# Supplementary material for: The Cholinergic Selectivity of FDA-Approved and Metabolite Compounds Examined with Molecular-Docking-Based Virtual Screening
Source: Molecules. 2024 May 16;29(10):2333. doi: 10.3390/molecules29102333 (PMC11124253; doi:10.3390/molecules29102333)
Supplement: Supplementary file 1 [file molecules-29-02333-s001.zip › molecules-2998522-supplementary.pdf]

Supporting Information for

The Cholinergic Selectivity of FDA-Approved and Metabolite Compounds Examined with Molecular-Docking-Based Virtual Screening

Michael D. Gambardella [1,*], Yigui Wang [1,2,*] and JiongDong Pang [1,*]

[1] Department of Chemistry and Biochemistry, Southern Connecticut State University, New Haven, CT 06515, USA

[2] Department of Chemistry and Chemical & Biochemical Engineering, University of New Haven, West Haven, CT 06516, USA

* Correspondence: gambardellm6@southernct.edu (M.D.G.); wangy3@southernct.edu or ywang@newhaven.edu (Y.W.); pangj1@southernct.edu (J.P.)

Table of Contents

- Molecular dynamics procedure---S2
- **Table S1.** The configuration files in molecular docking ---S2
- **Table S2.** Box sizes (Å) for 1W6R and 4BDS and their relative positions in the proteins, the step-by-step docking results for representative ligands. ---S3-S31
- **Table S3.** Specific interactions between AChE or BChE and the selected FDA-approved compounds or ZINC000253700110--S31-S33
- **Figure S1.** 1W6R and 4BDS "MultiSep" alignment with VMD and the "Surf" representation with original ligands in Protein Crystals ---S34
- **Figure S2-S5.** Enzyme-ligand interaction energies over 50ns for select ligands---------------------------S35
- **Figure S6-S9.** RMSD of ligand heavy atoms over 50ns for select ligands------------------------------S35-S36
- **Figure S10-S17.** Radius of Gyration over 50ns for select enzyme-ligand complexes---------------S36-S37
- **Table S4.** The FDA-approved dataset Vina virtual screening results ranked by difference in binding affinities (kcal/mol) for AChE selective compounds--S37-S89
- **Table S5.** The FDA-approved dataset Vina virtual screening results ranked by difference in binding affinities (kcal/mol) for BChE selective compounds ---S89-S138
- **Table S6.** The metabolites-in+vivo-clean dataset Vina virtual screening results ranked by difference in binding affinities (kcal/mol) for AChE selective compounds -------------------------------------- S138-S844
- **Table S7.** The metabolites-in+vivo-clean dataset Vina virtual screening results ranked by difference in binding affinities (kcal/mol) for BChE selective compounds --------------------------------------S844-S1913

Molecular Dynamics Procedure:

Ambrisentan, Caffeine, Ergotamine, and ZINC000253700110 were selected for further analysis via molecular dynamics simulations using GROMACS software on account of their enzyme selectivity in docking [10]. X-ray crystallography-derived atomic structures of AChE and BChE were prepared for the production run using the CHARMM36 forcefield. The lowest energy binding mode from the 12 ligands was chosen as the starting biological conformation, and stream files were generated using the CHARMM General Forcefield (CGenFF) server. Solvent molecules of water and chlorine anions were added to the enzyme-ligand complex. NVT and NPT equilibration steps were carried out with the Berendsen thermostat set for 310.15 K. The Parrinello-Rahman barostat was used for the NPT equilibration step. The molecular dynamics production run was performed for 50 ns.

Table S1 The configuration files in molecular docking

1W6R	4BDS
receptor = 1w6r.pdbqt<br>ligand = ambrisentan.pdbqt<br>out = ambrisentan-all-25.pdbqt<br>center_x = 4.8<br>center_y = 65.6<br>center_z = 56.8<br>size_x = 25<br>size_y = 25<br>size_z = 25<br>num_modes = 36<br>energy_range = 5<br>exhaustiveness = 10	receptor = 4bds.pdbqt<br>ligand = ambrisentan.pdbqt<br>out = ambrisentan-all-20.pdbqt<br>center_x = 136.3<br>center_y = 116.0<br>center_z = 42.3<br>size_x = 20<br>size_y = 20<br>size_z = 20<br>num_modes = 36<br>energy_range = 5<br>exhaustiveness = 10
Spacing (Step size) = 1.000 Å	Spacing (Step Size) = 1.000 Å

Table S2 Box sizes (Å) for 1W6R and 4BDS and their relative positions in the proteins, the step-by-step docking results for representative ligands.

**box size (Å)**	**1W6R**	4BDS
**10x10x10**	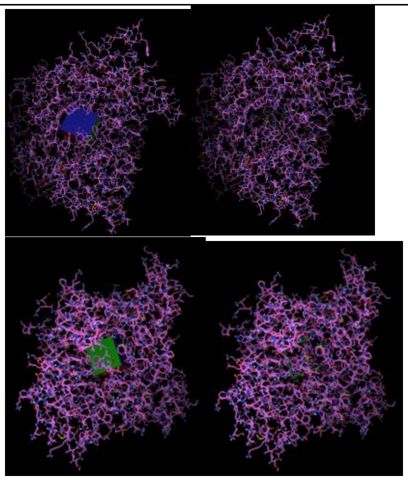	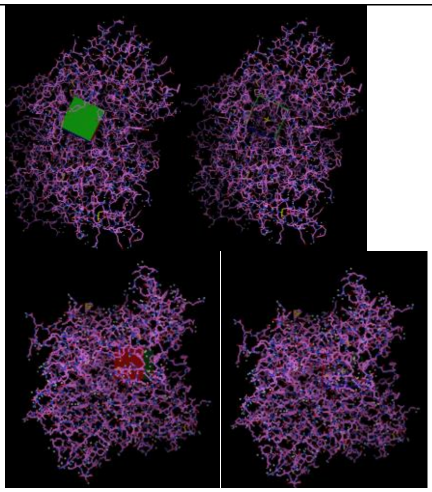
**16x16x16**	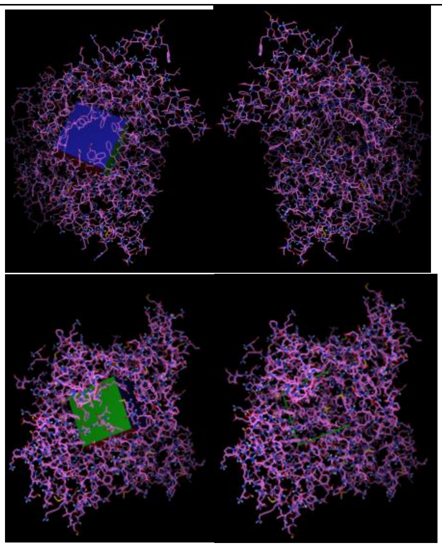	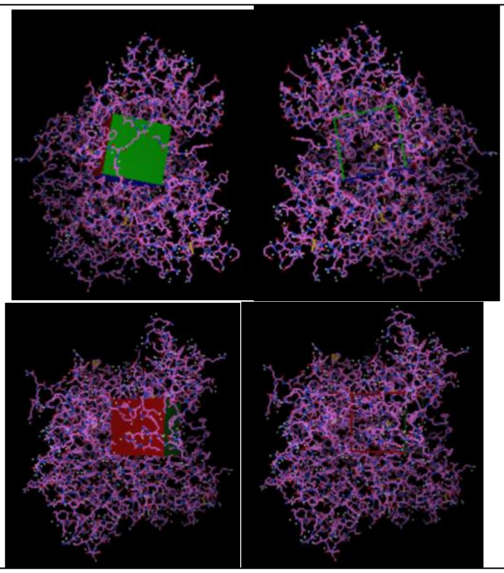

**Box size (Å)**	**1W6R**	4BDS
**20x20x20**	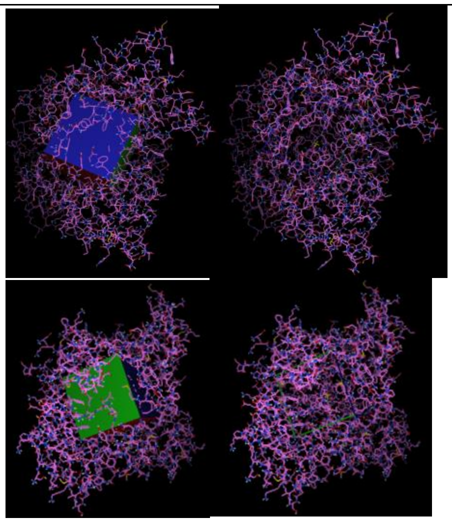	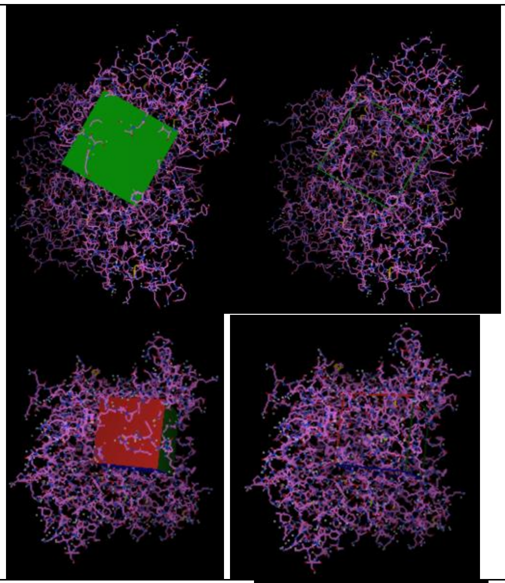
**24x24x24**	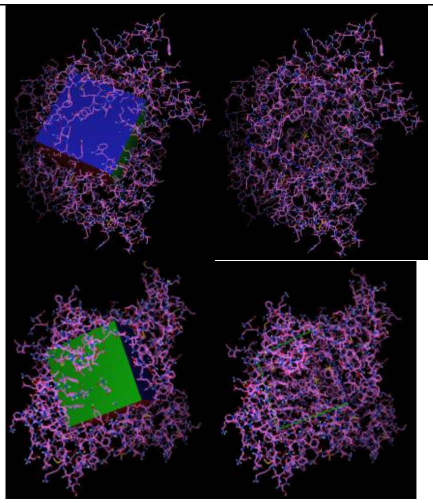	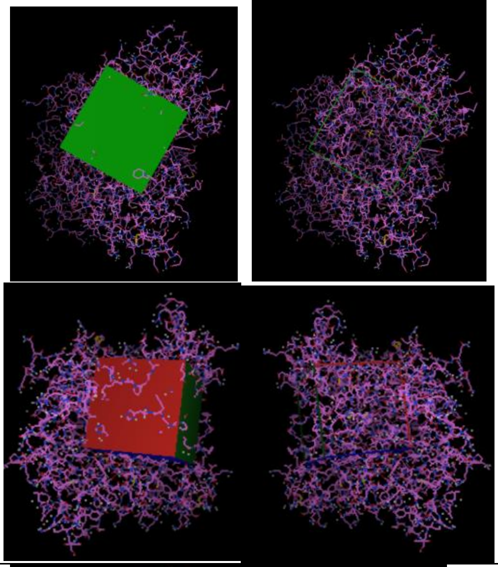
**30x30x30**	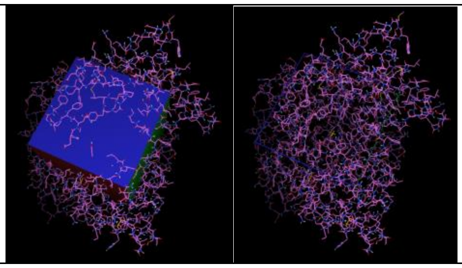	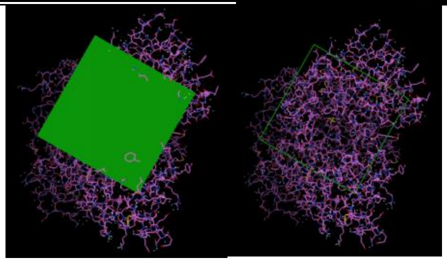

	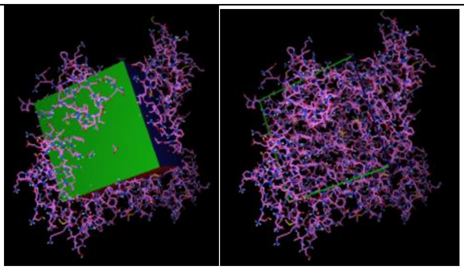	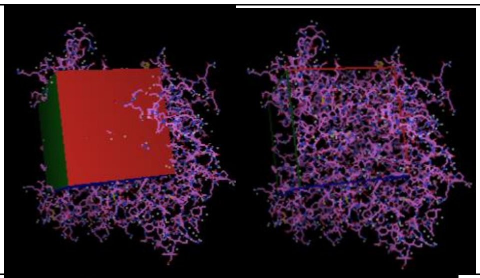
**40x40x40**	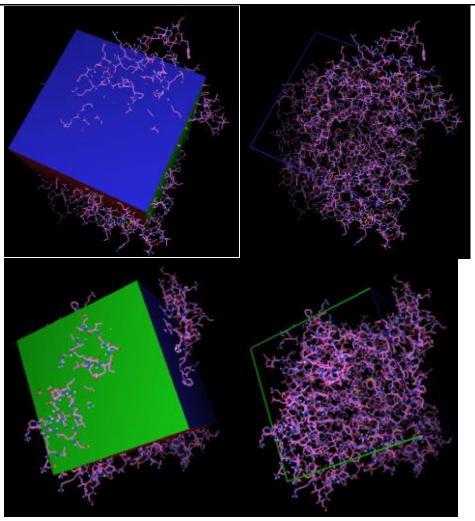	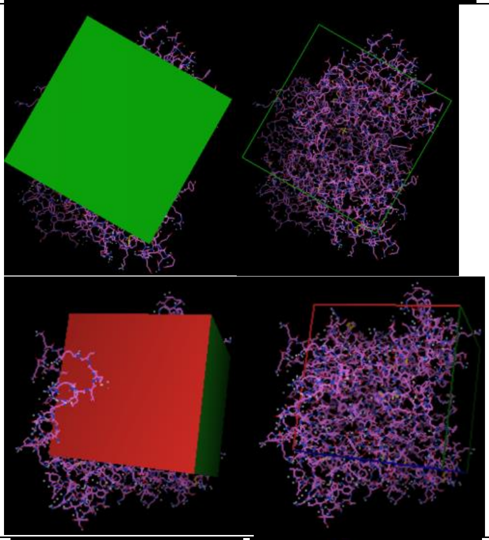
**50x50x50**	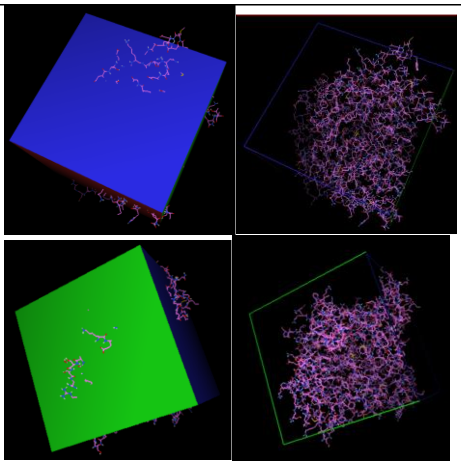	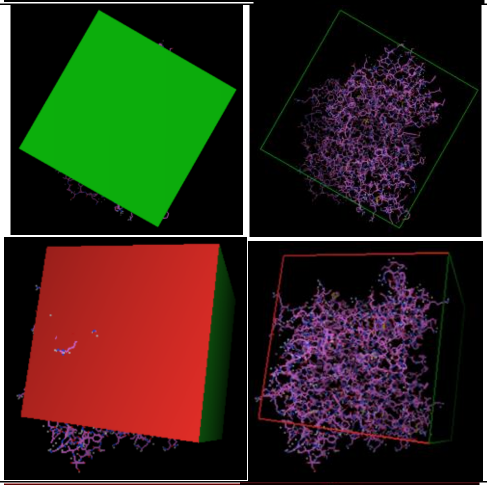
**60x60x60**	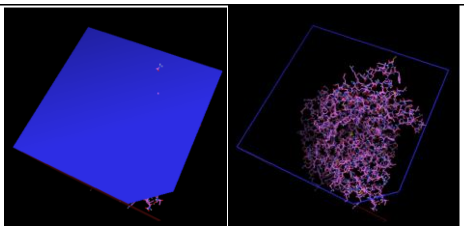	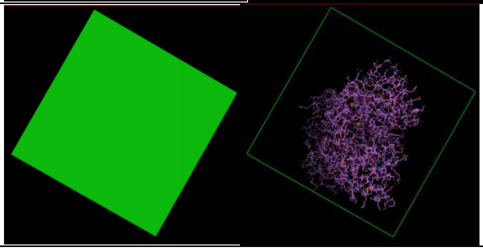

	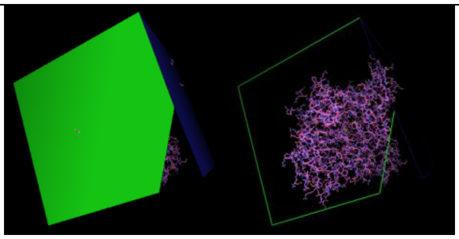	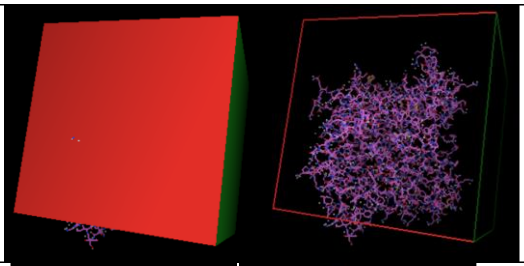
**70x70x70**	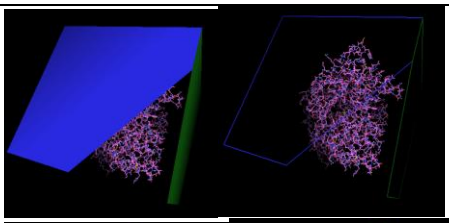<br>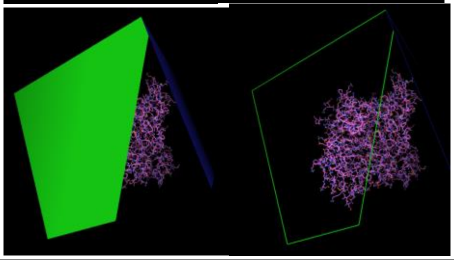	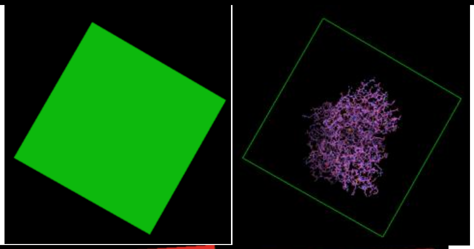<br>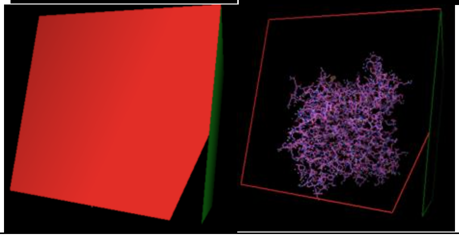
	**1W6R**	
	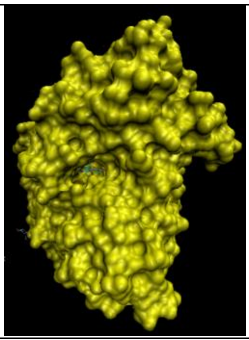 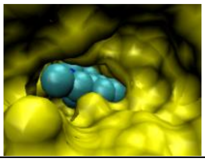	Size: 63.812, 61.080, 57.474<br>Center: 4.8, 64.8, 56.5<br>Ligand in Gorge: Galantamine<br>Dimension of GNT:<br>4.61, 6.68, 7.58 (233 Å^3)
	**4BDS**	
	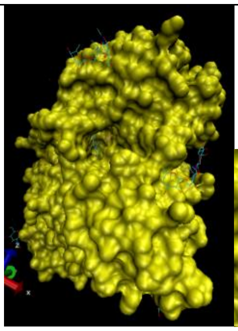 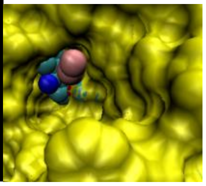	Size: 61.416, 59.514, 72.795<br>Center: 138.8, 123.3, 38.7<br>Ligands in Gorge:<br>Tacrine (THA),1-formyl-L-proline (FPK), and UnknownX (seven atoms in a chain)<br>Ligands Dimension:<br>10.77, 6.06, 5.05 (330 Å^3)<br>THA dimension:<br>3.76, 6.08, 5.86 (134 Å^3)
	ZINC_253700110	

	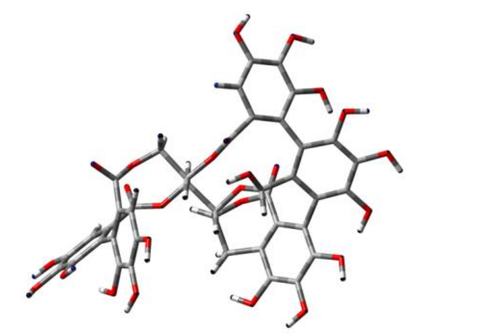	95 atoms MW:934.633<br>Center: 0.096, 0.094, -0.112<br>Size: 16.268, 10.901, 9.241 (1638.8 Å^3)
	1W6R	4BDS
	15x15x15 mode1 30x30x30<br>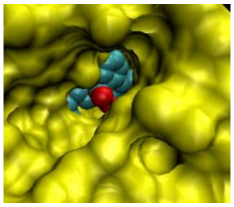 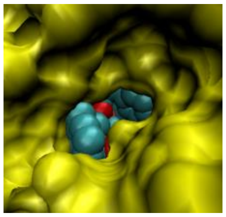<br>40 x40x40 70x70x70<br>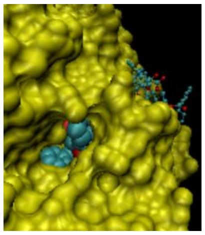 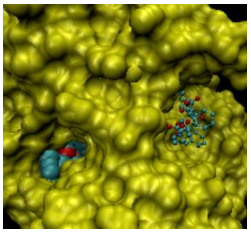	15x15x15 30x30x30 mode1<br>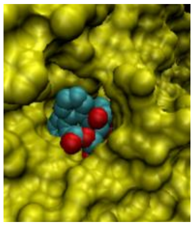 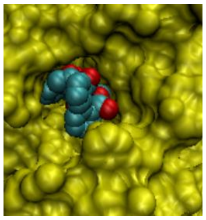<br>40x40x40 mode1 60x60x60 mode1<br>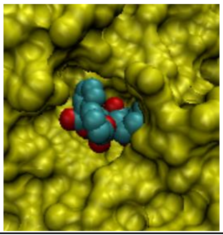 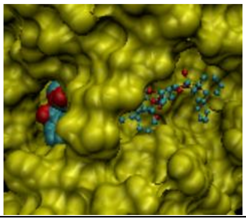
	**ZINC_257430040**	
	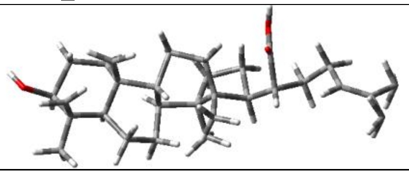	80 atoms MW:458.727<br>Center: 0.03, 0.103, 0.081<br>Size: 15.67, 6.61, 6.01 (622 Å^3)
	**1W6R**	**4BDS**
	25x25x25 mode1;<br>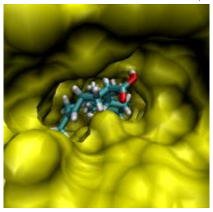<br>40x40x40<br>mode1, modes2,5,10, mode7<br>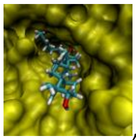, 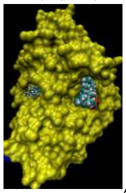, 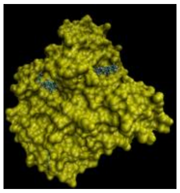	20x20x20 mode1; 70x70x70-mode1<br>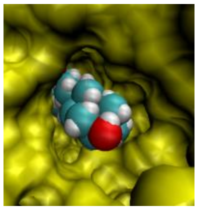 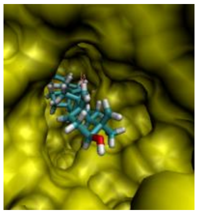
	**Ergotamine**	

	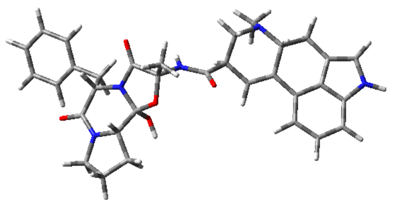	78 atoms, MW: 581.673<br>Center: -0.081, -0.010, 0.021<br>Size: 18.931, 10.122, 6.750 (1293 Å^3)
	**1W6R**	**4BDS**
	25x25x25 mode1, 30x30x30 mode1<br>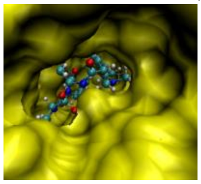 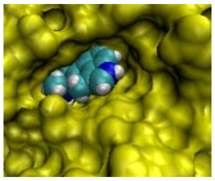<br>40x40x40<br>modes 1 and 8, modes 8 and 19<br>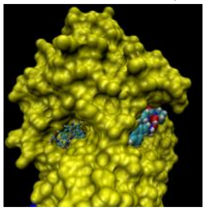 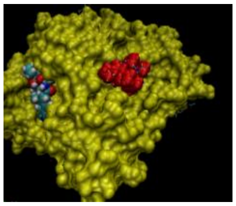	20x20x20 mode1; 70x70x70-mode1<br>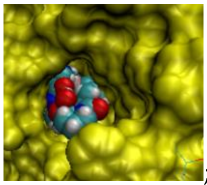; 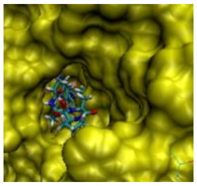
	**Ambrisentan**	
	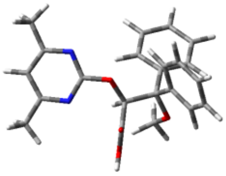	50 atoms MW: 378.4<br>Center:0.185, -0.158, -0.083<br>Size:10.48, 8.38, 7.60 (667 Å^3)
	**1W6R**	**4BDS**
	25x25x25 mode1<br>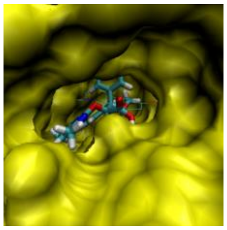<br>70x70x70<br>Mode1, mode8, modes 13, 17<br>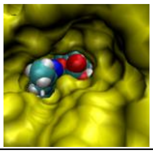 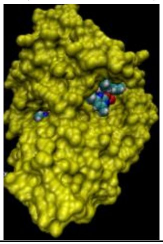 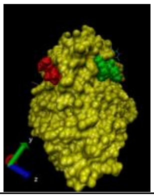	20x20x20 mode1; 70x70x70-mode1<br>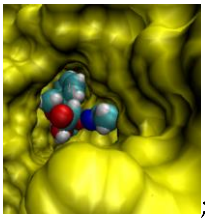; 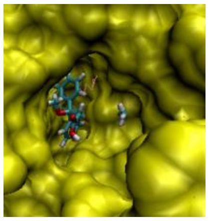
	**Caffein**	

	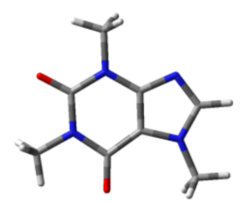	24 atoms MW 194.19 g/mol<br>Center: 0.063, 0.043, 0.000<br>Size: 7.326, 6.362, 1.799 (83.85 Å^3)
	**1W6R**	**4BDS**
	10x10x10 mode1 30x30x30 mode1<br>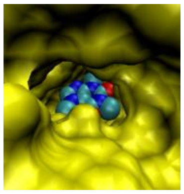 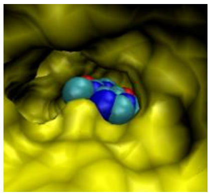<br>70x70x70 model1<br>(the same as 30x30x30)	10x10x10 mode1 30x30x30 mode1<br>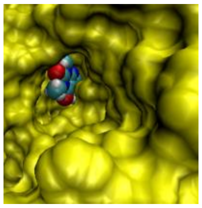 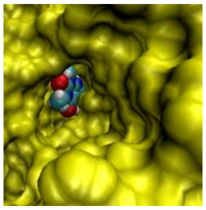<br>70x70x70 mode1<br>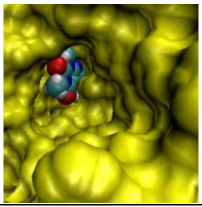
	**Avarol**	
	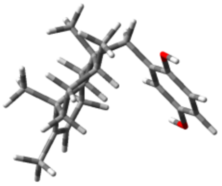	53 atoms, MW 314.5 g/mol<br>Center: -0.407, 0.231, -0.018<br>Size: 8.701, 7.904, 5.604 (385.4 Å^3)
	**1W6R**	**4BDS**
	10x10x10 mode1 30x30x30 mode1<br>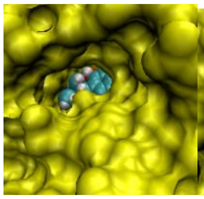 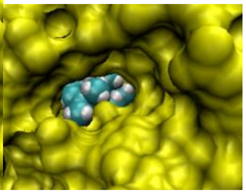<br>70x70x70 mode1<br>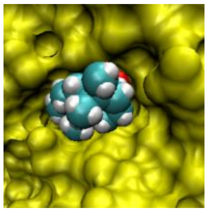	10x10x10 mode1 30x30x30 mode1<br>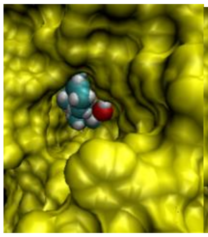 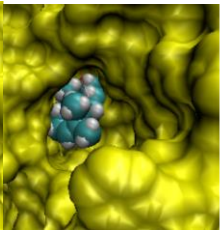<br>70x70x70 mode1<br>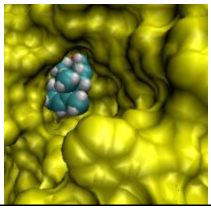
	**ZINC_253700110**	
**Box Size**	**1W6R**	4BDS
	mode | affinity | dist from best mode<br>| (kcal/mol) | rmsd l.b.| rmsd u.b.	mode | affinity | dist from best mode<br>| (kcal/mol) | rmsd l.b.| rmsd u.b.
**15x15x15**	1 248.0 0.000 0.000<br>2 250.3 2.057 6.318	1 20.8 0.000 0.000

**20x20x20**	1	147.3	0.000	0.000	1	4.2	0.000	0.000
					2	7.4	1.861	9.289
					3	7.9	2.603	4.647
					4	8.0	2.034	6.356
**25x25x25**	1	63.6	0.000	0.000	1	-6.2	0.000	0.000
					2	-4.4	2.452	9.645
					3	-4.3	2.461	5.969
					4	-2.9	2.327	6.669
					5	-2.1	2.626	6.083
**30x30x30**	1	51.0	0.000	0.000	1	-9.3	0.000	0.000
					2	-7.8	1.756	9.931
					3	-5.7	2.486	5.864
					4	-4.8	3.247	9.640
					5	-4.4	2.364	9.763
**40x40x40**	1	-6.7	0.000	0.000	1	-9.3	0.000	0.000
	2	-6.4	3.659	8.780	2	-9.2	4.071	11.792
	3	-6.1	2.995	10.122	3	-9.0	3.199	11.592
	4	-6.1	2.034	2.849	4	-8.8	3.511	11.932
	5	-6.0	2.479	10.185	5	-8.7	6.257	12.067
	6	-5.9	3.075	8.397	6	-8.5	4.012	8.012
	7	-5.9	2.371	9.549	7	-8.3	5.695	11.390
	8	-5.7	2.759	10.356	8	-8.2	3.129	10.993
	9	-5.7	2.782	10.412	9	-8.1	5.305	9.152
	10	-5.6	2.784	10.453	10	-8.1	4.634	9.391
	11	-5.5	2.687	9.908	11	-8.1	6.142	12.611
	12	-5.5	2.896	5.106	12	-8.1	6.809	10.798
	13	-5.5	3.260	6.219	13	-7.9	6.343	11.124
	14	-5.5	2.383	9.856	14	-7.8	1.744	9.934
	15	-5.3	2.619	4.362	15	-7.8	7.253	11.963
	16	-5.2	3.759	6.562	16	-7.8	4.482	10.754
	17	-5.2	3.827	7.561	17	-7.7	4.552	10.441
	18	-4.9	36.010	40.172	18	-7.6	6.816	11.143
	19	-4.8	2.299	3.240	19	-7.5	4.911	9.236
	20	-4.7	3.181	5.126	20	-7.2	5.270	10.671
**50x50x50**	1	-9.4	0.000	0.000	1	-9.2	0.000	0.000
	2	-8.9	2.794	3.756	2	-8.9	2.337	3.235
	3	-8.5	23.486	27.899	3	-8.9	2.619	4.168
	4	-7.9	2.877	6.045	4	-8.7	3.485	7.861
	5	-7.7	20.635	25.198	5	-8.6	2.956	9.593
	6	-7.6	11.794	14.906	6	-8.3	4.093	8.624
	7	-7.6	31.296	36.479	7	-8.1	3.422	9.208
	8	-7.5	21.915	26.323	8	-8.0	2.105	6.462
	9	-7.3	36.722	39.266	9	-8.0	28.193	31.794
	10	-7.2	32.682	37.176	10	-8.0	4.040	9.857
	11	-7.2	12.676	18.431	11	-8.0	5.228	10.372
	12	-7.2	32.276	35.936	12	-8.0	11.373	15.555
	13	-7.2	26.052	29.142	13	-7.9	3.916	5.872

	14	-7.1	36.300	40.616	14	-7.6	4.732	8.483
	15	-7.0	36.440	39.607	15	-7.5	28.897	33.808
	16	-6.8	34.865	38.112	16	-7.4	27.571	32.194
	17	-6.6	35.458	39.561	17	-7.3	28.580	31.934
	18	-6.6	20.355	24.988	18	-7.3	4.521	10.362
	19	-6.6	35.777	40.604	19	-7.2	3.152	10.082
	20	-6.4	36.489	40.670	20	-7.0	11.020	17.074
**60x60x60**	1	-10.5	0.000	0.000	1	-9.4	0.000	0.000
	2	-10.3	1.859	9.680	2	-9.4	3.302	9.582
	3	-10.2	1.601	2.308	3	-9.2	14.540	20.415
	4	-10.0	20.084	24.352	4	-9.2	24.953	28.779
	5	-9.2	2.556	4.854	5	-9.1	7.605	13.777
	6	-9.0	1.729	9.724	6	-9.0	3.741	6.895
	7	-8.5	24.678	28.577	7	-9.0	15.048	19.537
	8	-8.5	5.827	9.908	8	-8.8	15.140	19.197
	9	-8.5	2.691	4.383	9	-8.7	14.696	18.462
	10	-8.4	20.033	24.687	10	-8.4	29.002	33.624
	11	-8.0	24.943	30.553	11	-8.3	14.706	20.249
	12	-7.9	24.447	29.911	12	-8.2	23.346	28.473
	13	-7.8	4.993	9.283	13	-8.1	13.995	20.734
	14	-7.7	38.664	42.575	14	-8.1	25.421	28.707
	15	-7.6	22.474	27.815	15	-8.0	14.295	20.708
	16	-7.5	33.631	37.969	16	-8.0	14.948	21.142
	17	-7.5	38.127	42.605	17	-8.0	14.113	19.943
	18	-7.5	24.610	29.529	18	-7.8	15.703	18.892
	19	-7.4	38.089	42.557	19	-7.8	32.922	38.658
	20	-7.3	21.513	26.840	20	-7.6	14.209	19.620
**70x70x70**	1	-10.4	0.000	0.000	1	-10.1	0.000	0.000
	2	-10.2	1.629	2.330	2	-9.7	3.932	9.248
	3	-10.0	19.984	24.284	3	-9.4	1.747	2.099
	4	-9.4	3.524	8.783	4	-9.2	23.534	26.612
	5	-9.3	2.434	9.255	5	-9.0	16.451	21.135
	6	-8.8	4.312	10.233	6	-8.9	2.424	9.710
	7	-8.8	1.949	9.463	7	-8.5	18.036	21.373
	8	-8.6	5.083	9.196	8	-8.2	22.657	27.321
	9	-8.3	3.015	9.833	9	-8.2	3.300	9.564
	10	-8.1	23.375	28.482	10	-8.1	17.240	21.920
	11	-8.0	20.425	25.109	11	-7.9	15.839	19.755
	12	-8.0	24.884	30.506	12	-7.9	16.664	21.107
	13	-8.0	39.898	43.992	13	-7.9	2.579	3.442
	14	-7.9	24.343	29.763	14	-7.8	16.346	20.730
	15	-7.8	42.654	46.608	15	-7.6	15.875	20.827
	16	-7.8	3.493	8.353	16	-7.3	25.434	30.166
	17	-7.7	38.639	42.576	17	-7.2	16.265	21.882
	18	-7.6	40.479	44.766	18	-7.2	33.411	39.213
	19	-7.6	4.508	8.810	19	-7.2	2.600	6.978
	20	-7.6	29.090	34.230	20	-7.1	23.305	28.096

	**ZINC_257430040**							
Box Size(Å)	1W6R				4BDS			
	mode |	affinity | (kcal/mol) |	dist from best mode rmsd l.b.|	rmsd u.b.	mode |	affinity | (kcal/mol) |	dist from best mode rmsd l.b.|	rmsd u.b.
**10x10x10**	1	140.0	0.000	0.000	1	-7.5	0.000	0.000
	2	144.8	2.787	5.982	2	-6.1	2.540	6.737
**15x15x15**	1	37.9	0.000	0.000	1	-10.0	0.000	0.000
	2	39.1	1.160	1.580	2	-9.2	2.503	7.305
	3	39.2	1.987	7.428	3	-8.9	2.247	8.286
					4	-8.8	1.976	7.806
					5	-8.5	1.576	2.830
					6	-8.2	1.997	8.203
					7	-8.2	1.818	7.364
					8	-7.2	1.794	7.868
					9	-7.2	3.470	5.146
					10	-6.8	2.784	7.769
					11	-6.3	2.021	7.397
					12	-6.2	2.565	7.385
					13	-5.2	2.478	4.395
					14	-5.2	2.064	3.921
					15	-5.2	1.858	8.097
					16	-5.1	1.976	7.125
					17	-5.1	4.390	7.361
**20x20x20**	1	4.9	0.000	0.000	1	-10.0	0.000	0.000
	2	4.9	1.982	7.228	2	-9.5	1.496	2.025
	3	5.0	2.064	7.600	3	-9.5	2.239	4.210
	4	6.0	1.221	2.365	4	-9.2	2.487	7.307
	5	6.2	1.407	2.855	5	-9.2	1.566	2.507
	6	6.8	1.695	3.140	6	-9.1	1.982	7.884
	7	9.1	1.987	3.728	7	-8.9	2.027	8.295
					8	-8.9	2.221	8.268
					9	-8.8	2.644	4.752
					10	-8.8	2.285	3.541
					11	-8.7	2.203	7.544
					12	-8.5	1.653	7.479
					13	-8.5	2.426	4.410
					14	-8.5	2.153	3.912
					15	-8.4	1.981	3.977
					16	-8.4	2.142	7.986
					17	-8.4	2.204	3.683
					18	-8.4	1.710	2.574
					19	-8.3	3.413	5.375
					20	-8.3	2.383	4.939

**25x25x25**	1	-1.2	0.000	0.000	1	-9.9	0.000	0.000
	2	0.5	2.997	6.896	2	-9.5	2.203	4.169
	3	0.7	2.694	5.858	3	-9.1	2.691	7.249
	4	1.5	2.866	4.904	4	-9.1	1.505	2.407
	5	2.2	2.271	4.033	5	-9.1	3.186	4.522
	6	3.0	2.417	6.528	6	-9.0	1.675	2.186
	7	3.2	2.944	5.513	7	-8.9	2.370	4.210
	8	3.6	3.026	4.961	8	-8.9	1.790	8.067
	9	3.6	1.999	3.133	9	-8.9	1.921	8.211
					10	-8.8	2.223	8.259
					11	-8.8	2.835	4.438
					12	-8.6	2.683	4.884
					13	-8.6	2.165	7.776
					14	-8.5	2.731	4.246
					15	-8.3	1.823	7.853
					16	-8.3	3.142	5.078
					17	-8.2	2.153	8.646
					18	-8.1	3.004	5.070
					19	-8.1	2.528	4.834
					20	-8.0	2.184	4.391
**30x30x30**	1	-7.4	0.000	0.000	1	-10.0	0.000	0.000
	2	-5.5	1.952	2.889	2	-9.5	1.493	2.034
	3	-4.5	2.341	7.471	3	-9.5	2.258	4.212
	4	-4.0	2.262	8.083	4	-9.3	1.652	2.479
	5	-3.5	2.029	4.081	5	-9.2	3.198	4.655
					6	-8.9	2.435	4.285
					7	-8.8	2.562	4.575
					8	-8.8	1.935	8.025
					9	-8.7	2.165	7.425
					10	-8.6	3.346	5.182
					11	-8.6	2.812	4.875
					12	-8.5	2.189	7.866
					13	-8.4	2.589	4.570
					14	-8.3	2.407	3.635
					15	-8.2	2.976	4.989
					16	-8.1	2.579	4.959
					17	-8.1	2.246	8.598
					18	-8.0	1.918	3.886
					19	-7.9	3.079	4.816
					20	-7.8	2.691	9.002
**40x40x40**	1	-8.3	0.000	0.000	1	-9.9	0.000	0.000
	2	-8.1	1.733	7.882	2	-9.5	2.199	4.167
	3	-7.8	2.046	2.805	3	-9.4	1.326	1.764
	4	-7.0	20.472	23.394	4	-9.1	3.177	4.514
	5	-7.0	20.706	22.950	5	-9.0	1.516	2.433
	6	-6.9	20.838	23.139	6	-8.9	2.407	4.320
	7	-6.7	30.785	34.305	7	-8.8	1.957	8.223

	8	-6.6	30.817	32.871	8	-8.7	2.488	4.528
	9	-6.5	20.910	23.482	9	-8.7	2.813	4.960
	10	-6.4	20.557	23.012	10	-8.6	1.852	8.146
	11	-6.2	2.131	8.098	11	-8.6	2.029	7.992
	12	-6.0	30.508	33.522	12	-8.5	2.117	7.786
	13	-5.9	20.612	23.367	13	-8.3	2.523	4.588
	14	-5.8	30.780	32.649	14	-8.2	1.983	7.899
	15	-5.7	30.925	33.187	15	-8.0	2.786	9.092
	16	-5.7	31.083	32.974	16	-7.9	3.567	10.162
	17	-5.5	1.754	2.678	17	-7.9	2.211	4.375
	18	-5.5	16.524	19.139	18	-7.7	3.506	10.005
	19	-5.4	30.951	33.629	19	-7.4	3.298	9.510
	20	-5.4	17.754	20.229				
**50x50x50**	1	-8.7	0.000	0.000	1	-10.0	0.000	0.000
	2	-7.9	18.806	22.149	2	-9.5	2.315	4.341
	3	-7.8	20.875	22.561	3	-9.4	1.830	2.549
	4	-7.8	20.016	22.803	4	-9.2	1.576	2.497
	5	-7.7	2.117	7.378	5	-9.2	3.174	4.609
	6	-7.5	2.490	3.642	6	-9.1	2.202	7.443
	7	-7.4	3.519	7.566	7	-8.9	3.024	4.854
	8	-7.3	1.761	8.266	8	-8.8	1.814	8.161
	9	-7.3	2.698	7.282	9	-8.7	1.951	7.888
	10	-7.3	20.202	22.992	10	-8.7	2.194	7.470
	11	-7.2	1.493	7.280	11	-8.6	1.826	8.070
	12	-7.0	17.495	21.338	12	-8.5	2.265	4.250
	13	-6.9	2.235	3.581	13	-8.5	2.776	4.290
	14	-6.7	3.252	9.091	14	-8.4	14.288	17.997
	15	-6.6	23.527	26.604	15	-8.2	13.665	18.712
	16	-6.5	2.541	7.602	16	-8.2	1.935	7.891
	17	-6.4	21.831	25.904	17	-8.2	13.777	19.084
	18	-6.3	22.581	24.966	18	-8.1	2.739	9.023
	19	-6.2	23.150	26.731	19	-8.0	15.568	19.650
	20	-6.1	17.400	20.315	20	-7.9	3.353	5.291
**60x60x60**	1	-8.8	0.000	0.000	1	-10.0	0.000	0.000
	2	-8.7	2.383	3.317	2	-9.5	1.459	1.976
	3	-7.9	20.732	22.529	3	-9.2	3.169	4.611
	4	-7.9	2.910	4.218	4	-8.9	2.245	4.229
	5	-7.7	19.555	22.267	5	-8.8	2.296	8.426
	6	-7.7	19.979	22.621	6	-8.7	2.196	7.468
	7	-7.6	16.373	19.910	7	-8.7	2.827	5.165
	8	-7.5	2.421	4.020	8	-8.5	2.053	7.204
	9	-7.5	2.739	4.665	9	-8.5	2.684	7.333
	10	-7.4	2.467	8.044	10	-8.5	3.000	4.922
	11	-7.4	3.409	7.605	11	-8.4	1.970	8.262
	12	-7.4	20.251	23.133	12	-8.4	1.607	7.537
	13	-7.3	2.000	7.704	13	-8.4	2.084	7.422
	14	-7.3	1.550	7.385	14	-8.3	14.195	17.920

	15	-7.1	17.128	20.946	15	-8.2	3.194	5.366
	16	-7.0	1.821	7.938	16	-8.2	2.551	4.622
	17	-6.9	16.425	19.595	17	-8.0	15.564	19.669
	18	-6.8	17.112	20.911	18	-8.0	2.585	8.904
	19	-6.7	1.896	8.499	19	-8.0	2.948	4.489
	20	-6.6	16.702	19.389	20	-7.8	2.419	8.762
**70x70x70**	1	-8.9	0.000	0.000	1	-9.9	0.000	0.000
	2	-7.9	1.852	3.205	2	-9.4	2.208	4.240
	3	-7.8	19.299	22.220	3	-9.2	2.786	7.210
	4	-7.7	1.619	2.240	4	-8.9	3.020	4.278
	5	-7.5	1.391	1.810	5	-8.8	2.002	8.319
	6	-7.5	2.404	3.524	6	-8.8	2.175	8.186
	7	-7.4	1.805	8.496	7	-8.5	1.604	2.733
	8	-7.2	3.025	7.425	8	-8.3	2.136	7.736
	9	-7.0	3.139	8.064	9	-8.2	2.507	3.790
	10	-6.9	20.466	23.258	10	-8.1	2.137	4.248
	11	-6.9	2.754	9.237	11	-8.1	14.566	18.068
	12	-6.7	1.935	7.843	12	-8.1	2.164	8.585
	13	-6.6	16.174	19.634	13	-8.0	13.918	18.840
	14	-6.6	16.604	18.912	14	-7.9	3.000	5.006
	15	-6.5	21.915	24.764	15	-7.6	2.149	8.660
	16	-6.5	31.591	36.553	16	-7.4	3.454	9.917
	17	-6.4	39.708	42.277	17	-7.4	16.192	19.908
	18	-6.3	39.560	41.446	18	-7.3	3.531	9.833
	19	-6.3	18.496	21.844	19	-7.3	15.673	19.267
	20	-6.2	30.542	35.019	20	-6.9	5.001	10.861
	**Ergotamine**							
	1W6R				4BDS			
	mode |	affinity | (kcal/mol) |	dist from best mode rmsd l.b.|	rmsd u.b.	mode |	affinity | (kcal/mol) |	dist from best mode rmsd l.b.|	rmsd u.b.
**10x10x10**	1	200.1	0.000	0.000	1	-7.7	0.000	0.000
					2	-7.4	2.312	7.243
					3	-6.0	2.538	7.266
**15x15x15**	1	51.5	0.000	0.000	1	-13.2	0.000	0.000
	2	54.3	1.961	4.312	2	-13.2	2.090	5.197
	3	54.8	2.116	5.259	3	-12.7	1.795	4.576
					4	-12.4	1.461	1.969
					5	-12.1	2.192	7.246
					6	-11.8	1.999	6.920
					7	-11.6	2.400	6.760
					8	-11.3	2.098	6.702
					9	-10.8	2.192	7.788
					10	-10.7	2.399	7.523
					11	-10.2	2.520	8.044
					12	-9.7	1.876	4.853
					13	-9.6	2.591	6.296

					14	-9.5	2.327	5.903
					15	-9.3	1.990	3.838
					16	-9.1	1.994	2.764
					17	-8.9	2.628	7.067
					18	-8.7	2.284	7.061
**20x20x20**	1	13.5	0.000	0.000	1	-13.2	0.000	0.000
	2	15.4	2.057	5.671	2	-13.1	2.082	5.192
	3	15.8	1.560	2.015	3	-13.0	2.584	6.258
	4	16.3	1.572	3.034	4	-12.3	1.544	2.104
	5	17.7	2.327	7.435	5	-12.1	2.229	8.990
					6	-12.0	2.208	7.251
					7	-12.0	2.670	4.570
					8	-12.0	2.272	8.029
					9	-11.8	2.549	4.559
					10	-11.6	2.828	8.444
					11	-11.6	2.819	7.131
					12	-11.6	2.494	5.774
					13	-11.5	2.236	8.457
					14	-11.4	1.845	4.627
					15	-11.4	2.868	5.835
					16	-11.4	2.412	5.542
					17	-11.4	2.634	8.452
					18	-11.2	2.464	8.500
					19	-11.2	2.184	5.141
					20	-11.2	1.413	4.582
**25x25x25**	1	-2.5	0.000	0.000	1	-12.1	0.000	0.000
	2	1.6	1.776	2.832	2	-12.0	1.965	5.157
	3	2.2	1.822	4.981	3	-12.0	2.355	5.401
					4	-11.6	2.076	3.028
					5	-11.1	2.223	4.900
					6	-11.0	2.629	8.160
					7	-10.9	2.565	6.618
					8	-10.9	3.071	5.500
					9	-10.9	2.699	8.027
					10	-10.9	2.467	7.645
					11	-10.8	3.047	4.925
					12	-10.7	2.834	8.082
					13	-10.7	2.485	6.952
					14	-10.5	3.134	4.924
					15	-10.4	3.641	8.727
					16	-10.3	2.981	5.744
					17	-10.2	3.330	8.215
					18	-10.2	2.720	8.030
					19	-10.1	2.838	7.758
					20	-10.0	3.435	5.433
**30x30x30**	1	-10.9	0.000	0.000	1	-13.2	0.000	0.000
	2	-10.3	1.628	3.949	2	-13.1	2.078	5.242

	3 -6.3 1.981 6.915	3 -13.0 2.589 6.272<br>4 -12.9 2.699 7.180<br>5 -12.3 1.543 2.125<br>6 -12.2 2.270 7.504<br>7 -12.0 2.207 7.299<br>8 -12.0 2.669 4.552<br>9 -12.0 2.197 8.981<br>10 -11.9 2.098 8.360<br>11 -11.8 2.538 8.851<br>12 -11.7 2.600 4.549<br>13 -11.6 2.500 5.772<br>14 -11.6 2.747 9.263<br>15 -11.3 2.400 9.144<br>16 -11.2 3.187 5.783<br>17 -11.2 2.933 5.923<br>18 -11.2 2.472 8.229<br>19 -11.2 2.457 8.016<br>20 -11.1 2.783 5.360
**22x24x28**	**1 -10.3 0.000 0.000**<br>**2 -10.3 0.016 1.602**<br>**3 -9.4 1.760 4.411**<br>**4 -9.2 1.107 1.911**<br>**5 -8.9 2.102 4.995**<br>**6 -8.9 2.096 4.912**<br>**7 -8.8 6.964 10.443**<br>**8 -8.8 6.968 10.504**<br>**9 -8.7 1.511 4.299**<br>**10 -8.7 1.502 3.978**<br>**11 -8.7 1.627 2.393**<br>**12 -8.5 1.739 2.917**<br>**13 -8.4 7.348 10.980**<br>**14 -8.4 7.347 10.901**<br>**15 -8.4 1.734 3.110**<br>**16 -8.4 7.363 10.946**<br>**17 -8.3 1.905 3.614**<br>**18 -8.2 1.779 2.504**<br>**19 -7.8 2.405 5.883**<br>**20 -7.6 7.143 10.699**	**1 -8.8 0.000 0.000**<br>**2 -8.8 0.069 2.058**<br>**3 -8.8 2.028 3.886**<br>**4 -8.8 2.038 4.162**<br>**5 -8.7 1.871 4.091**<br>**6 -8.7 1.890 3.828**<br>**7 -8.6 3.468 6.542**<br>**8 -8.6 1.929 3.473**<br>**9 -8.6 1.929 3.328**
**40x40x40**	1 -11.9 0.000 0.000<br>2 -11.6 2.527 8.754<br>3 -11.5 6.612 12.664<br>4 -11.4 2.212 4.715<br>5 -11.0 2.740 9.106<br>6 -10.3 1.975 3.850<br>7 -10.2 14.172 16.101<br>8 -9.9 1.629 3.049<br>9 -9.9 14.365 15.946	1 -12.1 0.000 0.000<br>2 -12.0 2.362 5.334<br>3 -12.0 1.972 5.211<br>4 -11.1 2.256 4.912<br>5 -11.0 2.638 8.162<br>6 -11.0 3.062 5.518<br>7 -11.0 2.575 7.787<br>8 -10.6 2.543 7.627<br>9 -10.5 2.669 5.813

	10	-9.9	13.077	16.165	10	-10.5	3.565	8.828
	11	-9.6	13.622	16.555	11	-10.3	2.910	8.044
	12	-9.6	27.222	31.473	12	-10.3	3.012	5.722
	13	-9.5	13.059	14.977	13	-10.2	3.321	5.197
	14	-9.4	14.010	17.488	14	-10.2	2.711	7.765
	15	-9.4	14.073	18.165	15	-10.1	2.758	4.234
	16	-9.3	13.460	16.112	16	-10.1	3.182	8.690
	17	-9.3	14.027	16.832	17	-10.1	3.349	4.519
	18	-9.2	14.484	16.508	18	-10.0	2.900	5.993
	19	-9.2	13.427	16.693	19	-9.8	3.007	5.065
	20	-9.1	13.326	16.464	20	-9.8	3.753	5.962
**50x50x50**	1	-10.3	0.000	0.000	1	-13.2	0.000	0.000
	2	-10.0	6.305	10.125	2	-13.2	2.054	5.193
	3	-9.8	4.977	9.155	3	-13.1	2.542	6.150
	4	-9.7	3.374	9.020	4	-12.1	2.191	7.316
	5	-9.4	2.951	8.404	5	-12.1	2.654	4.185
	6	-9.4	17.609	20.435	6	-12.0	2.166	8.933
	7	-9.4	4.481	7.730	7	-11.7	2.351	8.600
	8	-9.3	4.490	9.265	8	-11.7	2.371	5.216
	9	-9.3	4.205	8.698	9	-11.7	2.426	5.644
	10	-9.2	16.353	21.088	10	-11.6	2.672	9.226
	11	-9.1	14.720	19.241	11	-11.5	3.166	8.760
	12	-9.0	14.995	18.747	12	-11.5	2.565	8.955
	13	-9.0	2.553	9.180	13	-11.5	2.515	8.388
	14	-9.0	3.566	9.175	14	-11.4	2.471	5.478
	15	-8.9	15.233	19.429	15	-11.3	19.183	21.888
	16	-8.9	15.681	19.669	16	-11.1	1.934	2.335
	17	-8.9	5.048	10.213	17	-11.1	3.363	9.177
	18	-8.8	15.324	19.457	18	-11.1	2.654	9.528
	19	-8.8	15.619	18.742	19	-11.0	14.451	18.278
	20	-8.6	3.238	9.110	20	-10.9	3.115	6.340
**60x60x60**	1	-10.3	0.000	0.000	1	-12.1	0.000	0.000
	2	-9.6	4.627	8.762	2	-12.0	2.367	5.412
	3	-9.4	2.960	8.410	3	-12.0	2.061	5.087
	4	-9.4	4.464	7.664	4	-11.9	2.122	4.323
	5	-9.2	6.843	10.698	5	-11.6	2.076	2.994
	6	-9.2	4.703	8.868	6	-11.0	3.038	5.431
	7	-9.2	2.722	9.064	7	-10.9	2.577	8.096
	8	-8.8	15.477	19.837	8	-10.6	2.934	4.682
	9	-8.8	5.222	10.354	9	-10.5	3.438	8.696
	10	-8.8	15.300	18.333	10	-10.4	3.322	4.923
	11	-8.7	2.646	5.654	11	-10.3	19.085	21.682
	12	-8.6	2.119	7.135	12	-10.3	2.755	7.925
	13	-8.4	1.805	5.401	13	-10.1	2.625	7.387
	14	-8.4	4.363	6.603	14	-10.0	13.207	17.339
	15	-8.3	4.117	8.939	15	-9.9	2.283	5.272
	16	-8.3	15.384	19.313	16	-9.8	3.056	4.904

	1W6R mode	affinity	dist	from best mode	4BDS mode	affinity	dist	from best mode
	17	-8.3	2.697	8.693	17	-9.7	3.478	5.514
	18	-8.3	18.299	23.119	18	-9.6	3.506	8.191
	19	-8.2	3.319	8.499	19	-9.5	13.503	18.457
	20	-8.0	25.441	28.891	20	-9.5	3.637	6.179
**70x70x70**	1	-10.3	0.000	0.000	1	-13.3	0.000	0.000
	2	-9.8	5.084	9.258	2	-13.2	2.057	5.159
	3	-9.5	4.490	8.791	3	-13.1	2.554	6.206
	4	-9.4	2.099	7.038	4	-13.0	2.675	7.128
	5	-9.3	3.040	8.469	5	-12.7	1.786	4.555
	6	-9.3	4.380	7.659	6	-12.1	2.196	8.960
	7	-9.3	4.520	9.263	7	-12.0	2.648	4.524
	8	-9.1	16.424	21.128	8	-11.9	3.080	4.544
	9	-8.9	3.657	9.072	9	-11.7	2.847	9.384
	10	-8.7	15.550	19.836	10	-11.5	2.570	8.901
	11	-8.7	4.160	9.075	11	-11.4	19.154	21.922
	12	-8.7	2.419	6.052	12	-11.4	2.636	8.429
	13	-8.7	18.826	23.595	13	-11.3	2.890	5.099
	14	-8.5	17.535	22.151	14	-11.0	14.541	18.345
	15	-8.4	15.867	19.922	15	-10.9	2.799	4.532
	16	-8.3	15.658	19.936	16	-10.8	14.172	18.447
	17	-8.3	4.296	6.451	17	-10.8	3.033	6.088
	18	-8.2	16.647	21.257	18	-10.7	2.843	9.546
	19	-8.1	15.880	19.599	19	-10.7	3.031	8.486
	20	-8.0	4.059	7.369	20	-10.6	2.364	5.385
**80x80x80**	1	-9.0	0.000	0.000	1	-12.1	0.000	0.000
	2	-8.8	2.402	3.438	2	-12.0	2.367	5.409
	3	-8.2	20.549	22.591	3	-11.9	2.122	4.321
	4	-8.2	2.340	3.841	4	-11.6	2.081	3.054
	5	-8.2	1.843	2.957	5	-11.3	2.296	5.208
	6	-8.1	18.966	22.169	6	-11.0	2.552	7.759
	7	-8.0	2.727	4.045	7	-11.0	3.092	5.552
	8	-7.8	17.219	20.254	8	-10.9	2.665	7.974
	9	-7.7	19.506	21.912	9	-10.5	3.451	8.721
	10	-7.1	3.094	7.353	10	-10.4	2.754	5.608
	11	-7.1	2.890	7.528	11	-10.3	19.086	21.650
	12	-7.0	3.864	7.557	12	-10.2	3.558	5.568
	13	-7.0	18.722	22.029	13	-10.0	2.909	7.839
	14	-7.0	16.304	18.617	14	-10.0	2.494	5.598
	15	-6.8	39.896	42.322	15	-9.9	2.670	4.809
	16	-6.8	28.085	31.337	16	-9.6	3.536	6.649
	17	-6.7	2.010	7.789	17	-9.6	3.032	5.912
	18	-6.6	39.748	41.699	18	-9.6	3.704	5.591
	19	-6.4	16.699	20.129	19	-9.2	2.634	7.709
	20	-6.4	36.755	40.280	20	-9.2	2.996	5.484

	**Ambrisentan**	
	**1W6R**	**4BDS**
	mode | affinity | dist from best mode	mode | affinity | dist from best mode

	l	(kcal/mol) l	rmsd l.b.l	rmsd u.b.	l	(kcal/mol) l	rmsd l.b.l	rmsd u.b.
**10x10x10**	1	56.2	0.000	0.000	1	-8.7	0.000	0.000
					2	-8.7	1.756	4.496
					3	-8.7	1.741	4.227
					4	-8.7	0.081	1.845
					5	-7.8	1.687	3.911
					6	-7.5	2.111	5.786
					7	-7.5	2.177	5.794
					8	-7.4	2.201	5.096
					9	-7.4	2.199	4.966
					10	-7.2	1.476	3.994
					11	-7.2	1.630	3.172
					12	-7.2	2.164	5.551
					13	-7.1	2.145	5.796
					14	-7.0	2.225	5.843
					15	-7.0	2.195	5.690
					16	-6.9	2.187	5.775
					17	-6.9	1.365	2.746
					18	-6.9	2.077	6.438
					19	-6.6	1.491	2.384
					20	-6.6	2.195	4.052
**15x15x15**	1	2.2	0.000	0.000	1	-8.8	0.000	0.000
	2	4.6	2.315	6.120	2	-8.8	0.059	1.603
	3	4.7	2.446	6.159	3	-8.7	2.067	4.109
	4	5.9	2.313	6.224	4	-8.7	1.884	4.281
	5	6.0	2.366	6.103	5	-8.5	1.902	4.827
	6	6.1	2.016	3.753	6	-8.5	2.491	5.403
	7	6.2	2.000	4.099	7	-8.4	2.124	5.705
	8	7.1	2.357	6.254	8	-8.3	2.402	6.025
					9	-8.2	2.433	5.943
					10	-8.2	1.596	2.662
					11	-8.1	1.809	3.478
					12	-8.1	1.699	3.774
					13	-8.1	1.959	5.850
					14	-8.0	1.818	4.010
					15	-8.0	2.116	4.457
					16	-8.0	2.197	4.872
					17	-8.0	2.790	5.797
					18	-8.0	1.670	3.676
					19	-8.0	1.994	3.604
					20	-7.8	2.018	3.705
**20x20x20**	1	-7.2	0.000	0.000	1	-8.8	0.000	0.000
	2	-7.1	0.078	1.845	2	-8.8	0.052	2.058
	3	-6.7	1.207	4.148	3	-8.8	2.072	4.084
	4	-6.7	1.170	3.758	4	-8.7	1.867	4.514
	5	-6.2	1.161	4.412	5	-8.6	2.909	6.417
	6	-6.2	1.160	4.093	6	-8.6	2.899	6.431

	7	-6.2	2.367	4.997	7	-8.5	2.489	5.514
	8	-6.1	1.669	2.976	8	-8.5	1.791	3.293
	9	-6.0	1.656	3.069	9	-8.4	2.059	5.786
	10	-5.8	2.097	5.308	10	-8.3	2.702	6.287
	11	-3.9	1.490	3.943	11	-8.3	2.767	5.681
	12	-2.5	2.175	6.167	12	-8.3	2.422	5.934
	13	-2.3	2.100	5.912	13	-8.2	1.687	2.471
	14	-2.2	1.349	4.065	14	-8.1	2.284	4.626
					15	-8.1	1.797	3.448
					16	-7.8	2.526	5.168
					17	-7.7	2.000	3.736
					18	-7.7	2.389	3.361
					19	-7.6	3.192	5.373
					20	-7.6	3.198	5.385
**25x25x25**	1	-10.1	0.000	0.000	1	-8.8	0.000	0.000
	2	-10.0	0.073	1.845	2	-8.8	2.026	4.112
	3	-9.7	1.164	2.035	3	-8.8	2.023	3.846
	4	-9.1	1.425	3.890	4	-8.7	1.889	3.884
	5	-9.0	1.457	4.192	5	-8.6	1.942	3.467
	6	-8.3	1.671	3.887	6	-8.5	3.437	6.492
	7	-8.3	1.691	4.162	7	-8.5	3.083	6.345
	8	-8.2	1.659	3.910	8	-8.5	3.398	6.537
	9	-8.1	1.026	2.566	9	-8.5	3.076	6.535
	10	-8.0	1.715	3.614	10	-8.3	1.623	3.596
	11	-8.0	1.031	1.573	11	-8.3	2.561	6.041
	12	-7.9	1.642	3.948	12	-8.2	3.057	5.765
	13	-7.9	2.059	5.621	13	-8.2	3.051	5.898
	14	-7.8	2.054	5.618	14	-8.1	1.497	2.159
	15	-7.2	2.151	4.776	15	-8.1	2.474	4.564
	16	-7.2	1.661	3.761	16	-8.1	1.508	2.844
	17	-6.8	2.048	4.790	17	-8.1	2.457	5.039
	18	-6.7	2.660	5.365	18	-8.1	2.277	4.955
	19	-6.7	2.212	5.780	19	-8.0	2.318	4.559
	20	-6.4	2.002	5.881	20	-8.0	3.012	5.051
**30x30x30**	1	-10.4	0.000	0.000	1	-8.8	0.000	0.000
	2	-10.3	0.009	1.844	2	-8.8	0.053	1.602
	3	-9.4	1.762	4.370	3	-8.7	2.080	4.057
	4	-9.4	1.773	4.588	4	-8.7	1.890	4.579
	5	-8.9	1.856	4.795	5	-8.7	1.876	4.276
	6	-8.9	1.855	4.623	6	-8.6	1.892	4.822
	7	-8.7	1.501	3.989	7	-8.5	2.493	5.360
	8	-8.6	1.623	2.568	8	-8.5	1.765	3.295
	9	-8.6	1.163	3.899	9	-8.4	2.150	5.728
	10	-8.4	2.084	4.407	10	-8.3	2.405	6.027
	11	-8.4	1.744	3.234	11	-8.3	2.694	6.381
	12	-8.2	1.647	2.311	12	-8.2	2.473	5.916
	13	-7.8	1.948	3.443	13	-8.2	2.381	4.919

	14	-7.8	1.737	4.204	14	-8.2	1.636	2.408
	15	-7.7	1.742	3.848	15	-8.2	1.693	2.878
	16	-7.6	1.968	3.578	16	-8.1	1.797	3.462
	17	-7.6	2.296	6.074	17	-8.1	1.656	3.795
	18	-7.2	1.979	4.247	18	-8.1	1.876	3.701
	19	-7.1	2.223	6.225	19	-8.1	2.891	6.458
	20	-7.1	2.301	6.035	20	-8.0	2.325	3.719
**40x40x40**	1	-10.4	0.000	0.000	1	-8.8	0.000	0.000
	2	-10.4	0.010	1.602	2	-8.8	2.022	3.844
	3	-9.4	1.749	4.576	3	-8.8	2.030	4.110
	4	-9.3	1.790	4.432	4	-8.7	1.891	3.862
	5	-8.9	2.093	4.984	5	-8.7	1.855	4.153
	6	-8.9	2.099	4.907	6	-8.6	1.935	3.339
	7	-8.6	1.617	2.545	7	-8.6	3.454	6.539
	8	-8.6	1.610	2.557	8	-8.6	3.442	6.567
	9	-7.2	1.687	3.567	9	-8.5	3.060	6.309
	10	-7.0	2.106	6.190	10	-8.1	2.090	6.110
	11	-7.0	13.359	15.519	11	-8.0	2.674	5.843
	12	-6.8	13.710	15.789	12	-8.0	1.489	2.879
	13	-6.2	26.385	29.026	13	-8.0	2.591	5.977
	14	-6.2	26.380	29.033	14	-7.9	1.481	2.870
	15	-6.1	26.436	28.571	15	-7.8	2.998	5.797
	16	-6.1	26.470	28.630	16	-7.7	2.596	5.241
	17	-6.1	26.478	29.090	17	-7.7	13.068	15.631
	18	-6.0	13.411	15.088	18	-7.6	13.072	15.580
	19	-5.9	26.518	28.595	19	-7.6	2.920	4.193
	20	-5.8	27.109	29.973	20	-7.6	2.986	5.980
**50x50x50**	1	-8.6	0.000	0.000	1	-8.8	0.000	0.000
	2	-8.2	1.518	4.104	2	-8.8	0.046	1.844
	3	-8.2	1.524	4.139	3	-8.7	2.114	4.084
	4	-7.6	19.742	21.968	4	-8.6	2.924	6.443
	5	-7.6	19.746	21.932	5	-8.5	2.931	6.473
	6	-7.3	18.144	20.580	6	-8.5	2.503	5.539
	7	-7.2	1.964	4.280	7	-8.4	1.771	3.349
	8	-7.2	3.071	5.725	8	-8.3	2.400	5.948
	9	-7.2	19.559	21.879	9	-8.3	2.232	4.790
	10	-7.2	3.076	5.605	10	-8.2	2.031	3.773
	11	-7.2	19.898	22.194	11	-8.1	2.047	4.498
	12	-7.1	18.363	20.453	12	-8.1	2.044	4.783
	13	-7.0	2.666	4.675	13	-8.1	1.860	3.144
	14	-7.0	20.921	23.721	14	-8.0	2.424	4.936
	15	-7.0	19.801	22.380	15	-7.9	1.809	2.983
	16	-6.9	18.503	20.664	16	-7.8	2.397	3.422
	17	-6.8	19.451	22.082	17	-7.7	2.004	3.633
	18	-6.8	20.659	23.779	18	-7.6	2.417	6.011
	19	-6.8	36.776	40.124	19	-7.6	2.104	4.733
	20	-6.7	19.567	21.935	20	-7.6	1.778	3.017

**60x60x60**	1	-7.1	0.000	0.000	1	-8.8	0.000	0.000
	2	-6.9	2.759	5.635	2	-8.8	0.048	1.844
	3	-6.8	0.855	2.034	3	-8.7	1.879	4.350
	4	-6.6	2.926	6.346	4	-8.7	1.869	4.513
	5	-6.6	2.473	6.256	5	-8.6	1.894	4.931
	6	-6.6	2.479	6.297	6	-8.6	1.895	4.826
	7	-6.5	2.621	6.470	7	-8.5	2.900	6.509
	8	-6.4	2.163	4.738	8	-8.4	2.480	5.382
	9	-6.4	32.444	35.425	9	-8.4	2.054	5.816
	10	-6.4	2.776	6.453	10	-8.3	2.044	5.692
	11	-6.3	2.645	6.599	11	-8.3	2.763	5.686
	12	-6.2	2.876	6.081	12	-8.2	2.385	4.912
	13	-6.2	2.485	4.692	13	-8.1	1.652	2.727
	14	-6.2	34.663	38.355	14	-8.1	2.855	6.388
	15	-6.2	34.905	38.021	15	-8.1	1.988	4.024
	16	-6.2	32.346	35.092	16	-8.1	2.197	5.311
	17	-6.2	40.318	43.558	17	-8.0	2.239	4.823
	18	-6.1	2.200	6.189	18	-8.0	1.762	2.905
	19	-5.9	4.112	6.235	19	-7.9	2.463	5.265
	20	-5.9	32.426	35.685	20	-7.7	1.559	3.754
**70x70x70**	1	-10.3	0.000	0.000	1	-8.7	0.000	0.000
	2	-9.4	1.764	4.539	2	-8.5	2.501	5.570
	3	-9.3	1.753	4.405	3	-8.5	2.889	6.401
	4	-8.9	2.090	4.976	4	-8.4	1.721	2.888
	5	-8.8	2.052	4.785	5	-8.4	2.008	5.592
	6	-8.4	7.327	10.876	6	-8.4	2.513	5.293
	7	-7.4	7.447	10.795	7	-8.3	2.439	6.059
	8	-7.4	19.639	22.038	8	-8.1	2.841	5.773
	9	-7.2	19.832	21.578	9	-8.1	2.215	4.939
	10	-7.1	20.115	22.586	10	-8.1	1.629	2.908
	11	-7.0	20.011	21.958	11	-8.1	1.809	3.116
	12	-7.0	20.274	22.212	12	-8.0	2.493	6.275
	13	-6.9	30.334	33.790	13	-8.0	1.874	3.958
	14	-6.9	20.283	22.687	14	-7.9	1.627	2.428
	15	-6.8	20.823	22.721	15	-7.7	1.622	2.637
	16	-6.7	20.855	23.309	16	-7.7	1.917	5.532
	17	-6.7	24.433	27.572	17	-7.6	13.873	16.180
	18	-6.7	18.284	21.121	18	-7.6	13.821	16.168
	19	-6.6	20.113	22.590	19	-7.6	1.920	2.783
	20	-6.5	20.315	22.944	20	-7.3	2.766	5.083
	**Caffeine**							
	**1W6R**				**4BDS**			
**Size (Å³)**	mode |	affinity | (kcal/mol)	dist from best mode | rmsd l.b.	rmsd u.b.	mode |	affinity | (kcal/mol)	dist from best mode | rmsd l.b.	rmsd u.b.
**10x10x10**	1	18.1	0.000	0.000	1	-6.5	0.000	0.000
	2	19.2	1.442	3.019	2	-6.2	1.423	3.691
					3	-6.1	1.487	3.087

					4	-6.0	1.703	4.808
					5	-6.0	1.655	3.692
					6	-6.0	1.625	3.090
					7	-6.0	1.651	4.198
					8	-5.9	2.802	3.758
					9	-5.8	1.283	3.476
					10	-5.8	5.535	6.056
					11	-5.8	1.859	4.435
					12	-5.7	1.895	3.860
					13	-5.7	3.023	4.572
					14	-5.6	2.653	4.881
					15	-5.6	2.099	3.782
					16	-5.6	2.550	3.360
					17	-5.5	2.765	3.960
					18	-5.5	6.020	7.219
					19	-5.4	2.842	3.356
**15x15x15**	1	-3.6	0.000	0.000	1	-6.5	0.000	0.000
	2	-3.5	1.626	3.704	2	-6.2	1.448	3.667
	3	-2.7	2.354	4.969	3	-6.1	1.481	3.087
	4	-2.4	1.432	2.987	4	-6.0	1.786	3.725
	5	-2.4	2.366	4.512	5	-6.0	1.728	2.863
	6	-1.8	1.784	2.348	6	-6.0	1.662	4.761
	7	-1.8	1.776	4.705	7	-5.9	1.842	4.213
	8	-1.6	2.293	3.533	8	-5.9	1.378	3.919
	9	-1.3	1.565	3.788	9	-5.9	2.804	3.749
	10	-0.8	2.254	4.372	10	-5.8	1.848	4.449
	11	0.6	2.937	5.089	11	-5.8	2.386	4.305
					12	-5.8	5.908	6.972
					13	-5.7	6.232	6.647
					14	-5.7	2.736	5.105
					15	-5.6	5.993	7.093
					16	-5.6	5.795	6.330
					17	-5.6	2.796	4.421
					18	-5.5	6.054	7.249
					19	-5.5	2.214	3.803
					20	-5.5	2.869	3.732
**20x20x20**	1	-6.7	0.000	0.000	1	-6.5	0.000	0.000
	2	-6.7	2.406	3.989	2	-6.2	1.455	3.681
	3	-6.6	2.978	4.159	3	-6.1	1.482	3.088
	4	-6.5	2.517	3.463	4	-6.1	1.671	3.679
	5	-6.4	1.704	3.683	5	-6.0	1.733	2.891
	6	-6.3	2.375	4.167	6	-6.0	1.670	4.759
	7	-6.3	1.561	3.060	7	-6.0	1.096	3.489
	8	-6.3	2.055	2.696	8	-5.9	1.649	4.268
	9	-6.2	1.988	3.808	9	-5.8	2.400	4.318
	10	-6.2	1.642	4.228	10	-5.8	5.509	6.045
	11	-6.2	3.352	4.826	11	-5.7	2.736	5.098

	12	-6.2	2.581	4.901	12	-5.6	5.835	6.363
	13	-6.1	3.371	4.255	13	-5.6	5.995	7.094
	14	-6.1	1.824	4.023	14	-5.5	5.761	6.606
	15	-6.1	1.083	3.538	15	-5.5	2.599	3.500
	16	-6.0	2.817	4.118	16	-5.5	2.382	2.545
	17	-5.9	2.076	3.941	17	-5.5	2.872	3.735
					18	-5.4	2.448	3.947
					19	-5.4	3.458	4.661
					20	-5.4	2.842	3.347
**25x25x25**	1	-6.7	0.000	0.000	1	-6.5	0.000	0.000
	2	-6.7	2.424	4.009	2	-6.2	1.425	3.684
	3	-6.6	3.037	4.176	3	-6.1	1.714	4.393
	4	-6.6	1.317	3.567	4	-6.1	1.487	3.089
	5	-6.6	1.928	4.214	5	-6.1	1.709	3.690
	6	-6.5	1.792	4.051	6	-6.1	1.691	3.400
	7	-6.5	1.779	3.708	7	-6.0	1.707	4.817
	8	-6.4	2.668	3.673	8	-5.9	1.766	3.516
	9	-6.4	2.678	4.955	9	-5.9	1.638	4.227
	10	-6.4	2.373	2.984	10	-5.9	2.816	3.736
	11	-6.3	2.363	4.171	11	-5.8	1.467	2.217
	12	-6.3	1.538	3.051	12	-5.8	2.400	4.322
	13	-6.2	2.390	4.673	13	-5.8	5.863	6.942
	14	-6.2	3.413	4.806	14	-5.7	6.242	6.659
	15	-6.2	2.190	4.061	15	-5.7	1.593	3.742
	16	-6.2	3.375	4.241	16	-5.7	2.902	4.460
	17	-6.2	2.367	4.917	17	-5.7	2.764	5.123
	18	-6.1	1.811	4.014	18	-5.6	5.998	7.097
	19	-5.8	2.989	3.228	19	-5.6	2.547	3.357
	20	-5.7	1.929	3.179	20	-5.6	6.087	7.291
**30x30x30**	1	-6.7	0.000	0.000	1	-6.5	0.000	0.000
	2	-6.7	2.404	3.990	2	-6.2	1.423	3.683
	3	-6.6	3.297	4.306	3	-6.1	1.697	4.387
	4	-6.6	1.331	3.579	4	-6.1	1.488	3.093
	5	-6.6	1.873	4.199	5	-6.0	1.790	3.726
	6	-6.5	1.786	4.040	6	-5.9	1.926	4.228
	7	-6.5	1.765	3.706	7	-5.9	2.821	3.731
	8	-6.5	2.522	3.473	8	-5.8	16.350	17.023
	9	-6.4	2.665	4.936	9	-5.8	1.906	3.872
	10	-6.4	2.423	3.005	10	-5.8	5.866	6.945
	11	-6.4	3.305	4.706	11	-5.7	16.365	16.919
	12	-6.3	2.376	4.168	12	-5.7	6.234	6.649
	13	-6.2	2.375	4.691	13	-5.7	3.114	4.645
	14	-6.2	3.356	4.803	14	-5.6	5.827	6.354
	15	-6.2	2.422	4.851	15	-5.6	16.546	16.962
	16	-6.2	1.703	4.624	16	-5.6	16.414	17.094
	17	-6.0	2.643	4.047	17	-5.5	16.412	17.130

	18	-6.0	2.028	3.651	18	-5.5	16.604	17.388
	19	-5.9	2.035	3.404	19	-5.4	3.182	4.342
	20	-5.8	3.377	5.108	20	-5.4	16.558	17.307
**40x40x40**	1	-6.7	0.000	0.000	1	-6.5	0.000	0.000
	2	-6.7	2.424	4.011	2	-6.2	1.417	3.685
	3	-6.7	3.266	4.289	3	-6.1	1.487	3.087
	4	-6.6	1.913	4.202	4	-5.9	16.309	16.996
	5	-6.6	1.325	3.577	5	-5.9	1.921	3.415
	6	-6.5	1.771	4.018	6	-5.8	2.810	3.666
	7	-6.5	2.503	3.477	7	-5.8	16.491	17.040
	8	-6.4	2.675	4.949	8	-5.8	2.387	4.274
	9	-6.4	3.336	4.756	9	-5.8	5.848	6.930
	10	-6.2	2.403	3.805	10	-5.7	6.229	6.645
	11	-6.1	3.378	4.259	11	-5.6	16.604	17.252
	12	-6.0	2.683	4.113	12	-5.6	17.703	18.303
	13	-6.0	2.032	3.319	13	-5.6	16.830	17.766
	14	-5.9	2.091	4.894	14	-5.5	16.373	17.093
	15	-5.7	7.578	9.692	15	-5.5	21.475	22.233
	16	-5.7	19.128	20.466	16	-5.5	5.710	6.550
	17	-5.5	18.827	20.013	17	-5.5	5.630	6.092
	18	-5.4	17.338	17.926	18	-5.4	2.870	3.983
	19	-5.4	17.429	17.897	19	-5.4	3.157	4.077
	20	-5.3	17.256	17.982	20	-5.3	16.660	17.454
**50x50x50**	1	-6.7	0.000	0.000	1	-6.5	0.000	0.000
	2	-6.7	2.411	3.992	2	-6.2	1.440	3.680
	3	-6.6	1.910	4.205	3	-6.1	1.486	3.092
	4	-6.4	2.672	4.945	4	-6.0	1.672	4.769
	5	-6.3	1.543	3.052	5	-5.9	2.813	3.724
	6	-6.2	3.031	3.477	6	-5.9	16.346	17.015
	7	-6.1	2.465	3.894	7	-5.8	16.379	16.933
	8	-6.0	1.768	4.702	8	-5.6	16.406	17.084
	9	-5.8	7.435	9.464	9	-5.5	21.174	22.009
	10	-5.7	7.772	9.559	10	-5.5	17.654	18.130
	11	-5.7	7.587	9.699	11	-5.5	21.206	21.875
	12	-5.7	7.629	9.561	12	-5.4	17.418	18.073
	13	-5.7	19.130	20.483	13	-5.4	17.727	18.256
	14	-5.4	18.831	19.932	14	-5.4	21.238	21.822
	15	-5.4	17.329	17.947	15	-5.3	17.336	18.012
	16	-5.4	18.848	20.015	16	-5.2	4.968	5.555
	17	-5.3	19.052	20.177	17	-5.1	18.342	18.892
	18	-5.3	17.265	18.335	18	-5.0	2.307	4.009
	19	-5.3	8.241	9.847	19	-4.9	4.028	5.249
	20	-5.2	18.924	20.281	20	-4.9	17.167	17.684
**60x60x60**	1	-6.8	0.000	0.000	1	-6.5	0.000	0.000
	2	-6.6	2.997	4.179	2	-6.2	1.413	3.675
	3	-6.6	1.897	4.200	3	-6.1	1.481	3.088
	4	-6.6	1.328	3.578	4	-6.0	1.792	3.726

	5	-6.6	3.432	3.788	5	-6.0	1.730	2.876
	6	-6.5	1.778	4.029	6	-6.0	1.671	4.754
	7	-6.4	2.663	4.937	7	-6.0	1.143	3.511
	8	-6.2	1.867	3.648	8	-5.9	16.336	17.002
	9	-6.0	1.986	2.791	9	-5.9	2.811	3.729
	10	-5.8	7.425	9.453	10	-5.9	1.944	3.431
	11	-5.7	7.570	9.683	11	-5.8	2.403	4.319
	12	-5.7	7.767	9.553	12	-5.7	5.867	6.945
	13	-5.7	7.703	9.633	13	-5.7	16.362	16.916
	14	-5.7	19.138	20.478	14	-5.6	6.027	7.138
	15	-5.5	18.829	20.059	15	-5.6	2.803	4.399
	16	-5.5	7.454	9.046	16	-5.6	16.773	17.436
	17	-5.4	18.818	20.142	17	-5.6	6.103	7.291
	18	-5.4	18.849	20.024	18	-5.5	5.599	6.079
	19	-5.3	17.226	18.337	19	-5.5	16.187	16.844
	20	-5.2	7.618	9.089	20	-5.5	21.171	22.006
**70x70x70**	1	-6.7	0.000	0.000	1	-6.5	0.000	0.000
	2	-6.7	2.436	4.019	2	-6.2	1.431	3.687
	3	-6.6	1.914	4.203	3	-6.1	1.485	3.088
	4	-6.5	3.401	3.788	4	-6.0	1.677	4.760
	5	-6.5	1.777	3.708	5	-5.9	1.967	4.201
	6	-6.4	2.644	4.900	6	-5.9	2.814	3.737
	7	-5.7	7.579	9.693	7	-5.9	16.318	17.004
	8	-5.7	7.669	9.602	8	-5.9	1.137	3.433
	9	-5.7	19.131	20.468	9	-5.8	2.397	4.317
	10	-5.5	18.808	19.947	10	-5.8	1.852	4.419
	11	-5.4	7.455	9.046	11	-5.8	5.857	6.935
	12	-5.4	17.328	17.940	12	-5.7	16.298	16.847
	13	-5.4	19.003	19.842	13	-5.6	6.018	7.100
	14	-5.4	18.841	19.841	14	-5.6	16.429	17.105
	15	-5.4	18.829	20.143	15	-5.5	5.477	5.969
	16	-5.4	8.046	9.422	16	-5.5	24.857	26.602
	17	-5.3	7.789	9.439	17	-5.5	21.481	22.241
	18	-5.2	7.474	9.437	18	-5.4	21.187	21.779
	19	-5.2	7.985	9.359	19	-5.4	3.339	4.529
	20	-5.1	7.246	9.048	20	-5.1	25.431	27.134
	**Avarol**							
	**1W6R**				**4BDS**			
	mode	affinity (kcal/mol)	dist from best mode rmsd l.b.	rmsd u.b.	mode	affinity (kcal/mol)	dist from best mode rmsd l.b.	rmsd u.b.
**10x10x10**	1	57.5	0.000	0.000	1	-8.5	0.000	0.000
					2	-8.4	3.147	5.359
					3	-8.3	1.429	2.986
					4	-8.2	2.768	5.110
					5	-8.2	1.299	2.209
					6	-8.1	1.625	2.404
					7	-8.1	2.978	6.040

					8	-7.9	2.595	5.230
					9	-7.9	3.049	5.841
					10	-7.8	1.537	3.539
					11	-7.6	1.433	3.346
					12	-7.4	2.790	5.880
					13	-7.4	1.429	2.897
					14	-7.2	3.127	5.666
					15	-7.1	1.790	2.986
					16	-7.1	3.406	5.624
					17	-7.1	3.029	5.653
					18	-6.8	1.818	3.828
					19	-6.7	2.499	5.168
**15x15x15**	1	6.7	0.000	0.000	1	-9.7	0.000	0.000
	2	8.4	1.228	1.967	2	-9.6	1.456	3.321
	3	11.5	2.531	6.256	3	-9.3	2.403	4.665
					4	-9.3	2.579	6.366
					5	-9.1	2.461	6.557
					6	-9.1	3.015	5.080
					7	-9.0	2.883	4.964
					8	-9.0	2.509	4.498
					9	-9.0	1.599	3.037
					10	-9.0	2.264	4.513
					11	-8.9	3.266	5.041
					12	-8.9	2.909	5.314
					13	-8.8	2.493	4.710
					14	-8.8	1.869	2.919
					15	-8.7	3.269	6.455
					16	-8.5	2.102	4.201
					17	-8.5	2.597	5.066
					18	-8.5	3.011	6.023
					19	-8.5	1.947	3.598
					20	-8.3	2.431	5.208
**20x20x20**	1	-6.7	0.000	0.000	1	-9.7	0.000	0.000
	2	-6.1	1.841	3.813	2	-9.5	1.484	3.328
	3	-6.0	1.930	4.476	3	-9.2	2.367	4.600
	4	-5.9	2.010	5.292	4	-9.2	1.762	3.738
	5	-5.4	1.311	4.173	5	-9.1	2.449	6.564
	6	-4.3	1.072	2.736	6	-9.1	2.970	4.993
	7	-4.2	2.506	4.321	7	-9.0	1.862	3.131
	8	-4.2	2.052	4.214	8	-9.0	2.781	6.795
	9	-3.7	1.411	2.731	9	-9.0	2.871	4.924
	10	-3.6	1.330	2.465	10	-8.9	2.979	5.344
	11	-3.4	1.581	3.888	11	-8.9	3.258	5.037
	12	-2.4	2.592	4.748	12	-8.8	2.459	4.582
	13	-2.1	2.354	5.810	13	-8.7	3.197	6.363
	14	-2.0	2.470	5.034	14	-8.6	1.916	2.994
	15	-1.8	2.072	5.168	15	-8.5	1.618	2.592

	16	-1.7	2.548	5.301	16	-8.4	2.573	5.115
					17	-8.4	3.276	5.852
					18	-8.4	2.948	6.129
					19	-8.3	2.609	3.764
					20	-8.3	1.831	3.815
**25x25x25**	1	-8.1	0.000	0.000	1	-9.8	0.000	0.000
	2	-7.7	1.654	4.795	2	-9.6	1.459	3.332
	3	-7.4	1.974	3.746	3	-9.4	1.879	3.256
	4	-7.3	2.118	5.213	4	-9.2	2.309	4.498
	5	-7.1	2.213	5.259	5	-9.2	2.562	6.344
	6	-7.0	2.075	5.485	6	-9.1	3.175	5.214
	7	-6.5	2.145	5.153	7	-9.1	2.438	6.545
	8	-6.2	2.270	5.318	8	-9.0	1.738	3.157
	9	-6.2	2.463	5.572	9	-9.0	1.861	3.124
	10	-6.0	1.998	4.193	10	-9.0	2.905	4.971
	11	-5.9	2.408	5.752	11	-8.9	2.188	4.324
	12	-5.9	1.946	5.185	12	-8.9	1.618	3.246
	13	-5.7	1.617	3.876	13	-8.9	3.285	5.072
	14	-5.5	2.023	2.756	14	-8.8	2.456	4.605
	15	-5.5	2.343	5.178	15	-8.7	2.446	5.033
	16	-5.0	2.062	4.902	16	-8.7	1.957	2.983
	17	-4.7	2.592	5.205	17	-8.6	2.546	4.680
	18	-4.7	2.768	5.117	18	-8.6	3.160	6.323
	19	-4.1	2.412	5.477	19	-8.5	2.406	5.488
					20	-8.4	2.814	5.468
**30x30x30**	1	-8.1	0.000	0.000	1	-9.7	0.000	0.000
	2	-7.8	1.712	4.832	2	-9.6	1.425	3.368
	3	-7.3	1.958	3.745	3	-9.3	2.407	4.659
	4	-7.1	2.199	5.247	4	-9.2	2.562	6.334
	5	-7.1	2.030	5.134	5	-9.1	2.985	5.042
	6	-7.0	2.055	5.481	6	-9.0	2.919	4.973
	7	-6.9	2.404	5.460	7	-9.0	2.002	3.275
	8	-6.7	1.012	1.149	8	-8.9	2.875	6.852
	9	-6.7	1.340	2.985	9	-8.9	1.661	3.401
	10	-6.7	2.094	3.070	10	-8.9	3.266	5.047
	11	-6.4	2.126	5.088	11	-8.9	2.329	4.865
	12	-6.2	2.189	5.847	12	-8.9	2.403	4.806
	13	-6.1	2.393	6.063	13	-8.9	2.653	6.489
	14	-6.0	1.969	4.200	14	-8.8	2.611	4.644
	15	-5.9	1.510	3.773	15	-8.6	1.443	2.953
	16	-5.1	2.271	5.336	16	-8.6	3.384	5.847
	17	-5.1	2.211	3.638	17	-8.5	2.947	5.992
	18	-4.7	2.147	5.809	18	-8.5	2.067	3.185
	19	-4.7	2.264	5.009	19	-8.4	2.664	4.986
	20	-4.6	1.966	3.752	20	-8.3	1.738	3.444
**40x40x40**	1	-8.1	0.000	0.000	1	-9.8	0.000	0.000
	2	-7.9	19.674	22.057	2	-9.7	1.460	3.329

	3	-7.9	19.827	22.213	3	-9.2	2.304	4.502
	4	-7.8	22.320	24.665	4	-9.1	3.214	5.236
	5	-7.7	19.345	21.886	5	-9.0	2.428	6.547
	6	-7.5	32.414	33.994	6	-9.0	2.914	4.984
	7	-7.4	21.500	23.742	7	-9.0	1.694	3.106
	8	-7.4	20.691	23.069	8	-9.0	1.615	3.193
	9	-7.3	32.303	33.755	9	-8.9	2.651	6.408
	10	-7.3	32.801	34.375	10	-8.9	2.192	4.323
	11	-7.2	1.498	2.780	11	-8.9	2.940	5.298
	12	-7.2	32.346	33.912	12	-8.9	3.275	5.074
	13	-7.1	23.669	25.868	13	-8.8	2.657	6.521
	14	-6.9	31.625	33.091	14	-8.7	2.497	4.803
	15	-6.9	32.663	34.093	15	-8.4	2.939	6.146
	16	-6.9	13.022	14.481	16	-8.4	1.886	3.608
	17	-6.9	1.737	3.511	17	-8.3	3.042	5.889
	18	-6.8	31.965	33.573	18	-8.3	2.414	4.735
	19	-6.8	1.702	3.267	19	-8.2	19.493	21.156
	20	-6.7	32.513	34.056	20	-8.2	3.100	6.159
**50x50x50**	1	-7.8	0.000	0.000	1	-9.8	0.000	0.000
	2	-7.8	2.358	4.529	2	-9.6	1.493	3.355
	3	-7.7	3.082	5.847	3	-9.1	2.994	5.008
	4	-7.6	19.803	21.968	4	-9.0	2.782	6.797
	5	-7.5	1.718	2.264	5	-9.0	2.899	4.925
	6	-7.5	1.494	3.184	6	-9.0	1.697	3.107
	7	-7.2	2.477	5.569	7	-9.0	2.433	6.580
	8	-7.2	7.670	10.686	8	-8.9	2.324	4.782
	9	-7.1	20.656	22.811	9	-8.9	3.263	5.063
	10	-7.0	2.976	6.017	10	-8.7	2.628	6.676
	11	-6.9	2.749	6.075	11	-8.5	3.341	5.847
	12	-6.8	2.868	5.248	12	-8.4	2.881	5.177
	13	-6.7	18.918	22.391	13	-8.4	1.861	3.857
	14	-6.7	19.592	21.309	14	-8.4	14.404	16.534
	15	-6.6	20.492	22.310	15	-8.2	2.370	4.899
	16	-6.5	1.998	3.448	16	-8.2	2.994	6.085
	17	-6.4	2.369	5.424	17	-8.2	19.496	21.170
	18	-6.3	20.303	22.410	18	-8.2	2.762	5.481
	19	-6.3	36.026	38.288	19	-8.0	14.357	17.161
	20	-6.2	35.952	38.264	20	-7.8	14.421	17.365
**60x60x60**	1	-7.8	0.000	0.000	1	-9.7	0.000	0.000
	2	-7.6	3.101	5.895	2	-9.5	1.527	3.340
	3	-7.6	19.879	22.041	3	-9.2	3.318	5.288
	4	-7.5	1.694	2.243	4	-9.1	2.185	4.364
	5	-7.3	2.333	5.254	5	-9.1	2.447	6.567
	6	-7.3	1.676	2.817	6	-9.0	2.869	4.936
	7	-7.2	2.923	6.086	7	-9.0	2.499	4.474
	8	-7.1	20.730	22.889	8	-8.9	2.995	5.336
	9	-7.0	2.017	4.356	9	-8.9	3.252	5.059

	10	-7.0	2.927	5.954	10	-8.7	1.905	3.015
	11	-7.0	1.497	3.214	11	-8.7	1.877	3.771
	12	-6.9	2.317	5.578	12	-8.7	3.245	6.397
	13	-6.8	20.260	22.111	13	-8.7	2.140	4.227
	14	-6.7	20.211	22.654	14	-8.5	1.970	3.737
	15	-6.7	20.815	23.235	15	-8.4	3.277	5.874
	16	-6.6	20.134	22.194	16	-8.4	14.385	16.578
	17	-6.6	19.781	22.350	17	-8.4	1.699	3.094
	18	-6.6	19.785	21.799	18	-8.3	13.586	16.031
	19	-6.5	3.042	6.010	19	-8.2	2.853	6.003
	20	-6.3	19.719	21.884	20	-8.2	19.483	21.150
**70x70x70**	1	-7.9	0.000	0.000	1	-9.0	0.000	0.000
	2	-7.6	19.861	22.091	2	-8.7	2.161	4.482
	3	-7.1	2.899	5.935	3	-8.6	2.309	5.504
	4	-7.1	2.717	5.870	4	-8.6	3.436	5.652
	5	-7.1	20.407	22.846	5	-8.4	2.966	5.984
	6	-7.1	19.860	22.033	6	-8.4	3.056	4.607
	7	-6.8	1.504	3.711	7	-8.4	1.473	2.643
	8	-6.8	19.400	21.258	8	-8.3	1.903	3.117
	9	-6.8	3.208	6.198	9	-8.3	3.103	5.725
	10	-6.8	19.931	22.614	10	-8.2	3.232	5.544
	11	-6.7	19.784	22.403	11	-7.9	3.434	5.628
	12	-6.7	2.148	4.049	12	-7.9	1.721	3.009
	13	-6.6	20.496	22.319	13	-7.8	14.726	16.661
	14	-6.6	20.425	22.861	14	-7.7	2.782	6.055
	15	-6.6	2.574	5.812	15	-7.7	2.086	5.273
	16	-6.4	1.591	2.244	16	-7.5	15.098	17.580
	17	-6.3	2.762	5.466	17	-7.4	19.907	21.799
	18	-6.3	19.528	21.948	18	-7.4	2.520	4.356
	19	-6.3	20.398	22.866	19	-7.4	15.124	17.829
	20	-6.3	30.612	33.100	20	-7.3	3.109	6.117

Table S3. Specific interactions between AChE or BChE and the selected FDA-approved compounds or ZINC000253700110

Name	Structure	MW (g/mol)<br>Size: x,y,z (Å)<br>V(Å^3)	AChE<br>30x30x30 box	BChE<br>30x30x30 box
Ambrisentan	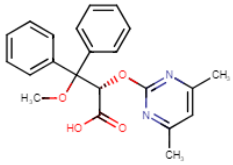	378.428<br>10.48, 8.4, 7.6<br>667.05	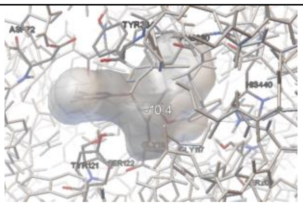	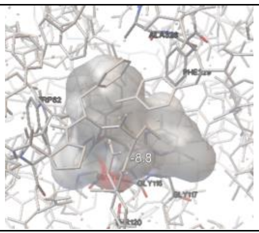

Methadone	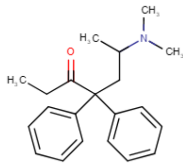	309.445<br>10.02,8.21,6.06<br>498.62	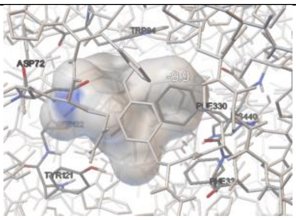	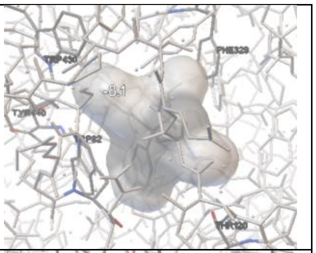
Triamterene	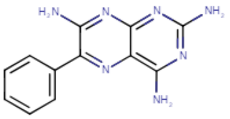	253.2626<br>12.01,7.14,4.32<br>370.221	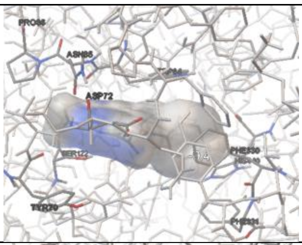	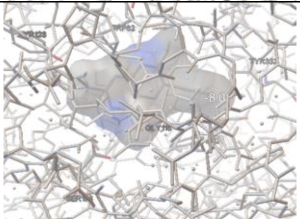
Prednisone	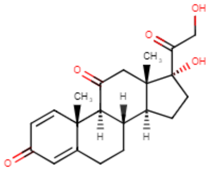	358.4281<br>13.11, 5.73,4.83<br>362.85	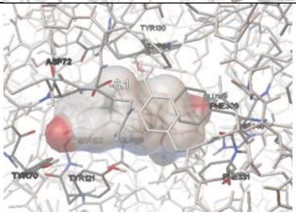	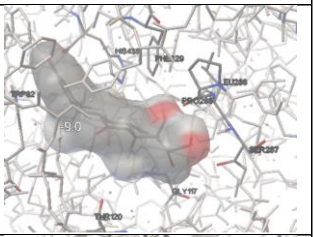
Metaxalone	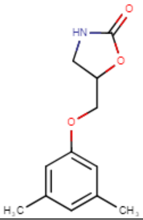	221.2524<br>9.165,6.34,4.10<br>238.14	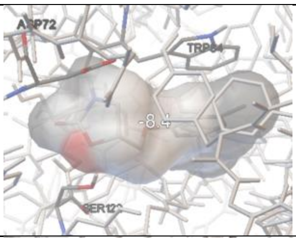	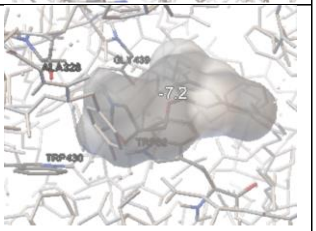
Doxapram	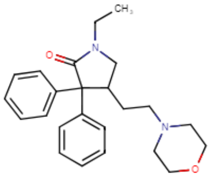	378.5072<br>12.03, 9.93,8.25<br>985.52	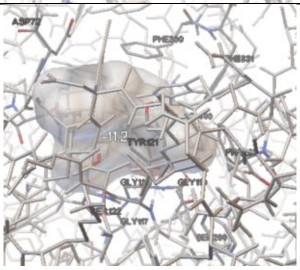	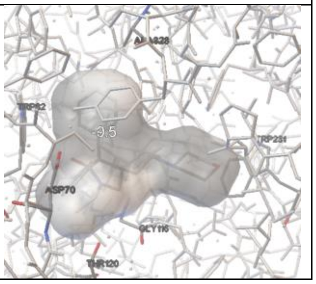

Name	Structure	MW (g/mol)<br>Size: x,y,z (Å)<br>V(Å^3)	AChE<br>30x30x30 box	BChE<br>30x30x30 box
Ergotamine	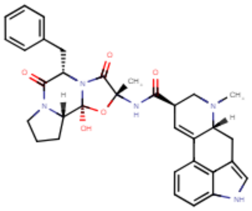	581.6615<br>18.93,10.12,6.75<br>1293.432	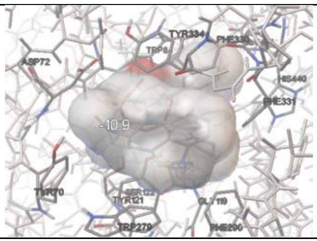	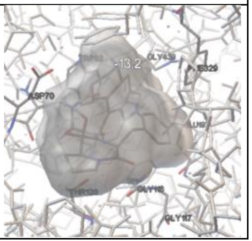

Ciclesonide	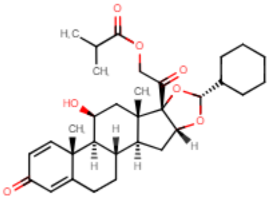	540.697<br>15.24, 11.42,7.05<br>1227.74	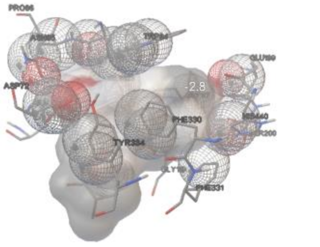	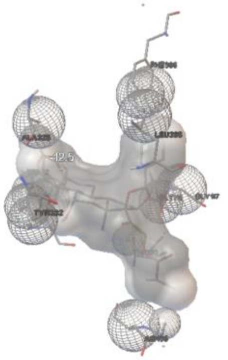
Suvorexant	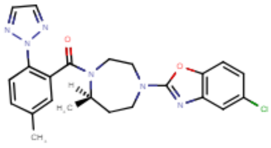	450.93<br>12.93,9.01,6.91<br>804.63	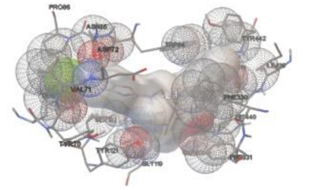	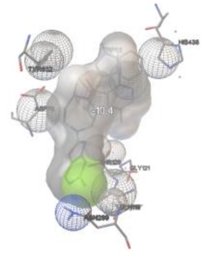
Nintedanib	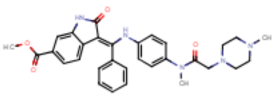	539.6248<br>22.38,8.26,6.695<br>1238.45	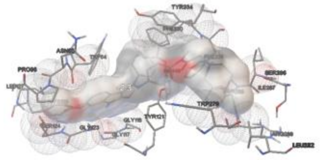	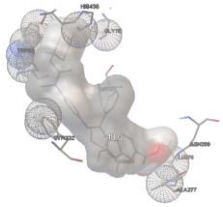
Lurasidone	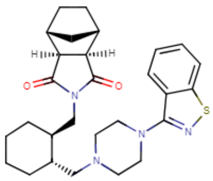	492.676<br>17.36,9.33,5.43<br>878.415	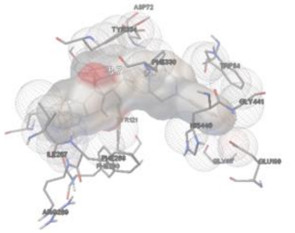	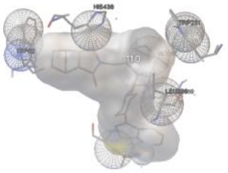
Amcinonide	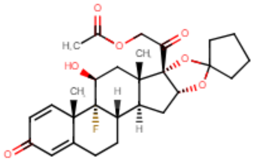	502.5717<br>13.85,8.52,6.20<br>731.48	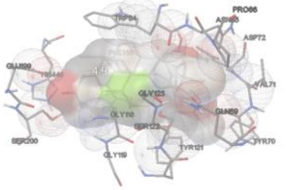	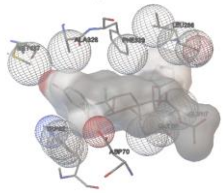
ZINC000253<br>700110	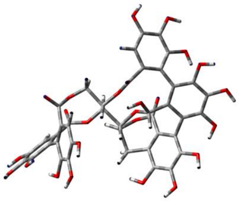	934.633<br>16.27,10.90,9.24<br>1638.8	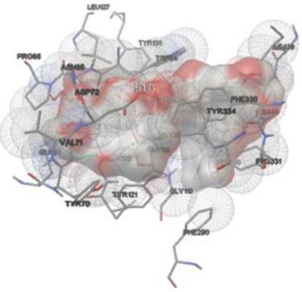	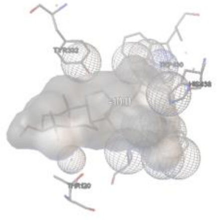

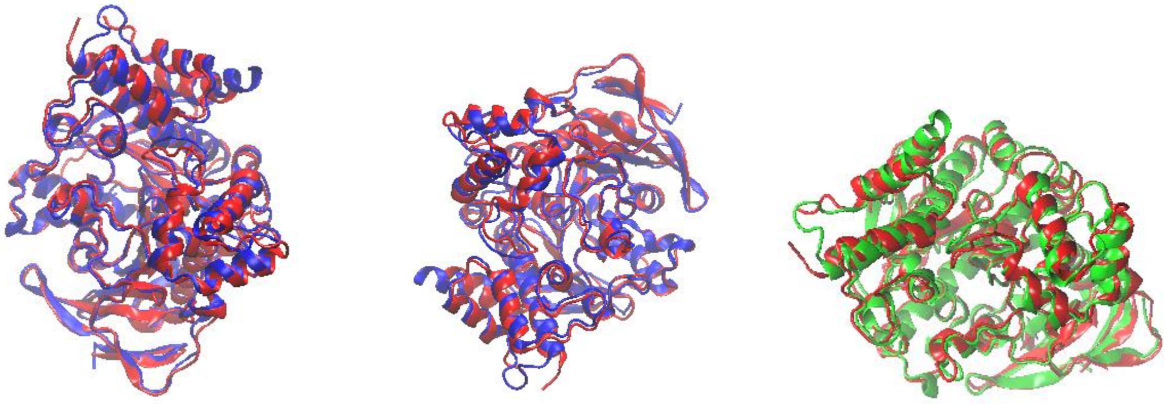

(a) 1W6R and 4BDS "MultiSep" alignment with VMD

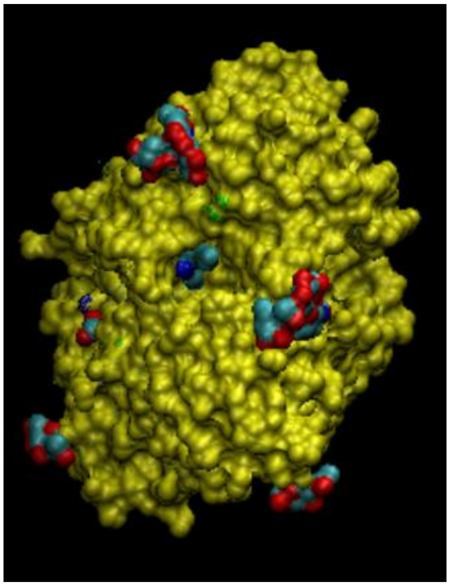

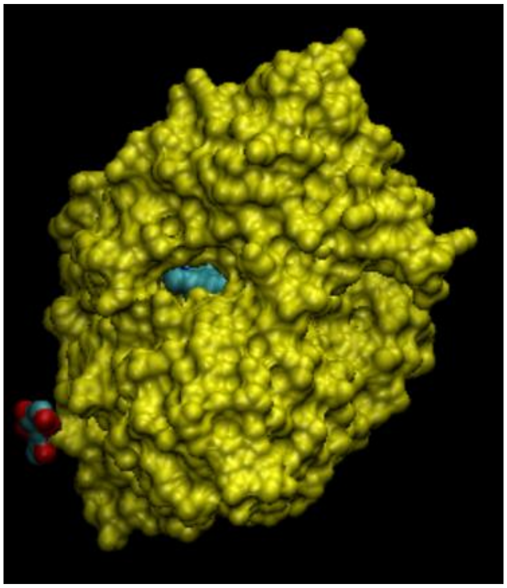

4BDS 1W6R

(b) the "Surf" representation and original ligands in Protein Crystals

Figure S1. (a) 1W6R and 4BDS "MultiSep" alignment with VMD; (b) the "Surf" representation with original ligands in Protein Crystals

S2

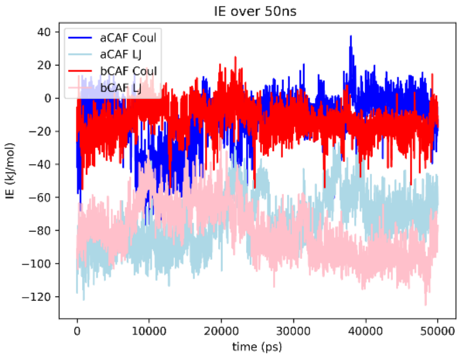

S3

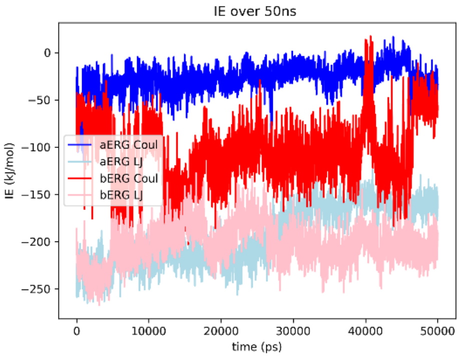

S4

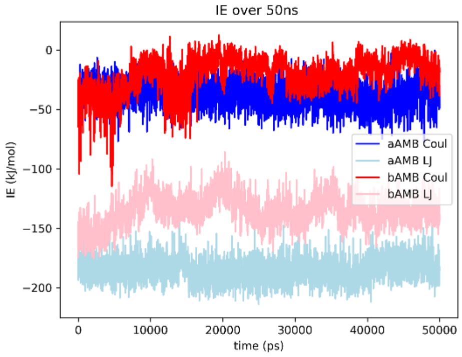

S5

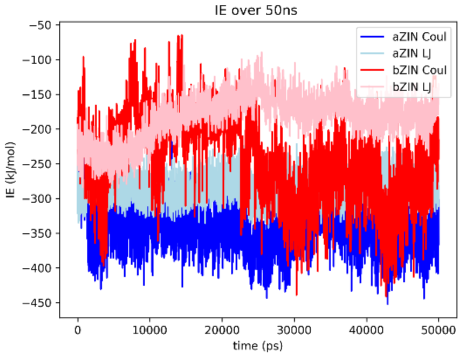

Figures S2-S5. Short-range intermolecular coulombic (blue, red) and Lennard-Jones (light blue, pink) interaction energy over 50 ns between atoms of ligands caffeine (**S2**), ergotamine (**S3**), ambrisentan (**S4**), and ZINC000253700110 (**S5**), and AChE (blue, light blue) and BChE (red, pink) active sites.

S6

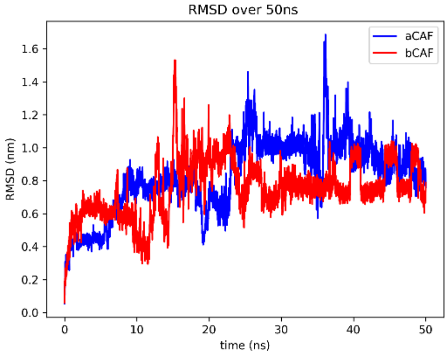

S7

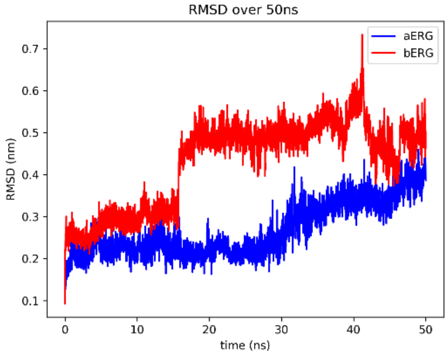

S8

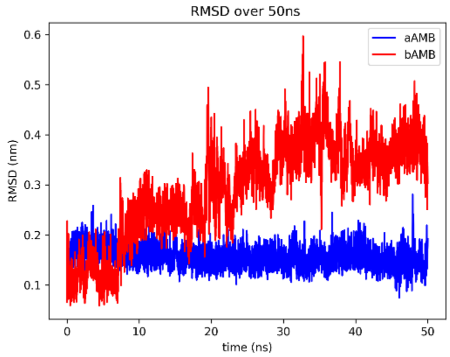

S9

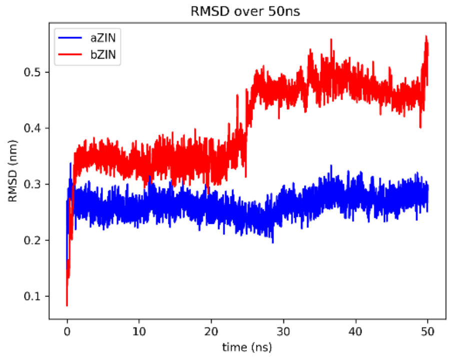

Figures S6-S9. RMSD over 50 ns of ligand heavy atoms with respect to the enzyme backbone of ligands caffeine (**S6**), ergotamine (**S7**), ambrisentan (**S8**), and ZINC000253700110 (**S9**). AChE-ligand complexes are in blue and BChE-ligand complexes are in red.

S10

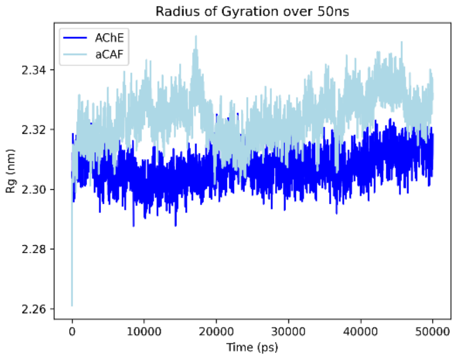

S11

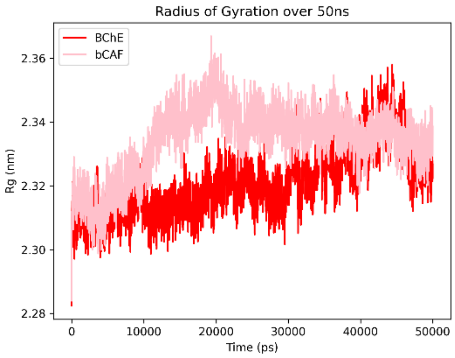

S12

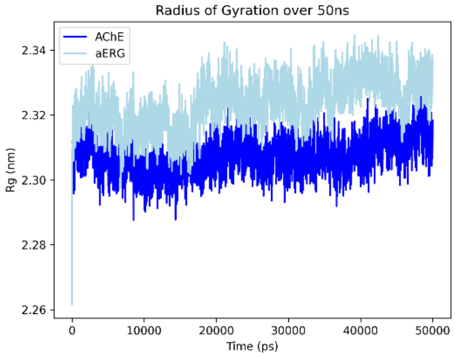

S13

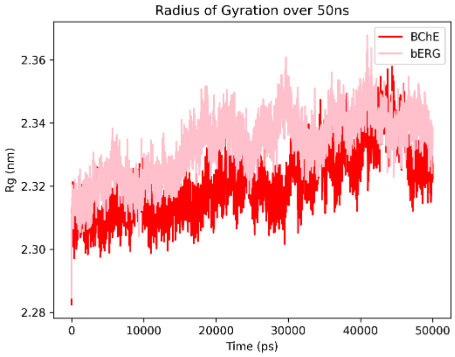

S14

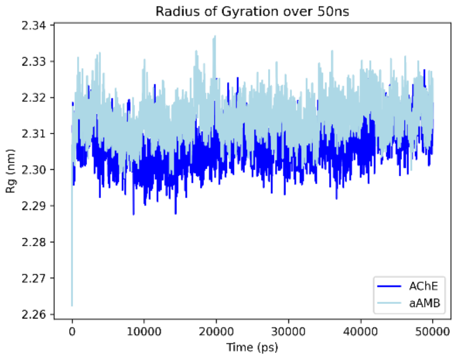

S15

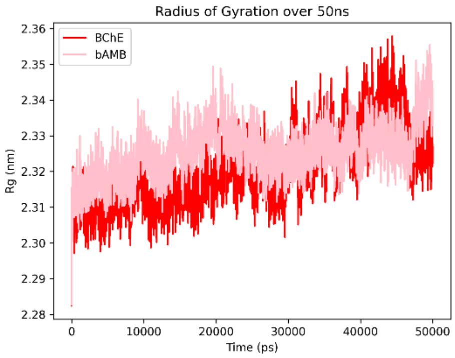

S16

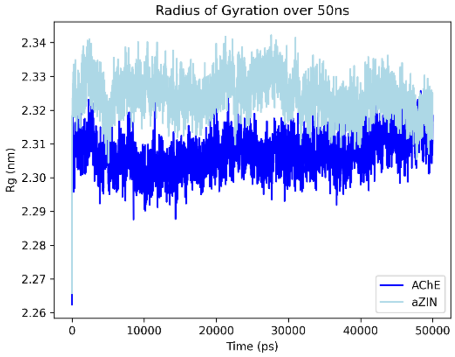

S17

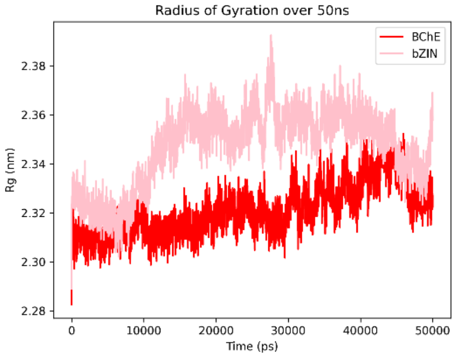

Figures S10-S17. Radius of gyration over 50 ns of protein for uninhibited AChE/BChE (blue, red) and AChE/BChE-ligand complexes (light blue, pink) for (**S10, S11**), ergotamine(**S12, S13**), ambrisentan (**S14, S15**), and ZINC000253700110 (**S16, S17**).

Table S4. AChE inhibitors from FDA-approved dataset (20 modes).

Ea diff.	Ea 1st	Ea mean	Ea SD	rmsd/l.b. mean	rmsd/l.b. SD	rmsd/u.b. mean	rmsd/u.b. SD	no. modes	no. rank	ZINC ID#
1.9	-10.8	-8.6	1.14	1.713	0.483	3.709	1.400	20	1	ZINC000000538627
1.8	-11.0	-8.6	1.23	1.642	0.304	4.355	2.029	5	2	ZINC000003875357
1.8	-9.8	-8.8	0.67	1.757	0.341	4.420	0.771	20	3	ZINC000001530707

1.8	-9.6	-8.2	0.63	1.685	0.309	4.417	0.850	20	4	ZINC000001530706
1.8	-10.4	-7.8	1.11	2.224	0.265	4.837	0.957	18	5	ZINC000000607939
1.8	-10.1	-7.7	0.81	2.219	0.392	5.110	1.559	20	6	ZINC000003843198
1.7	-10.6	-8.5	0.75	2.942	0.571	5.050	1.640	20	7	ZINC000000020253
1.6	-10.7	-9.2	0.61	1.463	0.173	4.420	0.300	20	8	ZINC000003807804
1.6	-10.7	-8.2	1.14	2.424	0.830	4.852	1.277	10	9	ZINC000006094354
1.6	-9.7	-7.8	0.66	2.145	0.381	4.848	1.033	20	10	ZINC000000000973
1.6	-8.8	-7.7	0.57	2.323	1.011	3.758	1.888	20	11	ZINC000000000471
1.6	-8.9	-7.5	0.48	1.981	0.423	4.189	1.003	20	12	ZINC000007997952
1.5	-10.7	-8.8	1.28	1.429	0.114	4.905	1.271	7	13	ZINC000253498282
1.5	-10.9	-8.9	1.28	1.683	0.482	4.294	1.188	9	14	ZINC000019632668
1.5	-8.7	-7.7	0.51	2.421	0.874	4.240	1.630	20	15	ZINC000012661824
1.4	-8.8	-6.7	1.04	2.322	0.410	4.629	1.440	20	16	ZINC000100001965
1.4	-7.1	-6.5	0.52	2.014	1.268	3.383	1.190	18	17	ZINC000000968256
1.4	-10.1	-7.7	1.34	2.623	1.117	4.730	1.959	16	18	ZINC000003875259

1.4	-8.9	-8.1	0.44	2.201	0.318	4.422	0.614	20	19	ZINC000000057253
1.4	-8.2	-7.2	0.72	1.982	0.747	3.589	0.709	20	20	ZINC000003812933
1.3	-9.4	-8.4	0.51	2.510	0.504	4.286	0.782	20	21	ZINC000000006156
1.3	-7.7	-7.1	0.54	1.763	0.746	3.178	0.734	20	22	ZINC000003831430
1.3	-8.7	-6.7	0.74	3.020	1.017	4.539	1.826	19	23	ZINC000000000850
1.3	-7.6	-6.6	0.41	2.305	0.511	3.983	0.789	20	24	ZINC000003830347
1.3	-8.9	-6.8	0.95	1.862	0.374	4.316	1.763	20	25	ZINC000100037890
1.3	-7.7	-6.9	0.39	1.808	0.336	3.996	0.968	20	26	ZINC000012503076
1.3	-9.5	-7.4	1.01	1.975	0.402	4.649	1.369	19	27	ZINC000000968336
1.3	-10.5	-8.3	1.12	2.798	0.522	4.993	1.068	18	28	ZINC000003813003
1.3	-10.5	-7.9	1.24	2.313	0.323	4.181	0.463	10	29	ZINC000003812983
1.3	-9.5	-7.6	0.78	2.617	0.478	4.583	1.051	20	30	ZINC000019362735
1.3	-8.3	-7.0	0.55	2.164	0.426	4.328	0.861	20	31	ZINC000000155531
1.3	-10.2	-8.4	0.94	2.145	0.351	3.971	0.483	14	32	ZINC000003812984
1.3	-8.7	-7.3	0.47	2.198	0.537	3.794	0.926	20	33	ZINC000000014257

1.3	-7.1	-6.5	0.34	1.610	0.810	2.714	0.887	19	34	ZINC000000967521
1.3	-8.1	-6.8	0.58	2.125	0.202	4.237	0.475	20	35	ZINC000001530930
1.2	-8.4	-7.8	0.27	2.277	0.479	4.215	0.663	20	36	ZINC000000968233
1.2	-8.6	-6.3	1.18	2.821	0.524	5.061	1.476	16	37	ZINC000000897229
1.2	-9.5	-8.1	0.65	3.437	1.426	4.820	2.116	20	38	ZINC000000004840
1.2	-9.2	-8.2	0.43	2.749	0.584	4.044	1.660	20	39	ZINC000002570817
1.2	-9.9	-8.6	0.71	2.146	0.619	4.584	1.521	20	40	ZINC000000000449
1.2	-8.4	-7.3	0.54	2.180	0.392	3.666	0.933	20	41	ZINC000036294079
1.2	-8.8	-6.6	0.97	2.104	0.391	4.522	0.662	16	42	ZINC000000020250
1.2	-9.2	-8.4	0.39	2.596	0.489	4.525	0.837	20	43	ZINC000000004009
1.2	-10.8	-8.8	0.94	2.938	0.723	4.685	1.172	19	44	ZINC000000001370
1.2	-9.9	-7.6	1.00	2.903	0.607	4.601	0.427	18	45	ZINC000000896595
1.2	-9.2	-8.2	0.57	2.149	0.654	4.421	1.482	20	46	ZINC000000896968
1.2	-8.6	-8.0	0.43	2.480	0.931	4.393	1.534	20	47	ZINC000001530635
1.2	-7.8	-7.1	0.52	1.867	0.888	3.449	0.783	19	48	ZINC000003831429

1.2	-6.9	-6.2	0.35	1.567	0.797	3.150	0.807	20	49	ZINC000000967566
1.2	-9.5	-8.2	0.73	2.836	0.497	4.863	1.184	20	50	ZINC000003800706
1.2	-9.1	-7.8	0.57	2.546	0.620	4.442	1.671	20	51	ZINC000000001984
1.2	-9.3	-8.5	0.58	1.804	0.303	4.322	1.244	20	52	ZINC000001530618
1.1	-8.4	-7.8	0.28	2.280	0.323	4.116	0.658	20	53	ZINC000000001728
1.1	-9.2	-8.2	0.48	2.281	0.505	4.413	1.501	20	54	ZINC000000584092
1.1	-8.9	-8.0	0.50	3.096	0.650	4.778	0.950	20	55	ZINC000000001281
1.1	-8.5	-7.9	0.39	2.389	0.580	4.034	1.380	20	56	ZINC000009224016
1.1	-8.5	-7.7	0.37	2.316	0.375	4.208	0.906	20	57	ZINC000000009342
1.1	-9.0	-7.9	0.66	1.792	0.446	3.884	1.755	19	58	ZINC000100027531
1.1	-8.9	-7.8	0.54	1.830	0.277	4.609	0.832	20	59	ZINC000001530769
1.1	-10.2	-7.7	1.11	2.061	0.419	3.856	0.564	13	60	ZINC000000402954
1.1	-8.5	-7.3	0.44	2.996	0.909	4.520	1.317	20	61	ZINC000012466082
1.1	-8.2	-7.3	0.38	2.500	0.622	4.261	0.863	20	62	ZINC000000001958
1.1	-8.4	-7.5	0.42	2.352	0.365	4.261	0.769	20	63	ZINC000000001681

1.1	-9.0	-8.3	0.31	3.170	0.727	5.022	1.234	20	64	ZINC000001530637
1.1	-7.9	-7.1	0.39	3.027	0.623	3.872	0.913	20	65	ZINC000003872277
1.1	-8.4	-7.3	0.43	2.452	0.499	4.676	0.713	20	66	ZINC000000113404
1.1	-9.4	-8.2	0.48	3.001	0.833	4.370	1.476	20	67	ZINC000000020243
1.1	-8.4	-7.6	0.35	2.255	0.794	3.539	1.612	20	68	ZINC000000897322
1.1	-9.0	-7.9	0.46	2.532	0.578	4.228	1.321	20	69	ZINC000000001655
1.1	-8.3	-7.6	0.41	2.018	0.578	4.410	1.849	20	70	ZINC000096014710
1.1	-7.5	-7.0	0.31	1.961	0.633	4.315	0.565	20	71	ZINC000001883067
1.1	-7.9	-6.8	0.62	2.645	1.277	4.684	2.148	20	72	ZINC000001542002
1.1	-8.9	-8.1	0.52	1.811	0.305	4.530	1.162	20	73	ZINC000001530617
1.1	-8.2	-6.9	0.48	2.919	0.721	4.287	1.062	20	74	ZINC000018203737
1.0	-8.4	-7.6	0.46	2.120	0.371	4.164	0.654	20	75	ZINC000002548959
1.0	-8.4	-7.5	0.40	2.494	0.410	4.503	0.950	20	76	ZINC000000001408
1.0	-8.3	-7.2	0.48	2.057	0.358	3.997	0.967	20	77	ZINC000000000853
1.0	-9.1	-6.7	0.91	2.123	0.256	4.688	0.741	14	78	ZINC000030726187

1.0	-10.1	-7.8	1.46	2.318	0.586	4.795	1.915	11	79	ZINC000004098633
1.0	-8.4	-7.3	0.62	2.240	0.352	4.392	1.048	20	80	ZINC000001530726
1.0	-8.6	-7.3	0.55	2.288	0.379	4.389	0.753	20	81	ZINC000002522648
1.0	-7.4	-6.5	0.46	2.070	0.409	4.213	1.515	20	82	ZINC000000001792
1.0	-6.7	-6.0	0.38	1.587	0.565	3.115	0.694	19	83	ZINC000043763856
1.0	-8.3	-7.0	0.45	2.575	0.604	4.425	1.388	20	84	ZINC000001530863
1.0	-8.7	-8.1	0.34	2.665	0.454	4.928	0.887	20	85	ZINC000001530575
1.0	-9.1	-7.0	0.95	2.091	0.238	4.402	1.030	19	86	ZINC000085205448
1.0	-8.2	-7.5	0.40	2.716	0.696	4.246	0.822	20	87	ZINC000000001979
1.0	-10.2	-8.2	1.44	2.061	0.353	3.735	0.856	9	88	ZINC000003875483
1.0	-9.0	-6.7	1.09	2.179	0.321	5.386	1.028	18	89	ZINC000019632713
1.0	-8.5	-7.6	0.54	2.041	0.561	4.646	0.734	20	90	ZINC000000020244
1.0	-8.2	-7.0	0.50	2.210	0.628	4.218	1.254	19	91	ZINC000000896455
1.0	-8.8	-7.7	0.53	2.285	0.527	4.498	1.563	20	92	ZINC000001543916
1.0	-7.6	-6.8	0.34	2.085	0.362	4.226	1.005	20	93	ZINC000000056427

1.0	-6.8	-6.4	0.29	1.560	0.592	2.690	0.877	19	94	ZINC000000967520
1.0	-7.9	-7.0	0.52	2.959	0.468	5.279	1.137	20	95	ZINC000019144231
1.0	-10.1	-8.6	0.74	2.574	0.869	4.585	1.331	20	96	ZINC000001530922
1.0	-7.4	-6.8	0.33	1.870	0.212	3.344	0.750	20	97	ZINC000000897244
1.0	-9.6	-7.7	0.79	2.331	0.452	4.371	1.394	20	98	ZINC000000120286
1.0	-9.8	-8.7	0.64	2.035	0.286	4.556	1.217	19	99	ZINC000100006264
1.0	-8.3	-7.5	0.47	2.316	0.536	4.621	0.921	20	100	ZINC000000001931
1.0	-7.7	-6.8	0.34	2.314	0.558	4.889	1.574	20	101	ZINC000001530567
1.0	-6.4	-5.7	0.29	2.492	0.636	3.736	0.914	20	102	ZINC000001554392
1.0	-9.5	-7.2	1.12	2.653	0.264	4.629	0.484	18	103	ZINC000003201907
0.9	-9.9	-7.8	0.88	1.535	0.402	5.404	1.569	20	104	ZINC000003831332
0.9	-8.0	-7.5	0.29	2.618	0.743	5.036	1.208	20	105	ZINC000036766734
0.9	-8.9	-6.7	1.04	3.337	1.052	5.314	2.102	19	106	ZINC000003792789
0.9	-9.0	-7.0	1.31	1.976	0.392	4.410	1.790	18	107	ZINC000013648755
0.9	-9.6	-7.7	0.95	2.406	0.254	5.007	1.351	17	108	ZINC000003782807

0.9	-7.7	-6.7	0.29	2.322	0.465	3.926	0.860	19	109	ZINC000000057206
0.9	-9.1	-7.6	0.75	2.063	0.343	4.609	1.165	18	110	ZINC000001530968
0.9	-8.6	-7.3	0.65	2.068	0.465	3.838	0.836	20	111	ZINC000000113428
0.9	-7.4	-6.5	0.59	1.920	0.245	3.730	0.615	19	112	ZINC000022010382
0.9	-10.5	-8.3	1.45	1.733	0.555	4.681	1.265	9	113	ZINC000003973334
0.9	-7.1	-6.5	0.29	2.537	0.716	4.338	1.052	20	114	ZINC000022056030
0.9	-8.4	-7.4	0.51	2.828	0.858	4.229	1.225	20	115	ZINC000000057464
0.9	-8.4	-7.6	0.59	2.907	0.698	5.124	1.398	20	116	ZINC000000014360
0.9	-8.4	-7.1	0.91	2.109	0.305	4.250	0.695	20	117	ZINC000000896711
0.9	-8.6	-6.7	0.66	1.684	0.303	4.699	1.583	20	118	ZINC000000897002
0.9	-8.1	-7.5	0.37	2.473	0.503	4.210	0.908	20	119	ZINC000001530803
0.9	-10.0	-7.6	0.81	3.287	0.821	5.428	1.538	20	120	ZINC000000897408
0.9	-8.2	-7.1	0.68	2.579	0.587	5.626	1.535	19	121	ZINC000000596731
0.9	-7.7	-7.0	0.37	2.189	0.605	3.983	0.807	20	122	ZINC000003874950
0.9	-7.5	-7.0	0.30	2.438	0.655	3.948	0.887	20	123	ZINC000013545634

0.9	-6.5	-5.8	0.50	1.693	0.887	4.631	1.442	20	124	ZINC000001530820
0.9	-8.0	-7.3	0.39	2.529	0.724	4.318	1.381	20	125	ZINC000000020228
0.9	-9.9	-8.5	0.80	1.966	0.485	4.526	1.519	20	126	ZINC000003830579
0.9	-8.6	-7.7	0.46	2.131	0.807	4.488	1.651	20	127	ZINC000000006310
0.9	-10.1	-8.7	0.55	2.421	0.672	4.741	1.356	20	128	ZINC000049643479
0.9	-8.5	-7.4	0.52	3.231	1.116	4.964	1.568	20	129	ZINC000003872605
0.9	-8.7	-7.7	0.40	2.069	0.313	4.297	1.317	20	130	ZINC000001536779
0.9	-9.4	-7.8	1.01	2.788	0.584	4.436	1.074	20	131	ZINC000000006427
0.9	-7.6	-6.9	0.29	2.275	0.341	4.572	1.645	20	132	ZINC000000000257
0.9	-8.1	-7.4	0.35	2.407	0.526	4.281	1.124	20	133	ZINC000000002043
0.9	-7.5	-6.9	0.36	2.656	0.444	4.953	0.900	20	134	ZINC000011680943
0.9	-8.2	-7.4	0.40	2.541	0.651	4.862	1.881	20	135	ZINC000003875368
0.9	-9.2	-7.6	0.94	1.839	0.332	4.522	1.456	20	136	ZINC000003831586
0.9	-8.4	-7.2	0.56	1.945	0.435	4.368	1.139	20	137	ZINC000000155269
0.9	-9.4	-7.9	0.72	1.959	0.660	4.178	1.923	20	138	ZINC000100036760

0.9	-6.7	-6.2	0.37	1.926	1.006	3.409	0.972	19	139	ZINC000100052205
0.9	-9.4	-7.8	0.82	1.777	0.518	4.145	2.036	20	140	ZINC000253476027
0.9	-6.9	-6.1	0.27	2.875	0.573	4.085	0.566	20	141	ZINC000000395010
0.9	-9.3	-7.1	1.09	1.874	0.423	5.027	1.045	19	142	ZINC000000968275
0.9	-7.9	-6.6	0.59	2.201	0.322	4.460	1.006	19	143	ZINC000000006016
0.9	-7.4	-6.9	0.35	2.340	0.574	3.838	1.003	20	144	ZINC000013545636
0.9	-10.3	-8.9	0.69	1.916	0.392	4.303	1.308	20	145	ZINC000084589076
0.9	-6.0	-5.7	0.17	1.993	0.494	4.032	1.413	20	146	ZINC000001530572
0.9	-8.4	-7.3	0.55	2.077	0.659	4.718	1.162	20	147	ZINC000001530751
0.9	-9.0	-7.5	0.64	2.064	0.577	4.678	1.731	20	148	ZINC000000538283
0.9	-7.5	-6.9	0.33	2.541	0.557	4.025	0.895	19	149	ZINC000000020783
0.9	-7.6	-7.1	0.24	1.975	0.498	4.688	1.182	20	150	ZINC000003785276
0.9	-6.9	-6.3	0.32	2.051	0.250	3.681	0.641	20	151	ZINC000003979899
0.8	-9.3	-7.3	0.75	2.885	0.471	5.210	1.166	20	152	ZINC000000004076
0.8	-9.4	-7.1	1.16	2.161	0.278	4.798	1.027	18	153	ZINC000000591993

0.8	-6.0	-5.8	0.11	2.477	0.558	4.437	0.589	20	154	ZINC000008214625
0.8	-8.9	-7.4	1.17	2.581	0.538	4.773	1.208	18	155	ZINC000000003642
0.8	-8.0	-6.7	0.49	2.918	0.624	5.440	1.290	20	156	ZINC000008214402
0.8	-8.4	-7.9	0.33	2.647	0.717	4.978	1.129	20	157	ZINC000001530752
0.8	-8.3	-7.1	0.47	2.262	0.463	4.042	1.174	20	158	ZINC000004632106
0.8	-8.9	-7.7	0.59	1.896	0.439	4.240	1.878	20	159	ZINC000100009280
0.8	-9.0	-7.4	0.53	2.083	0.735	3.804	1.710	19	160	ZINC000009302201
0.8	-6.1	-5.6	0.22	2.380	0.591	3.261	0.626	20	161	ZINC000001530806
0.8	-6.3	-5.6	0.26	2.472	0.960	3.238	1.044	20	162	ZINC000001530805
0.8	-8.5	-6.5	0.81	2.356	0.527	4.874	0.651	20	163	ZINC000000056647
0.8	-10.2	-7.6	1.11	2.640	0.573	5.409	1.864	19	164	ZINC000013818943
0.8	-8.0	-7.0	0.41	2.240	0.235	4.525	1.092	20	165	ZINC000087515509
0.8	-8.0	-7.5	0.22	3.134	0.610	4.411	0.923	20	166	ZINC000100032379
0.8	-9.8	-8.7	0.56	2.020	0.429	4.452	1.837	20	167	ZINC000100036756
0.8	-7.5	-6.6	0.39	2.433	0.504	4.260	1.536	20	168	ZINC000002019954

0.8	-8.8	-7.9	0.46	2.473	0.465	4.723	0.867	20	169	ZINC000000001758
0.8	-9.9	-7.6	1.25	2.148	0.328	3.964	0.709	12	170	ZINC000001280665
0.8	-7.2	-6.6	0.34	2.205	0.481	3.743	0.709	20	171	ZINC000003651680
0.8	-8.9	-7.5	0.63	2.393	0.576	4.368	1.790	20	172	ZINC000004392649
0.8	-7.2	-6.5	0.34	2.286	0.584	4.628	1.869	20	173	ZINC000003830891
0.8	-10.2	-7.9	1.16	2.420	1.037	4.443	1.563	6	174	ZINC000256433946
0.8	-8.7	-8.0	0.41	2.549	0.572	4.099	1.431	20	175	ZINC000003842753
0.8	-8.6	-7.4	0.42	2.247	0.415	3.809	0.965	20	176	ZINC000000896703
0.8	-9.6	-8.3	0.71	2.073	0.647	4.578	1.674	20	177	ZINC000003813083
0.8	-8.1	-7.2	0.40	2.410	0.467	4.817	1.533	20	178	ZINC000000001382
0.8	-8.0	-7.2	0.37	2.732	0.462	4.604	0.806	20	179	ZINC000000113410
0.8	-7.3	-6.4	0.43	2.372	0.497	3.883	0.868	20	180	ZINC000000896484
0.8	-9.6	-8.7	0.46	2.827	1.005	4.424	1.726	20	181	ZINC000100014475
0.8	-8.1	-7.3	0.39	1.803	0.278	4.437	0.657	20	182	ZINC000000000607
0.8	-6.9	-6.2	0.31	2.999	0.755	3.629	0.976	20	183	ZINC000084843283

0.8	-7.2	-6.6	0.28	2.124	0.424	3.612	0.546	20	184	ZINC000000000053
0.8	-9.1	-8.0	0.51	2.277	0.572	4.175	1.667	20	185	ZINC000005162311
0.8	-7.9	-6.8	0.64	2.717	0.677	4.261	1.271	20	186	ZINC000000002159
0.8	-8.7	-7.8	0.49	2.088	0.559	3.444	1.228	20	187	ZINC000000119717
0.8	-10.4	-8.2	1.40	2.099	0.297	4.505	0.974	9	188	ZINC000000001773
0.8	-9.7	-8.5	0.46	2.003	0.453	4.985	0.616	20	189	ZINC000012503099
0.8	-7.6	-7.0	0.30	2.219	0.378	4.827	1.674	20	190	ZINC000000896523
0.8	-8.0	-7.2	0.34	2.491	0.648	5.047	1.618	20	191	ZINC000000007673
0.8	-10.0	-8.6	0.73	2.209	0.449	4.560	1.113	20	192	ZINC000014961096
0.8	-9.3	-7.5	1.37	1.900	0.368	4.802	1.501	15	193	ZINC000001542930
0.8	-9.1	-7.7	0.61	2.127	0.633	4.630	1.501	20	194	ZINC000000000346
0.8	-8.3	-7.2	0.49	2.644	0.656	3.700	1.298	20	195	ZINC000000013156
0.8	-9.0	-8.0	0.41	1.785	0.417	4.618	0.861	20	196	ZINC000007997897
0.8	-8.0	-7.3	0.41	2.143	0.501	4.484	0.936	20	197	ZINC000000000196
0.8	-8.8	-7.1	0.48	3.052	0.462	5.557	0.925	20	198	ZINC000000601250

0.8	-9.8	-8.7	0.50	2.714	0.579	4.953	1.202	20	199	ZINC000001530973
0.8	-7.8	-7.0	0.45	2.072	0.321	4.692	1.616	20	200	ZINC000001530570
0.8	-7.4	-6.5	0.54	2.172	0.361	4.120	1.040	20	201	ZINC000003876069
0.8	-6.7	-6.3	0.17	1.366	0.213	2.741	0.502	20	202	ZINC000038140873
0.8	-8.7	-7.9	0.39	2.326	0.554	5.318	1.568	20	203	ZINC000000897258
0.8	-9.5	-8.8	0.31	2.802	0.787	4.490	1.661	20	204	ZINC000035342789
0.8	-8.5	-7.3	0.57	2.130	0.284	4.187	1.056	20	205	ZINC000001530725
0.8	-7.7	-6.9	0.38	2.005	0.416	4.248	0.938	20	206	ZINC000017285869
0.7	-7.2	-6.4	0.41	2.678	1.397	4.140	1.485	20	207	ZINC000001531009
0.7	-6.1	-5.3	0.30	3.024	1.005	3.780	1.074	20	208	ZINC000000001706
0.7	-8.5	-7.4	0.42	2.024	0.508	4.452	1.182	20	209	ZINC000001530981
0.7	-7.9	-6.9	0.50	2.932	1.027	4.472	1.729	20	210	ZINC000000002647
0.7	-9.9	-8.2	1.40	1.951	0.313	6.497	0.136	4	211	ZINC000004097440
0.7	-6.4	-6.1	0.21	2.525	0.723	3.899	0.883	20	212	ZINC000001530701
0.7	-6.6	-6.1	0.33	1.679	0.592	3.035	0.856	20	213	ZINC000100036955

0.7	-9.9	-8.1	0.98	2.130	0.436	4.829	1.285	20	214	ZINC000019632670
0.7	-8.1	-7.2	0.37	2.475	0.510	4.120	0.576	20	215	ZINC000035999642
0.7	-9.3	-7.4	0.99	2.113	0.887	5.156	1.570	8	216	ZINC000003815424
0.7	-7.7	-6.7	0.58	2.043	0.321	4.139	0.835	20	217	ZINC000000000506
0.7	-9.6	-7.6	1.01	2.917	0.712	5.083	1.670	19	218	ZINC000000527386
0.7	-9.8	-7.1	1.37	2.422	1.034	3.725	2.154	5	219	ZINC000000897222
0.7	-8.6	-7.7	0.42	2.407	0.517	4.566	1.488	20	220	ZINC000000002191
0.7	-10.5	-7.8	1.09	2.600	0.506	4.590	1.221	15	221	ZINC000095626706
0.7	-5.7	-5.2	0.20	2.385	0.939	3.423	1.079	20	222	ZINC000001530710
0.7	-8.6	-7.1	1.10	2.843	0.806	4.682	1.043	19	223	ZINC000001481833
0.7	-8.2	-6.2	0.86	2.939	0.557	4.538	1.118	20	224	ZINC000095452610
0.7	-8.4	-7.3	0.52	2.281	0.613	4.985	1.367	20	225	ZINC000000001317
0.7	-8.3	-7.5	0.39	2.230	0.431	4.177	1.382	20	226	ZINC000000057532
0.7	-9.3	-7.8	0.72	1.889	0.473	4.197	1.637	20	227	ZINC000001530814
0.7	-9.0	-6.6	0.74	3.994	0.887	5.722	1.623	19	228	ZINC000034220093

0.7	-9.3	-7.1	1.08	2.543	0.572	4.446	1.058	20	229	ZINC000095616603
0.7	-6.7	-5.9	0.37	2.752	0.752	4.724	1.894	20	230	ZINC000001530736
0.7	-6.8	-6.1	0.38	2.249	0.676	3.323	1.061	20	231	ZINC000000896663
0.7	-6.5	-5.8	0.35	2.232	0.610	3.525	0.929	20	232	ZINC000001482164
0.7	-7.3	-6.6	0.24	3.237	0.541	4.808	0.926	20	233	ZINC000000056652
0.7	-9.6	-8.1	0.53	1.748	0.659	5.115	2.537	19	234	ZINC000003812865
0.7	-8.5	-7.8	0.41	2.346	0.331	4.252	0.826	20	235	ZINC000000057254
0.7	-7.7	-7.0	0.37	1.952	0.775	4.159	1.461	20	236	ZINC000001633887
0.7	-5.4	-4.7	0.25	2.403	0.676	3.703	0.847	20	237	ZINC000003831050
0.7	-7.2	-6.4	0.39	2.194	0.549	3.783	1.090	20	238	ZINC000022010379
0.7	-9.7	-8.3	0.60	2.859	0.467	4.301	0.834	19	239	ZINC000002020233
0.7	-7.9	-7.0	0.48	2.553	0.530	4.640	0.772	20	240	ZINC000001530776
0.7	-7.2	-6.6	0.34	2.278	0.595	4.232	1.361	20	241	ZINC000000000215
0.7	-8.9	-8.0	0.44	2.974	0.705	4.941	1.658	20	242	ZINC000000000323
0.7	-8.4	-7.7	0.42	2.088	0.515	4.065	1.474	20	243	ZINC000013973998

0.7	-8.1	-7.1	0.50	2.791	0.797	4.727	1.048	20	244	ZINC000002519740
0.7	-9.4	-7.4	1.17	2.108	0.291	4.700	0.645	19	245	ZINC000000004778
0.7	-9.2	-7.5	0.90	2.085	0.520	4.560	1.617	20	246	ZINC000253476025
0.7	-10.1	-7.7	1.11	2.344	0.327	4.993	1.910	8	247	ZINC000030691679
0.7	-9.0	-8.3	0.33	2.120	0.513	4.883	1.867	20	248	ZINC000001530775
0.7	-8.0	-7.4	0.26	2.244	0.575	4.835	1.465	20	249	ZINC000001530569
0.7	-7.8	-7.0	0.37	2.527	0.541	4.114	1.687	20	250	ZINC000001530636
0.7	-8.0	-6.5	0.75	2.489	0.510	4.195	1.100	20	251	ZINC000003787060
0.7	-6.9	-6.2	0.31	2.415	0.327	5.056	1.419	20	252	ZINC000001530728
0.7	-9.0	-8.2	0.50	2.194	0.621	4.501	1.810	20	253	ZINC000100037020
0.7	-7.6	-6.8	0.38	1.700	0.570	3.680	1.595	20	254	ZINC000000020237
0.7	-9.4	-8.1	0.62	2.091	0.389	4.408	1.059	20	255	ZINC000001530764
0.7	-7.3	-6.4	0.42	2.555	0.505	4.335	1.308	20	256	ZINC000002019953
0.7	-7.3	-6.4	0.37	3.007	0.539	4.167	0.830	19	257	ZINC000100005670
0.7	-6.2	-5.6	0.25	2.242	0.395	3.363	0.775	20	258	ZINC000001843047

0.7	-6.4	-5.8	0.47	2.608	0.474	4.383	0.695	20	259	ZINC000100299039
0.7	-8.0	-7.2	0.39	1.881	0.340	4.393	0.909	20	260	ZINC000000000242
0.7	-9.2	-7.8	0.53	2.906	0.931	5.530	1.852	20	261	ZINC000007997966
0.7	-6.7	-5.9	0.41	2.375	0.677	3.792	0.892	20	262	ZINC000008403947
0.7	-9.3	-7.6	0.94	2.532	0.498	4.523	1.124	19	263	ZINC000001530948
0.7	-10.5	-10.5	0.00	0.000	0.000	0.000	0.000	1	264	ZINC000072481720
0.7	-8.1	-7.3	0.34	2.565	0.518	4.298	1.056	20	265	ZINC000007997568
0.7	-7.0	-6.4	0.29	2.485	0.575	3.952	1.353	20	266	ZINC000000113446
0.7	-7.7	-6.8	0.46	2.669	0.755	3.843	1.016	19	267	ZINC000006733300
0.6	-9.8	-7.8	1.02	2.677	0.610	4.482	1.055	16	268	ZINC000000009073
0.6	-6.1	-5.7	0.24	2.722	0.827	4.004	1.201	20	269	ZINC000100008275
0.6	-7.9	-6.9	0.52	2.675	0.514	3.876	1.156	19	270	ZINC000019702309
0.6	-9.4	-8.3	0.56	1.972	0.467	4.481	1.070	20	271	ZINC000000968273
0.6	-9.2	-7.6	0.80	3.605	0.891	5.598	1.614	20	272	ZINC000000592419
0.6	-9.1	-8.5	0.27	2.660	0.494	5.697	1.254	20	273	ZINC000001530759

0.6	-10.8	-8.9	1.43	1.708	0.286	3.110	0.448	6	274	ZINC000005764759
0.6	-7.7	-6.9	0.46	2.258	0.435	4.425	1.469	19	275	ZINC000013597823
0.6	-6.5	-5.9	0.30	3.278	0.783	4.587	1.045	20	276	ZINC000001530660
0.6	-8.1	-7.5	0.22	2.785	1.096	4.061	1.333	19	277	ZINC000003861768
0.6	-6.1	-5.6	0.23	2.415	0.707	3.661	0.952	19	278	ZINC000000155694
0.6	-8.2	-7.0	0.63	2.641	0.554	4.015	1.351	20	279	ZINC000003872055
0.6	-7.2	-6.6	0.33	2.434	0.524	4.094	1.376	20	280	ZINC000000020255
0.6	-10.0	-8.7	0.70	2.541	0.600	4.465	0.930	18	281	ZINC000000004785
0.6	-7.9	-7.2	0.34	2.960	0.762	4.036	0.832	20	282	ZINC000003872738
0.6	-9.6	-9.1	0.30	2.183	0.439	3.953	0.668	20	283	ZINC000002510358
0.6	-8.6	-7.6	0.47	1.656	0.309	4.521	0.691	20	284	ZINC000000968305
0.6	-5.0	-4.6	0.17	2.536	0.583	3.925	0.843	20	285	ZINC000100017404
0.6	-8.4	-7.3	0.66	1.929	0.638	4.131	1.285	20	286	ZINC000001530613
0.6	-6.4	-5.9	0.36	2.520	0.547	3.688	0.758	19	287	ZINC000000010163
0.6	-6.2	-5.6	0.26	2.607	0.400	4.170	0.723	20	288	ZINC000001849548

0.6	-8.9	-6.5	1.14	3.701	0.805	5.652	1.350	17	289	ZINC000003929486
0.6	-7.2	-6.7	0.26	2.918	0.814	4.556	1.389	20	290	ZINC000000001115
0.6	-6.5	-5.9	0.30	2.287	0.600	3.752	0.773	19	291	ZINC000000001798
0.6	-8.7	-7.4	0.57	1.682	0.287	4.492	1.540	20	292	ZINC000001530812
0.6	-6.4	-5.6	0.32	3.246	0.726	4.569	0.566	20	293	ZINC000001530659
0.6	-8.2	-7.2	0.67	2.472	0.562	4.253	1.494	20	294	ZINC000000968255
0.6	-9.8	-7.7	1.18	2.703	0.554	5.200	1.582	14	295	ZINC000003964126
0.6	-6.5	-5.7	0.33	2.289	1.026	3.438	0.999	20	296	ZINC000000896666
0.6	-8.6	-7.5	0.51	2.122	0.231	4.646	1.161	20	297	ZINC000001554010
0.6	-7.0	-6.4	0.30	2.540	0.819	3.909	1.440	20	298	ZINC000000075008
0.6	-7.2	-6.4	0.51	1.877	0.269	4.292	1.577	20	299	ZINC000004217475
0.6	-6.2	-5.9	0.19	1.641	0.589	4.270	0.754	20	300	ZINC000000000905
0.6	-6.2	-5.6	0.24	2.261	0.643	3.594	0.772	19	301	ZINC000003830990
0.6	-7.4	-6.7	0.41	2.155	0.588	3.805	0.358	18	302	ZINC000000968345
0.6	-9.7	-7.1	1.30	2.145	0.405	4.230	0.990	12	303	ZINC000000403533

0.6	-10.6	-8.8	0.95	2.410	0.710	4.310	1.594	20	304	ZINC000000000903
0.6	-9.4	-7.7	0.64	3.144	0.843	5.780	1.661	20	305	ZINC000252679615
0.6	-9.4	-7.4	0.99	3.059	0.671	4.554	0.949	20	306	ZINC000000000431
0.6	-9.8	-8.5	0.67	1.963	0.534	4.830	1.462	19	307	ZINC000000601316
0.6	-10.0	-8.4	0.65	2.384	0.663	4.510	1.178	20	308	ZINC000001550499
0.6	-8.0	-7.4	0.31	1.997	0.548	3.903	1.168	19	309	ZINC000000002041
0.6	-7.9	-7.4	0.31	1.865	0.390	4.272	0.717	20	310	ZINC000000508068
0.6	-7.0	-6.3	0.37	2.385	0.419	4.241	0.632	20	311	ZINC000002847375
0.6	-8.9	-7.6	0.61	2.119	0.584	4.471	1.760	20	312	ZINC000100009278
0.6	-7.8	-7.3	0.30	2.241	0.291	4.116	0.990	20	313	ZINC000000137884
0.6	-8.2	-7.1	0.49	2.073	0.372	4.526	1.485	20	314	ZINC000000020221
0.6	-9.4	-8.2	0.48	2.412	0.285	4.979	0.574	20	315	ZINC000000020251
0.6	-8.4	-7.6	0.35	2.392	0.349	4.651	0.762	20	316	ZINC000000020240
0.6	-9.8	-7.9	1.10	1.838	0.306	4.608	1.484	9	317	ZINC000118912393
0.6	-7.7	-6.8	0.36	2.082	0.600	3.776	1.010	20	318	ZINC000002005550

0.6	-9.4	-7.1	0.89	2.469	0.446	4.335	1.283	18	319	ZINC000003940470
0.6	-8.1	-7.4	0.38	3.349	1.193	5.012	1.755	20	320	ZINC000252678020
0.6	-6.6	-5.9	0.33	2.627	0.599	3.364	0.657	20	321	ZINC000000001132
0.6	-8.8	-7.1	0.72	2.571	0.662	4.494	1.558	20	322	ZINC000000057435
0.6	-8.1	-7.5	0.24	2.559	0.686	5.636	0.985	20	323	ZINC000004474564
0.6	-6.8	-6.2	0.30	2.831	0.496	4.840	1.224	20	324	ZINC000008214614
0.6	-7.1	-6.4	0.34	2.685	0.663	4.213	1.125	20	325	ZINC000000000507
0.6	-7.7	-6.8	0.55	2.336	0.465	4.548	1.164	20	326	ZINC000003981610
0.6	-8.4	-7.8	0.27	2.712	0.975	3.760	1.398	20	327	ZINC000000007782
0.6	-6.5	-5.6	0.35	2.538	0.554	3.675	0.539	20	328	ZINC000001547851
0.6	-9.2	-7.9	0.54	2.204	0.513	4.271	0.892	20	329	ZINC000019632912
0.6	-8.7	-8.0	0.34	2.579	0.665	4.345	1.569	20	330	ZINC000096006009
0.6	-7.9	-7.1	0.39	2.363	0.658	3.837	1.204	20	331	ZINC000000000271
0.6	-6.8	-6.2	0.29	2.187	0.596	3.697	1.204	20	332	ZINC000000020257
0.6	-8.3	-7.6	0.44	2.280	0.423	4.383	1.086	20	333	ZINC000002525885

0.6	-7.6	-7.1	0.27	2.341	0.549	4.132	0.849	20	334	ZINC000000002281
0.6	-6.4	-5.7	0.26	3.074	0.844	4.835	1.330	20	335	ZINC000018099446
0.6	-10.3	-8.1	0.94	2.107	0.535	5.434	1.478	19	336	ZINC000100018598
0.6	-7.2	-6.7	0.31	2.443	0.756	3.707	1.442	20	337	ZINC000005179119
0.5	-8.6	-7.3	0.48	2.210	0.398	5.496	1.170	20	338	ZINC000003785268
0.5	-8.7	-8.0	0.40	2.699	0.405	4.432	0.872	20	339	ZINC000003831139
0.5	-8.6	-7.2	0.72	2.402	0.612	4.394	1.271	20	340	ZINC000019362737
0.5	-4.8	-4.5	0.19	2.955	0.939	4.129	1.225	20	341	ZINC000003079336
0.5	-8.6	-7.7	0.67	2.541	0.579	4.193	1.491	19	342	ZINC000000018635
0.5	-9.3	-7.4	1.03	3.153	1.374	5.245	2.207	20	343	ZINC000003831417
0.5	-6.3	-5.7	0.26	2.437	0.894	4.131	1.030	20	344	ZINC000001531008
0.5	-8.6	-7.4	0.58	2.753	0.404	4.516	1.060	20	345	ZINC000000608101
0.5	-9.4	-6.9	1.13	2.517	0.633	4.389	0.950	17	346	ZINC000004215736
0.5	-8.8	-7.5	0.79	2.391	0.541	4.821	1.461	19	347	ZINC000000968327
0.5	-7.4	-6.8	0.28	1.308	0.597	5.320	1.851	20	348	ZINC000019855121

0.5	-8.7	-7.7	0.58	2.725	0.935	3.995	1.708	18	349	ZINC000003812944
0.5	-9.5	-8.3	0.90	2.082	0.386	4.230	0.882	18	350	ZINC000001999487
0.5	-4.8	-4.5	0.21	0.943	0.409	2.783	0.308	20	351	ZINC000245224194
0.5	-8.6	-7.2	0.66	2.522	0.543	4.658	1.514	20	352	ZINC000003604264
0.5	-9.3	-7.9	0.53	2.885	0.534	5.020	1.064	18	353	ZINC000000643055
0.5	-5.3	-4.7	0.27	2.865	1.188	3.515	1.260	20	354	ZINC000003581355
0.5	-7.2	-6.8	0.20	2.774	0.552	4.628	0.701	20	355	ZINC000000000469
0.5	-5.5	-4.8	0.30	2.901	1.274	3.496	1.373	20	356	ZINC000003645145
0.5	-7.4	-6.8	0.41	2.068	0.511	4.187	1.699	20	357	ZINC000000004413
0.5	-9.4	-7.7	0.72	2.951	0.524	4.756	0.716	19	358	ZINC000019632917
0.5	-9.2	-7.3	1.07	2.674	0.473	5.731	1.237	19	359	ZINC000000001850
0.5	-9.6	-7.2	1.38	2.354	0.675	4.178	1.149	10	360	ZINC000000389747
0.5	-7.0	-6.4	0.27	2.396	0.634	4.366	1.523	19	361	ZINC000018089317
0.5	-6.5	-5.8	0.28	2.697	0.673	3.318	0.812	20	362	ZINC000000002055
0.5	-6.1	-5.3	0.29	2.943	1.022	4.211	0.989	20	363	ZINC000001846431

0.5	-6.8	-6.0	0.39	1.904	0.193	6.430	1.219	20	364	ZINC000022447798
0.5	-9.9	-8.1	1.06	2.781	0.684	4.324	1.335	19	365	ZINC000000000575
0.5	-5.1	-4.8	0.15	3.157	0.801	4.837	1.026	20	366	ZINC000003843378
0.5	-9.8	-8.5	1.05	1.323	0.130	4.673	1.996	7	367	ZINC000003807917
0.5	-8.0	-7.3	0.32	3.414	0.545	5.296	0.753	20	368	ZINC000000002216
0.5	-6.8	-6.3	0.28	2.432	0.650	3.946	0.668	20	369	ZINC000000001084
0.5	-7.6	-6.7	0.38	2.670	0.671	4.060	1.265	20	370	ZINC000000049153
0.5	-8.0	-7.5	0.33	3.349	1.157	5.059	1.627	20	371	ZINC000001543475
0.5	-6.3	-5.8	0.28	2.914	1.072	4.465	1.800	20	372	ZINC000100019007
0.5	-8.5	-7.8	0.35	3.016	0.475	4.868	0.685	20	373	ZINC000000089763
0.5	-7.0	-6.1	0.40	2.341	0.722	3.466	1.120	18	374	ZINC000001482197
0.5	-6.5	-6.0	0.24	2.515	0.717	3.789	0.783	20	375	ZINC000000967597
0.5	-8.4	-7.8	0.29	3.179	1.230	4.826	1.652	20	376	ZINC000006627681
0.5	-8.6	-6.2	1.09	2.396	0.872	5.647	1.170	18	377	ZINC000001530601
0.5	-6.5	-5.9	0.33	2.206	0.466	3.496	0.917	20	378	ZINC000000010164

0.5	-8.9	-7.9	0.64	1.773	0.381	4.258	1.173	20	379	ZINC000019156872
0.5	-7.9	-6.8	0.48	2.641	0.538	5.183	0.780	20	380	ZINC000019144226
0.5	-6.5	-6.1	0.21	1.966	0.552	4.585	1.380	20	381	ZINC000006920384
0.5	-7.8	-7.0	0.37	2.311	0.215	4.369	0.532	20	382	ZINC000000014037
0.5	-7.1	-6.5	0.40	2.369	0.636	4.975	1.901	20	383	ZINC000001530717
0.5	-8.5	-7.5	0.56	2.666	0.607	4.469	1.570	20	384	ZINC000000035804
0.5	-9.1	-8.3	0.38	2.520	0.825	5.042	1.789	20	385	ZINC000009212654
0.5	-7.9	-6.9	0.46	2.119	0.712	3.630	1.519	20	386	ZINC000000020230
0.5	-6.1	-5.4	0.32	2.408	0.279	3.674	0.558	20	387	ZINC000000000711
0.5	-9.6	-7.7	1.07	1.919	0.704	5.010	1.486	17	388	ZINC000013520815
0.5	-9.5	-8.3	0.43	2.892	0.774	5.219	1.202	20	389	ZINC000000005823
0.5	-8.8	-7.1	0.61	2.314	0.544	4.513	1.581	20	390	ZINC000005456939
0.5	-7.7	-7.0	0.30	2.079	0.396	3.672	1.087	20	391	ZINC000001530929
0.5	-7.8	-6.8	0.33	3.722	1.186	4.565	1.501	20	392	ZINC000000006481
0.5	-6.5	-6.0	0.32	1.804	0.982	3.295	1.249	20	393	ZINC000001532179

0.5	-8.8	-7.7	0.58	2.551	0.419	5.001	1.207	20	394	ZINC000001690324
0.5	-6.3	-6.0	0.20	2.272	0.566	4.006	0.790	20	395	ZINC000003830569
0.5	-6.6	-6.1	0.29	2.187	0.800	4.496	1.029	20	396	ZINC000004978673
0.5	-6.6	-6.0	0.31	2.807	0.651	5.454	1.361	19	397	ZINC000001530737
0.5	-7.1	-6.7	0.23	1.824	0.543	4.289	1.254	20	398	ZINC000006845963
0.5	-9.8	-7.8	0.98	2.613	0.379	4.838	0.803	18	399	ZINC000000007295
0.5	-9.4	-8.1	0.59	2.473	0.555	5.261	0.639	20	400	ZINC000001850377
0.5	-9.6	-7.1	1.36	2.249	0.502	3.887	1.748	5	401	ZINC000256433948
0.5	-8.2	-7.4	0.42	2.318	0.776	4.187	1.487	19	402	ZINC000000136138
0.5	-9.0	-7.6	0.82	2.553	0.699	4.868	1.896	20	403	ZINC000004654889
0.5	-9.1	-7.5	0.57	1.922	0.356	6.742	1.040	19	404	ZINC000038945666
0.5	-8.6	-7.6	0.48	2.160	0.544	4.104	1.484	19	405	ZINC000003775644
0.5	-7.1	-6.4	0.27	3.564	0.645	4.425	0.755	20	406	ZINC000000000061
0.5	-8.3	-7.2	0.45	2.275	0.549	4.635	1.278	20	407	ZINC000000005895
0.5	-9.0	-6.9	1.12	2.349	0.427	4.393	0.806	20	408	ZINC000000491073

0.5	-9.5	-7.9	0.82	2.641	0.823	5.437	0.868	20	409	ZINC000000968263
0.4	-5.8	-5.4	0.22	2.983	0.722	3.787	0.906	20	410	ZINC000000005152
0.4	-7.2	-6.4	0.29	2.398	0.722	4.938	1.794	20	411	ZINC000001530811
0.4	-8.2	-7.1	0.69	2.734	0.378	4.705	1.124	20	412	ZINC000000896569
0.4	-8.3	-6.0	1.23	3.388	1.212	5.100	1.664	9	413	ZINC000001542199
0.4	-8.5	-7.4	0.45	2.692	0.380	4.675	0.905	20	414	ZINC000003802690
0.4	-6.3	-5.7	0.28	2.504	0.867	3.231	0.971	20	415	ZINC000000004949
0.4	-8.1	-6.9	0.48	2.674	0.543	6.232	1.765	20	416	ZINC000000538509
0.4	-6.1	-5.6	0.27	3.340	0.577	4.247	0.700	20	417	ZINC000100007011
0.4	-8.8	-7.9	0.44	2.659	0.754	5.058	0.976	20	418	ZINC000000003911
0.4	-9.6	-7.2	1.40	1.326	0.100	6.524	0.031	4	419	ZINC000004428529
0.4	-6.3	-5.6	0.29	2.805	1.006	4.238	0.968	20	420	ZINC000001530283
0.4	-9.3	-7.0	1.01	2.177	0.427	4.544	1.605	18	421	ZINC000003831282
0.4	-6.8	-6.2	0.27	2.574	0.552	4.216	1.364	19	422	ZINC000001482049
0.4	-9.1	-8.2	0.43	2.678	0.796	4.397	0.669	20	423	ZINC000000001016

0.4	-7.1	-6.3	0.41	2.892	0.832	4.052	0.981	20	424	ZINC000000056568
0.4	-6.7	-6.0	0.32	2.875	0.780	4.041	0.804	20	425	ZINC000013550868
0.4	-8.3	-6.2	0.80	2.509	0.492	5.736	2.017	20	426	ZINC000013587680
0.4	-8.1	-7.6	0.25	2.503	0.452	4.664	1.105	20	427	ZINC000004258316
0.4	-10.1	-7.8	1.12	1.638	0.300	4.688	1.491	9	428	ZINC000022065398
0.4	-6.9	-6.1	0.52	2.547	0.579	3.842	1.389	20	429	ZINC000012503151
0.4	-9.7	-7.9	1.01	2.176	0.451	4.627	1.376	20	430	ZINC000000968257
0.4	-9.4	-8.3	0.63	2.841	0.710	4.358	1.633	20	431	ZINC000003874498
0.4	-6.6	-6.0	0.37	2.273	0.659	3.359	1.198	20	432	ZINC000006021043
0.4	-8.3	-7.2	0.40	2.614	0.548	5.876	0.727	20	433	ZINC000001530939
0.4	-7.3	-6.6	0.34	3.116	0.738	4.361	1.548	20	434	ZINC000000119344
0.4	-8.1	-7.0	0.61	2.406	0.503	4.102	1.497	20	435	ZINC000021303210
0.4	-6.8	-6.1	0.37	2.205	0.633	3.818	1.222	20	436	ZINC000000057062
0.4	-9.4	-8.3	0.64	3.030	0.491	4.810	1.234	20	437	ZINC000000057255
0.4	-8.5	-7.4	0.56	3.313	0.740	5.271	1.559	19	438	ZINC000000016154

0.4	-7.7	-6.8	0.44	2.290	0.584	4.592	1.641	20	439	ZINC000001530568
0.4	-6.6	-6.0	0.26	2.436	0.550	3.542	0.595	20	440	ZINC000000001411
0.4	-7.2	-6.8	0.25	2.831	0.389	5.098	1.009	19	441	ZINC000019594594
0.4	-6.9	-6.5	0.28	2.014	0.412	4.027	0.788	20	442	ZINC000000968303
0.4	-9.9	-8.3	1.02	2.250	0.406	4.456	0.974	18	443	ZINC000001530625
0.4	-8.6	-6.9	0.89	2.153	0.501	4.779	1.919	20	444	ZINC000000538386
0.4	-7.6	-7.1	0.31	3.367	0.472	5.988	1.287	20	445	ZINC000145808877
0.4	-8.3	-7.5	0.37	2.011	0.456	3.724	1.568	20	446	ZINC000001530713
0.4	-7.6	-6.9	0.42	2.859	0.624	4.267	1.305	20	447	ZINC000000083315
0.4	-6.7	-5.9	0.35	2.544	0.808	3.377	0.975	20	448	ZINC000000388462
0.4	-7.4	-6.6	0.38	2.966	0.319	4.999	1.229	20	449	ZINC000004097225
0.4	-8.7	-7.0	0.90	3.494	0.832	5.450	1.438	17	450	ZINC000000020220
0.4	-7.6	-6.8	0.54	2.931	0.861	4.423	1.182	20	451	ZINC000000896918
0.4	-7.6	-7.1	0.36	2.277	0.560	3.841	1.555	20	452	ZINC000003813010
0.4	-10.0	-8.7	0.53	1.895	0.301	4.065	1.792	20	453	ZINC000043207851

0.4	-6.8	-6.3	0.35	2.945	0.711	3.641	0.849	19	454	ZINC000000105196
0.4	-9.5	-8.6	0.36	3.114	0.666	5.280	1.521	20	455	ZINC000000000416
0.4	-7.9	-7.1	0.56	1.744	0.322	4.229	1.479	19	456	ZINC000000154964
0.4	-9.6	-8.6	0.46	2.912	0.698	4.945	1.366	20	457	ZINC000003818726
0.4	-8.0	-6.6	0.78	2.833	0.466	4.557	0.946	19	458	ZINC000034781704
0.4	-8.3	-7.0	0.61	1.754	0.338	4.294	1.531	20	459	ZINC000000000456
0.4	-4.9	-4.1	0.26	4.843	1.743	5.849	1.766	20	460	ZINC000001530427
0.4	-6.6	-5.9	0.43	2.166	0.813	3.485	1.395	20	461	ZINC000008466459
0.4	-10.3	-9.1	0.87	2.531	0.743	4.615	1.490	20	462	ZINC000001996117
0.4	-9.1	-7.1	1.00	2.259	0.430	5.080	1.226	16	463	ZINC000001997125
0.4	-4.5	-4.4	0.17	1.484	1.568	3.163	1.352	20	464	ZINC000086040406
0.4	-8.3	-7.6	0.38	2.264	0.411	4.725	1.553	20	465	ZINC000000057533
0.4	-7.0	-6.3	0.52	2.512	1.016	3.951	2.018	19	466	ZINC000003831006
0.4	-6.8	-6.2	0.32	3.192	1.714	4.769	1.946	20	467	ZINC000001533877
0.4	-7.3	-6.5	0.49	2.998	1.513	4.954	2.019	20	468	ZINC000000001673

0.4	-5.0	-4.6	0.17	2.280	0.481	3.538	0.850	20	469	ZINC000003831051
0.4	-6.4	-5.7	0.32	2.522	0.772	3.461	0.766	19	470	ZINC000000000490
0.4	-9.3	-7.2	1.21	2.796	0.484	5.508	1.774	16	471	ZINC000035653007
0.4	-9.0	-8.3	0.42	2.024	0.419	3.744	1.364	20	472	ZINC000000002272
0.4	-8.1	-7.5	0.33	2.131	0.328	4.871	1.776	20	473	ZINC000035342787
0.4	-7.6	-7.0	0.36	2.121	0.297	3.658	1.066	20	474	ZINC000000156395
0.4	-6.2	-5.4	0.35	2.861	0.649	3.550	0.772	20	475	ZINC000100071256
0.4	-8.5	-7.5	0.55	2.024	0.438	4.299	1.398	20	476	ZINC000000968301
0.4	-8.0	-7.0	0.44	2.673	0.950	5.013	1.843	20	477	ZINC000000537957
0.4	-8.0	-7.4	0.38	2.070	0.410	3.969	0.856	20	478	ZINC000000001267
0.4	-7.5	-7.1	0.21	2.220	0.701	4.571	1.216	19	479	ZINC000004474603
0.4	-6.2	-5.8	0.26	2.224	0.533	3.360	0.972	20	480	ZINC000006021033
0.4	-6.3	-5.6	0.38	2.349	0.790	3.596	1.172	20	481	ZINC000006036847
0.4	-8.8	-7.8	0.61	2.191	0.446	4.713	1.872	20	482	ZINC000100015775
0.4	-6.7	-6.3	0.24	3.591	0.816	5.227	0.949	20	483	ZINC000018043251

0.4	-7.4	-6.6	0.46	1.894	0.435	4.116	1.546	20	484	ZINC000001633889
0.4	-7.0	-6.6	0.23	2.257	0.498	4.437	1.690	20	485	ZINC000001530938
0.4	-7.1	-6.6	0.26	2.355	0.700	3.688	1.519	20	486	ZINC000019168887
0.4	-9.9	-7.8	1.06	2.406	0.644	5.172	1.854	7	487	ZINC000036520252
0.4	-8.8	-7.6	0.66	1.935	0.774	4.092	1.907	20	488	ZINC000100035385
0.4	-9.2	-8.2	0.60	2.021	0.581	4.375	1.935	20	489	ZINC000100036751
0.4	-6.1	-5.7	0.25	2.068	0.696	3.731	0.572	19	490	ZINC000100024116
0.4	-6.4	-6.0	0.27	2.378	0.550	3.820	0.668	20	491	ZINC000003794711
0.4	-8.9	-7.8	0.62	2.014	0.503	3.855	1.603	20	492	ZINC000003818808
0.4	-8.8	-7.5	0.57	2.567	0.589	4.268	1.366	20	493	ZINC000000015515
0.4	-6.1	-5.6	0.25	2.528	1.118	3.721	1.090	20	494	ZINC000001531036
0.4	-9.8	-7.1	1.09	2.164	0.362	4.392	1.426	15	495	ZINC000100074252
0.4	-7.6	-7.2	0.24	2.414	0.463	4.355	1.277	20	496	ZINC000001530862
0.4	-7.8	-7.2	0.30	2.678	0.766	4.982	1.650	20	497	ZINC000000001899
0.4	-6.0	-5.1	0.27	2.485	1.013	4.244	1.078	20	498	ZINC000100009383

0.4	-10.1	-7.8	1.23	2.404	0.529	4.207	1.226	15	499	ZINC000011677376
0.4	-7.9	-6.9	0.37	1.922	0.429	5.813	1.882	20	500	ZINC000021982937
0.4	-8.7	-7.6	0.53	2.160	0.399	4.399	0.935	20	501	ZINC000001530688
0.4	-6.4	-6.0	0.25	1.748	0.295	3.703	0.748	19	502	ZINC000000391812
0.4	-9.0	-7.3	0.96	2.835	0.902	5.105	1.968	20	503	ZINC000003812869
0.4	-8.8	-8.0	0.48	3.437	0.812	5.553	1.358	20	504	ZINC000001530638
0.4	-7.4	-6.0	0.82	2.450	0.430	4.813	0.989	20	505	ZINC000003830961
0.4	-8.9	-7.9	0.62	2.559	0.581	5.345	1.143	20	506	ZINC000003778874
0.4	-6.0	-5.6	0.22	1.622	0.661	3.514	0.546	20	507	ZINC000245204924
0.4	-7.2	-6.4	0.38	2.617	0.606	4.181	1.476	20	508	ZINC000000057340
0.3	-7.7	-6.7	0.51	2.586	0.464	4.636	0.747	20	509	ZINC000000000096
0.3	-5.5	-5.2	0.16	1.353	0.407	4.545	1.901	20	510	ZINC000001529266
0.3	-6.0	-5.4	0.24	1.754	0.249	3.673	0.647	20	511	ZINC000004097426
0.3	-6.1	-5.7	0.23	1.855	0.697	3.040	1.353	20	512	ZINC000001481910
0.3	-9.7	-7.2	1.23	2.531	0.441	5.549	1.489	19	513	ZINC000084400879

0.3	-6.6	-6.0	0.27	2.608	0.692	4.644	1.406	20	514	ZINC000003781664
0.3	-8.6	-7.7	0.49	2.297	0.429	4.825	1.131	20	515	ZINC000000000856
0.3	-9.6	-7.2	1.19	2.382	0.333	5.129	1.274	13	516	ZINC000019796087
0.3	-9.7	-7.7	0.98	2.264	0.448	4.906	1.243	20	517	ZINC000000003876
0.3	-6.3	-5.7	0.26	2.338	0.310	3.657	0.683	20	518	ZINC000000002009
0.3	-9.1	-7.4	0.83	2.224	0.646	4.214	1.769	20	519	ZINC000000000596
0.3	-9.0	-8.3	0.34	2.455	0.528	5.213	1.434	20	520	ZINC000001530760
0.3	-9.9	-7.3	1.12	2.974	0.836	4.775	1.352	16	521	ZINC000000897085
0.3	-8.6	-7.5	0.57	1.993	0.383	5.400	1.591	19	522	ZINC000012468792
0.3	-8.7	-7.1	0.69	2.167	0.511	4.515	1.545	20	523	ZINC000001530912
0.3	-8.9	-7.8	0.60	2.702	0.536	5.558	2.062	20	524	ZINC000000968330
0.3	-9.6	-8.7	0.56	1.997	0.499	4.053	1.265	18	525	ZINC000000006157
0.3	-6.8	-6.5	0.22	2.188	0.431	3.293	1.202	20	526	ZINC000000000693
0.3	-10.5	-8.4	0.78	2.072	0.702	4.932	2.054	19	527	ZINC000000896560
0.3	-9.2	-8.1	0.45	2.507	0.482	4.781	0.940	20	528	ZINC000000403010

0.3	-9.0	-8.0	0.54	2.792	0.735	5.315	1.775	18	529	ZINC000002568036
0.3	-6.6	-6.1	0.30	3.588	0.683	4.691	0.842	20	530	ZINC000000034157
0.3	-9.0	-8.0	0.51	2.476	0.465	5.422	1.196	19	531	ZINC000003873296
0.3	-7.5	-6.9	0.34	2.113	0.286	5.253	1.180	20	532	ZINC000003875342
0.3	-4.9	-4.5	0.17	2.975	0.878	4.060	1.153	20	533	ZINC000003079342
0.3	-9.4	-8.5	0.44	3.010	0.647	5.191	1.401	20	534	ZINC000000004319
0.3	-6.0	-5.5	0.22	2.830	0.892	4.101	1.004	20	535	ZINC000000049154
0.3	-7.3	-6.7	0.46	2.303	0.485	4.512	1.435	20	536	ZINC000000002176
0.3	-6.4	-6.0	0.25	3.211	0.420	4.218	0.600	20	537	ZINC000012358719
0.3	-8.6	-7.3	0.55	2.197	0.482	3.840	1.055	19	538	ZINC000000113426
0.3	-8.5	-7.5	0.44	2.890	0.847	4.443	1.619	20	539	ZINC000053084692
0.3	-8.5	-6.8	0.91	2.038	0.296	4.802	1.639	15	540	ZINC000100037885
0.3	-8.5	-6.3	1.13	2.334	0.357	4.435	1.740	19	541	ZINC000003830215
0.3	-9.0	-7.4	0.56	2.532	0.441	5.418	1.435	19	542	ZINC000000006300
0.3	-7.2	-6.4	0.42	2.307	0.630	3.817	1.292	20	543	ZINC000009302239

0.3	-8.7	-7.6	0.54	2.158	0.478	4.671	0.942	20	544	ZINC000000020245
0.3	-5.0	-4.7	0.15	2.393	0.759	3.510	1.024	20	545	ZINC000003830314
0.3	-7.5	-6.9	0.30	2.241	0.507	4.143	1.421	20	546	ZINC000001530816
0.3	-6.9	-6.5	0.30	2.813	0.716	4.472	1.609	20	547	ZINC000035024346
0.3	-6.4	-5.7	0.33	2.537	0.727	3.386	1.065	20	548	ZINC000000000373
0.3	-7.1	-6.6	0.21	2.906	0.641	4.304	0.928	20	549	ZINC000001482184
0.3	-8.6	-7.4	0.51	3.146	0.679	4.970	1.207	20	550	ZINC000000001464
0.3	-7.4	-6.7	0.33	2.999	0.700	4.399	1.028	20	551	ZINC000000001735
0.3	-10.1	-7.6	1.75	1.509	0.474	3.406	1.348	3	552	ZINC000004655029
0.3	-7.0	-6.5	0.23	3.415	0.958	4.322	1.270	20	553	ZINC000000085733
0.3	-7.0	-6.5	0.26	2.655	0.644	4.042	1.200	20	554	ZINC000000895199
0.3	-6.2	-5.8	0.32	2.700	0.763	3.612	1.220	20	555	ZINC000001690604
0.3	-3.6	-3.0	0.20	4.047	1.486	4.326	1.481	20	556	ZINC000001487602
0.3	-8.2	-7.3	0.45	2.074	0.428	4.357	1.007	20	557	ZINC000000000565
0.3	-8.7	-7.7	0.42	2.680	0.850	4.595	1.919	18	558	ZINC000000008667

0.3	-8.7	-8.1	0.28	2.448	0.627	4.485	1.313	19	559	ZINC000000020241
0.3	-8.0	-6.7	0.89	2.395	0.510	4.535	1.407	20	560	ZINC000100001964
0.3	-9.2	-6.8	1.54	2.051	0.429	4.723	1.833	7	561	ZINC000003882036
0.3	-5.8	-5.4	0.19	3.173	1.019	4.403	1.136	20	562	ZINC000100006770
0.3	-7.1	-6.8	0.17	2.737	0.648	4.752	0.718	20	563	ZINC000000000746
0.3	-9.4	-8.0	0.90	2.082	0.524	4.642	1.329	20	564	ZINC000100036536
0.3	-6.3	-5.9	0.26	2.528	0.522	3.909	0.957	20	565	ZINC000000001341
0.3	-6.7	-6.0	0.41	2.514	0.651	3.876	1.136	20	566	ZINC000000020259
0.3	-5.7	-5.2	0.22	3.014	0.880	3.891	0.710	20	567	ZINC000000006226
0.3	-5.9	-5.4	0.25	3.142	0.764	3.795	0.878	19	568	ZINC000100061056
0.3	-7.4	-6.0	0.76	2.245	0.503	4.534	1.125	20	569	ZINC000005819214
0.3	-7.6	-6.4	0.58	2.268	0.552	3.853	1.560	20	570	ZINC000000004166
0.3	-4.7	-3.9	0.27	3.956	2.383	4.539	2.525	20	571	ZINC000034676245
0.3	-7.9	-7.1	0.43	2.468	0.331	4.378	1.277	20	572	ZINC000000896819
0.3	-5.9	-5.5	0.22	1.792	0.656	3.351	1.136	20	573	ZINC000008437287

0.3	-9.1	-8.2	0.51	3.126	0.572	5.645	1.120	20	574	ZINC000003830842
0.3	-7.7	-6.9	0.40	2.659	0.377	5.117	1.187	20	575	ZINC000008220878
0.3	-8.7	-7.7	0.51	2.762	0.442	5.047	0.910	19	576	ZINC000000014864
0.3	-8.9	-7.4	0.89	1.881	0.232	4.006	0.707	19	577	ZINC000014263142
0.3	-7.2	-6.6	0.35	2.322	0.543	4.231	1.582	20	578	ZINC000000007601
0.3	-8.5	-7.6	0.54	2.635	0.506	4.816	1.050	20	579	ZINC000003794601
0.3	-6.1	-5.5	0.23	2.283	1.129	3.875	1.026	20	580	ZINC000002539702
0.3	-6.3	-5.9	0.27	2.857	0.944	3.595	1.187	20	581	ZINC000000002101
0.3	-5.8	-5.1	0.28	2.656	1.027	4.456	1.056	20	582	ZINC000018279893
0.3	-7.3	-6.6	0.33	2.729	0.588	4.402	1.058	20	583	ZINC000016929327
0.3	-9.3	-7.6	0.69	3.311	0.402	5.732	1.236	20	584	ZINC000019632614
0.3	-5.0	-4.7	0.18	2.372	0.668	3.461	1.057	20	585	ZINC000000000083
0.3	-8.3	-7.1	0.58	2.855	0.850	4.184	1.594	19	586	ZINC000000896731
0.3	-7.3	-6.4	0.47	2.623	0.695	4.165	0.999	20	587	ZINC000000020231
0.3	-9.3	-7.2	1.21	3.022	0.775	4.971	2.079	15	588	ZINC000000601305

0.3	-8.7	-7.6	0.68	2.006	0.769	4.452	1.817	19	589	ZINC000100052267
0.3	-7.7	-7.1	0.38	2.751	0.427	4.633	0.907	20	590	ZINC000000000122
0.3	-10.1	-7.9	1.29	1.914	0.651	4.123	1.534	6	591	ZINC000003920719
0.3	-10.7	-8.4	1.04	2.891	0.631	4.753	0.871	20	592	ZINC000003874185
0.3	-7.8	-6.8	0.51	2.091	0.326	3.761	0.963	20	593	ZINC000003876068
0.3	-9.2	-7.2	0.98	2.365	0.645	5.259	1.914	16	594	ZINC000003830500
0.3	-4.3	-3.9	0.19	4.930	2.222	5.618	2.225	20	595	ZINC000000895034
0.3	-9.4	-7.0	1.24	2.289	0.587	4.338	1.895	8	596	ZINC000256433952
0.3	-9.7	-7.6	0.71	3.039	0.722	4.794	0.612	19	597	ZINC000028973441
0.3	-8.0	-7.1	0.56	2.682	0.513	4.643	1.475	19	598	ZINC000002539827
0.3	-7.1	-6.5	0.30	2.230	0.560	4.579	1.621	20	599	ZINC000001530951
0.3	-9.6	-7.5	1.20	1.717	0.152	5.112	0.835	16	600	ZINC000003874715
0.3	-8.3	-7.6	0.35	2.177	0.382	4.242	1.136	20	601	ZINC000003830339
0.3	-4.8	-4.6	0.13	3.514	1.139	4.366	1.142	20	602	ZINC000003782550
0.3	-8.3	-7.5	0.53	2.063	0.569	4.986	2.123	20	603	ZINC000003813078

0.3	-7.6	-6.8	0.36	2.794	1.154	3.769	1.371	20	604	ZINC000012360535
0.3	-7.4	-6.0	0.85	3.036	0.748	5.527	1.880	19	605	ZINC000043207237
0.3	-8.1	-7.0	0.54	2.223	0.389	4.434	0.653	20	606	ZINC000000896709
0.3	-8.6	-7.8	0.38	2.519	0.525	4.498	0.804	20	607	ZINC000003960338
0.3	-6.6	-6.2	0.21	1.905	0.703	4.135	1.200	20	608	ZINC000006845860
0.3	-9.7	-7.4	1.30	1.609	0.503	4.614	2.079	9	609	ZINC000003813047
0.3	-8.6	-7.1	0.77	2.838	0.420	4.624	1.283	20	610	ZINC000000895154
0.3	-6.5	-6.1	0.32	2.588	0.626	3.478	1.161	20	611	ZINC000000001644
0.3	-9.9	-8.6	0.49	3.135	0.599	5.701	1.455	20	612	ZINC000002032615
0.3	-7.1	-6.6	0.31	2.546	0.586	4.087	1.321	20	613	ZINC000000403079
0.3	-7.5	-6.6	0.50	1.923	0.657	4.371	1.413	20	614	ZINC000017285872
0.3	-6.9	-6.2	0.39	2.587	0.349	4.489	1.222	19	615	ZINC000253917094
0.2	-9.3	-7.7	0.89	2.439	0.575	4.740	0.881	19	616	ZINC000000004724
0.2	-5.6	-5.3	0.25	2.569	0.862	3.670	1.018	20	617	ZINC000000113442
0.2	-5.3	-5.0	0.20	3.243	1.814	4.314	1.705	20	618	ZINC000001482113

0.2	-8.0	-7.5	0.22	2.904	0.656	4.718	1.139	20	619	ZINC000001530703
0.2	-8.0	-7.2	0.44	2.903	0.656	5.528	0.990	20	620	ZINC000002000707
0.2	-6.3	-5.6	0.28	2.538	1.140	3.427	1.306	20	621	ZINC000000001554
0.2	-8.4	-7.8	0.29	2.685	0.551	4.408	1.040	20	622	ZINC000053045054
0.2	-6.7	-6.2	0.26	2.563	0.773	3.490	0.884	20	623	ZINC000000008492
0.2	-9.0	-7.7	0.74	2.460	0.740	4.408	1.371	20	624	ZINC000000001175
0.2	-7.3	-6.5	0.42	2.104	0.293	5.971	1.329	20	625	ZINC000008214635
0.2	-7.5	-6.6	0.44	2.117	0.734	3.726	1.876	20	626	ZINC000019166988
0.2	-9.3	-7.0	1.22	2.560	0.401	4.765	0.812	20	627	ZINC000000020248
0.2	-6.2	-5.6	0.27	2.419	0.642	3.325	1.007	20	628	ZINC000003872520
0.2	-9.0	-8.0	0.48	2.481	0.704	4.557	1.345	20	629	ZINC000033943508
0.2	-7.2	-6.5	0.51	2.280	0.476	3.900	1.463	20	630	ZINC000003795098
0.2	-3.9	-3.7	0.14	2.998	0.882	4.219	0.771	20	631	ZINC000002539484
0.2	-6.0	-5.5	0.30	2.796	1.026	4.099	1.078	20	632	ZINC000003803652
0.2	-4.9	-4.6	0.14	3.656	2.854	5.393	2.474	20	633	ZINC000006827695

0.2	-8.1	-7.1	0.48	2.589	0.467	4.438	1.343	19	634	ZINC000004228258
0.2	-7.9	-7.3	0.38	2.653	0.653	4.733	1.784	20	635	ZINC000001530652
0.2	-6.0	-5.5	0.28	2.557	0.457	3.888	0.813	20	636	ZINC000000057001
0.2	-4.1	-3.7	0.24	2.999	1.628	3.845	1.610	20	637	ZINC000001532728
0.2	-7.1	-6.6	0.27	3.099	0.549	4.813	0.928	20	638	ZINC000000002273
0.2	-7.5	-6.5	0.54	2.483	0.343	4.557	0.993	20	639	ZINC000000896698
0.2	-8.6	-7.3	0.72	2.321	0.633	5.120	1.809	20	640	ZINC000001546066
0.2	-8.1	-7.4	0.36	1.914	0.385	4.410	1.052	20	641	ZINC000001842633
0.2	-8.8	-7.8	0.44	2.717	0.471	5.180	1.643	20	642	ZINC000000018087
0.2	-4.5	-4.0	0.27	4.249	2.887	5.390	2.784	20	643	ZINC000008101109
0.2	-8.9	-8.2	0.33	2.173	0.354	4.829	1.222	20	644	ZINC000000896463
0.2	-8.0	-6.6	0.65	2.301	0.563	4.642	1.964	20	645	ZINC000022002214
0.2	-8.0	-7.5	0.25	2.481	0.512	4.812	0.704	20	646	ZINC000001543873
0.2	-7.4	-6.7	0.32	3.332	0.645	4.486	0.650	20	647	ZINC000000004321
0.2	-7.7	-6.4	0.52	2.221	0.296	5.248	1.001	19	648	ZINC000003929508

0.2	-7.8	-6.8	0.43	2.911	0.792	4.905	1.244	20	649	ZINC000001530654
0.2	-6.7	-6.3	0.17	2.095	0.352	4.241	1.695	20	650	ZINC000018115268
0.2	-8.1	-7.4	0.33	3.275	0.383	5.650	1.162	19	651	ZINC000001530940
0.2	-6.8	-6.3	0.31	1.611	0.375	3.825	0.810	20	652	ZINC000019632633
0.2	-4.4	-3.9	0.25	1.822	0.610	3.302	0.613	20	653	ZINC000000896409
0.2	-7.6	-6.8	0.58	2.514	0.539	4.520	1.545	20	654	ZINC000001035331
0.2	-7.1	-6.5	0.37	2.913	1.191	5.467	1.822	20	655	ZINC000001530756
0.2	-7.9	-7.1	0.44	2.364	0.306	4.135	0.536	18	656	ZINC000004228257
0.2	-9.5	-7.0	1.25	1.409	0.409	5.090	2.432	8	657	ZINC000003833821
0.2	-8.3	-6.9	0.57	2.510	0.758	4.853	1.910	20	658	ZINC000012484958
0.2	-8.7	-7.2	0.68	2.434	0.622	5.337	1.671	19	659	ZINC000003792417
0.2	-8.2	-6.7	0.71	2.132	0.414	4.804	1.145	20	660	ZINC000019632706
0.2	-6.6	-5.9	0.38	2.205	0.527	3.347	0.775	20	661	ZINC000001639567
0.2	-9.4	-7.1	1.20	2.948	0.661	4.807	1.526	19	662	ZINC000000601283
0.2	-5.2	-4.9	0.17	2.320	1.440	3.933	1.393	20	663	ZINC000003830813

0.2	-8.7	-7.6	0.70	2.835	0.623	4.598	1.257	20	664	ZINC000000057313
0.2	-8.5	-7.2	0.54	2.852	0.654	5.079	1.492	20	665	ZINC000004693575
0.2	-8.3	-7.2	0.56	2.351	0.786	4.397	1.950	20	666	ZINC000002008866
0.2	-7.8	-7.1	0.41	2.364	0.415	4.849	1.756	20	667	ZINC000004095696
0.2	-7.5	-6.8	0.44	2.659	0.584	5.316	1.298	20	668	ZINC000000057513
0.2	-9.1	-7.4	0.83	2.926	0.890	4.890	1.732	20	669	ZINC000000537822
0.2	-9.2	-8.1	0.68	2.565	0.512	4.849	1.393	19	670	ZINC000001530947
0.2	-8.1	-7.4	0.41	2.694	0.663	4.365	1.502	20	671	ZINC000019594599
0.2	-8.8	-7.5	0.47	3.144	0.636	5.298	1.008	20	672	ZINC000019364230
0.2	-10.1	-9.0	0.57	1.683	0.437	4.679	1.741	20	673	ZINC000012503068
0.2	-9.5	-7.0	1.11	3.088	0.714	4.938	1.567	16	674	ZINC000003786192
0.2	-6.8	-6.2	0.33	3.253	0.619	4.186	0.786	20	675	ZINC000000266964
0.2	-9.4	-7.9	0.84	2.788	0.679	5.383	1.765	20	676	ZINC000001530689
0.2	-7.2	-6.6	0.34	2.241	0.535	3.948	1.487	20	677	ZINC000001530950
0.2	-8.5	-7.3	0.77	3.529	0.466	5.813	0.821	20	678	ZINC000019364222

0.2	-8.1	-7.2	0.44	2.228	0.360	5.024	1.439	20	679	ZINC000003831165
0.2	-6.9	-6.3	0.24	3.032	0.594	4.961	1.238	19	680	ZINC000001530555
0.2	-2.7	-2.4	0.18	3.675	1.590	4.030	1.467	20	681	ZINC000005224188
0.2	-4.5	-4.2	0.19	2.195	0.863	3.542	0.880	19	682	ZINC000003831475
0.2	-7.9	-5.7	1.01	2.227	0.506	4.661	1.909	20	683	ZINC000003875393
0.2	-9.6	-7.4	1.28	2.510	0.329	4.455	0.779	10	684	ZINC000000004351
0.2	-7.0	-6.6	0.24	2.593	0.452	4.795	1.664	20	685	ZINC000001530716
0.2	-7.2	-6.7	0.24	2.741	0.933	4.432	1.593	20	686	ZINC000001530600
0.2	-9.1	-7.7	0.73	3.551	0.652	5.842	1.121	19	687	ZINC000000538564
0.2	-7.8	-6.8	0.39	2.487	0.636	4.265	1.117	20	688	ZINC000000897291
0.2	-6.6	-6.2	0.21	2.034	0.523	5.194	1.397	20	689	ZINC000014881137
0.2	-7.9	-6.9	0.60	3.310	1.058	4.695	1.534	18	690	ZINC000003871541
0.2	-6.1	-5.6	0.27	3.457	1.377	4.795	1.461	20	691	ZINC000006382803
0.2	-8.1	-6.1	1.15	2.495	0.467	4.486	0.964	19	692	ZINC000096942201
0.2	-8.1	-7.4	0.36	2.740	0.519	4.492	1.243	20	693	ZINC000000156792

0.2	-5.1	-4.6	0.26	2.875	1.278	3.623	1.399	20	694	ZINC000001532522
0.2	-8.5	-7.8	0.31	2.014	0.345	4.359	1.856	20	695	ZINC000000000301
0.2	-7.9	-7.2	0.47	2.314	0.257	4.564	1.284	20	696	ZINC000008855117
0.2	-9.8	-7.7	1.27	2.182	0.200	4.806	1.016	15	697	ZINC000003812988
0.2	-8.1	-7.4	0.40	1.940	0.576	4.040	1.832	20	698	ZINC000000518554
0.2	-9.7	-7.1	1.18	2.216	0.487	5.405	1.802	20	699	ZINC000009212427
0.2	-6.5	-5.9	0.27	3.043	0.649	4.662	1.014	20	700	ZINC000000039089
0.2	-8.2	-7.3	0.51	2.271	0.624	4.536	1.799	20	701	ZINC000000389149
0.2	-8.2	-7.0	0.79	3.231	1.300	4.705	2.085	19	702	ZINC000013512456
0.2	-8.7	-7.5	0.67	2.184	0.584	4.568	1.151	20	703	ZINC000000896958
0.2	-10.2	-8.5	0.99	1.778	0.378	4.503	1.936	20	704	ZINC000034608502
0.2	-8.4	-6.4	1.11	2.745	0.597	4.616	1.540	18	705	ZINC000000897232
0.2	-7.5	-6.8	0.43	1.797	0.396	4.397	1.768	20	706	ZINC000022448097
0.1	-3.5	-2.9	0.30	4.641	4.001	5.331	4.034	20	707	ZINC000008101126
0.1	-4.1	-3.5	0.20	6.065	3.931	6.435	3.814	20	708	ZINC000008034120

0.1	-3.8	-3.4	0.22	3.089	1.970	4.014	1.949	20	709	ZINC000003079337
0.1	-3.3	-2.8	0.25	4.243	2.060	4.714	2.073	20	710	ZINC000000901159
0.1	-8.0	-7.1	0.52	2.189	0.483	4.711	1.190	20	711	ZINC000000105216
0.1	-4.0	-3.6	0.22	3.934	2.338	4.610	2.207	20	712	ZINC000001532517
0.1	-8.6	-7.7	0.45	2.747	0.360	5.540	0.858	20	713	ZINC000000968326
0.1	-8.2	-7.4	0.49	2.035	0.278	3.908	0.875	20	714	ZINC000000056556
0.1	-6.5	-5.7	0.39	2.483	0.907	3.345	0.930	20	715	ZINC000019166991
0.1	-7.4	-6.9	0.28	1.949	0.343	4.274	1.697	20	716	ZINC000000113415
0.1	-6.7	-5.7	0.36	2.877	1.038	3.594	1.218	20	717	ZINC000000001688
0.1	-9.0	-6.7	1.07	1.662	0.237	5.387	2.032	7	718	ZINC000003881640
0.1	-7.0	-6.5	0.35	1.947	0.339	3.682	0.621	20	719	ZINC000019875504
0.1	-8.7	-7.1	0.86	3.242	0.741	5.282	1.510	20	720	ZINC000019364229
0.1	-9.0	-7.5	0.83	2.882	0.834	4.280	1.300	19	721	ZINC000000896634
0.1	-6.8	-6.3	0.26	2.733	0.506	4.198	0.630	20	722	ZINC000001576892
0.1	-7.3	-6.7	0.34	3.048	0.742	5.150	1.520	20	723	ZINC000004214955

0.1	-7.2	-6.6	0.34	2.015	0.379	3.997	1.841	20	724	ZINC000000014007
0.1	-8.4	-6.2	1.18	2.627	0.500	5.549	1.599	20	725	ZINC000038197764
0.1	-8.3	-6.8	0.76	2.418	0.701	5.244	1.692	19	726	ZINC000100008319
0.1	-4.6	-4.3	0.13	3.052	1.851	4.140	1.866	20	727	ZINC000000895032
0.1	-8.2	-7.4	0.47	2.680	0.597	5.429	1.916	20	728	ZINC000012495062
0.1	-7.8	-7.1	0.31	2.095	0.328	3.809	0.867	20	729	ZINC000000056645
0.1	-8.3	-7.5	0.48	2.627	0.516	6.011	1.024	20	730	ZINC000003929022
0.1	-4.7	-4.1	0.24	3.051	1.147	3.525	1.165	20	731	ZINC000000114127
0.1	-7.9	-6.0	1.08	2.431	0.591	4.502	1.553	19	732	ZINC000022002218
0.1	-8.1	-7.0	0.51	2.226	0.354	4.681	1.447	20	733	ZINC000000000494
0.1	-5.4	-5.2	0.17	2.170	0.711	3.398	0.786	20	734	ZINC000003008621
0.1	-5.7	-5.1	0.27	3.701	0.957	4.871	1.318	19	735	ZINC000003830387
0.1	-9.2	-6.8	1.37	1.955	0.783	4.367	1.842	9	736	ZINC000003814395
0.1	-8.6	-7.1	0.78	2.763	0.672	5.536	1.988	20	737	ZINC000003927198
0.1	-9.2	-6.9	1.05	3.376	0.776	5.637	1.407	15	738	ZINC000004474443

0.1	-6.9	-6.1	0.43	2.389	0.477	4.976	1.296	20	739	ZINC000000403609
0.1	-6.2	-5.5	0.32	2.208	0.582	3.602	1.191	20	740	ZINC000000394284
0.1	-7.0	-6.2	0.34	2.231	0.359	4.101	1.447	20	741	ZINC000002015035
0.1	-7.4	-6.7	0.29	2.273	0.683	4.416	1.253	20	742	ZINC000002016258
0.1	-4.8	-4.4	0.23	2.527	1.460	3.672	1.283	20	743	ZINC000003831474
0.1	-4.7	-4.4	0.19	2.303	1.018	3.346	1.061	19	744	ZINC000003831477
0.1	-5.2	-5.0	0.13	3.680	1.417	4.380	1.558	20	745	ZINC000038212689
0.1	-7.1	-6.3	0.38	3.663	0.758	4.281	0.866	20	746	ZINC000006409735
0.1	-8.2	-7.2	0.44	2.614	0.518	5.119	1.223	20	747	ZINC000001530935
0.1	-8.1	-7.5	0.36	2.390	0.662	4.095	1.517	19	748	ZINC000000120319
0.1	-8.1	-7.2	0.44	2.445	0.703	4.233	1.178	20	749	ZINC000000001145
0.1	-8.3	-6.9	0.73	3.275	1.192	5.361	1.866	20	750	ZINC000008383240
0.1	-8.9	-7.2	1.02	1.821	0.218	4.664	1.943	10	751	ZINC000003875980
0.1	-8.6	-7.5	0.60	2.757	0.495	4.931	1.218	20	752	ZINC000003813061
0.1	-7.9	-7.2	0.36	2.017	0.308	5.219	1.530	20	753	ZINC000008214703

0.1	-9.7	-7.7	1.04	3.172	0.369	4.902	0.500	18	754	ZINC000000004893
0.1	-5.6	-5.1	0.21	3.449	1.137	4.305	1.326	20	755	ZINC000006661227
0.1	-8.9	-8.3	0.42	2.404	0.596	4.766	1.655	20	756	ZINC000000002279
0.1	-5.7	-5.0	0.23	3.010	1.029	3.616	1.098	20	757	ZINC000000001795
0.1	-8.4	-6.6	1.14	1.781	0.574	5.483	3.205	15	758	ZINC000003798734
0.1	-5.1	-4.6	0.22	3.796	1.610	4.722	1.706	20	759	ZINC000000901736
0.1	-6.9	-6.5	0.28	2.208	0.492	3.781	0.832	19	760	ZINC000022010375
0.1	-9.9	-8.4	0.63	3.101	0.672	5.429	1.235	20	761	ZINC000031274852
0.1	-8.8	-7.1	1.06	2.707	0.594	4.190	1.459	20	762	ZINC000004475353
0.1	-7.5	-6.8	0.35	3.756	0.897	4.858	0.694	20	763	ZINC000001530599
0.1	-6.2	-5.7	0.24	3.585	0.731	4.158	0.818	19	764	ZINC000004640636
0.1	-8.6	-7.5	0.61	2.705	0.495	5.541	1.640	20	765	ZINC000001542392
0.1	-8.3	-7.4	0.52	2.339	0.812	4.091	1.695	20	766	ZINC000000057534
0.1	-9.1	-7.0	1.13	2.039	0.345	4.458	0.504	15	767	ZINC000000000509
0.1	-7.1	-6.7	0.24	2.977	0.744	5.001	1.334	20	768	ZINC000000056653

0.1	-8.9	-8.0	0.46	1.818	0.375	4.562	1.231	20	769	ZINC000000968274
0.1	-10.0	-8.1	1.07	2.984	0.344	4.883	0.582	14	770	ZINC000000001261
0.1	-8.4	-7.8	0.31	2.717	0.775	4.879	1.162	20	771	ZINC000000057278
0.1	-7.5	-6.8	0.34	2.431	0.509	4.508	1.335	20	772	ZINC000000001505
0.1	-7.0	-6.4	0.40	2.303	0.432	5.331	1.688	20	773	ZINC000001530718
0.1	-8.9	-6.9	1.23	4.006	0.733	6.876	1.414	15	774	ZINC000003776970
0.1	-4.9	-4.6	0.19	3.301	0.668	4.086	0.735	20	775	ZINC000001529425

Table S5. BChE inhibitors from FDA-approved dataset (20 modes).

Ea diff.	Ea 1st	Ea mean	Ea SD	rmsd/l.b. mean	rmsd/l.b. SD	rmsd/u.b. mean	rmsd/u.b. SD	no. modes	no. rank	ZINC ID#
82.3	26.7	26.7	0.00	0.000	0.000	0.000	0.000	1	1	ZINC000253387843
78.3	7.3	8.5	1.41	1.768	0.724	6.713	5.413	3	2	ZINC000150338819
72.0	13.1	14.8	1.70	2.275	0.000	9.998	0.000	2	3	ZINC000096006018
69.5	76.9	77.1	0.20	0.882	0.000	1.420	0.000	2	4	ZINC000252286878
67.5	5.0	5.1	0.10	1.240	0.000	1.422	0.000	2	5	ZINC000169621230

66.2	76.9	79.4	1.75	2.244	1.433	7.301	5.869	3	6	ZINC000252286877
65.8	95.1	95.1	0.00	0.000	0.000	0.000	0.000	1	7	ZINC000252286875
65.6	13.0	14.9	1.73	2.431	0.290	7.397	2.751	4	8	ZINC000169677008
65.5	4.3	6.1	1.47	2.038	0.333	7.833	4.925	3	9	ZINC000169289767
62.4	-3.3	-1.6	1.70	2.180	0.000	4.399	0.000	2	10	ZINC000203686879
62.3	-5.5	-4.5	1.05	1.587	0.000	5.330	0.000	2	11	ZINC000169621215
57.9	14.2	14.2	0.00	0.000	0.000	0.000	0.000	1	12	ZINC000169621228
57.6	-5.3	-3.8	1.15	1.597	0.085	2.101	0.079	3	13	ZINC000169621223
51.5	-8.6	-7.3	1.07	2.134	0.308	6.655	2.900	12	14	ZINC000085432544
50.7	7.1	8.6	1.55	1.039	0.000	1.622	0.000	2	15	ZINC000169621231
49.1	-2.6	-0.8	1.80	3.035	0.000	8.620	0.000	2	16	ZINC000169621200
48.4	-9.7	-8.3	1.14	1.918	0.367	5.906	3.394	9	17	ZINC000085432549
48.1	-10.4	-8.6	0.97	2.186	0.249	8.160	2.304	11	18	ZINC000087496429
45.1	81.1	82.7	1.60	0.882	0.000	1.107	0.000	2	19	ZINC000252286876
42.0	-10.0	-8.6	1.19	1.930	0.731	6.460	4.269	3	20	ZINC000096006024

40.1	20.7	22.6	1.25	2.627	0.245	8.109	0.213	4	21	ZINC000169289388
40.1	-8.1	-6.4	0.89	2.030	0.251	8.365	2.783	20	22	ZINC000150601177
37.8	-3.4	-1.4	1.47	2.131	0.612	4.752	2.104	4	23	ZINC000095617678
37.5	-7.1	-4.9	1.09	1.938	0.301	6.404	3.606	15	24	ZINC000150588351
36.6	-4.7	-2.8	1.29	2.595	1.040	5.714	2.674	8	25	ZINC000150338708
36.5	-8.9	-7.3	1.25	2.198	0.219	7.221	2.351	8	26	ZINC000085555528
35.1	-11.9	-10.2	1.02	2.422	0.256	6.376	2.942	19	27	ZINC000253632968
33.4	-10.4	-8.0	1.43	2.106	0.478	5.104	2.478	15	28	ZINC000009574770
33.0	-11.8	-9.5	1.21	2.471	0.596	5.851	2.800	10	29	ZINC000164760756
32.9	-5.2	-3.5	1.39	2.500	0.077	5.784	0.002	3	30	ZINC000253630390
32.3	-8.5	-7.0	1.27	2.791	0.209	6.773	1.876	10	31	ZINC000053683271
31.8	-8.0	-5.8	1.10	2.465	0.517	5.953	2.311	11	32	ZINC000218037687
31.4	-6.4	-4.4	1.18	2.362	0.239	7.624	1.070	5	33	ZINC000169344691
27.9	-5.9	-3.9	1.30	2.948	0.188	7.950	0.466	4	34	ZINC000085536990
27.2	-6.5	-5.2	0.84	3.218	0.353	7.926	1.597	8	35	ZINC000164528615

26.0	-10.2	-8.0	1.08	2.323	0.552	7.067	3.359	15	36	ZINC000085537011
25.7	-7.8	-5.6	1.16	1.934	0.240	6.979	1.176	7	37	ZINC000238809662
25.4	-8.6	-6.6	0.93	2.215	0.245	6.614	2.649	18	38	ZINC000095617679
24.9	-7.2	-4.5	1.27	2.597	0.397	5.829	2.333	7	39	ZINC000059111167
24.7	-8.2	-6.2	1.32	1.934	0.255	7.027	1.565	18	40	ZINC000238809665
23.7	-5.3	-3.0	1.00	2.141	0.234	7.065	1.261	9	41	ZINC000169289411
23.5	-8.4	-5.8	1.08	1.763	0.309	8.105	1.795	19	42	ZINC000238809663
23.0	-9.9	-8.7	1.22	1.865	0.413	6.689	3.332	10	43	ZINC000004097404
22.9	-8.7	-7.6	1.05	0.957	0.000	1.534	0.000	2	44	ZINC000085537026
22.9	-9.8	-8.5	0.90	1.665	0.276	6.355	3.229	9	45	ZINC000004097383
22.8	-9.1	-7.7	1.79	2.205	0.754	4.861	3.001	3	46	ZINC000169621220
22.2	-11.2	-9.0	1.15	2.620	0.386	7.311	2.817	17	47	ZINC000001612996
22.1	-8.2	-5.6	1.10	1.706	0.239	5.910	1.936	15	48	ZINC000096006013
21.6	-9.3	-6.9	1.32	2.315	0.677	5.503	2.652	8	49	ZINC000261527196
21.6	-9.0	-7.7	1.02	2.232	0.648	5.018	3.292	3	50	ZINC000008220909

21.5	-8.3	-6.4	1.28	1.970	0.465	5.915	3.883	6	51	ZINC000242548690
21.1	-12.1	-10.4	0.95	3.146	0.794	7.107	2.219	19	52	ZINC000150338755
19.8	-10.0	-7.7	1.48	2.361	0.770	4.750	2.334	4	53	ZINC000150338699
19.7	-7.8	-5.3	1.02	1.935	0.132	8.089	1.474	19	54	ZINC000238809664
19.4	-13.2	-11.8	0.67	2.408	0.334	6.779	2.092	19	55	ZINC000003978005
19.3	-7.8	-5.6	1.17	2.119	0.466	5.497	2.703	12	56	ZINC000085534098
18.6	-10.1	-8.6	1.02	2.645	0.227	7.232	1.260	15	57	ZINC000164760874
18.4	-12.7	-9.8	0.98	2.369	0.549	6.531	2.709	19	58	ZINC000203757351
18.3	-7.9	-5.7	1.20	2.103	0.532	6.419	2.039	9	59	ZINC000085534336
17.7	-9.4	-8.1	0.99	1.937	0.367	5.506	3.361	19	60	ZINC000053229445
17.4	-8.0	-5.7	1.03	2.605	0.418	6.946	2.030	10	61	ZINC000054053579
16.4	-11.5	-10.3	0.57	2.744	0.652	6.970	2.619	20	62	ZINC000053683151
15.6	-9.5	-7.0	1.04	3.047	0.786	6.470	1.016	16	63	ZINC000306122005
15.5	-10.9	-9.2	1.17	2.533	0.611	6.503	3.406	15	64	ZINC000066166864
15.0	-6.3	-4.7	1.11	3.178	0.136	9.518	0.178	4	65	ZINC000095551509

14.7	-11.8	-9.0	1.10	3.103	0.292	6.943	1.178	17	66	ZINC000100013130
13.8	-9.5	-8.1	0.93	2.808	0.505	7.589	3.300	19	67	ZINC000014879972
13.5	-10.5	-7.9	1.08	2.396	0.496	6.812	2.688	12	68	ZINC000004099009
13.3	-10.2	-8.3	0.90	2.224	0.324	6.621	2.765	20	69	ZINC000068204830
13.0	-9.2	-7.0	1.22	3.303	0.898	6.243	1.141	18	70	ZINC000254134439
12.6	-12.0	-11.0	0.52	2.578	0.352	6.767	2.818	20	71	ZINC000052955754
12.6	-10.8	-9.4	0.44	3.220	0.533	7.204	1.609	20	72	ZINC000008101127
12.3	-9.7	-8.6	0.61	3.012	0.674	6.770	2.431	19	73	ZINC000003938482
11.6	-10.1	-8.4	0.77	3.176	0.971	6.933	2.593	19	74	ZINC000028232755
11.1	-10.1	-8.3	1.01	3.135	1.070	6.519	2.533	20	75	ZINC000011616852
11.1	-11.8	-10.7	0.52	2.479	0.648	5.779	2.413	20	76	ZINC000014261579
10.9	-11.7	-10.2	0.53	2.115	0.392	6.012	3.391	19	77	ZINC000012503187
10.6	-10.1	-8.9	0.51	1.980	0.355	6.968	3.509	20	78	ZINC000087496092
10.4	-11.2	-9.8	0.51	2.701	0.238	6.465	1.160	20	79	ZINC000004097344
9.7	-10.5	-9.4	0.56	2.658	0.561	6.421	2.934	20	80	ZINC000028639340

9.7	-11.5	-10.7	0.41	1.857	0.347	4.830	2.846	20	81	ZINC000022443609
9.4	-9.9	-8.9	0.38	1.910	0.363	5.368	3.395	18	82	ZINC000094566092
9.4	-11.1	-9.6	0.85	2.450	0.525	6.136	3.039	20	83	ZINC000004099008
9.2	-9.3	-8.2	0.72	3.918	0.809	7.985	1.708	20	84	ZINC000049783788
9.0	-8.5	-6.0	1.05	1.905	0.202	7.474	1.995	20	85	ZINC000256097218
8.9	-10.3	-8.5	0.63	4.047	0.736	7.941	1.528	20	86	ZINC000028232750
8.8	-11.2	-10.5	0.31	2.432	0.405	7.279	2.397	20	87	ZINC000027990463
8.4	-10.5	-9.8	0.35	2.198	0.250	6.456	1.779	20	88	ZINC000085537014
8.4	-11.2	-9.5	0.55	3.137	0.757	7.040	2.685	20	89	ZINC000004097343
8.2	-10.2	-9.4	0.49	3.078	0.672	5.763	1.515	20	90	ZINC000003993846
8.1	-10.5	-9.2	0.62	2.748	0.374	5.971	1.461	20	91	ZINC000028232746
8.1	-10.4	-9.7	0.34	2.448	0.611	5.823	1.666	20	92	ZINC000049637509
8.0	-9.9	-9.2	0.45	2.553	0.593	5.822	2.082	20	93	ZINC000003920266
7.7	-9.9	-8.1	1.08	3.036	0.727	6.179	2.288	20	94	ZINC000043200832
7.6	-10.6	-9.4	0.49	3.699	0.842	6.476	2.227	20	95	ZINC000003938704

7.6	-9.9	-8.7	0.70	2.243	0.318	6.858	3.750	20	96	ZINC000096309558
7.5	-10.5	-8.9	0.64	2.517	0.487	6.135	2.441	20	97	ZINC000003938684
7.4	-10.4	-9.4	0.36	1.962	0.322	6.502	2.145	20	98	ZINC000049841054
7.2	-10.5	-9.9	0.30	2.821	0.866	5.737	3.033	19	99	ZINC000049036447
7.1	-10.7	-10.0	0.31	2.828	0.615	5.956	2.085	20	100	ZINC000148723177
6.8	-9.8	-9.1	0.32	3.376	0.625	5.942	2.013	20	101	ZINC000095616937
6.7	-10.0	-9.0	0.46	2.125	0.311	5.981	1.674	20	102	ZINC000096903163
6.7	-10.9	-9.8	0.43	2.325	0.314	7.040	0.836	20	103	ZINC000003914596
6.7	-11.5	-10.2	0.46	2.292	0.379	6.686	1.464	20	104	ZINC000026985532
6.7	-9.6	-8.6	0.60	2.969	0.426	6.486	2.098	20	105	ZINC000003918087
6.6	-12.4	-10.6	0.68	2.425	0.806	5.112	2.799	20	106	ZINC000000896717
6.6	-8.9	-8.1	0.39	2.917	0.447	6.064	2.373	20	107	ZINC000003989268
6.6	-7.2	-6.3	0.46	1.813	0.092	6.333	0.808	20	108	ZINC000256097227
6.6	-11.1	-10.0	0.63	2.690	0.482	5.334	1.501	20	109	ZINC000003915154
6.6	-8.9	-7.7	0.74	2.594	0.408	5.254	0.997	20	110	ZINC000100371951

6.3	-11.6	-10.4	0.52	2.865	1.366	5.265	2.989	20	111	ZINC000003932831
6.3	-9.7	-8.1	0.55	2.074	0.308	5.315	1.849	20	112	ZINC000003941496
6.2	-12.1	-11.3	0.46	2.378	0.358	5.981	1.551	20	113	ZINC000100378061
6.2	-8.4	-8.0	0.24	2.443	0.699	6.468	2.047	20	114	ZINC000028108825
6.2	-8.9	-7.8	0.55	2.380	0.317	6.300	1.298	20	115	ZINC000150338698
6.2	-9.6	-8.3	0.44	3.016	0.612	6.996	2.360	20	116	ZINC000029571072
6.1	-9.8	-9.0	0.38	3.424	0.448	6.060	1.076	20	117	ZINC000003782599
6.1	-7.3	-6.5	0.48	1.856	0.263	6.528	2.162	20	118	ZINC000256097213
6.0	-9.2	-8.8	0.23	3.265	0.614	7.175	2.605	20	119	ZINC000096006023
5.9	-11.8	-10.9	0.49	2.415	0.501	6.483	1.606	20	120	ZINC000026664090
5.9	-9.6	-8.7	0.38	2.494	0.640	5.789	2.065	20	121	ZINC000008214681
5.9	-9.9	-8.8	0.56	2.924	0.696	6.277	1.610	19	122	ZINC000003917708
5.8	-8.9	-8.2	0.35	2.478	0.381	5.661	2.049	20	123	ZINC000060183170
5.8	-10.8	-9.9	0.31	2.395	0.396	5.706	2.040	20	124	ZINC000003977978
5.7	-9.9	-8.9	0.56	2.874	0.641	6.141	2.318	20	125	ZINC000052245489

5.7	-8.7	-8.2	0.22	3.423	0.640	7.855	1.774	20	126	ZINC000060392785
5.6	-10.8	-9.2	0.72	2.634	0.534	6.052	1.404	20	127	ZINC000003913937
5.6	-11.1	-9.7	0.57	3.100	0.585	8.371	3.177	20	128	ZINC000084668739
5.4	-9.5	-8.4	0.57	2.223	0.624	6.046	3.174	20	129	ZINC000003938746
5.4	-9.8	-9.1	0.38	2.379	0.340	6.504	2.214	20	130	ZINC000003944422
5.4	-9.8	-8.3	0.49	2.787	0.400	6.105	1.612	19	131	ZINC000014879992
5.3	-10.6	-9.5	0.38	2.271	0.276	6.919	1.623	20	132	ZINC000029416466
5.2	-10.9	-10.1	0.31	2.071	0.320	5.636	2.376	20	133	ZINC000003951740
5.2	-11.0	-9.8	0.47	2.445	0.538	4.631	0.918	20	134	ZINC000011681563
5.1	-10.8	-10.0	0.37	3.204	0.343	6.227	1.573	20	135	ZINC000003977777
5.0	-7.5	-6.4	0.60	1.933	0.144	6.229	2.502	20	136	ZINC000256097222
5.0	-9.3	-8.4	0.43	2.607	0.400	5.794	2.723	19	137	ZINC000001611274
5.0	-10.3	-8.9	0.63	3.012	0.769	5.531	1.774	20	138	ZINC000003831128
4.9	-10.2	-9.6	0.29	2.513	0.959	5.213	2.420	20	139	ZINC000000537928
4.9	-10.4	-9.4	0.37	2.961	0.236	6.639	0.713	20	140	ZINC000001538857

4.8	-11.2	-10.1	0.47	2.601	0.639	5.641	1.486	20	141	ZINC000003927822
4.8	-10.4	-9.1	0.46	2.952	0.417	6.240	0.777	20	142	ZINC000204073689
4.8	-10.4	-9.5	0.43	2.971	0.382	5.955	1.694	20	143	ZINC000003992105
4.8	-10.3	-9.1	0.47	2.321	0.283	5.900	1.537	20	144	ZINC000004212851
4.7	-11.4	-10.6	0.35	3.159	1.053	5.131	1.761	20	145	ZINC000003939013
4.7	-11.0	-10.5	0.29	2.551	0.358	7.275	1.776	20	146	ZINC000003833846
4.6	-10.3	-9.4	0.38	2.522	0.570	4.936	2.053	20	147	ZINC000004213474
4.6	-8.2	-7.7	0.31	2.284	0.481	5.790	2.976	20	148	ZINC000242437511
4.6	-9.7	-9.0	0.37	2.768	0.391	7.113	2.564	20	149	ZINC000009164421
4.5	-9.4	-8.9	0.27	2.330	0.538	4.817	2.015	20	150	ZINC000003938751
4.5	-10.5	-9.6	0.45	2.222	0.561	4.682	1.955	19	151	ZINC000003977981
4.5	-11.1	-10.5	0.45	3.261	0.734	8.250	2.961	20	152	ZINC000036701290
4.4	-9.6	-8.3	0.54	2.901	0.479	5.625	1.975	20	153	ZINC000008214483
4.4	-9.2	-7.0	0.89	3.514	0.774	7.049	0.728	20	154	ZINC000003873832
4.4	-11.1	-10.6	0.29	2.981	0.698	6.317	2.212	20	155	ZINC000014210876

4.3	-10.5	-10.0	0.25	2.467	0.435	5.516	1.969	20	156	ZINC000100014909
4.3	-9.7	-9.0	0.37	2.781	0.495	5.606	1.907	19	157	ZINC000084441937
4.2	-9.6	-8.7	0.40	3.208	0.444	7.895	2.890	20	158	ZINC000003830441
4.2	-10.3	-9.2	0.62	3.509	0.924	5.953	2.334	20	159	ZINC000011677837
4.2	-9.9	-9.0	0.38	2.558	0.407	5.074	1.602	18	160	ZINC000004097308
4.2	-8.7	-8.0	0.36	2.492	0.573	5.110	2.592	20	161	ZINC000242437513
4.1	-9.5	-8.8	0.39	2.917	0.834	5.708	2.818	20	162	ZINC000003871960
4.1	-8.9	-7.5	0.39	2.386	0.485	5.342	2.082	20	163	ZINC000242437514
4.1	-11.1	-9.8	0.42	2.564	0.548	5.710	1.643	20	164	ZINC000019632618
4.0	-11.8	-10.5	0.47	3.243	0.752	5.948	1.549	20	165	ZINC000100022637
4.0	-9.8	-9.1	0.41	2.706	0.668	5.362	1.658	20	166	ZINC000005752191
4.0	-8.7	-7.7	0.36	2.628	0.524	5.826	2.970	20	167	ZINC000003871923
4.0	-8.3	-7.8	0.26	3.090	0.775	5.793	1.976	20	168	ZINC000095616599
4.0	-8.5	-7.9	0.26	2.538	0.519	6.850	3.022	20	169	ZINC000095616600
4.0	-8.3	-7.7	0.23	2.095	0.428	5.684	2.812	20	170	ZINC000008551178

3.9	-10.0	-9.4	0.27	3.138	0.399	6.681	1.515	20	171	ZINC000000643138
3.9	-11.8	-10.2	0.60	3.034	0.681	6.407	1.658	20	172	ZINC000001530886
3.9	-11.3	-8.9	1.12	3.221	0.880	6.685	2.364	20	173	ZINC000043100709
3.9	-9.3	-8.5	0.38	2.643	0.581	5.975	2.278	20	174	ZINC000003914809
3.9	-9.6	-8.9	0.34	2.445	0.402	7.735	3.109	20	175	ZINC000094566093
3.8	-10.1	-9.5	0.41	3.020	0.551	6.579	1.567	20	176	ZINC000011615926
3.8	-9.7	-9.0	0.39	2.601	0.430	6.493	1.671	20	177	ZINC000003916214
3.8	-9.8	-9.2	0.22	2.351	0.490	5.325	2.090	20	178	ZINC000003977764
3.7	-8.9	-8.4	0.27	2.661	0.376	6.225	2.160	20	179	ZINC000003920027
3.7	-10.8	-9.0	0.51	2.578	0.511	6.098	1.497	20	180	ZINC000014210457
3.7	-9.9	-9.0	0.48	2.590	0.679	5.143	1.573	20	181	ZINC000004097286
3.7	-9.8	-9.2	0.31	2.874	0.718	6.311	1.995	20	182	ZINC000052509463
3.6	-11.3	-9.9	0.44	3.442	0.759	6.558	1.798	19	183	ZINC000100016058
3.6	-8.6	-8.3	0.19	4.326	1.618	6.627	2.575	20	184	ZINC000085537017
3.6	-10.3	-9.6	0.31	2.270	0.431	6.770	2.217	20	185	ZINC000003820029

3.5	-10.7	-9.9	0.33	2.061	0.311	5.944	1.911	20	186	ZINC000022448696
3.5	-7.6	-7.1	0.25	2.569	0.763	5.835	1.460	20	187	ZINC000085540223
3.5	-8.6	-7.7	0.37	2.679	0.560	6.001	1.961	20	188	ZINC000008551177
3.4	-9.4	-8.9	0.30	2.532	0.567	5.399	1.225	20	189	ZINC000100037855
3.4	-8.3	-7.6	0.31	2.396	0.326	5.841	2.335	19	190	ZINC000242437512
3.3	-9.1	-8.3	0.33	2.491	0.441	6.129	1.959	20	191	ZINC000003943279
3.3	-9.6	-8.8	0.39	2.466	0.300	5.816	1.573	19	192	ZINC000004097467
3.3	-9.6	-8.7	0.32	2.563	0.372	6.620	0.708	20	193	ZINC000004097309
3.3	-8.6	-8.0	0.25	2.451	0.382	5.747	2.021	20	194	ZINC000003812306
3.3	-10.2	-9.2	0.49	3.021	0.482	6.788	1.470	20	195	ZINC000096272772
3.3	-10.6	-9.4	0.47	2.534	0.384	6.298	1.484	20	196	ZINC000100070954
3.3	-11.0	-10.3	0.41	2.720	0.931	5.325	2.099	20	197	ZINC000003817234
3.3	-10.1	-8.8	0.50	2.706	0.501	5.232	1.691	18	198	ZINC000001484334
3.2	-11.0	-10.0	0.38	2.620	0.426	6.542	1.686	20	199	ZINC000004074875
3.1	-9.7	-9.1	0.30	3.339	0.497	6.242	1.038	20	200	ZINC000018324776

3.1	-11.8	-10.8	0.37	3.436	0.664	5.487	1.047	20	201	ZINC000040430143
3.1	-9.9	-9.5	0.22	3.428	0.834	5.701	1.684	20	202	ZINC000003797541
3.1	-10.4	-9.7	0.31	2.870	0.795	5.561	2.055	20	203	ZINC000003831151
3.1	-9.5	-8.5	0.44	2.616	0.602	6.394	1.950	20	204	ZINC000000601253
3.1	-11.0	-9.9	0.37	2.642	0.480	5.439	0.939	20	205	ZINC000001895505
3.1	-9.7	-9.1	0.28	2.572	0.691	5.378	1.476	20	206	ZINC000003995809
3.1	-9.6	-7.6	1.04	3.050	0.459	6.319	0.844	19	207	ZINC000003920657
3.0	-11.0	-10.3	0.39	3.628	0.697	6.502	1.205	20	208	ZINC000001550477
3.0	-9.5	-8.5	0.48	3.268	0.879	5.630	1.180	19	209	ZINC000030691420
3.0	-10.2	-9.5	0.36	2.296	0.666	5.505	2.960	20	210	ZINC000003784182
3.0	-9.4	-8.6	0.35	3.571	0.729	7.208	2.449	20	211	ZINC000003921872
3.0	-9.9	-8.2	0.55	3.291	0.778	5.348	1.556	19	212	ZINC000003978029
3.0	-10.0	-8.5	0.50	2.921	0.562	5.771	1.114	20	213	ZINC000003875439
2.9	-9.9	-8.8	0.42	2.334	0.710	5.469	1.942	20	214	ZINC000043450326
2.9	-9.0	-8.3	0.22	4.086	0.851	8.057	2.807	20	215	ZINC000100907004

2.8	-9.4	-7.7	0.54	2.518	0.501	5.503	0.950	19	216	ZINC000000621853
2.8	-10.9	-9.9	0.37	3.345	1.049	6.655	3.116	20	217	ZINC000001481956
2.8	-9.4	-8.8	0.27	2.559	0.694	6.073	2.262	20	218	ZINC000006858022
2.8	-7.4	-6.5	0.36	1.992	0.536	4.992	1.783	20	219	ZINC000003830943
2.8	-10.6	-9.5	0.39	3.852	0.913	6.943	2.002	20	220	ZINC000003918453
2.8	-7.6	-6.7	0.38	3.051	0.709	5.769	1.537	20	221	ZINC000003830959
2.7	-9.5	-8.6	0.44	3.084	1.116	5.090	2.140	20	222	ZINC000003787097
2.7	-7.6	-6.8	0.38	2.905	0.507	5.707	1.369	20	223	ZINC000003830958
2.7	-10.6	-9.3	0.44	3.522	0.659	5.773	1.142	20	224	ZINC000060325170
2.7	-10.6	-10.0	0.30	3.012	0.480	6.977	2.588	20	225	ZINC000070466416
2.7	-9.7	-9.2	0.30	3.337	0.443	6.483	1.754	20	226	ZINC000004212809
2.7	-7.7	-7.0	0.30	2.468	0.571	5.523	2.484	19	227	ZINC000008214692
2.7	-7.4	-7.0	0.20	2.253	0.579	5.520	1.611	20	228	ZINC000003830946
2.7	-11.5	-10.4	0.60	2.573	0.588	4.197	1.442	20	229	ZINC000000538312
2.6	-9.5	-8.3	0.51	2.897	0.779	5.884	1.878	20	230	ZINC000003800008

2.6	-9.5	-9.1	0.29	2.522	0.461	5.601	1.309	20	231	ZINC000003926298
2.6	-11.0	-9.6	0.64	3.296	0.812	6.548	2.911	20	232	ZINC000003925861
2.6	-7.6	-6.9	0.32	2.483	0.733	6.104	1.829	20	233	ZINC000003830957
2.6	-8.7	-8.1	0.32	2.111	0.441	6.129	2.435	20	234	ZINC000003876023
2.6	-9.5	-8.6	0.39	2.396	0.707	5.088	1.747	19	235	ZINC000013540519
2.5	-11.5	-10.7	0.30	2.943	0.555	6.319	1.414	20	236	ZINC000004097427
2.5	-10.5	-9.8	0.32	3.347	0.925	6.689	2.373	20	237	ZINC000100003902
2.5	-9.5	-8.4	0.49	3.524	0.792	5.999	1.197	19	238	ZINC000000537891
2.5	-11.1	-10.0	0.35	3.280	0.624	7.039	1.563	20	239	ZINC000011679756
2.5	-9.1	-8.1	0.48	2.183	0.471	4.980	2.112	20	240	ZINC000118913560
2.5	-10.0	-9.3	0.34	2.448	0.405	5.378	1.103	18	241	ZINC000003861599
2.5	-9.2	-8.2	0.45	2.428	0.577	5.447	2.360	20	242	ZINC000014210455
2.5	-8.4	-7.9	0.33	2.628	0.425	5.791	1.909	20	243	ZINC000008551179
2.4	-10.2	-9.3	0.33	3.278	0.830	7.781	2.989	20	244	ZINC000000643153
2.4	-9.9	-9.2	0.31	3.189	0.541	6.677	1.435	20	245	ZINC000019796168

2.4	-11.6	-10.6	0.36	2.890	0.453	5.200	1.560	20	246	ZINC000004214700
2.4	-9.2	-8.8	0.21	2.391	0.585	6.156	2.832	20	247	ZINC000013911941
2.4	-10.2	-8.6	0.53	4.035	1.075	5.791	1.459	19	248	ZINC000011726211
2.3	-7.5	-6.8	0.29	2.845	0.668	6.924	1.207	20	249	ZINC000003830960
2.3	-9.4	-8.7	0.38	2.600	0.609	5.042	1.401	20	250	ZINC000004083606
2.3	-9.8	-9.0	0.29	3.925	1.494	6.639	2.890	20	251	ZINC000011616925
2.3	-11.4	-11.0	0.27	3.354	0.436	6.498	1.260	20	252	ZINC000068153186
2.3	-11.0	-9.9	0.39	2.870	0.715	4.981	1.633	20	253	ZINC000000537805
2.3	-9.1	-8.6	0.22	3.368	0.850	6.952	2.268	20	254	ZINC000004641374
2.2	-9.7	-8.7	0.51	4.093	0.585	5.938	1.258	20	255	ZINC000100017856
2.2	-8.6	-7.8	0.34	2.927	0.449	5.776	1.206	19	256	ZINC000043195697
2.2	-12.8	-11.8	0.44	3.484	0.630	6.523	1.001	20	257	ZINC000006716957
2.2	-8.9	-8.0	0.31	2.388	0.760	6.028	1.479	20	258	ZINC000000643046
2.2	-9.1	-8.6	0.26	2.411	0.517	4.955	1.700	19	259	ZINC000003876136
2.2	-10.3	-9.5	0.29	2.140	0.546	6.289	1.413	20	260	ZINC000100052681

2.1	-9.4	-8.4	0.41	2.325	0.438	4.851	1.849	20	261	ZINC000003938744
2.1	-8.8	-8.3	0.22	4.118	1.091	7.336	2.352	20	262	ZINC000040899447
2.1	-9.7	-8.7	0.43	2.699	0.607	5.961	2.073	19	263	ZINC000014164617
2.1	-10.2	-9.2	0.38	2.597	0.593	5.375	1.367	20	264	ZINC000001530975
2.1	-9.6	-8.8	0.45	1.890	0.442	4.683	1.680	20	265	ZINC000095619100
2.1	-7.4	-6.6	0.40	2.677	0.676	5.935	1.227	20	266	ZINC000008035377
2.1	-9.0	-8.2	0.32	3.077	0.556	5.976	1.849	20	267	ZINC000003826253
2.0	-9.3	-8.4	0.42	2.700	0.760	5.128	2.040	20	268	ZINC000100004345
2.0	-9.3	-8.1	0.44	2.202	0.455	4.912	1.523	19	269	ZINC000011680067
2.0	-9.9	-9.1	0.44	1.878	0.293	5.028	1.515	20	270	ZINC000100052688
2.0	-10.1	-8.9	0.39	2.705	0.545	5.197	1.057	20	271	ZINC000003816514
2.0	-9.5	-8.8	0.31	2.288	0.379	5.708	1.926	20	272	ZINC000098023177
2.0	-8.7	-8.1	0.24	2.891	0.686	5.311	2.033	20	273	ZINC000003938695
2.0	-9.1	-8.6	0.35	2.090	0.479	4.737	2.482	20	274	ZINC000003914808
2.0	-5.9	-5.6	0.17	2.418	0.403	7.511	1.550	20	275	ZINC000008214629

2.0	-9.9	-9.2	0.35	3.312	0.600	5.843	1.072	20	276	ZINC000043207238
1.9	-9.2	-8.5	0.40	3.167	0.876	5.161	1.872	20	277	ZINC000013682481
1.9	-8.2	-7.5	0.40	3.169	0.968	5.129	2.157	20	278	ZINC000003830993
1.9	-8.8	-7.9	0.36	2.358	0.536	5.721	1.291	20	279	ZINC000003805768
1.9	-9.4	-8.6	0.31	2.346	0.728	6.139	3.277	20	280	ZINC000100015048
1.9	-9.1	-7.9	0.38	2.895	0.547	6.860	2.879	20	281	ZINC000004215234
1.9	-5.7	-5.2	0.24	2.409	0.254	7.032	1.663	20	282	ZINC000008214662
1.9	-10.7	-9.7	0.37	3.371	0.682	5.801	1.087	20	283	ZINC000016052277
1.9	-10.4	-9.5	0.39	2.509	0.579	4.759	1.522	18	284	ZINC000003881958
1.9	-9.4	-8.9	0.32	2.875	0.983	5.724	3.069	20	285	ZINC000034806477
1.9	-9.3	-8.6	0.29	2.838	0.507	5.832	1.754	20	286	ZINC000003920028
1.8	-10.5	-9.6	0.35	2.252	0.444	6.658	1.203	20	287	ZINC000001530788
1.8	-10.8	-9.6	0.54	3.061	0.608	5.282	1.458	20	288	ZINC000019796080
1.8	-11.2	-10.2	0.31	3.162	0.798	5.832	1.665	20	289	ZINC000222731806
1.8	-9.9	-8.8	0.39	3.641	0.647	6.141	1.312	20	290	ZINC000019418959

1.8	-10.2	-9.6	0.30	3.097	0.510	5.714	0.787	20	291	ZINC000000602632
1.8	-8.7	-8.2	0.23	3.681	0.632	6.645	1.437	19	292	ZINC000095616601
1.8	-7.3	-6.5	0.33	2.700	0.628	5.361	1.692	19	293	ZINC000150339323
1.7	-8.3	-7.8	0.26	2.360	0.298	6.030	0.975	20	294	ZINC000004393164
1.7	-7.6	-7.0	0.34	2.276	0.622	5.071	2.190	20	295	ZINC000085540215
1.7	-8.8	-7.8	0.46	3.328	0.891	4.582	1.601	20	296	ZINC000003830999
1.7	-9.8	-9.1	0.34	2.650	0.620	5.385	1.918	20	297	ZINC000003872994
1.7	-9.3	-8.7	0.34	2.679	0.677	6.350	1.723	20	298	ZINC000043450324
1.7	-9.6	-8.9	0.36	2.824	0.752	5.640	2.173	20	299	ZINC000003802417
1.7	-10.4	-9.6	0.40	2.901	0.682	4.751	1.355	20	300	ZINC000051951647
1.7	-9.4	-8.4	0.36	2.282	0.497	5.553	1.027	20	301	ZINC000100052691
1.7	-8.8	-8.3	0.29	2.693	0.431	6.332	2.715	20	302	ZINC000028467879
1.7	-10.0	-9.6	0.26	2.767	0.299	5.146	1.401	20	303	ZINC000000643143
1.7	-9.6	-8.9	0.32	2.848	0.569	5.121	1.589	20	304	ZINC000011677857
1.7	-11.0	-10.0	0.61	2.850	0.618	5.574	2.101	20	305	ZINC000072318121

1.7	-10.1	-9.3	0.41	4.296	1.500	7.065	3.103	20	306	ZINC000028957444
1.6	-11.3	-10.4	0.34	3.813	0.860	6.869	1.816	20	307	ZINC000064033452
1.6	-9.5	-8.6	0.35	3.866	1.010	6.053	1.765	20	308	ZINC000000643114
1.6	-8.5	-8.1	0.24	2.699	0.352	5.850	1.690	20	309	ZINC000004235575
1.6	-9.1	-7.9	0.52	2.626	0.762	6.211	1.522	19	310	ZINC000013763987
1.6	-9.5	-9.0	0.23	2.066	0.266	5.651	1.129	20	311	ZINC000100052685
1.6	-9.3	-8.8	0.31	2.994	0.532	5.398	1.672	20	312	ZINC000004214603
1.6	-11.6	-10.8	0.34	2.344	0.401	5.490	1.455	20	313	ZINC000014210642
1.6	-8.7	-8.2	0.24	2.388	0.457	5.645	1.161	20	314	ZINC000004097310
1.6	-10.5	-9.7	0.33	2.361	0.414	4.983	1.015	20	315	ZINC000013986658
1.6	-9.8	-8.6	0.52	2.353	0.680	5.520	2.312	19	316	ZINC000003914810
1.6	-10.0	-9.4	0.37	3.493	0.413	6.699	1.115	20	317	ZINC000011615927
1.5	-10.6	-9.6	0.31	3.112	0.553	6.138	1.265	20	318	ZINC000011616882
1.5	-8.5	-7.6	0.34	2.890	0.534	6.033	0.523	20	319	ZINC000022016976
1.5	-9.2	-8.5	0.32	2.255	0.376	5.656	2.159	20	320	ZINC000003920355

1.5	-8.6	-8.1	0.23	3.443	0.947	6.026	1.287	20	321	ZINC000001530695
1.5	-8.9	-8.4	0.33	2.683	0.501	6.846	1.824	20	322	ZINC000004212945
1.5	-9.2	-8.0	0.51	3.664	0.449	6.650	1.221	20	323	ZINC000100004343
1.5	-8.4	-7.7	0.28	3.150	0.512	5.223	1.170	20	324	ZINC000003808779
1.5	-10.3	-9.1	0.46	3.368	0.812	5.970	1.682	20	325	ZINC000003873160
1.5	-11.7	-9.8	0.68	2.662	0.512	5.715	0.858	19	326	ZINC000003993855
1.5	-8.7	-8.0	0.31	2.903	0.912	6.688	3.238	19	327	ZINC000000897251
1.5	-10.6	-9.8	0.42	2.917	0.660	5.056	1.590	20	328	ZINC000014768621
1.5	-10.7	-10.2	0.25	3.008	0.671	5.237	1.411	20	329	ZINC000011617039
1.5	-10.5	-9.8	0.41	3.136	0.497	5.657	0.924	20	330	ZINC000001999441
1.5	-9.4	-8.3	0.36	2.129	0.490	5.255	2.158	19	331	ZINC000005029557
1.5	-12.5	-10.5	0.61	3.917	0.878	6.606	1.719	20	332	ZINC000000538658
1.5	-9.2	-8.2	0.41	3.430	0.755	5.508	1.502	20	333	ZINC000019228902
1.5	-10.2	-9.3	0.45	2.364	0.721	3.870	1.582	20	334	ZINC000000538483
1.5	-8.2	-7.7	0.24	2.246	0.295	6.532	2.474	20	335	ZINC000008551180

1.5	-10.8	-9.0	0.56	3.251	1.088	5.100	1.803	20	336	ZINC000005844792
1.4	-8.6	-8.2	0.23	2.224	0.486	5.646	2.619	20	337	ZINC000004474414
1.4	-7.6	-7.0	0.32	2.675	0.677	5.320	1.799	20	338	ZINC000003830944
1.4	-9.4	-8.4	0.38	2.172	0.287	4.718	1.102	19	339	ZINC000001853205
1.4	-9.7	-8.6	0.44	3.051	0.797	5.458	1.538	19	340	ZINC000003876186
1.4	-10.7	-9.8	0.44	2.791	0.889	5.336	1.885	20	341	ZINC000011681534
1.4	-10.6	-9.6	0.39	2.940	0.392	5.896	1.167	20	342	ZINC000003872931
1.4	-9.7	-8.8	0.37	2.142	0.659	5.081	1.947	20	343	ZINC000003945984
1.4	-8.9	-8.2	0.31	3.202	0.686	6.129	1.214	20	344	ZINC000001530697
1.4	-9.0	-7.9	0.37	2.425	0.372	6.075	1.141	20	345	ZINC000014879975
1.4	-7.5	-6.5	0.31	2.718	0.666	5.880	0.926	20	346	ZINC000008214418
1.4	-7.6	-7.0	0.29	3.086	0.504	6.156	1.458	20	347	ZINC000008143864
1.4	-9.6	-9.0	0.25	3.412	0.395	6.819	1.156	20	348	ZINC000003873936
1.4	-9.7	-9.2	0.33	2.676	0.539	5.175	1.541	20	349	ZINC000034089131
1.3	-8.6	-8.3	0.22	2.882	0.615	4.542	1.256	20	350	ZINC000000538273

1.3	-9.2	-8.3	0.36	3.171	0.453	6.222	0.632	20	351	ZINC000003780893
1.3	-9.8	-9.0	0.36	3.826	0.781	6.372	2.018	20	352	ZINC000095915244
1.3	-8.2	-7.7	0.30	2.449	0.439	5.337	1.430	20	353	ZINC000003798763
1.3	-8.9	-7.7	0.48	2.822	0.358	5.829	1.049	20	354	ZINC000000607790
1.3	-9.4	-7.9	0.50	2.843	0.738	4.948	1.664	20	355	ZINC000035653009
1.3	-10.0	-8.9	0.52	2.864	0.772	5.242	1.686	20	356	ZINC000035801098
1.3	-10.8	-9.6	0.49	3.200	0.892	5.028	1.579	20	357	ZINC000000403566
1.3	-10.4	-9.6	0.46	3.341	0.400	6.694	0.933	19	358	ZINC000035328014
1.3	-9.6	-9.0	0.28	3.319	0.970	5.801	1.876	20	359	ZINC000003976838
1.3	-5.7	-5.2	0.22	2.542	0.609	6.701	2.053	20	360	ZINC000003830276
1.3	-9.8	-8.7	0.44	4.982	0.979	6.654	1.290	20	361	ZINC000000601301
1.3	-8.7	-8.0	0.39	2.458	0.580	4.426	1.698	20	362	ZINC000003607120
1.3	-9.8	-8.8	0.44	2.719	0.600	6.039	2.867	20	363	ZINC000072316335
1.3	-10.8	-10.0	0.45	3.349	1.186	5.882	1.575	20	364	ZINC000004213946
1.2	-9.5	-8.8	0.36	2.830	0.636	5.453	1.560	20	365	ZINC000022448983

1.2	-11.1	-9.6	0.52	2.543	0.683	5.253	1.240	20	366	ZINC000000608382
1.2	-9.5	-8.9	0.30	3.958	0.693	7.249	2.040	20	367	ZINC000004629876
1.2	-9.4	-8.8	0.28	2.497	0.648	4.583	1.615	20	368	ZINC000001530571
1.2	-8.5	-7.9	0.22	2.336	0.411	5.318	1.173	20	369	ZINC000003812888
1.2	-9.0	-8.1	0.34	2.908	0.965	4.622	1.957	20	370	ZINC000003831531
1.2	-10.9	-9.7	0.47	3.010	0.823	5.124	1.882	20	371	ZINC000068202099
1.2	-8.8	-8.1	0.30	2.777	0.697	5.210	1.853	19	372	ZINC000410428674
1.2	-9.7	-8.7	0.44	2.882	0.385	5.421	0.706	20	373	ZINC000003801163
1.2	-10.5	-9.6	0.42	3.039	0.732	5.956	1.817	20	374	ZINC000013831130
1.2	-9.9	-9.1	0.31	2.679	0.458	5.387	1.310	20	375	ZINC000000538065
1.2	-8.7	-7.9	0.34	3.543	0.703	6.551	1.475	20	376	ZINC000009212428
1.2	-8.4	-7.4	0.31	2.789	0.587	4.864	1.217	20	377	ZINC000003982483
1.2	-9.6	-8.7	0.35	2.514	0.426	5.527	1.357	20	378	ZINC000003798537
1.2	-9.3	-8.1	0.51	2.455	0.660	5.432	1.641	20	379	ZINC000003798757
1.2	-7.5	-7.2	0.20	2.341	0.221	5.768	2.039	20	380	ZINC000003830635

1.2	-10.2	-9.4	0.45	2.553	0.426	4.985	1.325	20	381	ZINC000000537795
1.2	-9.4	-9.0	0.19	2.795	0.544	6.480	2.914	20	382	ZINC000003986735
1.1	-9.8	-8.3	0.51	2.910	0.455	4.873	1.060	18	383	ZINC000019796158
1.1	-10.6	-9.8	0.40	2.867	0.613	6.317	2.020	20	384	ZINC000003824921
1.1	-8.8	-8.1	0.31	3.060	0.483	5.512	1.259	20	385	ZINC000019632834
1.1	-8.0	-7.4	0.27	3.153	0.635	5.459	1.663	20	386	ZINC000003978028
1.1	-8.8	-8.1	0.29	4.032	0.693	5.362	0.982	19	387	ZINC000000601254
1.1	-9.8	-8.3	0.65	2.818	0.967	5.369	1.156	19	388	ZINC000052957434
1.1	-9.6	-8.8	0.50	2.567	0.795	5.435	1.265	20	389	ZINC000085205451
1.1	-9.6	-8.3	0.46	3.342	1.101	5.355	1.806	20	390	ZINC000100018854
1.1	-10.4	-9.4	0.45	2.915	0.296	5.249	0.914	20	391	ZINC000022010649
1.1	-10.0	-9.3	0.37	3.490	1.023	5.272	1.422	20	392	ZINC000000537964
1.0	-9.9	-9.2	0.30	2.612	0.374	6.942	1.498	20	393	ZINC000003872566
1.0	-8.7	-7.7	0.34	2.993	0.724	4.841	1.677	20	394	ZINC000003922770
1.0	-9.1	-8.2	0.45	2.797	0.602	5.475	1.930	20	395	ZINC000068247389

1.0	-9.3	-8.7	0.29	3.121	0.481	5.049	0.599	20	396	ZINC000022116608
1.0	-7.5	-6.8	0.39	2.992	0.391	6.228	1.461	20	397	ZINC000003830947
1.0	-8.6	-7.9	0.36	2.877	0.635	6.348	1.513	20	398	ZINC000072267023
1.0	-9.6	-8.8	0.30	2.885	0.742	5.116	1.188	19	399	ZINC000003776633
1.0	-9.3	-8.9	0.27	3.042	1.051	5.911	1.589	20	400	ZINC000003990451
1.0	-10.7	-9.9	0.36	2.734	0.533	5.300	1.347	20	401	ZINC000003782818
1.0	-9.5	-9.1	0.19	5.447	1.630	7.663	2.390	20	402	ZINC000043100953
1.0	-9.1	-8.4	0.42	3.517	0.922	5.364	1.290	20	403	ZINC000000000931
1.0	-9.6	-8.5	0.40	2.093	0.402	4.615	1.442	20	404	ZINC000004095858
1.0	-9.4	-8.8	0.39	3.632	0.423	5.953	0.537	20	405	ZINC000003827556
1.0	-9.2	-8.6	0.26	3.512	0.691	5.810	1.072	20	406	ZINC000053683345
1.0	-7.6	-7.0	0.24	2.580	0.590	5.602	1.603	20	407	ZINC000085540219
1.0	-9.3	-8.4	0.43	2.443	0.530	5.545	1.905	20	408	ZINC000095619101
1.0	-9.1	-8.1	0.46	2.977	0.569	5.105	1.524	20	409	ZINC000003806063
0.9	-7.0	-6.5	0.28	2.212	0.758	4.954	1.766	20	410	ZINC000008143866

0.9	-10.8	-9.8	0.33	3.124	0.758	5.863	0.973	20	411	ZINC000001542113
0.9	-10.0	-9.3	0.31	3.098	0.597	5.809	0.950	20	412	ZINC000019361042
0.9	-9.1	-8.3	0.34	3.326	0.764	6.057	1.850	20	413	ZINC000000597013
0.9	-10.3	-9.4	0.41	3.393	1.199	5.443	2.283	19	414	ZINC000052716421
0.9	-9.5	-8.3	0.45	3.040	0.594	6.219	2.073	20	415	ZINC000003871978
0.9	-8.7	-7.3	0.44	3.280	1.378	4.655	1.417	20	416	ZINC000002169830
0.9	-9.6	-8.7	0.37	3.204	1.007	5.259	1.767	20	417	ZINC000000000347
0.9	-10.7	-9.7	0.48	2.904	0.806	4.640	1.354	20	418	ZINC000000002688
0.9	-8.7	-8.1	0.31	3.320	0.434	5.881	1.097	20	419	ZINC000001535101
0.9	-9.9	-9.3	0.27	3.456	1.066	5.685	2.277	20	420	ZINC000035902489
0.9	-9.5	-8.7	0.40	2.772	1.028	5.685	3.608	20	421	ZINC000018516586
0.9	-9.2	-8.5	0.30	2.504	0.292	5.539	0.686	20	422	ZINC000001552908
0.9	-9.4	-8.6	0.39	2.273	0.489	5.129	1.648	20	423	ZINC000004212854
0.9	-7.2	-6.5	0.28	2.836	0.667	4.970	0.838	20	424	ZINC000004097476
0.9	-10.1	-9.3	0.29	2.968	0.509	6.084	1.159	20	425	ZINC000003938686

0.9	-8.7	-7.6	0.39	3.916	1.244	6.040	1.708	20	426	ZINC000013129998
0.8	-9.4	-8.7	0.31	3.050	0.610	5.420	1.539	20	427	ZINC000003819138
0.8	-9.7	-9.0	0.33	3.682	0.828	7.654	2.918	20	428	ZINC000003831490
0.8	-8.4	-7.2	0.43	4.742	1.014	6.912	1.224	20	429	ZINC000000121480
0.8	-10.1	-9.1	0.34	2.695	0.565	4.931	1.176	20	430	ZINC000000897240
0.8	-7.0	-6.5	0.24	2.576	0.648	5.572	1.728	20	431	ZINC000008035395
0.8	-9.9	-9.1	0.39	2.561	0.625	5.292	1.625	20	432	ZINC000002522669
0.8	-10.1	-9.4	0.43	2.832	0.962	4.395	1.809	20	433	ZINC000001489478
0.8	-9.8	-8.7	0.38	2.927	0.487	5.321	1.040	20	434	ZINC000003814422
0.8	-10.4	-9.2	0.44	3.118	0.724	6.029	1.083	20	435	ZINC000003810860
0.8	-9.3	-8.4	0.42	2.725	0.816	4.727	1.544	20	436	ZINC000001530694
0.8	-8.1	-7.4	0.31	2.396	0.600	5.719	1.407	20	437	ZINC000022016981
0.8	-10.0	-9.4	0.35	2.995	0.493	5.371	1.375	19	438	ZINC000003977767
0.8	-11.0	-10.1	0.39	3.328	0.726	5.540	1.772	20	439	ZINC000000538550
0.8	-8.9	-8.4	0.28	2.165	0.406	5.038	1.424	20	440	ZINC000026011099

0.8	-7.0	-6.6	0.24	2.244	0.585	6.191	1.311	20	441	ZINC000003830945
0.8	-9.6	-8.8	0.34	2.635	0.805	4.769	1.770	19	442	ZINC000021981454
0.8	-8.9	-8.2	0.37	3.023	0.499	6.327	1.380	20	443	ZINC000003871967
0.8	-11.3	-10.7	0.28	2.280	0.611	4.710	1.340	20	444	ZINC000004175630
0.8	-8.4	-7.6	0.32	2.479	0.580	5.065	1.745	20	445	ZINC000003991624
0.8	-9.1	-8.0	0.44	3.402	1.095	5.409	2.132	19	446	ZINC000019632628
0.8	-8.5	-7.8	0.26	2.265	0.753	5.457	1.755	20	447	ZINC000003930376
0.7	-10.5	-9.5	0.48	3.030	0.837	6.045	1.319	20	448	ZINC000000000655
0.7	-9.9	-8.6	0.50	2.384	0.534	4.844	1.477	19	449	ZINC000003875484
0.7	-8.3	-7.6	0.32	3.229	0.522	5.411	1.153	20	450	ZINC000000621893
0.7	-9.5	-8.6	0.35	2.799	0.408	5.660	0.777	20	451	ZINC000000538621
0.7	-8.6	-7.7	0.39	3.658	0.970	6.423	1.688	20	452	ZINC000019364224
0.7	-10.0	-9.3	0.39	2.242	0.367	4.658	1.369	20	453	ZINC000001530579
0.7	-10.4	-9.4	0.47	2.908	1.034	4.328	1.669	20	454	ZINC000000897301
0.7	-9.2	-8.3	0.43	2.665	0.660	5.220	1.596	20	455	ZINC000118913562

0.7	-8.0	-7.1	0.33	2.796	0.516	5.141	1.588	20	456	ZINC000004097283
0.7	-8.8	-7.8	0.36	2.700	0.560	4.947	1.739	20	457	ZINC000003831405
0.7	-9.1	-7.9	0.50	2.702	0.712	5.016	1.300	20	458	ZINC000012404516
0.7	-7.9	-6.9	0.34	2.238	0.381	4.553	1.148	20	459	ZINC000003977952
0.7	-8.0	-7.4	0.27	3.787	0.562	6.593	1.449	20	460	ZINC000003830449
0.7	-9.5	-8.6	0.46	2.578	0.802	5.113	1.811	20	461	ZINC000030691727
0.7	-8.9	-8.1	0.33	2.405	0.473	6.171	0.945	19	462	ZINC000004102194
0.7	-8.5	-8.1	0.22	2.962	0.579	6.252	1.935	20	463	ZINC000022059926
0.7	-9.1	-8.2	0.32	3.031	0.718	5.467	1.571	20	464	ZINC000000607971
0.7	-9.0	-7.4	0.53	3.412	0.893	5.221	1.385	19	465	ZINC000003830218
0.7	-8.5	-7.9	0.28	3.195	0.598	5.592	1.510	20	466	ZINC000000537752
0.7	-11.2	-9.0	0.59	2.588	0.633	4.984	1.537	19	467	ZINC000001539579
0.7	-9.8	-9.0	0.30	2.827	0.578	5.965	1.233	20	468	ZINC000003830716
0.7	-10.5	-9.3	0.41	2.453	0.669	5.874	2.323	20	469	ZINC000003955219
0.7	-9.7	-9.0	0.38	2.864	0.742	5.335	1.601	20	470	ZINC000084758479

0.7	-9.7	-8.9	0.41	2.315	0.456	4.955	1.626	18	471	ZINC000118912517
0.7	-9.1	-8.4	0.29	2.645	0.386	5.605	1.437	19	472	ZINC000003938652
0.7	-5.8	-4.5	0.41	6.069	2.766	6.631	2.739	20	473	ZINC000100070937
0.7	-9.6	-8.7	0.41	2.140	0.571	5.539	1.697	17	474	ZINC000003875560
0.6	-7.9	-7.4	0.28	2.209	0.481	5.559	1.565	20	475	ZINC000022059268
0.6	-9.0	-8.2	0.45	2.746	0.792	6.109	2.577	20	476	ZINC000011615928
0.6	-8.9	-8.2	0.30	3.116	0.575	5.553	1.411	20	477	ZINC000019796018
0.6	-9.5	-8.8	0.37	2.655	0.552	5.460	1.731	20	478	ZINC000016159083
0.6	-10.3	-9.5	0.36	2.634	0.593	5.122	1.468	20	479	ZINC000000537791
0.6	-9.5	-8.8	0.30	3.061	0.678	5.474	1.554	20	480	ZINC000001530639
0.6	-9.8	-9.2	0.33	3.199	0.801	5.801	1.674	20	481	ZINC000001534965
0.6	-8.9	-8.3	0.27	3.889	1.064	5.470	1.567	20	482	ZINC000003927870
0.6	-9.6	-8.9	0.32	3.831	1.009	6.186	1.588	20	483	ZINC000019203912
0.6	-8.8	-8.0	0.34	2.630	0.677	5.810	1.267	20	484	ZINC000101489663
0.6	-8.1	-7.4	0.36	3.224	0.856	5.506	1.260	20	485	ZINC000000044027

0.6	-7.8	-7.3	0.24	1.923	0.391	4.233	1.201	20	486	ZINC000033965961
0.6	-8.6	-7.8	0.34	2.879	0.665	6.317	1.048	20	487	ZINC000003830212
0.6	-9.1	-8.2	0.43	2.479	0.602	5.878	1.667	20	488	ZINC000008214619
0.6	-9.9	-9.2	0.32	2.720	0.829	5.089	1.269	20	489	ZINC000100296832
0.6	-9.6	-8.5	0.48	3.721	0.698	5.883	0.799	20	490	ZINC000003812897
0.6	-9.3	-8.2	0.39	2.623	0.719	4.702	1.663	20	491	ZINC000000403011
0.6	-9.8	-8.4	0.59	2.737	0.773	5.050	1.420	20	492	ZINC000003795819
0.6	-10.8	-10.0	0.36	2.209	0.289	4.490	0.942	20	493	ZINC000003860453
0.6	-8.4	-7.9	0.29	3.384	0.758	6.154	1.754	20	494	ZINC000003830405
0.6	-8.7	-8.1	0.26	2.401	0.664	4.564	2.250	20	495	ZINC000004099032
0.6	-9.5	-8.0	0.53	4.085	1.145	5.218	1.525	20	496	ZINC000006467621
0.6	-10.8	-10.0	0.36	3.102	0.711	5.687	1.167	20	497	ZINC000027428713
0.6	-8.9	-8.4	0.24	3.585	1.486	5.361	2.251	20	498	ZINC000003830986
0.6	-9.1	-7.9	0.40	2.827	0.706	5.567	1.913	20	499	ZINC000003800475
0.5	-9.0	-8.0	0.32	2.617	0.448	5.582	1.137	20	500	ZINC000000601281

0.5	-9.0	-7.9	0.40	3.812	1.176	7.005	2.218	20	501	ZINC000003952881
0.5	-8.6	-7.7	0.40	3.682	0.990	5.877	1.755	19	502	ZINC000004676424
0.5	-8.7	-8.2	0.35	2.296	0.806	3.953	1.347	20	503	ZINC000021297660
0.5	-9.2	-8.7	0.27	2.759	0.741	4.915	1.642	20	504	ZINC000000896543
0.5	-10.2	-8.5	0.49	3.450	0.966	6.388	1.277	20	505	ZINC000000896557
0.5	-8.5	-7.6	0.37	3.347	0.878	4.701	1.307	20	506	ZINC000000402909
0.5	-9.1	-8.8	0.18	3.977	0.668	7.207	1.904	20	507	ZINC000001529323
0.5	-8.4	-7.9	0.27	2.492	0.600	5.882	1.568	20	508	ZINC000049933061
0.5	-9.4	-9.0	0.23	2.610	0.578	5.861	1.810	20	509	ZINC000003807172
0.5	-9.8	-9.1	0.39	3.035	0.652	5.429	1.736	20	510	ZINC000003819392
0.5	-9.8	-9.2	0.33	3.375	1.044	6.993	3.061	20	511	ZINC000058581064
0.5	-8.0	-7.2	0.34	3.297	0.671	5.522	1.002	20	512	ZINC000003875392
0.5	-8.3	-7.4	0.38	2.810	0.749	5.045	1.023	20	513	ZINC000096942202
0.5	-9.2	-8.5	0.32	2.826	0.622	4.864	1.414	20	514	ZINC000003873371
0.5	-8.1	-7.2	0.35	3.296	0.704	5.393	1.293	20	515	ZINC000008552123

0.5	-9.0	-8.1	0.42	3.247	0.656	5.992	1.591	20	516	ZINC000000602128
0.5	-5.9	-5.1	0.34	4.859	2.555	6.619	2.593	20	517	ZINC000001530810
0.5	-9.9	-8.8	0.52	3.192	0.745	5.647	1.835	20	518	ZINC000100036924
0.5	-9.3	-8.7	0.32	2.708	0.309	6.241	1.226	20	519	ZINC000145401681
0.5	-8.8	-8.2	0.29	3.071	0.696	5.958	1.190	20	520	ZINC000002005305
0.5	-9.1	-8.6	0.21	2.763	0.738	5.758	1.577	19	521	ZINC000008577218
0.5	-8.4	-7.4	0.33	3.077	0.660	5.510	0.903	20	522	ZINC000000000751
0.5	-6.1	-5.3	0.29	3.537	2.268	4.655	1.887	20	523	ZINC000000000882
0.5	-10.6	-9.8	0.35	2.582	0.649	4.916	1.248	19	524	ZINC000000601229
0.5	-8.3	-7.7	0.28	3.468	0.362	6.645	0.915	19	525	ZINC000012402836
0.5	-8.3	-7.8	0.27	2.706	0.659	5.075	1.394	20	526	ZINC000004693574
0.5	-9.0	-8.0	0.37	3.254	0.891	5.539	2.019	20	527	ZINC000004468778
0.5	-9.5	-9.1	0.23	2.812	0.697	4.321	1.448	20	528	ZINC000000606383
0.5	-8.7	-8.3	0.17	2.594	0.732	5.209	1.451	20	529	ZINC000004474682
0.5	-9.1	-8.5	0.30	4.148	1.253	6.396	0.808	20	530	ZINC000001530741

0.5	-9.2	-8.5	0.31	2.444	0.569	4.978	1.355	18	531	ZINC000253530025
0.5	-8.8	-7.7	0.43	2.571	0.473	4.549	1.149	20	532	ZINC000030691763
0.4	-8.8	-8.1	0.28	2.932	0.850	5.120	1.794	20	533	ZINC000003871703
0.4	-10.0	-9.2	0.27	2.506	0.839	4.320	1.464	20	534	ZINC000000057522
0.4	-10.1	-9.1	0.44	3.225	0.824	5.766	1.897	20	535	ZINC000001886617
0.4	-10.1	-9.2	0.41	2.701	0.878	4.369	1.556	20	536	ZINC000103105084
0.4	-9.1	-8.2	0.36	2.628	0.544	4.902	1.334	19	537	ZINC000003831404
0.4	-8.6	-7.9	0.31	3.261	0.711	5.322	1.144	20	538	ZINC000002599970
0.4	-9.2	-8.5	0.35	2.857	0.547	6.461	1.570	20	539	ZINC000030691736
0.4	-9.0	-7.9	0.48	2.967	0.913	5.063	1.444	20	540	ZINC000100015780
0.4	-9.2	-8.5	0.38	2.534	0.553	5.516	1.158	20	541	ZINC000003861806
0.4	-9.4	-8.9	0.32	2.172	0.244	5.720	1.268	20	542	ZINC000001530580
0.4	-9.6	-8.8	0.29	3.240	0.508	5.896	1.319	20	543	ZINC000001485935
0.4	-9.7	-9.1	0.25	2.380	0.817	4.792	1.541	20	544	ZINC000001530977
0.4	-8.7	-8.0	0.35	2.855	0.995	4.519	1.774	20	545	ZINC000000057512

0.4	-10.1	-8.7	0.44	2.754	0.639	5.002	1.177	19	546	ZINC000245204949
0.4	-9.5	-8.9	0.30	2.524	0.555	4.691	1.536	20	547	ZINC000100296828
0.4	-9.5	-8.5	0.44	2.218	0.473	5.312	1.876	20	548	ZINC000004097304
0.4	-8.8	-8.1	0.32	2.429	0.779	4.322	1.902	20	549	ZINC000001843099
0.4	-9.7	-9.0	0.39	3.138	0.779	5.349	1.307	20	550	ZINC000001851149
0.4	-8.2	-7.6	0.33	2.468	0.222	5.157	0.968	20	551	ZINC000000622123
0.4	-9.5	-8.5	0.46	3.130	0.939	5.663	1.367	20	552	ZINC000000075126
0.4	-8.6	-7.9	0.31	3.016	0.564	5.110	1.809	20	553	ZINC000003830264
0.4	-9.0	-8.5	0.27	3.154	1.124	5.516	1.964	20	554	ZINC000001536109
0.4	-9.1	-8.7	0.22	2.233	0.412	5.485	1.150	20	555	ZINC000100055899
0.4	-8.2	-7.6	0.36	2.347	0.569	4.702	1.737	20	556	ZINC000053045055
0.4	-8.3	-7.4	0.26	2.554	0.416	5.175	1.373	20	557	ZINC000000897256
0.4	-9.3	-8.5	0.42	2.168	0.640	4.736	1.571	20	558	ZINC000003781943
0.4	-8.6	-7.3	0.38	2.837	0.669	4.611	1.054	20	559	ZINC000001997127
0.4	-9.8	-8.9	0.32	2.673	0.459	4.402	1.321	20	560	ZINC000000002212

0.4	-6.1	-5.2	0.46	2.502	1.229	5.026	1.862	20	561	ZINC000019364219
0.4	-7.3	-6.7	0.24	3.762	0.956	6.318	0.931	20	562	ZINC000012414057
0.4	-10.0	-9.2	0.38	4.131	0.875	6.439	1.433	20	563	ZINC000000025958
0.4	-3.7	-3.1	0.28	6.466	3.327	6.972	3.323	20	564	ZINC000000895318
0.4	-9.9	-8.9	0.41	2.292	0.525	6.031	1.381	20	565	ZINC000003936683
0.4	-9.1	-8.2	0.36	3.101	0.997	4.592	1.530	20	566	ZINC000000011012
0.4	-8.1	-7.5	0.29	2.329	0.411	5.354	1.246	20	567	ZINC000003871832
0.4	-9.1	-8.0	0.47	2.610	0.985	4.903	1.488	20	568	ZINC000000000941
0.4	-6.0	-5.6	0.38	3.785	3.528	5.288	3.369	20	569	ZINC000008214651
0.3	-8.8	-8.1	0.33	2.312	0.381	4.779	0.948	20	570	ZINC000100001918
0.3	-10.5	-9.2	0.41	3.584	1.299	5.980	1.677	20	571	ZINC000005844788
0.3	-8.9	-8.0	0.37	3.328	0.949	5.396	1.195	20	572	ZINC000002008310
0.3	-8.3	-7.5	0.34	3.612	0.625	6.178	0.923	19	573	ZINC000000005151
0.3	-7.5	-6.8	0.34	3.360	0.698	4.959	1.058	20	574	ZINC000004340269
0.3	-10.4	-9.8	0.34	3.590	0.912	6.050	0.887	20	575	ZINC000000968264

0.3	-8.9	-7.9	0.44	2.571	0.803	4.771	1.678	20	576	ZINC000001550766
0.3	-9.4	-8.3	0.40	2.744	0.834	4.537	1.227	20	577	ZINC000013831141
0.3	-4.1	-3.6	0.25	5.097	2.973	5.725	2.984	20	578	ZINC000004658562
0.3	-8.9	-7.7	0.39	4.141	1.241	5.410	1.526	20	579	ZINC000003798247
0.3	-9.6	-8.5	0.42	3.053	1.011	4.656	1.138	20	580	ZINC000034125842
0.3	-10.8	-10.2	0.34	2.595	0.768	4.375	1.445	20	581	ZINC000001481815
0.3	-9.9	-9.0	0.47	3.329	1.172	5.442	1.906	19	582	ZINC000000537931
0.3	-10.2	-9.7	0.28	3.062	0.488	5.556	0.601	20	583	ZINC000100016084
0.3	-5.5	-4.7	0.35	5.613	2.745	6.509	2.445	19	584	ZINC000000002028
0.3	-9.1	-8.3	0.38	4.089	0.899	5.520	0.910	20	585	ZINC000022116612
0.3	-5.8	-5.2	0.24	3.394	0.980	4.379	1.195	20	586	ZINC000004658290
0.3	-4.1	-3.6	0.25	6.048	2.939	6.784	2.979	20	587	ZINC000004658560
0.3	-9.8	-8.8	0.55	2.655	0.941	5.072	2.064	20	588	ZINC000030690433
0.3	-5.5	-4.8	0.30	3.769	1.675	4.816	1.724	20	589	ZINC000000002005
0.3	-9.5	-8.7	0.31	3.187	0.702	5.528	1.365	20	590	ZINC000001850374

0.3	-8.8	-8.2	0.24	2.843	0.433	5.425	1.051	20	591	ZINC000000896740
0.3	-9.4	-8.4	0.50	2.230	0.370	4.800	0.976	19	592	ZINC000000968375
0.3	-9.4	-8.7	0.32	3.460	0.739	5.728	1.327	20	593	ZINC000002036848
0.3	-10.2	-8.9	0.42	3.339	0.566	5.015	1.046	20	594	ZINC000000897089
0.3	-7.2	-6.1	0.39	2.912	1.296	4.489	1.561	19	595	ZINC000022010387
0.3	-10.1	-9.4	0.36	3.127	0.937	4.839	2.010	20	596	ZINC000084758235
0.2	-9.9	-9.5	0.18	2.157	0.565	5.335	1.160	20	597	ZINC000030691797
0.2	-9.2	-8.2	0.35	2.730	0.873	4.804	1.707	20	598	ZINC000034051848
0.2	-9.5	-8.7	0.32	2.744	0.547	5.736	1.892	20	599	ZINC000019594557
0.2	-9.7	-8.7	0.47	3.847	0.767	6.527	1.504	20	600	ZINC000043206370
0.2	-9.5	-8.9	0.25	3.535	1.062	5.771	1.783	20	601	ZINC000003873921
0.2	-7.4	-6.7	0.28	2.992	1.202	5.056	1.409	20	602	ZINC000000057146
0.2	-7.8	-7.3	0.29	2.785	0.487	5.592	2.270	20	603	ZINC000004468780
0.2	-7.6	-6.7	0.34	2.972	1.110	4.867	1.206	20	604	ZINC000000057147
0.2	-5.6	-4.7	0.33	3.472	1.650	4.818	1.642	20	605	ZINC000012859773

0.2	-9.4	-8.7	0.30	2.798	0.722	4.779	1.094	20	606	ZINC000003823475
0.2	-9.2	-8.5	0.26	3.312	0.365	5.465	0.594	20	607	ZINC000012503291
0.2	-6.0	-5.4	0.30	3.188	0.871	5.025	1.241	20	608	ZINC000001530303
0.2	-8.2	-7.3	0.51	3.249	0.957	5.623	2.074	20	609	ZINC000003806413
0.2	-4.4	-4.0	0.22	5.581	2.622	6.696	2.444	20	610	ZINC000001532805
0.2	-7.5	-6.4	0.41	3.041	1.297	5.057	1.618	19	611	ZINC000001530817
0.2	-7.2	-6.3	0.34	3.555	1.433	4.592	1.715	20	612	ZINC000000012346
0.2	-7.7	-7.2	0.26	2.307	0.536	5.177	0.976	20	613	ZINC000003831138
0.2	-7.3	-6.7	0.28	3.039	0.620	4.339	1.211	20	614	ZINC000001530621
0.2	-9.4	-9.2	0.18	2.776	0.547	5.628	1.165	20	615	ZINC000100018594
0.2	-8.8	-8.2	0.37	3.028	0.824	5.254	2.663	20	616	ZINC000003964325
0.2	-10.0	-9.5	0.24	2.526	0.724	5.316	1.890	20	617	ZINC000000538275
0.2	-9.0	-8.5	0.34	2.858	1.042	4.891	1.695	20	618	ZINC000000009689
0.2	-9.4	-8.8	0.33	2.129	0.588	5.233	1.917	20	619	ZINC000000601317
0.2	-8.9	-8.4	0.26	3.096	0.437	5.159	1.138	20	620	ZINC000000538266

0.2	-8.0	-7.5	0.22	2.434	0.407	4.768	0.809	20	621	ZINC000003806104
0.2	-3.7	-3.3	0.20	5.193	3.754	5.968	3.638	20	622	ZINC000004658557
0.2	-4.1	-3.5	0.26	5.335	2.710	5.641	2.759	20	623	ZINC000000895042
0.2	-8.1	-7.0	0.34	2.467	0.608	3.793	1.239	20	624	ZINC000003779042
0.2	-8.7	-8.2	0.33	2.569	0.289	5.396	1.120	20	625	ZINC000003812863
0.2	-5.4	-4.8	0.32	4.034	2.654	5.364	2.591	20	626	ZINC000003812960
0.2	-9.3	-8.5	0.32	2.788	0.473	5.075	1.358	20	627	ZINC000003873295
0.2	-7.5	-6.6	0.31	3.682	1.417	5.073	1.814	20	628	ZINC000017146904
0.2	-8.4	-7.7	0.33	3.144	0.531	4.926	1.023	20	629	ZINC000000897385
0.2	-8.7	-7.9	0.28	2.304	0.504	5.240	1.520	20	630	ZINC000004474405
0.2	-4.0	-3.5	0.26	6.329	3.061	6.614	3.082	20	631	ZINC000004658603
0.2	-8.5	-7.6	0.39	3.167	0.967	4.750	1.594	20	632	ZINC000004099200
0.2	-5.3	-4.5	0.26	4.173	1.272	5.122	1.168	20	633	ZINC000003831551
0.2	-5.7	-4.9	0.39	5.419	2.550	6.169	2.260	20	634	ZINC000000895302
0.2	-9.3	-8.7	0.37	3.364	0.753	5.827	1.339	20	635	ZINC000095619105

0.2	-4.6	-4.0	0.23	4.227	1.762	5.198	1.917	20	636	ZINC000000895103
0.2	-5.7	-5.5	0.14	4.074	1.765	5.148	1.949	20	637	ZINC000001532525
0.2	-9.6	-8.9	0.40	2.774	1.351	5.045	1.106	19	638	ZINC000000968310
0.2	-5.5	-4.9	0.30	3.935	2.575	5.239	2.706	20	639	ZINC000004097392
0.2	-6.5	-5.7	0.35	4.016	1.528	5.270	1.664	20	640	ZINC000003813042
0.2	-4.5	-4.0	0.22	5.834	2.614	6.735	2.367	20	641	ZINC000000599985
0.2	-3.6	-3.2	0.22	6.780	4.345	7.011	4.405	20	642	ZINC000004658552
0.2	-9.6	-8.7	0.37	2.977	0.784	6.285	2.403	20	643	ZINC000004474460
0.2	-7.9	-6.8	0.41	3.288	1.312	4.539	1.673	20	644	ZINC000018279854
0.2	-9.4	-8.5	0.50	2.959	0.944	5.218	1.722	20	645	ZINC000013537284
0.2	-8.0	-6.8	0.44	3.341	0.556	4.754	1.035	19	646	ZINC000003972949
0.2	-7.5	-6.9	0.30	2.850	0.620	5.928	2.143	20	647	ZINC000003830453
0.2	-10.3	-9.5	0.38	3.110	0.751	5.301	1.464	20	648	ZINC000052509366
0.2	-4.7	-4.2	0.32	3.329	1.596	5.368	1.673	20	649	ZINC000019364225
0.2	-9.3	-8.2	0.47	3.406	1.353	5.439	1.376	20	650	ZINC000000001331

0.2	-9.5	-8.9	0.28	3.141	0.998	6.070	1.674	20	651	ZINC000003830321
0.2	-4.3	-4.0	0.25	5.071	4.089	6.227	3.699	20	652	ZINC000000896695
0.2	-8.0	-7.4	0.22	2.271	0.374	5.043	1.368	20	653	ZINC000003812913
0.2	-5.4	-4.9	0.31	3.471	2.674	5.024	2.798	20	654	ZINC000003776875
0.2	-9.2	-8.4	0.41	2.716	0.644	5.040	1.390	20	655	ZINC000001996784
0.1	-7.9	-7.2	0.28	2.409	0.431	4.686	1.136	20	656	ZINC000012503177
0.1	-8.1	-7.6	0.31	2.938	0.635	4.930	1.429	20	657	ZINC000000004028
0.1	-9.3	-8.6	0.34	2.117	0.455	4.471	1.222	19	658	ZINC000004097416
0.1	-8.7	-8.1	0.34	3.384	0.933	4.825	1.236	20	659	ZINC000005733652
0.1	-8.5	-7.6	0.41	2.990	0.741	4.982	1.616	20	660	ZINC000001995484
0.1	-7.1	-6.3	0.29	3.944	1.114	5.938	1.286	20	661	ZINC000003977737
0.1	-7.0	-6.4	0.27	2.376	0.407	5.677	1.459	20	662	ZINC000001530762
0.1	-6.2	-5.2	0.43	5.058	2.203	5.945	2.039	20	663	ZINC000012503156
0.1	-9.3	-8.4	0.48	3.720	1.514	5.369	2.445	20	664	ZINC000051133897
0.1	-10.2	-9.5	0.33	3.703	0.489	5.864	1.156	20	665	ZINC000000607986

0.1	-9.8	-9.2	0.30	3.041	0.950	4.859	1.374	20	666	ZINC000028973446
0.1	-8.9	-8.3	0.24	3.071	0.276	5.635	1.110	20	667	ZINC000029319828
0.1	-8.6	-7.9	0.34	2.434	0.607	5.458	1.682	20	668	ZINC000003791297
0.1	-9.1	-8.4	0.36	3.036	0.678	5.059	1.638	20	669	ZINC000002016037
0.1	-5.4	-5.1	0.17	2.530	1.244	4.613	1.358	20	670	ZINC000002041302
0.1	-8.8	-8.2	0.28	3.441	0.987	5.031	1.461	20	671	ZINC000000000740
0.1	-9.9	-9.0	0.30	4.046	1.411	5.563	1.950	20	672	ZINC000002570895
0.1	-8.8	-8.3	0.29	2.203	0.668	4.811	1.809	20	673	ZINC000001530761
0.1	-7.8	-7.2	0.25	2.323	0.382	5.181	1.217	20	674	ZINC000019632718
0.1	-10.1	-9.5	0.30	4.444	0.699	7.430	1.128	20	675	ZINC000003816287
0.1	-4.7	-3.9	0.30	5.215	2.762	5.745	2.854	20	676	ZINC000000114124
0.1	-8.4	-7.4	0.40	4.125	1.395	5.821	1.364	20	677	ZINC000003806262
0.1	-6.2	-5.6	0.36	4.244	1.724	5.653	1.697	20	678	ZINC000000000353
0.1	-6.1	-5.4	0.26	3.937	2.493	4.800	2.214	20	679	ZINC000013298313
0.1	-3.9	-3.6	0.17	4.897	1.845	5.414	1.883	20	680	ZINC000001187543

0.1	-8.6	-7.9	0.38	3.102	0.701	6.287	2.155	20	681	ZINC000022059930
0.1	-7.3	-6.8	0.21	2.580	0.780	4.361	1.969	20	682	ZINC000043194409
0.1	-9.9	-9.0	0.40	2.680	0.466	5.466	1.455	19	683	ZINC000004097305
0.1	-5.5	-4.8	0.30	3.948	1.125	5.172	1.403	20	684	ZINC000001554588
0.1	-4.9	-4.5	0.22	4.467	1.706	5.742	1.658	20	685	ZINC000003079340
0.1	-3.5	-3.2	0.22	5.146	3.641	5.746	3.593	20	686	ZINC000000895316
0.1	-9.5	-8.5	0.33	2.466	0.518	4.538	1.854	20	687	ZINC000003978006
0.1	-4.7	-4.1	0.26	4.500	1.935	5.751	1.780	20	688	ZINC000000388081
0.1	-6.4	-5.6	0.31	5.310	1.362	6.700	1.095	20	689	ZINC000000113355
0.1	-5.7	-4.9	0.34	4.015	1.613	4.848	1.644	20	690	ZINC000052971887
0.1	-7.9	-7.3	0.30	3.483	0.734	5.915	1.552	20	691	ZINC000004038341
0.1	-9.3	-8.8	0.27	3.519	0.692	5.875	1.354	20	692	ZINC000000599734
0.1	-4.1	-3.8	0.17	5.856	2.368	6.237	2.391	20	693	ZINC000003809490
0.1	-8.7	-8.0	0.40	2.846	0.771	5.011	1.558	20	694	ZINC000003812851
0.1	-7.2	-6.4	0.24	3.829	1.171	4.836	1.434	20	695	ZINC000003629271

0.1	-5.5	-5.1	0.19	3.477	1.189	4.777	1.197	20	696	ZINC000003812862
0.1	-5.3	-4.7	0.25	6.188	2.775	7.180	2.253	20	697	ZINC000005133378
0.1	-7.8	-7.4	0.18	2.495	0.430	5.344	1.165	20	698	ZINC000027641461
0.1	-9.4	-8.5	0.35	3.479	0.830	5.638	1.734	20	699	ZINC000000121541
0.1	-9.2	-8.6	0.24	3.374	0.532	5.510	1.080	20	700	ZINC000003816292
0.1	-9.5	-8.7	0.40	2.221	0.513	4.942	1.907	20	701	ZINC000003812841
0.1	-8.1	-7.2	0.41	4.274	0.911	6.224	0.971	19	702	ZINC000003956788
0.1	-8.4	-7.5	0.32	2.945	0.446	5.208	0.869	20	703	ZINC000003918138
0.1	-4.7	-4.1	0.27	4.840	2.236	5.582	2.355	20	704	ZINC000000895099
0.1	-3.2	-2.9	0.16	5.668	2.044	6.318	1.970	20	705	ZINC000000901552
0.1	-3.2	-2.9	0.18	5.417	2.403	6.062	2.284	20	706	ZINC000000901555
0.1	-8.5	-7.3	0.41	3.967	1.540	5.107	1.776	20	707	ZINC000003798064
0.1	-9.6	-9.3	0.17	3.483	1.104	6.074	3.258	20	708	ZINC000006745272
0.1	-9.8	-9.1	0.38	4.477	0.716	6.765	1.131	20	709	ZINC000116473771
0.1	-7.6	-6.9	0.28	2.588	0.441	4.563	1.209	20	710	ZINC000003812867

0.1	-5.2	-4.9	0.20	3.745	1.885	4.675	1.792	20	711	ZINC000001532526
0.1	-3.6	-3.2	0.25	4.385	3.200	5.070	3.162	20	712	ZINC000008214573
0.1	-8.8	-8.1	0.36	3.254	0.879	5.476	0.985	20	713	ZINC000000005560
0.1	-8.7	-8.2	0.23	2.908	0.599	5.257	1.497	20	714	ZINC000030691760
0.1	-5.1	-4.2	0.36	3.115	1.010	4.125	1.005	20	715	ZINC000003589203
0.1	-8.2	-7.4	0.31	2.970	0.878	5.189	1.373	20	716	ZINC000000056646
0.1	-7.2	-6.7	0.24	3.238	0.632	5.526	1.251	20	717	ZINC000003953037
0.1	-7.9	-7.2	0.33	2.867	0.470	4.715	0.860	18	718	ZINC000013585233
0.1	-5.2	-4.6	0.22	3.994	1.397	5.100	1.433	20	719	ZINC000000155905
0.1	-8.7	-8.2	0.30	2.108	0.604	4.286	1.690	20	720	ZINC000001571009
0.1	-7.3	-6.4	0.40	3.526	1.281	4.870	1.851	20	721	ZINC000000057341
0.1	-8.2	-7.5	0.37	3.948	1.316	5.290	1.502	20	722	ZINC000004216238
0.1	-9.2	-8.5	0.33	2.106	0.479	5.002	1.800	20	723	ZINC000003875332
0.1	-7.0	-6.3	0.34	4.714	1.005	6.831	1.732	20	724	ZINC000000113382
0.1	-7.5	-7.0	0.21	1.996	0.326	4.992	1.294	20	725	ZINC000022851765

0.1	-5.8	-5.1	0.29	3.013	1.292	4.890	1.285	20	726	ZINC000000895040
0.1	-4.9	-4.5	0.20	3.931	3.405	5.688	3.074	20	727	ZINC000008015016
0.1	-4.9	-4.2	0.37	5.303	2.310	6.119	2.401	20	728	ZINC000000895360
0.1	-3.4	-2.9	0.22	6.400	4.775	6.735	4.606	19	729	ZINC000008214514

Table S6. AChE inhibitors from Metabolites-Clean-In+Vivo dataset (6 modes).

Ea diff.	Ea 1st	Ea mean	Ea SD	rmsd/l.b. mean	rmsd/l.b. SD	rmsd/u.b. mean	rmsd/u.b. SD	no. modes	no. rank	ZINC ID#
2.7	-12.4	-12.4	0.00	0.000	0.000	0.000	0.000	1	1	ZINC000000899892
2.5	-10.7	-8.8	1.14	1.760	0.177	6.558	0.112	4	2	ZINC000014728221
2.2	-11.1	-9.9	0.68	1.553	0.281	4.417	1.803	6	3	ZINC000014856933
2.1	-10.2	-8.3	0.95	1.937	0.515	6.013	1.280	6	4	ZINC000001721694
2.1	-10.2	-8.2	1.02	1.518	0.162	4.134	0.490	6	5	ZINC000230091164
2.0	-10.3	-9.0	0.85	1.840	0.415	3.731	0.648	6	6	ZINC000257391395
2.0	-10.2	-9.0	0.76	1.808	0.326	4.091	1.855	6	7	ZINC000100825111
2.0	-10.8	-9.1	1.12	3.609	0.226	5.710	0.474	4	8	ZINC000002568171

1.9	-9.8	-9.3	0.23	1.907	0.332	3.759	1.022	6	9	ZINC000100782014
1.9	-7.9	-7.5	0.20	1.885	0.264	3.728	0.335	6	10	ZINC000001641695
1.9	-7.7	-6.8	0.51	2.352	0.895	3.329	1.044	6	11	ZINC000012358780
1.9	-7.9	-7.5	0.21	1.982	0.229	3.577	0.347	6	12	ZINC000001641697
1.9	-11.4	-9.7	1.08	1.832	0.379	3.854	1.644	5	13	ZINC000049073510
1.9	-7.9	-7.5	0.19	1.928	0.315	3.608	0.337	6	14	ZINC000001641694
1.9	-10.7	-9.3	0.78	2.708	0.501	4.454	0.645	6	15	ZINC000002569743
1.9	-10.4	-9.5	0.56	2.099	1.079	3.772	1.505	6	16	ZINC000002570182
1.8	-9.6	-8.2	0.67	1.714	0.336	4.231	0.478	6	17	ZINC000006072099
1.8	-10.3	-8.8	0.92	1.471	0.267	3.825	1.833	5	18	ZINC000257492754
1.8	-9.4	-8.6	0.51	1.692	0.800	4.369	0.917	6	19	ZINC000014636785
1.8	-10.2	-8.9	0.78	1.781	0.263	3.723	0.963	6	20	ZINC000014497825
1.8	-10.1	-8.9	0.74	1.989	0.269	4.899	1.771	6	21	ZINC000000898356
1.8	-10.6	-9.4	0.81	1.923	0.476	3.189	1.216	6	22	ZINC000053199482
1.8	-10.8	-9.3	0.87	1.665	0.151	4.529	0.057	5	23	ZINC000015059550

1.8	-11.6	-11.6	0.00	0.000	0.000	0.000	0.000	1	24	ZINC000254823513
1.8	-10.2	-8.9	0.86	1.130	0.329	5.926	1.849	6	25	ZINC000014436345
1.8	-10.1	-8.9	0.59	1.983	0.292	4.268	0.669	6	26	ZINC000257440791
1.8	-9.9	-8.9	0.82	1.236	0.603	3.788	2.189	6	27	ZINC000085933459
1.7	-8.9	-7.9	0.62	1.580	0.207	3.596	0.533	6	28	ZINC000197875000
1.7	-9.3	-8.3	0.58	1.753	0.078	3.318	0.142	6	29	ZINC000001850972
1.7	-10.2	-8.4	1.02	1.721	0.268	4.042	1.984	6	30	ZINC000101705819
1.7	-8.6	-7.5	0.55	2.126	0.377	3.117	1.294	6	31	ZINC000034125583
1.7	-8.9	-7.9	0.66	1.625	0.128	3.864	0.336	6	32	ZINC000014413209
1.7	-10.2	-9.0	0.68	1.254	0.244	5.850	1.988	6	33	ZINC000014643597
1.7	-8.8	-8.0	0.40	1.379	0.122	3.431	0.347	6	34	ZINC000014973042
1.7	-9.0	-8.6	0.30	1.777	0.132	3.881	1.073	6	35	ZINC000001844836
1.7	-9.7	-8.7	0.69	1.723	0.214	3.947	0.956	6	36	ZINC000257558388
1.7	-8.9	-8.2	0.51	1.328	0.190	3.662	0.198	6	37	ZINC000005762484
1.7	-8.1	-7.6	0.35	1.866	0.822	3.542	0.864	6	38	ZINC000012418177

1.7	-10.7	-9.4	0.75	1.681	0.283	2.783	0.490	4	39	ZINC000013399000
1.7	-9.0	-8.3	0.35	0.946	0.273	3.503	0.891	6	40	ZINC000002384326
1.7	-9.1	-8.7	0.27	1.854	0.201	4.471	0.373	6	41	ZINC000001844839
1.7	-8.4	-8.1	0.18	1.796	0.438	3.813	0.610	6	42	ZINC000000410077
1.7	-10.5	-9.3	0.64	1.937	0.190	3.535	0.639	6	43	ZINC000014636754
1.7	-9.6	-8.0	0.97	1.826	0.225	4.174	0.836	6	44	ZINC000015055179
1.7	-7.9	-7.1	0.46	1.973	0.316	3.517	0.428	6	45	ZINC000014652213
1.7	-9.9	-8.5	0.70	1.764	0.247	4.012	0.269	6	46	ZINC000014807112
1.7	-8.8	-8.0	0.37	1.974	0.278	3.111	0.824	6	47	ZINC000014688572
1.7	-10.2	-9.7	0.27	1.908	0.360	4.743	1.114	6	48	ZINC000100781944
1.7	-9.5	-8.2	0.79	1.389	0.160	3.751	1.201	6	49	ZINC000238777918
1.7	-11.2	-9.1	1.02	1.549	0.504	4.058	1.826	6	50	ZINC000008552351
1.7	-9.2	-8.7	0.48	1.206	0.209	3.448	0.539	6	51	ZINC000100826334
1.7	-9.5	-8.3	0.65	1.689	0.379	3.495	1.620	6	52	ZINC000015214557
1.7	-10.8	-10.1	0.60	1.604	0.612	4.712	1.878	6	53	ZINC000013520048

1.6	-8.2	-7.8	0.20	1.742	0.555	4.497	0.331	6	54	ZINC000000394557
1.6	-8.8	-7.8	0.52	1.792	0.324	3.905	0.349	6	55	ZINC000012153513
1.6	-9.9	-8.3	0.88	1.676	0.216	4.295	0.563	6	56	ZINC000257466071
1.6	-10.8	-9.7	0.82	1.434	0.119	6.023	0.117	3	57	ZINC000003983997
1.6	-10.0	-9.4	0.54	1.117	0.547	5.853	1.965	6	58	ZINC000006092566
1.6	-10.1	-8.5	0.86	2.339	0.237	4.493	0.206	6	59	ZINC000257465010
1.6	-8.9	-8.5	0.34	1.634	0.171	5.107	0.475	6	60	ZINC000013660176
1.6	-10.3	-9.8	0.25	1.790	0.252	4.640	1.147	6	61	ZINC000100781941
1.6	-10.3	-8.8	0.88	1.878	0.276	3.329	0.881	4	62	ZINC000015155032
1.6	-10.1	-8.9	1.06	1.583	0.265	3.862	1.206	6	63	ZINC000100780119
1.6	-11.2	-9.8	0.75	1.465	0.345	5.025	1.673	6	64	ZINC000100775055
1.6	-8.8	-7.8	0.64	2.292	0.419	4.917	1.564	6	65	ZINC000141363803
1.6	-8.9	-8.6	0.30	1.186	0.397	3.823	0.234	6	66	ZINC000085850254
1.6	-8.8	-8.1	0.34	1.400	0.093	2.972	1.090	6	67	ZINC000015214528
1.6	-9.0	-8.4	0.38	1.729	0.509	2.901	0.617	6	68	ZINC000100778584

1.6	-10.2	-9.6	0.57	1.179	0.374	5.803	1.976	6	69	ZINC000004096945
1.6	-7.3	-6.8	0.33	2.357	0.362	3.375	0.584	6	70	ZINC000031484861
1.6	-7.7	-6.9	0.38	1.933	0.437	3.146	0.476	6	71	ZINC000008580496
1.6	-8.2	-7.4	0.41	2.273	0.256	3.668	0.575	6	72	ZINC000014860417
1.6	-9.0	-8.7	0.18	1.755	0.136	5.015	0.885	6	73	ZINC000013660178
1.6	-9.4	-8.5	0.64	1.871	0.170	4.464	0.316	6	74	ZINC000257572694
1.6	-7.7	-6.8	0.48	1.833	0.306	3.213	0.430	6	75	ZINC000014652207
1.6	-10.2	-8.7	0.80	2.022	0.336	4.114	0.934	6	76	ZINC000257450129
1.6	-7.7	-7.1	0.28	2.127	0.522	3.509	0.748	6	77	ZINC000005273747
1.6	-9.3	-8.4	0.43	1.559	0.136	3.238	0.822	6	78	ZINC000257403666
1.6	-10.6	-10.3	0.28	1.138	0.624	4.202	2.479	6	79	ZINC000003874317
1.6	-9.6	-9.0	0.44	2.450	0.185	4.625	1.016	6	80	ZINC000025757012
1.6	-8.0	-7.3	0.36	2.481	0.297	3.843	0.527	6	81	ZINC000006030770
1.6	-9.9	-8.7	0.63	1.985	0.306	3.535	1.150	6	82	ZINC000015054825
1.6	-9.0	-8.3	0.47	1.878	0.271	3.614	1.143	6	83	ZINC000014765294

1.5	-8.8	-8.1	0.41	1.728	0.277	2.898	1.049	6	84	ZINC000014442507
1.5	-10.8	-8.9	1.37	1.664	0.120	2.639	0.721	3	85	ZINC000257375472
1.5	-9.8	-9.6	0.16	0.728	0.349	6.069	2.088	6	86	ZINC000013340101
1.5	-9.6	-8.8	0.50	1.834	0.424	3.513	1.308	6	87	ZINC000256600544
1.5	-9.4	-8.7	0.39	2.136	0.755	3.770	1.547	6	88	ZINC000034031079
1.5	-10.5	-10.2	0.25	1.134	0.000	6.307	0.000	2	89	ZINC000005178763
1.5	-10.5	-9.4	0.68	1.935	0.486	4.510	1.685	6	90	ZINC000014728050
1.5	-9.5	-8.1	1.04	1.796	0.231	4.125	0.811	6	91	ZINC000257561485
1.5	-9.9	-9.0	0.59	2.484	0.676	5.060	1.187	6	92	ZINC000085993650
1.5	-10.9	-9.2	0.82	1.772	0.253	4.917	1.367	6	93	ZINC000013308679
1.5	-11.1	-9.6	0.84	2.267	0.368	2.992	0.497	5	94	ZINC000028822655
1.5	-10.5	-8.9	0.77	2.625	0.372	4.499	0.966	6	95	ZINC000002569742
1.5	-9.7	-8.9	0.54	1.255	0.418	4.072	2.190	6	96	ZINC000013382386
1.5	-10.4	-8.8	1.20	1.857	0.008	5.455	1.413	3	97	ZINC000014414919
1.5	-10.1	-8.7	0.68	1.885	0.276	5.494	1.665	6	98	ZINC000014727928

1.5	-9.9	-8.8	0.59	3.113	0.713	5.884	1.264	6	99	ZINC000004097050
1.5	-8.9	-8.7	0.17	1.914	0.272	3.561	0.277	6	100	ZINC000014884279
1.5	-10.0	-8.8	0.64	1.665	0.162	3.332	1.031	6	101	ZINC000257391394
1.5	-10.0	-9.4	0.36	1.344	0.291	4.375	0.300	6	102	ZINC000100053171
1.5	-8.6	-8.0	0.30	1.504	0.084	3.463	0.688	6	103	ZINC000005431438
1.5	-11.1	-9.8	0.68	1.459	0.333	5.036	1.663	6	104	ZINC000100775059
1.5	-9.4	-8.0	0.72	1.516	0.192	4.224	0.546	6	105	ZINC000100780332
1.5	-9.4	-8.5	0.43	1.541	0.329	3.005	0.770	6	106	ZINC000034294930
1.5	-8.5	-7.7	0.36	2.296	0.553	3.870	0.681	6	107	ZINC000001850427
1.5	-9.8	-9.1	0.39	1.973	0.589	3.695	1.270	6	108	ZINC000013378220
1.5	-8.8	-8.1	0.44	1.945	0.740	4.291	0.658	6	109	ZINC000000020244
1.5	-9.2	-8.7	0.34	2.099	0.382	4.713	1.436	6	110	ZINC000014593989
1.5	-8.7	-8.1	0.33	1.586	0.656	2.766	1.433	6	111	ZINC000005191516
1.5	-9.7	-8.8	0.62	2.005	0.386	2.972	0.867	6	112	ZINC000015056374
1.5	-10.1	-8.4	1.05	1.488	0.141	4.556	1.934	6	113	ZINC000015061065

1.5	-10.1	-9.0	0.80	1.862	0.366	3.314	0.941	6	114	ZINC000006094132
1.5	-8.9	-8.6	0.20	1.305	0.107	3.197	0.434	6	115	ZINC000015115276
1.5	-9.3	-8.7	0.42	1.989	0.989	5.145	0.619	6	116	ZINC000000056434
1.5	-10.7	-9.0	1.02	2.013	0.457	3.615	1.205	6	117	ZINC000002572820
1.5	-9.2	-8.8	0.27	2.375	0.803	4.509	1.458	6	118	ZINC000034084081
1.5	-11.1	-9.8	0.64	1.554	0.285	4.892	1.544	6	119	ZINC000100775056
1.5	-10.2	-9.7	0.47	1.362	0.103	4.429	0.220	6	120	ZINC000100053167
1.5	-8.1	-7.5	0.34	2.188	0.391	4.068	1.015	6	121	ZINC000013460056
1.5	-8.8	-8.5	0.25	2.260	0.688	4.941	1.066	6	122	ZINC000004802475
1.5	-7.4	-6.7	0.42	2.233	0.715	3.459	0.435	6	123	ZINC000014589814
1.5	-10.6	-9.4	1.09	1.164	0.270	3.988	2.351	5	124	ZINC000085614649
1.5	-10.5	-9.7	0.45	1.952	0.667	4.529	2.291	6	125	ZINC000253590073
1.5	-8.4	-7.8	0.37	1.876	0.529	3.347	1.780	6	126	ZINC000085661242
1.5	-9.2	-8.3	0.56	1.774	0.214	3.817	0.744	6	127	ZINC000002169365
1.5	-9.3	-8.5	0.50	1.714	0.361	3.779	0.526	6	128	ZINC000100782013

1.5	-7.8	-7.5	0.20	2.376	0.684	4.013	0.957	6	129	ZINC000001850908
1.5	-10.1	-9.5	0.36	1.279	0.146	4.154	1.087	6	130	ZINC000002504597
1.5	-9.6	-9.4	0.15	1.880	0.764	3.396	0.675	6	131	ZINC000100781933
1.5	-7.8	-7.5	0.26	1.827	0.285	3.412	0.284	6	132	ZINC000001850663
1.5	-7.7	-7.2	0.33	1.700	0.672	3.649	1.344	6	133	ZINC000004582605
1.5	-11.1	-9.8	0.66	1.444	0.326	5.034	1.664	6	134	ZINC000100775058
1.5	-10.6	-9.8	0.55	1.959	0.532	4.476	1.937	6	135	ZINC000018185774
1.5	-9.4	-8.7	0.33	1.987	0.218	4.658	1.512	6	136	ZINC000034031080
1.5	-7.9	-7.2	0.36	1.647	0.419	2.807	0.850	6	137	ZINC000014587710
1.5	-7.9	-7.5	0.18	1.901	0.370	3.419	0.486	6	138	ZINC000001641696
1.4	-10.0	-8.5	0.71	1.965	0.211	3.812	1.039	6	139	ZINC000013486728
1.4	-8.0	-7.5	0.25	1.976	0.439	3.446	1.170	6	140	ZINC000014822344
1.4	-9.3	-8.8	0.29	1.464	0.524	3.692	1.447	6	141	ZINC000013340546
1.4	-8.8	-8.1	0.33	1.641	0.155	4.010	0.618	6	142	ZINC000014442511
1.4	-9.0	-8.6	0.24	1.579	0.651	4.346	0.478	6	143	ZINC000059778328

1.4	-9.5	-9.4	0.11	1.865	0.764	3.063	0.851	6	144	ZINC000100781937
1.4	-10.9	-9.3	0.98	1.469	0.447	4.096	1.682	5	145	ZINC000033587910
1.4	-10.5	-9.9	0.38	1.638	0.658	3.770	2.058	6	146	ZINC000100152142
1.4	-9.7	-8.0	1.08	1.755	0.320	4.195	1.424	6	147	ZINC000085669179
1.4	-8.4	-7.7	0.36	2.403	0.613	4.353	0.899	6	148	ZINC000001685984
1.4	-9.1	-8.7	0.33	1.910	0.653	3.528	1.112	6	149	ZINC000100781957
1.4	-8.0	-7.5	0.28	2.559	0.307	4.222	0.571	6	150	ZINC000001685818
1.4	-9.8	-9.4	0.27	1.819	0.559	5.136	0.407	6	151	ZINC000040897283
1.4	-10.5	-9.8	0.44	1.862	0.743	4.626	2.041	6	152	ZINC000014822283
1.4	-8.6	-8.1	0.40	1.395	0.269	4.556	0.432	6	153	ZINC000100825794
1.4	-8.2	-7.7	0.36	1.246	0.190	3.179	0.698	6	154	ZINC000001850503
1.4	-9.5	-8.8	0.46	2.022	0.195	3.999	1.093	6	155	ZINC000257379323
1.4	-8.7	-8.2	0.24	1.440	0.479	4.204	0.415	6	156	ZINC000059066461
1.4	-9.0	-8.2	0.51	1.567	0.202	3.906	0.606	6	157	ZINC000015160950
1.4	-9.0	-8.2	0.40	2.205	0.770	4.359	0.087	6	158	ZINC000104368037

1.4	-8.9	-8.3	0.35	1.855	0.413	4.353	1.645	6	159	ZINC000013340549
1.4	-8.9	-8.2	0.40	1.991	1.038	3.788	1.895	6	160	ZINC000014858363
1.4	-9.4	-8.9	0.31	2.037	0.632	4.042	1.892	6	161	ZINC000001850101
1.4	-9.9	-8.3	0.81	2.215	1.186	3.699	1.626	6	162	ZINC000004228246
1.4	-8.5	-8.1	0.24	1.623	0.647	3.400	1.380	6	163	ZINC000014764778
1.4	-9.0	-7.9	0.52	1.712	0.196	3.933	0.178	6	164	ZINC000014919115
1.4	-10.1	-8.5	0.87	2.194	0.462	3.841	1.161	6	165	ZINC000002383187
1.4	-10.4	-8.8	1.04	1.498	0.141	3.869	1.822	6	166	ZINC000015061064
1.4	-10.5	-9.6	0.56	2.073	0.541	3.382	1.079	6	167	ZINC000038601228
1.4	-10.3	-9.6	0.42	1.154	0.321	3.001	0.506	6	168	ZINC000104383261
1.4	-10.5	-9.3	1.05	2.187	0.162	4.190	0.686	6	169	ZINC000038476614
1.4	-10.3	-9.5	0.49	1.164	0.315	3.134	0.612	6	170	ZINC000104383257
1.4	-9.0	-8.2	0.37	1.646	0.275	3.453	0.849	6	171	ZINC000006037458
1.4	-8.7	-7.7	0.59	1.157	0.298	2.651	0.879	6	172	ZINC000015115280
1.4	-7.3	-6.8	0.37	1.753	0.890	2.820	0.935	6	173	ZINC000014680002

1.4	-10.5	-9.9	0.47	1.747	0.425	4.379	1.946	6	174	ZINC000000058117
1.4	-10.6	-9.1	0.84	1.768	0.365	4.773	1.216	6	175	ZINC000000402671
1.4	-10.3	-9.5	0.45	1.121	0.298	2.691	0.592	6	176	ZINC000104383255
1.4	-10.5	-9.2	0.72	2.163	0.347	4.590	2.149	6	177	ZINC000100152139
1.4	-8.5	-8.0	0.24	2.022	0.120	4.278	0.515	6	178	ZINC000033950748
1.4	-9.5	-8.7	0.45	1.765	0.759	3.327	1.438	6	179	ZINC000085994612
1.4	-8.3	-7.7	0.31	1.942	0.473	3.569	0.511	6	180	ZINC000100779593
1.4	-9.5	-9.0	0.43	1.769	0.381	3.823	1.442	6	181	ZINC000005762114
1.4	-8.7	-7.5	0.63	2.465	0.946	4.767	2.133	6	182	ZINC000014651781
1.4	-10.0	-8.4	0.87	2.608	0.951	4.322	2.193	6	183	ZINC000100363890
1.4	-11.2	-10.0	0.78	1.685	0.479	3.830	1.405	6	184	ZINC000100784075
1.4	-10.3	-9.5	0.46	1.109	0.291	2.700	0.571	6	185	ZINC000104383251
1.4	-10.5	-9.7	0.61	1.625	0.673	2.892	1.854	6	186	ZINC000253590071
1.4	-8.3	-7.8	0.29	1.309	0.157	3.441	0.590	6	187	ZINC000038634844
1.4	-7.6	-7.1	0.34	1.801	0.348	3.595	0.495	6	188	ZINC000014652210

1.4	-8.7	-8.4	0.16	1.650	0.475	3.416	0.857	6	189	ZINC000053599708
1.4	-8.6	-8.3	0.18	2.123	0.486	4.504	0.992	6	190	ZINC000012417829
1.4	-9.5	-8.9	0.52	2.030	0.335	3.721	1.255	6	191	ZINC000013486729
1.4	-8.3	-7.6	0.77	2.106	0.495	3.747	0.870	6	192	ZINC000014713576
1.4	-8.7	-8.0	0.32	1.376	0.168	3.902	0.451	6	193	ZINC000100780396
1.4	-8.5	-7.8	0.51	1.405	0.091	3.624	0.311	6	194	ZINC000005431440
1.4	-7.9	-7.0	0.47	2.411	0.676	3.489	1.048	6	195	ZINC000003999316
1.4	-9.2	-8.2	0.48	2.039	0.799	4.089	1.926	6	196	ZINC000005925407
1.4	-9.5	-9.2	0.19	2.852	0.973	3.958	1.551	6	197	ZINC000095620791
1.4	-7.3	-7.0	0.11	1.652	0.472	4.128	1.333	6	198	ZINC000062233804
1.4	-9.9	-8.5	0.71	1.904	0.156	4.719	1.009	6	199	ZINC000015214561
1.4	-10.5	-9.5	0.78	1.604	0.571	2.983	1.728	6	200	ZINC000205122910
1.4	-8.9	-7.6	0.78	1.932	0.340	4.492	1.227	6	201	ZINC000085897787
1.4	-8.8	-8.2	0.31	1.715	0.294	3.324	0.769	6	202	ZINC000005103848
1.4	-8.6	-8.0	0.33	1.792	0.397	4.074	0.334	6	203	ZINC000100780402

1.4	-8.7	-8.3	0.33	1.610	0.265	3.903	0.413	6	204	ZINC000005132067
1.4	-9.6	-9.2	0.33	2.050	0.388	3.825	0.796	6	205	ZINC000067665433
1.4	-7.6	-7.4	0.11	2.527	0.564	5.047	0.642	6	206	ZINC000001532860
1.4	-10.9	-10.9	0.00	0.000	0.000	0.000	0.000	1	207	ZINC000005955119
1.4	-10.4	-8.9	1.00	1.710	0.253	4.961	0.066	4	208	ZINC000013486155
1.4	-9.3	-7.8	0.97	2.213	0.528	5.090	1.871	6	209	ZINC000014642915
1.4	-10.5	-9.9	0.42	1.876	0.769	4.560	2.221	6	210	ZINC000014822285
1.4	-11.2	-10.0	0.73	1.746	0.508	3.721	1.327	6	211	ZINC000257657037
1.4	-9.1	-8.4	0.38	1.834	0.243	3.332	0.425	6	212	ZINC000100824246
1.4	-9.3	-8.2	0.71	1.661	0.177	4.142	0.508	6	213	ZINC000015060167
1.4	-8.9	-8.1	0.42	1.669	0.417	3.329	1.082	6	214	ZINC000033953590
1.4	-10.2	-9.8	0.28	1.049	0.543	4.296	2.182	6	215	ZINC000014436799
1.4	-8.1	-7.5	0.49	1.561	0.793	3.620	0.468	6	216	ZINC000014818208
1.4	-7.8	-7.2	0.30	2.316	0.459	3.480	0.405	6	217	ZINC000006030769
1.4	-7.6	-7.3	0.20	2.473	0.364	3.303	0.536	6	218	ZINC000006030766

1.4	-9.7	-8.9	0.43	1.964	0.347	4.223	0.339	6	219	ZINC000015160852
1.4	-9.9	-8.7	0.76	1.550	0.243	5.501	1.328	6	220	ZINC000257474048
1.4	-7.2	-6.9	0.18	1.850	0.883	3.075	0.971	6	221	ZINC000014657479
1.4	-8.7	-8.3	0.26	1.910	0.448	4.185	0.391	6	222	ZINC000053599699
1.3	-9.6	-8.6	0.52	2.180	0.203	3.744	0.871	6	223	ZINC000252426445
1.3	-9.8	-9.0	0.41	1.950	0.302	4.568	2.013	6	224	ZINC000001003581
1.3	-9.7	-8.2	0.76	2.140	0.834	4.071	0.390	6	225	ZINC000006070386
1.3	-7.2	-6.6	0.32	2.324	0.457	3.483	0.550	6	226	ZINC000100079245
1.3	-8.6	-8.2	0.23	1.196	0.104	3.500	0.329	6	227	ZINC000086035230
1.3	-7.0	-6.3	0.42	2.105	0.558	3.745	0.387	6	228	ZINC000100778218
1.3	-8.0	-7.5	0.27	1.711	0.525	3.568	0.824	6	229	ZINC000038219001
1.3	-8.7	-7.9	0.43	1.625	0.183	3.229	0.766	6	230	ZINC000257398324
1.3	-8.8	-8.1	0.43	1.585	0.270	4.286	1.108	6	231	ZINC000100825791
1.3	-8.6	-8.0	0.33	1.633	0.124	3.970	0.704	6	232	ZINC000005731856
1.3	-7.2	-6.6	0.34	2.256	0.398	3.540	0.714	6	233	ZINC000100582767

1.3	-9.0	-8.2	0.45	1.761	0.286	3.838	0.704	6	234	ZINC000002037433
1.3	-11.1	-10.1	0.86	1.725	0.986	4.093	0.973	6	235	ZINC000257507658
1.3	-7.8	-7.5	0.15	1.735	0.357	3.347	0.486	6	236	ZINC000014860420
1.3	-9.5	-8.7	0.51	2.182	0.997	4.620	1.869	6	237	ZINC000090698218
1.3	-9.7	-8.8	0.56	2.049	0.172	4.256	0.704	6	238	ZINC000100780338
1.3	-11.2	-10.0	0.73	1.748	0.505	3.726	1.325	6	239	ZINC000014693987
1.3	-8.4	-8.2	0.21	1.416	0.282	3.708	0.250	6	240	ZINC000014918738
1.3	-10.1	-8.6	0.92	1.860	0.155	4.249	0.443	6	241	ZINC000100780114
1.3	-11.1	-10.1	0.74	1.710	1.019	4.142	1.004	6	242	ZINC000100779693
1.3	-8.8	-8.4	0.38	1.721	0.531	4.329	1.902	6	243	ZINC000014413387
1.3	-7.2	-6.6	0.32	2.168	0.482	3.471	0.427	6	244	ZINC000004098408
1.3	-7.2	-6.9	0.16	1.942	0.900	3.342	1.148	6	245	ZINC000001532633
1.3	-8.9	-8.7	0.37	1.126	0.617	4.396	2.443	6	246	ZINC000000006787
1.3	-8.5	-7.9	0.41	1.960	0.231	4.435	0.315	6	247	ZINC000149473035
1.3	-7.2	-6.8	0.22	1.635	0.355	2.763	0.389	6	248	ZINC000001850007

1.3	-8.2	-7.5	0.44	1.673	0.233	3.761	0.344	6	249	ZINC000006030959
1.3	-11.5	-10.8	0.41	1.113	0.294	4.606	2.037	6	250	ZINC000257375604
1.3	-8.9	-8.6	0.23	1.220	0.611	4.302	1.665	6	251	ZINC000002381590
1.3	-7.2	-6.6	0.30	2.183	0.468	3.181	0.598	6	252	ZINC000101168100
1.3	-11.5	-10.6	0.48	1.174	0.234	4.600	1.771	5	253	ZINC000257375603
1.3	-8.1	-7.7	0.22	2.389	0.618	3.664	1.645	6	254	ZINC000004096399
1.3	-9.4	-8.6	0.50	1.857	0.355	3.440	0.956	6	255	ZINC000015219998
1.3	-9.3	-8.7	0.38	1.461	0.057	3.704	1.438	6	256	ZINC000005352276
1.3	-8.5	-7.7	0.59	2.825	0.285	5.784	0.955	6	257	ZINC000014825018
1.3	-8.5	-7.6	0.53	1.605	0.328	2.994	0.655	6	258	ZINC000002020094
1.3	-8.1	-7.6	0.28	2.653	0.504	4.511	1.179	6	259	ZINC000005752020
1.3	-9.4	-8.5	0.57	1.822	0.348	4.708	1.527	6	260	ZINC000014504553
1.3	-10.8	-9.2	1.02	1.548	0.159	4.521	1.193	6	261	ZINC000002029390
1.3	-7.8	-7.0	0.41	2.016	0.333	3.754	0.714	6	262	ZINC000001850336
1.3	-10.3	-9.0	0.84	1.931	0.363	3.279	0.953	6	263	ZINC000015203705

1.3	-11.2	-10.0	0.77	1.683	0.478	3.827	1.404	6	264	ZINC000257657039
1.3	-9.4	-8.5	0.43	2.377	0.326	3.859	0.996	6	265	ZINC000025757016
1.3	-10.8	-9.3	0.72	1.487	0.160	4.790	1.145	6	266	ZINC000100784262
1.3	-11.5	-10.5	0.70	1.245	0.509	4.323	1.553	6	267	ZINC000257375605
1.3	-9.2	-8.7	0.46	1.981	0.325	3.169	0.733	6	268	ZINC000001319187
1.3	-9.9	-9.1	0.45	2.204	0.479	3.756	1.678	6	269	ZINC000001569924
1.3	-8.6	-8.0	0.39	1.308	0.160	4.145	0.474	6	270	ZINC000100825795
1.3	-8.3	-7.9	0.39	1.939	0.683	4.147	2.056	6	271	ZINC000104373884
1.3	-10.4	-10.0	0.24	2.816	0.586	4.520	0.803	6	272	ZINC000014616909
1.3	-7.5	-6.9	0.29	2.217	0.458	4.050	1.964	6	273	ZINC000005380682
1.3	-11.5	-10.7	0.50	1.053	0.283	4.775	1.603	6	274	ZINC000100780890
1.3	-7.6	-7.3	0.18	1.438	0.178	3.099	0.460	6	275	ZINC000015253562
1.3	-10.1	-8.5	0.75	1.955	0.336	4.688	1.892	6	276	ZINC000085933453
1.3	-9.3	-8.5	0.57	1.959	0.086	3.772	0.825	6	277	ZINC000257482482
1.3	-7.9	-7.7	0.14	1.989	0.340	4.621	1.061	6	278	ZINC000004629861

1.3	-9.2	-8.6	0.29	2.388	0.751	4.275	1.780	6	279	ZINC000064560765
1.3	-8.8	-8.1	0.44	1.788	0.197	3.690	0.535	6	280	ZINC000015209390
1.3	-8.0	-7.7	0.22	2.231	0.188	4.446	0.272	6	281	ZINC000095620528
1.3	-9.2	-8.6	0.33	1.637	0.294	3.652	0.718	6	282	ZINC000002572845
1.3	-8.3	-7.0	0.81	2.107	0.415	3.492	1.312	6	283	ZINC000032840981
1.3	-8.4	-8.1	0.21	1.440	0.197	3.499	0.573	6	284	ZINC000085880361
1.3	-9.1	-8.3	0.50	2.147	0.382	3.888	1.099	6	285	ZINC000005438241
1.3	-9.2	-8.4	0.54	1.790	1.058	3.450	2.188	6	286	ZINC000014858358
1.3	-10.8	-9.2	0.83	1.501	0.171	5.043	0.975	6	287	ZINC000257517028
1.3	-7.2	-6.6	0.32	2.104	0.337	3.208	0.308	6	288	ZINC000000967596
1.3	-7.5	-7.3	0.11	1.711	0.139	4.438	1.205	6	289	ZINC000085599280
1.3	-9.0	-8.3	0.33	1.803	0.214	4.653	0.441	6	290	ZINC000230085901
1.3	-8.9	-7.7	0.58	1.732	0.213	3.623	0.876	6	291	ZINC000257476496
1.3	-8.3	-7.8	0.27	1.420	0.200	3.017	0.693	6	292	ZINC000100779591
1.3	-11.2	-9.9	0.85	1.608	0.424	3.614	1.292	6	293	ZINC000257657038

1.3	-7.2	-6.6	0.30	2.017	0.364	3.389	0.447	6	294	ZINC000001081121
1.3	-7.6	-7.2	0.17	2.065	0.446	3.411	0.990	6	295	ZINC000002010910
1.3	-9.2	-8.1	0.56	2.638	0.825	5.431	1.712	6	296	ZINC000014858355
1.3	-9.7	-8.8	0.58	2.070	0.245	3.768	0.970	6	297	ZINC000015056382
1.3	-8.3	-7.3	0.65	1.706	0.228	3.960	0.754	6	298	ZINC000014635817
1.3	-8.9	-8.3	0.39	1.413	0.337	3.660	0.553	6	299	ZINC000030731547
1.3	-11.1	-10.1	0.78	1.692	1.016	4.292	1.064	6	300	ZINC000100779689
1.3	-8.7	-8.5	0.18	1.586	0.384	3.854	1.797	6	301	ZINC000013109226
1.3	-8.5	-7.8	0.40	1.869	0.774	3.990	0.363	6	302	ZINC000002040950
1.3	-10.0	-8.8	0.97	1.811	0.515	5.651	1.788	6	303	ZINC000000119983
1.3	-8.1	-7.7	0.23	1.704	0.419	3.814	1.438	6	304	ZINC000034961802
1.3	-9.1	-8.6	0.32	1.660	0.304	3.870	0.996	6	305	ZINC000257391554
1.3	-8.7	-8.3	0.28	1.467	0.188	4.002	0.118	6	306	ZINC000100781835
1.3	-9.5	-8.5	0.67	1.697	0.422	4.764	2.193	6	307	ZINC000000899787
1.3	-9.3	-8.0	0.62	2.252	0.148	5.297	0.743	6	308	ZINC000005999120

1.3	-8.4	-7.8	0.39	1.621	0.126	3.153	0.500	6	309	ZINC000015255941
1.3	-7.6	-7.3	0.21	1.832	0.266	3.187	0.705	6	310	ZINC000014860418
1.3	-9.6	-8.7	0.50	1.964	0.258	4.295	0.941	6	311	ZINC000014927465
1.3	-9.8	-8.2	0.94	1.798	0.512	5.288	1.606	6	312	ZINC000022061252
1.3	-10.0	-9.5	0.51	2.019	0.105	6.288	0.046	3	313	ZINC000015058647
1.3	-7.9	-7.3	0.32	2.457	0.583	3.662	0.679	6	314	ZINC000006037451
1.3	-9.7	-8.8	0.69	2.253	0.390	5.498	1.143	6	315	ZINC000003833872
1.3	-8.3	-7.8	0.27	1.737	0.239	3.452	0.791	6	316	ZINC000014616542
1.3	-11.5	-10.3	0.89	1.627	0.325	2.987	0.184	4	317	ZINC000100780892
1.3	-10.8	-9.3	0.72	1.441	0.157	4.453	1.609	6	318	ZINC000086010678
1.3	-10.2	-9.4	0.75	1.612	0.000	6.665	0.000	2	319	ZINC000032022340
1.3	-9.7	-9.0	0.54	1.962	0.247	3.869	0.591	6	320	ZINC000257440792
1.3	-11.2	-9.9	0.81	1.667	0.466	3.741	1.348	6	321	ZINC000100784074
1.3	-8.2	-7.5	0.32	1.577	0.333	3.533	0.574	6	322	ZINC000012153514
1.3	-8.6	-8.1	0.24	2.869	0.622	4.946	1.992	6	323	ZINC000017175239

1.3	-10.7	-10.6	0.10	1.700	0.000	2.462	0.000	2	324	ZINC000013539005
1.3	-7.0	-6.6	0.26	2.555	0.141	4.180	0.544	6	325	ZINC000002384605
1.3	-7.2	-6.6	0.29	2.277	0.478	3.451	0.558	6	326	ZINC000001694437
1.3	-9.6	-8.7	0.55	1.919	0.389	3.707	0.997	6	327	ZINC000252426443
1.3	-9.2	-8.1	0.57	1.904	0.129	4.119	0.318	6	328	ZINC000005438164
1.3	-8.6	-7.9	0.39	2.016	0.226	4.096	0.392	6	329	ZINC000005731858
1.3	-7.2	-6.6	0.32	2.260	0.359	3.478	0.663	6	330	ZINC000100079248
1.3	-9.5	-8.4	0.71	1.978	0.439	4.571	2.188	6	331	ZINC000000968078
1.3	-8.0	-7.2	0.47	2.154	0.557	4.024	0.873	6	332	ZINC000015119192
1.3	-8.6	-8.1	0.26	2.858	0.677	4.863	1.790	6	333	ZINC000001303441
1.3	-9.2	-8.8	0.46	2.320	0.449	3.648	0.608	6	334	ZINC000257471010
1.3	-7.7	-7.0	0.37	2.036	0.411	3.186	0.397	6	335	ZINC000006037450
1.3	-10.5	-9.0	0.76	1.990	0.161	4.239	2.014	6	336	ZINC000015262111
1.3	-7.1	-6.9	0.19	2.102	0.211	3.123	1.083	6	337	ZINC000095619235
1.3	-8.3	-7.8	0.35	1.483	0.265	3.157	1.095	6	338	ZINC000059560109

1.3	-9.5	-9.1	0.37	1.904	0.414	3.879	0.898	6	339	ZINC000043732283
1.3	-8.6	-7.8	0.47	2.141	0.394	3.954	0.907	6	340	ZINC000014593788
1.3	-8.5	-8.1	0.27	3.050	0.076	5.118	0.892	6	341	ZINC000012428336
1.3	-10.8	-8.5	1.03	2.423	0.376	4.402	1.307	6	342	ZINC000014612199
1.3	-7.2	-6.8	0.28	2.160	0.310	4.117	0.915	6	343	ZINC000002077816
1.3	-8.6	-8.3	0.28	1.758	0.450	4.840	1.931	6	344	ZINC000100825941
1.3	-7.9	-7.3	0.33	1.895	0.293	3.242	0.804	6	345	ZINC000002565574
1.3	-8.3	-8.0	0.30	2.011	0.185	5.365	1.930	6	346	ZINC000014720653
1.3	-9.5	-8.9	0.41	2.036	0.159	4.050	0.705	6	347	ZINC000014453721
1.3	-9.7	-8.5	0.78	1.886	0.260	6.493	0.209	6	348	ZINC000014806338
1.3	-10.6	-8.8	1.09	1.750	0.162	4.230	0.376	4	349	ZINC000013340818
1.3	-7.7	-7.1	0.43	2.286	0.818	3.589	1.041	6	350	ZINC000014589768
1.3	-8.1	-7.5	0.39	2.280	0.236	3.667	0.810	6	351	ZINC000013462238
1.3	-8.5	-8.0	0.34	1.758	0.334	3.805	0.474	6	352	ZINC000012153511
1.3	-8.1	-7.9	0.14	2.035	0.574	4.812	1.448	6	353	ZINC000033820503

1.3	-9.2	-8.5	0.50	1.351	0.890	2.644	1.690	6	354	ZINC000257532154
1.3	-7.9	-7.5	0.19	2.275	0.192	3.941	0.599	6	355	ZINC000014860465
1.3	-10.8	-9.3	0.72	1.446	0.155	4.458	1.601	6	356	ZINC000100784255
1.3	-10.8	-9.3	0.72	1.443	0.157	4.454	1.607	6	357	ZINC000100784257
1.3	-11.5	-10.7	0.47	1.144	0.294	4.880	1.642	6	358	ZINC000257375606
1.3	-7.3	-6.8	0.34	1.549	0.774	3.537	0.763	6	359	ZINC000001747260
1.3	-8.2	-7.5	0.32	2.365	0.291	4.361	0.565	6	360	ZINC000100827549
1.3	-10.0	-8.5	0.88	1.597	0.199	4.379	1.526	6	361	ZINC000004026295
1.3	-7.8	-7.4	0.20	1.891	0.785	4.833	1.628	6	362	ZINC000003197533
1.3	-10.8	-9.3	0.72	1.442	0.155	4.454	1.608	6	363	ZINC000257517027
1.3	-9.8	-9.0	0.52	2.267	0.659	3.923	1.509	6	364	ZINC000050027749
1.3	-8.9	-7.8	0.66	1.686	0.564	3.865	1.669	6	365	ZINC000013361115
1.3	-7.8	-7.1	0.36	2.242	0.690	3.412	0.893	6	366	ZINC000014680083
1.3	-8.0	-7.4	0.46	1.324	0.349	3.494	1.589	6	367	ZINC000100776285
1.2	-10.3	-9.0	0.74	2.275	0.349	4.184	0.908	6	368	ZINC000018210005

1.2	-8.8	-7.8	0.49	1.928	0.179	3.271	0.644	6	369	ZINC000014823314
1.2	-9.2	-8.2	0.62	2.012	0.951	4.480	1.778	6	370	ZINC000257532153
1.2	-6.5	-5.9	0.34	2.236	0.215	3.236	0.312	6	371	ZINC000005752223
1.2	-8.5	-8.0	0.33	1.811	0.216	4.024	0.280	6	372	ZINC000003978566
1.2	-8.3	-7.4	0.45	3.065	1.131	3.489	1.382	6	373	ZINC000002566542
1.2	-9.2	-8.5	0.55	1.936	0.317	4.977	1.762	6	374	ZINC000000968074
1.2	-7.7	-7.5	0.17	1.986	0.538	3.316	1.067	6	375	ZINC000001608615
1.2	-7.9	-7.4	0.28	3.074	0.767	5.009	0.615	6	376	ZINC000013377432
1.2	-8.3	-7.6	0.31	2.078	0.786	3.054	1.494	6	377	ZINC000002575489
1.2	-9.4	-8.1	0.75	1.870	0.275	3.189	0.814	6	378	ZINC000033771409
1.2	-11.2	-11.2	0.00	0.000	0.000	0.000	0.000	1	379	ZINC000004097376
1.2	-9.7	-8.7	0.59	1.797	0.234	4.068	1.067	6	380	ZINC000038318756
1.2	-9.9	-8.5	0.98	1.963	0.373	3.830	1.209	6	381	ZINC000100775771
1.2	-8.4	-8.0	0.25	1.353	0.193	3.872	0.296	6	382	ZINC000085600726
1.2	-10.8	-10.0	0.62	1.677	0.548	4.246	0.821	6	383	ZINC000100779702

1.2	-8.2	-7.5	0.31	2.761	0.396	3.902	0.859	6	384	ZINC000006117321
1.2	-9.5	-8.9	0.27	2.426	0.181	6.656	0.177	6	385	ZINC000015249534
1.2	-10.1	-9.3	0.60	1.565	0.467	3.718	1.293	6	386	ZINC000013340033
1.2	-7.0	-6.7	0.22	1.616	0.776	3.917	0.515	6	387	ZINC000013376124
1.2	-7.2	-7.0	0.19	2.499	1.021	4.373	0.510	6	388	ZINC000001570892
1.2	-8.3	-8.1	0.12	2.479	0.649	4.209	1.171	6	389	ZINC000040165549
1.2	-8.9	-8.3	0.33	2.345	1.002	4.353	1.560	6	390	ZINC000034084084
1.2	-6.8	-6.4	0.22	2.673	0.514	4.554	0.290	6	391	ZINC000002040973
1.2	-10.8	-10.0	0.60	1.659	0.560	4.229	0.854	6	392	ZINC000100779703
1.2	-7.7	-7.4	0.21	1.427	0.247	3.267	0.379	6	393	ZINC000002585474
1.2	-9.7	-8.5	1.03	1.838	0.436	4.431	1.706	3	394	ZINC000014410367
1.2	-7.4	-7.1	0.22	1.560	0.218	3.517	0.490	6	395	ZINC000000410088
1.2	-9.6	-9.0	0.47	2.222	0.460	4.960	2.118	6	396	ZINC000002585769
1.2	-10.8	-10.2	0.53	1.487	0.464	4.396	0.794	6	397	ZINC000100779697
1.2	-8.4	-7.8	0.33	1.755	0.208	3.499	0.692	6	398	ZINC000015169238

1.2	-8.0	-7.4	0.31	2.003	0.345	4.913	1.032	6	399	ZINC000001557676
1.2	-10.2	-8.9	0.93	1.796	0.379	3.970	1.716	6	400	ZINC000014887294
1.2	-6.9	-6.6	0.14	1.663	0.200	2.567	0.482	6	401	ZINC000002037836
1.2	-10.7	-9.7	0.51	2.398	0.797	5.558	1.432	6	402	ZINC000004352598
1.2	-8.8	-8.2	0.41	1.635	0.411	3.960	0.742	6	403	ZINC000064634151
1.2	-9.1	-8.2	0.48	2.024	0.231	3.556	0.679	6	404	ZINC000100824682
1.2	-8.7	-8.2	0.39	1.903	0.393	3.713	0.529	6	405	ZINC000085880358
1.2	-10.8	-10.0	0.61	1.657	0.560	4.227	0.846	6	406	ZINC000100779706
1.2	-7.7	-7.2	0.33	1.451	0.607	3.960	1.107	6	407	ZINC000001532071
1.2	-10.0	-9.0	0.72	1.987	0.307	4.202	1.883	6	408	ZINC000014806341
1.2	-8.3	-7.8	0.36	1.385	0.238	3.448	0.391	6	409	ZINC000038634836
1.2	-6.5	-6.0	0.28	1.562	0.299	2.921	0.441	6	410	ZINC000032167028
1.2	-10.0	-8.6	0.79	2.316	0.479	4.504	0.905	6	411	ZINC000059206758
1.2	-9.9	-8.6	0.67	1.927	0.132	3.683	1.251	6	412	ZINC000014497817
1.2	-7.2	-7.0	0.15	2.288	0.655	3.496	0.896	6	413	ZINC000014657476

1.2	-9.2	-8.2	0.59	2.230	1.020	4.488	2.116	6	414	ZINC000014858357
1.2	-8.7	-7.6	0.60	1.743	0.134	3.686	0.148	6	415	ZINC000014441668
1.2	-8.6	-8.1	0.38	1.481	0.262	4.011	0.262	6	416	ZINC000008220462
1.2	-8.8	-7.9	0.68	1.966	0.387	4.317	0.965	6	417	ZINC000257476497
1.2	-8.5	-7.9	0.30	1.759	0.190	3.995	0.307	6	418	ZINC000005431441
1.2	-9.5	-8.4	0.54	1.794	0.380	3.849	0.376	6	419	ZINC000001850335
1.2	-8.0	-7.5	0.26	2.418	0.209	4.313	0.764	6	420	ZINC000031706641
1.2	-8.1	-7.6	0.26	1.668	0.635	2.847	0.754	6	421	ZINC000001583510
1.2	-8.7	-8.3	0.31	1.206	0.127	3.582	0.536	6	422	ZINC000059778565
1.2	-7.9	-7.3	0.35	2.135	0.665	4.002	0.994	6	423	ZINC000245246074
1.2	-8.1	-7.9	0.16	1.319	0.162	3.766	0.726	6	424	ZINC000230090968
1.2	-7.7	-7.5	0.17	1.687	0.110	4.037	0.968	6	425	ZINC000027644505
1.2	-8.3	-7.4	0.39	2.671	0.319	4.931	0.574	6	426	ZINC000100823536
1.2	-8.8	-8.0	0.46	1.807	0.300	3.770	0.698	6	427	ZINC000015209388
1.2	-8.5	-8.1	0.21	2.419	1.053	4.550	1.871	6	428	ZINC000013376485

1.2	-9.2	-8.2	0.66	2.197	0.962	4.911	1.829	6	429	ZINC000257532155
1.2	-10.2	-9.0	1.08	1.873	0.041	4.298	1.357	3	430	ZINC000005967137
1.2	-6.8	-6.6	0.15	2.096	0.437	4.572	1.114	6	431	ZINC000012954494
1.2	-8.9	-8.3	0.30	1.741	0.234	4.053	0.506	6	432	ZINC000038588222
1.2	-6.6	-6.3	0.16	1.866	0.178	3.262	0.233	6	433	ZINC000001626973
1.2	-9.5	-8.5	0.54	2.496	0.488	4.723	0.702	6	434	ZINC000014486964
1.2	-8.6	-8.0	0.38	2.566	0.499	5.364	1.766	6	435	ZINC000013549756
1.2	-8.2	-7.5	0.35	1.940	0.535	3.331	1.292	6	436	ZINC000006038030
1.2	-8.7	-8.0	0.49	2.413	0.390	3.959	0.940	6	437	ZINC000002528942
1.2	-9.9	-8.6	0.74	2.152	0.437	3.847	1.143	6	438	ZINC000015054830
1.2	-8.4	-8.0	0.30	1.768	0.171	3.686	0.596	6	439	ZINC000014957313
1.2	-9.0	-8.2	0.45	1.906	0.412	3.386	0.498	6	440	ZINC000257378315
1.2	-10.2	-9.4	0.63	1.851	0.381	6.275	1.810	6	441	ZINC000006092198
1.2	-7.4	-6.7	0.37	2.098	0.556	3.622	0.995	6	442	ZINC000003978811
1.2	-8.6	-8.1	0.25	2.601	0.384	3.754	1.490	6	443	ZINC000000002159

1.2	-9.7	-9.1	0.54	2.100	0.421	5.097	1.581	6	444	ZINC000014820460
1.2	-7.0	-6.5	0.33	1.728	0.925	2.621	0.801	6	445	ZINC000012358735
1.2	-7.2	-6.8	0.24	1.724	0.356	2.732	0.748	6	446	ZINC000002037835
1.2	-9.0	-8.7	0.30	1.225	0.290	2.644	0.503	6	447	ZINC000005183072
1.2	-8.3	-7.8	0.32	1.814	0.200	3.678	0.872	6	448	ZINC000013307544
1.2	-8.1	-7.2	0.58	1.977	0.423	3.490	0.773	6	449	ZINC000000121456
1.2	-7.1	-6.6	0.23	1.670	0.123	2.965	0.531	6	450	ZINC000004284443
1.2	-9.0	-8.0	0.57	1.785	0.067	4.279	0.863	6	451	ZINC000257590926
1.2	-7.4	-7.1	0.19	2.072	0.186	4.192	1.066	6	452	ZINC000004726636
1.2	-11.2	-10.0	0.77	1.682	0.480	3.824	1.407	6	453	ZINC000014693989
1.2	-7.9	-7.5	0.23	1.449	0.420	3.921	1.102	6	454	ZINC000085644695
1.2	-9.7	-8.8	0.42	2.414	0.472	3.877	0.566	6	455	ZINC000015220001
1.2	-9.8	-8.7	0.66	2.128	0.358	4.961	2.010	6	456	ZINC000008681597
1.2	-9.3	-8.7	0.47	2.176	0.285	4.086	0.261	6	457	ZINC000013381474
1.2	-7.9	-7.5	0.44	1.766	1.279	2.761	1.498	6	458	ZINC000030731184

1.2	-7.8	-7.2	0.31	2.530	0.337	3.794	0.388	6	459	ZINC000006030767
1.2	-8.4	-8.0	0.35	1.154	0.198	3.170	0.657	6	460	ZINC000100780746
1.2	-10.8	-9.2	1.16	1.522	0.315	3.567	2.035	3	461	ZINC000014887305
1.2	-8.4	-7.7	0.39	1.903	0.273	4.244	0.534	6	462	ZINC000014018383
1.2	-8.2	-7.7	0.32	2.146	0.284	3.135	0.839	6	463	ZINC000013538213
1.2	-7.6	-7.0	0.30	1.814	0.398	2.857	0.785	6	464	ZINC000001850044
1.2	-8.1	-7.9	0.13	2.071	0.615	5.435	0.696	6	465	ZINC000004655407
1.2	-8.5	-7.5	0.51	1.912	0.066	3.399	0.340	6	466	ZINC000090697510
1.2	-9.4	-9.2	0.15	1.563	0.555	5.767	2.148	6	467	ZINC000006092209
1.2	-8.5	-7.9	0.41	1.541	0.140	3.207	0.869	6	468	ZINC000015169305
1.2	-10.3	-9.6	0.60	1.962	0.388	3.557	1.506	6	469	ZINC000014756811
1.2	-9.6	-8.7	0.50	1.787	0.285	4.142	1.301	6	470	ZINC000014497820
1.2	-7.2	-6.7	0.33	1.478	0.151	3.073	0.510	6	471	ZINC000000080850
1.2	-9.8	-8.6	0.64	1.688	0.852	4.547	2.103	6	472	ZINC000238740518
1.2	-8.7	-8.0	0.37	1.733	0.083	3.638	0.638	6	473	ZINC000095627992

1.2	-7.8	-7.3	0.41	1.737	0.840	6.121	1.603	6	474	ZINC000003870733
1.2	-10.6	-8.7	1.32	1.811	0.000	3.976	1.071	3	475	ZINC000001653612
1.2	-6.9	-6.3	0.30	1.746	0.512	2.768	0.583	6	476	ZINC000004623761
1.2	-8.5	-7.8	0.56	1.622	0.231	4.006	0.850	6	477	ZINC000014635813
1.2	-10.1	-8.8	0.72	1.799	0.301	3.277	0.778	6	478	ZINC000015216796
1.2	-9.4	-8.6	0.56	1.808	0.205	3.406	0.793	6	479	ZINC000014449460
1.2	-8.5	-8.0	0.28	2.162	0.359	4.658	0.682	6	480	ZINC000005820845
1.2	-7.3	-7.1	0.21	1.984	0.540	3.141	0.394	6	481	ZINC000013508926
1.2	-8.1	-7.5	0.31	2.182	0.486	4.402	1.437	6	482	ZINC000014679998
1.2	-10.8	-10.2	0.51	1.459	0.470	4.362	0.848	6	483	ZINC000257558734
1.2	-8.4	-7.7	0.45	1.674	0.314	3.772	0.631	6	484	ZINC000100776133
1.2	-7.7	-7.3	0.30	1.732	0.182	2.891	0.240	6	485	ZINC000000388663
1.2	-7.0	-6.4	0.26	2.173	0.119	3.173	0.499	6	486	ZINC000002379161
1.2	-8.5	-8.1	0.27	3.122	0.181	5.129	0.927	6	487	ZINC000004096578
1.2	-9.5	-8.8	0.43	1.717	0.399	4.049	2.195	6	488	ZINC000006093399

1.2	-9.1	-8.6	0.32	2.246	0.701	5.044	1.632	6	489	ZINC000002561222
1.2	-8.2	-7.8	0.24	2.171	0.220	3.935	1.108	6	490	ZINC000013460054
1.2	-9.3	-8.7	0.31	2.472	0.366	4.168	0.830	6	491	ZINC000095620792
1.2	-8.8	-8.2	0.40	1.753	0.204	3.600	0.795	6	492	ZINC000005751122
1.2	-8.2	-7.7	0.34	1.754	0.133	3.411	0.220	6	493	ZINC000256069559
1.2	-8.8	-7.7	0.54	1.838	0.088	3.828	0.797	6	494	ZINC000014442509
1.2	-8.2	-7.4	0.41	2.446	0.308	3.994	0.538	6	495	ZINC000014652216
1.2	-7.6	-7.4	0.13	2.043	0.825	3.547	1.127	6	496	ZINC000001850909
1.2	-9.2	-8.2	0.62	1.882	0.607	4.204	2.016	6	497	ZINC000014502767
1.2	-9.0	-7.8	0.60	1.767	0.202	4.149	1.360	6	498	ZINC000086000065
1.2	-9.8	-8.8	0.79	1.578	0.430	4.180	1.110	6	499	ZINC000006484855
1.2	-10.8	-10.0	0.60	1.662	0.556	4.230	0.849	6	500	ZINC000257558733
1.2	-10.8	-9.7	0.71	2.036	0.378	5.590	1.219	6	501	ZINC000100780488
1.2	-8.2	-7.9	0.15	1.421	0.259	3.889	0.131	6	502	ZINC000059560110
1.2	-8.9	-8.2	0.57	1.885	0.202	4.094	1.273	6	503	ZINC000015214570

1.2	-10.2	-8.7	1.13	1.474	0.125	4.717	0.622	5	504	ZINC000257558884
1.2	-8.7	-8.1	0.42	1.795	0.454	4.088	0.433	6	505	ZINC000085600699
1.2	-8.5	-8.1	0.23	1.663	0.504	3.477	0.467	6	506	ZINC000013457480
1.2	-8.1	-7.2	0.56	2.548	0.812	4.318	1.839	6	507	ZINC000002509772
1.2	-10.0	-9.3	0.48	2.055	0.444	4.475	1.768	6	508	ZINC000000156701
1.2	-9.0	-8.8	0.18	1.001	0.575	4.279	2.692	6	509	ZINC000000014036
1.2	-8.4	-8.0	0.32	1.819	0.321	4.168	0.319	6	510	ZINC000085600702
1.2	-9.2	-8.2	0.62	1.920	1.017	4.222	2.015	6	511	ZINC000014858356
1.2	-8.4	-8.1	0.25	1.775	0.115	5.882	1.618	6	512	ZINC000003978525
1.2	-10.4	-9.3	1.10	1.463	0.589	2.654	0.852	3	513	ZINC000006069746
1.2	-9.9	-8.8	0.71	2.291	0.933	4.976	2.313	6	514	ZINC000014642759
1.2	-9.1	-8.5	0.31	2.173	0.398	5.056	1.302	6	515	ZINC000005735437
1.2	-7.3	-6.8	0.30	2.036	0.410	3.150	0.416	6	516	ZINC000084397766
1.2	-9.0	-8.2	0.37	2.183	0.105	4.439	0.430	6	517	ZINC000059778576
1.2	-10.4	-9.2	0.77	1.639	0.298	3.284	1.531	6	518	ZINC000003871017

1.2	-10.1	-9.1	0.67	2.302	0.282	4.340	0.964	6	519	ZINC000015264159
1.2	-9.3	-8.5	0.49	1.902	0.402	3.666	0.814	6	520	ZINC000014691905
1.2	-8.2	-7.8	0.30	1.592	0.285	3.554	0.875	6	521	ZINC000100779603
1.2	-8.5	-8.2	0.24	1.805	0.409	4.056	0.747	6	522	ZINC000000968236
1.2	-7.6	-7.1	0.32	1.566	0.290	3.696	0.504	6	523	ZINC000000410085
1.2	-11.5	-10.3	0.64	1.819	0.326	4.252	1.594	6	524	ZINC000100780562
1.2	-7.6	-7.2	0.26	1.935	0.398	3.931	0.494	6	525	ZINC000001850068
1.2	-9.8	-8.9	0.74	2.128	0.418	5.972	1.110	6	526	ZINC000000968075
1.2	-9.9	-9.1	0.46	1.884	0.133	4.768	0.279	6	527	ZINC000169479300
1.2	-9.3	-8.7	0.31	1.430	0.530	2.606	1.315	6	528	ZINC000014453724
1.2	-8.4	-7.6	0.40	1.825	0.375	3.497	1.158	6	529	ZINC000006031150
1.2	-8.3	-8.0	0.23	1.475	0.407	2.978	0.447	6	530	ZINC000000394916
1.2	-9.2	-8.5	0.41	1.360	0.206	3.805	1.156	6	531	ZINC000014449456
1.2	-7.9	-7.2	0.34	2.557	0.393	3.880	0.340	6	532	ZINC000004284429
1.2	-10.7	-9.6	0.66	1.660	0.223	4.499	1.528	6	533	ZINC000015253954

1.2	-8.3	-7.8	0.36	1.547	0.779	3.835	0.627	6	534	ZINC000002539631
1.2	-8.9	-8.0	0.51	1.649	0.336	2.824	1.125	6	535	ZINC000013371336
1.2	-7.3	-7.0	0.14	2.365	0.325	4.955	0.488	6	536	ZINC000001576485
1.2	-7.8	-7.2	0.42	1.658	0.458	3.084	1.202	6	537	ZINC000013333971
1.2	-10.9	-10.1	0.66	1.659	0.801	4.874	1.214	6	538	ZINC000012496246
1.1	-8.1	-7.7	0.33	1.760	0.547	5.322	1.178	6	539	ZINC000012496414
1.1	-9.1	-8.4	0.33	1.741	0.212	4.340	0.559	6	540	ZINC000015060164
1.1	-8.4	-7.9	0.23	1.560	0.250	3.431	1.087	6	541	ZINC000014764776
1.1	-8.7	-8.2	0.42	1.638	0.324	3.864	0.542	6	542	ZINC000085880356
1.1	-7.7	-7.2	0.26	2.373	0.692	3.434	0.445	6	543	ZINC000014588443
1.1	-8.9	-8.1	0.37	1.191	0.086	3.299	0.564	6	544	ZINC000056874634
1.1	-8.0	-6.9	0.82	2.304	0.436	5.161	1.611	6	545	ZINC000001643526
1.1	-10.2	-8.6	0.93	2.559	0.186	4.657	0.797	6	546	ZINC000014612198
1.1	-9.0	-8.3	0.47	1.846	0.364	3.689	0.744	6	547	ZINC000013143015
1.1	-8.9	-8.0	0.56	2.121	0.381	4.830	2.367	6	548	ZINC000014419490

1.1	-9.3	-8.8	0.39	3.264	0.519	5.519	1.189	6	549	ZINC000040165415
1.1	-6.7	-6.3	0.22	2.718	0.951	3.739	1.441	6	550	ZINC000002384572
1.1	-9.3	-8.7	0.38	1.762	0.207	3.243	1.306	6	551	ZINC000015058667
1.1	-9.6	-8.7	0.54	1.987	0.650	4.026	0.777	6	552	ZINC000003881431
1.1	-8.5	-8.2	0.21	1.765	0.340	4.586	2.330	6	553	ZINC000014720651
1.1	-9.2	-8.5	0.49	2.361	1.018	4.367	1.577	6	554	ZINC000004261903
1.1	-8.9	-8.3	0.38	1.270	0.367	3.075	0.931	6	555	ZINC000100776602
1.1	-6.6	-6.4	0.11	2.119	0.748	3.005	0.816	6	556	ZINC000100256102
1.1	-8.3	-7.9	0.20	1.475	0.186	3.566	0.875	6	557	ZINC000257398325
1.1	-10.9	-9.8	0.72	1.737	0.608	4.179	0.902	6	558	ZINC000014586956
1.1	-9.2	-8.4	0.40	2.458	0.820	3.944	1.817	6	559	ZINC000000395627
1.1	-8.8	-7.9	0.48	1.751	0.229	5.774	1.920	6	560	ZINC000014720657
1.1	-8.7	-7.9	0.40	1.644	0.144	3.385	0.584	6	561	ZINC000257542123
1.1	-7.8	-7.4	0.26	1.600	0.095	3.848	0.427	6	562	ZINC000015169218
1.1	-8.6	-8.0	0.34	2.088	0.587	3.216	1.182	6	563	ZINC000013783203

1.1	-8.6	-8.0	0.34	1.554	0.100	4.051	0.333	6	564	ZINC000014635815
1.1	-8.2	-7.9	0.25	1.733	0.405	3.955	0.316	6	565	ZINC000003881381
1.1	-6.2	-6.0	0.13	2.624	1.293	3.719	0.457	6	566	ZINC000001850686
1.1	-7.2	-7.0	0.16	1.348	0.219	2.646	0.453	6	567	ZINC000001710004
1.1	-8.6	-8.0	0.41	1.593	0.919	2.811	1.390	6	568	ZINC000008217123
1.1	-10.7	-9.1	0.99	1.557	0.278	3.032	0.731	4	569	ZINC000257525428
1.1	-8.9	-8.3	0.41	1.270	0.369	3.075	0.934	6	570	ZINC000100776600
1.1	-6.9	-6.4	0.29	2.002	0.463	3.601	0.812	6	571	ZINC000001577445
1.1	-7.6	-7.0	0.30	1.992	0.609	3.796	0.513	6	572	ZINC000012362794
1.1	-10.2	-9.3	0.54	1.545	0.353	5.005	0.972	6	573	ZINC000015150613
1.1	-8.6	-8.1	0.41	2.258	0.886	4.108	0.957	6	574	ZINC000005767707
1.1	-9.1	-8.2	0.46	1.149	0.360	3.308	1.968	6	575	ZINC000013340111
1.1	-8.8	-7.8	0.76	1.741	0.331	4.862	1.862	6	576	ZINC000257418691
1.1	-11.2	-9.6	1.13	2.482	0.204	3.169	0.328	3	577	ZINC000014610057
1.1	-6.9	-6.6	0.22	2.100	0.976	3.621	0.523	6	578	ZINC000001850660

1.1	-8.5	-8.2	0.19	2.139	0.304	3.505	0.584	6	579	ZINC000014594784
1.1	-8.8	-8.4	0.18	1.752	0.505	4.000	0.371	6	580	ZINC000085600895
1.1	-9.2	-8.8	0.20	2.050	0.278	4.533	0.269	6	581	ZINC000014919032
1.1	-9.1	-8.6	0.30	2.147	0.624	3.000	0.513	6	582	ZINC000006095281
1.1	-6.4	-6.1	0.20	1.806	0.755	3.522	1.094	6	583	ZINC000005132507
1.1	-6.9	-6.6	0.21	1.440	0.705	4.064	1.102	6	584	ZINC000003861298
1.1	-8.7	-8.5	0.23	1.733	0.502	4.656	1.488	6	585	ZINC000027638934
1.1	-7.9	-7.6	0.13	2.075	0.310	5.543	1.145	6	586	ZINC000085695221
1.1	-7.8	-7.3	0.32	1.360	0.600	4.016	2.588	6	587	ZINC000014504259
1.1	-9.5	-8.7	0.61	2.160	0.609	3.846	1.259	6	588	ZINC000006116532
1.1	-10.1	-9.2	0.51	1.918	0.360	4.023	1.549	6	589	ZINC000013379156
1.1	-8.5	-7.9	0.43	1.767	0.486	3.480	1.235	6	590	ZINC000000394327
1.1	-9.2	-8.3	0.77	1.716	0.317	3.973	0.868	6	591	ZINC000257561484
1.1	-10.7	-10.0	0.40	1.606	0.197	4.782	1.448	6	592	ZINC000257802860
1.1	-9.3	-8.5	0.56	1.768	0.562	3.345	0.610	6	593	ZINC000100822123

1.1	-10.7	-9.9	0.53	1.568	0.276	5.068	1.498	6	594	ZINC000257802861
1.1	-8.9	-7.9	0.58	1.830	0.108	3.982	2.061	6	595	ZINC000257507101
1.1	-8.7	-8.0	0.32	2.138	0.291	4.887	0.985	6	596	ZINC000015160972
1.1	-8.6	-8.2	0.23	1.759	0.480	4.286	1.108	6	597	ZINC000027645229
1.1	-8.2	-8.0	0.18	1.552	0.131	3.633	0.134	6	598	ZINC000015214531
1.1	-9.8	-8.7	0.57	2.320	0.688	3.763	1.486	6	599	ZINC000095617645
1.1	-9.0	-7.8	0.90	1.633	0.194	4.119	0.547	6	600	ZINC000257561486
1.1	-11.3	-10.3	0.66	1.271	0.176	3.193	0.945	6	601	ZINC000095619420
1.1	-11.3	-10.8	0.24	1.400	0.418	3.996	2.013	6	602	ZINC000257454023
1.1	-10.2	-9.1	0.56	2.317	0.503	4.719	0.906	6	603	ZINC000014445178
1.1	-8.1	-7.8	0.21	1.639	0.451	2.614	0.587	6	604	ZINC000002018181
1.1	-7.9	-7.3	0.29	2.055	0.349	3.109	0.590	6	605	ZINC000013462240
1.1	-7.7	-7.1	0.28	2.122	0.989	2.888	1.129	6	606	ZINC000002018981
1.1	-9.4	-9.0	0.37	1.920	0.740	3.111	0.987	6	607	ZINC000002383046
1.1	-8.3	-7.9	0.29	1.781	0.320	3.830	0.584	6	608	ZINC000014854737

1.1	-10.0	-8.9	0.78	1.918	0.478	4.107	2.053	6	609	ZINC000257799583
1.1	-7.1	-6.8	0.18	1.948	0.511	2.804	0.573	6	610	ZINC000008613400
1.1	-10.7	-10.1	0.40	1.565	0.153	4.641	1.521	5	611	ZINC000100780542
1.1	-7.2	-7.0	0.09	2.054	0.567	3.747	0.812	6	612	ZINC000004521483
1.1	-8.9	-7.7	0.74	1.933	0.607	4.449	2.408	6	613	ZINC000257507103
1.1	-7.6	-7.1	0.22	1.753	0.484	4.052	1.538	6	614	ZINC000003197530
1.1	-8.0	-7.5	0.27	2.631	0.276	5.528	0.870	6	615	ZINC000014679996
1.1	-10.3	-9.3	0.62	2.186	0.261	5.654	1.264	6	616	ZINC000000073693
1.1	-8.0	-7.6	0.33	1.399	0.228	4.159	0.307	6	617	ZINC000015214521
1.1	-6.2	-6.0	0.11	2.051	0.472	3.701	0.600	6	618	ZINC000001529847
1.1	-9.6	-8.3	0.88	2.188	0.337	4.513	0.487	6	619	ZINC000100780334
1.1	-8.5	-8.3	0.12	1.624	0.556	4.340	2.237	6	620	ZINC000002389524
1.1	-7.5	-7.3	0.09	1.388	0.127	3.199	0.241	6	621	ZINC000003870303
1.1	-8.7	-7.7	0.53	1.818	0.267	3.238	0.968	6	622	ZINC000013371324
1.1	-10.9	-9.9	0.63	1.538	0.494	4.161	0.902	6	623	ZINC000100779686

1.1	-9.0	-8.1	0.47	2.808	0.352	4.474	1.157	6	624	ZINC000005115873
1.1	-9.3	-8.3	0.57	1.811	0.777	5.039	0.671	6	625	ZINC000002585551
1.1	-9.2	-8.5	0.41	1.491	0.220	2.763	1.472	6	626	ZINC000005352272
1.1	-8.0	-7.4	0.30	1.858	0.303	3.300	1.145	6	627	ZINC000006037706
1.1	-7.6	-7.3	0.21	2.499	0.624	4.059	0.445	6	628	ZINC000038644160
1.1	-8.6	-8.0	0.34	1.112	0.234	2.780	0.708	6	629	ZINC000015115274
1.1	-10.0	-9.1	0.67	1.961	0.321	5.894	1.344	6	630	ZINC000014806930
1.1	-8.5	-8.0	0.35	1.554	0.403	3.794	1.215	6	631	ZINC000015201060
1.1	-8.5	-7.8	0.45	1.636	0.186	3.012	0.768	6	632	ZINC000015169303
1.1	-8.3	-7.9	0.28	1.354	0.193	3.823	0.126	6	633	ZINC000070454715
1.1	-7.7	-7.1	0.39	2.470	0.357	3.729	0.713	6	634	ZINC000032302828
1.1	-8.6	-8.2	0.30	1.691	0.335	3.895	0.831	6	635	ZINC000014413206
1.1	-9.7	-9.2	0.27	2.159	0.165	4.427	0.700	6	636	ZINC000000901754
1.1	-8.3	-8.0	0.19	2.294	0.389	3.799	0.671	6	637	ZINC000100776450
1.1	-8.7	-8.0	0.38	1.846	0.577	3.822	0.721	6	638	ZINC000085599410

1.1	-9.4	-7.9	0.88	1.697	0.459	4.803	1.908	6	639	ZINC000033963724
1.1	-7.6	-6.9	0.34	2.253	0.668	3.740	1.210	6	640	ZINC000000391550
1.1	-8.9	-8.1	0.46	2.085	0.354	3.903	1.894	6	641	ZINC000013350928
1.1	-9.2	-8.1	0.57	2.182	0.158	5.216	1.487	6	642	ZINC000002525300
1.1	-10.1	-9.0	0.80	1.628	0.494	5.263	1.803	6	643	ZINC000012496354
1.1	-10.5	-9.4	0.79	1.556	0.205	4.530	1.686	5	644	ZINC000006090917
1.1	-10.2	-9.8	0.31	1.216	0.223	6.093	2.091	6	645	ZINC000000519621
1.1	-7.0	-6.5	0.29	2.571	0.396	3.983	0.517	6	646	ZINC000005761926
1.1	-6.4	-6.0	0.22	1.915	0.309	2.687	0.431	6	647	ZINC000100828322
1.1	-8.8	-8.2	0.50	2.043	0.160	3.945	0.373	6	648	ZINC000257540250
1.1	-7.8	-7.6	0.15	1.063	0.202	2.898	1.062	6	649	ZINC000015219238
1.1	-9.2	-8.1	0.70	1.436	0.313	3.825	0.478	6	650	ZINC000257579929
1.1	-7.3	-6.8	0.30	2.053	0.531	3.434	0.573	6	651	ZINC000257547802
1.1	-9.8	-8.7	0.75	1.695	0.651	4.577	1.930	6	652	ZINC000008681596
1.1	-7.3	-6.9	0.25	2.183	0.701	3.472	0.782	6	653	ZINC000014588889

1.1	-7.3	-6.9	0.25	2.175	0.700	3.531	0.698	6	654	ZINC000014588888
1.1	-8.6	-8.0	0.31	1.779	0.386	4.307	0.399	6	655	ZINC000085880170
1.1	-7.3	-7.0	0.16	2.393	0.552	4.574	1.489	6	656	ZINC000014610795
1.1	-11.4	-10.4	0.62	1.679	0.336	3.777	1.471	4	657	ZINC000100780554
1.1	-7.6	-7.2	0.20	1.703	0.278	3.972	0.657	6	658	ZINC000004654657
1.1	-8.3	-8.2	0.13	1.384	0.135	3.446	0.094	6	659	ZINC000085883134
1.1	-10.3	-9.1	0.84	1.652	0.090	4.415	1.547	5	660	ZINC000013308681
1.1	-9.1	-7.8	0.70	1.736	0.339	3.996	0.275	6	661	ZINC000014441664
1.1	-8.7	-7.8	0.43	1.483	0.555	4.886	1.354	6	662	ZINC000001575138
1.1	-10.0	-8.5	0.81	2.604	0.311	5.576	0.655	6	663	ZINC000031318338
1.1	-7.9	-7.5	0.27	2.639	0.507	4.015	1.346	6	664	ZINC000004096483
1.1	-10.1	-9.5	0.40	2.020	0.676	4.725	2.135	6	665	ZINC000000004935
1.1	-6.9	-6.6	0.18	2.144	0.473	4.151	0.456	6	666	ZINC000000391812
1.1	-8.2	-7.8	0.24	1.387	0.316	3.439	0.601	6	667	ZINC000085967184
1.1	-9.4	-8.5	0.50	1.749	0.305	6.293	1.377	6	668	ZINC000005998787

1.1	-8.8	-8.4	0.33	1.784	0.259	4.177	0.471	6	669	ZINC000014441666
1.1	-6.6	-6.4	0.10	1.690	0.710	2.535	0.575	6	670	ZINC000005761276
1.1	-9.6	-8.3	0.84	1.683	0.511	3.422	1.623	5	671	ZINC000104883842
1.1	-8.7	-8.1	0.33	1.840	0.210	3.611	0.581	6	672	ZINC000257453298
1.1	-8.2	-7.6	0.37	2.351	0.195	4.238	0.434	6	673	ZINC000015169242
1.1	-10.5	-9.0	0.78	1.707	0.411	4.410	1.523	6	674	ZINC000014918951
1.1	-7.2	-6.8	0.20	2.476	0.552	4.172	0.696	6	675	ZINC000095618078
1.1	-6.4	-5.9	0.25	2.041	0.596	3.024	0.842	6	676	ZINC000000336705
1.1	-7.7	-7.2	0.24	2.178	0.419	3.665	0.696	6	677	ZINC000004023242
1.1	-5.8	-5.6	0.10	1.073	0.147	2.836	0.590	6	678	ZINC000002039808
1.1	-8.9	-8.3	0.50	1.711	0.203	3.644	0.333	6	679	ZINC000085767734
1.1	-6.6	-6.3	0.17	1.247	0.080	3.727	1.492	6	680	ZINC000001648322
1.1	-9.8	-8.6	0.58	2.094	0.450	3.685	0.943	6	681	ZINC000015155029
1.1	-10.1	-9.3	0.88	1.075	0.007	2.155	0.090	3	682	ZINC000253604820
1.1	-9.3	-8.4	0.49	1.770	0.485	4.019	1.170	6	683	ZINC000095617918

1.1	-8.8	-8.0	0.40	1.973	0.182	5.709	1.090	6	684	ZINC000257635843
1.1	-11.3	-10.2	0.81	1.854	0.917	4.049	0.905	6	685	ZINC000095619421
1.1	-6.5	-6.2	0.18	1.552	0.226	3.303	1.018	6	686	ZINC000078275012
1.1	-7.4	-6.9	0.28	1.804	0.343	2.977	0.684	6	687	ZINC000000080846
1.1	-8.1	-7.4	0.36	1.298	0.082	2.919	1.075	6	688	ZINC000100776280
1.1	-9.6	-9.2	0.27	2.501	0.514	3.736	0.528	6	689	ZINC000100779994
1.1	-7.8	-7.3	0.23	2.830	0.572	4.318	0.702	6	690	ZINC000100774483
1.1	-7.7	-7.4	0.25	2.396	0.466	4.251	0.968	6	691	ZINC000261496280
1.1	-8.5	-7.9	0.34	1.809	0.122	3.732	0.506	6	692	ZINC000012153512
1.1	-8.6	-8.1	0.31	2.548	0.432	3.050	0.518	6	693	ZINC000000025672
1.1	-9.6	-9.2	0.32	2.123	0.784	3.248	0.802	6	694	ZINC000100779996
1.1	-9.6	-8.7	0.52	2.270	0.286	4.202	1.568	6	695	ZINC000014618589
1.1	-8.8	-8.0	0.43	1.505	0.180	3.134	1.186	6	696	ZINC000086000068
1.1	-10.4	-8.7	0.89	2.232	0.344	4.622	1.464	5	697	ZINC000013328438
1.1	-8.5	-7.9	0.33	1.646	0.066	3.365	0.428	6	698	ZINC000257542122

1.1	-8.4	-8.0	0.27	1.184	0.076	3.575	0.753	6	699	ZINC000100780737
1.1	-8.1	-7.6	0.31	1.828	0.658	3.195	1.440	6	700	ZINC000006031585
1.1	-7.9	-7.6	0.29	1.581	1.357	3.998	0.810	6	701	ZINC000015122123
1.1	-11.3	-10.2	0.81	1.411	0.308	3.656	0.630	6	702	ZINC000095619423
1.1	-7.9	-7.5	0.22	1.610	0.274	3.609	0.368	6	703	ZINC000001850504
1.1	-9.3	-9.0	0.27	2.217	0.496	4.083	0.476	6	704	ZINC000015160853
1.1	-7.1	-6.8	0.18	2.136	0.444	4.061	0.484	6	705	ZINC000003869622
1.1	-9.6	-9.2	0.34	1.641	0.432	4.536	2.052	6	706	ZINC000014645987
1.1	-6.5	-6.2	0.16	2.653	0.339	3.214	0.426	6	707	ZINC000005191778
1.1	-7.6	-7.5	0.09	1.389	0.194	5.270	2.336	6	708	ZINC000014725859
1.1	-6.7	-6.2	0.28	2.176	1.187	3.400	1.844	6	709	ZINC000034065042
1.1	-8.2	-7.7	0.35	2.101	0.342	4.020	0.585	6	710	ZINC000014688567
1.1	-10.2	-9.5	0.68	1.976	0.157	5.152	1.516	3	711	ZINC000002561261
1.1	-10.7	-9.4	1.07	1.851	0.394	4.861	1.499	5	712	ZINC000100780550
1.1	-7.9	-7.5	0.24	1.659	0.067	3.301	0.676	6	713	ZINC000015169224

1.1	-10.3	-9.4	0.65	1.707	0.770	4.606	2.268	6	714	ZINC000077312860
1.1	-10.9	-9.9	0.72	1.659	0.653	4.206	0.844	6	715	ZINC000257507657
1.1	-9.5	-8.6	0.50	2.151	0.446	4.956	1.553	6	716	ZINC000002565013
1.1	-9.5	-8.4	0.64	1.753	0.344	4.224	2.248	6	717	ZINC000012153524
1.1	-8.4	-8.0	0.24	2.014	0.553	4.664	0.218	6	718	ZINC000100772843
1.1	-9.7	-8.9	0.62	1.965	0.301	4.871	1.514	6	719	ZINC000095620871
1.1	-7.2	-6.4	0.37	2.244	0.669	4.081	1.460	6	720	ZINC000006031308
1.1	-7.8	-7.5	0.18	2.560	0.480	4.513	0.823	6	721	ZINC000001850073
1.1	-8.9	-8.0	0.47	1.725	0.293	3.452	1.187	6	722	ZINC000015220873
1.1	-8.8	-8.4	0.21	1.785	0.271	4.162	0.487	6	723	ZINC000014441680
1.1	-7.4	-7.1	0.20	1.934	0.345	3.360	0.218	6	724	ZINC000000899402
1.1	-9.2	-8.7	0.26	2.320	0.354	5.431	1.042	6	725	ZINC000014502737
1.1	-8.0	-7.6	0.27	1.860	0.287	3.985	0.295	6	726	ZINC000257545327
1.1	-11.4	-10.3	0.62	1.823	0.326	4.254	1.592	6	727	ZINC000257537975
1.1	-6.5	-6.4	0.08	1.632	0.454	4.454	1.242	6	728	ZINC000003875772

1.1	-7.3	-7.0	0.19	2.076	0.407	3.357	1.533	6	729	ZINC000015261530
1.1	-8.0	-7.8	0.13	1.756	0.087	3.776	0.501	6	730	ZINC000034569092
1.1	-8.1	-7.6	0.28	2.704	0.729	3.989	0.957	6	731	ZINC000030731200
1.1	-9.7	-9.2	0.31	1.585	0.334	4.324	1.124	6	732	ZINC000014693981
1.1	-8.0	-7.8	0.16	2.326	0.558	4.217	0.644	6	733	ZINC000014593123
1.1	-10.2	-9.3	0.63	1.640	0.497	2.970	1.686	6	734	ZINC000013302910
1.1	-10.2	-9.2	0.84	1.618	0.430	5.732	2.122	6	735	ZINC000000895953
1.1	-8.5	-8.2	0.17	1.545	0.366	4.231	0.267	6	736	ZINC000008234281
1.1	-7.7	-7.0	0.37	2.298	0.547	3.465	1.398	6	737	ZINC000005933671
1.1	-6.6	-6.4	0.10	1.791	0.661	2.598	0.577	6	738	ZINC000100256099
1.1	-10.9	-10.0	0.64	1.510	0.510	4.365	0.824	6	739	ZINC000100779691
1.1	-7.7	-7.2	0.23	2.376	0.636	3.400	0.286	6	740	ZINC000014588444
1.1	-8.6	-8.0	0.33	1.780	0.160	4.744	0.901	6	741	ZINC000014507220
1.1	-11.3	-10.7	0.46	1.464	0.368	5.088	1.608	6	742	ZINC000257454022
1.1	-7.5	-7.1	0.17	1.660	0.172	3.072	0.439	6	743	ZINC000100780395

1.1	-8.2	-7.8	0.28	1.678	0.172	3.548	0.721	6	744	ZINC000015169220
1.1	-9.9	-9.0	0.43	1.879	0.120	5.490	1.339	6	745	ZINC000014636764
1.1	-7.9	-7.3	0.37	2.529	0.405	5.158	0.266	6	746	ZINC000001576266
1.1	-7.4	-7.1	0.15	1.516	0.108	3.080	0.664	6	747	ZINC000003870304
1.1	-9.5	-8.8	0.47	1.880	0.166	5.138	1.872	6	748	ZINC000006093219
1.1	-10.9	-9.7	1.07	1.455	0.204	4.307	1.480	6	749	ZINC000002039832
1.1	-6.9	-6.6	0.19	2.016	0.180	3.639	0.557	6	750	ZINC000005808686
1.1	-7.6	-7.3	0.20	1.689	0.293	4.470	0.659	6	751	ZINC000004655406
1.1	-7.9	-7.7	0.11	2.127	0.518	4.417	0.887	6	752	ZINC000014449721
1.1	-7.9	-7.6	0.28	1.300	0.144	3.489	0.491	6	753	ZINC000100779588
1.1	-6.6	-6.4	0.11	2.067	0.820	3.045	0.771	6	754	ZINC000005761501
1.1	-9.5	-8.8	0.63	1.698	0.365	3.687	1.140	6	755	ZINC000015055176
1.1	-8.1	-7.3	0.41	1.482	0.353	4.228	1.045	6	756	ZINC000003650145
1.1	-7.5	-7.2	0.22	1.654	0.338	3.773	0.394	6	757	ZINC000014588965
1.1	-6.9	-6.4	0.25	2.098	0.337	3.376	0.583	6	758	ZINC000015274294

1.1	-8.0	-7.5	0.29	1.654	0.411	5.154	1.803	6	759	ZINC000012496824
1.1	-6.6	-6.0	0.36	2.125	0.395	3.115	0.299	6	760	ZINC000251663874
1.1	-8.1	-7.2	0.43	1.989	0.594	3.775	1.125	6	761	ZINC000013437345
1.1	-11.3	-10.2	0.77	1.462	0.348	3.553	0.658	6	762	ZINC000095619422
1.1	-10.4	-9.3	0.76	1.667	0.372	4.070	1.078	6	763	ZINC000004046842
1.1	-7.9	-7.4	0.25	2.815	0.333	4.393	1.074	6	764	ZINC000012495791
1.1	-7.3	-6.8	0.29	1.859	0.478	3.322	0.644	6	765	ZINC000257547803
1.1	-8.3	-8.0	0.17	1.762	0.299	3.905	0.155	6	766	ZINC000086021153
1.1	-8.2	-7.8	0.32	2.625	0.345	4.697	1.673	6	767	ZINC000004096827
1.1	-8.9	-8.0	0.48	2.110	0.403	3.942	1.885	6	768	ZINC000257507102
1.1	-8.8	-8.6	0.12	2.086	0.371	6.108	0.211	6	769	ZINC000000899567
1.1	-7.5	-7.2	0.18	3.131	0.718	4.813	1.334	6	770	ZINC000000393724
1.1	-7.3	-6.8	0.29	1.862	0.466	3.389	0.615	6	771	ZINC000014588886
1.1	-7.3	-6.9	0.25	2.166	0.708	3.522	0.789	6	772	ZINC000014588887
1.1	-8.9	-8.3	0.28	1.963	0.417	3.521	1.586	6	773	ZINC000006067251

1.1	-7.2	-6.6	0.30	2.155	0.333	3.650	0.392	6	774	ZINC000086034091
1.1	-9.3	-8.8	0.35	2.017	0.347	3.037	0.651	6	775	ZINC000086036336
1.1	-7.1	-6.6	0.29	2.419	0.235	3.868	0.211	6	776	ZINC000000389504
1.1	-6.5	-6.4	0.10	2.333	0.803	3.156	0.858	6	777	ZINC000000407077
1.1	-7.0	-6.6	0.22	2.633	0.591	3.339	0.831	6	778	ZINC000005457663
1.1	-6.3	-5.9	0.21	2.563	1.369	3.398	1.430	6	779	ZINC000095619614
1.1	-9.0	-8.2	0.55	1.815	0.219	3.851	0.562	6	780	ZINC000013322532
1.1	-9.2	-8.2	0.49	2.162	0.222	4.452	0.354	6	781	ZINC000015055155
1.1	-7.9	-7.7	0.11	2.318	0.143	3.613	0.328	6	782	ZINC000034278895
1.1	-7.1	-6.5	0.36	2.345	0.769	3.723	0.626	6	783	ZINC000001729185
1.1	-6.9	-6.6	0.25	1.754	0.590	2.972	0.570	6	784	ZINC000002567866
1.1	-6.7	-6.4	0.18	1.718	0.312	3.089	0.325	6	785	ZINC000001747064
1.1	-8.6	-8.0	0.29	1.941	0.441	4.547	0.494	6	786	ZINC000013374018
1.1	-6.9	-6.4	0.28	1.990	0.317	3.439	0.641	6	787	ZINC000013429398
1.1	-10.0	-8.4	1.04	1.183	0.105	4.237	1.590	5	788	ZINC000253604821

1.1	-10.8	-9.2	0.99	2.083	0.670	4.579	0.500	4	789	ZINC000100891773
1.1	-9.7	-8.8	0.63	2.280	0.358	5.058	1.757	6	790	ZINC000014728045
1.1	-9.3	-8.6	0.52	2.275	0.629	4.407	1.332	6	791	ZINC000050027755
1.1	-11.3	-10.7	0.45	1.467	0.376	5.088	1.608	6	792	ZINC000257454021
1.1	-9.6	-8.8	0.63	2.160	0.236	5.045	1.486	6	793	ZINC000014811638
1.1	-8.4	-7.5	0.52	1.658	0.079	3.826	0.635	6	794	ZINC000034085486
1.1	-6.6	-6.2	0.21	1.277	0.256	2.567	0.407	6	795	ZINC000002038648
1.1	-8.1	-7.5	0.40	2.117	0.183	4.085	0.436	6	796	ZINC000005412472
1.1	-8.5	-8.2	0.16	1.497	0.075	3.105	0.549	6	797	ZINC000005762483
1.1	-9.6	-8.9	0.45	1.737	0.142	3.896	0.962	6	798	ZINC000100825885
1.1	-9.7	-8.6	0.66	1.572	0.406	4.912	1.966	6	799	ZINC000014814445
1.1	-9.7	-9.1	0.33	2.591	0.564	4.388	1.330	6	800	ZINC000034921122
1.1	-10.6	-9.1	0.95	1.617	0.243	2.816	0.110	4	801	ZINC000005104405
1.1	-8.7	-8.2	0.27	1.530	0.255	3.727	1.335	6	802	ZINC000015060166
1.1	-9.1	-8.4	0.41	2.070	0.507	3.656	1.412	6	803	ZINC000006067252

1.1	-7.8	-7.5	0.16	1.781	0.225	5.007	0.947	6	804	ZINC000002165898
1.1	-7.2	-6.8	0.21	1.739	0.288	2.919	0.763	6	805	ZINC000001087344
1.1	-11.3	-10.4	0.95	1.456	0.000	5.315	0.000	2	806	ZINC000014636695
1.1	-9.7	-8.9	0.38	2.047	0.241	5.113	0.648	6	807	ZINC000022060952
1.1	-8.9	-7.7	0.70	2.064	0.219	4.369	1.094	6	808	ZINC000005689010
1.1	-8.7	-7.8	0.41	1.585	0.169	3.304	0.907	6	809	ZINC000100825788
1.1	-9.5	-9.1	0.34	1.795	0.228	4.225	1.112	6	810	ZINC000014826955
1.1	-10.6	-9.2	1.45	2.117	0.000	5.029	0.000	2	811	ZINC000014410875
1.1	-8.8	-8.1	0.35	1.469	0.386	3.952	1.068	6	812	ZINC000059200498
1.1	-9.6	-9.0	0.47	2.076	0.395	3.542	0.946	6	813	ZINC000169440689
1.0	-9.1	-8.7	0.31	1.915	0.288	3.622	1.050	6	814	ZINC000257502422
1.0	-6.6	-6.2	0.17	1.443	0.235	3.185	0.663	6	815	ZINC000001699441
1.0	-9.0	-8.5	0.28	1.551	0.247	2.376	0.462	6	816	ZINC000004578920
1.0	-10.4	-9.7	0.59	1.404	0.488	3.604	1.101	6	817	ZINC000100779671
1.0	-7.9	-7.8	0.08	2.255	0.426	3.377	0.920	6	818	ZINC000000901470

1.0	-8.2	-7.8	0.20	1.580	0.164	3.264	0.594	6	819	ZINC000013457484
1.0	-11.0	-9.9	0.88	1.964	0.522	4.705	1.643	6	820	ZINC000257530220
1.0	-8.1	-7.6	0.34	1.737	0.509	3.705	0.948	6	821	ZINC000004216848
1.0	-7.2	-6.9	0.20	2.587	0.531	4.093	1.132	6	822	ZINC000014588969
1.0	-9.5	-8.2	0.67	2.089	0.238	3.845	1.001	6	823	ZINC000013486153
1.0	-6.7	-6.2	0.27	1.593	0.389	3.163	1.068	6	824	ZINC000014438709
1.0	-6.8	-6.5	0.16	1.312	0.403	2.657	0.505	6	825	ZINC000000968028
1.0	-7.8	-7.2	0.28	2.005	0.437	3.813	0.694	6	826	ZINC000005273745
1.0	-9.4	-8.4	0.56	1.799	0.244	4.637	1.924	6	827	ZINC000014593852
1.0	-7.4	-6.9	0.23	1.807	0.229	4.216	1.171	6	828	ZINC000014455358
1.0	-7.2	-6.7	0.28	1.967	0.724	3.947	1.251	6	829	ZINC000000081248
1.0	-9.5	-8.0	1.03	1.631	0.332	4.539	1.974	6	830	ZINC000257554795
1.0	-7.7	-7.1	0.26	1.816	0.295	4.306	0.852	6	831	ZINC000008860474
1.0	-9.6	-8.1	0.79	1.821	0.249	4.330	1.058	5	832	ZINC000015216878
1.0	-7.1	-6.6	0.26	2.346	0.597	4.015	0.805	6	833	ZINC000000001080

1.0	-8.6	-8.1	0.28	1.600	0.716	4.331	2.209	6	834	ZINC000013537352
1.0	-7.0	-6.6	0.25	1.833	0.855	4.246	0.854	6	835	ZINC000001713247
1.0	-7.8	-7.4	0.26	2.053	0.635	3.206	1.505	6	836	ZINC000000895844
1.0	-9.0	-8.6	0.24	2.001	0.877	3.298	0.832	6	837	ZINC000104378338
1.0	-8.1	-7.5	0.42	1.669	0.177	3.947	1.152	6	838	ZINC000014635819
1.0	-8.2	-7.9	0.28	2.683	0.561	4.775	0.880	6	839	ZINC000256082972
1.0	-8.0	-7.6	0.22	2.056	0.097	4.577	0.506	6	840	ZINC000012495586
1.0	-6.4	-6.0	0.19	2.211	0.912	3.966	1.179	6	841	ZINC000001677787
1.0	-8.5	-7.9	0.36	2.262	0.474	4.454	1.624	6	842	ZINC000013482896
1.0	-8.3	-8.0	0.26	1.518	0.386	5.245	1.900	6	843	ZINC000004655435
1.0	-11.0	-10.0	0.68	1.587	0.365	4.816	1.709	6	844	ZINC000100780564
1.0	-8.3	-7.7	0.37	2.190	0.708	3.615	0.569	6	845	ZINC000030728460
1.0	-9.7	-8.9	0.53	2.317	0.535	4.173	1.399	6	846	ZINC000257428490
1.0	-7.5	-7.2	0.26	2.503	0.874	4.167	0.706	6	847	ZINC000003999315
1.0	-7.6	-7.1	0.38	1.290	0.617	4.291	1.439	6	848	ZINC000001570047

1.0	-9.3	-9.0	0.36	1.800	0.422	4.158	1.731	6	849	ZINC000014728051
1.0	-9.7	-9.4	0.16	2.301	0.387	4.287	1.740	6	850	ZINC000003978494
1.0	-8.7	-8.5	0.16	1.576	0.219	3.640	0.551	6	851	ZINC000015160952
1.0	-8.8	-7.7	0.57	1.776	0.145	4.126	1.263	6	852	ZINC000261497624
1.0	-7.4	-6.7	0.34	3.695	1.340	5.081	0.863	6	853	ZINC000000896284
1.0	-8.6	-8.4	0.15	1.715	0.567	4.875	1.756	6	854	ZINC000002513716
1.0	-9.0	-8.6	0.27	2.332	1.007	3.815	1.000	6	855	ZINC000100783136
1.0	-9.1	-7.7	0.66	1.674	0.147	4.238	1.131	6	856	ZINC000257466070
1.0	-8.9	-8.4	0.44	2.620	0.456	4.966	1.177	6	857	ZINC000012501482
1.0	-8.0	-7.8	0.16	1.879	0.273	4.154	0.485	6	858	ZINC000100779600
1.0	-7.4	-7.2	0.09	2.901	0.568	4.773	0.489	6	859	ZINC000014588970
1.0	-11.0	-10.2	0.42	1.616	0.312	3.863	2.129	6	860	ZINC000257530219
1.0	-7.7	-7.5	0.12	1.665	0.221	3.373	0.457	6	861	ZINC000001850505
1.0	-8.4	-8.1	0.20	1.646	0.478	3.449	1.698	6	862	ZINC000013340553
1.0	-7.8	-7.4	0.30	1.481	0.485	3.861	1.299	6	863	ZINC000070451064

1.0	-10.9	-10.1	0.38	1.186	0.199	3.557	1.063	6	864	ZINC000100775943
1.0	-6.4	-6.3	0.04	1.589	0.315	4.718	0.646	6	865	ZINC000001530705
1.0	-10.6	-9.2	0.70	1.850	0.359	4.076	1.288	6	866	ZINC000257446473
1.0	-7.8	-7.5	0.18	2.334	0.695	4.114	0.554	6	867	ZINC000001850826
1.0	-8.4	-7.6	0.38	2.745	0.345	4.672	1.315	6	868	ZINC000005114090
1.0	-9.7	-8.9	0.65	1.858	0.372	4.602	1.270	6	869	ZINC000257791956
1.0	-9.1	-8.3	0.53	1.169	0.316	3.235	1.092	6	870	ZINC000100775068
1.0	-9.8	-9.2	0.37	1.441	0.426	5.246	2.588	6	871	ZINC000014437249
1.0	-11.3	-10.3	0.64	1.849	0.337	4.223	1.554	6	872	ZINC000257537976
1.0	-10.2	-8.5	0.82	1.439	0.328	4.751	1.532	6	873	ZINC000003875364
1.0	-10.2	-9.0	0.64	1.784	0.209	3.916	1.761	6	874	ZINC000003651278
1.0	-8.6	-7.8	0.40	1.195	0.208	2.846	0.745	6	875	ZINC000015115278
1.0	-10.2	-9.1	0.71	1.767	0.231	4.218	2.197	6	876	ZINC000000265501
1.0	-6.5	-6.4	0.08	2.344	1.204	3.833	1.559	6	877	ZINC000002567598
1.0	-7.7	-7.2	0.24	1.722	0.590	2.411	0.619	6	878	ZINC000002559095

1.0	-8.9	-7.8	0.62	1.777	0.423	3.556	1.228	6	879	ZINC000238755630
1.0	-8.5	-8.2	0.28	1.502	0.207	3.197	0.416	6	880	ZINC000015120365
1.0	-8.5	-8.1	0.42	1.253	0.338	2.882	0.430	6	881	ZINC000014720699
1.0	-7.4	-6.8	0.28	2.331	0.276	3.876	0.313	6	882	ZINC000014695395
1.0	-8.7	-8.0	0.46	2.125	0.529	4.557	1.416	6	883	ZINC000014687857
1.0	-6.2	-6.0	0.18	1.916	0.147	3.858	0.337	6	884	ZINC000001638430
1.0	-9.0	-8.7	0.25	2.222	0.126	4.612	0.846	6	885	ZINC000100772314
1.0	-7.1	-6.7	0.24	1.318	0.600	3.651	0.791	6	886	ZINC000000347833
1.0	-9.2	-8.1	0.53	1.797	0.255	3.480	0.738	6	887	ZINC000013838497
1.0	-6.8	-6.6	0.13	2.430	0.415	3.929	0.797	6	888	ZINC000038611097
1.0	-9.8	-8.8	0.85	1.954	0.374	4.406	1.530	6	889	ZINC000014887298
1.0	-9.2	-7.8	0.79	1.861	0.215	4.162	0.844	6	890	ZINC000015055173
1.0	-8.9	-8.0	0.47	1.972	0.325	4.072	1.815	6	891	ZINC000257507104
1.0	-7.6	-7.1	0.21	2.397	0.688	3.719	0.912	6	892	ZINC000012496253
1.0	-8.7	-7.9	0.40	2.006	0.342	4.174	0.417	6	893	ZINC000005767672

1.0	-7.7	-7.3	0.25	1.674	0.337	4.642	0.863	6	894	ZINC000004655387
1.0	-10.4	-9.0	0.97	1.626	0.208	3.863	1.552	4	895	ZINC000014887284
1.0	-10.6	-9.5	0.90	1.573	0.469	5.164	1.744	6	896	ZINC000004023792
1.0	-9.4	-8.8	0.41	1.950	0.557	5.168	1.191	6	897	ZINC000000895551
1.0	-6.5	-6.2	0.15	2.592	0.324	4.266	0.405	6	898	ZINC000001707854
1.0	-9.1	-8.4	0.41	2.204	0.480	5.751	1.486	6	899	ZINC000004655109
1.0	-8.3	-7.9	0.27	1.736	0.104	4.509	1.959	6	900	ZINC000100825940
1.0	-7.5	-6.9	0.32	1.901	0.595	3.420	1.712	6	901	ZINC000034039988
1.0	-7.8	-7.6	0.11	2.146	0.471	4.969	0.782	6	902	ZINC000004546585
1.0	-8.6	-7.8	0.37	2.265	0.290	3.993	0.541	6	903	ZINC000014556676
1.0	-8.4	-8.0	0.20	1.694	0.132	3.294	0.377	6	904	ZINC000003978564
1.0	-8.0	-7.8	0.15	1.332	0.213	3.416	1.225	6	905	ZINC000086032743
1.0	-10.8	-9.1	0.84	1.929	0.140	7.117	0.330	6	906	ZINC000100828781
1.0	-7.9	-7.3	0.38	2.130	0.530	3.280	1.049	6	907	ZINC000006072259
1.0	-8.3	-7.2	0.72	2.533	0.477	4.520	1.063	6	908	ZINC000034125584

1.0	-10.4	-9.3	0.57	2.286	0.592	5.362	1.063	6	909	ZINC000014612644
1.0	-7.4	-7.1	0.18	1.931	0.939	4.021	0.850	6	910	ZINC000002015842
1.0	-9.8	-7.9	0.86	1.872	0.373	3.547	1.501	6	911	ZINC000032286633
1.0	-10.9	-10.0	0.43	1.488	0.633	3.364	0.995	6	912	ZINC000100775946
1.0	-11.3	-10.3	0.66	1.831	0.337	4.220	1.554	6	913	ZINC000257537977
1.0	-9.8	-8.3	1.10	1.719	0.340	4.154	1.621	5	914	ZINC000085956013
1.0	-9.2	-8.1	0.61	1.324	0.214	3.329	0.686	6	915	ZINC000100780081
1.0	-8.2	-7.9	0.27	1.976	0.046	3.823	0.342	6	916	ZINC000015274345
1.0	-10.1	-9.1	0.47	2.007	0.253	3.668	1.377	6	917	ZINC000001857526
1.0	-11.0	-10.0	0.74	1.604	0.345	4.818	1.699	6	918	ZINC000100780573
1.0	-9.1	-8.3	0.49	1.358	0.264	3.602	0.506	6	919	ZINC000100775067
1.0	-10.0	-8.9	0.76	2.118	0.560	4.356	1.383	6	920	ZINC000253592129
1.0	-7.8	-7.5	0.23	1.435	0.121	3.196	0.511	6	921	ZINC000028541806
1.0	-7.2	-6.9	0.20	2.719	0.786	4.521	1.497	6	922	ZINC000050027395
1.0	-7.3	-6.9	0.35	1.643	0.872	3.360	0.622	6	923	ZINC000001651948

1.0	-8.9	-8.0	0.48	2.074	0.381	3.897	1.907	6	924	ZINC000014419492
1.0	-9.3	-8.8	0.43	1.989	0.497	4.748	1.327	6	925	ZINC000001668225
1.0	-8.3	-7.8	0.46	1.615	0.269	4.278	1.812	6	926	ZINC000013303571
1.0	-8.0	-7.8	0.15	2.036	0.296	2.845	0.648	6	927	ZINC000014722288
1.0	-7.5	-7.1	0.28	2.298	0.538	3.676	0.989	6	928	ZINC000014588972
1.0	-9.3	-8.2	0.49	1.892	0.174	3.455	1.150	6	929	ZINC000014758847
1.0	-8.9	-8.3	0.32	2.080	0.458	4.104	1.040	6	930	ZINC000014589843
1.0	-7.8	-7.4	0.20	2.020	0.300	5.965	0.534	6	931	ZINC000008860448
1.0	-8.3	-7.7	0.33	2.243	0.747	3.611	0.663	6	932	ZINC000030728463
1.0	-8.9	-8.0	0.50	2.383	1.031	3.999	1.365	6	933	ZINC000008215728
1.0	-7.8	-7.2	0.37	2.151	0.176	2.932	0.438	6	934	ZINC000006024696
1.0	-8.3	-7.8	0.25	1.912	0.456	4.629	1.065	6	935	ZINC000013462910
1.0	-8.3	-7.7	0.32	2.503	1.043	4.181	1.959	6	936	ZINC000100822508
1.0	-7.4	-6.9	0.31	2.917	0.266	5.118	0.357	6	937	ZINC000050027593
1.0	-7.4	-7.0	0.29	1.581	0.219	3.239	0.347	6	938	ZINC000003870305

1.0	-7.6	-7.3	0.26	2.145	0.338	3.213	0.441	6	939	ZINC000006037454
1.0	-9.6	-8.5	0.60	1.926	0.252	4.997	1.588	6	940	ZINC000005999176
1.0	-7.4	-7.2	0.17	2.528	0.477	3.645	0.808	6	941	ZINC000000409238
1.0	-10.5	-8.8	1.24	1.724	0.057	4.073	1.799	3	942	ZINC000001711822
1.0	-7.6	-7.3	0.15	2.038	0.321	4.606	2.001	6	943	ZINC000014610794
1.0	-10.5	-9.2	0.70	1.313	0.165	4.147	2.104	6	944	ZINC000014814435
1.0	-9.2	-8.1	0.60	1.295	0.198	3.541	0.313	6	945	ZINC000257399408
1.0	-11.2	-10.8	0.25	1.553	0.390	4.378	1.566	6	946	ZINC000257454024
1.0	-7.8	-7.3	0.30	2.148	0.476	3.912	1.643	6	947	ZINC000012495542
1.0	-9.0	-7.9	0.60	1.869	0.384	7.276	0.177	6	948	ZINC000034316958
1.0	-10.9	-9.9	0.75	1.867	0.462	3.846	1.122	6	949	ZINC000014715678
1.0	-7.5	-7.0	0.42	2.604	0.459	4.170	0.667	6	950	ZINC000000409239
1.0	-6.7	-6.6	0.15	1.734	0.450	3.633	1.008	6	951	ZINC000014448169
1.0	-9.4	-8.8	0.46	1.308	0.211	3.060	0.902	6	952	ZINC000004102407
1.0	-7.8	-7.4	0.23	1.990	0.584	3.403	1.226	6	953	ZINC000006066725

1.0	-10.6	-9.6	0.74	1.459	0.472	4.247	1.100	6	954	ZINC000100779646
1.0	-11.0	-10.1	0.45	1.597	0.378	4.837	1.690	6	955	ZINC000257530221
1.0	-9.7	-8.9	0.41	1.559	0.208	6.717	0.226	6	956	ZINC000003870338
1.0	-8.7	-8.2	0.31	1.552	0.256	3.450	1.302	6	957	ZINC000257397014
1.0	-9.9	-9.6	0.25	1.485	0.413	4.391	1.553	6	958	ZINC000000388657
1.0	-7.0	-6.7	0.18	1.886	0.325	3.685	0.643	6	959	ZINC000005440783
1.0	-9.9	-8.8	0.77	1.952	0.204	4.479	0.388	6	960	ZINC000230086078
1.0	-9.3	-8.5	0.36	1.808	0.176	3.882	0.382	6	961	ZINC000015220979
1.0	-8.1	-7.9	0.16	1.520	0.363	3.977	1.666	6	962	ZINC000012496423
1.0	-9.5	-8.8	0.71	1.527	0.565	3.883	2.261	6	963	ZINC000000119978
1.0	-10.3	-9.1	0.91	1.558	0.097	4.692	0.883	3	964	ZINC000014715660
1.0	-7.4	-7.0	0.22	2.181	0.855	4.032	0.558	6	965	ZINC000095620537
1.0	-6.7	-6.4	0.18	2.644	0.734	4.106	0.507	6	966	ZINC000006469367
1.0	-9.6	-8.9	0.36	1.584	0.237	4.499	1.908	6	967	ZINC000014684783
1.0	-9.2	-8.2	0.52	1.188	0.213	3.142	0.999	6	968	ZINC000257399409

1.0	-6.9	-6.6	0.20	1.498	0.742	3.593	0.697	6	969	ZINC000000156386
1.0	-8.5	-8.1	0.21	1.653	0.335	3.341	0.561	6	970	ZINC000013457482
1.0	-8.5	-7.7	0.46	1.660	0.329	4.546	0.257	6	971	ZINC000015147894
1.0	-9.0	-8.1	0.59	2.064	0.431	4.058	1.094	6	972	ZINC000086032441
1.0	-10.6	-9.9	0.60	1.292	0.343	3.350	1.318	6	973	ZINC000257446472
1.0	-8.9	-8.0	0.55	1.671	0.212	4.361	0.364	6	974	ZINC000100778048
1.0	-8.1	-7.8	0.19	1.631	0.484	3.646	1.145	6	975	ZINC000013481840
1.0	-10.0	-9.3	0.70	2.014	0.437	5.724	1.263	6	976	ZINC000005998597
1.0	-7.3	-7.2	0.12	2.596	0.592	4.731	1.167	6	977	ZINC000001850071
1.0	-9.7	-9.0	0.49	1.664	0.282	5.739	1.863	6	978	ZINC000100825624
1.0	-9.8	-8.2	0.82	1.855	0.095	4.526	1.868	6	979	ZINC000085923591
1.0	-8.0	-7.6	0.24	2.285	0.343	4.776	0.702	6	980	ZINC000014449823
1.0	-8.7	-7.8	0.43	2.599	0.199	5.028	0.443	6	981	ZINC000034286751
1.0	-8.5	-7.9	0.39	2.463	0.414	4.205	0.847	6	982	ZINC000014688565
1.0	-7.4	-7.2	0.15	1.901	0.377	3.523	0.309	6	983	ZINC000100780393

1.0	-6.5	-6.2	0.18	1.853	0.481	4.604	0.262	6	984	ZINC000078275009
1.0	-9.2	-8.3	0.41	2.087	0.598	4.011	0.669	6	985	ZINC000042685596
1.0	-10.6	-9.5	0.91	1.631	0.547	5.664	1.522	6	986	ZINC000013542978
1.0	-9.0	-8.3	0.34	1.546	0.385	3.668	0.863	6	987	ZINC000014453727
1.0	-7.9	-7.5	0.19	1.661	0.309	3.217	0.569	6	988	ZINC000015169316
1.0	-9.1	-8.2	0.50	2.294	0.321	4.371	0.834	6	989	ZINC000015058666
1.0	-10.9	-10.0	0.43	1.484	0.624	3.356	0.998	6	990	ZINC000104357499
1.0	-9.1	-8.4	0.55	1.907	0.331	3.871	1.465	6	991	ZINC000014765289
1.0	-9.2	-8.3	0.56	2.133	0.341	3.569	0.651	6	992	ZINC000013143016
1.0	-7.8	-7.1	0.36	2.350	0.253	4.137	1.000	6	993	ZINC000015062581
1.0	-8.2	-8.0	0.16	2.677	0.575	4.719	0.947	6	994	ZINC000035898716
1.0	-9.8	-8.8	1.37	1.465	0.109	4.065	2.246	3	995	ZINC000105249470
1.0	-9.7	-8.6	0.63	1.691	0.590	4.984	2.443	6	996	ZINC000014918974
1.0	-9.2	-8.0	0.73	1.716	0.146	3.012	1.177	6	997	ZINC000257437533
1.0	-8.1	-7.3	0.42	2.649	0.521	4.056	1.483	6	998	ZINC000012496701

1.0	-8.3	-7.8	0.29	2.134	0.425	2.763	0.736	6	999	ZINC000030731204
1.0	-10.1	-9.1	0.75	1.426	0.227	4.865	1.195	6	1000	ZINC000004082211
1.0	-8.5	-7.5	0.43	1.873	0.381	4.323	0.520	6	1001	ZINC000015147947
1.0	-9.2	-8.3	0.58	1.448	0.649	3.321	1.021	6	1002	ZINC000257399407
1.0	-8.3	-7.8	0.32	2.235	0.741	3.621	0.642	6	1003	ZINC000100029136
1.0	-9.7	-8.7	0.53	1.697	0.596	5.174	1.487	6	1004	ZINC000014778644
1.0	-10.5	-9.3	0.66	1.815	0.349	4.001	1.252	6	1005	ZINC000257437534
1.0	-10.9	-9.8	1.10	1.866	0.000	5.453	0.000	2	1006	ZINC000011612565
1.0	-9.8	-9.3	0.43	1.745	0.382	3.661	1.770	6	1007	ZINC000000895951
1.0	-8.6	-7.9	0.35	1.841	0.248	4.863	0.890	6	1008	ZINC000014507217
1.0	-7.4	-7.2	0.14	2.137	0.555	3.120	0.924	6	1009	ZINC000006037453
1.0	-5.9	-5.5	0.28	1.975	0.703	3.149	1.300	6	1010	ZINC000000391110
1.0	-9.7	-8.5	1.03	2.476	0.327	4.550	0.778	4	1011	ZINC000095619844
1.0	-8.2	-7.2	0.53	2.129	0.443	5.211	1.777	6	1012	ZINC000013303578
1.0	-6.8	-6.4	0.20	3.166	0.812	4.487	0.996	6	1013	ZINC000032167026

1.0	-7.9	-7.7	0.14	2.457	0.793	5.458	1.655	6	1014	ZINC000005999167
1.0	-8.3	-8.0	0.26	2.128	0.567	3.150	0.612	6	1015	ZINC000012410298
1.0	-9.3	-8.8	0.30	1.534	0.713	4.939	1.688	6	1016	ZINC000015249391
1.0	-10.1	-9.4	0.34	1.704	0.370	4.425	1.840	6	1017	ZINC000000895958
1.0	-9.5	-9.0	0.42	1.426	0.410	5.916	1.999	6	1018	ZINC000014643592
1.0	-10.6	-9.3	0.69	1.516	0.259	5.334	1.852	5	1019	ZINC000257525430
1.0	-9.5	-9.0	0.51	1.782	0.347	3.995	0.522	6	1020	ZINC000256600546
1.0	-8.2	-7.7	0.29	1.768	0.508	3.111	0.661	6	1021	ZINC000100779849
1.0	-6.1	-6.0	0.09	2.606	1.288	3.710	0.448	6	1022	ZINC000001850685
1.0	-7.1	-6.9	0.12	1.843	0.489	3.190	0.491	6	1023	ZINC000001850005
1.0	-8.7	-8.0	0.45	2.116	0.335	4.233	0.637	6	1024	ZINC000100824247
1.0	-8.2	-7.8	0.27	2.012	0.334	4.265	1.314	6	1025	ZINC000002563374
1.0	-6.7	-6.4	0.29	1.654	0.412	3.001	0.710	6	1026	ZINC000000388339
1.0	-9.2	-7.6	0.76	1.840	0.198	4.578	0.736	6	1027	ZINC000095619976
1.0	-6.8	-6.8	0.05	3.128	0.144	4.579	0.345	6	1028	ZINC000026675136

1.0	-9.0	-8.5	0.35	1.337	0.600	4.643	1.478	6	1029	ZINC000000899938
1.0	-9.0	-8.4	0.40	1.913	0.477	3.421	1.055	6	1030	ZINC000100782059
1.0	-7.1	-6.8	0.22	1.949	0.491	3.106	0.688	6	1031	ZINC000012494222
1.0	-9.2	-8.1	0.61	1.330	0.195	3.320	0.669	6	1032	ZINC000100780078
1.0	-10.3	-9.1	0.65	1.855	0.215	5.532	0.442	6	1033	ZINC000095618068
1.0	-7.6	-7.1	0.28	2.014	0.244	3.225	0.712	6	1034	ZINC000002390999
1.0	-10.4	-10.4	0.00	0.000	0.000	0.000	0.000	1	1035	ZINC000004025169
1.0	-10.5	-9.3	0.74	2.001	0.672	4.770	1.235	6	1036	ZINC000257437532
1.0	-12.1	-12.1	0.00	0.000	0.000	0.000	0.000	1	1037	ZINC000100828051
1.0	-8.6	-7.6	0.55	2.034	0.543	4.528	1.509	6	1038	ZINC000014593820
1.0	-7.3	-6.7	0.46	2.126	0.219	4.084	0.259	6	1039	ZINC000100778221
1.0	-10.5	-9.1	0.74	1.950	0.299	4.369	1.272	6	1040	ZINC000015055161
1.0	-7.3	-6.9	0.22	2.384	0.208	4.472	1.185	6	1041	ZINC000034173951
1.0	-8.1	-7.6	0.26	2.669	0.301	4.371	0.585	6	1042	ZINC000005761214
1.0	-10.3	-10.1	0.16	1.559	0.737	6.081	0.613	6	1043	ZINC000005854444

1.0	-7.8	-7.5	0.18	2.514	0.349	4.606	1.635	6	1044	ZINC000004321698
1.0	-8.7	-8.0	0.39	1.412	0.116	2.623	1.105	6	1045	ZINC000014854738
1.0	-9.3	-8.4	0.79	1.842	0.212	4.482	0.434	6	1046	ZINC000100780449
1.0	-8.7	-8.3	0.20	1.420	0.261	4.159	0.525	6	1047	ZINC000014413213
1.0	-10.6	-9.5	0.91	1.564	0.466	5.170	1.747	6	1048	ZINC000006500050
1.0	-7.3	-6.9	0.26	3.027	0.931	4.856	1.151	6	1049	ZINC000248191972
1.0	-7.7	-7.1	0.30	1.881	0.583	4.029	1.058	6	1050	ZINC000252370201
1.0	-8.0	-7.6	0.37	1.737	0.418	3.402	0.975	6	1051	ZINC000015209579
1.0	-9.4	-8.4	0.63	1.843	0.341	3.095	0.280	6	1052	ZINC000040892347
1.0	-6.4	-6.2	0.17	1.461	0.161	3.192	0.357	6	1053	ZINC000005132594
1.0	-7.1	-7.0	0.09	1.577	0.270	2.942	0.218	6	1054	ZINC000001710003
1.0	-10.6	-9.5	0.91	1.570	0.470	5.172	1.747	6	1055	ZINC000004023795
1.0	-9.5	-8.3	0.60	1.970	0.485	4.215	1.661	6	1056	ZINC000014761154
1.0	-10.6	-9.6	0.74	1.530	0.484	4.423	0.858	6	1057	ZINC000257446474
1.0	-11.0	-10.3	0.37	1.614	0.292	3.871	2.122	6	1058	ZINC000100780567

1.0	-6.3	-6.0	0.16	2.041	0.365	3.014	0.812	6	1059	ZINC000001530366
1.0	-10.9	-10.1	0.39	1.183	0.200	3.553	1.057	6	1060	ZINC000104357494
1.0	-9.5	-8.8	0.57	1.776	0.125	4.894	0.804	6	1061	ZINC000095617951
1.0	-7.6	-7.5	0.07	2.193	0.120	3.628	0.827	6	1062	ZINC000015261551
1.0	-10.0	-8.7	0.83	2.506	0.348	4.473	0.372	4	1063	ZINC000015155026
1.0	-9.0	-7.9	0.62	1.747	0.387	3.507	1.169	6	1064	ZINC000100778052
1.0	-9.1	-8.7	0.24	1.660	0.293	2.961	0.445	6	1065	ZINC000257639977
1.0	-6.1	-5.9	0.21	2.429	1.177	3.508	1.256	6	1066	ZINC000032167536
1.0	-10.5	-8.9	0.85	1.785	0.406	5.319	1.822	6	1067	ZINC000100779895
1.0	-8.5	-8.2	0.22	1.727	0.327	4.308	1.575	6	1068	ZINC000015249388
1.0	-9.3	-8.6	0.35	1.878	0.733	4.157	2.196	6	1069	ZINC000014677567
1.0	-6.8	-6.7	0.09	1.514	0.897	3.006	0.971	6	1070	ZINC000113510027
1.0	-8.3	-7.8	0.25	2.535	0.312	5.724	1.023	6	1071	ZINC000085661247
1.0	-9.2	-8.3	0.48	1.834	0.615	6.182	1.129	6	1072	ZINC000014811594
1.0	-8.7	-8.4	0.29	1.804	0.246	3.690	0.643	6	1073	ZINC000100781166

1.0	-9.9	-8.9	0.64	1.828	0.314	4.671	0.527	6	1074	ZINC000100780113
1.0	-6.6	-6.3	0.19	1.931	0.661	3.000	1.089	6	1075	ZINC000005191814
1.0	-8.6	-7.3	0.95	2.041	0.583	4.223	1.254	6	1076	ZINC000014411637
1.0	-6.1	-6.0	0.09	1.219	0.309	3.508	0.543	6	1077	ZINC000100778613
1.0	-8.5	-8.2	0.26	1.438	0.245	2.892	0.778	6	1078	ZINC000257387898
1.0	-8.8	-8.1	0.50	2.589	0.212	6.488	0.407	6	1079	ZINC000040470653
1.0	-8.1	-7.7	0.28	1.501	0.212	3.807	0.400	6	1080	ZINC000015214525
1.0	-10.4	-9.1	0.74	1.741	0.220	4.670	1.191	6	1081	ZINC000000402695
1.0	-8.9	-8.3	0.38	2.263	0.662	3.636	1.416	6	1082	ZINC000032839149
1.0	-8.9	-8.0	0.49	1.950	0.283	3.153	0.796	6	1083	ZINC000013350932
1.0	-8.9	-8.3	0.30	1.458	0.313	3.620	1.629	6	1084	ZINC000230084385
1.0	-8.6	-8.0	0.41	2.432	0.494	4.777	1.387	6	1085	ZINC000014444133
1.0	-7.6	-7.1	0.32	2.002	0.457	3.717	0.945	6	1086	ZINC000000163858
1.0	-7.6	-6.9	0.35	2.207	0.157	4.335	1.258	6	1087	ZINC000003869692
1.0	-11.0	-10.1	0.53	1.889	0.355	4.915	1.739	6	1088	ZINC000257530222

1.0	-9.2	-8.5	0.46	2.645	0.711	4.088	1.592	6	1089	ZINC000001637998
1.0	-8.1	-7.5	0.34	1.622	0.172	3.528	0.672	6	1090	ZINC000197874988
1.0	-7.0	-6.8	0.13	1.502	0.303	2.873	0.292	6	1091	ZINC000002572145
1.0	-10.5	-9.3	0.63	1.310	0.077	3.703	1.424	6	1092	ZINC000014857746
1.0	-10.4	-9.7	0.56	1.338	0.343	4.098	1.291	6	1093	ZINC000086028139
1.0	-8.2	-7.8	0.34	1.411	0.207	3.530	0.419	6	1094	ZINC000038634841
1.0	-7.4	-7.1	0.15	2.028	0.549	2.911	0.521	6	1095	ZINC000014613371
1.0	-7.2	-6.5	0.37	1.899	0.181	3.927	1.152	6	1096	ZINC000095619718
1.0	-10.7	-9.1	1.02	1.578	0.207	3.691	1.668	5	1097	ZINC000005764527
1.0	-8.7	-8.2	0.27	1.908	0.360	3.006	0.764	6	1098	ZINC000015261559
1.0	-6.6	-6.3	0.19	2.180	0.995	3.429	1.066	6	1099	ZINC000001850881
1.0	-8.3	-8.0	0.22	1.849	0.549	3.914	0.365	6	1100	ZINC000005358268
1.0	-7.7	-7.2	0.31	1.727	0.259	4.281	0.598	6	1101	ZINC000014684362
1.0	-8.7	-8.2	0.36	1.629	0.152	4.924	1.084	6	1102	ZINC000015249400
1.0	-9.8	-9.0	0.54	2.163	0.423	4.921	1.460	6	1103	ZINC000014811617

1.0	-8.3	-7.8	0.42	2.700	0.296	5.771	1.661	6	1104	ZINC000027211157
1.0	-10.8	-9.9	0.45	1.762	0.568	4.420	2.084	6	1105	ZINC000100780631
1.0	-8.6	-7.5	0.76	2.798	0.626	4.930	1.164	6	1106	ZINC000012496134
1.0	-9.6	-8.8	0.40	1.920	0.199	4.122	1.617	6	1107	ZINC000257515695
1.0	-7.9	-7.5	0.25	1.900	0.490	4.398	1.236	6	1108	ZINC000095617634
1.0	-10.3	-9.5	0.57	2.204	0.263	5.841	1.354	6	1109	ZINC000003872070
1.0	-10.5	-9.2	0.97	1.487	0.448	4.505	1.741	6	1110	ZINC000003881977
1.0	-9.4	-8.7	0.46	1.961	0.315	3.967	0.848	6	1111	ZINC000257379324
1.0	-8.4	-8.0	0.26	1.548	0.210	3.683	1.009	6	1112	ZINC000015147899
1.0	-8.5	-8.0	0.29	2.164	0.666	3.985	1.026	6	1113	ZINC000100781841
1.0	-9.5	-8.3	0.79	1.801	0.402	3.821	1.264	6	1114	ZINC000030726889
1.0	-8.6	-8.1	0.28	1.204	0.098	3.528	0.303	6	1115	ZINC000095914189
1.0	-6.6	-6.4	0.15	1.720	0.357	4.504	1.928	6	1116	ZINC000033650000
1.0	-10.6	-9.5	0.89	1.635	0.541	5.674	1.523	6	1117	ZINC000008551557
1.0	-9.0	-8.6	0.24	2.041	0.895	3.397	0.827	6	1118	ZINC000085845251

1.0	-9.2	-9.0	0.24	1.475	0.190	4.130	1.674	6	1119	ZINC000005352274
1.0	-9.0	-8.0	0.52	1.568	0.451	3.151	0.875	6	1120	ZINC000059200502
1.0	-8.3	-7.7	0.40	1.721	0.161	3.267	0.717	6	1121	ZINC000015209689
1.0	-8.1	-7.9	0.21	1.630	0.322	3.542	0.344	6	1122	ZINC000255997553
1.0	-8.4	-7.9	0.31	1.418	0.287	3.980	0.219	6	1123	ZINC000100782233
1.0	-9.9	-8.8	0.63	2.427	0.515	4.814	1.526	6	1124	ZINC000000968076
1.0	-7.7	-7.4	0.21	2.301	0.105	4.269	0.328	6	1125	ZINC000005761204
1.0	-6.9	-6.6	0.17	1.339	0.245	2.802	0.384	6	1126	ZINC000000967571
1.0	-9.2	-8.6	0.41	1.781	0.576	5.141	2.006	6	1127	ZINC000014811582
1.0	-9.7	-9.0	0.47	1.955	0.521	3.192	1.039	6	1128	ZINC000013829478
1.0	-9.3	-8.7	0.30	1.710	0.432	4.161	2.150	6	1129	ZINC000014645949
1.0	-10.9	-10.1	0.38	1.186	0.204	3.556	1.062	6	1130	ZINC000100775955
1.0	-8.5	-8.2	0.38	1.678	0.246	3.950	0.402	6	1131	ZINC000059065197
1.0	-8.3	-7.8	0.41	2.395	0.260	5.102	0.265	6	1132	ZINC000004534013
1.0	-6.8	-6.2	0.28	1.962	0.709	3.499	1.133	6	1133	ZINC000014438705

1.0	-9.1	-8.1	0.67	1.680	0.577	3.986	0.433	6	1134	ZINC000257579930
1.0	-7.7	-7.2	0.23	1.744	0.505	2.305	0.671	6	1135	ZINC000005933618
1.0	-7.6	-7.0	0.29	3.366	0.345	5.189	0.935	6	1136	ZINC000002018980
1.0	-10.4	-9.8	0.46	1.309	0.370	3.845	1.208	6	1137	ZINC000100779667
1.0	-6.5	-6.1	0.18	1.929	0.363	3.771	0.996	6	1138	ZINC000085972378
1.0	-10.2	-8.5	1.24	2.268	0.216	5.057	0.309	3	1139	ZINC000014715684
1.0	-8.3	-7.7	0.38	2.008	0.269	3.810	0.353	6	1140	ZINC000033950766
1.0	-8.4	-8.2	0.19	1.342	0.119	3.725	0.616	6	1141	ZINC000100784267
1.0	-7.8	-7.5	0.16	2.002	0.545	3.594	1.319	6	1142	ZINC000034018756
1.0	-8.1	-7.8	0.37	1.665	0.080	5.548	1.165	6	1143	ZINC000013350895
1.0	-7.3	-6.9	0.30	1.961	0.382	3.880	2.117	6	1144	ZINC000261499749
1.0	-10.6	-9.3	0.67	1.425	0.253	5.553	1.742	6	1145	ZINC000257525431
1.0	-8.2	-7.5	0.34	1.382	0.117	3.572	0.989	6	1146	ZINC000086032739
1.0	-10.6	-9.5	0.91	1.569	0.472	5.164	1.744	6	1147	ZINC000004023794
1.0	-7.8	-7.2	0.38	2.998	1.039	4.929	1.788	6	1148	ZINC000004096329

1.0	-10.1	-8.9	0.91	1.598	0.418	3.086	1.677	6	1149	ZINC000014723711
1.0	-8.1	-7.5	0.37	2.552	0.479	4.469	0.999	6	1150	ZINC000050027582
1.0	-8.8	-8.4	0.22	1.560	0.623	3.537	1.833	6	1151	ZINC000100828806
1.0	-8.1	-7.4	0.49	1.712	0.348	3.249	0.964	6	1152	ZINC000038613726
1.0	-8.3	-7.8	0.26	1.939	0.261	4.088	1.165	6	1153	ZINC000005767277
1.0	-8.7	-8.2	0.30	1.270	0.041	3.395	0.502	6	1154	ZINC000015160907
1.0	-10.9	-10.0	0.45	1.220	0.206	3.265	1.020	6	1155	ZINC000100775949
1.0	-8.3	-8.1	0.12	1.571	0.252	5.419	1.671	6	1156	ZINC000015249382
1.0	-9.1	-8.1	0.51	2.372	0.683	4.059	0.963	6	1157	ZINC000013783211
1.0	-7.1	-6.5	0.34	2.040	0.303	3.362	0.399	6	1158	ZINC000059587954
1.0	-8.3	-7.9	0.63	2.707	0.161	6.347	1.382	6	1159	ZINC000014886643
1.0	-6.5	-6.2	0.21	1.701	0.292	3.187	0.669	6	1160	ZINC000001626974
1.0	-7.4	-7.1	0.24	2.207	0.483	3.662	0.519	6	1161	ZINC000013380489
1.0	-9.2	-8.1	0.64	1.574	0.585	3.668	0.370	6	1162	ZINC000230085967
1.0	-7.2	-7.1	0.12	1.758	0.365	2.911	0.532	6	1163	ZINC000004098346

1.0	-8.2	-7.9	0.22	1.727	0.182	3.923	1.642	6	1164	ZINC000257420301
1.0	-8.8	-8.4	0.35	1.716	0.759	3.911	1.454	6	1165	ZINC000012353732
1.0	-7.7	-7.2	0.24	1.921	0.403	2.361	0.584	6	1166	ZINC000005933622
1.0	-10.9	-9.7	0.94	1.854	0.316	4.168	1.275	6	1167	ZINC000014715676
1.0	-9.1	-8.3	0.46	1.348	0.289	3.669	0.473	6	1168	ZINC000257579928
1.0	-9.9	-8.3	0.98	1.358	0.180	4.184	1.828	5	1169	ZINC000003814360
1.0	-7.6	-7.0	0.32	1.924	0.441	3.871	0.581	6	1170	ZINC000095619540
1.0	-6.5	-6.3	0.07	1.436	0.137	2.992	0.458	6	1171	ZINC000006031128
1.0	-7.4	-7.1	0.22	2.040	0.418	5.483	0.691	6	1172	ZINC000004544317
1.0	-7.9	-7.6	0.22	1.957	0.265	4.092	0.683	6	1173	ZINC000002572266
1.0	-9.2	-8.5	0.40	1.575	0.398	5.120	1.736	6	1174	ZINC000257452998
1.0	-7.7	-7.4	0.18	1.978	0.815	4.213	1.874	6	1175	ZINC000034403408
1.0	-10.6	-9.5	0.91	1.564	0.467	5.166	1.745	6	1176	ZINC000004175377
1.0	-8.3	-7.6	0.33	1.809	0.200	3.997	0.601	6	1177	ZINC000014442513
1.0	-9.5	-8.7	0.49	2.295	0.373	4.327	0.987	6	1178	ZINC000086036325

1.0	-8.7	-8.0	0.72	1.927	0.540	4.073	1.828	6	1179	ZINC000001704485
1.0	-8.1	-7.5	0.29	2.392	0.348	4.328	0.800	6	1180	ZINC000013460044
1.0	-6.9	-6.4	0.25	2.871	0.824	4.301	0.861	6	1181	ZINC000032174383
1.0	-8.0	-7.6	0.32	1.257	0.662	2.164	0.613	6	1182	ZINC000100825046
1.0	-7.6	-7.4	0.11	1.648	0.215	4.857	1.989	6	1183	ZINC000014860254
1.0	-8.4	-8.0	0.21	1.794	0.197	3.679	0.355	6	1184	ZINC000033950659
1.0	-6.8	-6.5	0.25	1.580	0.886	3.381	0.518	6	1185	ZINC000000967332
0.9	-8.4	-7.9	0.32	1.918	0.531	3.188	1.248	6	1186	ZINC000033953589
0.9	-9.3	-8.6	0.38	1.856	0.550	4.394	2.286	6	1187	ZINC000013374804
0.9	-7.5	-7.1	0.24	2.661	0.567	4.721	1.118	6	1188	ZINC000168751887
0.9	-8.1	-7.7	0.28	1.660	0.518	3.236	0.973	6	1189	ZINC000013377497
0.9	-5.6	-5.3	0.13	1.606	0.485	2.475	0.366	6	1190	ZINC000001850920
0.9	-7.5	-7.3	0.09	1.951	0.299	3.931	1.144	6	1191	ZINC000015219235
0.9	-8.1	-7.6	0.35	1.749	0.686	3.454	1.810	6	1192	ZINC000015115327
0.9	-8.6	-7.5	0.64	1.590	0.568	3.936	1.506	6	1193	ZINC000015115271

0.9	-8.7	-8.3	0.30	2.002	0.404	4.041	0.474	6	1194	ZINC000006072098
0.9	-9.0	-8.3	0.59	2.067	0.376	4.264	0.524	6	1195	ZINC000015056377
0.9	-6.6	-6.2	0.24	2.253	0.278	3.260	0.683	6	1196	ZINC000031484864
0.9	-10.5	-9.5	0.81	1.433	0.399	5.059	1.869	6	1197	ZINC000004467880
0.9	-9.9	-8.9	0.48	1.421	0.243	4.538	1.086	6	1198	ZINC000013334424
0.9	-6.8	-6.6	0.15	1.664	0.679	3.829	1.385	6	1199	ZINC000004556536
0.9	-9.4	-8.3	0.51	1.667	0.357	4.212	1.432	6	1200	ZINC000257401483
0.9	-9.4	-9.0	0.31	1.438	0.378	3.836	2.344	6	1201	ZINC000014677565
0.9	-6.4	-5.8	0.31	2.109	0.400	3.063	0.697	6	1202	ZINC000002001177
0.9	-8.6	-8.3	0.16	1.645	0.163	4.139	0.994	6	1203	ZINC000014452647
0.9	-5.9	-5.7	0.15	2.438	0.377	3.951	0.748	6	1204	ZINC000003644164
0.9	-9.5	-8.6	0.47	1.430	0.252	4.729	1.261	6	1205	ZINC000013486217
0.9	-8.4	-7.5	0.47	1.881	0.561	3.745	1.935	6	1206	ZINC000015115269
0.9	-7.5	-7.4	0.07	1.795	0.506	3.622	1.532	6	1207	ZINC000257554912
0.9	-5.8	-5.5	0.17	1.710	0.257	2.943	0.523	6	1208	ZINC000095620504

0.9	-9.4	-9.1	0.31	2.132	0.586	3.850	1.603	6	1209	ZINC000000056472
0.9	-10.2	-8.6	0.80	2.111	0.248	3.917	0.625	6	1210	ZINC000028109109
0.9	-8.2	-7.7	0.31	1.970	0.701	3.284	0.466	6	1211	ZINC000000173360
0.9	-10.5	-9.8	0.66	1.445	0.445	5.601	1.695	5	1212	ZINC000003814411
0.9	-7.9	-7.3	0.28	2.561	0.600	4.903	0.970	6	1213	ZINC000015122071
0.9	-7.9	-7.4	0.32	1.435	0.205	3.872	1.402	6	1214	ZINC000100776455
0.9	-8.2	-7.9	0.17	2.393	0.232	5.591	0.923	6	1215	ZINC000014886652
0.9	-6.7	-6.3	0.24	1.770	0.756	2.609	1.311	6	1216	ZINC000000968037
0.9	-9.2	-8.8	0.34	2.142	0.316	3.974	0.996	6	1217	ZINC000212580656
0.9	-9.1	-8.7	0.27	1.950	0.163	4.712	1.760	6	1218	ZINC000001026053
0.9	-9.1	-8.4	0.35	1.550	0.246	3.498	0.702	6	1219	ZINC000100781169
0.9	-7.1	-6.4	0.34	2.365	0.412	3.518	0.444	6	1220	ZINC000100880082
0.9	-8.3	-8.1	0.15	1.588	0.150	3.528	0.247	6	1221	ZINC000085880168
0.9	-9.0	-8.5	0.27	2.076	0.683	4.170	1.902	6	1222	ZINC000000043479
0.9	-8.0	-7.8	0.20	1.259	0.159	2.596	0.777	6	1223	ZINC000001850501

0.9	-6.5	-6.1	0.21	1.488	0.220	3.984	0.831	6	1224	ZINC000085972335
0.9	-8.8	-8.4	0.33	2.367	0.645	3.647	1.523	6	1225	ZINC000002384992
0.9	-8.5	-8.0	0.35	2.534	0.649	4.377	1.285	6	1226	ZINC000015657741
0.9	-9.1	-8.3	0.55	1.714	0.224	3.575	1.135	6	1227	ZINC000261499502
0.9	-6.0	-5.6	0.17	2.555	1.367	4.305	1.323	6	1228	ZINC000001850466
0.9	-6.5	-6.2	0.21	1.889	1.012	3.066	0.595	6	1229	ZINC000000403494
0.9	-7.1	-6.9	0.15	1.649	0.824	4.256	1.832	6	1230	ZINC000000153660
0.9	-7.8	-7.4	0.23	1.537	0.573	2.743	0.484	6	1231	ZINC000100778988
0.9	-5.7	-5.2	0.23	1.677	0.223	2.547	0.369	6	1232	ZINC000100829306
0.9	-8.6	-8.2	0.24	1.791	0.291	4.152	2.341	6	1233	ZINC000006505385
0.9	-9.7	-8.8	0.65	1.680	0.277	3.719	1.794	6	1234	ZINC000013382475
0.9	-8.7	-8.3	0.32	1.633	0.577	4.408	1.533	6	1235	ZINC000150373202
0.9	-8.3	-7.8	0.32	1.478	0.607	2.831	0.330	6	1236	ZINC000100781102
0.9	-10.5	-9.5	0.97	1.600	0.491	4.829	1.839	5	1237	ZINC000013298301
0.9	-7.7	-7.3	0.20	1.988	0.370	3.685	0.924	6	1238	ZINC000001685819

0.9	-9.1	-8.6	0.40	1.709	0.131	3.608	0.228	6	1239	ZINC000015160851
0.9	-8.5	-8.2	0.24	2.126	0.389	4.145	0.368	6	1240	ZINC000085883131
0.9	-7.1	-6.8	0.20	1.524	0.359	3.336	0.359	6	1241	ZINC000033830723
0.9	-8.8	-7.8	0.46	2.695	0.466	4.470	1.078	6	1242	ZINC000013377649
0.9	-9.7	-9.0	0.56	1.696	0.480	4.381	1.913	6	1243	ZINC000014728220
0.9	-7.4	-6.6	0.40	2.559	0.495	3.579	0.298	6	1244	ZINC000004532783
0.9	-9.0	-8.6	0.24	1.837	0.991	3.309	0.844	6	1245	ZINC000100783142
0.9	-7.9	-7.6	0.19	1.257	0.172	2.854	1.302	6	1246	ZINC000100776283
0.9	-8.3	-8.0	0.15	1.254	0.075	2.902	0.966	6	1247	ZINC000083260318
0.9	-7.3	-7.2	0.09	1.955	0.522	4.106	1.468	6	1248	ZINC000004655401
0.9	-8.0	-7.2	0.45	1.730	0.624	3.567	0.840	6	1249	ZINC000001705987
0.9	-8.5	-8.3	0.18	1.789	0.296	4.375	1.388	6	1250	ZINC000004654967
0.9	-7.1	-6.5	0.31	2.369	0.392	3.495	0.406	6	1251	ZINC000100638520
0.9	-7.6	-7.1	0.24	1.574	0.338	4.948	1.394	6	1252	ZINC000001850796
0.9	-7.5	-7.1	0.19	2.317	0.258	2.960	0.278	6	1253	ZINC000001849753

0.9	-7.7	-7.5	0.15	1.816	0.508	4.317	1.554	6	1254	ZINC000015219346
0.9	-7.8	-7.3	0.28	2.022	0.686	4.439	1.268	6	1255	ZINC000011568127
0.9	-8.5	-8.1	0.22	1.824	0.401	3.480	1.259	6	1256	ZINC000015201058
0.9	-9.3	-8.5	0.42	1.841	1.032	3.337	0.830	6	1257	ZINC000100826956
0.9	-6.3	-5.8	0.31	2.537	0.901	3.420	0.900	6	1258	ZINC000001081105
0.9	-10.7	-10.0	0.61	1.391	0.685	3.638	1.022	6	1259	ZINC000100779679
0.9	-8.3	-7.8	0.28	1.188	0.396	2.428	0.514	6	1260	ZINC000100781105
0.9	-8.0	-7.2	0.40	3.149	0.541	4.961	0.729	6	1261	ZINC000004081371
0.9	-7.8	-7.3	0.26	2.364	0.420	4.391	1.916	6	1262	ZINC000001529646
0.9	-6.6	-6.1	0.28	2.586	0.766	3.992	0.959	6	1263	ZINC000012495942
0.9	-7.7	-7.4	0.19	2.288	0.397	3.563	1.009	6	1264	ZINC000006069019
0.9	-8.2	-7.9	0.27	2.469	0.553	4.162	1.093	6	1265	ZINC000100783456
0.9	-7.0	-6.6	0.26	2.187	0.516	4.218	1.166	6	1266	ZINC000002558114
0.9	-6.5	-6.2	0.13	2.301	0.233	3.797	0.940	6	1267	ZINC000004283567
0.9	-9.0	-8.7	0.32	1.432	0.250	3.432	1.754	6	1268	ZINC000013382388

0.9	-8.1	-7.4	0.52	1.499	0.673	2.936	1.323	6	1269	ZINC000001622033
0.9	-7.8	-7.2	0.30	1.698	0.127	4.458	1.730	6	1270	ZINC000014680245
0.9	-9.6	-9.0	0.41	1.762	0.254	3.135	0.761	6	1271	ZINC000014919096
0.9	-9.4	-8.2	0.67	1.652	0.257	5.316	2.061	6	1272	ZINC000001663391
0.9	-8.1	-7.5	0.31	2.249	0.327	3.976	0.620	6	1273	ZINC000005934232
0.9	-7.3	-6.8	0.33	1.930	0.393	3.791	1.315	6	1274	ZINC000014856839
0.9	-9.4	-9.1	0.21	2.020	0.238	3.880	0.812	6	1275	ZINC000015054827
0.9	-8.0	-7.6	0.24	1.867	0.448	2.669	0.743	6	1276	ZINC000001569529
0.9	-6.8	-6.6	0.13	2.038	0.441	3.260	1.019	6	1277	ZINC000001529264
0.9	-10.7	-10.0	0.48	1.506	0.470	4.384	0.700	6	1278	ZINC000014516843
0.9	-7.6	-7.2	0.23	1.856	0.467	4.462	0.709	6	1279	ZINC000095619683
0.9	-5.6	-5.3	0.16	1.460	0.150	3.008	0.603	6	1280	ZINC000002040188
0.9	-10.7	-10.1	0.61	1.647	1.059	4.142	0.979	6	1281	ZINC000257430475
0.9	-8.7	-8.4	0.16	2.276	0.555	3.844	0.281	6	1282	ZINC000006069908
0.9	-7.0	-6.8	0.17	1.826	0.552	2.984	0.949	6	1283	ZINC000012494217

0.9	-7.5	-7.3	0.12	2.385	0.220	3.019	0.291	6	1284	ZINC000005967123
0.9	-9.1	-8.9	0.16	0.794	0.121	2.547	0.252	6	1285	ZINC000100823446
0.9	-10.5	-9.7	0.59	1.850	0.689	2.936	1.509	4	1286	ZINC000044020714
0.9	-7.9	-7.5	0.23	1.881	0.308	4.018	0.722	6	1287	ZINC000013432658
0.9	-10.1	-9.3	0.40	2.035	0.535	3.958	0.502	6	1288	ZINC000013482849
0.9	-7.6	-7.1	0.28	2.470	0.821	3.505	0.435	6	1289	ZINC000100823364
0.9	-7.6	-7.1	0.29	2.324	0.650	3.706	0.716	6	1290	ZINC000095619239
0.9	-8.3	-8.0	0.24	1.500	0.192	3.570	0.622	6	1291	ZINC000100780399
0.9	-6.2	-5.9	0.18	1.605	0.289	2.809	0.628	6	1292	ZINC000001529846
0.9	-6.5	-6.2	0.20	2.372	0.477	3.759	0.566	6	1293	ZINC000014588937
0.9	-9.3	-9.0	0.22	1.401	0.485	3.168	1.697	6	1294	ZINC000014918972
0.9	-8.5	-8.2	0.21	1.649	0.375	4.117	0.439	6	1295	ZINC000005751120
0.9	-9.7	-8.5	0.98	2.152	0.456	4.346	1.525	6	1296	ZINC000008681590
0.9	-8.9	-8.4	0.36	1.404	0.647	3.587	1.672	6	1297	ZINC000015249546
0.9	-6.9	-6.8	0.11	2.587	0.423	4.888	0.472	6	1298	ZINC000014502358

0.9	-8.2	-7.9	0.30	2.710	0.510	4.740	0.851	6	1299	ZINC000095626050
0.9	-9.7	-9.2	0.43	1.256	0.219	4.222	0.383	6	1300	ZINC000100199161
0.9	-9.7	-8.3	0.69	1.538	0.127	4.596	2.056	6	1301	ZINC000040164529
0.9	-9.1	-9.0	0.11	0.823	0.137	2.764	0.411	6	1302	ZINC000257563234
0.9	-6.8	-6.2	0.31	1.798	0.332	3.302	1.304	6	1303	ZINC000000157405
0.9	-7.0	-6.6	0.19	2.121	0.456	3.382	1.250	6	1304	ZINC000000895840
0.9	-6.0	-5.7	0.20	2.317	0.905	2.974	1.140	6	1305	ZINC000032163564
0.9	-5.5	-5.4	0.19	2.143	1.344	4.190	0.472	6	1306	ZINC000003875920
0.9	-8.9	-8.6	0.29	1.534	0.782	3.309	1.290	6	1307	ZINC000013340290
0.9	-10.4	-8.6	0.84	2.405	0.414	4.808	0.653	6	1308	ZINC000006018921
0.9	-7.6	-7.2	0.25	1.581	0.140	3.203	0.784	6	1309	ZINC000014588968
0.9	-6.9	-6.8	0.17	2.156	0.534	3.720	1.597	6	1310	ZINC000006425539
0.9	-7.8	-7.4	0.27	1.877	0.489	5.225	0.938	6	1311	ZINC000001557677
0.9	-10.4	-8.9	1.07	1.978	0.082	7.357	0.464	3	1312	ZINC000038940469
0.9	-9.2	-8.5	0.47	2.292	1.045	4.170	1.554	6	1313	ZINC000001532216

0.9	-8.9	-8.3	0.35	2.066	0.755	3.623	2.081	6	1314	ZINC000014594783
0.9	-9.1	-9.0	0.11	0.827	0.139	2.769	0.400	6	1315	ZINC000100823444
0.9	-10.0	-9.0	0.62	1.529	0.220	5.581	1.363	6	1316	ZINC000034275400
0.9	-8.3	-8.0	0.17	1.507	0.394	3.728	0.354	6	1317	ZINC000085883124
0.9	-7.4	-7.0	0.18	2.135	0.470	3.657	1.145	6	1318	ZINC000015261409
0.9	-7.5	-7.3	0.15	2.103	0.662	3.251	1.044	6	1319	ZINC000257550527
0.9	-7.0	-6.6	0.30	2.522	0.939	3.731	0.796	6	1320	ZINC000000166877
0.9	-7.7	-7.5	0.19	1.700	0.505	4.171	1.461	6	1321	ZINC000003809850
0.9	-9.9	-9.2	0.38	2.277	0.286	4.334	0.451	6	1322	ZINC000015203708
0.9	-7.8	-7.5	0.19	1.678	0.602	3.053	0.682	6	1323	ZINC000013521393
0.9	-6.9	-6.3	0.29	2.837	0.815	4.059	1.041	6	1324	ZINC000095618106
0.9	-8.5	-8.0	0.27	1.539	0.122	3.591	0.638	6	1325	ZINC000100825789
0.9	-6.4	-5.8	0.31	2.184	0.688	2.931	0.605	6	1326	ZINC000015045392
0.9	-9.5	-8.6	0.47	1.429	0.247	4.732	1.261	6	1327	ZINC000100829337
0.9	-7.5	-7.2	0.17	2.719	0.682	4.755	1.166	6	1328	ZINC000033839283

0.9	-8.8	-8.2	0.33	2.051	0.605	3.324	0.866	6	1329	ZINC000013408254
0.9	-6.6	-6.5	0.05	1.142	0.068	2.597	0.505	6	1330	ZINC000015169330
0.9	-7.5	-7.2	0.15	1.998	0.358	3.325	0.904	6	1331	ZINC000014651629
0.9	-8.3	-7.6	0.42	1.444	0.258	3.261	0.898	6	1332	ZINC000230086290
0.9	-8.2	-7.9	0.20	2.037	0.272	4.414	1.030	6	1333	ZINC000027638889
0.9	-7.2	-7.0	0.13	1.698	0.451	3.636	1.355	6	1334	ZINC000001851038
0.9	-7.7	-7.4	0.22	1.693	0.366	4.074	1.299	6	1335	ZINC000014655148
0.9	-9.4	-8.5	0.44	2.214	0.462	4.977	1.143	6	1336	ZINC000001721693
0.9	-8.9	-8.6	0.32	1.804	0.680	3.973	2.248	6	1337	ZINC000013334896
0.9	-8.9	-8.5	0.27	1.547	0.758	3.899	2.122	6	1338	ZINC000014505480
0.9	-10.6	-9.7	0.72	1.451	0.360	4.375	0.902	6	1339	ZINC000100779651
0.9	-6.4	-6.1	0.21	2.058	0.528	3.278	0.737	6	1340	ZINC000005441043
0.9	-6.2	-5.8	0.20	2.190	0.413	2.713	0.405	6	1341	ZINC000100828265
0.9	-10.5	-9.5	0.89	1.631	0.546	5.672	1.522	6	1342	ZINC000034051634
0.9	-10.9	-9.2	1.06	2.036	0.353	3.960	1.388	4	1343	ZINC000014686333

0.9	-7.9	-7.5	0.19	2.073	0.563	5.170	0.897	6	1344	ZINC000012496298
0.9	-6.8	-6.5	0.27	1.563	0.435	3.406	0.260	6	1345	ZINC000001699439
0.9	-7.5	-6.9	0.33	2.098	0.214	5.604	0.647	6	1346	ZINC000014494695
0.9	-7.0	-6.6	0.22	2.299	1.001	4.531	0.955	6	1347	ZINC000000056550
0.9	-6.5	-6.1	0.22	2.153	0.204	3.365	0.440	6	1348	ZINC000002032473
0.9	-8.9	-8.6	0.41	1.871	0.315	3.217	1.046	6	1349	ZINC000015249563
0.9	-7.8	-7.5	0.21	2.131	0.193	4.758	0.971	6	1350	ZINC000013356177
0.9	-7.5	-7.2	0.17	2.743	0.717	4.823	1.130	6	1351	ZINC000100059038
0.9	-6.3	-6.0	0.21	2.049	0.173	3.969	0.190	6	1352	ZINC000005922165
0.9	-10.4	-9.1	1.14	1.181	0.086	4.875	1.637	4	1353	ZINC000004343508
0.9	-8.8	-8.4	0.27	2.183	0.423	3.828	1.215	6	1354	ZINC000040471659
0.9	-8.7	-8.2	0.40	1.798	0.383	4.228	0.632	6	1355	ZINC000100782234
0.9	-6.0	-5.6	0.20	1.661	0.188	2.558	0.466	6	1356	ZINC000095618214
0.9	-7.1	-6.5	0.31	2.305	0.284	3.431	0.306	6	1357	ZINC000005742757
0.9	-8.9	-8.7	0.23	2.070	0.432	3.782	0.477	6	1358	ZINC000001648536

0.9	-10.7	-10.7	0.00	0.000	0.000	0.000	0.000	1	1359	ZINC000014688366
0.9	-8.2	-7.8	0.32	1.913	0.310	3.564	0.479	6	1360	ZINC000015169240
0.9	-7.8	-7.5	0.19	1.707	0.592	3.073	0.670	6	1361	ZINC000100778989
0.9	-8.7	-7.8	0.64	1.722	0.194	4.450	1.514	6	1362	ZINC000012496564
0.9	-7.2	-6.7	0.23	2.823	1.119	3.946	1.949	6	1363	ZINC000095618103
0.9	-8.0	-7.6	0.25	2.233	0.718	3.810	1.114	6	1364	ZINC000014488862
0.9	-7.2	-6.9	0.16	1.718	0.363	3.348	0.771	6	1365	ZINC000001850051
0.9	-8.6	-7.8	0.42	1.695	0.113	3.530	0.967	6	1366	ZINC000014516056
0.9	-7.7	-7.4	0.21	1.377	0.759	2.179	0.579	6	1367	ZINC000005851109
0.9	-8.5	-8.0	0.40	1.731	0.389	5.467	1.663	6	1368	ZINC000104373883
0.9	-9.4	-8.4	0.54	1.669	0.490	3.277	1.704	6	1369	ZINC000014643594
0.9	-9.7	-8.2	0.81	1.665	0.322	3.903	1.615	6	1370	ZINC000022062283
0.9	-8.5	-7.8	0.38	1.768	0.131	3.572	0.822	6	1371	ZINC000015147949
0.9	-10.5	-9.5	0.86	1.636	0.550	5.670	1.522	6	1372	ZINC000021986282
0.9	-7.7	-7.2	0.25	2.365	0.342	4.617	0.903	6	1373	ZINC000013387628

0.9	-7.9	-7.7	0.19	2.076	0.328	3.864	0.713	6	1374	ZINC000014593122
0.9	-8.5	-8.1	0.26	2.645	0.444	4.693	1.580	6	1375	ZINC000001530234
0.9	-8.1	-7.6	0.31	2.071	0.504	4.573	1.886	6	1376	ZINC000014886649
0.9	-7.8	-7.5	0.19	1.704	0.601	3.070	0.672	6	1377	ZINC000002034811
0.9	-8.6	-8.2	0.30	2.654	0.340	3.820	1.120	6	1378	ZINC000012153650
0.9	-8.8	-7.7	0.63	1.829	0.037	5.166	0.529	6	1379	ZINC000012496568
0.9	-9.5	-8.5	0.71	1.713	0.424	4.926	1.954	4	1380	ZINC000100779934
0.9	-9.2	-8.3	0.65	2.037	0.724	4.046	1.705	6	1381	ZINC000017141864
0.9	-9.0	-8.5	0.57	1.300	0.089	2.821	0.366	6	1382	ZINC000409421828
0.9	-6.4	-6.2	0.10	2.477	0.325	4.256	0.648	6	1383	ZINC000005273796
0.9	-9.2	-8.5	0.47	1.967	0.361	4.696	1.551	6	1384	ZINC000257390696
0.9	-10.5	-9.6	0.70	1.406	0.402	4.822	2.178	6	1385	ZINC000004023797
0.9	-8.5	-7.8	0.38	2.413	0.514	4.388	1.666	6	1386	ZINC000004787345
0.9	-7.7	-7.4	0.21	2.256	0.506	4.553	0.630	6	1387	ZINC000013550169
0.9	-10.9	-10.2	0.45	1.632	0.424	5.614	1.216	6	1388	ZINC000257448384

0.9	-7.2	-6.9	0.21	1.803	0.475	2.803	1.081	6	1389	ZINC000001850813
0.9	-6.9	-6.4	0.32	2.483	0.186	3.779	0.563	6	1390	ZINC000039618913
0.9	-8.3	-7.8	0.27	1.162	0.407	2.710	0.190	6	1391	ZINC000014690788
0.9	-6.8	-6.6	0.13	1.404	0.384	3.039	0.249	6	1392	ZINC000000967570
0.9	-8.8	-7.9	0.44	1.863	0.280	4.031	1.876	6	1393	ZINC000100825943
0.9	-9.0	-8.6	0.23	2.157	0.871	3.801	0.736	6	1394	ZINC000257525166
0.9	-7.9	-7.7	0.10	1.099	0.539	2.036	0.491	6	1395	ZINC000100024169
0.9	-9.2	-8.5	0.48	1.955	0.347	4.668	1.561	6	1396	ZINC000100781025
0.9	-10.0	-9.0	0.79	1.092	0.204	6.129	1.706	6	1397	ZINC000012494057
0.9	-7.8	-7.5	0.17	1.698	0.593	3.065	0.671	6	1398	ZINC000100601092
0.9	-11.7	-10.7	0.69	1.466	0.238	4.623	0.853	5	1399	ZINC000053277634
0.9	-7.7	-7.3	0.20	2.546	0.505	4.948	1.310	6	1400	ZINC000014855860
0.9	-8.2	-7.7	0.47	1.723	1.027	3.072	1.624	6	1401	ZINC000019735126
0.9	-7.2	-7.0	0.15	1.873	0.415	3.196	1.279	6	1402	ZINC000238760920
0.9	-9.1	-8.4	0.39	2.373	0.475	3.971	0.701	6	1403	ZINC000033831284

0.9	-8.2	-8.0	0.13	2.031	0.327	3.224	0.622	6	1404	ZINC000014855880
0.9	-8.5	-7.9	0.35	2.195	0.603	4.059	1.469	6	1405	ZINC000027731561
0.9	-8.1	-7.7	0.29	1.254	0.186	3.039	0.498	6	1406	ZINC000015115267
0.9	-7.9	-7.4	0.25	1.976	0.586	4.263	0.729	6	1407	ZINC000000057754
0.9	-8.2	-7.9	0.19	1.376	0.188	3.560	0.666	6	1408	ZINC000085880353
0.9	-6.1	-5.8	0.16	2.511	0.867	3.214	0.918	6	1409	ZINC000100828266
0.9	-7.3	-7.1	0.13	2.060	0.448	3.833	0.803	6	1410	ZINC000014764735
0.9	-6.4	-6.2	0.11	1.795	0.403	4.263	0.483	6	1411	ZINC000085972365
0.9	-10.5	-9.2	0.97	1.508	0.620	3.910	2.020	6	1412	ZINC000003814412
0.9	-7.8	-7.4	0.22	2.511	1.531	3.897	2.076	6	1413	ZINC000008215849
0.9	-9.5	-8.3	0.70	1.898	0.444	3.883	1.143	6	1414	ZINC000104370412
0.9	-6.3	-5.6	0.34	2.379	0.243	3.318	0.187	6	1415	ZINC000005736008
0.9	-8.5	-8.1	0.24	1.213	0.206	3.435	0.582	6	1416	ZINC000100826331
0.9	-7.4	-7.2	0.22	2.057	0.151	3.262	0.469	6	1417	ZINC000005706881
0.9	-7.8	-7.7	0.12	1.975	0.196	3.865	1.036	6	1418	ZINC000013306629

0.9	-7.8	-7.6	0.23	1.698	0.575	2.813	1.344	6	1419	ZINC000000410082
0.9	-9.4	-8.7	0.36	2.107	0.665	4.330	1.943	6	1420	ZINC000230066008
0.9	-8.0	-7.4	0.27	1.887	0.310	3.599	1.080	6	1421	ZINC000031475165
0.9	-8.2	-7.8	0.19	2.495	0.461	3.778	0.935	6	1422	ZINC000013510524
0.9	-8.5	-7.9	0.32	1.639	0.435	4.122	1.556	6	1423	ZINC000027638922
0.9	-6.4	-5.8	0.31	2.357	0.810	3.604	0.954	6	1424	ZINC000002027286
0.9	-6.5	-6.4	0.08	2.018	0.563	3.741	1.004	6	1425	ZINC000013546051
0.9	-7.1	-6.5	0.32	2.446	0.425	3.587	0.497	6	1426	ZINC000100776461
0.9	-10.5	-8.9	0.80	1.729	0.423	5.118	2.127	6	1427	ZINC000100779897
0.9	-10.6	-9.4	0.79	1.610	0.302	4.229	1.723	6	1428	ZINC000000402672
0.9	-7.3	-6.8	0.24	2.199	0.322	3.315	0.387	6	1429	ZINC000006494870
0.9	-7.8	-7.4	0.21	1.997	0.075	3.086	0.643	6	1430	ZINC000005752287
0.9	-7.8	-7.4	0.24	2.300	0.216	4.522	1.574	6	1431	ZINC000015261546
0.9	-6.5	-6.3	0.15	3.236	0.561	4.472	0.299	6	1432	ZINC000005821035
0.9	-7.9	-7.4	0.26	1.565	0.479	2.814	1.372	6	1433	ZINC000014767029

0.9	-7.8	-7.5	0.19	1.715	0.611	3.090	0.670	6	1434	ZINC000013521389
0.9	-8.3	-8.0	0.25	1.654	0.800	3.810	1.104	6	1435	ZINC000000968233
0.9	-9.6	-8.7	0.69	1.971	0.513	3.200	1.200	6	1436	ZINC000257547496
0.9	-6.9	-6.8	0.15	1.526	0.379	3.743	0.828	6	1437	ZINC000005752359
0.9	-9.2	-8.4	0.45	2.513	0.587	4.376	0.819	6	1438	ZINC000257437531
0.9	-6.5	-6.2	0.16	1.885	0.226	3.589	0.269	6	1439	ZINC000005159450
0.9	-7.6	-7.2	0.22	2.529	0.284	4.970	0.484	6	1440	ZINC000034951645
0.9	-10.6	-9.5	0.53	1.245	0.174	6.088	1.513	6	1441	ZINC000257525429
0.9	-7.2	-6.6	0.47	2.589	0.710	3.543	0.822	6	1442	ZINC000000404287
0.9	-7.5	-6.9	0.44	1.629	0.795	3.583	0.395	6	1443	ZINC000000173167
0.9	-9.4	-9.2	0.17	2.039	0.118	5.303	2.119	6	1444	ZINC000014646117
0.9	-6.8	-6.6	0.14	3.315	0.821	4.402	1.179	6	1445	ZINC000005447650
0.9	-7.2	-6.8	0.20	2.940	0.178	5.403	0.399	6	1446	ZINC000001530487
0.9	-9.2	-8.5	0.49	1.729	0.374	3.591	1.536	6	1447	ZINC000014619348
0.9	-8.2	-7.9	0.24	2.490	0.518	4.303	0.999	6	1448	ZINC000200632209

0.9	-8.1	-7.9	0.24	1.635	0.252	3.505	0.454	6	1449	ZINC000015274344
0.9	-8.2	-7.7	0.31	1.405	0.251	4.069	0.294	6	1450	ZINC000005431436
0.9	-8.5	-8.3	0.19	1.521	0.458	3.404	1.349	6	1451	ZINC000257387900
0.9	-6.2	-6.0	0.23	1.895	0.751	2.924	1.208	6	1452	ZINC000002515951
0.9	-8.1	-7.9	0.12	2.157	0.608	4.517	1.045	6	1453	ZINC000016347026
0.9	-6.9	-6.7	0.22	1.655	0.988	3.255	0.594	6	1454	ZINC000032162511
0.9	-9.2	-8.5	0.50	2.328	0.590	4.852	1.356	6	1455	ZINC000100781028
0.9	-8.7	-7.6	0.59	1.988	0.520	5.185	1.108	6	1456	ZINC000013439573
0.9	-8.5	-7.8	0.40	1.782	0.223	3.519	1.108	6	1457	ZINC000100783419
0.9	-11.0	-9.6	0.90	1.543	0.272	3.795	1.542	4	1458	ZINC000013485103
0.9	-7.7	-7.5	0.15	2.260	0.267	3.780	1.095	6	1459	ZINC000050027193
0.9	-9.6	-7.6	1.01	1.908	0.360	5.238	0.610	5	1460	ZINC000014950040
0.9	-6.9	-6.6	0.16	2.023	0.552	3.952	1.155	6	1461	ZINC000002040978
0.9	-9.2	-8.5	0.43	2.844	0.848	4.953	1.668	6	1462	ZINC000035967558
0.9	-9.0	-8.4	0.39	2.072	0.633	4.886	1.687	6	1463	ZINC000000051924

0.9	-8.5	-8.0	0.34	1.633	0.625	3.213	0.469	6	1464	ZINC000100783048
0.9	-8.2	-7.9	0.18	1.943	0.481	3.687	1.287	6	1465	ZINC000001539904
0.9	-8.1	-7.5	0.26	2.320	0.242	4.232	0.360	6	1466	ZINC000001664327
0.9	-7.5	-7.1	0.27	2.684	0.815	3.493	0.928	6	1467	ZINC000001849749
0.9	-6.6	-6.5	0.07	1.237	0.115	2.775	0.246	6	1468	ZINC000015169332
0.9	-8.5	-8.0	0.26	1.439	0.321	4.450	0.439	6	1469	ZINC000100825792
0.9	-7.8	-7.4	0.31	2.636	0.554	4.097	1.138	6	1470	ZINC000000135391
0.9	-8.1	-7.7	0.22	2.338	0.663	3.467	1.100	6	1471	ZINC000004095850
0.9	-10.9	-10.1	0.66	1.603	0.391	5.682	1.266	6	1472	ZINC000257448383
0.9	-9.7	-8.8	0.47	1.772	0.368	5.027	1.776	6	1473	ZINC000014820466
0.9	-8.6	-8.2	0.28	2.011	0.300	3.961	0.807	6	1474	ZINC000006030793
0.9	-7.5	-7.1	0.23	2.336	0.303	4.203	0.738	6	1475	ZINC000149825977
0.9	-8.2	-7.8	0.29	1.488	0.295	3.935	0.684	6	1476	ZINC000059200503
0.9	-8.4	-8.0	0.24	2.496	0.786	3.840	0.684	6	1477	ZINC000015120006
0.9	-6.3	-6.0	0.16	1.966	0.715	3.373	0.378	6	1478	ZINC000002020096

0.9	-7.3	-7.0	0.21	1.814	0.554	3.034	0.645	6	1479	ZINC000004284428
0.9	-9.1	-9.0	0.11	0.824	0.137	2.769	0.407	6	1480	ZINC000100823448
0.9	-7.1	-6.5	0.31	2.271	0.316	3.358	0.391	6	1481	ZINC000100880090
0.9	-6.7	-6.6	0.07	2.163	0.441	4.470	0.981	6	1482	ZINC000002036970
0.9	-8.6	-8.2	0.27	1.844	0.362	4.211	2.339	6	1483	ZINC000013533857
0.9	-8.3	-7.9	0.22	2.014	0.262	4.020	0.718	6	1484	ZINC000014616540
0.9	-6.5	-5.9	0.33	2.892	0.381	4.369	0.278	6	1485	ZINC000095620557
0.9	-8.1	-7.3	0.40	2.289	0.456	4.320	0.498	6	1486	ZINC000086031218
0.9	-9.5	-8.6	0.47	1.431	0.247	4.729	1.262	6	1487	ZINC000013486219
0.9	-8.8	-7.6	0.58	1.830	0.359	4.337	0.769	6	1488	ZINC000015220402
0.9	-7.6	-7.4	0.17	2.095	0.355	4.541	0.984	6	1489	ZINC000034961768
0.9	-6.1	-5.8	0.20	1.396	0.211	2.545	0.385	6	1490	ZINC000004975178
0.9	-9.2	-7.6	0.77	1.825	0.165	3.714	0.347	6	1491	ZINC000257527275
0.9	-6.5	-6.0	0.31	1.567	0.316	3.457	0.762	6	1492	ZINC000033610759
0.9	-9.1	-8.4	0.45	2.049	0.440	3.631	0.877	6	1493	ZINC000257502423

0.9	-7.4	-7.1	0.23	1.567	0.261	3.044	1.125	6	1494	ZINC000004284430
0.9	-7.9	-7.1	0.42	1.981	0.261	4.232	1.615	6	1495	ZINC000014653879
0.9	-8.4	-7.9	0.49	3.657	0.200	5.843	0.319	6	1496	ZINC000008215945
0.9	-9.2	-8.5	0.47	1.966	0.356	4.686	1.539	6	1497	ZINC000257390695
0.9	-8.8	-8.7	0.10	1.711	0.370	4.387	1.874	6	1498	ZINC000015249394
0.9	-6.4	-6.0	0.20	3.012	0.711	4.019	0.858	6	1499	ZINC000032163565
0.9	-6.7	-6.1	0.34	2.258	0.897	3.472	1.010	6	1500	ZINC000001997121
0.9	-6.8	-6.5	0.18	2.672	0.272	3.872	0.661	6	1501	ZINC000100826429
0.9	-8.6	-7.9	0.43	1.384	0.404	3.841	0.171	6	1502	ZINC000085850251
0.9	-9.1	-8.6	0.29	1.719	0.661	3.851	0.642	6	1503	ZINC000000968478
0.9	-9.5	-8.6	0.65	1.848	0.136	5.039	0.906	6	1504	ZINC000014826957
0.9	-8.1	-7.7	0.22	1.906	0.765	3.824	1.930	6	1505	ZINC000017255170
0.9	-10.5	-9.5	0.89	1.637	0.546	5.671	1.522	6	1506	ZINC000003814413
0.9	-7.1	-6.8	0.21	2.221	0.888	3.602	0.505	6	1507	ZINC000033951476
0.9	-8.4	-8.2	0.27	1.265	0.146	3.337	0.624	6	1508	ZINC000059778570

0.9	-8.1	-7.5	0.34	1.433	0.368	5.819	0.807	6	1509	ZINC000004262101
0.9	-7.9	-7.5	0.21	1.865	0.228	4.658	1.596	6	1510	ZINC000014556665
0.9	-9.8	-9.2	0.45	2.016	0.579	2.850	0.873	6	1511	ZINC000014619349
0.9	-7.3	-6.8	0.24	2.463	0.359	4.559	0.327	6	1512	ZINC000003978810
0.9	-9.0	-8.6	0.23	1.841	0.759	3.584	0.742	6	1513	ZINC000014860419
0.9	-6.2	-6.0	0.24	2.589	0.475	3.905	0.467	6	1514	ZINC000000407078
0.9	-8.9	-8.2	0.42	1.581	0.491	3.735	0.599	6	1515	ZINC000085826897
0.9	-8.7	-8.0	0.49	2.257	0.575	3.371	1.239	6	1516	ZINC000005923859
0.9	-7.6	-7.0	0.33	2.416	0.750	3.678	0.701	6	1517	ZINC000095619240
0.9	-10.2	-8.7	0.88	1.614	0.139	5.178	1.230	6	1518	ZINC000000402719
0.9	-7.1	-6.5	0.31	2.355	0.411	3.503	0.432	6	1519	ZINC000039204664
0.9	-9.6	-8.7	0.66	2.102	0.146	4.929	1.876	6	1520	ZINC000000968073
0.9	-8.8	-8.0	0.41	2.176	0.543	4.868	1.159	6	1521	ZINC000015160973
0.9	-7.5	-7.3	0.21	1.551	0.216	3.321	0.161	6	1522	ZINC000000163859
0.9	-9.0	-8.5	0.26	1.764	1.019	3.102	0.982	6	1523	ZINC000085845240

0.9	-7.3	-7.0	0.15	1.374	0.177	3.121	0.483	6	1524	ZINC000015253565
0.9	-6.2	-5.9	0.21	1.779	0.454	3.129	0.389	6	1525	ZINC000002020134
0.9	-6.2	-5.8	0.23	2.820	1.004	4.551	0.550	6	1526	ZINC000015272539
0.9	-6.6	-6.2	0.21	1.947	0.284	3.566	0.570	6	1527	ZINC000014588589
0.9	-7.7	-7.0	0.45	2.381	0.383	3.544	1.066	6	1528	ZINC000030731196
0.9	-8.2	-7.8	0.32	1.869	0.163	3.597	0.535	6	1529	ZINC000015274347
0.9	-9.0	-8.6	0.23	2.153	0.862	3.796	0.726	6	1530	ZINC000257525165
0.9	-9.5	-8.6	0.47	1.430	0.248	4.730	1.261	6	1531	ZINC000100829340
0.9	-7.8	-7.5	0.19	1.727	0.606	3.091	0.663	6	1532	ZINC000100601089
0.9	-8.8	-8.5	0.18	1.910	0.102	5.050	1.474	6	1533	ZINC000257534558
0.9	-10.0	-8.8	0.90	1.268	0.167	6.063	1.835	6	1534	ZINC000012494021
0.9	-7.9	-7.4	0.28	2.255	0.270	4.124	0.642	6	1535	ZINC000005761430
0.9	-9.5	-8.6	0.47	1.432	0.249	4.730	1.261	6	1536	ZINC000257499925
0.9	-10.7	-9.9	0.78	1.495	0.672	4.015	0.861	6	1537	ZINC000100779675
0.9	-10.5	-9.5	0.86	1.493	0.407	4.945	2.005	6	1538	ZINC000026958925

0.9	-7.0	-6.8	0.15	1.989	0.423	3.431	0.462	6	1539	ZINC000014613370
0.9	-9.9	-8.9	0.65	1.730	0.527	3.270	1.678	6	1540	ZINC000100060558
0.9	-6.1	-6.0	0.08	1.750	0.390	3.986	0.322	6	1541	ZINC000039236335
0.9	-8.7	-8.0	0.36	1.569	0.221	3.814	2.272	6	1542	ZINC000100825939
0.9	-7.5	-7.2	0.16	2.343	0.659	4.389	1.293	6	1543	ZINC000100779837
0.9	-8.5	-8.0	0.30	1.962	0.178	4.253	0.220	6	1544	ZINC000015265065
0.9	-8.3	-7.6	0.38	2.457	0.422	3.805	0.899	6	1545	ZINC000035940762
0.9	-9.2	-8.7	0.35	2.045	0.440	3.817	1.266	6	1546	ZINC000014689493
0.9	-7.9	-7.6	0.19	2.805	0.336	4.336	0.866	6	1547	ZINC000013828020
0.9	-6.5	-6.3	0.09	1.874	0.728	2.893	1.036	6	1548	ZINC000095619759
0.9	-7.3	-7.0	0.19	1.682	0.288	3.056	0.875	6	1549	ZINC000008616482
0.9	-7.4	-7.2	0.12	1.455	0.281	4.198	2.479	6	1550	ZINC000014725865
0.9	-9.8	-8.2	0.94	1.799	0.292	5.185	1.075	6	1551	ZINC000257551982
0.9	-9.3	-8.4	0.64	1.909	0.308	3.616	1.139	6	1552	ZINC000257547495
0.9	-9.4	-8.2	0.92	1.550	0.377	3.960	1.515	6	1553	ZINC000014643666

0.9	-8.5	-7.9	0.28	1.640	0.291	3.332	1.055	6	1554	ZINC000014854734
0.9	-7.6	-7.2	0.21	1.693	0.341	3.102	0.933	6	1555	ZINC000031475157
0.9	-8.3	-7.8	0.30	2.036	0.560	4.656	0.859	6	1556	ZINC000014776358
0.9	-6.9	-6.4	0.23	1.726	0.415	3.350	1.028	6	1557	ZINC000001850470
0.9	-7.9	-7.8	0.07	2.149	0.295	4.278	0.993	6	1558	ZINC000000895837
0.9	-8.3	-8.1	0.21	1.181	0.070	3.273	0.468	6	1559	ZINC000056874637
0.9	-10.9	-10.3	0.33	1.426	0.370	4.561	2.054	6	1560	ZINC000100780586
0.9	-6.6	-6.1	0.32	1.833	0.372	3.517	0.450	6	1561	ZINC000000967523
0.9	-8.5	-8.3	0.17	1.121	0.169	3.109	0.678	6	1562	ZINC000100780741
0.9	-9.0	-7.7	0.67	1.464	0.085	3.719	1.651	6	1563	ZINC000015046811
0.9	-10.6	-9.4	0.80	1.725	0.242	3.730	1.435	6	1564	ZINC000014887279
0.9	-7.2	-7.0	0.11	1.836	0.651	4.355	1.841	6	1565	ZINC000002516116
0.9	-6.6	-6.4	0.11	2.532	0.297	3.936	0.873	6	1566	ZINC000001529575
0.9	-8.3	-8.0	0.17	1.660	0.378	3.812	1.558	6	1567	ZINC000100781828
0.9	-6.5	-6.3	0.20	2.272	0.431	3.427	0.625	6	1568	ZINC000001580553

0.9	-8.0	-7.5	0.36	2.805	0.863	5.112	2.119	6	1569	ZINC000004517830
0.9	-7.7	-7.2	0.24	2.721	0.346	3.888	0.564	6	1570	ZINC000002570889
0.9	-9.2	-8.0	0.82	1.714	0.177	5.193	0.241	4	1571	ZINC000086010705
0.9	-7.1	-6.5	0.32	2.388	0.341	3.340	0.448	6	1572	ZINC000012153566
0.9	-8.9	-8.1	0.62	1.953	0.572	4.052	1.970	6	1573	ZINC000013334897
0.9	-8.7	-8.2	0.24	1.869	0.438	5.215	2.155	6	1574	ZINC000013532102
0.9	-9.5	-8.8	0.41	2.139	0.264	4.237	1.722	6	1575	ZINC000064560766
0.9	-9.0	-8.5	0.25	1.861	0.980	3.202	0.913	6	1576	ZINC000100783134
0.9	-9.0	-8.6	0.28	1.758	0.550	3.779	1.531	6	1577	ZINC000013340547
0.9	-9.0	-8.6	0.23	2.151	0.861	3.793	0.726	6	1578	ZINC000257525167
0.9	-6.5	-6.2	0.21	2.698	0.758	4.312	1.028	6	1579	ZINC000261499611
0.9	-6.0	-5.8	0.11	1.997	0.559	3.843	0.364	6	1580	ZINC000078954729
0.9	-6.7	-6.3	0.20	2.160	0.223	3.532	0.698	6	1581	ZINC000005419315
0.9	-10.9	-9.6	1.08	1.691	0.534	6.026	0.326	6	1582	ZINC000257448385
0.9	-9.1	-9.0	0.11	0.822	0.138	2.756	0.402	6	1583	ZINC000100823449

0.9	-7.7	-7.3	0.29	1.870	0.935	3.322	1.828	6	1584	ZINC000001644136
0.9	-8.0	-7.5	0.34	2.050	0.450	4.122	1.572	6	1585	ZINC000085662224
0.9	-9.1	-8.7	0.31	2.962	0.442	5.450	0.681	6	1586	ZINC000100051679
0.9	-7.7	-7.2	0.34	1.873	0.351	2.975	0.794	6	1587	ZINC000095617959
0.9	-8.3	-7.7	0.31	1.892	0.384	3.866	0.697	6	1588	ZINC000230541799
0.9	-10.5	-9.4	0.88	1.431	0.382	4.319	1.983	6	1589	ZINC000003814414
0.9	-7.1	-6.8	0.16	2.186	0.476	3.321	1.274	6	1590	ZINC000003869623
0.9	-7.5	-6.9	0.27	3.157	0.590	5.466	0.501	6	1591	ZINC000100779841
0.9	-6.7	-6.6	0.07	2.671	0.582	4.517	1.061	6	1592	ZINC000014511113
0.9	-9.1	-8.7	0.26	2.208	0.277	4.352	1.031	6	1593	ZINC000015153341
0.9	-10.5	-9.5	0.89	1.631	0.554	5.668	1.522	6	1594	ZINC000004175380
0.9	-8.0	-7.5	0.24	1.561	0.321	3.090	0.807	6	1595	ZINC000015257097
0.9	-8.3	-7.8	0.29	1.170	0.401	2.720	0.203	6	1596	ZINC000100781100
0.9	-10.5	-9.0	1.05	1.666	0.527	4.169	1.706	6	1597	ZINC000005764361
0.9	-5.8	-5.4	0.21	1.598	0.383	2.938	0.782	6	1598	ZINC000002001176

0.9	-7.0	-6.7	0.23	1.676	0.870	2.657	0.673	6	1599	ZINC000000281856
0.9	-7.0	-6.4	0.31	1.348	0.163	4.821	0.043	6	1600	ZINC000031290674
0.9	-9.2	-8.5	0.75	1.898	0.322	4.073	0.931	6	1601	ZINC000257558390
0.9	-9.0	-7.9	0.51	1.793	0.190	3.822	1.267	6	1602	ZINC000006070284
0.9	-9.3	-8.3	0.61	2.230	0.200	4.334	0.957	6	1603	ZINC000006116533
0.9	-7.3	-6.9	0.29	2.477	0.573	4.449	0.359	6	1604	ZINC000095620536
0.9	-7.1	-6.9	0.18	2.751	0.941	4.188	1.086	6	1605	ZINC000100774485
0.9	-9.8	-8.7	0.73	1.900	0.451	3.603	0.840	6	1606	ZINC000013461655
0.9	-9.5	-8.5	0.60	1.534	0.331	5.053	1.050	6	1607	ZINC000013486220
0.9	-7.0	-6.6	0.24	2.112	0.409	3.877	0.436	6	1608	ZINC000005179609
0.9	-8.2	-7.7	0.32	1.797	0.401	3.818	1.077	6	1609	ZINC000059588065
0.9	-8.0	-7.5	0.29	1.584	0.294	3.351	0.707	6	1610	ZINC000006037626
0.9	-9.8	-7.6	1.10	1.256	0.144	4.309	0.321	5	1611	ZINC000257406935
0.9	-7.0	-6.7	0.18	3.564	0.530	4.813	0.628	6	1612	ZINC000014504598
0.9	-9.5	-8.6	0.47	1.428	0.254	4.729	1.262	6	1613	ZINC000257499926

0.9	-10.9	-10.0	0.87	1.575	0.387	5.021	1.388	6	1614	ZINC000100780578
0.9	-9.1	-8.0	0.69	2.006	0.326	4.290	1.230	6	1615	ZINC000015061510
0.9	-8.0	-7.8	0.17	2.331	0.752	2.960	0.770	6	1616	ZINC000014619019
0.9	-7.8	-6.9	0.39	2.569	0.483	4.618	1.330	6	1617	ZINC000005157173
0.9	-9.5	-8.6	0.47	1.427	0.250	4.729	1.262	6	1618	ZINC000100829339
0.9	-9.6	-8.6	0.68	2.305	0.571	3.910	1.677	6	1619	ZINC000035653092
0.9	-10.4	-9.7	0.56	1.405	0.486	3.618	1.068	6	1620	ZINC000230084434
0.9	-8.2	-7.5	0.50	2.388	0.390	4.428	0.173	6	1621	ZINC000238731716
0.9	-8.9	-8.2	0.32	1.918	0.365	3.311	0.579	6	1622	ZINC000085506308
0.9	-9.0	-8.6	0.30	1.908	0.939	3.331	0.792	6	1623	ZINC000085845247
0.9	-8.5	-8.1	0.29	2.044	1.077	4.207	1.067	6	1624	ZINC000000895404
0.9	-9.2	-7.7	0.73	2.233	0.654	5.037	1.843	6	1625	ZINC000002565773
0.9	-7.5	-7.0	0.28	2.912	0.682	5.015	1.051	6	1626	ZINC000034866769
0.9	-7.0	-6.4	0.29	3.049	1.082	4.570	1.653	6	1627	ZINC000095618095
0.9	-9.0	-8.6	0.29	1.876	1.021	3.367	0.890	6	1628	ZINC000100783140

0.9	-9.9	-9.2	0.35	2.444	0.327	4.195	0.608	6	1629	ZINC000004099031
0.9	-8.0	-7.6	0.25	1.753	0.561	4.267	1.468	6	1630	ZINC000036440830
0.9	-8.6	-8.2	0.26	1.641	0.220	3.594	0.498	6	1631	ZINC000100780742
0.9	-8.5	-7.8	0.34	1.907	0.395	3.526	0.857	6	1632	ZINC000015209386
0.9	-7.8	-7.5	0.21	1.704	0.374	4.015	0.873	6	1633	ZINC000015219230
0.9	-7.4	-7.2	0.26	1.775	0.379	3.617	1.279	6	1634	ZINC000014494692
0.9	-8.5	-8.2	0.25	1.955	0.498	4.344	1.636	6	1635	ZINC000257387897
0.9	-9.2	-8.3	0.47	1.577	0.350	5.647	1.935	6	1636	ZINC000001531191
0.9	-6.4	-5.9	0.28	2.759	0.978	3.651	0.977	6	1637	ZINC000012153686
0.9	-6.2	-5.7	0.24	2.009	0.383	2.723	0.244	6	1638	ZINC000000407092
0.9	-8.0	-7.3	0.34	1.486	0.183	3.197	0.451	6	1639	ZINC000005735747
0.9	-7.1	-6.9	0.18	1.637	0.335	3.236	0.071	6	1640	ZINC000013480060
0.9	-10.5	-9.0	0.77	1.818	0.391	5.324	1.817	6	1641	ZINC000100779893
0.9	-6.7	-6.3	0.24	2.284	0.467	3.469	0.740	6	1642	ZINC000013429394
0.9	-9.2	-8.6	0.48	2.205	0.329	3.778	0.931	6	1643	ZINC000015151397

0.9	-6.1	-5.8	0.19	1.604	0.168	3.440	0.135	6	1644	ZINC000261497510
0.9	-10.0	-8.6	0.83	1.658	0.229	3.889	1.486	6	1645	ZINC000015055024
0.9	-8.7	-8.1	0.43	1.549	0.215	4.114	0.243	6	1646	ZINC000105054083
0.9	-7.1	-6.5	0.31	1.991	0.739	3.016	0.970	6	1647	ZINC000038613343
0.9	-10.4	-9.6	0.63	1.508	0.477	4.371	0.926	6	1648	ZINC000086028132
0.9	-7.4	-7.0	0.30	1.547	0.765	3.350	0.547	6	1649	ZINC000014822225
0.9	-6.5	-6.1	0.21	1.653	0.754	2.537	1.264	6	1650	ZINC000085972363
0.9	-7.1	-6.9	0.13	2.046	0.125	4.004	0.147	6	1651	ZINC000014588995
0.9	-8.2	-7.6	0.36	2.470	0.654	3.749	0.818	6	1652	ZINC000100029132
0.9	-10.9	-10.1	0.63	1.668	0.470	5.592	1.201	6	1653	ZINC000257448386
0.9	-9.0	-8.6	0.24	2.163	0.873	3.821	0.751	6	1654	ZINC000257525164
0.9	-10.8	-9.8	0.56	1.787	0.457	4.104	1.723	6	1655	ZINC000100780634
0.9	-9.4	-8.6	0.55	1.787	0.083	4.427	1.369	6	1656	ZINC000014826960
0.9	-8.7	-8.4	0.27	1.471	0.185	3.815	0.591	6	1657	ZINC000044607970
0.9	-6.8	-6.5	0.14	1.798	0.189	2.955	0.276	6	1658	ZINC000004521894

0.9	-8.1	-7.9	0.16	2.503	0.928	4.211	1.900	6	1659	ZINC000001081581
0.9	-6.3	-6.0	0.24	1.840	0.295	2.645	0.353	6	1660	ZINC000100828320
0.9	-7.9	-7.4	0.37	2.013	0.534	2.655	1.091	6	1661	ZINC000000901361
0.9	-8.6	-8.0	0.41	1.787	0.369	3.900	1.323	6	1662	ZINC000015209575
0.9	-7.6	-7.0	0.33	2.296	0.652	3.709	0.686	6	1663	ZINC000100823363
0.9	-8.6	-8.1	0.33	1.594	0.345	3.773	0.786	6	1664	ZINC000087528988
0.9	-6.8	-6.6	0.18	1.454	0.085	2.903	0.260	6	1665	ZINC000025720799
0.9	-8.5	-8.0	0.34	1.897	0.762	3.564	0.727	6	1666	ZINC000100783047
0.9	-7.4	-7.1	0.26	1.593	0.467	2.960	1.205	6	1667	ZINC000014651626
0.9	-6.8	-6.5	0.18	1.784	0.414	3.077	0.400	6	1668	ZINC000005457565
0.9	-7.4	-6.6	0.37	2.574	0.567	3.613	0.611	6	1669	ZINC000004632126
0.9	-8.5	-8.2	0.20	1.464	0.134	3.542	0.912	6	1670	ZINC000100826335
0.9	-8.9	-8.1	0.54	1.988	0.721	3.598	1.361	6	1671	ZINC000085994611
0.9	-12.3	-12.3	0.00	0.000	0.000	0.000	0.000	1	1672	ZINC000059735702
0.9	-8.5	-7.9	0.33	2.691	0.672	4.312	1.761	6	1673	ZINC000036478809

0.9	-8.1	-7.9	0.21	1.389	0.125	3.887	0.527	6	1674	ZINC000005767209
0.9	-7.3	-6.6	0.34	2.949	0.946	4.099	1.420	6	1675	ZINC000004234860
0.9	-9.2	-8.8	0.28	2.637	0.224	4.168	0.531	6	1676	ZINC000013381476
0.9	-8.7	-7.6	0.59	2.191	0.293	4.742	0.577	6	1677	ZINC000001844838
0.8	-8.7	-8.0	0.59	1.605	0.433	4.252	0.715	6	1678	ZINC000059200500
0.8	-8.8	-8.4	0.29	1.820	0.329	4.054	0.171	6	1679	ZINC000085599405
0.8	-9.3	-7.8	0.83	2.812	0.227	5.837	1.141	6	1680	ZINC000014820468
0.8	-7.0	-6.7	0.16	2.059	0.388	3.603	0.455	6	1681	ZINC000001597137
0.8	-8.7	-7.8	0.45	1.927	0.358	3.972	0.851	6	1682	ZINC000013371321
0.8	-8.1	-7.3	0.48	2.194	0.299	4.717	0.941	6	1683	ZINC000230103228
0.8	-7.2	-7.0	0.20	2.085	0.249	4.422	1.388	6	1684	ZINC000014610450
0.8	-9.9	-8.9	0.63	1.965	0.559	3.745	0.828	6	1685	ZINC000015160890
0.8	-5.7	-5.6	0.07	1.939	0.417	3.131	0.382	6	1686	ZINC000095620503
0.8	-7.4	-7.1	0.20	3.253	0.472	4.301	0.468	6	1687	ZINC000001849924
0.8	-7.3	-6.9	0.26	2.774	0.512	3.793	0.678	6	1688	ZINC000016041099

0.8	-7.9	-7.5	0.35	2.742	0.666	4.141	0.580	6	1689	ZINC000005769987
0.8	-6.8	-6.5	0.19	1.842	0.505	2.990	1.774	6	1690	ZINC000002043162
0.8	-8.8	-8.3	0.43	1.486	0.288	2.564	0.588	6	1691	ZINC000257639975
0.8	-6.6	-6.1	0.29	3.067	0.905	3.840	0.641	6	1692	ZINC000005133244
0.8	-7.6	-7.2	0.24	1.775	0.157	2.978	0.621	6	1693	ZINC000031500056
0.8	-8.1	-7.4	0.49	1.395	0.223	3.804	0.441	6	1694	ZINC000013381726
0.8	-5.9	-5.6	0.13	2.591	0.474	3.971	0.425	6	1695	ZINC000002578956
0.8	-7.9	-7.4	0.41	1.449	0.251	3.105	1.043	6	1696	ZINC000100776288
0.8	-9.3	-8.8	0.37	1.936	0.238	3.329	0.750	6	1697	ZINC000004651219
0.8	-7.4	-7.0	0.24	3.792	0.873	5.167	1.118	6	1698	ZINC000013508949
0.8	-5.3	-5.0	0.22	2.104	0.606	3.119	0.785	6	1699	ZINC000031351564
0.8	-8.2	-7.4	0.38	2.168	0.305	3.453	0.713	6	1700	ZINC000001575524
0.8	-9.9	-9.0	0.57	1.839	0.505	4.010	0.781	6	1701	ZINC000015160889
0.8	-8.8	-7.9	0.70	2.436	0.333	4.594	0.536	6	1702	ZINC000013436567
0.8	-6.1	-6.0	0.15	2.092	0.732	3.210	1.288	6	1703	ZINC000013827879

0.8	-6.9	-6.6	0.16	2.034	0.625	3.834	0.502	6	1704	ZINC000015169319
0.8	-8.0	-7.7	0.23	1.540	0.821	2.242	0.579	6	1705	ZINC000004791944
0.8	-6.4	-6.1	0.19	2.826	0.875	3.925	1.142	6	1706	ZINC000003860323
0.8	-8.4	-7.8	0.44	1.535	0.227	4.024	1.105	6	1707	ZINC000257416554
0.8	-7.4	-7.1	0.15	2.660	0.365	4.783	0.990	6	1708	ZINC000000035111
0.8	-7.2	-7.0	0.13	2.170	0.717	3.573	1.524	6	1709	ZINC000003869583
0.8	-7.6	-7.2	0.19	1.750	0.148	3.856	1.715	6	1710	ZINC000238775504
0.8	-7.3	-7.2	0.11	2.458	0.554	3.818	0.633	6	1711	ZINC000001532634
0.8	-7.9	-7.2	0.37	2.412	0.148	4.626	1.243	6	1712	ZINC000040165383
0.8	-6.6	-6.3	0.19	2.412	0.513	4.249	0.478	6	1713	ZINC000005512459
0.8	-7.5	-7.3	0.21	1.412	0.523	3.916	0.727	6	1714	ZINC000001693431
0.8	-6.8	-6.5	0.15	1.279	0.222	2.763	0.338	6	1715	ZINC000002038672
0.8	-7.2	-6.9	0.15	2.236	0.248	3.103	0.662	6	1716	ZINC000100778222
0.8	-8.4	-7.5	0.46	1.839	0.289	5.210	0.476	6	1717	ZINC000257517910
0.8	-7.6	-7.3	0.19	1.745	0.878	4.492	0.849	6	1718	ZINC000001504642

0.8	-7.1	-6.7	0.25	2.896	1.346	4.317	1.607	6	1719	ZINC000082395907
0.8	-6.3	-6.0	0.17	2.625	0.830	3.508	0.988	6	1720	ZINC000032163569
0.8	-9.8	-7.9	1.01	1.750	0.527	4.232	1.898	5	1721	ZINC000003814379
0.8	-8.0	-7.5	0.37	2.382	0.723	4.754	1.694	6	1722	ZINC000005191517
0.8	-7.5	-7.0	0.32	1.853	0.388	3.458	1.525	6	1723	ZINC000000295922
0.8	-9.1	-8.3	0.60	1.808	0.171	5.210	1.029	6	1724	ZINC000012496092
0.8	-7.6	-7.1	0.33	2.115	0.222	5.583	0.965	6	1725	ZINC000001532512
0.8	-7.5	-7.1	0.29	2.164	0.648	3.647	1.249	6	1726	ZINC000000391177
0.8	-8.1	-7.7	0.26	1.073	0.080	2.939	0.992	6	1727	ZINC000100780744
0.8	-6.8	-6.5	0.13	2.017	0.541	3.381	1.081	6	1728	ZINC000000967799
0.8	-7.5	-7.2	0.26	2.133	0.705	3.365	1.550	6	1729	ZINC000013372532
0.8	-8.4	-8.1	0.21	1.535	0.255	3.330	0.843	6	1730	ZINC000100781837
0.8	-6.8	-6.6	0.14	2.407	0.540	3.616	0.703	6	1731	ZINC000013433792
0.8	-9.6	-8.8	0.36	1.980	0.400	4.667	0.777	6	1732	ZINC000013380528
0.8	-7.8	-6.9	0.57	2.078	1.074	4.313	1.843	6	1733	ZINC000014494689

0.8	-7.6	-7.1	0.32	2.209	0.434	3.382	1.366	6	1734	ZINC000034273693
0.8	-10.0	-9.0	0.79	1.092	0.204	6.128	1.705	6	1735	ZINC000261496105
0.8	-9.7	-9.0	0.59	1.911	0.329	3.704	1.402	6	1736	ZINC000013327852
0.8	-6.1	-5.8	0.19	3.748	0.659	4.929	0.509	6	1737	ZINC000005513289
0.8	-6.9	-6.5	0.21	1.972	0.327	3.728	0.555	6	1738	ZINC000017149354
0.8	-6.4	-6.0	0.21	1.866	0.430	3.707	0.457	6	1739	ZINC000002040466
0.8	-8.5	-8.0	0.29	2.251	0.354	3.712	0.361	6	1740	ZINC000006093727
0.8	-9.9	-9.6	0.20	1.570	0.726	3.362	1.691	6	1741	ZINC000004096947
0.8	-8.5	-7.1	0.70	2.039	0.494	4.855	1.802	6	1742	ZINC000014411632
0.8	-6.5	-6.1	0.20	2.229	0.356	4.434	0.644	6	1743	ZINC000032166986
0.8	-7.6	-7.3	0.24	1.331	0.282	2.956	0.257	6	1744	ZINC000100018123
0.8	-5.0	-4.9	0.07	1.685	0.548	3.160	0.348	6	1745	ZINC000000398630
0.8	-9.8	-9.5	0.17	2.812	0.468	5.162	0.892	6	1746	ZINC000001865983
0.8	-7.6	-7.2	0.25	2.454	0.504	4.487	1.622	6	1747	ZINC000002570876
0.8	-7.9	-7.5	0.21	1.887	0.277	3.031	0.274	6	1748	ZINC000002384631

0.8	-7.3	-7.0	0.20	1.460	0.202	4.782	1.252	6	1749	ZINC000001850064
0.8	-6.8	-6.2	0.28	2.475	0.917	3.654	1.149	6	1750	ZINC000012494296
0.8	-10.5	-9.1	0.69	1.836	0.384	6.049	1.390	6	1751	ZINC000100779892
0.8	-8.3	-8.0	0.19	2.431	0.213	5.069	1.652	6	1752	ZINC000005138489
0.8	-10.4	-9.6	0.66	1.687	0.251	4.242	1.172	6	1753	ZINC000100780489
0.8	-6.1	-6.0	0.14	1.325	0.461	3.442	0.501	6	1754	ZINC000001648299
0.8	-7.6	-7.3	0.14	2.424	0.760	3.953	1.278	6	1755	ZINC000004811698
0.8	-6.7	-6.5	0.14	2.664	0.536	4.590	0.741	6	1756	ZINC000005053120
0.8	-8.4	-6.8	0.91	1.882	0.436	3.972	1.507	6	1757	ZINC000257567680
0.8	-9.9	-9.0	0.59	1.825	0.486	4.000	0.794	6	1758	ZINC000256516078
0.8	-6.7	-6.4	0.17	2.193	0.394	4.217	0.866	6	1759	ZINC000039090699
0.8	-6.4	-6.2	0.18	1.514	0.403	3.784	1.046	6	1760	ZINC000000336939
0.8	-10.4	-9.6	0.56	1.609	0.498	3.308	0.723	6	1761	ZINC000005159763
0.8	-7.5	-7.1	0.31	1.681	0.259	2.636	0.470	6	1762	ZINC000004991433
0.8	-9.3	-8.8	0.28	2.194	0.412	4.080	0.486	6	1763	ZINC000014619351

0.8	-7.9	-7.8	0.13	2.618	0.439	4.467	1.677	6	1764	ZINC000014855851
0.8	-8.1	-7.6	0.42	2.343	0.504	4.246	1.474	6	1765	ZINC000005706874
0.8	-7.1	-6.8	0.18	3.379	0.784	4.524	0.913	6	1766	ZINC000001720924
0.8	-7.9	-7.6	0.26	1.520	0.504	2.711	0.940	6	1767	ZINC000001644079
0.8	-7.6	-7.3	0.18	1.885	0.147	4.782	1.336	6	1768	ZINC000014610446
0.8	-10.1	-9.2	0.71	2.522	0.428	4.618	0.585	6	1769	ZINC000004098221
0.8	-7.2	-6.3	0.42	3.820	0.170	5.258	0.370	6	1770	ZINC000082395908
0.8	-8.3	-7.0	0.68	2.343	0.751	4.616	2.016	6	1771	ZINC000257499260
0.8	-7.6	-7.4	0.17	1.963	0.452	3.609	0.930	6	1772	ZINC000002044619
0.8	-6.2	-6.1	0.07	1.908	1.171	4.107	0.662	6	1773	ZINC000001646630
0.8	-8.1	-7.7	0.25	2.160	0.913	4.440	1.263	6	1774	ZINC000095620809
0.8	-8.4	-7.8	0.34	1.737	0.242	4.167	0.230	6	1775	ZINC000033950651
0.8	-9.1	-7.6	0.96	2.159	0.358	4.545	2.097	5	1776	ZINC000014642741
0.8	-6.8	-6.6	0.10	2.826	0.517	4.358	0.381	6	1777	ZINC000014636772
0.8	-9.5	-8.3	0.59	2.186	0.455	4.523	1.979	6	1778	ZINC000000135453

0.8	-5.5	-5.4	0.11	0.493	0.813	3.254	0.561	6	1779	ZINC000003860474
0.8	-7.9	-7.7	0.13	2.177	0.230	3.800	1.021	6	1780	ZINC000142643559
0.8	-6.6	-6.5	0.07	1.577	0.067	2.799	0.291	6	1781	ZINC000000391989
0.8	-10.9	-8.6	1.05	2.096	0.484	4.971	1.035	6	1782	ZINC000014613038
0.8	-7.5	-7.1	0.23	1.760	0.383	3.200	1.645	6	1783	ZINC000004991439
0.8	-8.3	-8.0	0.16	1.465	0.206	3.411	0.691	6	1784	ZINC000002573806
0.8	-6.4	-5.8	0.26	1.950	0.210	4.230	0.208	6	1785	ZINC000095620596
0.8	-6.8	-6.5	0.19	2.252	0.441	4.744	1.208	6	1786	ZINC000001576294
0.8	-10.7	-9.4	1.05	1.561	0.250	5.118	0.519	5	1787	ZINC000014715670
0.8	-7.8	-7.2	0.41	2.172	0.974	2.768	0.894	6	1788	ZINC000006071082
0.8	-8.6	-7.9	0.56	1.632	0.447	3.517	0.917	6	1789	ZINC000100782244
0.8	-7.7	-7.2	0.25	2.128	0.296	4.944	0.372	6	1790	ZINC000014494726
0.8	-9.1	-8.6	0.29	2.186	0.195	3.881	0.265	6	1791	ZINC000100823694
0.8	-8.3	-7.9	0.27	1.779	0.441	3.975	1.648	6	1792	ZINC000095617969
0.8	-8.6	-7.9	0.35	1.703	0.209	4.812	0.790	6	1793	ZINC000257507767

0.8	-10.8	-9.6	0.73	2.039	0.383	5.598	1.225	6	1794	ZINC000100780487
0.8	-8.7	-8.5	0.12	2.159	0.195	5.329	0.716	6	1795	ZINC000090516505
0.8	-6.8	-6.4	0.20	1.980	0.588	3.510	0.645	6	1796	ZINC000100778219
0.8	-6.7	-6.5	0.16	1.912	1.037	3.027	1.081	6	1797	ZINC000013526620
0.8	-6.8	-6.4	0.21	2.404	0.473	3.755	0.864	6	1798	ZINC000242649151
0.8	-9.3	-8.5	0.43	1.912	0.428	4.679	0.904	6	1799	ZINC000019624717
0.8	-8.8	-7.8	0.57	1.822	0.149	4.603	1.146	6	1800	ZINC000012496572
0.8	-10.6	-9.5	0.86	1.521	0.205	3.853	1.445	4	1801	ZINC000015148353
0.8	-6.9	-6.5	0.22	1.544	0.082	2.838	0.574	6	1802	ZINC000015169338
0.8	-6.3	-6.0	0.20	1.799	0.223	3.284	1.023	6	1803	ZINC000261499724
0.8	-9.2	-8.8	0.41	1.733	0.421	5.965	0.811	6	1804	ZINC000014437693
0.8	-7.9	-7.6	0.20	1.983	0.746	3.667	0.658	6	1805	ZINC000001680841
0.8	-9.2	-8.4	0.40	1.552	0.390	5.150	1.769	6	1806	ZINC000257452997
0.8	-5.3	-5.2	0.05	1.413	0.321	3.123	0.718	6	1807	ZINC000001606156
0.8	-8.4	-7.8	0.32	2.109	0.714	3.394	1.552	6	1808	ZINC000001319891

0.8	-6.1	-6.0	0.07	2.386	0.874	3.884	1.147	6	1809	ZINC000095618192
0.8	-6.1	-5.9	0.11	2.097	0.352	3.655	0.892	6	1810	ZINC000095619999
0.8	-6.3	-5.7	0.28	2.209	0.401	3.535	0.835	6	1811	ZINC000002567751
0.8	-9.5	-8.0	0.96	1.956	0.176	5.332	1.214	6	1812	ZINC000015266626
0.8	-5.5	-5.4	0.04	2.016	0.729	3.771	0.986	6	1813	ZINC000001606158
0.8	-5.5	-5.2	0.14	2.319	0.348	3.623	0.896	6	1814	ZINC000003860971
0.8	-8.0	-7.6	0.33	1.721	0.432	3.263	1.502	6	1815	ZINC000015219232
0.8	-10.0	-9.0	0.79	1.090	0.203	6.128	1.705	6	1816	ZINC000085589778
0.8	-7.9	-7.3	0.41	1.990	0.827	2.720	0.812	6	1817	ZINC000001730678
0.8	-6.2	-5.9	0.16	1.923	0.904	3.728	0.866	6	1818	ZINC000002038280
0.8	-6.9	-6.8	0.10	0.914	0.547	2.858	0.540	6	1819	ZINC000100826347
0.8	-9.0	-8.2	0.55	2.295	0.376	4.278	0.483	6	1820	ZINC000100824680
0.8	-8.5	-8.0	0.31	1.540	0.693	3.473	1.366	6	1821	ZINC000071254667
0.8	-8.7	-8.0	0.30	1.534	0.231	3.530	1.622	6	1822	ZINC000015113372
0.8	-8.3	-7.9	0.26	2.400	0.459	4.179	1.422	6	1823	ZINC000012494419

0.8	-8.8	-7.9	0.44	1.696	0.157	3.686	1.354	6	1824	ZINC000006070293
0.8	-9.5	-8.0	0.73	2.673	1.229	4.411	1.979	6	1825	ZINC000001631259
0.8	-6.0	-5.8	0.16	2.293	0.780	3.811	0.576	6	1826	ZINC000002560610
0.8	-7.9	-7.5	0.25	2.103	0.652	3.489	1.592	6	1827	ZINC000060037129
0.8	-10.3	-8.8	0.95	1.642	0.127	3.934	1.704	4	1828	ZINC000013302605
0.8	-7.1	-6.5	0.29	2.371	0.233	4.706	0.204	6	1829	ZINC000034267179
0.8	-6.6	-6.4	0.13	2.638	1.063	4.522	1.502	6	1830	ZINC000013398042
0.8	-6.3	-6.0	0.18	1.778	0.308	3.032	0.466	6	1831	ZINC000033610758
0.8	-7.8	-7.5	0.23	1.527	1.073	2.193	0.771	6	1832	ZINC000006071114
0.8	-6.4	-6.0	0.20	2.859	0.302	4.410	0.569	6	1833	ZINC000001737952
0.8	-8.4	-8.3	0.11	1.738	0.656	4.091	1.231	6	1834	ZINC000096023517
0.8	-7.8	-7.4	0.33	2.155	0.997	2.759	0.929	6	1835	ZINC000006071115
0.8	-7.1	-7.0	0.17	2.005	0.222	3.599	0.461	5	1836	ZINC000014613382
0.8	-10.7	-8.6	1.13	2.151	0.376	4.079	1.678	5	1837	ZINC000014613036
0.8	-7.8	-7.6	0.15	1.491	0.405	2.707	0.455	6	1838	ZINC000100128039

0.8	-7.1	-6.9	0.13	2.798	0.361	5.187	0.068	6	1839	ZINC000013545749
0.8	-9.0	-8.4	0.34	1.774	0.347	4.556	1.036	6	1840	ZINC000100772709
0.8	-8.3	-7.7	0.34	2.707	0.638	3.307	1.126	6	1841	ZINC000000057722
0.8	-7.1	-6.9	0.11	1.356	0.218	3.978	0.585	6	1842	ZINC000008689957
0.8	-9.0	-8.4	0.43	1.922	0.241	3.489	0.447	6	1843	ZINC000139882088
0.8	-6.4	-6.1	0.23	1.919	0.535	3.905	0.406	6	1844	ZINC000002040464
0.8	-8.3	-8.0	0.18	1.631	0.519	3.885	1.160	6	1845	ZINC000000073700
0.8	-8.1	-7.6	0.28	2.258	0.717	3.863	1.491	6	1846	ZINC000014827121
0.8	-8.3	-7.6	0.44	2.936	0.318	6.099	0.301	6	1847	ZINC000014858364
0.8	-6.4	-6.1	0.18	3.362	0.598	4.518	0.722	6	1848	ZINC000095619762
0.8	-6.8	-6.5	0.17	2.102	0.338	4.415	0.625	6	1849	ZINC000006020524
0.8	-6.4	-5.9	0.27	2.304	0.531	3.578	0.721	6	1850	ZINC000005513256
0.8	-8.8	-7.5	0.66	2.021	0.474	4.453	1.997	6	1851	ZINC000257531032
0.8	-10.1	-9.4	0.58	1.444	0.330	4.353	1.599	6	1852	ZINC000004098690
0.8	-6.4	-6.1	0.16	2.204	0.557	4.072	0.982	6	1853	ZINC000002035304

0.8	-8.8	-8.1	0.55	2.014	0.310	4.130	0.669	6	1854	ZINC000006030777
0.8	-8.0	-7.8	0.14	1.582	0.374	3.346	0.726	6	1855	ZINC000038613732
0.8	-9.9	-9.1	0.51	1.636	0.580	3.204	1.124	6	1856	ZINC000256516071
0.8	-6.1	-5.9	0.12	1.894	0.759	3.138	0.863	6	1857	ZINC000100825052
0.8	-8.5	-7.9	0.36	1.714	0.655	3.315	0.547	6	1858	ZINC000014770819
0.8	-7.3	-7.0	0.22	1.831	0.513	3.281	1.174	6	1859	ZINC000015119191
0.8	-8.5	-8.3	0.20	1.643	0.081	2.720	0.656	6	1860	ZINC000013371348
0.8	-7.1	-6.8	0.20	2.526	0.698	4.359	1.376	6	1861	ZINC000015261397
0.8	-7.4	-7.1	0.17	1.710	0.733	4.253	1.044	6	1862	ZINC000299888585
0.8	-7.6	-7.3	0.29	2.045	0.618	4.716	0.667	6	1863	ZINC000020231212
0.8	-9.2	-8.6	0.42	1.893	0.205	3.620	0.774	6	1864	ZINC000014449458
0.8	-7.2	-6.9	0.24	1.791	1.037	3.115	1.351	6	1865	ZINC000000403119
0.8	-8.5	-7.8	0.30	2.798	0.765	5.760	1.460	6	1866	ZINC000001608665
0.8	-8.7	-8.1	0.32	1.952	0.132	4.756	1.047	6	1867	ZINC000015249372
0.8	-10.2	-8.7	0.89	2.138	0.131	4.263	0.621	6	1868	ZINC000004102209

0.8	-9.3	-8.3	0.52	2.612	0.060	5.310	0.246	6	1869	ZINC000050026923
0.8	-7.1	-6.7	0.25	2.025	0.503	3.268	0.516	6	1870	ZINC000004098411
0.8	-7.9	-7.5	0.33	1.699	0.495	3.385	1.427	6	1871	ZINC000004095494
0.8	-8.6	-6.7	1.12	2.680	0.271	5.803	1.906	4	1872	ZINC000021985931
0.8	-8.2	-8.1	0.07	1.473	0.339	3.851	0.406	6	1873	ZINC000085880179
0.8	-7.8	-7.4	0.29	1.790	0.442	2.652	0.349	6	1874	ZINC000000967412
0.8	-9.3	-8.5	0.50	1.572	0.251	5.148	1.214	6	1875	ZINC000257451987
0.8	-6.7	-6.2	0.29	1.618	0.798	3.124	1.165	6	1876	ZINC000002035755
0.8	-7.4	-7.1	0.19	2.888	1.014	4.702	0.892	6	1877	ZINC000002566085
0.8	-8.8	-8.0	0.43	2.864	0.205	4.828	1.537	6	1878	ZINC000095617653
0.8	-8.2	-7.9	0.19	1.807	0.801	4.552	2.211	6	1879	ZINC000030731410
0.8	-6.4	-6.2	0.13	2.731	0.633	3.976	1.058	6	1880	ZINC000005820911
0.8	-8.6	-8.0	0.38	1.826	0.244	3.763	1.622	6	1881	ZINC000014590814
0.8	-9.2	-8.4	0.61	2.060	0.164	3.382	0.170	6	1882	ZINC000015151400
0.8	-9.2	-8.5	0.39	1.478	0.336	4.713	2.084	6	1883	ZINC000257452999

0.8	-6.6	-6.1	0.27	1.697	0.429	3.363	0.894	6	1884	ZINC000014438715
0.8	-7.1	-6.7	0.29	3.514	0.740	5.184	0.524	6	1885	ZINC000095618096
0.8	-9.4	-8.5	0.63	1.664	0.318	4.516	1.519	5	1886	ZINC000085956421
0.8	-8.0	-7.5	0.23	3.248	0.408	4.662	0.922	6	1887	ZINC000000394998
0.8	-6.7	-6.3	0.21	2.152	0.512	3.237	0.740	6	1888	ZINC000005179608
0.8	-6.5	-6.2	0.23	1.809	0.601	3.231	0.701	6	1889	ZINC000000896722
0.8	-6.9	-6.4	0.28	1.789	0.443	3.429	0.964	6	1890	ZINC000014438713
0.8	-7.2	-6.9	0.21	2.195	0.341	4.257	1.305	6	1891	ZINC000257416748
0.8	-8.2	-7.7	0.26	1.760	0.349	3.878	2.011	6	1892	ZINC000040472353
0.8	-8.8	-8.2	0.32	2.034	0.352	4.320	0.649	6	1893	ZINC000015160671
0.8	-8.5	-8.1	0.17	2.049	0.507	4.058	0.455	6	1894	ZINC000013408251
0.8	-8.1	-7.4	0.35	2.634	0.489	4.367	1.156	6	1895	ZINC000002390894
0.8	-5.7	-5.5	0.10	2.524	0.468	3.455	0.602	6	1896	ZINC000005844372
0.8	-8.1	-7.7	0.21	2.153	0.156	4.322	1.052	6	1897	ZINC000095618259
0.8	-8.4	-8.0	0.19	1.611	0.135	3.541	0.972	6	1898	ZINC000100780404

0.8	-7.5	-7.1	0.29	1.772	0.376	3.283	1.584	6	1899	ZINC000001228645
0.8	-9.0	-8.5	0.28	1.121	0.314	2.573	0.192	6	1900	ZINC000100302414
0.8	-9.3	-8.8	0.33	1.840	0.384	3.075	0.674	6	1901	ZINC000014443427
0.8	-7.9	-7.3	0.28	2.768	0.590	3.473	0.483	6	1902	ZINC000002560357
0.8	-9.0	-8.3	0.33	2.809	0.285	4.508	0.956	6	1903	ZINC000001640621
0.8	-8.4	-8.2	0.19	2.081	0.208	5.034	0.722	6	1904	ZINC000027638896
0.8	-9.7	-8.1	0.98	1.727	0.430	3.920	1.437	6	1905	ZINC000257387724
0.8	-7.8	-7.5	0.20	2.252	0.443	3.452	0.454	6	1906	ZINC000014636752
0.8	-9.3	-9.1	0.31	1.480	0.418	4.406	1.900	6	1907	ZINC000015249519
0.8	-7.2	-7.0	0.17	2.827	0.506	4.621	1.519	6	1908	ZINC000050026919
0.8	-7.5	-7.1	0.20	2.220	0.638	3.339	1.256	6	1909	ZINC000001644138
0.8	-9.0	-8.3	0.41	2.272	0.124	4.427	0.446	6	1910	ZINC000100822121
0.8	-8.8	-8.2	0.32	2.362	0.280	5.333	0.821	6	1911	ZINC000001529568
0.8	-6.6	-6.3	0.22	3.807	0.734	5.196	0.858	6	1912	ZINC000002040426
0.8	-8.5	-8.0	0.29	2.154	0.619	3.704	0.647	6	1913	ZINC000013461958

0.8	-6.7	-6.6	0.19	1.421	0.651	3.517	0.715	6	1914	ZINC000000967715
0.8	-8.4	-7.7	0.36	1.879	0.435	3.461	1.431	6	1915	ZINC000040439764
0.8	-9.5	-8.4	0.56	2.382	0.390	4.303	1.225	6	1916	ZINC000015263482
0.8	-6.0	-5.8	0.09	2.322	0.917	4.023	1.099	6	1917	ZINC000001531061
0.8	-6.8	-6.5	0.15	1.648	0.387	3.230	0.361	6	1918	ZINC000005765889
0.8	-6.9	-6.7	0.20	2.228	0.585	3.733	1.070	6	1919	ZINC000002384607
0.8	-5.4	-5.2	0.10	1.803	0.288	2.614	0.456	6	1920	ZINC000034745388
0.8	-8.0	-7.5	0.46	1.196	0.585	2.442	0.730	6	1921	ZINC000004791946
0.8	-8.2	-8.1	0.07	1.819	0.293	3.606	0.915	6	1922	ZINC000015265063
0.8	-10.2	-9.6	0.44	1.764	0.208	5.121	1.948	6	1923	ZINC000014648331
0.8	-7.5	-7.1	0.22	2.437	0.740	4.050	1.456	6	1924	ZINC000095618081
0.8	-8.3	-8.0	0.18	1.503	0.166	3.955	0.584	6	1925	ZINC000257398323
0.8	-7.5	-7.1	0.25	2.057	0.442	2.985	1.611	6	1926	ZINC000004095742
0.8	-6.6	-6.2	0.22	1.482	0.307	2.856	0.209	6	1927	ZINC000001594272
0.8	-5.5	-5.1	0.21	1.604	0.441	2.937	1.376	6	1928	ZINC000002569363

0.8	-6.6	-6.2	0.22	2.361	0.879	3.471	1.193	6	1929	ZINC000014616840
0.8	-9.1	-8.4	0.42	1.528	0.378	3.054	1.771	6	1930	ZINC000014919030
0.8	-11.2	-11.2	0.00	0.000	0.000	0.000	0.000	1	1931	ZINC000257393420
0.8	-7.0	-6.6	0.24	2.479	0.758	3.989	0.606	6	1932	ZINC000095620538
0.8	-8.1	-7.9	0.16	2.423	0.744	4.628	1.338	6	1933	ZINC000014588966
0.8	-8.5	-7.9	0.34	1.764	0.268	4.869	1.624	6	1934	ZINC000004097767
0.8	-9.4	-8.1	0.73	1.866	0.134	3.905	0.904	6	1935	ZINC000100772312
0.8	-9.2	-8.8	0.28	2.059	0.296	5.303	0.471	6	1936	ZINC000014723713
0.8	-7.7	-7.3	0.20	3.082	1.045	4.992	1.926	6	1937	ZINC000004096090
0.8	-6.5	-6.1	0.21	2.124	0.585	3.466	0.563	6	1938	ZINC000000333860
0.8	-6.5	-6.4	0.11	1.823	0.829	4.445	0.583	6	1939	ZINC000001586420
0.8	-8.4	-8.0	0.40	2.134	0.562	4.468	1.878	6	1940	ZINC000034382705
0.8	-7.5	-7.3	0.11	2.360	0.257	5.259	1.063	6	1941	ZINC000002554974
0.8	-7.7	-7.3	0.18	1.517	0.560	2.882	1.300	6	1942	ZINC000003875791
0.8	-8.1	-7.8	0.19	2.524	0.653	4.014	0.439	6	1943	ZINC000001850029

0.8	-5.5	-5.4	0.07	1.443	0.235	3.017	0.522	6	1944	ZINC000002039807
0.8	-6.9	-6.6	0.21	3.431	0.182	5.734	0.697	6	1945	ZINC000002516012
0.8	-6.9	-6.6	0.18	1.893	0.323	3.940	0.457	6	1946	ZINC000004429364
0.8	-8.9	-8.7	0.20	2.185	0.439	4.327	1.285	6	1947	ZINC000095620806
0.8	-10.2	-8.5	1.10	2.349	0.414	5.103	1.381	6	1948	ZINC000253476806
0.8	-8.1	-7.8	0.22	1.681	0.489	4.525	1.653	6	1949	ZINC000015165680
0.8	-7.3	-7.1	0.16	1.968	0.247	3.816	0.115	6	1950	ZINC000000410087
0.8	-9.2	-8.5	0.37	1.728	0.292	4.638	0.425	6	1951	ZINC000019624722
0.8	-7.1	-6.8	0.14	2.202	0.853	3.716	0.578	6	1952	ZINC000033951149
0.8	-5.4	-5.0	0.17	1.554	0.361	3.247	0.655	6	1953	ZINC000002522807
0.8	-8.5	-7.9	0.29	1.934	0.660	3.885	0.728	6	1954	ZINC000013322992
0.8	-8.8	-7.9	0.55	2.057	0.408	5.451	1.079	6	1955	ZINC000013384421
0.8	-8.6	-8.1	0.34	1.388	0.466	4.045	0.534	6	1956	ZINC000038139375
0.8	-7.2	-6.8	0.22	2.903	0.570	4.974	1.432	6	1957	ZINC000015261578
0.8	-8.5	-8.1	0.33	1.540	0.251	3.817	0.397	6	1958	ZINC000059778561

0.8	-7.8	-7.1	0.36	2.864	0.616	4.461	1.163	6	1959	ZINC000014719901
0.8	-6.8	-6.4	0.24	1.960	0.346	3.936	0.680	6	1960	ZINC000014588532
0.8	-9.0	-8.6	0.25	1.071	0.306	2.363	0.333	6	1961	ZINC000100302422
0.8	-7.1	-6.5	0.29	2.190	0.674	3.687	1.259	6	1962	ZINC000006037901
0.8	-9.3	-8.7	0.42	1.809	0.245	3.902	1.264	6	1963	ZINC000257515694
0.8	-6.5	-6.4	0.09	2.251	0.197	4.272	0.460	6	1964	ZINC000005820603
0.8	-7.5	-7.3	0.19	2.092	0.385	4.384	1.457	6	1965	ZINC000034961806
0.8	-8.4	-7.8	0.44	2.412	0.306	4.929	0.253	6	1966	ZINC000040471113
0.8	-7.9	-7.4	0.32	2.388	0.643	4.294	1.739	6	1967	ZINC000015113351
0.8	-8.8	-7.9	0.51	1.881	0.337	3.161	1.014	6	1968	ZINC000033831283
0.8	-5.0	-4.6	0.19	1.869	0.417	3.155	0.282	6	1969	ZINC000031500678
0.8	-9.4	-8.5	0.64	1.610	0.333	4.912	2.053	6	1970	ZINC000014820462
0.8	-9.5	-8.5	0.64	1.709	0.265	4.470	1.758	6	1971	ZINC000014776038
0.8	-7.4	-6.7	0.33	1.836	0.190	3.131	1.006	6	1972	ZINC000004720697
0.8	-10.2	-8.9	0.92	1.471	0.135	4.119	0.525	6	1973	ZINC000014857235

0.8	-6.9	-6.7	0.11	3.497	0.815	5.131	0.883	6	1974	ZINC000050027585
0.8	-5.7	-5.4	0.24	2.328	1.023	3.430	1.161	6	1975	ZINC000000157429
0.8	-8.4	-7.6	0.42	1.614	0.194	4.387	1.177	6	1976	ZINC000012496194
0.8	-7.9	-7.3	0.36	2.392	0.565	4.429	0.343	6	1977	ZINC000014652219
0.8	-8.1	-7.6	0.38	1.768	0.315	4.909	1.326	6	1978	ZINC000005601487
0.8	-7.9	-7.3	0.31	1.737	0.165	4.875	1.672	6	1979	ZINC000013340551
0.8	-7.1	-6.9	0.14	2.585	0.448	3.917	0.561	6	1980	ZINC000039116380
0.8	-6.1	-5.9	0.18	2.091	0.905	3.386	0.860	6	1981	ZINC000012496492
0.8	-9.5	-8.4	0.75	2.203	0.363	3.871	0.912	6	1982	ZINC000001667297
0.8	-9.9	-9.0	0.56	1.890	0.620	3.938	0.752	6	1983	ZINC000015160888
0.8	-7.0	-6.9	0.13	1.881	0.938	3.164	1.823	6	1984	ZINC000014822499
0.8	-6.6	-6.3	0.21	2.821	1.042	4.237	1.685	6	1985	ZINC000034065041
0.8	-7.4	-7.2	0.09	1.913	0.471	4.108	1.557	6	1986	ZINC000014616304
0.8	-8.3	-7.8	0.29	2.141	0.600	2.833	1.127	6	1987	ZINC000001569732
0.8	-9.2	-8.8	0.44	1.939	0.596	4.459	1.977	6	1988	ZINC000006472605

0.8	-6.2	-5.9	0.22	2.071	0.879	3.508	0.735	6	1989	ZINC000001602945
0.8	-8.0	-7.5	0.37	1.665	1.054	3.142	1.344	6	1990	ZINC000100825049
0.8	-10.5	-10.5	0.00	0.000	0.000	0.000	0.000	1	1991	ZINC000100823697
0.8	-10.1	-8.4	1.17	2.061	0.515	3.066	0.624	5	1992	ZINC000005104583
0.8	-7.3	-6.9	0.25	2.453	0.321	4.279	0.265	6	1993	ZINC000001850769
0.8	-8.9	-8.5	0.21	2.469	0.244	4.597	0.485	6	1994	ZINC000095619456
0.8	-8.6	-7.9	0.34	1.631	0.419	4.316	1.435	6	1995	ZINC000014507223
0.8	-7.8	-7.6	0.15	1.485	0.417	2.693	0.461	6	1996	ZINC000100823686
0.8	-9.1	-8.5	0.42	1.514	0.120	3.690	1.397	6	1997	ZINC000257639976
0.8	-5.6	-5.1	0.33	2.351	1.024	3.251	1.125	6	1998	ZINC000001586316
0.8	-9.4	-8.8	0.49	1.458	0.232	3.419	1.390	6	1999	ZINC000257401484
0.8	-7.9	-7.6	0.20	1.407	0.164	3.313	0.994	6	2000	ZINC000085967179
0.8	-7.5	-7.2	0.16	3.245	0.169	5.133	0.480	6	2001	ZINC000003650547
0.8	-7.0	-6.8	0.11	2.603	0.774	4.207	1.280	6	2002	ZINC000013515329
0.8	-7.7	-7.4	0.14	1.410	0.067	5.463	0.778	6	2003	ZINC000040165199

0.8	-9.8	-8.4	0.97	1.561	0.466	4.205	2.222	5	2004	ZINC000015061062
0.8	-7.6	-7.4	0.18	0.844	0.050	2.807	0.297	6	2005	ZINC000059778294
0.8	-6.7	-6.4	0.15	2.626	0.494	4.216	0.696	6	2006	ZINC000004528590
0.8	-8.1	-7.5	0.48	1.780	0.324	3.948	0.565	6	2007	ZINC000006037768
0.8	-9.9	-9.0	0.52	1.213	0.334	4.411	1.970	6	2008	ZINC000005998952
0.8	-8.4	-8.0	0.35	1.408	0.667	5.176	1.838	6	2009	ZINC000015249397
0.8	-10.1	-8.0	1.08	1.739	0.228	3.608	1.255	5	2010	ZINC000257395488
0.8	-6.9	-6.5	0.20	1.934	0.359	3.907	0.499	6	2011	ZINC000004829970
0.8	-7.0	-6.5	0.26	1.938	0.537	2.759	1.205	6	2012	ZINC000040471222
0.8	-10.1	-8.7	0.83	1.804	0.139	4.636	1.148	6	2013	ZINC000257702707
0.8	-8.7	-8.1	0.39	1.763	0.433	4.230	0.355	6	2014	ZINC000003978565
0.8	-6.7	-6.3	0.27	2.210	0.485	3.287	0.987	6	2015	ZINC000242649153
0.8	-8.0	-7.6	0.21	1.739	0.155	4.746	1.292	6	2016	ZINC000040165301
0.8	-6.1	-5.8	0.14	2.299	1.407	4.328	1.277	6	2017	ZINC000000895176
0.8	-8.7	-7.5	0.64	2.037	0.348	4.617	1.504	6	2018	ZINC000014828288

0.8	-7.8	-7.6	0.22	1.916	0.288	3.654	0.595	6	2019	ZINC000014652174
0.8	-7.1	-6.8	0.20	2.631	0.956	3.717	0.805	6	2020	ZINC000000396313
0.8	-9.1	-8.9	0.13	1.597	0.382	2.781	0.779	6	2021	ZINC000013382463
0.8	-9.0	-8.5	0.29	1.035	0.314	2.545	0.181	6	2022	ZINC000100254112
0.8	-9.9	-9.0	0.52	1.790	0.668	3.276	1.023	6	2023	ZINC000015160887
0.8	-7.8	-7.1	0.46	1.788	0.697	3.729	1.709	6	2024	ZINC000014618748
0.8	-8.7	-8.2	0.35	3.102	0.432	4.939	1.273	6	2025	ZINC000255984291
0.8	-7.1	-7.0	0.12	1.756	0.889	4.945	1.308	6	2026	ZINC000000152561
0.8	-6.2	-5.8	0.20	2.368	1.079	3.651	1.021	6	2027	ZINC000014438727
0.8	-10.1	-8.5	1.11	2.036	0.187	4.652	0.380	4	2028	ZINC000013308654
0.8	-8.1	-7.6	0.28	2.575	0.133	4.309	0.532	6	2029	ZINC000002030622
0.8	-7.9	-7.6	0.23	1.629	0.463	3.040	1.349	6	2030	ZINC000014859995
0.8	-9.6	-8.1	0.67	2.282	0.571	5.186	0.378	6	2031	ZINC000003870336
0.8	-8.7	-7.6	0.72	2.568	0.316	5.160	0.848	6	2032	ZINC000008216134
0.8	-8.4	-7.8	0.28	2.342	0.566	4.224	1.259	6	2033	ZINC000004228258

0.8	-6.3	-6.1	0.14	1.227	0.162	3.066	0.556	6	2034	ZINC000100778610
0.8	-8.3	-7.9	0.27	2.216	0.565	4.487	1.418	6	2035	ZINC000001605475
0.8	-7.0	-6.6	0.26	2.446	0.754	4.051	1.445	6	2036	ZINC000013526174
0.8	-7.9	-7.5	0.27	2.049	0.661	3.255	1.685	6	2037	ZINC000003869498
0.8	-7.9	-7.5	0.28	2.031	0.596	3.810	1.067	6	2038	ZINC000013397373
0.8	-7.4	-7.2	0.11	2.390	0.621	4.192	1.033	6	2039	ZINC000001850354
0.8	-6.9	-6.5	0.20	1.917	0.324	3.746	0.545	6	2040	ZINC000004829971
0.8	-7.0	-6.9	0.09	2.026	0.486	3.860	1.929	6	2041	ZINC000095617701
0.8	-7.6	-7.4	0.14	0.841	0.197	2.633	0.442	6	2042	ZINC000100087103
0.8	-7.4	-7.2	0.09	2.786	0.540	4.803	1.168	6	2043	ZINC000095617813
0.8	-6.6	-6.5	0.07	1.820	0.536	3.814	0.997	6	2044	ZINC000005260607
0.8	-9.1	-8.3	0.60	1.739	0.673	4.852	1.153	6	2045	ZINC000005161463
0.8	-7.3	-7.1	0.15	1.406	0.514	3.051	0.572	6	2046	ZINC000014822214
0.8	-9.2	-7.3	1.32	1.824	0.948	4.821	1.784	6	2047	ZINC000013451932
0.8	-7.2	-6.5	0.33	3.938	0.274	5.312	0.344	6	2048	ZINC000004234861

0.8	-9.1	-8.4	0.54	1.909	0.421	4.715	1.731	6	2049	ZINC000014689152
0.8	-6.6	-6.4	0.18	2.243	0.518	3.648	1.080	6	2050	ZINC000004098711
0.8	-9.7	-9.1	0.34	2.099	0.274	5.340	0.493	6	2051	ZINC000095620813
0.8	-6.4	-6.0	0.20	1.748	0.731	3.244	0.869	6	2052	ZINC000013306845
0.8	-7.7	-7.2	0.37	1.740	0.181	4.034	1.495	6	2053	ZINC000001844924
0.8	-8.3	-8.1	0.27	1.793	0.166	3.916	0.692	6	2054	ZINC000015160949
0.8	-7.0	-6.7	0.15	2.734	0.329	3.897	0.485	6	2055	ZINC000000389503
0.8	-8.2	-7.6	0.32	2.201	0.581	4.567	2.159	6	2056	ZINC000015115329
0.8	-7.3	-6.9	0.21	2.666	0.535	3.689	0.546	6	2057	ZINC000000899546
0.8	-6.2	-6.1	0.12	1.683	0.166	3.172	0.904	6	2058	ZINC000003869728
0.8	-8.4	-8.0	0.21	1.803	0.389	4.114	0.463	6	2059	ZINC000000968462
0.8	-6.4	-5.9	0.24	2.337	0.441	3.813	0.586	6	2060	ZINC000002031351
0.8	-9.2	-8.6	0.32	1.961	0.731	4.774	1.367	6	2061	ZINC000036470466
0.8	-6.3	-6.1	0.12	1.300	0.596	2.991	0.438	6	2062	ZINC000001577167
0.8	-10.1	-8.0	1.08	2.829	0.313	5.979	1.488	5	2063	ZINC000257433291

0.8	-6.9	-6.5	0.20	2.249	0.650	3.888	0.466	6	2064	ZINC000008613255
0.8	-7.8	-7.4	0.30	1.558	0.097	4.909	1.195	6	2065	ZINC000032821792
0.8	-7.2	-7.1	0.08	1.877	0.595	3.968	1.004	6	2066	ZINC000015261744
0.8	-7.7	-7.1	0.33	2.369	0.391	4.439	0.595	6	2067	ZINC000014695397
0.8	-8.7	-8.1	0.45	3.454	0.138	5.898	0.297	6	2068	ZINC000008217141
0.8	-6.5	-6.3	0.13	2.809	1.386	4.308	0.477	6	2069	ZINC000095620526
0.8	-10.0	-9.2	0.49	2.068	0.538	4.332	1.312	6	2070	ZINC000014586979
0.8	-9.1	-8.4	0.53	1.841	0.182	4.091	0.979	6	2071	ZINC000257465011
0.8	-10.6	-9.0	0.83	1.531	0.168	4.552	1.360	6	2072	ZINC000000402705
0.8	-9.2	-8.2	0.50	2.043	0.227	4.275	0.414	6	2073	ZINC000015220916
0.8	-9.3	-8.5	0.36	1.658	0.289	3.517	0.553	6	2074	ZINC000001532881
0.8	-7.5	-6.8	0.37	2.447	0.471	3.874	0.743	6	2075	ZINC000002034648
0.8	-6.7	-6.6	0.07	1.624	0.348	3.282	1.258	6	2076	ZINC000015274081
0.8	-8.6	-7.8	0.41	1.863	0.223	5.236	1.134	6	2077	ZINC000014507214
0.8	-6.7	-6.2	0.30	1.496	0.741	3.419	1.036	6	2078	ZINC000002035757

0.8	-6.4	-6.2	0.09	2.273	0.721	4.132	0.731	6	2079	ZINC000001529497
0.8	-6.4	-6.0	0.17	2.420	1.163	3.719	1.158	6	2080	ZINC000001693632
0.8	-9.4	-8.8	0.51	1.512	0.158	3.434	1.447	6	2081	ZINC000015220397
0.8	-7.1	-6.8	0.21	2.436	1.044	3.339	1.887	6	2082	ZINC000001586250
0.8	-8.5	-7.8	0.36	2.746	0.184	5.120	0.084	6	2083	ZINC000034286752
0.8	-9.6	-8.3	0.59	1.837	0.310	4.134	0.946	6	2084	ZINC000015219699
0.8	-7.8	-7.3	0.31	1.861	0.248	3.274	1.657	6	2085	ZINC000014657913
0.8	-9.7	-8.5	0.57	2.295	0.394	4.341	1.232	6	2086	ZINC000033650137
0.8	-8.9	-8.7	0.13	1.318	1.017	3.617	1.132	6	2087	ZINC000000102690
0.8	-7.3	-6.9	0.34	2.389	0.351	4.780	0.448	6	2088	ZINC000003869916
0.8	-6.7	-6.4	0.17	2.312	0.161	3.565	0.717	6	2089	ZINC000004528591
0.8	-7.5	-7.3	0.15	2.893	0.246	5.535	0.324	6	2090	ZINC000100779842
0.8	-7.6	-7.3	0.31	2.434	0.309	5.153	1.230	6	2091	ZINC000085662223
0.8	-6.7	-6.4	0.23	2.471	0.892	3.854	2.241	6	2092	ZINC000005116865
0.8	-9.2	-8.6	0.35	1.212	0.232	2.765	0.658	6	2093	ZINC000002138728

0.8	-7.5	-7.2	0.20	2.246	0.744	4.044	1.151	6	2094	ZINC000002555622
0.8	-7.0	-6.6	0.24	2.806	0.317	4.048	0.656	6	2095	ZINC000033951137
0.8	-9.3	-7.8	0.83	2.269	0.612	5.545	1.428	6	2096	ZINC000014811061
0.8	-9.5	-7.4	0.96	2.201	0.472	4.150	1.102	6	2097	ZINC000100775777
0.8	-9.2	-8.3	0.49	1.521	0.304	4.527	1.476	6	2098	ZINC000100783926
0.8	-9.2	-8.4	0.43	1.722	0.378	4.816	1.222	6	2099	ZINC000100783928
0.8	-7.6	-7.2	0.27	2.107	0.284	4.633	1.715	6	2100	ZINC000003870218
0.8	-7.8	-7.2	0.41	2.327	0.915	2.813	0.941	6	2101	ZINC000006071083
0.8	-8.0	-7.8	0.21	1.885	0.274	3.278	0.555	6	2102	ZINC000033953588
0.8	-6.7	-6.4	0.18	2.539	0.449	4.013	0.772	6	2103	ZINC000242649149
0.8	-6.1	-5.9	0.16	2.467	0.279	3.484	0.527	6	2104	ZINC000032163581
0.8	-7.3	-6.8	0.30	2.416	0.883	4.597	1.847	6	2105	ZINC000033995176
0.8	-7.8	-7.3	0.32	3.165	0.588	4.429	0.960	6	2106	ZINC000005769936
0.8	-7.8	-7.6	0.14	1.489	0.416	2.753	0.370	6	2107	ZINC000100128032
0.8	-8.6	-8.0	0.29	2.249	0.565	4.441	0.419	6	2108	ZINC000085826891

0.8	-9.7	-7.7	1.21	2.856	0.207	5.542	0.090	4	2109	ZINC000014829122
0.8	-7.2	-6.8	0.24	2.428	0.516	3.951	1.112	6	2110	ZINC000003978809
0.8	-7.9	-7.7	0.12	1.126	0.518	2.302	0.283	6	2111	ZINC000000967579
0.8	-6.9	-6.5	0.24	2.180	0.653	3.858	0.464	6	2112	ZINC000004829973
0.8	-7.4	-7.2	0.18	2.933	0.893	4.360	1.040	6	2113	ZINC000000410090
0.8	-9.6	-9.1	0.35	1.845	0.487	3.713	1.728	6	2114	ZINC000014443420
0.8	-6.7	-6.2	0.31	1.525	0.576	3.100	0.483	6	2115	ZINC000100079234
0.8	-5.0	-4.9	0.07	1.801	0.247	2.468	0.722	6	2116	ZINC000043618605
0.8	-7.9	-7.7	0.10	0.952	0.537	2.221	0.368	6	2117	ZINC000000967580
0.8	-8.5	-8.0	0.40	2.272	1.067	3.907	1.801	6	2118	ZINC000035799632
0.8	-8.1	-7.8	0.22	1.470	0.303	4.776	1.549	6	2119	ZINC000040164458
0.8	-7.2	-6.7	0.30	2.513	0.417	4.830	0.178	6	2120	ZINC000013531489
0.8	-7.2	-6.5	0.31	2.007	0.717	4.161	0.105	6	2121	ZINC000001850046
0.8	-8.0	-7.5	0.24	2.216	0.236	4.295	0.947	6	2122	ZINC000013459402
0.8	-9.3	-8.5	0.43	1.311	0.263	3.823	1.357	6	2123	ZINC000085846266

0.8	-8.8	-8.5	0.18	1.940	0.339	4.776	1.601	6	2124	ZINC000013508092
0.8	-9.2	-8.5	0.37	1.927	0.980	3.678	0.979	6	2125	ZINC000100826954
0.8	-8.6	-8.0	0.31	2.893	0.391	5.740	1.311	6	2126	ZINC000000395628
0.8	-7.7	-7.3	0.31	2.098	0.404	3.715	1.419	6	2127	ZINC000001849897
0.8	-9.0	-7.5	1.07	1.869	0.191	4.472	0.558	6	2128	ZINC000014810540
0.8	-8.7	-8.4	0.19	1.907	0.536	4.439	0.437	6	2129	ZINC000070455184
0.8	-7.0	-6.5	0.36	3.163	1.364	4.399	1.420	6	2130	ZINC000032163576
0.8	-8.8	-8.3	0.36	2.179	0.549	3.260	1.643	6	2131	ZINC000014455177
0.8	-10.1	-9.3	0.57	1.565	0.770	4.103	1.668	6	2132	ZINC000086028109
0.8	-6.6	-6.2	0.28	1.805	0.616	3.006	0.882	6	2133	ZINC000001718570
0.8	-6.6	-6.5	0.07	2.016	0.352	3.375	0.558	6	2134	ZINC000012493811
0.8	-9.7	-9.3	0.29	1.753	0.602	3.417	1.440	6	2135	ZINC000001669780
0.8	-8.7	-7.2	0.84	2.041	0.277	4.618	0.670	6	2136	ZINC000014828298
0.8	-8.8	-8.4	0.21	2.001	0.355	4.575	1.335	6	2137	ZINC000100053206
0.8	-7.6	-6.9	0.30	2.301	0.792	3.794	1.196	6	2138	ZINC000095620539

0.8	-7.6	-7.4	0.18	2.289	0.850	3.777	2.276	6	2139	ZINC000034279676
0.8	-7.1	-6.9	0.12	1.866	0.855	4.380	1.409	6	2140	ZINC000014817963
0.8	-6.2	-5.9	0.13	2.343	0.749	3.518	1.069	6	2141	ZINC000012358983
0.8	-6.0	-5.6	0.20	2.332	0.767	3.632	1.036	6	2142	ZINC000029786592
0.8	-8.5	-7.9	0.34	1.952	0.248	5.285	1.279	6	2143	ZINC000013481405
0.8	-7.0	-6.5	0.28	1.366	0.272	2.413	0.307	6	2144	ZINC000000968029
0.8	-9.0	-8.6	0.40	1.974	0.605	3.664	1.139	6	2145	ZINC000003947563
0.8	-8.2	-7.9	0.20	1.767	0.316	3.949	0.878	6	2146	ZINC000014957310
0.8	-8.1	-7.8	0.27	2.553	0.549	4.912	0.385	6	2147	ZINC000013542046
0.8	-6.7	-6.3	0.19	2.504	0.414	3.804	0.948	6	2148	ZINC000004521350
0.8	-6.4	-6.2	0.12	1.806	0.848	3.098	1.217	6	2149	ZINC000085972331
0.8	-9.9	-8.3	0.93	2.197	0.777	4.074	1.546	5	2150	ZINC000252253177
0.8	-8.0	-7.2	0.45	2.417	0.446	4.056	1.777	6	2151	ZINC000014589709
0.8	-7.8	-7.4	0.22	2.563	0.896	4.837	2.218	6	2152	ZINC000005319055
0.8	-9.5	-8.9	0.52	1.641	0.357	5.422	1.843	6	2153	ZINC000000898889

0.8	-7.8	-7.6	0.09	2.102	0.340	4.126	0.445	6	2154	ZINC000085925347
0.8	-7.1	-6.9	0.16	1.925	0.454	3.325	0.519	6	2155	ZINC000008616481
0.8	-7.8	-7.6	0.17	1.619	0.502	3.157	1.423	6	2156	ZINC000038613729
0.8	-8.0	-7.6	0.33	1.726	0.605	4.184	1.359	6	2157	ZINC000008689961
0.8	-6.9	-6.5	0.21	2.304	0.464	3.309	1.180	6	2158	ZINC000012496486
0.8	-5.7	-5.4	0.18	2.094	0.433	3.419	0.275	6	2159	ZINC000000157427
0.8	-7.3	-7.2	0.08	2.760	0.416	4.524	0.808	6	2160	ZINC000040479014
0.8	-7.9	-7.6	0.19	2.328	0.360	4.433	1.083	6	2161	ZINC000100779845
0.8	-7.1	-6.7	0.32	1.693	1.096	2.682	1.210	6	2162	ZINC000000388090
0.8	-6.3	-6.1	0.12	2.609	0.742	3.951	0.729	6	2163	ZINC000001675443
0.8	-8.7	-7.8	0.61	1.548	0.438	4.032	2.149	6	2164	ZINC000014413384
0.8	-7.0	-6.8	0.17	2.644	0.841	3.370	1.051	6	2165	ZINC000005965557
0.8	-7.9	-7.7	0.12	1.134	0.518	2.262	0.262	6	2166	ZINC000100024175
0.8	-8.5	-7.6	0.48	2.044	0.115	4.565	1.478	6	2167	ZINC000033949427
0.8	-8.0	-7.7	0.16	1.813	0.519	3.477	1.686	6	2168	ZINC000005766910

0.8	-8.0	-7.8	0.17	2.651	0.309	4.136	0.797	6	2169	ZINC000013481657
0.8	-6.8	-6.2	0.28	2.389	0.418	3.973	0.731	6	2170	ZINC000005359087
0.8	-7.7	-7.3	0.25	2.022	0.884	3.112	1.388	6	2171	ZINC000000391099
0.8	-8.5	-7.9	0.39	1.759	0.761	3.283	0.557	6	2172	ZINC000014770821
0.8	-8.4	-8.2	0.08	1.958	0.230	4.442	1.114	6	2173	ZINC000015256866
0.8	-7.0	-6.2	0.40	1.827	0.501	3.297	1.432	6	2174	ZINC000002575133
0.8	-8.6	-7.6	0.45	1.789	0.392	4.466	0.736	6	2175	ZINC000013439579
0.8	-5.9	-5.4	0.28	2.365	0.946	3.473	1.002	6	2176	ZINC000002038706
0.8	-8.4	-8.0	0.34	1.748	0.514	3.325	1.281	6	2177	ZINC000000967335
0.8	-7.7	-7.0	0.68	2.162	0.396	3.825	1.237	6	2178	ZINC000014713578
0.8	-9.1	-8.6	0.33	2.595	0.239	4.940	0.791	6	2179	ZINC000004096383
0.8	-6.1	-5.9	0.18	1.754	0.896	3.249	0.522	6	2180	ZINC000000056578
0.8	-5.4	-5.2	0.22	2.186	0.910	3.027	0.959	6	2181	ZINC000001690036
0.8	-8.2	-7.9	0.21	2.351	0.560	5.684	0.787	6	2182	ZINC000004655402
0.8	-8.9	-7.5	0.99	2.261	1.127	5.214	1.656	6	2183	ZINC000014756839

0.8	-10.3	-9.5	0.50	1.885	0.305	4.192	0.971	6	2184	ZINC000257424794
0.8	-7.9	-7.5	0.34	2.081	0.166	3.427	1.254	6	2185	ZINC000001643527
0.8	-9.6	-8.4	1.04	1.497	0.402	2.991	1.790	5	2186	ZINC000014826975
0.8	-10.8	-9.8	0.58	1.928	0.266	5.037	1.420	6	2187	ZINC000257399887
0.8	-8.2	-8.0	0.13	1.429	0.087	3.556	0.706	6	2188	ZINC000083260317
0.8	-8.4	-6.4	1.18	2.361	0.669	5.803	1.636	4	2189	ZINC000014822205
0.8	-9.9	-8.9	0.59	1.862	0.168	6.641	0.402	6	2190	ZINC000005158950
0.8	-7.3	-6.9	0.29	1.508	0.254	2.790	1.339	6	2191	ZINC000001850730
0.8	-7.8	-7.6	0.23	1.509	0.424	2.999	0.507	6	2192	ZINC000014721619
0.8	-7.8	-7.4	0.20	2.330	0.667	5.053	0.971	6	2193	ZINC000004546338
0.8	-8.2	-7.8	0.37	1.542	0.232	3.682	0.544	6	2194	ZINC000257506994
0.8	-7.8	-7.3	0.32	1.855	0.322	3.199	0.397	6	2195	ZINC000014724749
0.8	-6.7	-6.5	0.14	2.276	0.918	2.859	1.329	6	2196	ZINC000005140707
0.8	-9.2	-7.9	0.97	2.403	0.780	5.171	2.258	6	2197	ZINC000014642753
0.8	-8.9	-8.4	0.25	2.004	0.327	3.268	0.407	6	2198	ZINC000015219994

0.8	-10.8	-9.5	0.83	1.992	0.373	5.496	1.180	6	2199	ZINC000257399889
0.8	-8.8	-8.4	0.24	1.971	0.346	5.080	0.319	6	2200	ZINC000004214983
0.8	-7.8	-7.5	0.16	1.471	0.142	2.637	0.768	6	2201	ZINC000014585729
0.8	-5.8	-5.5	0.19	2.054	0.822	3.030	1.024	6	2202	ZINC000001849640
0.8	-10.8	-9.4	0.83	1.893	0.480	6.095	0.351	6	2203	ZINC000104370512
0.8	-7.7	-7.5	0.13	2.012	0.193	6.134	0.377	6	2204	ZINC000032821797
0.8	-7.6	-7.3	0.19	2.276	0.855	3.897	1.264	6	2205	ZINC000003861373
0.8	-7.7	-7.4	0.24	2.046	0.482	3.149	1.626	6	2206	ZINC000095617744
0.8	-7.6	-7.2	0.24	1.811	0.082	4.426	1.263	6	2207	ZINC000071758305
0.8	-9.8	-8.7	0.73	1.668	0.382	4.992	1.729	6	2208	ZINC000014820464
0.8	-8.7	-8.3	0.32	1.585	0.241	5.006	1.768	6	2209	ZINC000257647442
0.8	-8.6	-7.8	0.43	1.932	0.215	4.030	1.235	6	2210	ZINC000100781825
0.8	-9.2	-8.4	0.45	1.472	0.309	5.080	1.671	6	2211	ZINC000100783924
0.8	-9.6	-9.0	0.27	1.639	0.487	4.569	1.154	6	2212	ZINC000013481200
0.8	-7.8	-7.3	0.39	2.171	0.783	3.269	1.752	6	2213	ZINC000001530464

0.8	-9.3	-8.4	0.45	1.867	0.201	5.169	1.049	6	2214	ZINC000005765529
0.8	-8.2	-8.0	0.12	2.313	0.440	4.888	1.591	6	2215	ZINC000012153161
0.8	-9.3	-8.3	0.72	1.961	0.284	5.045	1.466	6	2216	ZINC000005999177
0.8	-8.6	-7.7	0.47	1.928	0.232	4.473	0.809	6	2217	ZINC000001844837
0.8	-8.2	-7.9	0.23	2.516	0.424	4.323	1.673	6	2218	ZINC000004228260
0.8	-6.5	-6.4	0.09	1.966	0.306	4.470	0.999	6	2219	ZINC000095618159
0.8	-7.8	-7.4	0.30	1.894	0.656	3.736	0.795	6	2220	ZINC000014585727
0.8	-9.9	-8.8	0.80	1.871	0.420	4.723	1.627	6	2221	ZINC000035645028
0.8	-6.3	-6.1	0.15	2.416	0.979	3.240	1.322	6	2222	ZINC000039365142
0.8	-8.1	-7.7	0.30	2.087	0.552	3.737	1.383	6	2223	ZINC000014651351
0.8	-6.8	-6.5	0.16	2.614	0.735	3.238	0.882	6	2224	ZINC000100823375
0.8	-6.2	-5.9	0.16	2.202	0.702	3.370	0.692	6	2225	ZINC000002510213
0.8	-9.7	-9.0	0.54	2.358	0.559	4.461	1.812	6	2226	ZINC000000056583
0.8	-6.4	-6.1	0.17	1.354	0.286	3.342	1.267	6	2227	ZINC000003861297
0.8	-8.5	-7.8	0.47	2.155	0.299	4.138	1.546	6	2228	ZINC000005274046

0.8	-8.7	-8.2	0.29	1.643	0.399	4.692	1.811	6	2229	ZINC000014919033
0.8	-6.0	-5.5	0.22	4.716	1.549	6.380	1.323	6	2230	ZINC000002510059
0.8	-6.7	-6.5	0.15	1.861	0.763	2.944	0.940	6	2231	ZINC000002016091
0.8	-8.6	-8.2	0.27	1.925	0.636	4.594	1.650	6	2232	ZINC000015120364
0.8	-6.7	-6.5	0.13	2.630	0.945	3.766	0.991	6	2233	ZINC000014439077
0.8	-10.8	-9.8	0.56	1.926	0.263	5.072	1.448	6	2234	ZINC000257399888
0.8	-7.6	-6.9	0.35	3.176	0.202	4.010	0.425	6	2235	ZINC000004632590
0.8	-8.2	-7.7	0.26	1.986	0.269	3.278	0.855	6	2236	ZINC000015274346
0.8	-10.0	-8.5	0.87	1.768	1.002	3.281	2.029	6	2237	ZINC000005104406
0.8	-7.6	-7.2	0.28	1.382	0.202	2.895	0.228	6	2238	ZINC000100018127
0.8	-8.2	-7.5	0.34	2.783	0.462	5.564	1.280	6	2239	ZINC000002566273
0.8	-6.2	-5.8	0.20	1.867	0.425	3.734	1.234	6	2240	ZINC000021999673
0.8	-10.6	-9.5	0.63	1.808	0.510	3.753	0.984	6	2241	ZINC000100051405
0.8	-8.2	-7.7	0.24	1.756	0.287	2.807	0.711	6	2242	ZINC000015256869
0.8	-7.4	-7.0	0.29	2.106	0.676	3.884	1.023	6	2243	ZINC000005766979

0.8	-9.1	-8.2	0.65	1.738	0.096	3.527	1.249	6	2244	ZINC000013486730
0.8	-6.9	-6.8	0.08	2.773	0.711	5.216	0.789	6	2245	ZINC000083409148
0.8	-6.3	-6.1	0.12	2.694	0.612	3.392	0.797	6	2246	ZINC000000156517
0.8	-6.4	-5.9	0.24	1.705	0.532	2.883	0.648	6	2247	ZINC000001850757
0.8	-6.9	-6.6	0.24	1.183	0.745	4.155	1.791	6	2248	ZINC000001634150
0.8	-8.1	-7.6	0.28	1.491	0.220	3.527	0.667	6	2249	ZINC000100779594
0.8	-10.5	-9.3	0.67	1.654	0.370	3.868	1.358	6	2250	ZINC000014715668
0.8	-6.6	-6.4	0.12	1.830	0.211	3.983	0.838	6	2251	ZINC000004096102
0.8	-7.1	-6.8	0.21	2.472	0.311	4.223	0.691	6	2252	ZINC000014491184
0.8	-6.8	-6.5	0.17	2.160	0.701	4.078	0.494	6	2253	ZINC000002555297
0.8	-6.0	-5.9	0.08	1.654	0.319	2.965	0.859	6	2254	ZINC000001693595
0.8	-8.0	-7.6	0.19	2.527	0.566	4.269	1.518	6	2255	ZINC000005273845
0.8	-7.6	-7.4	0.20	0.880	0.089	2.561	0.596	6	2256	ZINC000100087107
0.8	-8.3	-7.7	0.34	2.185	0.291	4.368	1.571	6	2257	ZINC000014952276
0.8	-10.4	-9.6	0.64	1.800	0.321	5.631	1.403	6	2258	ZINC000005764492

0.8	-6.9	-6.4	0.24	2.248	0.859	3.706	1.653	6	2259	ZINC000040472595
0.8	-7.9	-7.6	0.15	1.964	0.172	5.184	0.719	6	2260	ZINC000012496408
0.8	-6.5	-6.1	0.30	1.958	0.373	3.744	0.771	6	2261	ZINC000014588465
0.8	-6.7	-6.3	0.18	2.747	0.642	4.676	0.925	6	2262	ZINC000015146060
0.8	-8.7	-8.1	0.33	1.290	0.127	2.810	0.597	6	2263	ZINC000015160908
0.8	-8.2	-8.0	0.10	2.214	0.546	3.315	0.605	6	2264	ZINC000001686938
0.8	-6.7	-6.1	0.34	1.566	0.557	3.122	0.481	6	2265	ZINC000100079236
0.8	-8.2	-7.6	0.43	2.277	0.561	4.040	1.413	6	2266	ZINC000014486947
0.8	-8.4	-8.3	0.17	1.707	0.441	4.421	0.464	6	2267	ZINC000100783422
0.8	-9.8	-8.8	0.56	1.509	0.422	4.918	2.205	6	2268	ZINC000006067695
0.8	-8.3	-7.7	0.32	2.202	0.608	4.867	1.586	6	2269	ZINC000040472704
0.8	-7.9	-7.5	0.18	1.543	0.238	3.515	0.635	6	2270	ZINC000014724750
0.8	-9.1	-8.7	0.21	1.911	0.634	4.191	1.710	6	2271	ZINC000006093290
0.8	-7.4	-7.0	0.28	2.470	0.561	3.591	0.662	6	2272	ZINC000014613369
0.8	-8.9	-8.7	0.13	2.075	0.091	4.353	0.563	6	2273	ZINC000013381475

0.8	-6.0	-5.8	0.13	2.055	0.472	3.330	1.173	6	2274	ZINC000002446417
0.8	-6.9	-6.5	0.22	2.910	1.132	4.131	1.704	6	2275	ZINC000095618100
0.8	-7.3	-6.9	0.31	2.676	0.625	4.949	1.213	6	2276	ZINC000050027581
0.8	-10.1	-9.3	0.58	1.384	0.353	4.137	1.584	6	2277	ZINC000230084367
0.8	-9.9	-9.3	0.34	1.530	0.613	3.372	1.197	6	2278	ZINC000256516075
0.8	-8.0	-7.7	0.25	3.035	0.563	4.422	1.554	6	2279	ZINC000018286013
0.8	-9.3	-9.2	0.13	1.631	0.468	4.042	2.062	6	2280	ZINC000014919086
0.8	-7.3	-7.0	0.16	2.482	0.240	3.949	0.370	6	2281	ZINC000014491186
0.8	-6.3	-5.9	0.20	2.076	0.968	3.027	1.113	6	2282	ZINC000002572237
0.8	-6.4	-6.2	0.12	2.205	0.579	4.123	1.087	6	2283	ZINC000001529540
0.8	-10.0	-8.8	0.81	1.739	0.258	5.012	1.130	6	2284	ZINC000085615625
0.8	-9.2	-8.7	0.52	1.833	0.902	5.035	1.539	6	2285	ZINC000022061255
0.8	-7.5	-7.3	0.13	2.830	0.752	4.361	1.126	6	2286	ZINC000035259491
0.8	-6.9	-6.5	0.21	2.032	0.572	3.539	0.999	6	2287	ZINC000000967595
0.8	-7.6	-7.3	0.31	2.177	0.177	3.737	1.047	6	2288	ZINC000005706883

0.8	-7.9	-7.3	0.34	1.971	0.179	3.914	0.620	6	2289	ZINC000100783424
0.8	-10.4	-9.1	0.71	2.005	0.223	5.004	1.100	6	2290	ZINC000257617627
0.8	-9.5	-8.0	0.86	1.618	0.193	4.522	1.574	6	2291	ZINC000100780639
0.8	-8.3	-7.9	0.25	2.345	0.338	3.768	0.771	6	2292	ZINC000095617664
0.8	-8.4	-7.9	0.29	1.763	0.392	3.797	1.628	6	2293	ZINC000095617971
0.8	-8.6	-8.3	0.19	1.829	0.295	4.263	0.364	6	2294	ZINC000015160951
0.8	-7.2	-7.0	0.14	1.575	0.156	3.413	0.275	6	2295	ZINC000013829462
0.8	-8.3	-7.8	0.26	2.357	0.398	3.596	0.429	6	2296	ZINC000014619020
0.8	-7.3	-7.0	0.17	2.408	0.573	3.797	0.668	6	2297	ZINC000014917021
0.8	-10.7	-9.6	0.75	1.782	0.149	4.513	1.391	3	2298	ZINC000013302609
0.8	-7.6	-7.5	0.07	1.718	0.278	3.480	0.450	6	2299	ZINC000085925336
0.8	-7.7	-7.3	0.23	2.323	0.550	3.786	0.477	6	2300	ZINC000021989120
0.8	-5.5	-5.3	0.14	2.213	0.944	3.799	1.160	6	2301	ZINC000001675676
0.8	-7.7	-7.1	0.27	2.335	0.408	4.948	0.390	6	2302	ZINC000034410584
0.8	-9.5	-9.0	0.38	1.754	0.331	5.512	1.822	6	2303	ZINC000015271738

0.8	-7.8	-7.2	0.37	2.154	0.397	3.183	1.059	6	2304	ZINC000000901363
0.8	-6.9	-6.7	0.15	1.634	0.160	2.963	0.808	6	2305	ZINC000001850048
0.8	-9.3	-8.8	0.30	1.824	0.457	3.413	0.943	6	2306	ZINC000251666744
0.8	-8.1	-7.4	0.39	1.565	0.150	3.646	1.144	6	2307	ZINC000100776454
0.8	-8.5	-8.0	0.27	2.420	0.508	4.380	0.549	6	2308	ZINC000100824245
0.8	-8.8	-8.3	0.47	2.104	0.467	3.769	1.479	6	2309	ZINC000097759147
0.8	-9.2	-8.3	0.43	2.243	0.459	5.788	0.977	6	2310	ZINC000000896041
0.8	-6.2	-6.1	0.16	2.202	0.440	4.504	0.345	6	2311	ZINC000261499646
0.8	-8.5	-7.9	0.40	1.798	0.231	2.579	0.226	6	2312	ZINC000005425259
0.8	-7.1	-6.9	0.11	2.608	0.544	4.191	0.717	6	2313	ZINC000012493681
0.8	-9.8	-9.4	0.30	2.394	0.536	4.407	1.361	6	2314	ZINC000013380527
0.8	-8.7	-7.8	0.43	2.099	0.073	5.425	1.085	6	2315	ZINC000012495519
0.8	-7.4	-6.8	0.40	1.825	0.495	3.266	1.025	6	2316	ZINC000252370200
0.8	-8.0	-6.9	0.51	2.683	0.469	4.448	1.135	6	2317	ZINC000013538216
0.7	-9.8	-9.0	0.55	1.918	0.536	3.573	0.581	6	2318	ZINC000256516066

0.7	-10.1	-8.7	0.87	1.580	0.510	3.738	1.477	6	2319	ZINC000100780627
0.7	-7.2	-7.1	0.07	1.817	0.596	2.760	0.547	6	2320	ZINC000001849752
0.7	-6.1	-5.9	0.15	3.499	1.232	5.147	1.966	6	2321	ZINC000002509716
0.7	-6.2	-5.8	0.29	1.970	0.756	2.475	0.999	6	2322	ZINC000032163585
0.7	-10.3	-8.6	0.84	1.799	0.331	5.210	0.786	6	2323	ZINC000006017711
0.7	-9.0	-8.7	0.18	1.689	0.748	2.861	0.983	6	2324	ZINC000104367278
0.7	-5.0	-4.8	0.12	2.150	0.412	2.924	0.549	6	2325	ZINC000002509980
0.7	-8.9	-8.7	0.15	1.395	0.330	3.345	1.091	6	2326	ZINC000100193759
0.7	-9.4	-8.5	0.47	1.379	0.117	3.332	0.422	6	2327	ZINC000001599733
0.7	-5.7	-5.4	0.15	2.072	1.007	3.021	1.007	6	2328	ZINC000001648312
0.7	-9.0	-8.7	0.21	1.690	0.761	2.812	1.048	6	2329	ZINC000100778709
0.7	-6.7	-6.5	0.11	2.322	0.324	3.749	0.613	6	2330	ZINC000005419310
0.7	-8.1	-7.8	0.28	2.349	0.768	3.657	1.532	6	2331	ZINC000100822509
0.7	-9.4	-8.1	1.11	1.404	0.296	5.362	1.585	5	2332	ZINC000012493502
0.7	-6.0	-5.7	0.14	2.903	0.653	3.847	0.860	6	2333	ZINC000002510285

0.7	-9.2	-8.6	0.43	2.188	0.748	4.954	1.149	6	2334	ZINC000085700578
0.7	-7.2	-6.9	0.15	2.641	0.492	3.572	0.800	6	2335	ZINC000000409284
0.7	-8.9	-8.5	0.28	1.693	0.299	4.705	0.606	6	2336	ZINC000013383921
0.7	-7.9	-7.5	0.26	2.422	0.685	4.514	0.708	6	2337	ZINC000205770902
0.7	-8.1	-7.7	0.50	2.485	0.657	4.862	1.165	6	2338	ZINC000014858365
0.7	-9.6	-7.7	1.13	1.504	0.382	4.765	2.304	4	2339	ZINC000257777844
0.7	-8.1	-7.9	0.11	2.169	0.273	3.982	0.579	6	2340	ZINC000095617657
0.7	-7.9	-7.4	0.28	1.815	0.390	3.077	1.224	6	2341	ZINC000035898717
0.7	-6.1	-5.9	0.12	2.399	0.277	3.970	0.449	6	2342	ZINC000001531619
0.7	-6.0	-5.8	0.12	2.901	0.947	4.084	1.481	6	2343	ZINC000032155223
0.7	-6.8	-6.4	0.21	2.457	0.904	3.648	1.773	6	2344	ZINC000095618097
0.7	-7.8	-7.6	0.12	2.831	1.106	4.674	1.414	6	2345	ZINC000000901356
0.7	-9.8	-8.5	1.32	1.978	0.540	3.908	1.155	5	2346	ZINC000013302911
0.7	-8.4	-8.1	0.20	1.461	0.112	3.990	0.483	6	2347	ZINC000005762533
0.7	-8.3	-7.9	0.24	1.960	0.662	4.003	1.556	6	2348	ZINC000002115314

0.7	-9.4	-8.5	0.49	1.532	0.115	4.996	1.203	6	2349	ZINC000014814194
0.7	-8.0	-7.8	0.21	2.087	0.402	5.064	0.457	6	2350	ZINC000033954298
0.7	-7.5	-7.4	0.15	2.247	0.240	4.961	1.024	6	2351	ZINC000015120892
0.7	-7.1	-6.7	0.24	2.552	0.440	4.631	1.536	6	2352	ZINC000050026849
0.7	-7.1	-6.8	0.16	3.145	0.290	4.448	0.362	6	2353	ZINC000003869576
0.7	-9.2	-8.5	0.40	1.986	0.773	4.181	1.706	6	2354	ZINC000014728223
0.7	-6.8	-6.4	0.21	1.963	0.298	2.587	0.485	6	2355	ZINC000033798297
0.7	-8.9	-8.2	0.40	2.585	0.851	4.022	1.891	6	2356	ZINC000005439110
0.7	-7.5	-7.4	0.09	1.866	0.845	4.453	1.027	6	2357	ZINC000013397381
0.7	-7.0	-6.6	0.22	1.974	0.518	3.389	1.435	6	2358	ZINC000000389873
0.7	-7.0	-6.5	0.23	1.862	0.082	4.657	0.848	6	2359	ZINC000100772468
0.7	-7.9	-7.6	0.15	2.777	0.560	4.991	1.580	6	2360	ZINC000014859826
0.7	-9.8	-8.9	0.55	1.515	0.387	4.452	1.567	6	2361	ZINC000100824256
0.7	-9.2	-8.6	0.45	2.165	0.765	4.977	1.126	6	2362	ZINC000257403775
0.7	-8.6	-8.2	0.33	1.599	0.374	3.066	0.708	6	2363	ZINC000100782246

0.7	-7.4	-6.8	0.33	2.870	0.402	3.743	0.194	6	2364	ZINC000000388112
0.7	-9.2	-8.5	0.45	1.707	0.236	3.210	1.544	6	2365	ZINC000100783061
0.7	-7.9	-7.5	0.19	1.564	0.731	3.821	1.021	6	2366	ZINC000004474603
0.7	-6.1	-5.8	0.21	1.922	0.595	3.626	1.162	6	2367	ZINC000032166984
0.7	-8.8	-8.5	0.30	1.688	0.349	3.211	1.357	6	2368	ZINC000005352269
0.7	-8.7	-7.6	0.63	2.520	0.295	3.734	1.086	6	2369	ZINC000014713562
0.7	-8.1	-8.0	0.15	2.625	0.479	3.854	0.833	6	2370	ZINC000015120005
0.7	-9.5	-7.8	0.90	2.212	0.407	3.938	1.783	6	2371	ZINC000095617850
0.7	-9.5	-8.8	0.47	2.083	0.314	3.959	1.048	6	2372	ZINC000015054822
0.7	-8.6	-8.1	0.42	2.428	0.785	4.123	1.686	6	2373	ZINC000004095845
0.7	-6.3	-6.0	0.16	2.699	1.267	3.676	1.345	6	2374	ZINC000012153446
0.7	-6.4	-6.0	0.21	1.473	0.232	3.844	0.345	6	2375	ZINC000005513417
0.7	-7.8	-7.2	0.33	1.920	0.221	5.493	0.773	6	2376	ZINC000100770154
0.7	-7.1	-7.0	0.09	2.681	0.138	4.272	0.823	6	2377	ZINC000004533738
0.7	-7.5	-7.2	0.20	1.066	0.520	2.744	0.631	6	2378	ZINC000261496102

0.7	-6.3	-6.0	0.26	2.198	0.401	3.203	1.359	6	2379	ZINC000002389359
0.7	-5.4	-5.1	0.19	1.850	0.604	3.024	0.871	6	2380	ZINC000038609494
0.7	-9.9	-9.4	0.46	2.271	0.440	4.091	0.743	6	2381	ZINC000001611775
0.7	-7.9	-7.4	0.26	2.033	0.383	3.733	1.766	6	2382	ZINC000008461972
0.7	-6.3	-6.0	0.17	2.523	0.710	4.439	0.684	6	2383	ZINC000013433426
0.7	-6.1	-5.9	0.16	2.537	0.982	3.984	1.086	6	2384	ZINC000001850428
0.7	-8.5	-8.0	0.38	1.795	0.646	2.532	0.515	6	2385	ZINC000100099468
0.7	-6.2	-6.0	0.14	2.379	0.313	4.409	0.783	6	2386	ZINC000095618190
0.7	-7.5	-7.3	0.12	2.312	0.262	4.891	0.579	6	2387	ZINC000014616314
0.7	-5.9	-5.8	0.08	2.517	0.265	4.068	0.245	6	2388	ZINC000032180834
0.7	-7.0	-6.7	0.27	2.771	0.349	4.857	0.324	6	2389	ZINC000014822496
0.7	-9.8	-8.8	0.67	1.541	0.512	4.807	2.192	6	2390	ZINC000006067693
0.7	-9.6	-7.8	1.27	1.742	0.757	2.949	1.809	3	2391	ZINC000015210025
0.7	-9.0	-8.7	0.21	1.664	0.805	3.070	0.948	6	2392	ZINC000100778713
0.7	-6.7	-6.0	0.32	3.315	1.115	4.574	0.952	6	2393	ZINC000032163586

0.7	-8.5	-8.0	0.30	2.341	0.920	4.636	0.578	6	2394	ZINC000000000076
0.7	-9.2	-8.2	0.53	1.899	0.261	4.890	1.012	6	2395	ZINC000014757320
0.7	-8.6	-7.8	0.46	2.318	0.280	4.344	1.187	6	2396	ZINC000085951537
0.7	-10.0	-9.4	0.36	1.132	0.582	3.276	0.690	6	2397	ZINC000257625697
0.7	-6.4	-6.0	0.20	2.833	1.094	4.075	1.062	6	2398	ZINC000001693633
0.7	-9.9	-9.1	0.62	1.743	0.618	4.494	1.687	6	2399	ZINC000014778674
0.7	-6.2	-6.0	0.12	2.321	0.967	3.515	1.380	6	2400	ZINC000100776157
0.7	-8.4	-8.2	0.17	2.263	0.470	4.686	0.565	6	2401	ZINC000013399012
0.7	-10.1	-8.6	0.84	1.987	0.288	6.658	0.209	4	2402	ZINC000004097840
0.7	-7.7	-7.4	0.19	2.619	0.346	4.109	1.135	6	2403	ZINC000025725624
0.7	-8.1	-7.8	0.21	2.906	0.739	4.362	1.435	6	2404	ZINC000100028524
0.7	-7.4	-6.7	0.32	2.101	0.282	3.867	0.790	6	2405	ZINC000014487668
0.7	-8.8	-8.2	0.44	2.035	0.319	3.943	1.595	6	2406	ZINC000257621361
0.7	-7.8	-7.4	0.26	2.190	0.417	3.905	1.170	6	2407	ZINC000095617659
0.7	-9.6	-8.9	0.36	2.389	0.294	4.282	0.603	6	2408	ZINC000018038211

0.7	-7.6	-7.3	0.16	2.867	0.480	3.795	0.619	6	2409	ZINC000000058111
0.7	-8.0	-7.5	0.35	2.003	0.381	5.231	1.554	6	2410	ZINC000004095589
0.7	-7.7	-7.1	0.42	1.843	0.344	2.542	0.575	6	2411	ZINC000006024682
0.7	-7.6	-7.3	0.23	2.081	0.565	4.016	1.260	6	2412	ZINC000008860475
0.7	-8.6	-8.1	0.38	1.948	0.462	4.128	1.076	6	2413	ZINC000014687445
0.7	-10.8	-9.7	0.93	2.051	0.678	6.086	0.286	6	2414	ZINC000257456807
0.7	-5.6	-5.4	0.12	1.498	0.780	3.015	0.273	6	2415	ZINC000000409293
0.7	-7.4	-7.0	0.26	2.416	0.681	3.637	1.143	6	2416	ZINC000003869580
0.7	-8.5	-8.0	0.40	1.789	0.564	2.781	0.452	6	2417	ZINC000100684054
0.7	-6.3	-6.0	0.15	2.742	0.741	3.998	0.635	6	2418	ZINC000095619761
0.7	-11.1	-10.0	0.79	1.905	0.357	4.499	1.337	6	2419	ZINC000100774351
0.7	-7.4	-7.1	0.21	2.452	0.471	3.137	0.880	6	2420	ZINC000000006556
0.7	-6.7	-6.5	0.10	2.518	0.188	4.378	0.782	6	2421	ZINC000006360467
0.7	-6.5	-6.2	0.16	2.297	0.260	3.182	0.791	6	2422	ZINC000001575508
0.7	-5.4	-5.1	0.19	2.017	0.797	3.251	0.938	6	2423	ZINC000002034880

0.7	-5.6	-5.3	0.26	2.041	0.821	3.220	1.143	6	2424	ZINC000005225061
0.7	-6.2	-6.0	0.14	1.314	0.632	2.719	0.466	6	2425	ZINC000019735105
0.7	-8.3	-7.9	0.35	2.646	0.555	4.016	1.487	6	2426	ZINC000082304985
0.7	-10.0	-9.4	0.35	0.880	0.186	3.489	0.639	6	2427	ZINC000100781033
0.7	-7.0	-6.7	0.21	1.979	0.305	2.939	1.121	6	2428	ZINC000000402766
0.7	-9.8	-8.8	0.63	1.694	0.389	4.266	1.357	6	2429	ZINC000230084481
0.7	-7.6	-7.1	0.31	1.985	0.391	3.266	1.562	6	2430	ZINC000006024697
0.7	-8.9	-7.8	0.66	2.092	0.727	4.011	1.858	6	2431	ZINC000014811619
0.7	-9.3	-8.6	0.52	1.924	0.347	5.102	1.657	6	2432	ZINC000002558134
0.7	-8.2	-7.7	0.23	1.915	0.318	4.778	1.347	6	2433	ZINC000257426780
0.7	-8.4	-7.8	0.30	2.078	0.355	3.781	0.805	6	2434	ZINC000014827119
0.7	-6.9	-6.5	0.25	1.831	0.356	3.647	1.564	6	2435	ZINC000001591038
0.7	-8.5	-8.2	0.26	2.194	0.418	4.444	1.292	6	2436	ZINC000013549753
0.7	-8.0	-7.8	0.15	1.823	0.323	3.967	0.743	6	2437	ZINC000257547804
0.7	-6.5	-6.0	0.38	2.916	0.464	4.391	0.884	6	2438	ZINC000001575509

0.7	-7.9	-7.6	0.17	2.459	1.228	4.474	0.406	6	2439	ZINC000000968250
0.7	-7.7	-7.4	0.19	2.173	0.692	3.796	0.677	6	2440	ZINC000008616483
0.7	-6.5	-6.1	0.26	1.679	0.426	2.761	0.859	6	2441	ZINC000002030901
0.7	-7.8	-7.4	0.21	2.071	0.407	4.305	1.373	6	2442	ZINC000095620853
0.7	-7.5	-7.4	0.13	2.606	0.536	4.948	1.535	6	2443	ZINC000014684217
0.7	-9.6	-8.4	1.03	1.538	0.248	4.417	1.390	6	2444	ZINC000014615084
0.7	-8.0	-7.8	0.13	1.982	0.334	4.620	0.977	6	2445	ZINC000005706877
0.7	-6.7	-6.4	0.17	2.184	0.596	4.348	0.339	6	2446	ZINC000001640853
0.7	-7.9	-7.8	0.15	1.504	0.137	3.455	0.798	6	2447	ZINC000012494625
0.7	-6.3	-5.9	0.21	1.714	0.709	2.383	0.898	6	2448	ZINC000001680828
0.7	-9.4	-8.5	0.44	2.037	0.207	5.402	0.593	6	2449	ZINC000001684139
0.7	-10.2	-8.4	0.84	2.799	0.594	4.854	1.417	6	2450	ZINC000002561253
0.7	-6.8	-6.3	0.32	3.096	0.778	4.910	1.062	6	2451	ZINC000095618094
0.7	-9.1	-8.5	0.31	2.015	0.394	4.752	1.154	6	2452	ZINC000014774933
0.7	-9.2	-8.7	0.44	2.164	0.301	3.367	0.686	6	2453	ZINC000015220977

0.7	-6.5	-6.4	0.09	1.633	0.179	3.854	1.311	6	2454	ZINC000095619616
0.7	-8.2	-7.8	0.21	1.452	0.295	4.257	0.699	6	2455	ZINC000000314951
0.7	-7.4	-7.2	0.11	2.465	0.514	5.500	1.247	6	2456	ZINC000095617688
0.7	-7.3	-6.6	0.45	2.769	0.427	5.293	1.150	6	2457	ZINC000005663064
0.7	-6.6	-6.1	0.27	1.623	0.447	3.846	1.028	6	2458	ZINC000014438721
0.7	-7.9	-7.6	0.21	2.133	0.473	4.006	0.833	6	2459	ZINC000001731789
0.7	-6.1	-5.8	0.17	1.923	0.168	4.109	0.333	6	2460	ZINC000100828978
0.7	-7.3	-6.7	0.35	2.089	0.610	3.276	1.403	6	2461	ZINC000004556663
0.7	-7.1	-6.6	0.32	1.653	0.434	2.715	0.798	6	2462	ZINC000001850419
0.7	-10.8	-10.0	0.75	1.650	0.214	4.717	1.697	5	2463	ZINC000257456804
0.7	-7.1	-6.7	0.23	2.529	0.291	4.695	0.371	6	2464	ZINC000050027208
0.7	-6.7	-6.5	0.09	2.736	0.781	5.570	0.461	6	2465	ZINC000012493817
0.7	-8.1	-7.9	0.17	1.749	0.097	3.261	0.491	6	2466	ZINC000014616541
0.7	-9.8	-8.1	1.11	1.990	0.398	5.732	0.718	6	2467	ZINC000002526197
0.7	-7.8	-7.7	0.11	0.761	0.105	3.338	0.278	6	2468	ZINC000001666852

0.7	-7.3	-7.2	0.08	1.719	0.870	3.315	0.785	6	2469	ZINC000013545758
0.7	-8.3	-8.0	0.29	2.095	0.872	3.415	1.542	6	2470	ZINC000001579641
0.7	-10.3	-9.2	0.87	1.732	0.578	4.649	2.525	6	2471	ZINC000100780822
0.7	-6.3	-6.0	0.27	1.426	0.243	2.084	0.321	6	2472	ZINC000006660884
0.7	-7.6	-7.4	0.15	1.790	0.249	4.923	1.000	6	2473	ZINC000014449825
0.7	-9.8	-8.8	0.65	1.922	0.873	4.049	1.540	6	2474	ZINC000257527008
0.7	-7.0	-6.8	0.11	2.452	0.685	3.700	0.908	6	2475	ZINC000013536561
0.7	-8.9	-8.7	0.17	1.484	0.352	3.428	0.966	6	2476	ZINC000100193754
0.7	-8.2	-6.7	0.79	2.171	0.838	4.805	1.890	6	2477	ZINC000014720693
0.7	-9.1	-8.1	0.69	1.789	0.837	3.329	1.445	6	2478	ZINC000085994610
0.7	-7.6	-7.2	0.20	1.825	0.220	4.821	1.368	6	2479	ZINC000071758298
0.7	-7.7	-7.1	0.32	1.668	0.277	3.569	1.467	6	2480	ZINC000006069801
0.7	-9.4	-8.4	0.51	1.622	0.196	4.323	1.274	6	2481	ZINC000014919098
0.7	-6.2	-5.7	0.24	2.963	0.518	4.305	0.654	6	2482	ZINC000032163574
0.7	-7.4	-7.3	0.07	2.091	0.141	5.048	0.307	6	2483	ZINC000001850291

0.7	-7.4	-7.1	0.16	1.611	0.553	4.194	1.404	6	2484	ZINC000257557765
0.7	-9.0	-8.1	0.73	1.828	1.035	3.346	1.947	6	2485	ZINC000014444063
0.7	-9.8	-8.4	0.95	1.724	0.346	4.234	1.784	6	2486	ZINC000100777064
0.7	-6.7	-6.2	0.23	2.100	0.889	3.513	1.047	6	2487	ZINC000004283873
0.7	-7.9	-7.1	0.50	1.818	0.490	2.612	0.408	6	2488	ZINC000000083665
0.7	-7.0	-6.9	0.10	1.915	0.255	3.126	0.550	6	2489	ZINC000257521760
0.7	-7.6	-7.5	0.09	2.485	0.752	4.141	1.683	6	2490	ZINC000022060236
0.7	-7.9	-7.6	0.16	2.094	0.652	4.013	0.961	6	2491	ZINC000014708247
0.7	-9.0	-7.9	1.04	3.501	0.331	5.535	1.298	4	2492	ZINC000100028707
0.7	-7.0	-6.8	0.12	3.419	0.470	4.643	0.549	6	2493	ZINC000000392004
0.7	-5.7	-5.5	0.14	1.645	0.624	2.440	0.732	6	2494	ZINC000001683674
0.7	-6.8	-6.3	0.27	1.976	0.374	3.561	1.015	6	2495	ZINC000014588531
0.7	-8.6	-7.9	0.37	1.821	0.305	5.696	0.618	6	2496	ZINC000257507769
0.7	-7.5	-7.2	0.19	1.681	0.290	4.435	1.152	6	2497	ZINC000014592862
0.7	-8.4	-7.7	0.40	1.905	0.351	4.691	2.050	6	2498	ZINC000014855570

0.7	-7.8	-7.3	0.28	1.546	0.184	3.788	0.960	6	2499	ZINC000015169307
0.7	-7.1	-6.5	0.29	2.606	1.200	3.943	1.806	6	2500	ZINC000038673530
0.7	-6.9	-6.6	0.18	1.881	0.849	2.677	0.455	6	2501	ZINC000000154698
0.7	-8.4	-7.8	0.30	1.645	0.442	3.887	0.448	6	2502	ZINC000100781844
0.7	-6.2	-5.9	0.15	2.887	0.735	4.138	0.316	6	2503	ZINC000002003741
0.7	-6.5	-6.4	0.05	2.408	0.708	4.913	0.638	6	2504	ZINC000003860440
0.7	-9.6	-9.0	0.39	1.950	0.394	5.245	0.533	6	2505	ZINC000095620787
0.7	-8.6	-8.2	0.21	2.648	0.523	4.212	1.730	6	2506	ZINC000000895903
0.7	-7.3	-7.2	0.08	1.632	0.231	4.820	2.182	6	2507	ZINC000005888062
0.7	-7.8	-7.6	0.17	2.233	0.632	4.348	1.304	6	2508	ZINC000013460734
0.7	-9.6	-8.2	0.74	1.741	0.345	2.974	0.713	6	2509	ZINC000014927463
0.7	-9.6	-8.6	0.71	1.109	0.211	6.231	1.764	6	2510	ZINC000003881360
0.7	-8.8	-8.2	0.32	1.339	0.264	2.819	0.794	6	2511	ZINC000015115282
0.7	-8.5	-7.8	0.40	2.223	0.564	3.604	0.955	6	2512	ZINC000238772098
0.7	-7.8	-7.7	0.10	1.481	0.209	3.582	0.374	6	2513	ZINC000038634839

0.7	-7.7	-7.5	0.21	1.886	0.154	3.324	0.199	6	2514	ZINC000100779846
0.7	-5.9	-5.6	0.12	2.035	0.327	3.211	0.866	6	2515	ZINC000032166960
0.7	-5.9	-5.6	0.16	2.452	0.677	4.338	0.548	6	2516	ZINC000002539248
0.7	-7.4	-7.1	0.20	2.109	0.222	3.086	0.737	6	2517	ZINC000005273746
0.7	-6.5	-6.3	0.15	1.397	0.339	3.086	0.478	6	2518	ZINC000002040513
0.7	-8.5	-8.2	0.23	1.435	0.140	2.974	0.380	6	2519	ZINC000015120362
0.7	-8.8	-8.0	0.41	2.150	0.538	4.371	1.897	6	2520	ZINC000027646101
0.7	-9.3	-8.8	0.37	2.704	0.266	4.405	1.041	6	2521	ZINC000004096380
0.7	-6.9	-6.6	0.20	2.623	0.589	4.764	0.380	6	2522	ZINC000012495653
0.7	-9.3	-8.8	0.27	2.424	0.664	4.907	1.533	6	2523	ZINC000002575152
0.7	-8.0	-7.6	0.31	1.546	0.642	4.661	1.779	6	2524	ZINC000014709493
0.7	-8.7	-8.1	0.45	2.499	0.975	4.783	0.246	6	2525	ZINC000014822244
0.7	-7.2	-6.9	0.15	2.281	0.460	3.943	1.446	6	2526	ZINC000004533994
0.7	-8.8	-7.8	0.46	2.487	0.745	4.804	1.433	6	2527	ZINC000004095542
0.7	-8.3	-7.8	0.32	1.808	0.374	4.685	0.947	6	2528	ZINC000085954092

0.7	-7.7	-7.3	0.21	1.650	0.325	3.653	0.876	6	2529	ZINC000005103853
0.7	-6.8	-6.3	0.33	1.735	0.953	2.866	1.210	6	2530	ZINC000001848437
0.7	-7.4	-7.0	0.22	2.283	0.675	3.056	0.694	6	2531	ZINC000005934751
0.7	-9.0	-8.7	0.24	1.990	0.522	3.382	1.187	6	2532	ZINC000069486111
0.7	-6.1	-5.9	0.15	1.743	0.267	3.828	0.393	6	2533	ZINC000095620000
0.7	-8.7	-7.0	1.19	1.864	0.261	4.345	2.126	6	2534	ZINC000014642991
0.7	-7.5	-7.1	0.30	1.829	0.651	2.813	0.887	6	2535	ZINC000003683317
0.7	-8.6	-8.1	0.36	1.147	0.483	4.450	1.780	6	2536	ZINC000005999087
0.7	-5.7	-5.5	0.12	1.627	0.370	2.673	0.456	6	2537	ZINC000001850693
0.7	-9.2	-8.5	0.50	2.175	0.754	5.014	1.140	6	2538	ZINC000100777199
0.7	-7.4	-7.0	0.24	1.862	0.299	2.951	0.799	6	2539	ZINC000014487674
0.7	-5.8	-5.5	0.16	1.822	0.772	3.098	1.031	6	2540	ZINC000000388194
0.7	-6.7	-6.5	0.12	1.801	0.189	2.818	0.725	6	2541	ZINC000001626972
0.7	-7.5	-7.2	0.27	1.592	0.076	3.691	0.690	6	2542	ZINC000001532584
0.7	-9.3	-8.5	0.65	2.386	0.264	4.906	1.383	3	2543	ZINC000003644903

0.7	-6.4	-5.9	0.29	2.462	1.037	3.355	1.181	6	2544	ZINC000095619758
0.7	-6.5	-6.2	0.16	2.649	1.092	3.831	1.187	6	2545	ZINC000001850883
0.7	-7.3	-6.9	0.23	1.750	0.199	3.345	0.259	6	2546	ZINC000001849926
0.7	-9.8	-8.3	0.93	1.242	0.229	2.484	0.474	4	2547	ZINC000014650356
0.7	-8.2	-7.9	0.22	2.931	0.293	4.955	0.642	6	2548	ZINC000013783212
0.7	-7.9	-7.5	0.26	1.403	0.291	3.435	0.388	6	2549	ZINC000100821652
0.7	-9.3	-7.8	0.91	1.735	0.251	3.650	0.787	5	2550	ZINC000100823129
0.7	-8.2	-7.7	0.31	1.351	0.310	2.942	0.819	6	2551	ZINC000100780735
0.7	-8.1	-7.9	0.21	2.342	0.362	3.114	0.320	6	2552	ZINC000100780355
0.7	-6.8	-6.6	0.11	2.215	0.743	3.390	0.594	6	2553	ZINC000000391851
0.7	-6.8	-6.4	0.20	1.587	0.318	4.277	0.785	6	2554	ZINC000031290671
0.7	-7.6	-7.2	0.28	2.404	0.792	4.102	0.954	6	2555	ZINC000001547195
0.7	-9.1	-7.8	0.96	1.658	0.056	3.061	0.049	3	2556	ZINC000095618492
0.7	-8.3	-8.0	0.22	2.111	0.980	3.501	0.598	6	2557	ZINC000100781122
0.7	-7.6	-7.1	0.27	2.055	0.441	3.525	1.623	6	2558	ZINC000003872731

0.7	-11.1	-10.0	0.84	1.844	0.354	4.458	1.315	6	2559	ZINC000257421590
0.7	-7.5	-7.1	0.36	1.805	0.577	2.509	1.113	6	2560	ZINC000001832155
0.7	-9.0	-8.3	0.41	1.540	0.215	3.862	1.229	6	2561	ZINC000001850165
0.7	-5.7	-5.3	0.18	1.963	0.535	2.482	0.607	6	2562	ZINC000000388713
0.7	-9.0	-8.7	0.24	1.992	0.864	3.308	1.055	6	2563	ZINC000104367271
0.7	-7.3	-7.0	0.16	2.285	0.738	3.232	0.596	6	2564	ZINC000100776203
0.7	-8.2	-7.7	0.29	2.373	0.526	3.802	0.652	6	2565	ZINC000000896202
0.7	-10.2	-8.7	0.83	1.370	0.297	4.252	1.771	5	2566	ZINC000003861550
0.7	-8.5	-8.1	0.30	1.627	0.592	2.401	0.649	6	2567	ZINC000101269492
0.7	-8.7	-8.3	0.25	2.112	0.223	4.540	1.238	6	2568	ZINC000016051666
0.7	-8.5	-8.2	0.22	1.595	0.524	2.306	0.493	6	2569	ZINC000100687736
0.7	-8.2	-7.9	0.24	2.243	0.628	3.811	1.981	6	2570	ZINC000006069529
0.7	-9.2	-8.5	0.52	2.181	0.747	5.044	1.122	6	2571	ZINC000038956900
0.7	-6.5	-6.4	0.08	2.112	0.582	3.122	0.867	6	2572	ZINC000002384606
0.7	-9.1	-8.3	0.46	1.973	0.353	3.977	0.342	6	2573	ZINC000100825887

0.7	-8.0	-7.8	0.15	2.329	0.730	3.714	0.603	6	2574	ZINC000014823208
0.7	-6.7	-6.3	0.22	2.010	0.830	2.765	0.915	6	2575	ZINC000000000490
0.7	-8.2	-7.5	0.48	1.892	0.608	3.248	1.119	6	2576	ZINC000013334984
0.7	-6.7	-6.3	0.25	2.238	0.744	3.226	0.778	6	2577	ZINC000000395645
0.7	-7.5	-7.1	0.17	1.507	0.246	1.873	0.407	6	2578	ZINC000000004358
0.7	-6.1	-6.0	0.09	1.058	0.125	2.927	0.374	6	2579	ZINC000100778608
0.7	-8.3	-8.2	0.11	2.248	0.493	6.218	2.253	6	2580	ZINC000014720655
0.7	-6.4	-6.0	0.21	1.849	0.260	3.741	0.892	6	2581	ZINC000100828985
0.7	-8.2	-7.8	0.30	1.873	0.180	4.055	1.023	6	2582	ZINC000015263561
0.7	-6.3	-5.9	0.27	2.769	0.751	3.844	1.260	6	2583	ZINC000031555018
0.7	-6.0	-5.7	0.18	2.467	0.526	4.168	0.242	6	2584	ZINC000012495421
0.7	-7.9	-7.5	0.33	1.364	0.268	2.310	0.473	6	2585	ZINC000001851053
0.7	-8.5	-7.7	0.40	2.139	0.188	3.665	1.049	6	2586	ZINC000230086244
0.7	-8.9	-8.5	0.27	2.138	0.709	4.390	1.342	6	2587	ZINC000004098527
0.7	-7.9	-7.6	0.22	1.885	0.442	3.087	0.577	6	2588	ZINC000003581319

0.7	-8.0	-7.6	0.17	2.257	0.104	3.853	0.671	6	2589	ZINC000005934430
0.7	-11.1	-10.2	0.69	1.668	0.483	3.659	1.821	6	2590	ZINC000100774356
0.7	-8.0	-7.7	0.16	2.267	0.882	5.003	1.900	6	2591	ZINC000013508857
0.7	-6.7	-6.3	0.28	2.147	0.307	3.252	0.717	6	2592	ZINC000013429396
0.7	-10.3	-9.7	0.37	1.433	0.352	3.700	0.901	6	2593	ZINC000100779648
0.7	-8.1	-7.4	0.38	2.273	0.442	4.480	1.137	6	2594	ZINC000005767142
0.7	-5.5	-5.3	0.11	1.208	0.065	2.759	0.434	6	2595	ZINC000002518855
0.7	-6.0	-5.9	0.04	2.470	0.501	3.873	0.821	6	2596	ZINC000032180007
0.7	-7.3	-7.2	0.13	1.862	0.437	3.749	0.254	6	2597	ZINC000003860296
0.7	-6.9	-6.6	0.19	1.945	0.796	3.910	1.409	6	2598	ZINC000257525585
0.7	-8.8	-8.2	0.31	1.971	0.518	3.881	1.264	6	2599	ZINC000012495328
0.7	-7.5	-7.1	0.25	2.148	1.077	3.395	0.709	6	2600	ZINC000001667585
0.7	-9.2	-8.9	0.19	1.673	0.447	4.968	1.780	6	2601	ZINC000013382470
0.7	-7.7	-7.4	0.12	2.441	0.526	4.316	0.391	6	2602	ZINC000100823347
0.7	-7.9	-7.5	0.25	1.303	0.280	3.238	0.473	6	2603	ZINC000100821653

0.7	-9.8	-9.0	0.51	1.475	0.384	4.595	1.208	6	2604	ZINC000257527007
0.7	-6.8	-6.5	0.22	1.844	0.932	3.657	0.766	6	2605	ZINC000000105246
0.7	-8.8	-8.5	0.23	1.937	0.370	3.390	0.998	6	2606	ZINC000015220974
0.7	-9.4	-8.8	0.52	1.907	0.183	5.793	0.696	6	2607	ZINC000014504544
0.7	-8.3	-8.1	0.20	1.480	0.234	5.242	2.137	6	2608	ZINC000247798767
0.7	-6.1	-5.9	0.13	1.697	0.224	2.803	0.373	6	2609	ZINC000002566224
0.7	-7.6	-7.3	0.19	2.491	0.608	4.437	1.243	6	2610	ZINC000100779844
0.7	-7.0	-6.6	0.21	2.543	0.379	3.879	0.379	6	2611	ZINC000006037729
0.7	-6.9	-6.7	0.09	2.082	0.953	3.499	1.107	6	2612	ZINC000001747853
0.7	-6.4	-6.0	0.22	1.920	1.018	3.082	1.116	6	2613	ZINC000014495084
0.7	-7.6	-7.3	0.14	2.234	0.141	5.279	0.616	6	2614	ZINC000001532511
0.7	-5.9	-5.5	0.19	1.623	0.339	3.409	0.905	6	2615	ZINC000001566476
0.7	-8.1	-7.5	0.47	2.158	1.026	4.023	1.740	6	2616	ZINC000000097168
0.7	-6.4	-6.0	0.31	2.509	0.278	3.812	0.710	6	2617	ZINC000003865059
0.7	-6.4	-6.2	0.13	2.510	0.702	3.173	0.701	6	2618	ZINC000033951470

0.7	-9.5	-8.0	0.74	1.959	0.201	4.172	1.772	6	2619	ZINC000238761677
0.7	-6.3	-6.0	0.20	2.144	0.664	3.323	0.910	6	2620	ZINC000005239363
0.7	-8.8	-7.9	0.52	2.098	0.455	3.853	1.117	6	2621	ZINC000006037459
0.7	-8.0	-7.6	0.39	1.620	0.341	3.562	0.532	6	2622	ZINC000014511122
0.7	-4.9	-4.6	0.19	1.803	0.310	2.951	0.510	6	2623	ZINC000001685644
0.7	-7.3	-7.1	0.11	1.890	0.574	3.069	0.318	6	2624	ZINC000095618206
0.7	-6.8	-6.1	0.35	1.566	0.307	2.707	0.867	6	2625	ZINC000014438711
0.7	-7.1	-6.8	0.19	2.031	0.756	3.217	0.748	6	2626	ZINC000000161294
0.7	-8.0	-7.8	0.23	1.491	0.632	4.080	1.188	6	2627	ZINC000008689960
0.7	-6.0	-5.6	0.20	2.096	0.683	3.673	0.405	6	2628	ZINC000005821721
0.7	-8.4	-7.7	0.41	1.689	0.332	4.703	1.480	6	2629	ZINC000014855568
0.7	-9.0	-8.8	0.12	1.861	0.209	3.241	0.240	6	2630	ZINC000115696265
0.7	-7.3	-7.0	0.19	2.335	0.396	3.676	0.902	6	2631	ZINC000004661832
0.7	-7.0	-6.8	0.12	2.608	0.308	3.602	0.547	6	2632	ZINC000000967598
0.7	-10.0	-9.4	0.35	1.316	0.353	4.512	1.535	6	2633	ZINC000014814387

0.7	-7.0	-6.7	0.19	2.942	0.744	4.330	0.719	6	2634	ZINC000000393698
0.7	-7.4	-7.2	0.11	2.167	0.223	4.158	1.886	6	2635	ZINC000001529647
0.7	-5.6	-5.3	0.14	1.270	0.501	2.714	0.424	6	2636	ZINC000008294954
0.7	-9.5	-8.2	1.17	1.492	0.426	4.577	1.669	5	2637	ZINC000006067691
0.7	-8.4	-7.8	0.39	1.695	0.183	3.848	1.246	6	2638	ZINC000015147898
0.7	-6.3	-6.0	0.17	2.732	0.727	4.472	0.973	6	2639	ZINC000012495945
0.7	-8.1	-7.6	0.35	2.331	0.483	3.924	0.946	6	2640	ZINC000014688570
0.7	-7.3	-7.0	0.14	2.149	0.481	3.872	0.915	6	2641	ZINC000012496250
0.7	-9.5	-8.2	0.78	1.403	0.297	4.996	1.412	5	2642	ZINC000003814418
0.7	-6.8	-6.6	0.15	1.678	0.357	3.828	0.715	6	2643	ZINC000014639163
0.7	-6.7	-6.3	0.19	2.275	0.267	3.775	0.650	6	2644	ZINC000014588533
0.7	-8.4	-7.7	0.42	1.739	0.302	5.179	1.695	6	2645	ZINC000014855566
0.7	-7.9	-7.2	0.35	2.343	0.332	5.829	1.241	6	2646	ZINC000059172212
0.7	-9.0	-8.7	0.23	2.000	0.897	3.233	1.026	6	2647	ZINC000086038749
0.7	-8.3	-8.0	0.22	2.390	0.716	3.574	0.569	6	2648	ZINC000100781119

0.7	-8.4	-8.0	0.28	2.504	0.619	4.081	1.256	6	2649	ZINC000014688574
0.7	-8.2	-7.6	0.30	1.672	0.385	4.846	2.052	6	2650	ZINC000015115286
0.7	-7.7	-7.5	0.25	1.009	0.781	4.083	0.991	6	2651	ZINC000001841214
0.7	-8.0	-7.7	0.19	2.693	0.806	4.676	0.268	6	2652	ZINC000013380510
0.7	-6.5	-6.1	0.19	2.631	0.736	3.497	0.889	6	2653	ZINC000001692499
0.7	-10.1	-9.4	0.97	2.183	0.188	4.718	1.558	3	2654	ZINC000049601330
0.7	-8.3	-7.7	0.36	2.295	0.120	4.428	1.105	6	2655	ZINC000005260863
0.7	-8.1	-7.7	0.30	1.517	0.143	3.867	1.370	6	2656	ZINC000031539577
0.7	-10.0	-9.0	0.79	1.177	0.282	5.167	2.132	6	2657	ZINC000090711972
0.7	-6.1	-5.9	0.13	2.274	0.406	4.017	0.496	6	2658	ZINC000095620553
0.7	-9.4	-8.6	0.41	1.542	0.315	3.805	0.679	6	2659	ZINC000257470336
0.7	-8.2	-8.0	0.15	1.667	0.501	2.840	1.272	6	2660	ZINC000085643429
0.7	-7.8	-7.3	0.49	2.071	0.294	2.601	0.552	6	2661	ZINC000000051120
0.7	-7.1	-6.8	0.16	2.443	0.583	3.580	0.349	6	2662	ZINC000005133008
0.7	-7.6	-7.2	0.19	2.427	0.533	4.694	0.640	6	2663	ZINC000005158581

0.7	-8.3	-7.9	0.19	1.723	0.258	4.465	1.197	6	2664	ZINC000040165445
0.7	-8.4	-7.6	0.48	1.861	0.177	3.845	1.888	6	2665	ZINC000014855563
0.7	-5.9	-5.6	0.17	2.342	0.523	3.572	1.187	6	2666	ZINC000001737786
0.7	-7.1	-6.7	0.21	2.226	0.538	2.991	0.743	6	2667	ZINC000000031165
0.7	-6.7	-6.3	0.23	1.539	0.513	4.134	0.956	6	2668	ZINC000014438663
0.7	-7.9	-7.4	0.37	2.004	0.260	3.640	1.147	6	2669	ZINC000142643352
0.7	-7.2	-7.1	0.11	2.625	0.969	4.034	1.412	6	2670	ZINC000004102315
0.7	-8.2	-8.0	0.18	1.473	0.255	3.451	0.458	6	2671	ZINC000015160899
0.7	-8.2	-7.8	0.21	1.945	0.247	4.798	1.288	6	2672	ZINC000014637061
0.7	-7.4	-7.1	0.24	3.157	0.478	5.045	0.610	6	2673	ZINC000095617758
0.7	-8.5	-8.2	0.16	1.196	0.403	3.178	0.833	6	2674	ZINC000005509740
0.7	-7.2	-6.7	0.30	2.498	0.635	3.566	1.013	6	2675	ZINC000000031164
0.7	-6.7	-6.5	0.12	2.535	0.470	4.110	1.118	6	2676	ZINC000001575553
0.7	-8.0	-7.9	0.09	1.736	0.219	4.302	0.344	6	2677	ZINC000231195452
0.7	-8.2	-7.9	0.14	1.616	0.546	3.672	0.769	6	2678	ZINC000085643426

0.7	-8.4	-8.0	0.24	2.240	0.386	3.734	0.680	6	2679	ZINC000013461956
0.7	-6.1	-5.9	0.12	2.891	0.604	4.674	0.415	6	2680	ZINC000003014482
0.7	-8.3	-8.0	0.15	2.140	0.395	5.207	1.936	6	2681	ZINC000034309949
0.7	-9.7	-8.9	0.50	1.633	0.226	3.662	1.274	6	2682	ZINC000257433876
0.7	-9.0	-7.2	0.97	1.939	0.353	5.333	1.735	5	2683	ZINC000095620846
0.7	-7.8	-7.6	0.15	1.715	0.552	3.333	1.614	6	2684	ZINC000001850817
0.7	-6.1	-5.9	0.11	2.744	0.870	3.822	0.830	6	2685	ZINC000032166951
0.7	-6.8	-6.4	0.21	3.042	0.503	4.281	0.333	6	2686	ZINC000000000868
0.7	-9.3	-8.6	0.48	3.012	0.477	5.758	0.254	6	2687	ZINC000008215530
0.7	-7.8	-7.4	0.21	1.687	0.598	3.543	1.153	6	2688	ZINC000005510216
0.7	-6.9	-6.7	0.17	2.368	0.214	4.385	0.836	6	2689	ZINC000002564637
0.7	-7.6	-7.1	0.30	2.486	0.799	4.226	0.627	6	2690	ZINC000014588993
0.7	-9.0	-8.3	0.41	2.061	0.262	4.595	1.438	6	2691	ZINC000100772710
0.7	-6.9	-6.7	0.21	2.086	0.602	3.537	1.593	6	2692	ZINC000033992254
0.7	-5.8	-5.6	0.13	2.646	0.779	3.983	0.552	6	2693	ZINC000095620554

0.7	-6.8	-6.7	0.06	1.708	0.432	3.808	0.999	6	2694	ZINC000014591999
0.7	-9.1	-8.5	0.42	2.237	0.459	4.290	0.630	6	2695	ZINC000257465009
0.7	-8.1	-7.8	0.28	2.101	0.145	3.456	1.066	6	2696	ZINC000095617660
0.7	-10.1	-9.4	0.43	1.813	1.053	3.568	1.115	6	2697	ZINC000100780739
0.7	-7.3	-7.1	0.13	2.355	0.119	4.550	0.926	6	2698	ZINC000014440794
0.7	-9.2	-8.5	0.50	2.156	0.767	5.014	1.135	6	2699	ZINC000039002493
0.7	-6.9	-6.5	0.20	2.370	0.481	3.777	0.848	6	2700	ZINC000002040440
0.7	-6.7	-6.4	0.21	3.287	0.459	5.313	0.510	6	2701	ZINC000034050194
0.7	-8.6	-7.2	1.04	2.031	0.505	7.165	0.096	3	2702	ZINC000000899853
0.7	-10.3	-9.6	0.44	1.446	0.360	4.300	0.891	6	2703	ZINC000257446471
0.7	-5.8	-5.5	0.17	2.299	0.443	3.633	0.469	6	2704	ZINC000000399366
0.7	-6.8	-6.7	0.05	0.829	0.525	2.914	0.593	6	2705	ZINC000100826343
0.7	-8.5	-8.0	0.39	1.323	0.671	4.129	1.367	6	2706	ZINC000015113766
0.7	-7.1	-6.8	0.29	1.781	0.370	4.621	1.903	6	2707	ZINC000006760986
0.7	-8.1	-7.8	0.23	2.368	0.202	4.755	1.369	6	2708	ZINC000004899446

0.7	-10.3	-9.0	0.94	1.796	0.460	4.127	2.162	6	2709	ZINC000257418755
0.7	-8.7	-8.4	0.21	1.690	0.483	2.770	1.360	6	2710	ZINC000002562337
0.7	-6.8	-6.7	0.05	0.638	0.274	2.758	0.479	6	2711	ZINC000070451052
0.7	-9.8	-9.0	0.61	1.497	0.443	4.218	1.430	6	2712	ZINC000100824254
0.7	-7.3	-7.0	0.17	1.896	0.328	3.336	0.795	6	2713	ZINC000195800476
0.7	-6.8	-6.3	0.28	2.383	0.564	3.689	0.897	6	2714	ZINC000001575515
0.7	-7.8	-7.5	0.20	2.511	0.160	4.628	1.155	6	2715	ZINC000050027451
0.7	-7.2	-7.0	0.21	2.711	0.225	5.297	0.148	6	2716	ZINC000014440796
0.7	-8.2	-7.7	0.33	1.684	0.219	3.489	1.070	6	2717	ZINC000013432655
0.7	-9.2	-8.7	0.39	1.770	0.602	3.244	1.309	6	2718	ZINC000080572978
0.7	-6.7	-6.3	0.22	1.658	0.586	3.375	0.756	6	2719	ZINC000002510282
0.7	-9.2	-8.5	0.51	2.183	0.747	5.042	1.128	6	2720	ZINC000257403776
0.7	-7.2	-6.9	0.20	2.684	0.760	5.626	0.783	6	2721	ZINC000000116202
0.7	-8.3	-7.9	0.25	1.873	0.771	3.681	1.769	6	2722	ZINC000005998227
0.7	-8.4	-7.5	0.47	1.999	0.213	5.818	0.441	6	2723	ZINC000014504524

0.7	-5.6	-5.2	0.20	2.303	0.663	3.558	0.800	6	2724	ZINC000001589669
0.7	-8.7	-8.0	0.45	2.153	0.361	3.730	1.311	6	2725	ZINC000257542065
0.7	-6.3	-6.1	0.21	1.641	0.551	2.866	0.231	6	2726	ZINC000100828317
0.7	-5.8	-5.3	0.29	2.220	0.373	3.467	0.797	6	2727	ZINC000000157430
0.7	-7.4	-7.2	0.11	1.472	0.292	3.980	1.416	6	2728	ZINC000257557767
0.7	-5.6	-5.3	0.21	2.457	0.197	3.718	0.590	6	2729	ZINC000000158146
0.7	-8.1	-7.5	0.46	1.594	0.236	3.470	0.795	6	2730	ZINC000015147896
0.7	-10.8	-10.1	0.52	1.602	0.141	4.826	1.713	6	2731	ZINC000257456805
0.7	-9.8	-9.4	0.25	2.125	0.215	4.299	0.991	6	2732	ZINC000014591311
0.7	-10.1	-9.4	0.45	1.843	1.022	3.839	1.372	6	2733	ZINC000257394845
0.7	-8.9	-8.4	0.28	1.615	0.472	5.309	1.943	6	2734	ZINC000014642315
0.7	-9.4	-8.8	0.51	2.052	0.227	4.238	1.005	6	2735	ZINC000104370417
0.7	-7.7	-7.2	0.26	2.522	0.820	3.673	0.823	6	2736	ZINC000002006632
0.7	-9.0	-8.4	0.37	2.198	0.486	4.955	0.546	6	2737	ZINC000015216542
0.7	-10.0	-9.0	0.79	1.088	0.199	6.128	1.705	6	2738	ZINC000085589772

0.7	-7.9	-7.6	0.21	2.651	0.820	3.802	1.609	6	2739	ZINC000002575475
0.7	-8.8	-7.6	0.64	1.924	0.242	5.380	0.523	6	2740	ZINC000261497623
0.7	-6.5	-6.3	0.15	2.162	0.926	4.100	0.723	6	2741	ZINC000006030496
0.7	-8.6	-7.6	0.69	2.859	0.602	4.775	1.476	6	2742	ZINC000004490939
0.7	-7.1	-6.7	0.26	3.254	0.871	4.750	1.270	6	2743	ZINC000095620535
0.7	-5.7	-5.2	0.22	1.781	0.711	4.330	0.093	6	2744	ZINC000001871730
0.7	-8.7	-8.5	0.18	1.798	0.398	4.335	2.058	6	2745	ZINC000001608669
0.7	-8.3	-8.1	0.21	1.702	0.449	6.171	1.557	6	2746	ZINC000014690458
0.7	-7.0	-6.8	0.15	2.770	1.544	4.042	0.716	6	2747	ZINC000001706140
0.7	-9.4	-8.7	0.33	1.747	0.526	4.398	2.075	6	2748	ZINC000028361970
0.7	-7.6	-7.1	0.28	2.337	0.765	4.295	0.723	6	2749	ZINC000034276228
0.7	-9.0	-8.7	0.19	1.681	0.765	3.055	0.981	6	2750	ZINC000086038753
0.7	-7.2	-6.5	0.39	2.074	0.352	3.772	0.759	6	2751	ZINC000001591826
0.7	-5.8	-5.6	0.13	1.865	0.311	2.896	0.800	6	2752	ZINC000004521745
0.7	-7.7	-7.2	0.24	1.788	0.553	3.409	1.133	6	2753	ZINC000004661833

0.7	-10.8	-10.0	0.83	1.843	0.499	5.444	1.215	6	2754	ZINC000100780540
0.7	-6.9	-6.3	0.29	2.414	1.183	3.489	1.172	6	2755	ZINC000095619765
0.7	-10.1	-9.4	0.45	1.759	1.086	3.660	1.110	6	2756	ZINC000257394847
0.7	-9.1	-8.7	0.34	1.292	0.069	3.184	1.298	6	2757	ZINC000257606001
0.7	-8.1	-7.8	0.15	1.483	0.559	2.855	1.618	6	2758	ZINC000013376489
0.7	-9.8	-8.8	0.58	1.485	0.430	4.887	2.251	6	2759	ZINC000006067696
0.7	-7.0	-6.8	0.15	2.598	0.366	4.155	0.504	6	2760	ZINC000000001217
0.7	-10.7	-9.7	0.58	2.272	0.477	4.804	0.909	6	2761	ZINC000003823232
0.7	-6.1	-5.9	0.13	3.111	0.717	3.703	0.861	6	2762	ZINC000002516013
0.7	-6.4	-6.0	0.25	2.401	0.797	4.346	0.528	6	2763	ZINC000095619615
0.7	-7.3	-7.0	0.27	2.007	0.427	3.336	0.722	6	2764	ZINC000013380487
0.7	-6.7	-6.4	0.18	2.099	0.603	3.066	1.154	6	2765	ZINC000004528592
0.7	-6.0	-5.7	0.21	2.677	0.677	4.254	0.923	6	2766	ZINC000095620555
0.7	-7.2	-7.0	0.24	1.817	0.217	4.213	1.963	6	2767	ZINC000005888070
0.7	-8.3	-7.9	0.27	2.423	0.482	4.263	1.446	6	2768	ZINC000012494415

0.7	-7.0	-6.4	0.28	1.949	0.564	3.216	0.971	6	2769	ZINC000001849842
0.7	-11.7	-11.7	0.00	0.000	0.000	0.000	0.000	1	2770	ZINC000038298810
0.7	-9.3	-8.9	0.54	1.599	0.541	4.984	1.567	6	2771	ZINC000004098319
0.7	-8.1	-7.7	0.30	1.444	0.157	5.036	0.241	6	2772	ZINC000015147951
0.7	-8.3	-7.8	0.31	2.126	0.766	3.720	1.746	6	2773	ZINC000032839147
0.7	-11.1	-9.9	0.89	1.966	0.424	4.724	1.522	6	2774	ZINC000257421589
0.7	-7.7	-7.4	0.21	1.665	0.134	4.162	1.310	6	2775	ZINC000085588773
0.7	-6.4	-6.2	0.15	3.214	0.764	4.364	1.058	6	2776	ZINC000005734944
0.7	-6.2	-6.0	0.17	2.711	0.921	3.955	0.962	6	2777	ZINC000037866093
0.7	-9.9	-8.9	0.92	1.811	0.383	4.183	1.632	6	2778	ZINC000014778655
0.7	-8.5	-7.4	0.63	2.165	0.445	5.345	1.495	6	2779	ZINC000026387734
0.7	-7.1	-6.6	0.24	2.959	0.457	4.321	0.640	6	2780	ZINC000000394884
0.7	-7.8	-7.5	0.18	2.158	0.680	3.652	1.323	6	2781	ZINC000000406939
0.7	-7.5	-7.3	0.19	2.144	0.210	4.994	0.681	6	2782	ZINC000014494723
0.7	-6.3	-6.0	0.14	3.358	0.627	5.099	0.213	6	2783	ZINC000038251274

0.7	-8.4	-8.0	0.22	1.857	0.433	3.567	1.253	6	2784	ZINC000015256931
0.7	-9.0	-8.3	0.43	2.481	0.611	4.119	0.932	6	2785	ZINC000069486147
0.7	-7.7	-7.1	0.35	2.219	0.778	4.103	2.269	6	2786	ZINC000031706539
0.7	-9.3	-8.1	0.75	2.340	0.332	4.365	2.112	6	2787	ZINC000014806174
0.7	-7.3	-7.1	0.13	1.684	0.446	4.727	1.954	6	2788	ZINC000085589841
0.7	-6.4	-6.2	0.15	2.218	0.704	3.528	1.305	6	2789	ZINC000004095998
0.7	-10.0	-8.3	1.19	1.235	0.392	2.943	0.892	3	2790	ZINC000015119967
0.7	-6.6	-6.4	0.15	2.362	0.876	3.572	1.380	6	2791	ZINC000004521351
0.7	-7.1	-6.6	0.25	3.028	0.615	4.193	0.802	6	2792	ZINC000004098478
0.7	-8.3	-7.9	0.27	3.130	0.567	4.590	0.750	6	2793	ZINC000040471112
0.7	-8.6	-8.4	0.15	1.988	1.479	4.086	1.286	6	2794	ZINC000001672966
0.7	-7.5	-7.2	0.20	1.973	0.374	3.287	1.174	6	2795	ZINC000015213096
0.7	-7.8	-7.1	0.43	2.683	0.802	3.599	0.737	6	2796	ZINC000000192311
0.7	-8.9	-8.3	0.31	1.393	0.129	5.590	1.277	6	2797	ZINC000014764521
0.7	-9.8	-9.1	0.51	1.819	0.292	3.616	1.495	6	2798	ZINC000014636756

0.7	-9.8	-8.4	1.00	1.399	0.181	5.114	1.394	5	2799	ZINC000005339055
0.7	-6.1	-5.6	0.24	2.788	1.487	3.546	1.548	6	2800	ZINC000014507030
0.7	-7.9	-7.6	0.18	2.214	0.274	4.073	0.576	6	2801	ZINC000056874350
0.7	-6.1	-5.7	0.20	2.131	0.514	3.500	0.624	6	2802	ZINC000003074808
0.7	-6.9	-6.5	0.25	1.836	0.445	5.242	0.250	6	2803	ZINC000014817950
0.7	-7.6	-7.1	0.27	2.394	0.754	3.933	0.655	6	2804	ZINC000014588996
0.7	-9.3	-8.4	0.67	1.818	0.622	3.116	1.529	6	2805	ZINC000100825945
0.7	-6.3	-6.0	0.17	2.822	0.707	4.544	0.625	6	2806	ZINC000095618189
0.7	-5.5	-5.3	0.16	3.302	1.016	4.027	0.490	6	2807	ZINC000032163567
0.7	-7.3	-6.8	0.27	2.014	0.240	3.607	0.451	6	2808	ZINC000014651624
0.7	-5.7	-5.2	0.25	2.182	1.165	4.029	1.145	6	2809	ZINC000001871732
0.7	-9.4	-9.0	0.23	1.634	0.165	3.517	0.341	6	2810	ZINC000005416256
0.7	-8.4	-7.7	0.38	1.649	0.328	4.399	1.861	6	2811	ZINC000257522825
0.7	-8.7	-8.3	0.35	1.873	0.467	3.285	0.799	6	2812	ZINC000100072466
0.7	-9.3	-9.0	0.24	1.819	0.323	5.139	1.766	3	2813	ZINC000014820481

0.7	-7.8	-7.6	0.15	2.365	0.731	4.896	1.038	6	2814	ZINC000013484895
0.7	-10.8	-10.3	0.47	1.967	0.372	3.435	0.062	6	2815	ZINC000100777811
0.7	-7.1	-6.9	0.08	2.608	0.699	3.894	1.101	6	2816	ZINC000005274093
0.7	-7.5	-7.1	0.21	2.715	0.480	4.796	0.982	6	2817	ZINC000100779838
0.7	-9.4	-8.6	0.38	2.413	0.289	4.548	1.143	6	2818	ZINC000050027596
0.7	-7.5	-7.3	0.09	2.423	0.480	5.110	1.643	6	2819	ZINC000013356164
0.7	-6.2	-5.9	0.17	2.946	0.751	4.475	0.800	6	2820	ZINC000001693594
0.7	-8.6	-7.9	0.53	2.318	0.779	3.587	1.117	6	2821	ZINC000090697506
0.7	-6.8	-6.6	0.13	1.610	0.321	2.628	0.261	6	2822	ZINC000005820027
0.7	-9.2	-8.5	0.52	2.177	0.744	5.051	1.104	6	2823	ZINC000100777198
0.7	-7.6	-7.1	0.28	2.275	0.901	3.571	1.263	6	2824	ZINC000038350357
0.7	-8.7	-8.2	0.31	2.012	0.419	3.692	1.757	6	2825	ZINC000000057912
0.7	-8.3	-8.0	0.21	1.902	0.379	3.956	0.541	6	2826	ZINC000085954086
0.7	-7.7	-7.1	0.43	1.734	0.939	5.040	0.876	6	2827	ZINC000100016181
0.7	-8.3	-8.1	0.16	1.520	0.226	6.134	1.545	6	2828	ZINC000247798773

0.7	-7.5	-7.2	0.17	1.220	0.210	2.727	0.427	6	2829	ZINC000100018138
0.7	-8.8	-8.4	0.44	1.919	0.368	4.299	0.592	6	2830	ZINC000014687095
0.7	-8.6	-8.1	0.62	2.989	1.288	4.733	1.666	6	2831	ZINC000012360703
0.7	-6.7	-6.5	0.21	1.933	0.488	3.923	1.870	6	2832	ZINC000001856482
0.7	-6.8	-6.4	0.21	2.487	0.523	4.207	0.472	6	2833	ZINC000034449169
0.7	-6.9	-6.6	0.14	2.721	0.347	4.631	0.270	6	2834	ZINC000012495649
0.7	-5.6	-5.5	0.19	1.402	0.698	3.012	0.504	6	2835	ZINC000032152670
0.7	-7.9	-7.5	0.27	2.685	0.771	5.443	1.577	6	2836	ZINC000015261253
0.7	-7.9	-7.4	0.21	2.110	0.407	4.082	0.462	6	2837	ZINC000095617658
0.7	-7.2	-7.1	0.06	1.702	0.298	4.259	0.624	6	2838	ZINC000003802189
0.7	-8.5	-8.0	0.36	1.949	0.153	3.706	0.406	6	2839	ZINC000257454434
0.7	-8.4	-8.0	0.28	1.758	0.463	3.440	1.151	6	2840	ZINC000230080207
0.7	-6.4	-6.0	0.28	2.155	0.695	2.901	0.622	6	2841	ZINC000013306848
0.7	-7.6	-7.3	0.25	2.253	0.275	4.283	0.523	6	2842	ZINC000013377375
0.7	-8.3	-8.0	0.21	1.487	0.396	4.048	1.272	6	2843	ZINC000013377872

0.7	-9.1	-8.5	0.41	1.821	0.244	3.400	0.431	6	2844	ZINC000003947475
0.7	-8.6	-8.3	0.28	1.719	0.302	3.055	1.774	6	2845	ZINC000013303512
0.7	-5.4	-5.1	0.15	1.514	0.531	2.656	0.470	6	2846	ZINC000000409274
0.7	-10.0	-9.4	0.32	0.829	0.155	2.988	1.065	6	2847	ZINC000257625698
0.7	-6.7	-6.4	0.17	1.697	0.258	3.218	1.232	6	2848	ZINC000086034554
0.7	-6.9	-6.7	0.18	1.709	0.807	3.179	0.463	6	2849	ZINC000014818206
0.7	-9.1	-8.7	0.26	1.973	0.792	4.322	1.951	6	2850	ZINC000014811615
0.7	-8.1	-7.6	0.30	2.179	0.830	3.887	1.299	6	2851	ZINC000014720613
0.7	-9.0	-8.6	0.29	1.236	0.685	2.921	1.732	6	2852	ZINC000015249557
0.7	-8.7	-8.4	0.16	1.947	0.546	3.671	1.139	6	2853	ZINC000095099889
0.7	-9.8	-9.1	0.53	1.478	0.387	4.539	1.316	6	2854	ZINC000100824253
0.7	-7.3	-6.7	0.27	1.294	0.582	3.150	1.031	6	2855	ZINC000001532070
0.7	-7.5	-7.3	0.21	1.428	0.211	3.073	0.363	6	2856	ZINC000013551720
0.7	-7.9	-7.7	0.18	1.532	0.213	3.064	0.855	6	2857	ZINC000104883029
0.7	-7.6	-7.1	0.28	2.008	0.954	3.421	1.369	6	2858	ZINC000034276224

0.7	-6.8	-6.4	0.23	2.706	0.688	4.787	1.907	6	2859	ZINC000005116863
0.7	-6.4	-6.0	0.23	2.469	0.555	3.810	1.267	6	2860	ZINC000033844979
0.7	-10.0	-9.4	0.37	1.221	0.551	3.235	0.650	6	2861	ZINC000100781036
0.7	-6.0	-5.7	0.18	2.565	1.157	3.938	1.390	6	2862	ZINC000005113046
0.7	-5.0	-4.8	0.12	1.277	0.291	2.664	0.400	6	2863	ZINC000001845895
0.7	-7.5	-7.4	0.07	2.158	0.491	4.307	1.258	6	2864	ZINC000015213112
0.7	-8.2	-7.8	0.25	1.459	0.255	3.962	0.531	6	2865	ZINC000002169363
0.7	-7.7	-7.2	0.24	1.880	0.206	3.484	1.394	6	2866	ZINC000085588750
0.7	-8.1	-7.9	0.15	1.858	0.408	4.142	1.771	6	2867	ZINC000014720635
0.7	-7.5	-7.2	0.24	1.388	0.748	3.584	0.811	6	2868	ZINC000040164573
0.7	-9.0	-8.4	0.31	2.062	0.761	5.169	1.782	6	2869	ZINC000000006047
0.7	-8.5	-8.1	0.20	2.091	0.501	4.103	2.021	6	2870	ZINC000069486134
0.7	-8.0	-7.2	0.43	2.340	0.840	4.147	1.927	6	2871	ZINC000230070585
0.7	-7.4	-7.1	0.19	2.243	1.075	5.138	1.400	6	2872	ZINC000001532786
0.7	-8.3	-8.2	0.15	1.792	0.237	4.194	1.868	6	2873	ZINC000038965442

0.7	-8.9	-8.3	0.44	1.565	0.751	4.105	0.394	6	2874	ZINC000085491480
0.7	-7.3	-7.0	0.17	1.694	0.331	3.628	1.086	6	2875	ZINC000014487670
0.7	-9.8	-8.5	0.95	1.866	0.675	4.165	1.295	5	2876	ZINC000100824313
0.7	-7.0	-6.8	0.15	3.008	0.772	4.811	0.479	6	2877	ZINC000000056609
0.7	-8.3	-7.8	0.30	1.731	0.303	4.838	1.012	6	2878	ZINC000085954084
0.7	-6.8	-6.5	0.17	1.662	0.673	3.639	1.027	6	2879	ZINC000000393126
0.7	-5.2	-4.9	0.18	2.356	0.709	3.582	0.773	6	2880	ZINC000014589726
0.7	-9.4	-8.7	0.49	1.294	0.153	3.495	1.165	6	2881	ZINC000257391555
0.7	-6.3	-6.0	0.16	2.686	0.899	4.344	0.822	6	2882	ZINC000014590678
0.7	-7.0	-6.7	0.18	2.053	0.773	2.803	1.047	6	2883	ZINC000095618098
0.7	-9.4	-8.9	0.38	1.925	0.456	4.170	1.667	6	2884	ZINC000000519174
0.7	-7.2	-6.6	0.33	3.183	0.669	4.265	0.749	6	2885	ZINC000000058257
0.7	-6.9	-6.5	0.24	3.135	1.055	4.414	1.528	6	2886	ZINC000033992257
0.7	-7.3	-7.0	0.14	1.767	0.332	3.361	1.160	6	2887	ZINC000001841752
0.7	-8.3	-8.0	0.19	2.333	0.627	3.483	0.368	6	2888	ZINC000100781124

0.7	-8.5	-8.2	0.27	1.899	0.359	3.488	1.610	6	2889	ZINC000034264743
0.7	-8.8	-8.0	0.47	1.973	0.281	3.909	1.536	6	2890	ZINC000085847766
0.7	-8.1	-7.3	0.44	1.988	0.193	3.527	1.354	6	2891	ZINC000013350899
0.7	-9.1	-9.0	0.09	1.577	0.448	4.119	0.907	6	2892	ZINC000000001904
0.7	-6.2	-6.0	0.11	1.802	0.347	4.137	0.397	6	2893	ZINC000003074809
0.7	-10.3	-9.7	0.37	1.438	0.344	3.687	0.913	6	2894	ZINC000100779644
0.7	-7.0	-6.7	0.23	1.710	0.322	4.240	0.940	6	2895	ZINC000212538847
0.7	-6.1	-5.9	0.12	2.203	0.897	3.315	1.214	6	2896	ZINC000000156048
0.7	-9.2	-8.5	0.48	1.881	0.400	5.286	1.932	6	2897	ZINC000005998641
0.7	-5.7	-5.3	0.23	2.483	0.317	3.514	0.785	6	2898	ZINC000001529501
0.7	-7.0	-6.6	0.22	1.874	0.842	2.710	0.903	6	2899	ZINC000006037917
0.7	-8.6	-8.2	0.19	2.189	0.339	4.555	0.510	6	2900	ZINC000001089845
0.7	-6.6	-6.4	0.11	2.612	0.921	4.438	1.589	6	2901	ZINC000005113157
0.7	-7.4	-7.1	0.15	1.493	0.256	3.579	1.406	6	2902	ZINC000100820653
0.7	-7.0	-6.8	0.14	2.251	0.205	5.482	1.446	6	2903	ZINC000095617687

0.7	-8.3	-7.7	0.34	2.152	0.526	4.409	1.647	6	2904	ZINC000015120564
0.7	-6.8	-6.6	0.11	1.231	0.066	2.570	0.549	6	2905	ZINC000002517078
0.7	-7.5	-7.2	0.18	1.835	0.263	3.342	1.311	6	2906	ZINC000409418451
0.7	-8.7	-8.0	0.46	2.155	0.691	4.597	1.278	6	2907	ZINC000006030349
0.7	-9.0	-7.8	0.95	1.947	0.517	4.235	1.877	6	2908	ZINC000017263727
0.7	-7.9	-7.5	0.21	1.870	0.331	4.194	1.242	6	2909	ZINC000034521288
0.7	-8.1	-7.6	0.35	2.823	0.940	5.252	2.205	6	2910	ZINC000002123545
0.7	-5.7	-5.2	0.25	2.972	1.471	4.955	0.870	6	2911	ZINC000001871733
0.7	-6.4	-6.0	0.19	2.224	0.496	3.774	0.724	6	2912	ZINC000003869518
0.7	-9.8	-9.1	0.50	1.476	0.389	4.592	1.221	6	2913	ZINC000238771963
0.7	-7.2	-6.7	0.28	2.447	0.566	4.304	0.914	6	2914	ZINC000005963484
0.7	-9.2	-8.6	0.45	2.159	0.757	4.995	1.102	6	2915	ZINC000257403774
0.7	-5.9	-5.5	0.18	2.588	1.304	4.629	0.484	6	2916	ZINC000033951616
0.7	-7.0	-6.7	0.17	2.384	0.514	4.276	1.476	6	2917	ZINC000004556547
0.7	-9.6	-9.2	0.20	1.665	0.462	5.091	0.555	6	2918	ZINC000000388659

0.7	-7.4	-7.1	0.15	1.602	0.451	4.208	1.402	6	2919	ZINC000100820652
0.7	-7.0	-6.7	0.19	2.514	0.944	5.010	0.843	6	2920	ZINC000005758641
0.7	-8.8	-8.5	0.27	1.657	0.833	4.442	1.782	6	2921	ZINC000002526471
0.7	-7.9	-7.7	0.16	2.954	0.115	4.826	0.539	6	2922	ZINC000012495479
0.7	-9.5	-8.2	1.02	2.172	0.485	4.031	1.747	6	2923	ZINC000005359804
0.7	-5.6	-5.4	0.10	2.006	1.338	3.837	1.510	6	2924	ZINC000002019768
0.7	-6.1	-5.9	0.19	2.012	0.921	3.175	1.136	6	2925	ZINC000001850596
0.7	-10.1	-9.5	0.41	1.283	0.798	3.599	0.616	6	2926	ZINC000100779500
0.7	-9.6	-8.8	0.58	2.191	0.601	4.462	1.476	6	2927	ZINC000013328289
0.7	-8.3	-7.7	0.36	1.784	0.770	4.712	0.611	6	2928	ZINC000044431718
0.7	-7.9	-7.7	0.11	1.718	0.401	4.515	1.344	6	2929	ZINC000015165684
0.7	-5.7	-5.5	0.19	2.056	1.081	2.830	1.130	6	2930	ZINC000000409322
0.7	-7.3	-7.1	0.11	1.772	0.697	3.017	0.276	6	2931	ZINC000095618207
0.7	-6.5	-6.3	0.14	1.909	0.420	3.772	0.897	6	2932	ZINC000014588938
0.7	-8.2	-7.8	0.22	2.302	0.426	4.827	1.123	6	2933	ZINC000039268170

0.7	-6.9	-6.7	0.24	1.290	0.183	2.789	0.372	6	2934	ZINC000032840809
0.7	-8.2	-7.7	0.27	2.080	0.395	3.710	1.185	6	2935	ZINC000000001311
0.7	-6.2	-5.8	0.25	1.578	0.389	3.041	0.845	6	2936	ZINC000014438707
0.7	-7.4	-7.1	0.13	1.341	0.347	3.921	1.396	6	2937	ZINC000100820655
0.7	-10.1	-9.0	0.66	2.415	0.240	4.933	0.352	6	2938	ZINC000000898731
0.7	-9.8	-9.2	0.34	2.031	0.542	5.525	0.564	6	2939	ZINC000038420626
0.7	-8.5	-8.1	0.33	1.400	0.268	3.195	0.366	6	2940	ZINC000015120363
0.7	-8.7	-8.2	0.30	1.647	0.365	4.203	0.821	6	2941	ZINC000013383574
0.7	-8.2	-7.8	0.27	1.362	0.444	2.369	0.959	6	2942	ZINC000100779315
0.7	-10.0	-9.4	0.34	1.131	0.585	3.269	0.685	6	2943	ZINC000100781031
0.7	-5.6	-5.4	0.15	2.173	0.470	3.587	1.115	6	2944	ZINC000000157424
0.7	-9.1	-8.4	0.43	2.328	0.463	5.204	0.938	6	2945	ZINC000257562930
0.7	-8.5	-8.0	0.26	1.961	0.458	4.072	2.025	6	2946	ZINC000028631418
0.7	-7.3	-7.1	0.12	1.758	0.733	2.998	0.273	6	2947	ZINC000100776202
0.7	-5.2	-5.0	0.10	2.088	0.409	3.249	1.184	6	2948	ZINC000095620710

0.7	-6.1	-5.7	0.21	2.295	0.702	4.062	1.001	6	2949	ZINC000100048378
0.7	-7.6	-7.2	0.26	1.907	0.565	4.089	1.343	6	2950	ZINC000100028946
0.7	-6.7	-6.4	0.16	1.886	0.853	3.943	0.951	6	2951	ZINC000004557091
0.7	-8.4	-8.0	0.25	1.263	0.189	3.582	0.477	6	2952	ZINC000086035225
0.7	-7.6	-7.1	0.32	2.664	0.689	4.271	0.544	6	2953	ZINC000014588994
0.7	-7.8	-7.4	0.21	1.869	0.050	4.514	0.489	6	2954	ZINC000095618200
0.7	-6.7	-6.4	0.16	3.474	1.084	4.715	1.458	6	2955	ZINC000014439083
0.7	-7.2	-7.0	0.12	2.776	1.137	4.165	1.149	6	2956	ZINC000001680640
0.7	-8.7	-8.2	0.40	2.093	0.457	3.828	1.235	6	2957	ZINC000034290028
0.7	-7.2	-7.0	0.20	1.350	0.444	3.391	1.400	6	2958	ZINC000100825957
0.7	-6.4	-6.0	0.21	2.752	1.118	3.851	1.453	6	2959	ZINC000095620430
0.7	-6.4	-6.2	0.11	1.886	0.852	4.525	1.158	6	2960	ZINC000085972373
0.7	-8.2	-7.8	0.22	1.702	0.458	4.255	1.276	6	2961	ZINC000257426777
0.7	-5.4	-4.9	0.25	2.368	0.911	3.749	1.197	6	2962	ZINC000001599379
0.7	-9.1	-8.4	0.44	2.699	0.322	5.143	0.891	6	2963	ZINC000257562928

0.7	-8.1	-7.6	0.28	3.329	0.523	4.899	0.303	6	2964	ZINC000003014490
0.7	-7.1	-7.0	0.07	2.154	0.573	3.584	1.582	6	2965	ZINC000002579085
0.7	-8.2	-7.3	0.52	1.656	0.211	3.572	0.703	6	2966	ZINC000095622610
0.7	-7.3	-7.1	0.15	2.233	0.276	4.300	0.930	6	2967	ZINC000001580404
0.7	-8.4	-7.8	0.44	1.767	0.796	4.257	1.232	6	2968	ZINC000000394686
0.7	-8.8	-8.6	0.09	1.874	0.213	4.183	1.020	6	2969	ZINC000230089202
0.7	-6.1	-5.9	0.15	2.858	1.160	4.592	0.602	6	2970	ZINC000022058494
0.7	-6.0	-5.7	0.21	2.035	0.614	3.689	0.595	6	2971	ZINC000002164100
0.7	-7.7	-7.5	0.13	2.874	1.087	4.219	1.741	6	2972	ZINC000015203321
0.7	-9.8	-8.5	0.89	1.795	0.505	3.697	1.723	6	2973	ZINC000014689080
0.7	-10.1	-9.5	0.40	1.791	1.061	3.299	1.093	6	2974	ZINC000100780738
0.7	-8.3	-8.1	0.18	1.498	0.440	4.076	2.267	6	2975	ZINC000014690456
0.7	-8.6	-8.2	0.24	2.327	0.220	3.800	0.667	6	2976	ZINC000005751118
0.7	-6.8	-6.5	0.17	1.958	0.645	2.802	0.662	6	2977	ZINC000033951136
0.7	-9.1	-8.4	0.53	2.088	0.435	4.224	1.566	6	2978	ZINC000014488318

0.7	-8.5	-7.9	0.30	2.267	0.559	3.384	1.126	6	2979	ZINC000140984829
0.7	-7.9	-7.5	0.19	2.003	0.304	4.929	1.147	6	2980	ZINC000027645241
0.7	-9.2	-8.4	0.51	2.442	0.425	4.965	0.663	6	2981	ZINC000257590928
0.7	-7.5	-7.2	0.19	1.809	0.410	4.090	1.067	6	2982	ZINC000006066774
0.7	-10.3	-9.5	0.71	1.924	0.554	5.580	1.571	4	2983	ZINC000100780826
0.7	-9.1	-8.3	0.45	2.450	0.555	5.507	0.700	6	2984	ZINC000100777203
0.7	-5.7	-5.4	0.18	2.716	1.058	3.415	1.607	6	2985	ZINC000014680219
0.7	-11.1	-10.1	0.68	1.848	0.316	3.846	1.681	6	2986	ZINC000257421591
0.7	-9.7	-8.2	0.87	1.817	0.328	4.364	1.584	6	2987	ZINC000014815043
0.7	-8.2	-7.8	0.43	1.979	0.194	4.437	1.250	6	2988	ZINC000013374015
0.7	-6.2	-6.0	0.18	2.045	0.698	3.944	1.039	6	2989	ZINC000095618187
0.7	-5.8	-5.4	0.22	3.038	1.482	4.008	1.447	6	2990	ZINC000022065336
0.7	-5.7	-5.1	0.27	2.656	0.918	4.694	0.385	6	2991	ZINC000001871735
0.7	-9.7	-9.3	0.27	2.114	0.128	3.462	0.826	6	2992	ZINC000014619350
0.7	-8.6	-8.1	0.35	2.340	0.230	3.910	1.032	6	2993	ZINC000013408183

0.7	-7.4	-7.1	0.15	1.678	0.153	5.154	0.314	6	2994	ZINC000257557766
0.7	-8.8	-7.5	0.58	2.010	0.282	5.352	0.470	6	2995	ZINC000100770480
0.7	-7.6	-7.2	0.23	1.793	0.441	3.279	1.282	6	2996	ZINC000034276222
0.7	-8.0	-7.8	0.22	1.651	0.503	4.505	1.780	6	2997	ZINC000015219339
0.7	-8.4	-8.2	0.22	1.757	0.298	3.727	0.735	6	2998	ZINC000100781974
0.7	-6.6	-6.4	0.17	2.043	0.285	3.787	0.308	6	2999	ZINC000077050241
0.7	-7.2	-6.9	0.18	2.543	0.516	4.619	1.498	6	3000	ZINC000027639972
0.7	-5.4	-5.1	0.23	1.911	0.565	3.632	0.723	6	3001	ZINC000002167283
0.7	-9.2	-8.5	0.33	1.954	0.483	3.994	1.250	6	3002	ZINC000257591085
0.7	-9.4	-8.8	0.54	1.532	0.252	4.428	1.440	6	3003	ZINC000015150645
0.7	-9.8	-8.3	1.11	2.081	0.302	4.447	1.747	5	3004	ZINC000014505838
0.7	-7.9	-7.3	0.40	2.111	0.416	4.928	1.322	6	3005	ZINC000014618750
0.7	-8.3	-8.0	0.22	2.327	0.631	3.411	0.373	6	3006	ZINC000014614800
0.7	-7.6	-7.4	0.14	1.845	0.425	3.393	1.165	6	3007	ZINC000085700605
0.7	-7.0	-6.7	0.15	2.708	0.763	3.442	0.619	6	3008	ZINC000001847459

0.7	-6.7	-6.4	0.31	2.197	0.885	2.825	0.958	6	3009	ZINC000000156863
0.7	-8.2	-7.5	0.47	1.345	0.670	3.383	1.742	6	3010	ZINC000014817866
0.7	-8.1	-7.6	0.29	2.167	0.324	3.857	0.674	6	3011	ZINC000085847757
0.7	-9.0	-8.6	0.32	1.767	0.225	4.727	0.307	6	3012	ZINC000014453720
0.7	-8.3	-7.9	0.20	2.211	0.653	4.478	1.583	6	3013	ZINC000013704159
0.7	-6.2	-6.0	0.16	2.279	0.520	3.439	0.600	6	3014	ZINC000014616890
0.7	-10.0	-9.4	0.29	1.148	0.571	3.013	0.605	6	3015	ZINC000100781035
0.7	-8.4	-8.1	0.18	2.612	0.390	4.000	0.484	6	3016	ZINC000085589364
0.7	-9.5	-8.1	1.01	1.827	0.318	5.647	0.084	5	3017	ZINC000257474046
0.7	-7.0	-6.5	0.28	2.893	0.454	4.388	0.251	6	3018	ZINC000001850864
0.7	-5.6	-5.4	0.18	2.026	0.505	2.883	0.458	6	3019	ZINC000001648304
0.7	-5.7	-5.6	0.07	2.359	1.177	3.704	0.839	6	3020	ZINC000095619760
0.7	-6.2	-5.9	0.13	1.740	0.184	3.484	0.225	6	3021	ZINC000015274293
0.7	-9.3	-8.5	0.38	1.646	0.240	2.797	0.293	6	3022	ZINC000014682734
0.7	-7.6	-7.2	0.28	1.270	0.393	3.792	0.922	6	3023	ZINC000000392595

0.7	-6.0	-5.9	0.10	2.657	0.367	4.225	0.505	6	3024	ZINC000001708200
0.7	-6.1	-5.9	0.13	2.568	0.493	3.193	0.633	6	3025	ZINC000002009976
0.7	-6.4	-6.0	0.18	1.936	0.109	3.713	0.858	6	3026	ZINC000014588466
0.7	-9.6	-9.0	0.33	1.946	0.753	4.759	0.949	6	3027	ZINC000000388658
0.7	-7.5	-7.3	0.21	1.650	0.344	2.992	0.986	6	3028	ZINC000209934384
0.7	-7.0	-6.7	0.15	1.056	0.370	3.507	0.966	6	3029	ZINC000000967794
0.7	-7.4	-7.2	0.13	2.408	0.650	3.481	1.104	6	3030	ZINC000003869639
0.7	-4.9	-4.8	0.19	1.210	0.599	2.527	0.300	6	3031	ZINC000002383464
0.7	-8.0	-7.8	0.20	1.818	0.155	5.099	0.911	6	3032	ZINC000004543890
0.7	-8.8	-7.6	0.67	1.936	0.144	5.456	0.490	6	3033	ZINC000012496559
0.7	-6.0	-5.9	0.11	1.938	0.646	3.074	0.117	6	3034	ZINC000005651182
0.7	-7.6	-7.1	0.40	2.056	1.120	3.536	1.409	6	3035	ZINC000000967333
0.7	-8.4	-7.8	0.42	1.523	0.563	3.533	1.764	6	3036	ZINC000014720695
0.7	-8.3	-8.0	0.20	2.092	0.540	4.288	0.948	6	3037	ZINC000003639975
0.7	-8.7	-8.4	0.21	2.437	0.603	3.984	1.689	6	3038	ZINC000004228273

0.7	-7.1	-6.8	0.19	3.056	0.360	4.421	0.224	6	3039	ZINC000001666982
0.7	-8.1	-7.8	0.23	1.272	0.139	3.674	0.710	6	3040	ZINC000059193058
0.7	-6.8	-6.7	0.09	1.406	0.281	5.043	0.476	6	3041	ZINC000001669521
0.7	-7.5	-7.2	0.20	1.325	0.270	2.804	0.348	6	3042	ZINC000001081099
0.7	-8.3	-7.0	0.81	1.660	0.344	4.030	1.601	6	3043	ZINC000014720697
0.7	-8.7	-8.6	0.11	1.377	0.691	2.645	0.652	6	3044	ZINC000004098881
0.7	-8.3	-8.0	0.15	1.478	0.422	4.033	2.186	6	3045	ZINC000014690454
0.7	-6.4	-6.1	0.20	3.010	0.516	5.415	0.732	6	3046	ZINC000050027561
0.7	-7.0	-6.6	0.22	1.909	0.380	2.673	0.511	6	3047	ZINC000069485773
0.7	-10.8	-10.0	0.83	1.851	0.521	5.464	1.214	6	3048	ZINC000257456806
0.7	-8.4	-7.7	0.40	1.829	0.360	5.939	0.895	6	3049	ZINC000257522824
0.7	-5.3	-5.0	0.17	1.398	0.505	3.967	0.382	6	3050	ZINC000001648281
0.7	-9.8	-9.1	0.49	1.443	0.401	4.434	1.522	6	3051	ZINC000100824255
0.7	-7.6	-7.3	0.22	1.883	0.266	3.383	0.567	6	3052	ZINC000001693693
0.7	-6.9	-6.7	0.15	1.936	0.589	2.955	0.691	6	3053	ZINC000001683675

0.7	-7.6	-7.3	0.16	2.480	0.557	5.293	0.757	6	3054	ZINC000014616307
0.7	-7.9	-7.6	0.20	2.147	0.944	4.118	0.432	6	3055	ZINC000000156635
0.7	-8.3	-7.9	0.29	1.583	0.132	3.543	0.375	6	3056	ZINC000013382502
0.7	-6.6	-6.3	0.17	1.825	0.298	4.743	2.172	6	3057	ZINC000095621422
0.7	-8.5	-7.9	0.37	1.703	0.397	3.548	1.452	6	3058	ZINC000015160974
0.7	-7.9	-7.9	0.05	1.782	0.253	3.232	0.663	6	3059	ZINC000014616539
0.7	-5.7	-5.5	0.09	1.834	0.150	4.098	0.513	6	3060	ZINC000002564113
0.7	-8.7	-8.6	0.08	1.346	0.536	3.123	1.774	6	3061	ZINC000261499720
0.7	-9.2	-8.7	0.50	1.813	0.136	4.394	1.640	6	3062	ZINC000000898283
0.7	-7.9	-7.5	0.29	1.656	0.602	2.253	0.614	6	3063	ZINC000001591842
0.7	-7.8	-7.6	0.20	1.627	0.252	3.965	0.770	6	3064	ZINC000001595414
0.7	-7.6	-7.0	0.27	1.815	0.375	4.505	1.133	6	3065	ZINC000014684363
0.7	-10.6	-9.0	1.03	1.595	0.223	4.883	0.731	6	3066	ZINC000005732865
0.7	-6.5	-6.1	0.26	2.638	1.032	3.804	1.266	6	3067	ZINC000095620502
0.7	-9.2	-8.5	0.42	2.003	0.319	3.976	0.959	6	3068	ZINC000015153339

0.7	-8.9	-8.1	0.58	2.782	0.498	4.488	0.827	6	3069	ZINC000000004773
0.7	-6.6	-6.1	0.29	2.344	0.356	3.821	0.750	6	3070	ZINC000001529576
0.7	-8.8	-7.3	0.79	1.769	0.135	4.467	0.730	6	3071	ZINC000014828125
0.7	-7.3	-7.0	0.16	1.930	0.700	3.324	1.030	6	3072	ZINC000001850500
0.7	-6.6	-6.5	0.12	2.218	0.449	3.380	0.408	6	3073	ZINC000001699440
0.7	-6.2	-6.0	0.11	2.792	0.930	4.904	0.519	6	3074	ZINC000005133461
0.7	-9.0	-8.7	0.23	1.791	0.689	3.103	0.973	6	3075	ZINC000100778711
0.7	-9.9	-9.1	0.56	1.657	0.405	2.913	0.983	6	3076	ZINC000013461652
0.7	-6.2	-6.0	0.14	2.490	0.761	4.148	1.346	6	3077	ZINC000100776155
0.7	-5.5	-5.2	0.12	2.621	0.469	4.463	0.417	6	3078	ZINC000015204642
0.7	-7.1	-6.8	0.16	1.057	0.051	4.683	0.597	6	3079	ZINC000064219762
0.7	-7.2	-6.9	0.16	3.011	0.252	4.023	0.631	6	3080	ZINC000014614123
0.7	-5.7	-5.3	0.24	2.246	0.911	3.425	1.233	6	3081	ZINC000005844820
0.7	-8.4	-8.0	0.29	1.977	0.179	2.654	0.265	6	3082	ZINC000013380362
0.7	-5.4	-5.1	0.17	3.133	0.914	3.635	0.976	6	3083	ZINC000001556980

0.7	-6.9	-6.4	0.23	2.213	0.392	3.217	0.493	6	3084	ZINC000000391878
0.7	-9.4	-8.0	0.77	2.364	0.377	3.949	1.531	6	3085	ZINC000085923594
0.7	-8.4	-7.7	0.43	2.548	1.011	4.738	1.535	6	3086	ZINC000013307187
0.7	-8.0	-7.6	0.20	1.963	0.190	3.975	0.946	6	3087	ZINC000085491749
0.7	-8.3	-8.1	0.20	1.705	0.451	6.170	1.558	6	3088	ZINC000014690452
0.7	-8.2	-7.9	0.24	1.522	0.146	3.189	1.033	6	3089	ZINC000100781982
0.7	-7.6	-7.0	0.35	2.251	0.387	3.491	1.381	6	3090	ZINC000002526418
0.7	-7.1	-6.8	0.20	2.401	0.284	4.186	0.988	6	3091	ZINC000034183021
0.7	-8.9	-7.2	0.75	1.706	0.470	4.959	1.658	6	3092	ZINC000014643690
0.7	-5.7	-5.5	0.22	2.087	0.917	3.547	1.454	6	3093	ZINC000002041165
0.7	-9.9	-9.3	0.53	1.504	0.349	3.828	1.799	6	3094	ZINC000000057674
0.7	-8.9	-8.1	0.44	1.970	0.138	4.136	0.661	6	3095	ZINC000013371345
0.7	-7.3	-7.0	0.25	1.742	0.333	3.766	1.447	6	3096	ZINC000001681500
0.7	-6.2	-5.9	0.19	1.838	0.336	3.498	0.549	6	3097	ZINC000005159654
0.7	-6.5	-6.4	0.09	2.121	0.447	3.796	1.079	6	3098	ZINC000002040946

0.7	-9.4	-8.5	0.53	1.568	0.200	4.613	1.421	6	3099	ZINC000100783550
0.7	-8.1	-7.8	0.20	2.089	0.475	4.335	1.132	6	3100	ZINC000014496298
0.7	-9.0	-8.6	0.27	1.842	0.845	3.290	0.917	6	3101	ZINC000100778707
0.7	-8.4	-7.4	0.48	1.923	0.220	5.184	0.865	6	3102	ZINC000004097781
0.7	-7.2	-6.5	0.32	2.834	0.720	4.925	1.500	6	3103	ZINC000004556703
0.7	-5.7	-5.6	0.07	1.766	0.507	3.162	0.972	6	3104	ZINC000086050396
0.7	-9.8	-9.0	0.48	1.437	0.401	3.665	1.792	6	3105	ZINC000095620821
0.7	-9.7	-9.4	0.22	1.691	0.157	6.057	0.175	6	3106	ZINC000085795143
0.7	-8.2	-7.4	0.49	1.980	0.266	3.474	0.931	6	3107	ZINC000005767132
0.7	-8.3	-7.4	0.56	1.744	0.261	4.426	2.001	6	3108	ZINC000014717739
0.7	-9.4	-8.3	0.60	1.675	0.311	4.723	1.905	6	3109	ZINC000100783553
0.7	-7.4	-7.2	0.15	1.514	0.885	3.981	0.772	6	3110	ZINC000002041162
0.7	-6.0	-5.8	0.17	3.367	1.238	5.090	1.950	6	3111	ZINC000013433458
0.6	-7.0	-6.5	0.32	2.616	1.272	4.513	1.225	6	3112	ZINC000032163578
0.6	-8.1	-7.6	0.24	2.098	0.371	4.940	2.057	6	3113	ZINC000050027879

0.6	-8.5	-8.2	0.19	1.652	0.293	5.769	1.782	6	3114	ZINC000015249510
0.6	-8.3	-8.1	0.19	2.477	0.375	4.360	1.079	6	3115	ZINC000100772814
0.6	-7.3	-6.9	0.20	2.090	0.117	3.532	0.941	6	3116	ZINC000004720696
0.6	-7.1	-6.6	0.24	2.102	0.429	3.487	1.543	6	3117	ZINC000002384856
0.6	-7.8	-7.4	0.27	1.338	0.438	3.504	1.244	6	3118	ZINC000001999341
0.6	-5.3	-5.0	0.12	2.900	0.964	4.280	0.969	6	3119	ZINC000002019686
0.6	-7.6	-7.1	0.31	2.279	0.211	3.446	0.607	6	3120	ZINC000002243723
0.6	-9.1	-8.4	0.41	2.317	0.450	5.172	0.917	6	3121	ZINC000100777201
0.6	-6.7	-6.5	0.09	3.875	0.302	5.506	0.555	6	3122	ZINC000014583660
0.6	-10.5	-8.9	0.82	1.616	0.211	4.676	1.854	6	3123	ZINC000100781248
0.6	-6.7	-6.5	0.21	2.934	1.172	4.152	1.382	6	3124	ZINC000095618092
0.6	-7.3	-7.1	0.21	2.288	0.228	3.202	0.436	6	3125	ZINC000001589378
0.6	-9.3	-9.0	0.17	2.315	0.586	3.988	1.521	6	3126	ZINC000000056757
0.6	-7.2	-6.7	0.21	2.550	0.481	3.626	0.886	6	3127	ZINC000015119268
0.6	-6.4	-6.1	0.17	1.771	0.197	2.995	0.645	6	3128	ZINC000000135384

0.6	-8.0	-7.6	0.25	1.953	0.131	3.925	0.749	6	3129	ZINC000049181235
0.6	-5.5	-5.1	0.23	2.532	0.405	3.386	0.568	6	3130	ZINC000095618234
0.6	-9.2	-8.0	1.00	1.884	0.102	3.639	0.730	5	3131	ZINC000015059548
0.6	-5.7	-5.5	0.09	1.753	0.753	3.935	0.422	6	3132	ZINC000039167973
0.6	-9.5	-8.6	0.68	1.110	0.212	6.231	1.764	6	3133	ZINC000012402601
0.6	-7.9	-7.7	0.11	1.617	0.207	3.883	0.442	6	3134	ZINC000059560115
0.6	-8.3	-7.9	0.27	1.891	0.376	4.402	0.760	6	3135	ZINC000085954093
0.6	-6.9	-6.8	0.15	1.670	0.447	2.794	0.770	6	3136	ZINC000006488104
0.6	-4.9	-4.6	0.18	1.368	0.492	2.872	0.364	6	3137	ZINC000001845884
0.6	-7.4	-7.2	0.11	2.740	0.359	4.381	0.838	6	3138	ZINC000050027764
0.6	-8.1	-7.8	0.15	1.113	0.393	2.381	0.503	6	3139	ZINC000000968099
0.6	-7.6	-7.2	0.30	2.343	0.179	4.982	1.221	6	3140	ZINC000038439057
0.6	-7.8	-7.5	0.21	1.968	0.437	4.383	1.201	6	3141	ZINC000002036789
0.6	-8.8	-8.1	0.44	2.192	0.292	4.870	1.066	6	3142	ZINC000004098847
0.6	-9.0	-7.8	1.15	2.436	0.000	4.228	0.000	2	3143	ZINC000100778725

0.6	-8.1	-7.5	0.28	2.248	0.307	3.879	1.295	6	3144	ZINC000000057060
0.6	-10.4	-9.4	0.48	1.637	0.333	5.033	1.185	6	3145	ZINC000100781258
0.6	-8.7	-8.3	0.25	2.175	0.973	3.360	1.920	6	3146	ZINC000040165421
0.6	-11.1	-10.0	1.08	2.036	0.653	4.146	1.222	5	3147	ZINC000006092239
0.6	-5.9	-5.7	0.17	1.565	0.170	3.457	0.212	6	3148	ZINC000100773208
0.6	-8.7	-8.5	0.17	1.671	0.462	4.012	2.116	6	3149	ZINC000006525249
0.6	-7.8	-7.5	0.34	1.697	0.423	2.623	0.724	6	3150	ZINC000001622034
0.6	-9.2	-7.9	0.60	2.658	0.381	5.183	0.986	6	3151	ZINC000085993651
0.6	-6.0	-5.5	0.24	2.056	1.229	3.011	1.069	6	3152	ZINC000014438703
0.6	-10.0	-8.9	0.83	1.155	0.283	5.155	2.131	6	3153	ZINC000061926651
0.6	-8.6	-8.0	0.41	1.280	0.371	5.074	1.967	6	3154	ZINC000015260792
0.6	-10.2	-9.2	0.56	1.900	0.271	4.753	1.536	6	3155	ZINC000100829597
0.6	-7.6	-7.3	0.21	1.801	0.232	5.027	1.254	6	3156	ZINC000001532513
0.6	-9.4	-9.0	0.28	1.860	0.336	4.749	1.272	6	3157	ZINC000005812872
0.6	-6.3	-5.9	0.21	3.285	0.362	4.506	0.324	6	3158	ZINC000002391067

0.6	-6.6	-6.3	0.23	2.574	0.569	4.124	0.986	6	3159	ZINC000000967515
0.6	-8.8	-8.5	0.12	2.500	0.179	5.391	0.880	6	3160	ZINC000015113824
0.6	-8.1	-8.0	0.12	2.844	0.277	5.132	0.730	6	3161	ZINC000036478810
0.6	-6.7	-6.5	0.13	0.799	0.258	3.064	0.639	6	3162	ZINC000001692473
0.6	-10.2	-8.5	0.89	1.603	0.111	4.915	1.669	6	3163	ZINC000013308689
0.6	-6.1	-5.9	0.11	3.453	1.129	4.469	1.049	6	3164	ZINC000013413605
0.6	-7.0	-6.7	0.14	1.051	0.386	3.506	0.970	6	3165	ZINC000002019620
0.6	-7.2	-6.7	0.24	2.078	0.321	3.447	0.997	6	3166	ZINC000003869769
0.6	-7.8	-7.4	0.21	1.937	0.310	5.176	0.678	6	3167	ZINC000034961770
0.6	-6.7	-6.2	0.23	2.859	0.922	3.903	1.554	6	3168	ZINC000033798295
0.6	-9.0	-7.6	0.87	1.873	0.443	4.998	1.686	5	3169	ZINC000014820392
0.6	-10.7	-9.2	1.12	2.193	0.037	4.923	0.911	4	3170	ZINC000014618717
0.6	-9.9	-8.3	1.01	1.372	0.127	5.207	0.778	4	3171	ZINC000013384887
0.6	-6.5	-6.4	0.09	2.419	1.271	4.637	0.460	6	3172	ZINC000013458468
0.6	-8.2	-7.7	0.25	2.899	0.397	5.223	1.093	6	3173	ZINC000100014157

0.6	-6.7	-6.5	0.12	3.130	0.379	6.063	0.080	6	3174	ZINC000005273930
0.6	-3.8	-3.8	0.00	0.197	0.094	2.460	0.175	6	3175	ZINC000008214515
0.6	-7.5	-7.1	0.31	1.625	0.966	3.384	1.677	6	3176	ZINC000001680376
0.6	-9.0	-7.3	0.85	1.941	0.312	4.327	0.672	6	3177	ZINC000086010681
0.6	-9.6	-7.9	1.23	1.867	0.293	3.698	1.325	6	3178	ZINC000100823132
0.6	-5.5	-5.4	0.13	1.646	0.209	2.467	0.800	6	3179	ZINC000014592713
0.6	-10.5	-9.1	0.73	1.590	0.150	4.378	1.768	6	3180	ZINC000100781250
0.6	-9.3	-7.8	1.02	1.501	0.490	4.454	1.637	6	3181	ZINC000100775744
0.6	-7.2	-7.0	0.17	2.625	0.183	5.393	1.192	6	3182	ZINC000034349842
0.6	-7.4	-7.2	0.15	2.161	0.521	4.021	1.778	6	3183	ZINC000095617718
0.6	-7.8	-7.5	0.17	1.799	0.623	3.099	1.359	6	3184	ZINC000014859996
0.6	-8.6	-7.9	0.34	1.650	0.191	3.573	1.519	6	3185	ZINC000095619965
0.6	-6.5	-6.1	0.27	2.525	0.498	4.270	0.865	6	3186	ZINC000001576200
0.6	-7.9	-7.4	0.25	3.021	0.342	5.559	1.339	6	3187	ZINC000011677074
0.6	-8.8	-8.5	0.22	1.494	0.856	4.289	1.470	6	3188	ZINC000002557414

0.6	-6.3	-5.8	0.28	2.344	1.025	3.619	1.045	6	3189	ZINC000014590586
0.6	-8.1	-7.9	0.12	1.731	0.316	3.446	1.199	6	3190	ZINC000005434378
0.6	-9.2	-8.5	0.51	1.472	0.272	2.892	0.860	6	3191	ZINC000095617910
0.6	-9.7	-7.9	0.98	2.226	0.370	5.915	1.787	6	3192	ZINC000100777066
0.6	-6.9	-6.7	0.16	1.838	0.894	3.006	0.564	6	3193	ZINC000000896098
0.6	-6.2	-6.0	0.13	2.369	0.473	3.515	0.244	6	3194	ZINC000001850889
0.6	-6.2	-6.0	0.15	3.912	0.420	5.309	0.338	6	3195	ZINC000001578620
0.6	-8.8	-8.4	0.27	2.169	0.723	3.486	1.603	6	3196	ZINC000034290098
0.6	-7.8	-7.4	0.24	2.035	0.427	4.689	1.045	6	3197	ZINC000085592672
0.6	-8.7	-8.1	0.41	1.790	0.636	4.178	0.582	6	3198	ZINC000095618180
0.6	-9.0	-8.2	0.64	1.508	0.554	4.202	2.665	5	3199	ZINC000006484601
0.6	-8.1	-6.5	0.93	1.947	0.046	3.532	1.366	6	3200	ZINC000015248705
0.6	-10.0	-8.4	1.01	1.819	0.067	4.851	1.416	4	3201	ZINC000100782616
0.6	-7.9	-7.8	0.11	1.931	0.795	2.886	1.127	6	3202	ZINC000002556747
0.6	-6.1	-5.8	0.17	2.291	0.827	3.449	0.993	6	3203	ZINC000002516016

0.6	-8.4	-7.9	0.31	2.295	0.415	5.063	2.096	6	3204	ZINC000050027030
0.6	-7.0	-6.6	0.20	2.109	0.269	3.485	0.432	6	3205	ZINC000001578707
0.6	-10.1	-9.4	0.46	2.350	1.139	3.624	0.986	6	3206	ZINC000257394844
0.6	-8.6	-8.1	0.32	1.202	0.268	4.268	1.963	6	3207	ZINC000257449731
0.6	-8.2	-8.0	0.24	1.726	0.419	3.823	2.293	6	3208	ZINC000004096453
0.6	-9.3	-8.3	0.77	2.174	0.335	4.303	1.004	6	3209	ZINC000005762649
0.6	-4.9	-4.7	0.14	2.109	0.815	2.911	0.986	6	3210	ZINC000003079336
0.6	-6.5	-6.2	0.23	0.865	0.423	3.556	0.583	6	3211	ZINC000000388546
0.6	-6.7	-6.3	0.24	2.570	1.082	3.177	1.098	6	3212	ZINC000000157525
0.6	-5.9	-5.4	0.29	1.814	0.992	2.709	0.859	6	3213	ZINC000014495041
0.6	-6.1	-5.7	0.23	3.038	0.688	4.725	0.453	6	3214	ZINC000021999682
0.6	-7.8	-7.7	0.06	1.780	0.673	3.169	0.520	6	3215	ZINC000100071097
0.6	-6.5	-6.1	0.24	2.171	0.113	3.427	0.276	6	3216	ZINC000014588555
0.6	-8.8	-8.5	0.19	1.338	0.640	4.024	2.301	6	3217	ZINC000015218406
0.6	-9.3	-8.4	0.60	1.933	0.156	4.242	1.303	6	3218	ZINC000015061516

0.6	-8.6	-8.3	0.31	1.874	0.333	3.145	0.725	6	3219	ZINC000015248717
0.6	-6.4	-6.2	0.11	1.612	0.119	3.111	0.355	6	3220	ZINC000006037750
0.6	-5.6	-5.4	0.19	2.868	0.897	3.735	0.995	6	3221	ZINC000003649921
0.6	-5.7	-5.4	0.20	1.800	0.762	2.705	0.852	6	3222	ZINC000002556430
0.6	-5.1	-5.0	0.15	2.720	0.306	3.938	0.337	6	3223	ZINC000095619431
0.6	-7.1	-6.9	0.14	1.708	0.515	3.337	0.672	6	3224	ZINC000002573799
0.6	-6.9	-6.7	0.16	2.207	0.397	3.188	0.982	6	3225	ZINC000004556678
0.6	-8.2	-7.8	0.23	1.857	0.235	3.178	1.122	6	3226	ZINC000019850522
0.6	-9.5	-8.2	0.66	1.665	0.361	2.828	0.447	6	3227	ZINC000006070288
0.6	-8.6	-8.2	0.21	1.437	0.748	3.392	1.095	6	3228	ZINC000085643423
0.6	-7.0	-6.6	0.20	2.542	0.378	3.625	0.635	6	3229	ZINC000014588468
0.6	-9.1	-8.2	0.50	1.837	0.492	4.108	1.017	6	3230	ZINC000014776087
0.6	-8.1	-7.5	0.29	2.332	0.244	3.426	0.993	6	3231	ZINC000014713558
0.6	-9.3	-8.4	0.53	1.679	0.359	4.435	1.532	6	3232	ZINC000100783551
0.6	-6.3	-6.1	0.11	3.195	1.155	5.164	0.268	6	3233	ZINC000001531618

0.6	-5.8	-5.6	0.18	2.311	0.493	3.720	0.701	6	3234	ZINC000002173157
0.6	-9.3	-8.9	0.34	1.671	0.312	3.111	0.875	6	3235	ZINC000251666750
0.6	-6.4	-6.2	0.15	1.662	0.524	2.846	0.287	6	3236	ZINC000001723266
0.6	-5.7	-5.5	0.20	2.812	1.128	3.715	1.241	6	3237	ZINC000033953242
0.6	-8.7	-8.1	0.41	1.773	0.658	4.147	0.581	6	3238	ZINC000201708936
0.6	-7.0	-6.7	0.20	3.615	0.458	5.080	0.404	6	3239	ZINC000095618077
0.6	-9.8	-9.3	0.40	1.904	0.952	5.308	1.693	6	3240	ZINC000013376981
0.6	-9.5	-8.6	0.63	1.916	0.230	4.229	1.353	6	3241	ZINC000014415765
0.6	-6.8	-6.5	0.21	2.856	0.715	3.786	1.155	6	3242	ZINC000003861699
0.6	-5.5	-5.2	0.20	1.740	0.476	3.087	0.344	6	3243	ZINC000002010676
0.6	-8.3	-7.9	0.17	1.978	0.709	4.535	2.151	6	3244	ZINC000014825020
0.6	-7.7	-6.9	0.42	1.939	0.353	4.298	1.574	6	3245	ZINC000014556679
0.6	-10.2	-9.1	0.66	1.841	0.352	4.710	1.470	6	3246	ZINC000100829593
0.6	-10.1	-9.4	0.39	1.676	0.435	5.084	2.074	6	3247	ZINC000003881558
0.6	-9.7	-9.1	0.47	1.872	0.282	4.836	1.407	6	3248	ZINC000001636654

0.6	-7.5	-7.3	0.11	2.268	0.724	4.698	1.658	6	3249	ZINC000299888584
0.6	-7.0	-6.6	0.20	2.541	0.385	3.688	0.624	6	3250	ZINC000014588471
0.6	-8.9	-8.2	0.51	1.789	0.513	3.956	1.711	6	3251	ZINC000014758993
0.6	-8.2	-7.8	0.22	2.100	0.253	3.445	0.698	6	3252	ZINC000038613728
0.6	-10.0	-8.9	0.66	2.275	0.289	4.414	0.727	6	3253	ZINC000013385026
0.6	-10.2	-9.3	0.80	1.747	0.312	3.781	1.362	6	3254	ZINC000257802859
0.6	-7.0	-6.4	0.40	2.401	1.065	4.328	1.194	6	3255	ZINC000095619768
0.6	-8.1	-7.9	0.10	1.921	0.132	4.527	0.480	6	3256	ZINC000000388318
0.6	-8.5	-7.9	0.28	1.736	0.392	3.922	1.382	6	3257	ZINC000014768806
0.6	-6.6	-6.1	0.24	2.684	0.452	3.538	0.541	6	3258	ZINC000001699884
0.6	-7.9	-7.3	0.58	2.073	0.340	3.675	0.641	6	3259	ZINC000014827122
0.6	-9.7	-8.6	0.85	2.381	0.317	4.243	0.527	6	3260	ZINC000100053131
0.6	-5.5	-5.3	0.19	3.156	0.889	4.602	0.852	6	3261	ZINC000001850763
0.6	-4.3	-4.1	0.12	1.991	1.401	2.579	1.256	6	3262	ZINC000001692445
0.6	-6.6	-6.4	0.13	2.431	0.654	3.593	1.018	6	3263	ZINC000095618089

0.6	-5.9	-5.6	0.20	1.763	0.420	3.144	0.780	6	3264	ZINC000039374927
0.6	-9.6	-9.0	0.34	2.381	0.336	4.337	1.939	6	3265	ZINC000000000857
0.6	-7.6	-7.2	0.26	1.844	0.745	3.326	1.025	6	3266	ZINC000100828847
0.6	-5.8	-5.6	0.18	1.941	0.359	2.969	0.542	6	3267	ZINC000002026704
0.6	-9.4	-8.9	0.22	2.371	0.442	4.761	1.728	6	3268	ZINC000000056584
0.6	-8.6	-8.1	0.32	2.190	0.521	3.675	1.525	6	3269	ZINC000000143992
0.6	-5.9	-5.8	0.09	2.752	0.347	4.051	0.597	6	3270	ZINC000095620552
0.6	-8.6	-8.1	0.27	1.914	0.288	3.903	1.218	6	3271	ZINC000261498157
0.6	-8.1	-7.7	0.25	2.067	0.472	4.180	1.037	6	3272	ZINC000085886056
0.6	-6.0	-5.6	0.21	2.277	0.984	3.265	1.064	6	3273	ZINC000095618232
0.6	-6.9	-6.5	0.24	2.200	0.481	3.462	0.767	6	3274	ZINC000001850936
0.6	-10.0	-8.9	0.83	1.154	0.284	5.157	2.129	6	3275	ZINC000025723214
0.6	-6.6	-6.4	0.14	2.475	0.496	3.878	0.889	6	3276	ZINC000004228277
0.6	-7.5	-7.4	0.10	1.287	0.197	2.659	1.008	6	3277	ZINC000033830725
0.6	-6.6	-6.2	0.25	2.032	0.320	3.802	0.953	6	3278	ZINC000034383086

0.6	-8.0	-7.4	0.32	2.058	0.327	4.677	0.356	6	3279	ZINC000059588329
0.6	-10.3	-8.8	1.19	1.917	0.560	3.916	1.513	5	3280	ZINC000100780828
0.6	-8.9	-8.0	0.46	1.841	0.354	3.666	1.434	6	3281	ZINC000006070283
0.6	-7.5	-7.2	0.20	1.989	0.665	2.522	0.799	6	3282	ZINC000000136391
0.6	-5.4	-4.9	0.27	2.413	0.790	3.231	1.121	6	3283	ZINC000002034319
0.6	-6.0	-5.8	0.15	2.032	0.711	3.636	0.774	6	3284	ZINC000095618315
0.6	-9.2	-8.7	0.48	1.946	0.337	4.313	0.848	6	3285	ZINC000014453943
0.6	-7.3	-7.1	0.11	2.109	0.450	2.734	0.754	6	3286	ZINC000008577164
0.6	-8.2	-7.9	0.15	2.185	0.417	4.210	1.080	6	3287	ZINC000002507487
0.6	-7.5	-7.1	0.24	2.066	0.564	4.675	1.195	6	3288	ZINC000001532175
0.6	-5.3	-4.9	0.18	1.979	0.452	3.145	0.397	6	3289	ZINC000029786610
0.6	-9.5	-8.4	0.65	1.513	0.089	4.768	1.708	6	3290	ZINC000005339053
0.6	-6.4	-6.2	0.11	1.748	0.683	3.387	0.386	6	3291	ZINC000001648223
0.6	-7.5	-7.3	0.20	1.668	0.715	3.533	0.419	6	3292	ZINC000013480058
0.6	-9.4	-9.0	0.43	2.069	0.358	4.372	1.702	6	3293	ZINC000014774634

0.6	-6.8	-6.7	0.07	1.720	0.470	3.724	0.597	6	3294	ZINC000086034071
0.6	-9.3	-8.5	0.46	1.474	0.227	3.152	0.443	6	3295	ZINC000004098725
0.6	-6.1	-5.7	0.20	4.042	1.523	5.725	1.745	6	3296	ZINC000001656421
0.6	-6.7	-6.4	0.19	2.521	0.967	3.922	1.782	6	3297	ZINC000095618093
0.6	-8.2	-7.7	0.27	1.611	0.412	3.919	1.280	6	3298	ZINC000014637059
0.6	-9.1	-7.7	0.82	2.862	0.135	5.005	0.927	6	3299	ZINC000014559010
0.6	-9.3	-7.9	0.97	2.004	0.427	4.672	1.121	6	3300	ZINC000013383386
0.6	-6.8	-6.7	0.09	2.138	0.426	2.795	0.292	6	3301	ZINC000001532018
0.6	-6.2	-6.1	0.09	2.660	0.783	4.879	0.406	6	3302	ZINC000001693596
0.6	-6.8	-6.5	0.16	2.100	0.447	2.638	0.431	6	3303	ZINC000001530092
0.6	-8.1	-7.9	0.14	1.186	0.415	2.599	0.200	6	3304	ZINC000000968101
0.6	-8.6	-8.1	0.33	1.873	0.162	4.334	0.596	6	3305	ZINC000015214565
0.6	-8.8	-7.4	0.75	1.560	0.262	3.689	1.197	6	3306	ZINC000104370405
0.6	-7.2	-6.9	0.26	1.715	0.382	3.900	1.345	6	3307	ZINC000095619684
0.6	-6.4	-6.2	0.09	1.526	0.316	2.901	0.681	6	3308	ZINC000001626971

0.6	-7.2	-7.0	0.17	1.844	0.672	3.456	0.952	6	3309	ZINC000001850495
0.6	-8.8	-8.3	0.29	2.128	0.621	2.934	1.004	6	3310	ZINC000001638008
0.6	-8.8	-8.5	0.21	1.890	0.442	4.648	2.020	6	3311	ZINC000001704484
0.6	-6.8	-6.5	0.15	2.265	0.311	3.503	1.060	6	3312	ZINC000001540301
0.6	-6.3	-5.9	0.23	2.515	0.446	3.832	0.958	6	3313	ZINC000001637976
0.6	-10.6	-9.7	0.50	1.602	0.344	4.633	1.644	6	3314	ZINC000257486692
0.6	-8.5	-8.2	0.16	1.678	0.719	4.693	0.407	6	3315	ZINC000000968466
0.6	-6.9	-6.5	0.26	2.487	0.920	3.660	0.835	6	3316	ZINC000000388061
0.6	-7.0	-6.7	0.20	2.529	0.412	3.669	0.652	6	3317	ZINC000257609908
0.6	-9.4	-8.6	0.44	1.407	0.160	4.099	0.712	6	3318	ZINC000257470337
0.6	-5.6	-5.5	0.07	1.357	0.813	2.592	0.600	6	3319	ZINC000090375175
0.6	-8.0	-7.7	0.32	2.233	0.646	5.297	1.624	6	3320	ZINC000033650329
0.6	-6.5	-5.9	0.43	3.154	0.528	4.762	0.808	6	3321	ZINC000001575510
0.6	-8.1	-7.4	0.31	2.016	0.241	5.990	1.259	6	3322	ZINC000008215630
0.6	-7.4	-7.1	0.30	1.459	0.340	3.252	0.398	6	3323	ZINC000001850974

0.6	-6.7	-6.3	0.20	1.162	0.161	3.737	0.776	6	3324	ZINC000257559499
0.6	-7.4	-7.2	0.13	2.271	0.334	4.197	0.636	6	3325	ZINC000031706719
0.6	-7.1	-6.6	0.23	1.715	0.329	4.195	0.975	6	3326	ZINC000257441136
0.6	-7.5	-7.3	0.24	2.507	0.172	5.050	0.491	6	3327	ZINC000005273846
0.6	-6.9	-6.7	0.11	2.084	0.399	3.334	1.035	6	3328	ZINC000033992256
0.6	-9.2	-8.7	0.32	1.499	0.167	4.442	2.128	6	3329	ZINC000100783060
0.6	-7.4	-7.2	0.14	1.052	0.270	2.617	0.241	6	3330	ZINC000100018136
0.6	-6.0	-5.7	0.18	1.762	0.277	3.201	0.815	6	3331	ZINC000100024297
0.6	-7.3	-7.1	0.10	3.035	0.416	3.848	0.392	6	3332	ZINC000100361515
0.6	-7.9	-7.7	0.16	2.323	0.552	3.712	1.505	6	3333	ZINC000100782471
0.6	-5.7	-5.6	0.15	2.816	0.979	4.248	1.246	6	3334	ZINC000031284479
0.6	-11.0	-9.8	1.25	2.022	0.000	4.175	0.000	2	3335	ZINC000014814266
0.6	-8.1	-7.6	0.30	2.164	0.788	4.445	1.866	6	3336	ZINC000004800919
0.6	-7.4	-7.1	0.14	1.514	0.264	4.229	1.403	6	3337	ZINC000100820651
0.6	-8.5	-7.7	0.56	2.524	0.937	3.800	1.420	6	3338	ZINC000002170233

0.6	-9.3	-7.9	0.66	1.495	0.372	4.212	1.278	6	3339	ZINC000257481852
0.6	-5.9	-5.6	0.18	2.671	0.619	3.419	0.788	6	3340	ZINC000032153274
0.6	-9.4	-9.2	0.15	1.315	0.611	5.580	1.946	6	3341	ZINC000100825074
0.6	-7.8	-7.6	0.21	1.830	0.473	3.553	1.019	6	3342	ZINC000060037124
0.6	-7.6	-7.5	0.16	1.836	0.525	2.483	0.337	6	3343	ZINC000030731188
0.6	-8.3	-8.1	0.13	1.856	0.787	4.166	2.181	6	3344	ZINC000012493443
0.6	-7.7	-7.4	0.14	2.028	0.194	5.274	0.653	6	3345	ZINC000027645272
0.6	-7.9	-7.7	0.12	1.832	0.450	4.678	0.572	6	3346	ZINC000062233813
0.6	-6.2	-6.0	0.09	1.448	0.274	3.338	0.265	6	3347	ZINC000085947594
0.6	-6.6	-6.2	0.21	1.511	0.235	3.971	1.733	6	3348	ZINC000095619617
0.6	-5.2	-4.9	0.21	1.131	0.337	2.852	0.286	6	3349	ZINC000001699558
0.6	-7.3	-6.9	0.20	1.916	0.578	2.960	1.787	6	3350	ZINC000004533995
0.6	-8.6	-8.1	0.32	1.207	0.274	4.267	1.963	6	3351	ZINC000257449733
0.6	-6.7	-6.5	0.12	2.259	0.569	3.957	1.009	6	3352	ZINC000031439572
0.6	-7.4	-7.1	0.28	1.913	0.855	2.735	0.709	6	3353	ZINC000034499765

0.6	-8.2	-7.8	0.28	2.168	0.300	5.502	1.040	6	3354	ZINC000027638928
0.6	-7.1	-6.7	0.18	1.811	0.346	4.881	0.746	6	3355	ZINC000100820648
0.6	-7.3	-7.0	0.22	2.209	0.590	4.472	1.485	6	3356	ZINC000003871295
0.6	-5.8	-5.6	0.10	2.354	0.552	3.393	0.941	6	3357	ZINC000013549379
0.6	-9.4	-8.8	0.28	2.070	0.392	4.834	1.966	6	3358	ZINC000014806934
0.6	-6.7	-6.4	0.23	2.679	0.562	3.464	0.984	6	3359	ZINC000000157490
0.6	-7.6	-7.2	0.23	1.630	0.179	2.197	0.264	6	3360	ZINC000001576267
0.6	-6.5	-6.1	0.21	1.957	0.840	3.343	1.356	6	3361	ZINC000005761930
0.6	-6.8	-6.5	0.12	1.771	0.627	3.197	0.493	6	3362	ZINC000005688565
0.6	-7.8	-7.3	0.29	2.036	0.754	3.105	1.033	6	3363	ZINC000002545403
0.6	-7.8	-7.7	0.07	1.666	0.464	3.497	0.490	6	3364	ZINC000001693280
0.6	-6.7	-6.1	0.32	1.743	0.248	3.598	0.765	6	3365	ZINC000014438725
0.6	-8.2	-7.4	0.39	2.028	0.312	5.276	1.480	6	3366	ZINC000027645250
0.6	-8.4	-7.4	0.54	2.038	0.483	4.087	1.549	5	3367	ZINC000006031379
0.6	-7.1	-6.9	0.16	1.876	0.263	3.480	0.337	6	3368	ZINC000001850768

0.6	-7.6	-7.4	0.10	1.656	0.261	3.224	0.861	6	3369	ZINC000034961804
0.6	-6.7	-6.4	0.15	2.004	0.198	4.149	0.486	6	3370	ZINC000014583658
0.6	-6.7	-6.4	0.21	2.240	0.516	4.048	0.298	6	3371	ZINC000001577444
0.6	-7.9	-7.5	0.22	2.899	0.337	4.329	0.880	6	3372	ZINC000013828017
0.6	-10.6	-9.1	0.95	1.853	0.288	4.304	1.501	6	3373	ZINC000014686331
0.6	-9.3	-7.9	0.99	2.198	0.439	4.339	0.891	6	3374	ZINC000100780637
0.6	-7.3	-6.7	0.27	1.881	0.499	4.143	1.375	6	3375	ZINC000012496868
0.6	-6.7	-6.5	0.11	1.860	0.636	3.384	1.050	6	3376	ZINC000012495662
0.6	-9.9	-9.5	0.35	1.553	0.342	3.709	1.065	6	3377	ZINC000095619159
0.6	-8.6	-8.4	0.14	1.546	0.124	4.323	1.994	6	3378	ZINC000015218412
0.6	-8.5	-7.9	0.33	1.686	0.149	4.122	0.635	6	3379	ZINC000014829563
0.6	-7.0	-6.8	0.09	1.776	0.362	4.882	2.199	6	3380	ZINC000014439791
0.6	-6.7	-6.5	0.08	2.270	0.958	4.323	0.707	6	3381	ZINC000018125667
0.6	-5.3	-5.0	0.20	2.498	1.020	3.808	0.725	6	3382	ZINC000001849645
0.6	-5.8	-5.5	0.15	2.072	0.621	3.656	0.208	6	3383	ZINC000005822039

0.6	-8.4	-8.1	0.23	1.567	0.523	3.482	1.296	6	3384	ZINC000085880175
0.6	-8.3	-7.8	0.33	2.203	0.703	4.527	0.681	6	3385	ZINC000000900156
0.6	-9.7	-8.6	0.81	1.914	0.271	4.283	0.580	6	3386	ZINC000015219706
0.6	-9.3	-9.0	0.23	1.545	0.728	5.295	1.849	6	3387	ZINC000257619164
0.6	-8.6	-7.8	0.41	1.815	0.253	4.914	0.968	6	3388	ZINC000008860487
0.6	-5.8	-5.7	0.08	2.469	0.583	3.300	0.723	6	3389	ZINC000022066124
0.6	-7.5	-7.4	0.08	1.557	0.514	3.778	1.293	6	3390	ZINC000004521470
0.6	-8.5	-7.9	0.41	2.227	0.459	4.440	1.831	6	3391	ZINC000005274045
0.6	-7.0	-6.7	0.16	1.056	0.372	3.514	0.965	6	3392	ZINC000000967562
0.6	-5.8	-5.6	0.12	2.018	0.702	3.236	1.115	6	3393	ZINC000003649505
0.6	-7.4	-7.1	0.18	2.178	0.944	3.756	0.714	6	3394	ZINC000033951475
0.6	-9.8	-9.3	0.47	1.577	0.257	4.627	1.519	6	3395	ZINC000004098704
0.6	-6.9	-6.7	0.13	1.797	0.521	2.792	1.216	6	3396	ZINC000050027440
0.6	-5.7	-5.2	0.26	3.440	0.132	4.450	0.243	6	3397	ZINC000002039800
0.6	-8.5	-8.0	0.39	1.868	0.601	3.835	1.584	6	3398	ZINC000257413646

0.6	-8.7	-8.5	0.17	1.975	1.231	4.491	1.251	6	3399	ZINC000000899119
0.6	-9.0	-8.0	0.94	1.744	0.236	5.776	1.355	6	3400	ZINC000100778835
0.6	-10.6	-9.2	1.12	1.471	0.491	2.559	0.840	5	3401	ZINC000257547167
0.6	-7.6	-7.1	0.34	1.893	0.937	3.904	1.288	6	3402	ZINC000012495217
0.6	-10.6	-10.0	0.51	1.829	0.456	5.742	1.264	6	3403	ZINC000100823548
0.6	-7.0	-6.8	0.15	2.570	0.914	3.803	0.928	6	3404	ZINC000005965554
0.6	-9.0	-8.4	0.35	1.656	0.188	5.976	1.538	6	3405	ZINC000100825070
0.6	-7.2	-7.0	0.13	2.506	0.517	5.329	1.566	6	3406	ZINC000040472688
0.6	-7.8	-7.0	0.36	1.746	0.110	4.284	0.704	6	3407	ZINC000014859992
0.6	-7.5	-7.3	0.17	1.697	0.710	3.069	0.649	6	3408	ZINC000013480059
0.6	-7.9	-7.6	0.23	2.458	0.439	4.472	0.910	6	3409	ZINC000013356169
0.6	-7.7	-7.2	0.30	1.606	0.435	3.316	1.234	6	3410	ZINC000005510218
0.6	-10.2	-8.8	1.45	2.000	0.000	3.163	0.000	2	3411	ZINC000015115198
0.6	-6.2	-6.1	0.13	1.919	0.524	3.388	0.341	6	3412	ZINC000100828315
0.6	-8.7	-8.4	0.24	1.502	0.307	2.894	0.215	6	3413	ZINC000013519962

0.6	-7.5	-6.8	0.52	1.894	0.665	3.848	1.381	6	3414	ZINC000100776361
0.6	-5.9	-5.6	0.15	3.266	0.632	4.037	0.752	6	3415	ZINC000000896810
0.6	-5.9	-5.5	0.21	2.887	0.543	3.716	0.608	6	3416	ZINC000005819403
0.6	-10.0	-8.7	1.00	1.255	0.175	6.042	1.838	6	3417	ZINC000004096636
0.6	-10.4	-9.7	0.57	1.641	0.646	4.176	0.887	6	3418	ZINC000230084503
0.6	-7.2	-6.8	0.17	2.028	0.316	4.939	0.703	6	3419	ZINC000005819939
0.6	-9.3	-8.6	0.40	1.963	0.780	5.274	1.694	6	3420	ZINC000014758886
0.6	-6.8	-6.4	0.31	2.047	0.952	3.140	1.370	6	3421	ZINC000008215740
0.6	-9.1	-8.4	0.37	1.900	0.148	4.981	1.109	6	3422	ZINC000004521870
0.6	-7.2	-6.8	0.24	2.395	0.546	3.235	0.659	6	3423	ZINC000005385354
0.6	-8.1	-7.9	0.12	1.212	0.402	2.617	0.200	6	3424	ZINC000000968100
0.6	-6.9	-6.8	0.13	1.929	0.266	3.895	0.874	6	3425	ZINC000001689463
0.6	-8.4	-7.7	0.56	1.623	0.456	4.381	2.051	6	3426	ZINC000015111918
0.6	-9.7	-8.9	0.55	1.864	0.522	3.470	1.688	5	3427	ZINC000001532408
0.6	-6.4	-6.1	0.19	2.617	1.208	4.214	1.454	6	3428	ZINC000095620589

0.6	-6.2	-5.9	0.17	2.146	0.622	3.709	0.909	6	3429	ZINC000100828982
0.6	-5.8	-5.6	0.11	1.500	0.450	3.528	0.697	6	3430	ZINC000059727266
0.6	-7.1	-7.0	0.14	1.025	0.155	4.601	0.733	6	3431	ZINC000001704768
0.6	-9.4	-8.6	0.47	2.231	0.454	5.015	1.819	6	3432	ZINC000014806948
0.6	-7.1	-6.9	0.16	1.373	0.356	3.099	0.929	6	3433	ZINC000014489944
0.6	-7.2	-6.8	0.22	1.973	0.238	3.654	0.999	6	3434	ZINC000014587709
0.6	-7.4	-7.1	0.20	1.332	0.194	3.677	1.059	6	3435	ZINC000085759858
0.6	-7.9	-7.7	0.21	2.461	0.666	4.378	1.167	6	3436	ZINC000070454139
0.6	-5.8	-5.4	0.21	2.106	0.376	3.494	0.631	6	3437	ZINC000013380322
0.6	-9.0	-8.3	0.49	1.900	0.209	3.631	1.290	6	3438	ZINC000014682893
0.6	-6.8	-6.4	0.21	2.150	1.106	3.712	0.653	6	3439	ZINC000000895999
0.6	-8.4	-8.2	0.17	1.997	0.305	3.119	0.732	6	3440	ZINC000015255009
0.6	-6.9	-6.8	0.08	2.527	0.188	4.824	0.341	6	3441	ZINC000006020362
0.6	-8.5	-7.9	0.32	2.099	0.418	5.820	1.746	6	3442	ZINC000028631421
0.6	-8.5	-8.0	0.36	2.273	0.666	3.570	1.805	6	3443	ZINC000040472580

0.6	-9.1	-8.7	0.28	1.840	0.718	4.713	2.418	6	3444	ZINC000000391976
0.6	-7.9	-7.4	0.40	1.371	0.156	2.613	1.203	6	3445	ZINC000001694875
0.6	-10.6	-9.7	0.52	1.604	0.342	4.797	1.279	6	3446	ZINC000257486690
0.6	-8.8	-7.4	0.81	1.836	0.394	4.726	1.613	6	3447	ZINC000257455463
0.6	-8.4	-7.2	0.63	1.977	0.557	5.565	1.574	6	3448	ZINC000013783220
0.6	-8.2	-7.3	0.55	2.606	0.512	5.019	1.907	6	3449	ZINC000014826091
0.6	-9.1	-7.8	0.66	1.805	0.349	4.871	0.679	6	3450	ZINC000015263495
0.6	-6.7	-6.4	0.16	2.365	0.296	4.724	1.253	6	3451	ZINC000001572037
0.6	-8.0	-7.4	0.30	1.832	0.400	2.630	0.284	6	3452	ZINC000095619946
0.6	-7.4	-7.1	0.23	1.749	0.328	4.072	1.694	6	3453	ZINC000013531922
0.6	-9.9	-8.5	1.18	2.040	0.480	4.613	0.687	3	3454	ZINC000004097860
0.6	-8.1	-7.7	0.22	1.822	0.164	4.678	1.568	6	3455	ZINC000015046814
0.6	-8.0	-7.4	0.30	1.867	0.433	2.637	0.347	6	3456	ZINC000261499647
0.6	-7.7	-7.2	0.34	2.630	0.561	4.222	1.222	6	3457	ZINC000013481684
0.6	-10.0	-9.0	0.79	1.091	0.204	6.127	1.705	6	3458	ZINC000006067627

0.6	-6.4	-6.0	0.21	2.407	0.458	3.305	0.787	6	3459	ZINC000039069556
0.6	-7.8	-7.5	0.18	1.767	0.461	4.330	1.291	6	3460	ZINC000015165678
0.6	-7.7	-7.3	0.28	2.130	0.796	3.378	1.498	6	3461	ZINC000001849896
0.6	-8.3	-8.1	0.14	2.023	0.137	5.360	0.455	6	3462	ZINC000014414830
0.6	-6.9	-6.5	0.21	2.456	0.392	5.267	0.293	6	3463	ZINC000038654414
0.6	-6.7	-6.5	0.16	1.260	0.411	2.849	0.307	6	3464	ZINC000001592406
0.6	-7.2	-6.8	0.22	2.254	0.869	3.432	0.671	6	3465	ZINC000006071999
0.6	-8.7	-8.2	0.33	3.267	0.349	6.045	0.714	6	3466	ZINC000005114091
0.6	-8.7	-8.5	0.21	1.690	0.627	3.516	1.328	6	3467	ZINC000015113755
0.6	-5.7	-5.4	0.16	2.775	1.105	3.810	1.221	6	3468	ZINC000001850847
0.6	-7.2	-6.9	0.23	2.238	0.684	3.437	0.303	6	3469	ZINC000095620680
0.6	-6.4	-6.2	0.13	2.828	0.406	3.868	0.586	6	3470	ZINC000095619763
0.6	-6.1	-5.8	0.15	2.451	0.819	3.784	0.466	6	3471	ZINC000005649494
0.6	-6.5	-6.0	0.29	2.096	0.393	3.359	0.822	6	3472	ZINC000014588463
0.6	-6.6	-6.3	0.16	2.793	0.248	4.344	0.345	6	3473	ZINC000040470479

0.6	-6.2	-5.9	0.15	2.092	0.549	3.996	0.682	6	3474	ZINC000261499752
0.6	-8.5	-8.3	0.18	2.511	0.912	4.661	1.599	6	3475	ZINC000034290100
0.6	-5.2	-4.8	0.19	2.627	0.667	3.127	0.857	6	3476	ZINC000001850318
0.6	-8.7	-8.2	0.27	2.216	0.743	5.111	1.197	6	3477	ZINC000014445259
0.6	-6.6	-6.5	0.09	2.785	1.005	3.820	1.206	6	3478	ZINC000014583662
0.6	-8.6	-7.6	0.73	1.965	0.435	4.626	1.415	6	3479	ZINC000013307197
0.6	-7.0	-6.8	0.14	1.845	0.746	3.933	1.258	6	3480	ZINC000014822497
0.6	-7.6	-7.2	0.21	2.744	0.846	4.272	0.750	6	3481	ZINC000034278894
0.6	-7.2	-6.9	0.21	2.411	0.748	3.591	1.269	6	3482	ZINC000095908875
0.6	-7.3	-7.2	0.07	2.399	0.382	4.623	0.542	6	3483	ZINC000050027624
0.6	-7.7	-7.6	0.07	1.878	0.390	4.766	1.452	6	3484	ZINC000027647165
0.6	-8.1	-7.7	0.35	1.674	0.140	3.314	0.231	6	3485	ZINC000013382503
0.6	-5.6	-5.4	0.11	2.652	0.518	3.303	0.604	6	3486	ZINC000100029282
0.6	-10.1	-9.8	0.16	1.708	0.484	3.728	1.061	6	3487	ZINC000257657040
0.6	-8.9	-8.6	0.21	2.672	0.332	5.288	0.505	6	3488	ZINC000015121943

0.6	-6.0	-5.6	0.21	2.426	1.295	3.393	1.273	6	3489	ZINC000014507033
0.6	-7.6	-7.4	0.13	2.041	1.047	4.127	1.873	6	3490	ZINC000000157118
0.6	-8.5	-7.9	0.35	1.772	0.267	5.150	0.305	6	3491	ZINC000257517911
0.6	-6.9	-6.6	0.26	3.054	0.573	4.557	0.598	6	3492	ZINC000095619609
0.6	-6.0	-5.6	0.21	1.667	0.402	2.870	0.624	6	3493	ZINC000002567753
0.6	-6.5	-6.4	0.13	2.105	0.422	5.358	0.531	6	3494	ZINC000038654406
0.6	-7.1	-6.8	0.22	2.213	0.330	5.014	1.879	6	3495	ZINC000002047887
0.6	-8.3	-7.9	0.33	2.368	0.512	4.009	1.315	6	3496	ZINC000040471114
0.6	-6.2	-5.8	0.21	1.973	0.614	3.304	0.774	6	3497	ZINC000002030900
0.6	-8.3	-8.1	0.15	2.422	0.535	4.998	0.250	6	3498	ZINC000034286750
0.6	-6.6	-6.2	0.20	2.525	0.662	3.653	0.827	6	3499	ZINC000001594276
0.6	-9.0	-8.4	0.34	1.957	0.068	6.153	0.849	6	3500	ZINC000014438695
0.6	-7.7	-7.3	0.20	2.400	0.746	4.655	1.206	6	3501	ZINC000002572265
0.6	-7.4	-7.1	0.21	2.667	0.432	5.043	0.804	6	3502	ZINC000006031098
0.6	-7.5	-7.3	0.18	1.690	0.719	3.066	0.649	6	3503	ZINC000033830724

0.6	-6.8	-6.4	0.25	2.123	0.617	3.355	1.085	6	3504	ZINC000014588556
0.6	-9.5	-8.6	0.68	1.107	0.210	6.230	1.764	6	3505	ZINC000012402603
0.6	-7.9	-7.3	0.36	2.405	0.679	3.514	1.935	6	3506	ZINC000005116993
0.6	-6.6	-6.3	0.15	2.522	0.469	3.868	1.104	6	3507	ZINC000040470480
0.6	-6.2	-5.9	0.27	1.123	0.589	3.569	0.261	6	3508	ZINC000002040666
0.6	-9.0	-8.6	0.21	1.762	0.292	3.075	1.029	6	3509	ZINC000033771410
0.6	-8.0	-7.7	0.26	2.517	0.821	4.096	1.672	6	3510	ZINC000032839145
0.6	-7.3	-7.0	0.15	2.131	0.742	3.349	1.172	6	3511	ZINC000002015645
0.6	-6.7	-6.4	0.16	2.016	0.777	4.593	2.426	6	3512	ZINC000001529165
0.6	-8.8	-8.3	0.29	1.916	0.497	6.000	1.366	6	3513	ZINC000014811592
0.6	-7.3	-6.8	0.28	2.031	0.546	3.022	0.611	6	3514	ZINC000013379105
0.6	-8.5	-8.0	0.27	2.170	0.607	3.440	1.322	6	3515	ZINC000000057344
0.6	-5.6	-5.4	0.08	3.467	1.827	4.134	1.745	6	3516	ZINC000001631379
0.6	-6.7	-6.6	0.10	1.356	0.431	3.538	0.706	6	3517	ZINC000001699438
0.6	-6.0	-5.7	0.16	2.159	0.805	3.627	1.016	6	3518	ZINC000000119667

0.6	-6.6	-6.4	0.14	2.463	0.495	3.921	0.787	6	3519	ZINC000003861695
0.6	-5.8	-5.6	0.13	1.966	0.409	3.545	0.512	6	3520	ZINC000032166982
0.6	-8.8	-8.5	0.22	2.274	0.486	4.986	0.310	6	3521	ZINC000095620006
0.6	-9.2	-8.6	0.33	1.518	0.158	4.651	1.864	6	3522	ZINC000231215761
0.6	-6.6	-6.3	0.15	2.242	0.527	3.200	0.597	6	3523	ZINC000012153444
0.6	-7.9	-7.7	0.15	2.086	0.557	4.247	1.177	6	3524	ZINC000014588967
0.6	-10.4	-9.7	0.57	1.647	0.648	4.180	0.881	6	3525	ZINC000100779678
0.6	-5.7	-5.5	0.14	2.537	0.620	3.457	0.616	6	3526	ZINC000000409241
0.6	-7.9	-7.3	0.38	2.304	0.569	4.998	1.764	6	3527	ZINC000013373497
0.6	-7.1	-6.8	0.20	2.447	0.636	3.203	0.884	6	3528	ZINC000000153019
0.6	-10.0	-8.9	0.80	1.155	0.284	5.158	2.128	6	3529	ZINC000261496247
0.6	-7.2	-6.6	0.39	2.724	0.663	4.205	0.805	6	3530	ZINC000001849665
0.6	-7.8	-7.5	0.26	2.093	0.254	3.603	1.130	6	3531	ZINC000013307163
0.6	-7.1	-6.8	0.14	1.975	0.230	2.740	0.602	6	3532	ZINC000040479092
0.6	-9.5	-8.1	0.65	2.242	0.348	4.612	0.491	6	3533	ZINC000100150718

0.6	-6.5	-6.0	0.29	3.175	1.077	4.641	1.187	6	3534	ZINC000032151579
0.6	-8.2	-7.9	0.23	1.485	0.207	5.119	1.620	6	3535	ZINC000004655432
0.6	-6.2	-5.8	0.30	3.153	0.992	5.023	0.671	6	3536	ZINC000001531601
0.6	-7.4	-7.2	0.13	2.435	0.775	3.790	1.841	6	3537	ZINC000040470616
0.6	-7.3	-6.8	0.29	2.416	0.268	4.368	1.243	6	3538	ZINC000034183017
0.6	-6.2	-6.0	0.12	2.736	1.121	4.448	0.823	6	3539	ZINC000001731785
0.6	-8.7	-8.1	0.41	1.795	0.638	4.131	0.603	6	3540	ZINC000095618178
0.6	-10.2	-9.0	0.64	1.923	0.250	4.902	1.265	6	3541	ZINC000257564788
0.6	-10.6	-10.1	0.45	1.685	0.345	5.105	1.542	6	3542	ZINC000100823543
0.6	-8.5	-7.9	0.48	1.794	0.393	4.038	0.777	6	3543	ZINC000014441682
0.6	-5.6	-5.3	0.17	2.377	0.773	3.212	0.702	6	3544	ZINC000014438654
0.6	-7.4	-7.0	0.21	2.323	0.960	4.233	0.738	6	3545	ZINC000014764738
0.6	-6.6	-6.4	0.13	2.297	0.788	3.613	1.166	6	3546	ZINC000001663619
0.6	-6.2	-6.0	0.12	2.234	0.531	3.799	1.304	6	3547	ZINC000033639585
0.6	-6.3	-6.0	0.21	2.846	0.424	4.004	0.434	6	3548	ZINC000069485965

0.6	-5.7	-5.6	0.11	2.028	0.337	3.859	0.224	6	3549	ZINC000212675501
0.6	-5.8	-5.5	0.25	2.174	1.037	3.374	1.013	6	3550	ZINC000039374930
0.6	-5.3	-5.0	0.14	1.743	0.410	3.740	0.311	6	3551	ZINC000002034780
0.6	-6.3	-6.0	0.20	2.840	0.599	4.004	0.766	6	3552	ZINC000005647271
0.6	-6.2	-6.0	0.11	2.153	1.244	3.549	1.172	6	3553	ZINC000000155456
0.6	-6.6	-6.1	0.26	1.778	0.226	3.300	0.915	6	3554	ZINC000006067338
0.6	-9.0	-7.8	0.74	1.974	0.625	3.702	1.485	6	3555	ZINC000015161030
0.6	-5.7	-5.3	0.24	2.848	0.616	3.585	0.971	6	3556	ZINC000000896814
0.6	-7.3	-6.7	0.31	2.724	0.755	4.082	1.643	6	3557	ZINC000014947825
0.6	-6.3	-6.0	0.20	3.303	1.103	4.280	1.423	6	3558	ZINC000013543038
0.6	-5.8	-5.6	0.19	2.603	1.046	4.473	1.124	6	3559	ZINC000014444683
0.6	-8.2	-8.0	0.14	2.509	0.561	3.897	1.574	6	3560	ZINC000001644308
0.6	-6.6	-6.4	0.11	1.794	0.092	3.852	0.808	6	3561	ZINC000005179611
0.6	-9.2	-7.2	1.39	1.826	0.835	4.314	2.903	3	3562	ZINC000013451936
0.6	-6.3	-6.0	0.12	3.949	0.327	5.450	0.392	6	3563	ZINC000019364679

0.6	-7.5	-7.3	0.23	2.305	0.280	4.042	1.242	6	3564	ZINC000095617747
0.6	-6.6	-6.3	0.17	3.430	0.579	4.220	0.521	6	3565	ZINC000001597267
0.6	-6.5	-6.4	0.14	2.128	0.320	3.544	1.117	6	3566	ZINC000005220248
0.6	-8.6	-7.7	0.63	1.490	0.583	2.931	0.649	6	3567	ZINC000238787523
0.6	-9.4	-8.9	0.30	2.177	0.438	4.560	0.548	6	3568	ZINC000013412788
0.6	-9.3	-8.6	0.44	1.956	0.662	4.771	1.862	6	3569	ZINC000028361975
0.6	-6.3	-6.0	0.15	2.426	0.412	3.483	0.894	6	3570	ZINC000001849638
0.6	-6.6	-6.3	0.19	2.508	0.527	4.036	0.865	6	3571	ZINC000001482164
0.6	-8.3	-8.1	0.21	2.502	0.364	4.365	1.074	6	3572	ZINC000100772812
0.6	-4.8	-4.5	0.25	1.464	0.448	2.355	0.523	6	3573	ZINC000001995154
0.6	-8.5	-8.1	0.26	1.990	0.256	3.964	1.621	6	3574	ZINC000034264742
0.6	-8.2	-7.9	0.20	2.149	0.687	3.073	0.544	6	3575	ZINC000247798770
0.6	-6.6	-6.3	0.21	2.104	0.943	3.297	1.842	6	3576	ZINC000005820598
0.6	-9.6	-8.7	0.47	1.836	0.209	4.281	0.739	6	3577	ZINC000014453942
0.6	-8.7	-8.2	0.31	2.359	0.878	3.842	1.628	6	3578	ZINC000004095844

0.6	-6.3	-6.0	0.24	2.489	0.967	4.208	1.072	6	3579	ZINC000002041121
0.6	-7.0	-6.8	0.16	2.693	0.522	4.599	0.496	6	3580	ZINC000059408387
0.6	-9.2	-8.9	0.23	1.358	0.629	5.354	1.844	6	3581	ZINC000257534559
0.6	-5.9	-5.8	0.10	1.722	0.402	3.091	0.689	6	3582	ZINC000001693593
0.6	-5.9	-5.7	0.12	2.717	1.202	3.922	0.852	6	3583	ZINC000002018675
0.6	-8.0	-7.9	0.07	1.749	0.268	5.099	0.862	6	3584	ZINC000027638941
0.6	-8.0	-7.4	0.31	1.805	0.193	5.384	0.607	6	3585	ZINC000040165304
0.6	-8.8	-7.8	0.47	2.212	0.352	3.981	0.838	6	3586	ZINC000014687441
0.6	-6.2	-6.0	0.15	2.689	0.708	4.298	0.969	6	3587	ZINC000095620561
0.6	-6.6	-6.3	0.14	2.770	0.312	4.433	0.455	6	3588	ZINC000040470477
0.6	-8.5	-8.2	0.29	1.923	0.359	4.596	2.067	6	3589	ZINC000014919012
0.6	-9.0	-8.3	0.52	1.729	0.366	4.865	1.811	6	3590	ZINC000014919008
0.6	-7.4	-7.2	0.10	2.314	0.379	6.869	0.123	6	3591	ZINC000013382915
0.6	-6.2	-6.0	0.10	2.501	0.301	3.995	0.186	6	3592	ZINC000002387302
0.6	-10.4	-9.7	0.56	1.650	0.649	4.185	0.870	6	3593	ZINC000014586946

0.6	-6.0	-5.7	0.21	1.763	0.665	2.265	0.839	6	3594	ZINC000000001592
0.6	-7.6	-7.0	0.28	2.050	0.280	3.399	0.848	6	3595	ZINC000013333974
0.6	-8.4	-8.2	0.14	2.081	0.352	3.920	0.604	6	3596	ZINC000002037431
0.6	-7.3	-7.1	0.13	2.209	0.505	3.631	1.195	6	3597	ZINC000003870000
0.6	-7.8	-7.5	0.26	2.141	0.548	3.355	0.746	6	3598	ZINC000090225589
0.6	-6.5	-6.2	0.28	2.310	0.455	3.458	0.510	6	3599	ZINC000039061874
0.6	-9.0	-8.1	0.57	2.215	0.385	4.076	1.222	6	3600	ZINC000257554796
0.6	-5.9	-5.5	0.26	1.980	0.364	3.110	0.627	6	3601	ZINC000001693359
0.6	-6.3	-6.0	0.24	2.755	0.412	4.201	0.822	6	3602	ZINC000033843063
0.6	-8.6	-7.4	0.62	1.876	0.216	3.924	0.585	6	3603	ZINC000257741509
0.6	-6.3	-6.0	0.16	1.343	0.226	3.154	0.210	6	3604	ZINC000085947592
0.6	-6.6	-6.4	0.18	1.806	0.367	4.360	0.928	6	3605	ZINC000013327181
0.6	-6.0	-5.9	0.13	2.263	0.689	3.803	0.807	6	3606	ZINC000001675421
0.6	-6.9	-6.4	0.26	2.599	0.332	3.594	0.294	6	3607	ZINC000001597294
0.6	-7.4	-6.9	0.27	3.175	0.683	5.276	1.348	6	3608	ZINC000002018982

0.6	-8.2	-7.9	0.25	1.726	0.167	4.026	0.485	6	3609	ZINC000002169368
0.6	-6.2	-5.9	0.16	2.557	0.467	3.709	0.450	6	3610	ZINC000001843705
0.6	-7.6	-7.1	0.25	3.089	0.953	5.774	1.221	6	3611	ZINC000040472564
0.6	-7.6	-7.2	0.33	1.923	0.231	3.015	1.558	6	3612	ZINC000014613563
0.6	-7.4	-7.2	0.09	1.579	0.347	2.786	0.304	6	3613	ZINC000000120337
0.6	-8.1	-7.8	0.28	2.546	0.538	4.526	1.370	6	3614	ZINC000002384941
0.6	-7.7	-7.4	0.22	1.826	0.304	4.013	1.260	6	3615	ZINC000032821770
0.6	-10.2	-8.5	0.97	2.056	0.219	4.252	1.024	6	3616	ZINC000014775896
0.6	-9.0	-8.0	0.56	1.520	0.277	5.414	1.938	6	3617	ZINC000014778687
0.6	-5.8	-5.6	0.17	3.867	1.141	5.177	1.396	6	3618	ZINC000003869224
0.6	-7.8	-7.6	0.11	2.082	0.235	4.025	0.880	6	3619	ZINC000257433631
0.6	-8.0	-7.7	0.18	2.054	0.708	4.367	1.754	6	3620	ZINC000001850772
0.6	-8.0	-7.4	0.27	1.889	0.479	2.491	0.365	6	3621	ZINC000100826909
0.6	-6.5	-6.1	0.21	2.080	0.567	2.930	0.604	6	3622	ZINC000000164392
0.6	-7.3	-7.0	0.23	1.859	0.457	2.790	0.996	6	3623	ZINC000005167572

0.6	-6.5	-6.2	0.16	2.539	0.405	3.292	0.351	6	3624	ZINC000001747833
0.6	-5.1	-4.8	0.14	3.065	1.341	3.981	1.046	6	3625	ZINC000001840969
0.6	-6.8	-6.5	0.13	2.669	0.557	5.004	0.642	6	3626	ZINC000034277894
0.6	-10.3	-9.3	0.51	2.108	0.260	4.944	0.741	6	3627	ZINC000005996059
0.6	-8.1	-7.5	0.28	2.415	0.504	3.924	0.700	6	3628	ZINC000014708250
0.6	-9.0	-8.3	0.47	1.803	0.170	3.552	0.515	6	3629	ZINC000095618402
0.6	-8.0	-7.4	0.38	2.606	0.624	4.604	1.632	6	3630	ZINC000040470839
0.6	-6.8	-6.5	0.19	2.273	0.517	3.509	0.424	6	3631	ZINC000002574224
0.6	-7.6	-7.2	0.20	2.346	0.754	4.041	0.943	6	3632	ZINC000006031633
0.6	-5.9	-5.4	0.25	2.859	0.862	3.801	1.403	6	3633	ZINC000014593063
0.6	-8.2	-7.6	0.28	2.781	0.640	3.801	0.937	6	3634	ZINC000014447816
0.6	-7.4	-7.2	0.09	2.223	0.485	3.655	1.362	6	3635	ZINC000002047203
0.6	-9.3	-8.9	0.28	1.887	0.473	3.015	0.662	6	3636	ZINC000251666747
0.6	-6.1	-5.9	0.20	2.224	0.940	3.765	1.131	6	3637	ZINC000002578904
0.6	-7.7	-7.2	0.31	2.004	0.443	3.045	0.638	6	3638	ZINC000013311063

0.6	-9.0	-8.3	0.44	2.038	0.458	4.877	1.662	6	3639	ZINC000014758849
0.6	-6.3	-6.2	0.09	1.741	0.517	3.901	0.299	6	3640	ZINC000013385871
0.6	-9.3	-8.5	0.43	1.660	0.347	3.816	0.665	6	3641	ZINC000100783555
0.6	-7.1	-6.7	0.19	1.518	0.269	4.052	1.371	6	3642	ZINC000100820647
0.6	-8.3	-7.9	0.25	2.083	0.694	3.277	1.305	6	3643	ZINC000257453297
0.6	-9.3	-8.9	0.20	1.633	0.295	2.797	0.639	6	3644	ZINC000014684784
0.6	-7.5	-7.2	0.25	2.655	0.590	4.123	1.489	6	3645	ZINC000000001070
0.6	-7.4	-7.1	0.21	1.839	0.311	4.404	1.420	6	3646	ZINC000257472117
0.6	-5.7	-5.2	0.21	3.145	0.563	3.935	0.792	6	3647	ZINC000002039792
0.6	-9.0	-7.9	0.70	1.904	0.052	4.115	0.691	6	3648	ZINC000015220394
0.6	-7.8	-7.5	0.16	2.402	0.562	4.264	1.395	6	3649	ZINC000245246073
0.6	-6.6	-6.4	0.10	2.111	0.413	3.469	0.609	6	3650	ZINC000014588925
0.6	-8.9	-8.3	0.31	2.737	1.082	4.568	2.148	6	3651	ZINC000040478997
0.6	-8.2	-7.7	0.24	1.457	0.265	3.596	1.601	6	3652	ZINC000014725142
0.6	-5.7	-5.4	0.20	2.263	0.623	3.871	0.900	6	3653	ZINC000038611958

0.6	-9.3	-8.4	0.55	2.490	0.988	3.964	2.288	6	3654	ZINC000004095501
0.6	-5.8	-5.6	0.09	1.817	0.198	3.682	0.237	6	3655	ZINC000003634658
0.6	-6.6	-6.4	0.11	1.804	0.369	3.595	1.117	6	3656	ZINC000238742506
0.6	-5.7	-5.6	0.10	1.839	0.292	2.697	0.902	6	3657	ZINC000082946719
0.6	-7.0	-6.7	0.18	2.207	0.793	2.747	1.190	6	3658	ZINC000002037834
0.6	-7.4	-7.1	0.20	2.271	0.539	4.819	1.186	6	3659	ZINC000004655397
0.6	-8.1	-7.5	0.32	1.837	0.135	4.730	1.225	6	3660	ZINC000003813088
0.6	-7.2	-6.7	0.23	2.661	0.252	3.646	0.364	6	3661	ZINC000001850751
0.6	-6.2	-5.9	0.16	2.063	0.291	3.476	0.387	6	3662	ZINC000004234841
0.6	-5.8	-5.4	0.20	2.097	0.465	3.536	0.556	6	3663	ZINC000002382899
0.6	-8.1	-7.9	0.16	1.968	0.078	5.992	0.206	6	3664	ZINC000015256922
0.6	-8.3	-8.1	0.21	2.556	0.368	4.846	1.277	6	3665	ZINC000100772811
0.6	-9.2	-8.5	0.49	1.682	0.553	4.263	1.810	6	3666	ZINC000100771517
0.6	-8.0	-7.7	0.21	2.640	0.315	3.577	0.250	6	3667	ZINC000100780351
0.6	-10.2	-9.2	0.56	1.909	0.262	4.760	1.523	6	3668	ZINC000257564791

0.6	-5.0	-4.8	0.17	2.455	0.793	3.242	0.400	6	3669	ZINC000003627724
0.6	-7.3	-6.7	0.39	2.037	0.591	3.230	1.217	6	3670	ZINC000000073686
0.6	-8.1	-7.8	0.21	1.225	0.409	2.746	0.331	6	3671	ZINC000000967533
0.6	-10.4	-9.9	0.32	1.045	0.185	2.997	0.896	6	3672	ZINC000257473766
0.6	-8.0	-7.6	0.31	2.190	0.326	4.377	0.988	6	3673	ZINC000013462256
0.6	-8.4	-8.1	0.14	2.426	0.558	3.465	1.125	6	3674	ZINC000002018324
0.6	-10.4	-9.8	0.36	1.092	0.170	2.870	0.798	6	3675	ZINC000257473764
0.6	-5.9	-5.6	0.22	2.040	0.843	2.806	0.950	6	3676	ZINC000002561201
0.6	-9.7	-8.6	0.82	1.299	0.082	3.101	2.356	4	3677	ZINC000004731261
0.6	-8.1	-7.5	0.37	1.633	0.267	4.456	0.754	6	3678	ZINC000085661960
0.6	-8.8	-8.3	0.35	1.919	0.684	3.309	1.264	6	3679	ZINC000008586438
0.6	-7.1	-6.9	0.11	1.696	0.478	2.963	0.447	6	3680	ZINC000015119523
0.6	-9.7	-8.4	1.15	2.017	0.312	3.143	0.154	3	3681	ZINC000014819561
0.6	-6.2	-5.9	0.15	2.526	1.059	3.709	1.299	6	3682	ZINC000032192158
0.6	-7.6	-7.1	0.26	2.194	0.743	3.720	0.725	6	3683	ZINC000095620531

0.6	-7.8	-7.5	0.21	1.840	0.649	4.849	2.284	6	3684	ZINC000002383225
0.6	-5.6	-5.4	0.09	2.747	0.767	4.400	0.664	6	3685	ZINC000013437571
0.6	-7.9	-7.6	0.23	1.956	0.365	2.893	0.791	6	3686	ZINC000040479041
0.6	-7.9	-7.5	0.28	1.683	0.219	4.479	0.559	6	3687	ZINC000257520815
0.6	-6.5	-6.3	0.12	2.270	0.424	3.893	0.777	6	3688	ZINC000014766723
0.6	-8.3	-7.8	0.37	2.283	0.164	4.953	0.932	6	3689	ZINC000100829937
0.6	-10.2	-9.3	0.56	1.910	0.264	4.757	1.538	6	3690	ZINC000100829594
0.6	-8.2	-7.9	0.21	2.289	0.644	4.558	1.447	6	3691	ZINC000013461548
0.6	-7.7	-7.4	0.24	1.921	0.931	4.537	0.788	6	3692	ZINC000000967716
0.6	-7.8	-7.4	0.20	2.686	0.751	5.167	1.239	6	3693	ZINC000014590685
0.6	-6.0	-5.8	0.16	2.625	0.898	4.454	0.888	6	3694	ZINC000002041265
0.6	-9.8	-9.4	0.29	1.648	0.317	4.542	1.232	6	3695	ZINC000013341236
0.6	-7.8	-7.3	0.43	1.908	0.282	4.268	0.498	6	3696	ZINC000001842389
0.6	-7.2	-6.9	0.18	2.442	0.492	4.364	0.256	6	3697	ZINC000014491216
0.6	-6.0	-5.8	0.14	1.916	0.241	3.662	0.850	6	3698	ZINC000029786589

0.6	-7.7	-7.5	0.14	1.833	0.423	4.212	1.428	6	3699	ZINC000014616313
0.6	-8.3	-8.0	0.36	2.451	0.398	4.228	1.154	6	3700	ZINC000257623055
0.6	-7.4	-7.1	0.16	2.853	0.303	4.759	0.696	6	3701	ZINC000002165950
0.6	-8.7	-7.9	0.37	1.718	0.144	4.610	1.263	6	3702	ZINC000014659765
0.6	-8.6	-7.9	0.51	1.389	0.573	4.423	1.889	6	3703	ZINC000015260794
0.6	-10.3	-8.9	1.14	1.728	0.523	3.407	1.684	6	3704	ZINC000100780820
0.6	-5.6	-5.4	0.07	1.674	0.327	2.787	0.424	6	3705	ZINC000001850692
0.6	-7.3	-7.1	0.19	2.110	0.642	4.658	1.447	6	3706	ZINC000040165300
0.6	-7.2	-6.8	0.25	1.905	0.552	3.467	1.748	6	3707	ZINC000001690306
0.6	-10.0	-8.7	0.98	1.256	0.181	6.047	1.829	6	3708	ZINC000261496248
0.6	-6.7	-6.3	0.32	2.863	1.639	4.035	1.422	6	3709	ZINC000256086294
0.6	-7.5	-7.2	0.25	1.744	0.727	3.740	0.326	6	3710	ZINC000033830722
0.6	-10.1	-8.6	0.70	1.551	0.397	5.313	1.565	6	3711	ZINC000257395798
0.6	-9.7	-9.2	0.33	2.467	0.816	5.415	1.607	6	3712	ZINC000014658270
0.6	-6.6	-6.3	0.23	2.578	0.569	4.075	1.073	6	3713	ZINC000002038603

0.6	-6.8	-6.6	0.13	1.830	0.320	2.305	0.349	6	3714	ZINC000001605724
0.6	-6.5	-6.4	0.09	2.764	0.866	3.653	1.102	6	3715	ZINC000000404389
0.6	-8.3	-8.0	0.26	2.511	0.363	4.836	1.270	6	3716	ZINC000257623157
0.6	-6.6	-6.2	0.28	1.864	0.680	2.696	0.567	6	3717	ZINC000001529532
0.6	-7.7	-7.5	0.15	2.187	0.489	3.111	0.872	6	3718	ZINC000002572599
0.6	-5.2	-4.9	0.12	1.789	0.653	3.139	1.010	6	3719	ZINC000001631214
0.6	-6.5	-6.3	0.17	1.840	0.983	3.481	0.741	6	3720	ZINC000000157773
0.6	-7.1	-6.8	0.17	1.503	0.165	3.725	1.006	6	3721	ZINC000257441138
0.6	-8.6	-8.1	0.33	2.493	0.349	4.023	1.018	6	3722	ZINC000034290101
0.6	-10.4	-9.7	0.56	1.654	0.663	4.193	0.887	6	3723	ZINC000100779674
0.6	-8.2	-7.6	0.41	1.730	0.239	4.573	0.142	6	3724	ZINC000257547805
0.6	-5.7	-5.6	0.10	3.417	0.472	4.171	0.498	6	3725	ZINC000000154095
0.6	-7.4	-7.1	0.24	1.840	0.880	2.427	0.857	6	3726	ZINC000034499759
0.6	-8.8	-7.9	0.45	1.792	0.363	4.893	2.036	6	3727	ZINC000015113370
0.6	-7.0	-6.9	0.09	1.948	0.278	2.627	0.272	6	3728	ZINC000069486034

0.6	-8.9	-8.6	0.18	1.877	0.354	3.234	0.769	6	3729	ZINC000001571783
0.6	-7.5	-7.1	0.39	2.184	0.142	5.498	1.068	6	3730	ZINC000004623738
0.6	-8.1	-7.8	0.17	2.281	0.860	3.941	0.299	6	3731	ZINC000000058121
0.6	-6.6	-6.4	0.20	2.101	0.829	3.529	1.325	6	3732	ZINC000000400150
0.6	-6.9	-6.7	0.11	2.677	0.770	4.553	1.438	6	3733	ZINC000012494450
0.6	-8.2	-7.8	0.26	1.740	0.367	4.932	1.696	6	3734	ZINC000015256919
0.6	-6.4	-6.2	0.12	1.772	0.399	3.320	0.650	6	3735	ZINC000013429402
0.6	-8.2	-7.8	0.32	2.299	0.280	4.865	1.779	6	3736	ZINC000001619591
0.6	-6.9	-6.6	0.18	1.826	0.482	3.403	0.910	6	3737	ZINC000014822381
0.6	-7.5	-6.8	0.33	2.128	0.548	2.671	0.465	6	3738	ZINC000000120144
0.6	-8.9	-8.4	0.36	2.067	0.359	5.443	0.400	6	3739	ZINC000005273981
0.6	-6.8	-6.7	0.07	1.939	0.251	5.145	0.784	6	3740	ZINC000001693415
0.6	-6.1	-5.9	0.12	2.807	0.559	4.060	0.529	6	3741	ZINC000005647278
0.6	-6.4	-6.2	0.14	1.962	0.844	3.119	1.160	6	3742	ZINC000014439080
0.6	-10.2	-9.2	0.56	1.821	0.362	4.714	1.520	6	3743	ZINC000257564793

0.6	-9.7	-8.0	1.24	2.288	0.114	4.001	0.232	3	3744	ZINC000257480396
0.6	-7.0	-6.7	0.16	2.114	0.107	3.625	0.795	6	3745	ZINC000001677783
0.6	-6.4	-6.2	0.17	2.214	0.464	3.393	0.807	6	3746	ZINC000000899774
0.6	-6.8	-6.5	0.14	3.249	0.490	5.363	0.508	6	3747	ZINC000002384839
0.6	-7.0	-6.6	0.20	2.539	0.380	3.686	0.619	6	3748	ZINC000014588470
0.6	-8.3	-7.2	0.77	1.879	0.453	4.334	1.744	6	3749	ZINC000013408228
0.6	-7.6	-7.2	0.35	1.752	0.197	4.181	0.551	6	3750	ZINC000015148008
0.6	-8.3	-7.9	0.22	2.485	0.799	4.388	1.529	6	3751	ZINC000018847046
0.6	-6.8	-6.4	0.21	2.607	0.895	3.341	0.924	6	3752	ZINC000001600505
0.6	-7.2	-6.6	0.31	2.322	0.659	4.172	0.913	6	3753	ZINC000014490908
0.6	-5.5	-5.3	0.15	2.758	1.462	3.643	0.510	6	3754	ZINC000002039788
0.6	-7.1	-7.0	0.11	3.000	0.485	4.884	0.410	6	3755	ZINC000069485801
0.6	-5.5	-5.3	0.11	2.908	1.370	3.610	1.028	6	3756	ZINC000005956077
0.6	-7.1	-6.7	0.18	1.898	0.512	4.857	0.611	6	3757	ZINC000257441137
0.6	-6.3	-5.9	0.18	2.484	0.528	3.488	1.123	6	3758	ZINC000001582457

0.6	-6.5	-6.1	0.29	2.234	0.414	4.342	0.663	6	3759	ZINC000245204666
0.6	-10.0	-8.8	0.82	1.153	0.280	5.150	2.137	6	3760	ZINC000038458574
0.6	-5.9	-5.6	0.15	2.392	0.570	3.903	0.268	6	3761	ZINC000000902052
0.6	-9.2	-8.8	0.35	2.568	0.549	5.530	1.778	6	3762	ZINC000034349847
0.6	-7.2	-6.9	0.14	1.916	0.373	3.543	0.757	6	3763	ZINC000014818202
0.6	-7.4	-7.2	0.15	1.810	0.353	2.896	0.295	6	3764	ZINC000000120332
0.6	-7.5	-7.3	0.15	2.382	0.644	5.356	0.662	6	3765	ZINC000005082886
0.6	-7.4	-7.2	0.17	2.457	1.067	4.378	1.365	6	3766	ZINC000005178949
0.6	-6.8	-6.5	0.16	2.838	1.018	3.893	1.022	6	3767	ZINC000001687374
0.6	-7.5	-7.1	0.31	1.189	0.568	2.753	0.375	6	3768	ZINC000100769858
0.6	-7.3	-6.8	0.24	2.698	0.466	3.953	0.689	6	3769	ZINC000000394812
0.6	-7.3	-7.0	0.17	2.945	0.625	4.321	1.444	6	3770	ZINC000000895849
0.6	-6.9	-6.7	0.11	1.771	0.029	2.894	0.463	6	3771	ZINC000004098388
0.6	-6.0	-5.9	0.06	2.055	0.194	4.013	0.531	6	3772	ZINC000032166964
0.6	-6.6	-6.5	0.08	1.856	0.766	3.714	1.063	6	3773	ZINC000012493948

0.6	-6.7	-6.1	0.32	2.710	0.822	3.941	1.065	6	3774	ZINC000000967630
0.6	-9.9	-7.8	1.10	1.958	0.271	4.631	1.996	5	3775	ZINC000005104581
0.6	-8.6	-8.2	0.34	3.070	0.225	6.244	1.371	6	3776	ZINC000014616073
0.6	-7.0	-6.6	0.20	2.541	0.383	3.664	0.655	6	3777	ZINC000204978128
0.6	-9.7	-9.0	0.44	1.575	0.672	5.041	1.612	6	3778	ZINC000014778630
0.6	-9.5	-8.9	0.42	1.773	0.833	4.180	2.215	6	3779	ZINC000014728402
0.6	-6.8	-6.5	0.16	2.991	0.934	5.262	1.112	6	3780	ZINC000005160449
0.6	-7.4	-7.2	0.15	1.814	0.385	3.000	0.245	6	3781	ZINC000000120326
0.6	-9.3	-8.7	0.41	1.565	0.460	3.863	2.032	6	3782	ZINC000000900254
0.6	-7.0	-6.6	0.18	2.179	0.855	3.007	0.576	6	3783	ZINC000001530228
0.6	-6.4	-6.2	0.12	1.964	0.747	3.071	0.525	6	3784	ZINC000014439085
0.6	-6.2	-5.9	0.27	3.256	0.815	4.820	1.486	6	3785	ZINC000033950845
0.6	-7.5	-7.4	0.19	1.828	0.182	5.076	1.359	6	3786	ZINC000005442554
0.6	-6.7	-6.4	0.14	1.375	0.181	3.378	0.367	6	3787	ZINC000015169340
0.6	-9.6	-9.0	0.37	1.664	0.854	2.683	1.077	6	3788	ZINC000100780005

0.6	-10.1	-9.4	0.47	1.762	1.088	3.623	1.140	6	3789	ZINC000257394846
0.6	-10.1	-8.9	0.64	1.642	0.437	3.952	1.496	6	3790	ZINC000014450714
0.6	-7.3	-7.0	0.16	2.259	0.578	3.633	1.020	6	3791	ZINC000000391935
0.6	-7.0	-7.0	0.05	1.745	0.285	3.665	1.136	6	3792	ZINC000257620012
0.6	-6.8	-6.6	0.09	2.326	1.027	4.115	1.130	6	3793	ZINC000014592788
0.6	-5.6	-5.3	0.20	3.035	0.279	4.312	0.451	6	3794	ZINC000013437574
0.6	-8.4	-7.8	0.54	2.313	0.742	4.321	1.849	6	3795	ZINC000257499262
0.6	-7.2	-7.0	0.17	1.732	0.248	4.805	1.212	6	3796	ZINC000001529966
0.6	-8.3	-8.0	0.19	1.790	0.236	4.328	0.557	6	3797	ZINC000006037688
0.6	-7.2	-6.9	0.16	1.908	0.569	3.257	1.000	6	3798	ZINC000002557900
0.6	-8.9	-8.0	0.51	1.813	0.203	4.460	1.633	6	3799	ZINC000257441914
0.6	-6.3	-5.8	0.28	2.809	0.982	4.447	1.008	6	3800	ZINC000038220510
0.6	-10.1	-8.9	0.67	1.576	0.330	4.759	1.525	6	3801	ZINC000014450720
0.6	-7.0	-6.7	0.18	1.396	1.059	3.508	0.968	6	3802	ZINC000002019619
0.6	-6.1	-5.9	0.16	3.373	0.311	4.664	0.460	6	3803	ZINC000002010484

0.6	-5.6	-5.4	0.11	2.156	1.168	3.425	0.387	6	3804	ZINC000000334875
0.6	-6.5	-6.4	0.07	1.764	0.337	3.241	0.210	6	3805	ZINC000100488727
0.6	-8.7	-8.1	0.41	1.569	0.611	4.092	0.279	6	3806	ZINC000196553233
0.6	-8.1	-7.8	0.21	1.613	0.258	3.640	1.354	6	3807	ZINC000013432660
0.6	-6.3	-6.0	0.15	2.275	0.437	3.549	0.711	6	3808	ZINC000005441040
0.6	-6.9	-6.6	0.18	2.077	0.324	4.238	0.723	6	3809	ZINC000100772470
0.6	-9.4	-9.1	0.21	1.696	0.386	4.961	0.845	6	3810	ZINC000015273417
0.6	-8.1	-7.8	0.28	1.834	0.198	4.851	1.073	6	3811	ZINC000012496925
0.6	-7.5	-7.2	0.27	2.146	0.417	4.711	2.155	6	3812	ZINC000003870223
0.6	-6.7	-5.9	0.42	2.343	0.412	3.622	0.858	6	3813	ZINC000039618912
0.6	-10.3	-8.7	1.30	1.566	0.491	4.565	1.722	5	3814	ZINC000257418756
0.6	-10.2	-9.0	0.64	1.828	0.357	4.825	1.317	6	3815	ZINC000257564792
0.6	-7.0	-6.8	0.11	2.548	0.399	4.219	0.226	6	3816	ZINC000005273806
0.6	-9.3	-8.9	0.28	1.611	0.287	3.004	0.894	6	3817	ZINC000251666753
0.6	-6.6	-6.3	0.21	2.458	0.563	3.931	1.056	6	3818	ZINC000002000305

0.6	-8.6	-7.1	0.70	1.571	0.147	3.972	1.029	6	3819	ZINC000014828122
0.6	-7.4	-7.0	0.19	3.780	0.183	4.621	0.298	6	3820	ZINC000004899513
0.6	-7.6	-7.3	0.24	2.863	0.713	4.285	1.575	6	3821	ZINC000003635934
0.6	-9.8	-9.8	0.00	0.000	0.000	0.000	0.000	1	3822	ZINC000004096117
0.6	-6.6	-6.4	0.16	2.495	0.500	3.944	0.802	6	3823	ZINC000003831031
0.6	-7.7	-7.4	0.20	2.446	0.371	5.260	0.697	6	3824	ZINC000004095908
0.6	-6.4	-6.3	0.05	2.716	1.319	4.168	0.776	6	3825	ZINC000005956065
0.6	-7.0	-6.6	0.28	2.067	0.404	4.733	1.068	6	3826	ZINC000015122227
0.6	-8.6	-8.2	0.24	1.821	0.303	3.653	1.457	6	3827	ZINC000013380363
0.6	-6.2	-5.9	0.11	2.624	0.479	4.851	0.544	6	3828	ZINC000001849837
0.6	-7.9	-7.5	0.25	2.284	0.667	3.694	1.031	6	3829	ZINC000100780006
0.6	-9.5	-8.6	0.68	1.110	0.212	6.231	1.764	6	3830	ZINC000100891776
0.6	-8.0	-7.6	0.21	2.226	0.306	3.774	0.963	6	3831	ZINC000014827117
0.6	-10.1	-8.6	0.74	1.566	0.309	4.366	2.012	6	3832	ZINC000257395799
0.6	-9.6	-8.3	0.62	2.026	0.257	4.487	0.708	6	3833	ZINC000095635912

0.6	-7.3	-6.8	0.25	2.566	0.538	4.084	0.404	6	3834	ZINC000006205881
0.6	-6.5	-6.3	0.13	2.143	0.379	3.171	0.492	6	3835	ZINC000242649207
0.6	-6.7	-6.2	0.36	2.530	1.745	3.817	1.494	6	3836	ZINC000000388545
0.6	-10.6	-9.7	0.90	2.363	0.000	3.760	0.000	2	3837	ZINC000014613037
0.6	-7.6	-7.2	0.23	1.922	0.851	3.276	0.753	6	3838	ZINC000100361631
0.6	-6.0	-5.8	0.16	1.952	0.226	3.310	0.113	6	3839	ZINC000000156245
0.6	-6.8	-6.4	0.17	1.706	0.418	3.829	0.941	6	3840	ZINC000004556936
0.6	-6.4	-6.1	0.14	2.419	0.786	4.333	0.615	6	3841	ZINC000006037250
0.6	-6.3	-5.9	0.21	2.245	0.560	3.739	0.749	6	3842	ZINC000000155368
0.6	-7.9	-7.6	0.17	1.759	0.407	2.669	0.602	6	3843	ZINC000000097165
0.6	-6.2	-6.0	0.14	1.614	0.619	3.315	0.618	6	3844	ZINC000100773214
0.6	-6.2	-5.8	0.21	2.867	0.809	3.491	1.005	6	3845	ZINC000002033904
0.6	-5.3	-5.2	0.05	2.848	1.531	4.103	0.647	6	3846	ZINC000261499725
0.6	-7.7	-7.4	0.29	1.487	0.257	3.733	1.210	6	3847	ZINC000015257102
0.6	-10.6	-9.5	0.65	1.679	0.286	5.333	0.602	6	3848	ZINC000100783994

0.6	-7.4	-7.1	0.16	2.386	0.606	3.918	1.661	6	3849	ZINC000040470615
0.6	-7.4	-7.3	0.07	1.791	0.379	3.053	0.215	6	3850	ZINC000000120329
0.6	-7.9	-7.4	0.41	1.576	0.299	3.587	0.925	6	3851	ZINC000014505050
0.6	-6.2	-5.7	0.28	1.850	0.522	3.286	0.768	6	3852	ZINC000001850874
0.6	-8.4	-8.2	0.15	1.766	0.474	3.644	1.104	6	3853	ZINC000085489325
0.6	-8.6	-8.1	0.39	2.639	0.293	5.578	1.553	6	3854	ZINC000013523894
0.6	-6.8	-6.6	0.18	2.354	0.312	3.636	0.536	6	3855	ZINC000001850478
0.6	-5.3	-4.9	0.32	2.640	0.725	3.148	0.843	6	3856	ZINC000002168242
0.6	-7.0	-6.7	0.16	2.798	0.483	3.407	0.728	6	3857	ZINC000004098416
0.6	-5.6	-5.3	0.17	2.355	0.757	4.170	0.738	6	3858	ZINC000002015944
0.6	-7.7	-7.3	0.27	1.936	0.731	3.248	0.632	6	3859	ZINC000004027288
0.6	-5.9	-5.7	0.16	2.512	0.584	3.999	0.966	6	3860	ZINC000002034874
0.6	-7.1	-6.8	0.20	2.383	0.155	4.595	0.558	6	3861	ZINC000012495938
0.6	-7.5	-7.0	0.36	2.147	0.812	3.692	1.811	6	3862	ZINC000003869638
0.6	-7.3	-7.0	0.27	2.065	0.287	5.120	0.406	6	3863	ZINC000001531529

0.6	-5.3	-4.8	0.35	2.444	1.139	3.043	1.249	6	3864	ZINC000001586746
0.6	-8.5	-7.5	0.49	1.936	0.279	4.019	1.215	6	3865	ZINC000004097827
0.6	-9.0	-8.6	0.24	1.823	0.146	3.649	0.585	6	3866	ZINC000015058363
0.6	-6.2	-6.1	0.11	2.487	0.498	3.816	0.985	6	3867	ZINC000005159624
0.6	-7.7	-7.5	0.07	2.775	0.237	4.522	0.748	6	3868	ZINC000002573801
0.6	-8.4	-8.2	0.12	1.869	0.240	3.748	0.688	6	3869	ZINC000005751121
0.6	-7.4	-7.0	0.24	2.054	0.669	3.067	0.771	6	3870	ZINC000000151915
0.6	-8.5	-8.3	0.14	1.465	0.293	3.445	0.609	6	3871	ZINC000085599408
0.6	-6.3	-5.9	0.33	3.082	1.410	4.099	1.478	6	3872	ZINC000032156158
0.6	-8.1	-7.4	0.37	2.617	0.515	4.095	1.170	6	3873	ZINC000002384818
0.6	-7.6	-7.2	0.23	2.326	0.723	3.850	0.748	6	3874	ZINC000100776178
0.6	-7.1	-6.7	0.22	3.154	0.909	4.279	1.307	6	3875	ZINC000082395906
0.6	-5.8	-5.5	0.14	1.870	0.463	3.616	1.488	6	3876	ZINC000012153711
0.6	-8.6	-7.8	0.40	2.080	0.142	5.040	1.092	6	3877	ZINC000012496636
0.6	-6.0	-5.6	0.24	2.131	0.659	3.441	1.082	6	3878	ZINC000082946718

0.6	-8.2	-7.6	0.29	1.721	0.050	5.123	0.820	6	3879	ZINC000012496277
0.6	-8.9	-8.2	0.38	1.913	0.192	3.666	0.832	6	3880	ZINC000013323228
0.6	-6.4	-6.2	0.16	2.623	0.721	4.469	1.351	6	3881	ZINC000005761927
0.6	-6.2	-6.0	0.09	2.552	0.371	4.354	0.733	6	3882	ZINC000059659244
0.6	-8.2	-7.8	0.21	1.680	0.440	4.186	1.178	6	3883	ZINC000014637057
0.6	-9.9	-8.3	1.04	1.447	0.266	4.624	1.778	6	3884	ZINC000003881403
0.6	-8.9	-8.6	0.28	1.772	0.278	6.222	0.368	6	3885	ZINC000257497785
0.6	-6.9	-6.6	0.21	2.116	0.322	3.411	1.402	6	3886	ZINC000014657637
0.6	-8.5	-8.0	0.24	1.786	0.136	4.057	2.080	6	3887	ZINC000013334898
0.6	-6.8	-6.4	0.20	3.004	0.722	5.485	1.556	6	3888	ZINC000040470862
0.6	-5.9	-5.7	0.13	1.884	0.766	3.101	0.715	6	3889	ZINC000021984838
0.6	-7.5	-7.2	0.24	1.092	0.580	3.153	0.500	6	3890	ZINC000040164574
0.6	-5.7	-5.3	0.20	2.053	0.463	3.545	0.632	6	3891	ZINC000050179659
0.6	-7.3	-6.9	0.33	3.688	0.399	5.971	0.137	6	3892	ZINC000014947822
0.6	-6.6	-6.3	0.25	2.420	0.112	3.593	0.611	6	3893	ZINC000001575518

0.6	-7.0	-6.6	0.21	1.945	0.470	3.111	0.939	6	3894	ZINC000004972521
0.6	-8.3	-7.6	0.35	1.773	0.188	5.113	1.289	6	3895	ZINC000015256916
0.6	-6.8	-6.6	0.16	2.585	1.080	3.815	1.409	6	3896	ZINC000095618091
0.6	-9.2	-8.4	0.48	1.965	0.427	4.721	1.096	6	3897	ZINC000000985403
0.6	-7.3	-7.0	0.23	1.485	0.355	4.670	2.037	6	3898	ZINC000004523259
0.6	-7.8	-7.4	0.27	2.237	0.977	4.063	0.841	6	3899	ZINC000001842391
0.6	-6.6	-6.2	0.21	2.983	0.993	5.431	1.570	6	3900	ZINC000013398039
0.6	-8.1	-7.8	0.25	1.404	0.189	3.525	0.360	6	3901	ZINC000085883128
0.6	-6.7	-6.5	0.14	1.572	0.459	3.908	1.267	6	3902	ZINC000031290680
0.6	-7.4	-7.2	0.18	1.224	0.423	2.930	0.294	6	3903	ZINC000000968128
0.6	-8.2	-7.9	0.18	1.537	0.314	3.655	0.485	6	3904	ZINC000015160905
0.6	-7.1	-6.8	0.17	1.859	0.501	4.240	1.183	6	3905	ZINC000100820650
0.6	-9.4	-8.9	0.39	1.664	0.307	5.731	0.419	6	3906	ZINC000257566292
0.6	-6.7	-6.3	0.27	2.982	0.921	4.864	0.503	6	3907	ZINC000001850898
0.6	-6.1	-5.8	0.21	2.776	1.297	4.382	0.438	6	3908	ZINC000002017409

0.6	-6.0	-5.9	0.09	2.373	0.691	3.696	0.825	6	3909	ZINC000095620562
0.6	-7.3	-7.2	0.08	3.147	0.624	3.721	0.638	6	3910	ZINC000004654765
0.6	-8.4	-7.9	0.33	2.430	0.422	5.324	1.346	6	3911	ZINC000014445238
0.6	-6.6	-6.0	0.31	2.119	0.270	3.626	0.706	6	3912	ZINC000001637975
0.6	-7.5	-7.3	0.11	1.616	0.218	5.637	2.118	6	3913	ZINC000014860251
0.6	-8.6	-8.5	0.07	1.350	0.659	3.282	1.849	6	3914	ZINC000014491827
0.6	-6.7	-6.5	0.10	2.600	0.528	4.058	0.744	6	3915	ZINC000001758084
0.6	-9.1	-7.7	0.86	1.627	0.439	4.138	1.324	6	3916	ZINC000014927139
0.6	-8.4	-8.0	0.32	2.059	0.237	2.525	0.254	6	3917	ZINC000004096931
0.6	-9.2	-8.8	0.43	1.908	0.402	5.718	1.114	6	3918	ZINC000000895955
0.6	-6.3	-6.1	0.11	3.533	0.701	5.091	0.746	6	3919	ZINC000001578619
0.6	-7.5	-7.0	0.30	1.969	0.333	3.391	0.931	6	3920	ZINC000034381101
0.6	-7.5	-7.4	0.13	2.826	0.485	4.702	1.586	6	3921	ZINC000095617714
0.6	-10.3	-8.7	0.90	1.548	0.167	4.821	0.943	6	3922	ZINC000013308687
0.6	-8.1	-7.9	0.15	2.689	0.393	3.783	1.091	6	3923	ZINC000018169010

0.6	-6.9	-6.6	0.17	1.832	0.588	3.683	0.904	6	3924	ZINC000000968225
0.6	-5.9	-5.4	0.31	2.483	0.556	3.527	0.849	6	3925	ZINC000002039799
0.6	-9.0	-8.5	0.40	1.382	0.458	5.032	1.505	6	3926	ZINC000004649679
0.6	-5.5	-5.3	0.12	1.955	0.926	3.041	0.875	6	3927	ZINC000005923283
0.6	-8.6	-8.4	0.19	2.330	0.948	4.084	1.778	6	3928	ZINC000034290102
0.6	-5.8	-5.5	0.21	2.243	0.817	3.409	1.286	6	3929	ZINC000002575203
0.6	-9.7	-9.0	0.37	1.869	0.200	4.917	1.089	6	3930	ZINC000014825436
0.6	-8.4	-8.4	0.05	2.508	0.868	4.728	1.472	6	3931	ZINC000008215971
0.6	-6.4	-6.3	0.09	2.418	0.839	3.821	0.665	6	3932	ZINC000032164300
0.6	-6.7	-6.2	0.23	1.747	0.315	3.934	0.438	6	3933	ZINC000015217519
0.6	-9.1	-8.4	0.43	2.378	0.493	5.000	0.885	6	3934	ZINC000257562929
0.6	-8.6	-8.2	0.32	2.703	0.727	4.684	1.228	6	3935	ZINC000001703461
0.6	-6.2	-6.0	0.14	1.709	0.516	3.420	0.501	6	3936	ZINC000100773212
0.6	-6.0	-5.7	0.20	1.903	0.442	3.437	0.235	6	3937	ZINC000019735106
0.6	-9.2	-8.7	0.30	1.555	0.126	4.598	1.927	6	3938	ZINC000231215705

0.6	-7.0	-6.6	0.25	2.501	0.264	3.694	0.511	6	3939	ZINC000000901642
0.6	-8.9	-8.3	0.72	2.175	0.674	3.544	1.554	6	3940	ZINC000014691903
0.6	-9.1	-8.4	0.38	2.722	0.452	4.784	1.620	6	3941	ZINC000033943508
0.6	-6.6	-6.4	0.10	2.325	0.257	4.235	0.441	6	3942	ZINC000033791470
0.6	-10.1	-8.8	0.66	1.592	0.412	4.621	1.668	6	3943	ZINC000014450716
0.6	-8.5	-8.0	0.34	1.838	0.393	2.682	0.640	6	3944	ZINC000100687730
0.6	-7.8	-7.6	0.15	1.628	0.410	3.904	1.361	6	3945	ZINC000008234296
0.6	-7.3	-7.0	0.20	2.619	0.430	4.405	1.582	6	3946	ZINC000027206940
0.6	-7.1	-6.6	0.22	1.649	0.274	3.717	0.876	6	3947	ZINC000100820649
0.6	-8.9	-8.5	0.29	1.620	0.548	4.296	2.043	6	3948	ZINC000257441913
0.6	-7.5	-7.2	0.16	1.807	0.425	5.392	0.748	6	3949	ZINC000014592866
0.6	-7.2	-6.9	0.26	2.117	0.708	3.136	0.781	6	3950	ZINC000014503322
0.6	-7.6	-7.2	0.29	2.454	0.250	3.966	0.384	6	3951	ZINC000001695462
0.6	-9.0	-8.5	0.22	1.869	0.228	4.297	1.912	6	3952	ZINC000014811608
0.6	-8.6	-7.8	0.53	1.302	0.203	4.372	1.985	6	3953	ZINC000015260799

0.6	-8.4	-7.7	0.35	2.050	0.671	4.520	1.737	6	3954	ZINC000002384847
0.6	-7.6	-7.3	0.23	1.945	0.160	4.869	1.717	6	3955	ZINC000014556685
0.6	-9.6	-9.2	0.32	2.045	0.402	5.204	1.832	6	3956	ZINC000015116110
0.6	-7.7	-7.1	0.31	2.065	0.613	3.144	0.894	6	3957	ZINC000100827440
0.6	-6.1	-5.9	0.11	1.711	0.281	4.043	0.433	6	3958	ZINC000001638431
0.6	-7.8	-7.5	0.39	1.909	0.296	5.321	0.752	6	3959	ZINC000014616312
0.6	-9.3	-8.4	0.60	1.716	0.486	5.841	0.714	6	3960	ZINC000238752405
0.6	-8.6	-7.9	0.40	1.308	0.337	5.095	1.931	6	3961	ZINC000257449732
0.6	-7.2	-6.9	0.18	2.514	0.200	4.449	0.485	6	3962	ZINC000034267181
0.6	-8.4	-8.2	0.15	2.267	0.606	3.867	1.018	6	3963	ZINC000014594786
0.6	-6.1	-5.9	0.13	1.973	0.376	3.359	0.569	6	3964	ZINC000014588936
0.6	-7.9	-7.6	0.19	1.634	0.190	3.570	0.653	6	3965	ZINC000005731857
0.6	-8.5	-8.2	0.21	1.520	0.319	3.344	1.665	6	3966	ZINC000001531858
0.6	-5.9	-5.7	0.14	2.336	0.563	3.104	0.751	6	3967	ZINC000003860659
0.6	-9.7	-8.6	0.70	2.129	0.343	3.868	1.059	6	3968	ZINC000256080887

0.6	-7.7	-7.3	0.25	2.158	0.369	3.701	1.836	6	3969	ZINC000261499747
0.6	-10.2	-9.1	0.54	1.856	0.347	5.396	0.512	6	3970	ZINC000100829592
0.6	-9.5	-8.5	0.77	1.628	0.295	4.284	1.291	6	3971	ZINC000257492752
0.6	-8.0	-7.3	0.35	1.822	0.369	3.303	1.429	6	3972	ZINC000095619945
0.6	-7.5	-7.3	0.18	1.703	0.698	3.070	0.669	6	3973	ZINC000013480057
0.6	-9.2	-8.7	0.32	1.733	0.744	3.439	1.804	6	3974	ZINC000001665640
0.6	-6.7	-6.4	0.21	1.800	0.889	4.904	1.986	6	3975	ZINC000001633880
0.6	-7.0	-6.9	0.06	2.488	0.898	4.146	1.419	6	3976	ZINC000005758685
0.6	-10.0	-8.9	0.81	1.159	0.290	5.166	2.117	6	3977	ZINC000261496246
0.6	-9.3	-8.5	0.62	1.981	0.662	4.271	2.242	6	3978	ZINC000013339711
0.6	-9.2	-7.3	1.22	2.447	0.215	4.761	1.290	4	3979	ZINC000014723715
0.6	-8.2	-7.7	0.33	2.416	0.284	4.114	0.795	6	3980	ZINC000238733293
0.6	-5.1	-5.0	0.11	1.701	0.121	2.943	0.345	6	3981	ZINC000019877638
0.6	-5.8	-5.4	0.21	1.639	0.424	2.887	0.810	6	3982	ZINC000001850618
0.6	-6.9	-6.7	0.23	2.088	0.369	5.496	1.469	6	3983	ZINC000095617792

0.6	-7.6	-7.1	0.33	2.370	0.387	3.980	0.714	6	3984	ZINC000002504554
0.6	-8.4	-7.6	0.43	1.977	0.499	4.749	1.520	6	3985	ZINC000257559219
0.6	-7.4	-7.0	0.31	2.728	0.977	3.724	1.407	6	3986	ZINC000034499768
0.6	-7.2	-6.9	0.16	2.242	0.577	3.672	0.559	6	3987	ZINC000000156963
0.6	-8.0	-7.0	0.56	2.185	0.330	5.146	1.217	6	3988	ZINC000014411628
0.6	-6.9	-6.5	0.28	2.212	0.304	3.339	1.006	6	3989	ZINC000034114361
0.6	-6.7	-6.4	0.22	2.677	0.441	3.886	1.006	6	3990	ZINC000001601796
0.6	-6.3	-6.2	0.11	1.796	0.872	3.331	0.677	6	3991	ZINC000013386352
0.6	-5.6	-5.2	0.27	1.504	0.386	2.164	0.549	6	3992	ZINC000001718566
0.6	-6.6	-6.4	0.18	2.591	0.575	3.989	0.837	6	3993	ZINC000000967511
0.6	-8.3	-8.0	0.24	1.595	0.541	4.047	1.028	6	3994	ZINC000000120283
0.6	-6.8	-6.1	0.31	2.421	0.902	4.301	0.697	6	3995	ZINC000095620591
0.6	-8.6	-8.1	0.32	1.205	0.266	4.267	1.963	6	3996	ZINC000015260796
0.6	-6.6	-6.3	0.20	1.979	0.399	3.263	0.642	6	3997	ZINC000001586646
0.6	-7.7	-7.4	0.18	2.639	0.360	4.148	0.453	6	3998	ZINC000095617663

0.6	-8.4	-8.0	0.43	2.227	0.180	4.210	0.526	6	3999	ZINC000006030775
0.6	-5.3	-5.0	0.17	1.682	0.696	2.517	0.730	6	4000	ZINC000001672808
0.6	-7.9	-7.8	0.08	1.883	0.310	2.900	0.776	6	4001	ZINC000005759355
0.6	-6.6	-6.3	0.14	2.610	0.495	4.063	1.182	6	4002	ZINC000040470478
0.6	-7.6	-7.4	0.19	2.228	0.525	3.708	1.205	6	4003	ZINC000002534722
0.6	-9.9	-8.5	0.73	1.628	0.242	3.498	1.160	6	4004	ZINC000006068778
0.6	-8.6	-7.9	0.35	2.125	0.487	5.533	0.414	6	4005	ZINC000004097770
0.6	-8.0	-7.8	0.20	2.245	0.339	3.143	0.574	6	4006	ZINC000050027481
0.6	-7.4	-7.2	0.16	2.325	0.682	5.015	0.520	6	4007	ZINC000008860439
0.6	-9.5	-7.8	1.10	1.233	0.277	2.245	0.195	4	4008	ZINC000013484750
0.6	-8.1	-7.9	0.27	1.373	0.247	2.989	0.554	6	4009	ZINC000015115288
0.6	-8.3	-8.0	0.25	1.795	0.875	3.055	1.415	6	4010	ZINC000100780001
0.6	-4.8	-4.5	0.13	2.241	0.284	3.468	0.792	6	4011	ZINC000002045861
0.6	-10.1	-9.4	0.42	1.872	0.867	5.318	1.606	6	4012	ZINC000014727219
0.6	-6.5	-6.2	0.27	1.866	1.234	2.974	1.380	6	4013	ZINC000001670361

0.6	-6.9	-6.7	0.13	2.003	0.238	4.971	1.650	6	4014	ZINC000034893634
0.6	-5.6	-5.1	0.29	2.042	0.321	3.016	0.777	6	4015	ZINC000002037749
0.6	-7.6	-7.3	0.19	2.858	0.710	4.298	1.570	6	4016	ZINC000000155696
0.6	-5.1	-4.8	0.18	2.464	0.829	3.291	1.032	6	4017	ZINC000000334460
0.6	-7.7	-7.5	0.12	2.770	0.207	4.602	0.762	6	4018	ZINC000033976926
0.6	-7.8	-7.2	0.28	1.544	0.140	3.303	0.512	6	4019	ZINC000005103850
0.6	-10.4	-9.0	0.74	1.567	0.307	3.580	2.030	6	4020	ZINC000090614937
0.6	-6.3	-5.9	0.22	1.607	0.312	3.794	0.737	6	4021	ZINC000095620597
0.6	-7.9	-7.8	0.11	1.217	0.596	3.789	0.862	6	4022	ZINC000001685159
0.6	-7.0	-6.5	0.27	2.260	0.332	3.477	0.918	6	4023	ZINC000004557162
0.6	-8.2	-7.6	0.36	1.062	0.466	5.412	0.603	6	4024	ZINC000015146156
0.6	-9.3	-8.8	0.39	2.028	0.270	5.851	1.461	6	4025	ZINC000005662684
0.6	-7.8	-7.4	0.41	1.986	0.521	2.784	0.260	6	4026	ZINC000002047002
0.6	-8.8	-8.4	0.36	2.253	0.212	4.254	0.604	6	4027	ZINC000257591086
0.6	-6.4	-6.2	0.11	1.624	0.689	4.690	0.463	6	4028	ZINC000015261745

0.6	-5.9	-5.5	0.25	1.648	0.541	2.701	0.432	6	4029	ZINC000012495565
0.6	-8.7	-8.3	0.26	1.899	0.337	3.210	0.801	6	4030	ZINC000003847505
0.6	-6.9	-6.7	0.15	2.222	0.871	3.947	1.526	6	4031	ZINC000003978812
0.6	-8.7	-8.1	0.41	1.784	0.645	4.169	0.587	6	4032	ZINC000100776135
0.6	-6.9	-6.6	0.18	1.647	0.441	3.230	0.622	6	4033	ZINC000000080853
0.6	-9.4	-7.7	0.84	2.869	0.287	6.184	1.325	6	4034	ZINC000014778667
0.6	-7.8	-7.5	0.16	1.806	0.180	3.851	0.830	6	4035	ZINC000257433630
0.6	-10.1	-9.5	0.43	1.269	0.800	3.205	1.054	6	4036	ZINC000100779497
0.6	-7.3	-6.9	0.23	1.560	0.585	2.331	0.338	6	4037	ZINC000001680382
0.6	-5.9	-5.6	0.21	2.100	0.619	3.350	0.910	6	4038	ZINC000095619613
0.6	-8.2	-7.9	0.18	1.733	0.321	4.587	1.663	6	4039	ZINC000256078939
0.6	-6.4	-6.2	0.12	2.532	0.755	3.918	1.028	6	4040	ZINC000001737810
0.6	-9.5	-8.6	0.68	1.110	0.212	6.230	1.764	6	4041	ZINC000044963243
0.6	-8.6	-7.9	0.43	1.457	0.218	3.927	0.703	6	4042	ZINC000015160906
0.6	-6.2	-6.0	0.13	2.686	0.403	3.663	0.582	6	4043	ZINC000039365143

0.6	-8.7	-8.6	0.14	1.404	0.739	3.197	1.829	6	4044	ZINC000015249537
0.6	-7.2	-6.9	0.21	2.195	0.405	3.140	1.345	6	4045	ZINC000040439671
0.6	-5.5	-5.1	0.23	2.518	0.629	3.102	0.584	6	4046	ZINC000032166944
0.6	-6.8	-6.5	0.22	2.375	0.398	4.317	0.730	6	4047	ZINC000000157077
0.6	-6.5	-6.3	0.12	2.165	0.475	3.212	0.881	6	4048	ZINC000095618090
0.6	-8.0	-7.3	0.56	1.831	0.352	4.562	2.118	6	4049	ZINC000013303506
0.6	-7.3	-7.0	0.16	2.721	0.196	3.622	0.440	6	4050	ZINC000012495916
0.6	-7.0	-6.9	0.10	1.967	0.244	3.676	0.812	6	4051	ZINC000001850293
0.6	-7.2	-6.9	0.14	2.800	0.780	4.337	0.957	6	4052	ZINC000013508950
0.6	-8.5	-7.3	0.69	1.984	0.206	5.629	0.323	6	4053	ZINC000261497625
0.6	-7.0	-6.9	0.09	1.941	0.362	3.603	1.460	6	4054	ZINC000257416747
0.6	-6.4	-6.0	0.19	3.183	0.614	4.624	0.734	6	4055	ZINC000038251276
0.6	-8.1	-7.9	0.14	2.236	0.636	3.823	1.324	6	4056	ZINC000014688569
0.6	-6.1	-5.7	0.21	1.990	0.448	3.781	0.571	6	4057	ZINC000003606265
0.6	-7.2	-6.8	0.24	1.470	0.442	3.075	1.532	6	4058	ZINC000034126691

0.6	-5.7	-5.2	0.30	1.926	0.277	3.154	0.454	6	4059	ZINC000001586317
0.6	-7.8	-7.4	0.25	2.995	0.560	3.988	0.223	6	4060	ZINC000000409269
0.6	-9.3	-8.6	0.41	1.880	0.387	2.705	0.670	6	4061	ZINC000013461790
0.6	-7.1	-6.9	0.09	1.970	0.336	3.361	0.699	6	4062	ZINC000000410076
0.6	-6.3	-6.1	0.14	2.050	0.268	3.745	0.579	6	4063	ZINC000148097133
0.6	-7.1	-6.9	0.10	2.246	0.349	4.182	1.322	6	4064	ZINC000001850294
0.6	-7.2	-6.9	0.20	2.701	0.818	4.074	1.075	6	4065	ZINC000002031123
0.6	-7.4	-7.0	0.23	2.474	0.309	4.225	0.843	6	4066	ZINC000001648505
0.6	-7.8	-7.7	0.07	1.664	0.724	3.106	0.521	6	4067	ZINC000100071089
0.6	-6.7	-6.2	0.25	1.595	0.662	2.712	0.587	6	4068	ZINC000014516984
0.6	-8.0	-7.9	0.07	2.120	1.292	3.688	1.349	6	4069	ZINC000095625973
0.6	-10.1	-8.9	0.67	1.579	0.328	4.761	1.522	6	4070	ZINC000014450718
0.6	-5.6	-5.3	0.18	2.261	0.701	2.904	0.648	6	4071	ZINC000032228394
0.6	-6.9	-6.8	0.09	1.916	0.505	3.026	1.321	6	4072	ZINC000016041098
0.6	-5.8	-5.3	0.23	2.220	0.405	2.996	0.551	6	4073	ZINC000022065339

0.6	-8.0	-7.5	0.27	1.914	0.649	2.998	0.969	6	4074	ZINC000014720615
0.6	-7.6	-7.2	0.22	1.827	0.332	3.566	0.867	6	4075	ZINC000034276223
0.6	-10.4	-9.5	0.90	2.027	0.000	6.672	0.000	2	4076	ZINC000004655389
0.6	-8.4	-8.0	0.21	2.254	0.484	3.994	1.348	6	4077	ZINC000005767143
0.6	-8.9	-8.3	0.37	1.987	0.229	6.407	0.547	6	4078	ZINC000014590707
0.6	-7.4	-6.8	0.32	1.837	0.679	2.918	0.784	6	4079	ZINC000020389897
0.6	-7.2	-7.0	0.16	3.353	1.134	4.400	1.593	6	4080	ZINC000001692494
0.6	-8.2	-7.7	0.28	1.601	0.331	4.415	1.516	6	4081	ZINC000013377650
0.6	-7.6	-7.2	0.20	2.023	0.546	4.018	1.084	6	4082	ZINC000002038252
0.6	-6.7	-6.4	0.16	2.110	0.219	4.288	0.473	6	4083	ZINC000002000964
0.6	-7.9	-7.5	0.22	2.489	0.342	5.251	1.115	6	4084	ZINC000027645264
0.6	-8.4	-8.2	0.24	2.640	0.420	4.838	1.263	6	4085	ZINC000100772815
0.6	-7.2	-6.9	0.19	2.326	0.454	3.813	1.311	6	4086	ZINC000002583437
0.6	-6.3	-6.1	0.12	2.443	0.410	3.858	0.888	6	4087	ZINC000002020126
0.6	-8.4	-7.8	0.42	1.821	0.522	4.969	0.303	6	4088	ZINC000013377648

0.6	-5.5	-5.1	0.21	1.692	0.225	3.087	1.005	6	4089	ZINC000001599553
0.6	-8.8	-8.2	0.30	2.877	0.771	4.236	1.705	6	4090	ZINC000095619324
0.6	-6.2	-6.0	0.13	2.635	1.118	4.173	1.031	6	4091	ZINC000001731762
0.6	-8.7	-7.9	0.53	2.084	0.328	4.003	1.296	6	4092	ZINC000013482103
0.6	-6.6	-6.4	0.15	2.333	0.553	4.002	1.132	6	4093	ZINC000050027725
0.6	-6.0	-5.6	0.19	2.700	0.618	3.415	0.821	6	4094	ZINC000052541469
0.6	-5.5	-5.0	0.29	3.041	1.278	4.067	1.581	6	4095	ZINC000001665895
0.6	-7.2	-7.0	0.13	2.513	0.650	3.819	0.514	6	4096	ZINC000095911093
0.6	-8.4	-7.7	0.62	2.168	0.828	3.195	1.194	6	4097	ZINC000002038824
0.6	-8.9	-8.1	0.42	1.994	0.199	5.803	1.721	6	4098	ZINC000014590706
0.6	-10.6	-9.6	0.64	1.693	0.254	5.337	0.595	6	4099	ZINC000257486691
0.6	-5.9	-5.6	0.18	2.363	0.655	3.579	0.362	6	4100	ZINC000003875729
0.6	-10.6	-10.2	0.29	1.662	0.344	5.613	1.224	6	4101	ZINC000257469498
0.6	-8.5	-8.3	0.21	1.771	0.576	5.641	2.010	6	4102	ZINC000014593990
0.6	-9.5	-8.6	0.68	1.106	0.209	6.230	1.764	6	4103	ZINC000012493629

0.6	-9.4	-8.5	0.57	1.981	0.678	5.468	1.767	6	4104	ZINC000000899106
0.6	-6.4	-6.2	0.08	1.796	0.500	3.566	0.567	6	4105	ZINC000003861537
0.6	-7.7	-7.6	0.06	2.651	0.243	4.582	0.582	6	4106	ZINC000004025995
0.6	-7.9	-7.4	0.34	2.071	0.627	3.684	2.125	6	4107	ZINC000014766089
0.6	-5.4	-5.2	0.18	2.044	0.664	3.309	1.069	6	4108	ZINC000001609498
0.6	-7.4	-7.1	0.21	2.062	0.457	4.304	1.129	6	4109	ZINC000014719713
0.6	-6.9	-6.7	0.14	2.230	0.307	3.594	0.558	6	4110	ZINC000069485993
0.6	-6.8	-6.6	0.09	2.394	0.276	3.455	0.252	6	4111	ZINC000001578706
0.6	-7.2	-6.8	0.33	2.354	0.566	4.777	1.385	6	4112	ZINC000005758915
0.6	-6.8	-6.6	0.13	1.794	0.400	3.760	0.875	6	4113	ZINC000002002623
0.6	-6.9	-6.5	0.26	1.219	0.226	4.511	0.940	6	4114	ZINC000257559500
0.6	-8.1	-7.8	0.23	2.284	0.836	4.253	1.120	6	4115	ZINC000000199415
0.6	-6.7	-6.1	0.27	1.905	0.340	3.659	0.749	6	4116	ZINC000012153709
0.6	-7.8	-7.6	0.13	2.422	0.424	4.668	1.514	6	4117	ZINC000015148367
0.6	-8.5	-8.2	0.21	2.349	0.105	4.893	0.151	6	4118	ZINC000006030794

0.6	-8.0	-7.8	0.17	1.837	0.773	3.668	1.747	6	4119	ZINC000005457567
0.6	-5.7	-5.6	0.12	2.157	1.066	3.745	1.264	6	4120	ZINC000001706746
0.6	-8.0	-7.6	0.37	1.552	0.185	3.506	0.421	6	4121	ZINC000005762559
0.6	-6.8	-6.4	0.18	2.639	0.555	3.892	0.768	6	4122	ZINC000005239470
0.6	-10.5	-9.3	1.20	2.630	0.000	3.750	0.000	2	4123	ZINC000031622970
0.6	-6.7	-6.5	0.15	2.954	0.832	4.185	0.882	6	4124	ZINC000001843706
0.6	-9.4	-7.7	0.89	1.635	0.179	3.203	0.997	5	4125	ZINC000100778662
0.6	-6.9	-6.8	0.14	1.952	0.248	2.668	0.459	6	4126	ZINC000050027476
0.6	-8.2	-7.8	0.20	2.431	0.734	4.659	2.212	6	4127	ZINC000085552064
0.6	-7.7	-7.4	0.20	2.674	0.527	3.767	0.491	6	4128	ZINC000013480131
0.6	-6.4	-6.1	0.13	1.605	0.596	2.727	0.477	6	4129	ZINC000001591814
0.6	-9.6	-8.4	0.64	1.422	0.310	4.051	2.092	6	4130	ZINC000257474047
0.6	-5.3	-5.1	0.15	2.333	1.326	3.506	0.791	6	4131	ZINC000001435981
0.6	-7.7	-7.6	0.04	2.253	0.728	4.058	1.207	6	4132	ZINC000004025994
0.6	-9.4	-7.6	0.99	1.574	0.275	4.774	1.134	5	4133	ZINC000015061063

0.6	-9.2	-8.2	0.52	1.920	0.283	5.153	1.373	6	4134	ZINC000000899851
0.6	-5.8	-5.6	0.13	2.353	0.296	3.608	0.532	6	4135	ZINC000005839426
0.6	-9.4	-8.3	0.64	1.488	0.304	5.047	1.714	6	4136	ZINC000257470335
0.6	-6.9	-6.5	0.27	1.737	0.424	3.404	1.500	6	4137	ZINC000002561200
0.6	-7.0	-6.6	0.24	2.627	0.347	3.830	0.432	6	4138	ZINC000014588469
0.6	-6.2	-6.0	0.11	2.468	0.373	3.617	0.353	6	4139	ZINC000026671806
0.6	-6.3	-6.1	0.07	2.379	0.444	3.219	0.550	6	4140	ZINC000005751251
0.6	-6.5	-6.3	0.12	2.740	0.768	4.227	0.864	6	4141	ZINC000095618107
0.6	-7.5	-7.2	0.22	1.942	0.419	3.607	1.257	6	4142	ZINC000001841751
0.6	-10.5	-9.1	0.72	1.682	0.132	3.929	1.521	6	4143	ZINC000100781251
0.6	-9.4	-9.1	0.25	1.514	0.868	5.611	2.289	6	4144	ZINC000000391977
0.6	-9.8	-8.2	0.99	2.368	0.787	4.580	1.842	5	4145	ZINC000104883775
0.6	-7.9	-7.6	0.30	1.740	0.173	2.914	0.449	6	4146	ZINC000002560957
0.5	-7.4	-7.3	0.16	1.813	0.567	5.116	1.144	6	4147	ZINC000003785276
0.5	-8.8	-8.2	0.55	1.306	0.000	2.247	0.000	2	4148	ZINC000014587143

0.5	-7.6	-7.2	0.24	1.947	0.757	3.675	2.109	6	4149	ZINC000095618364
0.5	-7.2	-7.0	0.14	1.676	0.554	3.037	0.920	6	4150	ZINC000100136301
0.5	-6.8	-6.3	0.36	2.821	1.173	3.978	1.020	6	4151	ZINC000032163588
0.5	-8.7	-8.0	0.50	1.964	0.411	4.058	0.427	6	4152	ZINC000257558387
0.5	-6.7	-6.4	0.18	2.285	0.597	4.925	0.554	6	4153	ZINC000001731775
0.5	-9.3	-8.8	0.53	1.344	0.186	4.399	1.980	6	4154	ZINC000014642684
0.5	-7.3	-7.0	0.22	2.478	1.065	3.420	1.572	6	4155	ZINC000014504268
0.5	-5.3	-4.9	0.24	2.087	0.666	2.826	0.906	6	4156	ZINC000000388082
0.5	-8.5	-8.1	0.24	1.767	0.133	4.300	1.388	6	4157	ZINC000013509284
0.5	-7.1	-6.9	0.12	2.794	0.509	5.116	1.033	6	4158	ZINC000002522618
0.5	-7.1	-6.7	0.20	2.439	0.372	3.398	0.865	6	4159	ZINC000000156346
0.5	-7.5	-7.4	0.13	2.805	0.473	4.718	1.598	6	4160	ZINC000095617715
0.5	-7.2	-6.8	0.29	2.137	0.338	3.215	1.135	6	4161	ZINC000002530790
0.5	-9.4	-8.4	0.72	2.215	0.342	4.398	2.080	6	4162	ZINC000000338284
0.5	-5.9	-5.9	0.05	2.352	0.980	4.033	0.893	6	4163	ZINC000012496488

0.5	-8.8	-8.4	0.30	0.865	0.596	4.228	2.383	6	4164	ZINC000238763600
0.5	-6.3	-6.1	0.10	1.524	0.208	3.294	0.278	6	4165	ZINC000100778614
0.5	-5.2	-5.0	0.14	2.349	1.673	3.370	1.191	6	4166	ZINC000257481072
0.5	-6.3	-5.8	0.23	2.325	0.537	4.163	0.933	6	4167	ZINC000002048317
0.5	-7.9	-7.6	0.25	1.837	0.172	3.391	0.886	6	4168	ZINC000015147954
0.5	-5.2	-5.0	0.12	2.337	1.663	3.359	1.201	6	4169	ZINC000100829303
0.5	-6.5	-6.3	0.15	2.582	0.356	4.135	0.560	6	4170	ZINC000012153418
0.5	-6.6	-6.4	0.17	2.027	0.832	3.708	1.003	6	4171	ZINC000000967594
0.5	-6.2	-5.9	0.15	2.561	0.348	4.352	0.430	6	4172	ZINC000001575498
0.5	-5.6	-5.5	0.09	3.157	0.721	4.594	0.767	6	4173	ZINC000002581450
0.5	-8.0	-7.7	0.20	2.645	0.440	4.338	0.811	6	4174	ZINC000038392781
0.5	-8.7	-7.9	0.49	1.707	0.239	4.732	2.161	6	4175	ZINC000013382409
0.5	-8.9	-8.4	0.37	1.816	0.345	4.494	1.810	6	4176	ZINC000014590709
0.5	-9.8	-8.3	1.01	2.104	0.402	4.036	1.081	6	4177	ZINC000015053509
0.5	-8.0	-7.6	0.28	1.982	0.689	4.193	1.225	6	4178	ZINC000059778317

0.5	-7.1	-6.9	0.11	1.711	0.487	2.956	0.435	6	4179	ZINC000015119522
0.5	-7.1	-6.9	0.11	2.570	0.475	4.418	1.632	6	4180	ZINC000006018751
0.5	-9.2	-8.2	0.75	1.815	0.737	4.694	1.897	6	4181	ZINC000014616594
0.5	-7.6	-7.3	0.26	2.382	0.686	3.508	1.242	6	4182	ZINC000000400176
0.5	-9.4	-8.2	0.87	1.922	0.143	4.809	1.296	6	4183	ZINC000031155506
0.5	-7.3	-7.0	0.15	1.904	0.345	3.838	0.987	6	4184	ZINC000001850911
0.5	-9.2	-8.4	0.41	2.683	0.870	4.477	1.568	6	4185	ZINC000035967561
0.5	-8.5	-8.2	0.20	1.656	0.313	3.176	1.064	6	4186	ZINC000015160971
0.5	-4.7	-4.5	0.15	1.908	0.473	2.999	0.592	6	4187	ZINC000032150628
0.5	-7.8	-7.4	0.34	2.100	0.271	3.889	0.273	6	4188	ZINC000090697509
0.5	-8.5	-7.8	0.39	1.541	0.331	4.020	1.969	6	4189	ZINC000014919010
0.5	-7.2	-6.8	0.21	2.792	0.639	4.111	1.372	6	4190	ZINC000000895850
0.5	-6.9	-6.6	0.26	1.915	0.612	2.743	0.374	6	4191	ZINC000004141385
0.5	-7.4	-7.2	0.13	2.587	0.385	4.481	0.889	6	4192	ZINC000011568128
0.5	-7.8	-7.3	0.45	1.534	0.375	3.973	1.607	6	4193	ZINC000015115284

0.5	-8.9	-8.2	0.41	2.251	0.160	4.707	0.951	6	4194	ZINC000015263489
0.5	-7.3	-7.1	0.15	2.475	0.982	3.396	1.380	6	4195	ZINC000030691055
0.5	-5.8	-5.6	0.11	2.571	0.333	3.457	0.402	6	4196	ZINC000001674080
0.5	-8.7	-7.9	0.47	1.806	0.269	4.430	0.840	6	4197	ZINC000100825889
0.5	-8.7	-8.2	0.23	1.718	0.316	4.616	1.556	6	4198	ZINC000085923585
0.5	-8.3	-7.6	0.47	2.883	0.888	3.808	1.507	6	4199	ZINC000029566459
0.5	-6.3	-6.2	0.08	2.140	0.969	4.025	0.887	6	4200	ZINC000001700020
0.5	-7.2	-6.8	0.25	2.615	0.453	4.264	1.231	6	4201	ZINC000002534721
0.5	-7.6	-7.4	0.15	2.330	0.490	4.404	1.308	6	4202	ZINC000100779848
0.5	-5.1	-4.9	0.11	2.248	0.418	3.691	0.221	6	4203	ZINC000095619430
0.5	-7.2	-7.0	0.11	1.351	0.338	2.954	0.832	6	4204	ZINC000255987427
0.5	-5.7	-5.5	0.11	2.341	0.314	3.854	0.424	6	4205	ZINC000095620551
0.5	-6.3	-6.1	0.19	2.027	1.105	2.949	1.185	6	4206	ZINC000001641352
0.5	-4.8	-4.7	0.09	2.661	1.301	3.536	0.927	6	4207	ZINC000002002624
0.5	-6.3	-6.2	0.07	2.477	0.749	4.212	0.690	6	4208	ZINC000000388567

0.5	-7.5	-7.2	0.28	2.617	1.280	4.829	2.165	6	4209	ZINC000005226618
0.5	-9.3	-8.8	0.34	1.745	0.525	4.460	2.084	6	4210	ZINC000003604261
0.5	-6.2	-5.9	0.21	1.686	0.324	3.322	0.864	6	4211	ZINC000095620599
0.5	-6.9	-6.6	0.18	1.785	0.452	3.404	1.340	6	4212	ZINC000014619628
0.5	-6.8	-6.5	0.16	2.423	0.464	3.466	0.512	6	4213	ZINC000006071095
0.5	-8.7	-8.3	0.34	1.669	0.592	4.107	0.915	6	4214	ZINC000008234284
0.5	-9.9	-8.3	0.90	1.707	0.219	2.569	0.740	5	4215	ZINC000014681213
0.5	-5.5	-5.2	0.15	3.329	1.140	3.776	1.248	6	4216	ZINC000014451129
0.5	-6.1	-6.0	0.05	2.295	0.147	3.348	0.476	6	4217	ZINC000001849639
0.5	-8.1	-7.4	0.46	1.692	0.336	5.140	2.157	6	4218	ZINC000015113190
0.5	-8.6	-8.0	0.48	2.317	0.672	4.646	1.445	6	4219	ZINC000257615707
0.5	-7.3	-7.1	0.10	2.287	0.391	4.759	1.366	6	4220	ZINC000003156382
0.5	-6.1	-5.9	0.10	1.927	0.713	3.956	0.713	6	4221	ZINC000001408068
0.5	-8.8	-8.1	0.41	1.454	0.456	4.344	2.135	6	4222	ZINC000257411769
0.5	-8.0	-7.3	0.50	2.017	0.181	5.334	0.641	6	4223	ZINC000100770489

0.5	-8.1	-7.6	0.29	2.653	0.203	5.336	0.312	6	4224	ZINC000027731568
0.5	-6.4	-6.3	0.07	1.788	0.819	3.795	1.659	6	4225	ZINC000085972342
0.5	-8.5	-7.1	0.78	1.914	0.277	3.836	0.587	6	4226	ZINC000257741510
0.5	-7.5	-7.3	0.21	2.487	1.204	4.047	2.037	6	4227	ZINC000252444101
0.5	-8.0	-7.4	0.42	1.878	0.453	4.257	1.426	6	4228	ZINC000261499263
0.5	-4.4	-4.3	0.06	3.576	0.288	4.017	0.237	6	4229	ZINC000001850481
0.5	-7.9	-7.5	0.36	2.068	0.284	2.574	0.618	6	4230	ZINC000012496482
0.5	-6.3	-6.0	0.16	3.362	0.719	4.833	0.427	6	4231	ZINC000095620530
0.5	-9.0	-8.8	0.22	2.220	0.782	5.066	1.521	6	4232	ZINC000096014710
0.5	-8.8	-7.4	0.67	1.985	0.293	4.310	1.347	6	4233	ZINC000000899905
0.5	-6.2	-6.0	0.13	2.493	0.408	4.456	0.665	6	4234	ZINC000095620590
0.5	-7.2	-6.7	0.29	2.769	0.768	4.775	1.701	6	4235	ZINC000004556662
0.5	-5.0	-4.9	0.07	3.142	1.407	4.142	1.046	6	4236	ZINC000029786463
0.5	-6.2	-5.9	0.21	2.624	0.658	3.089	0.722	6	4237	ZINC000035653780
0.5	-4.7	-4.5	0.18	1.886	0.813	2.726	0.825	6	4238	ZINC000015204986

0.5	-8.4	-7.8	0.36	2.442	0.467	4.154	1.139	6	4239	ZINC000002391070
0.5	-8.6	-8.3	0.21	2.518	0.318	4.361	0.783	6	4240	ZINC000002560983
0.5	-6.8	-6.4	0.24	2.891	0.497	3.854	0.640	6	4241	ZINC000000157134
0.5	-7.3	-6.7	0.32	2.311	0.737	4.225	1.466	6	4242	ZINC000000085744
0.5	-9.2	-8.2	0.70	1.772	0.276	4.471	1.420	6	4243	ZINC000004026296
0.5	-8.3	-7.7	0.45	1.618	0.981	3.420	0.827	6	4244	ZINC000100780389
0.5	-7.6	-7.4	0.12	2.207	0.192	4.829	0.649	6	4245	ZINC000034961763
0.5	-6.9	-6.5	0.29	2.487	0.225	4.023	0.589	6	4246	ZINC000002560966
0.5	-6.7	-6.6	0.05	2.072	0.405	4.177	1.782	6	4247	ZINC000001532666
0.5	-10.4	-9.7	0.38	2.098	0.399	4.456	1.856	6	4248	ZINC000257498240
0.5	-8.1	-7.4	0.32	1.660	0.114	4.069	1.779	6	4249	ZINC000001589494
0.5	-6.6	-6.2	0.28	2.145	1.267	3.932	0.918	6	4250	ZINC000013460359
0.5	-5.6	-5.4	0.17	2.672	0.865	3.453	0.879	6	4251	ZINC000000164508
0.5	-6.6	-6.2	0.22	2.393	0.181	4.376	0.552	6	4252	ZINC000034946853
0.5	-7.2	-6.9	0.26	1.556	0.504	4.664	0.833	6	4253	ZINC000033822146

0.5	-6.2	-6.0	0.13	2.264	0.811	3.127	1.170	6	4254	ZINC000095619611
0.5	-7.5	-7.1	0.22	2.537	1.150	4.167	1.922	6	4255	ZINC000100297478
0.5	-8.8	-8.4	0.25	2.618	0.205	4.263	0.738	6	4256	ZINC000000056973
0.5	-8.8	-8.5	0.20	1.623	0.251	3.884	0.813	6	4257	ZINC000038198783
0.5	-8.3	-8.1	0.16	1.719	0.380	3.564	1.045	6	4258	ZINC000100779312
0.5	-6.6	-6.3	0.15	1.811	0.313	3.658	0.827	6	4259	ZINC000005179610
0.5	-8.4	-7.7	0.44	1.838	0.061	4.955	1.555	6	4260	ZINC000014919014
0.5	-8.1	-7.8	0.22	2.356	0.454	4.088	2.042	6	4261	ZINC000003590784
0.5	-9.4	-8.2	0.61	2.194	0.396	4.714	0.509	6	4262	ZINC000000135449
0.5	-8.8	-8.0	0.40	2.654	0.552	4.633	1.590	6	4263	ZINC000095619326
0.5	-8.2	-7.7	0.23	2.330	0.741	3.749	1.597	6	4264	ZINC000014719903
0.5	-7.2	-7.0	0.20	2.086	0.473	4.735	1.201	6	4265	ZINC000005923344
0.5	-7.2	-7.0	0.10	1.368	0.347	2.969	0.813	6	4266	ZINC000255987428
0.5	-6.0	-5.6	0.24	2.527	0.119	3.794	0.357	6	4267	ZINC000005134856
0.5	-5.0	-4.8	0.13	2.344	0.601	3.464	1.029	6	4268	ZINC000043618606

0.5	-6.6	-6.4	0.10	2.791	0.969	4.606	0.819	6	4269	ZINC000004529321
0.5	-6.6	-6.2	0.20	2.808	0.661	5.290	1.585	6	4270	ZINC000006361320
0.5	-7.0	-6.8	0.17	2.185	1.055	3.611	1.028	6	4271	ZINC000100779453
0.5	-6.8	-6.6	0.12	2.314	0.557	3.464	1.048	6	4272	ZINC000100469657
0.5	-7.0	-6.7	0.20	2.179	0.293	4.048	1.580	6	4273	ZINC000000156055
0.5	-7.7	-7.4	0.14	1.847	0.229	3.525	0.994	6	4274	ZINC000257433629
0.5	-6.8	-6.7	0.07	1.699	0.677	4.536	0.805	6	4275	ZINC000014822185
0.5	-9.4	-8.5	0.79	1.568	0.218	5.195	1.785	5	4276	ZINC000008379778
0.5	-7.5	-7.2	0.26	3.203	0.657	4.211	0.778	6	4277	ZINC000006471895
0.5	-8.5	-8.2	0.15	1.702	0.513	2.719	0.614	6	4278	ZINC000100155602
0.5	-7.9	-7.6	0.20	1.454	0.085	3.364	0.598	6	4279	ZINC000100830157
0.5	-9.5	-9.1	0.29	0.787	0.088	3.314	0.797	6	4280	ZINC000086035237
0.5	-8.4	-8.2	0.12	2.703	0.618	3.628	1.483	6	4281	ZINC000001708201
0.5	-9.1	-8.8	0.27	1.947	0.355	3.302	1.515	6	4282	ZINC000013382469
0.5	-6.9	-6.5	0.23	1.983	0.911	2.901	1.112	6	4283	ZINC000008318019

0.5	-8.7	-8.3	0.21	1.258	0.104	3.432	1.102	6	4284	ZINC000085599406
0.5	-7.3	-7.1	0.16	2.704	0.645	3.615	0.469	6	4285	ZINC000100823361
0.5	-7.1	-6.9	0.13	2.198	0.253	4.926	0.663	6	4286	ZINC000095620627
0.5	-7.3	-6.8	0.34	2.137	0.708	3.444	1.849	6	4287	ZINC000000050188
0.5	-7.6	-7.2	0.21	1.835	0.105	4.688	1.369	6	4288	ZINC000071758304
0.5	-7.9	-7.4	0.24	2.449	0.446	4.929	1.946	6	4289	ZINC000039326130
0.5	-6.3	-6.1	0.11	3.066	0.610	4.466	0.429	6	4290	ZINC000033844977
0.5	-7.3	-6.9	0.24	2.024	0.438	5.061	1.285	6	4291	ZINC000013340360
0.5	-4.9	-4.8	0.09	2.183	1.677	3.814	0.944	6	4292	ZINC000255976620
0.5	-5.5	-5.2	0.15	3.225	0.847	4.133	0.868	6	4293	ZINC000006037016
0.5	-8.0	-7.2	0.51	2.918	1.148	4.155	1.745	6	4294	ZINC000012494290
0.5	-7.0	-6.8	0.13	2.437	0.949	3.901	0.821	6	4295	ZINC000100779455
0.5	-7.1	-6.9	0.18	1.924	0.758	4.575	1.194	6	4296	ZINC000008860490
0.5	-5.6	-5.4	0.25	1.770	0.659	2.562	0.877	6	4297	ZINC000005225057
0.5	-7.4	-7.2	0.13	1.922	0.348	5.557	1.128	6	4298	ZINC000014714820

0.5	-5.1	-4.8	0.25	3.087	0.475	4.088	0.541	6	4299	ZINC000005761863
0.5	-6.4	-6.0	0.27	1.862	0.414	3.778	0.821	6	4300	ZINC000261499612
0.5	-10.3	-9.2	0.87	1.856	0.289	3.931	1.244	6	4301	ZINC000014819291
0.5	-5.5	-5.3	0.12	2.114	1.297	3.449	1.432	6	4302	ZINC000100772441
0.5	-8.9	-8.6	0.18	1.550	0.616	3.955	1.661	6	4303	ZINC000013340870
0.5	-7.7	-7.4	0.23	2.013	0.555	3.507	1.727	6	4304	ZINC000014684263
0.5	-7.1	-6.8	0.16	2.790	0.866	3.971	1.459	6	4305	ZINC000002522642
0.5	-6.7	-6.6	0.11	3.682	0.720	5.115	0.652	6	4306	ZINC000001576211
0.5	-5.2	-4.9	0.20	1.405	0.322	2.176	0.642	6	4307	ZINC000001850403
0.5	-7.9	-7.6	0.12	3.104	0.495	4.949	0.537	6	4308	ZINC000006091886
0.5	-9.0	-8.5	0.31	1.828	0.278	3.452	0.510	6	4309	ZINC000015220004
0.5	-6.2	-5.8	0.21	1.710	0.453	3.089	0.430	6	4310	ZINC000016892206
0.5	-7.7	-7.3	0.38	2.798	0.923	3.754	1.156	6	4311	ZINC000030731193
0.5	-9.8	-9.1	0.42	1.532	0.463	3.282	0.736	6	4312	ZINC000257436631
0.5	-6.6	-6.4	0.14	2.346	0.317	4.138	0.718	6	4313	ZINC000002566914

0.5	-7.7	-7.6	0.13	1.844	0.708	4.393	1.172	6	4314	ZINC000033955138
0.5	-6.1	-5.9	0.17	2.207	1.083	4.203	0.424	6	4315	ZINC000001672226
0.5	-6.8	-6.7	0.07	1.757	0.917	2.665	0.449	6	4316	ZINC000004098911
0.5	-7.7	-7.6	0.07	2.232	0.957	4.489	0.914	6	4317	ZINC000001687322
0.5	-9.6	-9.0	0.35	1.761	0.493	3.423	0.676	6	4318	ZINC000257525225
0.5	-6.6	-6.5	0.07	2.121	0.654	3.585	1.641	6	4319	ZINC000050027598
0.5	-9.0	-8.4	0.37	2.231	0.279	4.338	1.294	6	4320	ZINC000014774781
0.5	-5.5	-5.5	0.05	1.637	0.581	2.819	0.413	6	4321	ZINC000000967534
0.5	-8.8	-8.3	0.37	2.029	0.193	6.001	0.816	6	4322	ZINC000014854276
0.5	-4.1	-3.8	0.19	2.723	1.014	3.831	0.991	6	4323	ZINC000002031649
0.5	-8.1	-7.9	0.17	1.860	0.445	3.020	0.868	6	4324	ZINC000100781106
0.5	-8.0	-7.6	0.38	1.708	0.548	3.959	1.667	6	4325	ZINC000261499261
0.5	-5.4	-5.1	0.20	3.018	0.660	3.864	0.638	6	4326	ZINC000001850854
0.5	-9.7	-8.3	0.93	1.432	0.165	4.335	1.543	6	4327	ZINC000014859457
0.5	-6.2	-6.0	0.13	2.693	0.355	3.656	0.512	6	4328	ZINC000095620532

0.5	-7.5	-6.9	0.43	2.178	0.366	5.143	0.683	6	4329	ZINC000014616309
0.5	-7.9	-7.5	0.28	2.427	0.218	5.771	0.687	6	4330	ZINC000085808389
0.5	-6.7	-6.5	0.09	1.968	0.171	3.414	0.966	6	4331	ZINC000038654420
0.5	-6.3	-5.9	0.19	2.287	0.637	4.128	0.649	6	4332	ZINC000014614316
0.5	-8.7	-8.5	0.19	1.079	0.052	4.331	2.406	6	4333	ZINC000013340107
0.5	-6.8	-6.6	0.13	1.749	0.882	3.308	0.270	6	4334	ZINC000000900176
0.5	-7.8	-7.6	0.22	1.671	0.839	4.147	1.854	6	4335	ZINC000095620810
0.5	-7.3	-7.2	0.04	1.241	0.458	2.725	0.477	6	4336	ZINC000100779746
0.5	-9.0	-8.4	0.31	2.234	0.295	4.332	1.302	6	4337	ZINC000014774784
0.5	-5.9	-5.4	0.22	2.159	0.889	2.969	1.175	6	4338	ZINC000003860658
0.5	-8.3	-7.8	0.30	2.036	0.562	4.354	1.400	6	4339	ZINC000006037122
0.5	-7.7	-7.4	0.21	1.927	0.654	3.277	1.412	6	4340	ZINC000257705866
0.5	-9.0	-8.7	0.29	1.574	0.389	4.825	0.742	6	4341	ZINC000013383922
0.5	-9.5	-9.1	0.30	0.776	0.133	3.318	0.788	6	4342	ZINC000059588754
0.5	-8.0	-7.8	0.17	1.595	0.349	3.796	0.494	6	4343	ZINC000012496734

0.5	-7.2	-6.9	0.14	1.882	0.165	3.937	0.756	6	4344	ZINC000014818089
0.5	-8.1	-7.9	0.13	1.603	0.416	2.635	0.691	6	4345	ZINC000014614797
0.5	-7.8	-7.4	0.26	2.029	0.585	3.603	1.700	6	4346	ZINC000000895841
0.5	-6.8	-6.6	0.11	2.105	0.583	3.320	0.720	6	4347	ZINC000000156504
0.5	-8.3	-7.8	0.32	2.535	0.095	5.150	0.198	6	4348	ZINC000050027626
0.5	-7.3	-7.0	0.21	1.643	0.387	3.647	0.450	6	4349	ZINC000209934352
0.5	-8.4	-7.9	0.27	2.356	0.285	4.912	1.417	6	4350	ZINC000027646107
0.5	-8.1	-7.7	0.27	2.316	0.287	3.360	1.157	6	4351	ZINC000000895330
0.5	-8.1	-7.8	0.23	2.742	1.237	3.826	1.516	6	4352	ZINC000030728433
0.5	-7.5	-7.3	0.17	1.801	0.602	3.189	0.711	6	4353	ZINC000001664401
0.5	-6.1	-5.8	0.18	1.320	0.506	2.984	0.434	6	4354	ZINC000032169152
0.5	-9.0	-7.9	0.53	2.497	1.164	4.116	1.734	6	4355	ZINC000003860156
0.5	-9.8	-8.9	0.55	2.339	0.459	4.073	1.136	6	4356	ZINC000053275063
0.5	-7.0	-6.8	0.12	1.656	0.733	2.991	1.747	6	4357	ZINC000029488972
0.5	-4.8	-4.5	0.24	2.145	0.562	2.924	0.714	6	4358	ZINC000001697400

0.5	-6.9	-6.7	0.13	2.145	0.641	3.444	0.663	6	4359	ZINC000003846058
0.5	-6.6	-6.3	0.15	1.987	0.324	4.141	0.688	6	4360	ZINC000014638745
0.5	-7.0	-6.7	0.21	2.711	0.295	4.909	0.695	6	4361	ZINC000038417737
0.5	-8.6	-7.9	0.47	1.951	0.424	5.058	1.823	6	4362	ZINC000014455179
0.5	-8.1	-7.8	0.17	2.623	0.814	4.907	0.982	6	4363	ZINC000005115783
0.5	-7.6	-7.1	0.25	2.310	0.218	4.104	0.652	6	4364	ZINC000001850337
0.5	-7.0	-6.8	0.15	1.602	0.703	2.744	0.285	6	4365	ZINC000257411065
0.5	-7.8	-7.6	0.16	2.601	0.439	4.166	1.824	6	4366	ZINC000000001601
0.5	-5.6	-5.3	0.19	1.814	0.410	3.128	0.543	6	4367	ZINC000095618215
0.5	-6.0	-5.7	0.18	2.387	0.393	3.568	0.277	6	4368	ZINC000002027353
0.5	-7.1	-7.0	0.08	2.439	0.106	4.365	0.953	6	4369	ZINC000004556546
0.5	-8.1	-7.0	0.61	1.579	0.284	4.492	1.681	6	4370	ZINC000014436005
0.5	-9.1	-7.9	1.02	1.599	0.276	4.081	1.836	3	4371	ZINC000014587138
0.5	-8.3	-7.9	0.31	2.408	0.116	4.161	0.519	6	4372	ZINC000013387719
0.5	-6.6	-6.3	0.19	2.120	0.588	3.601	0.861	6	4373	ZINC000014588464

0.5	-6.9	-6.7	0.13	1.829	0.310	3.654	0.695	6	4374	ZINC000003846060
0.5	-9.5	-8.4	0.86	1.823	0.206	3.984	1.277	6	4375	ZINC000015059547
0.5	-7.2	-6.9	0.23	2.198	0.589	3.704	0.760	6	4376	ZINC000014828986
0.5	-9.1	-8.7	0.37	1.375	0.755	4.552	0.942	6	4377	ZINC000001996094
0.5	-8.0	-7.7	0.22	2.223	0.454	6.510	0.228	6	4378	ZINC000033650330
0.5	-5.7	-5.5	0.13	2.214	0.689	3.400	0.444	6	4379	ZINC000032150481
0.5	-5.6	-5.4	0.21	2.896	0.863	3.527	1.374	6	4380	ZINC000005543437
0.5	-7.0	-6.6	0.22	1.725	0.232	2.783	0.809	6	4381	ZINC000000002137
0.5	-10.3	-9.8	0.35	1.103	0.181	2.876	0.816	6	4382	ZINC000100776086
0.5	-5.3	-4.7	0.32	2.715	0.553	3.503	0.721	6	4383	ZINC000005822249
0.5	-7.4	-7.1	0.17	1.430	0.141	3.200	0.478	6	4384	ZINC000006031050
0.5	-6.3	-6.0	0.14	2.318	0.568	4.331	0.478	6	4385	ZINC000002567986
0.5	-10.0	-9.2	0.73	2.009	0.106	4.128	1.423	6	4386	ZINC000100780658
0.5	-8.3	-7.8	0.46	2.174	0.765	3.846	0.495	6	4387	ZINC000100780390
0.5	-6.9	-6.7	0.22	1.849	1.025	2.764	1.020	6	4388	ZINC000000161958

0.5	-8.9	-8.4	0.38	1.885	0.470	5.146	1.888	6	4389	ZINC000257767903
0.5	-9.1	-8.5	0.32	1.745	0.135	3.729	0.710	6	4390	ZINC000257557769
0.5	-7.5	-7.3	0.20	2.166	0.756	3.523	0.683	6	4391	ZINC000014723446
0.5	-6.4	-6.0	0.18	2.864	0.735	4.362	0.485	6	4392	ZINC000002577416
0.5	-9.0	-8.5	0.36	2.614	1.100	4.343	1.997	6	4393	ZINC000014445254
0.5	-8.6	-7.6	0.60	1.874	0.210	4.863	1.441	6	4394	ZINC000261497626
0.5	-6.4	-6.0	0.18	2.713	0.586	4.375	1.004	6	4395	ZINC000038251275
0.5	-10.3	-9.7	0.45	1.068	0.144	3.156	0.603	6	4396	ZINC000257473765
0.5	-8.5	-8.1	0.21	2.663	1.096	3.461	1.741	6	4397	ZINC000255984290
0.5	-6.5	-6.3	0.17	1.982	0.594	4.062	0.866	6	4398	ZINC000002566007
0.5	-7.1	-6.9	0.15	2.551	0.420	4.609	0.437	6	4399	ZINC000034951648
0.5	-7.9	-7.3	0.31	1.896	0.131	4.168	0.870	6	4400	ZINC000004098732
0.5	-7.7	-7.3	0.25	2.705	1.260	4.890	1.863	6	4401	ZINC000004097100
0.5	-8.0	-6.7	0.95	1.964	0.394	3.544	1.754	6	4402	ZINC000021985929
0.5	-6.9	-6.7	0.11	1.834	0.467	4.927	2.216	6	4403	ZINC000014439790

0.5	-8.6	-8.0	0.41	1.750	0.616	4.276	0.443	6	4404	ZINC000231138600
0.5	-5.5	-5.2	0.14	3.052	0.787	3.683	0.706	6	4405	ZINC000006036939
0.5	-6.4	-6.2	0.13	2.100	0.296	3.496	0.852	6	4406	ZINC000004521128
0.5	-9.6	-8.4	0.76	2.278	0.231	5.348	1.776	6	4407	ZINC000257430013
0.5	-5.3	-5.1	0.21	2.493	1.147	3.418	1.209	6	4408	ZINC000002041110
0.5	-7.1	-6.9	0.16	2.629	0.575	3.885	1.559	6	4409	ZINC000040165321
0.5	-8.9	-8.5	0.32	2.070	0.224	4.789	1.624	6	4410	ZINC000000119985
0.5	-8.0	-7.9	0.08	1.789	0.304	5.513	1.629	6	4411	ZINC000013340552
0.5	-5.4	-5.2	0.16	2.073	1.285	4.115	0.719	6	4412	ZINC000015042997
0.5	-7.0	-6.8	0.12	1.992	0.682	3.348	1.688	6	4413	ZINC000038417739
0.5	-6.1	-5.6	0.23	2.263	0.194	3.624	0.707	6	4414	ZINC000002004603
0.5	-5.9	-5.8	0.06	1.781	0.436	2.842	0.907	6	4415	ZINC000001638432
0.5	-5.6	-5.2	0.22	1.849	0.652	3.275	1.048	6	4416	ZINC000002164143
0.5	-7.7	-7.5	0.18	2.436	0.685	4.335	2.001	6	4417	ZINC000040471351
0.5	-7.1	-6.9	0.17	2.223	0.208	3.810	0.713	6	4418	ZINC000013380486

0.5	-5.9	-5.9	0.05	1.445	0.931	3.113	0.734	6	4419	ZINC000000968269
0.5	-7.4	-7.1	0.19	2.051	1.001	3.316	1.445	6	4420	ZINC000002015646
0.5	-6.6	-6.3	0.13	2.627	0.151	4.562	0.691	6	4421	ZINC000012493697
0.5	-7.0	-6.6	0.27	2.771	0.385	4.728	0.463	6	4422	ZINC000050027382
0.5	-8.4	-7.9	0.24	3.325	0.323	5.937	1.313	6	4423	ZINC000013681007
0.5	-7.6	-7.3	0.16	1.981	0.136	3.768	0.410	6	4424	ZINC000257545326
0.5	-6.6	-6.4	0.12	1.874	0.624	3.922	0.796	6	4425	ZINC000014590681
0.5	-7.8	-7.4	0.20	2.600	0.737	5.369	1.370	6	4426	ZINC000014590684
0.5	-8.3	-8.0	0.18	1.805	0.328	3.794	1.447	6	4427	ZINC000014768812
0.5	-9.2	-8.8	0.22	1.937	0.081	5.937	0.826	6	4428	ZINC000013515177
0.5	-5.7	-5.4	0.13	3.237	0.389	3.970	0.395	6	4429	ZINC000000159554
0.5	-10.1	-9.0	0.56	1.502	0.314	3.750	1.050	6	4430	ZINC000015206027
0.5	-6.4	-6.2	0.13	1.992	0.383	3.551	0.472	6	4431	ZINC000033610757
0.5	-4.9	-4.7	0.12	2.400	1.076	2.959	0.924	6	4432	ZINC000000407074
0.5	-7.0	-6.5	0.37	3.868	0.664	4.624	0.794	6	4433	ZINC000000154564

0.5	-7.2	-6.9	0.23	2.075	0.589	3.734	1.979	6	4434	ZINC000085738439
0.5	-7.3	-7.0	0.16	2.286	0.318	4.383	1.500	6	4435	ZINC000095617719
0.5	-7.0	-6.8	0.25	1.744	0.468	3.281	0.710	6	4436	ZINC000002566202
0.5	-5.8	-5.6	0.15	1.489	0.551	3.225	0.619	6	4437	ZINC000004501392
0.5	-8.6	-8.6	0.04	1.152	0.454	2.308	0.432	6	4438	ZINC000014684782
0.5	-6.6	-6.2	0.21	2.552	0.609	3.175	0.570	6	4439	ZINC000000157414
0.5	-5.3	-5.1	0.13	0.750	0.388	2.570	0.234	6	4440	ZINC000001574315
0.5	-7.0	-6.8	0.13	2.319	0.333	2.946	0.445	6	4441	ZINC000006092467
0.5	-8.8	-6.8	0.95	2.247	0.348	3.939	1.371	6	4442	ZINC000014763461
0.5	-8.0	-7.8	0.19	1.943	0.082	3.957	0.203	6	4443	ZINC000005766881
0.5	-6.8	-6.4	0.20	2.813	0.527	5.096	0.889	6	4444	ZINC000040472596
0.5	-8.2	-7.7	0.30	2.459	1.126	3.426	1.498	6	4445	ZINC000001605732
0.5	-7.3	-7.1	0.19	2.725	0.654	3.628	0.503	6	4446	ZINC000095619238
0.5	-6.9	-6.6	0.18	2.045	0.443	3.298	0.536	6	4447	ZINC000000388085
0.5	-8.0	-7.7	0.22	2.474	0.460	4.059	0.853	6	4448	ZINC000035874817

0.5	-6.5	-6.1	0.18	2.371	0.152	3.836	0.532	6	4449	ZINC000004096278
0.5	-8.8	-8.4	0.27	1.452	0.372	3.227	1.895	6	4450	ZINC000000897738
0.5	-7.2	-7.0	0.13	1.715	0.497	2.813	0.753	6	4451	ZINC000100781742
0.5	-7.2	-6.8	0.29	2.806	0.205	5.440	0.476	6	4452	ZINC000100891527
0.5	-8.6	-8.4	0.30	1.669	0.771	5.715	1.729	6	4453	ZINC000014647927
0.5	-8.0	-7.6	0.26	1.992	0.693	4.588	2.280	6	4454	ZINC000040470654
0.5	-5.5	-5.2	0.22	1.878	0.784	3.204	0.740	6	4455	ZINC000002584406
0.5	-7.2	-7.0	0.19	1.915	0.364	3.903	1.284	6	4456	ZINC000005923355
0.5	-6.0	-5.8	0.17	1.978	0.702	3.818	0.814	6	4457	ZINC000002038279
0.5	-7.2	-6.9	0.20	2.210	1.332	3.042	1.535	6	4458	ZINC000004654887
0.5	-7.3	-7.1	0.11	2.356	0.443	5.139	0.725	6	4459	ZINC000040165324
0.5	-10.2	-9.9	0.23	1.512	0.270	4.540	2.110	6	4460	ZINC000003869685
0.5	-6.1	-5.6	0.29	2.078	0.807	3.745	0.732	6	4461	ZINC000012495724
0.5	-5.4	-5.2	0.10	2.182	0.945	3.904	0.838	6	4462	ZINC000002034328
0.5	-5.1	-4.9	0.10	2.062	0.562	3.433	0.962	6	4463	ZINC000005820719

0.5	-7.5	-7.3	0.18	2.100	0.083	4.791	1.035	6	4464	ZINC000000002169
0.5	-8.9	-8.3	0.40	1.872	0.243	5.756	1.658	6	4465	ZINC000014854255
0.5	-9.5	-7.9	0.98	1.823	0.227	4.693	1.544	6	4466	ZINC000013302908
0.5	-5.9	-5.3	0.28	2.266	1.009	4.030	0.710	6	4467	ZINC000001850619
0.5	-6.5	-6.2	0.22	1.782	0.526	3.169	1.232	6	4468	ZINC000001693678
0.5	-5.0	-4.9	0.08	2.483	1.100	4.516	0.677	6	4469	ZINC000002015915
0.5	-7.5	-7.2	0.18	1.815	0.825	3.022	0.790	6	4470	ZINC000004098901
0.5	-6.0	-5.8	0.14	1.739	0.359	3.260	0.794	6	4471	ZINC000002040376
0.5	-4.5	-4.4	0.11	2.116	0.233	3.204	0.764	6	4472	ZINC000001648986
0.5	-5.2	-4.8	0.18	1.835	0.570	3.049	0.650	6	4473	ZINC000001586750
0.5	-9.1	-8.4	0.34	2.988	0.320	5.525	0.998	6	4474	ZINC000015115002
0.5	-7.2	-7.0	0.11	1.583	0.929	3.576	0.782	6	4475	ZINC000014764736
0.5	-6.7	-6.5	0.11	2.394	0.831	3.913	1.947	6	4476	ZINC000005410797
0.5	-10.4	-9.1	0.93	1.869	0.177	4.245	1.491	5	4477	ZINC000100780584
0.5	-8.6	-7.9	0.43	1.826	0.221	5.759	0.535	6	4478	ZINC000014504521

0.5	-7.1	-6.9	0.13	1.838	0.639	3.092	0.622	6	4479	ZINC000100779755
0.5	-8.5	-8.4	0.09	2.308	0.267	5.626	1.501	6	4480	ZINC000033832846
0.5	-6.7	-6.4	0.24	2.731	0.468	3.723	0.635	6	4481	ZINC000000409286
0.5	-7.3	-7.1	0.15	2.745	0.622	4.476	0.361	6	4482	ZINC000027639979
0.5	-9.0	-8.3	0.36	2.424	0.706	3.837	1.583	6	4483	ZINC000004228242
0.5	-7.9	-7.7	0.15	1.266	0.324	4.675	1.635	6	4484	ZINC000013522693
0.5	-7.1	-6.9	0.15	2.218	0.380	3.605	0.458	6	4485	ZINC000005759011
0.5	-8.5	-8.1	0.29	1.870	0.322	4.076	1.475	6	4486	ZINC000085589183
0.5	-6.7	-6.4	0.16	1.593	0.455	2.492	1.664	6	4487	ZINC000001576295
0.5	-5.3	-5.2	0.08	2.888	1.154	3.634	1.123	6	4488	ZINC000014448311
0.5	-4.9	-4.7	0.12	3.167	0.790	3.421	0.580	6	4489	ZINC000001722340
0.5	-5.4	-5.2	0.08	2.307	0.962	3.835	0.556	6	4490	ZINC000001850303
0.5	-7.7	-7.4	0.26	2.743	0.771	4.791	1.815	6	4491	ZINC000001708195
0.5	-8.5	-8.2	0.25	1.767	0.696	4.004	1.566	6	4492	ZINC000257387899
0.5	-4.5	-4.4	0.07	3.006	0.631	3.428	0.534	6	4493	ZINC000013449981

0.5	-7.1	-6.9	0.10	2.294	0.507	4.810	0.240	6	4494	ZINC000050027588
0.5	-6.3	-5.8	0.24	1.887	1.084	2.913	1.091	6	4495	ZINC000002039295
0.5	-8.9	-8.5	0.34	1.989	0.196	5.646	1.069	6	4496	ZINC000257767899
0.5	-7.1	-6.5	0.34	2.203	0.293	3.602	0.433	6	4497	ZINC000001693657
0.5	-9.8	-9.2	0.36	1.723	0.548	3.207	0.860	6	4498	ZINC000257436629
0.5	-7.7	-7.4	0.33	1.960	0.382	5.135	1.550	6	4499	ZINC000256078938
0.5	-7.2	-6.9	0.19	2.004	0.430	4.134	0.937	6	4500	ZINC000015213104
0.5	-7.1	-6.9	0.10	2.211	0.471	3.939	1.200	6	4501	ZINC000001532632
0.5	-10.4	-9.2	0.74	1.534	0.309	4.941	1.147	6	4502	ZINC000100781260
0.5	-10.9	-9.7	1.17	2.208	0.667	4.104	1.093	6	4503	ZINC000013533343
0.5	-6.6	-6.2	0.23	2.442	0.327	3.396	0.499	6	4504	ZINC000000164490
0.5	-5.9	-5.7	0.10	2.910	1.135	3.825	1.109	6	4505	ZINC000001995292
0.5	-7.8	-7.6	0.13	1.644	0.416	3.004	0.756	6	4506	ZINC000008700366
0.5	-7.6	-7.3	0.18	2.024	0.955	3.548	0.622	6	4507	ZINC000013130109
0.5	-8.3	-7.6	0.37	1.817	0.299	3.499	0.754	6	4508	ZINC000013383221

0.5	-6.9	-6.3	0.33	2.325	0.419	4.283	0.716	6	4509	ZINC000004556677
0.5	-8.5	-8.2	0.20	2.179	0.286	4.003	0.922	6	4510	ZINC000001867781
0.5	-6.0	-5.8	0.11	3.122	0.626	4.317	0.473	6	4511	ZINC000001708199
0.5	-5.3	-5.0	0.14	2.183	1.142	4.039	1.221	6	4512	ZINC000002019414
0.5	-7.9	-7.6	0.21	1.293	0.288	3.344	0.588	6	4513	ZINC000100830155
0.5	-8.6	-8.3	0.24	1.764	0.574	4.821	1.081	6	4514	ZINC000004098623
0.5	-7.3	-7.2	0.07	1.971	0.701	4.485	1.967	6	4515	ZINC000014684306
0.5	-7.6	-7.3	0.29	2.260	0.445	3.858	1.295	6	4516	ZINC000095617745
0.5	-7.3	-7.1	0.17	2.328	0.688	3.535	0.381	6	4517	ZINC000100823358
0.5	-6.3	-5.9	0.22	2.826	0.758	4.029	1.401	6	4518	ZINC000028631778
0.5	-7.4	-7.3	0.11	2.304	0.436	3.910	1.278	6	4519	ZINC000040479013
0.5	-5.8	-5.5	0.17	2.738	0.945	4.258	0.828	6	4520	ZINC000001850534
0.5	-9.9	-9.0	0.56	1.691	0.141	5.229	0.963	6	4521	ZINC000085889337
0.5	-6.2	-6.1	0.09	2.270	0.329	4.904	0.400	6	4522	ZINC000085972376
0.5	-6.2	-6.0	0.14	2.140	1.155	3.981	0.913	6	4523	ZINC000014591088

0.5	-6.6	-6.3	0.20	2.983	0.368	5.911	0.068	6	4524	ZINC000040439733
0.5	-7.2	-7.0	0.15	1.404	0.176	2.541	0.820	6	4525	ZINC000100781729
0.5	-6.7	-6.1	0.29	2.847	0.684	3.797	0.730	6	4526	ZINC000000331715
0.5	-6.0	-5.6	0.27	2.014	0.733	3.471	1.032	6	4527	ZINC000014723523
0.5	-6.8	-6.7	0.09	2.835	0.860	4.317	1.040	6	4528	ZINC000001576892
0.5	-6.0	-5.8	0.13	2.482	0.706	4.167	0.947	6	4529	ZINC000032180904
0.5	-5.7	-5.4	0.24	2.958	1.128	4.245	1.012	6	4530	ZINC000032153276
0.5	-8.6	-8.0	0.38	2.261	0.724	3.655	1.628	6	4531	ZINC000013480041
0.5	-6.8	-6.5	0.19	2.103	0.495	4.149	0.516	6	4532	ZINC000001851030
0.5	-7.3	-7.1	0.15	1.869	0.179	4.561	1.499	6	4533	ZINC000004726637
0.5	-9.2	-8.0	0.73	1.767	0.314	3.375	0.786	6	4534	ZINC000004096994
0.5	-8.1	-7.8	0.16	2.444	0.734	4.506	2.033	6	4535	ZINC000085552062
0.5	-9.1	-8.8	0.22	2.654	1.009	4.700	1.485	6	4536	ZINC000095620822
0.5	-4.4	-4.2	0.14	3.471	2.080	4.291	1.676	6	4537	ZINC000005783274
0.5	-5.8	-5.7	0.12	2.075	0.319	3.822	0.305	6	4538	ZINC000002164098

0.5	-5.9	-5.6	0.18	2.463	1.067	3.343	1.511	6	4539	ZINC000002016613
0.5	-5.4	-5.3	0.09	2.312	0.577	3.440	1.021	6	4540	ZINC000078160318
0.5	-5.9	-5.7	0.12	2.311	0.611	3.212	0.154	6	4541	ZINC000024398176
0.5	-6.1	-6.0	0.13	2.227	1.029	3.388	1.071	6	4542	ZINC000001586382
0.5	-9.1	-8.7	0.22	1.446	0.144	2.907	1.125	6	4543	ZINC000014449461
0.5	-5.0	-4.9	0.07	3.036	1.553	3.779	1.111	6	4544	ZINC000000003184
0.5	-6.2	-6.2	0.05	2.045	0.749	3.552	1.351	6	4545	ZINC000034595211
0.5	-8.8	-8.3	0.37	2.090	0.420	4.163	0.408	6	4546	ZINC000013381473
0.5	-4.7	-4.5	0.11	2.372	0.314	2.966	0.521	6	4547	ZINC000002509929
0.5	-5.0	-4.6	0.17	2.613	0.977	3.307	1.154	6	4548	ZINC000032150846
0.5	-5.5	-5.1	0.23	2.585	0.926	3.107	1.156	6	4549	ZINC000003645145
0.5	-6.7	-6.5	0.13	2.859	0.593	4.514	0.608	6	4550	ZINC000004531655
0.5	-5.4	-5.4	0.08	1.912	0.747	2.444	0.753	6	4551	ZINC000014590677
0.5	-6.8	-6.5	0.23	2.829	0.515	5.057	0.186	6	4552	ZINC000014822501
0.5	-7.9	-7.7	0.21	2.710	0.519	5.079	1.465	6	4553	ZINC000014858366

0.5	-8.7	-7.9	0.70	1.798	0.407	4.960	2.148	3	4554	ZINC000014820489
0.5	-8.6	-8.1	0.35	2.496	0.712	3.865	1.483	6	4555	ZINC000257427190
0.5	-8.3	-7.5	0.40	3.293	0.661	5.207	1.071	6	4556	ZINC000040472351
0.5	-7.0	-6.8	0.09	2.940	0.558	4.893	0.380	6	4557	ZINC000069485822
0.5	-9.5	-8.9	0.52	1.651	0.083	4.130	1.397	5	4558	ZINC000100780893
0.5	-10.0	-8.6	0.77	1.499	0.195	4.556	1.696	6	4559	ZINC000001319752
0.5	-7.5	-7.1	0.20	2.058	0.610	3.579	1.323	6	4560	ZINC000002391078
0.5	-6.7	-6.6	0.07	2.168	0.282	3.504	1.084	6	4561	ZINC000040484081
0.5	-8.5	-8.3	0.08	1.457	0.141	3.342	1.799	6	4562	ZINC000015218409
0.5	-7.1	-6.9	0.15	2.387	0.632	4.021	0.797	6	4563	ZINC000004556673
0.5	-8.1	-7.4	0.62	1.506	0.512	3.668	1.895	6	4564	ZINC000257427754
0.5	-6.8	-6.7	0.07	2.214	0.829	3.237	0.887	6	4565	ZINC000003860771
0.5	-8.0	-7.5	0.37	1.684	0.500	5.141	2.031	6	4566	ZINC000261499260
0.5	-6.4	-6.2	0.17	3.316	1.482	4.580	1.698	6	4567	ZINC000095620548
0.5	-9.9	-8.5	0.87	1.785	0.357	4.540	0.430	6	4568	ZINC000014612852

0.5	-7.1	-6.5	0.29	2.451	0.403	4.751	1.169	6	4569	ZINC000001605718
0.5	-8.0	-7.8	0.24	1.648	0.751	5.941	1.686	6	4570	ZINC000014817868
0.5	-7.4	-7.3	0.07	1.617	0.160	4.289	2.393	6	4571	ZINC000014725861
0.5	-9.3	-7.9	1.05	1.682	0.368	4.586	1.793	5	4572	ZINC000013545017
0.5	-6.9	-6.7	0.15	1.785	0.441	2.779	0.670	6	4573	ZINC000100066097
0.5	-8.5	-8.0	0.45	1.623	0.269	2.664	0.503	6	4574	ZINC000013540500
0.5	-9.8	-9.2	0.41	1.399	0.515	3.368	1.121	6	4575	ZINC000015160930
0.5	-8.0	-7.7	0.28	2.893	0.858	4.653	1.476	6	4576	ZINC000012496759
0.5	-5.0	-4.9	0.11	1.798	0.296	3.106	0.754	6	4577	ZINC000001615324
0.5	-6.5	-6.2	0.22	2.693	0.958	4.012	1.109	6	4578	ZINC000032135581
0.5	-6.9	-6.5	0.22	2.345	0.322	4.469	1.008	6	4579	ZINC000013347379
0.5	-9.1	-8.8	0.16	1.684	0.413	4.189	1.487	6	4580	ZINC000013143013
0.5	-8.4	-7.9	0.28	1.826	0.222	5.386	1.151	6	4581	ZINC000014589774
0.5	-7.3	-7.0	0.21	2.161	0.710	3.506	1.425	6	4582	ZINC000014588971
0.5	-5.5	-5.2	0.14	3.053	0.941	4.110	0.840	6	4583	ZINC000014451123

0.5	-8.3	-8.1	0.16	1.314	0.625	2.584	0.351	6	4584	ZINC000034461449
0.5	-6.5	-6.2	0.17	2.864	0.938	3.940	1.022	6	4585	ZINC000095620501
0.5	-7.5	-7.2	0.21	1.958	0.828	3.266	0.700	6	4586	ZINC000004654766
0.5	-7.7	-7.4	0.28	2.338	0.411	3.583	1.070	6	4587	ZINC000034273694
0.5	-7.5	-7.4	0.09	2.203	0.059	3.839	0.791	6	4588	ZINC000257563117
0.5	-7.1	-6.9	0.14	2.307	0.422	3.889	1.546	6	4589	ZINC000040165322
0.5	-7.3	-7.1	0.15	2.053	0.461	3.545	0.612	6	4590	ZINC000038329368
0.5	-5.5	-5.1	0.18	3.477	1.723	4.435	1.905	6	4591	ZINC000095617999
0.5	-6.8	-6.3	0.26	1.865	0.628	2.907	0.630	6	4592	ZINC000000900396
0.5	-8.0	-7.4	0.32	2.163	0.464	4.841	0.162	6	4593	ZINC000002391137
0.5	-6.8	-6.5	0.15	1.938	0.687	3.832	1.145	6	4594	ZINC000012493883
0.5	-7.1	-6.9	0.13	1.843	0.630	3.100	0.621	6	4595	ZINC000257389433
0.5	-8.5	-8.0	0.41	1.848	0.543	3.623	1.469	6	4596	ZINC000013540496
0.5	-7.7	-7.2	0.39	2.047	0.491	2.951	1.465	6	4597	ZINC000001579826
0.5	-6.5	-6.4	0.11	2.410	1.079	3.312	1.652	6	4598	ZINC000095618104

0.5	-6.9	-6.5	0.34	2.391	1.227	4.159	1.530	6	4599	ZINC000095619767
0.5	-8.8	-8.3	0.41	2.071	0.228	4.436	1.003	6	4600	ZINC000015263559
0.5	-7.5	-7.3	0.11	2.526	0.758	4.196	0.482	6	4601	ZINC000006067069
0.5	-7.9	-7.6	0.18	1.856	0.339	3.610	0.499	6	4602	ZINC000014614595
0.5	-8.0	-7.6	0.33	1.916	0.544	4.916	1.717	6	4603	ZINC000015259902
0.5	-7.4	-7.2	0.15	1.520	0.507	3.836	1.473	6	4604	ZINC000011681054
0.5	-9.4	-8.7	0.68	1.284	0.354	2.555	0.601	6	4605	ZINC000014919099
0.5	-6.9	-6.7	0.17	1.996	0.583	3.087	0.802	6	4606	ZINC000100778138
0.5	-5.9	-5.5	0.18	2.307	0.328	3.804	0.806	6	4607	ZINC000003644165
0.5	-8.0	-7.8	0.15	2.123	0.265	3.591	0.445	6	4608	ZINC000257554747
0.5	-6.2	-6.0	0.14	2.296	0.556	4.495	0.556	6	4609	ZINC000004642624
0.5	-5.6	-5.4	0.18	1.479	0.321	3.123	0.440	6	4610	ZINC000014724461
0.5	-10.0	-8.4	0.72	2.199	0.361	4.341	1.591	6	4611	ZINC000014612197
0.5	-6.9	-6.6	0.16	3.066	0.393	4.517	0.569	6	4612	ZINC000001676257
0.5	-6.9	-6.7	0.15	2.069	0.669	3.732	0.693	6	4613	ZINC000005735761

0.5	-6.7	-6.5	0.08	2.035	0.518	3.620	1.083	6	4614	ZINC000002037928
0.5	-6.1	-5.9	0.13	1.493	0.230	3.563	0.521	6	4615	ZINC000002572383
0.5	-7.2	-6.9	0.18	2.566	0.743	4.099	1.135	6	4616	ZINC000002038847
0.5	-5.2	-5.0	0.08	1.616	0.328	3.164	0.195	6	4617	ZINC000245228117
0.5	-7.2	-7.0	0.16	1.611	0.735	2.641	1.363	6	4618	ZINC000013341254
0.5	-7.7	-7.4	0.20	2.240	0.427	3.892	1.443	6	4619	ZINC000040487039
0.5	-6.7	-6.4	0.22	3.075	0.300	4.314	0.534	6	4620	ZINC000095620547
0.5	-5.6	-5.4	0.17	2.433	0.895	3.287	0.901	6	4621	ZINC000002557901
0.5	-8.3	-7.2	0.51	1.941	0.214	5.162	0.929	6	4622	ZINC000100770152
0.5	-7.4	-7.0	0.37	1.954	0.826	2.601	0.802	6	4623	ZINC000034499762
0.5	-8.6	-8.1	0.52	2.152	0.584	4.545	1.589	6	4624	ZINC000017141863
0.5	-8.1	-6.7	1.01	1.594	1.026	4.748	1.390	6	4625	ZINC000095619687
0.5	-7.2	-7.0	0.11	1.283	0.294	2.915	0.813	6	4626	ZINC000015169334
0.5	-8.1	-7.8	0.19	3.149	0.291	5.580	0.760	6	4627	ZINC000003869856
0.5	-8.7	-8.2	0.29	1.757	0.376	3.747	1.190	6	4628	ZINC000014453725

0.5	-7.0	-6.6	0.26	2.636	0.452	3.332	0.833	6	4629	ZINC000005141599
0.5	-7.0	-6.4	0.27	1.849	0.231	3.621	1.486	6	4630	ZINC000005618627
0.5	-5.1	-4.8	0.18	1.553	0.050	2.589	0.387	6	4631	ZINC000001850401
0.5	-7.0	-6.7	0.19	1.763	0.656	5.624	1.835	6	4632	ZINC000014439788
0.5	-6.0	-5.6	0.19	2.076	0.260	3.496	0.588	6	4633	ZINC000001850362
0.5	-5.5	-5.3	0.11	2.385	0.433	3.898	0.777	6	4634	ZINC000001850423
0.5	-6.6	-6.5	0.05	2.447	0.248	4.543	0.724	6	4635	ZINC000002040979
0.5	-6.7	-6.4	0.18	1.220	0.176	3.971	1.067	6	4636	ZINC000085972888
0.5	-8.1	-7.9	0.14	1.574	0.383	2.619	0.676	6	4637	ZINC000100781108
0.5	-7.1	-6.8	0.19	1.546	0.453	2.586	1.126	6	4638	ZINC000000020255
0.5	-7.9	-7.5	0.30	2.034	0.263	2.535	0.605	6	4639	ZINC000044608165
0.5	-5.9	-5.3	0.29	2.155	0.442	3.699	0.377	6	4640	ZINC000001693597
0.5	-8.2	-7.8	0.20	2.124	0.338	4.075	1.330	6	4641	ZINC000013462906
0.5	-9.9	-9.0	0.56	1.689	0.142	5.227	0.964	6	4642	ZINC000257538893
0.5	-7.6	-7.4	0.20	2.467	0.226	4.537	1.147	6	4643	ZINC000095617726

0.5	-7.8	-7.4	0.24	2.078	0.286	3.157	0.836	6	4644	ZINC000001673578
0.5	-9.1	-8.6	0.30	2.245	0.723	3.851	1.423	6	4645	ZINC000014805954
0.5	-7.2	-6.7	0.23	1.736	0.323	3.323	0.809	6	4646	ZINC000034866779
0.5	-5.7	-5.5	0.14	1.875	0.772	3.598	0.840	6	4647	ZINC000001668235
0.5	-6.9	-6.8	0.13	1.903	0.432	2.474	0.560	6	4648	ZINC000001575283
0.5	-9.0	-8.6	0.25	1.959	0.766	3.621	1.575	6	4649	ZINC000014612910
0.5	-6.9	-6.7	0.12	2.156	0.625	3.484	0.675	6	4650	ZINC000001597139
0.5	-6.7	-6.5	0.25	1.624	0.304	2.536	0.439	6	4651	ZINC000006031292
0.5	-6.7	-6.4	0.15	2.729	0.918	4.332	1.389	6	4652	ZINC000005273653
0.5	-7.3	-7.1	0.19	2.642	0.770	3.754	0.493	6	4653	ZINC000100823357
0.5	-3.8	-3.5	0.19	3.535	1.934	4.573	1.727	6	4654	ZINC000002034885
0.5	-8.5	-8.2	0.20	2.305	0.252	4.078	0.325	6	4655	ZINC000001638006
0.5	-6.7	-6.6	0.08	1.868	0.381	6.285	0.242	6	4656	ZINC000022930737
0.5	-9.3	-8.2	0.55	2.162	0.188	4.365	0.971	6	4657	ZINC000013323232
0.5	-11.0	-10.0	0.46	1.751	0.349	4.818	1.480	6	4658	ZINC000100828887

0.5	-7.0	-6.5	0.26	2.654	0.594	4.385	0.292	6	4659	ZINC000001850603
0.5	-6.9	-6.6	0.22	2.238	0.285	4.524	1.611	6	4660	ZINC000050027620
0.5	-6.4	-6.2	0.17	2.427	1.223	3.926	1.375	6	4661	ZINC000013516321
0.5	-8.1	-7.7	0.35	2.586	0.159	4.895	0.248	6	4662	ZINC000050027238
0.5	-6.6	-6.4	0.11	1.654	0.394	2.579	0.508	6	4663	ZINC000031290836
0.5	-7.2	-7.0	0.11	2.048	0.363	3.731	0.311	6	4664	ZINC000014917023
0.5	-5.3	-5.0	0.27	2.280	0.742	3.231	0.855	6	4665	ZINC000002038642
0.5	-5.3	-5.0	0.23	1.633	0.271	3.010	0.913	6	4666	ZINC000005954694
0.5	-7.7	-7.5	0.17	1.960	0.205	3.782	0.814	6	4667	ZINC000100822077
0.5	-7.1	-6.9	0.09	1.391	0.191	3.210	0.660	6	4668	ZINC000100058345
0.5	-7.1	-6.9	0.17	2.304	0.365	3.361	0.851	6	4669	ZINC000002570884
0.5	-9.0	-8.0	0.66	2.060	0.614	4.169	1.658	6	4670	ZINC000104370409
0.5	-6.7	-6.3	0.29	1.255	0.221	4.372	0.877	6	4671	ZINC000085972900
0.5	-5.6	-5.4	0.13	2.572	1.187	3.124	1.393	6	4672	ZINC000001648290
0.5	-8.2	-7.9	0.14	1.805	0.119	5.776	0.589	6	4673	ZINC000015112948

0.5	-7.2	-6.8	0.20	2.472	0.332	4.736	1.560	6	4674	ZINC000005112939
0.5	-6.2	-5.9	0.21	2.947	0.846	4.800	0.373	6	4675	ZINC000002037147
0.5	-10.8	-8.8	0.99	1.549	0.403	4.547	1.846	6	4676	ZINC000004655107
0.5	-7.1	-7.0	0.09	2.102	0.179	4.029	1.270	6	4677	ZINC000014856837
0.5	-7.9	-7.5	0.39	1.993	0.223	3.178	1.692	6	4678	ZINC000044608166
0.5	-5.5	-5.3	0.13	1.443	0.196	3.509	0.203	6	4679	ZINC000002040187
0.5	-7.6	-7.5	0.11	2.170	0.215	5.481	1.017	6	4680	ZINC000004899811
0.5	-7.0	-6.8	0.13	1.603	0.741	2.751	0.302	6	4681	ZINC000015119517
0.5	-6.9	-6.8	0.11	1.823	0.408	2.965	0.563	6	4682	ZINC000100778136
0.5	-7.3	-7.0	0.20	2.058	0.294	2.916	0.842	6	4683	ZINC000002561059
0.5	-9.9	-8.9	0.69	1.716	0.174	4.418	1.429	6	4684	ZINC000100779852
0.5	-5.9	-5.7	0.14	2.601	0.920	3.382	1.218	6	4685	ZINC000032163579
0.5	-5.9	-5.7	0.15	3.230	0.516	3.910	0.468	6	4686	ZINC000033820275
0.5	-7.2	-6.9	0.21	1.971	0.579	3.347	0.455	6	4687	ZINC000095620679
0.5	-10.0	-8.9	0.54	2.104	0.494	4.204	1.666	6	4688	ZINC000100775230

0.5	-6.1	-5.7	0.25	1.878	0.852	4.379	0.328	6	4689	ZINC000001849954
0.5	-7.0	-6.8	0.13	2.325	0.735	4.443	2.012	6	4690	ZINC000006020363
0.5	-7.3	-7.1	0.11	2.276	0.390	5.053	0.820	6	4691	ZINC000032839133
0.5	-7.3	-7.0	0.20	1.809	0.182	4.422	0.416	6	4692	ZINC000001850292
0.5	-8.6	-8.0	0.30	1.644	0.147	3.778	0.336	6	4693	ZINC000014441678
0.5	-9.1	-7.8	0.65	2.425	0.262	4.283	1.136	6	4694	ZINC000100051467
0.5	-8.0	-7.6	0.29	2.594	1.031	4.697	2.138	6	4695	ZINC000000049153
0.5	-7.8	-7.6	0.10	3.113	0.860	4.771	1.740	6	4696	ZINC000003872190
0.5	-9.8	-9.2	0.41	1.467	0.525	3.621	1.407	6	4697	ZINC000015160928
0.5	-7.3	-7.0	0.15	2.568	0.650	4.389	1.723	6	4698	ZINC000034361789
0.5	-5.7	-5.4	0.20	2.032	0.619	3.271	0.690	6	4699	ZINC000039374928
0.5	-5.3	-5.2	0.12	1.292	0.373	2.778	0.235	6	4700	ZINC000002563377
0.5	-5.4	-5.2	0.15	3.339	1.214	4.088	1.271	6	4701	ZINC000001853865
0.5	-6.2	-6.0	0.11	2.902	0.106	5.166	0.488	6	4702	ZINC000001601798
0.5	-7.1	-6.8	0.26	2.439	0.499	3.411	0.836	6	4703	ZINC000040472505

0.5	-9.1	-7.8	1.02	1.585	0.296	4.072	1.847	3	4704	ZINC000014587136
0.5	-7.3	-7.2	0.09	2.263	0.394	4.438	0.383	6	4705	ZINC000033979285
0.5	-8.1	-7.9	0.15	2.695	0.686	5.189	1.547	6	4706	ZINC000040165423
0.5	-6.9	-6.9	0.05	1.967	0.199	2.640	0.393	6	4707	ZINC000069486031
0.5	-8.7	-8.4	0.23	1.665	0.249	4.047	1.750	6	4708	ZINC000014885095
0.5	-6.2	-5.9	0.15	2.501	0.741	3.853	1.019	6	4709	ZINC000095620560
0.5	-6.3	-6.0	0.14	1.658	0.300	3.500	0.570	6	4710	ZINC000033610756
0.5	-5.5	-5.3	0.13	1.302	0.563	3.004	0.620	6	4711	ZINC000000895845
0.5	-6.4	-6.2	0.07	2.301	0.394	4.462	0.418	6	4712	ZINC000002575042
0.5	-9.0	-8.5	0.30	2.258	0.247	5.910	0.636	6	4713	ZINC000014612909
0.5	-7.6	-7.5	0.13	2.255	0.191	4.473	1.566	6	4714	ZINC000012153244
0.5	-8.0	-7.4	0.36	1.478	0.509	5.379	1.655	6	4715	ZINC000004096694
0.5	-8.5	-7.9	0.31	2.221	0.672	3.669	1.729	6	4716	ZINC000013380364
0.5	-5.2	-4.8	0.20	1.731	0.299	2.826	0.546	6	4717	ZINC000013459858
0.5	-6.5	-6.4	0.13	2.045	0.415	3.644	0.437	6	4718	ZINC000014766721

0.5	-5.5	-5.2	0.15	2.883	1.024	3.869	1.060	6	4719	ZINC000006021147
0.5	-8.6	-8.2	0.23	1.600	0.191	5.231	0.809	6	4720	ZINC000014587270
0.5	-7.8	-7.6	0.13	2.292	0.146	4.309	1.091	6	4721	ZINC000002525884
0.5	-6.8	-6.6	0.15	2.274	0.811	3.754	0.874	6	4722	ZINC000031290833
0.5	-8.0	-7.6	0.18	2.399	0.733	3.835	1.559	6	4723	ZINC000004534012
0.5	-7.3	-7.0	0.20	2.151	0.840	3.780	1.414	6	4724	ZINC000013341231
0.5	-7.3	-7.0	0.20	2.129	0.655	3.612	1.100	6	4725	ZINC000005765464
0.5	-6.7	-6.5	0.22	1.690	0.278	3.223	1.141	6	4726	ZINC000031290677
0.5	-8.7	-8.0	0.31	2.383	0.435	4.565	1.013	6	4727	ZINC000001865985
0.5	-6.4	-6.2	0.11	2.482	0.157	4.042	0.255	6	4728	ZINC000001529541
0.5	-8.2	-7.9	0.37	2.659	0.087	4.589	0.212	6	4729	ZINC000002384780
0.5	-7.2	-6.8	0.29	1.833	0.222	3.343	1.655	6	4730	ZINC000014507211
0.5	-5.8	-5.6	0.12	2.909	0.605	4.177	0.676	6	4731	ZINC000002169346
0.5	-5.5	-5.2	0.14	2.299	0.344	3.577	0.576	6	4732	ZINC000001583724
0.5	-9.8	-9.0	0.45	2.488	0.275	4.990	1.071	6	4733	ZINC000001576167

0.5	-8.1	-7.8	0.20	2.332	0.474	5.126	1.754	6	4734	ZINC000004095997
0.5	-5.5	-5.4	0.07	4.853	1.400	6.285	1.284	6	4735	ZINC000003869232
0.5	-8.4	-7.7	0.47	2.557	0.761	3.790	1.649	6	4736	ZINC000006485142
0.5	-9.6	-8.9	0.41	1.755	0.496	3.154	0.527	6	4737	ZINC000257525224
0.5	-6.5	-6.4	0.11	2.167	0.455	4.035	0.397	6	4738	ZINC000005512462
0.5	-5.9	-5.5	0.26	3.055	1.083	5.113	0.980	6	4739	ZINC000032180821
0.5	-7.5	-6.9	0.28	3.615	0.384	4.712	0.478	6	4740	ZINC000000901599
0.5	-6.6	-6.4	0.14	2.006	0.654	3.031	0.409	6	4741	ZINC000001631205
0.5	-10.4	-9.8	0.28	1.944	0.262	3.632	1.538	6	4742	ZINC000257498242
0.5	-9.8	-8.8	0.73	1.634	0.198	4.662	1.401	6	4743	ZINC000002029389
0.5	-6.5	-6.3	0.09	1.171	0.190	3.020	0.501	6	4744	ZINC000039217112
0.5	-7.2	-6.9	0.19	2.142	0.819	3.277	1.288	6	4745	ZINC000001850902
0.5	-8.3	-8.0	0.27	2.608	0.419	4.829	1.342	6	4746	ZINC000257623156
0.5	-9.8	-8.9	0.93	1.209	0.144	4.976	1.620	4	4747	ZINC000003861661
0.5	-7.7	-7.6	0.07	1.727	0.326	3.903	0.267	6	4748	ZINC000013381727

0.5	-9.0	-8.3	0.35	1.353	0.276	2.823	0.520	6	4749	ZINC000012341951
0.5	-7.2	-7.0	0.13	1.345	0.336	2.904	0.783	6	4750	ZINC000001599676
0.5	-7.2	-7.0	0.11	1.350	0.341	2.961	0.823	6	4751	ZINC000100781740
0.5	-6.5	-6.2	0.16	2.702	0.746	4.401	1.224	6	4752	ZINC000005524327
0.5	-10.2	-9.6	0.37	1.380	0.278	4.174	1.026	6	4753	ZINC000100779664
0.5	-8.7	-8.3	0.21	1.615	0.313	3.340	1.159	6	4754	ZINC000008632336
0.5	-7.4	-7.4	0.00	1.980	0.318	4.421	0.998	6	4755	ZINC000015120478
0.5	-7.1	-6.8	0.18	2.370	0.690	4.205	1.280	6	4756	ZINC000004492883
0.5	-8.3	-8.0	0.21	1.768	0.214	4.299	0.813	6	4757	ZINC000014854744
0.5	-6.5	-6.2	0.16	3.246	0.619	4.895	0.800	6	4758	ZINC000034746388
0.5	-7.9	-7.5	0.31	2.166	0.471	2.575	0.655	6	4759	ZINC000004556977
0.5	-7.3	-6.6	0.34	2.134	0.300	4.443	0.425	6	4760	ZINC000012496871
0.5	-7.2	-7.0	0.12	1.430	0.444	2.640	0.563	6	4761	ZINC000257507465
0.5	-7.5	-7.2	0.15	1.855	0.187	2.726	0.324	6	4762	ZINC000015213737
0.5	-7.7	-7.4	0.18	1.934	0.631	2.604	0.744	6	4763	ZINC000000057733

0.5	-4.6	-4.5	0.11	2.187	0.390	3.004	0.376	6	4764	ZINC000001532883
0.5	-7.2	-6.6	0.30	3.247	0.731	5.449	1.532	6	4765	ZINC000038673533
0.5	-5.7	-5.5	0.17	2.214	0.316	4.044	0.894	6	4766	ZINC000001850952
0.5	-7.3	-6.8	0.28	2.100	0.764	3.667	0.902	6	4767	ZINC000001706892
0.5	-7.2	-6.9	0.25	1.764	0.359	2.697	0.814	6	4768	ZINC000001841755
0.5	-7.6	-7.2	0.27	1.548	0.266	2.972	0.617	6	4769	ZINC000002561199
0.5	-7.2	-6.9	0.23	1.934	0.584	3.310	0.484	6	4770	ZINC000095620678
0.5	-8.4	-7.6	0.44	1.458	0.400	5.092	2.160	6	4771	ZINC000257500234
0.5	-8.8	-8.7	0.10	0.968	0.397	4.144	1.981	6	4772	ZINC000008437752
0.5	-7.2	-6.7	0.27	1.401	0.151	4.516	1.343	6	4773	ZINC000013406033
0.5	-6.6	-6.4	0.11	2.700	0.442	3.944	0.893	6	4774	ZINC000028630750
0.5	-10.3	-8.8	1.11	1.820	0.082	5.526	0.391	4	4775	ZINC000013360030
0.5	-9.3	-8.4	0.64	1.684	0.375	4.534	1.180	6	4776	ZINC000015206004
0.5	-10.3	-9.8	0.38	1.082	0.178	2.848	0.788	6	4777	ZINC000100776091
0.5	-7.3	-7.0	0.17	2.252	0.208	5.251	0.882	6	4778	ZINC000004544321

0.5	-8.3	-7.8	0.24	1.862	0.544	3.674	1.405	6	4779	ZINC000004578919
0.5	-5.4	-5.2	0.18	2.438	0.967	3.441	1.063	6	4780	ZINC000001529768
0.5	-7.1	-6.6	0.28	2.248	0.470	3.613	0.343	6	4781	ZINC000001673347
0.5	-7.3	-7.1	0.13	1.812	0.639	3.142	1.029	6	4782	ZINC000014919958
0.5	-7.9	-7.9	0.08	2.277	0.362	3.868	0.873	6	4783	ZINC000013387718
0.5	-7.8	-7.3	0.25	1.956	0.705	3.491	0.732	6	4784	ZINC000001715917
0.5	-6.2	-5.9	0.17	2.862	0.213	3.753	0.190	6	4785	ZINC000050027417
0.5	-5.4	-5.3	0.06	2.563	0.745	3.533	0.761	6	4786	ZINC000001850304
0.5	-7.4	-7.2	0.12	1.277	0.358	2.814	0.746	6	4787	ZINC000004292977
0.5	-6.7	-6.6	0.08	2.630	0.420	5.251	0.493	6	4788	ZINC000008076544
0.5	-8.6	-7.5	0.82	1.599	0.132	5.526	0.565	6	4789	ZINC000014491589
0.5	-9.9	-9.1	0.79	2.527	0.264	3.831	1.432	6	4790	ZINC000014651993
0.5	-6.6	-6.3	0.18	2.211	0.985	3.486	0.988	6	4791	ZINC000000120316
0.5	-6.9	-6.5	0.23	2.445	0.240	4.342	0.439	6	4792	ZINC000002522658
0.5	-6.9	-6.9	0.08	2.552	0.641	3.542	0.914	6	4793	ZINC000000388671

0.5	-9.2	-8.4	0.54	1.339	0.124	4.436	1.352	6	4794	ZINC000257492753
0.5	-5.9	-5.6	0.18	2.245	0.583	3.979	0.986	6	4795	ZINC000100048384
0.5	-9.4	-8.4	0.55	1.983	0.546	3.075	0.804	6	4796	ZINC000004096654
0.5	-6.4	-6.1	0.17	2.130	0.425	3.075	1.064	6	4797	ZINC000004533813
0.5	-7.2	-6.7	0.27	1.773	0.492	3.620	1.122	6	4798	ZINC000005820126
0.5	-7.9	-7.6	0.14	1.752	0.147	3.448	0.854	6	4799	ZINC000015169309
0.5	-5.9	-5.7	0.12	3.059	0.574	4.405	0.866	6	4800	ZINC000000120539
0.5	-7.8	-7.6	0.18	1.742	0.260	3.531	0.720	6	4801	ZINC000012153076
0.5	-9.0	-8.4	0.45	2.148	0.642	4.759	0.546	6	4802	ZINC000005933760
0.5	-6.8	-6.6	0.11	2.774	0.542	3.743	0.843	6	4803	ZINC000008951862
0.5	-7.2	-6.8	0.29	2.544	0.757	4.016	1.180	6	4804	ZINC000006031949
0.5	-5.4	-5.1	0.20	2.037	0.407	3.409	0.517	6	4805	ZINC000001720584
0.5	-7.2	-6.9	0.16	2.077	0.303	5.059	1.990	6	4806	ZINC000005955041
0.5	-4.3	-4.0	0.25	2.197	0.547	2.978	0.805	6	4807	ZINC000000407006
0.5	-6.4	-6.1	0.23	2.002	1.232	3.257	1.867	6	4808	ZINC000015120357

0.5	-6.7	-6.5	0.11	2.566	0.383	3.686	0.501	6	4809	ZINC000001693667
0.5	-6.4	-5.8	0.30	2.415	0.553	3.784	0.736	6	4810	ZINC000013437588
0.5	-6.5	-6.0	0.31	2.187	1.013	3.942	1.012	6	4811	ZINC000032152855
0.5	-7.4	-6.7	0.31	1.746	0.471	4.103	1.301	6	4812	ZINC000005999104
0.5	-7.6	-7.2	0.24	2.700	0.471	4.241	1.168	6	4813	ZINC000013460042
0.5	-6.3	-6.1	0.16	2.215	0.236	3.807	0.877	6	4814	ZINC000002018446
0.5	-6.2	-5.9	0.18	1.974	0.529	3.535	0.835	6	4815	ZINC000038220508
0.5	-7.9	-7.7	0.11	2.198	0.314	4.221	0.330	6	4816	ZINC000013397394
0.5	-10.5	-10.1	0.27	1.645	0.332	5.612	1.222	6	4817	ZINC000257469500
0.5	-5.2	-4.9	0.16	3.080	1.324	3.851	0.994	6	4818	ZINC000257481074
0.5	-8.9	-8.6	0.22	2.105	0.294	5.839	0.990	6	4819	ZINC000257441915
0.5	-7.8	-7.4	0.21	2.257	0.226	4.286	0.983	6	4820	ZINC000015261459
0.5	-6.8	-6.6	0.14	2.400	1.335	3.950	1.943	6	4821	ZINC000000001239
0.5	-6.1	-5.7	0.21	2.961	0.859	4.683	0.330	6	4822	ZINC000002037148
0.5	-9.4	-8.8	0.30	1.907	0.578	3.715	1.407	6	4823	ZINC000000086978

0.5	-6.1	-5.9	0.15	2.368	1.082	3.753	1.432	6	4824	ZINC000038220509
0.5	-9.0	-8.8	0.10	2.525	0.897	3.768	0.706	6	4825	ZINC000100072583
0.5	-7.9	-7.4	0.29	1.817	0.178	3.643	0.956	6	4826	ZINC000257803964
0.5	-7.1	-7.0	0.12	2.277	0.448	2.950	0.460	6	4827	ZINC000008216889
0.5	-6.9	-6.8	0.09	2.320	0.301	3.967	1.246	6	4828	ZINC000000153644
0.5	-8.5	-8.2	0.17	1.562	0.543	3.378	1.264	6	4829	ZINC000257532966
0.5	-7.3	-7.2	0.04	1.242	0.453	2.728	0.471	6	4830	ZINC000100779748
0.5	-7.7	-7.4	0.19	2.460	0.386	5.244	0.657	6	4831	ZINC000003861769
0.5	-5.5	-5.2	0.15	2.683	1.227	3.712	1.312	6	4832	ZINC000014451127
0.5	-5.4	-5.2	0.17	2.692	0.720	3.980	0.699	6	4833	ZINC000035021146
0.5	-8.3	-8.1	0.18	2.894	0.339	5.554	0.695	6	4834	ZINC000014420733
0.5	-7.6	-7.4	0.17	1.853	0.296	3.981	1.280	6	4835	ZINC000004097164
0.5	-4.9	-4.8	0.14	3.218	1.318	4.138	1.412	6	4836	ZINC000026672172
0.5	-9.3	-8.7	0.33	1.812	0.529	4.118	0.801	6	4837	ZINC000257382662
0.5	-7.1	-6.9	0.14	2.353	0.580	4.423	1.681	6	4838	ZINC000001529671

0.5	-7.6	-7.0	0.32	2.140	0.274	4.805	1.804	6	4839	ZINC000095617756
0.5	-5.5	-5.4	0.11	3.230	0.933	4.350	1.098	6	4840	ZINC000001532838
0.5	-8.2	-7.7	0.24	2.065	0.422	3.551	0.954	6	4841	ZINC000014823206
0.5	-7.3	-7.0	0.20	1.937	0.409	3.185	0.998	6	4842	ZINC000100779451
0.5	-6.2	-6.0	0.15	3.213	0.467	3.875	0.704	6	4843	ZINC000100029290
0.5	-7.0	-6.7	0.17	2.423	0.690	2.964	0.869	6	4844	ZINC000000895911
0.5	-6.0	-5.8	0.11	2.025	0.388	2.956	0.602	6	4845	ZINC000002019416
0.5	-7.5	-7.1	0.36	2.609	0.172	7.200	0.177	6	4846	ZINC000095618362
0.5	-8.9	-8.2	0.35	2.248	0.207	3.939	0.642	6	4847	ZINC000015153295
0.5	-8.9	-8.5	0.30	2.047	0.293	5.654	1.724	6	4848	ZINC000257767901
0.5	-8.7	-8.0	0.43	2.009	0.376	4.348	1.182	6	4849	ZINC000014682706
0.5	-7.6	-7.2	0.25	2.008	0.487	3.440	0.800	6	4850	ZINC000001825278
0.5	-8.6	-8.2	0.19	2.332	0.529	4.354	0.327	6	4851	ZINC000015264646
0.5	-9.5	-9.1	0.23	1.857	0.380	3.900	1.534	6	4852	ZINC000003895655
0.5	-8.5	-7.8	0.42	1.947	0.496	3.401	1.310	6	4853	ZINC000013540498

0.5	-10.4	-9.8	0.31	2.141	0.396	3.744	1.475	6	4854	ZINC000257498243
0.5	-9.9	-9.0	0.56	1.682	0.143	5.225	0.963	6	4855	ZINC000100779857
0.5	-5.2	-5.1	0.11	1.298	0.265	3.033	0.732	6	4856	ZINC000001995224
0.5	-8.0	-7.8	0.15	1.990	0.877	2.539	0.777	6	4857	ZINC000100225688
0.5	-7.7	-7.4	0.30	1.400	0.647	2.892	1.580	6	4858	ZINC000256007008
0.5	-7.9	-7.5	0.23	1.692	0.312	5.439	0.712	6	4859	ZINC000040165306
0.5	-10.2	-9.5	0.44	1.627	0.330	4.281	1.062	6	4860	ZINC000100779669
0.5	-6.9	-6.5	0.26	2.167	0.448	3.787	1.604	6	4861	ZINC000004533963
0.5	-6.6	-6.4	0.17	2.070	0.310	2.847	0.772	6	4862	ZINC000013282550
0.5	-6.6	-6.2	0.23	2.049	0.584	2.926	0.800	6	4863	ZINC000000152578
0.5	-9.2	-8.4	0.39	1.846	0.430	3.828	1.206	6	4864	ZINC000014658334
0.5	-8.5	-7.8	0.34	1.895	0.293	3.632	0.957	6	4865	ZINC000038588218
0.5	-9.5	-9.1	0.38	1.476	0.243	4.575	1.427	6	4866	ZINC000004098644
0.5	-6.4	-6.3	0.07	2.462	0.475	4.238	0.311	6	4867	ZINC000002522619
0.5	-5.5	-5.4	0.21	2.029	0.765	2.981	0.931	6	4868	ZINC000005977893

0.5	-6.4	-6.1	0.16	2.635	0.756	3.805	0.775	6	4869	ZINC000005819412
0.5	-6.8	-6.5	0.11	2.199	0.246	4.015	1.216	6	4870	ZINC000005663069
0.5	-7.3	-7.1	0.15	1.839	0.604	3.140	1.036	6	4871	ZINC000100779447
0.5	-7.2	-7.0	0.13	1.528	0.217	3.006	0.866	6	4872	ZINC000015169532
0.5	-7.5	-7.2	0.17	2.196	0.264	4.638	1.556	6	4873	ZINC000050027288
0.5	-8.6	-8.2	0.32	2.633	0.413	5.182	0.453	6	4874	ZINC000100772270
0.5	-6.4	-6.1	0.18	2.610	0.492	4.527	0.929	6	4875	ZINC000040439673
0.5	-7.8	-7.5	0.21	1.632	0.401	3.646	1.876	6	4876	ZINC000100822355
0.5	-8.6	-8.2	0.30	1.265	0.207	3.265	1.406	6	4877	ZINC000038954072
0.5	-11.0	-10.0	0.49	1.727	0.349	4.155	1.799	6	4878	ZINC000100828885
0.5	-7.3	-7.0	0.21	2.202	0.647	3.824	1.863	6	4879	ZINC000000394813
0.5	-6.0	-5.9	0.11	2.652	0.485	4.685	0.185	6	4880	ZINC000006069856
0.5	-9.4	-8.3	0.73	2.007	0.223	4.338	1.206	6	4881	ZINC000015162852
0.5	-8.6	-7.8	0.50	2.671	0.484	4.352	1.330	6	4882	ZINC000050027351
0.5	-6.2	-5.9	0.21	2.152	1.086	2.858	1.440	6	4883	ZINC000095619612

0.5	-6.1	-5.6	0.24	2.322	0.766	2.938	0.585	6	4884	ZINC000000896073
0.5	-5.5	-5.0	0.31	2.851	1.365	3.234	1.585	6	4885	ZINC000014680216
0.5	-7.1	-6.9	0.09	2.343	0.400	3.661	1.438	6	4886	ZINC000069485808
0.5	-7.7	-7.5	0.17	3.367	0.647	5.868	1.821	6	4887	ZINC000004492895
0.5	-7.4	-7.2	0.16	1.953	0.409	4.527	1.532	6	4888	ZINC000257472116
0.5	-7.1	-6.6	0.27	2.870	0.220	4.880	0.295	6	4889	ZINC000069485831
0.5	-7.3	-7.1	0.19	2.506	0.653	3.695	0.401	6	4890	ZINC000100823359
0.5	-9.0	-8.8	0.15	1.548	0.323	3.929	0.939	6	4891	ZINC000230084409
0.5	-6.9	-6.5	0.20	2.180	0.926	3.458	1.723	6	4892	ZINC000032841041
0.5	-8.2	-8.0	0.10	1.803	0.236	4.429	1.852	6	4893	ZINC000257563233
0.5	-7.5	-7.2	0.21	1.898	0.308	3.825	1.029	6	4894	ZINC000015169314
0.5	-7.0	-6.8	0.18	2.418	0.649	3.652	1.455	6	4895	ZINC000038417740
0.5	-7.2	-6.9	0.16	1.685	0.340	2.273	0.273	6	4896	ZINC000014684305
0.5	-10.3	-8.7	1.09	2.186	0.610	5.271	1.585	6	4897	ZINC000005854460
0.5	-7.5	-7.1	0.27	3.151	1.028	5.789	1.575	6	4898	ZINC000252444100

0.5	-7.7	-7.5	0.14	2.722	0.190	5.288	0.697	6	4899	ZINC000033976414
0.5	-9.0	-8.4	0.31	2.186	0.274	4.237	1.372	6	4900	ZINC000014774777
0.5	-6.3	-5.9	0.23	1.514	0.587	2.776	0.969	6	4901	ZINC000001693629
0.5	-5.6	-5.4	0.17	3.295	0.167	4.039	0.271	6	4902	ZINC000001765597
0.5	-7.9	-7.7	0.11	1.702	0.364	4.535	1.375	6	4903	ZINC000085695226
0.5	-7.1	-6.7	0.30	2.720	0.977	4.531	1.744	6	4904	ZINC000000000166
0.5	-9.4	-8.8	0.29	2.002	0.613	4.982	0.973	6	4905	ZINC000014774910
0.5	-6.3	-6.2	0.07	2.746	0.366	4.567	0.348	6	4906	ZINC000034383089
0.5	-10.1	-9.1	0.54	1.849	0.460	5.169	1.648	6	4907	ZINC000006093351
0.5	-7.0	-6.8	0.12	1.447	0.717	2.612	0.433	6	4908	ZINC000257411066
0.5	-6.6	-6.4	0.13	1.531	0.201	2.606	0.367	6	4909	ZINC000005510517
0.5	-7.0	-6.7	0.23	1.455	0.823	2.329	1.303	6	4910	ZINC000001850349
0.5	-7.5	-7.2	0.17	2.116	0.867	3.221	0.769	6	4911	ZINC000100471170
0.5	-6.1	-5.9	0.17	2.104	0.509	3.452	0.440	6	4912	ZINC000002020095
0.5	-7.3	-7.1	0.21	2.626	0.794	3.563	0.556	6	4913	ZINC000095619237

0.5	-7.5	-7.1	0.34	1.539	0.737	3.326	0.986	6	4914	ZINC000261496101
0.5	-9.8	-9.2	0.37	1.714	0.690	3.716	0.578	6	4915	ZINC000015160927
0.5	-8.2	-7.5	0.34	2.067	0.520	3.685	0.591	6	4916	ZINC000015169243
0.5	-6.5	-6.2	0.23	2.096	0.423	3.563	0.689	6	4917	ZINC000257497039
0.5	-6.5	-6.4	0.04	2.090	0.430	4.814	1.412	6	4918	ZINC000085649275
0.5	-8.4	-8.0	0.21	2.227	0.438	4.131	1.403	6	4919	ZINC000015112781
0.5	-9.2	-8.3	0.49	2.130	0.686	3.949	1.553	6	4920	ZINC000034046038
0.5	-8.4	-7.8	0.37	1.634	0.225	3.620	0.708	6	4921	ZINC000100781168
0.5	-8.6	-8.1	0.43	1.544	0.391	3.447	1.057	6	4922	ZINC000006037475
0.5	-6.6	-6.4	0.11	2.691	0.442	3.895	0.878	6	4923	ZINC000004095951
0.5	-5.5	-5.3	0.23	1.772	0.507	2.884	0.588	6	4924	ZINC000014591971
0.5	-8.4	-8.0	0.24	1.785	0.147	4.218	1.730	6	4925	ZINC000005167610
0.5	-6.2	-6.0	0.11	2.388	0.408	3.753	0.970	6	4926	ZINC000005159516
0.5	-5.2	-5.0	0.12	2.541	1.430	3.327	1.157	6	4927	ZINC000100829305
0.5	-6.6	-6.4	0.14	2.609	0.940	4.897	0.159	6	4928	ZINC000012493851

0.5	-8.0	-7.6	0.31	1.924	0.542	5.584	1.581	6	4929	ZINC000261499262
0.5	-6.0	-5.8	0.12	1.818	0.395	4.995	1.127	6	4930	ZINC000100051224
0.5	-9.6	-7.9	0.78	1.712	0.195	6.247	1.459	6	4931	ZINC000001531481
0.5	-7.8	-7.4	0.21	2.722	0.450	4.067	1.217	6	4932	ZINC000012495781
0.5	-8.7	-8.3	0.25	2.286	0.486	4.216	1.247	6	4933	ZINC000000000867
0.5	-8.1	-7.8	0.19	1.840	0.221	4.548	1.230	6	4934	ZINC000257426778
0.5	-6.7	-6.3	0.19	2.979	0.900	3.987	0.915	6	4935	ZINC000000403022
0.5	-5.9	-5.6	0.16	3.039	1.579	3.773	1.716	6	4936	ZINC000004097510
0.5	-6.1	-5.9	0.15	2.461	0.688	3.660	0.669	6	4937	ZINC000004095899
0.5	-7.5	-6.9	0.30	1.643	0.284	3.361	0.184	6	4938	ZINC000038611099
0.5	-9.5	-8.9	0.30	2.402	0.442	4.551	1.320	6	4939	ZINC000000265504
0.5	-6.9	-6.8	0.09	2.296	0.754	3.247	1.145	6	4940	ZINC000001633996
0.5	-8.5	-7.8	0.41	1.822	0.567	4.371	1.013	6	4941	ZINC000003882638
0.5	-9.9	-8.5	0.83	2.368	0.385	5.396	1.381	4	4942	ZINC000013449409
0.5	-5.6	-5.4	0.13	1.862	0.228	2.729	0.622	6	4943	ZINC000032228393

0.5	-9.8	-9.3	0.32	1.453	0.546	3.498	1.428	6	4944	ZINC000257436630
0.5	-7.5	-7.0	0.27	2.769	0.446	5.302	0.418	6	4945	ZINC000005273582
0.5	-9.4	-8.7	0.52	1.281	0.656	4.400	1.105	6	4946	ZINC000003860369
0.5	-6.9	-6.5	0.26	2.640	0.279	4.455	0.779	6	4947	ZINC000050027703
0.5	-7.1	-6.9	0.14	1.840	0.919	3.394	0.490	6	4948	ZINC000002566012
0.5	-5.3	-4.9	0.23	1.973	0.726	3.457	0.925	6	4949	ZINC000002034380
0.5	-7.5	-7.1	0.26	1.890	0.593	3.590	2.008	6	4950	ZINC000008469300
0.5	-8.3	-7.7	0.35	1.889	0.487	3.131	0.820	6	4951	ZINC000013783213
0.5	-6.3	-6.1	0.10	1.656	0.274	4.588	0.363	6	4952	ZINC000085972368
0.5	-8.1	-7.8	0.23	1.645	0.421	3.411	1.185	6	4953	ZINC000257398326
0.5	-4.3	-4.0	0.17	3.489	1.076	4.206	1.095	6	4954	ZINC000100828536
0.5	-6.6	-6.2	0.23	2.480	0.318	2.962	0.427	6	4955	ZINC000000154476
0.5	-7.3	-7.1	0.15	1.770	0.259	4.065	1.517	6	4956	ZINC000014719715
0.5	-5.9	-5.5	0.25	2.469	1.463	4.317	1.041	6	4957	ZINC000004712490
0.5	-5.5	-5.3	0.12	2.655	0.367	3.098	0.677	6	4958	ZINC000002146674

0.5	-5.5	-5.2	0.20	2.047	0.736	3.030	0.640	6	4959	ZINC000033839259
0.5	-7.9	-7.6	0.15	1.687	0.578	4.275	1.454	6	4960	ZINC000004467867
0.5	-10.4	-9.7	0.37	1.979	0.248	4.473	1.841	6	4961	ZINC000015169388
0.5	-8.7	-8.3	0.29	3.139	0.462	4.987	0.715	6	4962	ZINC000006070482
0.5	-7.4	-7.0	0.20	1.734	0.307	3.556	1.315	6	4963	ZINC000256069558
0.5	-9.8	-8.9	0.77	1.523	0.279	4.461	1.483	6	4964	ZINC000100780904
0.5	-8.9	-8.3	0.33	1.482	0.388	3.914	2.100	6	4965	ZINC000257441912
0.5	-6.0	-5.6	0.20	1.790	0.246	3.560	0.346	6	4966	ZINC000032167034
0.5	-6.2	-6.0	0.11	2.326	0.831	3.981	0.575	6	4967	ZINC000001680379
0.5	-6.1	-5.9	0.16	1.883	0.971	4.072	0.618	6	4968	ZINC000002003738
0.5	-7.1	-6.9	0.09	2.616	0.462	5.086	1.488	6	4969	ZINC000032839122
0.5	-6.6	-6.4	0.13	2.450	0.507	3.442	0.869	6	4970	ZINC000013520486
0.5	-5.5	-5.1	0.20	1.914	0.533	2.456	0.606	6	4971	ZINC000032166514
0.5	-6.3	-6.1	0.15	2.662	0.742	4.686	0.750	6	4972	ZINC000004582587
0.5	-6.8	-6.6	0.12	3.624	0.949	5.326	1.386	6	4973	ZINC000002584391

0.5	-7.7	-7.4	0.21	2.071	0.977	3.870	1.553	6	4974	ZINC000001575527
0.5	-5.6	-5.5	0.09	2.310	0.408	3.200	0.579	6	4975	ZINC000001730614
0.5	-7.1	-6.8	0.18	2.042	0.491	3.591	0.694	6	4976	ZINC000015253236
0.5	-8.1	-7.6	0.21	1.711	0.542	4.423	1.623	6	4977	ZINC000014637062
0.5	-9.9	-9.4	0.30	1.685	0.382	5.076	0.555	6	4978	ZINC000095099109
0.5	-8.3	-8.0	0.17	1.708	0.170	5.622	0.565	6	4979	ZINC000012495318
0.5	-8.0	-7.5	0.37	2.670	0.877	5.125	1.925	6	4980	ZINC000040470841
0.5	-5.8	-5.6	0.15	1.991	0.557	3.193	0.912	6	4981	ZINC000001850311
0.5	-8.9	-7.9	0.61	1.687	0.773	4.643	1.888	6	4982	ZINC000085923581
0.5	-9.1	-7.8	0.65	1.681	0.606	4.253	2.050	6	4983	ZINC000003819459
0.5	-5.5	-5.4	0.08	2.482	0.560	3.184	0.628	6	4984	ZINC000005133103
0.5	-9.2	-8.2	0.67	1.987	0.415	4.506	1.665	6	4985	ZINC000006032043
0.5	-7.2	-7.0	0.13	1.295	0.308	2.950	0.784	6	4986	ZINC000100136309
0.5	-8.0	-7.5	0.31	2.143	0.353	4.207	1.468	6	4987	ZINC000141596430
0.5	-7.5	-7.4	0.10	2.548	0.202	4.610	1.066	6	4988	ZINC000050027219

0.5	-8.0	-7.8	0.21	1.786	0.587	4.133	2.073	6	4989	ZINC000033650328
0.5	-6.7	-6.4	0.14	2.358	0.162	3.911	1.126	6	4990	ZINC000012494607
0.5	-9.1	-7.9	1.02	1.622	0.273	4.097	1.827	3	4991	ZINC000014587134
0.5	-7.9	-7.5	0.23	2.056	0.254	3.368	0.592	6	4992	ZINC000001575525
0.5	-5.6	-5.2	0.22	2.203	0.330	2.887	0.641	6	4993	ZINC000000388712
0.5	-9.9	-9.0	0.56	1.685	0.146	5.227	0.964	6	4994	ZINC000100779855
0.5	-5.5	-5.1	0.16	1.853	0.460	2.951	0.916	6	4995	ZINC000004404468
0.5	-6.8	-6.6	0.10	2.434	0.289	3.716	0.757	6	4996	ZINC000006360470
0.5	-8.2	-8.0	0.22	2.848	0.203	5.079	0.714	6	4997	ZINC000017860887
0.5	-8.1	-7.6	0.22	1.662	0.280	3.231	0.542	6	4998	ZINC000015169222
0.5	-7.9	-7.6	0.24	2.065	0.558	4.924	2.106	6	4999	ZINC000004787313
0.5	-8.6	-8.1	0.35	1.597	0.656	3.961	0.423	6	5000	ZINC000100776137
0.5	-8.5	-7.9	0.42	1.664	0.663	5.671	1.647	6	5001	ZINC000002518378
0.5	-7.1	-7.0	0.09	1.634	0.932	3.259	0.831	6	5002	ZINC000001532172
0.5	-6.9	-6.6	0.14	2.580	0.644	3.637	0.853	6	5003	ZINC000002242980

0.5	-8.0	-7.9	0.11	2.115	0.583	4.254	1.350	6	5004	ZINC000027731575
0.5	-7.5	-7.1	0.31	2.723	1.191	4.991	1.964	6	5005	ZINC000003881598
0.5	-5.9	-5.6	0.18	2.846	0.478	4.307	0.970	6	5006	ZINC000002384801
0.5	-8.5	-8.0	0.44	3.026	0.318	4.963	1.033	6	5007	ZINC000008217150
0.5	-7.5	-7.2	0.14	1.657	0.576	3.416	1.262	6	5008	ZINC000004474696
0.5	-7.1	-6.8	0.17	2.101	0.554	4.128	0.503	6	5009	ZINC000015253231
0.5	-8.7	-8.2	0.28	2.077	0.215	6.598	0.296	6	5010	ZINC000257487946
0.5	-9.8	-9.0	0.46	1.572	0.471	4.021	0.807	6	5011	ZINC000257436628
0.5	-8.4	-8.0	0.19	2.476	0.479	3.526	0.961	6	5012	ZINC000000056963
0.5	-7.2	-6.9	0.15	1.695	0.604	4.633	1.146	6	5013	ZINC000014616294
0.5	-9.3	-8.3	0.50	2.264	0.288	4.637	0.996	6	5014	ZINC000001570254
0.5	-8.9	-8.2	0.36	1.761	0.377	4.844	1.405	6	5015	ZINC000100770506
0.5	-7.6	-7.4	0.13	2.115	0.552	3.458	1.315	6	5016	ZINC000001731788
0.5	-7.6	-7.3	0.16	2.498	0.400	4.696	0.519	6	5017	ZINC000012494128
0.5	-8.4	-7.8	0.35	2.015	0.319	3.799	1.305	6	5018	ZINC000261498158

0.5	-7.1	-6.7	0.33	2.115	0.219	4.008	1.083	6	5019	ZINC000002580736
0.5	-9.6	-8.4	0.99	1.870	0.378	4.197	1.611	6	5020	ZINC000085586196
0.5	-5.5	-5.4	0.04	1.175	0.803	2.667	0.491	6	5021	ZINC000078366699
0.5	-7.5	-7.4	0.07	2.225	0.347	4.548	1.430	6	5022	ZINC000015148369
0.5	-5.9	-5.6	0.19	2.517	1.089	3.987	1.450	6	5023	ZINC000002003668
0.5	-7.1	-6.7	0.22	1.528	0.201	3.505	1.154	6	5024	ZINC000257441139
0.5	-7.2	-7.0	0.11	1.330	0.330	2.958	0.791	6	5025	ZINC000015169530
0.5	-5.1	-4.8	0.20	2.321	0.728	3.228	0.916	6	5026	ZINC000001586745
0.5	-8.7	-7.9	0.58	2.122	0.413	5.549	0.570	6	5027	ZINC000005283951
0.5	-9.3	-8.0	0.63	2.901	0.298	6.054	1.229	6	5028	ZINC000013409862
0.5	-6.6	-6.4	0.13	2.650	0.483	3.952	0.856	6	5029	ZINC000004095952
0.5	-6.7	-6.5	0.13	3.064	0.375	4.200	0.325	6	5030	ZINC000238789269
0.5	-6.6	-6.4	0.15	2.432	0.168	3.225	0.138	6	5031	ZINC000002384789
0.5	-9.7	-8.7	0.47	2.413	0.382	4.860	0.413	6	5032	ZINC000100779387
0.5	-10.5	-9.6	0.62	2.040	0.405	4.733	0.303	6	5033	ZINC000014618716

0.5	-5.8	-5.5	0.20	2.550	0.394	3.582	0.370	6	5034	ZINC000001671473
0.5	-7.2	-6.7	0.25	2.125	0.370	4.393	0.496	6	5035	ZINC000034267180
0.5	-9.3	-7.5	1.17	1.570	0.688	4.754	1.171	6	5036	ZINC000013403754
0.5	-9.0	-8.3	0.38	1.516	0.407	3.363	0.533	6	5037	ZINC000001448969
0.5	-6.3	-6.1	0.11	1.992	0.330	3.085	0.404	6	5038	ZINC000014616892
0.5	-10.3	-9.8	0.27	1.043	0.199	2.971	0.921	6	5039	ZINC000100776090
0.5	-5.8	-5.7	0.07	2.070	0.962	3.145	1.160	6	5040	ZINC000014614421
0.5	-9.0	-8.2	0.47	1.912	0.522	3.201	0.761	6	5041	ZINC000100778588
0.5	-7.9	-7.3	0.40	2.023	0.146	5.693	0.453	6	5042	ZINC000012496233
0.5	-7.5	-7.2	0.23	2.666	0.582	4.145	1.080	6	5043	ZINC000006024680
0.5	-10.4	-9.4	0.48	1.616	0.311	5.017	1.168	6	5044	ZINC000100781255
0.5	-8.7	-8.2	0.31	2.359	0.722	4.466	1.599	6	5045	ZINC000013108258
0.5	-9.9	-9.5	0.24	1.545	0.451	3.344	0.926	6	5046	ZINC000231162070
0.5	-6.4	-6.1	0.25	1.592	0.327	5.772	1.736	6	5047	ZINC000095621421
0.5	-6.0	-5.9	0.07	2.246	0.412	4.217	0.688	6	5048	ZINC000095619998

0.5	-7.4	-7.0	0.22	1.933	0.245	4.936	0.837	6	5049	ZINC000012496381
0.5	-7.4	-7.1	0.22	1.684	0.551	3.803	1.369	6	5050	ZINC000000895821
0.5	-9.1	-7.9	0.69	1.564	0.487	4.480	1.460	6	5051	ZINC000015112557
0.5	-6.9	-6.6	0.13	2.834	0.725	4.833	0.405	6	5052	ZINC000000406974
0.5	-7.4	-7.2	0.10	1.529	0.104	5.292	2.330	6	5053	ZINC000255970901
0.5	-5.9	-5.5	0.22	1.494	0.299	2.702	0.378	6	5054	ZINC000034746446
0.5	-7.2	-7.0	0.19	2.244	0.685	3.265	0.455	6	5055	ZINC000095620681
0.5	-5.6	-5.4	0.11	1.873	0.563	3.661	0.729	6	5056	ZINC000014590680
0.5	-6.6	-6.2	0.26	2.373	0.704	2.994	0.744	6	5057	ZINC000000002055
0.5	-7.7	-7.4	0.21	1.979	0.095	3.159	1.251	6	5058	ZINC000014657921
0.5	-8.4	-7.9	0.28	2.155	0.786	4.465	1.013	6	5059	ZINC000000057731
0.5	-7.6	-7.5	0.07	1.499	0.207	4.503	2.254	6	5060	ZINC000014725863
0.5	-9.0	-8.2	0.59	1.985	0.523	4.867	1.515	6	5061	ZINC000013384422
0.5	-8.5	-8.0	0.31	1.954	0.131	3.991	0.821	6	5062	ZINC000015061507
0.5	-5.9	-5.8	0.09	2.169	0.984	3.649	0.298	6	5063	ZINC000004654603

0.5	-5.5	-5.2	0.15	2.751	1.120	4.085	0.963	6	5064	ZINC000006030233
0.5	-5.5	-5.2	0.15	1.730	0.313	3.096	0.403	6	5065	ZINC000002040185
0.5	-6.8	-6.5	0.15	2.210	0.277	3.983	0.425	6	5066	ZINC000050027628
0.5	-5.7	-5.5	0.17	2.253	0.431	2.976	0.524	6	5067	ZINC000000157062
0.5	-7.6	-7.4	0.11	2.275	0.702	3.319	1.432	6	5068	ZINC000003869640
0.5	-6.7	-6.5	0.13	1.761	0.600	3.652	0.645	6	5069	ZINC000086034077
0.5	-7.9	-7.7	0.11	1.473	0.452	3.923	2.080	6	5070	ZINC000028128258
0.5	-8.8	-8.2	0.49	1.676	0.387	5.122	1.186	6	5071	ZINC000003801131
0.5	-7.4	-7.2	0.17	2.185	0.560	3.592	1.727	6	5072	ZINC000095617770
0.5	-8.9	-8.4	0.32	1.615	0.067	3.848	0.766	6	5073	ZINC000014811640
0.5	-9.0	-8.2	0.71	1.574	0.379	2.358	0.557	5	5074	ZINC000014413380
0.5	-7.2	-7.1	0.07	2.496	0.329	4.528	0.796	6	5075	ZINC000034248037
0.5	-6.4	-5.9	0.26	1.262	0.336	2.814	1.361	6	5076	ZINC000014590585
0.5	-6.3	-5.9	0.20	4.476	0.650	6.352	0.373	6	5077	ZINC000003869734
0.5	-6.7	-6.5	0.12	2.118	0.506	3.845	1.370	6	5078	ZINC000005510509

0.5	-7.3	-7.1	0.13	1.820	0.636	3.154	1.017	6	5079	ZINC000104368697
0.5	-7.2	-7.0	0.13	1.345	0.337	2.907	0.778	6	5080	ZINC000257507464
0.5	-7.8	-7.5	0.25	2.547	0.167	4.828	0.813	6	5081	ZINC000033641280
0.5	-8.0	-7.4	0.28	1.937	0.414	4.531	1.453	6	5082	ZINC000012496235
0.5	-9.2	-8.2	0.53	2.439	0.201	4.961	0.670	6	5083	ZINC000015263491
0.5	-5.2	-5.0	0.14	1.780	0.656	2.822	0.492	6	5084	ZINC000005509366
0.5	-5.9	-5.9	0.05	1.554	0.157	4.006	1.574	6	5085	ZINC000100051225
0.5	-7.8	-7.3	0.38	2.162	0.733	3.995	1.295	6	5086	ZINC000013397369
0.5	-5.6	-5.5	0.07	2.634	1.464	4.255	0.865	6	5087	ZINC000012495437
0.5	-6.6	-6.3	0.14	2.961	0.177	4.166	0.557	6	5088	ZINC000003869626
0.5	-8.5	-8.0	0.28	2.140	0.607	4.210	0.583	6	5089	ZINC000013461961
0.5	-7.5	-7.1	0.24	2.050	0.456	4.658	1.434	6	5090	ZINC000030730673
0.5	-5.0	-4.8	0.12	4.298	1.573	5.056	1.799	6	5091	ZINC000005955801
0.5	-6.6	-6.3	0.21	1.786	0.518	2.817	0.502	6	5092	ZINC000001849800
0.5	-7.9	-7.8	0.12	1.922	0.376	3.373	0.638	6	5093	ZINC000085826893

0.5	-5.7	-5.5	0.11	2.411	0.338	3.312	0.468	6	5094	ZINC000002027357
0.5	-6.5	-6.4	0.09	2.119	0.380	4.133	0.945	6	5095	ZINC000015146062
0.5	-6.8	-6.5	0.22	1.735	0.750	4.215	1.106	6	5096	ZINC000014592000
0.5	-7.7	-7.3	0.22	1.952	0.259	4.542	1.298	6	5097	ZINC000012496405
0.5	-9.1	-8.7	0.26	2.142	0.554	4.334	0.485	6	5098	ZINC000252426444
0.5	-7.7	-7.3	0.24	2.695	1.257	4.889	1.862	6	5099	ZINC000004097101
0.5	-7.0	-6.8	0.16	2.349	0.407	3.053	0.609	6	5100	ZINC000069486026
0.5	-9.9	-9.0	0.56	1.693	0.140	5.224	0.967	6	5101	ZINC000257538892
0.5	-5.6	-5.5	0.09	2.331	1.200	4.052	0.216	6	5102	ZINC000005688625
0.5	-8.5	-8.0	0.29	1.938	0.104	6.117	0.796	6	5103	ZINC000014438580
0.5	-5.5	-5.3	0.09	2.616	0.506	3.245	0.571	6	5104	ZINC000005133096
0.5	-10.1	-8.8	0.93	1.677	0.403	4.269	1.778	6	5105	ZINC000257418753
0.5	-6.3	-6.0	0.17	3.064	0.763	5.105	1.483	6	5106	ZINC000050027714
0.5	-5.7	-5.5	0.12	2.048	0.311	3.354	0.288	6	5107	ZINC000021984837
0.5	-8.2	-7.5	0.43	2.463	0.240	4.872	0.353	6	5108	ZINC000034018996

0.5	-6.2	-6.0	0.17	2.816	0.815	4.088	1.268	6	5109	ZINC000021999701
0.5	-6.1	-5.9	0.15	1.912	0.098	4.104	1.361	6	5110	ZINC000013439020
0.5	-8.8	-8.3	0.29	1.554	0.497	4.554	2.088	6	5111	ZINC000015251365
0.5	-5.2	-5.0	0.12	2.328	1.628	3.347	1.155	6	5112	ZINC000257481073
0.5	-6.4	-6.1	0.17	2.929	0.646	5.155	1.344	6	5113	ZINC000027650877
0.5	-5.8	-5.6	0.14	2.711	1.115	3.792	1.208	6	5114	ZINC000001613340
0.5	-5.7	-5.5	0.14	1.529	0.175	2.317	0.447	6	5115	ZINC000014723424
0.5	-9.7	-7.8	1.02	2.126	0.181	6.769	1.512	6	5116	ZINC000257503691
0.5	-7.0	-6.8	0.13	2.441	0.915	3.897	0.794	6	5117	ZINC000257534096
0.5	-7.7	-7.4	0.26	2.187	0.718	3.884	1.286	6	5118	ZINC000002558954
0.5	-6.3	-6.0	0.21	1.820	0.242	3.365	0.537	6	5119	ZINC000080052522
0.5	-8.8	-8.3	0.47	1.074	0.587	5.146	2.286	6	5120	ZINC000015251371
0.5	-8.0	-7.6	0.30	2.310	0.482	2.914	0.666	6	5121	ZINC000100031823
0.5	-8.1	-7.9	0.10	2.825	0.275	5.098	0.616	6	5122	ZINC000095617488
0.5	-7.1	-6.7	0.20	2.858	0.262	4.227	1.210	6	5123	ZINC000095617683

0.5	-10.3	-9.9	0.26	2.088	0.384	4.575	1.472	6	5124	ZINC000013374803
0.5	-6.8	-6.5	0.16	2.061	0.465	3.603	0.720	6	5125	ZINC000014588924
0.5	-6.3	-5.8	0.36	1.769	1.017	3.873	1.156	6	5126	ZINC000100627414
0.5	-5.5	-5.4	0.07	2.049	0.473	3.239	0.695	6	5127	ZINC000095618246
0.5	-6.8	-6.7	0.21	1.950	0.464	3.736	1.215	6	5128	ZINC000001613603
0.5	-6.3	-6.0	0.18	1.999	0.989	3.663	1.751	6	5129	ZINC000012153149
0.5	-7.5	-7.0	0.25	2.811	0.322	5.298	0.403	6	5130	ZINC000004899435
0.5	-4.9	-4.8	0.12	2.418	1.045	2.970	0.894	6	5131	ZINC000002004972
0.5	-6.4	-6.1	0.14	1.488	0.233	4.010	0.554	6	5132	ZINC000079566582
0.5	-6.2	-6.0	0.10	3.232	0.724	4.837	0.336	6	5133	ZINC000032176972
0.5	-7.0	-6.7	0.15	1.878	0.578	4.154	1.337	6	5134	ZINC000004533737
0.5	-7.4	-7.0	0.43	1.723	1.130	2.780	1.608	6	5135	ZINC000001600828
0.5	-8.4	-8.0	0.31	2.441	0.153	5.037	0.666	6	5136	ZINC000001605477
0.5	-5.4	-5.1	0.14	2.512	1.110	3.674	0.753	6	5137	ZINC000002019417
0.5	-8.6	-8.3	0.19	1.811	0.536	4.160	1.190	6	5138	ZINC000015120459

0.5	-6.0	-5.5	0.24	2.400	0.375	4.393	0.477	6	5139	ZINC000003788202
0.5	-8.1	-7.8	0.26	2.422	0.409	4.445	0.878	6	5140	ZINC000257417379
0.5	-10.3	-9.7	0.45	1.070	0.149	3.162	0.600	6	5141	ZINC000257473767
0.5	-8.1	-7.9	0.20	1.415	0.129	3.447	0.487	6	5142	ZINC000231183737
0.5	-8.5	-7.9	0.28	2.637	0.648	4.754	1.559	6	5143	ZINC000013585233
0.5	-5.6	-5.5	0.09	1.882	0.365	3.548	1.044	6	5144	ZINC000005842751
0.5	-5.5	-5.4	0.08	2.403	0.632	3.114	0.673	6	5145	ZINC000100029286
0.5	-8.5	-8.1	0.22	1.808	0.104	4.347	1.402	6	5146	ZINC000027641268
0.5	-7.0	-6.8	0.15	1.788	0.890	2.929	0.413	6	5147	ZINC000100781684
0.5	-10.3	-9.8	0.30	1.023	0.180	2.972	0.909	6	5148	ZINC000100776088
0.5	-7.1	-6.7	0.23	2.468	0.680	4.594	1.789	6	5149	ZINC000034279674
0.5	-8.5	-8.1	0.29	1.869	0.196	5.603	1.834	6	5150	ZINC000014916933
0.5	-7.8	-7.4	0.18	2.437	0.286	4.530	0.542	6	5151	ZINC000261499748
0.5	-8.0	-6.9	0.82	2.013	0.591	3.796	2.165	5	5152	ZINC000004521935
0.5	-6.6	-6.3	0.15	2.896	0.665	4.111	0.955	6	5153	ZINC000002561012

0.5	-5.4	-5.1	0.20	3.063	0.896	3.768	0.895	6	5154	ZINC000002575440
0.5	-7.8	-7.4	0.19	2.423	0.312	4.185	0.466	6	5155	ZINC000261499750
0.5	-7.1	-6.8	0.16	2.345	0.796	4.615	2.172	6	5156	ZINC000034267187
0.5	-8.0	-7.9	0.07	3.010	0.256	4.754	1.122	6	5157	ZINC000034382706
0.5	-10.0	-9.3	0.40	2.059	0.331	3.714	1.066	6	5158	ZINC000014697120
0.5	-7.3	-7.2	0.09	2.024	0.672	4.525	1.758	6	5159	ZINC000032839129
0.5	-6.0	-5.9	0.08	2.107	0.623	3.977	1.008	6	5160	ZINC000003881710
0.5	-6.8	-6.2	0.36	2.807	1.123	4.046	1.392	6	5161	ZINC000095620563
0.5	-6.0	-5.8	0.12	2.712	0.960	3.955	1.524	6	5162	ZINC000005513439
0.5	-9.3	-8.6	0.49	1.836	0.497	4.091	0.900	6	5163	ZINC000100782066
0.5	-5.3	-5.1	0.15	2.269	0.835	3.238	0.845	6	5164	ZINC000000158145
0.5	-8.8	-8.5	0.20	1.559	0.156	3.564	1.066	6	5165	ZINC000100779661
0.5	-7.8	-7.3	0.29	2.576	0.874	3.248	1.229	6	5166	ZINC000013379106
0.5	-9.5	-8.9	0.43	2.099	0.889	3.292	0.916	6	5167	ZINC000100780003
0.5	-6.0	-5.7	0.21	2.162	1.029	3.583	0.833	6	5168	ZINC000001676373

0.5	-5.3	-4.9	0.23	1.863	0.653	2.631	0.639	6	5169	ZINC000001586315
0.5	-7.9	-7.5	0.34	2.041	0.252	2.554	0.590	6	5170	ZINC000004556978
0.5	-7.9	-7.6	0.17	2.018	0.749	4.534	1.031	6	5171	ZINC000004474564
0.5	-6.3	-6.2	0.09	2.391	0.595	3.821	0.746	6	5172	ZINC000002015520
0.5	-7.3	-7.0	0.23	1.928	0.158	3.727	0.945	6	5173	ZINC000238754057
0.5	-7.5	-7.4	0.11	2.031	0.316	4.355	0.559	6	5174	ZINC000032303249
0.5	-6.4	-6.3	0.11	1.800	0.126	3.221	0.338	6	5175	ZINC000014588939
0.5	-7.7	-7.2	0.38	2.409	1.273	4.086	1.707	6	5176	ZINC000004096363
0.5	-6.2	-6.0	0.10	1.714	0.348	4.038	1.092	6	5177	ZINC000001680439
0.5	-8.1	-7.3	0.64	2.351	0.373	6.241	1.427	6	5178	ZINC000095617972
0.5	-6.1	-5.8	0.22	2.131	0.433	3.746	0.908	6	5179	ZINC000095618193
0.5	-8.5	-8.3	0.16	1.408	0.286	2.247	0.422	6	5180	ZINC000100155605
0.5	-8.6	-8.2	0.24	1.928	0.219	5.215	1.714	6	5181	ZINC000014916929
0.5	-6.6	-6.3	0.17	2.162	0.342	5.076	0.778	6	5182	ZINC000095618217
0.5	-5.9	-5.8	0.10	1.950	0.357	3.584	0.847	6	5183	ZINC000001716732

0.5	-9.0	-7.1	1.07	2.099	0.444	4.183	0.527	5	5184	ZINC000238738645
0.5	-8.3	-7.8	0.29	2.284	0.111	5.213	1.178	6	5185	ZINC000001747302
0.5	-5.9	-5.6	0.16	2.524	0.421	3.076	0.533	6	5186	ZINC000000388057
0.5	-8.1	-7.6	0.37	2.316	0.436	4.328	1.104	6	5187	ZINC000014486949
0.5	-5.7	-5.4	0.22	2.382	0.355	3.419	0.575	6	5188	ZINC000035021147
0.5	-7.1	-7.0	0.11	2.516	0.566	3.251	0.486	6	5189	ZINC000001081286
0.5	-7.5	-6.9	0.46	1.644	0.520	4.309	1.040	6	5190	ZINC000031169598
0.5	-8.5	-7.8	0.51	1.567	0.176	2.552	0.376	6	5191	ZINC000034042772
0.5	-5.1	-4.8	0.15	2.802	0.843	3.164	0.820	6	5192	ZINC000000900814
0.5	-7.1	-6.9	0.13	2.140	0.553	3.904	0.555	6	5193	ZINC000015253239
0.5	-9.2	-8.7	0.38	2.140	0.270	5.348	1.063	6	5194	ZINC000022061258
0.5	-9.8	-9.2	0.40	1.503	0.500	4.190	0.700	6	5195	ZINC000015160929
0.5	-4.7	-4.6	0.12	1.891	0.646	2.854	0.979	6	5196	ZINC000002005192
0.5	-7.5	-7.1	0.26	1.209	0.584	3.372	0.578	6	5197	ZINC000261496104
0.5	-5.7	-5.6	0.13	2.790	1.057	4.079	0.439	6	5198	ZINC000002019685

0.5	-5.3	-4.8	0.28	2.730	1.452	3.299	1.585	6	5199	ZINC000005836963
0.5	-8.2	-7.8	0.24	1.837	0.626	4.343	1.519	6	5200	ZINC000027638954
0.5	-8.8	-7.7	0.86	1.559	0.727	4.945	1.797	6	5201	ZINC000014641887
0.5	-8.8	-7.8	0.65	1.647	0.458	3.558	1.942	6	5202	ZINC000257430724
0.5	-9.0	-8.4	0.35	2.125	0.311	4.978	1.441	6	5203	ZINC000257619566
0.5	-4.4	-4.2	0.11	3.120	1.505	3.938	1.190	6	5204	ZINC000005783272
0.5	-8.0	-7.7	0.24	1.953	0.329	3.929	1.466	6	5205	ZINC000085951548
0.5	-8.7	-7.8	0.43	2.330	0.729	5.020	2.122	6	5206	ZINC000000395629
0.5	-8.7	-7.5	0.57	3.401	0.692	5.227	1.446	6	5207	ZINC000029566455
0.5	-5.2	-4.8	0.21	1.988	0.471	2.485	0.428	6	5208	ZINC000000394775
0.5	-6.9	-6.4	0.29	2.251	0.717	4.545	1.258	6	5209	ZINC000005662850
0.5	-8.8	-8.4	0.37	1.707	0.450	3.239	0.974	6	5210	ZINC000100775768
0.5	-7.3	-7.1	0.21	3.184	0.134	4.800	0.331	6	5211	ZINC000013508536
0.5	-6.7	-6.4	0.14	2.059	0.170	3.300	0.663	6	5212	ZINC000000896001
0.5	-6.1	-5.8	0.17	2.008	0.343	3.109	0.582	6	5213	ZINC000002169190

0.5	-9.0	-8.1	0.59	2.136	0.484	3.607	1.056	6	5214	ZINC000090636267
0.5	-8.8	-8.2	0.36	1.573	0.501	3.530	1.809	6	5215	ZINC000015251374
0.5	-6.8	-6.6	0.14	2.159	0.634	3.218	0.815	6	5216	ZINC000000494255
0.5	-8.0	-7.6	0.29	2.467	0.362	4.839	1.671	6	5217	ZINC000002560698
0.5	-4.5	-4.4	0.07	2.975	0.585	3.852	0.793	6	5218	ZINC000033951264
0.5	-5.2	-5.0	0.13	1.858	0.650	3.395	0.415	6	5219	ZINC000019877642
0.5	-5.4	-5.1	0.20	2.362	1.264	4.243	0.528	6	5220	ZINC000005651192
0.5	-10.1	-9.0	0.75	1.923	0.538	5.975	1.365	6	5221	ZINC000257418754
0.5	-7.6	-7.3	0.20	2.745	0.329	4.075	0.835	6	5222	ZINC000025725630
0.5	-7.0	-6.8	0.13	1.878	0.765	3.253	1.092	6	5223	ZINC000013383237
0.5	-7.2	-7.0	0.12	1.342	0.326	2.905	0.784	6	5224	ZINC000100781726
0.5	-5.9	-5.6	0.20	1.886	0.679	2.828	0.544	6	5225	ZINC000000395594
0.5	-6.9	-6.6	0.18	1.593	0.625	2.964	0.408	6	5226	ZINC000000162515
0.5	-7.1	-6.5	0.29	1.970	0.502	4.280	1.383	6	5227	ZINC000005999105
0.5	-9.0	-8.5	0.26	1.959	0.684	4.282	0.208	6	5228	ZINC000013485386

0.5	-6.6	-6.3	0.14	2.323	0.586	4.209	0.523	6	5229	ZINC000044710259
0.5	-6.5	-6.4	0.11	3.688	0.735	4.472	0.782	6	5230	ZINC000004430890
0.5	-5.9	-5.7	0.15	2.750	0.577	3.828	0.567	6	5231	ZINC000005839582
0.5	-6.6	-6.4	0.12	2.919	0.690	4.814	0.409	6	5232	ZINC000082395905
0.5	-7.9	-7.8	0.11	2.614	0.360	3.535	1.046	6	5233	ZINC000018275062
0.5	-7.7	-7.3	0.23	2.127	0.320	4.271	1.479	6	5234	ZINC000034800261
0.5	-7.1	-6.9	0.24	3.003	0.320	5.261	0.192	6	5235	ZINC000034361790
0.5	-10.5	-9.4	0.94	1.757	0.170	4.544	1.453	3	5236	ZINC000013302612
0.5	-6.5	-6.2	0.19	2.841	0.764	4.635	1.388	6	5237	ZINC000050027402
0.5	-8.1	-7.7	0.31	1.691	0.356	2.179	0.688	6	5238	ZINC000001605729
0.5	-9.3	-8.2	0.61	1.708	0.363	5.844	1.817	6	5239	ZINC000015219255
0.5	-7.7	-7.5	0.09	2.225	0.709	3.058	0.680	6	5240	ZINC000000084153
0.5	-6.1	-6.0	0.07	2.144	0.259	3.407	0.294	6	5241	ZINC000001850888
0.5	-8.6	-6.7	1.01	1.721	0.150	5.079	1.427	6	5242	ZINC000006031721
0.5	-7.9	-7.8	0.10	2.538	0.254	4.621	0.951	6	5243	ZINC000002561096

0.5	-8.2	-7.8	0.26	2.290	0.476	5.604	1.353	6	5244	ZINC000014720633
0.5	-8.1	-7.6	0.30	2.400	0.380	4.679	1.208	6	5245	ZINC000004899447
0.5	-7.2	-7.1	0.07	2.632	0.346	4.196	0.850	6	5246	ZINC000002011298
0.5	-7.5	-7.0	0.33	2.515	0.683	3.359	0.750	6	5247	ZINC000000895906
0.5	-6.5	-6.2	0.15	2.092	0.431	3.108	0.673	6	5248	ZINC000004528589
0.5	-5.4	-5.2	0.13	2.359	1.118	4.136	0.848	6	5249	ZINC000005651512
0.5	-8.8	-7.2	0.84	1.686	0.289	3.783	1.375	5	5250	ZINC000257452935
0.5	-8.8	-8.1	0.37	1.390	0.576	3.559	1.795	6	5251	ZINC000015251368
0.5	-5.4	-5.0	0.24	2.086	0.774	2.727	0.891	6	5252	ZINC000014438701
0.5	-8.1	-8.0	0.18	1.758	0.314	3.292	0.643	6	5253	ZINC000100781980
0.5	-7.7	-7.6	0.09	1.797	0.849	4.638	1.596	6	5254	ZINC000008860491
0.5	-9.6	-9.0	0.34	1.711	0.532	2.987	0.674	6	5255	ZINC000257525223
0.5	-10.1	-8.8	0.76	2.032	0.541	4.479	1.462	6	5256	ZINC000015264163
0.5	-9.7	-9.1	0.57	1.435	0.262	2.908	1.220	6	5257	ZINC000003861630
0.5	-5.9	-5.7	0.12	1.943	0.442	3.373	0.995	6	5258	ZINC000001668232

0.5	-6.7	-6.6	0.12	2.221	0.325	3.680	0.682	6	5259	ZINC000014588923
0.5	-8.9	-8.7	0.15	2.731	0.639	4.352	1.543	6	5260	ZINC000034349835
0.5	-9.9	-9.4	0.59	1.829	0.428	5.349	0.464	6	5261	ZINC000029056208
0.5	-8.0	-7.6	0.31	1.753	0.390	5.233	1.947	6	5262	ZINC000015259911
0.5	-7.1	-6.9	0.10	1.330	0.143	3.038	0.757	6	5263	ZINC000100007130
0.5	-7.9	-6.7	0.78	1.996	0.663	5.093	1.944	6	5264	ZINC000013480187
0.5	-7.0	-6.8	0.16	1.900	0.748	3.785	0.708	6	5265	ZINC000013383238
0.5	-7.2	-7.1	0.09	2.147	0.310	4.295	1.110	6	5266	ZINC000034410586
0.5	-8.0	-7.5	0.39	2.605	0.211	5.900	0.776	6	5267	ZINC000040165382
0.5	-6.8	-6.8	0.08	1.757	0.920	2.753	0.568	6	5268	ZINC000100002169
0.5	-9.3	-8.6	0.41	1.679	0.296	4.524	0.719	6	5269	ZINC000100782061
0.5	-5.3	-5.0	0.22	1.987	0.431	3.043	0.920	6	5270	ZINC000002011664
0.5	-5.9	-5.6	0.23	2.143	0.825	3.376	0.997	6	5271	ZINC000031703222
0.5	-8.3	-7.6	0.35	1.764	0.271	3.696	0.983	6	5272	ZINC000014453726
0.5	-9.9	-9.0	0.56	1.693	0.141	5.226	0.963	6	5273	ZINC000100779853

0.5	-9.1	-7.5	1.22	2.094	0.180	4.394	1.283	6	5274	ZINC000100778728
0.5	-7.8	-7.4	0.20	2.278	0.392	5.224	1.070	6	5275	ZINC000002561050
0.5	-10.2	-8.7	0.91	1.945	0.444	3.421	1.377	6	5276	ZINC000079041147
0.5	-6.6	-6.4	0.11	2.238	0.179	3.167	0.468	6	5277	ZINC000000901722
0.5	-9.6	-9.0	0.35	1.808	0.464	3.404	0.651	6	5278	ZINC000257525222
0.5	-6.5	-6.3	0.17	1.947	0.613	4.020	0.230	6	5279	ZINC000001577442
0.5	-8.4	-8.2	0.16	1.856	0.553	4.991	1.689	6	5280	ZINC000014455492
0.5	-8.9	-8.4	0.26	1.993	0.037	6.393	0.443	6	5281	ZINC000014438693
0.5	-8.3	-8.0	0.27	1.832	0.292	5.362	1.153	6	5282	ZINC000014448743
0.5	-6.2	-6.0	0.10	1.801	0.427	3.987	0.640	6	5283	ZINC000055169533
0.5	-7.6	-7.1	0.37	1.886	0.949	3.821	0.969	6	5284	ZINC000012495221
0.5	-8.5	-7.7	0.45	2.377	0.365	4.087	0.808	6	5285	ZINC000014508331
0.5	-7.2	-6.9	0.23	2.200	0.718	3.312	0.466	6	5286	ZINC000100829374
0.5	-5.9	-5.6	0.20	3.073	0.632	3.671	0.642	6	5287	ZINC000002031376
0.5	-7.3	-7.1	0.21	2.245	0.499	3.683	1.262	6	5288	ZINC000100774490

0.5	-7.5	-7.3	0.15	2.293	0.274	4.854	0.854	6	5289	ZINC000034961808
0.5	-7.0	-6.8	0.15	1.805	0.888	2.946	0.377	6	5290	ZINC000015119516
0.5	-6.9	-6.5	0.19	1.997	0.442	3.558	1.180	6	5291	ZINC000014722253
0.5	-7.8	-7.5	0.20	1.786	0.250	3.520	0.606	6	5292	ZINC000013432656
0.5	-6.7	-6.4	0.19	1.955	0.665	3.673	1.087	6	5293	ZINC000001677815
0.5	-6.6	-6.5	0.05	1.916	0.459	3.839	0.728	6	5294	ZINC000085589570
0.5	-5.0	-4.7	0.26	1.639	0.423	2.259	0.327	6	5295	ZINC000001693578
0.5	-4.9	-4.8	0.07	3.928	1.635	4.464	1.744	6	5296	ZINC000150338756
0.5	-7.6	-7.4	0.16	2.201	0.221	4.988	1.726	6	5297	ZINC000012496578
0.5	-6.4	-6.2	0.15	2.019	0.453	3.683	0.725	6	5298	ZINC000008234325
0.5	-9.7	-8.3	1.00	1.724	0.281	5.062	1.217	4	5299	ZINC000238749825
0.5	-5.8	-5.3	0.28	2.336	1.136	3.112	1.105	6	5300	ZINC000005225119
0.5	-7.7	-7.5	0.16	2.489	0.747	3.616	0.381	6	5301	ZINC000000158606
0.5	-6.6	-6.4	0.13	2.678	0.452	3.937	0.885	6	5302	ZINC000013520481
0.5	-7.8	-7.7	0.10	2.188	0.635	4.403	0.543	6	5303	ZINC000100829759

0.5	-7.8	-7.5	0.18	2.669	0.762	5.163	1.255	6	5304	ZINC000014590686
0.5	-6.4	-6.1	0.20	1.832	0.303	3.159	0.795	6	5305	ZINC000145910892
0.5	-6.8	-6.5	0.21	2.723	0.345	3.571	0.642	6	5306	ZINC000001699969
0.5	-5.8	-5.6	0.12	2.543	0.427	4.252	0.574	6	5307	ZINC000095620542
0.5	-6.9	-6.6	0.16	2.112	0.352	3.521	1.089	6	5308	ZINC000002557905
0.5	-7.0	-6.8	0.12	2.452	0.914	3.906	0.794	6	5309	ZINC000166269186
0.5	-6.9	-6.6	0.17	1.949	0.442	3.621	0.682	6	5310	ZINC000103211661
0.5	-5.7	-5.5	0.16	2.338	1.114	3.581	0.912	6	5311	ZINC000004353766
0.5	-7.9	-7.7	0.14	1.921	0.175	3.465	0.934	6	5312	ZINC000014657485
0.5	-10.6	-9.3	1.30	2.342	0.000	3.610	0.000	2	5313	ZINC000013302792
0.5	-8.0	-7.3	0.37	3.019	0.498	4.227	1.132	6	5314	ZINC000013379107
0.5	-3.4	-3.1	0.24	2.929	1.229	3.378	1.063	6	5315	ZINC000004658628
0.5	-8.1	-7.8	0.25	2.251	0.407	3.673	1.489	6	5316	ZINC000257406494
0.5	-9.1	-7.9	0.76	2.283	0.079	4.086	1.059	6	5317	ZINC000100775773
0.5	-6.3	-6.0	0.18	3.756	0.546	5.624	0.311	6	5318	ZINC000002557556

0.5	-7.1	-6.9	0.13	2.400	0.694	4.125	1.835	6	5319	ZINC000032839125
0.5	-10.1	-9.3	0.41	2.413	0.287	3.635	1.467	6	5320	ZINC000014651996
0.5	-7.6	-7.5	0.07	1.427	0.142	4.168	2.468	6	5321	ZINC000255970900
0.5	-6.6	-6.4	0.13	2.681	0.429	4.030	0.781	6	5322	ZINC000005211474
0.5	-8.0	-7.5	0.41	1.756	0.384	5.030	1.808	6	5323	ZINC000013374017
0.5	-5.4	-5.2	0.19	2.233	1.007	4.031	0.977	6	5324	ZINC000005863713
0.5	-4.9	-4.7	0.12	2.780	1.320	3.752	1.134	6	5325	ZINC000032163935
0.5	-10.7	-9.1	1.10	1.542	0.071	5.020	0.822	4	5326	ZINC000014636693
0.5	-6.4	-6.2	0.10	2.254	0.660	3.951	0.827	6	5327	ZINC000015120276
0.5	-6.7	-6.5	0.09	2.417	0.595	3.364	1.167	6	5328	ZINC000001845768
0.5	-9.0	-8.4	0.39	2.256	0.296	4.391	1.287	6	5329	ZINC000014774780
0.5	-7.8	-7.3	0.24	2.707	0.196	4.224	0.673	6	5330	ZINC000000402960
0.5	-8.2	-8.0	0.09	1.853	0.543	4.712	0.930	6	5331	ZINC000095619951
0.5	-8.0	-7.2	0.46	2.076	0.564	3.948	1.758	6	5332	ZINC000085661244
0.5	-7.4	-7.0	0.24	2.046	0.358	4.918	1.395	6	5333	ZINC000015219342

0.5	-8.5	-7.5	0.71	2.112	0.253	4.623	1.571	6	5334	ZINC000257442235
0.5	-7.2	-6.9	0.23	2.347	0.886	3.493	1.198	6	5335	ZINC000000393723
0.5	-10.4	-9.7	0.40	2.083	0.392	4.433	1.872	6	5336	ZINC000015169390
0.5	-7.0	-6.8	0.13	1.170	0.286	4.274	0.691	6	5337	ZINC000001704769
0.5	-5.3	-5.2	0.08	1.467	0.081	3.621	0.514	6	5338	ZINC000001606159
0.5	-7.4	-7.0	0.25	2.050	0.220	4.180	0.690	6	5339	ZINC000001874360
0.5	-7.8	-7.5	0.19	2.439	0.455	4.299	1.444	6	5340	ZINC000014684262
0.5	-8.0	-7.8	0.11	1.873	0.270	4.247	0.942	6	5341	ZINC000014556668
0.5	-8.9	-8.1	0.55	2.269	0.351	4.162	1.011	6	5342	ZINC000014486966
0.5	-8.4	-7.9	0.24	2.249	0.567	3.984	1.264	6	5343	ZINC000000284966
0.5	-6.7	-6.3	0.19	2.556	0.321	4.535	0.399	6	5344	ZINC000004556737
0.5	-6.5	-6.2	0.14	1.996	0.388	3.251	0.493	6	5345	ZINC000000967796
0.5	-7.9	-7.8	0.09	2.062	0.477	4.370	1.301	6	5346	ZINC000059588412
0.5	-6.9	-6.6	0.13	1.563	0.747	3.050	0.563	6	5347	ZINC000000897468
0.5	-7.5	-7.3	0.24	2.198	1.228	3.993	2.059	6	5348	ZINC000044675977

0.5	-6.7	-6.3	0.21	2.509	0.340	3.439	0.329	6	5349	ZINC000000395644
0.5	-6.7	-6.3	0.23	2.479	0.358	4.556	0.883	6	5350	ZINC000095618158
0.5	-6.7	-6.4	0.18	1.781	0.375	3.870	1.107	6	5351	ZINC000039090697
0.5	-6.4	-6.0	0.21	2.580	0.711	3.372	0.493	6	5352	ZINC000001530244
0.5	-8.3	-7.9	0.22	1.869	0.494	4.145	0.610	6	5353	ZINC000085826899
0.5	-4.9	-4.7	0.14	1.916	0.853	2.713	0.713	6	5354	ZINC000032153930
0.5	-8.8	-7.7	0.73	1.928	0.430	4.213	0.491	6	5355	ZINC000014810546
0.5	-6.8	-6.5	0.20	2.163	0.880	3.937	1.960	6	5356	ZINC000095620471
0.5	-10.4	-9.2	0.73	1.670	0.229	5.335	0.899	6	5357	ZINC000100781253
0.5	-6.8	-6.5	0.19	3.900	0.263	4.837	0.240	6	5358	ZINC000034676246
0.5	-5.7	-5.4	0.20	3.298	1.096	4.355	1.108	6	5359	ZINC000001850507
0.5	-7.3	-7.0	0.15	2.925	0.350	3.981	0.444	6	5360	ZINC000002169060
0.5	-7.4	-6.9	0.30	2.595	0.314	4.701	0.622	6	5361	ZINC000001786139
0.5	-8.7	-7.9	0.41	2.473	0.483	4.046	1.003	6	5362	ZINC000257376831
0.5	-8.6	-8.0	0.29	2.257	0.477	3.962	1.585	6	5363	ZINC000002561124

0.5	-6.6	-6.3	0.18	2.086	0.458	3.881	0.690	6	5364	ZINC000004262096
0.5	-10.1	-9.3	0.49	1.948	0.452	3.925	1.271	6	5365	ZINC000085989478
0.5	-7.2	-6.9	0.24	1.929	0.589	3.307	0.466	6	5366	ZINC000100829375
0.5	-9.9	-9.0	0.56	1.691	0.143	5.226	0.966	6	5367	ZINC000257538894
0.5	-8.3	-8.0	0.17	1.973	0.245	4.084	1.630	6	5368	ZINC000257557768
0.5	-6.8	-6.7	0.09	1.571	0.326	3.352	1.645	6	5369	ZINC000014858401
0.5	-8.5	-8.0	0.40	1.663	0.516	3.581	1.240	6	5370	ZINC000002334300
0.5	-6.5	-6.3	0.13	2.761	0.812	4.022	1.165	6	5371	ZINC000095618105
0.5	-6.0	-5.7	0.21	2.113	0.572	3.162	0.847	6	5372	ZINC000001530365
0.5	-6.6	-6.3	0.17	2.064	0.397	3.585	0.678	6	5373	ZINC000014774720
0.5	-7.1	-6.9	0.13	2.576	0.734	4.184	1.070	6	5374	ZINC000100774488
0.5	-5.4	-5.1	0.17	2.622	1.017	3.970	0.509	6	5375	ZINC000001850395
0.5	-8.7	-8.3	0.22	2.501	0.769	4.139	1.828	6	5376	ZINC000012495324
0.5	-7.5	-7.0	0.27	2.858	0.161	4.735	0.844	6	5377	ZINC000005273581
0.5	-8.7	-8.3	0.30	2.273	0.218	3.979	0.974	6	5378	ZINC000006032027

0.5	-8.6	-7.8	0.41	1.875	0.139	5.022	1.239	6	5379	ZINC000008860486
0.5	-6.6	-6.4	0.12	2.123	0.756	3.926	1.035	6	5380	ZINC000014639011
0.5	-6.9	-6.7	0.17	1.896	0.377	2.772	0.674	6	5381	ZINC000230080068
0.5	-8.1	-7.9	0.16	1.754	0.420	2.792	0.693	6	5382	ZINC000014614798
0.5	-8.7	-8.2	0.37	1.443	0.187	5.949	1.538	6	5383	ZINC000257464256
0.5	-5.5	-5.2	0.16	1.561	0.165	2.330	0.477	6	5384	ZINC000002019498
0.5	-7.9	-7.5	0.31	2.042	0.259	2.547	0.596	6	5385	ZINC000002558630
0.5	-9.0	-8.4	0.33	2.199	0.284	4.330	1.301	6	5386	ZINC000257619565
0.5	-7.0	-6.7	0.21	2.418	0.594	4.091	1.511	6	5387	ZINC000038417738
0.5	-6.5	-6.2	0.16	1.366	0.446	2.804	0.301	6	5388	ZINC000001085207
0.5	-7.9	-7.5	0.22	2.495	0.419	4.423	0.835	6	5389	ZINC000013307161
0.5	-8.9	-8.5	0.27	2.116	0.176	5.572	1.052	6	5390	ZINC000014854258
0.5	-7.5	-7.3	0.12	2.873	0.709	4.425	1.293	6	5391	ZINC000095617783
0.5	-6.7	-6.6	0.09	2.513	0.515	4.088	0.951	6	5392	ZINC000095620534
0.5	-5.8	-5.6	0.09	2.906	1.194	4.099	1.163	6	5393	ZINC000014593064

0.5	-7.0	-6.8	0.17	2.187	1.068	3.984	0.799	6	5394	ZINC000136148979
0.5	-5.8	-5.5	0.18	1.852	0.572	2.709	0.635	6	5395	ZINC000001850690
0.5	-7.6	-7.4	0.13	2.868	0.986	4.413	1.461	6	5396	ZINC000001669442
0.5	-6.9	-6.8	0.13	2.078	0.338	3.652	0.705	6	5397	ZINC000001576487
0.5	-7.2	-7.0	0.13	1.341	0.354	3.199	0.851	6	5398	ZINC000001599675
0.5	-6.6	-5.9	0.35	2.454	0.508	3.985	1.454	6	5399	ZINC000036092735
0.5	-6.3	-5.6	0.35	1.886	0.489	3.516	0.545	6	5400	ZINC000025695868
0.5	-5.5	-5.2	0.15	3.156	0.948	3.836	0.943	6	5401	ZINC000014451125
0.5	-6.5	-6.2	0.13	2.635	0.659	3.561	0.944	6	5402	ZINC000004654822
0.5	-6.9	-6.7	0.12	2.170	0.618	3.511	0.635	6	5403	ZINC000003846059
0.5	-7.8	-7.5	0.23	2.342	0.285	3.787	0.628	6	5404	ZINC000001575526
0.5	-8.9	-8.1	0.65	1.749	0.329	4.684	1.748	5	5405	ZINC000000900298
0.5	-6.9	-6.7	0.13	2.444	0.433	4.058	1.088	6	5406	ZINC000006068107
0.5	-8.5	-8.2	0.32	2.200	0.560	2.969	0.399	6	5407	ZINC000004096036
0.5	-6.7	-6.4	0.18	3.086	0.674	4.303	0.716	6	5408	ZINC000000405277

0.5	-7.2	-7.0	0.11	1.353	0.339	2.963	0.824	6	5409	ZINC000015169336
0.5	-5.6	-5.4	0.19	2.546	0.632	3.411	0.705	6	5410	ZINC000001081110
0.5	-8.5	-8.1	0.31	1.919	0.619	3.272	1.601	6	5411	ZINC000003882640
0.5	-8.7	-8.1	0.37	2.195	0.523	3.900	1.526	6	5412	ZINC000257530774
0.5	-7.4	-6.9	0.27	2.665	0.795	3.191	0.790	6	5413	ZINC000000003252
0.5	-6.5	-6.4	0.17	2.582	0.880	3.789	1.387	6	5414	ZINC000004529333
0.5	-8.0	-7.6	0.18	1.833	0.296	2.784	0.437	6	5415	ZINC000014720617
0.5	-7.2	-7.0	0.16	2.262	0.228	4.603	0.764	6	5416	ZINC000034397691
0.5	-7.7	-7.3	0.26	2.095	0.143	5.765	0.505	6	5417	ZINC000012496421
0.5	-7.1	-6.8	0.18	2.067	0.201	5.683	0.960	6	5418	ZINC000015122232
0.5	-6.9	-6.5	0.21	2.268	0.597	4.317	0.494	6	5419	ZINC000012495658
0.5	-7.4	-7.2	0.24	1.828	0.195	4.795	1.694	6	5420	ZINC000014714823
0.5	-5.6	-5.4	0.11	2.496	0.371	3.739	0.557	6	5421	ZINC000001850422
0.5	-6.2	-5.9	0.22	3.117	1.348	3.572	1.639	6	5422	ZINC000001530546
0.5	-7.2	-6.7	0.27	2.509	0.271	3.682	1.121	6	5423	ZINC000002562537

0.5	-8.2	-7.7	0.30	2.902	0.774	4.516	1.605	6	5424	ZINC000008215624
0.5	-6.7	-6.5	0.15	1.913	0.350	3.868	1.052	6	5425	ZINC000014639014
0.5	-7.1	-6.8	0.19	2.085	0.328	5.731	1.073	6	5426	ZINC000015122231
0.5	-9.0	-8.7	0.15	3.111	0.427	5.187	0.910	6	5427	ZINC000001730395
0.5	-9.8	-8.8	0.73	2.499	0.224	4.463	1.041	6	5428	ZINC000015044664
0.5	-8.7	-8.3	0.24	2.160	0.335	6.050	1.585	6	5429	ZINC000001561970
0.5	-6.6	-6.5	0.09	2.638	0.723	3.804	0.905	6	5430	ZINC000001692500
0.5	-7.0	-6.9	0.08	2.364	0.115	4.951	0.186	6	5431	ZINC000085589987
0.5	-8.0	-7.5	0.38	1.500	0.205	3.633	0.729	6	5432	ZINC000005103851
0.5	-9.0	-8.5	0.26	2.391	0.223	4.262	0.908	6	5433	ZINC000014486968
0.5	-9.3	-9.1	0.13	1.750	0.073	4.197	0.753	6	5434	ZINC000013484977
0.5	-6.8	-6.7	0.07	2.103	0.356	3.360	0.603	6	5435	ZINC000001087345
0.4	-6.4	-6.2	0.16	2.143	0.783	3.280	1.150	6	5436	ZINC000013542648
0.4	-8.9	-8.3	0.38	2.183	0.817	4.102	1.325	6	5437	ZINC000031440253
0.4	-6.3	-6.0	0.18	2.663	0.603	3.530	0.806	6	5438	ZINC000012493904

0.4	-6.7	-6.5	0.17	1.643	0.532	2.651	0.824	6	5439	ZINC000038673528
0.4	-6.8	-6.8	0.05	2.902	0.344	4.460	1.164	6	5440	ZINC000002545093
0.4	-5.2	-5.0	0.20	2.022	0.434	2.732	0.748	6	5441	ZINC000038613495
0.4	-5.8	-5.8	0.05	1.576	1.756	3.848	0.863	6	5442	ZINC000000004042
0.4	-7.3	-7.2	0.15	2.523	0.184	4.464	0.895	6	5443	ZINC000039818922
0.4	-8.7	-7.6	0.66	1.996	1.110	3.527	2.229	6	5444	ZINC000005554084
0.4	-5.4	-4.9	0.30	2.983	0.505	3.687	0.647	6	5445	ZINC000001850698
0.4	-7.5	-7.3	0.13	1.998	0.327	3.667	1.075	6	5446	ZINC000004096695
0.4	-6.7	-6.5	0.12	2.175	0.575	3.502	0.735	6	5447	ZINC000014588922
0.4	-7.0	-6.8	0.12	2.554	0.264	4.345	1.165	6	5448	ZINC000006036096
0.4	-7.3	-6.9	0.26	2.956	0.537	3.846	0.492	6	5449	ZINC000009280616
0.4	-6.2	-5.9	0.16	3.576	0.965	4.652	1.379	6	5450	ZINC000013413602
0.4	-5.3	-5.2	0.09	3.054	1.069	4.173	0.949	6	5451	ZINC000002510290
0.4	-7.4	-7.1	0.15	2.925	0.244	4.515	0.291	6	5452	ZINC000005501042
0.4	-7.9	-7.7	0.15	2.375	0.499	3.925	0.678	6	5453	ZINC000004097613

0.4	-6.8	-6.5	0.20	1.129	0.162	4.368	1.060	6	5454	ZINC000085972896
0.4	-8.9	-8.6	0.25	1.634	0.390	6.414	0.188	3	5455	ZINC000014858773
0.4	-8.6	-7.9	0.31	2.173	0.287	4.366	0.752	6	5456	ZINC000015061513
0.4	-5.6	-5.4	0.10	2.806	1.074	3.809	1.383	6	5457	ZINC000014680221
0.4	-7.7	-7.4	0.22	2.264	0.525	4.757	1.673	6	5458	ZINC000005641395
0.4	-8.5	-8.0	0.28	1.416	0.166	3.342	1.323	6	5459	ZINC000002010941
0.4	-6.0	-5.7	0.16	1.468	0.678	3.137	0.337	6	5460	ZINC000032169150
0.4	-5.8	-5.6	0.12	2.716	0.238	4.010	0.702	6	5461	ZINC000033844381
0.4	-5.1	-4.8	0.18	2.301	0.347	2.925	0.473	6	5462	ZINC000004293904
0.4	-8.7	-8.3	0.21	1.512	0.388	3.165	1.711	6	5463	ZINC000257579859
0.4	-5.1	-4.8	0.24	1.513	0.233	3.199	1.115	6	5464	ZINC000004218505
0.4	-7.7	-7.5	0.16	2.191	0.449	2.935	0.665	6	5465	ZINC000004096220
0.4	-3.8	-3.6	0.15	3.439	1.373	4.160	1.236	6	5466	ZINC000001675771
0.4	-6.8	-6.6	0.14	2.463	0.722	4.026	0.639	6	5467	ZINC000000902118
0.4	-8.2	-7.0	0.87	1.869	0.254	4.109	1.270	6	5468	ZINC000095617911

0.4	-7.7	-7.5	0.09	2.165	0.758	3.752	1.581	6	5469	ZINC000003869497
0.4	-9.5	-7.8	1.17	2.007	0.549	5.649	1.350	5	5470	ZINC000257382462
0.4	-5.9	-5.5	0.22	2.990	1.167	4.402	1.767	6	5471	ZINC000004557151
0.4	-9.0	-7.9	0.62	1.679	0.103	4.118	0.841	6	5472	ZINC000015113367
0.4	-10.0	-9.0	0.49	2.172	0.580	4.324	1.426	6	5473	ZINC000014697118
0.4	-8.8	-8.6	0.15	1.471	0.708	4.093	2.059	6	5474	ZINC000257417246
0.4	-9.4	-9.0	0.32	2.617	0.592	5.037	1.594	6	5475	ZINC000001624462
0.4	-7.9	-7.7	0.14	2.021	0.153	4.717	0.781	6	5476	ZINC000012496411
0.4	-7.4	-7.0	0.24	2.081	0.749	3.645	1.110	6	5477	ZINC000002534775
0.4	-8.2	-8.0	0.14	1.891	0.658	3.225	1.445	6	5478	ZINC000001999286
0.4	-6.5	-6.4	0.09	1.891	0.403	3.178	1.284	6	5479	ZINC000095620527
0.4	-7.0	-6.8	0.11	2.028	0.154	5.428	1.061	6	5480	ZINC000023355305
0.4	-8.7	-7.9	0.54	2.546	0.671	4.892	1.748	6	5481	ZINC000038581018
0.4	-5.3	-5.2	0.06	2.080	0.415	3.066	0.517	6	5482	ZINC000013480032
0.4	-4.9	-4.9	0.05	2.748	1.755	3.870	1.057	6	5483	ZINC000014822471

0.4	-7.2	-6.9	0.19	2.553	0.677	4.143	0.761	6	5484	ZINC000000154632
0.4	-8.0	-7.5	0.24	3.036	0.311	4.662	0.334	6	5485	ZINC000005134156
0.4	-7.3	-7.1	0.21	2.042	0.216	3.236	0.749	6	5486	ZINC000002384836
0.4	-10.0	-8.6	0.75	2.108	0.794	6.360	0.434	6	5487	ZINC000015266816
0.4	-5.9	-5.5	0.20	2.117	0.155	4.019	0.435	6	5488	ZINC000002038253
0.4	-9.3	-8.6	0.41	1.831	0.544	4.196	0.939	6	5489	ZINC000257382663
0.4	-6.7	-6.5	0.15	2.250	0.358	4.373	1.822	6	5490	ZINC000095617790
0.4	-4.9	-4.6	0.15	2.617	0.920	3.734	0.390	6	5491	ZINC000000396075
0.4	-4.3	-4.2	0.08	2.883	1.537	3.985	1.153	6	5492	ZINC000006037891
0.4	-8.0	-7.8	0.14	2.375	0.694	3.545	1.450	6	5493	ZINC000040493557
0.4	-6.7	-6.5	0.13	2.475	0.814	3.899	1.081	6	5494	ZINC000001680836
0.4	-5.8	-5.7	0.05	1.863	0.138	4.828	1.271	6	5495	ZINC000033839263
0.4	-6.1	-6.0	0.07	4.027	1.234	4.782	1.302	6	5496	ZINC000013517187
0.4	-8.8	-7.4	0.79	1.922	0.317	4.475	1.919	6	5497	ZINC000014811614
0.4	-9.1	-8.1	0.44	2.742	0.997	5.347	1.986	6	5498	ZINC000014916789

0.4	-5.0	-4.6	0.19	2.907	0.783	3.576	0.870	6	5499	ZINC000032166516
0.4	-5.3	-5.1	0.12	3.570	1.683	5.062	1.502	6	5500	ZINC000003869933
0.4	-10.3	-9.5	0.42	1.699	0.400	5.041	1.391	6	5501	ZINC000100778183
0.4	-5.1	-4.9	0.13	2.394	0.860	3.081	0.694	6	5502	ZINC000013462805
0.4	-6.2	-5.9	0.21	3.058	0.995	4.085	0.773	6	5503	ZINC000000896000
0.4	-9.4	-7.8	1.04	1.556	0.410	4.655	2.216	4	5504	ZINC000005276361
0.4	-8.9	-8.5	0.37	1.951	0.204	3.909	0.948	6	5505	ZINC000000056582
0.4	-6.7	-6.5	0.11	1.681	0.545	3.613	1.071	6	5506	ZINC000002000965
0.4	-6.3	-5.9	0.24	2.303	0.969	4.261	0.447	6	5507	ZINC000014591089
0.4	-6.0	-5.9	0.10	3.739	1.328	5.068	1.772	6	5508	ZINC000033839282
0.4	-8.0	-7.7	0.20	2.085	0.429	3.942	1.400	6	5509	ZINC000014589899
0.4	-6.9	-6.6	0.14	2.235	0.615	3.932	1.335	6	5510	ZINC000001629225
0.4	-6.0	-5.8	0.17	2.057	1.146	2.935	0.717	6	5511	ZINC000001698748
0.4	-8.6	-7.4	0.88	1.829	0.266	3.996	0.545	6	5512	ZINC000257741511
0.4	-6.5	-6.2	0.18	2.113	0.502	3.258	0.722	6	5513	ZINC000000895928

0.4	-7.0	-6.8	0.14	2.570	0.817	3.972	0.884	6	5514	ZINC000004482687
0.4	-9.1	-8.4	0.56	1.437	0.255	3.792	1.221	6	5515	ZINC000085887481
0.4	-4.3	-4.2	0.09	3.086	1.406	3.957	1.183	6	5516	ZINC000006031296
0.4	-7.5	-7.3	0.17	2.376	0.190	5.399	0.318	6	5517	ZINC000033822372
0.4	-7.8	-7.4	0.32	1.486	0.747	3.930	1.242	6	5518	ZINC000000057020
0.4	-7.5	-7.1	0.27	2.413	1.276	3.003	1.592	6	5519	ZINC000000039798
0.4	-9.5	-8.3	0.70	1.848	0.082	5.186	0.936	6	5520	ZINC000230086108
0.4	-8.7	-8.0	0.37	1.935	0.451	3.337	1.703	6	5521	ZINC000095619754
0.4	-6.3	-6.2	0.08	2.141	0.211	4.489	1.429	6	5522	ZINC000050027299
0.4	-6.7	-6.4	0.21	1.933	0.431	3.568	1.072	6	5523	ZINC000050027381
0.4	-6.6	-6.3	0.21	2.230	0.705	3.345	1.302	6	5524	ZINC000002384609
0.4	-6.7	-6.4	0.16	2.036	0.551	3.820	0.936	6	5525	ZINC000001849844
0.4	-6.0	-5.7	0.19	2.419	1.238	3.512	1.275	6	5526	ZINC000001641350
0.4	-8.2	-8.0	0.15	1.241	0.544	4.029	1.644	6	5527	ZINC000001095292
0.4	-7.4	-7.0	0.19	2.470	0.432	3.963	0.991	6	5528	ZINC000003776573

0.4	-8.1	-7.9	0.14	1.434	0.063	3.166	1.256	6	5529	ZINC000014586897
0.4	-6.6	-6.4	0.12	1.764	0.392	2.898	0.381	6	5530	ZINC000005699887
0.4	-9.0	-8.7	0.25	0.969	0.557	3.354	1.755	6	5531	ZINC000257563699
0.4	-6.9	-6.7	0.12	2.904	0.827	4.566	0.727	6	5532	ZINC000040472594
0.4	-7.5	-7.1	0.22	2.534	0.316	5.042	0.511	6	5533	ZINC000034018757
0.4	-6.9	-6.7	0.15	3.082	1.313	4.288	1.724	6	5534	ZINC000044710258
0.4	-6.9	-6.5	0.19	2.027	0.649	3.069	0.648	6	5535	ZINC000257379870
0.4	-6.7	-6.3	0.30	2.050	0.397	3.728	0.805	6	5536	ZINC000095619764
0.4	-6.9	-6.7	0.17	2.429	0.853	4.069	2.107	6	5537	ZINC000002572103
0.4	-5.6	-5.3	0.17	1.418	0.516	2.425	0.366	6	5538	ZINC000005771137
0.4	-5.1	-4.9	0.15	1.863	0.636	3.078	0.623	6	5539	ZINC000005225143
0.4	-8.2	-7.9	0.37	2.602	0.371	3.771	0.597	6	5540	ZINC000004038394
0.4	-9.5	-8.8	0.68	1.422	0.226	2.440	0.659	6	5541	ZINC000059774636
0.4	-6.9	-6.6	0.20	1.905	0.601	2.670	0.312	6	5542	ZINC000014505663
0.4	-8.6	-8.0	0.36	2.112	0.359	3.675	1.498	6	5543	ZINC000257530772

0.4	-8.5	-7.7	0.49	1.896	0.712	3.620	1.696	6	5544	ZINC000257485927
0.4	-7.5	-7.3	0.13	1.681	0.453	3.687	1.111	6	5545	ZINC000014771119
0.4	-6.8	-6.5	0.17	2.143	0.611	3.287	1.563	6	5546	ZINC000034279055
0.4	-7.7	-7.2	0.29	2.405	0.360	4.766	0.833	6	5547	ZINC000004556745
0.4	-7.9	-7.6	0.18	1.627	0.128	2.280	0.422	6	5548	ZINC000014455634
0.4	-6.1	-5.9	0.16	2.310	0.320	3.693	0.545	6	5549	ZINC000005514849
0.4	-8.9	-8.3	0.47	2.234	0.285	5.032	1.001	6	5550	ZINC000014727930
0.4	-9.5	-8.3	0.69	1.747	0.132	4.866	0.652	6	5551	ZINC000100780126
0.4	-6.6	-6.2	0.20	2.941	0.385	5.087	0.667	6	5552	ZINC000095617788
0.4	-8.8	-8.4	0.31	1.829	0.542	4.418	0.982	6	5553	ZINC000006494597
0.4	-6.2	-6.1	0.12	2.440	0.344	4.101	0.500	6	5554	ZINC000001731782
0.4	-8.5	-7.9	0.49	1.773	0.271	4.460	1.285	6	5555	ZINC000015159846
0.4	-5.3	-4.9	0.24	2.120	0.743	3.409	0.680	6	5556	ZINC000001529451
0.4	-7.7	-7.3	0.24	2.702	0.343	3.685	0.796	6	5557	ZINC000001611085
0.4	-6.9	-6.7	0.13	2.865	0.672	5.433	0.582	6	5558	ZINC000083409146

0.4	-7.1	-6.9	0.11	2.400	0.220	3.089	0.363	6	5559	ZINC000014657634
0.4	-6.2	-6.0	0.15	2.423	0.894	3.842	1.757	6	5560	ZINC000001578621
0.4	-7.7	-7.6	0.11	1.964	0.458	4.546	1.185	6	5561	ZINC000003982574
0.4	-5.7	-5.3	0.20	1.829	0.475	3.775	0.653	6	5562	ZINC000001850963
0.4	-6.6	-6.4	0.12	2.349	0.318	4.015	0.602	6	5563	ZINC000004082316
0.4	-5.2	-4.9	0.15	2.371	0.476	3.436	0.670	6	5564	ZINC000005840154
0.4	-9.4	-9.0	0.32	1.432	0.157	3.900	1.303	6	5565	ZINC000100783845
0.4	-10.2	-8.3	1.11	1.708	0.219	4.131	2.252	5	5566	ZINC000003881412
0.4	-6.2	-6.1	0.11	2.019	1.109	3.167	0.976	6	5567	ZINC000001690956
0.4	-8.5	-7.8	0.37	2.622	0.907	4.984	1.621	6	5568	ZINC000004228232
0.4	-6.5	-6.4	0.07	2.188	0.223	3.423	0.735	6	5569	ZINC000001841361
0.4	-7.0	-6.7	0.16	2.755	0.756	4.529	1.720	6	5570	ZINC000035024346
0.4	-7.6	-7.2	0.19	1.838	0.138	4.743	1.504	6	5571	ZINC000014610448
0.4	-5.4	-5.1	0.15	2.205	0.772	3.006	0.718	6	5572	ZINC000005841345
0.4	-9.5	-9.0	0.36	1.743	0.174	5.324	0.888	6	5573	ZINC000004096852

0.4	-5.5	-5.2	0.17	1.912	1.057	2.968	1.352	6	5574	ZINC000002037537
0.4	-7.6	-7.3	0.21	2.499	0.673	4.546	1.590	6	5575	ZINC000014855862
0.4	-5.1	-5.0	0.10	1.797	0.198	2.967	0.352	6	5576	ZINC000002384645
0.4	-8.3	-7.8	0.26	1.704	0.446	3.630	0.863	6	5577	ZINC000013130932
0.4	-5.5	-5.4	0.05	2.670	0.226	4.025	0.217	6	5578	ZINC000001691303
0.4	-8.6	-8.3	0.19	2.141	0.766	5.101	2.193	6	5579	ZINC000001550030
0.4	-6.2	-5.9	0.20	2.172	1.300	4.033	0.998	6	5580	ZINC000000901307
0.4	-6.1	-6.0	0.07	2.313	0.467	3.655	0.975	6	5581	ZINC000005360037
0.4	-6.2	-6.0	0.10	2.872	0.099	4.533	0.331	6	5582	ZINC000033949574
0.4	-7.0	-6.5	0.22	1.821	0.393	3.913	1.503	6	5583	ZINC000100772469
0.4	-7.7	-7.5	0.10	2.339	0.077	4.365	1.479	6	5584	ZINC000006091890
0.4	-6.8	-6.6	0.11	2.255	0.659	3.984	2.040	6	5585	ZINC000034946855
0.4	-7.6	-7.5	0.07	2.166	0.641	4.230	2.288	6	5586	ZINC000003869417
0.4	-6.5	-6.2	0.16	2.020	0.371	3.704	1.134	6	5587	ZINC000001529263
0.4	-5.7	-5.5	0.13	1.916	0.420	2.744	0.780	6	5588	ZINC000001997105

0.4	-8.5	-6.7	1.11	2.163	0.424	4.359	2.152	5	5589	ZINC000003830216
0.4	-8.0	-7.5	0.32	2.420	0.543	3.437	0.605	6	5590	ZINC000100778150
0.4	-6.7	-6.6	0.06	1.429	0.286	3.622	0.660	6	5591	ZINC000000001086
0.4	-7.9	-7.4	0.38	2.503	0.440	4.735	0.654	6	5592	ZINC000014486943
0.4	-8.5	-8.2	0.28	1.906	0.371	3.833	1.372	6	5593	ZINC000100773217
0.4	-7.8	-7.2	0.32	2.136	0.548	3.730	1.056	6	5594	ZINC000014590663
0.4	-6.7	-6.5	0.12	3.071	0.612	4.713	1.187	6	5595	ZINC000095617773
0.4	-10.5	-9.8	0.48	1.459	0.888	2.672	0.419	6	5596	ZINC000103556301
0.4	-8.9	-7.9	0.51	1.843	0.443	4.354	2.124	6	5597	ZINC000013327491
0.4	-8.9	-8.4	0.27	1.439	0.244	2.613	0.594	6	5598	ZINC000253497769
0.4	-9.2	-8.7	0.40	1.785	0.651	3.775	1.508	6	5599	ZINC000100771516
0.4	-8.0	-7.5	0.24	1.980	0.553	4.723	1.500	6	5600	ZINC000027645258
0.4	-5.9	-5.8	0.06	2.524	0.999	4.607	0.972	6	5601	ZINC000033950846
0.4	-5.2	-5.0	0.15	2.783	0.824	3.556	0.689	6	5602	ZINC000002031908
0.4	-5.5	-5.2	0.29	2.846	0.647	3.472	0.879	6	5603	ZINC000001587816

0.4	-9.1	-8.2	0.52	2.027	0.369	5.755	0.693	6	5604	ZINC000014642306
0.4	-4.9	-4.9	0.05	4.241	0.044	4.739	0.299	6	5605	ZINC000000407028
0.4	-9.3	-8.6	0.48	1.539	0.191	3.909	1.691	6	5606	ZINC000100780644
0.4	-5.4	-5.1	0.16	3.231	1.339	4.462	1.112	6	5607	ZINC000000347124
0.4	-4.5	-4.4	0.09	3.332	1.556	3.968	1.355	6	5608	ZINC000005765023
0.4	-8.6	-8.5	0.07	2.294	1.046	4.713	1.411	6	5609	ZINC000000047553
0.4	-8.5	-7.9	0.40	2.583	0.672	4.701	1.770	6	5610	ZINC000002020212
0.4	-7.0	-6.9	0.13	2.211	1.120	4.116	1.210	6	5611	ZINC000006093443
0.4	-6.2	-5.9	0.17	2.612	0.539	3.827	0.895	6	5612	ZINC000001575496
0.4	-6.0	-5.5	0.27	1.577	0.377	3.476	0.837	6	5613	ZINC000001677780
0.4	-7.3	-7.1	0.13	2.042	0.258	5.046	1.386	6	5614	ZINC000014455360
0.4	-9.0	-8.2	0.41	2.155	0.310	4.480	1.572	6	5615	ZINC000257508719
0.4	-6.1	-5.7	0.35	2.532	1.182	3.924	1.437	6	5616	ZINC000002039411
0.4	-6.8	-6.6	0.17	2.095	0.671	3.292	1.094	6	5617	ZINC000000901641
0.4	-5.2	-4.9	0.22	1.819	0.525	3.334	0.501	6	5618	ZINC000001688155

0.4	-8.6	-8.3	0.19	1.907	0.280	4.373	1.228	6	5619	ZINC000002525299
0.4	-9.5	-8.1	0.75	1.558	0.330	4.518	2.348	6	5620	ZINC000105249481
0.4	-6.9	-6.6	0.17	2.394	0.847	4.364	0.897	6	5621	ZINC000005830072
0.4	-8.6	-8.4	0.27	1.793	1.051	4.213	1.031	6	5622	ZINC000008579417
0.4	-9.4	-9.0	0.34	1.437	0.107	3.855	1.790	6	5623	ZINC000100783844
0.4	-9.4	-9.0	0.38	1.929	0.656	4.582	2.151	6	5624	ZINC000002149675
0.4	-8.8	-8.4	0.31	1.760	0.167	4.169	1.654	6	5625	ZINC000014593853
0.4	-9.5	-8.8	0.40	1.206	0.125	4.271	1.955	6	5626	ZINC000004082210
0.4	-7.2	-6.8	0.23	3.456	0.577	4.810	0.573	6	5627	ZINC000017952885
0.4	-3.9	-3.7	0.16	1.653	1.161	2.722	1.127	6	5628	ZINC000001627227
0.4	-6.6	-6.5	0.09	1.926	0.840	2.811	1.015	6	5629	ZINC000000900177
0.4	-6.0	-5.8	0.15	2.131	0.348	3.424	0.624	6	5630	ZINC000001581357
0.4	-6.1	-5.8	0.18	2.608	0.784	4.082	1.458	6	5631	ZINC000001532909
0.4	-7.7	-7.3	0.38	2.276	0.408	5.004	0.715	6	5632	ZINC000013377376
0.4	-5.3	-5.1	0.13	3.034	0.786	3.996	1.089	6	5633	ZINC000001850892

0.4	-6.9	-6.7	0.13	1.897	0.158	4.257	0.377	6	5634	ZINC000004899613
0.4	-5.7	-5.4	0.17	2.702	1.008	3.917	1.240	6	5635	ZINC000002558610
0.4	-6.1	-5.7	0.26	3.005	0.801	4.055	1.088	6	5636	ZINC000003650146
0.4	-8.0	-7.6	0.33	1.783	0.535	3.260	1.374	6	5637	ZINC000014720619
0.4	-6.3	-6.1	0.17	2.550	0.651	4.020	0.537	6	5638	ZINC000001410681
0.4	-7.6	-7.4	0.14	1.835	0.138	3.874	0.885	6	5639	ZINC000100822072
0.4	-6.6	-6.3	0.21	2.935	0.826	3.633	0.971	6	5640	ZINC000001529427
0.4	-9.5	-8.1	0.64	1.810	0.190	5.504	1.964	6	5641	ZINC000257568396
0.4	-7.4	-7.2	0.14	2.669	0.333	3.128	0.252	6	5642	ZINC000040470269
0.4	-7.5	-7.2	0.16	2.146	0.796	4.858	1.184	6	5643	ZINC000027644415
0.4	-8.1	-7.8	0.26	3.204	0.448	4.624	0.638	6	5644	ZINC000030728438
0.4	-8.2	-7.6	0.36	1.588	0.391	3.898	1.218	6	5645	ZINC000039236424
0.4	-8.1	-7.9	0.15	2.698	0.685	5.194	1.556	6	5646	ZINC000004096023
0.4	-7.1	-6.9	0.12	2.774	0.448	3.998	0.290	6	5647	ZINC000013451458
0.4	-6.0	-5.7	0.15	2.291	0.346	4.034	1.249	6	5648	ZINC000003870097

0.4	-10.0	-9.6	0.23	1.246	0.536	3.793	0.617	6	5649	ZINC000012363891
0.4	-8.6	-8.2	0.28	1.943	0.237	4.896	1.582	6	5650	ZINC000257402422
0.4	-9.7	-8.3	0.67	1.943	0.692	3.614	1.514	6	5651	ZINC000014410370
0.4	-5.3	-5.2	0.12	1.437	0.392	3.573	0.904	6	5652	ZINC000001648294
0.4	-6.6	-6.4	0.17	2.394	0.348	3.285	0.803	6	5653	ZINC000005192424
0.4	-7.5	-7.2	0.18	1.649	0.477	3.378	0.449	6	5654	ZINC000004410593
0.4	-6.2	-5.9	0.21	3.518	0.885	4.653	1.360	6	5655	ZINC000014948997
0.4	-7.6	-7.3	0.22	2.732	0.613	4.350	1.845	6	5656	ZINC000003795098
0.4	-9.1	-8.4	0.36	2.221	0.558	4.421	0.478	6	5657	ZINC000012153083
0.4	-6.5	-6.0	0.28	2.633	0.674	4.339	1.510	6	5658	ZINC000002384798
0.4	-7.0	-6.7	0.21	1.902	0.239	3.052	0.949	6	5659	ZINC000003869770
0.4	-5.8	-5.5	0.13	2.405	1.316	3.650	0.924	6	5660	ZINC000039070276
0.4	-6.8	-6.5	0.21	2.311	0.998	5.512	1.345	6	5661	ZINC000000899863
0.4	-8.6	-8.3	0.13	1.384	0.274	3.297	0.331	6	5662	ZINC000257532967
0.4	-5.5	-5.4	0.10	2.641	0.933	3.724	0.821	6	5663	ZINC000000163290

0.4	-8.6	-8.3	0.23	1.861	0.290	4.996	1.344	6	5664	ZINC000013359966
0.4	-5.5	-5.3	0.21	2.250	0.761	3.510	0.837	6	5665	ZINC000039374929
0.4	-7.9	-7.5	0.19	2.794	0.966	3.717	1.404	6	5666	ZINC000000057378
0.4	-9.1	-8.8	0.16	1.456	0.264	2.738	0.928	6	5667	ZINC000256430651
0.4	-6.6	-6.2	0.20	2.476	0.162	4.112	0.155	6	5668	ZINC000257524205
0.4	-6.6	-6.1	0.33	2.724	1.247	4.069	1.713	6	5669	ZINC000095620558
0.4	-6.5	-6.4	0.07	2.127	0.568	5.165	0.416	6	5670	ZINC000095618216
0.4	-6.7	-6.5	0.08	2.155	0.189	2.643	0.285	6	5671	ZINC000003913406
0.4	-7.9	-7.5	0.20	2.051	0.309	5.147	1.649	6	5672	ZINC000015261360
0.4	-8.0	-7.5	0.34	1.849	0.319	5.393	0.549	6	5673	ZINC000004097121
0.4	-8.2	-7.8	0.25	2.840	0.269	4.275	0.646	6	5674	ZINC000003872521
0.4	-9.9	-9.5	0.24	1.542	0.453	3.333	0.905	6	5675	ZINC000257453447
0.4	-8.0	-7.5	0.29	2.344	0.434	4.308	1.677	6	5676	ZINC000040479044
0.4	-8.4	-7.4	0.50	2.534	0.128	5.452	1.168	6	5677	ZINC000014643306
0.4	-8.7	-8.3	0.34	2.086	0.685	4.212	0.988	6	5678	ZINC000059206760

0.4	-6.6	-6.2	0.19	2.572	0.898	4.413	0.845	6	5679	ZINC000002560662
0.4	-7.0	-6.7	0.23	2.149	0.153	3.475	1.380	6	5680	ZINC000095617797
0.4	-9.1	-8.5	0.41	1.725	0.482	4.134	0.365	4	5681	ZINC000100823130
0.4	-6.2	-5.9	0.21	2.152	0.419	3.789	0.977	6	5682	ZINC000095620587
0.4	-8.7	-7.7	0.70	2.191	0.155	4.027	0.429	6	5683	ZINC000257390697
0.4	-7.4	-6.8	0.30	2.423	0.483	4.014	0.439	6	5684	ZINC000000394965
0.4	-8.1	-8.0	0.13	1.625	0.267	3.128	1.188	6	5685	ZINC000001851016
0.4	-9.3	-8.8	0.41	1.965	0.837	3.924	1.871	6	5686	ZINC000000899682
0.4	-9.0	-8.6	0.34	1.648	0.229	4.455	1.328	6	5687	ZINC000014725180
0.4	-6.0	-5.7	0.23	1.497	0.518	3.996	0.741	6	5688	ZINC000001531008
0.4	-9.6	-9.1	0.33	1.797	0.185	4.763	1.062	5	5689	ZINC000253389145
0.4	-4.9	-4.7	0.11	2.267	0.918	3.456	0.804	6	5690	ZINC000032152465
0.4	-6.8	-6.6	0.13	2.547	0.648	3.978	1.162	6	5691	ZINC000002560982
0.4	-5.2	-5.0	0.11	1.350	0.142	3.215	0.558	6	5692	ZINC000071775220
0.4	-9.5	-8.3	0.70	1.736	0.139	4.863	0.642	6	5693	ZINC000100780125

0.4	-7.2	-7.0	0.16	2.199	0.516	4.573	1.232	6	5694	ZINC000013341252
0.4	-6.3	-6.0	0.14	2.197	0.360	3.354	0.667	6	5695	ZINC000001764662
0.4	-8.4	-8.1	0.17	1.840	0.945	4.754	0.248	6	5696	ZINC000001679978
0.4	-6.5	-6.3	0.14	1.921	0.207	4.169	1.006	6	5697	ZINC000100771706
0.4	-9.6	-8.3	1.07	1.787	0.386	5.086	1.189	6	5698	ZINC000000402709
0.4	-9.1	-8.6	0.39	1.654	0.245	4.646	1.271	6	5699	ZINC000100782055
0.4	-6.7	-6.3	0.22	1.887	0.291	4.346	1.129	6	5700	ZINC000039090698
0.4	-7.0	-6.6	0.30	1.746	0.472	2.445	0.567	6	5701	ZINC000038612043
0.4	-9.1	-8.0	0.54	1.869	0.697	3.660	1.201	6	5702	ZINC000100778208
0.4	-8.9	-8.6	0.20	1.736	0.457	4.830	2.166	6	5703	ZINC000013508093
0.4	-5.9	-5.6	0.21	2.523	0.118	3.692	0.579	6	5704	ZINC000005134905
0.4	-5.8	-5.5	0.18	1.810	0.516	3.266	0.635	6	5705	ZINC000001672954
0.4	-7.0	-6.9	0.13	2.112	0.554	3.917	0.549	6	5706	ZINC000015253234
0.4	-8.2	-7.8	0.31	1.148	0.445	5.258	0.824	6	5707	ZINC000014825618
0.4	-10.5	-9.7	0.52	2.606	1.381	3.952	1.475	6	5708	ZINC000100091857

0.4	-4.7	-4.5	0.12	2.508	0.516	3.081	0.605	6	5709	ZINC000032175836
0.4	-9.1	-8.5	0.27	1.636	0.320	4.274	1.130	6	5710	ZINC000013136661
0.4	-5.2	-4.8	0.28	1.955	0.584	2.887	1.152	6	5711	ZINC000000409359
0.4	-8.1	-7.7	0.23	2.036	0.742	3.538	1.240	6	5712	ZINC000013380360
0.4	-4.4	-4.1	0.13	3.448	0.085	4.131	0.206	6	5713	ZINC000005227203
0.4	-7.5	-7.2	0.18	1.649	0.476	3.372	0.446	6	5714	ZINC000100082089
0.4	-6.0	-5.8	0.15	1.957	0.326	3.155	0.524	6	5715	ZINC000001850597
0.4	-6.9	-6.8	0.07	2.526	0.219	4.412	0.708	6	5716	ZINC000040439604
0.4	-8.4	-8.1	0.23	2.430	0.622	4.104	1.248	6	5717	ZINC000040165389
0.4	-3.9	-3.8	0.11	0.846	0.447	2.829	0.172	6	5718	ZINC000001242720
0.4	-5.4	-5.1	0.16	1.496	0.449	2.509	0.425	6	5719	ZINC000000409361
0.4	-7.8	-7.4	0.23	2.519	0.860	4.853	1.567	6	5720	ZINC000014590687
0.4	-9.9	-8.6	0.63	1.798	0.567	4.597	1.395	6	5721	ZINC000031622969
0.4	-5.2	-5.1	0.07	1.249	0.274	3.284	0.405	6	5722	ZINC000001606157
0.4	-6.1	-6.0	0.15	1.833	0.188	3.562	1.088	6	5723	ZINC000100828979

0.4	-8.7	-8.1	0.46	1.918	0.250	4.129	1.144	6	5724	ZINC000100781027
0.4	-6.6	-6.0	0.32	2.318	0.914	3.264	0.692	6	5725	ZINC000000001554
0.4	-6.9	-6.7	0.25	2.272	0.569	3.877	1.730	6	5726	ZINC000005618628
0.4	-9.4	-8.6	0.69	1.579	0.348	3.680	1.262	6	5727	ZINC000014654137
0.4	-9.1	-8.2	0.57	1.914	0.237	3.054	0.664	6	5728	ZINC000100257126
0.4	-6.8	-6.5	0.19	2.480	0.864	4.215	1.071	6	5729	ZINC000013375684
0.4	-8.3	-7.9	0.21	2.814	0.281	4.912	1.233	6	5730	ZINC000017994680
0.4	-6.2	-5.8	0.22	2.345	0.779	3.553	0.761	6	5731	ZINC000034926229
0.4	-10.7	-9.6	0.94	1.561	0.427	4.423	1.727	5	5732	ZINC000001531300
0.4	-6.3	-6.1	0.15	2.570	0.946	3.921	0.617	6	5733	ZINC000034287734
0.4	-7.1	-6.8	0.17	2.207	0.666	4.661	1.956	6	5734	ZINC000015120278
0.4	-7.6	-7.4	0.12	2.687	0.813	4.751	0.974	6	5735	ZINC000014616262
0.4	-8.0	-7.6	0.26	2.114	0.621	3.329	1.686	6	5736	ZINC000002583634
0.4	-10.6	-9.5	0.57	1.911	0.290	5.074	1.432	6	5737	ZINC000257383245
0.4	-5.4	-5.2	0.16	1.948	0.483	3.236	0.473	6	5738	ZINC000038609497

0.4	-5.7	-5.5	0.11	2.363	0.829	3.528	0.608	6	5739	ZINC000000967331
0.4	-10.5	-9.8	0.49	2.139	1.486	3.895	1.537	6	5740	ZINC000253633069
0.4	-9.1	-8.4	0.35	2.321	0.560	4.659	0.527	6	5741	ZINC000012153082
0.4	-10.2	-9.6	0.99	2.487	0.681	5.495	1.452	5	5742	ZINC000002019691
0.4	-6.8	-6.6	0.12	2.413	0.215	4.604	0.226	6	5743	ZINC000050027757
0.4	-5.2	-4.9	0.23	2.073	0.707	3.050	0.816	6	5744	ZINC000001648285
0.4	-7.4	-7.1	0.17	1.971	0.391	3.125	1.550	6	5745	ZINC000005116874
0.4	-6.9	-6.8	0.14	2.289	0.375	3.058	0.564	6	5746	ZINC000040471223
0.4	-10.0	-8.9	0.62	1.849	0.248	4.211	0.732	6	5747	ZINC000005924185
0.4	-9.5	-9.2	0.23	2.844	0.598	3.940	0.872	6	5748	ZINC000013374802
0.4	-6.9	-6.6	0.12	3.013	1.002	4.689	1.278	6	5749	ZINC000001576296
0.4	-6.7	-6.6	0.07	2.717	0.724	4.320	0.783	6	5750	ZINC000014636769
0.4	-10.0	-8.7	0.93	2.043	0.383	4.162	1.358	4	5751	ZINC000014650485
0.4	-7.5	-7.2	0.16	2.436	0.366	4.463	0.408	6	5752	ZINC000030730678
0.4	-6.2	-6.0	0.10	2.090	0.384	4.533	0.541	6	5753	ZINC000095620593

0.4	-9.5	-8.4	0.63	1.825	0.228	5.218	0.681	6	5754	ZINC000257374302
0.4	-7.0	-6.6	0.25	2.400	0.465	4.352	0.802	6	5755	ZINC000050027217
0.4	-6.7	-6.5	0.19	2.994	0.741	5.007	1.006	6	5756	ZINC000040470649
0.4	-7.2	-6.9	0.20	1.933	0.742	3.910	1.240	6	5757	ZINC000005758961
0.4	-7.7	-7.3	0.26	1.722	0.055	3.699	0.290	6	5758	ZINC000006031048
0.4	-8.2	-8.0	0.21	1.736	0.201	3.980	0.865	6	5759	ZINC000257385229
0.4	-7.6	-7.4	0.19	1.237	0.231	3.117	1.148	6	5760	ZINC000064634147
0.4	-10.0	-9.0	0.75	1.958	0.360	4.597	1.015	6	5761	ZINC000014763050
0.4	-6.8	-6.7	0.07	2.493	0.421	3.442	0.836	6	5762	ZINC000002516165
0.4	-8.7	-8.3	0.34	2.281	0.678	4.290	0.975	6	5763	ZINC000096828964
0.4	-8.5	-8.2	0.25	2.781	0.573	4.951	1.667	6	5764	ZINC000001678872
0.4	-5.4	-5.0	0.17	2.722	1.324	3.760	1.453	6	5765	ZINC000013351056
0.4	-6.5	-6.2	0.18	2.930	1.104	4.772	1.283	6	5766	ZINC000004521597
0.4	-7.7	-7.3	0.18	2.371	0.224	4.313	0.402	6	5767	ZINC000015122073
0.4	-7.0	-6.9	0.07	1.344	0.166	3.063	0.736	6	5768	ZINC000100019326

0.4	-5.3	-5.0	0.15	2.746	0.574	3.660	0.524	6	5769	ZINC000000409299
0.4	-5.4	-5.2	0.10	2.181	0.492	3.837	0.234	6	5770	ZINC000000167136
0.4	-8.7	-8.2	0.38	1.959	0.822	3.735	1.678	6	5771	ZINC000013352019
0.4	-9.4	-8.2	0.56	1.388	0.426	5.039	1.892	6	5772	ZINC000014642303
0.4	-7.2	-6.7	0.27	2.518	0.853	3.748	1.389	6	5773	ZINC000014504265
0.4	-8.3	-7.9	0.29	1.694	0.405	3.779	0.727	6	5774	ZINC000015112255
0.4	-9.1	-8.8	0.23	1.609	0.328	3.120	0.836	6	5775	ZINC000256430665
0.4	-9.5	-8.8	0.39	1.580	0.128	2.779	1.271	6	5776	ZINC000100257432
0.4	-8.3	-7.7	0.32	2.511	0.348	4.425	0.553	6	5777	ZINC000086032423
0.4	-8.0	-7.5	0.36	1.968	0.389	5.309	1.940	6	5778	ZINC000015259905
0.4	-9.0	-8.2	0.57	2.036	0.418	4.072	1.750	6	5779	ZINC000001581503
0.4	-7.3	-7.0	0.19	2.584	0.494	3.607	1.163	6	5780	ZINC000001592424
0.4	-10.6	-9.2	0.75	1.869	0.461	5.294	1.798	6	5781	ZINC000257383244
0.4	-5.5	-5.3	0.11	1.847	0.463	3.669	1.165	6	5782	ZINC000000394340
0.4	-6.9	-6.7	0.12	2.180	0.302	2.657	0.364	6	5783	ZINC000001530093

0.4	-6.5	-6.1	0.19	1.460	0.488	4.308	0.183	6	5784	ZINC000079566589
0.4	-5.4	-5.0	0.26	2.489	0.356	3.273	0.303	6	5785	ZINC000015119985
0.4	-6.5	-6.4	0.11	1.852	0.175	3.378	0.191	6	5786	ZINC000022923898
0.4	-8.7	-8.1	0.35	3.332	0.372	5.408	1.461	6	5787	ZINC000035051048
0.4	-4.8	-4.6	0.14	3.151	0.736	4.601	0.518	6	5788	ZINC000002510287
0.4	-6.5	-6.2	0.16	2.411	0.239	5.068	0.348	6	5789	ZINC000002572323
0.4	-8.2	-7.8	0.44	2.333	0.651	3.749	1.086	6	5790	ZINC000014448741
0.4	-9.1	-7.8	0.83	1.982	0.354	4.232	1.507	6	5791	ZINC000038723882
0.4	-8.2	-7.7	0.30	1.807	0.172	4.605	0.639	6	5792	ZINC000014611608
0.4	-9.3	-8.8	0.29	1.629	0.295	4.105	0.837	6	5793	ZINC000257382665
0.4	-8.3	-7.8	0.38	1.779	0.243	4.068	0.385	6	5794	ZINC000085872098
0.4	-5.6	-5.4	0.10	2.468	1.001	4.307	1.848	6	5795	ZINC000004096184
0.4	-7.3	-7.2	0.07	1.283	0.469	5.364	2.159	6	5796	ZINC000014860252
0.4	-8.7	-7.9	0.37	2.598	0.089	5.418	1.299	6	5797	ZINC000013517438
0.4	-7.7	-7.5	0.15	1.532	0.544	2.716	1.040	6	5798	ZINC000000968147

0.4	-8.5	-8.1	0.24	2.624	0.137	4.958	0.882	6	5799	ZINC000015261563
0.4	-9.8	-8.5	0.68	1.765	0.126	4.566	1.083	6	5800	ZINC000059774507
0.4	-8.2	-8.0	0.13	1.761	0.382	3.325	1.268	6	5801	ZINC000013306523
0.4	-7.7	-7.3	0.20	2.022	0.223	4.957	0.741	6	5802	ZINC000027638901
0.4	-8.1	-7.6	0.27	2.289	0.226	5.122	1.511	6	5803	ZINC000002507625
0.4	-7.9	-7.6	0.19	1.966	0.768	4.065	1.592	6	5804	ZINC000257485925
0.4	-7.2	-7.1	0.12	1.965	0.416	4.911	1.182	6	5805	ZINC000005819976
0.4	-8.7	-8.5	0.27	1.909	0.229	4.644	1.393	6	5806	ZINC000257487947
0.4	-9.0	-8.3	0.38	1.768	0.193	4.773	1.325	6	5807	ZINC000014920373
0.4	-6.0	-5.9	0.09	2.173	0.354	3.534	1.076	6	5808	ZINC000003137564
0.4	-6.9	-6.7	0.11	1.822	0.757	4.264	0.476	6	5809	ZINC000001587602
0.4	-7.5	-7.3	0.15	3.189	0.856	4.470	1.424	6	5810	ZINC000002001392
0.4	-6.4	-6.3	0.12	1.682	0.352	3.833	1.039	6	5811	ZINC000001627247
0.4	-9.1	-8.4	0.35	2.318	0.562	4.657	0.529	6	5812	ZINC000239274937
0.4	-7.7	-7.7	0.05	2.454	0.507	4.250	0.968	6	5813	ZINC000085798750

0.4	-7.1	-7.0	0.08	1.557	0.168	3.395	1.098	6	5814	ZINC000014487672
0.4	-9.1	-8.3	0.61	1.705	0.279	4.997	1.604	6	5815	ZINC000257561799
0.4	-7.6	-7.4	0.15	2.329	0.259	3.980	0.896	6	5816	ZINC000001605452
0.4	-6.6	-6.2	0.26	2.819	1.465	4.453	1.794	6	5817	ZINC000000074770
0.4	-8.0	-7.7	0.25	1.832	0.810	2.853	0.781	6	5818	ZINC000014721627
0.4	-7.4	-7.1	0.19	2.023	0.376	4.438	0.596	6	5819	ZINC000004726635
0.4	-7.7	-7.4	0.20	1.947	0.544	3.921	0.827	6	5820	ZINC000000057908
0.4	-4.7	-4.5	0.13	3.986	0.349	4.766	0.200	6	5821	ZINC000002034716
0.4	-6.8	-6.6	0.13	1.709	0.464	2.129	0.553	6	5822	ZINC000000158629
0.4	-8.0	-7.5	0.27	2.023	0.392	4.342	0.293	6	5823	ZINC000014854747
0.4	-6.1	-5.9	0.10	1.752	0.566	4.032	0.437	6	5824	ZINC000038789220
0.4	-6.5	-6.2	0.20	2.434	0.996	3.946	0.947	6	5825	ZINC000014439079
0.4	-6.4	-6.1	0.15	1.789	0.332	2.821	0.594	6	5826	ZINC000021999704
0.4	-9.0	-8.7	0.19	1.369	0.426	5.085	2.022	6	5827	ZINC000015251447
0.4	-4.9	-4.9	0.05	2.506	1.414	3.723	0.985	6	5828	ZINC000029786458

0.4	-8.8	-7.7	0.53	2.558	0.849	4.278	1.567	6	5829	ZINC000001713365
0.4	-6.5	-6.3	0.11	1.949	0.991	3.764	0.825	6	5830	ZINC000000900185
0.4	-6.5	-6.4	0.07	2.622	0.828	4.480	0.483	6	5831	ZINC000005457464
0.4	-9.6	-8.2	1.02	1.666	0.066	5.899	0.114	3	5832	ZINC000230076868
0.4	-8.8	-7.7	0.51	2.578	0.866	4.280	1.613	6	5833	ZINC000004096882
0.4	-7.0	-6.9	0.07	2.278	0.398	4.426	1.410	6	5834	ZINC000015261532
0.4	-6.4	-6.0	0.23	1.828	0.613	3.181	1.239	6	5835	ZINC000032166988
0.4	-6.4	-6.0	0.19	2.887	0.235	4.715	0.592	6	5836	ZINC000002522672
0.4	-6.0	-5.7	0.15	2.396	0.942	4.461	0.982	6	5837	ZINC000021999687
0.4	-8.5	-8.3	0.15	1.875	0.549	3.456	1.328	6	5838	ZINC000085589370
0.4	-5.6	-5.4	0.13	2.305	1.075	3.492	0.896	6	5839	ZINC000013306796
0.4	-9.3	-8.8	0.36	2.350	0.547	4.977	1.743	6	5840	ZINC000014807116
0.4	-8.2	-7.9	0.17	1.992	0.363	3.703	1.101	6	5841	ZINC000004096381
0.4	-9.4	-7.9	0.76	1.994	0.373	5.349	1.421	6	5842	ZINC000004521569
0.4	-6.9	-6.5	0.23	2.340	0.676	3.426	1.089	6	5843	ZINC000000968248

0.4	-9.8	-8.9	0.48	1.548	0.111	5.278	0.947	6	5844	ZINC000015222342
0.4	-9.1	-8.5	0.36	2.246	0.390	4.563	0.850	6	5845	ZINC000015153336
0.4	-7.0	-6.7	0.16	3.050	0.665	3.868	0.887	6	5846	ZINC000000895711
0.4	-6.4	-6.2	0.14	2.338	0.895	4.307	0.735	6	5847	ZINC000012493608
0.4	-8.3	-7.6	0.39	1.865	0.126	4.732	1.368	6	5848	ZINC000012496087
0.4	-8.9	-8.0	0.50	1.823	0.160	3.048	0.255	6	5849	ZINC000014682736
0.4	-7.1	-6.9	0.16	1.850	0.467	3.375	0.851	6	5850	ZINC000014822384
0.4	-9.9	-9.5	0.24	1.524	0.469	3.328	0.893	6	5851	ZINC000100829373
0.4	-6.6	-6.5	0.05	1.778	0.212	3.700	0.353	6	5852	ZINC000014722255
0.4	-8.4	-8.2	0.18	1.433	0.608	2.160	0.320	6	5853	ZINC000014491830
0.4	-7.5	-7.2	0.24	1.678	0.255	5.025	1.593	6	5854	ZINC000085589376
0.4	-5.0	-4.9	0.07	1.594	0.151	2.956	0.612	6	5855	ZINC000002034630
0.4	-7.1	-6.7	0.26	2.245	0.357	4.514	1.230	6	5856	ZINC000015213109
0.4	-8.3	-8.0	0.17	1.464	0.678	4.099	2.012	6	5857	ZINC000257559218
0.4	-7.6	-7.4	0.19	2.072	0.500	3.874	1.104	6	5858	ZINC000149827764

0.4	-6.7	-6.6	0.21	1.716	1.004	2.579	1.001	6	5859	ZINC000002040153
0.4	-8.7	-8.3	0.26	1.658	0.891	3.464	0.984	6	5860	ZINC000104373812
0.4	-7.7	-7.4	0.25	2.226	0.182	3.890	0.288	6	5861	ZINC000210988066
0.4	-5.1	-5.0	0.07	2.810	0.496	3.575	0.665	6	5862	ZINC000095620441
0.4	-5.6	-5.4	0.12	2.224	0.615	3.405	0.665	6	5863	ZINC000008294957
0.4	-8.6	-8.0	0.38	1.925	0.190	4.823	1.707	6	5864	ZINC000014916934
0.4	-7.5	-7.2	0.18	2.607	0.435	4.288	0.741	6	5865	ZINC000015119193
0.4	-7.0	-6.8	0.17	2.309	0.587	3.583	1.457	6	5866	ZINC000015261370
0.4	-6.4	-6.1	0.15	2.315	0.792	3.707	0.667	6	5867	ZINC000001850742
0.4	-7.6	-7.3	0.17	1.701	0.359	3.710	1.771	6	5868	ZINC000013377373
0.4	-5.6	-5.2	0.16	2.118	0.211	3.568	0.707	6	5869	ZINC000005838977
0.4	-7.6	-7.4	0.13	1.934	0.346	3.487	0.688	6	5870	ZINC000012494123
0.4	-9.0	-8.7	0.28	2.316	0.418	4.023	1.524	6	5871	ZINC000050027339
0.4	-5.5	-5.4	0.08	2.214	0.396	4.143	0.615	6	5872	ZINC000100004207
0.4	-4.6	-4.5	0.14	1.685	0.519	2.516	1.013	6	5873	ZINC000032211379

0.4	-6.5	-6.3	0.14	1.835	0.422	2.951	0.797	6	5874	ZINC000000120430
0.4	-5.7	-5.3	0.20	2.226	1.253	3.715	1.053	6	5875	ZINC000095617997
0.4	-8.1	-7.8	0.18	2.213	0.941	3.085	1.260	6	5876	ZINC000001532617
0.4	-5.8	-5.4	0.25	2.206	0.808	3.890	0.758	6	5877	ZINC000002041115
0.4	-6.2	-5.9	0.14	2.653	0.775	4.099	1.286	6	5878	ZINC000095618191
0.4	-6.4	-6.3	0.11	2.025	0.682	3.053	0.402	6	5879	ZINC000000331671
0.4	-8.8	-8.5	0.27	1.286	0.646	4.871	2.164	6	5880	ZINC000015251502
0.4	-8.8	-8.5	0.25	1.375	0.710	4.920	2.129	6	5881	ZINC000015251506
0.4	-9.1	-8.7	0.31	1.638	0.275	5.261	1.069	6	5882	ZINC000085887478
0.4	-6.3	-6.2	0.13	2.242	1.359	2.988	1.032	6	5883	ZINC000000152996
0.4	-6.7	-6.6	0.07	1.540	0.664	2.980	0.703	6	5884	ZINC000005157392
0.4	-6.3	-6.1	0.15	2.069	0.638	3.126	0.888	6	5885	ZINC000069485976
0.4	-6.4	-6.1	0.18	3.579	0.692	5.111	1.155	6	5886	ZINC000034746386
0.4	-8.9	-7.2	0.86	1.618	0.125	4.474	0.450	6	5887	ZINC000257511803
0.4	-4.3	-4.2	0.08	3.080	1.382	4.031	1.125	6	5888	ZINC000006031279

0.4	-5.2	-5.0	0.13	1.605	0.488	2.590	0.709	6	5889	ZINC000015119986
0.4	-7.2	-6.8	0.27	2.184	0.199	4.074	0.656	6	5890	ZINC000087492869
0.4	-6.3	-6.0	0.20	0.861	0.423	3.850	1.098	6	5891	ZINC000003581021
0.4	-9.3	-8.7	0.34	1.869	0.561	4.153	0.816	6	5892	ZINC000100782063
0.4	-4.4	-4.2	0.12	1.970	1.143	3.368	0.622	6	5893	ZINC000000150863
0.4	-7.0	-6.7	0.13	3.455	0.160	4.811	0.314	6	5894	ZINC000033963312
0.4	-7.0	-6.7	0.16	1.700	0.766	3.197	1.690	6	5895	ZINC000035307742
0.4	-5.7	-5.6	0.04	2.689	0.550	3.765	0.776	6	5896	ZINC000000157016
0.4	-6.1	-5.6	0.34	2.706	0.644	4.018	1.002	6	5897	ZINC000095620569
0.4	-7.1	-6.8	0.21	2.142	0.198	5.115	1.539	6	5898	ZINC000002242694
0.4	-6.6	-6.4	0.20	1.875	1.041	2.803	0.950	6	5899	ZINC000001577074
0.4	-7.8	-7.3	0.31	2.042	0.469	3.173	0.925	6	5900	ZINC000169418208
0.4	-7.1	-6.8	0.32	2.236	0.127	3.011	0.144	6	5901	ZINC000002384788
0.4	-9.7	-8.4	1.06	2.035	0.569	4.535	1.535	6	5902	ZINC000013674168
0.4	-7.5	-7.1	0.35	1.945	0.148	5.439	0.690	6	5903	ZINC000012496280

0.4	-6.2	-6.1	0.07	2.372	0.433	3.782	1.273	6	5904	ZINC000001601797
0.4	-6.7	-6.5	0.07	2.178	0.340	2.789	0.473	6	5905	ZINC000003913405
0.4	-4.8	-4.7	0.12	2.253	0.717	2.964	0.972	6	5906	ZINC000014590710
0.4	-4.4	-4.2	0.17	1.546	0.445	3.484	0.825	6	5907	ZINC000000404268
0.4	-5.9	-5.8	0.07	2.351	0.930	4.923	1.177	6	5908	ZINC000001638429
0.4	-8.7	-8.5	0.13	2.738	0.670	4.569	0.656	6	5909	ZINC000014824069
0.4	-5.9	-5.7	0.17	2.436	0.227	3.610	0.672	6	5910	ZINC000003801919
0.4	-6.6	-6.3	0.18	2.129	0.304	3.391	0.321	6	5911	ZINC000001850935
0.4	-7.5	-7.2	0.17	1.539	0.407	3.169	0.233	6	5912	ZINC000001081109
0.4	-8.7	-8.4	0.14	1.999	0.213	5.000	1.026	6	5913	ZINC000013307639
0.4	-6.4	-6.2	0.16	2.750	0.611	3.112	0.591	6	5914	ZINC000053194795
0.4	-7.4	-6.8	0.35	3.029	0.253	5.340	0.304	6	5915	ZINC000002391099
0.4	-8.1	-7.3	0.45	1.859	0.249	5.112	0.646	6	5916	ZINC000012496274
0.4	-9.0	-8.3	0.53	2.099	0.584	5.810	1.023	6	5917	ZINC000000901752
0.4	-6.2	-6.0	0.12	2.815	0.887	4.250	0.803	6	5918	ZINC000004234800

0.4	-10.2	-9.6	0.37	1.382	0.280	4.177	1.022	6	5919	ZINC000257433569
0.4	-10.5	-9.8	0.45	2.060	1.529	3.805	1.612	6	5920	ZINC000253633074
0.4	-5.9	-5.6	0.16	2.706	0.922	4.261	0.883	6	5921	ZINC000012153710
0.4	-5.5	-5.2	0.24	2.275	1.939	4.088	1.410	6	5922	ZINC000000388410
0.4	-9.0	-8.5	0.33	1.359	0.150	4.033	2.489	6	5923	ZINC000014811610
0.4	-9.6	-8.9	0.46	1.730	0.242	3.532	0.767	6	5924	ZINC000014616912
0.4	-9.8	-9.0	0.39	2.218	0.823	4.195	1.095	6	5925	ZINC000100257438
0.4	-8.0	-7.6	0.33	2.034	0.462	2.569	0.603	6	5926	ZINC000005392428
0.4	-8.7	-7.8	0.66	2.165	0.203	4.277	0.651	6	5927	ZINC000257390694
0.4	-9.0	-8.7	0.21	0.869	0.610	4.114	2.209	6	5928	ZINC000015251439
0.4	-8.5	-8.2	0.23	1.048	0.682	3.390	1.965	6	5929	ZINC000257444827
0.4	-6.6	-6.3	0.23	2.663	0.966	3.902	1.438	6	5930	ZINC000015113207
0.4	-7.8	-7.5	0.24	1.731	0.532	3.804	1.357	6	5931	ZINC000013481841
0.4	-6.2	-6.0	0.13	2.585	0.569	3.646	0.853	6	5932	ZINC000000164581
0.4	-6.6	-6.4	0.14	2.609	0.634	3.953	1.344	6	5933	ZINC000002047359

0.4	-8.9	-8.5	0.29	2.225	0.601	3.525	0.921	6	5934	ZINC000050027637
0.4	-6.6	-6.2	0.19	1.697	0.496	2.988	0.923	6	5935	ZINC000000001507
0.4	-5.9	-5.7	0.13	2.893	0.724	4.417	0.343	6	5936	ZINC000002510293
0.4	-6.8	-6.4	0.23	3.033	0.838	4.249	1.143	6	5937	ZINC000014488962
0.4	-10.5	-9.8	0.51	2.289	1.478	4.045	1.419	6	5938	ZINC000100091866
0.4	-6.1	-5.8	0.18	3.656	0.224	4.565	0.541	6	5939	ZINC000002516010
0.4	-8.0	-7.6	0.31	2.073	0.496	4.431	1.694	6	5940	ZINC000100385307
0.4	-5.4	-5.1	0.21	1.562	0.202	2.440	0.157	6	5941	ZINC000001532776
0.4	-7.2	-6.9	0.20	2.519	0.545	4.553	1.156	6	5942	ZINC000008437013
0.4	-6.8	-6.4	0.26	2.084	0.605	3.125	1.073	6	5943	ZINC000000968247
0.4	-7.0	-6.9	0.09	1.630	0.466	5.296	0.729	6	5944	ZINC000014454646
0.4	-7.4	-7.1	0.23	2.087	0.657	2.913	1.018	6	5945	ZINC000030724120
0.4	-6.0	-5.7	0.14	2.305	0.467	3.042	0.449	6	5946	ZINC000100002478
0.4	-6.9	-6.4	0.27	2.100	0.531	4.211	1.595	6	5947	ZINC000004899467
0.4	-7.1	-6.8	0.18	2.104	0.506	2.829	0.762	6	5948	ZINC000015261401

0.4	-8.1	-7.7	0.25	2.046	0.389	3.692	0.882	6	5949	ZINC000013371333
0.4	-8.4	-8.0	0.32	2.240	0.328	4.036	0.435	6	5950	ZINC000015265060
0.4	-6.6	-6.3	0.16	2.451	1.120	3.887	1.802	6	5951	ZINC000004556685
0.4	-7.3	-7.0	0.18	2.546	0.444	5.118	0.441	6	5952	ZINC000014439176
0.4	-6.3	-6.0	0.27	2.155	0.901	3.337	1.816	6	5953	ZINC000005511592
0.4	-9.8	-9.0	0.36	2.011	0.976	3.460	1.443	6	5954	ZINC000257530569
0.4	-7.7	-7.4	0.30	2.197	0.604	3.108	0.404	6	5955	ZINC000000287668
0.4	-4.7	-4.4	0.20	2.013	0.376	3.186	0.654	6	5956	ZINC000038611803
0.4	-9.1	-8.5	0.49	1.966	0.430	4.081	1.304	6	5957	ZINC000014450730
0.4	-5.8	-5.5	0.21	2.324	0.969	3.732	0.895	6	5958	ZINC000003634659
0.4	-9.9	-8.1	0.99	1.828	0.426	4.872	1.340	5	5959	ZINC000257405860
0.4	-8.3	-7.8	0.26	1.627	0.364	3.785	0.850	6	5960	ZINC000006069162
0.4	-7.0	-6.9	0.07	2.408	0.256	4.504	0.813	6	5961	ZINC000002564746
0.4	-7.6	-7.2	0.23	2.086	0.719	3.352	1.659	6	5962	ZINC000004095646
0.4	-6.0	-5.9	0.09	1.961	0.549	3.796	1.203	6	5963	ZINC000261499723

0.4	-6.3	-6.0	0.21	1.714	0.778	4.636	0.710	6	5964	ZINC000001549593
0.4	-6.3	-6.1	0.10	1.523	0.217	3.751	0.811	6	5965	ZINC000085972338
0.4	-7.4	-7.2	0.17	2.664	0.241	3.563	1.060	6	5966	ZINC000040470271
0.4	-9.6	-9.0	0.58	1.641	0.092	5.465	1.089	5	5967	ZINC000002029395
0.4	-8.4	-8.2	0.09	1.841	0.202	4.947	0.624	6	5968	ZINC000014587265
0.4	-8.0	-7.6	0.25	3.493	0.243	4.785	0.315	6	5969	ZINC000001728129
0.4	-9.0	-8.9	0.05	1.570	0.143	2.487	0.684	6	5970	ZINC000257377282
0.4	-8.4	-8.2	0.14	2.394	0.503	4.849	1.442	6	5971	ZINC000027644820
0.4	-7.4	-7.0	0.20	2.267	0.672	5.090	0.943	6	5972	ZINC000004214727
0.4	-5.6	-5.5	0.07	2.737	1.580	4.274	1.442	6	5973	ZINC000261495746
0.4	-7.4	-7.3	0.16	1.771	0.539	3.495	1.391	6	5974	ZINC000261496281
0.4	-6.7	-6.6	0.11	2.297	0.527	3.235	0.746	6	5975	ZINC000001095311
0.4	-6.2	-6.1	0.09	1.792	0.558	2.883	0.431	6	5976	ZINC000000896726
0.4	-6.3	-6.1	0.16	2.896	0.268	3.724	0.316	6	5977	ZINC000000156868
0.4	-8.0	-7.8	0.13	1.794	0.436	3.699	0.737	6	5978	ZINC000003472623

0.4	-6.6	-6.3	0.16	2.480	0.619	4.244	1.317	6	5979	ZINC000095617789
0.4	-6.6	-6.5	0.11	2.784	0.633	3.862	1.124	6	5980	ZINC000014455500
0.4	-6.9	-6.4	0.28	2.028	0.434	3.729	0.769	6	5981	ZINC000013328184
0.4	-8.6	-8.3	0.21	1.674	0.388	3.233	1.926	6	5982	ZINC000261499673
0.4	-8.3	-8.2	0.10	1.304	0.548	3.085	1.890	6	5983	ZINC000015249553
0.4	-7.3	-7.0	0.20	1.810	0.262	3.387	0.941	6	5984	ZINC000257444147
0.4	-7.2	-6.9	0.15	2.462	0.559	4.114	1.422	6	5985	ZINC000004097103
0.4	-6.2	-6.0	0.13	2.769	1.176	3.957	1.364	6	5986	ZINC000005820846
0.4	-9.9	-8.4	0.94	1.799	0.427	4.448	1.778	5	5987	ZINC000001711825
0.4	-7.6	-7.4	0.18	2.081	0.509	4.532	0.711	6	5988	ZINC000012494841
0.4	-6.3	-6.2	0.08	1.877	0.665	2.646	0.526	6	5989	ZINC000001680785
0.4	-6.8	-6.3	0.23	2.251	0.508	3.732	1.572	6	5990	ZINC000095618076
0.4	-8.5	-8.3	0.15	1.551	0.186	4.029	2.171	6	5991	ZINC000230097216
0.4	-5.8	-5.4	0.24	3.207	0.450	4.095	0.306	6	5992	ZINC000014614334
0.4	-6.3	-6.1	0.15	2.008	0.527	3.757	0.868	6	5993	ZINC000013515547

0.4	-9.1	-8.8	0.19	1.634	0.285	4.531	1.842	6	5994	ZINC000085887484
0.4	-7.4	-6.9	0.32	1.802	0.440	2.326	0.667	6	5995	ZINC000000012614
0.4	-6.3	-6.1	0.13	2.281	0.793	3.046	0.583	6	5996	ZINC000003874615
0.4	-7.2	-6.7	0.26	2.278	0.329	3.799	0.583	6	5997	ZINC000005759086
0.4	-9.0	-8.4	0.47	1.237	0.608	4.627	2.316	6	5998	ZINC000085795087
0.4	-5.4	-5.2	0.08	3.664	1.772	4.437	1.473	6	5999	ZINC000000406999
0.4	-6.7	-6.6	0.07	2.155	0.225	2.790	0.297	6	6000	ZINC000002568071
0.4	-5.7	-5.4	0.21	2.113	0.346	3.430	0.956	6	6001	ZINC000005752097
0.4	-6.0	-5.6	0.26	2.684	1.383	3.326	1.535	6	6002	ZINC000013437575
0.4	-5.2	-4.9	0.17	3.151	1.139	4.049	1.198	6	6003	ZINC000002509971
0.4	-8.9	-8.5	0.32	1.356	0.452	5.005	2.298	6	6004	ZINC000014436341
0.4	-10.0	-8.9	0.71	1.785	0.739	4.172	1.631	6	6005	ZINC000002019692
0.4	-6.7	-6.5	0.13	3.053	0.772	4.834	0.904	6	6006	ZINC000095617772
0.4	-6.7	-6.4	0.17	2.725	0.559	3.265	0.480	6	6007	ZINC000008801890
0.4	-9.1	-8.0	0.80	1.842	0.325	5.044	2.044	6	6008	ZINC000257463481

0.4	-8.4	-8.4	0.08	1.563	0.311	3.521	1.082	6	6009	ZINC000015249358
0.4	-6.4	-6.3	0.12	2.622	0.377	4.155	0.504	6	6010	ZINC000015120477
0.4	-7.4	-7.2	0.14	2.162	0.370	4.217	1.061	6	6011	ZINC000003869471
0.4	-6.0	-5.8	0.14	5.225	0.618	6.592	0.444	6	6012	ZINC000003634656
0.4	-10.1	-8.3	0.88	1.701	0.385	5.140	1.586	6	6013	ZINC000257424795
0.4	-4.6	-4.5	0.05	1.754	0.267	2.818	0.395	6	6014	ZINC000031500674
0.4	-8.0	-7.8	0.19	1.833	0.441	5.753	1.624	6	6015	ZINC000033650331
0.4	-6.3	-6.0	0.13	2.932	0.662	4.380	1.061	6	6016	ZINC000038251273
0.4	-6.4	-6.2	0.17	2.209	1.340	3.204	1.408	6	6017	ZINC000000388739
0.4	-8.9	-7.7	0.58	2.162	0.410	3.755	1.044	6	6018	ZINC000013408961
0.4	-7.1	-6.8	0.20	1.752	0.415	2.843	1.094	6	6019	ZINC000014487966
0.4	-5.8	-5.7	0.12	3.601	1.038	4.177	0.961	6	6020	ZINC000026895132
0.4	-7.4	-7.2	0.13	2.101	0.557	4.319	1.064	6	6021	ZINC000004097549
0.4	-9.4	-8.7	0.54	1.763	0.638	3.911	2.146	6	6022	ZINC000014758887
0.4	-7.2	-6.9	0.20	1.742	0.403	3.095	1.225	6	6023	ZINC000005766919

0.4	-7.7	-7.2	0.33	2.159	0.268	4.139	0.402	6	6024	ZINC000001584052
0.4	-6.6	-6.3	0.18	5.534	0.322	6.718	0.237	6	6025	ZINC000000895129
0.4	-9.0	-8.3	0.34	1.555	0.334	2.970	0.632	6	6026	ZINC000012341953
0.4	-5.8	-5.7	0.11	1.583	1.005	3.095	1.116	6	6027	ZINC000000388234
0.4	-6.5	-6.3	0.19	2.576	1.233	4.765	1.077	6	6028	ZINC000032163571
0.4	-6.2	-5.8	0.21	1.822	0.418	3.984	0.960	6	6029	ZINC000032166990
0.4	-8.0	-7.4	0.32	2.218	0.775	4.323	1.920	6	6030	ZINC000013377210
0.4	-7.0	-6.7	0.20	2.957	0.836	3.600	0.817	6	6031	ZINC000000105196
0.4	-8.7	-8.4	0.21	1.748	0.830	3.646	0.946	6	6032	ZINC000100825895
0.4	-8.9	-8.1	0.40	1.725	0.274	3.307	1.075	6	6033	ZINC000015153344
0.4	-5.3	-5.0	0.19	2.912	0.822	3.305	0.883	6	6034	ZINC000002031533
0.4	-6.7	-6.5	0.14	2.435	0.944	4.395	1.216	6	6035	ZINC000012493847
0.4	-8.4	-7.5	0.56	2.686	0.313	4.904	1.086	6	6036	ZINC000031356994
0.4	-6.7	-6.5	0.15	2.833	0.238	4.678	0.418	6	6037	ZINC000013375685
0.4	-6.1	-5.7	0.26	2.648	1.171	3.920	1.227	6	6038	ZINC000002039412

0.4	-8.8	-8.5	0.22	1.263	0.663	4.867	2.156	6	6039	ZINC000015251514
0.4	-4.9	-4.9	0.08	2.758	1.727	4.031	1.008	6	6040	ZINC000006031817
0.4	-5.9	-5.5	0.20	1.480	0.237	3.519	1.131	6	6041	ZINC000095618176
0.4	-7.2	-7.0	0.12	1.835	0.443	3.514	1.243	6	6042	ZINC000100776357
0.4	-9.2	-8.8	0.32	1.674	0.410	3.245	1.396	6	6043	ZINC000100783840
0.4	-9.7	-8.7	0.73	1.720	0.466	3.682	1.739	6	6044	ZINC000013484972
0.4	-8.6	-6.7	1.15	1.711	0.085	4.280	1.352	4	6045	ZINC000013399024
0.4	-7.5	-7.3	0.12	1.991	0.292	3.951	1.282	6	6046	ZINC000014556659
0.4	-6.3	-5.9	0.20	2.731	0.974	4.082	1.495	6	6047	ZINC000002039298
0.4	-5.9	-5.5	0.25	2.175	0.763	3.304	0.949	6	6048	ZINC000032167539
0.4	-8.5	-7.5	0.71	2.356	0.630	5.170	1.402	6	6049	ZINC000014820394
0.4	-8.0	-7.6	0.26	1.461	0.342	2.484	0.383	6	6050	ZINC000100778148
0.4	-4.7	-4.6	0.09	1.371	0.506	3.545	1.291	6	6051	ZINC000001586752
0.4	-8.2	-7.9	0.24	1.665	0.822	3.860	2.328	6	6052	ZINC000034309947
0.4	-4.6	-4.4	0.15	2.447	0.468	2.890	0.579	6	6053	ZINC000032175839

0.4	-8.1	-7.8	0.30	2.013	0.130	5.813	0.463	6	6054	ZINC000012496228
0.4	-5.2	-4.9	0.21	2.037	0.773	3.145	1.137	6	6055	ZINC000014500541
0.4	-6.2	-5.9	0.16	3.683	0.833	4.834	1.180	6	6056	ZINC000013413608
0.4	-5.7	-5.6	0.07	1.683	0.704	2.924	1.242	6	6057	ZINC000001850662
0.4	-6.5	-6.2	0.15	1.879	0.267	5.780	1.801	6	6058	ZINC000019364680
0.4	-6.5	-6.2	0.17	1.609	0.220	2.917	1.561	6	6059	ZINC000257453814
0.4	-6.5	-6.2	0.16	3.321	0.426	4.426	0.359	6	6060	ZINC000003869291
0.4	-5.3	-5.0	0.13	4.271	1.309	5.673	1.187	6	6061	ZINC000003869934
0.4	-5.7	-5.5	0.10	3.256	0.847	4.557	0.790	6	6062	ZINC000001569422
0.4	-7.5	-7.3	0.09	2.581	1.057	3.982	1.929	6	6063	ZINC000005127809
0.4	-9.3	-7.6	0.93	1.656	0.271	4.969	1.498	6	6064	ZINC000005764434
0.4	-8.5	-8.3	0.12	1.581	0.601	3.339	1.424	6	6065	ZINC000015120457
0.4	-8.1	-7.9	0.18	2.713	0.670	4.363	1.514	6	6066	ZINC000017176123
0.4	-9.0	-8.8	0.12	1.107	0.698	4.085	2.159	6	6067	ZINC000257563698
0.4	-7.1	-6.9	0.10	2.322	0.380	4.398	0.356	6	6068	ZINC000004533736

0.4	-6.7	-6.6	0.07	2.162	0.886	3.026	1.545	6	6069	ZINC000004753654
0.4	-9.2	-8.6	0.32	1.839	0.406	3.108	0.979	6	6070	ZINC000014593850
0.4	-8.9	-8.1	0.45	1.648	0.270	3.273	1.747	6	6071	ZINC000014758997
0.4	-5.5	-5.1	0.39	1.796	0.778	2.540	0.979	6	6072	ZINC000000407031
0.4	-5.4	-5.1	0.16	2.211	0.607	4.093	0.457	6	6073	ZINC000014590584
0.4	-5.3	-5.0	0.27	2.508	0.772	3.407	0.801	6	6074	ZINC000005225126
0.4	-4.8	-4.7	0.04	1.453	0.277	3.237	0.446	6	6075	ZINC000001699551
0.4	-8.6	-8.1	0.46	2.594	0.382	4.914	1.932	6	6076	ZINC000003875126
0.4	-7.0	-6.9	0.10	1.997	0.593	3.244	0.621	6	6077	ZINC000100779751
0.4	-9.0	-7.7	0.71	1.608	0.192	4.931	1.099	5	6078	ZINC000100779920
0.4	-8.9	-8.1	0.56	2.119	0.349	4.964	1.614	6	6079	ZINC000014728222
0.4	-7.0	-6.7	0.24	2.086	0.888	4.573	0.888	6	6080	ZINC000005761880
0.4	-8.6	-8.2	0.30	1.947	0.366	3.906	1.274	6	6081	ZINC000100772272
0.4	-8.4	-8.2	0.15	1.554	0.779	3.915	1.584	6	6082	ZINC000031157863
0.4	-9.2	-8.4	0.56	1.731	0.166	3.840	0.876	6	6083	ZINC000014820405

0.4	-6.3	-5.9	0.20	2.417	0.562	3.148	0.658	6	6084	ZINC000000968069
0.4	-9.1	-8.8	0.17	1.614	0.262	4.517	1.824	6	6085	ZINC000257480156
0.4	-6.6	-6.2	0.24	2.785	0.234	4.034	0.743	6	6086	ZINC000004557160
0.4	-6.0	-5.8	0.12	2.146	0.431	3.445	0.682	6	6087	ZINC000001529894
0.4	-6.4	-6.3	0.09	2.077	0.513	3.322	1.134	6	6088	ZINC000001532704
0.4	-5.4	-5.3	0.09	3.223	1.332	4.773	1.172	6	6089	ZINC000003869279
0.4	-5.6	-5.5	0.08	2.172	0.326	3.512	0.380	6	6090	ZINC000030731335
0.4	-7.4	-7.1	0.19	2.645	0.416	4.200	1.237	6	6091	ZINC000040470270
0.4	-6.7	-6.4	0.16	2.262	0.683	3.550	1.193	6	6092	ZINC000002146885
0.4	-6.1	-5.8	0.19	2.481	1.117	3.186	1.275	6	6093	ZINC000001677781
0.4	-8.6	-7.9	0.47	2.125	1.119	4.148	1.627	6	6094	ZINC000100778205
0.4	-7.9	-7.3	0.49	2.180	0.367	3.287	1.282	6	6095	ZINC000014713574
0.4	-7.0	-6.6	0.18	2.824	0.373	5.179	0.164	6	6096	ZINC000050027558
0.4	-4.3	-4.2	0.08	3.008	1.614	4.064	1.019	6	6097	ZINC000031274102
0.4	-5.6	-5.4	0.13	1.415	0.048	2.598	0.984	6	6098	ZINC000002522787

0.4	-9.2	-8.2	1.02	1.885	0.807	4.012	2.315	6	6099	ZINC000005650642
0.4	-8.5	-8.0	0.34	1.855	0.179	4.598	1.616	6	6100	ZINC000257402425
0.4	-7.6	-7.2	0.29	2.035	0.497	2.824	0.839	6	6101	ZINC000013538219
0.4	-6.9	-6.7	0.19	2.272	0.461	3.598	1.153	6	6102	ZINC000015261525
0.4	-6.2	-6.0	0.20	2.156	0.515	4.410	0.473	6	6103	ZINC000005751230
0.4	-9.5	-8.8	0.37	1.471	0.266	2.724	1.322	6	6104	ZINC000100257427
0.4	-4.7	-4.6	0.06	2.955	1.285	4.023	1.141	6	6105	ZINC000001562141
0.4	-6.8	-6.7	0.08	1.916	0.329	3.197	1.758	6	6106	ZINC000015122308
0.4	-6.2	-5.9	0.18	2.359	0.433	3.740	0.875	6	6107	ZINC000212675572
0.4	-5.1	-4.9	0.12	3.650	0.473	4.314	0.517	6	6108	ZINC000014592829
0.4	-4.9	-4.9	0.05	1.811	0.481	3.536	0.754	6	6109	ZINC000004261993
0.4	-6.7	-6.5	0.12	2.022	0.411	4.128	1.212	6	6110	ZINC000004097502
0.4	-5.3	-5.2	0.04	2.869	1.298	4.038	1.160	6	6111	ZINC000003860287
0.4	-4.3	-4.1	0.10	3.137	0.766	4.353	0.968	6	6112	ZINC000014679957
0.4	-7.1	-6.8	0.18	3.028	0.555	4.100	0.630	6	6113	ZINC000000410089

0.4	-6.5	-6.2	0.16	2.061	0.439	3.005	0.458	6	6114	ZINC000032175869
0.4	-6.5	-6.2	0.15	3.021	0.620	4.410	0.919	6	6115	ZINC000014504601
0.4	-8.7	-8.4	0.21	1.885	0.763	3.773	0.889	6	6116	ZINC000100825896
0.4	-7.3	-7.0	0.22	2.210	0.283	3.249	0.649	6	6117	ZINC000085808322
0.4	-5.7	-5.5	0.14	2.122	0.204	3.012	0.523	6	6118	ZINC000000156526
0.4	-7.5	-7.2	0.20	1.748	0.336	3.026	0.295	6	6119	ZINC000000388442
0.4	-5.1	-5.1	0.00	3.333	0.742	4.348	0.724	6	6120	ZINC000002575506
0.4	-8.8	-7.7	0.53	2.414	0.752	3.795	1.365	6	6121	ZINC000004096880
0.4	-7.0	-6.7	0.17	2.401	0.453	4.187	0.648	6	6122	ZINC000002024479
0.4	-7.4	-7.2	0.16	1.907	0.226	3.593	0.330	6	6123	ZINC000002557911
0.4	-7.5	-7.3	0.12	2.156	0.886	3.784	1.086	6	6124	ZINC000014517111
0.4	-5.8	-5.7	0.11	3.165	0.795	4.108	0.718	6	6125	ZINC000005761690
0.4	-10.4	-10.3	0.15	1.650	0.258	3.907	2.081	6	6126	ZINC000100778032
0.4	-7.8	-7.2	0.33	1.915	0.187	5.757	1.089	6	6127	ZINC000013303575
0.4	-9.0	-8.6	0.20	2.011	0.234	3.388	1.569	6	6128	ZINC000257546827

0.4	-8.9	-7.7	1.01	1.698	0.361	3.979	2.045	6	6129	ZINC000230076120
0.4	-6.5	-6.1	0.20	2.423	0.132	3.099	0.347	6	6130	ZINC000000388462
0.4	-6.7	-6.5	0.09	2.713	0.349	4.036	0.367	6	6131	ZINC000033992255
0.4	-6.4	-6.2	0.13	2.665	0.597	4.423	0.924	6	6132	ZINC000002390920
0.4	-6.2	-5.9	0.16	3.052	0.747	4.440	0.569	6	6133	ZINC000001529832
0.4	-7.5	-7.3	0.16	3.044	0.071	4.190	0.224	6	6134	ZINC000015121871
0.4	-5.4	-5.2	0.21	2.381	0.984	3.345	1.142	6	6135	ZINC000001849641
0.4	-7.1	-6.9	0.15	2.845	0.296	4.543	0.628	6	6136	ZINC000004899448
0.4	-5.6	-5.2	0.22	1.978	0.900	2.894	0.928	6	6137	ZINC000002575201
0.4	-4.6	-4.5	0.08	1.375	0.785	3.408	0.953	6	6138	ZINC000001691363
0.4	-10.2	-9.2	0.52	1.747	0.489	3.637	0.767	6	6139	ZINC000100051411
0.4	-7.9	-7.6	0.20	2.123	0.407	3.933	0.583	6	6140	ZINC000013377374
0.4	-7.6	-7.4	0.15	2.120	0.092	3.837	0.472	6	6141	ZINC000095617661
0.4	-8.8	-7.7	0.68	2.579	0.619	4.244	1.818	6	6142	ZINC000100825568
0.4	-5.9	-5.6	0.15	1.624	0.696	3.469	0.790	6	6143	ZINC000002038278

0.4	-6.3	-6.2	0.11	1.989	0.400	4.519	0.269	6	6144	ZINC000001680438
0.4	-9.0	-8.3	0.36	2.067	0.489	4.388	1.198	6	6145	ZINC000034046036
0.4	-7.9	-7.7	0.19	1.590	0.239	3.105	0.504	6	6146	ZINC000005766879
0.4	-6.0	-5.7	0.29	2.710	0.668	3.587	0.964	6	6147	ZINC000000001082
0.4	-3.9	-3.5	0.18	5.957	2.108	6.435	2.079	6	6148	ZINC000001699948
0.4	-8.8	-8.5	0.21	1.262	0.665	4.875	2.170	6	6149	ZINC000257417247
0.4	-6.4	-6.3	0.13	1.768	0.308	3.554	0.322	6	6150	ZINC000008418983
0.4	-10.1	-8.5	0.87	1.707	0.338	4.625	1.297	6	6151	ZINC000001557163
0.4	-8.5	-8.1	0.25	1.823	0.154	5.222	1.754	6	6152	ZINC000257402423
0.4	-7.9	-7.5	0.22	1.887	0.420	3.505	1.652	6	6153	ZINC000257485456
0.4	-8.7	-7.9	0.56	2.045	0.335	4.302	1.339	6	6154	ZINC000100781024
0.4	-4.8	-4.7	0.05	2.195	1.764	3.580	0.935	6	6155	ZINC000000404377
0.4	-7.5	-7.3	0.13	1.753	0.383	3.680	1.057	6	6156	ZINC000014771113
0.4	-8.0	-7.3	0.40	2.103	0.232	5.561	0.801	6	6157	ZINC000100829938
0.4	-6.0	-5.9	0.07	2.698	0.519	4.242	0.563	6	6158	ZINC000050027416

0.4	-7.6	-7.4	0.19	2.678	0.666	5.085	0.643	6	6159	ZINC000050027590
0.4	-5.3	-5.0	0.21	2.140	0.500	2.944	0.868	6	6160	ZINC000034743443
0.4	-7.2	-7.0	0.13	2.196	0.594	4.524	0.938	6	6161	ZINC000001850863
0.4	-6.9	-6.6	0.18	2.719	0.914	4.067	1.093	6	6162	ZINC000015119267
0.4	-6.7	-6.5	0.11	2.021	0.359	3.451	1.451	6	6163	ZINC000085808290
0.4	-5.3	-5.0	0.20	1.764	0.913	2.624	1.187	6	6164	ZINC000001693581
0.4	-7.4	-6.9	0.31	1.816	0.410	5.338	1.014	6	6165	ZINC000066331981
0.4	-7.0	-6.5	0.25	2.742	0.743	5.396	0.498	6	6166	ZINC000005112938
0.4	-6.5	-6.4	0.14	2.415	0.422	3.291	0.260	6	6167	ZINC000006437474
0.4	-10.8	-9.5	0.72	1.738	0.484	4.758	1.607	6	6168	ZINC000100774350
0.4	-9.1	-8.5	0.53	1.891	0.541	4.745	1.177	6	6169	ZINC000100782057
0.4	-6.5	-6.1	0.24	2.408	0.414	3.637	0.633	6	6170	ZINC000001680692
0.4	-8.1	-7.7	0.33	1.838	0.458	5.984	1.613	6	6171	ZINC000257765105
0.4	-7.3	-7.0	0.21	3.195	0.227	4.005	0.319	6	6172	ZINC000013481668
0.4	-8.2	-7.7	0.42	2.083	0.310	4.137	0.952	6	6173	ZINC000256070042

0.4	-5.4	-5.3	0.13	1.737	0.551	2.815	0.554	6	6174	ZINC000022000199
0.4	-8.0	-7.9	0.10	2.623	0.174	4.991	0.832	6	6175	ZINC000003014492
0.4	-9.3	-8.8	0.29	1.623	0.293	4.113	0.801	6	6176	ZINC000257382664
0.4	-6.6	-6.0	0.28	2.007	0.286	3.177	0.792	6	6177	ZINC000001684870
0.4	-7.1	-6.7	0.25	1.895	0.114	3.243	0.246	6	6178	ZINC000001849853
0.4	-9.3	-8.7	0.60	1.707	0.129	4.210	1.675	6	6179	ZINC000100780665
0.4	-5.8	-5.8	0.08	1.970	0.533	3.877	0.952	6	6180	ZINC000095618188
0.4	-8.3	-7.7	0.31	2.095	0.469	5.078	0.754	6	6181	ZINC000004097829
0.4	-6.3	-6.0	0.20	2.373	0.396	3.374	0.460	6	6182	ZINC000000388064
0.4	-6.8	-6.7	0.07	2.401	1.152	4.209	0.919	6	6183	ZINC000015113357
0.4	-6.7	-6.5	0.19	3.077	0.625	4.709	1.109	6	6184	ZINC000040470650
0.4	-7.7	-7.6	0.17	2.316	0.117	4.687	0.281	6	6185	ZINC000001605727
0.4	-8.7	-8.3	0.34	2.282	0.682	4.258	1.030	6	6186	ZINC000100232127
0.4	-6.4	-6.2	0.15	1.602	1.098	3.419	0.626	6	6187	ZINC000002242686
0.4	-9.1	-8.7	0.25	1.671	0.243	4.588	1.749	6	6188	ZINC000085887474

0.4	-8.7	-7.7	0.67	2.681	0.549	5.584	1.380	6	6189	ZINC000014820544
0.4	-5.7	-5.4	0.21	2.138	0.526	3.634	0.932	6	6190	ZINC000005735816
0.4	-8.7	-8.3	0.30	1.676	0.879	3.666	1.487	6	6191	ZINC000257631731
0.4	-8.1	-7.7	0.21	2.024	0.799	3.751	1.559	6	6192	ZINC000014859827
0.4	-6.5	-6.3	0.11	2.296	0.687	3.329	1.013	6	6193	ZINC000014719169
0.4	-7.8	-7.5	0.17	1.539	0.449	3.561	0.965	6	6194	ZINC000004655404
0.4	-10.6	-9.1	0.83	1.910	0.392	6.151	0.319	6	6195	ZINC000100780480
0.4	-8.0	-7.7	0.17	1.750	0.604	3.271	0.992	6	6196	ZINC000014614594
0.4	-7.6	-7.2	0.24	2.345	0.862	3.589	1.210	6	6197	ZINC000001576651
0.4	-6.6	-6.3	0.18	1.994	0.951	3.386	0.858	6	6198	ZINC000000338275
0.4	-8.6	-8.2	0.30	1.912	0.370	3.839	1.364	6	6199	ZINC000257467839
0.4	-5.4	-5.2	0.10	2.459	0.653	3.877	1.066	6	6200	ZINC000001701826
0.4	-7.2	-7.0	0.16	1.952	0.241	2.645	0.604	6	6201	ZINC000004545921
0.4	-6.2	-5.9	0.21	3.139	1.022	4.582	1.668	6	6202	ZINC000001529718
0.4	-7.7	-7.1	0.34	2.469	0.914	3.967	1.889	6	6203	ZINC000000895813

0.4	-7.5	-7.3	0.17	1.733	0.372	2.565	0.696	6	6204	ZINC000015213738
0.4	-6.9	-6.5	0.20	3.219	1.350	4.236	1.718	6	6205	ZINC000012410579
0.4	-9.1	-8.3	0.45	1.716	0.278	4.877	1.754	6	6206	ZINC000014919035
0.4	-6.2	-5.9	0.19	3.519	0.887	4.725	1.291	6	6207	ZINC000014948991
0.4	-8.2	-7.6	0.35	2.567	0.750	5.361	1.642	6	6208	ZINC000004899652
0.4	-6.3	-6.0	0.19	1.045	0.644	4.062	0.886	6	6209	ZINC000002013424
0.4	-6.1	-5.6	0.21	1.891	0.890	3.349	0.784	6	6210	ZINC000013380324
0.4	-6.8	-6.6	0.13	2.293	0.171	3.401	1.002	6	6211	ZINC000005274026
0.4	-10.6	-9.5	0.61	1.927	0.306	5.035	1.402	6	6212	ZINC000257383243
0.4	-6.9	-6.6	0.17	2.415	0.886	4.075	0.843	6	6213	ZINC000004095529
0.4	-5.7	-5.5	0.14	3.113	1.193	4.317	1.439	6	6214	ZINC000001605654
0.4	-6.6	-6.2	0.21	2.114	0.227	4.909	1.506	6	6215	ZINC000017147663
0.4	-8.8	-8.6	0.15	2.696	0.285	4.340	0.864	6	6216	ZINC000004099030
0.4	-8.4	-8.0	0.36	1.461	0.382	4.721	1.303	6	6217	ZINC000001850793
0.4	-7.5	-7.3	0.15	2.004	0.328	3.519	0.863	6	6218	ZINC000004095578

0.4	-6.8	-6.7	0.07	1.900	1.043	2.944	0.664	6	6219	ZINC000012362995
0.4	-6.1	-5.9	0.12	2.965	0.913	4.301	0.531	6	6220	ZINC000032176538
0.4	-6.4	-6.1	0.19	1.907	1.065	3.314	1.335	6	6221	ZINC000002003388
0.4	-4.3	-4.2	0.08	3.015	1.454	4.031	1.122	6	6222	ZINC000006037906
0.4	-6.1	-5.8	0.24	1.927	1.245	3.157	1.582	6	6223	ZINC000002039413
0.4	-7.6	-7.4	0.14	1.993	0.330	4.195	1.127	6	6224	ZINC000013515634
0.4	-7.1	-6.9	0.16	2.003	0.393	3.661	1.043	6	6225	ZINC000032821774
0.4	-5.4	-5.0	0.25	2.479	0.893	3.952	0.452	6	6226	ZINC000001576855
0.4	-6.1	-6.0	0.12	1.410	0.276	3.031	0.794	6	6227	ZINC000100828976
0.4	-6.9	-6.8	0.11	1.358	0.568	3.052	0.489	6	6228	ZINC000100781686
0.4	-6.5	-6.2	0.22	2.785	1.192	4.157	1.164	6	6229	ZINC000005339839
0.4	-6.7	-6.5	0.14	3.076	0.355	3.985	0.426	6	6230	ZINC000015253416
0.4	-9.4	-8.4	0.54	1.685	0.459	3.574	1.441	6	6231	ZINC000015112120
0.4	-8.6	-8.2	0.32	1.999	0.381	4.023	1.111	6	6232	ZINC000100772274
0.4	-7.1	-6.9	0.11	2.120	0.664	3.253	0.359	6	6233	ZINC000012496248

0.4	-5.4	-5.2	0.12	2.258	0.985	3.254	0.964	6	6234	ZINC000001683479
0.4	-9.9	-9.5	0.25	1.536	0.457	3.329	0.916	6	6235	ZINC000085666899
0.4	-7.4	-7.2	0.13	2.481	0.330	5.041	0.758	6	6236	ZINC000015122069
0.4	-7.4	-7.2	0.12	2.330	0.685	3.744	1.168	6	6237	ZINC000013481683
0.4	-6.0	-5.7	0.18	1.350	0.580	3.718	1.045	6	6238	ZINC000008551134
0.4	-8.7	-8.3	0.24	2.337	0.178	4.396	0.692	6	6239	ZINC000085876815
0.4	-5.8	-5.7	0.07	2.008	0.470	4.880	0.369	6	6240	ZINC000033839264
0.4	-10.4	-10.3	0.12	1.674	0.253	3.926	2.063	6	6241	ZINC000257468255
0.4	-6.3	-6.0	0.21	1.259	0.970	4.601	0.516	6	6242	ZINC000001532740
0.4	-3.8	-3.5	0.26	3.703	1.901	4.014	2.003	6	6243	ZINC000005650763
0.4	-7.5	-7.3	0.22	2.209	0.352	3.859	1.317	6	6244	ZINC000001579825
0.4	-8.0	-7.8	0.13	2.289	0.857	4.106	1.233	6	6245	ZINC000002566154
0.4	-8.3	-7.9	0.32	1.999	0.291	4.690	1.543	6	6246	ZINC000100782001
0.4	-6.6	-6.3	0.15	2.708	0.477	4.826	0.739	6	6247	ZINC000095617786
0.4	-8.1	-7.4	0.31	2.115	0.227	3.936	1.010	6	6248	ZINC000005618680

0.4	-7.7	-7.1	0.31	2.620	0.817	4.468	0.692	6	6249	ZINC000001850435
0.4	-9.0	-8.7	0.21	1.716	0.334	6.428	0.405	6	6250	ZINC000257497786
0.4	-8.9	-8.5	0.28	1.925	0.164	6.311	0.447	6	6251	ZINC000014438699
0.4	-7.2	-7.0	0.13	2.282	0.559	4.011	1.268	6	6252	ZINC000095618083
0.4	-8.3	-7.5	0.49	1.753	0.274	3.381	1.479	6	6253	ZINC000014413377
0.4	-8.9	-8.5	0.31	2.048	0.514	5.044	1.633	6	6254	ZINC000005640448
0.4	-9.9	-8.0	0.93	2.044	0.533	4.031	1.610	6	6255	ZINC000100829420
0.4	-4.9	-4.9	0.05	3.084	1.367	3.971	0.951	6	6256	ZINC000029786461
0.4	-9.4	-8.8	0.31	2.265	0.224	5.377	0.227	6	6257	ZINC000100198557
0.4	-4.3	-4.1	0.12	1.570	0.908	3.380	0.689	6	6258	ZINC000001674077
0.4	-9.4	-8.3	0.63	1.584	0.287	4.887	1.523	6	6259	ZINC000022062288
0.4	-9.3	-8.6	0.38	2.103	0.314	4.388	1.514	6	6260	ZINC000014805957
0.4	-7.3	-6.8	0.25	0.808	0.276	4.231	0.520	6	6261	ZINC000059585752
0.4	-8.4	-8.1	0.19	1.936	0.391	4.484	1.809	6	6262	ZINC000014679850
0.4	-9.2	-9.0	0.17	2.056	0.383	5.760	1.638	6	6263	ZINC000257424780

0.4	-4.4	-4.2	0.11	2.774	1.678	3.611	1.434	6	6264	ZINC000006072081
0.4	-8.0	-7.7	0.24	1.868	0.394	3.432	1.118	6	6265	ZINC000100779310
0.4	-7.0	-6.9	0.10	2.420	0.514	4.540	0.765	6	6266	ZINC000034893632
0.4	-9.1	-8.3	0.50	1.677	0.297	5.005	1.625	6	6267	ZINC000015249366
0.4	-8.1	-7.8	0.22	1.953	0.336	4.780	0.662	6	6268	ZINC000012496290
0.4	-6.0	-5.7	0.18	2.030	0.958	2.726	0.622	6	6269	ZINC000100002477
0.4	-8.0	-7.6	0.23	1.930	1.129	3.161	0.739	6	6270	ZINC000033833974
0.4	-4.5	-4.5	0.00	0.905	1.461	2.858	1.260	6	6271	ZINC000086040406
0.4	-8.7	-8.3	0.22	1.795	0.411	3.292	1.663	6	6272	ZINC000013481472
0.4	-6.9	-6.6	0.21	2.889	0.161	3.873	0.190	6	6273	ZINC000001529359
0.4	-5.3	-5.0	0.20	1.775	0.348	2.464	0.308	6	6274	ZINC000001663926
0.4	-7.4	-7.2	0.17	2.498	0.355	4.142	1.265	6	6275	ZINC000001888743
0.4	-5.9	-5.7	0.15	2.624	0.965	3.752	0.774	6	6276	ZINC000001632727
0.4	-10.5	-9.3	0.59	2.003	0.271	4.573	1.067	6	6277	ZINC000257800073
0.4	-9.1	-8.0	0.79	2.388	0.442	4.783	0.457	6	6278	ZINC000003785676

0.4	-5.0	-4.8	0.09	1.858	0.196	2.794	0.412	6	6279	ZINC000095620439
0.4	-4.8	-4.6	0.14	2.644	0.734	3.184	0.739	6	6280	ZINC000004262348
0.4	-8.9	-7.9	0.62	2.021	0.480	3.516	1.048	6	6281	ZINC000001532046
0.4	-8.4	-8.0	0.38	1.396	0.362	3.112	1.847	6	6282	ZINC000014919007
0.4	-5.6	-5.3	0.16	2.770	0.633	3.670	1.065	6	6283	ZINC000000409294
0.4	-7.8	-7.5	0.20	1.938	0.596	3.463	1.141	6	6284	ZINC000014691901
0.4	-7.9	-7.7	0.13	2.055	0.339	5.427	0.378	6	6285	ZINC000004654965
0.4	-9.5	-8.4	0.62	1.630	0.313	4.480	1.784	6	6286	ZINC000257374300
0.4	-6.5	-6.4	0.07	2.414	0.638	3.402	0.690	6	6287	ZINC000000895443
0.4	-9.3	-8.3	0.78	1.917	0.138	4.126	0.811	6	6288	ZINC000100825888
0.4	-8.1	-7.9	0.16	2.042	0.505	3.431	0.404	6	6289	ZINC000014823312
0.4	-9.3	-8.9	0.29	1.823	0.281	4.187	1.413	6	6290	ZINC000015217357
0.4	-5.1	-4.9	0.15	2.223	0.779	3.264	0.602	6	6291	ZINC000006069855
0.4	-8.6	-7.7	0.46	1.829	0.523	4.111	1.351	6	6292	ZINC000095617970
0.4	-6.4	-6.1	0.15	2.237	0.297	3.610	0.924	6	6293	ZINC000004533816

0.4	-5.2	-5.0	0.15	2.102	0.280	3.270	0.500	6	6294	ZINC000029786613
0.4	-8.7	-8.2	0.31	2.277	0.379	4.754	0.937	6	6295	ZINC000014643308
0.4	-9.9	-8.4	0.92	2.028	0.523	4.444	1.407	6	6296	ZINC000238730544
0.4	-5.4	-5.2	0.10	2.619	0.940	3.895	1.387	6	6297	ZINC000095619619
0.4	-7.1	-7.0	0.09	2.145	0.399	4.171	0.820	6	6298	ZINC000013346770
0.4	-7.3	-7.0	0.20	2.446	0.210	4.967	0.387	6	6299	ZINC000050027194
0.4	-6.7	-6.4	0.17	2.499	0.267	4.667	0.394	6	6300	ZINC000034946850
0.4	-7.3	-7.0	0.20	2.342	0.616	3.090	0.937	6	6301	ZINC000001995685
0.4	-9.6	-8.5	1.08	1.367	0.927	2.914	0.495	4	6302	ZINC000014646784
0.4	-8.3	-7.8	0.26	2.328	0.625	4.201	1.455	6	6303	ZINC000005260866
0.4	-5.6	-5.2	0.20	2.283	1.297	3.686	1.188	6	6304	ZINC000100772443
0.4	-6.5	-6.3	0.18	1.739	0.317	2.546	0.623	6	6305	ZINC000095618088
0.4	-8.8	-7.7	0.53	2.413	0.754	3.800	1.383	6	6306	ZINC000004096881
0.4	-7.6	-7.4	0.12	1.891	0.190	3.848	0.792	6	6307	ZINC000100822074
0.4	-7.7	-7.4	0.18	2.289	0.350	5.606	1.712	6	6308	ZINC000013896421

0.4	-7.9	-6.9	0.53	2.364	0.299	4.532	0.560	6	6309	ZINC000004096500
0.4	-6.3	-6.1	0.15	2.077	0.495	3.894	0.455	6	6310	ZINC000002019886
0.4	-7.0	-6.9	0.07	2.276	0.297	2.994	0.527	6	6311	ZINC000069486029
0.4	-5.9	-5.8	0.11	2.841	0.861	4.154	0.853	6	6312	ZINC000014592849
0.4	-10.6	-9.5	0.57	1.907	0.287	5.073	1.436	6	6313	ZINC000257383242
0.4	-6.1	-6.0	0.11	2.594	0.479	3.448	0.427	6	6314	ZINC000001574322
0.4	-6.3	-6.0	0.19	1.260	0.818	4.256	0.904	6	6315	ZINC000012359024
0.4	-9.9	-9.5	0.24	1.549	0.450	3.338	0.920	6	6316	ZINC000100829371
0.4	-9.9	-9.5	0.26	1.542	0.452	3.331	0.908	6	6317	ZINC000100829370
0.4	-6.6	-6.4	0.10	2.730	0.408	5.378	0.436	6	6318	ZINC000029545753
0.4	-5.7	-5.4	0.21	2.209	0.427	3.192	0.684	6	6319	ZINC000002561202
0.4	-7.4	-7.0	0.17	1.605	0.211	3.281	0.657	6	6320	ZINC000014724752
0.4	-8.7	-8.3	0.33	1.875	0.648	4.127	0.963	6	6321	ZINC000257631730
0.4	-8.9	-8.6	0.20	1.704	0.500	4.001	2.167	6	6322	ZINC000018825330
0.4	-8.2	-7.9	0.21	3.077	0.286	5.001	1.301	6	6323	ZINC000039208822

0.4	-8.0	-7.6	0.32	2.015	0.398	4.701	1.777	6	6324	ZINC000014616305
0.4	-6.9	-6.8	0.06	2.385	0.944	3.945	1.361	6	6325	ZINC000005274089
0.4	-6.7	-6.5	0.21	2.951	1.034	4.289	1.537	6	6326	ZINC000038673526
0.4	-8.4	-7.6	0.37	2.377	0.980	3.605	1.518	6	6327	ZINC000033994789
0.4	-5.5	-5.2	0.13	1.600	0.224	2.640	0.232	6	6328	ZINC000003860605
0.4	-7.0	-6.7	0.21	2.484	0.318	3.559	0.091	6	6329	ZINC000015261389
0.4	-7.9	-7.4	0.29	2.600	0.444	3.353	1.082	6	6330	ZINC000006019680
0.4	-6.0	-5.6	0.17	2.593	0.939	3.929	1.332	6	6331	ZINC000013541290
0.4	-6.4	-6.2	0.10	2.610	0.216	3.129	0.316	6	6332	ZINC000000002501
0.4	-5.6	-5.4	0.16	2.498	0.750	3.311	0.716	6	6333	ZINC000001529769
0.4	-8.6	-8.2	0.28	2.149	0.624	4.031	2.096	6	6334	ZINC000012496640
0.4	-6.1	-6.0	0.09	2.170	0.337	4.334	1.074	6	6335	ZINC000001637981
0.4	-7.6	-7.5	0.15	2.675	0.334	3.742	0.822	6	6336	ZINC000000002067
0.4	-7.3	-7.2	0.17	1.125	0.601	3.151	1.110	6	6337	ZINC000004096300
0.4	-4.0	-3.9	0.14	2.325	0.763	3.022	0.781	6	6338	ZINC000001707856

0.4	-6.7	-6.5	0.20	1.893	0.710	3.833	1.050	6	6339	ZINC000014488960
0.4	-9.0	-8.7	0.19	1.659	0.336	3.841	0.509	6	6340	ZINC000257421069
0.4	-6.4	-6.2	0.10	2.116	0.232	3.450	0.477	6	6341	ZINC000013429400
0.4	-6.3	-6.1	0.16	1.584	0.515	4.171	1.221	6	6342	ZINC000015269439
0.4	-5.1	-4.8	0.16	1.750	0.506	2.464	0.792	6	6343	ZINC000001586314
0.4	-7.7	-7.3	0.26	2.113	0.369	5.006	1.656	6	6344	ZINC000005641400
0.4	-8.3	-8.0	0.19	2.065	0.386	4.702	1.057	6	6345	ZINC000015120479
0.4	-7.3	-7.2	0.05	1.200	0.447	6.168	1.895	6	6346	ZINC000014860253
0.4	-8.6	-8.3	0.23	1.927	0.544	3.957	2.227	6	6347	ZINC000261499672
0.4	-8.4	-7.8	0.35	2.020	0.272	3.604	1.041	6	6348	ZINC000006068806
0.4	-7.7	-7.1	0.31	1.919	0.539	4.794	1.699	6	6349	ZINC000015149587
0.4	-9.3	-8.8	0.29	1.639	0.315	4.106	0.854	6	6350	ZINC000100782064
0.4	-6.3	-6.0	0.24	1.548	0.430	3.639	1.017	6	6351	ZINC000079566593
0.4	-7.0	-6.7	0.14	1.987	0.230	4.120	0.734	6	6352	ZINC000001849803
0.4	-9.1	-8.4	0.35	2.313	0.562	4.656	0.532	6	6353	ZINC000004521472

0.4	-10.4	-10.3	0.12	1.733	0.221	5.804	1.298	6	6354	ZINC000100778038
0.4	-8.2	-7.8	0.26	2.644	0.612	4.712	1.897	6	6355	ZINC000002567233
0.4	-7.4	-7.1	0.18	2.421	0.386	4.544	0.480	6	6356	ZINC000012495932
0.4	-9.7	-9.2	0.55	1.476	0.385	4.947	1.679	6	6357	ZINC000000001219
0.4	-7.1	-6.9	0.14	3.598	0.669	4.629	0.809	6	6358	ZINC000001849750
0.4	-10.5	-9.8	0.49	2.265	1.490	3.679	1.729	6	6359	ZINC000253633072
0.4	-6.1	-5.8	0.26	2.343	1.250	3.266	1.586	6	6360	ZINC000002039410
0.4	-5.9	-5.6	0.14	2.598	0.670	4.006	1.367	6	6361	ZINC000028631780
0.4	-5.6	-5.3	0.14	1.760	0.345	3.565	0.255	6	6362	ZINC000012495431
0.4	-6.6	-6.4	0.11	2.942	0.648	4.146	0.832	6	6363	ZINC000100771703
0.4	-8.6	-7.6	0.84	1.777	0.347	7.003	0.243	6	6364	ZINC000005732362
0.4	-6.7	-6.4	0.22	2.091	0.274	4.069	1.104	6	6365	ZINC000015121415
0.4	-8.6	-8.3	0.18	1.884	0.287	6.008	0.785	6	6366	ZINC000014438576
0.4	-8.7	-8.1	0.40	1.991	0.614	3.388	1.506	6	6367	ZINC000013311797
0.4	-7.9	-7.7	0.20	1.728	0.242	3.902	0.708	6	6368	ZINC000002040989

0.4	-6.8	-6.6	0.19	2.355	0.203	4.163	0.554	6	6369	ZINC000014722251
0.4	-6.3	-6.0	0.19	1.405	0.872	4.277	1.080	6	6370	ZINC000003860635
0.4	-6.9	-6.4	0.29	2.262	0.625	3.913	1.091	6	6371	ZINC000004082318
0.4	-8.3	-8.1	0.17	1.692	0.302	3.737	1.142	6	6372	ZINC000257617943
0.4	-5.3	-4.9	0.28	2.404	0.761	3.184	0.858	6	6373	ZINC000000391113
0.4	-9.0	-8.6	0.22	2.281	0.316	4.642	1.292	6	6374	ZINC000257375891
0.4	-7.5	-7.2	0.17	2.784	0.338	4.479	1.095	6	6375	ZINC000001846541
0.4	-8.1	-7.6	0.23	2.313	0.194	4.408	1.081	6	6376	ZINC000050027843
0.4	-7.4	-7.1	0.21	2.746	0.595	3.950	1.405	6	6377	ZINC000014766024
0.4	-6.7	-6.6	0.16	2.402	0.250	4.098	1.617	6	6378	ZINC000050027392
0.4	-7.4	-7.2	0.16	2.660	0.103	6.407	0.111	6	6379	ZINC000033995143
0.4	-7.1	-7.0	0.07	2.292	0.269	4.297	0.417	6	6380	ZINC000014491215
0.4	-9.5	-8.2	0.75	1.883	0.126	4.338	1.253	6	6381	ZINC000100780123
0.4	-8.0	-7.8	0.11	1.837	0.303	3.259	1.168	6	6382	ZINC000014556671
0.4	-7.0	-6.6	0.26	2.453	0.196	4.788	0.242	6	6383	ZINC000069485810

0.4	-10.5	-9.0	0.76	1.882	0.239	5.277	0.896	6	6384	ZINC000095620634
0.4	-10.3	-9.4	0.78	1.770	0.506	3.659	1.907	6	6385	ZINC000014762739
0.4	-8.9	-8.5	0.18	2.605	0.222	5.037	1.238	6	6386	ZINC000004090458
0.4	-10.2	-8.9	0.88	1.997	0.553	3.622	1.809	6	6387	ZINC000014612200
0.4	-7.2	-7.1	0.11	2.029	0.133	4.891	1.102	6	6388	ZINC000257496000
0.4	-9.4	-9.0	0.35	1.429	0.154	3.889	1.309	6	6389	ZINC000257388640
0.4	-6.4	-6.3	0.08	1.843	0.308	4.583	1.476	6	6390	ZINC000095618157
0.4	-7.1	-6.7	0.31	1.603	0.441	3.957	1.842	6	6391	ZINC000004228295
0.4	-5.8	-5.7	0.08	2.887	0.552	3.637	0.564	6	6392	ZINC000000358476
0.4	-9.9	-9.1	0.75	1.384	0.400	3.402	1.672	3	6393	ZINC000014827096
0.4	-8.7	-7.9	0.43	2.017	0.077	3.444	1.110	6	6394	ZINC000014682678
0.4	-5.6	-5.3	0.22	2.261	0.693	3.482	0.546	6	6395	ZINC000001849774
0.4	-9.7	-8.1	0.94	1.465	0.342	5.179	1.849	6	6396	ZINC000014715664
0.4	-6.3	-6.2	0.08	2.370	0.499	4.298	0.511	6	6397	ZINC000014723366
0.4	-6.6	-6.5	0.09	3.157	0.414	5.835	0.449	6	6398	ZINC000034946851

0.4	-9.5	-8.2	0.70	1.721	0.217	4.950	0.913	6	6399	ZINC000100780121
0.4	-7.5	-7.3	0.09	2.239	0.680	5.138	1.364	6	6400	ZINC000013372522
0.4	-8.4	-7.8	0.45	1.775	0.180	5.234	1.835	6	6401	ZINC000257402401
0.4	-6.2	-5.9	0.20	3.369	1.517	4.037	1.989	6	6402	ZINC000019440143
0.4	-7.9	-7.3	0.33	2.258	0.407	3.930	1.460	6	6403	ZINC000027744102
0.4	-7.3	-7.0	0.25	1.915	0.376	4.670	2.397	6	6404	ZINC000071756293
0.4	-7.7	-7.4	0.26	2.394	0.714	4.082	1.168	6	6405	ZINC000000156395
0.4	-7.5	-7.1	0.22	2.009	0.578	3.181	1.704	6	6406	ZINC000050027886
0.4	-7.6	-7.2	0.28	1.920	0.447	4.536	1.645	6	6407	ZINC000014591331
0.4	-10.5	-9.8	0.47	1.830	1.254	3.424	1.312	6	6408	ZINC000100091855
0.4	-6.8	-6.6	0.12	2.891	0.369	4.023	0.504	6	6409	ZINC000000388060
0.4	-8.2	-8.0	0.21	2.407	0.723	4.078	1.517	6	6410	ZINC000040478998
0.4	-6.5	-6.4	0.05	2.787	0.169	5.182	0.713	6	6411	ZINC000002384790
0.4	-6.0	-5.8	0.09	1.530	0.605	2.693	0.777	6	6412	ZINC000001699427
0.4	-4.2	-4.0	0.10	1.519	0.593	2.864	0.279	6	6413	ZINC000005765013

0.4	-10.3	-9.7	0.36	2.088	0.405	4.438	1.869	6	6414	ZINC000100776851
0.4	-7.6	-7.4	0.15	1.910	0.206	4.137	0.999	6	6415	ZINC000257433632
0.4	-4.3	-4.2	0.04	2.824	1.509	5.086	0.219	6	6416	ZINC000005132893
0.4	-8.9	-8.6	0.17	1.279	0.661	2.188	0.588	6	6417	ZINC000015249525
0.4	-4.8	-4.7	0.11	3.181	1.388	4.132	0.911	6	6418	ZINC000000897447
0.4	-8.0	-7.5	0.30	2.092	0.140	5.029	1.054	6	6419	ZINC000062233600
0.4	-7.9	-7.6	0.20	1.601	0.150	3.344	0.597	6	6420	ZINC000005762558
0.4	-7.1	-6.6	0.27	2.198	0.759	3.105	0.976	6	6421	ZINC000015253419
0.4	-8.3	-8.0	0.13	1.649	0.319	3.295	1.549	6	6422	ZINC000014491824
0.4	-4.7	-4.6	0.07	2.127	0.382	3.345	0.262	6	6423	ZINC000032163933
0.4	-5.9	-5.6	0.12	2.591	0.749	4.121	0.815	6	6424	ZINC000029786597
0.4	-8.5	-8.0	0.28	1.718	0.195	4.072	0.574	6	6425	ZINC000015121957
0.4	-7.6	-7.4	0.13	2.111	0.677	4.519	1.682	6	6426	ZINC000012494846
0.4	-6.6	-6.4	0.12	1.945	0.457	3.016	1.087	6	6427	ZINC000095620543
0.4	-9.5	-9.0	0.40	1.791	0.562	3.387	1.405	6	6428	ZINC000014728044

0.4	-9.2	-8.5	0.37	2.103	0.180	3.606	0.558	6	6429	ZINC000014593851
0.4	-6.0	-5.8	0.14	1.636	0.604	2.977	1.170	6	6430	ZINC000100113204
0.4	-7.0	-6.8	0.15	1.714	0.466	3.694	1.063	6	6431	ZINC000001677816
0.4	-8.0	-7.3	0.37	1.507	0.268	3.934	0.992	6	6432	ZINC000086032848
0.4	-5.9	-5.5	0.17	2.477	0.754	4.038	0.607	6	6433	ZINC000002570845
0.4	-6.5	-6.4	0.13	2.649	0.533	4.272	0.719	6	6434	ZINC000021999709
0.4	-9.9	-9.5	0.26	1.663	0.341	3.753	0.784	6	6435	ZINC000085666897
0.4	-5.2	-5.1	0.11	2.798	0.973	3.417	1.096	6	6436	ZINC000019735138
0.4	-6.9	-6.7	0.12	2.376	0.737	3.052	0.782	6	6437	ZINC000000087959
0.4	-4.9	-4.6	0.17	1.664	0.404	2.665	0.689	6	6438	ZINC000002041123
0.4	-6.6	-6.1	0.23	2.165	0.637	3.948	1.800	6	6439	ZINC000012495398
0.4	-8.1	-7.6	0.33	2.112	0.557	3.760	1.202	6	6440	ZINC000014766023
0.4	-7.8	-7.4	0.21	1.956	0.268	6.044	0.668	6	6441	ZINC000012496400
0.4	-7.8	-7.6	0.20	1.735	0.519	3.332	1.548	6	6442	ZINC000100822354
0.4	-8.9	-8.2	0.41	1.462	0.338	4.678	2.117	6	6443	ZINC000100900259

0.4	-8.1	-7.8	0.15	1.793	0.167	4.584	1.295	6	6444	ZINC000013208862
0.4	-7.4	-7.1	0.18	2.072	0.837	4.424	2.381	6	6445	ZINC000095618365
0.4	-5.3	-5.0	0.19	2.072	0.414	3.720	0.554	6	6446	ZINC000001680431
0.4	-10.2	-9.1	0.94	1.879	0.489	5.238	0.083	3	6447	ZINC000033616004
0.4	-6.4	-6.1	0.17	3.882	0.950	5.145	1.233	6	6448	ZINC000003869979
0.4	-6.9	-6.8	0.08	2.397	0.353	4.459	0.506	6	6449	ZINC000050027226
0.4	-8.3	-7.6	0.65	1.888	0.426	3.759	1.593	6	6450	ZINC000257530775
0.4	-6.6	-6.4	0.13	2.467	0.266	4.785	0.742	6	6451	ZINC000013347371
0.4	-6.0	-5.6	0.23	2.883	0.575	4.121	0.759	6	6452	ZINC000003875730
0.4	-6.8	-6.7	0.08	3.455	1.016	5.119	0.673	6	6453	ZINC000095618102
0.4	-8.8	-8.6	0.18	1.479	0.736	4.125	2.073	6	6454	ZINC000015251510
0.4	-5.6	-5.4	0.19	2.306	0.267	3.770	0.507	6	6455	ZINC000001848538
0.4	-7.7	-7.5	0.20	1.826	0.513	3.345	1.161	6	6456	ZINC000000384854
0.4	-6.9	-6.8	0.14	2.574	0.633	4.299	1.163	6	6457	ZINC000040472687
0.4	-8.3	-8.0	0.23	1.813	0.558	4.509	1.975	6	6458	ZINC000005116716

0.4	-9.1	-8.4	0.38	2.221	0.556	4.422	0.478	6	6459	ZINC000004081998
0.4	-6.3	-6.0	0.21	1.035	0.661	3.554	1.035	6	6460	ZINC000008551223
0.4	-6.6	-6.4	0.11	2.305	0.337	3.764	0.455	6	6461	ZINC000002018445
0.4	-9.4	-8.7	0.37	2.011	0.200	4.453	0.829	6	6462	ZINC000100824311
0.4	-9.3	-7.6	1.01	1.884	0.178	5.084	1.576	4	6463	ZINC000013308656
0.4	-6.5	-6.4	0.13	2.198	0.788	3.544	0.938	6	6464	ZINC000005819711
0.4	-7.4	-7.0	0.28	2.315	0.263	4.527	1.053	6	6465	ZINC000003636015
0.4	-4.7	-4.5	0.11	3.456	1.183	4.000	1.025	6	6466	ZINC000005159440
0.4	-7.7	-7.3	0.21	1.873	0.466	4.545	0.742	6	6467	ZINC000012496534
0.4	-6.3	-6.0	0.18	3.474	1.007	5.187	0.605	6	6468	ZINC000004234801
0.4	-6.8	-6.3	0.28	2.853	0.366	3.484	0.534	6	6469	ZINC000000213065
0.4	-5.8	-5.5	0.14	3.433	0.552	4.746	0.989	6	6470	ZINC000006020915
0.4	-6.7	-6.5	0.15	2.351	0.460	4.804	1.438	6	6471	ZINC000040472689
0.4	-6.1	-5.9	0.12	2.671	1.284	3.926	1.074	6	6472	ZINC000001693631
0.4	-9.4	-8.8	0.31	2.278	0.239	5.387	0.229	6	6473	ZINC000231215774

0.4	-7.9	-7.6	0.22	2.244	0.577	4.688	0.693	6	6474	ZINC000000338221
0.4	-8.6	-8.4	0.13	2.468	0.712	3.883	1.459	6	6475	ZINC000050027362
0.4	-6.7	-6.5	0.14	2.037	0.180	2.557	0.179	6	6476	ZINC000001574271
0.4	-10.3	-9.8	0.27	2.044	0.424	3.592	1.553	6	6477	ZINC000100776850
0.4	-4.9	-4.9	0.05	3.319	1.636	4.218	1.050	6	6478	ZINC000006066886
0.4	-6.8	-6.5	0.24	2.197	1.041	3.619	0.750	6	6479	ZINC000000409219
0.4	-5.3	-5.2	0.09	2.088	0.515	3.673	0.836	6	6480	ZINC000001712794
0.4	-7.0	-6.7	0.18	2.987	1.245	4.361	1.766	6	6481	ZINC000002504940
0.4	-9.4	-8.7	0.32	2.177	0.330	4.895	1.151	6	6482	ZINC000231215728
0.4	-8.0	-7.7	0.21	2.023	0.402	4.180	1.565	6	6483	ZINC000001842680
0.4	-8.6	-8.1	0.38	2.272	0.707	3.166	1.175	6	6484	ZINC000000037835
0.4	-6.1	-5.8	0.15	2.870	0.483	4.209	0.338	6	6485	ZINC000001569723
0.4	-6.6	-6.3	0.16	2.079	0.763	2.990	0.363	6	6486	ZINC000012153298
0.4	-8.3	-8.1	0.20	1.790	0.231	4.041	0.520	6	6487	ZINC000100781978
0.4	-5.6	-5.5	0.15	3.352	0.621	4.566	0.409	6	6488	ZINC000014616871

0.4	-9.4	-9.1	0.24	1.387	0.135	3.336	1.414	6	6489	ZINC000257388638
0.4	-5.7	-5.4	0.22	2.531	0.687	3.001	0.734	6	6490	ZINC000005196288
0.4	-7.2	-6.9	0.23	2.029	0.211	3.052	0.533	6	6491	ZINC000001589375
0.4	-9.4	-8.8	0.32	2.271	0.347	4.306	0.823	6	6492	ZINC000095617739
0.4	-7.2	-6.8	0.22	3.567	0.645	4.678	1.198	6	6493	ZINC000100361770
0.4	-9.6	-8.3	0.86	1.799	0.390	4.401	1.478	6	6494	ZINC000257525714
0.4	-4.5	-4.3	0.16	2.465	1.151	3.457	1.060	6	6495	ZINC000000388287
0.4	-8.6	-8.3	0.22	2.095	0.638	4.337	0.575	6	6496	ZINC000001618130
0.4	-7.3	-7.1	0.11	4.220	0.571	5.945	0.508	6	6497	ZINC000014419577
0.4	-7.9	-7.5	0.26	1.801	0.470	4.175	0.972	6	6498	ZINC000034961798
0.4	-7.4	-7.2	0.11	1.771	0.507	4.760	0.784	6	6499	ZINC000004655386
0.4	-7.2	-6.9	0.16	2.367	0.480	4.573	1.871	6	6500	ZINC000069485829
0.4	-8.1	-7.7	0.27	3.119	0.548	4.496	0.589	6	6501	ZINC000100029127
0.4	-9.1	-7.4	1.13	2.164	0.750	3.968	1.550	6	6502	ZINC000015203726
0.4	-6.7	-6.4	0.23	2.498	0.368	3.948	0.851	6	6503	ZINC000002022216

0.4	-9.9	-9.4	0.29	1.538	0.449	3.299	0.861	6	6504	ZINC000100829367
0.4	-8.8	-8.6	0.17	2.372	0.580	4.247	1.127	6	6505	ZINC000006358750
0.4	-6.0	-5.8	0.09	2.027	0.269	3.045	0.258	6	6506	ZINC000014888075
0.4	-6.7	-6.4	0.18	2.369	0.396	3.804	1.308	6	6507	ZINC000004556801
0.4	-5.0	-4.8	0.12	2.502	1.007	3.151	1.117	6	6508	ZINC000031351567
0.4	-6.1	-5.9	0.16	1.972	0.242	3.303	0.538	6	6509	ZINC000082402583
0.4	-6.7	-6.3	0.21	1.697	0.063	3.451	0.267	6	6510	ZINC000038235249
0.4	-8.1	-7.7	0.23	1.920	0.191	4.110	1.195	6	6511	ZINC000004544323
0.4	-8.5	-8.3	0.17	1.601	0.042	6.825	0.184	6	6512	ZINC000014491821
0.4	-8.5	-8.1	0.23	1.836	0.397	4.515	0.527	6	6513	ZINC000014587260
0.4	-6.9	-6.6	0.21	2.349	0.793	4.626	0.813	6	6514	ZINC000014814061
0.4	-4.6	-4.2	0.28	2.893	1.030	3.237	1.041	6	6515	ZINC000004262304
0.4	-7.2	-7.0	0.08	1.507	0.597	3.537	1.161	6	6516	ZINC000003777423
0.4	-6.6	-6.4	0.14	1.965	0.276	3.595	1.590	6	6517	ZINC000001848350
0.4	-8.3	-8.1	0.19	2.114	0.271	4.760	0.905	6	6518	ZINC000095620817

0.4	-7.2	-6.7	0.26	2.416	0.395	3.767	1.160	6	6519	ZINC000001532220
0.4	-5.9	-5.7	0.15	2.488	1.311	4.048	1.567	6	6520	ZINC000013549381
0.4	-7.5	-7.3	0.15	1.756	0.431	3.162	1.713	6	6521	ZINC000008469318
0.4	-5.7	-5.4	0.18	2.264	0.988	3.171	0.956	6	6522	ZINC000014616838
0.4	-7.6	-7.2	0.27	2.662	0.539	4.335	1.818	6	6523	ZINC000002583632
0.4	-6.3	-5.9	0.25	1.573	0.511	4.289	0.426	6	6524	ZINC000085972323
0.4	-9.4	-8.2	0.66	1.672	0.265	5.559	1.537	6	6525	ZINC000013385605
0.4	-7.1	-7.0	0.08	2.735	0.345	4.724	1.250	6	6526	ZINC000004899806
0.4	-8.6	-8.2	0.21	1.875	0.393	3.549	1.132	6	6527	ZINC000014613698
0.4	-7.8	-7.6	0.11	1.624	0.245	3.518	0.711	6	6528	ZINC000006031075
0.4	-6.4	-6.1	0.20	1.866	0.275	3.018	0.888	6	6529	ZINC000000487825
0.4	-6.2	-6.0	0.12	2.045	0.747	4.148	0.557	6	6530	ZINC000005273797
0.4	-6.5	-6.4	0.12	1.499	0.271	3.152	1.368	6	6531	ZINC000001687490
0.4	-7.4	-6.8	0.30	2.245	0.725	3.863	1.370	6	6532	ZINC000050027574
0.4	-5.6	-5.5	0.11	2.717	1.387	4.405	0.491	6	6533	ZINC000000154666

0.4	-9.5	-8.8	0.57	1.653	0.180	5.871	1.308	6	6534	ZINC000004424205
0.4	-7.2	-6.8	0.33	3.104	0.425	4.247	0.412	6	6535	ZINC000000388089
0.4	-6.5	-6.2	0.14	3.331	0.699	4.689	0.512	6	6536	ZINC000001581634
0.4	-8.4	-8.1	0.20	2.381	0.489	4.147	1.482	6	6537	ZINC000040439682
0.4	-8.9	-8.2	0.38	1.758	0.200	4.523	0.535	6	6538	ZINC000015220400
0.4	-10.4	-10.3	0.15	1.668	0.253	3.921	2.066	6	6539	ZINC000257468254
0.4	-7.9	-7.3	0.44	2.847	0.559	4.245	1.269	6	6540	ZINC000014455632
0.4	-9.7	-8.8	0.50	1.942	0.193	5.300	2.224	6	6541	ZINC000013374805
0.4	-7.5	-7.4	0.09	1.332	0.807	2.889	1.112	6	6542	ZINC000015251517
0.4	-6.3	-6.0	0.23	2.541	0.683	3.849	1.290	6	6543	ZINC000036092734
0.4	-9.6	-8.8	0.44	2.237	0.624	4.213	1.304	6	6544	ZINC000100779390
0.4	-8.8	-8.3	0.26	2.303	0.746	4.764	2.370	6	6545	ZINC000014517334
0.4	-6.5	-6.2	0.20	2.559	0.708	4.364	0.385	6	6546	ZINC000000159590
0.4	-8.7	-8.2	0.39	1.983	0.537	3.903	0.931	6	6547	ZINC000101885563
0.4	-8.2	-7.9	0.18	1.786	0.534	4.556	1.213	6	6548	ZINC000257637215

0.4	-6.6	-6.5	0.07	2.578	0.853	4.542	1.688	6	6549	ZINC000050026926
0.4	-6.2	-6.1	0.08	3.227	0.194	4.114	0.218	6	6550	ZINC000005334404
0.4	-7.0	-6.9	0.07	1.354	0.161	3.069	0.736	6	6551	ZINC000212507023
0.4	-8.1	-7.5	0.33	2.078	0.276	3.941	1.345	6	6552	ZINC000002575131
0.4	-6.8	-6.6	0.13	2.052	0.341	3.506	1.395	6	6553	ZINC000004974454
0.4	-5.9	-5.7	0.12	2.417	0.943	4.039	0.848	6	6554	ZINC000004262604
0.4	-10.0	-9.4	0.37	1.850	0.115	3.480	0.202	6	6555	ZINC000100782021
0.4	-6.1	-5.9	0.14	1.578	0.364	3.403	0.782	6	6556	ZINC000100828983
0.4	-9.4	-8.7	0.35	1.984	0.179	4.702	1.053	6	6557	ZINC000100782527
0.4	-8.1	-7.8	0.24	2.520	1.081	3.808	1.507	6	6558	ZINC000100029130
0.4	-6.5	-6.3	0.13	2.361	0.891	4.451	0.679	6	6559	ZINC000014590682
0.4	-9.0	-8.7	0.25	1.140	0.645	5.031	2.173	6	6560	ZINC000015251445
0.4	-9.1	-8.0	0.72	1.754	0.197	5.108	0.941	6	6561	ZINC000014489662
0.4	-10.0	-8.2	1.01	1.655	0.546	3.042	1.645	6	6562	ZINC000014416215
0.4	-9.4	-9.0	0.29	1.378	0.126	4.177	1.577	6	6563	ZINC000257388639

0.4	-5.7	-5.4	0.16	2.866	0.597	3.615	0.433	6	6564	ZINC000000394712
0.4	-7.1	-6.7	0.22	2.366	0.592	4.462	0.770	6	6565	ZINC000001664926
0.4	-7.6	-7.5	0.11	2.721	0.289	4.640	1.124	6	6566	ZINC000002149383
0.4	-7.8	-7.6	0.11	1.623	0.125	3.704	0.839	6	6567	ZINC000085661926
0.4	-7.0	-6.7	0.21	2.425	0.300	3.367	0.850	6	6568	ZINC000013481667
0.4	-10.0	-9.1	0.79	1.787	0.528	4.933	1.296	5	6569	ZINC000013308683
0.4	-9.6	-8.7	0.45	2.299	0.469	4.603	0.685	6	6570	ZINC000100779388
0.4	-7.5	-7.2	0.20	3.299	0.289	4.908	0.635	6	6571	ZINC000095617757
0.4	-6.5	-6.4	0.07	1.893	0.346	3.451	1.698	6	6572	ZINC000005274095
0.4	-6.1	-6.0	0.09	2.701	0.997	3.899	0.820	6	6573	ZINC000001690027
0.4	-4.4	-4.2	0.16	1.991	0.900	2.811	0.892	6	6574	ZINC000002020097
0.4	-9.5	-8.4	0.63	1.820	0.223	5.223	0.681	6	6575	ZINC000257374301
0.4	-7.1	-7.0	0.17	2.764	0.642	4.079	1.423	6	6576	ZINC000005112924
0.4	-8.2	-8.0	0.15	1.807	0.159	4.250	1.310	6	6577	ZINC000015256928
0.4	-5.7	-5.5	0.17	2.166	0.858	3.593	1.098	6	6578	ZINC000005735822

0.4	-4.9	-4.8	0.12	3.312	1.183	4.128	1.072	6	6579	ZINC000029786460
0.4	-6.3	-6.1	0.21	2.000	0.214	3.164	0.728	6	6580	ZINC000257497038
0.4	-8.7	-8.3	0.34	2.104	0.691	4.241	1.028	6	6581	ZINC000085644662
0.4	-5.5	-5.3	0.15	2.593	0.593	3.175	1.007	6	6582	ZINC000000895524
0.4	-5.8	-5.5	0.19	2.891	0.879	3.880	1.196	6	6583	ZINC000002017352
0.4	-7.1	-6.6	0.27	2.496	0.531	3.888	1.521	6	6584	ZINC000050026938
0.4	-6.8	-6.5	0.21	1.798	0.104	4.351	1.450	6	6585	ZINC000015122230
0.4	-9.7	-8.9	0.54	2.126	0.398	4.520	1.624	6	6586	ZINC000001531710
0.4	-5.3	-5.1	0.13	1.539	0.417	2.591	0.431	6	6587	ZINC000002528295
0.4	-8.6	-7.9	0.36	1.700	0.150	5.519	1.164	6	6588	ZINC000005358843
0.4	-4.8	-4.7	0.04	1.483	0.471	3.548	0.367	6	6589	ZINC000001632621
0.4	-5.7	-5.5	0.17	3.533	1.047	4.640	1.045	6	6590	ZINC000000005878
0.4	-7.6	-7.4	0.14	2.568	0.671	3.939	1.084	6	6591	ZINC000000336322
0.4	-7.8	-7.5	0.21	1.869	0.924	3.207	0.883	6	6592	ZINC000005082283
0.4	-6.9	-6.8	0.07	2.253	0.367	3.692	0.592	6	6593	ZINC000040484080

0.4	-7.6	-7.4	0.12	1.865	0.640	4.227	1.586	6	6594	ZINC000004543724
0.4	-8.9	-8.8	0.07	2.478	0.401	4.470	0.381	6	6595	ZINC000100646448
0.4	-9.1	-8.4	0.38	2.223	0.554	4.426	0.475	6	6596	ZINC000012153085
0.4	-8.1	-7.4	0.40	1.957	0.437	2.637	0.732	6	6597	ZINC000015248723
0.4	-7.3	-7.2	0.08	2.337	0.327	4.135	1.110	6	6598	ZINC000013514841
0.4	-9.7	-8.8	0.55	2.599	0.404	3.925	0.989	6	6599	ZINC000000898220
0.4	-8.6	-7.4	0.70	2.201	0.343	4.421	1.756	4	6600	ZINC000014820474
0.4	-7.5	-7.4	0.10	2.327	0.539	3.283	0.709	6	6601	ZINC000013305131
0.4	-8.4	-8.0	0.18	2.316	0.312	4.610	1.042	6	6602	ZINC000013408187
0.4	-9.0	-8.7	0.24	1.214	0.330	5.140	2.063	6	6603	ZINC000015251442
0.4	-9.4	-7.9	1.05	1.824	0.355	5.400	1.267	5	6604	ZINC000014645838
0.4	-4.5	-4.3	0.09	4.441	0.050	4.830	0.210	6	6605	ZINC000000407027
0.4	-7.2	-7.0	0.16	3.023	0.375	4.130	0.805	6	6606	ZINC000005765849
0.4	-7.5	-7.4	0.10	1.803	0.249	3.408	1.245	6	6607	ZINC000014556682
0.4	-7.9	-7.8	0.14	2.791	0.207	5.337	0.206	6	6608	ZINC000013513777

0.4	-7.1	-7.1	0.04	0.655	0.779	3.112	0.727	6	6609	ZINC000000967522
0.4	-7.0	-6.8	0.11	2.645	0.672	4.580	0.917	6	6610	ZINC000034266412
0.4	-9.6	-8.0	1.20	2.735	0.975	5.402	1.681	5	6611	ZINC000012496221
0.4	-4.8	-4.7	0.11	2.069	0.957	4.027	0.424	6	6612	ZINC000005650711
0.4	-7.5	-7.1	0.25	1.824	0.271	3.037	1.544	6	6613	ZINC000014657918
0.4	-8.1	-7.7	0.21	2.704	0.585	5.074	1.469	6	6614	ZINC000018155852
0.4	-8.0	-7.7	0.31	1.496	0.253	4.239	2.174	6	6615	ZINC000015259908
0.4	-5.0	-4.9	0.06	1.706	0.541	3.405	0.613	6	6616	ZINC000001602836
0.4	-6.8	-6.4	0.17	2.852	1.041	4.139	1.487	6	6617	ZINC000095618101
0.4	-9.4	-9.1	0.25	1.384	0.125	3.357	1.418	6	6618	ZINC000257388637
0.4	-9.8	-9.1	0.46	1.878	0.634	4.410	0.942	6	6619	ZINC000014885064
0.4	-9.1	-8.8	0.19	1.553	0.266	3.024	0.964	6	6620	ZINC000256430597
0.4	-5.9	-5.6	0.20	3.269	1.701	5.608	1.168	6	6621	ZINC000014453730
0.4	-6.9	-6.6	0.15	2.693	0.424	4.286	0.916	6	6622	ZINC000050027444
0.4	-7.5	-7.1	0.31	2.801	1.116	4.982	1.988	6	6623	ZINC000252444102

0.4	-9.1	-8.4	0.35	2.314	0.560	4.657	0.530	6	6624	ZINC000012153084
0.4	-7.1	-7.0	0.09	2.631	0.984	4.007	1.227	6	6625	ZINC000014855858
0.4	-6.1	-6.0	0.11	2.491	0.538	4.212	1.073	6	6626	ZINC000033639584
0.4	-4.8	-4.5	0.20	2.263	0.345	3.805	0.750	6	6627	ZINC000000391109
0.4	-7.5	-7.3	0.20	1.823	0.581	4.022	1.235	6	6628	ZINC000014655151
0.4	-8.5	-7.5	0.52	2.484	0.090	6.209	0.217	6	6629	ZINC000014490904
0.4	-4.9	-4.8	0.11	2.490	0.991	3.132	0.958	6	6630	ZINC000001663923
0.4	-7.1	-6.8	0.16	2.130	0.435	3.007	0.895	6	6631	ZINC000001605722
0.4	-9.0	-8.5	0.41	2.028	0.376	4.135	1.031	6	6632	ZINC000100780451
0.4	-8.5	-8.2	0.12	2.267	0.678	3.784	1.118	6	6633	ZINC000000080812
0.4	-5.2	-5.0	0.13	2.520	0.732	3.586	0.828	6	6634	ZINC000005956847
0.4	-6.6	-6.3	0.15	1.948	0.972	4.008	1.390	6	6635	ZINC000032163572
0.4	-8.5	-8.2	0.24	1.781	0.555	2.670	1.285	6	6636	ZINC000257467836
0.4	-6.7	-6.6	0.12	3.033	0.894	4.890	1.512	6	6637	ZINC000006072072
0.4	-6.9	-6.5	0.18	2.956	1.321	3.998	1.358	6	6638	ZINC000000034157

0.4	-8.6	-8.1	0.42	1.937	0.159	6.415	1.348	6	6639	ZINC000100775370
0.4	-8.3	-7.7	0.38	1.855	0.170	3.548	0.992	6	6640	ZINC000013783206
0.4	-8.1	-7.8	0.18	2.850	0.413	4.556	1.066	6	6641	ZINC000100390124
0.4	-5.0	-4.8	0.12	2.361	0.566	4.095	0.899	6	6642	ZINC000014593620
0.4	-6.5	-6.2	0.14	2.984	0.393	4.687	0.488	6	6643	ZINC000050027696
0.4	-10.4	-10.3	0.15	1.681	0.245	3.943	2.048	6	6644	ZINC000257468252
0.4	-9.0	-7.9	0.60	2.221	0.588	4.512	1.591	6	6645	ZINC000000056792
0.4	-5.8	-5.5	0.15	2.763	0.181	4.133	0.427	6	6646	ZINC000005838789
0.4	-9.2	-8.5	0.43	2.328	0.295	3.514	1.453	6	6647	ZINC000014919091
0.4	-6.6	-6.5	0.12	2.494	0.850	3.887	1.170	6	6648	ZINC000005447649
0.4	-8.6	-8.2	0.20	1.618	0.428	4.087	1.512	6	6649	ZINC000014503461
0.4	-6.3	-6.0	0.23	1.446	0.861	3.962	0.987	6	6650	ZINC000005783661
0.4	-8.9	-8.1	0.59	1.590	0.313	5.746	1.553	6	6651	ZINC000014858772
0.4	-5.0	-4.8	0.11	2.199	0.881	3.498	0.749	6	6652	ZINC000026673320
0.4	-6.1	-5.7	0.27	1.660	0.428	4.320	0.559	6	6653	ZINC000001677794

0.4	-9.5	-8.8	0.39	1.226	0.299	3.729	1.512	6	6654	ZINC000018038506
0.4	-8.7	-7.9	0.37	2.559	0.137	4.964	1.503	6	6655	ZINC000014687097
0.4	-7.3	-7.0	0.14	2.278	0.162	4.388	1.696	6	6656	ZINC000033976927
0.4	-5.4	-5.3	0.12	0.907	0.378	2.887	0.475	6	6657	ZINC000000967330
0.4	-7.9	-7.6	0.17	2.414	0.336	3.866	1.183	6	6658	ZINC000000066104
0.4	-7.2	-6.7	0.24	1.515	0.504	2.939	1.640	6	6659	ZINC000095099394
0.4	-6.1	-5.9	0.16	2.837	0.805	3.958	0.633	6	6660	ZINC000000120249
0.4	-7.7	-7.5	0.13	2.290	0.212	4.738	0.381	6	6661	ZINC000027647171
0.4	-7.8	-7.4	0.18	2.672	0.767	5.165	1.231	6	6662	ZINC000257534536
0.4	-7.9	-6.7	0.64	2.426	0.386	4.613	1.646	6	6663	ZINC000002020491
0.4	-6.7	-6.4	0.17	1.642	0.260	3.555	0.828	6	6664	ZINC000002040977
0.4	-7.6	-7.5	0.16	3.480	0.597	5.456	1.320	6	6665	ZINC000003872189
0.4	-8.1	-7.7	0.23	1.848	0.088	3.648	0.864	6	6666	ZINC000257476498
0.4	-7.1	-6.8	0.19	2.294	0.225	3.442	1.281	6	6667	ZINC000050027235
0.4	-7.0	-6.7	0.22	1.580	0.833	2.948	0.725	6	6668	ZINC000000388593

0.4	-8.7	-8.1	0.49	1.794	0.184	3.168	0.420	6	6669	ZINC000095620007
0.4	-7.3	-7.0	0.14	1.948	0.645	2.644	0.871	6	6670	ZINC000001563568
0.4	-5.6	-5.3	0.19	1.818	0.542	2.745	0.366	6	6671	ZINC000014495038
0.4	-5.9	-5.5	0.16	1.883	0.186	2.952	0.743	6	6672	ZINC000000157146
0.4	-6.0	-5.8	0.14	1.655	0.533	3.294	0.920	6	6673	ZINC000014860574
0.4	-7.6	-7.5	0.12	1.828	0.329	5.177	1.939	6	6674	ZINC000059067345
0.4	-5.6	-5.3	0.21	2.366	0.747	3.037	0.934	6	6675	ZINC000001648303
0.4	-5.9	-5.6	0.16	2.283	0.623	3.852	0.574	6	6676	ZINC000001532658
0.4	-5.2	-5.1	0.09	1.647	0.488	2.849	0.278	6	6677	ZINC000000001886
0.4	-5.7	-5.6	0.11	1.939	0.532	3.659	0.603	6	6678	ZINC000014616805
0.4	-5.9	-5.7	0.07	3.109	0.550	4.597	0.952	6	6679	ZINC000001711140
0.4	-6.5	-6.4	0.09	1.928	0.154	4.209	0.616	6	6680	ZINC000039090696
0.4	-6.5	-6.2	0.20	1.905	0.869	2.823	1.537	6	6681	ZINC000034294020
0.4	-4.8	-4.6	0.12	2.952	1.108	3.685	1.188	6	6682	ZINC000039067134
0.4	-6.4	-6.1	0.14	2.574	0.623	3.514	1.306	6	6683	ZINC000000895834

0.4	-7.5	-7.3	0.15	2.359	0.353	4.038	1.148	6	6684	ZINC000095617716
0.4	-5.9	-5.8	0.09	2.190	0.351	4.515	0.219	6	6685	ZINC000050026968
0.4	-8.6	-7.7	0.67	1.978	0.470	4.796	1.748	6	6686	ZINC000013359969
0.4	-8.5	-8.1	0.28	1.967	0.108	3.813	1.053	6	6687	ZINC000013330544
0.4	-7.4	-7.2	0.13	2.633	0.310	3.576	1.048	6	6688	ZINC000040470272
0.4	-5.9	-5.8	0.07	3.006	0.712	3.820	0.459	6	6689	ZINC000001850550
0.4	-8.6	-7.1	1.19	3.127	0.148	4.264	0.692	3	6690	ZINC000006484843
0.4	-4.7	-4.4	0.19	2.372	0.921	3.752	0.928	6	6691	ZINC000000388765
0.4	-7.2	-6.9	0.20	1.924	0.478	3.287	0.685	6	6692	ZINC000140122154
0.4	-9.2	-7.7	0.74	1.849	0.074	5.074	0.884	6	6693	ZINC000014762895
0.4	-8.9	-8.2	0.37	2.657	0.563	4.492	1.269	6	6694	ZINC000008215481
0.4	-5.7	-5.3	0.20	2.593	0.352	4.345	0.510	6	6695	ZINC000095618221
0.4	-8.1	-7.3	0.53	2.290	0.324	3.525	1.572	6	6696	ZINC000014713572
0.4	-7.2	-6.9	0.16	1.897	0.361	4.071	1.039	6	6697	ZINC000014822753
0.4	-5.6	-5.4	0.09	2.444	1.334	3.996	1.151	6	6698	ZINC000001850487

0.4	-7.6	-7.3	0.16	2.339	0.466	4.284	1.547	6	6699	ZINC000002124468
0.4	-7.5	-7.0	0.23	2.349	0.573	4.283	1.703	6	6700	ZINC000002168694
0.4	-6.1	-6.0	0.18	1.709	0.420	3.249	0.274	6	6701	ZINC000001850962
0.4	-7.4	-7.2	0.12	2.630	0.531	4.635	1.002	6	6702	ZINC000257437883
0.4	-5.8	-5.8	0.05	1.624	0.494	4.525	1.296	6	6703	ZINC000033839261
0.4	-10.4	-10.3	0.12	1.668	0.252	3.921	2.066	6	6704	ZINC000257468253
0.4	-7.1	-7.0	0.05	2.016	0.485	4.565	1.376	6	6705	ZINC000095620796
0.4	-7.6	-7.3	0.18	1.654	0.154	4.040	1.089	6	6706	ZINC000004096987
0.4	-10.1	-8.6	0.80	2.225	0.044	5.375	0.867	6	6707	ZINC000257421588
0.4	-8.9	-8.6	0.30	1.926	0.464	5.015	1.651	6	6708	ZINC000014805960
0.4	-6.4	-6.2	0.12	2.581	0.186	4.901	0.908	6	6709	ZINC000002516111
0.4	-6.1	-5.9	0.12	2.700	0.854	4.148	0.936	6	6710	ZINC000005131760
0.4	-4.9	-4.8	0.14	1.056	0.845	2.550	0.356	6	6711	ZINC000000967532
0.4	-8.4	-8.0	0.27	1.752	0.635	4.797	1.743	6	6712	ZINC000013376986
0.4	-6.3	-6.2	0.09	1.495	0.379	3.018	0.935	6	6713	ZINC000077050244

0.4	-9.0	-7.9	0.98	1.608	0.267	4.091	1.827	3	6714	ZINC000014587140
0.4	-5.7	-5.5	0.11	1.953	0.746	4.598	0.494	6	6715	ZINC000002036909
0.4	-7.6	-7.4	0.15	1.940	0.516	3.006	1.549	6	6716	ZINC000005633008
0.4	-6.0	-5.8	0.11	2.195	1.065	3.902	1.103	6	6717	ZINC000012405252
0.4	-8.9	-8.7	0.17	1.742	0.234	5.459	0.999	6	6718	ZINC000015249355
0.4	-8.2	-7.7	0.40	2.542	0.748	3.443	0.998	6	6719	ZINC000003594299
0.4	-5.4	-5.1	0.14	1.597	0.358	3.114	0.533	6	6720	ZINC000000388596
0.4	-9.3	-8.7	0.44	1.847	0.199	4.508	0.615	6	6721	ZINC000013383388
0.4	-6.4	-6.0	0.21	2.782	0.228	4.923	0.469	6	6722	ZINC000001763955
0.4	-10.5	-9.8	0.50	2.322	1.511	3.721	1.731	6	6723	ZINC000100091860
0.4	-9.7	-8.7	0.74	2.343	0.299	4.405	0.472	6	6724	ZINC000085730714
0.4	-8.1	-8.0	0.09	2.701	0.301	4.335	0.335	6	6725	ZINC000001530233
0.4	-6.1	-5.8	0.15	3.066	0.990	4.875	0.874	6	6726	ZINC000095620556
0.4	-7.5	-7.3	0.18	1.640	0.488	3.373	0.448	6	6727	ZINC000100073014
0.4	-7.6	-7.1	0.27	3.675	0.628	4.935	0.435	6	6728	ZINC000100006441

0.4	-7.5	-7.1	0.26	2.216	0.350	3.629	0.376	6	6729	ZINC000031475161
0.4	-7.8	-7.5	0.18	1.820	0.547	4.203	1.693	6	6730	ZINC000012496517
0.4	-5.5	-5.3	0.11	2.467	0.957	3.772	0.856	6	6731	ZINC000031302473
0.4	-6.5	-6.2	0.21	2.159	1.140	2.926	0.826	6	6732	ZINC000001747839
0.4	-7.4	-7.2	0.16	1.406	0.210	3.252	0.568	6	6733	ZINC000085973574
0.4	-4.3	-4.2	0.09	3.140	1.476	3.994	1.140	6	6734	ZINC000031274105
0.4	-5.0	-4.8	0.11	2.564	0.448	2.947	0.376	6	6735	ZINC000000152265
0.4	-8.7	-8.2	0.30	2.277	0.665	3.857	1.425	6	6736	ZINC000002384770
0.4	-7.7	-7.5	0.13	2.218	0.537	4.705	0.838	6	6737	ZINC000014619890
0.3	-7.8	-7.6	0.26	1.057	0.749	4.330	0.631	6	6738	ZINC000001481993
0.3	-8.1	-7.9	0.23	2.141	0.498	5.771	1.566	6	6739	ZINC000014720631
0.3	-7.9	-7.6	0.31	1.864	0.382	4.306	2.106	6	6740	ZINC000014886646
0.3	-9.5	-8.2	0.99	2.056	0.519	4.272	1.162	6	6741	ZINC000253595813
0.3	-6.6	-6.3	0.21	2.103	0.266	3.529	0.723	6	6742	ZINC000002557912
0.3	-6.9	-6.7	0.14	2.968	0.430	4.793	1.559	6	6743	ZINC000095617681

0.3	-4.9	-4.6	0.17	1.949	0.636	2.329	0.700	6	6744	ZINC000001699905
0.3	-3.9	-3.8	0.08	1.697	0.755	3.359	0.451	6	6745	ZINC000001718826
0.3	-9.2	-8.7	0.37	2.463	0.456	4.141	0.561	6	6746	ZINC000013412791
0.3	-9.1	-8.7	0.32	1.622	0.278	3.619	1.605	6	6747	ZINC000257430348
0.3	-8.7	-8.5	0.09	1.782	0.223	2.764	0.628	6	6748	ZINC000085795105
0.3	-5.2	-5.0	0.13	3.217	0.943	3.660	1.041	6	6749	ZINC000001558385
0.3	-5.0	-4.9	0.07	2.177	0.740	3.813	0.338	6	6750	ZINC000002510291
0.3	-8.4	-8.1	0.27	1.218	0.695	5.106	2.388	6	6751	ZINC000015251399
0.3	-7.2	-6.8	0.25	1.554	0.528	2.871	1.364	6	6752	ZINC000105057710
0.3	-8.2	-7.7	0.43	1.786	0.303	5.284	1.583	6	6753	ZINC000100781995
0.3	-6.1	-5.8	0.21	2.070	0.656	3.419	0.745	6	6754	ZINC000001655444
0.3	-9.4	-8.9	0.34	1.833	0.671	4.451	2.040	6	6755	ZINC000014811604
0.3	-6.8	-6.6	0.09	2.382	0.668	3.383	1.226	6	6756	ZINC000001850350
0.3	-7.7	-7.5	0.24	1.996	0.281	3.522	0.950	6	6757	ZINC000015148010
0.3	-7.6	-7.5	0.12	2.032	0.344	5.167	1.606	6	6758	ZINC000014590629

0.3	-6.9	-6.7	0.24	1.097	0.514	1.851	0.102	6	6759	ZINC000002037656
0.3	-7.7	-7.5	0.19	1.988	0.235	5.364	0.963	6	6760	ZINC000013374016
0.3	-8.6	-8.4	0.16	2.265	0.635	4.398	1.699	6	6761	ZINC000014811580
0.3	-7.5	-7.3	0.09	2.442	0.322	4.458	0.953	6	6762	ZINC000003869416
0.3	-7.1	-6.9	0.17	1.698	0.657	3.081	1.083	6	6763	ZINC000001850820
0.3	-9.4	-8.7	0.49	2.202	0.790	4.294	2.115	6	6764	ZINC000014691900
0.3	-4.4	-4.0	0.22	2.083	0.580	3.647	0.722	6	6765	ZINC000000388088
0.3	-6.5	-6.2	0.15	2.961	0.528	4.371	0.521	6	6766	ZINC000050026846
0.3	-8.3	-8.1	0.17	2.613	0.557	4.348	1.148	6	6767	ZINC000034290097
0.3	-7.9	-7.7	0.11	2.349	0.572	3.707	1.543	6	6768	ZINC000257726660
0.3	-9.1	-8.1	0.60	1.598	0.565	4.442	0.797	6	6769	ZINC000001568212
0.3	-7.1	-6.9	0.12	1.897	0.806	2.940	0.700	6	6770	ZINC000003847413
0.3	-7.0	-6.7	0.24	2.238	0.626	4.018	1.881	6	6771	ZINC000040470873
0.3	-8.2	-7.8	0.34	1.343	0.080	4.467	1.536	6	6772	ZINC000015113768
0.3	-4.9	-4.8	0.10	3.353	1.629	4.265	1.036	6	6773	ZINC000000003183

0.3	-6.6	-6.5	0.05	2.261	0.737	3.408	1.571	6	6774	ZINC000002384834
0.3	-5.5	-5.2	0.16	1.889	0.630	2.831	0.316	6	6775	ZINC000013462028
0.3	-5.6	-5.3	0.18	2.923	0.896	4.113	1.055	6	6776	ZINC000002015857
0.3	-9.0	-8.7	0.23	1.647	0.312	3.761	1.541	6	6777	ZINC000013143014
0.3	-6.0	-5.8	0.15	1.116	0.151	4.217	0.770	6	6778	ZINC000003869786
0.3	-8.5	-7.6	0.60	1.977	0.303	4.504	1.154	6	6779	ZINC000015274237
0.3	-7.7	-7.4	0.21	2.699	0.767	4.997	1.031	6	6780	ZINC000257534537
0.3	-7.8	-7.4	0.34	2.486	0.883	3.617	1.442	6	6781	ZINC000000083315
0.3	-7.3	-7.1	0.13	2.147	0.164	4.921	2.049	6	6782	ZINC000095617690
0.3	-8.8	-8.6	0.22	1.718	0.573	3.021	0.541	6	6783	ZINC000014502861
0.3	-4.1	-3.9	0.17	1.537	0.572	2.556	0.407	6	6784	ZINC000100828525
0.3	-5.5	-5.3	0.11	2.154	1.256	4.266	1.109	6	6785	ZINC000000895418
0.3	-7.4	-6.5	0.53	2.402	0.363	4.294	1.144	6	6786	ZINC000034126346
0.3	-6.1	-5.8	0.21	2.584	1.065	3.576	1.208	6	6787	ZINC000013437581
0.3	-4.7	-4.5	0.13	2.336	0.908	3.657	0.696	6	6788	ZINC000001640814

0.3	-6.0	-5.8	0.15	1.851	0.611	2.961	0.808	6	6789	ZINC000004995651
0.3	-4.9	-4.8	0.07	1.780	0.324	2.780	0.835	6	6790	ZINC000002034329
0.3	-8.8	-8.4	0.23	1.977	0.092	6.442	0.449	6	6791	ZINC000257497783
0.3	-9.8	-9.4	0.26	2.230	0.901	5.204	1.194	6	6792	ZINC000085996405
0.3	-6.0	-5.9	0.10	1.948	0.453	3.440	0.525	6	6793	ZINC000000049154
0.3	-6.2	-6.0	0.13	2.554	0.656	4.559	0.585	6	6794	ZINC000003804392
0.3	-7.1	-6.9	0.19	2.183	0.648	3.009	0.820	6	6795	ZINC000006576154
0.3	-8.1	-7.7	0.29	1.720	0.236	3.806	0.988	6	6796	ZINC000012153223
0.3	-8.8	-8.0	0.39	1.757	0.358	5.083	1.448	6	6797	ZINC000257435880
0.3	-6.6	-6.3	0.19	3.062	0.620	3.791	0.434	6	6798	ZINC000000393338
0.3	-4.8	-4.7	0.07	3.190	0.639	3.679	0.388	6	6799	ZINC000000405269
0.3	-8.5	-8.0	0.31	1.892	0.255	4.251	1.442	6	6800	ZINC000085837039
0.3	-7.0	-6.6	0.22	1.704	0.093	2.237	0.178	6	6801	ZINC000004533965
0.3	-7.2	-7.0	0.15	1.878	0.614	4.606	1.387	6	6802	ZINC000000052601
0.3	-4.8	-4.7	0.09	2.425	0.756	3.376	1.329	6	6803	ZINC000002389918

0.3	-6.6	-6.4	0.16	2.959	0.878	4.065	0.995	6	6804	ZINC000001576324
0.3	-7.0	-6.6	0.29	2.887	0.499	3.770	0.637	6	6805	ZINC000000056568
0.3	-9.3	-7.9	0.85	1.891	0.301	5.919	0.798	6	6806	ZINC000013411697
0.3	-6.1	-5.6	0.27	2.882	0.607	3.654	0.675	6	6807	ZINC000001677572
0.3	-6.0	-5.6	0.23	2.223	0.864	2.768	0.828	6	6808	ZINC000000157108
0.3	-7.2	-6.9	0.24	1.916	0.349	5.038	2.087	6	6809	ZINC000005955038
0.3	-6.4	-6.2	0.13	2.341	0.677	4.487	0.860	6	6810	ZINC000095618125
0.3	-5.6	-5.4	0.11	3.321	1.376	5.502	0.937	6	6811	ZINC000095619620
0.3	-8.7	-8.2	0.31	1.440	0.415	3.039	1.577	6	6812	ZINC000015046973
0.3	-5.6	-5.5	0.09	2.069	0.250	3.666	0.599	6	6813	ZINC000002164365
0.3	-6.6	-6.3	0.14	2.386	0.453	3.161	0.454	6	6814	ZINC000001531990
0.3	-6.3	-6.2	0.07	1.739	0.300	3.704	0.678	6	6815	ZINC000005132601
0.3	-8.3	-8.0	0.24	1.821	0.448	4.344	1.338	6	6816	ZINC000257517243
0.3	-5.6	-5.4	0.10	4.119	1.820	4.586	1.906	6	6817	ZINC000012366795
0.3	-7.4	-6.9	0.47	1.841	0.966	3.148	1.588	6	6818	ZINC000001719254

0.3	-5.3	-5.1	0.16	4.240	2.239	4.812	2.265	6	6819	ZINC000004095756
0.3	-5.2	-5.0	0.19	2.520	1.546	3.007	1.735	6	6820	ZINC000003581355
0.3	-8.3	-7.9	0.21	2.234	0.635	4.507	1.112	6	6821	ZINC000005925403
0.3	-3.6	-3.5	0.07	1.230	0.419	2.596	0.317	6	6822	ZINC000008214699
0.3	-5.2	-5.0	0.11	2.522	1.088	3.258	1.023	6	6823	ZINC000002575098
0.3	-5.2	-4.9	0.23	1.972	0.289	3.054	0.464	6	6824	ZINC000000158173
0.3	-7.0	-6.6	0.25	2.951	0.670	4.542	1.143	6	6825	ZINC000005273947
0.3	-6.4	-6.3	0.07	1.786	0.294	3.474	1.202	6	6826	ZINC000086034643
0.3	-7.6	-7.4	0.17	1.931	0.418	3.775	1.111	6	6827	ZINC000034961796
0.3	-7.1	-6.8	0.21	1.737	0.416	5.278	1.659	6	6828	ZINC000014858403
0.3	-6.2	-5.8	0.19	2.792	1.397	4.217	1.316	6	6829	ZINC000001843030
0.3	-8.4	-8.0	0.19	2.047	0.190	5.096	1.102	6	6830	ZINC000257374611
0.3	-8.7	-8.3	0.24	2.868	0.734	4.647	0.998	6	6831	ZINC000001871563
0.3	-5.4	-5.1	0.18	2.739	1.314	3.685	1.423	6	6832	ZINC000000155364
0.3	-8.8	-8.5	0.19	1.943	0.538	4.239	1.020	6	6833	ZINC000140682730

0.3	-9.3	-8.9	0.31	2.278	0.115	5.020	0.878	6	6834	ZINC000014636784
0.3	-8.7	-8.2	0.33	1.665	0.842	3.948	1.474	6	6835	ZINC000014504476
0.3	-7.0	-6.8	0.17	1.407	0.774	2.543	1.416	6	6836	ZINC000001691037
0.3	-8.3	-7.9	0.26	1.806	0.386	3.233	0.966	6	6837	ZINC000006070277
0.3	-7.6	-7.5	0.11	2.185	0.399	2.912	0.675	6	6838	ZINC000013514189
0.3	-7.7	-7.4	0.14	2.929	0.448	5.167	0.426	6	6839	ZINC000095619751
0.3	-4.3	-3.9	0.21	2.493	0.868	3.159	1.025	6	6840	ZINC000001850875
0.3	-5.9	-5.8	0.09	1.526	0.380	3.608	0.206	6	6841	ZINC000001530146
0.3	-6.0	-5.7	0.20	2.626	0.793	3.604	0.748	6	6842	ZINC000001577061
0.3	-6.2	-6.1	0.09	2.118	1.168	3.775	1.436	6	6843	ZINC000002558026
0.3	-6.2	-5.9	0.16	1.813	0.810	3.077	0.697	6	6844	ZINC000000389865
0.3	-10.1	-9.2	0.42	1.795	0.320	3.948	0.687	6	6845	ZINC000003644819
0.3	-5.7	-5.4	0.16	2.399	0.378	3.534	0.643	6	6846	ZINC000002570846
0.3	-6.1	-5.9	0.17	3.013	0.946	4.652	1.145	6	6847	ZINC000016697731
0.3	-5.8	-5.7	0.09	2.737	1.391	4.752	0.696	6	6848	ZINC000006361277

0.3	-7.1	-6.9	0.25	2.863	0.479	4.749	1.412	6	6849	ZINC000015261395
0.3	-6.6	-6.5	0.05	2.917	0.917	4.029	1.121	6	6850	ZINC000033951295
0.3	-6.6	-6.3	0.24	2.449	1.004	4.556	1.521	6	6851	ZINC000000077999
0.3	-8.2	-8.0	0.19	1.657	0.188	3.013	0.307	6	6852	ZINC000247949810
0.3	-9.5	-8.9	0.62	1.523	0.187	5.453	1.414	6	6853	ZINC000257438143
0.3	-8.5	-8.0	0.36	1.906	0.212	4.818	1.700	6	6854	ZINC000257402424
0.3	-6.5	-6.3	0.18	2.212	0.706	2.962	0.664	6	6855	ZINC000257692310
0.3	-10.4	-9.9	0.58	1.904	0.876	4.319	1.559	6	6856	ZINC000100780458
0.3	-5.7	-5.4	0.22	3.139	0.611	4.448	0.371	6	6857	ZINC000019364244
0.3	-6.3	-6.0	0.18	2.593	0.398	4.184	1.033	6	6858	ZINC000033841553
0.3	-6.5	-6.2	0.17	2.607	0.557	4.403	1.063	6	6859	ZINC000050026910
0.3	-6.3	-6.2	0.07	2.897	0.482	4.516	1.090	6	6860	ZINC000040473160
0.3	-7.0	-6.6	0.23	1.829	0.300	2.472	0.515	6	6861	ZINC000001605723
0.3	-8.9	-8.2	0.94	1.856	0.670	3.132	1.106	4	6862	ZINC000014410364
0.3	-5.3	-5.2	0.11	2.459	0.556	3.386	0.705	6	6863	ZINC000005956067

0.3	-6.0	-5.9	0.07	1.651	0.213	3.705	0.830	6	6864	ZINC000028631770
0.3	-8.9	-7.7	0.56	2.664	0.199	4.827	0.846	6	6865	ZINC000014820396
0.3	-10.0	-9.0	0.58	1.474	0.177	3.848	1.292	6	6866	ZINC000100257509
0.3	-7.4	-7.2	0.12	1.653	0.646	3.747	0.597	6	6867	ZINC000006119185
0.3	-6.9	-6.9	0.05	2.321	0.751	3.660	1.133	6	6868	ZINC000000391936
0.3	-8.9	-8.0	0.42	1.634	0.014	3.920	0.671	6	6869	ZINC000253592133
0.3	-8.2	-8.1	0.09	1.802	0.157	3.798	0.842	6	6870	ZINC000015120817
0.3	-7.9	-7.5	0.24	2.339	0.447	4.413	1.615	6	6871	ZINC000040479042
0.3	-6.7	-6.4	0.25	2.760	0.330	4.513	0.851	6	6872	ZINC000032787789
0.3	-6.7	-6.5	0.18	1.934	0.218	2.673	0.391	6	6873	ZINC000033963311
0.3	-6.5	-6.3	0.10	2.806	1.140	4.098	0.929	6	6874	ZINC000000391789
0.3	-5.5	-5.2	0.16	3.125	0.433	4.381	0.992	6	6875	ZINC000014616381
0.3	-6.9	-6.6	0.17	2.171	0.433	3.163	1.060	6	6876	ZINC000004556775
0.3	-8.2	-7.6	0.28	3.022	0.323	4.985	1.083	6	6877	ZINC000050027617
0.3	-8.0	-7.5	0.38	2.453	0.793	4.036	1.656	6	6878	ZINC000038338719

0.3	-5.8	-5.6	0.15	1.255	0.680	4.144	0.186	6	6879	ZINC000003861121
0.3	-5.2	-5.0	0.13	2.122	0.687	3.151	0.943	6	6880	ZINC000002040992
0.3	-8.4	-8.1	0.20	1.861	0.624	3.078	1.521	6	6881	ZINC000014859994
0.3	-9.2	-8.3	0.55	2.032	0.601	4.918	0.957	6	6882	ZINC000100780325
0.3	-8.5	-7.9	0.35	1.635	0.194	3.825	0.619	6	6883	ZINC000015060165
0.3	-7.6	-7.5	0.05	2.317	0.487	4.382	1.277	6	6884	ZINC000014855868
0.3	-8.5	-7.9	0.30	2.373	0.182	4.531	0.557	6	6885	ZINC000032209980
0.3	-8.0	-7.8	0.11	2.226	0.560	3.761	1.183	6	6886	ZINC000030728760
0.3	-7.6	-7.1	0.28	1.946	0.181	4.562	1.218	6	6887	ZINC000043762293
0.3	-9.0	-8.2	0.43	2.178	0.356	2.824	0.586	6	6888	ZINC000100825565
0.3	-9.2	-8.1	0.55	2.155	0.580	5.398	0.988	6	6889	ZINC000257470192
0.3	-5.5	-5.4	0.09	2.071	0.557	2.983	0.687	6	6890	ZINC000001596545
0.3	-5.0	-4.8	0.11	2.507	0.529	3.498	0.846	6	6891	ZINC000001529524
0.3	-7.8	-7.3	0.33	1.876	0.372	2.869	0.677	6	6892	ZINC000040484032
0.3	-8.6	-7.9	0.43	2.456	0.390	5.254	1.282	6	6893	ZINC000014504817

0.3	-7.7	-7.4	0.13	2.473	0.239	3.560	0.613	6	6894	ZINC000014713560
0.3	-7.1	-6.9	0.12	3.560	0.649	5.078	0.701	6	6895	ZINC000013372516
0.3	-5.3	-5.0	0.15	4.989	1.611	5.709	1.798	6	6896	ZINC000000002005
0.3	-8.1	-7.3	0.49	2.284	1.015	3.486	1.886	6	6897	ZINC000001569733
0.3	-7.6	-7.0	0.36	1.604	0.441	3.672	1.044	6	6898	ZINC000085589975
0.3	-5.9	-5.8	0.05	1.327	0.572	4.031	0.938	6	6899	ZINC000001648298
0.3	-10.3	-9.0	1.30	2.044	0.000	4.981	0.000	2	6900	ZINC000014688348
0.3	-6.8	-6.6	0.21	1.895	0.884	3.213	0.708	6	6901	ZINC000000002151
0.3	-7.2	-7.0	0.18	2.413	0.343	4.324	1.126	6	6902	ZINC000034961767
0.3	-6.7	-6.5	0.18	2.436	0.443	4.317	1.256	6	6903	ZINC000040439628
0.3	-7.8	-7.6	0.17	1.928	0.694	3.630	0.603	6	6904	ZINC000104359755
0.3	-6.0	-5.9	0.09	1.969	1.028	2.738	0.892	6	6905	ZINC000034828682
0.3	-8.5	-8.3	0.09	1.933	0.214	3.326	1.775	6	6906	ZINC000013524192
0.3	-5.7	-5.4	0.23	1.960	0.329	3.424	0.132	6	6907	ZINC000001656422
0.3	-8.0	-7.4	0.38	1.936	0.449	2.520	0.581	6	6908	ZINC000001605730

0.3	-7.9	-7.7	0.20	1.964	0.526	4.487	1.484	6	6909	ZINC000001532132
0.3	-7.6	-7.4	0.12	2.983	0.762	5.154	1.310	6	6910	ZINC000003872191
0.3	-9.2	-8.7	0.35	1.794	0.492	4.547	1.445	6	6911	ZINC000100771519
0.3	-8.9	-8.7	0.22	1.520	0.726	5.105	2.391	6	6912	ZINC000085795076
0.3	-7.4	-7.2	0.13	2.226	0.478	4.453	1.474	6	6913	ZINC000012496115
0.3	-8.2	-8.0	0.21	1.709	0.189	2.818	0.264	6	6914	ZINC000000851689
0.3	-7.1	-6.9	0.16	2.506	0.234	4.463	0.901	6	6915	ZINC000034266417
0.3	-6.3	-6.0	0.20	2.574	0.719	3.634	1.030	6	6916	ZINC000001850745
0.3	-8.6	-8.5	0.07	1.370	0.740	2.127	0.372	6	6917	ZINC000261499721
0.3	-9.1	-8.2	0.55	2.875	0.387	4.515	0.281	6	6918	ZINC000013330536
0.3	-6.4	-6.1	0.21	3.717	0.486	5.609	0.505	6	6919	ZINC000014491752
0.3	-7.5	-7.3	0.12	1.824	0.728	3.516	1.670	6	6920	ZINC000095620802
0.3	-9.6	-8.9	0.48	2.227	0.642	4.110	1.515	6	6921	ZINC000100068844
0.3	-5.3	-5.0	0.14	2.608	0.602	3.573	0.924	6	6922	ZINC000000159604
0.3	-7.5	-7.3	0.09	2.128	0.751	4.110	1.615	6	6923	ZINC000095619749

0.3	-7.5	-7.1	0.25	2.365	0.620	5.337	2.458	6	6924	ZINC000095618363
0.3	-7.7	-7.1	0.33	2.049	0.095	4.419	1.297	6	6925	ZINC000257496001
0.3	-7.3	-6.7	0.30	1.953	0.521	4.302	0.355	6	6926	ZINC000000393725
0.3	-8.8	-8.6	0.11	1.693	0.560	2.917	0.494	6	6927	ZINC000014502866
0.3	-10.7	-9.8	0.69	2.302	0.786	5.419	1.438	5	6928	ZINC000002019693
0.3	-6.0	-5.8	0.12	1.705	0.243	4.316	1.522	6	6929	ZINC000032839150
0.3	-8.8	-8.5	0.23	1.652	0.509	2.719	0.541	6	6930	ZINC000257619664
0.3	-8.5	-8.3	0.16	2.014	0.097	4.403	2.008	6	6931	ZINC000257527733
0.3	-4.7	-4.6	0.05	2.176	1.093	2.620	1.187	6	6932	ZINC000001850677
0.3	-7.8	-7.2	0.28	2.316	0.761	4.127	1.255	6	6933	ZINC000014590661
0.3	-8.0	-7.6	0.27	2.055	0.628	4.784	0.820	6	6934	ZINC000013484901
0.3	-8.3	-7.9	0.24	2.126	0.910	4.386	1.825	6	6935	ZINC000000899558
0.3	-9.5	-8.9	0.57	1.518	0.181	5.456	1.410	6	6936	ZINC000257438144
0.3	-6.4	-6.2	0.19	2.638	0.591	3.442	0.846	6	6937	ZINC000001575507
0.3	-6.7	-6.5	0.09	2.065	0.472	3.856	0.749	6	6938	ZINC000000158540

0.3	-6.4	-6.2	0.09	2.392	0.533	3.658	0.706	6	6939	ZINC000033611419
0.3	-7.7	-7.5	0.11	2.573	0.504	4.877	1.968	6	6940	ZINC000095617752
0.3	-8.8	-8.3	0.35	1.714	0.174	6.044	0.867	6	6941	ZINC000257497784
0.3	-7.0	-6.8	0.17	1.746	0.707	3.315	1.540	6	6942	ZINC000003802188
0.3	-8.5	-8.0	0.34	2.743	0.557	4.771	0.436	6	6943	ZINC000014590819
0.3	-7.8	-7.5	0.22	2.310	0.702	3.200	0.838	6	6944	ZINC000008584773
0.3	-7.6	-7.4	0.24	1.947	0.738	4.853	2.036	6	6945	ZINC000013308932
0.3	-6.3	-6.0	0.21	2.577	0.678	3.861	0.521	6	6946	ZINC000000895930
0.3	-8.8	-8.2	0.44	1.638	0.080	5.654	1.574	6	6947	ZINC000100828808
0.3	-6.3	-6.2	0.06	1.931	0.210	4.604	1.220	6	6948	ZINC000001650371
0.3	-7.1	-6.7	0.24	2.353	0.494	3.381	1.111	6	6949	ZINC000028631256
0.3	-4.1	-4.0	0.08	3.015	0.836	3.951	0.843	6	6950	ZINC000031361505
0.3	-5.4	-5.1	0.14	5.955	0.537	7.370	0.576	6	6951	ZINC000000389529
0.3	-8.0	-7.6	0.19	2.179	0.200	5.720	0.235	6	6952	ZINC000003805921
0.3	-6.5	-6.3	0.14	3.051	0.904	4.357	0.949	6	6953	ZINC000001758085

0.3	-6.3	-6.0	0.19	2.510	0.776	4.015	1.614	6	6954	ZINC000095620431
0.3	-6.3	-6.2	0.09	1.924	0.505	4.334	0.988	6	6955	ZINC000095618124
0.3	-4.4	-4.3	0.09	1.933	0.398	2.908	0.655	6	6956	ZINC000032211383
0.3	-6.5	-6.2	0.16	2.189	0.573	3.036	0.270	6	6957	ZINC000012153500
0.3	-5.2	-4.7	0.28	2.736	0.542	3.484	0.565	6	6958	ZINC000015272645
0.3	-7.4	-7.0	0.30	1.460	0.724	2.266	0.497	6	6959	ZINC000001719255
0.3	-8.8	-8.5	0.19	2.219	0.203	5.264	0.490	6	6960	ZINC000248317783
0.3	-7.0	-6.9	0.06	2.494	0.109	5.476	0.411	6	6961	ZINC000002575118
0.3	-9.6	-8.8	0.61	1.485	0.216	3.551	1.681	6	6962	ZINC000014443525
0.3	-8.2	-7.5	0.59	3.016	1.202	4.961	2.170	6	6963	ZINC000003873977
0.3	-4.3	-4.1	0.10	2.271	1.004	2.794	0.820	6	6964	ZINC000003880802
0.3	-7.9	-7.1	0.42	2.419	0.883	4.002	1.873	6	6965	ZINC000005141416
0.3	-9.1	-8.5	0.50	1.963	0.428	4.071	1.314	6	6966	ZINC000014450734
0.3	-8.7	-8.2	0.30	2.110	0.254	4.557	0.419	6	6967	ZINC000253529649
0.3	-6.1	-6.0	0.05	0.707	0.519	3.908	0.538	6	6968	ZINC000001532127

0.3	-6.0	-5.8	0.13	1.508	0.620	3.574	0.940	6	6969	ZINC000025669369
0.3	-8.4	-7.9	0.33	1.851	0.436	3.828	1.627	6	6970	ZINC000014659768
0.3	-9.2	-8.6	0.38	1.840	0.496	3.567	0.836	6	6971	ZINC000000898328
0.3	-6.6	-6.4	0.14	2.563	0.598	4.164	1.243	6	6972	ZINC000050026812
0.3	-10.4	-10.2	0.26	1.302	0.532	4.826	1.715	5	6973	ZINC000257389748
0.3	-5.1	-5.0	0.11	4.267	1.780	5.221	2.096	6	6974	ZINC000013351046
0.3	-7.0	-6.9	0.19	1.467	0.363	3.017	1.303	6	6975	ZINC000095620626
0.3	-10.4	-9.9	0.58	1.906	0.878	4.320	1.559	6	6976	ZINC000257389750
0.3	-8.0	-7.6	0.21	2.239	0.149	4.158	1.144	6	6977	ZINC000005455583
0.3	-6.4	-6.1	0.14	2.635	0.616	5.079	0.443	6	6978	ZINC000017147668
0.3	-5.8	-5.6	0.16	2.409	1.279	4.632	0.734	6	6979	ZINC000100349755
0.3	-9.3	-9.0	0.15	1.747	0.194	5.036	0.690	6	6980	ZINC000014437690
0.3	-7.4	-7.0	0.19	2.767	0.235	4.756	0.294	6	6981	ZINC000001572942
0.3	-7.6	-7.2	0.25	2.669	0.291	4.455	1.476	6	6982	ZINC000001562565
0.3	-6.4	-6.3	0.07	1.959	0.823	3.050	0.646	6	6983	ZINC000001576886

0.3	-8.1	-7.8	0.15	2.353	0.577	3.848	0.771	6	6984	ZINC000014490879
0.3	-8.2	-7.9	0.25	1.788	0.188	4.467	1.224	6	6985	ZINC000014414825
0.3	-9.6	-8.9	0.40	1.338	0.256	4.005	1.239	6	6986	ZINC000100779637
0.3	-4.9	-4.7	0.13	3.547	0.835	4.250	0.188	6	6987	ZINC000032166509
0.3	-5.0	-4.9	0.07	1.492	0.183	2.665	0.184	6	6988	ZINC000095620440
0.3	-5.1	-4.9	0.17	2.178	0.761	3.128	0.833	6	6989	ZINC000001680660
0.3	-4.7	-4.4	0.14	1.565	0.380	3.592	0.403	6	6990	ZINC000001683490
0.3	-7.1	-6.9	0.12	2.217	0.452	4.091	1.376	6	6991	ZINC000004096188
0.3	-6.8	-6.8	0.04	1.890	0.654	2.767	0.813	6	6992	ZINC000003612845
0.3	-7.1	-6.7	0.24	2.142	0.221	3.382	0.687	6	6993	ZINC000050027599
0.3	-8.1	-7.7	0.22	2.230	0.390	4.214	1.190	6	6994	ZINC000005618679
0.3	-6.8	-6.7	0.08	2.416	0.577	3.692	0.815	6	6995	ZINC000002038848
0.3	-9.2	-8.3	0.55	2.030	0.603	4.924	0.960	6	6996	ZINC000257470193
0.3	-8.1	-7.8	0.21	1.899	0.309	3.071	0.770	6	6997	ZINC000100823539
0.3	-5.8	-5.4	0.18	1.779	0.548	2.808	0.660	6	6998	ZINC000002035981

0.3	-6.0	-5.8	0.14	1.835	0.598	3.084	0.875	6	6999	ZINC000004995652
0.3	-8.3	-7.8	0.29	2.171	0.657	4.520	1.515	6	7000	ZINC000001569747
0.3	-8.4	-7.9	0.35	1.716	0.222	5.079	1.710	6	7001	ZINC000257402400
0.3	-8.4	-8.2	0.15	2.076	0.609	3.286	1.329	6	7002	ZINC000100371326
0.3	-8.3	-8.0	0.20	2.323	0.350	4.972	0.796	6	7003	ZINC000002384979
0.3	-8.4	-8.0	0.24	2.068	0.180	5.313	1.126	6	7004	ZINC000257374612
0.3	-6.7	-6.4	0.15	2.123	0.884	3.389	0.708	6	7005	ZINC000002041003
0.3	-7.1	-6.9	0.14	2.088	0.585	4.276	1.159	6	7006	ZINC000003875375
0.3	-8.6	-8.1	0.25	3.496	0.733	5.111	1.240	6	7007	ZINC000000037831
0.3	-7.8	-7.6	0.16	1.802	0.735	3.309	0.860	6	7008	ZINC000104359764
0.3	-6.0	-5.5	0.31	2.098	0.606	2.942	0.988	6	7009	ZINC000005964796
0.3	-8.3	-7.9	0.25	1.906	0.157	3.538	1.117	6	7010	ZINC000014516059
0.3	-10.4	-9.9	0.62	1.688	0.914	4.151	1.370	6	7011	ZINC000257389747
0.3	-7.0	-6.6	0.17	2.509	0.605	4.293	1.885	6	7012	ZINC000095617774
0.3	-6.6	-6.5	0.18	1.777	0.312	3.670	1.455	6	7013	ZINC000014817952

0.3	-6.0	-5.8	0.14	1.836	0.595	3.091	0.862	6	7014	ZINC000004995653
0.3	-7.6	-7.3	0.16	3.391	0.957	4.874	1.341	6	7015	ZINC000002001393
0.3	-7.1	-6.9	0.15	2.178	0.545	3.962	1.272	6	7016	ZINC000003869397
0.3	-8.4	-7.8	0.44	1.869	0.319	4.838	1.654	6	7017	ZINC000014504530
0.3	-5.8	-5.5	0.14	3.171	0.852	4.454	0.393	6	7018	ZINC000002548258
0.3	-6.6	-6.5	0.10	1.624	0.178	4.916	1.154	6	7019	ZINC000004097503
0.3	-8.5	-7.8	0.46	2.013	0.243	4.817	1.005	6	7020	ZINC000015274239
0.3	-8.7	-8.2	0.31	1.740	0.458	3.741	1.241	6	7021	ZINC000015112769
0.3	-5.9	-5.8	0.06	1.910	0.871	3.753	0.536	6	7022	ZINC000003860972
0.3	-8.0	-7.7	0.21	2.122	0.613	3.615	0.707	6	7023	ZINC000015116104
0.3	-5.9	-5.9	0.05	0.855	0.935	2.965	0.754	6	7024	ZINC000000968254
0.3	-6.4	-6.1	0.15	2.258	0.711	3.426	0.528	6	7025	ZINC000039217111
0.3	-6.1	-5.9	0.16	2.153	0.424	3.652	0.799	6	7026	ZINC000001575504
0.3	-6.6	-6.3	0.16	2.362	0.761	4.589	1.129	6	7027	ZINC000000056625
0.3	-6.7	-6.3	0.29	2.717	0.681	3.231	0.896	6	7028	ZINC000006731693

0.3	-8.2	-8.0	0.15	1.540	0.113	4.459	1.346	6	7029	ZINC000027638948
0.3	-6.2	-5.6	0.30	1.977	0.641	3.600	1.058	6	7030	ZINC000001566492
0.3	-9.1	-8.4	0.40	2.084	0.676	4.391	1.509	6	7031	ZINC000000899886
0.3	-3.2	-3.2	0.05	3.971	2.576	4.870	2.152	6	7032	ZINC000004658606
0.3	-6.4	-6.2	0.07	1.758	0.382	3.702	1.024	6	7033	ZINC000256081490
0.3	-6.6	-6.4	0.12	2.521	0.347	3.903	0.320	6	7034	ZINC000014588973
0.3	-8.4	-8.0	0.21	1.874	0.266	4.562	1.600	6	7035	ZINC000257374610
0.3	-5.0	-4.7	0.20	3.246	1.269	3.967	1.387	6	7036	ZINC000006424844
0.3	-8.5	-8.2	0.20	2.312	0.226	5.335	1.666	6	7037	ZINC000095620790
0.3	-8.5	-8.1	0.25	1.764	0.340	5.263	1.787	6	7038	ZINC000257475591
0.3	-6.9	-6.7	0.17	2.563	0.264	5.905	0.167	6	7039	ZINC000034161471
0.3	-5.9	-5.7	0.13	4.399	1.545	5.918	1.875	6	7040	ZINC000000895081
0.3	-9.7	-9.5	0.14	1.801	0.406	4.277	0.569	6	7041	ZINC000000402623
0.3	-8.2	-7.7	0.41	2.604	0.189	4.969	1.028	6	7042	ZINC000040439659
0.3	-7.4	-7.1	0.19	2.486	0.149	4.397	1.567	6	7043	ZINC000004545922

0.3	-8.7	-7.8	0.69	1.638	0.468	3.281	0.606	6	7044	ZINC000015248720
0.3	-5.7	-5.5	0.08	5.006	1.703	6.135	1.336	6	7045	ZINC000018153302
0.3	-7.4	-7.1	0.24	2.269	0.506	4.398	0.867	6	7046	ZINC000013372518
0.3	-6.0	-5.7	0.20	2.791	0.858	4.519	0.971	6	7047	ZINC000001532246
0.3	-8.4	-7.7	0.68	2.265	0.260	5.387	0.795	6	7048	ZINC000006020493
0.3	-9.0	-7.7	0.80	2.514	0.283	5.157	0.889	6	7049	ZINC000100830058
0.3	-6.7	-6.5	0.15	2.492	0.585	4.323	0.779	6	7050	ZINC000004974455
0.3	-6.1	-5.9	0.17	2.100	1.105	3.091	0.911	6	7051	ZINC000006579654
0.3	-9.1	-8.2	0.62	1.981	0.143	4.701	1.253	6	7052	ZINC000256455422
0.3	-7.3	-7.0	0.27	2.382	0.522	3.589	0.925	6	7053	ZINC000034161472
0.3	-6.4	-6.2	0.12	2.518	0.784	3.376	1.052	6	7054	ZINC000000001712
0.3	-8.6	-8.2	0.24	2.031	0.107	5.013	0.966	6	7055	ZINC000100772803
0.3	-5.3	-5.1	0.12	4.458	0.734	6.334	0.536	6	7056	ZINC000014584227
0.3	-6.2	-5.9	0.16	3.113	1.019	4.202	1.422	6	7057	ZINC000032839136
0.3	-6.5	-6.2	0.20	1.731	0.678	2.876	0.641	6	7058	ZINC000257692314

0.3	-6.9	-6.6	0.14	2.251	0.807	4.044	1.611	6	7059	ZINC000040472563
0.3	-9.5	-9.2	0.22	1.417	0.478	4.688	0.635	6	7060	ZINC000015273414
0.3	-9.3	-8.0	0.98	1.610	0.055	2.659	0.361	3	7061	ZINC000100778664
0.3	-7.1	-7.0	0.17	1.186	0.518	1.785	0.332	6	7062	ZINC000001530378
0.3	-6.4	-6.2	0.12	2.687	0.494	4.649	0.773	6	7063	ZINC000005820498
0.3	-7.1	-6.9	0.12	1.695	0.448	3.989	1.156	6	7064	ZINC000033953643
0.3	-5.8	-5.7	0.11	2.792	1.257	3.993	1.143	6	7065	ZINC000003634661
0.3	-6.7	-6.5	0.08	2.414	0.274	3.762	1.102	6	7066	ZINC000050027134
0.3	-3.6	-3.3	0.17	2.007	0.730	2.513	0.725	6	7067	ZINC000001757816
0.3	-5.6	-5.4	0.10	2.301	0.835	3.411	1.082	6	7068	ZINC000032149496
0.3	-6.7	-6.5	0.13	2.958	0.329	4.337	1.112	6	7069	ZINC000000170331
0.3	-6.2	-6.1	0.11	2.586	0.377	4.277	1.132	6	7070	ZINC000002522671
0.3	-6.0	-5.8	0.15	1.846	0.537	4.015	0.717	6	7071	ZINC000100028858
0.3	-8.5	-8.0	0.23	2.013	0.388	4.997	0.751	6	7072	ZINC000000403155
0.3	-6.4	-6.3	0.07	1.795	0.557	3.248	0.333	6	7073	ZINC000005688568

0.3	-7.3	-6.9	0.30	1.796	0.742	3.553	1.677	6	7074	ZINC000004095593
0.3	-7.8	-7.2	0.30	2.207	0.451	3.489	0.378	6	7075	ZINC000014652292
0.3	-7.5	-7.3	0.11	3.340	0.867	4.826	1.670	6	7076	ZINC000015203319
0.3	-9.2	-8.2	0.72	1.730	0.346	5.225	0.705	6	7077	ZINC000100780327
0.3	-7.6	-7.4	0.12	2.151	0.773	3.959	1.966	6	7078	ZINC000013372510
0.3	-8.7	-8.1	0.45	2.064	0.356	4.250	0.797	6	7079	ZINC000014504479
0.3	-5.8	-5.5	0.20	1.730	0.529	2.984	0.898	6	7080	ZINC000012495559
0.3	-8.5	-8.3	0.21	2.314	0.534	4.508	1.296	6	7081	ZINC000013481532
0.3	-7.3	-6.7	0.31	1.897	0.139	4.356	0.316	6	7082	ZINC000000410079
0.3	-6.8	-6.5	0.16	1.838	0.800	4.430	0.649	6	7083	ZINC000014957330
0.3	-5.5	-5.2	0.16	2.180	0.698	2.787	0.726	6	7084	ZINC000013462034
0.3	-8.9	-8.3	0.36	1.504	0.394	3.360	0.528	6	7085	ZINC000004303674
0.3	-7.9	-7.7	0.20	2.350	0.538	3.789	1.494	6	7086	ZINC000139369680
0.3	-5.4	-5.1	0.18	3.021	1.353	4.309	1.502	6	7087	ZINC000013351060
0.3	-8.6	-8.0	0.37	2.543	0.313	5.220	0.229	6	7088	ZINC000002560972

0.3	-7.0	-6.7	0.18	2.527	0.654	3.986	0.815	6	7089	ZINC000001597265
0.3	-8.5	-8.3	0.15	1.913	0.223	3.951	2.196	6	7090	ZINC000013524195
0.3	-8.1	-7.5	0.42	2.444	0.842	5.103	2.265	6	7091	ZINC000013744633
0.3	-6.1	-5.8	0.17	1.691	0.291	3.697	0.768	6	7092	ZINC000005924776
0.3	-8.1	-7.7	0.29	2.149	0.504	4.987	1.563	6	7093	ZINC000014859988
0.3	-6.1	-5.9	0.14	2.153	0.634	3.413	0.823	6	7094	ZINC000000152802
0.3	-7.2	-7.0	0.15	1.679	0.205	2.371	0.248	6	7095	ZINC000000119975
0.3	-6.6	-6.4	0.12	1.934	0.250	3.511	0.783	6	7096	ZINC000004082037
0.3	-6.1	-6.0	0.08	2.043	0.472	4.145	0.634	6	7097	ZINC000033838509
0.3	-7.7	-7.4	0.17	1.810	0.199	4.136	0.786	6	7098	ZINC000257803965
0.3	-10.5	-9.9	0.45	1.578	0.211	2.567	0.820	3	7099	ZINC000015148355
0.3	-4.6	-4.6	0.07	2.481	0.895	3.163	0.681	6	7100	ZINC000000538242
0.3	-7.1	-6.8	0.16	2.795	1.003	3.887	0.942	6	7101	ZINC000000402937
0.3	-7.3	-7.2	0.07	2.150	0.519	4.383	0.843	6	7102	ZINC000034204221
0.3	-6.3	-6.0	0.18	2.129	0.817	3.468	0.995	6	7103	ZINC000006382828

0.3	-8.4	-8.1	0.22	2.272	0.335	3.108	0.419	6	7104	ZINC000006117641
0.3	-6.4	-6.1	0.16	1.934	0.166	3.173	0.745	6	7105	ZINC000005356586
0.3	-9.6	-9.0	0.38	1.230	0.147	4.146	1.348	6	7106	ZINC000100779633
0.3	-6.1	-5.9	0.12	2.211	0.663	2.846	0.771	6	7107	ZINC000000896404
0.3	-6.5	-6.2	0.12	1.721	0.204	2.991	1.596	6	7108	ZINC000015120793
0.3	-4.9	-4.7	0.14	1.682	0.294	3.048	0.540	6	7109	ZINC000001602516
0.3	-9.4	-8.9	0.35	1.675	0.159	3.723	1.718	6	7110	ZINC000014919084
0.3	-5.6	-5.3	0.20	1.732	0.456	3.492	0.296	6	7111	ZINC000005839270
0.3	-6.8	-6.5	0.13	1.919	0.203	4.030	0.888	6	7112	ZINC000013432664
0.3	-9.7	-8.7	1.00	1.755	0.000	3.923	0.000	2	7113	ZINC000100822252
0.3	-6.0	-5.8	0.12	2.208	0.393	3.162	0.723	6	7114	ZINC000000391824
0.3	-8.5	-8.3	0.12	1.741	0.340	4.068	2.342	6	7115	ZINC000013524198
0.3	-6.1	-5.9	0.12	1.965	0.559	3.402	0.956	6	7116	ZINC000013433448
0.3	-8.4	-8.2	0.21	2.245	0.881	3.476	1.570	6	7117	ZINC000001638005
0.3	-8.7	-8.0	0.65	1.911	0.235	5.533	1.962	4	7118	ZINC000014820550

0.3	-7.9	-7.7	0.21	2.248	0.338	3.135	1.022	6	7119	ZINC000017993401
0.3	-8.7	-8.3	0.27	1.238	0.395	3.687	2.180	6	7120	ZINC000257579864
0.3	-7.3	-6.8	0.30	2.132	0.379	3.481	1.526	6	7121	ZINC000014439795
0.3	-6.3	-6.1	0.09	2.520	0.341	3.643	0.842	6	7122	ZINC000040439590
0.3	-8.6	-7.8	0.54	1.794	0.594	4.033	2.081	6	7123	ZINC000014505065
0.3	-7.1	-7.0	0.14	1.553	0.746	4.826	1.314	6	7124	ZINC000000073032
0.3	-7.5	-7.4	0.07	1.674	0.398	2.651	0.760	6	7125	ZINC000000057740
0.3	-8.2	-7.8	0.29	1.802	0.199	5.809	0.975	6	7126	ZINC000013382413
0.3	-5.4	-5.3	0.09	3.221	1.521	3.869	1.852	6	7127	ZINC000014680224
0.3	-6.2	-6.0	0.14	2.562	0.566	3.173	0.621	6	7128	ZINC000003875629
0.3	-8.6	-8.3	0.23	2.562	0.362	4.819	1.369	6	7129	ZINC000012501484
0.3	-9.1	-8.6	0.25	2.684	0.549	5.600	1.864	6	7130	ZINC000016026413
0.3	-9.1	-8.5	0.52	1.758	0.302	5.258	1.076	6	7131	ZINC000100782058
0.3	-7.0	-6.7	0.24	1.596	0.329	3.294	0.877	6	7132	ZINC000005360100
0.3	-5.8	-5.7	0.08	2.010	0.126	4.122	0.473	6	7133	ZINC000001708196

0.3	-9.5	-8.5	0.57	1.653	0.576	4.840	1.519	6	7134	ZINC000257388891
0.3	-6.6	-6.3	0.16	1.920	0.767	2.935	0.402	6	7135	ZINC000100826992
0.3	-5.6	-5.3	0.19	1.861	0.501	3.140	0.805	6	7136	ZINC000001718840
0.3	-9.0	-8.3	0.38	1.717	0.687	5.189	1.016	6	7137	ZINC000001531881
0.3	-7.2	-6.9	0.22	2.314	0.052	5.143	1.916	6	7138	ZINC000095617702
0.3	-7.9	-7.8	0.09	1.877	1.001	5.124	1.675	6	7139	ZINC000028128252
0.3	-9.6	-8.8	0.67	1.631	0.164	3.598	1.654	6	7140	ZINC000014443529
0.3	-7.5	-7.3	0.17	3.015	0.598	4.088	0.884	6	7141	ZINC000000407973
0.3	-9.3	-7.8	0.95	2.423	1.268	4.315	1.989	4	7142	ZINC000005920311
0.3	-7.8	-7.3	0.33	2.602	0.316	4.499	1.099	6	7143	ZINC000095617725
0.3	-4.8	-4.6	0.12	2.837	0.390	3.410	0.446	6	7144	ZINC000000967189
0.3	-5.7	-5.4	0.14	2.118	0.661	3.461	0.493	6	7145	ZINC000001850460
0.3	-6.7	-6.3	0.24	2.686	0.512	4.549	1.217	6	7146	ZINC000001532546
0.3	-8.0	-7.7	0.21	1.554	0.054	3.475	0.863	6	7147	ZINC000257520816
0.3	-5.6	-5.5	0.08	1.658	0.569	4.526	0.466	6	7148	ZINC000002516866

0.3	-9.8	-9.3	0.36	1.958	0.688	3.690	1.391	6	7149	ZINC000100778187
0.3	-7.1	-6.8	0.18	2.192	0.792	3.209	0.454	6	7150	ZINC000000057736
0.3	-8.7	-8.1	0.41	2.117	0.298	4.472	0.524	6	7151	ZINC000253529650
0.3	-6.5	-6.3	0.13	1.986	0.389	3.347	0.686	6	7152	ZINC000001680788
0.3	-7.5	-7.2	0.20	2.097	0.720	4.163	1.296	6	7153	ZINC000033980258
0.3	-9.6	-8.5	0.66	2.065	0.566	4.435	1.589	6	7154	ZINC000257433875
0.3	-7.4	-7.0	0.24	3.000	0.183	6.166	0.554	6	7155	ZINC000033995142
0.3	-8.7	-7.2	0.78	2.385	0.360	5.276	0.930	6	7156	ZINC000031351595
0.3	-8.4	-8.1	0.18	2.439	0.191	4.088	0.670	6	7157	ZINC000000038545
0.3	-6.5	-6.4	0.07	2.850	0.761	3.988	1.234	6	7158	ZINC000014412408
0.3	-5.8	-5.6	0.17	2.030	0.204	3.933	0.582	6	7159	ZINC000002036910
0.3	-8.2	-7.7	0.25	1.647	0.206	5.075	0.846	6	7160	ZINC000040164374
0.3	-10.4	-9.3	0.61	1.734	0.520	4.752	1.538	6	7161	ZINC000261494684
0.3	-6.2	-6.1	0.05	2.364	0.343	3.202	0.511	6	7162	ZINC000040471335
0.3	-6.2	-5.9	0.17	2.776	0.459	4.391	1.202	6	7163	ZINC000002390943

0.3	-9.2	-8.7	0.37	1.814	0.613	4.488	1.519	6	7164	ZINC000257419074
0.3	-5.6	-5.4	0.19	2.932	1.335	3.675	1.364	6	7165	ZINC000001850313
0.3	-7.8	-7.2	0.32	2.176	0.692	3.695	1.315	6	7166	ZINC000014590662
0.3	-7.1	-6.7	0.21	2.492	0.759	3.711	0.930	6	7167	ZINC000000901485
0.3	-4.7	-4.5	0.18	2.107	0.559	3.401	1.084	6	7168	ZINC000006071289
0.3	-6.3	-6.2	0.09	2.244	0.455	3.853	0.685	6	7169	ZINC000003869812
0.3	-6.3	-5.9	0.27	2.502	0.930	3.495	1.237	6	7170	ZINC000015120356
0.3	-5.4	-5.2	0.13	1.895	1.003	2.783	0.563	6	7171	ZINC000001688763
0.3	-10.1	-9.3	0.42	2.013	0.202	4.780	1.747	6	7172	ZINC000014651990
0.3	-8.4	-7.8	0.34	1.457	0.253	4.385	0.962	6	7173	ZINC000100772806
0.3	-5.7	-5.3	0.21	1.899	0.593	3.564	0.714	6	7174	ZINC000001693320
0.3	-7.4	-7.3	0.11	2.507	0.370	4.908	0.702	6	7175	ZINC000100823350
0.3	-7.5	-7.3	0.17	2.075	0.335	3.978	1.085	6	7176	ZINC000006031000
0.3	-8.4	-7.5	0.47	2.361	0.124	5.163	0.192	6	7177	ZINC000001708205
0.3	-8.4	-8.1	0.17	2.576	0.704	4.564	1.798	6	7178	ZINC000039208823

0.3	-9.4	-9.1	0.25	2.415	0.512	4.096	1.353	6	7179	ZINC000015169404
0.3	-8.4	-7.8	0.34	1.839	0.494	3.562	0.858	6	7180	ZINC000043875537
0.3	-4.0	-3.9	0.08	2.182	1.560	3.405	1.143	6	7181	ZINC000001532732
0.3	-8.6	-7.9	0.40	1.935	0.452	5.332	1.405	6	7182	ZINC000014610482
0.3	-7.9	-7.6	0.24	1.738	0.305	3.858	1.166	6	7183	ZINC000014444406
0.3	-6.2	-6.0	0.11	2.219	0.566	3.443	1.438	6	7184	ZINC000001601795
0.3	-5.5	-5.2	0.21	1.927	0.654	2.661	1.168	6	7185	ZINC000033839268
0.3	-6.6	-6.4	0.13	3.313	0.419	4.336	0.837	6	7186	ZINC000001590839
0.3	-5.3	-5.0	0.21	2.700	0.616	3.739	0.531	6	7187	ZINC000005820128
0.3	-6.9	-6.8	0.08	1.822	0.838	3.397	0.512	6	7188	ZINC000014719302
0.3	-6.1	-6.0	0.07	2.634	0.934	4.595	1.669	6	7189	ZINC000032839152
0.3	-6.0	-5.7	0.17	1.905	0.651	3.064	0.828	6	7190	ZINC000103200603
0.3	-7.6	-7.6	0.05	1.828	0.412	4.172	0.675	6	7191	ZINC000015253254
0.3	-7.8	-7.7	0.07	1.970	0.451	4.976	1.678	6	7192	ZINC000014720629
0.3	-5.3	-5.1	0.14	2.297	0.709	3.552	0.680	6	7193	ZINC000015119532

0.3	-8.6	-8.1	0.33	1.898	0.266	6.619	0.409	6	7194	ZINC000257475592
0.3	-6.5	-6.0	0.26	1.576	0.452	3.509	1.169	6	7195	ZINC000038642932
0.3	-6.4	-6.3	0.08	1.714	0.226	4.493	0.119	6	7196	ZINC000086034456
0.3	-7.1	-6.9	0.15	1.830	0.716	4.452	0.962	6	7197	ZINC000000153654
0.3	-9.1	-8.8	0.18	1.639	0.323	3.331	0.642	6	7198	ZINC000256430657
0.3	-6.1	-5.9	0.09	1.700	0.849	3.868	0.777	6	7199	ZINC000001593115
0.3	-6.5	-6.4	0.05	1.990	0.342	3.481	0.875	6	7200	ZINC000001709623
0.3	-5.8	-5.6	0.09	2.759	0.224	3.250	0.260	6	7201	ZINC000000388291
0.3	-5.4	-5.2	0.21	2.010	0.799	3.108	1.124	6	7202	ZINC000005820722
0.3	-9.6	-8.8	0.51	2.054	0.661	4.214	1.590	6	7203	ZINC000003874958
0.3	-6.6	-6.5	0.07	2.671	0.893	4.623	0.956	6	7204	ZINC000050027211
0.3	-6.2	-6.0	0.16	2.175	0.729	3.706	1.324	6	7205	ZINC000013383607
0.3	-6.8	-6.5	0.22	1.966	0.281	4.545	0.652	6	7206	ZINC000095619766
0.3	-6.2	-6.1	0.10	1.967	0.706	2.551	1.068	6	7207	ZINC000002039296
0.3	-3.4	-3.2	0.13	2.394	1.338	3.335	1.264	6	7208	ZINC000001597766

0.3	-7.9	-7.5	0.25	1.634	0.813	3.875	1.008	6	7209	ZINC000001653028
0.3	-10.3	-8.4	1.09	2.116	0.687	4.392	1.886	5	7210	ZINC000014696262
0.3	-8.8	-8.4	0.23	2.494	0.236	4.787	0.654	6	7211	ZINC000015153301
0.3	-8.5	-8.2	0.23	2.037	0.059	4.247	1.942	6	7212	ZINC000257527731
0.3	-6.0	-5.8	0.12	2.401	1.180	4.384	1.069	6	7213	ZINC000032151477
0.3	-10.3	-9.8	0.44	2.090	0.455	4.089	0.999	6	7214	ZINC000100824161
0.3	-6.6	-6.4	0.09	1.949	0.606	3.181	0.468	6	7215	ZINC000008616085
0.3	-9.7	-9.2	0.38	1.277	0.570	3.271	0.702	6	7216	ZINC000100781958
0.3	-5.3	-5.1	0.11	4.527	1.130	6.214	0.992	6	7217	ZINC000014584229
0.3	-6.6	-6.3	0.23	3.108	0.708	4.205	1.083	6	7218	ZINC000001541833
0.3	-5.0	-4.9	0.05	1.861	0.205	2.681	0.697	6	7219	ZINC000000388077
0.3	-7.3	-7.1	0.13	1.614	0.558	3.954	1.740	6	7220	ZINC000004096204
0.3	-6.5	-6.3	0.11	3.755	1.151	5.225	1.815	6	7221	ZINC000013357394
0.3	-8.2	-7.9	0.17	2.303	0.628	3.728	0.904	6	7222	ZINC000000388180
0.3	-8.4	-8.0	0.20	2.014	0.143	5.490	1.321	6	7223	ZINC000014679852

0.3	-8.4	-8.0	0.23	1.654	0.448	5.073	2.081	6	7224	ZINC000015251396
0.3	-8.5	-8.1	0.26	2.192	0.079	4.115	0.268	6	7225	ZINC000015113369
0.3	-7.3	-6.9	0.23	3.330	0.958	4.677	1.096	6	7226	ZINC000000057291
0.3	-8.4	-7.8	0.39	2.655	0.247	5.137	1.043	6	7227	ZINC000031356996
0.3	-4.9	-4.8	0.08	1.990	0.958	2.829	0.833	6	7228	ZINC000001680396
0.3	-8.1	-7.4	0.52	1.855	0.852	3.260	1.285	6	7229	ZINC000003861746
0.3	-5.9	-5.7	0.17	2.417	0.247	4.381	0.474	6	7230	ZINC000001532721
0.3	-5.6	-5.2	0.22	4.084	0.549	4.967	0.659	6	7231	ZINC000034633903
0.3	-5.9	-5.7	0.09	1.155	0.264	4.258	0.875	6	7232	ZINC000002040884
0.3	-5.9	-5.7	0.13	2.295	0.450	3.620	0.521	6	7233	ZINC000001532844
0.3	-5.6	-5.2	0.25	3.013	0.896	3.834	1.218	6	7234	ZINC000013437570
0.3	-6.4	-6.1	0.18	2.349	0.799	3.187	0.663	6	7235	ZINC000000896380
0.3	-4.8	-4.6	0.08	2.774	0.804	3.608	0.988	6	7236	ZINC000000389716
0.3	-8.9	-7.6	0.68	1.549	0.475	3.350	1.769	6	7237	ZINC000100829830
0.3	-7.2	-6.9	0.21	1.988	0.399	4.853	1.199	6	7238	ZINC000001567073

0.3	-8.5	-8.1	0.32	2.895	0.418	4.691	1.267	6	7239	ZINC000018059633
0.3	-5.0	-4.8	0.13	2.305	0.452	3.377	0.684	6	7240	ZINC000001742333
0.3	-9.2	-8.3	0.55	2.031	0.603	4.923	0.959	6	7241	ZINC000100780323
0.3	-8.8	-8.0	0.38	2.557	0.194	4.700	0.682	6	7242	ZINC000002384970
0.3	-6.0	-5.7	0.20	2.246	1.251	4.379	1.070	6	7243	ZINC000003876030
0.3	-7.2	-6.8	0.23	2.204	0.305	3.507	1.184	6	7244	ZINC000002391059
0.3	-8.1	-7.8	0.15	1.914	0.617	5.218	1.402	6	7245	ZINC000015120292
0.3	-8.6	-7.8	0.56	1.846	0.442	5.328	1.763	6	7246	ZINC000015116033
0.3	-8.9	-8.6	0.20	2.734	0.189	4.445	0.882	6	7247	ZINC000003861683
0.3	-7.4	-7.1	0.18	2.621	0.336	4.538	1.133	6	7248	ZINC000033995506
0.3	-5.2	-5.0	0.14	2.514	0.327	3.640	0.442	6	7249	ZINC000002041112
0.3	-5.7	-5.4	0.21	2.772	0.661	3.337	0.830	6	7250	ZINC000015272614
0.3	-9.1	-7.1	0.95	2.187	0.229	5.081	0.739	6	7251	ZINC000105495178
0.3	-6.0	-5.7	0.14	1.807	0.605	3.039	0.906	6	7252	ZINC000004995650
0.3	-8.3	-7.8	0.33	1.748	0.627	3.124	1.555	6	7253	ZINC000095866536

0.3	-5.4	-5.2	0.15	2.017	0.643	2.506	0.490	6	7254	ZINC000095619915
0.3	-5.9	-5.6	0.18	2.309	0.938	3.868	1.315	6	7255	ZINC000002036135
0.3	-8.3	-7.8	0.44	2.628	1.084	4.297	1.972	6	7256	ZINC000004096962
0.3	-6.8	-6.4	0.21	1.594	0.269	4.588	1.581	6	7257	ZINC000015122226
0.3	-6.5	-6.3	0.12	2.479	0.656	4.440	0.894	6	7258	ZINC000077303069
0.3	-7.3	-7.0	0.14	1.792	0.244	3.463	0.592	6	7259	ZINC000005510222
0.3	-6.8	-6.6	0.21	1.905	0.410	3.176	0.647	6	7260	ZINC000005997565
0.3	-5.9	-5.7	0.13	2.451	0.254	3.726	0.619	6	7261	ZINC000000156709
0.3	-7.5	-7.2	0.24	3.247	0.445	4.726	0.429	6	7262	ZINC000002384942
0.3	-5.3	-5.1	0.16	2.320	0.139	3.676	0.108	6	7263	ZINC000001532715
0.3	-6.8	-6.6	0.17	1.957	0.455	2.971	0.871	6	7264	ZINC000002568154
0.3	-8.4	-8.1	0.18	2.697	0.913	3.846	0.532	6	7265	ZINC000000391809
0.3	-8.9	-8.8	0.07	2.331	0.129	4.334	0.769	6	7266	ZINC000001692348
0.3	-6.2	-6.0	0.22	1.848	0.385	2.538	0.649	6	7267	ZINC000002039297
0.3	-5.3	-5.0	0.21	2.289	0.517	3.417	0.421	6	7268	ZINC000002041113

0.3	-7.6	-7.2	0.21	2.859	0.354	4.637	0.648	6	7269	ZINC000005923356
0.3	-4.0	-3.7	0.24	1.006	0.788	2.089	0.179	6	7270	ZINC000001680021
0.3	-6.0	-5.8	0.15	2.071	0.534	2.531	0.699	6	7271	ZINC000001529433
0.3	-5.3	-5.1	0.12	3.062	0.335	3.762	0.522	6	7272	ZINC000002569298
0.3	-7.5	-7.4	0.08	2.338	0.214	4.703	0.990	6	7273	ZINC000012496118
0.3	-5.9	-5.7	0.12	1.947	0.181	3.396	0.292	6	7274	ZINC000000895179
0.3	-6.6	-6.4	0.15	2.210	0.315	3.148	0.786	6	7275	ZINC000002560979
0.3	-8.4	-8.1	0.29	1.600	0.104	3.526	0.532	6	7276	ZINC000257403667
0.3	-7.3	-6.9	0.19	2.898	0.410	3.857	0.805	6	7277	ZINC000013549730
0.3	-7.7	-7.1	0.42	2.844	0.407	5.605	1.658	6	7278	ZINC000011677069
0.3	-6.7	-6.5	0.18	1.578	0.455	2.144	0.338	6	7279	ZINC000000001927
0.3	-5.9	-5.8	0.07	2.310	0.625	4.736	0.454	6	7280	ZINC000095618186
0.3	-7.4	-7.1	0.27	2.138	0.693	2.969	0.954	6	7281	ZINC000000897457
0.3	-9.2	-8.5	0.56	1.949	0.489	3.680	1.380	6	7282	ZINC000014590329
0.3	-7.0	-6.8	0.21	2.106	0.257	3.931	1.156	6	7283	ZINC000003870285

0.3	-6.6	-6.5	0.08	2.108	0.521	3.378	0.514	6	7284	ZINC000001850617
0.3	-5.8	-5.6	0.11	1.704	0.972	2.682	0.616	6	7285	ZINC000012358697
0.3	-7.5	-7.3	0.13	2.965	0.247	4.403	1.264	6	7286	ZINC000095617759
0.3	-5.9	-5.7	0.12	2.326	1.195	3.518	0.902	6	7287	ZINC000012495569
0.3	-8.4	-7.8	0.30	2.109	0.188	3.359	0.963	6	7288	ZINC000014682901
0.3	-7.1	-6.7	0.28	2.579	0.871	3.282	0.853	6	7289	ZINC000000158743
0.3	-5.8	-5.7	0.12	2.311	1.212	4.609	0.717	6	7290	ZINC000100349759
0.3	-6.0	-5.8	0.15	2.732	1.330	4.674	0.996	6	7291	ZINC000002004604
0.3	-8.1	-7.7	0.26	2.631	0.316	4.890	1.831	6	7292	ZINC000050027888
0.3	-4.9	-4.6	0.16	2.465	0.629	3.361	0.755	6	7293	ZINC000002003567
0.3	-5.7	-5.4	0.13	2.599	0.643	4.209	1.346	6	7294	ZINC000017877781
0.3	-6.9	-6.6	0.21	2.194	0.285	4.106	0.480	6	7295	ZINC000013373389
0.3	-6.0	-5.8	0.12	1.452	0.482	3.539	0.968	6	7296	ZINC000100028860
0.3	-7.0	-6.8	0.09	3.010	1.002	5.118	0.996	6	7297	ZINC000005273654
0.3	-9.5	-8.5	0.45	2.005	0.373	5.466	2.054	6	7298	ZINC000257527917

0.3	-8.6	-8.0	0.38	1.903	0.226	3.960	1.305	6	7299	ZINC000261494638
0.3	-5.4	-5.2	0.09	2.408	0.950	3.122	1.124	6	7300	ZINC000000410301
0.3	-7.9	-7.7	0.17	2.041	0.358	4.644	0.613	6	7301	ZINC000062233810
0.3	-7.7	-7.3	0.18	1.723	0.054	4.001	1.479	6	7302	ZINC000033358936
0.3	-9.1	-8.6	0.40	1.607	0.296	4.339	1.716	6	7303	ZINC000257561798
0.3	-3.7	-3.5	0.11	4.628	1.694	5.066	1.822	6	7304	ZINC000002038687
0.3	-6.4	-6.2	0.13	1.029	0.483	3.301	0.461	6	7305	ZINC000000406908
0.3	-8.8	-8.3	0.37	2.674	0.491	4.646	1.143	6	7306	ZINC000014445252
0.3	-6.4	-6.2	0.16	2.360	1.159	3.484	1.882	6	7307	ZINC000013433784
0.3	-9.8	-8.6	0.64	1.671	0.343	4.322	2.198	6	7308	ZINC000013860547
0.3	-6.0	-6.0	0.00	2.078	1.091	2.934	0.647	6	7309	ZINC000100002220
0.3	-7.5	-7.3	0.13	1.618	0.685	3.034	0.395	6	7310	ZINC000008582046
0.3	-8.5	-7.8	0.45	1.958	0.180	4.316	1.350	6	7311	ZINC000085837036
0.3	-6.2	-5.9	0.21	3.039	0.403	4.506	0.368	6	7312	ZINC000095620588
0.3	-8.7	-8.4	0.16	1.532	0.633	3.145	1.737	6	7313	ZINC000013481473

0.3	-6.5	-6.3	0.13	2.029	0.700	4.124	1.871	6	7314	ZINC000005820001
0.3	-6.8	-6.4	0.20	1.623	0.249	4.070	1.003	6	7315	ZINC000006067325
0.3	-9.5	-8.6	0.51	2.456	0.178	4.948	1.007	6	7316	ZINC000230078234
0.3	-7.3	-6.9	0.34	1.955	0.226	3.636	0.864	6	7317	ZINC000015062584
0.3	-7.0	-6.8	0.20	2.306	0.199	3.392	1.278	6	7318	ZINC000050027240
0.3	-4.2	-4.1	0.07	2.199	0.255	2.705	0.343	6	7319	ZINC000100013729
0.3	-7.2	-7.0	0.17	2.107	0.676	3.790	2.064	6	7320	ZINC000014855864
0.3	-6.5	-6.2	0.16	2.449	0.932	3.778	0.855	6	7321	ZINC000000039112
0.3	-9.6	-8.9	0.40	1.344	0.262	4.042	1.191	6	7322	ZINC000100779635
0.3	-7.8	-7.2	0.30	1.948	0.676	3.531	1.150	6	7323	ZINC000257487223
0.3	-8.9	-8.5	0.24	2.267	0.680	5.109	1.149	6	7324	ZINC000001617237
0.3	-7.7	-7.6	0.06	1.671	0.249	5.185	1.371	6	7325	ZINC000071758309
0.3	-9.0	-8.7	0.18	1.583	0.238	4.502	1.804	6	7326	ZINC000257480154
0.3	-6.3	-6.1	0.09	2.542	0.694	4.189	0.984	6	7327	ZINC000014588974
0.3	-6.2	-6.0	0.11	2.556	0.665	4.395	0.632	6	7328	ZINC000014418037

0.3	-6.3	-5.9	0.18	2.899	0.392	3.934	0.819	6	7329	ZINC000095620544
0.3	-5.9	-5.7	0.12	1.738	0.468	3.555	0.537	6	7330	ZINC000002041122
0.3	-7.4	-7.2	0.13	2.905	0.616	4.630	1.065	6	7331	ZINC000013514842
0.3	-5.4	-5.2	0.13	3.265	1.472	4.431	1.490	6	7332	ZINC000005543434
0.3	-4.9	-4.8	0.07	2.149	0.445	3.082	0.697	6	7333	ZINC000002169175
0.3	-6.1	-5.9	0.14	2.593	0.922	4.309	1.210	6	7334	ZINC000012495490
0.3	-6.5	-6.1	0.26	1.578	0.428	2.205	0.838	6	7335	ZINC000001693679
0.3	-7.5	-7.3	0.23	2.157	0.245	5.412	1.538	6	7336	ZINC000015261463
0.3	-7.0	-6.8	0.13	2.057	0.841	3.221	1.492	6	7337	ZINC000040470863
0.3	-9.5	-8.6	0.47	2.220	0.340	4.193	0.776	6	7338	ZINC000031457813
0.3	-7.3	-6.9	0.23	1.715	0.270	4.442	1.162	6	7339	ZINC000012496865
0.3	-8.5	-8.2	0.16	1.548	0.215	4.402	0.355	6	7340	ZINC000085600897
0.3	-8.4	-8.1	0.26	1.270	0.736	4.228	2.157	6	7341	ZINC000015251389
0.3	-8.8	-8.6	0.12	2.119	0.170	5.163	0.413	6	7342	ZINC000410428643
0.3	-5.8	-5.4	0.19	2.272	0.344	3.729	0.907	6	7343	ZINC000001605651

0.3	-9.8	-8.6	0.88	1.709	0.151	4.320	1.701	5	7344	ZINC000100782623
0.3	-7.8	-7.1	0.36	2.113	0.300	4.244	1.367	6	7345	ZINC000033807286
0.3	-8.5	-7.8	0.46	2.050	0.234	4.989	0.951	6	7346	ZINC000257618577
0.3	-8.7	-8.3	0.21	2.390	0.676	4.579	1.165	6	7347	ZINC000014489316
0.3	-5.5	-5.4	0.09	2.694	0.590	3.453	0.811	6	7348	ZINC000002036101
0.3	-8.1	-7.8	0.20	1.865	0.529	3.311	1.259	6	7349	ZINC000104366586
0.3	-6.2	-5.9	0.17	2.426	0.813	3.791	0.385	6	7350	ZINC000005157187
0.3	-9.1	-7.6	1.01	1.772	0.270	4.907	1.910	6	7351	ZINC000014416217
0.3	-6.8	-6.5	0.19	2.019	0.104	3.513	0.429	6	7352	ZINC000014817948
0.3	-5.1	-4.9	0.22	3.565	2.759	5.145	2.075	6	7353	ZINC000012404985
0.3	-6.9	-6.6	0.15	2.559	0.304	3.531	0.453	6	7354	ZINC000000281855
0.3	-7.0	-6.8	0.14	2.268	0.441	4.982	2.141	6	7355	ZINC000095617791
0.3	-6.4	-6.1	0.20	2.193	0.560	4.181	1.619	6	7356	ZINC000001576199
0.3	-7.5	-7.4	0.04	2.387	0.509	4.190	0.836	6	7357	ZINC000095619236
0.3	-6.5	-6.4	0.11	2.083	0.332	3.702	0.756	6	7358	ZINC000040439735

0.3	-6.5	-6.2	0.17	2.403	0.939	3.571	1.890	6	7359	ZINC000001576326
0.3	-6.8	-6.5	0.23	2.099	0.741	2.929	0.564	6	7360	ZINC000034279054
0.3	-7.9	-7.6	0.17	2.411	0.223	5.912	0.353	6	7361	ZINC000004655403
0.3	-5.0	-4.8	0.18	2.686	0.745	3.412	0.724	6	7362	ZINC000001841229
0.3	-7.3	-7.0	0.15	2.116	0.180	4.947	2.039	6	7363	ZINC000040165526
0.3	-7.0	-6.8	0.12	1.497	0.339	2.483	0.489	6	7364	ZINC000040470872
0.3	-8.6	-8.1	0.33	2.034	0.518	4.311	1.382	6	7365	ZINC000013311795
0.3	-8.8	-8.6	0.20	1.703	0.550	2.987	0.619	6	7366	ZINC000014502864
0.3	-6.1	-5.6	0.27	2.540	0.738	4.879	0.499	6	7367	ZINC000013437584
0.3	-8.7	-8.1	0.42	2.153	0.251	4.543	0.522	6	7368	ZINC000253529651
0.3	-9.4	-8.6	0.66	1.611	0.077	4.725	0.694	6	7369	ZINC000015149492
0.3	-5.1	-5.0	0.07	3.550	1.908	4.549	1.722	6	7370	ZINC000000391913
0.3	-8.7	-7.8	0.55	1.831	0.591	4.501	2.227	6	7371	ZINC000000899915
0.3	-6.7	-6.4	0.25	2.770	1.348	4.089	1.553	6	7372	ZINC000001850933
0.3	-7.5	-7.3	0.13	1.986	0.742	3.898	1.224	6	7373	ZINC000014771121

0.3	-5.8	-5.7	0.08	1.652	0.406	3.149	0.387	6	7374	ZINC000001586760
0.3	-7.6	-7.2	0.27	3.008	0.638	3.878	0.578	6	7375	ZINC000000083860
0.3	-7.7	-7.1	0.28	1.851	0.367	4.970	1.753	6	7376	ZINC000085592675
0.3	-7.9	-7.7	0.15	2.368	0.202	3.596	0.220	6	7377	ZINC000001747873
0.3	-8.3	-8.1	0.18	1.966	0.383	4.184	2.124	6	7378	ZINC000008855117
0.3	-7.4	-7.0	0.17	2.542	0.420	4.511	0.994	6	7379	ZINC000100770545
0.3	-7.4	-7.2	0.10	1.733	0.363	3.482	1.108	6	7380	ZINC000005158603
0.3	-7.6	-7.2	0.24	1.588	0.113	4.152	1.814	6	7381	ZINC000004095596
0.3	-8.7	-8.3	0.24	1.835	0.489	3.991	1.319	6	7382	ZINC000014504473
0.3	-6.5	-6.1	0.24	2.205	0.374	3.720	1.571	6	7383	ZINC000036092736
0.3	-4.5	-4.1	0.22	3.543	1.090	4.547	0.418	6	7384	ZINC000001850626
0.3	-8.7	-8.2	0.41	2.046	0.313	3.807	0.755	6	7385	ZINC000015055158
0.3	-7.8	-7.3	0.24	2.018	0.242	4.357	1.493	6	7386	ZINC000008860447
0.3	-7.6	-7.5	0.09	1.779	0.411	4.054	0.724	6	7387	ZINC000015253263
0.3	-8.9	-8.2	0.34	2.080	0.873	5.095	1.237	6	7388	ZINC000003979039

0.3	-6.6	-6.1	0.26	2.393	0.869	3.042	0.991	6	7389	ZINC000000047985
0.3	-5.3	-5.2	0.07	2.739	0.804	3.703	0.940	6	7390	ZINC000001609512
0.3	-8.8	-7.9	0.68	1.894	0.419	4.445	1.913	6	7391	ZINC000014505068
0.3	-7.7	-7.4	0.19	1.410	0.334	2.203	0.460	6	7392	ZINC000087496269
0.3	-5.6	-5.5	0.05	1.917	0.879	3.959	0.863	6	7393	ZINC000002019415
0.3	-6.8	-6.7	0.12	1.472	0.741	3.380	1.217	6	7394	ZINC000000021790
0.3	-5.7	-5.5	0.14	2.593	0.977	4.216	1.076	6	7395	ZINC000013383345
0.3	-7.0	-6.8	0.16	1.971	0.582	3.491	1.907	6	7396	ZINC000050027621
0.3	-5.4	-5.3	0.08	2.260	0.772	3.073	0.851	6	7397	ZINC000002384556
0.3	-6.7	-6.3	0.21	2.537	0.741	4.177	1.160	6	7398	ZINC000001700946
0.3	-8.4	-7.3	0.94	2.022	0.576	4.039	1.439	6	7399	ZINC000017141862
0.3	-7.3	-7.1	0.18	2.158	0.359	4.541	1.648	6	7400	ZINC000015261358
0.3	-4.4	-4.2	0.17	3.915	0.771	4.582	0.267	6	7401	ZINC000002575200
0.3	-9.1	-8.5	0.56	1.851	0.240	4.538	1.381	6	7402	ZINC000256455425
0.3	-4.2	-4.0	0.12	2.829	0.982	3.627	0.777	6	7403	ZINC000031361507

0.3	-9.7	-8.4	0.71	2.277	0.292	5.049	1.408	6	7404	ZINC000014645232
0.3	-5.7	-5.5	0.13	2.023	0.218	3.589	0.116	6	7405	ZINC000000399365
0.3	-7.5	-7.3	0.15	1.968	0.296	4.553	0.801	6	7406	ZINC000014616311
0.3	-6.3	-6.0	0.19	2.924	0.798	3.552	0.749	6	7407	ZINC000000002233
0.3	-5.9	-5.8	0.11	2.152	0.458	3.566	0.935	6	7408	ZINC000000409206
0.3	-8.1	-7.6	0.32	2.277	0.707	4.384	1.194	6	7409	ZINC000003645672
0.3	-8.9	-8.4	0.38	2.069	0.273	4.431	1.124	6	7410	ZINC000012495242
0.3	-6.7	-6.5	0.19	2.596	0.842	4.185	1.329	6	7411	ZINC000014639214
0.3	-9.4	-9.1	0.27	2.251	0.434	4.705	1.633	6	7412	ZINC000015169403
0.3	-8.3	-7.8	0.25	2.269	0.668	5.187	1.749	6	7413	ZINC000014660065
0.3	-5.4	-5.2	0.15	1.823	0.903	3.307	0.823	6	7414	ZINC000002556454
0.3	-6.5	-6.2	0.20	2.417	0.885	3.075	0.799	6	7415	ZINC000100774999
0.3	-5.0	-4.8	0.13	1.643	0.429	2.571	0.902	6	7416	ZINC000002169176
0.3	-9.2	-8.9	0.23	1.671	0.208	3.969	1.658	6	7417	ZINC000014815122
0.3	-6.7	-6.3	0.31	2.284	0.187	3.589	0.954	6	7418	ZINC000040472506

0.3	-5.1	-5.0	0.07	1.928	0.375	3.254	0.452	6	7419	ZINC000038886594
0.3	-6.3	-5.8	0.22	1.770	0.349	4.244	0.323	6	7420	ZINC000002036968
0.3	-7.2	-7.0	0.16	1.305	0.689	3.506	0.869	6	7421	ZINC000004096372
0.3	-6.1	-5.9	0.11	2.957	1.353	4.202	1.546	6	7422	ZINC000032135821
0.3	-4.3	-4.1	0.09	2.272	1.471	2.901	1.309	6	7423	ZINC000001574399
0.3	-6.3	-5.9	0.21	1.505	0.350	4.069	0.202	6	7424	ZINC000001680757
0.3	-8.5	-8.1	0.23	2.291	0.560	4.210	1.202	6	7425	ZINC000014594785
0.3	-6.6	-6.4	0.15	2.241	0.291	4.706	1.410	6	7426	ZINC000034266410
0.3	-8.2	-7.6	0.39	1.982	0.520	4.203	1.573	6	7427	ZINC000257485455
0.3	-7.9	-7.2	0.38	2.403	0.596	4.469	0.707	6	7428	ZINC000014592619
0.3	-8.8	-8.4	0.31	2.845	0.678	5.257	1.724	6	7429	ZINC000013514846
0.3	-9.7	-9.2	0.40	1.275	0.561	3.270	0.703	6	7430	ZINC000100781962
0.3	-8.5	-7.8	0.48	2.331	0.766	3.610	1.146	6	7431	ZINC000090697507
0.3	-7.6	-7.4	0.12	1.895	0.258	4.180	1.434	6	7432	ZINC000034961765
0.3	-6.8	-6.5	0.19	2.027	0.712	3.992	1.762	6	7433	ZINC000034279056

0.3	-6.2	-5.9	0.13	2.922	0.535	3.447	0.463	6	7434	ZINC000000401997
0.3	-6.9	-6.7	0.16	2.361	0.508	3.981	0.997	6	7435	ZINC000004528568
0.3	-5.5	-5.2	0.22	2.537	0.866	2.984	0.956	6	7436	ZINC000001765586
0.3	-8.5	-7.9	0.32	1.871	0.261	4.231	1.465	6	7437	ZINC000015274238
0.3	-7.5	-7.2	0.19	2.126	0.096	5.701	1.372	6	7438	ZINC000012496551
0.3	-6.7	-6.4	0.16	2.404	0.955	4.435	1.393	6	7439	ZINC000257525584
0.3	-9.1	-8.6	0.24	2.554	0.425	5.108	1.533	6	7440	ZINC000040439744
0.3	-6.9	-6.9	0.05	1.437	0.205	5.591	0.389	6	7441	ZINC000003860906
0.3	-8.5	-8.2	0.18	2.157	0.262	3.941	0.169	6	7442	ZINC000001638001
0.3	-5.7	-5.6	0.08	1.823	0.270	3.404	0.955	6	7443	ZINC000100776433
0.3	-6.9	-6.6	0.18	2.530	0.613	4.539	0.740	6	7444	ZINC000085589701
0.3	-7.9	-7.2	0.57	2.086	0.309	5.064	0.730	6	7445	ZINC000257542064
0.3	-8.5	-8.2	0.17	2.952	0.676	5.407	0.596	6	7446	ZINC000000000352
0.3	-6.6	-6.3	0.15	2.453	0.500	4.538	1.259	6	7447	ZINC000095617787
0.3	-7.9	-7.6	0.29	1.898	0.611	2.780	1.267	6	7448	ZINC000000057505

0.3	-9.3	-7.9	0.81	1.690	0.323	4.329	1.794	6	7449	ZINC000013310303
0.3	-6.4	-6.3	0.08	3.235	0.548	4.609	1.045	6	7450	ZINC000040471255
0.3	-6.0	-5.8	0.12	2.439	0.194	4.260	0.903	6	7451	ZINC000002043543
0.3	-7.8	-7.6	0.17	1.859	0.688	3.366	0.866	6	7452	ZINC000257532559
0.3	-6.8	-6.5	0.15	2.766	0.855	4.063	0.971	6	7453	ZINC000100770539
0.3	-6.0	-5.7	0.19	2.206	0.245	4.441	0.345	6	7454	ZINC000002384555
0.3	-7.0	-6.8	0.09	1.775	0.281	5.824	1.652	6	7455	ZINC000014439786
0.3	-8.9	-8.5	0.31	1.755	0.593	2.846	0.743	6	7456	ZINC000004213653
0.3	-5.3	-5.1	0.15	3.876	0.438	4.357	0.510	6	7457	ZINC000033844408
0.3	-7.0	-6.6	0.26	1.923	0.941	4.907	0.760	6	7458	ZINC000014616295
0.3	-8.5	-8.2	0.24	2.044	0.061	3.566	1.513	6	7459	ZINC000013524189
0.3	-6.8	-6.7	0.07	1.829	0.298	2.708	0.359	6	7460	ZINC000000409285
0.3	-8.1	-7.8	0.19	2.401	0.276	4.529	1.350	6	7461	ZINC000005274044
0.3	-5.3	-5.0	0.15	3.956	1.120	4.648	1.323	6	7462	ZINC000014680218
0.3	-6.8	-6.4	0.22	2.599	0.260	4.667	0.495	6	7463	ZINC000069485940

0.3	-8.5	-8.2	0.21	2.030	0.071	3.605	1.589	6	7464	ZINC000257527734
0.3	-6.5	-6.2	0.16	3.003	0.445	5.429	0.199	6	7465	ZINC000001709624
0.3	-8.8	-8.5	0.15	1.184	0.432	4.143	1.988	6	7466	ZINC000100772451
0.3	-6.5	-6.3	0.15	2.506	0.414	4.623	0.425	6	7467	ZINC000005512600
0.3	-8.2	-8.0	0.13	1.946	0.603	4.394	1.389	6	7468	ZINC000015120819
0.3	-6.3	-6.2	0.08	2.381	0.728	4.375	1.369	6	7469	ZINC000095618266
0.3	-6.5	-6.2	0.23	2.087	0.469	2.964	0.766	6	7470	ZINC000257692318
0.3	-9.3	-8.1	0.70	2.009	0.280	5.742	1.729	6	7471	ZINC000014712182
0.3	-8.8	-8.2	0.39	2.355	0.555	3.929	0.910	6	7472	ZINC000006070267
0.3	-4.6	-4.5	0.09	1.522	0.599	2.804	0.418	6	7473	ZINC000001690407
0.3	-6.1	-5.8	0.21	2.157	0.581	2.705	0.564	6	7474	ZINC000100395869
0.3	-7.5	-7.3	0.17	1.634	0.213	2.110	0.279	6	7475	ZINC000008469299
0.3	-6.4	-6.0	0.21	2.687	0.973	3.840	0.705	6	7476	ZINC000082402584
0.3	-6.3	-6.2	0.08	2.267	0.471	3.261	0.670	6	7477	ZINC000000001953
0.3	-7.0	-6.6	0.27	2.745	0.249	4.620	0.361	6	7478	ZINC000050027793

0.3	-8.9	-8.2	0.38	1.732	0.892	4.185	1.291	6	7479	ZINC000000393808
0.3	-8.2	-7.9	0.18	2.048	0.336	4.495	0.815	6	7480	ZINC000014652228
0.3	-7.8	-7.2	0.32	2.434	0.728	4.344	1.389	6	7481	ZINC000014590664
0.3	-6.4	-6.1	0.18	1.574	0.880	3.052	1.140	6	7482	ZINC000000332748
0.3	-8.0	-7.5	0.28	1.893	0.654	3.179	1.491	6	7483	ZINC000136185123
0.3	-9.4	-8.9	0.39	1.664	0.306	5.740	0.432	6	7484	ZINC000257566291
0.3	-8.1	-7.8	0.16	2.459	0.507	3.193	0.696	6	7485	ZINC000003913403
0.3	-8.3	-7.7	0.30	1.740	0.272	3.662	1.460	6	7486	ZINC000014556674
0.3	-6.6	-6.5	0.05	2.158	0.619	2.983	0.702	6	7487	ZINC000000896153
0.3	-10.2	-9.5	0.37	1.491	0.159	4.831	1.240	6	7488	ZINC000100778189
0.3	-9.5	-8.8	0.34	1.920	0.167	5.251	0.516	6	7489	ZINC000013341238
0.3	-9.2	-7.8	0.75	2.141	0.337	4.161	1.004	6	7490	ZINC000014681995
0.3	-6.1	-5.7	0.24	2.589	0.876	4.031	0.802	6	7491	ZINC000014495081
0.3	-9.6	-8.8	0.69	1.533	0.164	4.454	2.166	6	7492	ZINC000257555566
0.3	-5.3	-5.1	0.13	2.269	0.721	2.684	0.707	6	7493	ZINC000013380571

0.3	-7.9	-7.6	0.16	1.782	0.165	2.822	0.690	6	7494	ZINC000257433057
0.3	-5.5	-5.3	0.11	3.596	0.485	5.302	0.488	6	7495	ZINC000038190810
0.3	-5.7	-5.5	0.18	2.463	0.947	3.857	1.351	6	7496	ZINC000005761902
0.3	-4.1	-3.9	0.13	2.408	0.767	3.191	1.014	6	7497	ZINC000003861605
0.3	-6.9	-6.7	0.17	2.677	0.272	4.726	1.137	6	7498	ZINC000002522665
0.3	-6.8	-6.7	0.09	1.931	0.273	3.363	0.904	6	7499	ZINC000002522563
0.3	-6.7	-6.5	0.10	3.520	0.906	5.063	0.931	6	7500	ZINC000050027649
0.3	-5.0	-4.5	0.25	1.696	0.575	2.228	0.724	6	7501	ZINC000001697401
0.3	-8.8	-8.3	0.32	2.076	0.330	3.635	0.953	6	7502	ZINC000095619729
0.3	-7.5	-7.4	0.09	2.523	0.658	3.949	0.598	6	7503	ZINC000011631247
0.3	-8.6	-8.0	0.39	2.111	0.493	5.356	1.120	6	7504	ZINC000014727929
0.3	-6.1	-5.9	0.14	1.919	0.581	3.370	0.301	6	7505	ZINC000014888072
0.3	-8.2	-8.0	0.19	1.658	0.189	2.978	0.279	6	7506	ZINC000015169356
0.3	-5.5	-5.3	0.13	1.779	0.510	2.213	0.671	6	7507	ZINC000006481415
0.3	-5.7	-5.5	0.15	2.021	0.720	3.899	1.608	6	7508	ZINC000005819419

0.3	-8.4	-8.2	0.12	2.628	0.166	3.888	1.243	6	7509	ZINC000013408191
0.3	-6.5	-6.2	0.16	2.361	1.024	3.076	1.200	6	7510	ZINC000000033882
0.3	-6.8	-6.5	0.21	2.047	0.250	3.705	1.154	6	7511	ZINC000008577167
0.3	-8.5	-8.0	0.32	1.811	0.181	4.947	1.183	6	7512	ZINC000257402403
0.3	-5.5	-5.4	0.10	1.654	0.495	3.638	0.974	6	7513	ZINC000001532865
0.3	-7.3	-7.2	0.07	2.066	0.442	3.399	0.915	6	7514	ZINC000015261405
0.3	-6.5	-6.4	0.05	3.012	0.447	4.332	1.006	6	7515	ZINC000013477407
0.3	-7.7	-7.5	0.10	2.626	0.568	4.620	1.293	6	7516	ZINC000008568878
0.3	-8.5	-8.2	0.27	2.550	0.632	4.752	1.087	6	7517	ZINC000100772275
0.3	-8.2	-8.0	0.21	1.592	0.160	3.006	0.259	6	7518	ZINC000000851690
0.3	-5.1	-4.9	0.13	1.968	0.492	2.436	0.579	6	7519	ZINC000014592814
0.3	-5.7	-5.5	0.18	2.144	1.121	3.566	1.520	6	7520	ZINC000005822250
0.3	-4.8	-4.5	0.15	2.391	0.350	2.871	0.437	6	7521	ZINC000000409333
0.3	-6.2	-6.1	0.09	3.072	0.471	5.879	0.461	6	7522	ZINC000060261385
0.3	-8.0	-7.6	0.23	2.189	0.712	4.864	2.286	6	7523	ZINC000033967521

0.3	-6.0	-5.8	0.14	1.635	0.458	3.170	0.881	6	7524	ZINC000253483956
0.3	-6.1	-5.9	0.09	3.019	0.305	4.591	0.404	6	7525	ZINC000001578617
0.3	-6.3	-6.0	0.16	3.215	0.973	4.718	1.728	6	7526	ZINC000027650880
0.3	-5.3	-5.0	0.15	3.188	1.326	4.183	1.474	6	7527	ZINC000003869281
0.3	-8.3	-8.2	0.11	1.742	0.179	5.223	0.943	6	7528	ZINC000004654966
0.3	-9.2	-8.2	0.69	1.729	0.347	5.225	0.704	6	7529	ZINC000257470194
0.3	-6.3	-6.0	0.17	3.163	1.409	4.304	1.423	6	7530	ZINC000014439082
0.3	-6.4	-6.2	0.12	3.188	0.487	3.789	0.497	6	7531	ZINC000001555311
0.3	-5.4	-5.3	0.05	2.650	1.118	3.912	1.520	6	7532	ZINC000095619621
0.3	-9.1	-7.8	0.81	1.708	0.427	2.322	0.644	6	7533	ZINC000257532774
0.3	-8.9	-8.6	0.25	1.668	0.335	3.336	0.722	6	7534	ZINC000257421068
0.3	-5.8	-5.6	0.16	1.881	0.531	2.861	0.533	6	7535	ZINC000100023851
0.3	-8.8	-8.3	0.50	1.314	0.000	2.263	0.000	2	7536	ZINC000014587141
0.3	-6.2	-6.1	0.09	3.465	1.107	4.649	1.723	6	7537	ZINC000034259487
0.3	-8.9	-8.3	0.40	1.982	0.228	6.316	0.505	6	7538	ZINC000014854264

0.3	-10.5	-9.8	0.65	1.764	0.887	4.812	1.523	6	7539	ZINC000100780457
0.3	-6.4	-6.2	0.12	1.970	0.505	3.977	0.288	6	7540	ZINC000000164902
0.3	-5.1	-4.8	0.20	1.919	0.423	2.719	0.812	6	7541	ZINC000000388070
0.3	-4.6	-4.5	0.12	2.039	0.180	2.871	0.597	6	7542	ZINC000000409386
0.3	-7.5	-7.3	0.12	1.935	0.228	4.224	1.147	6	7543	ZINC000100823572
0.3	-8.1	-7.4	0.34	2.275	0.543	4.361	1.108	6	7544	ZINC000001611702
0.3	-4.8	-4.7	0.07	2.819	1.270	3.733	0.874	6	7545	ZINC000032163583
0.3	-9.0	-8.8	0.14	1.631	0.283	4.533	1.843	6	7546	ZINC000257480153
0.3	-9.0	-8.1	0.66	1.753	0.349	6.678	1.585	6	7547	ZINC000000898959
0.3	-10.0	-9.2	0.57	2.195	0.402	4.057	0.781	6	7548	ZINC000257425742
0.3	-6.6	-6.5	0.08	2.436	0.378	6.010	0.263	6	7549	ZINC000040439643
0.3	-6.5	-6.2	0.17	2.088	0.473	3.870	0.607	6	7550	ZINC000014588553
0.3	-6.9	-6.7	0.10	2.826	0.309	4.315	0.786	6	7551	ZINC000050027631
0.3	-8.1	-7.4	0.39	2.043	0.512	4.200	2.014	6	7552	ZINC000014504899
0.3	-5.7	-5.5	0.12	1.703	0.252	2.532	0.260	6	7553	ZINC000001850694

0.3	-5.4	-5.2	0.16	4.057	2.926	4.582	3.029	6	7554	ZINC000000901626
0.3	-8.5	-7.6	0.68	1.784	0.220	4.817	0.361	6	7555	ZINC000100829344
0.3	-5.6	-5.3	0.17	2.944	1.837	4.225	2.124	6	7556	ZINC000095617998
0.3	-6.5	-6.2	0.20	1.928	0.863	2.796	1.472	6	7557	ZINC000001569728
0.3	-7.6	-7.1	0.23	1.636	0.138	3.275	0.951	6	7558	ZINC000001593013
0.3	-6.9	-6.6	0.17	1.943	0.857	3.605	0.891	6	7559	ZINC000001576866
0.3	-9.7	-9.2	0.38	1.275	0.578	3.273	0.706	6	7560	ZINC000257555247
0.3	-9.0	-8.6	0.31	1.482	0.228	4.037	1.354	6	7561	ZINC000257480155
0.3	-6.3	-6.2	0.12	2.122	0.413	2.856	0.561	6	7562	ZINC000002039876
0.3	-6.6	-6.5	0.11	2.379	0.825	3.514	0.993	6	7563	ZINC000005140706
0.3	-6.3	-5.9	0.21	2.386	0.534	3.635	1.235	6	7564	ZINC000004556856
0.3	-5.8	-5.7	0.08	1.447	0.076	2.950	0.725	6	7565	ZINC000100773211
0.3	-10.1	-8.7	0.78	1.596	0.378	5.283	1.195	6	7566	ZINC000005360172
0.3	-6.9	-6.7	0.17	2.491	0.720	4.835	1.855	6	7567	ZINC000050027391
0.3	-6.8	-6.6	0.16	1.972	0.621	4.382	1.124	6	7568	ZINC000013513316

0.3	-7.6	-7.3	0.24	2.282	0.544	3.736	0.957	6	7569	ZINC000014719899
0.3	-9.5	-8.0	0.89	1.455	0.157	5.396	1.846	5	7570	ZINC000085588991
0.3	-7.9	-7.6	0.16	2.287	0.151	3.754	0.377	6	7571	ZINC000095617662
0.3	-8.7	-8.0	0.49	1.876	0.239	4.601	0.484	6	7572	ZINC000014504482
0.3	-6.0	-5.8	0.17	2.057	0.543	4.014	0.280	6	7573	ZINC000032166962
0.3	-7.5	-7.2	0.27	2.766	0.728	4.725	0.917	6	7574	ZINC000015122070
0.3	-5.9	-5.7	0.12	2.676	0.907	3.812	0.504	6	7575	ZINC000012495554
0.3	-7.6	-7.4	0.13	2.241	0.468	4.972	2.023	6	7576	ZINC000040472636
0.3	-8.6	-8.2	0.23	2.675	0.613	4.757	1.646	6	7577	ZINC000095617810
0.3	-9.7	-9.0	0.48	2.042	0.526	3.985	0.938	6	7578	ZINC000015203706
0.3	-8.5	-8.2	0.28	1.812	0.212	4.407	1.839	6	7579	ZINC000100775371
0.3	-5.3	-5.2	0.07	1.933	0.616	2.378	0.753	6	7580	ZINC000001482113
0.3	-6.9	-6.6	0.16	4.737	0.319	6.136	0.463	6	7581	ZINC000017819771
0.3	-8.5	-8.2	0.31	1.836	0.420	3.858	1.326	6	7582	ZINC000257467837
0.3	-6.0	-5.8	0.14	2.371	0.619	3.245	0.559	6	7583	ZINC000000155720

0.3	-8.4	-7.5	0.43	2.004	0.550	5.123	1.967	6	7584	ZINC000015116029
0.3	-8.0	-7.6	0.21	1.612	0.535	3.251	1.434	6	7585	ZINC000013282308
0.3	-4.0	-3.9	0.08	2.673	0.999	3.098	1.161	6	7586	ZINC000001850546
0.3	-5.6	-5.5	0.13	2.333	0.326	3.723	0.453	6	7587	ZINC000003649507
0.3	-9.6	-8.6	0.72	1.470	0.230	4.403	2.192	6	7588	ZINC000257555565
0.3	-7.7	-7.2	0.24	2.063	0.699	3.913	1.019	6	7589	ZINC000015122072
0.3	-3.6	-3.5	0.08	1.273	0.634	2.783	0.390	6	7590	ZINC000001699944
0.3	-9.4	-8.0	0.81	2.639	0.655	4.443	1.090	6	7591	ZINC000238779689
0.3	-8.6	-7.9	0.35	1.841	0.256	4.894	0.370	6	7592	ZINC000257507766
0.3	-4.7	-4.5	0.17	2.647	0.741	3.837	0.330	6	7593	ZINC000032175843
0.3	-6.6	-6.4	0.15	2.241	0.321	4.141	0.852	6	7594	ZINC000001532623
0.3	-7.4	-7.2	0.18	1.574	0.479	4.042	1.229	6	7595	ZINC000253617814
0.3	-8.7	-8.5	0.12	1.793	0.096	5.515	0.850	6	7596	ZINC000015249560
0.3	-8.0	-7.6	0.27	2.473	0.739	3.484	1.289	6	7597	ZINC000002558198
0.3	-9.5	-8.5	0.64	1.732	0.490	5.545	0.576	6	7598	ZINC000014947781

0.3	-5.5	-5.2	0.18	2.070	0.742	2.887	1.224	6	7599	ZINC000000388357
0.3	-6.3	-5.9	0.19	2.659	0.902	4.415	1.151	6	7600	ZINC000013437585
0.3	-8.4	-7.7	0.46	1.585	0.172	3.530	1.228	6	7601	ZINC000015159847
0.3	-4.8	-4.6	0.12	4.491	2.807	5.893	2.068	6	7602	ZINC000256081130
0.3	-8.0	-7.7	0.20	2.023	0.624	4.505	0.926	6	7603	ZINC000012495581
0.3	-5.3	-5.1	0.15	2.389	0.177	2.912	0.164	6	7604	ZINC000000388312
0.3	-6.1	-5.9	0.12	2.267	1.042	3.556	1.401	6	7605	ZINC000001693630
0.3	-8.5	-7.4	0.72	2.520	0.322	4.886	0.475	6	7606	ZINC000100823131
0.3	-6.2	-5.7	0.24	2.639	0.784	3.307	1.038	6	7607	ZINC000005134904
0.3	-5.8	-5.7	0.09	2.665	0.597	3.617	0.702	6	7608	ZINC000001736031
0.3	-7.5	-7.3	0.14	2.229	0.358	3.626	1.451	6	7609	ZINC000095617723
0.3	-4.8	-4.7	0.09	3.097	2.054	4.338	1.853	6	7610	ZINC000013459852
0.3	-8.6	-8.2	0.22	1.917	0.260	5.986	0.791	6	7611	ZINC000014438575
0.3	-5.1	-5.0	0.07	2.297	0.179	3.279	0.385	6	7612	ZINC000029786605
0.3	-5.2	-4.9	0.18	2.915	0.770	4.128	0.504	6	7613	ZINC000001850674

0.3	-7.4	-7.1	0.15	2.031	0.246	3.869	0.445	6	7614	ZINC000001677806
0.3	-6.3	-6.1	0.12	3.191	0.806	5.221	1.685	6	7615	ZINC000050026977
0.3	-6.1	-5.9	0.12	2.675	0.875	4.542	1.853	6	7616	ZINC000001842903
0.3	-5.8	-5.7	0.12	2.330	1.190	4.598	0.645	6	7617	ZINC000100830171
0.3	-7.9	-7.5	0.19	1.953	0.749	4.796	1.120	6	7618	ZINC000085954076
0.3	-8.6	-8.1	0.27	1.967	0.210	5.143	0.448	6	7619	ZINC000085588925
0.3	-8.3	-8.2	0.18	1.510	0.288	3.605	1.660	6	7620	ZINC000015249352
0.3	-7.4	-7.1	0.16	2.627	0.622	3.892	0.967	6	7621	ZINC000013379087
0.3	-7.1	-6.9	0.22	2.356	0.801	3.903	1.788	6	7622	ZINC000034279673
0.3	-7.0	-6.8	0.09	2.000	1.000	4.089	0.256	6	7623	ZINC000002555390
0.3	-7.3	-7.1	0.16	2.741	0.377	4.609	1.113	6	7624	ZINC000014855866
0.3	-6.3	-6.2	0.07	2.283	0.167	4.458	0.547	6	7625	ZINC000040439639
0.3	-8.4	-8.0	0.19	1.828	0.320	4.899	2.052	6	7626	ZINC000257374609
0.3	-6.9	-6.7	0.16	1.809	0.463	3.974	1.887	6	7627	ZINC000196921871
0.3	-6.6	-6.2	0.25	2.341	0.800	4.244	1.064	6	7628	ZINC000207654210

0.3	-8.7	-8.4	0.18	2.017	0.558	3.838	1.596	6	7629	ZINC000014689153
0.3	-5.4	-5.1	0.18	3.175	0.415	4.163	0.435	6	7630	ZINC000014616379
0.3	-6.9	-6.6	0.25	2.065	0.420	3.613	1.778	6	7631	ZINC000002047152
0.3	-6.1	-5.9	0.15	2.254	0.749	3.645	0.883	6	7632	ZINC000014614335
0.3	-8.7	-8.3	0.35	1.822	0.351	3.908	2.045	6	7633	ZINC000000051923
0.3	-6.0	-5.9	0.13	2.489	1.060	3.378	1.132	6	7634	ZINC000001530217
0.3	-5.3	-5.0	0.23	2.007	0.673	2.782	0.866	6	7635	ZINC000002510149
0.3	-7.3	-7.1	0.18	3.073	0.664	3.838	0.722	6	7636	ZINC000000158741
0.3	-7.2	-6.8	0.20	2.140	0.556	2.767	0.841	6	7637	ZINC000004545920
0.3	-5.8	-5.5	0.15	2.689	1.730	3.497	1.781	6	7638	ZINC000002146644
0.3	-5.0	-4.9	0.11	2.035	0.176	3.461	0.592	6	7639	ZINC000029786608
0.3	-8.5	-8.2	0.32	2.034	0.404	4.098	1.042	6	7640	ZINC000105161358
0.3	-7.1	-6.7	0.27	2.748	0.501	4.052	0.941	6	7641	ZINC000002575129
0.3	-8.7	-8.3	0.21	3.390	0.579	6.118	1.000	6	7642	ZINC000001865982
0.3	-8.1	-7.6	0.34	2.208	0.788	4.062	1.041	6	7643	ZINC000004023310

0.3	-8.0	-7.7	0.24	1.656	1.140	2.746	1.650	6	7644	ZINC000001605731
0.3	-6.8	-6.5	0.14	2.349	0.421	2.991	0.773	6	7645	ZINC000095617777
0.3	-9.2	-8.5	0.40	1.939	0.520	4.199	1.566	6	7646	ZINC000253595814
0.3	-4.9	-4.6	0.14	2.788	1.873	3.756	1.825	6	7647	ZINC000003869286
0.3	-6.6	-6.3	0.23	3.025	0.289	4.044	0.871	6	7648	ZINC000013538197
0.3	-6.2	-5.8	0.22	2.431	0.707	3.479	1.431	6	7649	ZINC000013549717
0.3	-8.7	-8.3	0.31	3.274	0.482	5.600	1.116	6	7650	ZINC000001997858
0.3	-6.2	-5.8	0.24	1.948	0.519	3.552	0.793	6	7651	ZINC000008383223
0.3	-9.6	-8.8	0.38	1.438	0.494	3.872	1.724	6	7652	ZINC000013340618
0.3	-7.6	-7.5	0.11	3.042	0.273	4.986	0.434	6	7653	ZINC000001605728
0.3	-6.5	-6.4	0.11	4.383	1.492	5.456	1.552	6	7654	ZINC000096321491
0.3	-8.2	-7.5	0.53	2.038	0.140	5.219	0.404	6	7655	ZINC000012494381
0.3	-5.7	-5.6	0.11	2.043	0.975	4.013	1.062	6	7656	ZINC000013437578
0.3	-9.7	-9.1	0.56	2.136	0.418	4.005	0.874	6	7657	ZINC000230067236
0.3	-7.5	-7.2	0.25	2.574	0.123	4.612	0.562	6	7658	ZINC000014449163

0.3	-5.4	-5.1	0.14	2.276	1.451	3.943	1.252	6	7659	ZINC000004501410
0.3	-5.9	-5.8	0.08	2.110	0.813	3.697	1.127	6	7660	ZINC000001531045
0.3	-5.1	-4.7	0.22	3.474	1.886	4.830	1.660	6	7661	ZINC000000895427
0.3	-5.4	-5.2	0.21	2.002	0.914	4.359	0.619	6	7662	ZINC000000160274
0.3	-6.5	-6.4	0.08	2.276	0.729	5.154	0.292	6	7663	ZINC000001575290
0.3	-6.8	-6.5	0.16	1.745	0.238	3.814	1.275	6	7664	ZINC000013432662
0.3	-6.6	-6.4	0.12	3.195	0.410	4.393	0.705	6	7665	ZINC000000388311
0.3	-5.4	-5.0	0.22	2.821	1.776	4.253	1.783	6	7666	ZINC000000388046
0.3	-7.1	-6.9	0.17	1.916	0.119	5.212	0.311	6	7667	ZINC000001529947
0.3	-8.7	-8.1	0.37	2.065	0.420	4.796	1.098	6	7668	ZINC000015104218
0.3	-8.6	-7.5	0.52	1.806	0.431	5.186	1.968	6	7669	ZINC000014758732
0.3	-6.0	-5.8	0.11	1.970	0.664	2.937	1.185	6	7670	ZINC000001888739
0.3	-9.0	-8.3	0.49	1.999	0.327	3.676	1.423	6	7671	ZINC000100782082
0.3	-8.1	-7.8	0.17	1.992	0.112	4.551	0.547	6	7672	ZINC000013377651
0.3	-5.9	-5.6	0.19	2.605	0.690	4.521	0.250	6	7673	ZINC000002389912

0.3	-5.5	-5.4	0.10	2.167	1.693	3.642	1.592	6	7674	ZINC000013549494
0.3	-5.6	-5.3	0.24	2.201	0.690	2.885	0.913	6	7675	ZINC000013437567
0.3	-6.0	-5.8	0.14	1.829	0.579	3.115	0.858	6	7676	ZINC000014860575
0.3	-7.9	-7.4	0.28	2.416	0.416	4.201	0.872	6	7677	ZINC000003913402
0.3	-7.8	-7.6	0.18	1.971	0.378	3.265	0.935	6	7678	ZINC000006411846
0.3	-7.8	-7.4	0.27	1.819	0.128	5.342	0.546	6	7679	ZINC000012503177
0.3	-7.9	-7.6	0.21	1.753	0.261	3.425	1.164	6	7680	ZINC000257433058
0.3	-4.7	-4.6	0.12	1.320	1.111	2.342	0.748	6	7681	ZINC000000007993
0.3	-6.5	-6.4	0.07	2.690	0.520	4.075	1.049	6	7682	ZINC000050027234
0.3	-6.4	-6.2	0.17	1.858	0.364	2.662	0.479	6	7683	ZINC000034294022
0.3	-5.6	-5.4	0.13	1.731	0.386	2.969	0.700	6	7684	ZINC000001850486
0.3	-5.8	-5.6	0.21	2.544	1.256	3.548	1.372	6	7685	ZINC000001731997
0.3	-6.0	-5.7	0.16	2.416	0.457	4.401	0.673	6	7686	ZINC000006119077
0.3	-7.8	-7.6	0.17	1.946	0.689	3.836	0.733	6	7687	ZINC000015169350
0.3	-9.9	-9.4	0.36	1.386	0.205	4.070	1.858	6	7688	ZINC000013334444

0.3	-7.1	-7.0	0.08	2.485	0.532	4.706	1.449	6	7689	ZINC000077303072
0.3	-6.5	-6.3	0.13	2.556	0.700	3.689	0.586	6	7690	ZINC000005134376
0.3	-8.0	-7.6	0.23	2.380	0.263	4.655	0.569	6	7691	ZINC000034382707
0.3	-6.9	-6.7	0.09	2.750	0.728	4.332	0.832	6	7692	ZINC000001586788
0.3	-5.4	-5.3	0.09	1.725	0.326	2.832	0.412	6	7693	ZINC000022000195
0.3	-8.1	-7.7	0.31	2.018	0.596	4.008	1.011	6	7694	ZINC000005842977
0.3	-9.2	-8.3	0.43	2.618	0.174	5.181	0.399	6	7695	ZINC000050027132
0.3	-6.3	-6.2	0.12	1.953	0.529	3.407	1.191	6	7696	ZINC000069485974
0.3	-7.3	-6.9	0.27	2.075	0.188	3.463	0.299	6	7697	ZINC000014651766
0.3	-5.5	-5.3	0.12	3.248	1.439	4.109	1.546	6	7698	ZINC000002567965
0.3	-6.8	-6.5	0.15	1.785	0.242	4.312	0.992	6	7699	ZINC000014957323
0.3	-8.2	-7.8	0.20	1.640	0.436	4.341	1.421	6	7700	ZINC000257426779
0.3	-5.5	-5.4	0.12	2.390	0.918	3.500	0.902	6	7701	ZINC000095618245
0.3	-8.6	-8.4	0.28	1.350	0.332	2.960	1.745	6	7702	ZINC000013382393
0.3	-5.1	-5.0	0.12	1.451	0.738	2.988	0.452	6	7703	ZINC000001670837

0.3	-9.9	-9.2	0.36	1.247	0.234	4.029	1.247	6	7704	ZINC000100779626
0.3	-4.7	-4.6	0.10	2.073	1.532	3.297	1.089	6	7705	ZINC000005362481
0.3	-5.9	-5.7	0.15	2.536	0.618	3.954	1.066	6	7706	ZINC000002034326
0.3	-6.3	-6.2	0.09	2.216	0.523	4.865	0.852	6	7707	ZINC000095618156
0.3	-5.9	-5.6	0.23	1.771	0.608	2.094	0.630	6	7708	ZINC000001668238
0.3	-6.5	-6.3	0.13	2.278	0.723	3.240	0.257	6	7709	ZINC000257692321
0.3	-7.9	-7.7	0.13	1.656	0.151	3.988	0.830	6	7710	ZINC000085599777
0.3	-9.7	-9.0	0.35	2.104	0.205	4.964	0.420	6	7711	ZINC000014887281
0.3	-8.7	-8.3	0.21	1.503	0.610	3.146	1.736	6	7712	ZINC000015046976
0.3	-8.7	-8.3	0.24	1.949	0.434	4.222	1.344	6	7713	ZINC000015055153
0.3	-8.0	-7.1	0.58	2.001	0.348	4.240	1.542	6	7714	ZINC000013303515
0.3	-6.0	-5.8	0.17	1.899	0.952	2.771	0.497	6	7715	ZINC000005134501
0.3	-6.6	-6.4	0.12	2.118	0.388	3.509	0.841	6	7716	ZINC000004523272
0.3	-6.8	-6.8	0.05	3.125	0.169	4.176	0.377	6	7717	ZINC000000967563
0.3	-6.2	-5.9	0.18	2.259	0.442	3.194	1.014	6	7718	ZINC000003200634

0.3	-8.8	-8.3	0.35	2.344	0.676	5.034	1.621	6	7719	ZINC000165067804
0.3	-6.4	-6.3	0.09	2.470	0.790	3.916	0.960	6	7720	ZINC000000388293
0.3	-4.7	-4.6	0.07	2.194	0.407	2.939	0.530	6	7721	ZINC000005966178
0.3	-9.2	-8.5	0.50	2.152	0.349	5.782	1.166	6	7722	ZINC000014806944
0.3	-8.4	-7.9	0.30	2.403	0.943	5.329	1.303	6	7723	ZINC000014445246
0.3	-7.6	-7.4	0.10	1.984	0.481	3.780	0.742	6	7724	ZINC000095619750
0.3	-6.8	-6.5	0.16	2.648	0.384	4.430	0.500	6	7725	ZINC000060147881
0.3	-5.7	-5.5	0.11	2.476	0.677	3.522	1.042	6	7726	ZINC000001532657
0.3	-6.8	-6.6	0.11	2.784	0.882	4.048	1.309	6	7727	ZINC000002039275
0.3	-7.3	-7.1	0.13	2.012	0.332	3.222	0.604	6	7728	ZINC000014613368
0.3	-7.4	-7.2	0.13	1.859	0.625	4.550	0.420	6	7729	ZINC000014771116
0.3	-6.7	-6.3	0.27	2.557	0.683	3.585	0.642	6	7730	ZINC000085589688
0.3	-6.2	-5.9	0.13	2.750	0.362	4.506	0.412	6	7731	ZINC000001529626
0.3	-9.1	-7.9	1.12	2.642	0.281	5.226	0.869	4	7732	ZINC000014689090
0.3	-7.7	-7.4	0.19	1.817	0.239	3.832	1.215	6	7733	ZINC000015256934

0.3	-6.3	-5.9	0.18	2.794	1.222	4.130	1.706	6	7734	ZINC000095620559
0.3	-9.4	-9.1	0.29	2.354	0.514	4.124	1.291	6	7735	ZINC000257620010
0.3	-5.1	-4.9	0.15	2.085	0.743	3.416	0.947	6	7736	ZINC000013551397
0.3	-7.2	-6.8	0.26	1.908	0.678	2.817	0.416	6	7737	ZINC000015120008
0.3	-4.4	-4.3	0.10	2.735	0.352	3.680	0.591	6	7738	ZINC000033951265
0.3	-10.3	-8.4	0.99	2.442	0.169	5.210	1.089	6	7739	ZINC000014613039
0.3	-7.1	-6.9	0.10	2.203	0.636	3.082	0.874	6	7740	ZINC000002510107
0.3	-8.1	-7.7	0.17	1.884	0.162	4.022	0.533	6	7741	ZINC000015265068
0.3	-8.6	-8.2	0.30	1.572	0.497	3.783	1.207	6	7742	ZINC000014811606
0.3	-6.9	-6.8	0.06	1.925	0.380	5.017	0.274	6	7743	ZINC000004474613
0.3	-6.7	-6.7	0.05	3.340	1.010	5.595	1.205	6	7744	ZINC000002560984
0.3	-6.9	-6.7	0.18	2.829	0.260	4.202	0.270	6	7745	ZINC000014636771
0.3	-6.5	-6.4	0.11	2.400	0.902	3.291	1.151	6	7746	ZINC000014719170
0.3	-8.9	-7.9	0.66	2.094	0.368	5.093	1.346	6	7747	ZINC000015161032
0.3	-5.4	-5.3	0.11	2.050	0.870	3.575	0.791	6	7748	ZINC000001850552

0.3	-6.5	-6.3	0.19	2.605	0.821	4.191	1.539	6	7749	ZINC000050027718
0.3	-5.3	-5.2	0.07	2.414	0.255	3.989	0.147	6	7750	ZINC000090412744
0.3	-9.7	-8.7	0.73	1.454	0.205	3.674	1.725	6	7751	ZINC000014859538
0.3	-6.1	-5.7	0.18	1.820	0.572	3.398	0.921	6	7752	ZINC000001531036
0.3	-8.5	-8.1	0.32	2.297	0.219	4.677	0.493	6	7753	ZINC000100889365
0.3	-7.1	-6.7	0.30	1.415	0.363	4.502	1.519	6	7754	ZINC000002041154
0.3	-6.5	-6.4	0.08	2.547	0.645	4.491	0.968	6	7755	ZINC000005134625
0.3	-8.8	-7.6	0.57	2.835	1.025	4.506	1.724	6	7756	ZINC000005930819
0.3	-7.7	-7.2	0.21	2.865	0.580	4.554	0.729	6	7757	ZINC000001572940
0.3	-8.3	-7.8	0.37	1.999	0.411	4.187	1.110	6	7758	ZINC000014516053
0.3	-7.6	-7.4	0.26	2.295	0.374	4.058	0.799	6	7759	ZINC000000135392
0.3	-9.6	-8.9	0.44	1.315	0.170	3.395	1.456	6	7760	ZINC000086028124
0.3	-5.3	-4.9	0.20	2.139	0.450	3.726	0.251	6	7761	ZINC000012402448
0.3	-6.7	-6.4	0.20	2.722	0.506	5.055	0.510	6	7762	ZINC000014583656
0.3	-8.4	-8.1	0.21	2.649	0.729	4.753	1.842	6	7763	ZINC000095617648

0.3	-7.7	-7.4	0.29	2.343	1.010	3.571	1.549	6	7764	ZINC000000039098
0.3	-6.2	-5.9	0.22	2.263	0.408	3.872	1.430	6	7765	ZINC000001687335
0.3	-8.6	-8.4	0.15	1.389	0.678	2.977	1.783	6	7766	ZINC000261499719
0.3	-8.5	-8.2	0.16	1.416	0.292	3.213	0.466	6	7767	ZINC000015120458
0.3	-6.4	-6.3	0.07	2.654	0.346	3.601	0.499	6	7768	ZINC000012153443
0.3	-9.0	-7.9	0.55	2.026	0.184	4.244	1.076	6	7769	ZINC000015053515
0.3	-9.3	-8.6	0.54	2.187	0.496	5.026	1.412	6	7770	ZINC000000899786
0.3	-6.5	-6.3	0.12	2.231	0.691	3.209	0.210	6	7771	ZINC000100774994
0.3	-5.6	-5.3	0.25	2.655	0.779	3.836	0.512	6	7772	ZINC000001627234
0.3	-6.3	-5.9	0.21	2.426	0.792	3.018	0.872	6	7773	ZINC000000001011
0.3	-6.4	-6.3	0.11	3.295	0.717	4.442	0.779	6	7774	ZINC000004685854
0.3	-9.1	-7.8	0.87	2.078	0.346	5.925	0.549	6	7775	ZINC000014762893
0.3	-9.1	-8.7	0.33	1.741	0.186	4.092	1.305	6	7776	ZINC000014450729
0.3	-7.3	-7.1	0.13	2.447	0.324	4.509	1.403	6	7777	ZINC000002168692
0.3	-5.1	-5.0	0.13	3.274	0.790	4.382	0.449	6	7778	ZINC000000897516

0.3	-3.3	-3.1	0.17	3.552	2.251	4.191	1.970	6	7779	ZINC000000895111
0.3	-6.7	-6.5	0.19	2.114	0.513	3.637	1.243	6	7780	ZINC000015261372
0.3	-7.0	-6.9	0.09	2.976	0.364	4.211	0.320	6	7781	ZINC000006471017
0.3	-7.7	-7.2	0.29	2.411	0.695	3.186	1.154	6	7782	ZINC000015121872
0.3	-5.9	-5.8	0.13	2.787	0.842	3.764	1.147	6	7783	ZINC000013437580
0.3	-6.5	-6.2	0.15	2.212	0.234	3.448	0.556	6	7784	ZINC000014723364
0.3	-5.9	-5.9	0.05	2.089	0.419	3.709	0.672	6	7785	ZINC000002036797
0.3	-8.4	-7.8	0.48	1.713	0.268	3.694	1.052	6	7786	ZINC000257396874
0.3	-9.1	-8.4	0.59	1.930	0.352	4.493	1.246	6	7787	ZINC000014450732
0.3	-9.1	-8.5	0.29	1.836	0.207	3.969	1.103	6	7788	ZINC000257466072
0.3	-7.0	-6.9	0.11	1.875	0.457	3.585	0.899	6	7789	ZINC000008580042
0.3	-8.9	-8.7	0.15	1.696	0.331	2.437	0.399	6	7790	ZINC000006505219
0.3	-6.3	-6.0	0.11	3.451	1.168	4.967	1.217	6	7791	ZINC000001763954
0.3	-6.5	-6.1	0.23	1.697	0.336	3.468	0.875	6	7792	ZINC000013429392
0.3	-7.1	-6.9	0.12	2.204	0.461	4.077	1.390	6	7793	ZINC000004095545

0.3	-7.9	-7.2	0.32	1.953	0.580	2.985	1.550	6	7794	ZINC000001575285
0.3	-8.4	-8.1	0.27	1.046	0.679	4.232	2.151	6	7795	ZINC000257444828
0.3	-7.2	-6.8	0.25	2.103	0.193	2.768	0.334	6	7796	ZINC000050027615
0.3	-9.7	-9.1	0.40	1.391	0.615	3.285	0.692	6	7797	ZINC000104372610
0.3	-7.2	-7.0	0.16	2.087	0.516	3.308	1.239	6	7798	ZINC000012496098
0.3	-8.7	-7.6	0.65	2.888	0.148	5.250	0.825	6	7799	ZINC000000338225
0.3	-9.4	-9.1	0.27	2.243	0.421	4.701	1.632	6	7800	ZINC000015169406
0.3	-8.4	-7.9	0.28	1.748	0.303	5.030	1.609	6	7801	ZINC000257377007
0.3	-6.8	-6.7	0.07	2.125	0.571	4.163	0.237	6	7802	ZINC000015169321
0.3	-7.1	-6.6	0.26	2.792	0.816	4.270	0.784	6	7803	ZINC000001687392
0.3	-8.7	-7.6	0.84	1.737	0.105	5.134	0.342	6	7804	ZINC000006067053
0.3	-5.5	-5.3	0.13	2.687	0.901	3.206	0.971	6	7805	ZINC000095620001
0.3	-5.8	-5.7	0.07	3.081	0.560	4.638	0.529	6	7806	ZINC000034259489
0.3	-8.2	-8.1	0.15	1.814	0.097	4.246	0.502	6	7807	ZINC000257385231
0.3	-9.1	-8.2	0.62	2.061	0.270	4.939	1.217	6	7808	ZINC000256455419

0.3	-8.4	-8.0	0.33	1.148	0.199	2.914	0.389	6	7809	ZINC000015115272
0.3	-6.5	-6.4	0.10	1.958	0.742	2.957	0.667	6	7810	ZINC000100774997
0.3	-5.9	-5.6	0.19	2.109	0.469	3.759	0.801	6	7811	ZINC000000119664
0.3	-8.1	-7.0	0.71	2.525	0.542	4.412	1.809	6	7812	ZINC000014826093
0.3	-7.1	-6.9	0.11	2.393	0.205	3.490	0.521	6	7813	ZINC000005273940
0.3	-6.2	-6.0	0.09	2.822	0.796	5.272	1.104	6	7814	ZINC000034191113
0.3	-6.6	-6.4	0.11	2.953	0.667	4.474	0.587	6	7815	ZINC000001848348
0.3	-6.8	-6.6	0.13	2.307	0.758	4.125	1.112	6	7816	ZINC000005647497
0.3	-6.0	-5.8	0.15	1.771	0.262	3.640	0.161	6	7817	ZINC000014614332
0.3	-9.6	-8.8	0.69	1.481	0.268	2.768	0.449	6	7818	ZINC000014443523
0.3	-6.3	-6.1	0.15	2.837	0.300	4.046	0.551	6	7819	ZINC000013538200
0.3	-6.3	-6.1	0.11	3.116	0.963	4.351	1.273	6	7820	ZINC000001529368
0.3	-6.5	-6.0	0.27	1.881	0.601	4.247	0.507	6	7821	ZINC000014438723
0.3	-5.6	-5.4	0.19	1.902	0.516	3.231	1.070	6	7822	ZINC000005761901
0.3	-5.3	-5.2	0.07	2.161	1.117	2.898	0.702	6	7823	ZINC000004623758

0.3	-6.6	-6.4	0.14	2.797	0.819	3.942	1.273	6	7824	ZINC000001575516
0.3	-5.8	-5.7	0.07	2.361	0.128	4.261	0.483	6	7825	ZINC000032166980
0.3	-7.6	-7.4	0.13	1.852	0.495	3.339	1.730	6	7826	ZINC000040472635
0.3	-6.3	-6.1	0.12	2.220	0.363	3.305	0.551	6	7827	ZINC000000896628
0.3	-9.6	-8.9	0.46	1.295	0.159	4.114	1.336	6	7828	ZINC000100779640
0.3	-8.0	-7.8	0.19	1.084	0.597	3.064	0.539	6	7829	ZINC000014930934
0.3	-5.8	-5.7	0.07	2.454	0.568	4.498	1.701	6	7830	ZINC000001532908
0.3	-6.4	-6.2	0.11	3.002	0.529	4.813	1.110	6	7831	ZINC000002556602
0.3	-8.5	-6.9	0.79	1.770	0.200	4.627	0.719	6	7832	ZINC000086010683
0.3	-6.0	-5.9	0.11	2.088	0.334	3.212	0.852	6	7833	ZINC000015060556
0.3	-5.8	-5.7	0.11	2.190	1.153	3.776	1.200	6	7834	ZINC000002572371
0.3	-9.3	-8.9	0.25	1.919	0.293	4.107	0.373	6	7835	ZINC000014455081
0.3	-5.5	-5.3	0.11	1.545	0.325	3.234	0.771	6	7836	ZINC000100028895
0.3	-4.9	-4.7	0.12	2.191	0.377	3.363	0.425	6	7837	ZINC000003130515
0.3	-7.3	-7.1	0.15	2.346	0.420	4.599	0.795	6	7838	ZINC000004533735

0.3	-6.6	-6.4	0.08	2.468	0.519	3.880	1.488	6	7839	ZINC000001677784
0.3	-7.1	-6.9	0.10	2.650	0.447	4.530	0.787	6	7840	ZINC000013549727
0.3	-8.7	-8.3	0.23	1.628	0.419	3.971	2.216	6	7841	ZINC000257579869
0.3	-6.6	-6.2	0.22	1.847	0.631	2.786	0.104	6	7842	ZINC000004098892
0.3	-7.7	-7.5	0.11	2.513	0.735	4.332	1.835	6	7843	ZINC000006091878
0.3	-8.1	-7.9	0.12	1.898	0.147	4.212	0.397	6	7844	ZINC000015256937
0.3	-5.1	-4.8	0.18	2.703	0.483	3.346	0.726	6	7845	ZINC000005760081
0.3	-9.3	-8.7	0.33	1.790	0.093	5.051	1.779	6	7846	ZINC000015043861
0.3	-8.9	-7.7	0.67	2.066	0.183	5.402	0.623	6	7847	ZINC000015153043
0.3	-4.5	-4.1	0.17	3.486	0.960	4.436	0.957	6	7848	ZINC000002510102
0.3	-6.8	-6.5	0.21	2.469	0.457	3.388	1.243	6	7849	ZINC000015261377
0.3	-8.5	-7.7	0.47	2.947	0.852	5.341	2.018	6	7850	ZINC000100053615
0.3	-6.1	-5.7	0.20	3.275	0.469	5.143	0.651	6	7851	ZINC000038339734
0.3	-6.6	-6.4	0.10	2.217	0.642	3.130	1.061	6	7852	ZINC000004556684
0.3	-8.6	-7.8	0.44	1.859	0.593	2.619	0.676	6	7853	ZINC000100825627

0.3	-8.3	-8.1	0.13	1.969	0.314	4.358	1.023	6	7854	ZINC000013485385
0.3	-7.3	-7.0	0.21	1.337	0.189	2.911	0.948	6	7855	ZINC000001850787
0.3	-5.7	-5.3	0.18	1.960	0.465	3.110	0.778	6	7856	ZINC000005838947
0.3	-5.7	-5.6	0.09	2.450	1.161	3.665	0.782	6	7857	ZINC000000901320
0.3	-4.2	-4.0	0.10	4.096	1.012	4.826	0.546	6	7858	ZINC000001661081
0.3	-6.6	-6.1	0.24	2.691	0.673	4.226	1.432	6	7859	ZINC000004557161
0.3	-9.1	-8.2	0.85	1.987	0.231	4.337	1.269	5	7860	ZINC000014927467
0.3	-8.3	-7.3	0.55	3.393	0.473	5.735	0.251	6	7861	ZINC000004261765
0.3	-8.6	-8.0	0.37	1.907	0.691	4.781	1.021	6	7862	ZINC000013380829
0.3	-5.1	-4.9	0.15	3.512	1.341	4.111	1.591	6	7863	ZINC000005836947
0.3	-8.3	-8.0	0.20	2.747	0.284	4.909	0.636	6	7864	ZINC000257591084
0.3	-8.1	-7.7	0.23	2.175	0.378	4.085	1.104	6	7865	ZINC000100823541
0.3	-9.8	-8.4	1.02	1.454	0.290	5.588	1.712	5	7866	ZINC000100770219
0.3	-7.5	-7.1	0.33	1.841	0.177	4.056	0.911	6	7867	ZINC000014618749
0.3	-7.1	-6.9	0.15	2.453	0.372	3.847	0.752	6	7868	ZINC000000517336

0.3	-4.1	-4.0	0.05	2.576	1.115	3.194	0.826	6	7869	ZINC000100047338
0.3	-6.7	-6.3	0.18	1.782	0.444	3.341	1.764	6	7870	ZINC000032787793
0.3	-6.7	-6.4	0.15	2.256	0.361	5.258	0.157	6	7871	ZINC000032839143
0.3	-5.9	-5.6	0.15	2.127	0.642	4.053	0.358	6	7872	ZINC000005157239
0.3	-7.8	-7.6	0.12	2.004	0.673	3.776	0.605	6	7873	ZINC000257532558
0.3	-5.0	-4.9	0.18	2.309	0.276	2.971	0.377	6	7874	ZINC000002041073
0.3	-5.1	-4.8	0.15	2.613	0.728	3.407	0.945	6	7875	ZINC000005765982
0.3	-4.8	-4.6	0.16	3.369	1.373	4.286	1.327	6	7876	ZINC000005339229
0.3	-7.1	-6.8	0.26	2.363	0.269	4.356	1.128	6	7877	ZINC000095620797
0.3	-5.1	-4.9	0.14	2.528	0.434	3.564	0.392	6	7878	ZINC000000114138
0.3	-7.2	-6.9	0.22	2.740	0.892	3.761	1.604	6	7879	ZINC000000895186
0.3	-6.3	-6.1	0.15	2.865	0.977	4.316	0.592	6	7880	ZINC000002040427
0.3	-6.6	-6.5	0.07	2.760	1.378	4.749	0.577	6	7881	ZINC000005410794
0.3	-7.9	-7.5	0.21	2.003	0.159	3.865	0.858	6	7882	ZINC000015148006
0.3	-6.7	-6.6	0.07	2.308	0.396	4.129	0.703	6	7883	ZINC000001849801

0.3	-6.6	-6.4	0.10	2.614	0.383	5.115	0.868	6	7884	ZINC000004556937
0.3	-6.3	-6.1	0.14	2.604	1.196	3.415	1.422	6	7885	ZINC000015119939
0.3	-6.0	-6.0	0.00	2.400	1.213	3.293	0.688	6	7886	ZINC000100002215
0.3	-5.7	-5.4	0.13	2.075	0.603	3.183	0.593	6	7887	ZINC000014723426
0.3	-6.2	-6.0	0.13	2.539	0.674	4.542	0.574	6	7888	ZINC000002568218
0.3	-9.2	-8.1	0.54	2.155	0.578	5.397	0.985	6	7889	ZINC000014453716
0.3	-6.6	-6.3	0.20	2.261	0.736	3.091	0.331	6	7890	ZINC000100203530
0.3	-6.7	-6.4	0.22	3.052	0.805	4.722	1.196	6	7891	ZINC000004228241
0.3	-6.2	-6.0	0.11	2.439	0.587	4.073	0.746	6	7892	ZINC000000112609
0.3	-8.1	-7.9	0.13	1.890	0.182	4.491	0.146	6	7893	ZINC000014414834
0.3	-7.6	-7.3	0.22	1.634	0.129	4.345	1.585	6	7894	ZINC000004095598
0.3	-8.4	-8.0	0.20	2.049	0.228	5.087	1.125	6	7895	ZINC000014679848
0.3	-8.4	-8.3	0.07	2.065	0.193	5.972	0.335	6	7896	ZINC000015256872
0.3	-8.2	-7.8	0.25	2.035	0.138	5.487	0.482	6	7897	ZINC000004095825
0.3	-4.6	-4.4	0.14	2.211	0.634	2.759	0.924	6	7898	ZINC000003860818

0.3	-6.1	-6.0	0.07	3.438	0.794	5.420	0.625	6	7899	ZINC000001569726
0.3	-6.3	-6.0	0.15	1.667	0.511	2.932	0.437	6	7900	ZINC000100773252
0.3	-4.7	-4.6	0.07	1.667	1.524	3.244	0.928	6	7901	ZINC000001693339
0.3	-7.4	-7.2	0.21	1.872	0.259	4.113	1.678	6	7902	ZINC000014714818
0.3	-9.1	-8.8	0.23	1.605	0.331	2.813	1.068	6	7903	ZINC000100783839
0.3	-5.9	-5.5	0.21	1.993	0.883	3.667	0.712	6	7904	ZINC000001531046
0.3	-7.3	-7.2	0.08	2.453	0.887	3.725	1.220	6	7905	ZINC000040164443
0.3	-7.1	-6.8	0.28	1.891	0.465	3.152	1.540	6	7906	ZINC000034173950
0.3	-9.2	-8.7	0.39	2.403	0.414	6.058	1.348	6	7907	ZINC000085995604
0.3	-7.4	-7.2	0.26	2.212	0.775	3.414	1.679	6	7908	ZINC000006019397
0.3	-5.1	-4.9	0.11	2.990	1.544	3.849	1.645	6	7909	ZINC000005955660
0.3	-10.4	-9.9	0.58	1.519	0.576	4.502	1.617	6	7910	ZINC000257389749
0.3	-10.3	-9.8	0.44	2.088	0.454	4.087	1.000	6	7911	ZINC000100824160
0.3	-9.1	-8.6	0.33	1.866	0.350	3.915	0.848	6	7912	ZINC000004096264
0.3	-7.7	-7.3	0.27	2.375	0.748	4.117	0.472	6	7913	ZINC000001697411

0.3	-5.2	-5.0	0.15	2.577	1.335	4.041	0.996	6	7914	ZINC000001849646
0.3	-4.5	-4.3	0.12	3.097	0.692	3.933	0.626	6	7915	ZINC000002034635
0.3	-8.2	-7.8	0.37	2.530	0.214	4.724	1.141	6	7916	ZINC000015261557
0.3	-4.5	-4.4	0.11	1.599	0.170	2.571	0.269	6	7917	ZINC000013460368
0.3	-9.9	-9.2	0.44	1.258	0.218	3.451	1.100	6	7918	ZINC000100779628
0.3	-6.6	-6.4	0.12	2.603	0.449	3.453	0.472	6	7919	ZINC000095617775
0.3	-7.2	-7.0	0.15	1.900	0.733	4.082	1.100	6	7920	ZINC000000394966
0.3	-6.9	-6.6	0.19	2.599	0.266	4.888	0.628	6	7921	ZINC000095617793
0.3	-8.6	-8.1	0.33	1.783	0.311	3.347	0.895	6	7922	ZINC000014503464
0.3	-7.7	-7.6	0.08	1.710	0.287	4.818	1.144	6	7923	ZINC000014610456
0.3	-5.8	-5.5	0.18	1.941	0.398	3.249	0.633	6	7924	ZINC000030731330
0.3	-7.0	-6.8	0.13	2.060	0.691	3.180	1.198	6	7925	ZINC000013508540
0.3	-6.6	-6.2	0.18	3.024	0.296	5.443	0.207	6	7926	ZINC000034050195
0.3	-10.0	-9.6	0.18	1.636	0.495	4.030	0.765	6	7927	ZINC000001504382
0.3	-9.0	-8.0	0.61	1.734	0.380	3.855	1.647	6	7928	ZINC000015216882

0.3	-8.7	-8.4	0.19	2.184	0.463	3.947	1.250	6	7929	ZINC000014687093
0.3	-5.7	-5.5	0.12	2.687	0.850	3.406	0.984	6	7930	ZINC000001570971
0.3	-8.1	-7.8	0.22	2.028	0.396	3.553	1.083	6	7931	ZINC000257447774
0.3	-3.2	-2.9	0.15	4.023	2.276	4.474	2.106	6	7932	ZINC000000901648
0.3	-8.2	-7.9	0.21	1.815	0.455	4.337	1.457	6	7933	ZINC000015112251
0.3	-8.4	-8.0	0.26	1.600	0.437	5.219	2.203	6	7934	ZINC000015251392
0.3	-8.8	-8.7	0.08	2.456	0.429	4.369	1.447	6	7935	ZINC000000897138
0.3	-7.4	-7.2	0.15	1.799	0.203	5.485	1.074	6	7936	ZINC000014714821
0.3	-7.9	-7.5	0.35	2.124	0.753	3.103	1.428	6	7937	ZINC000004672223
0.3	-7.2	-6.9	0.24	2.216	0.344	3.782	0.734	6	7938	ZINC000033807478
0.3	-7.3	-7.0	0.24	2.074	0.353	4.297	1.175	6	7939	ZINC000100776359
0.3	-7.8	-7.5	0.15	1.764	0.270	5.922	0.244	6	7940	ZINC000027645235
0.3	-9.3	-8.9	0.39	1.761	0.295	3.937	2.101	6	7941	ZINC000014436343
0.3	-8.2	-7.6	0.43	1.474	0.543	3.843	2.543	6	7942	ZINC000014642909
0.3	-6.8	-6.7	0.08	2.317	0.811	3.282	1.279	6	7943	ZINC000000088245

0.3	-5.3	-4.9	0.21	2.306	0.460	3.132	0.639	6	7944	ZINC000003581248
0.3	-5.5	-5.2	0.15	1.849	0.698	2.664	0.690	6	7945	ZINC000013462030
0.3	-7.6	-7.3	0.20	1.858	0.307	3.027	1.156	6	7946	ZINC000033980257
0.3	-6.3	-6.1	0.13	2.106	0.464	3.850	0.705	6	7947	ZINC000001850498
0.3	-9.7	-9.2	0.38	1.214	0.588	2.909	1.163	6	7948	ZINC000100781961
0.3	-6.3	-5.9	0.28	2.481	0.722	3.584	1.277	6	7949	ZINC000013515489
0.3	-5.9	-5.8	0.10	1.815	0.171	3.784	0.337	6	7950	ZINC000028631776
0.3	-9.0	-8.7	0.33	2.225	0.397	4.068	0.651	6	7951	ZINC000014486970
0.3	-9.9	-9.2	0.40	1.248	0.218	4.019	1.248	6	7952	ZINC000100779629
0.3	-7.3	-7.0	0.19	2.291	0.194	5.448	1.076	6	7953	ZINC000001643525
0.3	-5.2	-5.0	0.10	4.970	1.925	6.292	2.017	6	7954	ZINC000004096823
0.3	-8.8	-8.6	0.16	1.705	0.561	2.763	0.549	6	7955	ZINC000014502859
0.3	-7.5	-7.3	0.11	2.173	0.328	4.411	0.909	6	7956	ZINC000013514831
0.3	-7.0	-6.6	0.20	2.527	0.306	3.527	0.716	6	7957	ZINC000001596590
0.3	-6.3	-6.1	0.17	2.572	0.898	4.048	1.539	6	7958	ZINC000001590842

0.3	-7.9	-7.7	0.21	2.475	0.691	4.012	0.593	6	7959	ZINC000015116102
0.3	-7.9	-7.3	0.41	2.298	0.411	4.195	1.051	6	7960	ZINC000014486945
0.3	-6.5	-6.2	0.21	2.554	0.518	4.329	1.134	6	7961	ZINC000002556600
0.3	-6.8	-6.6	0.22	2.218	0.569	4.148	1.325	6	7962	ZINC000013387626
0.3	-4.8	-4.6	0.19	2.324	0.377	3.055	0.633	6	7963	ZINC000000409332
0.3	-7.0	-6.9	0.11	2.099	0.898	3.313	1.041	6	7964	ZINC000000098166
0.3	-4.7	-4.6	0.09	2.501	1.725	3.914	1.403	6	7965	ZINC000000003182
0.3	-6.3	-6.2	0.11	2.624	0.139	4.173	0.693	6	7966	ZINC000001576323
0.3	-8.3	-7.6	0.59	2.262	0.589	5.476	1.559	6	7967	ZINC000014820583
0.3	-9.0	-9.0	0.04	2.315	0.400	4.642	1.307	6	7968	ZINC000015256875
0.3	-5.4	-5.3	0.11	2.289	0.210	3.389	0.478	6	7969	ZINC000090412745
0.3	-8.9	-8.3	0.35	1.451	0.337	3.267	0.472	6	7970	ZINC000004303672
0.3	-6.3	-6.0	0.18	2.235	0.493	3.560	1.288	6	7971	ZINC000004556855
0.3	-5.3	-5.0	0.23	1.487	0.406	2.233	0.469	6	7972	ZINC000001757853
0.3	-5.7	-5.6	0.07	2.215	0.842	3.391	0.656	6	7973	ZINC000014887327

0.3	-7.4	-7.0	0.27	1.769	0.596	4.005	1.136	6	7974	ZINC000014494686
0.3	-6.2	-6.0	0.11	2.624	0.654	4.442	0.541	6	7975	ZINC000002567711
0.3	-10.7	-9.4	0.93	2.003	0.571	3.756	1.380	6	7976	ZINC000014715666
0.3	-7.2	-7.0	0.20	2.198	0.284	4.379	0.829	6	7977	ZINC000004654921
0.3	-6.5	-6.3	0.15	1.995	0.581	2.735	1.086	6	7978	ZINC000000389574
0.3	-9.2	-8.1	0.58	2.319	0.702	4.950	0.942	6	7979	ZINC000100780330
0.3	-6.8	-6.6	0.14	1.602	0.150	4.801	1.433	6	7980	ZINC000014454643
0.3	-8.3	-8.2	0.11	1.785	0.246	3.699	1.234	6	7981	ZINC000013383575
0.3	-8.8	-8.6	0.17	1.801	0.629	3.105	0.760	6	7982	ZINC000257619665
0.3	-5.4	-5.2	0.15	1.921	1.463	3.631	1.200	6	7983	ZINC000000388747
0.3	-6.6	-6.3	0.25	2.092	0.386	4.125	0.496	6	7984	ZINC000000968246
0.3	-8.0	-7.0	0.51	2.532	0.222	5.603	0.231	6	7985	ZINC000004097065
0.3	-9.9	-9.2	0.46	1.278	0.235	3.609	1.314	6	7986	ZINC000100779630
0.3	-5.5	-5.3	0.12	3.251	0.810	4.448	0.379	6	7987	ZINC000006863475
0.3	-8.0	-7.5	0.41	2.079	0.124	5.439	0.351	6	7988	ZINC000100770509

0.3	-10.2	-9.2	0.75	1.694	0.210	3.589	0.108	3	7989	ZINC000100773296
0.3	-5.6	-5.3	0.21	2.303	0.756	3.246	0.868	6	7990	ZINC000000001684
0.3	-6.8	-6.6	0.14	1.580	0.373	4.277	0.973	6	7991	ZINC000230071136
0.3	-7.2	-6.9	0.22	2.255	0.566	3.757	1.460	6	7992	ZINC000013341229
0.3	-6.5	-6.3	0.12	1.976	0.316	4.326	0.732	6	7993	ZINC000034329975
0.3	-9.7	-8.3	0.68	2.102	0.592	4.560	1.777	6	7994	ZINC000014489231
0.3	-9.5	-8.9	0.56	1.529	0.184	4.884	1.733	6	7995	ZINC000257438145
0.3	-6.8	-6.6	0.10	2.987	0.536	3.642	0.526	6	7996	ZINC000016051516
0.3	-5.5	-5.3	0.15	1.781	0.436	2.816	0.897	6	7997	ZINC000014592712
0.3	-9.2	-7.2	1.06	1.549	0.103	3.304	1.052	5	7998	ZINC000014926996
0.3	-8.9	-8.1	0.45	1.620	0.328	4.002	0.645	6	7999	ZINC000100783930
0.3	-7.6	-7.2	0.24	2.216	0.429	2.687	0.657	6	8000	ZINC000002583633
0.3	-6.9	-6.6	0.23	2.271	0.320	5.000	0.238	6	8001	ZINC000013549694
0.3	-6.5	-6.2	0.14	2.580	0.845	3.450	0.830	6	8002	ZINC000000409176
0.3	-6.6	-6.4	0.13	2.396	0.642	4.153	0.274	6	8003	ZINC000000967593

0.3	-5.5	-5.2	0.17	2.575	0.779	3.471	0.962	6	8004	ZINC000013462032
0.3	-7.4	-7.3	0.07	1.582	0.104	4.315	2.345	6	8005	ZINC000149655888
0.3	-7.4	-7.1	0.19	2.217	0.873	3.483	1.513	6	8006	ZINC000000402700
0.3	-7.8	-7.6	0.15	1.863	0.679	3.612	0.595	6	8007	ZINC000100822037
0.3	-7.0	-6.4	0.26	2.035	0.728	2.807	0.491	6	8008	ZINC000005161652
0.3	-8.8	-8.7	0.12	1.816	0.212	6.027	0.918	6	8009	ZINC000015249375
0.3	-4.7	-4.4	0.21	2.414	0.588	3.001	0.629	6	8010	ZINC000002560611
0.3	-6.5	-6.3	0.17	2.147	0.802	3.277	0.106	6	8011	ZINC000100774995
0.3	-6.0	-5.9	0.07	1.792	0.168	3.628	0.262	6	8012	ZINC000001586759
0.3	-5.3	-5.1	0.15	2.194	0.853	3.531	1.263	6	8013	ZINC000002504530
0.3	-6.1	-5.9	0.16	2.762	0.366	3.755	0.597	6	8014	ZINC000000895826
0.3	-8.6	-8.2	0.24	2.028	0.107	6.177	0.867	6	8015	ZINC000257475590
0.3	-5.4	-5.1	0.14	2.064	0.511	2.920	0.726	6	8016	ZINC000001677332
0.3	-3.9	-3.7	0.12	2.529	1.136	3.598	1.149	6	8017	ZINC000001586656
0.3	-6.4	-6.3	0.11	2.666	0.362	4.759	1.191	6	8018	ZINC000095618267

0.3	-5.6	-5.3	0.14	2.225	1.293	4.302	1.294	6	8019	ZINC000001656424
0.3	-7.7	-7.3	0.25	2.322	0.614	4.784	1.768	6	8020	ZINC000014018380
0.3	-8.4	-8.0	0.19	2.001	0.371	4.183	0.626	6	8021	ZINC000015263493
0.3	-5.8	-5.6	0.16	2.370	1.033	3.246	0.934	6	8022	ZINC000002019243
0.3	-8.5	-8.2	0.19	1.614	0.520	3.589	1.183	6	8023	ZINC000015120460
0.3	-7.6	-7.3	0.18	2.708	0.421	4.405	1.363	6	8024	ZINC000013513371
0.3	-5.4	-5.3	0.07	2.805	1.442	3.392	1.613	6	8025	ZINC000005543439
0.3	-10.5	-9.8	0.55	2.064	0.545	4.753	0.964	6	8026	ZINC000002008019
0.3	-9.4	-9.1	0.21	2.379	0.524	4.100	1.296	6	8027	ZINC000257620011
0.3	-9.8	-8.1	0.90	2.271	0.718	5.129	1.873	6	8028	ZINC000100821923
0.3	-6.9	-6.6	0.18	1.641	0.214	3.304	1.008	6	8029	ZINC000005861416
0.3	-8.3	-8.0	0.30	1.927	0.283	5.430	1.798	6	8030	ZINC000014916931
0.3	-7.2	-6.9	0.23	2.322	0.332	4.396	0.692	6	8031	ZINC000001687357
0.3	-4.8	-4.5	0.19	2.395	1.427	3.713	1.758	6	8032	ZINC000000388764
0.3	-5.0	-4.9	0.11	2.147	0.888	2.872	0.952	6	8033	ZINC000001531087

0.3	-7.0	-6.8	0.08	2.692	0.613	4.176	0.722	6	8034	ZINC000022116605
0.3	-6.5	-6.4	0.07	3.145	0.971	4.554	1.464	6	8035	ZINC000001847640
0.3	-5.6	-5.5	0.06	1.869	0.500	2.765	0.333	6	8036	ZINC000021303045
0.3	-6.0	-5.7	0.15	1.889	0.749	5.013	1.444	6	8037	ZINC000005131656
0.3	-4.4	-4.2	0.11	3.162	1.862	3.795	1.812	6	8038	ZINC000000409287
0.3	-6.3	-6.2	0.09	2.217	0.628	3.470	1.280	6	8039	ZINC000005820491
0.3	-9.5	-9.0	0.32	1.455	0.174	4.761	1.693	6	8040	ZINC000100781249
0.3	-8.5	-8.2	0.28	1.912	0.373	3.838	1.373	6	8041	ZINC000257467838
0.3	-6.6	-6.3	0.15	2.075	0.759	3.168	0.465	6	8042	ZINC000100203536
0.3	-7.4	-7.2	0.13	2.799	0.474	5.264	1.233	6	8043	ZINC000050027244
0.3	-9.6	-8.8	0.69	1.412	0.212	3.628	1.880	6	8044	ZINC000014443527
0.3	-7.8	-7.2	0.46	1.915	0.491	2.689	0.637	6	8045	ZINC000040471179
0.3	-8.5	-8.0	0.27	1.764	0.416	3.812	1.828	6	8046	ZINC000015274240
0.3	-4.5	-4.0	0.25	2.096	0.353	2.666	0.474	6	8047	ZINC000000897129
0.3	-7.8	-7.6	0.14	1.948	0.698	3.728	0.627	6	8048	ZINC000100822038

0.3	-10.3	-9.6	0.60	1.988	0.825	3.873	1.421	6	8049	ZINC000000306698
0.3	-7.0	-6.6	0.37	2.131	0.418	3.423	1.387	6	8050	ZINC000034277892
0.3	-6.1	-5.9	0.11	2.791	0.352	4.422	0.412	6	8051	ZINC000015146068
0.3	-7.8	-7.6	0.11	1.535	0.268	3.196	0.560	6	8052	ZINC000015169348
0.3	-6.7	-6.6	0.08	1.801	0.128	2.180	0.224	6	8053	ZINC000014657643
0.3	-9.4	-8.6	0.52	1.494	0.374	3.466	1.345	6	8054	ZINC000059774644
0.3	-5.8	-5.6	0.15	2.572	0.462	3.518	0.749	6	8055	ZINC000021999678
0.3	-8.0	-7.8	0.12	2.421	0.748	4.259	1.424	6	8056	ZINC000013461544
0.3	-7.1	-6.7	0.23	2.189	0.680	3.654	1.131	6	8057	ZINC000001533462
0.3	-6.3	-6.0	0.16	3.169	0.577	4.412	0.651	6	8058	ZINC000005859351
0.3	-9.0	-8.8	0.30	2.002	0.397	4.983	0.587	6	8059	ZINC000014764187
0.3	-8.7	-8.0	0.45	1.968	0.288	4.598	0.557	6	8060	ZINC000253529648
0.3	-6.5	-6.0	0.28	2.540	0.967	3.794	2.151	6	8061	ZINC000069485950
0.3	-5.6	-5.4	0.11	2.812	1.344	4.374	1.298	6	8062	ZINC000001656423
0.3	-3.5	-3.2	0.19	2.728	1.259	3.719	1.037	6	8063	ZINC000001648253

0.3	-8.5	-8.1	0.22	1.865	0.273	5.854	0.890	6	8064	ZINC000257475589
0.3	-10.3	-9.3	0.98	1.255	0.285	4.464	1.643	6	8065	ZINC000257469499
0.3	-9.5	-8.4	0.82	1.597	0.244	3.637	1.969	6	8066	ZINC000026974185
0.3	-8.5	-8.2	0.19	1.991	0.081	5.115	2.135	6	8067	ZINC000257527732
0.3	-7.6	-7.4	0.19	2.450	0.932	3.272	0.920	6	8068	ZINC000000336205
0.3	-6.5	-6.2	0.17	1.986	0.519	3.646	0.722	6	8069	ZINC000002506590
0.3	-8.4	-8.0	0.24	1.948	0.422	4.041	1.241	6	8070	ZINC000014652425
0.3	-9.5	-8.5	0.45	1.781	0.563	5.461	2.177	6	8071	ZINC000015058105
0.3	-7.6	-7.1	0.27	1.896	0.266	4.405	1.278	6	8072	ZINC000004655405
0.3	-8.6	-8.1	0.35	2.203	0.635	4.196	0.914	6	8073	ZINC000014687439
0.3	-6.8	-6.5	0.17	2.404	0.704	4.179	0.913	6	8074	ZINC000257467984
0.3	-8.9	-8.5	0.22	2.119	0.561	5.586	1.296	6	8075	ZINC000001617236
0.3	-4.8	-4.5	0.14	1.984	0.759	2.824	0.704	6	8076	ZINC000000391207
0.3	-7.1	-6.9	0.16	2.271	0.372	3.949	1.540	6	8077	ZINC000003875374
0.3	-10.2	-9.4	0.52	1.820	0.437	4.796	1.415	6	8078	ZINC000100778185

0.3	-8.7	-8.2	0.31	1.475	0.373	3.214	0.460	6	8079	ZINC000253531706
0.2	-8.2	-8.1	0.11	1.813	0.499	3.967	1.330	6	8080	ZINC000015120816
0.2	-7.3	-7.0	0.16	2.296	0.410	4.422	1.427	6	8081	ZINC000050027435
0.2	-5.3	-4.9	0.22	2.575	0.763	3.254	0.654	6	8082	ZINC000004538481
0.2	-6.3	-6.2	0.14	2.408	0.817	3.112	1.169	6	8083	ZINC000002557807
0.2	-6.4	-6.2	0.17	2.084	0.193	3.804	0.937	6	8084	ZINC000050027275
0.2	-6.2	-6.0	0.14	2.851	0.292	4.803	0.612	6	8085	ZINC000002384835
0.2	-4.9	-4.6	0.14	3.142	1.104	4.211	1.271	6	8086	ZINC000003079340
0.2	-6.0	-5.8	0.16	1.193	0.466	3.752	1.090	6	8087	ZINC000003869787
0.2	-7.3	-7.1	0.15	1.803	0.690	3.063	0.910	6	8088	ZINC000000291740
0.2	-8.3	-8.0	0.23	2.045	0.530	4.060	1.189	6	8089	ZINC000015119467
0.2	-6.0	-5.8	0.11	1.823	0.682	3.356	0.544	6	8090	ZINC000000895809
0.2	-8.8	-8.5	0.25	1.945	0.191	4.758	1.199	6	8091	ZINC000014854273
0.2	-6.5	-6.2	0.16	2.590	0.447	3.662	0.762	6	8092	ZINC000000388426
0.2	-8.8	-8.4	0.22	1.154	0.370	5.751	1.743	6	8093	ZINC000257693618

0.2	-5.5	-5.3	0.15	2.225	0.364	2.910	0.535	6	8094	ZINC000003649919
0.2	-5.2	-5.0	0.15	3.115	0.965	4.231	1.613	6	8095	ZINC000002111594
0.2	-5.4	-5.3	0.07	1.324	0.416	3.005	0.799	6	8096	ZINC000002040186
0.2	-9.1	-8.2	0.48	1.973	0.187	4.323	0.783	6	8097	ZINC000085564529
0.2	-7.6	-7.2	0.20	1.901	0.082	4.773	1.485	6	8098	ZINC000071758297
0.2	-8.5	-8.3	0.18	1.891	0.444	4.512	0.659	6	8099	ZINC000014489309
0.2	-5.5	-5.4	0.09	2.572	0.766	3.042	0.764	6	8100	ZINC000013307107
0.2	-5.8	-5.5	0.16	3.263	0.820	4.214	1.162	6	8101	ZINC000001569523
0.2	-6.5	-6.4	0.11	2.641	0.262	3.777	0.289	6	8102	ZINC000000901883
0.2	-5.1	-4.9	0.13	1.848	0.265	2.823	0.456	6	8103	ZINC000000895470
0.2	-6.9	-6.5	0.18	2.079	0.437	4.266	1.159	6	8104	ZINC000085589692
0.2	-7.4	-7.2	0.13	3.399	0.507	4.747	0.815	6	8105	ZINC000000057162
0.2	-8.1	-7.6	0.39	2.465	0.266	6.752	0.140	6	8106	ZINC000257589305
0.2	-5.0	-4.7	0.16	3.798	2.437	5.203	2.174	6	8107	ZINC000000901289
0.2	-6.6	-6.4	0.21	2.171	0.368	3.580	1.103	6	8108	ZINC000050027280

0.2	-5.6	-5.4	0.14	3.048	0.596	4.236	0.994	6	8109	ZINC000002039490
0.2	-6.8	-6.5	0.19	2.091	0.604	4.512	0.990	6	8110	ZINC000012418399
0.2	-6.5	-6.4	0.09	2.246	1.422	3.343	1.141	6	8111	ZINC000000119563
0.2	-9.0	-7.7	0.79	1.627	0.236	4.742	1.468	6	8112	ZINC000061389522
0.2	-6.6	-6.4	0.16	2.693	0.524	4.432	1.186	6	8113	ZINC000004899611
0.2	-6.5	-6.2	0.18	2.701	0.326	4.236	0.536	6	8114	ZINC000001541834
0.2	-8.3	-8.0	0.22	2.724	0.648	4.693	1.660	6	8115	ZINC000005413083
0.2	-9.9	-9.6	0.29	1.784	0.400	3.106	0.981	6	8116	ZINC000100051398
0.2	-5.2	-5.2	0.07	3.043	0.127	3.991	0.165	6	8117	ZINC000002384557
0.2	-8.4	-8.0	0.29	1.843	0.028	5.702	1.452	6	8118	ZINC000257402402
0.2	-9.3	-8.8	0.37	2.111	0.524	4.483	1.355	6	8119	ZINC000253389933
0.2	-7.4	-7.0	0.23	2.058	0.459	3.575	1.296	6	8120	ZINC000033995141
0.2	-6.3	-6.2	0.08	2.203	0.396	4.391	0.850	6	8121	ZINC000018140538
0.2	-10.0	-8.9	0.90	1.604	0.133	5.072	0.833	6	8122	ZINC000014442544
0.2	-9.1	-8.1	0.46	2.095	0.354	4.417	0.740	6	8123	ZINC000100780698

0.2	-8.7	-8.1	0.45	2.433	0.791	3.802	1.306	6	8124	ZINC000001637888
0.2	-5.6	-5.4	0.15	2.017	0.543	3.738	0.637	6	8125	ZINC000000327584
0.2	-8.7	-8.3	0.28	1.589	0.465	3.199	0.425	6	8126	ZINC000004104823
0.2	-7.9	-7.7	0.16	2.013	0.453	3.815	1.343	6	8127	ZINC000020431034
0.2	-10.3	-9.7	0.39	1.775	0.268	5.179	1.743	6	8128	ZINC000015169342
0.2	-8.9	-8.5	0.24	1.998	0.480	3.492	1.132	6	8129	ZINC000001529648
0.2	-7.6	-7.3	0.21	1.560	0.221	3.451	1.157	6	8130	ZINC000003872686
0.2	-9.4	-8.6	0.43	1.987	0.112	5.946	0.663	6	8131	ZINC000014859214
0.2	-6.7	-6.6	0.07	2.620	0.615	4.502	0.416	6	8132	ZINC000005273808
0.2	-6.5	-6.4	0.10	2.470	0.269	3.960	0.426	6	8133	ZINC000040471336
0.2	-7.7	-7.4	0.27	1.760	0.489	2.369	0.755	6	8134	ZINC000040471177
0.2	-5.1	-4.9	0.17	2.177	0.512	3.232	0.516	6	8135	ZINC000000388192
0.2	-10.7	-9.2	1.45	2.305	0.000	5.393	0.000	2	8136	ZINC000104376700
0.2	-8.0	-7.9	0.11	2.020	0.338	5.408	1.312	6	8137	ZINC000014454936
0.2	-5.9	-5.8	0.07	2.566	0.828	3.882	0.977	6	8138	ZINC000001532843

0.2	-5.5	-5.4	0.07	4.498	2.619	5.263	2.779	6	8139	ZINC000005187769
0.2	-9.5	-8.5	0.57	1.690	0.414	4.769	0.750	6	8140	ZINC000005360178
0.2	-8.5	-8.2	0.21	2.576	0.455	3.962	0.843	6	8141	ZINC000005648081
0.2	-8.2	-8.0	0.17	1.901	0.352	3.907	1.013	6	8142	ZINC000257752826
0.2	-8.1	-7.8	0.28	1.942	0.434	5.422	0.447	6	8143	ZINC000014828284
0.2	-5.8	-5.7	0.11	1.949	0.783	3.659	0.668	6	8144	ZINC000004715091
0.2	-7.0	-6.8	0.15	1.662	0.575	2.855	1.015	6	8145	ZINC000001850904
0.2	-6.1	-5.9	0.15	2.387	0.303	4.015	0.721	6	8146	ZINC000006931855
0.2	-4.2	-4.1	0.11	3.085	1.395	3.680	1.586	6	8147	ZINC000032163926
0.2	-6.6	-6.2	0.22	2.525	0.599	3.064	0.689	6	8148	ZINC000000270812
0.2	-9.9	-9.1	0.72	1.829	0.350	3.339	1.414	6	8149	ZINC000034386055
0.2	-5.3	-5.0	0.17	2.205	0.732	3.054	0.806	6	8150	ZINC000012153320
0.2	-6.8	-6.6	0.14	1.837	0.222	4.024	1.382	6	8151	ZINC000085649287
0.2	-8.2	-7.8	0.23	2.763	0.502	4.942	1.536	6	8152	ZINC000018036401
0.2	-6.7	-6.3	0.19	2.756	0.268	3.553	0.372	6	8153	ZINC000000395673

0.2	-5.5	-5.3	0.14	2.667	0.641	3.525	0.861	6	8154	ZINC000000388413
0.2	-6.5	-6.4	0.11	2.311	0.100	3.906	1.329	6	8155	ZINC000001235108
0.2	-6.2	-6.0	0.12	2.109	0.426	3.027	0.621	6	8156	ZINC000001481898
0.2	-6.6	-6.3	0.21	2.862	0.466	3.606	0.447	6	8157	ZINC000002242702
0.2	-8.5	-8.1	0.50	2.281	1.165	3.707	2.210	6	8158	ZINC000095617647
0.2	-5.9	-5.7	0.11	2.663	1.272	4.405	1.277	6	8159	ZINC000001850430
0.2	-6.8	-6.5	0.25	2.185	0.461	3.201	0.720	6	8160	ZINC000013350968
0.2	-9.2	-8.5	0.41	1.486	0.333	4.377	0.925	6	8161	ZINC000230083083
0.2	-8.3	-6.5	0.86	2.397	0.601	4.597	0.526	6	8162	ZINC000100781044
0.2	-7.1	-6.9	0.18	1.788	0.787	4.579	0.950	6	8163	ZINC000003870057
0.2	-7.4	-7.1	0.14	1.534	0.292	4.000	1.362	6	8164	ZINC000257557764
0.2	-7.4	-7.0	0.22	1.938	0.450	4.697	2.217	6	8165	ZINC000004666212
0.2	-8.2	-7.7	0.32	1.933	0.111	4.191	1.053	6	8166	ZINC000013408203
0.2	-9.1	-8.3	0.57	1.594	0.164	4.595	1.267	6	8167	ZINC000013373686
0.2	-9.3	-8.7	0.49	2.011	0.655	3.582	1.153	6	8168	ZINC000000896053

0.2	-7.5	-7.1	0.19	2.376	0.728	3.862	1.374	6	8169	ZINC000006031302
0.2	-6.2	-6.0	0.18	2.062	0.344	3.438	1.228	6	8170	ZINC000004533815
0.2	-9.5	-8.5	0.66	1.448	0.331	5.305	0.887	6	8171	ZINC000100782438
0.2	-6.7	-6.2	0.45	1.913	1.284	2.480	0.979	6	8172	ZINC000014505160
0.2	-7.0	-6.8	0.14	2.217	0.147	3.920	0.699	6	8173	ZINC000255983263
0.2	-5.6	-5.4	0.11	1.958	0.175	2.959	0.224	6	8174	ZINC000003861284
0.2	-5.6	-5.4	0.11	1.587	0.281	3.171	0.559	6	8175	ZINC000001850465
0.2	-7.7	-6.5	0.70	1.858	0.549	3.298	1.927	6	8176	ZINC000004492892
0.2	-6.4	-6.3	0.08	2.655	0.351	4.549	0.333	6	8177	ZINC000050026780
0.2	-3.7	-3.5	0.15	2.133	0.507	2.633	0.366	6	8178	ZINC000001846561
0.2	-8.7	-8.2	0.27	1.813	0.496	4.708	1.800	6	8179	ZINC000000899795
0.2	-8.3	-8.1	0.13	2.679	0.468	4.062	1.667	6	8180	ZINC000002539827
0.2	-9.0	-8.5	0.37	1.905	0.817	4.615	1.764	6	8181	ZINC000005273977
0.2	-6.8	-6.5	0.20	3.117	0.630	4.830	0.482	6	8182	ZINC000013347343
0.2	-5.7	-5.4	0.20	2.630	0.439	3.153	0.392	6	8183	ZINC000001605761

0.2	-7.8	-7.6	0.17	1.597	0.507	3.591	2.035	6	8184	ZINC000013373490
0.2	-5.3	-5.2	0.12	2.439	0.542	3.835	0.347	6	8185	ZINC000001532761
0.2	-7.1	-6.7	0.26	2.416	0.746	4.075	0.589	6	8186	ZINC000002015545
0.2	-3.8	-3.6	0.15	2.889	1.501	3.693	1.422	6	8187	ZINC000000901514
0.2	-4.9	-4.8	0.07	2.183	0.312	3.283	0.675	6	8188	ZINC000000895446
0.2	-7.0	-6.8	0.20	2.347	0.696	3.388	1.418	6	8189	ZINC000000402813
0.2	-5.8	-5.7	0.07	3.208	0.549	4.504	0.665	6	8190	ZINC000012503978
0.2	-8.3	-7.1	0.57	2.971	0.311	5.363	0.686	6	8191	ZINC000008220075
0.2	-5.6	-5.4	0.08	1.876	0.370	3.290	0.228	6	8192	ZINC000005842326
0.2	-8.6	-8.1	0.35	1.324	0.647	3.775	2.261	6	8193	ZINC000257464257
0.2	-6.6	-6.4	0.13	1.795	0.528	3.688	0.579	6	8194	ZINC000001648306
0.2	-9.3	-8.5	0.46	1.878	0.346	4.015	1.280	6	8195	ZINC000014635272
0.2	-5.0	-4.8	0.13	2.259	0.395	3.930	0.829	6	8196	ZINC000014593619
0.2	-8.5	-8.0	0.26	2.331	0.407	4.495	1.688	6	8197	ZINC000095619753
0.2	-7.4	-7.2	0.18	2.240	0.803	3.424	0.833	6	8198	ZINC000004262577

0.2	-6.3	-6.1	0.11	2.377	0.610	3.917	1.475	6	8199	ZINC000012493688
0.2	-9.1	-8.4	0.51	2.006	0.334	4.296	0.481	6	8200	ZINC000100780700
0.2	-8.2	-8.1	0.11	1.704	0.346	3.885	0.779	6	8201	ZINC000257385232
0.2	-7.4	-7.2	0.15	1.771	0.487	4.450	0.784	6	8202	ZINC000028631806
0.2	-8.6	-8.4	0.12	1.769	0.634	5.322	1.740	6	8203	ZINC000015263983
0.2	-8.3	-8.1	0.20	1.726	0.482	3.594	1.345	6	8204	ZINC000257469895
0.2	-6.4	-6.2	0.15	3.097	0.800	4.572	1.183	6	8205	ZINC000019334332
0.2	-9.4	-8.3	0.84	1.956	0.137	5.083	0.215	3	8206	ZINC000004098421
0.2	-5.3	-5.1	0.15	2.005	0.932	2.966	0.714	6	8207	ZINC000002031664
0.2	-6.1	-6.0	0.09	1.850	0.609	2.996	0.380	6	8208	ZINC000006661321
0.2	-6.5	-6.4	0.07	2.155	0.457	3.689	0.603	6	8209	ZINC000090756079
0.2	-7.0	-6.8	0.09	2.600	0.367	3.502	0.506	6	8210	ZINC000000388044
0.2	-10.4	-10.1	0.27	1.899	0.938	4.886	1.216	6	8211	ZINC000230067523
0.2	-6.7	-6.6	0.07	1.832	0.723	3.534	1.069	6	8212	ZINC000001532635
0.2	-8.4	-7.9	0.34	2.755	0.383	4.668	1.034	6	8213	ZINC000004228257

0.2	-6.6	-6.4	0.15	1.456	0.563	3.183	0.169	6	8214	ZINC000257405993
0.2	-8.2	-7.9	0.21	1.168	0.647	3.577	1.604	6	8215	ZINC000085795019
0.2	-5.6	-5.5	0.13	3.347	0.680	3.919	0.743	6	8216	ZINC000000895518
0.2	-6.5	-6.3	0.15	1.770	0.610	3.449	0.358	6	8217	ZINC000104369793
0.2	-8.0	-7.6	0.29	2.845	0.621	5.235	1.476	6	8218	ZINC000050026941
0.2	-9.4	-8.8	0.38	1.492	0.181	4.141	1.480	6	8219	ZINC000100823481
0.2	-5.0	-4.7	0.18	1.822	0.575	2.652	0.605	6	8220	ZINC000000391784
0.2	-6.0	-5.7	0.21	2.175	0.666	3.788	0.480	6	8221	ZINC000003860322
0.2	-6.1	-5.9	0.15	2.709	0.754	3.928	0.672	6	8222	ZINC000002510214
0.2	-4.9	-4.7	0.18	2.844	1.386	3.476	1.539	6	8223	ZINC000001529505
0.2	-6.5	-6.1	0.31	3.314	0.701	4.541	0.623	6	8224	ZINC000002164045
0.2	-7.5	-7.4	0.07	2.097	0.365	3.751	0.677	6	8225	ZINC000015253257
0.2	-6.6	-6.4	0.10	2.122	0.593	3.677	0.260	6	8226	ZINC000018043251
0.2	-8.5	-8.0	0.37	1.780	0.223	5.642	0.764	6	8227	ZINC000014438578
0.2	-9.3	-8.8	0.36	2.157	0.473	4.747	0.935	6	8228	ZINC000013330538

0.2	-9.5	-8.8	0.42	1.849	0.359	4.871	1.851	6	8229	ZINC000003875408
0.2	-8.5	-8.0	0.34	1.998	0.553	4.354	1.119	6	8230	ZINC000032840986
0.2	-6.5	-6.3	0.18	2.827	0.197	4.780	0.526	6	8231	ZINC000001572036
0.2	-6.3	-6.2	0.08	2.231	0.613	2.961	0.983	6	8232	ZINC000001637977
0.2	-5.2	-5.1	0.07	1.546	1.247	3.222	0.340	6	8233	ZINC000005133378
0.2	-8.4	-8.1	0.20	1.979	0.356	5.963	0.777	6	8234	ZINC000013372190
0.2	-6.6	-6.4	0.16	3.575	0.295	5.173	0.244	6	8235	ZINC000004096019
0.2	-9.1	-8.4	0.50	2.031	0.331	4.192	0.477	6	8236	ZINC000100780703
0.2	-5.6	-5.5	0.07	2.172	1.719	3.982	1.645	6	8237	ZINC000000334493
0.2	-9.6	-8.8	0.47	2.199	0.460	4.594	2.079	6	8238	ZINC000100776940
0.2	-8.3	-8.0	0.24	1.994	0.413	3.409	1.802	6	8239	ZINC000018123143
0.2	-6.9	-6.6	0.19	1.596	0.365	4.117	0.444	6	8240	ZINC000002567754
0.2	-6.0	-5.9	0.10	2.029	0.308	3.715	0.564	6	8241	ZINC000003869726
0.2	-6.2	-6.0	0.11	1.608	0.405	3.824	0.783	6	8242	ZINC000256081489
0.2	-7.0	-6.6	0.25	2.525	0.567	3.586	0.948	6	8243	ZINC000008383206

0.2	-4.6	-4.4	0.08	1.618	0.133	3.027	0.563	6	8244	ZINC000000409387
0.2	-8.7	-7.8	0.44	1.904	0.137	4.462	1.104	6	8245	ZINC000014682856
0.2	-7.4	-7.2	0.12	2.049	0.193	3.418	1.162	6	8246	ZINC000001575284
0.2	-4.7	-4.4	0.15	2.418	0.589	3.115	0.734	6	8247	ZINC000000391203
0.2	-9.1	-8.9	0.16	1.479	0.398	3.904	1.104	6	8248	ZINC000033832364
0.2	-7.5	-7.2	0.20	2.291	0.459	4.123	0.573	6	8249	ZINC000012153245
0.2	-9.9	-9.0	0.66	2.034	0.561	5.410	0.534	3	8250	ZINC000257501577
0.2	-6.9	-6.8	0.08	2.976	1.228	4.754	1.849	6	8251	ZINC000003869788
0.2	-9.7	-8.7	1.01	1.957	0.210	4.537	0.587	6	8252	ZINC000100829361
0.2	-5.8	-5.7	0.14	2.304	1.199	4.607	0.703	6	8253	ZINC000044171347
0.2	-8.4	-7.8	0.48	1.723	0.273	3.704	1.048	6	8254	ZINC000085691297
0.2	-4.2	-4.1	0.08	3.748	1.703	4.348	1.736	6	8255	ZINC000000156980
0.2	-6.3	-6.1	0.14	2.265	0.824	4.563	1.491	6	8256	ZINC000001709621
0.2	-6.1	-5.9	0.11	2.429	0.647	4.002	0.978	6	8257	ZINC000082402585
0.2	-8.8	-8.2	0.27	1.789	0.398	4.391	1.898	6	8258	ZINC000015116109

0.2	-4.3	-4.2	0.09	3.086	1.122	4.105	1.265	6	8259	ZINC000003880801
0.2	-8.2	-7.9	0.23	1.876	0.376	4.229	1.356	6	8260	ZINC000238761130
0.2	-9.1	-8.2	0.87	1.829	0.357	5.705	1.155	6	8261	ZINC000014918954
0.2	-5.6	-5.5	0.07	2.733	0.991	4.672	0.659	6	8262	ZINC000027645952
0.2	-8.7	-8.3	0.34	2.008	0.286	4.788	2.049	6	8263	ZINC000006525252
0.2	-8.4	-7.8	0.52	1.748	0.254	4.492	1.342	6	8264	ZINC000015159848
0.2	-6.2	-5.9	0.11	2.364	0.545	3.286	1.204	6	8265	ZINC000002597015
0.2	-4.5	-4.3	0.16	2.241	0.791	2.911	1.228	6	8266	ZINC000002510288
0.2	-8.2	-8.0	0.11	2.043	0.228	4.064	0.739	6	8267	ZINC000005358329
0.2	-8.7	-8.3	0.21	1.689	0.448	4.736	1.292	6	8268	ZINC000005812870
0.2	-9.1	-8.6	0.45	1.600	0.526	4.919	1.607	6	8269	ZINC000086049492
0.2	-9.0	-8.5	0.29	2.124	0.664	3.843	1.604	6	8270	ZINC000001850644
0.2	-6.0	-5.9	0.08	1.660	0.475	3.098	1.002	6	8271	ZINC000014616889
0.2	-7.8	-7.5	0.23	2.744	0.346	6.041	0.358	6	8272	ZINC000085954079
0.2	-5.5	-5.3	0.09	2.733	1.139	3.913	0.951	6	8273	ZINC000001679584

0.2	-7.7	-7.5	0.15	2.186	0.632	4.914	2.198	6	8274	ZINC000002583985
0.2	-9.9	-8.8	0.89	2.067	0.066	4.774	1.009	6	8275	ZINC000136865596
0.2	-7.4	-7.0	0.22	2.045	0.271	4.276	1.467	6	8276	ZINC000005180991
0.2	-8.4	-7.8	0.36	1.673	0.182	4.399	1.272	6	8277	ZINC000012495508
0.2	-4.7	-4.5	0.20	2.407	0.519	3.346	0.510	6	8278	ZINC000000404390
0.2	-9.9	-7.8	1.22	2.310	0.476	4.148	1.239	4	8279	ZINC000014684239
0.2	-4.9	-4.7	0.08	2.760	0.975	3.548	0.869	6	8280	ZINC000000388073
0.2	-6.8	-6.6	0.13	2.187	0.591	5.065	1.852	6	8281	ZINC000014618871
0.2	-7.9	-7.5	0.24	1.809	0.351	4.087	1.184	6	8282	ZINC000013459400
0.2	-5.6	-5.5	0.06	2.220	0.628	3.562	0.261	6	8283	ZINC000005840786
0.2	-4.7	-4.6	0.07	2.640	0.479	3.633	0.414	6	8284	ZINC000000404073
0.2	-8.9	-8.2	0.35	2.431	0.500	4.708	0.966	6	8285	ZINC000034263538
0.2	-6.9	-6.7	0.13	2.395	0.399	4.701	1.990	6	8286	ZINC000013347793
0.2	-4.5	-4.2	0.16	2.023	1.194	3.289	1.132	6	8287	ZINC000000164685
0.2	-4.4	-4.1	0.17	1.988	0.322	2.396	0.331	6	8288	ZINC000001697403

0.2	-4.8	-4.6	0.08	1.746	0.622	3.335	0.872	6	8289	ZINC000001530488
0.2	-9.5	-8.5	0.66	1.447	0.320	5.302	0.887	6	8290	ZINC000257391293
0.2	-7.1	-6.8	0.21	1.770	0.290	4.502	1.649	6	8291	ZINC000004097178
0.2	-8.7	-7.8	0.63	1.836	0.269	4.975	0.753	6	8292	ZINC000100772808
0.2	-9.6	-8.7	0.58	1.578	0.362	4.622	1.204	6	8293	ZINC000100821957
0.2	-5.6	-5.4	0.15	4.685	0.609	6.447	0.133	6	8294	ZINC000001575552
0.2	-8.9	-8.3	0.37	1.941	0.312	5.604	1.494	6	8295	ZINC000014590708
0.2	-6.8	-6.5	0.16	2.072	0.701	3.586	1.448	6	8296	ZINC000034279057
0.2	-7.5	-7.4	0.09	2.044	0.696	3.428	1.599	6	8297	ZINC000139369577
0.2	-6.1	-6.0	0.08	2.880	1.571	3.581	1.437	6	8298	ZINC000001529922
0.2	-6.5	-6.2	0.15	2.268	0.474	3.256	0.613	6	8299	ZINC000012153445
0.2	-10.3	-9.7	0.41	1.974	0.162	5.108	1.968	6	8300	ZINC000100256094
0.2	-8.8	-8.7	0.09	2.524	0.485	4.474	0.943	6	8301	ZINC000004805034
0.2	-5.2	-4.9	0.16	2.191	0.278	3.121	0.657	6	8302	ZINC000001529452
0.2	-8.3	-8.0	0.18	1.929	0.263	3.556	1.036	6	8303	ZINC000006032028

0.2	-7.7	-7.4	0.21	2.059	0.284	4.422	0.480	6	8304	ZINC000008652493
0.2	-6.8	-6.5	0.15	2.415	0.700	4.108	0.743	6	8305	ZINC000000388555
0.2	-8.0	-7.0	0.51	1.528	0.342	4.393	1.863	6	8306	ZINC000013481404
0.2	-5.4	-5.2	0.21	2.634	0.877	3.476	1.153	6	8307	ZINC000002037750
0.2	-6.6	-6.5	0.11	2.568	0.465	4.195	1.444	6	8308	ZINC000004533521
0.2	-6.2	-6.0	0.16	2.655	0.641	3.097	0.751	6	8309	ZINC000000388076
0.2	-6.3	-6.1	0.12	2.960	0.379	4.589	0.520	6	8310	ZINC000050027303
0.2	-4.7	-4.7	0.05	1.687	0.625	3.698	0.372	6	8311	ZINC000001683870
0.2	-5.2	-5.0	0.15	2.822	0.888	4.130	0.882	6	8312	ZINC000005863711
0.2	-7.4	-7.0	0.21	2.142	0.560	4.135	1.581	6	8313	ZINC000015261544
0.2	-7.4	-7.1	0.27	2.169	0.329	3.826	1.757	6	8314	ZINC000014766091
0.2	-8.8	-8.5	0.20	1.655	0.611	3.028	1.201	6	8315	ZINC000257730027
0.2	-8.5	-7.6	0.49	1.926	0.062	6.374	0.344	6	8316	ZINC000100779968
0.2	-6.2	-6.0	0.17	4.129	1.604	5.469	1.574	6	8317	ZINC000014419848
0.2	-8.8	-8.5	0.20	1.661	0.603	3.030	1.191	6	8318	ZINC000257730028

0.2	-6.9	-6.8	0.08	3.256	0.365	5.641	0.457	6	8319	ZINC000100776193
0.2	-4.6	-4.5	0.11	1.856	1.001	3.258	0.768	6	8320	ZINC000001646642
0.2	-5.3	-5.1	0.15	3.640	1.622	4.404	1.615	6	8321	ZINC000013351051
0.2	-6.2	-6.0	0.15	2.283	0.147	3.652	0.425	6	8322	ZINC000001575497
0.2	-5.7	-5.5	0.16	3.551	1.059	5.077	1.399	6	8323	ZINC000001532714
0.2	-9.1	-8.4	0.52	1.868	0.433	4.533	0.245	6	8324	ZINC000100780707
0.2	-7.0	-6.8	0.12	2.106	0.332	4.148	0.698	6	8325	ZINC000100825937
0.2	-6.5	-6.1	0.21	2.611	1.025	3.966	1.783	6	8326	ZINC000002561063
0.2	-7.1	-6.9	0.14	3.102	1.244	4.320	1.377	6	8327	ZINC000006394082
0.2	-8.8	-8.7	0.07	2.615	0.540	4.445	0.851	6	8328	ZINC000100053191
0.2	-7.0	-6.9	0.07	2.247	0.460	4.611	1.111	6	8329	ZINC000095617812
0.2	-8.1	-7.6	0.24	2.195	0.359	5.300	1.029	6	8330	ZINC000028631415
0.2	-9.5	-8.5	0.66	1.447	0.317	5.302	0.887	6	8331	ZINC000257391291
0.2	-8.9	-8.5	0.33	0.915	0.424	3.808	0.831	6	8332	ZINC000261497141
0.2	-5.9	-5.7	0.16	2.466	0.532	3.247	0.818	6	8333	ZINC000001730674

0.2	-6.8	-6.6	0.17	2.600	0.542	4.246	1.409	6	8334	ZINC000095620545
0.2	-6.8	-6.5	0.17	2.234	0.305	2.928	0.428	6	8335	ZINC000002561113
0.2	-7.3	-7.2	0.09	2.004	0.137	5.035	1.558	6	8336	ZINC000033977438
0.2	-5.6	-5.5	0.15	2.604	1.252	4.691	0.598	6	8337	ZINC000001736714
0.2	-6.0	-5.9	0.08	2.761	0.907	4.245	1.597	6	8338	ZINC000012495524
0.2	-5.7	-5.6	0.09	2.173	1.228	2.972	0.961	6	8339	ZINC000000897142
0.2	-7.7	-7.4	0.21	3.161	0.455	4.181	0.205	6	8340	ZINC000000185722
0.2	-6.3	-6.1	0.11	2.492	0.424	4.259	0.724	6	8341	ZINC000013537153
0.2	-4.9	-4.5	0.22	2.218	0.690	2.991	1.032	6	8342	ZINC000000388187
0.2	-8.1	-7.8	0.20	2.459	0.400	5.430	0.351	6	8343	ZINC000002384787
0.2	-9.0	-7.6	0.67	1.975	0.234	5.271	0.557	6	8344	ZINC000015153046
0.2	-6.7	-6.6	0.09	2.800	0.948	4.240	0.395	6	8345	ZINC000000120234
0.2	-7.9	-7.5	0.24	2.395	0.614	3.448	1.155	6	8346	ZINC000004899521
0.2	-6.0	-5.7	0.15	1.205	0.308	3.701	0.860	6	8347	ZINC000005131976
0.2	-6.3	-5.9	0.20	1.967	0.330	3.401	0.599	6	8348	ZINC000001532902

0.2	-9.0	-8.8	0.19	2.099	0.422	4.091	1.259	6	8349	ZINC000100780448
0.2	-7.1	-6.9	0.17	1.753	0.676	3.930	1.563	6	8350	ZINC000000895535
0.2	-6.6	-6.3	0.16	1.496	0.501	3.452	1.465	6	8351	ZINC000238757654
0.2	-8.4	-7.8	0.30	2.013	0.398	4.299	1.360	6	8352	ZINC000005283952
0.2	-8.8	-7.9	0.86	2.145	0.206	4.915	1.505	6	8353	ZINC000095620829
0.2	-8.7	-8.1	0.31	1.640	0.247	2.824	0.306	6	8354	ZINC000014682732
0.2	-3.7	-3.5	0.11	2.386	0.857	3.022	1.044	6	8355	ZINC000001850956
0.2	-7.3	-7.0	0.17	2.539	0.125	5.271	0.874	6	8356	ZINC000005501044
0.2	-8.9	-8.7	0.23	1.925	0.464	4.266	1.156	6	8357	ZINC000013108863
0.2	-6.7	-6.6	0.08	2.740	0.519	4.545	0.926	6	8358	ZINC000012494460
0.2	-4.9	-4.7	0.12	4.131	2.294	5.513	2.357	6	8359	ZINC000006827695
0.2	-6.1	-5.9	0.12	2.444	0.230	4.171	0.658	6	8360	ZINC000001575503
0.2	-6.4	-6.1	0.20	2.108	0.201	4.344	0.410	6	8361	ZINC000002560987
0.2	-6.3	-6.2	0.08	2.808	0.288	4.763	1.194	6	8362	ZINC000050027609
0.2	-6.5	-6.1	0.21	3.068	0.867	4.221	0.976	6	8363	ZINC000242554685

0.2	-7.6	-7.5	0.08	2.391	0.267	5.155	0.571	6	8364	ZINC000039268171
0.2	-4.2	-4.0	0.16	1.567	0.335	2.688	0.409	6	8365	ZINC000002035951
0.2	-5.9	-5.8	0.08	1.940	0.289	3.733	0.962	6	8366	ZINC000001813010
0.2	-6.0	-5.9	0.18	1.912	1.099	2.623	0.836	6	8367	ZINC000000895934
0.2	-8.5	-8.0	0.35	1.978	0.518	4.087	1.258	6	8368	ZINC000015119942
0.2	-6.8	-6.6	0.13	1.773	0.386	3.156	0.956	6	8369	ZINC000004533739
0.2	-6.6	-6.4	0.13	1.763	0.434	3.113	1.353	6	8370	ZINC000004974456
0.2	-3.6	-3.3	0.27	1.860	0.698	2.504	0.635	6	8371	ZINC000008101126
0.2	-6.1	-6.0	0.07	2.858	0.475	3.978	0.959	6	8372	ZINC000015221893
0.2	-5.8	-5.7	0.09	3.519	1.261	5.329	2.034	6	8373	ZINC000004557150
0.2	-6.0	-5.8	0.11	2.429	0.434	4.020	0.527	6	8374	ZINC000001734242
0.2	-5.8	-5.6	0.10	3.155	0.546	4.426	0.564	6	8375	ZINC000012503975
0.2	-8.0	-7.7	0.19	1.556	0.612	3.204	1.296	6	8376	ZINC000014657482
0.2	-5.0	-4.9	0.06	3.981	1.102	4.694	1.038	6	8377	ZINC000001081066
0.2	-6.1	-6.0	0.07	3.880	0.357	5.953	0.892	6	8378	ZINC000005273937

0.2	-6.2	-6.0	0.12	2.000	0.669	3.680	0.931	6	8379	ZINC000004533814
0.2	-8.3	-8.0	0.33	2.014	0.179	5.609	0.111	6	8380	ZINC000006070285
0.2	-8.9	-8.2	0.37	1.777	0.259	4.461	1.575	6	8381	ZINC000257791957
0.2	-6.6	-6.4	0.13	1.946	0.158	2.593	0.444	6	8382	ZINC000001530781
0.2	-4.8	-4.7	0.07	2.798	0.655	3.799	0.727	6	8383	ZINC000095618403
0.2	-9.0	-7.8	0.74	1.976	0.285	3.370	0.346	6	8384	ZINC000196380571
0.2	-9.5	-8.2	0.92	1.496	0.348	3.334	1.830	6	8385	ZINC000002561231
0.2	-8.1	-7.6	0.31	2.123	0.546	4.064	1.805	6	8386	ZINC000140106990
0.2	-8.4	-8.0	0.25	1.768	0.294	5.008	1.613	6	8387	ZINC000100775374
0.2	-10.4	-10.1	0.28	1.813	0.976	4.898	1.219	6	8388	ZINC000100773278
0.2	-4.3	-4.2	0.11	3.109	1.587	3.770	1.371	6	8389	ZINC000002034503
0.2	-6.9	-6.7	0.07	2.832	0.479	4.541	1.037	6	8390	ZINC000034263462
0.2	-5.2	-5.1	0.09	2.840	1.415	4.389	1.133	6	8391	ZINC000100004911
0.2	-8.9	-8.5	0.22	2.115	0.564	5.580	1.287	6	8392	ZINC000001617235
0.2	-8.2	-7.9	0.24	1.622	0.646	3.518	1.686	6	8393	ZINC000005116717

0.2	-6.6	-6.2	0.26	2.717	0.333	3.179	0.243	6	8394	ZINC000000394644
0.2	-5.1	-5.0	0.09	1.869	0.100	2.781	0.353	6	8395	ZINC000012341544
0.2	-8.2	-7.8	0.24	2.036	0.476	3.275	1.138	6	8396	ZINC000003014484
0.2	-9.4	-8.4	0.72	0.965	0.520	3.512	1.734	6	8397	ZINC000086005127
0.2	-7.7	-7.2	0.26	2.371	0.442	4.266	1.178	6	8398	ZINC000015219975
0.2	-8.7	-7.8	0.49	2.474	0.746	5.348	1.434	6	8399	ZINC000004217451
0.2	-8.2	-7.6	0.30	1.795	0.267	5.585	0.888	6	8400	ZINC000257501606
0.2	-8.9	-8.7	0.20	2.172	0.949	4.106	0.707	6	8401	ZINC000014590817
0.2	-7.2	-7.0	0.16	2.783	0.634	3.983	0.266	6	8402	ZINC000000057058
0.2	-8.1	-7.2	0.61	2.369	0.759	4.796	1.899	6	8403	ZINC000031357002
0.2	-9.2	-8.8	0.24	2.003	0.652	3.752	1.684	6	8404	ZINC000005669265
0.2	-5.8	-5.5	0.15	1.897	0.430	3.150	1.135	6	8405	ZINC000095618177
0.2	-8.5	-7.6	0.45	1.979	0.218	5.553	1.027	6	8406	ZINC000005998713
0.2	-8.0	-7.8	0.13	2.633	0.606	4.987	1.696	6	8407	ZINC000004762006
0.2	-7.9	-7.5	0.32	2.063	0.357	4.348	0.740	6	8408	ZINC000100822080

0.2	-6.5	-6.3	0.13	1.413	0.451	3.252	0.475	6	8409	ZINC000012153502
0.2	-4.9	-4.9	0.04	1.769	1.180	3.669	0.497	6	8410	ZINC000001653137
0.2	-8.1	-7.7	0.21	2.025	0.671	3.646	1.779	6	8411	ZINC000014719905
0.2	-8.1	-7.6	0.24	2.409	0.405	4.242	1.639	6	8412	ZINC000148751572
0.2	-5.7	-5.5	0.14	1.773	0.552	3.064	1.042	6	8413	ZINC000002510279
0.2	-7.7	-7.4	0.23	1.641	0.292	2.863	0.808	6	8414	ZINC000014444408
0.2	-9.3	-8.8	0.26	2.156	0.428	3.998	1.016	6	8415	ZINC000257464485
0.2	-6.9	-6.6	0.19	2.345	0.439	3.669	1.049	6	8416	ZINC000004097029
0.2	-8.8	-8.5	0.19	1.715	0.576	3.019	1.192	6	8417	ZINC000257730026
0.2	-9.3	-8.5	0.46	1.635	0.362	4.427	1.564	6	8418	ZINC000257470334
0.2	-8.1	-7.4	0.37	1.960	0.191	5.322	1.255	6	8419	ZINC000150373589
0.2	-9.0	-8.5	0.26	1.973	0.591	2.958	0.808	6	8420	ZINC000001590614
0.2	-5.8	-5.7	0.07	3.202	0.554	4.502	0.664	6	8421	ZINC000012503977
0.2	-6.9	-6.7	0.13	2.861	0.527	5.353	0.845	6	8422	ZINC000004556661
0.2	-7.3	-6.9	0.30	1.613	0.416	2.352	0.308	6	8423	ZINC000001719253

0.2	-7.6	-7.4	0.19	1.898	0.412	3.116	1.042	6	8424	ZINC000095618258
0.2	-5.8	-5.7	0.08	1.393	0.442	3.787	1.038	6	8425	ZINC000100029048
0.2	-4.7	-4.5	0.16	1.027	0.211	2.379	0.458	6	8426	ZINC000002039889
0.2	-6.8	-6.8	0.05	1.677	1.026	4.282	0.993	6	8427	ZINC000257493861
0.2	-4.3	-3.9	0.17	1.923	1.294	2.586	1.217	6	8428	ZINC000006037875
0.2	-5.5	-5.4	0.07	2.465	0.580	3.828	1.241	6	8429	ZINC000014611346
0.2	-6.6	-6.3	0.18	2.649	0.412	5.149	0.496	6	8430	ZINC000014443859
0.2	-5.8	-5.7	0.09	3.075	0.480	4.613	0.683	6	8431	ZINC000013434913
0.2	-6.0	-5.9	0.12	2.196	0.597	3.818	0.808	6	8432	ZINC000011535715
0.2	-8.8	-8.5	0.14	2.173	0.167	5.224	0.395	6	8433	ZINC000100053204
0.2	-7.7	-7.3	0.20	2.430	0.818	4.374	1.355	6	8434	ZINC000257544210
0.2	-8.4	-8.0	0.24	1.664	0.184	4.710	1.443	6	8435	ZINC000257377005
0.2	-4.5	-4.2	0.18	3.067	1.440	4.358	1.297	6	8436	ZINC000000391793
0.2	-7.1	-6.9	0.18	2.500	0.756	3.407	0.673	6	8437	ZINC000014592899
0.2	-6.7	-6.4	0.24	2.120	0.662	2.974	1.253	6	8438	ZINC000004222913

0.2	-8.5	-8.2	0.23	2.125	0.289	4.747	1.403	6	8439	ZINC000014504547
0.2	-8.6	-8.3	0.21	1.808	0.318	4.773	0.417	6	8440	ZINC000014587259
0.2	-7.9	-7.4	0.33	2.260	0.237	4.059	1.099	6	8441	ZINC000013783205
0.2	-6.6	-6.3	0.20	3.981	0.609	5.110	0.760	6	8442	ZINC000000402941
0.2	-8.3	-7.9	0.21	2.047	0.531	5.385	1.316	6	8443	ZINC000015120293
0.2	-7.8	-7.5	0.16	1.809	0.388	2.541	0.638	6	8444	ZINC000255982385
0.2	-10.3	-9.4	0.79	1.530	0.641	3.945	1.710	6	8445	ZINC000100780534
0.2	-8.3	-7.1	0.75	2.324	0.517	4.910	1.356	6	8446	ZINC000026387738
0.2	-6.8	-6.5	0.21	3.227	1.147	4.593	1.066	6	8447	ZINC000000391122
0.2	-4.6	-4.6	0.05	4.527	2.066	4.964	2.360	6	8448	ZINC000005339228
0.2	-5.8	-5.8	0.04	1.889	0.269	4.900	1.365	6	8449	ZINC000033839262
0.2	-6.6	-6.4	0.19	2.167	1.107	3.836	1.085	6	8450	ZINC000257405995
0.2	-8.1	-7.7	0.24	2.261	0.368	3.636	1.301	6	8451	ZINC000013403354
0.2	-7.7	-7.5	0.15	2.512	0.306	4.877	1.503	6	8452	ZINC000033977437
0.2	-8.4	-8.2	0.14	2.429	0.319	4.743	1.188	6	8453	ZINC000050026949

0.2	-8.0	-7.6	0.22	1.718	0.209	3.774	0.582	6	8454	ZINC000002040988
0.2	-6.4	-6.3	0.11	2.277	0.510	3.736	1.134	6	8455	ZINC000014588554
0.2	-8.2	-7.6	0.47	1.656	0.481	4.331	2.098	6	8456	ZINC000000899105
0.2	-7.4	-7.1	0.18	2.194	0.207	5.508	1.471	6	8457	ZINC000085808379
0.2	-6.1	-5.8	0.23	2.678	0.170	4.120	0.754	6	8458	ZINC000069485952
0.2	-7.7	-7.2	0.38	3.003	0.849	4.259	1.465	6	8459	ZINC000000083317
0.2	-6.5	-6.3	0.13	1.687	0.621	3.139	0.465	6	8460	ZINC000104369800
0.2	-5.8	-5.5	0.20	1.921	0.333	2.706	0.229	6	8461	ZINC000014723427
0.2	-8.2	-7.7	0.43	2.009	0.281	3.133	0.671	6	8462	ZINC000230064858
0.2	-8.1	-7.5	0.42	2.146	0.261	4.769	1.362	6	8463	ZINC000095617808
0.2	-4.7	-4.5	0.09	3.114	1.162	4.155	1.134	6	8464	ZINC000000403051
0.2	-8.3	-8.0	0.18	1.674	0.257	3.744	1.685	6	8465	ZINC000015249522
0.2	-6.2	-5.6	0.27	1.757	0.472	2.922	0.948	6	8466	ZINC000000389562
0.2	-5.1	-4.9	0.18	4.177	2.506	4.850	2.257	6	8467	ZINC000000895210
0.2	-6.1	-5.9	0.09	1.332	0.270	3.702	0.491	6	8468	ZINC000079566585

0.2	-6.0	-5.8	0.17	2.982	1.110	3.696	1.071	6	8469	ZINC000082069927
0.2	-7.0	-6.8	0.22	2.301	0.807	3.669	1.174	6	8470	ZINC000000058172
0.2	-6.3	-6.0	0.21	2.195	0.130	3.736	0.955	6	8471	ZINC000212543132
0.2	-7.7	-7.3	0.30	2.260	0.278	3.285	0.966	6	8472	ZINC000014766021
0.2	-7.6	-7.0	0.36	1.992	0.191	4.395	1.371	6	8473	ZINC000004544315
0.2	-3.7	-3.6	0.06	2.553	0.503	3.357	0.278	6	8474	ZINC000002563976
0.2	-7.8	-7.3	0.27	2.066	0.620	3.897	1.675	6	8475	ZINC000014859824
0.2	-4.6	-4.4	0.11	2.684	0.648	3.828	0.396	6	8476	ZINC000014593080
0.2	-6.1	-5.9	0.13	2.048	1.039	3.071	0.947	6	8477	ZINC000000896402
0.2	-9.6	-8.2	0.74	2.059	0.486	4.736	1.280	6	8478	ZINC000257433874
0.2	-8.7	-8.2	0.32	1.670	0.164	3.324	0.591	6	8479	ZINC000014714522
0.2	-7.8	-7.2	0.33	2.297	0.493	3.732	1.345	6	8480	ZINC000034382704
0.2	-5.7	-5.4	0.15	3.044	0.817	3.692	1.064	6	8481	ZINC000001605763
0.2	-8.4	-7.9	0.24	1.793	0.529	4.322	0.343	6	8482	ZINC000004803379
0.2	-9.0	-8.0	0.57	1.860	0.275	5.327	0.853	6	8483	ZINC000257592649

0.2	-4.1	-3.9	0.11	2.539	0.832	3.217	0.835	6	8484	ZINC000100828523
0.2	-6.3	-6.2	0.07	3.108	1.053	4.711	1.628	6	8485	ZINC000002389593
0.2	-8.3	-8.2	0.05	1.605	0.182	4.907	2.346	6	8486	ZINC000256122338
0.2	-7.9	-7.8	0.06	1.821	1.002	3.867	0.909	6	8487	ZINC000000164363
0.2	-6.6	-6.4	0.15	2.709	0.641	4.117	0.727	6	8488	ZINC000257524206
0.2	-6.1	-6.0	0.09	3.863	0.425	5.207	0.532	6	8489	ZINC000005113158
0.2	-6.4	-6.1	0.16	2.188	0.358	4.531	0.729	6	8490	ZINC000002390911
0.2	-5.6	-5.3	0.18	3.386	1.464	4.571	1.520	6	8491	ZINC000001575551
0.2	-7.7	-7.3	0.20	2.349	0.773	4.155	0.470	6	8492	ZINC000006066936
0.2	-8.4	-7.7	0.56	1.725	0.199	4.962	0.967	6	8493	ZINC000100779963
0.2	-6.9	-6.7	0.13	2.143	0.627	3.855	0.463	6	8494	ZINC000000001084
0.2	-7.3	-7.1	0.15	1.837	0.183	4.563	0.717	6	8495	ZINC000001532510
0.2	-7.7	-7.2	0.25	2.108	0.551	3.515	1.151	6	8496	ZINC000257487222
0.2	-8.1	-7.6	0.25	2.254	0.521	4.185	1.666	6	8497	ZINC000014928782
0.2	-10.3	-9.9	0.25	2.399	0.787	4.223	1.222	6	8498	ZINC000138793099

0.2	-8.5	-8.2	0.19	2.136	0.604	4.734	1.360	6	8499	ZINC000012661824
0.2	-7.6	-7.3	0.15	1.918	0.443	3.480	0.611	6	8500	ZINC000006031742
0.2	-7.5	-7.1	0.20	1.953	0.173	2.468	0.375	6	8501	ZINC000095617734
0.2	-6.9	-6.5	0.19	2.022	0.307	3.151	1.665	6	8502	ZINC000015261576
0.2	-8.4	-8.3	0.14	1.418	0.712	4.807	1.971	6	8503	ZINC000257617471
0.2	-3.8	-3.6	0.13	3.055	1.333	4.106	1.341	6	8504	ZINC000000895412
0.2	-5.7	-5.5	0.14	1.926	0.661	3.172	0.574	6	8505	ZINC000001712308
0.2	-9.4	-8.8	0.41	1.997	0.377	4.425	0.898	6	8506	ZINC000105495168
0.2	-5.3	-5.0	0.20	2.685	0.659	3.610	0.894	6	8507	ZINC000095620541
0.2	-8.5	-7.4	0.65	1.795	0.462	4.004	2.139	6	8508	ZINC000004534050
0.2	-7.7	-7.1	0.33	1.856	0.212	5.165	1.535	6	8509	ZINC000012496243
0.2	-4.8	-4.7	0.04	2.739	0.569	3.368	0.679	6	8510	ZINC000019735340
0.2	-9.9	-9.3	0.40	1.765	0.694	5.953	1.909	6	8511	ZINC000003875593
0.2	-6.8	-6.6	0.16	1.515	0.150	3.061	0.797	6	8512	ZINC000032840885
0.2	-8.7	-8.3	0.36	1.520	0.516	5.043	1.997	6	8513	ZINC000001609699

0.2	-5.8	-5.6	0.10	2.977	0.658	4.225	0.495	6	8514	ZINC000257681277
0.2	-8.7	-8.3	0.28	1.415	0.344	3.047	0.247	6	8515	ZINC000014443429
0.2	-5.5	-5.4	0.05	2.376	0.681	3.572	1.349	6	8516	ZINC000008602482
0.2	-7.4	-7.2	0.18	2.211	0.570	3.915	1.698	6	8517	ZINC000015261255
0.2	-9.7	-8.4	1.02	1.742	0.147	4.978	1.405	4	8518	ZINC000014410872
0.2	-6.8	-6.4	0.30	3.360	0.550	4.340	0.629	6	8519	ZINC000000404470
0.2	-9.0	-7.6	1.09	2.069	0.564	5.390	1.345	6	8520	ZINC000014820400
0.2	-4.2	-4.0	0.10	2.271	0.906	3.529	0.690	6	8521	ZINC000000901192
0.2	-9.1	-8.3	0.55	1.702	0.518	3.693	1.695	6	8522	ZINC000095617914
0.2	-7.0	-6.9	0.19	2.199	0.507	4.021	1.914	6	8523	ZINC000002575495
0.2	-4.1	-3.9	0.12	2.379	0.440	2.817	0.556	6	8524	ZINC000100828528
0.2	-7.9	-7.6	0.23	2.704	0.405	5.204	1.025	6	8525	ZINC000013508203
0.2	-7.8	-7.5	0.18	1.486	0.230	2.967	0.895	6	8526	ZINC000257433060
0.2	-8.6	-7.9	0.47	2.015	0.190	3.602	0.910	6	8527	ZINC000100828884
0.2	-8.4	-8.2	0.15	1.801	0.331	3.509	1.847	6	8528	ZINC000100828445

0.2	-9.6	-8.8	0.46	2.069	0.331	5.365	2.069	6	8529	ZINC000014452717
0.2	-7.1	-7.1	0.05	1.725	0.602	4.284	0.334	6	8530	ZINC000100034211
0.2	-10.5	-10.0	0.30	1.764	0.405	4.377	0.613	6	8531	ZINC000257749155
0.2	-7.6	-7.1	0.32	1.749	0.310	4.849	1.281	6	8532	ZINC000033822371
0.2	-5.0	-4.9	0.07	3.384	0.397	4.093	0.307	6	8533	ZINC000001532854
0.2	-9.6	-9.0	0.44	1.622	0.313	3.911	1.650	6	8534	ZINC000257564794
0.2	-6.4	-6.2	0.07	2.914	0.653	4.935	0.791	6	8535	ZINC000034329974
0.2	-7.1	-7.0	0.14	2.505	1.064	3.760	1.955	6	8536	ZINC000004533526
0.2	-7.2	-6.6	0.26	1.933	0.751	4.542	1.803	6	8537	ZINC000095099395
0.2	-9.6	-8.7	0.79	1.465	0.339	3.855	1.604	6	8538	ZINC000100821961
0.2	-10.1	-9.0	0.73	2.049	0.707	4.963	1.914	6	8539	ZINC000100830112
0.2	-8.2	-7.9	0.24	1.759	0.461	4.246	1.368	6	8540	ZINC000139182108
0.2	-8.9	-8.5	0.24	1.924	0.267	5.834	1.136	6	8541	ZINC000013517387
0.2	-6.5	-6.3	0.15	2.089	0.484	2.432	0.517	6	8542	ZINC000000206825
0.2	-4.1	-3.9	0.09	2.566	1.220	3.340	0.896	6	8543	ZINC000002009758

0.2	-6.0	-6.0	0.05	2.236	0.479	4.786	0.746	6	8544	ZINC000014712751
0.2	-5.6	-5.3	0.15	2.512	1.714	4.250	1.340	6	8545	ZINC000100030112
0.2	-5.2	-5.1	0.07	1.949	0.617	3.105	0.554	6	8546	ZINC000002560879
0.2	-5.1	-4.9	0.13	1.722	0.355	2.722	0.729	6	8547	ZINC000005818638
0.2	-9.0	-8.4	0.56	1.609	0.234	4.755	1.951	6	8548	ZINC000005999029
0.2	-9.6	-8.5	0.81	1.904	0.416	5.399	0.720	6	8549	ZINC000257564790
0.2	-7.5	-7.1	0.23	2.482	0.249	6.176	0.213	6	8550	ZINC000001643524
0.2	-7.4	-7.3	0.12	1.543	0.435	4.016	1.245	6	8551	ZINC000197960184
0.2	-6.2	-6.1	0.09	2.619	1.158	4.284	1.348	6	8552	ZINC000001575555
0.2	-5.5	-5.4	0.10	2.683	1.097	4.191	0.657	6	8553	ZINC000014616802
0.2	-8.4	-8.1	0.28	2.488	0.858	4.803	0.538	6	8554	ZINC000195853999
0.2	-4.4	-4.3	0.12	3.172	0.324	4.100	0.425	6	8555	ZINC000002013201
0.2	-7.2	-6.7	0.34	2.464	1.203	3.145	1.209	6	8556	ZINC000000145536
0.2	-9.9	-8.9	0.97	1.812	0.395	4.245	2.043	6	8557	ZINC000257569242
0.2	-8.7	-7.9	0.46	1.841	0.668	3.519	1.328	6	8558	ZINC000004096990

0.2	-6.9	-6.6	0.16	2.849	0.165	4.110	0.599	6	8559	ZINC000002575123
0.2	-6.3	-6.0	0.21	2.962	0.899	4.601	1.752	6	8560	ZINC000001578623
0.2	-7.5	-7.4	0.16	2.320	0.520	3.667	1.484	6	8561	ZINC000100782472
0.2	-6.0	-5.7	0.18	1.611	0.194	2.631	0.282	6	8562	ZINC000000900491
0.2	-11.0	-10.6	0.54	0.236	0.026	4.785	0.054	3	8563	ZINC000005845723
0.2	-5.5	-5.4	0.10	3.673	0.517	5.060	0.803	6	8564	ZINC000001569524
0.2	-6.3	-6.1	0.19	1.955	0.982	4.420	1.867	6	8565	ZINC000001633876
0.2	-8.5	-8.2	0.17	2.648	0.241	5.224	0.379	6	8566	ZINC000095617809
0.2	-8.1	-7.9	0.15	1.996	0.767	3.148	1.772	6	8567	ZINC000002169830
0.2	-8.7	-8.3	0.29	1.686	0.181	3.084	1.374	6	8568	ZINC000013323022
0.2	-6.6	-6.5	0.10	1.787	0.606	3.064	1.497	6	8569	ZINC000005274097
0.2	-7.5	-7.4	0.07	2.237	0.310	5.107	0.900	6	8570	ZINC000040165302
0.2	-5.7	-5.4	0.13	1.947	0.374	3.449	0.556	6	8571	ZINC000001850363
0.2	-6.9	-6.7	0.13	2.314	0.334	3.690	0.948	6	8572	ZINC000012494868
0.2	-7.6	-7.4	0.16	2.213	0.264	3.787	0.985	6	8573	ZINC000038919972

0.2	-7.1	-6.9	0.17	2.658	0.457	4.058	1.404	6	8574	ZINC000002522621
0.2	-7.1	-6.8	0.13	2.392	0.761	3.792	0.652	6	8575	ZINC000015205059
0.2	-6.6	-6.4	0.11	2.299	0.162	3.915	0.930	6	8576	ZINC000014638740
0.2	-7.1	-7.0	0.08	2.301	0.192	5.095	1.797	6	8577	ZINC000095617703
0.2	-7.8	-7.4	0.25	2.210	0.498	4.142	2.123	6	8578	ZINC000095619741
0.2	-5.8	-5.7	0.07	3.205	0.552	4.503	0.667	6	8579	ZINC000257681278
0.2	-8.4	-8.2	0.17	1.789	0.127	5.077	1.474	6	8580	ZINC000257617470
0.2	-5.8	-5.7	0.09	2.702	0.740	3.575	1.108	6	8581	ZINC000012503976
0.2	-8.2	-8.1	0.09	1.868	0.261	4.488	1.439	6	8582	ZINC000095620814
0.2	-6.1	-6.0	0.06	3.173	0.837	4.581	1.139	6	8583	ZINC000002036815
0.2	-6.7	-6.6	0.09	2.154	0.463	3.953	0.976	6	8584	ZINC000032839141
0.2	-4.3	-4.2	0.11	3.099	1.616	3.585	1.505	6	8585	ZINC000001696686
0.2	-5.7	-5.4	0.23	2.151	1.233	3.335	1.195	6	8586	ZINC000005821935
0.2	-7.7	-7.4	0.28	2.013	0.130	5.758	0.645	6	8587	ZINC000065731641
0.2	-6.2	-6.1	0.07	1.717	0.846	3.483	0.444	6	8588	ZINC000013385873

0.2	-7.2	-7.0	0.15	1.552	0.124	2.668	0.312	6	8589	ZINC000013433435
0.2	-6.5	-6.3	0.15	1.423	0.456	3.258	0.456	6	8590	ZINC000012153499
0.2	-8.5	-7.7	0.46	1.983	0.076	4.456	1.144	6	8591	ZINC000014826959
0.2	-8.6	-8.0	0.31	2.144	0.241	5.488	0.215	6	8592	ZINC000025723899
0.2	-6.8	-6.6	0.15	3.014	0.343	4.902	0.436	6	8593	ZINC000001532910
0.2	-8.4	-7.6	0.54	1.453	0.281	3.903	1.457	6	8594	ZINC000256116516
0.2	-9.1	-8.7	0.31	1.822	0.602	3.753	1.531	6	8595	ZINC000196583471
0.2	-6.4	-6.3	0.07	2.184	0.297	4.436	1.154	6	8596	ZINC000034114360
0.2	-8.1	-7.9	0.12	2.931	0.804	5.479	1.982	6	8597	ZINC000013347171
0.2	-6.5	-6.2	0.22	2.957	0.421	3.849	0.677	6	8598	ZINC000000403588
0.2	-5.2	-5.0	0.13	1.940	0.736	3.062	0.385	6	8599	ZINC000014495014
0.2	-9.5	-8.9	0.43	2.684	0.277	4.813	1.004	6	8600	ZINC000085996406
0.2	-6.5	-6.3	0.16	1.945	0.216	3.279	0.633	6	8601	ZINC000002516118
0.2	-8.7	-8.3	0.24	2.555	0.384	4.610	1.072	6	8602	ZINC000001576166
0.2	-8.7	-8.2	0.32	1.466	0.361	3.258	0.442	6	8603	ZINC000014443425

0.2	-9.6	-7.7	1.11	1.914	0.257	3.761	1.537	4	8604	ZINC000003984016
0.2	-5.3	-5.0	0.13	2.442	0.527	4.456	0.490	6	8605	ZINC000001532734
0.2	-8.2	-7.8	0.25	1.718	0.184	3.486	0.885	6	8606	ZINC000013783204
0.2	-9.3	-8.9	0.28	2.164	0.435	3.999	1.016	6	8607	ZINC000100780724
0.2	-9.0	-8.3	0.38	2.005	0.478	3.741	1.236	6	8608	ZINC000034046037
0.2	-8.5	-8.4	0.11	2.568	0.223	4.467	0.516	6	8609	ZINC000040165418
0.2	-5.2	-5.0	0.12	2.593	0.921	4.847	0.679	6	8610	ZINC000014584223
0.2	-6.3	-6.2	0.11	2.098	0.776	3.727	1.049	6	8611	ZINC000050027413
0.2	-8.2	-7.9	0.15	1.652	0.582	3.542	1.736	6	8612	ZINC000014859990
0.2	-9.6	-9.6	0.00	0.000	0.000	0.000	0.000	1	8613	ZINC000014635133
0.2	-6.4	-6.0	0.22	2.972	0.297	4.835	0.590	6	8614	ZINC000050027376
0.2	-9.4	-8.5	0.43	1.946	0.389	5.478	2.134	6	8615	ZINC000257527918
0.2	-4.9	-4.7	0.21	2.668	1.037	3.904	0.967	6	8616	ZINC000000158556
0.2	-7.9	-7.8	0.09	2.118	0.454	4.332	1.672	6	8617	ZINC000014953674
0.2	-8.4	-7.8	0.41	1.682	0.718	3.020	0.807	6	8618	ZINC000015046795

0.2	-7.0	-6.7	0.16	2.628	1.231	3.322	1.507	6	8619	ZINC000006092852
0.2	-5.7	-5.3	0.17	2.330	0.906	3.013	0.592	6	8620	ZINC000039204748
0.2	-5.4	-5.1	0.25	1.523	0.853	2.331	0.781	6	8621	ZINC000000407032
0.2	-9.9	-8.8	0.84	1.894	0.305	3.925	1.451	6	8622	ZINC000014717651
0.2	-5.0	-4.9	0.07	5.828	0.193	6.800	0.248	6	8623	ZINC000003873028
0.2	-5.9	-5.6	0.18	3.103	1.478	5.508	1.023	6	8624	ZINC000014453731
0.2	-6.3	-6.1	0.15	3.306	0.589	5.248	1.345	6	8625	ZINC000004899564
0.2	-5.8	-5.6	0.11	1.457	0.426	4.204	0.427	6	8626	ZINC000100028960
0.2	-9.8	-9.5	0.20	1.667	0.332	5.103	2.085	6	8627	ZINC000003869768
0.2	-6.8	-6.6	0.13	2.081	0.420	3.885	1.729	6	8628	ZINC000015261580
0.2	-8.1	-6.7	0.67	2.009	0.384	4.517	1.904	6	8629	ZINC000257561269
0.2	-6.5	-6.3	0.11	2.433	0.189	4.279	0.330	6	8630	ZINC000014817953
0.2	-9.5	-8.5	0.66	1.751	0.299	5.188	0.945	6	8631	ZINC000000402701
0.2	-8.6	-8.2	0.29	2.065	0.971	4.048	1.863	6	8632	ZINC000085994613
0.2	-6.6	-6.4	0.13	2.917	0.202	4.248	0.907	6	8633	ZINC000015261519

0.2	-5.5	-5.2	0.25	2.031	1.002	3.298	1.108	6	8634	ZINC000001684719
0.2	-9.1	-7.4	0.97	1.384	0.095	4.613	0.840	5	8635	ZINC000015119697
0.2	-6.4	-6.2	0.09	2.276	0.598	3.455	1.181	6	8636	ZINC000005440503
0.2	-8.3	-7.5	0.50	1.881	0.130	6.692	0.681	6	8637	ZINC000100779966
0.2	-6.7	-6.5	0.16	1.909	0.445	4.442	0.410	6	8638	ZINC000014588512
0.2	-6.3	-6.2	0.09	1.974	1.452	5.055	1.448	6	8639	ZINC000003861736
0.2	-8.6	-8.2	0.20	2.584	0.416	5.371	1.188	6	8640	ZINC000000404256
0.2	-7.3	-6.8	0.38	2.318	0.230	4.668	1.476	6	8641	ZINC000015261461
0.2	-9.1	-8.7	0.26	1.585	0.584	3.531	0.598	6	8642	ZINC000100783439
0.2	-5.1	-4.9	0.19	2.596	0.678	3.225	0.926	6	8643	ZINC000000164545
0.2	-6.9	-6.6	0.23	2.822	0.252	3.683	0.439	6	8644	ZINC000000039811
0.2	-6.1	-5.8	0.21	1.853	0.488	3.525	0.199	6	8645	ZINC000002566471
0.2	-9.3	-7.6	1.05	1.908	0.217	4.310	1.125	5	8646	ZINC000033831853
0.2	-5.2	-5.1	0.11	2.337	1.115	3.938	0.157	6	8647	ZINC000004262098
0.2	-5.5	-5.5	0.05	2.195	0.450	3.250	0.850	6	8648	ZINC000008602481

0.2	-8.8	-8.3	0.33	1.950	0.247	5.730	0.750	6	8649	ZINC000257514616
0.2	-7.8	-7.5	0.23	1.800	0.816	3.241	1.121	6	8650	ZINC000000066039
0.2	-6.2	-6.1	0.09	2.568	0.464	4.240	0.736	6	8651	ZINC000001575554
0.2	-8.4	-7.8	0.43	1.435	0.239	2.893	0.736	6	8652	ZINC000015046792
0.2	-8.7	-7.3	0.70	2.047	0.663	6.038	1.828	6	8653	ZINC000013546348
0.2	-10.3	-9.7	0.32	1.799	0.265	4.172	2.080	6	8654	ZINC000257467682
0.2	-8.7	-8.4	0.19	1.807	0.522	4.349	0.665	6	8655	ZINC000006523722
0.2	-9.2	-7.6	1.20	1.121	0.140	1.685	0.555	3	8656	ZINC000033503586
0.2	-5.8	-5.5	0.17	3.158	1.258	3.803	1.421	6	8657	ZINC000001845870
0.2	-8.4	-7.9	0.32	2.229	0.350	5.911	1.337	6	8658	ZINC000100828443
0.2	-5.7	-5.5	0.12	1.925	0.657	3.140	0.536	6	8659	ZINC000003861086
0.2	-6.9	-6.5	0.18	2.613	0.537	5.205	1.535	6	8660	ZINC000040470861
0.2	-6.6	-6.2	0.19	1.470	0.233	2.692	0.203	6	8661	ZINC000002384629
0.2	-7.2	-7.0	0.13	2.330	0.497	3.774	1.323	6	8662	ZINC000000901507
0.2	-5.7	-5.6	0.08	2.108	0.637	3.584	0.705	6	8663	ZINC000001532827

0.2	-6.5	-6.3	0.12	1.973	0.295	2.875	0.730	6	8664	ZINC000002560934
0.2	-4.8	-4.7	0.17	2.199	1.068	3.227	1.121	6	8665	ZINC000001615347
0.2	-7.2	-6.9	0.21	2.429	0.171	3.900	0.899	6	8666	ZINC000000526834
0.2	-5.9	-5.6	0.15	1.804	0.761	2.585	0.645	6	8667	ZINC000000901022
0.2	-5.5	-5.4	0.09	1.627	0.430	3.026	0.802	6	8668	ZINC000000388609
0.2	-9.1	-8.8	0.25	1.518	0.586	3.720	0.649	6	8669	ZINC000100783436
0.2	-5.2	-5.1	0.07	2.464	0.723	3.085	0.845	6	8670	ZINC000013437566
0.2	-6.6	-6.4	0.10	2.664	0.643	4.437	1.256	6	8671	ZINC000001529649
0.2	-9.0	-8.6	0.27	1.556	0.348	3.145	1.019	6	8672	ZINC000014504721
0.2	-9.1	-8.6	0.41	1.844	0.284	4.240	0.799	6	8673	ZINC000014453944
0.2	-7.7	-7.3	0.23	2.088	0.652	3.972	1.212	6	8674	ZINC000015219977
0.2	-6.0	-5.7	0.22	2.181	0.536	3.560	0.995	6	8675	ZINC000001605259
0.2	-4.5	-4.4	0.09	3.649	0.737	4.123	0.366	6	8676	ZINC000000164484
0.2	-7.1	-6.9	0.12	2.428	0.527	4.169	1.147	6	8677	ZINC000005274091
0.2	-7.2	-6.9	0.20	2.182	0.546	3.686	1.359	6	8678	ZINC000000002001

0.2	-7.8	-7.1	0.44	3.554	0.190	6.414	0.270	6	8679	ZINC000004228245
0.2	-4.3	-4.1	0.15	1.465	0.368	2.207	0.348	6	8680	ZINC000100828987
0.2	-8.3	-7.7	0.27	2.072	0.139	4.747	1.146	6	8681	ZINC000059207391
0.2	-9.2	-8.9	0.16	1.673	0.310	4.957	1.305	6	8682	ZINC000014725168
0.2	-7.5	-7.1	0.27	2.192	0.282	4.450	0.500	6	8683	ZINC000014556688
0.2	-8.0	-7.4	0.44	2.006	0.460	5.406	2.015	6	8684	ZINC000005998663
0.2	-8.0	-7.6	0.27	3.042	0.489	4.911	1.208	6	8685	ZINC000013520785
0.2	-6.6	-6.4	0.11	2.156	0.714	3.437	1.049	6	8686	ZINC000000388310
0.2	-7.3	-7.0	0.26	1.994	0.822	3.258	1.466	6	8687	ZINC000000396101
0.2	-8.3	-7.5	0.47	1.943	0.200	5.584	0.751	6	8688	ZINC000100770492
0.2	-6.6	-6.2	0.25	1.541	0.324	3.463	1.079	6	8689	ZINC000015204734
0.2	-6.7	-6.5	0.14	2.976	0.941	4.735	1.465	6	8690	ZINC000095618099
0.2	-8.6	-6.7	1.03	2.207	0.617	5.890	1.827	5	8691	ZINC000014811056
0.2	-9.6	-8.8	0.54	1.619	0.355	4.627	1.197	6	8692	ZINC000100821958
0.2	-8.9	-7.9	0.75	1.807	0.306	4.436	1.337	6	8693	ZINC000034084213

0.2	-8.3	-8.1	0.09	1.716	0.096	5.835	0.167	6	8694	ZINC000015249385
0.2	-9.4	-8.9	0.32	2.196	0.315	4.509	0.808	6	8695	ZINC000015219704
0.2	-5.5	-5.2	0.16	2.570	0.747	3.883	0.966	6	8696	ZINC000000896812
0.2	-6.4	-6.3	0.09	3.599	0.542	5.435	0.564	6	8697	ZINC000005273931
0.2	-5.2	-5.1	0.08	2.239	0.462	2.820	0.766	6	8698	ZINC000001081496
0.2	-6.3	-6.1	0.14	1.932	0.867	3.739	1.125	6	8699	ZINC000000120502
0.2	-7.5	-7.1	0.25	2.352	0.779	4.124	2.203	6	8700	ZINC000015261259
0.2	-7.2	-7.1	0.14	1.820	0.261	3.722	0.885	6	8701	ZINC000001841754
0.2	-7.8	-7.7	0.05	2.182	0.178	3.711	0.264	6	8702	ZINC000257468040
0.2	-9.3	-8.9	0.28	1.555	0.517	3.502	1.590	6	8703	ZINC000257471244
0.2	-5.8	-5.7	0.07	1.341	0.502	3.976	0.893	6	8704	ZINC000100029041
0.2	-5.4	-5.3	0.11	2.119	0.583	2.922	0.700	6	8705	ZINC000001532578
0.2	-6.8	-6.7	0.11	2.852	0.276	4.491	1.400	6	8706	ZINC000095617680
0.2	-6.2	-6.1	0.06	2.486	0.760	3.056	0.990	6	8707	ZINC000000393339
0.2	-6.4	-6.2	0.13	1.914	0.894	2.780	0.808	6	8708	ZINC000001673553

0.2	-6.4	-6.1	0.15	2.023	0.609	2.779	0.886	6	8709	ZINC000004521598
0.2	-7.7	-7.5	0.12	2.254	0.507	3.193	1.232	6	8710	ZINC000095617487
0.2	-7.5	-7.2	0.18	1.846	0.166	5.338	1.377	6	8711	ZINC000014590816
0.2	-8.6	-8.1	0.36	1.568	0.915	4.133	1.416	6	8712	ZINC000014652510
0.2	-8.3	-7.7	0.44	2.823	0.630	4.465	1.553	6	8713	ZINC000004095566
0.2	-6.1	-6.0	0.06	2.632	0.310	5.007	0.281	6	8714	ZINC000006032499
0.2	-5.6	-5.4	0.17	3.250	0.905	4.182	0.538	6	8715	ZINC000001587582
0.2	-9.4	-7.9	1.13	1.988	0.373	3.828	0.970	6	8716	ZINC000238741972
0.2	-6.6	-6.4	0.17	1.411	0.569	3.161	0.170	6	8717	ZINC000100824528
0.2	-9.3	-8.6	0.57	2.201	0.631	4.194	1.372	6	8718	ZINC000013330532
0.2	-5.5	-5.4	0.14	1.198	0.280	3.284	0.836	6	8719	ZINC000008551175
0.2	-4.9	-4.8	0.08	3.142	1.546	4.460	1.356	6	8720	ZINC000004228276
0.2	-5.4	-5.2	0.08	2.924	0.760	4.674	0.601	6	8721	ZINC000028631781
0.2	-6.6	-6.2	0.22	1.916	0.578	3.476	1.112	6	8722	ZINC000015204735
0.2	-5.7	-5.5	0.12	1.917	0.614	3.121	0.508	6	8723	ZINC000001712309

0.2	-7.1	-6.7	0.24	1.877	0.660	3.866	1.323	6	8724	ZINC000085589970
0.2	-4.7	-4.6	0.08	2.412	1.816	3.557	1.116	6	8725	ZINC000001673420
0.2	-8.5	-7.9	0.39	2.073	0.685	4.278	1.261	6	8726	ZINC000014685555
0.2	-6.8	-6.6	0.10	1.933	0.290	4.505	0.867	6	8727	ZINC000012496860
0.2	-6.0	-5.6	0.24	2.543	0.610	3.229	1.205	6	8728	ZINC000001532614
0.2	-6.5	-6.4	0.07	2.290	0.870	3.033	0.802	6	8729	ZINC000001765359
0.2	-3.0	-2.9	0.09	3.334	2.096	3.754	1.919	6	8730	ZINC000001484626
0.2	-7.4	-7.0	0.31	2.094	0.193	5.346	0.732	6	8731	ZINC000040165198
0.2	-6.7	-6.4	0.11	3.052	0.766	4.084	0.298	6	8732	ZINC000095617504
0.2	-9.5	-8.7	0.56	2.071	0.813	3.528	1.830	6	8733	ZINC000004102323
0.2	-6.8	-6.7	0.12	2.782	0.558	4.311	1.335	6	8734	ZINC000005115921
0.2	-7.7	-7.3	0.21	2.361	0.767	4.311	1.303	6	8735	ZINC000015219980
0.2	-6.3	-6.1	0.11	2.124	0.901	3.300	0.344	6	8736	ZINC000257419391
0.2	-6.7	-6.2	0.25	2.337	0.779	4.383	1.426	6	8737	ZINC000001709622
0.2	-8.0	-7.9	0.08	2.368	0.441	4.048	0.818	6	8738	ZINC000005574938

0.2	-7.5	-7.4	0.10	2.288	0.309	4.300	1.830	6	8739	ZINC000040472637
0.2	-5.1	-5.1	0.05	2.018	0.632	2.945	0.628	6	8740	ZINC000004521557
0.2	-7.9	-7.0	0.68	2.198	0.614	3.994	1.546	6	8741	ZINC000013303509
0.2	-7.3	-6.9	0.37	1.872	0.714	2.559	0.726	6	8742	ZINC000001719252
0.2	-6.6	-6.4	0.09	2.027	0.708	3.455	1.393	6	8743	ZINC000002516284
0.2	-4.8	-4.6	0.15	2.522	0.668	3.152	0.851	6	8744	ZINC000000586584
0.2	-8.1	-7.7	0.19	2.044	0.504	3.497	1.402	6	8745	ZINC000014928780
0.2	-6.6	-6.1	0.39	2.584	0.475	4.380	1.834	6	8746	ZINC000001999404
0.2	-6.8	-6.6	0.11	2.553	0.254	3.977	0.314	6	8747	ZINC000000393781
0.2	-8.1	-8.0	0.16	1.964	0.157	3.775	0.685	6	8748	ZINC000005934432
0.2	-9.5	-8.6	0.67	2.528	0.326	4.903	0.585	6	8749	ZINC000238743190
0.2	-8.1	-7.2	0.50	2.030	0.197	5.555	0.409	6	8750	ZINC000004096283
0.2	-5.4	-5.1	0.29	2.852	0.953	3.512	1.099	6	8751	ZINC000095619738
0.2	-6.6	-6.3	0.13	2.131	0.377	2.687	0.529	6	8752	ZINC000035307749
0.2	-10.3	-8.7	1.13	2.179	0.343	4.990	1.274	3	8753	ZINC000003980114

0.2	-6.7	-6.4	0.16	2.826	0.634	4.078	0.814	6	8754	ZINC000005274102
0.2	-8.1	-7.6	0.27	1.759	0.427	3.218	1.012	6	8755	ZINC000002522581
0.2	-6.4	-6.3	0.08	2.266	0.492	3.619	0.650	6	8756	ZINC000001529812
0.2	-9.1	-8.4	0.50	1.851	0.442	3.857	1.313	6	8757	ZINC000257557780
0.2	-8.0	-7.6	0.22	2.387	0.739	4.755	1.518	6	8758	ZINC000003598530
0.2	-5.6	-5.5	0.13	2.175	0.680	3.740	0.569	6	8759	ZINC000001561960
0.2	-8.6	-7.6	0.67	2.042	0.794	4.027	1.998	6	8760	ZINC000034290145
0.2	-9.3	-7.3	1.05	1.916	0.505	3.888	1.354	5	8761	ZINC000015257187
0.2	-7.8	-7.6	0.14	2.081	0.463	3.921	1.765	6	8762	ZINC000006091898
0.2	-8.9	-8.2	0.39	1.364	0.197	4.974	1.889	6	8763	ZINC000014647503
0.2	-6.1	-6.0	0.09	2.459	0.369	3.523	0.913	6	8764	ZINC000015221896
0.2	-7.0	-6.7	0.15	2.765	0.299	4.728	0.950	6	8765	ZINC000001586769
0.2	-9.5	-8.5	0.61	1.535	0.211	4.166	1.652	6	8766	ZINC000100780536
0.2	-10.7	-8.7	1.19	2.014	0.157	5.635	1.466	4	8767	ZINC000100829126
0.2	-8.1	-7.6	0.25	2.250	0.372	3.641	1.287	6	8768	ZINC000003814318

0.2	-7.5	-6.9	0.27	2.234	0.303	3.557	1.513	6	8769	ZINC000095617735
0.2	-9.0	-8.2	0.75	1.986	0.000	4.051	0.000	2	8770	ZINC000100779280
0.2	-9.1	-8.4	0.53	1.684	0.316	4.299	0.516	6	8771	ZINC000257557782
0.2	-6.4	-6.2	0.11	3.075	0.917	4.081	1.382	6	8772	ZINC000001569730
0.2	-8.6	-7.7	0.62	2.363	0.755	3.860	1.422	6	8773	ZINC000008952459
0.2	-6.6	-6.4	0.17	1.480	0.564	3.192	0.174	6	8774	ZINC000100824532
0.2	-9.4	-7.8	1.11	1.718	0.220	5.107	0.081	4	8775	ZINC000006094187
0.2	-5.9	-5.6	0.21	3.609	1.361	4.950	0.444	6	8776	ZINC000086033572
0.2	-8.9	-7.6	0.99	1.848	0.534	3.896	1.568	6	8777	ZINC000100781045
0.2	-6.7	-6.4	0.21	2.905	0.599	4.180	0.993	6	8778	ZINC000005663066
0.2	-7.3	-7.0	0.14	2.399	0.555	4.494	1.757	6	8779	ZINC000033995140
0.2	-5.3	-5.2	0.07	3.119	0.467	4.631	0.767	6	8780	ZINC000001765488
0.2	-9.8	-9.2	0.48	2.210	0.365	3.971	1.036	6	8781	ZINC000053194131
0.2	-5.4	-5.2	0.12	3.392	0.558	4.413	0.804	6	8782	ZINC000014680223
0.2	-7.4	-7.0	0.26	2.994	0.210	5.832	0.371	6	8783	ZINC000001786141

0.2	-8.9	-8.3	0.33	2.617	0.511	4.477	1.369	6	8784	ZINC000013383463
0.2	-7.1	-6.9	0.09	2.380	0.625	4.991	0.995	6	8785	ZINC000008860472
0.2	-9.0	-8.1	0.57	1.877	0.358	5.532	1.734	6	8786	ZINC000015058564
0.2	-6.5	-6.2	0.26	2.059	0.957	4.134	1.210	6	8787	ZINC000001684877
0.2	-9.5	-8.4	0.71	1.527	0.127	5.332	0.933	6	8788	ZINC000085592952
0.2	-8.1	-7.3	0.50	2.892	0.783	4.929	2.066	6	8789	ZINC000000099385
0.2	-7.1	-6.9	0.10	2.034	0.349	2.640	0.486	6	8790	ZINC000001532221
0.2	-8.2	-7.8	0.21	1.895	0.386	4.736	1.542	6	8791	ZINC000040165446
0.2	-7.8	-7.6	0.13	2.685	0.145	5.392	1.121	6	8792	ZINC000001605476
0.2	-5.9	-5.7	0.13	2.161	0.175	2.652	0.484	6	8793	ZINC000095620432
0.2	-7.1	-6.9	0.17	2.106	0.273	3.460	1.051	6	8794	ZINC000005618626
0.2	-9.7	-8.3	1.02	1.815	0.229	4.508	1.664	5	8795	ZINC000013302607
0.2	-5.5	-5.3	0.14	2.676	1.281	4.232	1.046	6	8796	ZINC000013551392
0.2	-5.9	-5.8	0.15	1.635	0.743	3.766	0.520	6	8797	ZINC000001677789
0.2	-6.4	-6.1	0.19	2.968	1.032	4.388	1.887	6	8798	ZINC000013357397

0.2	-5.7	-5.5	0.14	1.896	0.650	3.116	0.551	6	8799	ZINC000001712310
0.2	-9.1	-8.5	0.61	1.892	0.420	5.722	2.116	6	8800	ZINC000014776041
0.2	-5.6	-5.3	0.15	2.363	0.904	3.403	1.480	6	8801	ZINC000002579066
0.2	-6.9	-6.6	0.23	2.048	0.246	4.213	1.281	6	8802	ZINC000013541531
0.2	-8.4	-7.8	0.50	1.751	0.267	4.493	1.341	6	8803	ZINC000257396873
0.2	-6.7	-6.4	0.17	1.686	0.312	3.176	1.316	6	8804	ZINC000212539314
0.2	-6.6	-6.4	0.08	2.594	0.193	3.569	0.198	6	8805	ZINC000002037837
0.2	-6.5	-6.2	0.21	2.182	0.335	4.986	1.474	6	8806	ZINC000004683166
0.2	-6.8	-6.6	0.12	2.420	0.198	3.924	0.684	6	8807	ZINC000013411767
0.2	-9.1	-8.4	0.53	2.049	0.339	4.186	0.486	6	8808	ZINC000086001536
0.2	-6.4	-6.3	0.08	1.937	0.346	3.608	0.454	6	8809	ZINC000001577443
0.2	-6.3	-6.0	0.16	2.150	0.403	2.891	0.581	6	8810	ZINC000015120792
0.2	-6.8	-6.5	0.19	1.997	0.155	2.892	0.432	6	8811	ZINC000015261374
0.2	-5.7	-5.6	0.08	3.353	0.888	5.494	0.826	6	8812	ZINC000013374813
0.2	-7.8	-7.6	0.12	2.582	0.320	5.084	0.923	6	8813	ZINC000002384776

0.2	-5.2	-5.1	0.07	2.611	0.576	3.215	0.686	6	8814	ZINC000002011524
0.2	-5.9	-5.8	0.10	2.879	0.444	4.973	1.219	6	8815	ZINC000005113207
0.2	-7.4	-7.2	0.12	2.248	0.583	4.071	1.465	6	8816	ZINC000033995505
0.2	-4.3	-4.1	0.13	1.760	0.096	3.040	0.351	6	8817	ZINC000001849913
0.2	-4.4	-4.2	0.15	1.495	0.873	3.227	0.635	6	8818	ZINC000001691984
0.2	-9.9	-9.2	0.42	1.252	0.218	3.590	1.282	6	8819	ZINC000014586892
0.2	-8.7	-8.3	0.20	1.957	0.697	5.530	0.658	6	8820	ZINC000001562166
0.2	-9.5	-8.0	0.84	1.797	0.477	3.990	1.892	6	8821	ZINC000100783992
0.2	-9.4	-8.5	0.83	1.615	0.302	5.022	1.486	6	8822	ZINC000033985293
0.2	-8.8	-8.4	0.22	1.085	0.331	4.950	1.909	6	8823	ZINC000257693617
0.2	-6.8	-6.6	0.16	2.890	0.224	4.391	0.661	6	8824	ZINC000002598081
0.2	-7.6	-7.2	0.34	1.959	0.679	3.385	1.908	6	8825	ZINC000030725535
0.2	-8.7	-8.5	0.13	2.329	0.808	3.656	0.630	6	8826	ZINC000004705638
0.2	-4.8	-4.7	0.09	2.378	0.667	3.547	0.851	6	8827	ZINC000002522666
0.2	-6.1	-5.7	0.23	2.921	0.383	3.832	0.393	6	8828	ZINC000001532525

0.2	-4.6	-4.5	0.09	3.753	1.983	4.976	1.765	6	8829	ZINC000000388081
0.2	-4.9	-4.8	0.07	1.825	0.403	2.526	0.410	6	8830	ZINC000038192546
0.2	-6.1	-5.9	0.15	2.808	0.579	4.436	0.744	6	8831	ZINC000004523462
0.2	-5.2	-5.0	0.15	2.073	0.450	2.710	0.453	6	8832	ZINC000001529613
0.2	-8.8	-8.5	0.15	1.159	0.402	4.102	2.012	6	8833	ZINC000257693616
0.2	-6.6	-6.3	0.21	1.885	0.436	4.395	0.673	6	8834	ZINC000015121892
0.2	-5.9	-5.9	0.05	2.568	0.202	3.409	0.466	6	8835	ZINC000000112611
0.2	-9.6	-8.5	0.75	1.681	0.375	4.884	1.435	6	8836	ZINC000100821959
0.2	-8.8	-8.2	0.29	1.811	0.187	3.896	1.488	6	8837	ZINC000257568397
0.2	-5.4	-5.1	0.21	2.471	0.456	3.297	0.825	6	8838	ZINC000001532526
0.2	-8.4	-7.9	0.29	1.987	0.252	5.001	1.039	6	8839	ZINC000032296185
0.2	-7.5	-7.2	0.16	2.967	0.354	5.221	1.354	6	8840	ZINC000004095788
0.2	-9.0	-8.7	0.26	2.045	0.195	5.531	1.092	6	8841	ZINC000006521580
0.2	-9.2	-8.9	0.18	1.902	0.138	6.435	0.210	6	8842	ZINC000013523633
0.2	-6.8	-6.6	0.11	1.860	0.207	4.137	0.713	6	8843	ZINC000095620625

0.2	-6.3	-6.1	0.10	3.306	0.529	4.269	0.902	6	8844	ZINC000095617724
0.2	-3.0	-2.9	0.07	1.949	0.877	2.630	0.967	6	8845	ZINC000001529401
0.2	-6.6	-6.1	0.35	2.012	0.453	3.829	0.806	6	8846	ZINC000002564433
0.2	-5.2	-5.0	0.10	2.176	0.395	2.903	0.416	6	8847	ZINC000002035722
0.2	-6.5	-6.4	0.06	5.126	0.898	6.409	0.426	6	8848	ZINC000005116778
0.2	-5.4	-5.1	0.16	2.540	1.141	3.395	0.983	6	8849	ZINC000001561741
0.2	-6.5	-6.2	0.18	3.183	0.651	4.344	0.654	6	8850	ZINC000000057623
0.2	-6.9	-6.5	0.26	2.613	0.692	3.986	0.639	6	8851	ZINC000100770538
0.2	-7.0	-6.9	0.15	2.516	0.461	5.880	1.842	6	8852	ZINC000254081315
0.2	-5.9	-5.8	0.08	1.907	0.948	5.591	0.682	6	8853	ZINC000001729064
0.2	-5.3	-5.2	0.09	2.827	0.190	3.802	0.552	6	8854	ZINC000005831820
0.2	-6.7	-6.4	0.15	1.860	0.148	3.022	1.199	6	8855	ZINC000069485775
0.2	-4.9	-4.8	0.13	3.038	0.567	3.776	0.396	6	8856	ZINC000000895045
0.2	-6.2	-5.9	0.21	1.581	0.250	3.924	0.749	6	8857	ZINC000014588501
0.2	-6.0	-5.8	0.12	2.033	0.798	3.448	0.740	6	8858	ZINC000000164504

0.2	-5.4	-5.2	0.15	2.330	0.426	3.238	0.530	6	8859	ZINC000012358842
0.2	-7.5	-7.4	0.09	2.464	0.519	4.255	0.974	6	8860	ZINC000085798754
0.2	-6.0	-5.9	0.07	2.009	0.754	3.259	0.853	6	8861	ZINC000001641024
0.2	-6.6	-6.3	0.21	2.343	0.563	4.571	1.533	6	8862	ZINC000005274096
0.2	-5.9	-5.7	0.13	3.077	0.660	4.336	0.702	6	8863	ZINC000014446138
0.2	-9.9	-9.2	0.57	1.693	0.295	3.489	0.741	6	8864	ZINC000100051419
0.2	-7.3	-7.1	0.13	2.399	0.405	4.559	0.613	6	8865	ZINC000095619743
0.2	-7.3	-7.1	0.16	1.826	0.444	3.257	0.801	6	8866	ZINC000095620546
0.2	-6.6	-6.4	0.13	2.421	0.117	4.688	0.517	6	8867	ZINC000004533727
0.2	-7.5	-7.4	0.15	1.819	0.187	2.922	0.881	6	8868	ZINC000095617686
0.2	-9.5	-8.5	0.66	1.358	0.210	5.247	0.898	6	8869	ZINC000257391290
0.2	-6.9	-6.7	0.09	2.756	0.716	4.320	1.187	6	8870	ZINC000000034158
0.2	-6.7	-6.4	0.21	2.253	0.464	5.069	0.337	6	8871	ZINC000001731772
0.2	-9.5	-8.7	0.85	1.576	0.000	2.942	0.000	2	8872	ZINC000095620833
0.2	-5.6	-5.5	0.07	2.062	0.379	2.974	0.269	6	8873	ZINC000002004617

0.2	-8.4	-7.5	0.54	2.446	0.405	4.761	1.615	6	8874	ZINC000013414519
0.2	-5.9	-5.6	0.17	1.464	0.250	3.787	0.721	6	8875	ZINC000001849951
0.2	-6.2	-5.8	0.18	2.173	0.603	3.790	1.715	6	8876	ZINC000002020107
0.2	-9.6	-8.6	0.60	1.848	0.222	5.130	1.574	6	8877	ZINC000015216418
0.2	-6.0	-5.8	0.12	2.488	0.976	4.095	1.057	6	8878	ZINC000000157145
0.2	-5.1	-4.9	0.12	1.769	0.325	2.635	0.235	6	8879	ZINC000040454340
0.2	-6.8	-6.7	0.11	1.757	1.060	3.242	1.264	6	8880	ZINC000257493862
0.2	-9.5	-8.9	0.60	1.473	0.167	4.540	1.987	6	8881	ZINC000257438146
0.2	-9.8	-8.1	0.80	1.536	0.252	4.828	1.591	6	8882	ZINC000100771750
0.2	-9.3	-9.0	0.20	1.669	0.175	4.828	2.352	6	8883	ZINC000000388661
0.2	-5.2	-5.1	0.09	3.353	1.693	3.991	1.860	6	8884	ZINC000000164488
0.2	-6.6	-6.1	0.30	2.871	0.641	3.645	0.797	6	8885	ZINC000000164777
0.2	-6.8	-6.7	0.08	1.968	0.384	2.998	1.209	6	8886	ZINC000001575281
0.2	-6.4	-6.1	0.16	2.911	0.345	4.887	0.321	6	8887	ZINC000050026810
0.2	-7.5	-7.5	0.04	2.033	0.176	3.863	0.761	6	8888	ZINC000015253260

0.2	-5.7	-5.4	0.21	2.759	0.799	4.146	1.059	6	8889	ZINC000001593212
0.2	-7.0	-6.7	0.18	1.723	0.217	2.297	0.433	6	8890	ZINC000004556679
0.2	-5.2	-5.0	0.09	3.071	0.812	4.296	1.013	6	8891	ZINC000001529732
0.2	-6.6	-6.4	0.13	1.617	0.358	3.825	1.507	6	8892	ZINC000002563258
0.2	-6.0	-5.6	0.28	1.868	0.451	3.311	1.117	6	8893	ZINC000005202740
0.2	-6.5	-6.2	0.17	2.314	0.339	3.388	0.350	6	8894	ZINC000000391877
0.2	-7.3	-7.0	0.20	1.783	0.207	3.297	0.565	6	8895	ZINC000001586773
0.2	-7.5	-7.1	0.30	2.843	0.507	3.937	0.738	6	8896	ZINC000013379088
0.2	-8.2	-8.0	0.21	1.988	0.316	3.124	1.025	6	8897	ZINC000015255000
0.2	-6.6	-6.3	0.18	2.283	0.402	3.388	0.836	6	8898	ZINC000001848352
0.2	-9.7	-8.7	0.57	1.780	0.272	4.593	1.544	6	8899	ZINC000066123260
0.2	-8.4	-8.0	0.31	1.604	0.194	3.624	1.021	6	8900	ZINC000100775373
0.2	-4.8	-4.7	0.09	1.756	0.302	3.373	0.555	6	8901	ZINC000096014346
0.2	-4.3	-4.1	0.11	3.184	1.057	3.959	0.675	6	8902	ZINC000005227198
0.2	-9.5	-8.5	0.66	1.442	0.323	5.301	0.887	6	8903	ZINC000100782433

0.2	-9.4	-9.0	0.25	2.060	0.533	5.226	1.231	6	8904	ZINC000006070245
0.2	-6.1	-5.9	0.12	1.881	0.330	3.570	0.997	6	8905	ZINC000032166966
0.2	-9.3	-8.7	0.52	2.064	0.842	4.540	1.794	6	8906	ZINC000253389932
0.2	-7.2	-7.1	0.10	2.206	0.294	3.592	1.453	6	8907	ZINC000095617721
0.2	-10.3	-9.7	0.32	1.759	0.343	4.160	2.099	6	8908	ZINC000100256085
0.2	-7.1	-6.8	0.17	2.420	0.968	3.799	1.404	6	8909	ZINC000001506908
0.2	-11.2	-10.5	0.70	1.631	0.000	5.636	0.000	2	8910	ZINC000013376906
0.2	-8.2	-7.8	0.28	2.995	0.613	4.676	0.370	6	8911	ZINC000002384846
0.2	-8.4	-7.5	0.72	1.818	0.352	5.641	1.798	6	8912	ZINC000013783221
0.2	-8.4	-7.3	0.92	1.884	0.431	3.984	1.517	5	8913	ZINC000086021684
0.2	-10.2	-8.9	0.63	2.274	0.797	4.851	1.721	6	8914	ZINC000002019694
0.2	-7.3	-7.0	0.16	1.864	0.516	4.647	1.134	6	8915	ZINC000005922016
0.2	-5.2	-5.0	0.14	3.240	1.322	4.364	1.577	6	8916	ZINC000001529908
0.2	-7.5	-7.2	0.20	2.347	0.384	4.473	0.697	6	8917	ZINC000085876752
0.2	-8.2	-8.1	0.16	1.366	0.889	4.360	1.702	6	8918	ZINC000000236100

0.2	-6.3	-6.1	0.15	2.593	0.431	4.045	0.601	6	8919	ZINC000000897449
0.2	-5.6	-5.4	0.17	1.926	0.465	3.203	0.863	6	8920	ZINC000000395098
0.2	-6.1	-6.0	0.07	3.993	0.121	6.158	0.465	6	8921	ZINC000005273938
0.2	-6.3	-5.9	0.20	2.514	0.805	4.709	2.159	6	8922	ZINC000013433788
0.2	-7.1	-6.9	0.11	2.599	0.831	4.237	0.480	6	8923	ZINC000100043983
0.2	-7.3	-7.1	0.09	2.270	0.109	4.086	0.867	6	8924	ZINC000002037372
0.2	-5.4	-5.3	0.09	2.372	0.685	3.448	0.725	6	8925	ZINC000002038539
0.2	-6.6	-6.2	0.27	2.083	0.530	3.837	0.800	6	8926	ZINC000002585420
0.2	-4.6	-4.4	0.19	1.991	0.338	3.037	0.720	6	8927	ZINC000002031573
0.2	-5.6	-5.4	0.13	1.657	0.381	3.430	0.621	6	8928	ZINC000014616888
0.2	-6.3	-6.2	0.07	2.930	0.298	4.542	0.815	6	8929	ZINC000033841554
0.2	-7.8	-7.6	0.13	1.562	0.072	5.185	1.725	6	8930	ZINC000261499679
0.2	-8.0	-7.3	0.37	2.132	0.697	4.050	1.439	6	8931	ZINC000003861741
0.2	-6.2	-6.0	0.15	1.318	0.449	4.414	0.685	6	8932	ZINC000085972327
0.2	-5.2	-4.9	0.21	2.551	0.992	3.167	1.254	6	8933	ZINC000001583154

0.2	-5.2	-5.0	0.09	2.301	0.679	3.855	0.650	6	8934	ZINC000003870185
0.2	-7.8	-7.6	0.15	2.777	0.828	3.922	1.078	6	8935	ZINC000040470838
0.2	-7.8	-7.6	0.28	1.893	0.951	4.182	1.618	6	8936	ZINC000004762525
0.2	-8.7	-8.2	0.27	1.873	0.407	3.971	2.025	6	8937	ZINC000013334899
0.2	-7.7	-7.3	0.24	2.044	0.656	3.878	1.230	6	8938	ZINC000257544209
0.2	-10.6	-9.6	0.73	2.220	0.161	4.389	0.732	3	8939	ZINC000014618714
0.2	-7.4	-7.0	0.23	3.206	0.136	6.190	0.081	6	8940	ZINC000033995177
0.2	-5.2	-5.1	0.07	1.532	0.730	3.636	1.271	6	8941	ZINC000000388706
0.2	-8.1	-7.4	0.46	2.356	0.806	4.263	1.550	6	8942	ZINC000008215959
0.2	-7.7	-7.4	0.24	1.664	0.385	2.585	0.580	6	8943	ZINC000014455635
0.2	-9.6	-9.2	0.36	1.970	0.179	6.396	0.850	6	8944	ZINC000013340429
0.2	-8.2	-7.9	0.21	1.733	0.441	3.884	1.653	6	8945	ZINC000257517242
0.2	-9.9	-9.3	0.44	1.638	0.513	5.082	2.464	4	8946	ZINC000014689032
0.2	-4.7	-4.5	0.17	2.170	0.660	3.306	0.763	6	8947	ZINC000004528636
0.2	-4.6	-4.2	0.21	3.378	1.831	4.394	1.625	6	8948	ZINC000003593479

0.2	-5.7	-5.5	0.13	2.489	0.469	3.181	0.304	6	8949	ZINC000000895145
0.2	-5.6	-5.5	0.11	2.645	0.432	3.717	0.431	6	8950	ZINC000003650147
0.2	-5.2	-5.0	0.09	2.462	0.750	4.581	0.349	6	8951	ZINC000014584225
0.2	-5.0	-4.9	0.07	5.934	0.739	7.367	0.629	6	8952	ZINC000001532521
0.2	-8.8	-8.5	0.14	2.114	0.191	5.252	0.406	6	8953	ZINC000248317782
0.2	-8.5	-7.8	0.36	2.569	0.438	4.961	0.888	6	8954	ZINC000034263537
0.2	-8.8	-8.2	0.35	1.917	0.208	5.719	0.750	6	8955	ZINC000014590610
0.2	-6.9	-6.6	0.25	1.944	0.301	5.579	1.742	6	8956	ZINC000014618872
0.2	-8.4	-7.8	0.50	1.754	0.271	4.494	1.342	6	8957	ZINC000257396872
0.2	-6.2	-6.1	0.11	1.421	0.747	4.216	1.109	6	8958	ZINC000018042331
0.2	-5.8	-5.6	0.13	2.591	0.806	3.445	1.000	6	8959	ZINC000002384585
0.2	-6.0	-5.8	0.16	1.472	0.802	4.025	1.086	6	8960	ZINC000003876031
0.2	-7.7	-7.3	0.21	1.803	0.241	4.458	1.105	6	8961	ZINC000012494893
0.2	-7.9	-7.5	0.22	2.218	0.489	4.698	1.562	6	8962	ZINC000005662798
0.2	-5.9	-5.6	0.17	1.337	0.231	2.823	0.878	6	8963	ZINC000001684740

0.2	-8.0	-7.2	0.49	2.826	0.958	4.058	1.190	6	8964	ZINC000008215517
0.2	-5.8	-5.7	0.11	2.689	0.992	4.203	0.333	6	8965	ZINC000013533446
0.2	-5.7	-5.4	0.17	2.784	1.023	3.394	1.267	6	8966	ZINC000001605764
0.2	-7.9	-7.7	0.13	2.410	0.503	4.217	1.433	6	8967	ZINC000050026786
0.2	-6.4	-6.2	0.11	2.264	0.195	3.548	0.764	6	8968	ZINC000004787500
0.2	-9.3	-7.9	0.91	1.962	0.470	3.463	1.468	6	8969	ZINC000014762898
0.2	-7.7	-7.3	0.21	1.933	0.510	3.540	1.089	6	8970	ZINC000001529634
0.2	-5.9	-5.8	0.07	1.581	0.598	2.398	0.412	6	8971	ZINC000013512210
0.2	-7.5	-7.1	0.19	2.322	0.955	2.992	1.316	6	8972	ZINC000005518118
0.2	-6.3	-6.0	0.14	2.598	0.790	3.177	0.846	6	8973	ZINC000000000558
0.2	-6.5	-6.3	0.10	2.535	0.611	3.699	1.426	6	8974	ZINC000015261364
0.2	-8.4	-8.0	0.19	2.084	0.323	4.407	1.817	6	8975	ZINC000261499674
0.2	-8.6	-7.9	0.38	1.709	0.232	5.366	0.786	6	8976	ZINC000014587266
0.2	-8.0	-7.3	0.43	2.393	0.416	4.427	1.545	6	8977	ZINC000050027343
0.2	-5.8	-5.7	0.08	2.458	0.773	3.901	0.614	6	8978	ZINC000008576188

0.2	-5.5	-5.4	0.07	2.604	0.623	4.100	1.249	6	8979	ZINC000008584727
0.2	-8.4	-8.0	0.21	2.093	0.319	4.391	1.828	6	8980	ZINC000100828446
0.2	-3.2	-3.1	0.05	1.458	0.742	2.256	0.237	6	8981	ZINC000008437654
0.2	-7.4	-7.1	0.26	2.207	0.579	3.052	1.094	6	8982	ZINC000000389513
0.2	-4.9	-4.7	0.13	1.531	0.265	2.801	0.748	6	8983	ZINC000004261992
0.2	-4.9	-4.7	0.14	2.347	0.559	2.880	0.498	6	8984	ZINC000001532684
0.2	-7.9	-7.4	0.31	1.962	0.600	3.150	1.518	6	8985	ZINC000033994788
0.2	-6.2	-6.0	0.20	1.980	0.382	2.881	1.151	6	8986	ZINC000002047236
0.2	-8.9	-7.6	1.05	2.450	0.848	4.484	0.953	6	8987	ZINC000006066655
0.2	-7.2	-6.9	0.22	2.419	0.373	4.374	0.646	6	8988	ZINC000004096361
0.2	-5.2	-5.0	0.08	2.087	0.671	3.618	0.901	6	8989	ZINC000001529905
0.2	-6.4	-6.2	0.12	2.685	0.237	4.597	0.786	6	8990	ZINC000012493693
0.2	-4.7	-4.5	0.14	3.210	2.875	4.871	2.491	6	8991	ZINC000001532759
0.2	-9.7	-8.4	1.06	1.829	0.264	5.693	0.244	3	8992	ZINC000014421749
0.2	-6.3	-6.2	0.07	2.987	0.471	3.890	0.268	6	8993	ZINC000001708206

0.2	-9.5	-8.3	0.95	1.986	0.579	4.636	1.340	6	8994	ZINC000000402666
0.2	-8.5	-7.8	0.34	2.452	0.603	4.256	1.522	6	8995	ZINC000002047810
0.2	-7.1	-6.9	0.09	1.887	0.648	4.558	0.165	6	8996	ZINC000006070723
0.2	-9.1	-8.3	0.54	1.893	0.431	4.429	0.342	6	8997	ZINC000257557781
0.2	-4.4	-4.2	0.12	5.562	2.725	7.011	2.009	6	8998	ZINC000000895212
0.2	-9.6	-8.8	0.57	1.605	0.345	4.581	1.165	6	8999	ZINC000257564789
0.2	-7.9	-7.6	0.19	1.840	0.095	3.530	0.464	6	9000	ZINC000100822081
0.2	-7.4	-7.1	0.21	2.290	0.827	4.488	1.572	6	9001	ZINC000095620803
0.2	-6.2	-5.9	0.17	2.146	0.515	3.116	0.898	6	9002	ZINC000001637972
0.2	-7.2	-6.8	0.22	2.027	0.333	4.160	1.161	6	9003	ZINC000008860473
0.2	-9.1	-8.4	0.65	1.920	0.233	4.721	1.281	6	9004	ZINC000100780643
0.2	-6.6	-6.4	0.17	1.568	0.621	3.311	0.290	6	9005	ZINC000013375112
0.2	-5.4	-5.2	0.15	2.170	0.582	2.486	0.630	6	9006	ZINC000000388396
0.2	-8.1	-7.8	0.23	2.137	0.490	3.848	1.739	6	9007	ZINC000007998855
0.2	-7.5	-6.8	0.53	2.340	0.329	3.703	0.539	6	9008	ZINC000034126347

0.2	-5.8	-5.7	0.06	1.120	0.256	3.759	1.020	6	9009	ZINC000100029045
0.2	-6.6	-6.5	0.07	2.499	0.703	4.233	1.158	6	9010	ZINC000004029831
0.2	-6.2	-5.9	0.17	2.506	0.879	4.274	1.422	6	9011	ZINC000001888932
0.2	-5.4	-5.3	0.10	1.958	0.279	3.011	0.391	6	9012	ZINC000003860607
0.2	-4.6	-4.5	0.06	3.382	0.680	4.385	0.634	6	9013	ZINC000005998163
0.2	-8.4	-7.8	0.45	1.381	0.325	3.175	0.653	6	9014	ZINC000256116513
0.2	-6.7	-6.6	0.08	3.607	0.310	5.125	0.410	6	9015	ZINC000003869625
0.2	-8.2	-7.8	0.24	1.922	0.253	3.433	1.204	6	9016	ZINC000014682692
0.2	-5.5	-5.4	0.05	2.113	0.344	3.133	0.837	6	9017	ZINC000013818253
0.2	-7.5	-7.3	0.12	2.120	0.294	4.053	1.922	6	9018	ZINC000040472638
0.2	-7.2	-7.0	0.18	1.716	0.145	4.649	1.358	6	9019	ZINC000005442660
0.2	-10.3	-9.7	0.32	1.769	0.304	4.155	2.100	6	9020	ZINC000257467681
0.2	-8.7	-8.4	0.16	2.412	0.837	3.782	1.786	6	9021	ZINC000003641060
0.2	-8.0	-6.5	0.72	3.063	0.475	6.095	1.679	6	9022	ZINC000013744635
0.2	-8.1	-7.6	0.22	2.170	0.600	4.066	1.779	6	9023	ZINC000013403349

0.2	-7.6	-7.4	0.09	2.271	0.420	2.926	0.617	6	9024	ZINC000001589376
0.2	-6.3	-6.0	0.22	1.479	0.927	4.035	0.822	6	9025	ZINC000013533920
0.2	-7.2	-7.0	0.13	2.329	0.213	4.164	0.325	6	9026	ZINC000006186566
0.2	-7.6	-7.1	0.31	1.553	0.801	5.026	1.457	6	9027	ZINC000095619680
0.2	-9.1	-8.1	0.58	2.390	0.409	4.400	1.663	6	9028	ZINC000095617800
0.2	-6.8	-6.6	0.12	2.246	0.778	3.551	0.685	6	9029	ZINC000000152322
0.2	-8.2	-7.7	0.32	2.332	0.316	3.758	0.881	6	9030	ZINC000040472352
0.2	-9.3	-9.0	0.23	1.731	0.383	3.885	1.594	6	9031	ZINC000015169401
0.2	-7.0	-6.8	0.17	1.847	0.338	3.986	1.443	6	9032	ZINC000256109388
0.2	-5.2	-5.0	0.13	2.900	1.258	4.380	0.856	6	9033	ZINC000002037878
0.2	-9.5	-8.5	0.63	1.445	0.320	5.301	0.887	6	9034	ZINC000257391292
0.2	-6.2	-6.0	0.15	3.061	0.916	3.911	1.053	6	9035	ZINC000034800312
0.2	-5.7	-5.6	0.07	1.306	0.255	3.697	0.997	6	9036	ZINC000003869785
0.2	-7.0	-6.8	0.11	2.535	0.680	3.945	0.571	6	9037	ZINC000001657745
0.2	-9.5	-8.6	0.61	1.727	0.106	4.015	0.720	6	9038	ZINC000015112122

0.2	-6.8	-6.5	0.18	2.825	0.430	5.175	1.051	6	9039	ZINC000050027229
0.2	-8.8	-8.5	0.28	1.635	0.610	3.013	1.188	6	9040	ZINC000100826339
0.2	-4.8	-4.6	0.11	2.539	0.623	3.791	0.473	6	9041	ZINC000000403074
0.2	-8.4	-7.1	0.82	2.240	0.351	5.385	2.036	6	9042	ZINC000257567675
0.2	-4.9	-4.9	0.00	1.221	0.543	2.622	0.395	6	9043	ZINC000006119222
0.2	-8.2	-7.9	0.14	1.771	0.552	4.523	1.185	6	9044	ZINC000015120291
0.2	-5.7	-5.5	0.11	2.759	0.682	4.419	0.987	6	9045	ZINC000003870028
0.2	-8.9	-8.4	0.30	0.993	0.462	3.510	0.618	6	9046	ZINC000261497140
0.2	-8.2	-7.7	0.34	3.051	0.739	6.168	1.892	6	9047	ZINC000001529973
0.2	-5.2	-5.0	0.12	2.820	0.510	4.226	0.340	6	9048	ZINC000001765484
0.2	-8.4	-7.5	0.62	1.783	0.472	4.417	1.669	6	9049	ZINC000013481363
0.2	-4.4	-4.2	0.08	3.053	1.117	4.180	0.562	6	9050	ZINC000004720638
0.2	-7.1	-7.0	0.07	1.636	0.734	3.716	0.924	6	9051	ZINC000006004394
0.2	-9.5	-8.3	0.56	1.798	0.368	4.518	1.612	6	9052	ZINC000085589229
0.2	-9.1	-8.8	0.25	1.505	0.586	3.710	0.645	6	9053	ZINC000100783435

0.2	-7.1	-6.6	0.26	2.076	0.344	3.516	0.226	6	9054	ZINC000001677791
0.2	-8.8	-8.5	0.20	1.668	0.591	3.030	1.184	6	9055	ZINC000257730029
0.2	-3.8	-3.5	0.19	1.692	0.654	2.504	0.332	6	9056	ZINC000005736045
0.2	-7.0	-6.7	0.17	1.643	0.301	3.543	0.831	6	9057	ZINC000005820127
0.2	-6.1	-5.9	0.13	2.677	0.476	4.005	0.605	6	9058	ZINC000003014483
0.2	-6.6	-6.3	0.19	2.096	0.255	3.255	0.252	6	9059	ZINC000000388080
0.2	-6.4	-6.3	0.07	2.864	0.657	4.272	0.792	6	9060	ZINC000018188714
0.2	-6.8	-6.7	0.08	2.015	1.111	3.048	0.767	6	9061	ZINC000097056130
0.2	-5.8	-5.5	0.17	2.762	1.010	3.992	0.699	6	9062	ZINC000005116994
0.2	-6.3	-5.8	0.35	2.343	0.375	4.224	0.889	6	9063	ZINC000004899432
0.2	-6.9	-6.5	0.25	1.731	0.433	2.740	0.407	6	9064	ZINC000000895800
0.2	-6.5	-6.4	0.09	2.304	0.317	4.065	0.619	6	9065	ZINC000003869621
0.2	-6.0	-5.8	0.13	4.997	0.328	6.656	0.337	6	9066	ZINC000100006772
0.2	-8.6	-8.3	0.19	2.843	0.569	4.736	1.205	6	9067	ZINC000050027349
0.2	-6.2	-6.0	0.11	3.087	0.673	3.829	0.802	6	9068	ZINC000000388067

0.2	-6.1	-6.0	0.09	2.418	0.559	3.236	0.692	6	9069	ZINC000000332752
0.2	-7.7	-7.0	0.51	1.790	0.441	4.832	0.479	6	9070	ZINC000014828300
0.2	-7.3	-7.2	0.06	2.181	0.442	4.119	1.322	6	9071	ZINC000005180993
0.2	-6.8	-6.7	0.15	2.219	0.305	3.975	1.183	6	9072	ZINC000069485820
0.2	-6.4	-6.2	0.09	2.867	0.462	4.550	1.354	6	9073	ZINC000040439667
0.2	-5.2	-5.0	0.11	2.822	1.239	4.514	1.083	6	9074	ZINC000005131896
0.2	-5.9	-5.7	0.12	1.924	0.192	3.316	0.543	6	9075	ZINC000001532649
0.2	-9.1	-7.7	0.70	2.821	0.519	4.633	1.171	6	9076	ZINC000013480429
0.2	-7.1	-6.8	0.17	1.878	0.082	3.814	1.837	6	9077	ZINC000036456743
0.2	-4.4	-4.1	0.16	2.040	0.693	3.301	0.501	6	9078	ZINC000001849914
0.2	-8.4	-8.0	0.23	2.081	0.323	5.054	1.801	6	9079	ZINC000261499675
0.2	-7.2	-6.9	0.20	2.199	0.850	4.091	1.745	6	9080	ZINC000004556702
0.2	-7.6	-7.4	0.16	2.002	0.874	2.777	1.172	6	9081	ZINC000095620533
0.2	-7.0	-6.8	0.14	2.227	0.491	3.628	1.657	6	9082	ZINC000095617771
0.2	-9.2	-8.8	0.29	2.657	0.049	5.613	0.726	6	9083	ZINC000095620793

0.2	-7.6	-7.2	0.31	2.134	0.513	4.230	1.104	6	9084	ZINC000012153042
0.2	-6.8	-6.7	0.12	2.203	0.489	4.133	0.529	6	9085	ZINC000001849664
0.2	-5.2	-5.0	0.08	2.403	0.829	3.592	0.927	6	9086	ZINC000001530348
0.2	-8.7	-8.2	0.25	2.946	0.766	4.842	1.016	6	9087	ZINC000000037830
0.2	-8.2	-8.1	0.09	1.556	0.610	3.414	0.891	6	9088	ZINC000015249514
0.2	-5.7	-5.3	0.21	2.678	0.375	3.205	0.347	6	9089	ZINC000001605762
0.2	-5.2	-4.8	0.29	2.960	0.764	3.618	0.884	6	9090	ZINC000015270318
0.2	-8.5	-8.0	0.42	1.977	0.275	3.606	1.065	6	9091	ZINC000100781059
0.2	-6.8	-6.4	0.25	2.853	0.557	4.224	0.499	6	9092	ZINC000000155973
0.2	-6.8	-6.6	0.12	2.165	0.524	3.457	1.093	6	9093	ZINC000002384843
0.2	-8.9	-8.5	0.22	1.705	0.444	4.557	2.074	6	9094	ZINC000238753974
0.2	-5.7	-5.5	0.14	3.339	0.894	4.259	1.084	6	9095	ZINC000100053681
0.2	-6.6	-6.3	0.21	3.025	0.331	4.929	1.550	6	9096	ZINC000002384936
0.2	-8.8	-8.5	0.17	1.258	0.545	4.405	1.801	6	9097	ZINC000100772446
0.2	-9.3	-8.5	0.75	1.658	0.255	4.142	1.129	6	9098	ZINC000238766352

0.2	-7.8	-7.4	0.27	2.164	0.835	2.994	1.400	6	9099	ZINC000033994790
0.2	-8.4	-8.0	0.18	1.880	0.273	4.596	1.640	6	9100	ZINC000014679854
0.2	-9.0	-8.4	0.28	1.451	0.302	4.743	0.656	6	9101	ZINC000014725164
0.2	-10.3	-8.8	1.04	1.774	0.402	5.121	1.289	6	9102	ZINC000014715672
0.2	-8.2	-7.8	0.27	1.518	0.324	3.784	1.574	6	9103	ZINC000100298302
0.2	-5.8	-5.6	0.16	2.258	0.250	3.343	0.788	6	9104	ZINC000085589703
0.2	-4.6	-4.5	0.11	1.542	0.517	2.780	0.500	6	9105	ZINC000002037726
0.2	-6.6	-6.2	0.19	1.752	0.550	3.818	0.982	6	9106	ZINC000004726903
0.2	-6.3	-6.1	0.11	2.131	0.897	3.304	0.341	6	9107	ZINC000012153501
0.2	-6.0	-5.9	0.09	2.427	0.729	4.672	0.775	6	9108	ZINC000001665919
0.2	-9.1	-8.7	0.32	1.758	0.203	4.754	1.388	6	9109	ZINC000100824314
0.2	-5.3	-5.1	0.12	1.766	0.920	2.641	0.511	6	9110	ZINC000017886255
0.2	-8.3	-7.0	0.74	2.167	0.328	4.380	1.269	6	9111	ZINC000014810544
0.2	-7.0	-6.8	0.12	2.093	0.400	4.861	0.524	6	9112	ZINC000014454644
0.2	-9.0	-8.6	0.29	1.901	0.331	5.092	0.980	6	9113	ZINC000036177510

0.2	-6.5	-6.2	0.23	2.171	1.158	3.186	1.060	6	9114	ZINC000002034882
0.2	-5.9	-5.6	0.15	2.731	0.740	4.301	0.826	6	9115	ZINC000003869886
0.2	-4.4	-4.3	0.07	2.369	1.278	3.139	0.907	6	9116	ZINC000003791092
0.2	-8.4	-8.0	0.25	1.677	0.212	4.691	1.458	6	9117	ZINC000257377006
0.2	-9.6	-8.8	0.71	1.648	0.554	3.412	1.585	4	9118	ZINC000014688356
0.2	-4.5	-4.4	0.09	1.989	0.496	3.592	0.993	6	9119	ZINC000001555566
0.2	-6.8	-6.5	0.19	1.715	0.433	4.372	0.813	6	9120	ZINC000015121889
0.2	-6.2	-5.9	0.19	1.928	0.546	2.693	1.084	6	9121	ZINC000028631761
0.2	-8.1	-7.7	0.20	2.459	0.570	4.509	1.416	6	9122	ZINC000004095572
0.2	-7.6	-7.4	0.16	2.854	0.788	4.839	1.762	6	9123	ZINC000028631687
0.2	-7.1	-6.9	0.09	3.447	0.484	5.493	0.205	6	9124	ZINC000001532514
0.2	-7.1	-6.9	0.17	2.719	0.666	4.034	1.141	6	9125	ZINC000002036798
0.2	-6.7	-6.5	0.12	2.847	0.215	4.876	0.966	6	9126	ZINC000005115824
0.2	-9.2	-8.7	0.40	1.828	0.599	3.764	1.523	6	9127	ZINC000100771521
0.2	-6.3	-6.1	0.14	2.207	0.549	3.319	1.391	6	9128	ZINC000001530285

0.2	-7.0	-6.8	0.16	2.091	0.386	5.141	0.327	6	9129	ZINC000000389599
0.2	-8.2	-7.8	0.29	2.434	0.229	4.917	0.737	6	9130	ZINC000014503833
0.2	-7.5	-7.4	0.11	2.570	0.653	3.881	0.621	6	9131	ZINC000002037639
0.2	-7.7	-7.6	0.07	2.448	0.423	4.333	1.377	6	9132	ZINC000018181336
0.2	-6.8	-6.3	0.24	2.394	0.289	3.590	0.410	6	9133	ZINC000000388428
0.2	-7.0	-6.4	0.41	1.816	0.444	3.549	1.051	6	9134	ZINC000001684890
0.2	-8.0	-7.0	0.50	2.555	0.376	4.972	1.558	6	9135	ZINC000004096533
0.2	-6.1	-6.0	0.07	1.736	0.163	3.713	0.527	6	9136	ZINC000004095505
0.2	-6.2	-6.1	0.09	2.308	0.477	3.920	1.326	6	9137	ZINC000034113328
0.2	-8.2	-8.2	0.07	1.866	0.296	4.136	1.478	6	9138	ZINC000027638911
0.2	-9.4	-9.1	0.18	2.311	0.199	5.301	0.425	6	9139	ZINC000085996403
0.2	-7.4	-7.1	0.14	2.988	0.811	4.580	1.428	6	9140	ZINC000000004060
0.2	-10.6	-8.9	1.27	1.815	0.136	4.204	1.385	3	9141	ZINC000100773099
0.2	-7.1	-6.7	0.21	2.637	0.640	4.976	0.538	6	9142	ZINC000014494539
0.2	-6.0	-5.8	0.16	1.626	0.774	3.910	0.573	6	9143	ZINC000013481852

0.2	-8.4	-7.7	0.38	2.298	0.235	4.763	1.225	6	9144	ZINC000001708204
0.2	-9.4	-8.7	0.51	1.755	0.293	5.250	1.441	6	9145	ZINC000014859213
0.2	-8.8	-7.9	0.62	1.638	0.115	3.481	0.534	6	9146	ZINC000012153834
0.2	-6.5	-6.2	0.20	2.085	0.258	5.533	1.258	6	9147	ZINC000255970188
0.2	-7.9	-7.6	0.16	2.008	0.516	2.788	0.623	6	9148	ZINC000003847274
0.2	-9.7	-7.7	1.18	2.180	0.280	5.576	1.279	4	9149	ZINC000256080310
0.2	-8.3	-7.9	0.28	1.758	0.251	2.443	0.316	6	9150	ZINC000013380365
0.2	-6.5	-6.3	0.15	2.363	0.350	4.289	1.600	6	9151	ZINC000050027575
0.2	-9.3	-7.6	0.82	2.120	0.136	4.156	1.647	6	9152	ZINC000014490883
0.2	-8.7	-8.6	0.11	1.621	0.731	3.651	1.511	6	9153	ZINC000100825083
0.2	-6.5	-6.3	0.13	2.471	0.620	4.070	0.285	6	9154	ZINC000000901721
0.2	-9.5	-8.4	0.65	1.358	0.212	5.246	0.898	6	9155	ZINC000100782441
0.2	-8.4	-8.0	0.49	1.718	1.000	3.145	1.532	6	9156	ZINC000257615708
0.2	-6.5	-6.2	0.17	2.307	0.397	3.939	0.808	6	9157	ZINC000002561071
0.2	-6.9	-6.4	0.35	2.105	0.496	4.566	1.903	6	9158	ZINC000014455510

0.2	-8.4	-8.2	0.21	1.949	0.273	5.520	1.554	6	9159	ZINC000014855572
0.2	-8.1	-7.9	0.09	2.303	0.561	3.738	1.572	6	9160	ZINC000017129257
0.2	-7.9	-7.0	0.59	2.383	0.385	4.266	0.944	6	9161	ZINC000002560985
0.2	-9.9	-8.6	1.02	1.918	0.281	4.540	2.027	6	9162	ZINC000014717647
0.2	-5.0	-4.9	0.07	1.954	0.554	2.562	0.421	6	9163	ZINC000001867127
0.2	-6.1	-6.0	0.05	2.371	1.131	4.799	0.357	6	9164	ZINC000095618320
0.2	-6.1	-6.0	0.10	2.217	0.435	3.951	0.166	6	9165	ZINC000006929291
0.2	-4.5	-4.3	0.11	2.374	0.968	2.968	1.248	6	9166	ZINC000003860819
0.2	-6.8	-6.4	0.22	1.816	0.408	3.396	1.899	6	9167	ZINC000004556802
0.2	-5.1	-4.8	0.17	5.057	2.271	5.766	2.134	6	9168	ZINC000003861770
0.2	-4.7	-4.3	0.21	2.359	0.671	3.021	0.698	6	9169	ZINC000000895304
0.2	-6.7	-6.5	0.19	2.393	0.557	3.500	0.936	6	9170	ZINC000005274024
0.2	-9.5	-8.4	0.88	2.488	0.457	4.649	0.906	6	9171	ZINC000003869906
0.2	-6.3	-5.9	0.22	1.991	0.909	4.306	0.341	6	9172	ZINC000038642928
0.2	-5.8	-5.5	0.14	2.679	0.523	4.554	0.668	6	9173	ZINC000001532569

0.2	-5.8	-5.7	0.09	1.707	0.195	3.425	0.723	6	9174	ZINC000100006137
0.2	-6.5	-6.1	0.22	2.372	0.284	4.778	0.944	6	9175	ZINC000001848346
0.2	-7.7	-7.4	0.16	2.040	0.252	3.868	0.978	6	9176	ZINC000014616808
0.2	-7.5	-7.3	0.12	2.599	0.617	3.984	1.316	6	9177	ZINC000006031286
0.2	-5.9	-5.4	0.25	3.509	1.179	4.697	0.557	6	9178	ZINC000013831818
0.2	-6.4	-6.2	0.15	2.954	0.775	4.201	0.859	6	9179	ZINC000100017211
0.2	-3.8	-3.7	0.15	2.570	1.326	3.050	1.389	6	9180	ZINC000004262387
0.2	-8.9	-8.6	0.37	2.124	0.470	3.668	0.943	6	9181	ZINC000014591324
0.2	-5.0	-4.7	0.13	1.882	0.576	2.987	0.773	6	9182	ZINC000001731112
0.2	-8.8	-8.2	0.36	1.921	0.232	5.572	1.627	6	9183	ZINC000014854267
0.2	-8.9	-8.6	0.17	2.162	0.788	4.815	0.311	6	9184	ZINC000014652295
0.2	-7.8	-7.6	0.16	2.194	0.623	4.296	1.252	6	9185	ZINC000005715146
0.2	-9.3	-8.6	0.76	2.402	0.255	4.585	1.921	6	9186	ZINC000005386772
0.2	-5.1	-4.7	0.21	2.789	0.867	4.240	0.919	6	9187	ZINC000003130514
0.2	-5.7	-5.6	0.09	2.273	1.126	3.874	1.589	6	9188	ZINC000002522588

0.2	-5.0	-4.8	0.15	2.145	0.699	2.950	0.795	6	9189	ZINC000000388071
0.2	-6.4	-6.1	0.18	2.942	0.365	4.763	0.673	6	9190	ZINC000004533504
0.2	-7.5	-7.2	0.17	1.613	0.552	2.050	0.404	6	9191	ZINC000040471397
0.2	-6.1	-5.9	0.10	2.934	1.390	4.457	2.109	6	9192	ZINC000002522600
0.2	-7.8	-7.6	0.16	2.280	0.262	3.865	0.409	6	9193	ZINC000257574254
0.2	-7.6	-7.5	0.14	1.773	0.611	4.030	1.178	6	9194	ZINC000014496278
0.2	-6.6	-6.4	0.16	2.376	0.329	3.625	1.334	6	9195	ZINC000015261366
0.2	-8.8	-8.7	0.08	2.718	0.675	4.453	0.858	6	9196	ZINC000100053185
0.2	-6.5	-6.1	0.21	2.116	0.558	2.996	0.849	6	9197	ZINC000002379217
0.2	-8.8	-8.4	0.20	1.346	0.526	5.211	1.882	6	9198	ZINC000100772450
0.2	-6.7	-6.5	0.16	2.247	0.339	5.130	0.792	6	9199	ZINC000032839139
0.2	-6.7	-6.3	0.21	1.882	0.628	3.089	0.476	6	9200	ZINC000000388292
0.2	-9.2	-8.5	0.40	1.486	0.418	4.180	1.537	6	9201	ZINC000005567454
0.2	-7.2	-6.9	0.24	3.547	0.650	5.531	0.386	6	9202	ZINC000002522698
0.2	-7.7	-7.2	0.22	2.135	0.246	4.474	1.645	6	9203	ZINC000014684261

0.2	-9.5	-8.8	0.71	1.310	0.385	3.903	1.957	5	9204	ZINC000006067766
0.2	-7.1	-6.7	0.30	2.321	0.227	3.619	0.709	6	9205	ZINC000000144268
0.2	-6.5	-6.3	0.13	1.837	0.449	3.225	0.486	6	9206	ZINC000257419390
0.2	-6.6	-6.4	0.17	1.579	0.627	3.288	0.287	6	9207	ZINC000257405996
0.2	-6.6	-6.4	0.19	1.655	0.620	3.316	0.354	6	9208	ZINC000100824533
0.2	-5.9	-5.7	0.09	1.903	0.703	3.368	1.399	6	9209	ZINC000038647448
0.2	-7.9	-7.8	0.14	2.380	0.189	4.108	0.540	6	9210	ZINC000001605726
0.2	-6.8	-6.6	0.17	2.617	0.634	4.952	0.526	6	9211	ZINC000001850602
0.2	-7.0	-6.9	0.11	1.921	0.329	3.525	1.483	6	9212	ZINC000085589574
0.2	-8.0	-7.8	0.15	2.282	0.175	6.356	0.280	6	9213	ZINC000001531861
0.2	-9.6	-9.0	0.44	1.618	0.319	3.914	1.647	6	9214	ZINC000257564787
0.2	-5.3	-5.0	0.16	5.034	2.657	5.539	2.724	6	9215	ZINC000082071923
0.2	-5.5	-5.4	0.05	2.365	0.697	3.580	1.419	6	9216	ZINC000002516022
0.2	-6.4	-6.2	0.09	2.736	0.444	5.002	0.338	6	9217	ZINC000050027556
0.2	-7.4	-7.2	0.14	2.497	0.321	4.003	1.146	6	9218	ZINC000034022549

0.2	-6.7	-6.4	0.15	2.424	0.925	3.899	2.060	6	9219	ZINC000001589437
0.2	-6.3	-6.2	0.08	2.379	0.432	4.366	0.471	6	9220	ZINC000012493601
0.2	-8.1	-7.9	0.21	3.141	0.803	4.640	1.554	6	9221	ZINC000255984288
0.2	-6.6	-6.5	0.12	2.170	0.442	3.437	1.028	6	9222	ZINC000013347367
0.2	-5.8	-5.7	0.07	3.212	0.553	4.514	0.666	6	9223	ZINC000013434916
0.2	-9.3	-8.4	0.70	1.732	0.300	2.729	0.855	3	9224	ZINC000014689082
0.2	-7.2	-6.7	0.24	2.204	0.355	5.273	0.914	6	9225	ZINC000034961794
0.2	-5.4	-5.3	0.11	2.508	0.708	3.447	0.815	6	9226	ZINC000035822486
0.2	-8.6	-8.0	0.43	1.792	0.128	4.527	0.268	6	9227	ZINC000257401486
0.2	-6.9	-6.6	0.18	3.815	0.838	4.798	1.351	6	9228	ZINC000095953048
0.2	-8.8	-8.5	0.28	2.158	0.107	5.903	0.915	6	9229	ZINC000014854270
0.2	-10.3	-9.6	0.42	2.003	0.311	4.445	1.823	6	9230	ZINC000257467684
0.2	-9.0	-8.6	0.21	1.543	0.259	4.117	1.961	6	9231	ZINC000013382387
0.2	-8.3	-7.3	0.72	1.624	0.307	4.504	1.441	6	9232	ZINC000013385488
0.2	-5.5	-5.3	0.12	2.680	1.178	4.358	1.103	6	9233	ZINC000005131743

0.2	-5.7	-5.3	0.21	3.510	1.391	4.153	1.496	6	9234	ZINC000000001795
0.2	-7.7	-7.3	0.21	2.240	0.635	4.282	1.270	6	9235	ZINC000015219983
0.2	-6.3	-6.1	0.15	2.466	0.594	4.877	0.587	6	9236	ZINC000001576198
0.2	-8.0	-7.6	0.25	2.389	0.487	5.158	1.486	6	9237	ZINC000000899115
0.2	-5.4	-5.1	0.20	2.278	0.209	3.075	0.254	6	9238	ZINC000015119988
0.2	-5.5	-5.4	0.07	2.879	1.601	4.728	1.514	6	9239	ZINC000002046963
0.2	-6.9	-6.6	0.18	1.776	0.309	3.788	1.144	6	9240	ZINC000002561203
0.2	-9.3	-8.8	0.33	2.569	0.275	5.495	0.499	6	9241	ZINC000085996404
0.2	-8.5	-7.8	0.43	1.562	0.175	4.202	0.763	6	9242	ZINC000100779962
0.2	-8.7	-7.8	0.59	2.661	1.034	4.421	1.685	6	9243	ZINC000004228234
0.2	-10.3	-9.4	0.45	1.639	0.308	4.543	1.256	6	9244	ZINC000100780546
0.2	-6.6	-6.1	0.32	1.849	0.423	4.029	0.844	6	9245	ZINC000005339827
0.2	-6.7	-6.5	0.15	1.556	0.443	3.297	1.036	6	9246	ZINC000005881073
0.2	-5.2	-5.0	0.09	1.987	0.755	3.250	0.931	6	9247	ZINC000003870184
0.2	-8.4	-7.8	0.39	1.434	0.292	2.875	0.691	6	9248	ZINC000013481362

0.2	-4.6	-4.4	0.12	3.399	1.016	4.056	0.958	6	9249	ZINC000001069171
0.2	-9.9	-9.0	0.84	1.917	0.295	3.936	1.438	6	9250	ZINC000014717652
0.2	-7.5	-7.2	0.20	2.588	0.396	4.909	1.187	6	9251	ZINC000001529628
0.2	-8.6	-8.1	0.31	2.425	0.195	4.903	0.299	6	9252	ZINC000001569748
0.2	-9.1	-8.5	0.86	1.821	0.399	4.274	1.304	6	9253	ZINC000096014928
0.2	-8.4	-8.2	0.15	1.783	0.112	5.113	1.421	6	9254	ZINC000013372188
0.2	-7.3	-7.0	0.30	1.891	0.344	2.490	0.199	6	9255	ZINC000002382836
0.2	-6.7	-6.6	0.12	2.933	0.683	5.715	1.221	6	9256	ZINC000029489590
0.2	-4.4	-4.3	0.11	2.623	1.678	3.656	1.683	6	9257	ZINC000000388770
0.2	-6.9	-6.7	0.09	2.049	0.475	4.468	1.090	6	9258	ZINC000005112923
0.2	-7.8	-7.6	0.12	1.952	0.408	4.438	1.685	6	9259	ZINC000003644304
0.2	-6.2	-6.0	0.09	2.337	0.472	4.605	0.862	6	9260	ZINC000040471829
0.2	-5.5	-5.3	0.13	1.976	0.814	3.249	0.728	6	9261	ZINC000005820346
0.2	-7.3	-7.0	0.19	1.714	0.305	3.106	0.746	6	9262	ZINC000014652286
0.2	-7.3	-7.1	0.15	2.566	0.475	4.712	0.693	6	9263	ZINC000095619744

0.2	-7.9	-7.7	0.15	2.397	0.589	4.927	2.030	6	9264	ZINC000100822484
0.2	-6.3	-6.0	0.22	1.298	0.762	4.379	0.417	6	9265	ZINC000006072527
0.2	-6.2	-5.9	0.16	6.118	0.604	7.398	0.354	6	9266	ZINC000015274320
0.2	-7.9	-7.7	0.11	1.636	0.149	3.922	0.847	6	9267	ZINC000100823531
0.2	-8.2	-7.8	0.24	1.677	0.491	3.857	1.655	6	9268	ZINC000015112257
0.2	-6.9	-6.6	0.19	2.132	0.204	3.973	0.844	6	9269	ZINC000004095569
0.2	-3.5	-3.3	0.13	3.509	2.122	4.092	2.128	6	9270	ZINC000001532681
0.2	-6.0	-5.7	0.16	2.175	0.585	3.110	1.028	6	9271	ZINC000050027403
0.2	-6.9	-6.8	0.10	2.889	0.409	5.222	0.595	6	9272	ZINC000083409141
0.2	-5.8	-5.6	0.09	1.760	0.109	3.632	0.625	6	9273	ZINC000000895175
0.2	-3.8	-3.7	0.07	1.815	0.394	2.465	0.740	6	9274	ZINC000000161206
0.2	-6.8	-6.6	0.11	1.900	0.279	3.265	0.712	6	9275	ZINC000001599253
0.2	-6.6	-6.5	0.09	1.309	0.341	3.511	1.043	6	9276	ZINC000001846606
0.2	-5.8	-5.6	0.15	2.861	0.448	3.608	0.671	6	9277	ZINC000000153933
0.2	-10.3	-9.7	0.32	1.768	0.318	4.168	2.084	6	9278	ZINC000257467683

0.2	-4.9	-4.6	0.22	3.787	0.989	4.468	1.051	6	9279	ZINC000000967396
0.2	-7.6	-7.4	0.15	2.376	0.921	3.473	0.763	6	9280	ZINC000000967928
0.2	-4.8	-4.7	0.11	2.222	0.399	3.257	0.530	6	9281	ZINC000001663924
0.2	-5.8	-5.6	0.18	2.107	0.642	3.573	0.418	6	9282	ZINC000000388762
0.2	-7.1	-6.9	0.15	2.137	0.176	4.677	0.669	6	9283	ZINC000002030906
0.2	-8.8	-8.5	0.20	1.657	0.589	3.017	1.178	6	9284	ZINC000100826340
0.2	-8.2	-7.9	0.27	2.267	0.333	2.735	0.575	6	9285	ZINC000013548506
0.2	-8.4	-8.0	0.25	2.072	0.352	4.937	1.953	6	9286	ZINC000100828441
0.2	-9.8	-9.3	0.43	2.179	0.519	4.725	0.959	6	9287	ZINC000095618174
0.2	-7.2	-7.0	0.11	2.710	0.655	4.089	1.260	6	9288	ZINC000100891529
0.2	-6.0	-5.8	0.09	1.975	0.459	3.855	0.403	6	9289	ZINC000033838365
0.2	-8.2	-8.0	0.17	1.683	0.409	3.844	1.235	6	9290	ZINC000015120783
0.2	-9.1	-7.8	0.79	1.763	0.446	4.886	1.463	6	9291	ZINC000015112559
0.2	-5.2	-4.9	0.14	2.162	0.690	3.123	0.904	6	9292	ZINC000013480221
0.2	-7.2	-7.0	0.12	1.990	0.408	3.087	0.666	6	9293	ZINC000001607785

0.2	-5.3	-5.0	0.29	3.003	1.580	3.444	1.672	6	9294	ZINC000001529322
0.2	-5.9	-5.8	0.07	2.353	0.623	3.738	0.446	6	9295	ZINC000000895194
0.2	-7.3	-6.9	0.26	2.270	0.515	4.747	0.853	6	9296	ZINC000000517337
0.2	-4.6	-4.3	0.16	5.702	1.508	6.690	1.456	6	9297	ZINC000008383183
0.2	-9.6	-8.9	0.83	2.131	0.736	4.827	1.499	4	9298	ZINC000004352602
0.2	-6.4	-6.2	0.15	2.193	1.153	2.997	0.933	6	9299	ZINC000000330134
0.2	-9.6	-8.8	0.45	2.121	0.373	5.396	2.108	6	9300	ZINC000100776936
0.2	-6.5	-6.3	0.12	2.164	0.231	3.279	0.774	6	9301	ZINC000004533726
0.2	-9.3	-8.8	0.37	2.080	0.565	4.417	1.477	6	9302	ZINC000013330534
0.2	-5.8	-5.6	0.13	2.187	0.328	3.295	0.574	6	9303	ZINC000000145734
0.2	-7.5	-7.3	0.12	2.606	0.627	4.043	1.297	6	9304	ZINC000002384652
0.2	-5.0	-4.8	0.13	1.736	0.415	3.636	0.823	6	9305	ZINC000001586443
0.2	-8.4	-7.9	0.50	1.753	0.272	4.498	1.339	6	9306	ZINC000015159849
0.2	-5.5	-5.3	0.15	2.360	0.848	3.155	0.739	6	9307	ZINC000033839258
0.2	-5.8	-5.6	0.10	4.333	1.167	5.807	0.882	6	9308	ZINC000003860327

0.2	-7.9	-7.7	0.15	1.953	0.468	4.243	1.008	6	9309	ZINC000100823533
0.2	-4.6	-4.4	0.16	3.092	1.466	4.186	1.447	6	9310	ZINC000100216788
0.2	-7.9	-7.6	0.21	1.219	0.352	5.808	0.460	6	9311	ZINC000014771656
0.2	-8.7	-8.2	0.50	1.315	0.000	2.250	0.000	2	9312	ZINC000014587146
0.2	-6.5	-6.0	0.24	2.767	1.044	3.975	1.765	6	9313	ZINC000002516129
0.2	-6.2	-6.0	0.13	2.404	0.239	3.435	0.638	6	9314	ZINC000002567933
0.2	-6.1	-6.0	0.08	2.489	0.645	4.391	0.685	6	9315	ZINC000002559032
0.2	-5.4	-5.1	0.21	2.459	0.416	3.745	0.989	6	9316	ZINC000001529502
0.2	-5.2	-5.0	0.20	2.178	0.814	2.797	0.863	6	9317	ZINC000015119535
0.2	-8.6	-8.2	0.27	2.159	0.308	4.138	0.308	6	9318	ZINC000257375892
0.2	-8.0	-6.4	0.91	2.196	0.869	3.860	1.320	6	9319	ZINC000006037984
0.2	-8.7	-7.7	0.55	2.476	1.009	3.241	1.596	6	9320	ZINC000000967720
0.2	-9.2	-7.9	0.64	2.974	0.557	4.917	1.147	6	9321	ZINC000006576347
0.2	-9.4	-8.2	0.86	2.579	0.732	5.152	1.410	5	9322	ZINC000005104407
0.2	-6.9	-6.7	0.18	2.355	0.731	3.210	0.874	6	9323	ZINC000000388113

0.2	-3.7	-3.5	0.13	2.563	0.986	3.050	0.993	6	9324	ZINC000004521395
0.2	-8.8	-8.3	0.29	2.003	0.242	5.765	1.676	6	9325	ZINC000257487944
0.2	-9.2	-8.8	0.26	1.874	0.351	4.125	1.328	6	9326	ZINC000065742963
0.2	-6.9	-6.5	0.22	2.804	0.341	4.555	0.801	6	9327	ZINC000004556548
0.2	-7.4	-7.2	0.15	2.487	0.846	3.979	1.124	6	9328	ZINC000082164067
0.2	-9.5	-8.1	0.95	2.117	0.063	4.550	1.697	6	9329	ZINC000014762890
0.2	-8.2	-7.8	0.26	1.794	0.262	4.232	1.434	6	9330	ZINC000005999133
0.2	-7.4	-7.2	0.12	2.713	0.151	5.142	0.308	6	9331	ZINC000013514836
0.1	-7.6	-7.2	0.42	1.252	0.627	4.662	1.974	6	9332	ZINC000001583785
0.1	-5.9	-5.8	0.11	1.437	0.113	3.410	0.678	6	9333	ZINC000001530145
0.1	-9.2	-8.9	0.23	1.824	0.713	4.084	1.180	6	9334	ZINC000014828127
0.1	-5.9	-5.6	0.15	2.305	0.701	3.592	1.043	6	9335	ZINC000001694745
0.1	-6.9	-6.7	0.18	1.651	0.388	4.677	0.431	6	9336	ZINC000015121891
0.1	-4.5	-4.4	0.11	4.019	2.070	4.603	2.112	6	9337	ZINC000000901672
0.1	-5.2	-5.0	0.09	3.129	1.299	4.587	1.382	6	9338	ZINC000001635605

0.1	-6.9	-6.4	0.35	1.593	0.210	4.037	0.503	6	9339	ZINC000001850790
0.1	-9.2	-8.1	0.74	1.492	0.323	3.020	0.794	6	9340	ZINC000257590901
0.1	-8.8	-8.3	0.26	1.656	0.256	5.658	1.781	6	9341	ZINC000257627782
0.1	-6.8	-6.7	0.11	2.134	0.446	4.705	1.863	6	9342	ZINC000013507521
0.1	-6.4	-6.3	0.07	2.220	0.266	4.207	0.567	6	9343	ZINC000004899618
0.1	-8.2	-7.7	0.23	2.031	0.236	3.766	1.376	6	9344	ZINC000006070276
0.1	-9.7	-8.4	0.76	2.176	0.528	5.345	1.228	6	9345	ZINC000100777985
0.1	-4.2	-4.1	0.07	3.210	1.127	3.627	0.993	6	9346	ZINC000000901420
0.1	-7.1	-7.0	0.07	1.354	0.406	3.513	0.989	6	9347	ZINC000012153498
0.1	-8.2	-8.2	0.00	1.403	0.420	3.994	2.342	6	9348	ZINC000012153639
0.1	-9.5	-8.8	0.39	2.068	0.440	4.722	2.225	6	9349	ZINC000257496644
0.1	-7.3	-7.0	0.23	1.954	0.558	5.232	0.680	6	9350	ZINC000015122329
0.1	-8.7	-8.1	0.50	2.716	0.604	5.634	1.850	6	9351	ZINC000031159899
0.1	-8.1	-7.9	0.13	1.286	0.393	5.086	1.960	6	9352	ZINC000095617985
0.1	-9.7	-8.6	0.65	2.330	0.428	4.133	1.068	6	9353	ZINC000085730718

0.1	-6.7	-6.6	0.09	2.569	0.242	3.993	0.762	6	9354	ZINC000004899443
0.1	-9.3	-8.4	0.43	1.912	0.234	5.300	1.520	6	9355	ZINC000014859212
0.1	-6.1	-5.9	0.09	3.009	0.520	4.334	1.016	6	9356	ZINC000004523463
0.1	-5.2	-5.0	0.08	2.073	0.801	3.494	0.931	6	9357	ZINC000001532519
0.1	-4.0	-3.7	0.13	2.956	0.935	3.547	1.026	6	9358	ZINC000004501380
0.1	-9.7	-8.8	0.63	1.920	0.706	4.332	1.845	6	9359	ZINC000100777989
0.1	-6.8	-6.5	0.16	3.084	0.458	4.603	0.983	6	9360	ZINC000000002234
0.1	-6.9	-6.5	0.24	1.949	0.409	3.653	0.725	6	9361	ZINC000004073375
0.1	-5.0	-4.9	0.10	2.574	0.387	3.140	0.441	6	9362	ZINC000000153666
0.1	-8.7	-8.4	0.20	2.738	0.821	4.402	1.663	6	9363	ZINC000013383462
0.1	-6.5	-6.3	0.20	2.202	0.229	3.598	0.916	6	9364	ZINC000004787494
0.1	-5.8	-5.5	0.14	2.913	0.589	4.362	0.997	6	9365	ZINC000002169919
0.1	-8.7	-7.9	0.47	2.190	0.561	4.186	1.172	6	9366	ZINC000005274083
0.1	-8.4	-8.1	0.27	1.511	0.366	2.758	0.650	6	9367	ZINC000100114364
0.1	-6.9	-6.4	0.32	2.295	0.600	3.293	1.121	6	9368	ZINC000000034161

0.1	-7.9	-7.8	0.09	2.352	0.348	4.737	0.477	6	9369	ZINC000096014435
0.1	-5.5	-5.3	0.13	2.746	0.773	4.156	1.527	6	9370	ZINC000002560465
0.1	-7.4	-7.2	0.13	2.358	0.340	4.076	1.224	6	9371	ZINC000050027126
0.1	-8.8	-7.7	0.58	1.849	0.741	3.156	1.743	6	9372	ZINC000015058665
0.1	-7.7	-7.5	0.14	2.727	0.276	4.705	0.450	6	9373	ZINC000015044703
0.1	-9.8	-9.3	0.33	1.442	0.324	4.153	1.215	6	9374	ZINC000100779658
0.1	-3.7	-3.5	0.12	5.335	2.024	6.184	2.124	6	9375	ZINC000001683467
0.1	-6.5	-6.4	0.07	3.262	1.025	4.369	1.487	6	9376	ZINC000002570173
0.1	-9.8	-8.3	1.02	1.776	0.254	3.697	1.343	6	9377	ZINC000100782622
0.1	-7.1	-6.8	0.16	2.882	0.532	5.295	0.333	6	9378	ZINC000036373791
0.1	-6.4	-6.3	0.10	2.078	0.418	3.760	1.280	6	9379	ZINC000002047016
0.1	-8.1	-7.7	0.26	1.910	0.463	3.740	1.538	6	9380	ZINC000014765292
0.1	-8.8	-7.7	0.51	2.031	0.370	4.285	1.107	6	9381	ZINC000006018931
0.1	-8.3	-8.0	0.25	1.861	0.306	3.709	1.679	6	9382	ZINC000015044536
0.1	-8.0	-7.8	0.16	3.202	0.824	5.544	1.585	6	9383	ZINC000013437983

0.1	-6.4	-6.2	0.15	1.788	0.891	3.729	1.728	6	9384	ZINC000001673031
0.1	-6.7	-6.4	0.22	3.150	0.630	4.340	0.879	6	9385	ZINC000000034159
0.1	-5.4	-5.3	0.07	1.974	0.638	4.087	0.793	6	9386	ZINC000001532864
0.1	-9.3	-7.9	0.86	2.125	0.566	5.970	1.353	6	9387	ZINC000013308650
0.1	-7.7	-7.5	0.21	2.273	0.275	4.342	1.572	6	9388	ZINC000015248708
0.1	-6.6	-6.4	0.15	1.903	1.148	2.978	0.772	6	9389	ZINC000100053150
0.1	-7.7	-7.5	0.17	1.860	0.294	3.750	0.936	6	9390	ZINC000001529633
0.1	-8.8	-8.5	0.22	2.076	0.378	4.520	1.310	6	9391	ZINC000410428642
0.1	-7.5	-7.2	0.22	1.859	0.274	4.318	1.418	6	9392	ZINC000004096986
0.1	-6.7	-6.6	0.07	2.750	0.503	5.196	0.505	6	9393	ZINC000028631259
0.1	-9.5	-8.6	0.55	2.237	0.420	4.252	0.469	6	9394	ZINC000100579991
0.1	-6.1	-6.0	0.09	2.886	1.135	4.717	1.371	6	9395	ZINC000013538225
0.1	-8.2	-7.8	0.36	2.163	0.664	3.410	1.469	6	9396	ZINC000015261561
0.1	-4.0	-3.8	0.14	1.342	0.683	2.301	0.220	6	9397	ZINC000008214541
0.1	-7.5	-7.2	0.26	1.181	0.564	3.027	0.509	6	9398	ZINC000261496103

0.1	-6.5	-6.4	0.13	2.339	0.369	3.578	0.448	6	9399	ZINC000002041131
0.1	-10.3	-8.9	1.08	1.705	0.083	5.181	1.730	5	9400	ZINC000256402996
0.1	-9.2	-8.9	0.23	1.841	0.704	4.105	1.156	6	9401	ZINC000257458462
0.1	-5.3	-5.2	0.11	1.959	0.647	3.013	0.832	6	9402	ZINC000005837961
0.1	-5.7	-5.5	0.17	2.166	0.570	3.764	0.927	6	9403	ZINC000000902058
0.1	-7.1	-6.9	0.14	1.757	0.793	4.019	0.667	6	9404	ZINC000100034396
0.1	-6.8	-6.5	0.21	2.685	0.578	4.245	0.710	6	9405	ZINC000001591828
0.1	-7.2	-7.0	0.11	2.294	0.235	4.125	0.393	6	9406	ZINC000004096362
0.1	-5.2	-4.8	0.24	2.036	0.687	2.573	0.574	6	9407	ZINC000000170090
0.1	-6.6	-6.1	0.37	2.854	0.443	4.226	0.886	6	9408	ZINC000001695428
0.1	-8.2	-7.8	0.24	1.497	0.300	4.542	1.927	6	9409	ZINC000044790312
0.1	-6.1	-5.9	0.12	3.119	0.643	4.387	0.897	6	9410	ZINC000034016985
0.1	-6.9	-6.8	0.10	2.074	0.327	4.122	0.722	6	9411	ZINC000255983261
0.1	-5.6	-5.4	0.24	1.985	1.046	3.919	0.883	6	9412	ZINC000012405128
0.1	-7.6	-7.4	0.18	1.921	0.260	3.127	0.578	6	9413	ZINC000040484034

0.1	-7.6	-7.4	0.12	1.457	0.429	3.883	1.681	6	9414	ZINC000004096699
0.1	-9.4	-8.8	0.43	1.662	0.122	4.042	1.251	6	9415	ZINC000014763052
0.1	-7.9	-7.7	0.17	1.673	0.458	4.097	0.401	6	9416	ZINC000085600697
0.1	-5.1	-4.9	0.12	1.885	0.629	2.936	0.860	6	9417	ZINC000005829539
0.1	-5.1	-4.9	0.11	1.925	0.567	2.838	0.640	6	9418	ZINC000038613497
0.1	-7.7	-7.6	0.07	2.110	1.326	4.117	1.471	6	9419	ZINC000003978454
0.1	-5.9	-5.7	0.11	1.513	0.612	4.345	0.665	6	9420	ZINC000095618256
0.1	-7.0	-6.7	0.20	2.418	0.631	3.599	0.809	6	9421	ZINC000062592267
0.1	-4.2	-3.9	0.19	1.520	0.654	2.312	0.465	6	9422	ZINC000013480025
0.1	-6.3	-6.3	0.04	1.670	0.917	4.094	0.900	6	9423	ZINC000000001504
0.1	-8.7	-8.2	0.28	3.267	0.400	5.553	0.982	6	9424	ZINC000013408673
0.1	-4.9	-4.7	0.11	2.183	0.590	2.770	0.916	6	9425	ZINC000001532530
0.1	-8.5	-8.2	0.16	2.251	0.280	4.499	1.006	6	9426	ZINC000002384989
0.1	-7.8	-7.5	0.31	2.222	0.450	4.783	1.371	6	9427	ZINC000257485926
0.1	-5.1	-4.8	0.14	3.821	0.781	4.299	0.630	6	9428	ZINC000000158648

0.1	-6.9	-6.6	0.31	1.960	0.496	4.300	1.192	6	9429	ZINC000015121890
0.1	-8.2	-7.9	0.24	1.971	1.087	4.081	1.300	6	9430	ZINC000014652543
0.1	-8.8	-8.6	0.13	2.115	0.552	4.386	1.305	6	9431	ZINC000001549362
0.1	-8.2	-7.5	0.34	2.050	0.576	5.206	2.233	6	9432	ZINC000014491299
0.1	-5.0	-4.8	0.10	3.204	0.800	4.491	0.796	6	9433	ZINC000100013589
0.1	-7.9	-7.6	0.25	2.261	0.951	4.330	1.925	6	9434	ZINC000065739349
0.1	-8.3	-8.0	0.28	1.798	0.306	3.594	1.534	6	9435	ZINC000015044528
0.1	-6.9	-6.7	0.11	2.521	0.351	4.061	1.052	6	9436	ZINC000035307770
0.1	-9.5	-8.5	0.60	1.392	0.263	2.809	0.559	6	9437	ZINC000095617909
0.1	-9.3	-8.7	0.44	1.779	0.466	4.909	1.549	6	9438	ZINC000008379775
0.1	-8.2	-7.5	0.35	1.834	0.520	4.304	2.431	6	9439	ZINC000014491303
0.1	-7.9	-7.3	0.30	2.571	0.241	5.019	0.870	6	9440	ZINC000027210242
0.1	-7.6	-7.2	0.27	2.041	0.213	3.985	1.288	6	9441	ZINC000002522657
0.1	-9.7	-9.4	0.29	1.344	0.379	4.237	2.457	6	9442	ZINC000000517261
0.1	-5.6	-5.5	0.14	1.789	0.339	2.884	0.677	6	9443	ZINC000015119659

0.1	-8.5	-7.5	0.50	2.131	0.175	5.550	1.234	6	9444	ZINC000257628116
0.1	-9.2	-8.7	0.36	1.938	0.268	3.424	1.073	6	9445	ZINC000257641761
0.1	-6.5	-6.2	0.20	1.236	0.260	3.820	0.711	6	9446	ZINC000065743156
0.1	-6.6	-6.4	0.10	1.633	0.312	3.008	0.821	6	9447	ZINC000100821353
0.1	-7.8	-7.6	0.16	2.465	0.656	4.266	0.466	6	9448	ZINC000000968134
0.1	-5.7	-5.6	0.08	2.557	0.903	3.556	1.860	6	9449	ZINC000004556876
0.1	-6.1	-5.9	0.09	3.079	0.881	4.844	1.487	6	9450	ZINC000002391069
0.1	-10.1	-8.7	0.72	1.856	0.487	3.986	1.612	6	9451	ZINC000013130924
0.1	-8.1	-7.3	0.59	1.951	0.159	5.150	0.617	6	9452	ZINC000004096284
0.1	-5.1	-5.0	0.07	2.288	0.837	3.681	1.296	6	9453	ZINC000095619618
0.1	-7.2	-6.9	0.24	2.168	0.384	4.992	1.625	6	9454	ZINC000014722312
0.1	-7.5	-7.1	0.33	1.758	0.339	3.159	1.666	6	9455	ZINC000040472418
0.1	-5.7	-5.6	0.11	2.150	0.305	3.706	0.829	6	9456	ZINC000002527917
0.1	-4.8	-4.4	0.18	2.523	0.546	3.016	0.696	6	9457	ZINC000001648359
0.1	-7.7	-7.4	0.18	3.257	0.303	4.404	0.700	6	9458	ZINC000019418965

0.1	-8.3	-7.8	0.30	2.638	0.255	5.176	1.792	6	9459	ZINC000018130229
0.1	-4.6	-4.3	0.24	2.489	0.628	3.100	0.768	6	9460	ZINC000002034597
0.1	-5.0	-4.9	0.12	2.066	0.292	3.353	0.564	6	9461	ZINC000000114134
0.1	-5.2	-5.0	0.11	2.338	0.356	4.814	0.349	6	9462	ZINC000019363537
0.1	-9.0	-8.2	0.44	1.895	0.475	3.977	0.531	6	9463	ZINC000015203703
0.1	-6.2	-5.9	0.17	1.665	0.624	3.709	1.027	6	9464	ZINC000001693323
0.1	-6.5	-6.3	0.13	2.306	0.663	4.521	1.206	6	9465	ZINC000000388238
0.1	-8.4	-8.1	0.17	1.460	0.591	5.017	2.188	6	9466	ZINC000004228251
0.1	-8.6	-8.2	0.21	2.390	0.681	5.358	1.248	6	9467	ZINC000014693338
0.1	-8.5	-7.5	0.68	1.444	0.756	2.855	0.702	6	9468	ZINC000014610075
0.1	-9.1	-8.6	0.26	2.198	0.479	4.720	0.538	6	9469	ZINC000050027372
0.1	-7.0	-6.9	0.07	2.210	0.536	3.701	1.699	6	9470	ZINC000095617798
0.1	-6.5	-6.3	0.19	2.456	0.972	4.112	1.257	6	9471	ZINC000004096190
0.1	-7.2	-7.0	0.11	2.283	0.245	3.962	0.690	6	9472	ZINC000012496902
0.1	-9.9	-8.8	0.58	1.476	0.212	3.236	1.495	6	9473	ZINC000005409389

0.1	-6.7	-6.6	0.08	2.356	0.415	4.568	0.950	6	9474	ZINC000013515330
0.1	-6.9	-6.5	0.25	2.356	0.488	3.983	0.945	6	9475	ZINC000004096092
0.1	-5.7	-5.6	0.07	1.829	1.194	2.780	1.147	6	9476	ZINC000001587728
0.1	-4.9	-4.6	0.16	2.052	1.336	3.717	1.299	6	9477	ZINC000100054128
0.1	-8.4	-7.7	0.33	1.673	0.515	4.023	1.042	6	9478	ZINC000002558139
0.1	-4.6	-4.4	0.11	1.746	0.276	2.878	0.576	6	9479	ZINC000013460366
0.1	-8.3	-7.9	0.23	1.500	0.295	3.485	1.730	6	9480	ZINC000028825577
0.1	-6.3	-6.1	0.11	2.561	0.355	5.233	1.252	6	9481	ZINC000001677778
0.1	-8.8	-8.6	0.11	0.915	0.165	4.100	0.654	6	9482	ZINC000230081381
0.1	-8.2	-7.9	0.18	2.185	0.210	4.432	0.780	6	9483	ZINC000095619730
0.1	-6.9	-6.8	0.16	1.888	0.442	4.138	1.859	6	9484	ZINC000014858404
0.1	-4.8	-4.6	0.10	4.128	0.252	4.552	0.340	6	9485	ZINC000000130187
0.1	-10.4	-9.9	0.27	1.760	0.399	4.378	0.616	6	9486	ZINC000257749157
0.1	-6.8	-6.6	0.17	1.718	0.811	3.454	0.488	6	9487	ZINC000003875795
0.1	-8.6	-8.4	0.27	1.841	0.324	4.339	1.555	6	9488	ZINC000257474758

0.1	-6.4	-6.3	0.14	1.660	0.978	2.710	1.296	6	9489	ZINC000014505294
0.1	-8.1	-7.7	0.23	1.766	0.338	4.681	1.391	6	9490	ZINC000040165447
0.1	-6.7	-6.5	0.10	3.035	0.257	4.571	0.276	6	9491	ZINC000050026936
0.1	-6.1	-5.6	0.23	2.131	0.613	4.202	0.710	6	9492	ZINC000001531154
0.1	-8.4	-8.2	0.11	2.750	0.612	4.305	1.465	6	9493	ZINC000003641064
0.1	-6.8	-6.6	0.17	2.379	0.477	4.068	2.044	6	9494	ZINC000004533520
0.1	-8.6	-8.4	0.15	2.271	0.552	4.501	1.494	6	9495	ZINC000006018386
0.1	-6.5	-6.1	0.21	2.816	0.796	4.346	1.281	6	9496	ZINC000015113209
0.1	-7.7	-7.5	0.15	1.818	0.457	3.343	1.768	6	9497	ZINC000034018549
0.1	-6.4	-6.3	0.14	2.828	1.257	3.991	1.290	6	9498	ZINC000084462581
0.1	-7.7	-7.5	0.21	2.215	0.508	3.527	1.639	6	9499	ZINC000040479043
0.1	-8.2	-7.7	0.26	2.228	0.179	5.031	0.566	6	9500	ZINC000012494328
0.1	-6.4	-6.2	0.13	1.966	0.244	3.357	1.500	6	9501	ZINC000015120790
0.1	-6.5	-6.2	0.18	2.142	0.600	3.762	1.052	6	9502	ZINC000000056459
0.1	-5.5	-5.3	0.17	2.203	0.386	3.658	0.443	6	9503	ZINC000014438522

0.1	-5.0	-4.5	0.28	2.499	1.756	3.304	1.794	6	9504	ZINC000000394027
0.1	-6.6	-6.4	0.12	2.268	0.553	3.072	1.118	6	9505	ZINC000005274025
0.1	-8.2	-7.5	0.37	1.747	0.444	3.774	1.825	6	9506	ZINC000014491297
0.1	-8.9	-8.3	0.29	1.769	0.215	3.571	1.455	6	9507	ZINC000015220800
0.1	-8.0	-6.9	0.63	1.718	0.569	4.662	1.598	6	9508	ZINC000257533585
0.1	-6.8	-6.7	0.05	3.696	0.290	5.199	0.568	6	9509	ZINC000014491514
0.1	-8.7	-7.7	0.47	1.560	0.161	5.204	0.405	6	9510	ZINC000257592650
0.1	-9.5	-8.8	0.43	2.099	0.368	5.399	2.101	6	9511	ZINC000257496643
0.1	-9.0	-7.8	0.61	1.958	0.148	2.991	0.379	5	9512	ZINC000014610071
0.1	-8.0	-7.7	0.17	3.087	1.070	5.481	1.711	6	9513	ZINC000013437980
0.1	-8.8	-8.6	0.19	2.173	0.222	4.129	1.602	6	9514	ZINC000013371914
0.1	-6.7	-6.4	0.16	2.438	0.208	4.568	0.523	6	9515	ZINC000012494207
0.1	-6.6	-6.3	0.17	1.799	0.511	3.966	1.057	6	9516	ZINC000012504390
0.1	-4.3	-4.0	0.16	2.443	0.782	3.258	1.055	6	9517	ZINC000000388078
0.1	-7.1	-6.9	0.17	1.558	0.210	3.602	1.440	6	9518	ZINC000066331979

0.1	-5.6	-5.4	0.22	3.129	1.300	4.697	1.209	6	9519	ZINC000001575549
0.1	-6.5	-6.3	0.16	2.715	0.615	4.863	0.511	6	9520	ZINC000004556675
0.1	-6.0	-5.8	0.12	4.438	1.758	5.361	1.684	6	9521	ZINC000001843029
0.1	-4.2	-4.0	0.15	3.079	0.967	3.542	0.903	6	9522	ZINC000000901132
0.1	-8.1	-7.8	0.23	1.710	0.707	3.267	1.700	6	9523	ZINC000013461541
0.1	-8.8	-8.6	0.11	0.900	0.162	4.108	0.657	6	9524	ZINC000257417627
0.1	-9.9	-9.6	0.16	1.890	0.192	4.290	1.838	6	9525	ZINC000257693622
0.1	-9.0	-8.6	0.22	2.243	0.841	4.027	0.747	6	9526	ZINC000100777220
0.1	-7.8	-7.4	0.26	2.872	0.928	4.784	1.656	6	9527	ZINC000001569734
0.1	-9.7	-8.8	0.60	1.944	0.755	4.301	1.838	6	9528	ZINC000257537731
0.1	-3.5	-3.3	0.11	3.540	2.316	4.153	2.329	6	9529	ZINC000000895664
0.1	-6.5	-6.5	0.04	2.011	0.195	4.618	1.104	6	9530	ZINC000012495589
0.1	-6.6	-6.4	0.18	2.536	0.765	4.293	1.635	6	9531	ZINC000000056626
0.1	-8.5	-8.4	0.09	2.217	0.771	4.336	1.939	6	9532	ZINC000095620805
0.1	-7.5	-6.9	0.27	2.507	0.279	4.472	1.758	6	9533	ZINC000095617733

0.1	-8.0	-7.6	0.24	1.932	0.291	5.009	0.983	6	9534	ZINC000100770494
0.1	-7.3	-7.0	0.12	1.524	0.076	3.372	0.607	6	9535	ZINC000005735772
0.1	-5.0	-4.8	0.20	2.454	0.892	2.871	0.885	6	9536	ZINC000001529230
0.1	-8.2	-7.8	0.32	1.762	0.522	3.552	1.795	6	9537	ZINC000000897908
0.1	-6.9	-6.8	0.11	2.011	0.433	2.741	0.644	6	9538	ZINC000004654886
0.1	-7.3	-7.0	0.19	2.321	0.176	5.243	0.220	6	9539	ZINC000034961834
0.1	-9.2	-8.7	0.28	1.818	0.198	4.858	1.412	6	9540	ZINC000100824315
0.1	-7.0	-6.8	0.20	1.811	1.003	2.729	1.314	6	9541	ZINC000000493112
0.1	-7.3	-7.0	0.27	1.747	0.493	2.336	0.630	6	9542	ZINC000000895905
0.1	-8.8	-8.4	0.21	1.521	0.066	4.175	1.695	6	9543	ZINC000014764385
0.1	-9.4	-9.2	0.24	1.818	0.401	3.542	1.265	6	9544	ZINC000257419894
0.1	-7.1	-6.8	0.15	1.771	0.191	3.896	1.308	6	9545	ZINC000002002692
0.1	-7.7	-7.1	0.44	1.839	1.206	2.405	0.904	6	9546	ZINC000000073711
0.1	-9.9	-9.4	0.32	2.604	0.430	4.792	1.054	6	9547	ZINC000002383057
0.1	-9.4	-9.2	0.21	1.623	0.320	3.774	1.556	6	9548	ZINC000015057682

0.1	-3.2	-3.2	0.05	2.406	0.890	3.154	1.022	6	9549	ZINC000002004049
0.1	-6.1	-5.8	0.20	1.859	0.202	3.417	1.160	6	9550	ZINC000257379871
0.1	-5.9	-5.7	0.18	1.702	0.651	3.448	1.319	6	9551	ZINC000014419528
0.1	-5.8	-5.6	0.14	2.994	1.115	5.066	0.531	6	9552	ZINC000005134646
0.1	-5.7	-5.4	0.15	2.564	0.799	3.251	0.827	6	9553	ZINC000014419737
0.1	-8.5	-7.9	0.33	2.230	0.890	3.775	1.472	6	9554	ZINC000018169811
0.1	-9.1	-8.3	0.48	2.451	1.044	4.464	1.906	6	9555	ZINC000014760155
0.1	-7.7	-7.4	0.23	2.648	0.279	3.573	0.294	6	9556	ZINC000085588719
0.1	-9.1	-8.7	0.27	1.321	0.276	3.669	0.655	6	9557	ZINC000100783438
0.1	-8.2	-6.8	0.73	1.934	0.426	5.656	0.754	6	9558	ZINC000014828294
0.1	-6.5	-6.4	0.07	2.009	0.954	3.378	0.704	6	9559	ZINC000256066513
0.1	-7.6	-7.5	0.11	1.661	0.413	2.645	0.871	6	9560	ZINC000040484031
0.1	-8.0	-7.8	0.16	3.195	0.823	5.543	1.586	6	9561	ZINC000013437984
0.1	-8.9	-7.9	0.51	1.739	0.167	4.302	0.997	6	9562	ZINC000015216884
0.1	-8.2	-8.0	0.16	1.611	0.439	4.209	0.651	6	9563	ZINC000085934995

0.1	-8.1	-7.9	0.10	1.820	0.335	3.687	1.443	6	9564	ZINC000015256925
0.1	-5.4	-5.1	0.16	1.552	0.470	2.474	0.679	6	9565	ZINC000001529662
0.1	-9.1	-8.8	0.16	1.730	0.245	4.890	0.910	6	9566	ZINC000040949810
0.1	-8.5	-8.1	0.25	1.638	0.430	4.751	1.676	6	9567	ZINC000085794981
0.1	-8.0	-7.8	0.14	3.209	0.823	5.546	1.583	6	9568	ZINC000014964390
0.1	-6.7	-6.5	0.11	2.996	0.640	4.969	1.202	6	9569	ZINC000034267186
0.1	-9.9	-8.8	1.15	1.984	0.000	4.790	0.000	2	9570	ZINC000014688358
0.1	-4.9	-4.7	0.08	3.441	1.857	4.892	1.640	6	9571	ZINC000032786028
0.1	-8.1	-7.0	0.60	1.859	0.405	3.838	1.746	6	9572	ZINC000014443997
0.1	-6.4	-6.2	0.16	2.055	1.001	3.126	1.305	6	9573	ZINC000000402870
0.1	-8.5	-6.9	1.15	1.330	0.582	2.962	1.573	6	9574	ZINC000013545798
0.1	-5.3	-5.1	0.12	1.834	0.547	2.670	0.370	6	9575	ZINC000005820872
0.1	-4.0	-3.9	0.07	2.816	0.859	3.765	0.804	6	9576	ZINC000002568012
0.1	-8.1	-8.0	0.09	1.055	0.838	3.769	1.991	6	9577	ZINC000013377095
0.1	-6.5	-6.4	0.05	2.885	0.611	4.471	1.623	6	9578	ZINC000001611161

0.1	-8.2	-7.5	0.32	1.765	0.621	4.199	2.517	6	9579	ZINC000257398699
0.1	-5.9	-5.6	0.17	2.908	0.388	3.477	0.484	6	9580	ZINC000000152270
0.1	-7.0	-6.6	0.19	2.573	0.405	4.211	0.522	6	9581	ZINC000001757674
0.1	-10.3	-10.0	0.24	1.806	0.978	4.900	1.216	6	9582	ZINC000257409224
0.1	-6.6	-6.4	0.10	1.640	0.270	2.999	0.747	6	9583	ZINC000012153389
0.1	-8.8	-8.2	0.35	1.706	0.308	5.642	1.767	6	9584	ZINC000014775723
0.1	-9.0	-8.3	0.41	2.863	0.471	5.279	1.022	6	9585	ZINC000050027347
0.1	-4.6	-4.5	0.05	2.204	1.446	3.338	1.468	6	9586	ZINC000001850009
0.1	-7.6	-7.4	0.20	2.905	0.461	5.096	0.760	6	9587	ZINC000002002632
0.1	-6.4	-6.1	0.20	2.754	0.789	3.828	0.747	6	9588	ZINC000000057624
0.1	-7.7	-7.2	0.31	2.360	0.603	4.699	1.106	6	9589	ZINC000257555570
0.1	-9.4	-8.8	0.29	1.730	0.269	4.893	0.108	6	9590	ZINC000014646573
0.1	-7.8	-7.4	0.32	2.489	0.358	4.092	0.665	6	9591	ZINC000014592618
0.1	-7.6	-7.3	0.19	3.663	0.769	4.845	1.380	6	9592	ZINC000002020167
0.1	-7.7	-7.4	0.21	2.557	0.712	4.142	0.709	6	9593	ZINC000008738372

0.1	-8.8	-8.6	0.11	0.911	0.171	4.101	0.655	6	9594	ZINC000104366392
0.1	-6.6	-6.4	0.15	2.345	0.550	3.246	0.473	6	9595	ZINC000000396530
0.1	-6.1	-5.9	0.23	1.873	0.339	4.037	0.955	6	9596	ZINC000090440654
0.1	-7.5	-7.2	0.20	2.684	0.176	5.270	1.119	6	9597	ZINC000013480185
0.1	-6.9	-6.9	0.05	2.101	0.605	3.864	1.562	6	9598	ZINC000000073634
0.1	-7.9	-7.6	0.17	1.707	0.606	4.471	1.473	6	9599	ZINC000027638905
0.1	-6.7	-6.6	0.13	2.179	0.327	4.019	1.228	6	9600	ZINC000034027887
0.1	-7.1	-7.0	0.08	1.495	0.434	3.979	0.568	6	9601	ZINC000257630983
0.1	-6.3	-6.1	0.16	2.668	0.766	3.986	0.814	6	9602	ZINC000212543292
0.1	-7.5	-7.4	0.09	2.010	0.569	3.290	1.386	6	9603	ZINC000257563116
0.1	-9.6	-9.1	0.39	1.672	0.215	3.735	1.135	6	9604	ZINC000257429061
0.1	-4.1	-4.0	0.10	1.613	0.503	2.584	0.321	6	9605	ZINC000002034789
0.1	-9.3	-9.0	0.20	1.440	0.144	5.823	0.805	6	9606	ZINC000257601056
0.1	-10.0	-9.7	0.29	0.982	0.479	4.582	1.502	6	9607	ZINC000003872446
0.1	-5.9	-5.4	0.29	2.619	1.121	3.852	1.108	6	9608	ZINC000032163590

0.1	-8.3	-7.8	0.24	2.022	0.619	4.646	1.388	6	9609	ZINC000257384872
0.1	-9.6	-7.9	0.96	2.083	0.406	4.478	0.741	6	9610	ZINC000000899569
0.1	-7.7	-7.5	0.15	2.553	0.131	4.129	1.187	6	9611	ZINC000040473191
0.1	-9.6	-7.9	0.92	1.641	0.200	4.765	1.637	6	9612	ZINC000247807498
0.1	-6.5	-6.1	0.17	2.708	0.397	4.378	1.282	6	9613	ZINC000040542580
0.1	-6.3	-6.1	0.10	2.244	0.523	2.618	0.541	6	9614	ZINC000000403283
0.1	-9.5	-9.2	0.16	1.596	0.647	3.499	1.794	6	9615	ZINC000014650950
0.1	-9.9	-9.0	0.60	2.278	0.259	4.340	0.569	6	9616	ZINC000163794228
0.1	-9.9	-9.6	0.16	1.900	0.194	4.296	1.836	6	9617	ZINC000015169358
0.1	-4.8	-4.7	0.11	3.020	1.136	3.955	0.330	6	9618	ZINC000032166512
0.1	-9.6	-8.8	0.86	1.783	0.415	4.677	1.540	5	9619	ZINC000001711823
0.1	-3.4	-3.4	0.00	2.718	3.261	4.134	2.414	6	9620	ZINC000001692439
0.1	-6.6	-6.3	0.15	1.693	0.289	4.126	1.176	6	9621	ZINC000004726902
0.1	-5.7	-5.4	0.20	1.686	0.398	2.817	0.692	6	9622	ZINC000001529421
0.1	-4.4	-4.3	0.12	2.207	0.797	2.628	1.086	6	9623	ZINC000000409334

0.1	-9.4	-8.6	1.01	1.906	0.320	3.536	1.593	5	9624	ZINC000100782619
0.1	-7.6	-7.4	0.14	2.178	0.535	3.599	0.705	6	9625	ZINC000006031851
0.1	-7.0	-6.8	0.14	2.760	0.878	3.833	1.743	6	9626	ZINC000000001575
0.1	-5.4	-5.3	0.05	2.145	0.251	2.854	0.306	6	9627	ZINC000100029274
0.1	-6.0	-5.9	0.08	3.146	0.475	5.235	0.424	6	9628	ZINC000005500823
0.1	-7.1	-7.0	0.08	1.375	0.398	2.857	1.164	6	9629	ZINC000257630980
0.1	-9.4	-9.1	0.33	1.915	0.303	4.454	1.032	6	9630	ZINC000257419893
0.1	-8.8	-8.6	0.10	1.760	0.237	4.453	1.843	6	9631	ZINC000013382465
0.1	-6.9	-6.5	0.18	2.575	0.737	4.176	1.474	6	9632	ZINC000000402938
0.1	-8.8	-8.5	0.28	1.859	0.278	3.743	1.038	6	9633	ZINC000014453477
0.1	-8.3	-7.8	0.44	1.781	0.035	3.750	1.236	6	9634	ZINC000257420300
0.1	-8.3	-8.0	0.22	1.625	0.276	2.440	0.416	6	9635	ZINC000015044525
0.1	-6.6	-6.4	0.13	2.005	0.631	3.276	1.576	6	9636	ZINC000005274098
0.1	-4.9	-4.7	0.11	2.396	0.224	3.263	0.699	6	9637	ZINC000003861729
0.1	-5.9	-5.5	0.20	2.096	0.475	3.962	0.869	6	9638	ZINC000014589067

0.1	-4.5	-4.4	0.12	6.029	1.916	6.883	2.138	6	9639	ZINC000000895862
0.1	-9.8	-9.3	0.32	1.444	0.324	4.153	1.216	6	9640	ZINC000086028078
0.1	-6.2	-6.1	0.05	2.557	0.870	4.070	1.945	6	9641	ZINC000038339735
0.1	-5.9	-5.7	0.11	2.757	0.558	4.137	1.112	6	9642	ZINC000002575128
0.1	-8.5	-8.0	0.33	2.044	0.474	4.110	0.638	6	9643	ZINC000013408243
0.1	-5.4	-5.3	0.06	2.920	0.636	4.201	0.451	6	9644	ZINC000004706056
0.1	-9.0	-7.8	0.58	1.603	0.336	5.066	1.779	6	9645	ZINC000013411700
0.1	-8.0	-7.7	0.21	3.035	0.209	4.978	0.350	6	9646	ZINC000001569746
0.1	-8.8	-8.5	0.14	1.654	0.411	2.572	0.384	6	9647	ZINC000257377280
0.1	-8.2	-7.8	0.21	1.677	0.114	6.234	0.752	6	9648	ZINC000014817993
0.1	-8.4	-7.3	0.91	1.880	0.383	5.245	2.000	6	9649	ZINC000014811612
0.1	-8.5	-7.8	0.79	3.015	0.217	5.249	0.563	6	9650	ZINC000014820483
0.1	-8.6	-7.9	0.37	1.731	0.157	5.529	1.850	6	9651	ZINC000257418690
0.1	-4.6	-4.4	0.11	5.091	2.457	6.098	2.271	6	9652	ZINC000013526470
0.1	-5.6	-5.3	0.16	3.272	0.906	4.732	1.278	6	9653	ZINC000005131905

0.1	-9.1	-8.3	0.42	2.098	0.179	4.150	0.961	6	9654	ZINC000257591137
0.1	-7.7	-7.3	0.36	2.062	0.582	4.348	1.625	6	9655	ZINC000005863151
0.1	-6.6	-6.4	0.10	2.488	0.780	4.637	0.957	6	9656	ZINC000050027130
0.1	-3.8	-3.6	0.12	2.339	1.093	3.463	1.065	6	9657	ZINC000012495988
0.1	-7.0	-6.7	0.26	1.936	1.169	2.905	1.376	6	9658	ZINC000000493111
0.1	-9.2	-8.5	0.43	2.238	0.474	3.612	1.497	6	9659	ZINC000013461788
0.1	-8.3	-7.6	0.46	1.770	0.171	4.336	1.491	6	9660	ZINC000014765291
0.1	-8.6	-7.3	0.60	1.706	0.293	4.505	0.787	6	9661	ZINC000086010708
0.1	-5.8	-5.6	0.10	1.336	0.386	3.666	1.006	6	9662	ZINC000021983366
0.1	-5.1	-4.8	0.16	1.929	0.542	2.306	0.508	6	9663	ZINC000005224866
0.1	-7.4	-7.3	0.12	1.956	0.144	3.155	1.648	6	9664	ZINC000040471399
0.1	-5.4	-5.2	0.08	1.968	1.162	3.996	1.418	6	9665	ZINC000001532049
0.1	-5.3	-5.1	0.13	2.114	0.615	3.049	1.082	6	9666	ZINC000000895124
0.1	-8.2	-7.8	0.29	1.478	0.286	4.522	1.898	6	9667	ZINC000036532687
0.1	-3.2	-3.0	0.12	6.975	0.906	7.587	0.820	6	9668	ZINC000000901039

0.1	-9.3	-8.5	0.72	1.594	0.518	4.184	2.267	6	9669	ZINC000014437098
0.1	-7.7	-7.5	0.15	2.840	0.532	4.519	1.203	6	9670	ZINC000000056406
0.1	-8.1	-7.7	0.27	2.466	0.732	4.074	1.634	6	9671	ZINC000001579638
0.1	-9.6	-8.5	0.89	1.856	0.323	6.703	0.420	6	9672	ZINC000014689036
0.1	-9.2	-9.0	0.21	1.513	0.183	3.962	1.024	6	9673	ZINC000014828129
0.1	-5.9	-5.8	0.07	2.078	0.457	3.415	1.319	6	9674	ZINC000013383608
0.1	-8.7	-8.3	0.34	1.967	0.872	3.471	1.687	6	9675	ZINC000012343956
0.1	-7.0	-6.7	0.15	2.390	0.660	4.404	1.324	6	9676	ZINC000034361788
0.1	-7.4	-7.2	0.13	2.110	0.482	4.512	1.599	6	9677	ZINC000014616308
0.1	-7.4	-7.1	0.24	2.157	0.167	4.622	0.741	6	9678	ZINC000001609418
0.1	-8.9	-8.5	0.29	2.374	0.292	4.672	0.884	6	9679	ZINC000001570253
0.1	-8.9	-8.5	0.39	1.983	0.353	3.872	1.590	6	9680	ZINC000100783420
0.1	-9.7	-8.1	0.86	2.714	0.190	6.215	0.696	6	9681	ZINC000257505342
0.1	-4.9	-4.5	0.26	2.235	0.710	2.428	0.850	6	9682	ZINC000000391822
0.1	-8.6	-8.4	0.11	2.366	0.407	4.998	1.298	6	9683	ZINC000001617238

0.1	-7.4	-6.7	0.31	1.596	0.260	5.744	1.710	6	9684	ZINC000257516122
0.1	-9.1	-8.9	0.12	1.712	0.210	4.212	1.496	6	9685	ZINC000001667453
0.1	-5.4	-5.3	0.12	2.317	0.574	3.377	0.897	6	9686	ZINC000001997926
0.1	-6.2	-5.9	0.18	2.085	1.149	3.390	0.499	6	9687	ZINC000100003619
0.1	-7.7	-7.4	0.21	2.018	0.328	3.999	1.898	6	9688	ZINC000050027880
0.1	-7.8	-7.5	0.15	2.680	0.448	4.451	1.096	6	9689	ZINC000017129255
0.1	-7.0	-6.7	0.15	1.914	0.216	5.009	1.561	6	9690	ZINC000085649290
0.1	-7.4	-6.7	0.30	1.579	0.267	5.738	1.713	6	9691	ZINC000014921706
0.1	-8.9	-8.2	0.34	2.277	0.225	5.913	1.645	6	9692	ZINC000013311888
0.1	-5.1	-4.9	0.13	2.458	0.422	3.382	0.537	6	9693	ZINC000015272647
0.1	-9.0	-7.2	0.84	1.130	0.151	3.735	2.000	6	9694	ZINC000008217220
0.1	-5.2	-5.2	0.05	3.549	1.371	4.950	1.085	6	9695	ZINC000005543431
0.1	-2.4	-2.4	0.05	5.297	2.019	6.027	1.895	6	9696	ZINC000008216125
0.1	-6.9	-6.8	0.12	2.173	0.122	3.901	0.723	6	9697	ZINC000255983264
0.1	-7.7	-7.5	0.15	1.637	0.226	3.948	0.873	6	9698	ZINC000005103852

0.1	-6.7	-6.6	0.08	2.247	0.808	3.057	0.924	6	9699	ZINC000000034160
0.1	-7.8	-7.6	0.11	1.832	0.830	3.843	1.456	6	9700	ZINC000003869496
0.1	-5.9	-5.8	0.10	1.376	0.286	2.832	0.938	6	9701	ZINC000033839585
0.1	-6.1	-5.9	0.13	2.818	0.423	4.668	0.760	6	9702	ZINC000001678871
0.1	-5.0	-4.8	0.11	2.630	1.345	3.631	1.624	6	9703	ZINC000014500538
0.1	-6.1	-6.0	0.07	2.652	0.390	5.213	1.269	6	9704	ZINC000050027407
0.1	-7.4	-7.0	0.27	1.891	0.255	4.574	1.957	6	9705	ZINC000014610454
0.1	-8.4	-7.8	0.40	1.829	0.396	4.968	1.821	6	9706	ZINC000014642309
0.1	-5.5	-5.4	0.07	2.779	1.822	4.573	1.599	6	9707	ZINC000005020010
0.1	-8.8	-7.2	0.80	2.096	0.472	3.986	1.599	6	9708	ZINC000013334938
0.1	-5.8	-5.7	0.09	5.034	0.103	6.251	0.519	6	9709	ZINC000003860326
0.1	-8.6	-8.5	0.06	2.753	0.428	5.389	1.228	6	9710	ZINC000050027013
0.1	-7.0	-6.8	0.18	1.760	0.973	2.833	1.275	6	9711	ZINC000000493110
0.1	-6.2	-6.0	0.13	2.613	0.749	3.182	0.672	6	9712	ZINC000000049143
0.1	-6.7	-6.6	0.08	2.854	0.832	4.513	0.680	6	9713	ZINC000014636774

0.1	-5.5	-5.4	0.07	3.298	0.550	4.221	0.321	6	9714	ZINC000000114121
0.1	-7.7	-7.3	0.22	2.200	0.779	3.494	1.832	6	9715	ZINC000050027871
0.1	-7.8	-7.6	0.14	2.017	0.368	3.539	1.368	6	9716	ZINC000014414838
0.1	-5.5	-5.3	0.12	3.120	1.400	4.000	1.671	6	9717	ZINC000006119176
0.1	-6.8	-6.6	0.14	1.701	0.335	3.900	0.561	6	9718	ZINC000410428639
0.1	-7.1	-6.7	0.21	2.304	0.080	5.096	1.143	6	9719	ZINC000255986989
0.1	-8.3	-7.6	0.48	2.254	0.499	5.961	1.741	6	9720	ZINC000257469828
0.1	-8.5	-7.9	0.40	2.713	0.269	4.872	0.574	6	9721	ZINC000001638003
0.1	-7.6	-7.4	0.11	3.067	0.510	5.010	0.306	6	9722	ZINC000001589379
0.1	-8.0	-7.8	0.10	1.890	0.518	4.097	1.205	6	9723	ZINC000005360194
0.1	-5.4	-5.1	0.16	2.353	0.285	3.158	0.574	6	9724	ZINC000004202722
0.1	-6.2	-6.0	0.16	2.889	0.406	3.493	0.370	6	9725	ZINC000000406211
0.1	-8.3	-8.0	0.16	2.767	0.724	4.823	1.260	6	9726	ZINC000014489340
0.1	-3.1	-3.1	0.00	3.251	2.607	4.426	1.598	6	9727	ZINC000261499688
0.1	-6.6	-6.4	0.12	2.567	0.278	4.502	0.573	6	9728	ZINC000001572038

0.1	-8.3	-7.9	0.22	1.714	0.349	5.466	1.672	6	9729	ZINC000013517977
0.1	-4.4	-4.3	0.07	1.844	0.305	2.431	0.506	6	9730	ZINC000033839397
0.1	-7.9	-7.7	0.13	1.625	0.155	3.918	0.835	6	9731	ZINC000085599775
0.1	-7.1	-7.0	0.07	2.681	0.287	4.954	0.742	6	9732	ZINC000095617764
0.1	-7.5	-7.2	0.20	1.990	0.243	6.002	1.279	6	9733	ZINC000100820608
0.1	-2.6	-2.5	0.09	2.899	1.663	3.338	1.690	6	9734	ZINC000002031661
0.1	-8.4	-8.0	0.21	1.220	0.309	5.192	1.976	6	9735	ZINC000012953172
0.1	-7.7	-7.3	0.29	2.541	0.927	3.685	1.450	6	9736	ZINC000000039099
0.1	-9.0	-8.1	0.76	1.838	0.546	5.159	2.205	6	9737	ZINC000014612855
0.1	-6.9	-6.6	0.18	2.297	0.433	3.981	0.811	6	9738	ZINC000003869664
0.1	-8.3	-7.8	0.30	1.593	0.245	5.188	1.785	6	9739	ZINC000257621025
0.1	-9.9	-9.5	0.19	1.831	0.255	4.468	1.706	6	9740	ZINC000257693621
0.1	-7.4	-7.3	0.09	2.519	0.525	6.162	0.449	6	9741	ZINC000095617717
0.1	-7.1	-7.1	0.05	3.051	0.393	5.671	0.513	6	9742	ZINC000001786143
0.1	-7.9	-7.8	0.11	1.889	0.359	3.248	1.721	6	9743	ZINC000012358753

0.1	-6.1	-6.0	0.13	2.022	0.225	4.500	0.302	6	9744	ZINC000002384560
0.1	-5.4	-5.3	0.07	2.437	0.357	3.084	0.351	6	9745	ZINC000100029277
0.1	-7.4	-7.3	0.09	2.303	0.566	4.146	1.070	6	9746	ZINC000050026947
0.1	-4.9	-4.7	0.12	2.642	2.056	3.627	1.892	6	9747	ZINC000001532692
0.1	-8.0	-7.6	0.23	2.027	0.381	5.173	0.667	6	9748	ZINC000257765104
0.1	-8.1	-7.6	0.35	1.579	0.319	5.605	1.169	6	9749	ZINC000014725178
0.1	-7.1	-7.0	0.07	1.356	0.402	3.511	0.990	6	9750	ZINC000100824542
0.1	-9.5	-8.6	0.49	2.302	0.428	5.545	1.831	6	9751	ZINC000257496645
0.1	-5.1	-5.0	0.07	2.182	0.691	4.366	0.362	6	9752	ZINC000142307162
0.1	-6.3	-6.2	0.08	2.955	0.445	4.023	0.426	6	9753	ZINC000000119675
0.1	-7.4	-7.1	0.21	1.870	0.254	3.369	1.117	6	9754	ZINC000015149668
0.1	-8.8	-8.1	0.44	2.029	0.268	5.802	0.542	6	9755	ZINC000014590611
0.1	-5.6	-5.5	0.12	2.162	0.947	2.985	0.969	6	9756	ZINC000001693960
0.1	-5.4	-5.4	0.00	0.056	0.018	3.013	0.301	6	9757	ZINC000000391883
0.1	-5.7	-5.4	0.18	2.516	0.405	3.202	0.667	6	9758	ZINC000014419733

0.1	-5.7	-5.6	0.11	2.963	0.492	4.008	0.733	6	9759	ZINC000001734221
0.1	-10.2	-9.6	0.68	2.288	0.608	4.874	1.938	6	9760	ZINC000090586582
0.1	-6.3	-6.2	0.07	2.349	0.364	5.283	1.119	6	9761	ZINC000014491747
0.1	-7.9	-7.6	0.16	1.433	0.369	4.930	1.987	6	9762	ZINC000261499678
0.1	-8.6	-7.9	0.48	2.397	0.159	4.530	0.984	6	9763	ZINC000014489227
0.1	-7.1	-6.7	0.20	2.236	0.207	4.398	1.235	6	9764	ZINC000255986988
0.1	-7.0	-6.6	0.21	3.819	0.238	4.614	0.161	6	9765	ZINC000000266964
0.1	-6.8	-6.6	0.21	2.237	0.963	3.843	1.799	6	9766	ZINC000032190038
0.1	-8.9	-8.7	0.21	2.053	0.160	4.409	2.110	6	9767	ZINC000100776930
0.1	-6.3	-6.0	0.11	2.810	0.490	4.594	0.516	6	9768	ZINC000001576201
0.1	-7.7	-7.4	0.28	2.120	0.564	3.490	1.509	6	9769	ZINC000005863164
0.1	-8.0	-7.7	0.17	1.804	0.570	3.321	1.604	6	9770	ZINC000014660062
0.1	-9.0	-8.5	0.32	2.790	0.441	4.849	0.680	6	9771	ZINC000014493338
0.1	-7.6	-7.5	0.08	1.509	0.706	2.536	0.569	6	9772	ZINC000100019054
0.1	-8.2	-7.8	0.27	1.455	0.283	4.461	1.830	6	9773	ZINC000004217475

0.1	-3.9	-3.8	0.06	2.476	1.148	3.101	1.034	6	9774	ZINC000003860797
0.1	-7.8	-7.6	0.16	1.806	0.720	2.919	1.321	6	9775	ZINC000033641279
0.1	-6.3	-6.1	0.12	2.133	0.361	4.161	0.944	6	9776	ZINC000002512466
0.1	-5.1	-5.0	0.07	2.707	0.904	3.460	0.914	6	9777	ZINC000002047226
0.1	-5.6	-5.3	0.15	2.603	1.296	3.734	1.214	6	9778	ZINC000005821936
0.1	-9.3	-8.8	0.32	1.990	0.146	6.175	0.559	6	9779	ZINC000013660146
0.1	-6.9	-6.7	0.07	2.646	0.598	5.086	0.758	6	9780	ZINC000100776194
0.1	-6.6	-6.4	0.07	2.381	0.678	3.500	0.687	6	9781	ZINC000150307441
0.1	-8.4	-8.2	0.15	1.681	0.135	5.346	0.429	6	9782	ZINC000014771148
0.1	-4.9	-4.8	0.10	3.145	1.758	4.051	1.906	6	9783	ZINC000000895228
0.1	-10.3	-9.9	0.28	2.146	0.416	4.322	1.755	6	9784	ZINC000015169360
0.1	-5.0	-4.9	0.07	2.889	1.701	3.962	1.538	6	9785	ZINC000000895425
0.1	-9.1	-8.7	0.33	1.601	0.425	4.036	1.357	6	9786	ZINC000014588934
0.1	-9.0	-8.7	0.16	1.751	0.435	3.557	0.983	6	9787	ZINC000014504726
0.1	-6.5	-6.2	0.16	2.368	0.713	3.612	0.231	6	9788	ZINC000002573891

0.1	-9.2	-8.9	0.23	1.834	0.707	4.089	1.179	6	9789	ZINC000257458460
0.1	-8.6	-8.2	0.25	2.108	0.641	3.914	1.695	6	9790	ZINC000014856854
0.1	-8.7	-8.0	0.46	2.015	0.177	4.694	1.331	6	9791	ZINC000085589198
0.1	-5.5	-5.4	0.05	1.825	1.335	3.650	0.768	6	9792	ZINC000000388167
0.1	-8.8	-8.0	0.51	1.755	0.368	5.502	0.983	6	9793	ZINC000015058664
0.1	-7.0	-6.8	0.15	2.779	0.524	4.416	1.006	6	9794	ZINC000004899516
0.1	-7.5	-7.3	0.08	2.019	0.259	5.173	1.468	6	9795	ZINC000036532621
0.1	-4.9	-4.7	0.13	2.773	1.522	4.085	1.578	6	9796	ZINC000000895282
0.1	-7.4	-7.2	0.13	2.314	0.780	4.329	1.389	6	9797	ZINC000014590631
0.1	-8.3	-7.9	0.33	2.806	0.561	5.777	1.147	6	9798	ZINC000085589828
0.1	-9.7	-8.7	0.60	1.815	0.577	4.301	1.865	6	9799	ZINC000257537734
0.1	-3.5	-3.5	0.05	2.673	1.057	3.302	1.010	6	9800	ZINC000002560605
0.1	-6.4	-6.2	0.12	2.344	0.538	3.670	1.034	6	9801	ZINC000002555108
0.1	-8.3	-8.1	0.14	2.788	0.263	4.725	1.142	6	9802	ZINC000000895154
0.1	-6.3	-6.1	0.12	1.804	0.046	2.745	0.402	6	9803	ZINC000095618208

0.1	-4.3	-4.2	0.08	3.753	2.508	4.136	2.476	6	9804	ZINC000003861771
0.1	-9.8	-8.2	0.83	2.212	0.806	4.299	1.953	5	9805	ZINC000012496218
0.1	-9.1	-8.5	0.41	1.597	0.238	4.150	1.247	6	9806	ZINC000015220877
0.1	-4.9	-4.7	0.15	2.127	0.487	2.967	0.724	6	9807	ZINC000001641013
0.1	-4.0	-3.9	0.07	3.156	2.214	3.897	2.113	6	9808	ZINC000001532517
0.1	-6.6	-6.4	0.11	2.101	0.894	3.370	0.676	6	9809	ZINC000001856991
0.1	-9.1	-8.6	0.33	1.583	0.238	4.122	1.292	6	9810	ZINC000257378314
0.1	-5.8	-5.6	0.16	3.024	1.102	5.043	0.513	6	9811	ZINC000005134442
0.1	-6.8	-6.6	0.09	2.305	0.454	4.447	1.159	6	9812	ZINC000001576486
0.1	-6.6	-6.5	0.09	4.422	0.738	5.586	0.552	6	9813	ZINC000017783685
0.1	-6.7	-6.4	0.17	2.159	0.408	3.424	1.436	6	9814	ZINC000040439720
0.1	-6.6	-6.3	0.17	1.605	0.511	3.788	1.271	6	9815	ZINC000004726901
0.1	-9.0	-8.6	0.31	1.620	0.327	3.743	0.550	6	9816	ZINC000014504724
0.1	-6.3	-6.1	0.16	2.026	0.243	4.087	0.913	6	9817	ZINC000014511116
0.1	-7.9	-7.5	0.21	1.941	0.576	4.645	2.289	6	9818	ZINC000032821807

0.1	-8.7	-8.5	0.15	2.315	0.156	5.201	1.105	6	9819	ZINC000013482914
0.1	-9.7	-8.5	0.64	1.968	0.440	5.168	1.084	6	9820	ZINC000257537732
0.1	-4.2	-3.9	0.16	4.990	2.717	5.815	2.539	6	9821	ZINC000000895342
0.1	-5.7	-5.5	0.18	3.580	1.228	4.599	1.375	6	9822	ZINC000085587804
0.1	-7.6	-7.1	0.24	1.852	0.507	3.688	0.671	6	9823	ZINC000230080447
0.1	-7.4	-7.0	0.22	2.066	0.218	3.638	1.336	6	9824	ZINC000014494581
0.1	-7.1	-6.6	0.25	1.980	0.137	3.964	1.342	6	9825	ZINC000015122228
0.1	-7.0	-6.7	0.25	2.712	0.185	5.418	0.162	6	9826	ZINC000014440792
0.1	-2.9	-2.6	0.13	3.195	1.339	3.607	1.342	6	9827	ZINC000100083921
0.1	-6.6	-6.4	0.15	1.113	0.125	4.010	0.879	6	9828	ZINC000085972901
0.1	-7.3	-7.1	0.17	2.083	0.865	4.291	2.084	6	9829	ZINC000006072414
0.1	-9.4	-9.1	0.36	1.898	0.264	4.470	1.023	6	9830	ZINC000015057676
0.1	-7.7	-7.4	0.22	2.176	0.194	4.362	0.316	6	9831	ZINC000003913404
0.1	-5.5	-5.2	0.17	4.321	1.795	5.336	1.950	6	9832	ZINC000003869280
0.1	-6.8	-6.6	0.13	2.221	0.687	4.227	1.865	6	9833	ZINC000001529865

0.1	-7.7	-7.4	0.26	2.040	0.227	4.155	0.716	6	9834	ZINC000013377498
0.1	-9.0	-8.2	0.47	1.588	0.122	4.920	1.457	6	9835	ZINC000014588935
0.1	-6.1	-6.0	0.09	3.088	1.224	5.130	1.526	6	9836	ZINC000013538222
0.1	-8.6	-8.2	0.31	2.126	0.157	4.230	0.548	6	9837	ZINC000206507655
0.1	-6.8	-6.7	0.09	2.463	0.801	4.081	1.823	6	9838	ZINC000004533527
0.1	-7.2	-7.2	0.04	2.490	0.714	3.904	0.529	6	9839	ZINC000013460029
0.1	-6.2	-6.0	0.11	2.306	0.268	3.357	0.471	6	9840	ZINC000001622057
0.1	-5.4	-5.2	0.15	2.234	0.489	2.745	0.693	6	9841	ZINC000001529559
0.1	-7.6	-7.4	0.10	2.556	0.283	4.542	1.214	6	9842	ZINC000004762523
0.1	-6.9	-6.6	0.23	2.097	0.441	2.482	0.558	6	9843	ZINC000040471282
0.1	-6.4	-6.3	0.11	2.683	0.587	3.384	0.982	6	9844	ZINC000039626634
0.1	-6.2	-6.0	0.12	2.528	0.558	3.333	1.004	6	9845	ZINC000050026957
0.1	-8.8	-8.2	0.33	1.947	0.546	6.163	0.520	6	9846	ZINC000257423746
0.1	-9.0	-8.3	0.35	2.478	0.296	5.241	1.188	6	9847	ZINC000410428658
0.1	-7.0	-6.8	0.12	1.770	0.238	4.287	1.933	6	9848	ZINC000000402227

0.1	-6.8	-6.6	0.16	2.349	0.392	4.264	0.445	6	9849	ZINC000050027753
0.1	-4.4	-4.0	0.27	2.091	0.420	2.574	0.432	6	9850	ZINC000001867147
0.1	-6.1	-5.9	0.21	2.943	0.977	4.224	1.184	6	9851	ZINC000001529210
0.1	-5.7	-5.6	0.07	3.087	1.045	4.041	1.011	6	9852	ZINC000038645381
0.1	-6.6	-6.3	0.18	2.299	0.634	3.664	1.317	6	9853	ZINC000034371742
0.1	-7.4	-7.2	0.12	2.124	0.380	3.678	0.867	6	9854	ZINC000000406940
0.1	-4.6	-4.4	0.12	3.441	2.113	4.316	2.095	6	9855	ZINC000001591795
0.1	-7.9	-7.4	0.43	2.287	0.358	4.978	0.207	6	9856	ZINC000004534014
0.1	-6.8	-6.5	0.22	2.569	0.108	4.588	0.336	6	9857	ZINC000003791059
0.1	-5.7	-5.6	0.10	2.119	0.572	2.932	0.584	6	9858	ZINC000000897141
0.1	-7.4	-7.1	0.33	1.956	0.188	2.952	0.401	6	9859	ZINC000005442556
0.1	-4.5	-4.4	0.05	1.518	0.776	3.257	0.641	6	9860	ZINC000001648177
0.1	-6.4	-6.2	0.17	3.062	0.380	3.987	0.648	6	9861	ZINC000001529432
0.1	-4.8	-4.6	0.11	2.800	0.820	3.291	1.011	6	9862	ZINC000001532654
0.1	-7.3	-7.1	0.10	1.734	0.417	2.994	0.959	6	9863	ZINC000012402948

0.1	-7.0	-6.9	0.14	1.496	0.807	3.018	0.531	6	9864	ZINC000004096145
0.1	-6.4	-6.1	0.18	3.324	0.795	5.120	0.651	6	9865	ZINC000003869234
0.1	-7.6	-7.4	0.21	1.922	0.157	2.949	0.572	6	9866	ZINC000040484033
0.1	-9.7	-8.4	0.77	2.374	0.570	4.889	1.434	6	9867	ZINC000100777988
0.1	-6.0	-5.8	0.14	1.895	0.348	2.392	0.403	6	9868	ZINC000013549714
0.1	-8.8	-8.6	0.11	0.896	0.158	4.095	0.659	6	9869	ZINC000100778617
0.1	-7.8	-7.6	0.15	1.707	0.242	2.950	0.757	6	9870	ZINC000014444616
0.1	-9.0	-8.6	0.29	2.181	0.832	3.984	0.739	6	9871	ZINC000014493341
0.1	-8.2	-7.4	0.42	2.476	0.238	4.693	1.044	6	9872	ZINC000040164494
0.1	-9.7	-8.3	0.90	1.838	0.503	4.529	1.438	6	9873	ZINC000014442550
0.1	-8.2	-7.9	0.17	1.721	0.175	3.117	1.061	6	9874	ZINC000014682708
0.1	-6.7	-6.4	0.24	2.279	0.551	4.611	1.739	6	9875	ZINC000085994845
0.1	-9.0	-8.7	0.15	1.803	0.308	3.378	1.458	6	9876	ZINC000013570997
0.1	-5.6	-5.5	0.05	3.535	1.995	5.570	1.930	6	9877	ZINC000000000882
0.1	-6.8	-6.6	0.15	1.969	0.205	4.340	1.638	6	9878	ZINC000032839138

0.1	-7.2	-7.0	0.10	2.493	0.623	4.442	1.098	6	9879	ZINC000003869415
0.1	-8.1	-7.8	0.15	2.041	0.553	4.956	0.984	6	9880	ZINC000015120294
0.1	-5.3	-5.0	0.22	1.795	0.600	2.605	0.694	6	9881	ZINC000002034318
0.1	-5.8	-5.7	0.10	2.060	0.536	3.063	0.409	6	9882	ZINC000001693358
0.1	-9.1	-8.6	0.35	1.586	0.241	4.122	1.294	6	9883	ZINC000085767708
0.1	-8.2	-8.0	0.21	1.918	0.176	4.293	0.532	6	9884	ZINC000257385230
0.1	-6.0	-5.8	0.10	1.752	0.219	3.295	0.674	6	9885	ZINC000014589066
0.1	-6.5	-6.3	0.20	2.294	0.379	3.636	1.021	6	9886	ZINC000014504593
0.1	-7.4	-7.2	0.11	1.331	0.387	3.311	1.640	6	9887	ZINC000011681044
0.1	-9.3	-8.7	0.32	2.079	0.435	3.722	1.494	6	9888	ZINC000035051050
0.1	-9.2	-8.1	0.63	1.982	0.309	5.249	0.397	6	9889	ZINC000257525713
0.1	-6.2	-6.1	0.05	3.117	1.216	3.991	1.755	6	9890	ZINC000003869627
0.1	-8.4	-8.0	0.27	1.888	0.425	4.316	0.464	6	9891	ZINC000013408247
0.1	-9.4	-9.1	0.28	1.908	0.266	4.327	0.826	6	9892	ZINC000257419895
0.1	-8.8	-8.1	0.50	2.257	0.505	4.786	1.134	6	9893	ZINC000257554797

0.1	-7.9	-7.6	0.15	1.220	0.127	4.584	0.864	6	9894	ZINC000082156465
0.1	-5.1	-5.0	0.15	2.013	0.624	2.638	0.655	6	9895	ZINC000000388314
0.1	-7.1	-7.0	0.10	2.000	0.842	5.054	1.015	6	9896	ZINC000034334813
0.1	-9.1	-8.7	0.25	1.759	0.299	4.639	1.070	6	9897	ZINC000253500652
0.1	-6.5	-6.2	0.21	2.547	0.657	4.184	0.840	6	9898	ZINC000000056655
0.1	-8.4	-8.1	0.26	2.085	0.700	4.047	0.502	6	9899	ZINC000014651125
0.1	-8.7	-7.9	0.41	1.925	0.234	5.108	1.566	6	9900	ZINC000257647445
0.1	-9.3	-8.3	0.73	1.715	0.453	4.988	1.368	6	9901	ZINC000015119966
0.1	-9.5	-8.2	0.93	1.897	0.326	5.002	1.372	3	9902	ZINC000014635135
0.1	-8.1	-7.8	0.25	2.156	0.451	3.387	1.387	6	9903	ZINC000015261555
0.1	-7.0	-6.8	0.09	2.064	0.297	3.222	0.990	6	9904	ZINC000040471221
0.1	-7.7	-7.4	0.23	2.121	0.623	3.475	1.576	6	9905	ZINC000005863166
0.1	-3.6	-3.5	0.10	2.033	0.832	2.617	0.858	6	9906	ZINC000001691362
0.1	-8.2	-7.5	0.36	1.845	0.524	5.220	2.437	6	9907	ZINC000257398698
0.1	-8.7	-7.7	0.54	2.343	0.219	4.682	0.602	6	9908	ZINC000013311890

0.1	-5.5	-5.4	0.11	2.628	0.650	3.125	0.696	6	9909	ZINC000000001706
0.1	-8.8	-8.6	0.13	0.858	0.125	3.844	0.924	6	9910	ZINC000070454250
0.1	-9.6	-8.1	0.77	2.194	0.514	6.409	0.672	5	9911	ZINC000000900047
0.1	-6.3	-6.1	0.12	2.410	0.606	4.055	0.874	6	9912	ZINC000005274103
0.1	-5.9	-5.8	0.13	1.929	0.518	2.903	0.313	6	9913	ZINC000001090557
0.1	-6.1	-5.9	0.12	1.914	0.184	4.429	0.507	6	9914	ZINC000002522616
0.1	-6.5	-6.2	0.19	1.942	0.819	3.347	1.135	6	9915	ZINC000001532547
0.1	-7.1	-6.7	0.20	2.061	0.237	4.938	1.312	6	9916	ZINC000014722314
0.1	-6.3	-6.2	0.09	2.287	0.817	3.445	0.498	6	9917	ZINC000002011247
0.1	-6.2	-6.0	0.18	2.158	0.140	3.256	0.717	6	9918	ZINC000001575517
0.1	-6.9	-6.8	0.10	2.131	0.310	4.172	0.681	6	9919	ZINC000255983262
0.1	-5.5	-5.0	0.31	3.046	0.983	3.987	1.306	6	9920	ZINC000003599047
0.1	-8.6	-8.3	0.32	2.504	0.629	4.556	1.705	6	9921	ZINC000014651036
0.1	-7.6	-7.1	0.32	2.887	1.132	3.721	1.203	6	9922	ZINC000001997119
0.1	-5.0	-4.9	0.08	1.785	0.556	4.220	0.830	6	9923	ZINC000015119534

0.1	-5.0	-4.8	0.11	2.440	1.137	2.693	1.347	6	9924	ZINC000013480247
0.1	-8.3	-7.8	0.46	2.604	0.871	4.274	1.772	6	9925	ZINC000013522804
0.1	-10.3	-10.0	0.31	1.905	0.922	4.961	1.238	6	9926	ZINC000014857211
0.1	-9.5	-8.7	0.45	2.002	0.345	5.527	2.227	6	9927	ZINC000014452716
0.1	-8.3	-8.0	0.20	1.721	0.703	4.090	0.974	6	9928	ZINC000031166410
0.1	-9.1	-7.4	1.19	1.439	0.355	3.318	1.520	6	9929	ZINC000005808603
0.1	-7.1	-7.0	0.11	2.610	0.272	4.899	0.968	6	9930	ZINC000040470490
0.1	-9.0	-7.9	0.60	1.812	0.355	4.673	1.128	6	9931	ZINC000015216880
0.1	-7.6	-7.4	0.17	2.426	0.342	3.073	0.718	6	9932	ZINC000095617741
0.1	-9.4	-9.2	0.21	1.677	0.212	3.837	1.445	6	9933	ZINC000015057678
0.1	-8.0	-7.7	0.20	2.265	0.462	4.140	1.316	6	9934	ZINC000004762519
0.1	-9.5	-8.5	0.43	2.154	0.250	6.125	1.922	6	9935	ZINC000100776925
0.1	-9.9	-8.8	0.54	1.532	0.346	4.692	1.637	6	9936	ZINC000000402946
0.1	-4.4	-4.4	0.05	1.642	0.256	2.249	0.461	6	9937	ZINC000001482107
0.1	-5.8	-5.6	0.21	2.349	0.880	3.377	0.744	6	9938	ZINC000000898976

0.1	-8.9	-8.7	0.17	2.849	0.548	4.759	1.528	6	9939	ZINC000004899457
0.1	-5.3	-5.2	0.09	1.757	0.556	2.718	0.602	6	9940	ZINC000014616895
0.1	-9.6	-8.5	0.84	1.840	0.519	4.827	1.322	6	9941	ZINC000002039833
0.1	-6.8	-6.5	0.18	2.132	0.291	2.906	0.503	6	9942	ZINC000001605721
0.1	-6.8	-6.6	0.10	2.191	0.481	4.250	1.605	6	9943	ZINC000012496582
0.1	-9.0	-8.6	0.27	2.481	0.341	4.756	0.939	6	9944	ZINC000100037020
0.1	-6.9	-6.8	0.07	2.134	0.481	3.648	1.078	6	9945	ZINC000004095989
0.1	-8.0	-7.1	0.77	3.284	0.596	5.678	1.629	6	9946	ZINC000012501916
0.1	-7.1	-6.9	0.16	2.249	0.163	4.294	0.805	6	9947	ZINC000040487061
0.1	-9.3	-8.7	0.32	2.321	0.402	4.602	0.484	6	9948	ZINC000100051666
0.1	-6.0	-5.8	0.12	2.681	1.036	4.043	1.323	6	9949	ZINC000000391197
0.1	-7.4	-7.0	0.24	2.682	0.898	3.509	1.202	6	9950	ZINC000000043475
0.1	-5.0	-4.8	0.09	2.155	0.643	3.587	1.258	6	9951	ZINC000005509484
0.1	-3.9	-3.8	0.13	4.568	0.586	5.080	0.533	6	9952	ZINC000001591604
0.1	-6.0	-5.8	0.15	2.530	0.681	4.207	1.542	6	9953	ZINC000004096131

0.1	-6.9	-6.6	0.20	2.089	0.160	3.980	0.830	6	9954	ZINC000003953999
0.1	-10.4	-10.0	0.25	1.598	0.172	4.454	0.651	6	9955	ZINC000257749151
0.1	-6.8	-6.6	0.13	2.340	0.597	2.956	0.801	6	9956	ZINC000000008492
0.1	-6.5	-6.2	0.18	2.003	0.839	4.038	0.267	6	9957	ZINC000013551949
0.1	-3.8	-3.5	0.16	2.207	1.277	2.824	1.233	6	9958	ZINC000003639348
0.1	-6.6	-6.5	0.09	2.515	1.052	4.469	0.876	6	9959	ZINC000014439782
0.1	-7.0	-6.9	0.12	2.699	0.841	4.769	1.575	6	9960	ZINC000095618368
0.1	-7.7	-7.6	0.07	1.967	0.554	3.636	1.887	6	9961	ZINC000034022548
0.1	-5.9	-5.7	0.20	1.885	0.559	3.619	0.922	6	9962	ZINC000014419527
0.1	-5.5	-5.4	0.09	1.801	0.857	4.035	0.497	6	9963	ZINC000006037008
0.1	-9.1	-8.1	0.52	2.656	0.543	4.203	1.372	6	9964	ZINC000014612187
0.1	-7.8	-7.6	0.27	2.382	0.459	4.890	1.180	6	9965	ZINC000027210238
0.1	-6.4	-6.2	0.13	3.047	0.335	4.390	0.494	6	9966	ZINC000039626638
0.1	-6.5	-6.1	0.21	1.696	0.480	3.636	0.765	6	9967	ZINC000005132054
0.1	-6.4	-6.1	0.24	1.382	0.690	3.072	0.619	6	9968	ZINC000000157768

0.1	-8.3	-7.8	0.26	1.843	0.434	3.834	1.238	6	9969	ZINC000257384873
0.1	-9.0	-8.0	0.71	1.517	0.410	4.346	1.483	6	9970	ZINC000014927147
0.1	-7.7	-7.3	0.30	2.249	0.564	3.605	1.414	6	9971	ZINC000002384653
0.1	-7.8	-7.5	0.16	2.655	0.353	4.709	1.098	6	9972	ZINC000012495470
0.1	-6.8	-6.3	0.24	2.081	0.324	4.127	1.297	6	9973	ZINC000001572029
0.1	-5.5	-5.4	0.09	2.365	0.570	3.554	1.192	6	9974	ZINC000002385617
0.1	-9.2	-8.9	0.23	1.824	0.712	4.084	1.177	6	9975	ZINC000100775367
0.1	-8.2	-7.8	0.28	1.527	0.329	3.784	1.568	6	9976	ZINC000253498247
0.1	-8.8	-8.5	0.22	2.987	0.688	4.531	1.197	6	9977	ZINC000001637886
0.1	-6.0	-5.5	0.27	2.622	0.828	3.971	1.025	6	9978	ZINC000001529776
0.1	-6.6	-6.4	0.18	2.566	0.683	3.513	0.691	6	9979	ZINC000000001392
0.1	-8.3	-8.1	0.20	1.901	0.184	5.099	0.635	6	9980	ZINC000150373950
0.1	-5.9	-5.7	0.17	1.798	0.488	3.580	0.984	6	9981	ZINC000014419525
0.1	-4.1	-3.9	0.15	2.438	1.720	4.740	1.145	6	9982	ZINC000019230152
0.1	-6.6	-6.4	0.08	1.968	0.669	3.087	0.781	6	9983	ZINC000100821354

0.1	-8.3	-7.6	0.35	1.943	0.184	4.299	0.679	6	9984	ZINC000014828124
0.1	-9.4	-9.2	0.21	1.734	0.110	3.909	1.327	6	9985	ZINC000013321036
0.1	-5.7	-5.5	0.10	3.792	1.239	4.771	1.429	6	9986	ZINC000085587794
0.1	-5.8	-5.7	0.06	1.653	0.970	2.590	0.785	6	9987	ZINC000000896625
0.1	-6.6	-6.3	0.18	2.795	0.487	4.992	0.779	6	9988	ZINC000004556701
0.1	-6.7	-6.5	0.10	2.820	0.387	4.467	0.761	6	9989	ZINC000008577156
0.1	-7.0	-6.8	0.14	2.240	0.263	3.118	0.591	6	9990	ZINC000015261400
0.1	-5.6	-5.4	0.12	1.849	1.078	2.926	1.430	6	9991	ZINC000005822251
0.1	-6.6	-6.4	0.12	2.086	0.874	2.952	0.861	6	9992	ZINC000100053153
0.1	-6.5	-6.3	0.13	2.537	0.406	3.763	0.941	6	9993	ZINC000002564465
0.1	-9.4	-8.8	0.47	2.016	0.235	4.074	0.172	6	9994	ZINC000100772316
0.1	-8.3	-7.5	0.50	2.038	0.230	4.499	1.155	6	9995	ZINC000002015428
0.1	-6.7	-6.6	0.06	1.901	0.407	4.230	1.223	6	9996	ZINC000014502361
0.1	-8.3	-8.2	0.19	1.821	0.150	4.500	1.357	6	9997	ZINC000095620789
0.1	-7.9	-7.0	0.78	2.247	0.611	4.454	2.248	6	9998	ZINC000014642994

0.1	-6.7	-6.5	0.08	3.057	0.319	4.965	1.028	6	9999	ZINC000050027730
0.1	-7.4	-6.9	0.23	2.391	0.341	4.386	0.769	6	10000	ZINC000040484006
0.1	-9.4	-8.7	0.38	1.951	0.223	3.930	1.052	6	10001	ZINC000085996402
0.1	-8.0	-7.4	0.31	1.993	0.576	3.937	1.644	6	10002	ZINC000095618257
0.1	-5.7	-5.4	0.17	2.500	0.431	3.286	0.557	6	10003	ZINC000002526383
0.1	-5.1	-4.9	0.12	1.958	0.224	3.023	0.462	6	10004	ZINC000012358877
0.1	-8.4	-8.0	0.23	2.167	0.381	3.396	1.133	6	10005	ZINC000004095812
0.1	-9.0	-8.5	0.27	2.293	0.764	4.242	1.405	6	10006	ZINC000257510177
0.1	-9.4	-9.2	0.12	1.862	0.247	5.666	0.458	6	10007	ZINC000095619428
0.1	-8.3	-8.1	0.19	1.638	0.459	2.725	0.713	6	10008	ZINC000015044532
0.1	-6.2	-6.0	0.16	1.335	0.588	3.864	1.272	6	10009	ZINC000085972319
0.1	-6.8	-6.6	0.18	1.835	0.707	3.007	0.867	6	10010	ZINC000001672943
0.1	-8.3	-8.1	0.11	1.409	0.327	2.795	0.724	6	10011	ZINC000257469896
0.1	-5.4	-5.3	0.07	2.290	0.502	3.721	0.718	6	10012	ZINC000095618222
0.1	-8.7	-8.5	0.15	2.335	0.406	3.752	1.221	6	10013	ZINC000003204425

0.1	-5.5	-5.0	0.24	3.168	1.249	3.867	1.417	6	10014	ZINC000000902215
0.1	-6.4	-6.1	0.18	2.678	1.103	4.011	1.026	6	10015	ZINC000095620564
0.1	-9.4	-8.5	0.68	1.860	0.571	3.414	1.674	6	10016	ZINC000015155024
0.1	-9.1	-7.9	0.78	1.888	0.398	5.002	1.006	6	10017	ZINC000013408219
0.1	-5.8	-5.6	0.18	2.859	0.982	4.245	0.886	6	10018	ZINC000022115627
0.1	-6.8	-6.6	0.12	2.741	0.318	4.353	0.775	6	10019	ZINC000002043137
0.1	-7.7	-7.5	0.17	1.694	0.284	3.136	1.016	6	10020	ZINC000014616807
0.1	-4.2	-4.1	0.07	2.549	0.572	3.523	0.613	6	10021	ZINC000000409240
0.1	-7.0	-6.8	0.14	2.403	0.373	4.316	0.747	6	10022	ZINC000005274090
0.1	-8.8	-8.6	0.13	0.912	0.172	4.104	0.654	6	10023	ZINC000100778625
0.1	-5.9	-5.7	0.11	2.741	0.532	4.259	0.418	6	10024	ZINC000001575505
0.1	-5.6	-5.4	0.18	2.384	0.566	3.259	0.444	6	10025	ZINC000002584579
0.1	-5.0	-4.9	0.10	3.886	1.235	4.655	1.322	6	10026	ZINC000001529567
0.1	-7.4	-7.2	0.11	1.734	0.273	3.878	1.494	6	10027	ZINC000011681040
0.1	-6.1	-5.9	0.14	3.084	1.130	4.469	1.635	6	10028	ZINC000001578624

0.1	-6.5	-6.4	0.07	2.073	0.140	3.607	0.602	6	10029	ZINC000006032099
0.1	-7.5	-7.1	0.19	2.518	0.548	3.790	0.668	6	10030	ZINC000085949359
0.1	-5.9	-5.7	0.07	3.251	1.281	4.760	1.398	6	10031	ZINC000012493577
0.1	-6.2	-5.9	0.14	2.142	0.687	3.182	0.732	6	10032	ZINC000006068630
0.1	-7.4	-7.3	0.06	1.511	0.208	4.260	2.432	6	10033	ZINC000255970899
0.1	-8.2	-7.9	0.16	2.027	0.580	4.591	1.384	6	10034	ZINC000257637217
0.1	-8.5	-8.2	0.22	3.232	0.808	5.243	0.615	6	10035	ZINC000034349836
0.1	-7.4	-7.1	0.20	3.127	0.170	6.514	0.116	6	10036	ZINC000004096415
0.1	-7.1	-7.0	0.14	2.060	0.135	3.908	1.606	6	10037	ZINC000001671375
0.1	-7.1	-6.7	0.28	2.434	1.090	3.719	1.667	6	10038	ZINC000000402814
0.1	-7.7	-7.7	0.05	1.453	0.203	4.550	1.868	6	10039	ZINC000085795011
0.1	-8.8	-8.3	0.31	1.332	0.091	6.476	0.137	6	10040	ZINC000015251270
0.1	-9.1	-8.7	0.36	2.621	0.728	4.959	0.652	6	10041	ZINC000013282986
0.1	-5.6	-5.2	0.21	2.774	1.582	3.940	1.563	6	10042	ZINC000001529420
0.1	-8.5	-7.8	0.39	2.427	0.458	4.589	1.036	6	10043	ZINC000014687091

0.1	-6.5	-6.1	0.22	2.115	1.059	3.711	0.798	6	10044	ZINC000001529351
0.1	-5.1	-5.0	0.07	2.377	1.248	3.798	1.350	6	10045	ZINC000000154729
0.1	-5.9	-5.6	0.20	2.635	0.690	3.498	0.605	6	10046	ZINC000012358763
0.1	-7.5	-7.2	0.22	2.070	0.337	5.641	1.115	6	10047	ZINC000100820614
0.1	-7.2	-7.0	0.12	2.780	0.815	4.051	0.976	6	10048	ZINC000005783565
0.1	-3.9	-3.7	0.12	2.623	0.814	2.682	0.926	6	10049	ZINC000095619739
0.1	-9.2	-8.5	0.37	1.572	0.465	2.543	0.600	6	10050	ZINC000013409173
0.1	-6.3	-6.3	0.05	2.505	0.529	4.778	1.670	6	10051	ZINC000005500865
0.1	-4.2	-4.1	0.11	1.668	0.470	2.741	0.361	6	10052	ZINC000000388479
0.1	-3.2	-3.2	0.05	1.074	0.705	2.816	0.334	6	10053	ZINC000003860808
0.1	-9.3	-8.8	0.27	1.972	0.545	4.001	0.536	6	10054	ZINC000100780727
0.1	-5.6	-5.3	0.20	3.291	1.384	4.092	1.355	6	10055	ZINC000004087886
0.1	-5.7	-5.4	0.17	2.434	0.418	3.399	0.701	6	10056	ZINC000014419734
0.1	-8.2	-8.0	0.11	1.722	0.425	4.033	1.025	6	10057	ZINC000257752822
0.1	-8.6	-8.2	0.22	2.674	0.606	5.155	1.572	6	10058	ZINC000034349834

0.1	-9.1	-8.6	0.38	2.127	0.783	2.836	0.723	6	10059	ZINC000014728401
0.1	-8.1	-7.6	0.25	2.532	0.495	5.115	1.968	6	10060	ZINC000013461546
0.1	-6.9	-6.7	0.11	3.008	0.508	5.466	0.512	6	10061	ZINC000083409143
0.1	-4.5	-4.2	0.20	3.625	1.592	4.098	1.630	6	10062	ZINC000034689286
0.1	-5.4	-5.3	0.09	2.594	0.635	3.252	0.554	6	10063	ZINC000030728630
0.1	-6.1	-5.9	0.11	3.964	0.408	5.225	0.767	6	10064	ZINC000000402896
0.1	-6.8	-6.6	0.15	2.075	0.208	4.257	0.948	6	10065	ZINC000008419115
0.1	-4.5	-4.2	0.13	3.452	0.913	4.246	0.582	6	10066	ZINC000003861345
0.1	-8.1	-7.8	0.21	2.593	0.231	4.643	0.857	6	10067	ZINC000100051673
0.1	-6.5	-6.1	0.21	2.610	0.428	4.669	0.414	6	10068	ZINC000013347347
0.1	-6.6	-6.3	0.20	2.530	0.465	2.894	0.542	6	10069	ZINC000013480031
0.1	-8.0	-7.8	0.18	2.265	0.458	4.075	1.013	6	10070	ZINC000040439645
0.1	-8.7	-7.8	0.59	2.280	0.784	3.973	2.002	6	10071	ZINC000017141861
0.1	-8.1	-7.9	0.20	2.558	0.568	4.306	1.567	6	10072	ZINC000095617646
0.1	-5.3	-5.1	0.15	1.650	0.228	2.410	0.291	6	10073	ZINC000005922661

0.1	-7.7	-7.4	0.15	1.551	0.394	3.740	0.938	6	10074	ZINC000001849759
0.1	-5.3	-4.8	0.24	2.639	1.859	3.796	1.662	6	10075	ZINC000013459854
0.1	-5.2	-5.1	0.09	3.295	0.927	4.496	0.939	6	10076	ZINC000004095591
0.1	-8.2	-7.5	0.30	2.008	0.539	5.307	2.318	6	10077	ZINC000257398700
0.1	-6.6	-6.3	0.17	1.661	0.338	3.468	1.425	6	10078	ZINC000012504391
0.1	-6.1	-5.7	0.22	2.356	0.533	3.845	0.798	6	10079	ZINC000030725382
0.1	-7.7	-7.4	0.18	1.825	0.193	4.077	1.025	6	10080	ZINC000100822075
0.1	-8.1	-7.5	0.36	2.009	0.222	5.795	0.286	6	10081	ZINC000085994848
0.1	-5.8	-5.6	0.11	3.001	1.097	4.121	0.616	6	10082	ZINC000005134440
0.1	-4.2	-4.1	0.15	2.464	0.296	3.078	0.479	6	10083	ZINC000059217448
0.1	-8.9	-7.9	0.66	1.817	0.070	4.547	0.353	6	10084	ZINC000015059549
0.1	-5.8	-5.7	0.11	2.694	0.544	3.725	1.132	6	10085	ZINC000006119078
0.1	-9.2	-8.2	0.86	1.549	0.678	2.958	1.186	5	10086	ZINC000257590906
0.1	-9.7	-8.4	0.76	1.897	0.504	5.229	1.125	6	10087	ZINC000257537733
0.1	-7.0	-6.9	0.05	2.703	0.396	4.365	1.234	6	10088	ZINC000002560992

0.1	-4.5	-4.3	0.09	2.930	1.836	4.126	1.846	6	10089	ZINC000003593481
0.1	-8.7	-8.0	0.43	1.917	0.362	3.671	1.413	6	10090	ZINC000004521568
0.1	-4.4	-4.1	0.18	3.481	1.446	4.454	1.475	6	10091	ZINC000015043929
0.1	-8.5	-8.0	0.32	1.830	0.346	4.216	1.205	6	10092	ZINC000257528424
0.1	-10.5	-8.8	1.07	1.964	0.446	4.483	0.624	6	10093	ZINC000257515094
0.1	-4.2	-4.0	0.13	1.909	0.524	3.058	0.183	6	10094	ZINC000014592620
0.1	-4.7	-4.5	0.15	1.030	0.209	2.510	0.220	6	10095	ZINC000001680680
0.1	-8.4	-8.1	0.27	2.634	0.371	4.673	1.124	6	10096	ZINC000002575496
0.1	-5.8	-5.7	0.07	3.007	0.937	3.851	1.190	6	10097	ZINC000006090913
0.1	-6.4	-6.2	0.15	2.691	0.234	3.891	0.777	6	10098	ZINC000002504612
0.1	-7.4	-7.2	0.13	2.015	0.592	4.048	0.814	6	10099	ZINC000002384576
0.1	-7.1	-6.8	0.20	3.723	0.187	5.554	0.508	6	10100	ZINC000006091875
0.1	-7.0	-6.9	0.07	2.544	0.902	3.638	1.760	6	10101	ZINC000017969422
0.1	-9.0	-8.6	0.25	2.014	0.920	3.569	1.075	6	10102	ZINC000257510176
0.1	-7.8	-7.6	0.18	2.583	0.420	4.379	1.479	6	10103	ZINC000004787349

0.1	-9.5	-8.2	0.78	1.581	0.238	4.270	1.716	6	10104	ZINC000004534074
0.1	-6.0	-5.9	0.06	2.674	0.540	3.582	0.962	6	10105	ZINC000000164388
0.1	-9.5	-7.7	1.30	1.514	0.065	3.844	0.026	3	10106	ZINC000067912650
0.1	-9.0	-8.4	0.31	1.797	0.261	3.383	1.390	6	10107	ZINC000014497823
0.1	-9.1	-8.7	0.19	1.719	0.248	4.837	0.910	6	10108	ZINC000013660229
0.1	-10.4	-9.8	0.30	1.947	0.371	4.328	0.591	6	10109	ZINC000257749160
0.1	-8.4	-7.4	0.68	1.802	0.092	5.200	1.078	6	10110	ZINC000014771154
0.1	-9.6	-8.3	0.71	1.908	0.201	4.401	1.757	6	10111	ZINC000000899790
0.1	-6.7	-6.3	0.17	2.578	0.260	4.707	1.117	6	10112	ZINC000034893635
0.1	-9.1	-8.5	0.37	1.575	0.242	4.149	1.238	6	10113	ZINC000015220875
0.1	-6.5	-6.1	0.22	1.436	0.326	3.753	0.690	6	10114	ZINC000013551953
0.1	-4.7	-4.3	0.22	2.146	0.455	2.654	0.619	6	10115	ZINC000001592388
0.1	-5.7	-5.6	0.16	1.979	0.688	3.588	0.837	6	10116	ZINC000001848542
0.1	-4.8	-4.7	0.12	3.359	0.461	4.103	0.364	6	10117	ZINC000001668234
0.1	-9.4	-8.6	0.49	1.821	0.452	4.039	1.632	6	10118	ZINC000014779276

0.1	-5.8	-5.7	0.04	2.124	0.335	3.433	0.796	6	10119	ZINC000005514846
0.1	-6.3	-6.0	0.16	2.961	0.497	3.805	0.900	6	10120	ZINC000000388198
0.1	-7.6	-7.1	0.25	1.851	0.532	3.178	0.811	6	10121	ZINC000100823538
0.1	-7.0	-6.6	0.22	3.114	0.255	4.356	0.687	6	10122	ZINC000002559033
0.1	-4.8	-4.6	0.14	2.316	0.352	2.863	0.629	6	10123	ZINC000004342579
0.1	-8.3	-7.7	0.47	1.488	0.270	3.613	1.482	6	10124	ZINC000257592652
0.1	-9.8	-9.3	0.33	1.443	0.327	4.154	1.216	6	10125	ZINC000100779654
0.1	-7.0	-6.9	0.11	2.636	0.445	4.347	0.971	6	10126	ZINC000002384845
0.1	-8.8	-8.2	0.35	1.810	0.152	6.047	0.838	6	10127	ZINC000257514618
0.1	-6.5	-6.4	0.08	1.927	0.505	4.712	1.531	6	10128	ZINC000085589596
0.1	-5.3	-5.1	0.11	2.639	1.503	3.585	1.612	6	10129	ZINC000014614330
0.1	-6.6	-6.5	0.09	2.513	0.531	3.816	1.265	6	10130	ZINC000015261521
0.1	-6.3	-6.1	0.12	2.467	0.543	3.848	0.906	6	10131	ZINC000005273807
0.1	-6.8	-6.6	0.18	1.954	0.669	3.479	1.780	6	10132	ZINC000001605720
0.1	-8.4	-7.8	0.41	1.710	0.246	3.218	0.965	6	10133	ZINC000257454433

0.1	-3.3	-3.2	0.11	1.547	0.332	2.128	0.452	6	10134	ZINC000002039466
0.1	-8.7	-8.3	0.41	1.935	0.929	4.456	2.188	6	10135	ZINC000018847051
0.1	-8.3	-7.1	0.90	2.528	0.397	4.982	1.369	6	10136	ZINC000138792825
0.1	-6.5	-6.3	0.16	2.670	0.728	4.225	1.457	6	10137	ZINC000004556854
0.1	-6.9	-6.8	0.10	2.127	0.343	4.177	0.663	6	10138	ZINC000100825935
0.1	-7.4	-7.2	0.11	2.413	0.247	3.680	0.967	6	10139	ZINC000013387648
0.1	-6.6	-6.3	0.16	1.867	0.571	4.170	1.303	6	10140	ZINC000053683563
0.1	-6.9	-6.7	0.19	2.352	0.336	4.463	1.115	6	10141	ZINC000095617815
0.1	-10.3	-10.0	0.25	1.889	0.938	4.885	1.217	6	10142	ZINC000257409225
0.1	-5.5	-5.3	0.17	2.997	0.742	4.503	1.027	6	10143	ZINC000001605653
0.1	-4.2	-4.0	0.13	2.742	2.037	3.293	2.057	6	10144	ZINC000034676244
0.1	-6.2	-6.1	0.06	2.987	0.758	3.983	0.423	6	10145	ZINC000095618075
0.1	-7.0	-6.6	0.24	1.788	0.592	4.452	1.381	6	10146	ZINC000000402228
0.1	-8.8	-8.4	0.37	1.320	0.776	4.533	1.917	6	10147	ZINC000015251264
0.1	-9.0	-8.6	0.25	2.012	0.913	3.570	1.077	6	10148	ZINC000257510179

0.1	-9.2	-8.7	0.52	1.586	0.247	4.409	1.298	6	10149	ZINC000085884002
0.1	-7.0	-6.8	0.24	0.704	0.854	4.916	1.658	6	10150	ZINC000100016166
0.1	-5.9	-5.7	0.17	1.896	0.441	3.922	0.736	6	10151	ZINC000003982716
0.1	-6.8	-6.3	0.24	3.009	0.261	4.891	0.901	6	10152	ZINC000014504596
0.1	-7.5	-7.1	0.22	1.833	0.248	4.368	0.874	6	10153	ZINC000002018831
0.1	-5.1	-5.0	0.04	2.075	0.762	3.865	1.310	6	10154	ZINC000146944175
0.1	-7.1	-6.9	0.16	2.870	0.196	5.375	0.230	6	10155	ZINC000000895199
0.1	-6.9	-6.7	0.15	2.536	0.221	4.794	0.564	6	10156	ZINC000050027701
0.1	-5.2	-5.1	0.07	1.964	0.446	3.565	0.208	6	10157	ZINC000002031579
0.1	-6.5	-6.4	0.07	1.870	0.272	4.797	1.380	6	10158	ZINC000005160364
0.1	-5.0	-4.8	0.11	2.623	0.666	3.177	0.726	6	10159	ZINC000000160790
0.1	-5.0	-4.9	0.11	3.188	1.513	4.004	1.619	6	10160	ZINC000012953444
0.1	-6.7	-6.4	0.26	2.287	0.595	2.529	0.602	6	10161	ZINC000002581983
0.1	-9.3	-8.6	0.42	1.891	0.538	5.367	1.198	6	10162	ZINC000006032238
0.1	-7.8	-7.4	0.21	1.816	0.255	3.642	0.962	6	10163	ZINC000013481842

0.1	-8.2	-7.4	0.53	1.838	0.228	5.240	1.193	6	10164	ZINC000014776081
0.1	-3.3	-3.0	0.18	12.855	6.943	13.199	6.825	6	10165	ZINC000008383199
0.1	-6.6	-6.3	0.15	1.713	0.275	4.073	1.212	6	10166	ZINC000238738426
0.1	-10.4	-9.0	0.70	1.578	0.255	5.201	1.184	6	10167	ZINC000003807917
0.1	-4.4	-4.2	0.19	1.166	0.427	2.047	0.168	6	10168	ZINC000001481970
0.1	-6.3	-6.3	0.07	2.764	0.287	4.316	0.741	6	10169	ZINC000013304791
0.1	-6.1	-6.0	0.09	3.081	1.217	5.126	1.524	6	10170	ZINC000013538228
0.1	-6.4	-6.3	0.19	2.620	0.310	3.643	0.529	6	10171	ZINC000000113355
0.1	-9.1	-7.9	0.65	1.907	0.256	4.862	0.441	6	10172	ZINC000013429609
0.1	-6.3	-6.1	0.11	2.345	0.347	4.202	0.878	6	10173	ZINC000040471831
0.1	-8.9	-8.2	0.50	1.679	0.235	4.740	1.506	6	10174	ZINC000257551983
0.1	-7.3	-7.1	0.15	1.394	0.380	3.935	2.101	6	10175	ZINC000015113336
0.1	-5.7	-5.5	0.11	4.074	1.277	5.133	1.501	6	10176	ZINC000085587797
0.1	-8.5	-8.3	0.13	3.320	0.416	4.887	1.005	6	10177	ZINC000000037833
0.1	-5.8	-5.6	0.13	2.689	0.199	4.563	0.744	6	10178	ZINC000001605260

0.1	-5.1	-5.0	0.10	2.629	0.174	3.379	0.511	6	10179	ZINC000012496497
0.1	-5.7	-5.6	0.09	1.836	0.583	2.812	0.497	6	10180	ZINC000100023849
0.1	-3.3	-3.0	0.16	4.057	1.950	4.579	1.852	6	10181	ZINC000000901159
0.1	-7.8	-7.5	0.42	2.490	0.291	6.137	0.658	6	10182	ZINC000003830507
0.1	-7.1	-6.9	0.13	2.587	0.478	4.704	1.276	6	10183	ZINC000095617763
0.1	-8.7	-8.0	0.44	2.442	0.566	3.685	1.243	6	10184	ZINC000014820476
0.1	-9.2	-7.9	0.84	1.446	0.189	3.208	0.472	6	10185	ZINC000014920779
0.1	-9.9	-9.5	0.18	2.068	0.532	3.712	1.500	6	10186	ZINC000100822033
0.1	-6.0	-5.9	0.08	2.678	0.831	4.380	1.278	6	10187	ZINC000002522704
0.1	-6.3	-6.2	0.05	1.237	0.625	3.399	1.177	6	10188	ZINC000003860720
0.1	-8.4	-8.1	0.29	1.394	0.369	2.381	0.593	6	10189	ZINC000100162755
0.1	-9.0	-8.3	0.35	1.718	0.144	3.225	0.862	6	10190	ZINC000006505382
0.1	-6.2	-6.0	0.17	2.629	0.457	3.948	0.984	6	10191	ZINC000013549711
0.1	-6.4	-6.2	0.11	2.142	0.789	3.856	1.371	6	10192	ZINC000085550138
0.1	-9.6	-9.0	0.54	1.678	0.204	4.699	1.137	3	10193	ZINC000014719978

0.1	-8.1	-7.6	0.24	2.807	0.174	5.814	0.356	6	10194	ZINC000027210246
0.1	-7.7	-7.4	0.18	1.814	0.524	3.845	1.234	6	10195	ZINC000257560781
0.1	-6.2	-6.1	0.08	1.567	1.001	4.707	0.946	6	10196	ZINC000014616899
0.1	-9.3	-8.2	0.64	1.610	0.201	3.145	1.630	6	10197	ZINC000014416219
0.1	-8.5	-8.2	0.26	2.164	0.592	4.491	1.408	6	10198	ZINC000015119941
0.1	-7.1	-6.9	0.15	1.975	0.609	4.532	1.215	6	10199	ZINC000000058258
0.1	-7.8	-7.3	0.25	2.174	0.443	3.610	0.990	6	10200	ZINC000001605725
0.1	-6.8	-6.6	0.13	3.121	0.223	5.625	0.374	6	10201	ZINC000005274092
0.1	-5.3	-5.1	0.13	3.669	1.875	4.522	1.924	6	10202	ZINC000014592916
0.1	-5.7	-5.4	0.23	2.999	1.500	4.314	1.391	6	10203	ZINC000005844826
0.1	-5.8	-5.5	0.13	3.461	0.598	4.540	0.856	6	10204	ZINC000006036732
0.1	-9.4	-8.4	0.62	2.130	0.286	4.721	1.498	6	10205	ZINC000105495172
0.1	-5.3	-5.0	0.16	2.858	0.623	3.852	0.741	6	10206	ZINC000001609510
0.1	-6.3	-6.2	0.07	3.175	0.596	4.851	0.648	6	10207	ZINC000039626636
0.1	-6.3	-6.2	0.08	2.114	0.816	4.010	0.963	6	10208	ZINC000000013246

0.1	-8.1	-7.9	0.22	2.251	0.460	4.471	0.613	6	10209	ZINC000257457541
0.1	-4.9	-4.7	0.14	3.440	1.474	4.728	1.388	6	10210	ZINC000004521480
0.1	-7.3	-7.1	0.13	1.452	0.378	2.537	0.630	6	10211	ZINC000001584050
0.1	-4.7	-4.7	0.05	2.777	0.880	3.700	1.253	6	10212	ZINC000031500668
0.1	-5.9	-5.8	0.14	2.962	1.140	4.372	2.013	6	10213	ZINC000004557149
0.1	-4.9	-4.5	0.25	2.794	0.857	3.402	0.777	6	10214	ZINC000001634091
0.1	-5.6	-5.3	0.17	3.002	0.470	4.545	0.881	6	10215	ZINC000001529886
0.1	-8.8	-7.9	0.48	1.655	0.373	3.317	1.551	6	10216	ZINC000014723721
0.1	-6.8	-6.6	0.21	2.490	0.366	6.078	0.326	6	10217	ZINC000014455517
0.1	-5.1	-5.0	0.04	2.535	0.839	4.529	0.341	6	10218	ZINC000261499700
0.1	-5.4	-5.2	0.13	2.442	0.522	3.991	1.246	6	10219	ZINC000001532464
0.1	-8.9	-8.5	0.30	2.096	0.175	6.371	0.908	6	10220	ZINC000014854261
0.1	-7.9	-7.5	0.40	1.800	0.262	4.451	1.381	6	10221	ZINC000012496397
0.1	-5.3	-5.2	0.11	1.732	0.568	4.690	0.587	6	10222	ZINC000014711017
0.1	-8.6	-8.1	0.29	2.622	0.688	4.180	1.037	6	10223	ZINC000034290099

0.1	-9.0	-8.7	0.21	1.694	0.362	3.794	0.458	6	10224	ZINC000014504729
0.1	-8.7	-8.4	0.19	1.042	0.318	4.962	1.897	6	10225	ZINC000100772448
0.1	-8.3	-8.1	0.24	2.007	0.456	5.044	0.877	6	10226	ZINC000014642687
0.1	-6.2	-6.0	0.15	1.647	1.180	3.776	1.606	6	10227	ZINC000000087933
0.1	-8.4	-8.1	0.19	1.979	0.055	5.532	0.382	6	10228	ZINC000100770533
0.1	-5.7	-5.5	0.15	3.249	0.740	4.985	0.984	6	10229	ZINC000000404262
0.1	-8.8	-8.2	0.35	2.046	0.641	4.357	1.946	6	10230	ZINC000257524671
0.1	-6.9	-6.7	0.13	1.994	0.699	2.879	1.132	6	10231	ZINC000004095540
0.1	-8.6	-7.8	0.48	2.173	0.282	5.089	0.723	6	10232	ZINC000013408223
0.1	-7.1	-6.9	0.13	2.249	0.437	3.590	0.806	6	10233	ZINC000003870237
0.1	-8.8	-8.4	0.36	2.671	0.712	4.293	0.967	6	10234	ZINC000085993647
0.1	-7.8	-7.4	0.27	2.334	0.182	4.740	1.273	6	10235	ZINC000085951547
0.1	-8.9	-8.7	0.21	1.842	0.316	3.459	1.871	6	10236	ZINC000015058106
0.1	-6.0	-6.0	0.04	2.731	0.487	3.871	0.943	6	10237	ZINC000015221890
0.1	-7.1	-6.9	0.24	2.292	0.430	4.258	0.725	6	10238	ZINC000050026772

0.1	-7.0	-6.8	0.19	1.808	1.119	2.808	1.357	6	10239	ZINC000000493113
0.1	-8.3	-7.9	0.29	2.390	0.211	4.369	0.502	6	10240	ZINC000034290103
0.1	-5.3	-5.1	0.11	1.930	0.521	2.831	0.927	6	10241	ZINC000005841334
0.1	-6.6	-6.4	0.11	2.195	0.398	3.894	0.727	6	10242	ZINC000002561137
0.1	-7.0	-6.7	0.14	2.179	0.528	3.118	1.728	6	10243	ZINC000095617776
0.1	-4.1	-4.0	0.08	2.154	1.163	2.979	1.234	6	10244	ZINC000001666987
0.1	-7.4	-7.0	0.30	2.237	0.417	3.335	1.522	6	10245	ZINC000000154442
0.1	-7.2	-6.9	0.17	2.178	0.516	3.651	0.571	6	10246	ZINC000001687400
0.1	-8.7	-8.4	0.20	2.792	0.308	5.248	0.355	6	10247	ZINC000000119988
0.1	-6.9	-6.8	0.11	2.037	0.314	4.163	0.684	6	10248	ZINC000100825934
0.1	-4.1	-3.9	0.09	2.071	0.929	3.171	0.628	6	10249	ZINC000014592621
0.1	-5.6	-5.4	0.08	3.029	1.901	4.098	1.482	6	10250	ZINC000002004458
0.1	-4.2	-4.0	0.17	2.699	1.652	3.360	1.795	6	10251	ZINC000003861336
0.1	-4.9	-4.6	0.14	3.836	2.291	4.177	2.495	6	10252	ZINC000001571334
0.1	-8.5	-7.9	0.45	1.891	0.492	2.939	1.115	6	10253	ZINC000206507623

0.1	-8.2	-7.7	0.34	1.522	0.374	3.781	1.418	6	10254	ZINC000253498243
0.1	-9.0	-8.8	0.15	1.821	0.171	4.933	1.337	6	10255	ZINC000014920378
0.1	-6.5	-6.2	0.18	1.696	0.501	3.658	0.758	6	10256	ZINC000000897426
0.1	-5.4	-5.2	0.18	3.151	0.701	3.899	1.023	6	10257	ZINC000014616382
0.1	-4.4	-4.2	0.18	1.866	0.380	2.902	0.640	6	10258	ZINC000000391792
0.1	-8.8	-8.5	0.15	2.179	0.165	5.242	0.375	6	10259	ZINC000028631193
0.1	-10.0	-8.1	1.34	1.974	0.036	5.738	0.494	3	10260	ZINC000100777424
0.1	-6.6	-6.5	0.15	2.253	0.464	2.861	0.715	6	10261	ZINC000040471440
0.1	-7.4	-6.6	0.38	1.569	0.238	6.519	1.217	6	10262	ZINC000014921700
0.1	-8.1	-7.6	0.29	2.174	1.299	3.404	2.163	6	10263	ZINC000001529675
0.1	-7.7	-7.3	0.26	2.061	0.252	3.057	0.319	6	10264	ZINC000033980259
0.1	-5.8	-5.6	0.11	3.123	1.266	5.054	0.523	6	10265	ZINC000005134644
0.1	-6.1	-5.9	0.11	3.681	0.080	4.448	0.140	6	10266	ZINC000000000920
0.1	-9.4	-9.1	0.27	2.165	0.406	5.274	1.603	6	10267	ZINC000015057680
0.1	-8.6	-8.4	0.21	1.957	0.255	4.970	0.684	6	10268	ZINC000013339777

0.1	-9.0	-7.6	1.40	2.029	0.000	4.341	0.000	2	10269	ZINC000014681161
0.1	-9.1	-7.9	0.97	1.659	0.446	4.800	0.238	6	10270	ZINC000014648557
0.1	-5.3	-5.2	0.09	1.829	0.535	3.068	0.576	6	10271	ZINC000000895339
0.1	-6.1	-5.8	0.25	1.757	0.501	2.572	0.539	6	10272	ZINC000012405046
0.1	-7.9	-7.2	0.43	1.739	0.297	5.227	0.823	6	10273	ZINC000014616273
0.1	-7.4	-6.8	0.30	1.511	0.193	5.686	1.755	6	10274	ZINC000014921703
0.1	-8.8	-8.4	0.35	1.597	0.870	4.695	1.938	6	10275	ZINC000015251267
0.1	-5.7	-5.4	0.16	2.865	0.764	3.628	0.906	6	10276	ZINC000005759723
0.1	-9.3	-8.8	0.23	1.897	0.836	4.828	1.571	6	10277	ZINC000257382461
0.1	-6.4	-6.3	0.07	2.566	0.384	5.319	0.866	6	10278	ZINC000005113904
0.1	-6.8	-6.5	0.15	2.230	0.911	2.869	1.311	6	10279	ZINC000000034162
0.1	-8.2	-7.8	0.33	2.159	0.939	3.622	2.158	6	10280	ZINC000013414507
0.1	-5.4	-5.2	0.11	1.990	0.696	2.646	0.486	6	10281	ZINC000000897131
0.1	-8.0	-7.7	0.21	2.591	0.472	5.710	0.571	6	10282	ZINC000032821825
0.1	-3.7	-3.6	0.12	2.643	2.109	3.569	2.051	6	10283	ZINC000003079337

0.1	-6.4	-6.3	0.11	1.949	0.352	4.464	0.494	6	10284	ZINC000256081491
0.1	-8.7	-8.2	0.53	2.130	0.399	5.363	1.164	6	10285	ZINC000000338222
0.1	-9.0	-8.5	0.29	2.251	0.719	3.821	0.973	6	10286	ZINC000100777224
0.1	-5.0	-4.8	0.12	2.152	1.097	3.121	1.158	6	10287	ZINC000014589727
0.1	-5.6	-5.5	0.07	1.836	0.431	3.678	0.552	6	10288	ZINC000100776435
0.1	-4.0	-3.9	0.07	2.614	0.620	3.292	0.668	6	10289	ZINC000032163928
0.1	-8.6	-8.1	0.33	1.768	0.537	3.166	1.698	6	10290	ZINC000014687443
0.1	-7.4	-7.1	0.20	2.599	0.671	4.501	1.682	6	10291	ZINC000006037912
0.1	-6.6	-6.3	0.15	1.533	0.549	3.979	1.256	6	10292	ZINC000012504389
0.1	-5.6	-5.3	0.16	2.667	1.770	3.440	1.773	6	10293	ZINC000000895235
0.1	-6.4	-6.1	0.16	3.128	0.755	5.196	1.552	6	10294	ZINC000001576325
0.1	-7.0	-6.9	0.10	2.384	0.495	3.729	0.639	6	10295	ZINC000000056653
0.1	-4.6	-4.6	0.05	2.158	0.384	3.211	0.651	6	10296	ZINC000005360197
0.1	-8.7	-8.3	0.24	1.695	0.519	4.604	1.196	6	10297	ZINC000014768312
0.1	-9.4	-7.5	0.92	1.621	0.156	4.217	0.963	6	10298	ZINC000100829808

0.1	-8.2	-7.5	0.37	1.976	0.556	5.423	2.141	6	10299	ZINC000257398701
0.1	-5.4	-5.3	0.07	2.200	0.232	2.944	0.295	6	10300	ZINC000030728634
0.1	-7.9	-7.8	0.08	2.213	0.508	4.293	0.702	6	10301	ZINC000014859830
0.1	-6.5	-6.2	0.22	1.307	0.310	3.904	0.730	6	10302	ZINC000004097479
0.1	-6.6	-6.3	0.16	1.726	0.265	4.095	1.176	6	10303	ZINC000004726904
0.1	-5.1	-5.0	0.09	3.632	1.677	5.081	1.535	6	10304	ZINC000003869231
0.1	-4.5	-4.4	0.07	2.368	0.781	3.276	0.759	6	10305	ZINC000032175845
0.1	-5.9	-5.7	0.12	2.474	0.160	4.130	0.443	6	10306	ZINC000039386843
0.1	-6.9	-6.6	0.15	2.909	0.592	4.659	1.022	6	10307	ZINC000001532770
0.1	-8.5	-8.3	0.17	1.920	0.282	4.071	0.836	6	10308	ZINC000015055181
0.1	-8.1	-8.0	0.07	2.234	0.278	3.764	0.227	6	10309	ZINC000004533517
0.1	-4.9	-4.8	0.07	2.791	0.617	3.631	0.800	6	10310	ZINC000014685162
0.1	-9.5	-8.6	0.42	1.887	0.573	5.190	2.351	6	10311	ZINC000257527916
0.1	-6.5	-6.3	0.15	1.865	0.945	3.484	0.319	6	10312	ZINC000257405994
0.1	-4.8	-4.5	0.17	2.033	0.776	3.074	0.665	6	10313	ZINC000006020022

0.1	-5.8	-5.6	0.11	1.196	0.425	4.451	0.403	6	10314	ZINC000005131990
0.1	-8.3	-7.8	0.29	1.799	0.414	3.279	0.749	6	10315	ZINC000015044097
0.1	-4.4	-4.0	0.22	2.270	0.597	2.706	0.629	6	10316	ZINC000001867146
0.1	-5.4	-5.1	0.15	2.840	0.915	3.780	0.973	6	10317	ZINC000001574407
0.1	-6.8	-6.5	0.16	2.448	0.413	4.281	1.398	6	10318	ZINC000004097096
0.1	-8.2	-7.7	0.28	1.612	0.396	3.057	0.428	6	10319	ZINC000253498249
0.1	-7.1	-7.0	0.10	1.818	0.234	5.166	0.439	6	10320	ZINC000100363055
0.1	-7.5	-7.3	0.15	3.027	0.132	5.352	0.177	6	10321	ZINC000005116907
0.1	-6.1	-6.0	0.07	2.928	0.661	3.705	0.928	6	10322	ZINC000000404428
0.1	-5.2	-5.0	0.13	1.928	0.527	2.883	0.281	6	10323	ZINC000005821005
0.1	-8.1	-7.8	0.23	1.733	0.209	3.620	1.518	6	10324	ZINC000014682852
0.1	-5.1	-4.9	0.11	2.747	1.314	4.214	0.785	6	10325	ZINC000000900788
0.1	-8.1	-7.9	0.10	1.549	0.384	3.914	1.670	6	10326	ZINC000003983883
0.1	-9.1	-8.6	0.37	2.118	0.328	4.470	1.169	6	10327	ZINC000018152086
0.1	-9.7	-8.4	0.79	1.938	0.460	4.788	1.852	6	10328	ZINC000100777986

0.1	-6.2	-6.0	0.09	3.476	0.972	4.537	1.053	6	10329	ZINC000013438050
0.1	-8.3	-8.1	0.16	1.503	0.384	2.851	0.665	6	10330	ZINC000257469898
0.1	-9.2	-8.0	0.95	2.211	0.566	4.023	1.249	6	10331	ZINC000100780661
0.1	-7.0	-6.9	0.07	1.898	0.205	4.978	1.011	6	10332	ZINC000014439174
0.1	-10.5	-9.6	0.69	1.609	0.131	3.907	1.535	6	10333	ZINC000100783988
0.1	-8.2	-7.7	0.30	1.430	0.271	4.479	1.852	6	10334	ZINC000253498246
0.1	-6.8	-6.6	0.18	2.050	0.726	3.308	0.415	6	10335	ZINC000001672944
0.1	-6.3	-6.0	0.22	3.145	1.256	4.264	1.697	6	10336	ZINC000001590840
0.1	-5.4	-5.2	0.11	3.309	1.243	4.003	1.510	6	10337	ZINC000001529588
0.1	-7.6	-7.5	0.08	1.766	0.149	4.902	1.308	6	10338	ZINC000003870602
0.1	-7.9	-7.7	0.12	2.836	0.298	4.951	1.048	6	10339	ZINC000004787342
0.1	-4.3	-4.1	0.14	3.206	1.808	3.702	1.986	6	10340	ZINC000034781669
0.1	-9.2	-8.9	0.24	1.505	0.203	4.343	0.502	6	10341	ZINC000257458459
0.1	-6.6	-6.5	0.13	2.363	0.507	4.004	1.366	6	10342	ZINC000013514843
0.1	-8.5	-8.3	0.16	1.677	0.355	4.707	1.739	6	10343	ZINC000100828809

0.1	-8.7	-7.6	0.98	1.752	0.143	4.732	0.330	6	10344	ZINC000085589186
0.1	-7.9	-7.7	0.09	2.255	0.506	4.403	0.640	6	10345	ZINC000014859833
0.1	-7.2	-7.0	0.13	1.698	0.313	2.831	0.424	6	10346	ZINC000004533757
0.1	-4.7	-4.6	0.09	1.197	0.899	3.634	1.144	6	10347	ZINC000003860193
0.1	-8.4	-8.1	0.18	1.828	0.321	4.499	1.981	6	10348	ZINC000006067269
0.1	-7.3	-6.9	0.21	2.792	0.339	4.622	0.545	6	10349	ZINC000034248042
0.1	-8.5	-8.2	0.21	1.966	0.686	4.342	1.506	6	10350	ZINC000002584002
0.1	-8.8	-8.2	0.41	2.006	0.825	4.690	1.858	6	10351	ZINC000004654623
0.1	-5.9	-5.7	0.14	2.604	1.484	3.093	1.518	6	10352	ZINC000006092898
0.1	-4.7	-4.3	0.18	2.414	0.451	2.931	0.352	6	10353	ZINC000004658605
0.1	-7.2	-7.1	0.07	3.031	0.753	5.112	1.439	6	10354	ZINC000013545026
0.1	-3.9	-3.7	0.12	1.723	0.436	2.436	0.437	6	10355	ZINC000002169156
0.1	-7.0	-6.9	0.15	1.673	0.808	3.374	0.484	6	10356	ZINC000002386390
0.1	-10.2	-9.7	0.29	1.813	0.265	4.187	2.074	6	10357	ZINC000015169343
0.1	-8.4	-8.0	0.25	1.941	0.324	4.663	2.224	6	10358	ZINC000034264744

0.1	-9.9	-9.0	0.53	1.622	0.451	3.849	1.522	6	10359	ZINC000014592806
0.1	-8.2	-7.9	0.23	1.686	0.499	3.867	1.676	6	10360	ZINC000015112253
0.1	-8.6	-8.0	0.49	1.603	0.381	4.294	1.546	6	10361	ZINC000014922903
0.1	-7.9	-7.6	0.14	1.426	0.315	4.307	1.785	6	10362	ZINC000100828657
0.1	-9.4	-8.7	0.49	2.077	0.386	4.437	1.327	6	10363	ZINC000014779271
0.1	-7.2	-6.9	0.19	2.033	0.281	3.943	1.737	6	10364	ZINC000014503848
0.1	-6.6	-6.2	0.18	1.512	0.207	3.906	1.118	6	10365	ZINC000012504388
0.1	-3.9	-3.6	0.20	2.662	0.925	3.428	0.950	6	10366	ZINC000033842666
0.1	-7.2	-6.9	0.21	2.657	0.478	4.334	1.302	6	10367	ZINC000002384779
0.1	-6.0	-5.9	0.07	1.728	0.338	3.990	0.604	6	10368	ZINC000002038799
0.1	-7.9	-6.7	0.78	1.597	0.351	4.389	1.740	6	10369	ZINC000008602415
0.1	-6.5	-6.3	0.13	2.236	0.692	4.279	1.771	6	10370	ZINC000001530388
0.1	-6.2	-5.8	0.18	1.888	0.310	3.916	0.611	6	10371	ZINC000001731784
0.1	-6.8	-6.6	0.18	1.817	0.610	3.243	0.429	6	10372	ZINC000001672941
0.1	-8.7	-8.3	0.25	1.951	0.221	2.855	0.379	6	10373	ZINC000069486144

0.1	-7.9	-7.5	0.19	2.082	0.219	4.499	1.168	6	10374	ZINC000005358301
0.1	-7.0	-6.7	0.25	1.611	0.245	3.521	1.390	6	10375	ZINC000000895534
0.1	-5.6	-5.5	0.22	1.685	0.516	3.698	0.556	6	10376	ZINC000002040174
0.1	-9.3	-8.9	0.28	1.904	0.059	5.192	1.516	6	10377	ZINC000018061098
0.1	-8.2	-7.9	0.25	1.711	0.634	2.949	1.400	6	10378	ZINC000014859989
0.1	-4.3	-4.1	0.15	1.590	0.719	2.344	0.589	6	10379	ZINC000004658584
0.1	-6.7	-6.6	0.09	2.476	0.405	3.911	1.066	6	10380	ZINC000095617769
0.1	-7.0	-6.8	0.08	2.904	0.386	5.161	0.652	6	10381	ZINC000005274088
0.1	-8.9	-8.4	0.31	2.703	0.410	4.600	0.781	6	10382	ZINC000013383460
0.1	-6.7	-6.5	0.11	2.344	0.747	3.998	1.183	6	10383	ZINC000001575291
0.1	-6.9	-6.8	0.10	2.083	0.358	4.148	0.685	6	10384	ZINC000100825938
0.1	-8.1	-7.7	0.29	2.884	0.403	4.507	1.063	6	10385	ZINC000050027566
0.1	-9.1	-8.6	0.35	1.581	0.246	4.115	1.296	6	10386	ZINC000015220880
0.1	-5.9	-5.7	0.17	1.757	0.462	3.639	0.927	6	10387	ZINC000014419530
0.1	-5.0	-4.8	0.09	2.356	0.906	3.895	0.947	6	10388	ZINC000100017404

0.1	-6.7	-6.4	0.20	2.200	0.237	4.840	1.459	6	10389	ZINC000031500058
0.1	-7.2	-7.0	0.13	2.564	0.709	4.125	1.577	6	10390	ZINC000000057152
0.1	-6.1	-5.9	0.13	2.768	0.362	3.716	0.575	6	10391	ZINC000000403004
0.1	-4.9	-4.8	0.08	2.590	0.761	3.163	0.818	6	10392	ZINC000000163730
0.1	-5.1	-4.9	0.16	1.637	0.573	2.141	0.840	6	10393	ZINC000000388699
0.1	-6.5	-6.2	0.18	1.459	0.284	3.641	0.694	6	10394	ZINC000013551951
0.1	-5.3	-5.1	0.15	2.020	0.368	3.138	0.475	6	10395	ZINC000000001669
0.1	-5.7	-5.5	0.10	4.246	1.474	5.263	1.632	6	10396	ZINC000085587800
0.1	-7.2	-6.9	0.20	2.381	0.438	4.027	1.375	6	10397	ZINC000012495162
0.1	-8.1	-6.2	0.93	2.812	0.162	5.589	0.871	6	10398	ZINC000014643895
0.1	-6.8	-6.6	0.13	1.470	0.677	4.492	1.050	6	10399	ZINC000012358714
0.1	-7.2	-6.8	0.23	2.479	0.797	3.772	0.180	6	10400	ZINC000002034481
0.1	-4.9	-4.7	0.16	1.905	0.139	2.910	0.163	6	10401	ZINC000001532553
0.1	-5.8	-5.5	0.11	2.023	0.471	3.763	0.267	6	10402	ZINC000000895230
0.1	-8.5	-7.9	0.39	2.906	0.278	5.671	1.053	6	10403	ZINC000001997859

0.1	-6.1	-5.8	0.16	2.946	0.548	5.162	0.778	6	10404	ZINC000001731776
0.1	-6.0	-5.7	0.20	1.944	0.610	3.880	0.983	6	10405	ZINC000001850547
0.1	-4.1	-4.0	0.11	1.951	0.238	2.653	0.496	6	10406	ZINC000100828526
0.1	-7.6	-7.5	0.09	1.941	0.462	2.786	0.414	6	10407	ZINC000100019057
0.1	-9.2	-8.9	0.21	1.501	0.183	3.965	1.025	6	10408	ZINC000100775365
0.1	-6.8	-6.6	0.20	1.570	0.771	3.967	0.998	6	10409	ZINC000001532162
0.1	-5.0	-4.7	0.22	3.119	0.614	3.786	0.719	6	10410	ZINC000002560463
0.1	-8.8	-8.6	0.13	0.853	0.118	3.845	0.926	6	10411	ZINC000100778622
0.1	-8.9	-8.8	0.16	2.693	0.290	5.003	0.873	6	10412	ZINC000002546073
0.1	-8.5	-8.2	0.23	2.448	0.363	5.083	1.424	6	10413	ZINC000008681609
0.1	-5.4	-5.3	0.09	1.447	0.386	2.439	0.824	6	10414	ZINC000001532613
0.1	-7.1	-6.9	0.15	1.781	0.258	4.167	1.432	6	10415	ZINC000005923353
0.1	-8.7	-8.4	0.25	1.085	0.328	4.971	1.925	6	10416	ZINC000257693615
0.1	-4.9	-4.6	0.24	2.379	1.924	3.064	2.065	6	10417	ZINC000004975461
0.1	-8.8	-8.0	0.42	1.875	0.185	3.683	1.612	6	10418	ZINC000238737193

0.1	-7.9	-7.6	0.22	1.956	0.290	3.826	0.435	6	10419	ZINC000100822083
0.1	-5.4	-5.0	0.18	3.101	1.747	4.205	1.468	6	10420	ZINC000014491652
0.1	-8.9	-8.5	0.32	2.003	0.098	4.493	2.102	6	10421	ZINC000257569789
0.1	-8.0	-7.7	0.17	2.232	0.232	4.369	0.863	6	10422	ZINC000001590782
0.1	-5.8	-5.5	0.20	2.223	0.754	3.828	1.135	6	10423	ZINC000014453728
0.1	-6.6	-6.4	0.09	2.000	0.989	3.410	0.699	6	10424	ZINC000038523228
0.1	-8.5	-7.2	1.05	1.923	0.236	4.521	1.965	6	10425	ZINC000000899796
0.1	-6.6	-6.3	0.17	1.851	0.609	3.382	0.540	6	10426	ZINC000000388166
0.1	-9.0	-8.5	0.26	2.241	0.850	4.393	1.147	6	10427	ZINC000257510178
0.1	-9.8	-9.3	0.33	1.445	0.323	4.153	1.213	6	10428	ZINC000100779657
0.1	-8.7	-8.5	0.16	1.799	0.525	4.886	1.968	6	10429	ZINC000257487945
0.1	-6.1	-6.0	0.07	2.768	0.713	4.164	0.964	6	10430	ZINC000033838366
0.1	-5.1	-4.8	0.16	1.881	0.479	3.069	0.639	6	10431	ZINC000002011663
0.1	-3.8	-3.5	0.18	4.232	1.473	4.698	1.347	6	10432	ZINC000001687155
0.1	-6.1	-6.0	0.07	3.406	1.306	5.465	1.723	6	10433	ZINC000005273939

0.1	-5.7	-5.6	0.07	2.530	0.799	4.220	1.277	6	10434	ZINC000003869805
0.1	-7.9	-7.7	0.14	1.450	0.434	6.482	2.174	6	10435	ZINC000013340108
0.1	-5.9	-5.7	0.13	2.071	0.881	4.644	1.063	6	10436	ZINC000144018055
0.1	-8.5	-7.8	0.47	2.105	0.461	5.186	1.299	6	10437	ZINC000004228312
0.1	-7.9	-7.7	0.14	1.551	0.297	3.903	0.322	6	10438	ZINC000002169366
0.1	-9.3	-8.8	0.30	2.204	0.742	4.365	0.644	6	10439	ZINC000100780721
0.1	-7.9	-7.7	0.13	1.623	0.162	3.606	0.888	6	10440	ZINC000104366578
0.1	-5.5	-5.4	0.05	2.282	0.676	3.726	1.111	6	10441	ZINC000014491655
0.1	-7.0	-6.8	0.13	2.344	0.752	3.548	1.346	6	10442	ZINC000095620868
0.1	-8.3	-8.0	0.23	2.559	0.653	5.807	0.499	6	10443	ZINC000015270270
0.1	-6.0	-5.9	0.10	2.574	0.601	4.314	1.059	6	10444	ZINC000004556725
0.1	-6.3	-6.0	0.25	2.905	1.012	3.912	1.447	6	10445	ZINC000033967236
0.1	-8.1	-7.8	0.20	2.018	0.223	5.761	0.889	6	10446	ZINC000012496295
0.1	-9.2	-9.0	0.18	1.486	0.193	3.767	0.906	6	10447	ZINC000257458461
0.1	-8.2	-7.5	0.36	1.779	0.566	4.413	2.197	6	10448	ZINC000014491301

0.1	-8.6	-7.9	0.68	2.180	0.727	4.392	1.065	6	10449	ZINC000006031516
0.1	-6.0	-5.8	0.09	2.090	0.615	3.457	0.695	6	10450	ZINC000000896626
0.1	-5.2	-5.0	0.17	2.861	0.673	3.635	0.736	6	10451	ZINC000002017391
0.1	-8.3	-7.8	0.23	2.016	0.623	4.648	1.390	6	10452	ZINC000015044095
0.1	-9.7	-9.3	0.33	2.618	0.211	4.936	0.852	6	10453	ZINC000100776789
0.1	-9.8	-9.8	0.00	0.000	0.000	0.000	0.000	1	10454	ZINC000118937396
0.1	-9.1	-8.5	0.43	1.939	0.256	3.727	1.213	6	10455	ZINC000257591136
0.1	-5.1	-4.8	0.18	2.656	0.971	3.473	1.541	6	10456	ZINC000005160191
0.1	-8.0	-7.7	0.24	2.657	0.268	4.784	0.492	6	10457	ZINC000006070279
0.1	-8.5	-8.0	0.28	2.251	0.358	4.891	1.358	6	10458	ZINC000257562931
0.1	-7.0	-6.9	0.12	1.962	0.497	2.514	0.566	6	10459	ZINC000000155996
0.1	-7.9	-7.5	0.21	1.306	0.205	5.070	1.799	6	10460	ZINC000004096706
0.1	-7.9	-7.3	0.29	2.382	0.316	4.910	0.274	6	10461	ZINC000005662799
0.1	-8.7	-7.8	0.54	2.544	0.561	4.978	1.094	6	10462	ZINC000257380052
0.1	-7.9	-7.6	0.17	1.539	0.362	3.314	0.917	6	10463	ZINC000100823534

0.1	-6.5	-6.4	0.06	1.876	0.618	3.734	0.602	6	10464	ZINC000000038028
0.1	-9.1	-8.5	0.44	1.815	0.247	3.685	0.877	6	10465	ZINC000257638141
0.1	-8.4	-7.4	0.82	1.848	0.681	3.918	1.636	6	10466	ZINC000013480188
0.1	-8.8	-8.2	0.43	1.804	0.265	4.636	1.332	6	10467	ZINC000257514619
0.1	-6.8	-6.5	0.16	2.141	0.568	3.670	1.457	6	10468	ZINC000005662848
0.1	-8.3	-8.1	0.12	2.356	0.213	3.904	0.668	6	10469	ZINC000008552277
0.1	-7.0	-6.9	0.15	2.079	0.303	4.841	1.966	6	10470	ZINC000002516115
0.1	-6.0	-6.0	0.04	2.684	0.348	3.766	0.890	6	10471	ZINC000015221887
0.1	-10.3	-8.8	0.99	1.682	0.081	5.411	1.629	6	10472	ZINC000256403002
0.1	-8.9	-8.4	0.32	2.062	0.181	6.151	0.946	6	10473	ZINC000257767906
0.1	-9.2	-8.0	1.01	2.167	0.449	5.167	1.978	6	10474	ZINC000000901042
0.1	-5.0	-4.9	0.10	1.685	0.233	2.301	0.218	6	10475	ZINC000005818690
0.1	-8.1	-6.9	0.88	1.913	0.279	5.937	1.687	6	10476	ZINC000014820485
0.1	-6.8	-6.7	0.09	2.759	0.484	3.843	0.863	6	10477	ZINC000000388262
0.1	-7.8	-7.7	0.07	2.570	0.256	4.308	1.494	6	10478	ZINC000017721961

0.1	-5.3	-5.1	0.16	1.891	0.639	2.644	0.445	6	10479	ZINC000032163906
0.1	-3.9	-3.6	0.17	2.294	0.698	3.006	1.082	6	10480	ZINC000039260233
0.1	-10.7	-9.4	0.71	2.125	0.095	4.633	1.251	6	10481	ZINC000257393578
0.1	-7.9	-7.5	0.25	1.767	0.138	3.545	1.264	6	10482	ZINC000014444622
0.1	-6.4	-6.2	0.11	2.116	0.613	3.140	0.480	6	10483	ZINC000001602584
0.1	-4.9	-4.7	0.11	2.635	0.506	3.541	0.694	6	10484	ZINC000095618404
0.1	-5.9	-5.7	0.18	1.666	0.498	3.990	0.763	6	10485	ZINC000013351308
0.1	-7.2	-7.0	0.16	2.852	0.370	3.790	0.725	6	10486	ZINC000019074717
0.1	-7.8	-7.6	0.11	2.889	0.504	5.127	1.534	6	10487	ZINC000050027019
0.1	-7.1	-6.7	0.20	2.505	0.284	3.948	0.898	6	10488	ZINC000034248039
0.1	-8.0	-7.4	0.34	1.771	0.533	3.598	1.101	6	10489	ZINC000001649427
0.1	-3.9	-3.8	0.08	1.828	0.446	2.344	0.455	6	10490	ZINC000014724336
0.1	-6.1	-5.9	0.20	2.189	0.678	3.057	1.140	6	10491	ZINC000028631755
0.1	-4.3	-4.0	0.16	3.147	1.305	3.399	1.414	6	10492	ZINC000054009855
0.1	-6.2	-6.0	0.11	2.741	0.628	3.826	0.968	6	10493	ZINC000069485968

0.1	-7.4	-6.7	0.34	1.539	0.217	6.358	1.524	6	10494	ZINC000257516124
0.1	-8.9	-8.7	0.17	1.512	0.224	5.119	1.334	6	10495	ZINC000006094144
0.1	-7.2	-7.0	0.08	2.400	0.147	4.894	1.044	6	10496	ZINC000005273941
0.1	-7.1	-6.9	0.11	1.679	0.469	3.103	1.304	6	10497	ZINC000014610452
0.1	-7.4	-6.9	0.30	2.856	0.440	5.074	0.958	6	10498	ZINC000001572939
0.1	-7.1	-6.9	0.20	2.277	0.496	3.873	1.753	6	10499	ZINC000015261407
0.1	-4.9	-4.8	0.14	2.543	0.893	3.381	0.903	6	10500	ZINC000013509329
0.1	-6.4	-6.2	0.12	2.901	0.394	5.127	0.401	6	10501	ZINC000050027203
0.1	-6.5	-6.2	0.18	2.141	0.677	3.261	0.511	6	10502	ZINC000000388432
0.1	-7.9	-7.4	0.30	1.887	0.400	4.780	1.098	6	10503	ZINC000027638917
0.1	-5.4	-5.4	0.05	2.263	0.417	2.918	0.361	6	10504	ZINC000004556034
0.1	-6.5	-6.4	0.05	2.684	0.834	4.174	1.482	6	10505	ZINC000004533503
0.1	-9.9	-9.6	0.15	1.806	0.309	4.187	1.952	6	10506	ZINC000257693620
0.1	-6.1	-5.9	0.14	5.229	1.071	6.704	0.565	6	10507	ZINC000084403374
0.1	-7.3	-7.1	0.21	3.155	1.232	5.184	0.482	6	10508	ZINC000014504288

0.1	-8.4	-7.8	0.28	1.649	0.272	3.298	1.341	6	10509	ZINC000014642690
0.1	-6.4	-6.0	0.21	2.897	0.408	3.899	0.361	6	10510	ZINC000000142824
0.1	-4.2	-4.1	0.11	1.562	0.557	3.273	0.764	6	10511	ZINC000001532209
0.1	-9.6	-8.3	0.96	1.597	0.516	5.167	2.385	5	10512	ZINC000085841735
0.1	-6.6	-6.4	0.11	1.535	0.487	3.206	0.603	6	10513	ZINC000012153388
0.1	-8.0	-7.8	0.13	3.003	0.345	4.797	1.335	6	10514	ZINC000003869858
0.1	-4.6	-4.4	0.13	1.999	0.493	3.088	1.186	6	10515	ZINC000022000224
0.1	-8.5	-7.9	0.50	2.379	0.904	3.747	1.965	6	10516	ZINC000261494646
0.1	-5.7	-5.5	0.13	2.546	1.260	4.136	1.234	6	10517	ZINC000013508504
0.1	-6.0	-5.8	0.11	2.778	0.692	4.106	0.221	6	10518	ZINC000018531805
0.1	-6.6	-6.4	0.11	2.460	0.342	4.240	0.553	6	10519	ZINC000004096247
0.1	-8.8	-8.3	0.28	1.816	0.505	5.661	1.398	6	10520	ZINC000015251259
0.1	-6.8	-6.7	0.11	2.365	0.364	5.440	1.053	6	10521	ZINC000013514832
0.1	-7.7	-6.6	0.53	1.617	0.263	5.101	0.652	6	10522	ZINC000014828296
0.1	-6.2	-6.1	0.12	2.277	0.576	3.724	0.783	6	10523	ZINC000002504349

0.1	-7.7	-7.5	0.13	1.614	0.510	3.293	1.063	6	10524	ZINC000257560780
0.1	-5.1	-4.9	0.20	2.925	1.648	3.558	1.669	6	10525	ZINC000002504856
0.1	-6.6	-6.3	0.17	2.718	0.893	4.146	1.229	6	10526	ZINC000014588511
0.1	-6.8	-6.7	0.11	2.682	0.446	4.890	1.407	6	10527	ZINC000002384848
0.1	-6.6	-6.4	0.09	2.281	0.826	3.704	0.294	6	10528	ZINC000104375779
0.1	-6.1	-6.0	0.07	3.215	0.413	5.909	0.398	6	10529	ZINC000003869810
0.1	-7.9	-7.6	0.14	1.555	0.062	5.177	1.753	6	10530	ZINC000261499680
0.1	-3.3	-3.2	0.05	1.510	0.803	2.593	0.609	6	10531	ZINC000001686874
0.1	-5.7	-5.3	0.18	2.459	0.246	4.001	0.215	6	10532	ZINC000003870147
0.1	-6.7	-6.4	0.21	2.095	0.397	2.966	0.438	6	10533	ZINC000100770556
0.1	-7.7	-7.4	0.23	2.614	0.686	3.682	1.433	6	10534	ZINC000013386789
0.1	-6.8	-6.6	0.11	1.997	0.496	4.587	0.630	6	10535	ZINC000002584392
0.1	-8.9	-8.8	0.15	1.708	0.916	3.839	1.677	6	10536	ZINC000257617942
0.1	-7.5	-7.2	0.24	2.133	0.654	3.589	1.810	6	10537	ZINC000095617736
0.1	-4.2	-4.0	0.15	1.645	0.439	2.475	0.521	6	10538	ZINC000002035950

0.1	-9.8	-9.3	0.31	1.444	0.325	4.154	1.215	6	10539	ZINC000086028084
0.1	-6.6	-6.4	0.15	2.749	0.427	4.143	0.913	6	10540	ZINC000001572030
0.1	-8.0	-7.7	0.19	3.583	0.083	6.266	0.223	6	10541	ZINC000014964394
0.1	-8.1	-7.8	0.24	1.960	0.625	3.438	1.822	6	10542	ZINC000040472581
0.1	-6.1	-6.0	0.09	1.936	0.286	4.253	0.136	6	10543	ZINC000000902108
0.1	-6.8	-6.4	0.31	2.054	0.493	4.357	0.907	6	10544	ZINC000001731773
0.1	-7.7	-7.4	0.16	1.883	0.272	3.526	0.772	6	10545	ZINC000257560779
0.1	-9.3	-7.8	1.11	1.500	0.390	6.202	0.079	4	10546	ZINC000004743114
0.1	-7.2	-7.0	0.08	2.872	0.425	4.253	0.827	6	10547	ZINC000095617760
0.1	-9.1	-7.9	0.71	2.434	0.394	4.439	1.297	6	10548	ZINC000014612204
0.1	-6.5	-6.4	0.06	2.551	0.860	4.550	1.423	6	10549	ZINC000001731774
0.1	-5.4	-5.1	0.22	2.776	0.913	3.376	1.149	6	10550	ZINC000003599046
0.1	-6.0	-5.7	0.17	2.036	0.688	3.384	0.457	6	10551	ZINC000000388039
0.1	-6.4	-6.2	0.13	2.386	0.489	3.844	0.701	6	10552	ZINC000000395643
0.1	-8.3	-8.1	0.12	2.679	0.423	5.016	0.532	6	10553	ZINC000095617811

0.1	-6.1	-6.0	0.09	2.089	0.465	3.951	0.567	6	10554	ZINC000001530283
0.1	-6.3	-6.1	0.17	1.793	0.351	3.048	1.080	6	10555	ZINC000005512602
0.1	-9.3	-8.6	0.37	2.016	0.210	5.654	0.912	6	10556	ZINC000014723709
0.1	-5.7	-5.4	0.17	2.470	0.819	3.286	0.878	6	10557	ZINC000005759722
0.1	-6.9	-6.5	0.21	2.368	0.401	3.486	1.096	6	10558	ZINC000033963310
0.1	-8.2	-7.8	0.28	1.487	0.296	4.535	1.915	6	10559	ZINC000100298297
0.1	-9.7	-8.8	0.75	1.733	0.130	3.856	1.193	6	10560	ZINC000100780484
0.1	-5.2	-4.8	0.18	1.321	0.195	2.030	0.273	6	10561	ZINC000000391783
0.1	-6.1	-6.0	0.09	2.575	0.289	4.505	0.743	6	10562	ZINC000001532265
0.1	-6.6	-6.4	0.10	1.484	0.486	2.989	0.755	6	10563	ZINC000104355493
0.1	-7.6	-7.2	0.24	1.820	0.565	2.853	1.424	6	10564	ZINC000034022547
0.1	-10.2	-9.4	0.50	1.902	0.275	5.070	1.167	6	10565	ZINC000100823545
0.1	-5.3	-5.1	0.09	2.550	0.182	4.195	0.566	6	10566	ZINC000001532568
0.1	-7.0	-6.7	0.18	2.038	0.290	3.036	0.889	6	10567	ZINC000040439669
0.1	-5.7	-5.3	0.22	2.809	0.713	3.252	0.722	6	10568	ZINC000001701825

0.1	-8.1	-7.8	0.16	1.171	0.300	5.068	1.988	6	10569	ZINC000095617986
0.1	-6.4	-6.0	0.26	3.357	0.973	4.875	0.674	6	10570	ZINC000001637979
0.1	-8.0	-7.2	0.77	1.629	0.863	5.196	1.749	6	10571	ZINC000014643427
0.1	-9.1	-8.0	0.73	2.484	0.372	4.317	1.250	6	10572	ZINC000014612188
0.1	-9.2	-8.1	0.54	2.232	0.404	3.566	0.764	6	10573	ZINC000000900525
0.1	-4.6	-4.5	0.11	2.632	0.624	3.484	0.669	6	10574	ZINC000004972689
0.1	-7.3	-7.2	0.11	2.512	0.599	3.577	0.982	6	10575	ZINC000005997103
0.1	-6.6	-6.4	0.17	2.330	0.475	3.481	0.827	6	10576	ZINC000004556739
0.1	-6.2	-6.0	0.12	2.211	0.384	3.980	1.203	6	10577	ZINC000015060557
0.1	-8.5	-8.3	0.16	2.013	0.225	4.761	0.685	6	10578	ZINC000014587277
0.1	-8.1	-7.9	0.23	2.048	0.221	4.205	0.743	6	10579	ZINC000257572693
0.1	-9.3	-8.7	0.46	1.977	0.192	3.497	1.063	6	10580	ZINC000014636782
0.1	-6.5	-6.0	0.26	1.855	0.252	3.533	0.467	6	10581	ZINC000000397605
0.1	-6.0	-5.7	0.13	2.019	0.381	3.914	0.420	6	10582	ZINC000014616841
0.1	-5.9	-5.9	0.04	2.610	0.676	4.054	1.601	6	10583	ZINC000002043758

0.1	-8.4	-8.1	0.16	2.110	0.085	4.712	0.666	6	10584	ZINC000014587276

Table S7. BChE inhibitors from Metabolites-Clean-In+Vivo dataset (6 modes).

Ea diff.	Ea 1st	Ea mean	Ea SD	rmsd/l.b. mean	rmsd/l.b. SD	rmsd/u.b. mean	rmsd/u.b. SD	no. modes	no. rank	ZINC ID#
109.8	1.5	1.5	0.00	0.000	0.000	0.000	0.000	1	1	ZINC000253700110
95.0	-4.4	-4.2	0.15	1.507	0.000	2.179	0.000	2	2	ZINC000257477016
93.6	-10.8	-9.8	1.21	1.841	0.086	11.100	0.202	3	3	ZINC000100773716
92.3	2.8	2.8	0.00	0.000	0.000	0.000	0.000	1	4	ZINC000230145063
90.8	-10.3	-10.3	0.00	0.000	0.000	0.000	0.000	1	5	ZINC000257511708
89.2	-8.4	-7.9	0.50	1.818	0.000	11.547	0.000	2	6	ZINC000100773723
89.0	7.6	8.3	0.75	2.755	0.000	5.910	0.000	2	7	ZINC000230145081
88.7	6.2	6.2	0.00	0.000	0.000	0.000	0.000	1	8	ZINC000230145090
88.3	3.8	5.2	1.40	2.277	0.000	8.625	0.000	2	9	ZINC000253700114
88.2	2.8	3.2	0.30	0.361	0.369	4.007	3.987	6	10	ZINC000095618164
87.2	-9.4	-9.4	0.00	0.000	0.000	0.000	0.000	1	11	ZINC000100773732

86.1	-3.4	-2.6	0.66	1.903	0.684	9.051	4.455	5	12	ZINC000100773709
85.4	-2.9	-2.9	0.00	0.000	0.000	0.000	0.000	1	13	ZINC000195190176
85.3	-0.3	1.2	1.11	1.831	0.111	9.251	0.043	3	14	ZINC000198244759
84.9	-1.2	-1.2	0.00	0.000	0.000	0.000	0.000	1	15	ZINC000257477017
84.9	-9.5	-9.5	0.00	0.000	0.000	0.000	0.000	1	16	ZINC000100773719
83.0	-7.1	-5.7	1.45	2.194	0.000	11.552	0.000	2	17	ZINC000100773727
82.9	-8.8	-8.4	0.57	2.464	0.083	7.829	3.422	3	18	ZINC000100773712
81.5	-6.8	-6.1	0.44	1.735	0.226	9.061	3.739	4	19	ZINC000100773721
81.4	-9.3	-8.2	1.05	1.543	0.000	5.138	0.000	2	20	ZINC000085664967
79.9	6.3	7.4	1.30	2.607	0.231	5.162	1.490	3	21	ZINC000195192307
79.3	-3.9	-3.9	0.00	0.000	0.000	0.000	0.000	1	22	ZINC000230096408
78.8	-9.8	-8.4	0.97	2.376	0.604	6.602	4.350	3	23	ZINC000257541140
78.4	0.1	0.7	0.55	2.157	0.000	9.240	0.000	2	24	ZINC000257477018
78.3	-7.8	-7.3	0.50	1.627	0.000	2.202	0.000	2	25	ZINC000100773734
78.0	-5.0	-5.0	0.00	0.000	0.000	0.000	0.000	1	26	ZINC000150361833

76.6	-8.7	-7.6	0.85	1.590	0.433	4.426	3.914	5	27	ZINC000257549022
76.6	-2.0	-2.0	0.00	0.000	0.000	0.000	0.000	1	28	ZINC000253388225
76.6	-6.8	-6.0	0.72	1.783	0.381	11.464	0.085	4	29	ZINC000257541139
76.2	-8.1	-6.7	1.02	1.802	0.250	11.035	0.080	3	30	ZINC000257549023
75.3	-5.4	-4.5	0.59	1.884	0.447	3.844	1.447	5	31	ZINC000169358704
75.2	-6.1	-4.8	1.25	2.031	0.000	9.175	0.000	2	32	ZINC000150361836
75.0	-8.6	-8.6	0.00	0.000	0.000	0.000	0.000	1	33	ZINC000257541137
75.0	-1.0	-1.0	0.00	0.000	0.000	0.000	0.000	1	34	ZINC000242548690
74.5	-5.5	-4.0	0.94	2.357	0.204	7.921	2.156	5	35	ZINC000085664958
73.8	-6.3	-4.8	1.50	1.777	0.000	11.620	0.000	2	36	ZINC000100773729
73.5	-6.5	-6.3	0.20	1.681	0.000	11.759	0.000	2	37	ZINC000257541138
73.3	-7.3	-7.3	0.00	2.071	0.000	11.359	0.000	2	38	ZINC000257549024
73.0	-4.6	-3.1	1.45	1.839	0.000	12.058	0.000	2	39	ZINC000100773725
72.7	-3.9	-2.3	1.16	1.635	0.185	7.010	2.867	3	40	ZINC000150361840
72.6	6.6	6.6	0.00	0.000	0.000	0.000	0.000	1	41	ZINC000150364748

71.9	-9.0	-8.7	0.35	2.249	0.000	2.670	0.000	2	42	ZINC000085664963
71.7	-7.7	-6.6	1.15	1.672	0.000	2.926	0.000	2	43	ZINC000253951346
71.1	-5.2	-5.0	0.26	2.226	0.128	6.950	2.055	3	44	ZINC000257702865
70.9	3.5	4.9	1.40	2.492	0.000	6.202	0.000	2	45	ZINC000150364739
70.9	0.9	0.9	0.00	0.000	0.000	0.000	0.000	1	46	ZINC000150364741
70.4	8.9	9.9	0.73	0.817	0.072	2.134	0.305	3	47	ZINC000257655896
70.2	9.1	9.1	0.00	0.000	0.000	0.000	0.000	1	48	ZINC000257655895
70.1	9.3	9.3	0.00	0.000	0.000	0.000	0.000	1	49	ZINC000257655898
69.7	7.2	7.2	0.00	0.000	0.000	0.000	0.000	1	50	ZINC000257422891
69.6	-9.4	-7.7	1.20	2.546	0.091	10.412	0.083	3	51	ZINC000257511707
69.5	-8.0	-8.0	0.00	0.000	0.000	0.000	0.000	1	52	ZINC000257511710
69.5	-10.5	-9.1	1.40	2.055	0.000	11.369	0.000	2	53	ZINC000257549021
69.4	2.7	2.7	0.00	0.000	0.000	0.000	0.000	1	54	ZINC000150364745
69.4	7.5	8.9	1.45	1.160	0.000	2.402	0.000	2	55	ZINC000257422892
69.3	-3.2	-3.2	0.00	0.000	0.000	0.000	0.000	1	56	ZINC000198241786

69.3	7.5	7.5	0.00	0.000	0.000	0.000	0.000	1	57	ZINC000257422894
68.6	9.0	9.0	0.00	0.000	0.000	0.000	0.000	1	58	ZINC000253603711
68.6	8.4	8.9	0.45	0.717	0.000	1.835	0.000	2	59	ZINC000253603718
68.3	8.5	8.5	0.00	0.000	0.000	0.000	0.000	1	60	ZINC000257422893
68.2	0.2	0.9	0.70	2.128	0.000	8.310	0.000	2	61	ZINC000160792462
68.0	8.6	8.6	0.00	0.000	0.000	0.000	0.000	1	62	ZINC000253603713
68.0	-6.5	-5.7	0.89	2.431	0.285	8.998	3.008	5	63	ZINC000257511709
68.0	7.5	7.5	0.00	0.000	0.000	0.000	0.000	1	64	ZINC000257637121
67.9	-11.9	-10.0	1.15	1.700	0.232	4.421	0.244	4	65	ZINC000195921106
67.9	-10.6	-9.2	1.35	1.990	0.000	9.840	0.000	2	66	ZINC000257495326
67.8	-7.4	-6.2	0.95	2.363	0.070	10.291	0.083	3	67	ZINC000257454808
67.2	8.2	8.2	0.00	0.000	0.000	0.000	0.000	1	68	ZINC000257637123
67.1	8.4	8.4	0.00	0.000	0.000	0.000	0.000	1	69	ZINC000257637122
66.6	8.8	8.8	0.00	0.000	0.000	0.000	0.000	1	70	ZINC000257637124
66.1	-3.4	-3.4	0.00	0.000	0.000	0.000	0.000	1	71	ZINC000257702864

66.0	8.3	8.6	0.25	1.132	0.000	1.937	0.000	2	72	ZINC000257465483
66.0	-8.7	-8.1	0.65	1.792	0.000	3.852	0.000	2	73	ZINC000150352379
65.8	-5.8	-4.8	0.64	1.826	0.335	3.419	0.994	6	74	ZINC000257495325
65.5	8.6	8.6	0.00	0.000	0.000	0.000	0.000	1	75	ZINC000257465484
65.5	-4.2	-4.2	0.00	0.000	0.000	0.000	0.000	1	76	ZINC000150361837
65.2	-0.1	-0.1	0.00	0.000	0.000	0.000	0.000	1	77	ZINC000150351754
65.1	-8.1	-6.1	1.01	2.590	1.721	4.940	3.449	6	78	ZINC000257780649
65.0	-2.7	-2.7	0.00	0.000	0.000	0.000	0.000	1	79	ZINC000257477015
65.0	9.3	10.0	0.70	0.892	0.000	2.423	0.000	2	80	ZINC000257465485
64.9	-7.0	-5.9	1.10	1.913	0.000	2.402	0.000	2	81	ZINC000252338821
64.7	15.2	15.2	0.00	0.000	0.000	0.000	0.000	1	82	ZINC000257655897
64.7	8.8	10.1	0.93	0.960	0.246	1.951	0.143	3	83	ZINC000257429630
64.6	7.5	8.8	1.25	2.918	0.000	8.880	0.000	2	84	ZINC000150364003
64.6	8.9	8.9	0.05	0.713	0.000	1.801	0.000	2	85	ZINC000257429628
64.6	9.0	10.1	0.86	1.032	0.195	2.121	0.256	4	86	ZINC000257429627

64.5	8.9	8.9	0.00	0.000	0.000	0.000	0.000	1	87	ZINC000257429629
64.5	10.4	11.4	0.82	1.889	0.703	5.210	3.918	3	88	ZINC000230145075
64.3	-7.7	-6.6	1.10	1.322	0.000	1.735	0.000	2	89	ZINC000253951344
64.0	-0.3	-0.3	0.00	0.000	0.000	0.000	0.000	1	90	ZINC000230096416
63.8	9.3	9.8	0.46	3.061	0.520	10.249	0.595	3	91	ZINC000253388227
63.8	10.6	11.9	1.30	1.578	0.000	2.938	0.000	2	92	ZINC000257465482
63.3	-9.0	-7.8	0.73	2.158	0.255	7.863	2.972	4	93	ZINC000150363358
63.2	13.8	14.3	0.50	1.270	0.395	2.653	0.588	3	94	ZINC000253603715
63.2	-6.1	-5.1	0.69	3.755	1.797	7.407	4.317	3	95	ZINC000257780650
63.0	-11.6	-9.9	1.27	2.109	0.014	10.236	0.270	3	96	ZINC000257431867
63.0	-7.8	-7.8	0.00	0.000	0.000	0.000	0.000	1	97	ZINC000100773731
62.8	-0.3	-0.3	0.00	0.000	0.000	0.000	0.000	1	98	ZINC000257702866
62.6	-8.4	-7.1	0.80	1.972	0.137	11.855	0.055	4	99	ZINC000257410609
62.5	-4.7	-4.5	0.14	2.173	0.454	4.839	1.396	3	100	ZINC000150352323
62.3	-5.2	-4.3	0.88	4.046	1.717	8.419	3.832	6	101	ZINC000257780651

62.1	-8.3	-7.0	0.77	2.007	0.159	11.868	0.083	4	102	ZINC000257410610
62.1	-5.1	-3.6	1.10	4.039	1.926	8.655	4.319	6	103	ZINC000257780652
62.0	-10.4	-9.3	0.67	1.470	0.223	3.848	1.050	5	104	ZINC000230082733
62.0	-8.4	-7.1	0.85	2.017	0.181	11.853	0.063	4	105	ZINC000257410608
61.9	-8.4	-6.9	0.90	1.805	0.442	8.422	4.967	4	106	ZINC000257505796
61.8	-8.5	-7.1	0.79	1.651	0.617	9.320	4.400	5	107	ZINC000257410611
61.8	-8.3	-7.2	0.79	1.870	0.013	11.861	0.037	3	108	ZINC000257505797
61.6	-8.2	-6.9	0.92	1.972	0.103	11.878	0.110	4	109	ZINC000257505798
60.9	-12.2	-12.0	0.29	1.922	0.926	7.501	3.362	6	110	ZINC000257417778
60.9	-12.2	-11.8	0.40	2.249	0.353	7.433	3.286	6	111	ZINC000100775355
60.9	-12.2	-11.9	0.29	1.874	0.910	7.465	3.409	6	112	ZINC000257417779
60.9	-12.2	-11.2	0.56	1.788	0.493	5.179	4.273	6	113	ZINC000257417776
60.8	-12.2	-11.5	0.56	2.061	0.331	8.652	3.154	6	114	ZINC000100775353
60.8	-12.2	-12.0	0.20	1.856	0.897	8.773	2.788	6	115	ZINC000257417777
60.7	-6.9	-5.9	0.73	1.717	0.398	7.085	4.742	5	116	ZINC000257505795

60.6	9.1	9.3	0.20	2.542	0.000	10.841	0.000	2	117	ZINC000253388226
60.1	-2.1	-0.2	1.11	2.040	0.745	6.311	4.126	4	118	ZINC000257408867
60.0	-2.0	-0.8	1.15	2.303	0.000	11.975	0.000	2	119	ZINC000257408866
60.0	-2.0	-0.3	1.25	2.399	0.101	12.061	0.057	3	120	ZINC000257408865
60.0	-9.6	-9.6	0.00	0.000	0.000	0.000	0.000	1	121	ZINC000150352381
59.8	-8.2	-6.8	0.93	1.669	0.521	9.422	4.212	5	122	ZINC000257436872
59.7	-6.3	-6.0	0.30	2.526	0.000	4.452	0.000	2	123	ZINC000085853286
59.7	-3.3	-2.0	1.21	1.520	0.582	4.835	3.408	3	124	ZINC000150351751
59.6	-7.8	-6.6	0.83	1.348	0.651	6.830	4.935	5	125	ZINC000257382482
59.6	-7.7	-6.0	0.89	1.800	0.469	9.853	3.832	6	126	ZINC000257382481
59.6	7.5	8.8	1.25	2.942	0.000	8.887	0.000	2	127	ZINC000150364000
59.5	-8.4	-6.7	1.00	2.055	0.263	11.971	0.059	5	128	ZINC000257386945
59.5	-8.1	-6.4	0.98	2.086	0.284	11.895	0.091	5	129	ZINC000257789096
59.5	-7.7	-6.1	0.90	1.694	0.523	9.335	4.156	5	130	ZINC000257382484
59.5	-7.8	-6.5	1.30	5.881	0.000	11.989	0.000	2	131	ZINC000257500451

59.5	-7.7	-6.4	1.30	1.079	0.000	2.325	0.000	2	132	ZINC000257382483
59.4	-8.3	-6.9	1.45	2.385	0.000	12.042	0.000	2	133	ZINC000257386947
59.4	-8.0	-6.3	1.14	2.014	0.250	11.830	0.077	4	134	ZINC000257789091
59.4	-8.4	-6.9	1.50	2.011	0.000	11.956	0.000	2	135	ZINC000257386948
59.3	-8.3	-7.6	0.70	1.772	0.000	11.936	0.000	2	136	ZINC000257386946
59.3	-8.0	-6.5	0.96	1.994	0.336	11.848	0.078	4	137	ZINC000257789100
59.1	-8.2	-6.8	0.99	1.595	0.562	8.647	4.592	4	138	ZINC000257436873
58.9	-0.8	0.3	1.00	1.577	0.635	4.811	3.984	5	139	ZINC000257408868
58.8	2.0	3.5	1.50	2.560	0.000	9.977	0.000	2	140	ZINC000252338823
58.7	9.3	9.8	0.52	3.122	0.580	10.264	0.559	3	141	ZINC000253388224
58.7	-1.0	-1.0	0.00	0.000	0.000	0.000	0.000	1	142	ZINC000095911651
58.6	7.5	8.8	1.25	2.940	0.000	8.879	0.000	2	143	ZINC000150364009
58.6	7.5	8.8	1.25	2.949	0.000	8.887	0.000	2	144	ZINC000150364005
58.5	-10.5	-9.8	0.75	2.891	0.000	4.018	0.000	2	145	ZINC000085798105
58.4	-5.9	-5.9	0.00	0.000	0.000	0.000	0.000	1	146	ZINC000257500453

58.3	-5.9	-4.8	1.10	2.476	0.000	3.304	0.000	2	147	ZINC000257500452
58.3	-3.3	-1.8	0.89	2.490	0.173	8.016	0.961	4	148	ZINC000261499625
58.2	-3.7	-2.3	0.79	2.451	0.479	8.667	3.436	5	149	ZINC000085853295
58.2	-7.4	-6.5	0.56	1.917	0.441	6.913	3.943	6	150	ZINC000150369178
58.2	-7.7	-5.9	1.13	2.319	0.177	11.767	0.080	4	151	ZINC000257511880
58.1	-7.7	-6.2	0.78	2.037	0.165	11.683	0.087	5	152	ZINC000257511881
58.1	-7.7	-6.2	1.10	2.294	0.001	11.840	0.029	3	153	ZINC000257511879
58.1	-7.7	-6.2	1.07	2.201	0.146	11.796	0.077	3	154	ZINC000257511882
58.0	-9.4	-8.7	1.02	1.784	0.448	5.037	3.285	4	155	ZINC000150352383
58.0	-5.5	-4.4	0.95	4.043	1.547	7.474	4.175	3	156	ZINC000257500454
58.0	-10.0	-8.2	1.07	2.090	0.085	6.073	0.080	4	157	ZINC000150363974
57.9	-9.3	-8.1	1.20	2.351	0.000	6.264	0.000	2	158	ZINC000257617068
57.9	-9.5	-8.0	1.08	1.987	0.332	4.785	0.675	4	159	ZINC000150369635
57.8	-8.3	-7.2	1.04	1.253	0.404	2.301	0.274	3	160	ZINC000257442613
57.8	-8.4	-7.8	0.59	0.839	0.099	1.555	0.412	3	161	ZINC000257442612

57.8	-6.9	-5.7	1.08	1.788	0.364	7.991	4.768	6	162	ZINC000257436870
57.7	-8.3	-7.8	0.55	0.909	0.000	2.047	0.000	2	163	ZINC000118936959
57.7	-8.3	-8.3	0.00	0.000	0.000	0.000	0.000	1	164	ZINC000257442614
57.7	-8.3	-7.3	1.00	1.090	0.000	2.238	0.000	2	165	ZINC000118936958
57.7	-9.2	-8.9	0.30	1.902	0.000	10.464	0.000	2	166	ZINC000257617067
57.6	-8.3	-8.1	0.20	0.757	0.000	1.940	0.000	2	167	ZINC000118936957
57.6	-3.7	-2.3	1.14	1.774	0.095	3.091	0.212	3	168	ZINC000257477949
57.6	-8.2	-7.6	0.60	1.192	0.000	2.237	0.000	2	169	ZINC000257442611
57.6	-7.2	-5.3	1.20	2.303	0.429	6.999	3.220	5	170	ZINC000230162637
57.6	-3.6	-2.2	1.40	2.646	0.000	12.774	0.000	2	171	ZINC000257477946
57.5	-9.8	-8.4	1.11	1.909	0.082	3.678	0.882	3	172	ZINC000257532273
57.3	-3.2	-1.9	0.89	2.208	0.148	9.376	4.301	4	173	ZINC000257477948
57.1	-10.5	-8.6	1.13	2.266	0.179	7.920	0.738	4	174	ZINC000230162645
56.9	-10.5	-9.8	0.75	1.561	0.000	2.005	0.000	2	175	ZINC000196121689
56.3	-6.0	-4.4	0.86	2.293	0.053	8.736	1.229	5	176	ZINC000150352387

56.2	-11.6	-10.6	0.64	1.495	0.371	2.214	0.420	4	177	ZINC000257524086
56.2	-11.6	-10.8	0.75	1.873	0.000	2.736	0.000	2	178	ZINC000257524085
56.2	-10.7	-9.7	0.65	2.159	0.166	7.292	2.624	6	179	ZINC000257617065
56.2	-8.2	-7.1	0.57	1.548	0.554	9.283	4.375	5	180	ZINC000257542530
56.1	-11.5	-9.5	1.16	2.304	0.360	8.388	4.094	4	181	ZINC000257524087
56.1	-8.1	-6.5	1.06	2.093	0.175	11.931	0.068	4	182	ZINC000257542529
56.0	-11.0	-9.8	0.83	1.816	0.252	5.177	4.344	4	183	ZINC000247853265
56.0	-8.1	-7.3	0.59	1.236	0.612	6.709	4.989	3	184	ZINC000257452559
56.0	-11.4	-9.6	1.30	2.562	0.050	11.279	0.144	3	185	ZINC000257524084
56.0	-8.8	-8.2	0.45	2.758	0.146	7.780	2.284	3	186	ZINC000096086150
55.9	-9.7	-9.6	0.05	1.719	0.000	9.530	0.000	2	187	ZINC000085664960
55.9	-8.0	-6.4	1.06	2.034	0.251	11.880	0.058	5	188	ZINC000257542531
55.8	-1.8	-1.2	0.65	2.635	0.337	9.183	3.917	4	189	ZINC000257477947
55.8	-7.9	-6.7	0.81	1.662	0.566	9.731	4.017	6	190	ZINC000257452558
55.8	-7.9	-6.4	0.97	2.082	0.143	11.767	0.095	4	191	ZINC000257452557

55.8	-7.9	-6.4	0.92	1.789	0.587	9.403	4.202	5	192	ZINC000257452560
55.5	-12.6	-12.4	0.20	1.767	0.000	2.639	0.000	2	193	ZINC000257480635
55.5	-7.1	-6.4	0.72	1.432	0.288	2.266	0.035	5	194	ZINC000257436871
55.5	-12.5	-10.4	1.24	2.095	0.061	6.401	3.580	4	195	ZINC000100820551
55.5	-12.6	-12.3	0.25	1.728	0.000	1.955	0.000	2	196	ZINC000257480636
55.5	-12.6	-10.9	1.22	1.678	0.411	2.997	1.101	3	197	ZINC000100820548
55.5	-12.6	-11.9	0.79	1.358	0.284	2.071	0.489	3	198	ZINC000257480634
55.4	-0.6	0.9	0.84	2.338	1.365	3.548	1.294	6	199	ZINC000119363936
55.4	-12.5	-11.0	1.38	1.980	0.145	3.483	0.620	4	200	ZINC000257480633
55.3	-0.5	0.5	0.74	2.827	1.298	4.136	1.228	4	201	ZINC000253599614
55.3	-0.5	1.1	0.87	1.626	0.842	2.797	0.690	5	202	ZINC000253599627
55.3	-12.5	-11.6	0.99	1.713	0.063	2.130	0.091	3	203	ZINC000100820549
55.3	-0.5	0.8	0.77	2.700	1.258	3.821	1.266	4	204	ZINC000253599728
55.3	-0.5	0.8	0.73	2.102	1.443	3.463	1.286	6	205	ZINC000118913853
55.3	-1.6	-0.3	0.97	2.741	0.345	11.099	0.078	4	206	ZINC000085853281

55.2	-0.5	1.3	0.87	2.159	0.975	3.220	0.921	6	207	ZINC000253599618
55.2	-12.4	-10.7	1.32	1.946	0.210	5.292	3.568	5	208	ZINC000100820546
55.2	-0.5	0.6	0.78	2.595	1.193	3.621	1.221	6	209	ZINC000253599724
55.2	-0.5	0.9	0.79	2.094	1.415	3.426	1.281	6	210	ZINC000119364393
55.2	-0.5	1.0	0.87	1.534	1.261	2.628	1.370	5	211	ZINC000119364126
55.1	-7.0	-6.2	0.84	1.969	0.184	11.885	0.132	6	212	ZINC000257542532
55.0	-0.2	1.4	0.91	2.316	0.847	5.408	3.745	6	213	ZINC000118913854
55.0	-0.6	1.2	0.97	3.062	0.189	9.054	3.675	6	214	ZINC000257771241
55.0	-9.9	-8.7	0.87	2.294	0.327	3.318	1.217	3	215	ZINC000257408915
54.9	-10.0	-8.5	1.04	1.723	0.282	9.091	4.083	5	216	ZINC000247853255
54.9	-0.1	1.7	1.02	2.035	0.570	5.692	4.058	5	217	ZINC000253599731
54.8	-0.1	1.2	1.30	2.001	0.000	12.634	0.000	2	218	ZINC000118913852
54.8	-12.0	-11.0	0.84	1.499	0.416	5.069	4.176	4	219	ZINC000100825950
54.7	-11.9	-10.6	0.93	2.015	0.437	7.404	4.363	6	220	ZINC000100825948
54.7	3.1	4.0	1.16	3.026	0.271	5.093	1.117	4	221	ZINC000150351753

54.7	-12.0	-10.8	1.09	2.087	0.271	8.194	4.064	4	222	ZINC000100825951
54.6	-9.5	-7.8	1.11	1.968	0.491	7.839	4.654	6	223	ZINC000247853274
54.6	0.7	2.1	0.87	2.068	0.289	6.330	4.462	4	224	ZINC000257513439
54.6	-5.1	-3.2	0.85	2.209	0.619	2.812	0.741	6	225	ZINC000118936960
54.6	0.2	0.6	0.37	2.602	1.050	3.621	1.118	6	226	ZINC000118913856
54.5	0.9	2.0	0.90	2.195	0.286	7.923	4.718	3	227	ZINC000257513438
54.5	-0.2	1.1	0.90	2.581	0.816	8.746	4.298	6	228	ZINC000257771240
54.5	0.1	1.3	1.02	1.615	0.498	7.564	5.063	3	229	ZINC000257497521
54.5	0.3	1.7	0.94	1.592	1.143	4.726	4.135	5	230	ZINC000119364270
54.5	-1.2	0.1	0.83	1.187	0.424	2.543	0.416	4	231	ZINC000253598904
54.4	0.8	2.3	1.08	1.784	0.693	7.229	5.403	3	232	ZINC000257513437
54.4	0.0	1.2	0.77	2.445	0.861	7.732	4.363	6	233	ZINC000257771238
54.3	1.0	2.1	0.76	2.362	0.541	6.089	3.870	5	234	ZINC000257513440
54.3	0.2	1.4	0.62	2.742	0.931	3.837	0.898	6	235	ZINC000118937069
54.2	0.3	1.7	0.84	2.407	1.160	5.611	3.650	6	236	ZINC000118937068

54.2	0.6	1.5	0.59	2.595	1.242	4.103	1.188	6	237	ZINC000253599623
54.2	-11.1	-11.1	0.00	0.000	0.000	0.000	0.000	1	238	ZINC000257417639
54.2	-0.0	1.4	0.79	2.359	0.869	10.150	4.660	5	239	ZINC000257771239
54.1	-7.9	-6.8	0.77	2.410	0.280	7.480	3.856	5	240	ZINC000247853284
54.1	-0.8	0.2	0.82	1.514	0.813	7.167	5.150	3	241	ZINC000253598914
54.1	0.6	1.9	0.90	3.642	0.207	7.740	3.264	6	242	ZINC000253599726
54.1	-5.9	-5.1	0.82	2.315	0.189	8.480	2.180	6	243	ZINC000257454805
54.0	-12.4	-10.6	1.04	1.653	0.315	9.471	0.200	4	244	ZINC000257452118
54.0	0.3	2.0	1.00	1.903	0.459	5.376	4.206	5	245	ZINC000257497523
54.0	-11.6	-10.9	0.48	1.424	0.317	2.013	0.440	4	246	ZINC000257618700
54.0	-11.1	-11.1	0.00	0.000	0.000	0.000	0.000	1	247	ZINC000257417641
53.9	-12.1	-10.4	1.07	1.990	0.316	5.545	3.453	4	248	ZINC000230158897
53.9	-7.4	-6.1	0.84	1.982	0.427	5.251	1.454	5	249	ZINC000230162649
53.9	-11.5	-11.1	0.43	1.312	0.277	2.099	0.465	3	250	ZINC000257618697
53.9	-6.3	-5.1	0.88	2.539	0.296	10.341	0.177	3	251	ZINC000257454807

53.9	-11.6	-10.6	1.00	1.070	0.000	2.070	0.000	2	252	ZINC000257618698
53.8	-8.4	-7.7	0.70	2.265	0.000	9.995	0.000	2	253	ZINC000150369174
53.7	0.7	1.5	0.74	2.548	1.175	6.080	3.990	5	254	ZINC000118937070
53.5	-11.9	-11.3	0.41	1.913	0.545	7.077	3.583	6	255	ZINC000085897767
53.5	1.0	1.8	0.56	2.769	0.768	4.211	0.811	6	256	ZINC000257497524
53.3	-9.4	-9.2	0.25	1.887	0.000	4.222	0.000	2	257	ZINC000150363345
53.3	1.2	1.4	0.16	3.819	0.104	5.081	0.092	3	258	ZINC000118937071
53.1	-10.2	-9.2	0.77	2.347	0.452	4.937	3.899	5	259	ZINC000100825952
53.1	-7.1	-6.2	0.92	2.438	0.153	7.630	1.935	6	260	ZINC000230162657
53.1	1.4	2.0	0.53	3.109	0.794	8.895	3.679	3	261	ZINC000257497522
53.1	-13.7	-12.8	0.90	1.858	0.000	9.610	0.000	2	262	ZINC000230040885
53.1	-12.3	-10.5	1.02	1.539	0.321	6.377	3.109	5	263	ZINC000085741860
53.0	-3.7	-2.7	0.81	2.349	0.511	7.174	3.016	6	264	ZINC000150353194
53.0	-5.7	-3.9	1.07	2.208	0.013	7.855	0.697	4	265	ZINC000150366456
53.0	-3.8	-3.2	0.54	2.651	0.175	7.650	0.791	4	266	ZINC000261499626

52.9	0.4	1.4	1.00	2.631	0.000	12.354	0.000	2	267	ZINC000253598907
52.8	-10.5	-10.5	0.00	0.000	0.000	0.000	0.000	1	268	ZINC000150367453
52.8	-10.3	-8.8	1.25	2.009	0.159	6.089	3.970	4	269	ZINC000100829679
52.7	-10.2	-8.9	1.16	1.877	0.329	6.875	4.792	3	270	ZINC000257451253
52.7	-1.9	-0.4	0.76	2.525	0.782	8.849	4.311	6	271	ZINC000257440454
52.7	-12.0	-9.7	1.16	2.139	0.215	7.615	3.803	5	272	ZINC000257789765
52.6	-10.2	-9.7	0.45	1.778	0.011	2.621	0.153	3	273	ZINC000100829683
52.6	-10.2	-8.3	1.26	1.772	0.389	5.270	3.814	5	274	ZINC000257451250
52.6	-11.9	-10.2	1.21	1.785	0.386	4.734	3.288	6	275	ZINC000257789777
52.6	-11.9	-10.9	1.08	1.717	0.085	2.599	0.586	5	276	ZINC000257789781
52.6	-12.0	-10.4	1.23	1.929	0.302	4.662	3.318	6	277	ZINC000100829559
52.6	-6.2	-6.2	0.00	0.000	0.000	0.000	0.000	1	278	ZINC000085853291
52.5	-11.8	-11.3	0.50	1.666	0.000	1.902	0.000	2	279	ZINC000100777876
52.5	-10.1	-8.1	1.39	2.035	0.266	7.623	4.062	3	280	ZINC000257451251
52.5	-10.1	-8.6	1.22	1.762	0.424	4.777	3.540	6	281	ZINC000257451252

52.5	-11.7	-10.2	1.45	1.942	0.000	4.213	0.000	2	282	ZINC000100777878
52.5	-12.3	-11.2	0.90	2.089	0.537	6.367	3.441	6	283	ZINC000150353091
52.5	-11.9	-10.2	1.18	2.096	0.745	7.299	4.281	5	284	ZINC000257789771
52.5	-11.8	-11.8	0.00	0.000	0.000	0.000	0.000	1	285	ZINC000257552323
52.5	-9.9	-8.5	1.16	1.633	0.446	4.618	4.154	5	286	ZINC000100829680
52.5	-10.0	-8.2	0.89	2.153	0.084	8.548	1.784	5	287	ZINC000230158841
52.4	-11.7	-11.7	0.00	0.000	0.000	0.000	0.000	1	288	ZINC000100777875
52.4	-11.8	-10.5	1.17	1.796	0.411	4.833	3.680	5	289	ZINC000100829556
52.4	-11.7	-10.4	1.21	1.800	0.143	3.048	1.151	3	290	ZINC000100777877
52.4	-11.7	-10.3	1.40	1.881	0.000	4.182	0.000	2	291	ZINC000257552324
52.4	-11.7	-9.8	1.14	1.611	0.212	3.016	0.975	5	292	ZINC000257552322
52.4	-13.1	-11.9	0.87	2.058	0.229	6.809	4.589	3	293	ZINC000257755202
52.4	-11.8	-9.9	0.97	1.959	0.757	6.469	4.168	6	294	ZINC000100829555
52.4	-3.4	-2.3	0.84	3.020	0.326	8.320	1.254	5	295	ZINC000150363981
52.4	-1.5	-0.2	0.59	2.005	0.654	6.335	4.673	6	296	ZINC000257479316

52.4	-1.5	-1.1	0.54	2.224	0.385	7.885	4.557	3	297	ZINC000257455316
52.3	-11.6	-10.1	1.45	1.363	0.000	3.746	0.000	2	298	ZINC000257552325
52.3	-13.1	-11.6	1.10	2.183	0.117	6.931	4.468	3	299	ZINC000257755215
52.3	-13.1	-11.6	1.14	1.574	0.507	4.767	4.691	4	300	ZINC000257755212
52.3	-1.5	-0.5	0.60	2.196	0.808	8.359	4.584	6	301	ZINC000257479318
52.3	-11.3	-9.4	0.92	2.189	0.353	7.992	4.022	6	302	ZINC000100783537
52.3	-13.1	-12.1	0.87	1.717	0.576	6.551	4.846	3	303	ZINC000100777490
52.3	-13.1	-11.2	1.12	1.978	0.306	5.361	4.271	4	304	ZINC000100777492
52.3	-3.2	-2.9	0.20	2.860	0.205	8.701	0.971	5	305	ZINC000257545493
52.2	-11.2	-9.6	0.94	1.672	0.467	4.336	3.581	6	306	ZINC000257392925
52.2	-11.3	-9.7	0.90	2.158	0.420	7.649	4.440	6	307	ZINC000257392927
52.2	-11.2	-9.3	0.92	2.232	0.274	7.771	4.288	6	308	ZINC000257392924
52.2	-1.5	-0.3	0.89	1.943	0.442	5.381	4.116	5	309	ZINC000257455315
52.2	-12.0	-10.4	1.01	1.569	0.227	7.836	3.233	4	310	ZINC000230066849
52.2	-12.0	-11.7	0.35	1.232	0.000	1.896	0.000	2	311	ZINC000100829471

52.1	-11.3	-9.7	0.81	2.157	0.407	6.355	3.911	5	312	ZINC000085798091
52.1	-11.2	-9.3	0.96	2.243	0.352	9.321	3.373	5	313	ZINC000100783539
52.1	-12.0	-11.5	0.50	1.736	0.000	2.582	0.000	2	314	ZINC000257448927
52.0	-1.3	-0.6	0.75	2.856	0.000	4.624	0.000	2	315	ZINC000257440455
52.0	-11.0	-9.3	0.96	2.187	0.421	8.599	3.661	4	316	ZINC000100783541
52.0	-1.3	0.0	0.90	2.395	0.756	8.418	4.628	6	317	ZINC000257440453
52.0	-12.0	-10.7	1.27	2.006	0.197	7.084	4.467	3	318	ZINC000257448929
52.0	-9.7	-8.0	1.09	1.779	0.145	5.122	4.436	4	319	ZINC000257496519
52.0	-13.1	-12.1	0.74	1.439	0.654	4.792	4.668	4	320	ZINC000100777494
52.0	-12.0	-11.0	0.95	1.504	0.262	2.149	0.388	5	321	ZINC000100829473
51.9	-1.0	-0.8	0.32	2.464	0.602	10.278	3.623	6	322	ZINC000257479317
51.9	-9.9	-8.7	1.25	1.938	0.000	5.493	0.000	2	323	ZINC000150363354
51.8	-11.9	-10.6	1.21	1.964	0.272	7.060	4.498	3	324	ZINC000257448930
51.8	-9.5	-7.5	1.13	1.636	0.192	5.876	3.841	4	325	ZINC000100829682
51.7	-11.1	-9.4	0.82	2.376	0.477	9.838	3.077	6	326	ZINC000100829558

51.7	-12.8	-11.9	0.89	2.257	0.765	6.676	3.201	6	327	ZINC000100822111
51.7	-0.9	-0.6	0.28	2.048	0.793	6.587	4.678	6	328	ZINC000257479319
51.6	-10.7	-9.8	0.99	2.141	0.258	6.287	3.624	3	329	ZINC000257431868
51.6	-1.4	-0.2	1.04	1.578	0.680	4.644	3.795	6	330	ZINC000257544024
51.5	-1.4	-0.3	0.72	1.783	0.853	4.864	3.836	6	331	ZINC000257544026
51.5	-11.2	-9.7	1.09	2.265	0.231	11.495	0.210	3	332	ZINC000100829469
51.5	-1.4	-0.2	1.05	1.735	0.798	6.312	4.827	6	333	ZINC000257544027
51.5	-1.5	-0.1	1.15	1.894	0.730	6.588	4.613	6	334	ZINC000257544025
51.5	-1.5	0.0	1.03	1.657	0.851	6.299	4.892	6	335	ZINC000256079953
51.5	-0.9	0.0	0.64	2.077	0.849	6.286	4.636	6	336	ZINC000257440452
51.5	-12.0	-11.6	0.45	1.683	0.000	2.481	0.000	2	337	ZINC000257376829
51.4	-1.3	0.1	1.01	2.151	0.409	7.682	4.573	5	338	ZINC000256079958
51.3	-1.3	-0.0	0.99	1.921	0.582	6.543	4.669	6	339	ZINC000256079948
51.3	-1.3	0.3	0.96	2.565	0.054	12.265	0.054	4	340	ZINC000256079950
51.3	-3.7	-2.6	1.10	2.085	0.000	11.308	0.000	2	341	ZINC000100772714

51.3	-11.5	-9.9	0.91	2.181	0.369	7.142	2.581	6	342	ZINC000230040872
51.3	-0.6	0.0	0.50	2.405	0.338	4.291	0.770	4	343	ZINC000257455318
51.3	-5.2	-3.9	1.30	2.633	0.000	6.068	0.000	2	344	ZINC000150366453
51.2	-11.0	-9.2	1.02	1.894	0.364	6.383	4.048	6	345	ZINC000100829470
51.2	-0.5	0.3	0.61	2.571	0.151	12.477	0.090	3	346	ZINC000257455317
51.2	-1.0	0.6	1.24	2.990	0.067	7.983	3.232	3	347	ZINC000257612677
51.2	-11.5	-9.5	1.16	2.172	0.420	8.616	3.653	4	348	ZINC000257489783
51.2	-8.9	-7.9	1.01	2.341	0.525	6.775	3.757	6	349	ZINC000257618699
51.2	-11.8	-11.3	0.49	2.327	0.242	4.423	0.229	4	350	ZINC000095618168
51.2	-1.0	0.5	1.50	2.898	0.000	11.211	0.000	2	351	ZINC000257612676
51.1	-8.9	-8.1	0.88	2.300	0.223	7.710	2.839	3	352	ZINC000257410669
51.1	-11.3	-9.7	1.03	2.026	0.395	5.789	3.842	4	353	ZINC000257489781
51.1	-10.0	-9.0	0.74	1.822	0.006	6.921	3.717	3	354	ZINC000095618167
51.1	2.3	3.7	0.97	2.157	0.070	7.625	4.278	5	355	ZINC000253598910
51.1	-2.8	-1.9	1.07	2.544	0.011	9.523	0.144	3	356	ZINC000150357908

51.0	-11.1	-9.2	0.96	2.167	0.358	7.990	4.021	6	357	ZINC000257489782
51.0	-11.6	-10.6	1.14	1.835	0.580	6.678	4.833	3	358	ZINC000257557259
50.8	-13.1	-12.6	0.50	1.756	0.000	2.000	0.000	2	359	ZINC000257547844
50.8	-13.1	-12.6	0.50	1.707	0.000	1.954	0.000	2	360	ZINC000257547841
50.8	-13.1	-12.6	0.50	1.714	0.000	1.964	0.000	2	361	ZINC000100784168
50.8	-12.8	-11.5	1.03	2.071	0.235	5.628	3.325	6	362	ZINC000100828822
50.8	-8.4	-7.5	0.95	2.651	0.000	9.571	0.000	2	363	ZINC000257408916
50.7	-11.5	-9.7	1.25	1.602	0.462	5.820	3.859	4	364	ZINC000257557261
50.7	-8.9	-8.3	0.60	2.182	0.105	4.950	0.079	3	365	ZINC000015265887
50.7	-8.9	-8.0	0.78	2.306	0.228	7.715	2.843	3	366	ZINC000015265889
50.6	-9.1	-7.8	0.94	1.554	0.267	2.491	0.011	3	367	ZINC000085798104
50.5	-12.3	-11.3	0.71	1.653	0.588	6.100	3.958	5	368	ZINC000100828829
50.5	-9.9	-8.3	0.74	2.571	0.603	9.253	3.330	6	369	ZINC000085798099
50.4	-9.5	-8.7	0.73	2.392	0.476	9.674	3.287	6	370	ZINC000100783535
50.3	-9.2	-8.3	0.76	2.284	0.331	9.885	3.321	6	371	ZINC000257392926

50.2	-10.2	-8.7	0.78	2.055	0.317	8.506	3.713	5	372	ZINC000085827758
50.2	-11.2	-9.6	1.02	2.086	0.534	7.670	4.416	6	373	ZINC000100783045
50.2	-11.7	-10.5	1.19	1.409	0.238	1.972	0.547	3	374	ZINC000257557260
50.1	-9.2	-8.6	0.70	2.159	0.277	6.259	4.082	6	375	ZINC000100783040
50.0	-11.1	-9.6	0.98	2.221	0.445	7.682	4.431	6	376	ZINC000257489784
49.9	-10.2	-8.9	0.79	2.282	0.392	7.595	3.565	5	377	ZINC000100783039
49.9	0.4	1.4	0.92	2.182	0.789	9.679	4.357	5	378	ZINC000257612675
49.9	-13.1	-11.5	1.07	1.800	0.427	5.191	4.388	4	379	ZINC000100777491
49.9	-10.2	-8.9	0.79	2.213	0.292	7.707	3.648	5	380	ZINC000100783042
49.8	-2.2	-0.7	1.06	2.263	0.894	8.958	4.704	4	381	ZINC000257764615
49.7	-11.7	-10.2	1.22	1.942	0.613	8.182	4.550	4	382	ZINC000257557262
49.7	-11.0	-9.7	1.03	2.061	0.165	6.019	3.578	6	383	ZINC000230158891
49.7	-11.8	-10.5	1.27	1.969	0.289	7.057	4.534	3	384	ZINC000100826007
49.6	-11.9	-11.5	0.33	1.406	0.315	2.151	0.410	3	385	ZINC000257376828
49.6	-11.8	-10.4	1.29	1.590	0.517	4.984	4.668	4	386	ZINC000100826003

49.6	-11.9	-11.5	0.40	1.699	0.000	2.574	0.000	2	387	ZINC000100826001
49.5	-7.8	-6.7	0.78	1.848	0.217	5.476	1.843	6	388	ZINC000150363972
49.5	-11.8	-10.6	1.22	1.968	0.270	7.066	4.513	3	389	ZINC000100826005
49.5	-2.0	-1.1	0.57	1.550	0.411	2.867	0.599	5	390	ZINC000257619567
49.5	-10.0	-8.8	0.63	1.873	0.103	7.937	1.893	6	391	ZINC000257532272
49.5	-8.4	-7.0	1.01	2.120	0.026	9.925	0.040	3	392	ZINC000150363965
49.5	-1.8	-0.6	0.67	2.602	0.398	8.133	4.208	5	393	ZINC000257764611
49.4	-1.7	-0.2	0.73	1.984	0.739	5.152	3.878	6	394	ZINC000257764600
49.4	-2.0	-1.0	0.58	1.871	0.913	6.105	5.044	6	395	ZINC000257619568
49.4	-11.7	-10.1	1.29	2.114	0.226	8.489	4.183	4	396	ZINC000257376830
49.4	-2.0	-0.9	0.59	2.454	0.467	8.686	4.264	6	397	ZINC000257619570
49.3	-9.1	-8.2	0.65	2.004	0.628	8.037	4.185	6	398	ZINC000257448928
49.3	-11.6	-10.2	1.27	1.691	0.488	5.275	4.272	4	399	ZINC000257376827
49.3	-1.9	-1.0	0.45	1.867	0.784	4.738	3.874	6	400	ZINC000257619569
49.2	-7.7	-6.4	0.97	2.033	0.284	7.807	3.425	6	401	ZINC000257617069

49.0	-10.1	-9.6	0.50	2.089	0.000	6.337	0.000	2	402	ZINC000150363969
49.0	-11.7	-10.6	1.08	2.423	0.181	5.257	0.609	4	403	ZINC000230158851
49.0	-11.6	-10.3	0.96	2.336	0.477	7.232	3.422	5	404	ZINC000150353462
48.9	-8.1	-6.4	0.89	1.739	0.568	9.450	4.199	5	405	ZINC000257789105
48.9	2.5	2.5	0.00	0.000	0.000	0.000	0.000	1	406	ZINC000261499624
48.7	-12.7	-11.8	0.83	2.102	0.136	4.481	2.987	5	407	ZINC000100828826
48.7	-5.9	-4.3	0.85	2.564	0.376	6.845	1.847	5	408	ZINC000257617070
48.6	-12.4	-11.4	0.95	2.381	0.432	6.737	4.520	3	409	ZINC000100784171
48.6	-10.8	-9.4	0.90	1.979	0.541	4.817	3.747	5	410	ZINC000257382982
48.5	-12.9	-12.9	0.00	0.000	0.000	0.000	0.000	1	411	ZINC000095620352
48.5	-0.8	0.2	0.64	2.050	0.937	8.936	4.822	4	412	ZINC000257764605
48.5	-12.4	-11.6	0.62	1.815	0.099	2.129	0.161	3	413	ZINC000100784165
48.5	-9.4	-9.4	0.00	0.000	0.000	0.000	0.000	1	414	ZINC000150368768
48.4	-12.1	-10.7	0.65	2.215	0.704	6.239	3.543	6	415	ZINC000150353092
48.4	-13.3	-11.4	1.12	2.273	0.332	8.054	4.160	4	416	ZINC000100783695

48.4	-10.8	-9.4	0.72	1.860	0.511	2.683	0.538	6	417	ZINC000257382979
48.4	-6.2	-6.2	0.00	0.000	0.000	0.000	0.000	1	418	ZINC000257454806
48.3	-13.3	-12.2	1.30	2.002	0.519	6.256	4.510	3	419	ZINC000257431446
48.3	2.1	2.5	0.33	1.715	1.060	5.230	4.375	4	420	ZINC000257612674
48.3	-13.3	-12.2	1.30	1.990	0.511	6.260	4.516	3	421	ZINC000100783690
48.3	-13.3	-11.9	1.32	2.176	0.469	7.928	4.349	4	422	ZINC000257431448
48.2	-11.0	-9.9	1.05	1.889	0.000	2.055	0.000	2	423	ZINC000257417640
48.2	-3.0	-3.0	0.05	1.477	0.000	2.497	0.000	2	424	ZINC000257545492
48.1	-10.5	-9.6	0.54	2.713	0.215	6.350	3.303	4	425	ZINC000257547842
48.1	-12.2	-11.2	1.23	2.245	0.581	7.318	3.193	3	426	ZINC000257566122
48.1	-9.9	-9.6	0.29	1.914	0.389	4.570	1.478	6	427	ZINC000230158883
48.1	0.3	1.2	0.69	1.191	0.100	1.494	0.082	3	428	ZINC000257425935
48.1	-9.9	-8.7	1.07	2.624	0.910	7.491	4.139	6	429	ZINC000085798096
48.0	-10.8	-9.2	0.85	2.327	0.117	5.038	3.178	6	430	ZINC000257382981
48.0	-11.1	-11.1	0.00	0.000	0.000	0.000	0.000	1	431	ZINC000257417638

47.9	-13.0	-11.4	1.24	2.023	0.441	6.524	4.483	5	432	ZINC000095619774
47.9	-10.8	-9.0	1.11	2.285	0.141	6.986	4.027	5	433	ZINC000257382980
47.7	-7.8	-6.6	0.76	1.703	0.329	5.361	3.414	6	434	ZINC000257617066
47.5	-13.1	-11.8	0.91	2.187	0.461	5.187	4.294	4	435	ZINC000100784174
47.5	-9.4	-7.6	1.08	2.012	0.193	4.173	0.868	5	436	ZINC000257447172
47.4	-13.1	-11.6	1.05	1.676	0.390	5.089	3.446	4	437	ZINC000085897762
47.3	-13.1	-11.9	0.84	1.548	0.539	5.011	4.519	4	438	ZINC000257755207
47.3	-10.8	-9.2	1.11	2.196	0.556	5.632	3.221	4	439	ZINC000257609142
47.2	-10.6	-9.3	1.30	2.518	0.000	9.943	0.000	2	440	ZINC000257609141
47.2	-10.7	-9.7	0.74	1.216	0.467	3.070	1.576	3	441	ZINC000257609143
47.2	-10.7	-8.8	1.19	2.262	0.397	8.119	2.444	4	442	ZINC000257609144
47.1	-12.4	-11.2	0.71	2.257	0.147	5.927	2.625	4	443	ZINC000095618036
47.1	-11.5	-10.8	0.33	2.440	0.425	5.839	2.988	6	444	ZINC000100772795
47.0	-13.2	-12.1	0.81	1.955	0.507	4.439	3.140	5	445	ZINC000100828824
46.9	-11.9	-11.2	0.40	2.169	1.020	8.065	2.349	6	446	ZINC000100828831

46.9	1.9	3.2	0.96	2.223	0.353	9.435	4.514	4	447	ZINC000257468755
46.9	-8.1	-7.0	0.81	2.126	0.192	6.148	2.063	6	448	ZINC000095618165
46.8	1.9	3.7	1.25	2.254	0.279	9.545	4.340	4	449	ZINC000257468756
46.8	0.4	0.4	0.00	0.000	0.000	0.000	0.000	1	450	ZINC000252338822
46.8	2.0	2.9	0.70	1.425	0.296	2.608	0.440	3	451	ZINC000257468754
46.7	-11.1	-10.1	0.75	1.632	0.950	5.503	4.421	6	452	ZINC000150368553
46.6	-0.9	0.8	1.16	2.381	0.283	7.332	2.994	4	453	ZINC000150357904
46.5	-11.7	-10.4	1.10	2.221	0.425	4.341	0.311	3	454	ZINC000230096432
46.5	-12.3	-10.9	0.87	2.031	0.515	5.408	3.697	6	455	ZINC000257480099
46.5	-9.2	-8.4	0.43	2.473	0.278	6.162	1.037	6	456	ZINC000150352534
46.5	-11.0	-9.9	0.89	1.962	1.109	8.725	3.825	5	457	ZINC000150368556
46.4	-9.5	-8.2	0.97	2.072	0.249	4.819	2.558	6	458	ZINC000257447174
46.4	-9.2	-7.9	1.30	2.807	0.000	5.541	0.000	2	459	ZINC000257408913
46.3	-12.4	-10.9	0.72	2.232	0.505	6.831	3.203	6	460	ZINC000100772797
46.3	-0.1	1.4	1.07	2.103	0.422	9.702	0.227	4	461	ZINC000085646804

46.2	-12.6	-11.8	1.15	1.443	0.207	7.184	3.935	4	462	ZINC000085897759
46.2	-9.9	-8.8	0.77	2.307	0.328	7.227	0.655	4	463	ZINC000257452776
46.0	2.7	3.8	0.98	1.746	0.631	5.246	4.307	5	464	ZINC000257468753
46.0	-12.0	-10.8	0.98	1.653	0.642	8.332	2.802	5	465	ZINC000257485964
45.9	-6.2	-6.2	0.00	0.000	0.000	0.000	0.000	1	466	ZINC000150369169
45.9	-12.1	-10.4	0.99	1.635	0.296	6.769	3.178	5	467	ZINC000150352538
45.9	-7.7	-6.6	0.83	2.097	0.250	7.591	3.078	4	468	ZINC000257431869
45.8	-8.5	-7.3	1.20	2.590	0.000	9.457	0.000	2	469	ZINC000257408914
45.8	-12.0	-10.7	0.90	2.193	0.497	7.311	2.938	6	470	ZINC000257480101
45.7	-11.7	-11.0	0.64	1.938	0.108	3.807	0.528	5	471	ZINC000257394246
45.7	-13.0	-11.3	1.27	2.532	0.294	7.284	3.079	5	472	ZINC000257566123
45.5	-3.6	-2.7	1.23	2.647	0.143	5.284	1.249	3	473	ZINC000096086151
45.4	-6.7	-6.7	0.00	0.000	0.000	0.000	0.000	1	474	ZINC000150366458
45.4	-8.9	-7.6	0.78	2.124	0.290	6.869	3.143	4	475	ZINC000257554951
45.3	-10.4	-9.4	0.47	2.249	0.711	6.101	3.966	6	476	ZINC000257431449

45.2	-11.2	-10.5	0.38	2.199	0.422	5.599	3.123	6	477	ZINC000100772791
45.1	-12.6	-10.9	1.08	2.236	0.877	6.012	3.897	5	478	ZINC000257515264
45.0	-12.9	-11.8	0.90	2.052	0.290	6.370	3.360	5	479	ZINC000100828820
44.9	-4.2	-3.8	0.45	2.304	0.108	8.079	1.431	3	480	ZINC000252338824
44.9	-10.5	-9.3	0.77	2.593	0.224	6.647	3.459	6	481	ZINC000257547843
44.8	-11.8	-10.3	1.02	2.047	0.245	8.284	2.708	6	482	ZINC000230158873
44.8	-11.1	-10.2	0.52	2.314	0.896	7.097	3.796	6	483	ZINC000100772799
44.7	-12.5	-11.3	0.91	2.355	0.302	4.330	0.164	3	484	ZINC000257394245
44.7	-12.5	-10.9	1.08	2.197	0.327	3.842	0.682	4	485	ZINC000257394248
44.6	-8.2	-6.8	1.03	1.631	0.673	4.340	2.802	3	486	ZINC000257452777
44.6	-9.0	-8.6	0.38	2.063	0.331	6.109	3.782	6	487	ZINC000150366892
44.5	-11.0	-10.3	0.82	1.683	0.354	2.466	0.396	5	488	ZINC000257390211
44.5	-11.5	-9.8	0.81	2.769	0.636	6.127	3.242	6	489	ZINC000100772802
44.5	-9.8	-8.9	0.85	1.157	0.000	1.707	0.000	2	490	ZINC000257517755
44.4	-12.5	-11.4	1.10	2.652	0.000	4.483	0.000	2	491	ZINC000257394247

44.4	-7.5	-7.0	0.55	2.106	0.000	8.647	0.000	2	492	ZINC000150369401
44.3	-11.1	-10.7	0.39	2.119	0.824	5.656	3.311	6	493	ZINC000118936865
44.3	-11.1	-10.4	0.55	1.970	0.391	7.010	3.167	6	494	ZINC000118936864
44.3	-11.1	-10.7	0.36	1.680	0.202	5.096	3.702	6	495	ZINC000256430906
44.3	-11.1	-10.7	0.36	1.796	0.248	5.404	3.448	6	496	ZINC000256430895
44.3	-11.1	-10.8	0.27	1.708	0.237	3.832	3.016	6	497	ZINC000118936862
44.3	-11.1	-10.6	0.49	1.873	0.364	6.701	3.545	6	498	ZINC000256430911
44.2	-8.7	-8.0	0.79	1.220	0.309	2.243	0.373	6	499	ZINC000257620436
44.2	-13.0	-11.2	1.07	2.885	0.267	7.865	2.951	4	500	ZINC000257804825
44.2	-11.1	-10.5	0.46	2.262	0.820	7.205	2.957	6	501	ZINC000256430901
44.2	-13.0	-11.2	1.27	2.454	0.019	10.995	0.222	3	502	ZINC000095619777
44.1	-11.1	-10.4	0.40	2.275	0.795	6.918	3.385	6	503	ZINC000118936863
44.0	-9.1	-8.4	0.81	2.123	0.520	7.671	4.228	6	504	ZINC000150366896
44.0	-11.8	-10.9	0.82	2.020	0.453	9.043	3.649	6	505	ZINC000257466940
44.0	-9.0	-7.7	1.02	1.957	0.292	7.833	3.418	5	506	ZINC000230158858

44.0	-8.2	-8.2	0.00	0.000	0.000	0.000	0.000	1	507	ZINC000100822709
43.9	0.7	2.0	0.88	2.223	0.158	7.828	0.441	5	508	ZINC000261499627
43.9	-10.9	-9.8	0.86	2.481	0.303	7.659	1.421	5	509	ZINC000257447173
43.9	-11.8	-11.1	0.75	2.103	0.359	10.859	0.186	5	510	ZINC000257466942
43.8	-11.8	-10.6	1.03	2.091	0.451	8.696	3.841	5	511	ZINC000119384759
43.8	-11.7	-11.0	0.81	2.157	0.377	10.869	0.179	5	512	ZINC000119385173
43.8	-11.8	-10.6	0.75	1.619	0.225	7.901	3.896	4	513	ZINC000257466941
43.8	-11.8	-10.7	1.02	2.340	0.266	7.885	3.671	6	514	ZINC000119385038
43.8	-11.7	-10.8	0.88	1.902	0.641	7.553	4.204	6	515	ZINC000257466939
43.6	-9.0	-8.0	1.09	1.870	0.136	2.969	0.349	5	516	ZINC000257620434
43.6	-9.2	-8.9	0.19	1.987	0.104	10.946	0.075	6	517	ZINC000150366899
43.6	-13.3	-11.9	1.30	1.918	0.434	4.977	4.110	4	518	ZINC000100783691
43.5	-6.7	-5.1	1.04	2.266	0.251	7.131	2.635	4	519	ZINC000150369632
43.5	-11.3	-9.9	1.06	1.964	0.443	4.529	3.492	6	520	ZINC000119384871
43.3	-9.1	-8.9	0.15	1.860	0.489	7.460	4.026	6	521	ZINC000257620433

43.2	-11.1	-10.8	0.15	2.045	0.562	6.480	3.599	6	522	ZINC000257395081
43.1	-8.9	-8.6	0.28	1.902	0.496	5.880	3.999	6	523	ZINC000150366889
43.0	-13.0	-12.1	0.92	1.936	0.651	6.872	3.112	6	524	ZINC000257695303
42.9	-6.1	-4.6	1.13	2.348	0.668	5.646	2.319	5	525	ZINC000150368772
42.9	-10.8	-10.5	0.20	2.078	0.643	5.844	3.365	6	526	ZINC000257421664
42.9	-8.8	-8.3	0.58	1.272	0.435	2.401	0.300	5	527	ZINC000257620435
42.8	-6.7	-5.2	0.80	2.553	0.511	10.699	4.047	6	528	ZINC000004429047
42.7	-9.0	-8.3	0.65	2.356	0.000	9.828	0.000	2	529	ZINC000150369170
42.6	-10.3	-9.1	0.71	1.865	0.427	4.250	3.067	6	530	ZINC000257391276
42.5	-11.0	-9.9	0.77	1.643	0.277	3.912	3.183	5	531	ZINC000085907229
42.5	-11.0	-9.7	0.83	2.136	0.121	8.502	1.896	5	532	ZINC000230040894
42.5	-12.6	-11.4	0.63	1.772	0.243	5.516	3.878	6	533	ZINC000257468786
42.5	-11.4	-10.5	0.79	1.856	0.379	5.555	3.517	4	534	ZINC000257778807
42.4	-11.5	-10.7	0.62	1.762	0.073	10.297	0.075	3	535	ZINC000100777898
42.3	-8.9	-7.9	1.00	2.388	0.000	9.238	0.000	2	536	ZINC000261499653

42.3	-10.9	-10.5	0.40	3.258	0.000	5.121	0.000	2	537	ZINC000100823244
42.3	-10.5	-9.7	0.67	1.684	1.017	5.427	3.020	6	538	ZINC000100824436
42.2	-9.6	-9.6	0.00	0.000	0.000	0.000	0.000	1	539	ZINC000150367989
42.2	-13.3	-11.7	1.15	2.325	0.283	8.066	4.149	4	540	ZINC000257431447
42.2	-13.3	-11.8	1.27	2.223	0.397	7.970	4.278	4	541	ZINC000100783698
42.2	-10.8	-9.5	0.95	3.209	0.018	4.556	0.387	4	542	ZINC000100823245
42.2	-10.8	-9.4	0.94	3.201	0.111	4.740	0.261	5	543	ZINC000257384707
42.2	-10.8	-9.7	0.75	2.583	1.162	3.868	1.596	5	544	ZINC000257384708
42.1	-5.8	-4.3	0.93	1.750	0.582	7.292	4.458	6	545	ZINC000100772715
42.0	-9.0	-7.7	1.03	2.330	0.223	7.026	3.209	5	546	ZINC000095618035
42.0	-11.0	-10.3	0.54	2.033	0.393	6.730	3.613	6	547	ZINC000257390208
41.9	-10.1	-8.7	1.40	3.173	0.000	6.617	0.000	2	548	ZINC000086002047
41.9	-11.0	-10.4	0.41	2.172	0.432	8.287	2.916	6	549	ZINC000257390209
41.9	-11.0	-10.7	0.23	2.034	0.362	6.804	3.636	6	550	ZINC000118936921
41.9	-11.0	-10.2	0.90	2.027	0.269	2.618	0.737	6	551	ZINC000257390210

41.9	-11.0	-10.1	0.90	1.682	0.449	2.629	0.339	4	552	ZINC000118936918
41.8	-10.6	-10.3	0.18	2.770	0.583	6.816	3.226	6	553	ZINC000118937025
41.8	-11.0	-10.4	0.75	1.971	0.508	5.568	3.548	6	554	ZINC000118936920
41.8	-7.7	-6.5	1.19	1.338	0.813	2.385	1.149	3	555	ZINC000257452775
41.8	-10.2	-9.6	0.47	2.168	0.046	8.527	2.684	6	556	ZINC000100772800
41.8	-11.0	-10.7	0.22	2.053	0.365	6.817	3.627	6	557	ZINC000118936919
41.7	-12.7	-11.7	0.97	2.168	0.752	6.138	2.581	6	558	ZINC000085897747
41.7	-12.5	-11.7	0.48	2.046	0.639	5.599	3.621	6	559	ZINC000257804824
41.6	-7.6	-7.6	0.00	0.000	0.000	0.000	0.000	1	560	ZINC000096016073
41.6	-11.7	-11.0	0.38	1.978	0.603	7.269	3.888	6	561	ZINC000015121641
41.5	-11.7	-10.9	0.80	2.339	0.623	4.726	3.126	6	562	ZINC000257542157
41.5	-10.9	-9.6	0.91	2.162	0.937	8.662	2.608	6	563	ZINC000100772798
41.5	-11.6	-10.2	0.77	1.897	0.360	7.053	3.730	6	564	ZINC000257475640
41.5	-10.4	-10.4	0.00	0.000	0.000	0.000	0.000	1	565	ZINC000150368770
41.5	-9.9	-9.0	0.64	2.425	0.915	6.938	2.344	3	566	ZINC000100890127

41.5	-11.1	-10.4	0.65	2.161	0.354	5.320	2.891	6	567	ZINC000257485095
41.4	-12.0	-11.0	0.97	2.583	0.272	6.325	3.214	3	568	ZINC000086034644
41.4	-11.6	-10.9	0.52	2.299	0.614	7.653	3.975	6	569	ZINC000257542155
41.4	-11.1	-10.8	0.33	1.815	0.245	3.017	0.970	6	570	ZINC000257485092
41.4	-11.1	-10.8	0.34	1.997	0.437	4.641	3.309	6	571	ZINC000257485094
41.4	-10.4	-8.6	1.05	1.914	0.399	7.853	3.867	6	572	ZINC000100824479
41.4	-13.2	-11.5	1.03	2.444	0.153	7.366	1.466	4	573	ZINC000085852988
41.4	-11.1	-10.7	0.43	2.058	0.399	4.785	3.191	6	574	ZINC000257485093
41.3	-11.0	-10.6	0.35	1.917	0.260	6.614	3.339	6	575	ZINC000257389062
41.3	-12.7	-11.7	0.78	2.606	0.307	8.465	2.019	6	576	ZINC000100772793
41.3	-11.0	-10.0	0.50	2.975	0.323	7.180	2.447	6	577	ZINC000100782166
41.2	-10.5	-9.3	0.77	1.803	0.198	7.070	4.003	6	578	ZINC000100777903
41.2	-11.0	-10.6	0.24	1.848	0.308	6.735	3.177	6	579	ZINC000118936976
41.1	-11.3	-9.8	1.17	1.919	0.410	3.519	1.585	5	580	ZINC000230096425
41.1	-11.1	-10.8	0.26	1.668	0.459	6.676	3.389	6	581	ZINC000118937281

41.1	-9.9	-9.2	0.75	2.620	0.000	8.378	0.000	2	582	ZINC000257552148
41.1	-10.5	-10.0	0.33	1.982	0.614	5.551	2.780	6	583	ZINC000100782168
41.1	-10.5	-9.8	0.43	2.154	0.827	8.003	2.082	6	584	ZINC000261496439
41.0	-13.7	-12.4	0.84	2.714	0.545	5.332	2.403	6	585	ZINC000086034640
41.0	-10.5	-10.2	0.29	2.694	0.387	3.564	0.374	6	586	ZINC000261496438
41.0	-11.3	-10.1	1.13	2.306	0.417	8.838	3.032	5	587	ZINC000257542156
40.9	-10.5	-10.0	0.36	1.826	0.484	6.664	2.886	6	588	ZINC000261496440
40.9	-13.1	-12.8	0.36	1.866	0.719	6.067	3.343	5	589	ZINC000085897746
40.9	-9.5	-8.0	0.72	2.097	0.912	6.404	2.665	6	590	ZINC000150368685
40.9	-11.2	-10.5	0.69	2.279	0.606	6.151	3.826	6	591	ZINC000257542158
40.9	-9.6	-8.0	0.89	2.108	0.110	7.645	2.352	6	592	ZINC000230158864
40.8	-12.3	-11.6	0.54	1.785	0.177	6.759	2.911	6	593	ZINC000095617902
40.8	-12.9	-12.5	0.23	1.494	0.687	7.625	2.754	5	594	ZINC000085897741
40.8	-12.1	-11.5	0.66	2.478	0.200	6.706	2.682	6	595	ZINC000095617905
40.8	-12.9	-12.0	0.58	2.424	0.848	7.021	3.662	6	596	ZINC000257804826

40.8	-10.5	-10.1	0.35	2.037	0.721	6.744	2.795	6	597	ZINC000261496437
40.7	-9.9	-9.0	0.64	2.423	0.916	6.938	2.344	3	598	ZINC000100890131
40.7	-9.9	-9.0	0.62	2.426	0.912	6.950	2.333	3	599	ZINC000100829262
40.7	-9.9	-9.0	0.64	2.425	0.915	6.942	2.340	3	600	ZINC000100890134
40.7	-9.9	-9.0	0.64	2.425	0.917	6.939	2.344	3	601	ZINC000100829264
40.7	-12.2	-11.3	0.69	2.324	0.406	4.210	2.395	6	602	ZINC000095617904
40.6	30.0	31.5	1.15	1.851	0.581	4.559	2.919	3	603	ZINC000008220909
40.6	-9.8	-8.3	0.72	2.232	0.734	6.169	2.807	6	604	ZINC000150368691
40.6	-9.9	-8.7	0.68	1.672	1.001	4.266	3.146	5	605	ZINC000100890133
40.6	-11.7	-10.3	1.40	2.821	0.000	8.765	0.000	2	606	ZINC000095618166
40.6	1.0	1.0	0.00	0.000	0.000	0.000	0.000	1	607	ZINC000094313270
40.6	-10.8	-9.9	0.66	2.490	0.422	8.302	2.885	6	608	ZINC000257421661
40.6	-11.5	-10.3	0.61	1.862	0.332	5.743	2.812	6	609	ZINC000015216551
40.6	-12.5	-12.1	0.41	2.039	0.323	6.753	3.109	6	610	ZINC000100772783
40.6	-10.8	-10.5	0.22	2.088	0.652	5.851	3.360	6	611	ZINC000118936924

40.5	-10.8	-10.3	0.40	2.430	0.439	8.348	2.910	6	612	ZINC000257421663
40.5	-10.8	-10.4	0.30	2.021	0.585	7.042	3.486	6	613	ZINC000257421662
40.5	-10.0	-9.2	0.59	2.451	0.432	6.155	3.355	5	614	ZINC000085907237
40.5	-5.5	-5.0	0.50	2.395	0.000	7.382	0.000	2	615	ZINC000150357911
40.5	-9.5	-8.0	0.74	2.000	0.729	5.463	2.672	6	616	ZINC000150368687
40.4	-10.8	-10.4	0.37	2.438	0.452	8.369	2.906	6	617	ZINC000118936922
40.4	-9.5	-7.8	0.96	2.123	1.119	4.557	1.679	4	618	ZINC000150368689
40.4	-10.8	-10.5	0.23	2.345	0.348	7.170	3.358	6	619	ZINC000118936925
40.4	-10.8	-10.5	0.27	2.366	0.374	7.200	3.342	6	620	ZINC000118936923
40.4	-9.4	-8.8	0.57	2.218	0.124	7.009	1.188	6	621	ZINC000150352536
40.2	-10.5	-9.1	0.98	2.026	0.648	6.170	2.536	6	622	ZINC000100890110
40.2	-9.8	-9.4	0.38	1.745	0.338	3.792	1.516	4	623	ZINC000150338847
40.2	-13.0	-10.8	1.00	2.003	0.426	7.078	3.430	6	624	ZINC000095619776
40.2	-11.0	-10.5	0.26	1.940	0.300	5.595	3.240	6	625	ZINC000118937047
40.2	-12.8	-11.6	0.75	2.965	0.148	9.979	0.338	6	626	ZINC000150353465

40.1	-12.7	-12.1	0.56	1.745	0.715	6.601	2.473	6	627	ZINC000085897742
40.1	-10.5	-9.4	0.85	1.676	0.199	7.204	2.897	5	628	ZINC000100824437
40.1	-10.9	-10.6	0.24	2.462	0.524	8.036	2.746	6	629	ZINC000253500761
40.1	-10.5	-9.0	0.98	1.903	0.697	6.973	2.634	6	630	ZINC000100890112
40.1	-10.9	-10.6	0.21	2.412	0.553	6.698	3.294	6	631	ZINC000118913858
40.1	-10.5	-9.0	0.98	2.027	0.649	6.200	2.523	6	632	ZINC000100890109
40.1	-9.5	-8.3	1.02	1.770	0.315	4.426	2.819	6	633	ZINC000257391278
40.1	-10.9	-10.6	0.20	2.262	0.631	6.717	3.274	6	634	ZINC000253604295
40.1	-10.5	-9.0	1.13	1.674	0.304	7.332	2.944	5	635	ZINC000100890113
40.0	-10.5	-10.2	0.20	3.020	0.545	5.609	3.293	6	636	ZINC000119381040
40.0	-10.5	-10.2	0.20	2.775	0.531	8.185	2.742	6	637	ZINC000257765572
40.0	30.6	31.8	1.15	2.497	0.000	7.497	0.000	2	638	ZINC000169621220
40.0	-10.5	-10.2	0.24	2.950	0.685	8.177	2.736	6	639	ZINC000257765573
40.0	-10.5	-10.2	0.20	2.771	0.525	8.151	2.724	6	640	ZINC000257765574
40.0	-10.5	-10.2	0.29	2.732	0.540	8.130	2.730	6	641	ZINC000119380633

40.0	-10.5	-10.2	0.18	3.002	0.733	8.166	2.729	6	642	ZINC000257765575
40.0	-10.5	-10.2	0.18	2.996	0.721	8.171	2.732	6	643	ZINC000119380903
39.9	-10.4	-10.1	0.17	2.818	0.642	8.151	2.732	6	644	ZINC000119380779
39.8	-7.6	-6.5	0.70	2.026	0.221	5.053	2.468	5	645	ZINC000257517757
39.8	-9.7	-8.5	0.84	2.017	0.099	5.562	2.610	5	646	ZINC000150357041
39.8	-10.2	-9.8	0.31	1.279	0.557	8.009	4.291	6	647	ZINC000095620869
39.8	-9.1	-7.7	1.13	2.176	0.077	6.294	2.002	5	648	ZINC000196084903
39.6	-11.0	-10.5	0.32	1.889	0.269	5.579	3.258	6	649	ZINC000118937048
39.6	-11.6	-10.5	1.10	2.187	0.003	10.471	0.035	3	650	ZINC000257779510
39.6	-11.2	-10.1	1.05	3.283	0.000	5.801	0.000	2	651	ZINC000070454179
39.6	-11.0	-10.5	0.32	1.928	0.317	4.207	2.709	6	652	ZINC000256421913
39.6	-11.0	-10.5	0.34	1.955	0.304	5.567	3.278	6	653	ZINC000118937049
39.6	-11.2	-10.8	0.28	1.950	0.506	5.303	3.389	6	654	ZINC000257561965
39.6	-11.2	-10.8	0.28	2.164	0.588	6.704	3.357	6	655	ZINC000257561968
39.6	-11.0	-10.5	0.34	1.912	0.335	7.067	3.235	6	656	ZINC000256421903

39.6	-11.2	-10.9	0.23	2.108	0.889	6.389	3.698	6	657	ZINC000118936877
39.6	-11.0	-10.4	0.45	1.905	0.342	2.875	0.380	6	658	ZINC000256421894
39.6	-11.2	-10.8	0.26	2.464	0.809	8.038	2.708	6	659	ZINC000257561967
39.6	-11.0	-10.4	0.31	1.821	0.274	6.841	3.491	6	660	ZINC000118937046
39.5	-11.0	-10.5	0.33	1.973	0.297	5.653	3.190	6	661	ZINC000256421920
39.5	-11.2	-10.8	0.27	2.418	0.750	8.045	2.711	6	662	ZINC000118936874
39.5	-9.7	-8.7	0.79	2.092	0.036	6.417	2.465	4	663	ZINC000150357044
39.5	-11.2	-10.8	0.27	2.418	0.753	8.043	2.710	6	664	ZINC000118936876
39.5	-10.9	-10.4	0.24	2.204	0.320	6.996	2.976	6	665	ZINC000118936859
39.5	-11.2	-10.7	0.43	2.544	0.666	8.046	2.685	6	666	ZINC000118936875
39.5	-11.2	-10.8	0.26	2.634	0.453	8.026	2.741	6	667	ZINC000257561966
39.4	-10.7	-10.3	0.29	1.918	0.242	7.477	4.301	6	668	ZINC000257495940
39.4	-10.7	-10.1	0.34	1.938	0.232	4.208	3.315	6	669	ZINC000118936968
39.4	-10.7	-10.3	0.28	1.960	0.206	7.510	4.261	6	670	ZINC000118936967
39.4	-10.7	-10.3	0.26	1.950	0.217	7.603	4.318	6	671	ZINC000257495939

39.4	-12.9	-11.6	1.03	2.340	0.198	7.805	1.445	5	672	ZINC000257449087
39.4	-10.7	-10.3	0.27	1.664	0.352	5.614	4.253	6	673	ZINC000118936965
39.4	-11.4	-10.0	1.04	1.806	0.248	5.398	3.717	6	674	ZINC000100783811
39.4	-10.7	-10.4	0.21	2.137	0.368	6.116	4.084	6	675	ZINC000257495942
39.4	-10.6	-10.2	0.25	2.797	0.600	6.823	3.262	6	676	ZINC000118937023
39.3	-8.0	-7.0	0.66	2.124	0.256	6.023	2.496	4	677	ZINC000150369406
39.2	-12.5	-11.0	0.81	3.048	0.424	8.026	2.315	6	678	ZINC000100777664
39.1	-11.2	-10.8	0.25	2.011	0.801	8.040	2.708	6	679	ZINC000256408218
39.1	-12.2	-10.8	0.99	1.761	0.412	6.178	3.813	5	680	ZINC000257414050
39.0	-9.7	-9.0	0.53	1.816	0.136	8.895	2.951	6	681	ZINC000100777902
39.0	-11.2	-10.8	0.25	1.977	0.749	8.042	2.710	6	682	ZINC000118937280
39.0	-11.2	-10.8	0.26	2.010	0.825	8.037	2.707	6	683	ZINC000256408210
39.0	-11.2	-10.8	0.32	1.733	0.469	8.109	2.742	6	684	ZINC000118937282
39.0	-11.2	-10.9	0.21	1.759	0.849	6.379	3.706	6	685	ZINC000118937283
39.0	-11.2	-10.8	0.29	1.958	0.757	8.053	2.714	6	686	ZINC000256408227

39.0	-11.9	-11.5	0.27	2.099	0.367	5.903	3.555	6	687	ZINC000100783122
39.0	-11.1	-10.8	0.24	1.980	0.765	8.040	2.709	6	688	ZINC000256408202
38.9	-11.5	-10.5	0.56	2.544	0.409	5.420	2.532	6	689	ZINC000100783852
38.9	-11.9	-11.5	0.30	2.782	0.521	8.465	2.697	6	690	ZINC000100783124
38.9	-11.1	-10.0	0.82	1.910	0.375	5.880	3.429	6	691	ZINC000100826206
38.9	-8.8	-8.2	0.60	1.784	0.000	8.381	0.000	2	692	ZINC000257552146
38.9	-9.6	-8.9	0.58	1.619	0.729	3.969	3.305	6	693	ZINC000257535009
38.9	-10.8	-10.0	0.54	1.879	0.279	9.567	3.580	6	694	ZINC000118936834
38.9	-10.8	-10.3	0.28	1.942	0.495	6.083	4.265	6	695	ZINC000257524960
38.9	-9.5	-9.0	0.68	1.462	0.447	2.678	0.894	6	696	ZINC000100825335
38.9	-11.9	-11.4	0.38	2.332	0.631	6.002	3.052	6	697	ZINC000100783126
38.9	-9.5	-8.7	0.80	1.613	0.513	4.492	3.365	6	698	ZINC000257535008
38.9	-10.8	-10.2	0.31	1.668	0.337	5.786	4.196	6	699	ZINC000257524959
38.9	-10.8	-10.4	0.26	1.850	0.480	7.568	4.380	6	700	ZINC000118936835
38.9	-10.8	-10.4	0.24	1.693	0.585	5.645	4.606	6	701	ZINC000257524958

38.9	-10.8	-10.4	0.24	1.793	0.465	7.639	4.437	6	702	ZINC000118936837
38.8	-12.0	-10.6	0.78	2.373	0.630	7.350	3.657	6	703	ZINC000257566121
38.8	-9.5	-8.9	0.62	1.683	0.600	4.066	3.341	6	704	ZINC000257535006
38.8	-11.6	-11.0	0.41	2.647	0.411	8.996	2.611	6	705	ZINC000257515266
38.8	-10.9	-10.6	0.16	1.841	0.466	5.792	4.136	6	706	ZINC000257463853
38.8	-8.2	-6.7	0.73	1.936	0.245	6.196	1.977	6	707	ZINC000150357732
38.7	-2.6	-1.2	1.35	3.120	0.000	8.683	0.000	2	708	ZINC000150363987
38.7	-11.1	-10.7	0.28	1.869	0.325	5.469	3.290	6	709	ZINC000257395078
38.7	-10.9	-10.6	0.20	1.778	0.445	5.622	4.253	6	710	ZINC000119385591
38.7	-9.5	-8.7	0.75	1.795	0.541	4.355	3.285	6	711	ZINC000100825341
38.7	-10.9	-10.6	0.20	1.802	0.434	5.630	4.254	6	712	ZINC000119385501
38.7	-10.8	-10.5	0.20	2.753	0.667	6.677	3.303	6	713	ZINC000118937024
38.7	-10.9	-10.6	0.24	2.113	0.333	5.982	3.992	6	714	ZINC000257463852
38.7	-9.5	-8.5	0.84	2.130	0.508	6.126	3.723	6	715	ZINC000100825339
38.7	-10.9	-10.6	0.20	2.020	0.228	5.880	4.057	6	716	ZINC000119385707

38.7	-10.6	-10.0	0.29	2.736	0.872	6.569	3.916	6	717	ZINC000118936872
38.6	-11.1	-10.8	0.15	2.075	0.629	6.470	3.598	6	718	ZINC000118937279
38.6	-11.1	-10.7	0.39	1.864	0.319	6.543	3.664	6	719	ZINC000257395079
38.6	-9.4	-8.5	0.80	2.141	0.577	6.131	3.726	6	720	ZINC000257535007
38.6	-5.7	-4.1	1.13	1.978	0.007	7.696	0.022	3	721	ZINC000150368539
38.6	-11.1	-10.8	0.19	1.686	0.357	6.794	3.224	6	722	ZINC000118937278
38.6	-9.4	-8.7	0.69	2.045	0.203	9.293	3.283	6	723	ZINC000100827270
38.6	-9.3	-8.6	0.83	1.982	0.556	3.352	1.443	5	724	ZINC000257410100
38.6	-11.6	-11.2	0.29	2.707	0.427	8.474	2.886	6	725	ZINC000100783129
38.6	-11.1	-10.8	0.18	1.693	0.398	5.228	3.535	6	726	ZINC000118937277
38.6	-10.8	-10.4	0.20	2.655	0.500	6.727	3.312	6	727	ZINC000257540710
38.5	-9.3	-8.8	0.23	1.853	0.184	5.943	4.030	6	728	ZINC000257410102
38.5	-10.9	-10.5	0.24	3.109	0.658	5.833	3.031	6	729	ZINC000257540711
38.5	-11.0	-10.6	0.35	1.913	0.257	6.612	3.338	6	730	ZINC000257389063
38.5	-10.8	-10.3	0.36	2.722	0.582	5.730	3.099	6	731	ZINC000257540712

38.5	-11.0	-10.5	0.38	1.946	0.315	6.567	3.301	6	732	ZINC000118936975
38.5	-9.3	-8.7	0.28	1.919	0.118	9.266	3.269	6	733	ZINC000100827273
38.5	-12.0	-12.0	0.00	0.000	0.000	0.000	0.000	1	734	ZINC000257391277
38.5	-10.8	-10.3	0.30	2.243	0.669	5.444	3.270	6	735	ZINC000118937022
38.5	-10.9	-10.0	0.78	2.185	0.573	2.558	0.597	5	736	ZINC000257395080
38.5	-9.9	-9.0	0.90	3.613	0.000	4.071	0.000	2	737	ZINC000085646807
38.5	-11.1	-10.6	0.59	2.318	0.519	8.103	2.628	6	738	ZINC000118937276
38.5	-9.3	-8.5	0.63	1.449	0.426	2.712	1.032	6	739	ZINC000100825333
38.4	-11.1	-10.6	0.45	1.910	0.134	8.741	2.487	6	740	ZINC000100777784
38.4	-11.1	-10.6	0.25	2.063	0.280	6.873	2.968	6	741	ZINC000118936868
38.4	-9.2	-8.4	0.70	1.925	0.213	7.487	4.139	6	742	ZINC000257410101
38.3	-10.8	-10.3	0.33	2.686	0.553	5.684	3.129	6	743	ZINC000257540713
38.3	-9.2	-8.7	0.25	1.986	0.183	9.215	3.318	6	744	ZINC000100827276
38.3	-10.7	-9.9	0.51	1.944	0.251	3.718	0.850	6	745	ZINC000257404680
38.3	-10.7	-10.0	0.44	1.873	0.118	3.865	0.729	6	746	ZINC000257404679

38.3	-10.7	-10.1	0.39	1.959	0.228	3.690	0.851	6	747	ZINC000257404678
38.3	-6.1	-4.7	1.01	2.252	0.030	7.803	2.486	3	748	ZINC000150363349
38.3	-8.3	-7.3	0.74	2.019	0.683	6.221	2.982	5	749	ZINC000150367993
38.2	-11.6	-10.9	0.66	1.997	0.145	7.252	2.949	6	750	ZINC000257468787
38.2	-7.3	-6.1	0.95	2.063	0.176	5.788	3.369	3	751	ZINC000261499651
38.2	-9.1	-8.6	0.44	2.130	0.100	10.727	0.091	6	752	ZINC000257410099
38.1	-9.7	-8.7	0.79	2.080	0.043	6.410	2.482	4	753	ZINC000257449086
38.1	-12.1	-10.2	0.94	2.247	0.552	4.979	2.662	6	754	ZINC000257515267
38.1	-8.9	-8.7	0.10	1.763	0.296	6.070	3.836	6	755	ZINC000100827277
37.9	-11.3	-10.7	0.38	2.212	0.661	5.497	3.191	6	756	ZINC000004098868
37.8	-12.1	-11.5	0.37	2.664	0.514	8.396	3.059	5	757	ZINC000150353089
37.8	-10.9	-10.2	0.37	2.095	0.108	6.263	2.817	6	758	ZINC000085808842
37.8	-10.4	-9.5	0.64	1.664	0.335	4.135	3.002	6	759	ZINC000150367785
37.7	-10.6	-10.2	0.20	2.369	0.380	8.023	3.815	6	760	ZINC000257611505
37.7	-10.4	-9.3	1.03	1.716	0.548	4.953	3.709	4	761	ZINC000150367791

37.7	-10.6	-10.3	0.17	2.778	0.587	9.329	3.158	6	762	ZINC000118936853
37.7	-10.6	-10.2	0.22	2.536	0.459	9.396	3.139	6	763	ZINC000118936850
37.7	-11.9	-10.9	0.68	2.135	0.603	5.050	2.790	6	764	ZINC000150353464
37.7	-3.8	-1.8	1.01	2.643	0.169	6.385	1.032	5	765	ZINC000150368536
37.7	-10.6	-10.2	0.23	2.648	0.615	9.475	3.157	6	766	ZINC000118936852
37.7	-10.6	-10.2	0.24	2.092	0.815	6.213	4.092	6	767	ZINC000257611606
37.6	-11.4	-10.8	0.36	1.924	0.236	5.595	3.396	6	768	ZINC000257468785
37.6	-9.2	-8.2	0.85	1.759	0.035	10.642	0.083	5	769	ZINC000257421790
37.6	-11.3	-9.8	0.98	1.707	0.052	2.449	0.301	4	770	ZINC000257414051
37.6	-9.2	-8.6	0.37	1.624	0.285	7.309	4.128	6	771	ZINC000100822066
37.6	-9.2	-8.4	0.49	1.486	0.354	5.710	4.106	6	772	ZINC000257421789
37.5	-9.1	-8.4	0.47	1.371	0.363	5.513	4.248	6	773	ZINC000257421791
37.5	-7.9	-6.8	1.15	2.026	0.000	7.843	0.000	2	774	ZINC000257452778
37.5	-9.1	-8.6	0.33	1.538	0.445	7.327	4.144	6	775	ZINC000100822068
37.5	-9.1	-7.9	0.93	1.688	0.254	8.992	3.461	6	776	ZINC000257421792

37.4	-10.3	-10.1	0.15	1.918	0.244	6.087	3.836	6	777	ZINC000257611607
37.4	-10.9	-10.6	0.20	2.669	0.222	8.091	2.634	6	778	ZINC000253500764
37.4	-9.0	-8.5	0.32	1.803	0.116	9.017	3.152	6	779	ZINC000100822069
37.4	-10.9	-10.6	0.22	2.349	0.485	6.749	3.316	6	780	ZINC000118937132
37.3	-10.9	-10.5	0.21	2.440	0.459	6.782	3.191	6	781	ZINC000118914964
37.3	-10.9	-10.6	0.17	2.400	0.529	6.714	3.280	6	782	ZINC000253500763
37.3	-10.9	-10.6	0.24	2.345	0.489	6.762	3.300	6	783	ZINC000118913860
37.3	-10.9	-10.6	0.19	2.402	0.539	6.708	3.277	6	784	ZINC000118937131
37.3	-10.9	-10.6	0.20	2.419	0.554	6.704	3.287	6	785	ZINC000250898577
37.3	-10.7	-9.9	0.67	1.822	0.186	5.699	3.145	6	786	ZINC000100783855
37.3	-10.9	-10.6	0.20	2.125	0.517	5.429	3.291	6	787	ZINC000078817078
37.3	-10.9	-10.6	0.20	2.198	0.648	6.715	3.286	6	788	ZINC000253604296
37.3	-10.9	-10.5	0.21	2.194	0.611	5.352	3.190	6	789	ZINC000253604291
37.2	-10.9	-10.6	0.20	2.391	0.517	6.726	3.267	6	790	ZINC000253604292
37.2	-10.9	-10.6	0.24	2.337	0.487	6.752	3.301	6	791	ZINC000008143844

37.2	-2.4	-1.0	1.19	1.358	0.338	9.782	4.706	4	792	ZINC000095620835
37.2	-10.0	-8.5	1.24	2.866	1.274	6.237	4.082	6	793	ZINC000003830332
37.1	-12.8	-11.3	1.19	1.163	0.092	2.003	0.444	3	794	ZINC000257449088
37.1	-10.7	-10.0	0.30	2.601	1.029	6.415	4.091	6	795	ZINC000257437995
37.1	-8.7	-8.6	0.15	1.597	0.000	2.656	0.000	2	796	ZINC000100822067
37.1	-10.7	-10.1	0.55	1.956	0.463	2.569	0.287	6	797	ZINC000118937130
37.1	-11.0	-10.0	0.54	2.474	0.453	8.657	2.546	6	798	ZINC000257633055
37.1	-10.1	-8.1	1.03	2.135	0.393	6.585	3.189	5	799	ZINC000150352540
37.1	-12.6	-11.1	1.06	2.603	0.352	7.684	3.302	3	800	ZINC000100822710
37.1	-10.7	-10.0	0.38	2.990	0.830	8.057	3.323	6	801	ZINC000257437994
37.0	-9.8	-9.2	0.50	1.555	0.234	2.591	0.894	6	802	ZINC000257478883
37.0	-9.9	-9.6	0.22	1.323	0.356	2.292	0.336	6	803	ZINC000100825325
37.0	-10.7	-10.1	0.32	2.857	0.733	6.861	3.569	6	804	ZINC000118936871
37.0	-9.8	-9.3	0.43	1.571	0.297	2.770	0.797	6	805	ZINC000257478885
37.0	-9.8	-9.2	0.43	1.486	0.411	2.776	0.748	6	806	ZINC000100825330

37.0	-2.8	-2.8	0.00	0.000	0.000	0.000	0.000	1	807	ZINC000085646800
36.9	-9.8	-9.2	0.43	1.498	0.292	2.686	0.785	6	808	ZINC000100825328
36.9	-9.7	-9.2	0.47	1.586	0.337	4.062	3.392	6	809	ZINC000100825323
36.9	-9.8	-9.2	0.45	1.534	0.303	2.774	0.744	6	810	ZINC000257478884
36.9	-10.3	-9.0	0.95	2.802	1.133	4.920	3.288	6	811	ZINC000257448858
36.9	-9.8	-8.9	0.67	1.835	0.291	6.161	3.844	6	812	ZINC000257478882
36.9	-10.6	-10.1	0.24	2.425	0.922	5.174	3.853	6	813	ZINC000118936870
36.8	-10.7	-9.8	0.61	2.590	0.849	6.624	3.725	6	814	ZINC000118936873
36.8	-9.4	-8.4	0.69	2.686	0.206	8.227	1.831	5	815	ZINC000085907233
36.8	-10.9	-10.6	0.20	1.932	0.476	6.712	3.271	6	816	ZINC000030731556
36.8	-10.7	-10.1	0.27	2.796	0.875	6.764	3.722	6	817	ZINC000257437996
36.7	-10.9	-10.5	0.27	1.952	0.487	6.682	3.240	6	818	ZINC000008218968
36.7	-10.7	-9.6	0.87	3.358	0.207	9.721	0.116	6	819	ZINC000257437993
36.7	-12.3	-11.3	0.66	1.679	0.323	6.465	3.802	6	820	ZINC000100776738
36.7	-10.9	-10.5	0.23	2.249	0.226	8.080	2.639	6	821	ZINC000008234226

36.7	-11.2	-10.1	1.09	2.072	0.304	5.964	2.110	5	822	ZINC000150361644
36.6	-11.6	-10.9	0.59	1.753	0.633	2.967	0.588	6	823	ZINC000257503092
36.6	-12.7	-11.7	0.62	2.448	0.636	7.017	2.469	6	824	ZINC000257401056
36.5	-11.9	-10.7	0.75	1.923	0.368	5.527	3.476	6	825	ZINC000100783647
36.4	-2.5	-1.2	1.04	1.240	0.370	7.601	5.580	5	826	ZINC000095620824
36.4	-3.1	-2.2	0.58	1.735	0.548	10.521	4.238	6	827	ZINC000100828710
36.4	-9.7	-9.2	0.55	2.212	0.687	5.460	2.603	6	828	ZINC000100825799
36.3	-6.7	-5.7	0.74	1.622	0.044	7.946	0.071	3	829	ZINC000085907231
36.3	-9.5	-8.2	0.93	2.681	0.271	7.424	1.227	5	830	ZINC000042850970
36.3	-9.7	-8.8	0.95	1.911	0.000	11.337	0.000	2	831	ZINC000257551679
36.3	-9.8	-9.0	0.71	1.490	0.453	5.826	4.323	6	832	ZINC000257551677
36.3	-9.7	-8.8	0.86	1.686	0.612	8.736	3.879	5	833	ZINC000100828177
36.3	-6.3	-6.3	0.00	0.000	0.000	0.000	0.000	1	834	ZINC000150357726
36.3	-10.2	-9.3	0.90	1.605	0.127	2.067	0.100	3	835	ZINC000257481897
36.3	-9.7	-9.1	0.66	1.511	0.441	4.566	3.659	5	836	ZINC000100828178

36.2	-9.6	-8.6	1.01	1.774	0.786	2.736	0.655	5	837	ZINC000100828180
36.2	-11.2	-10.7	0.32	2.369	0.640	8.949	3.615	6	838	ZINC000257503094
36.2	-11.2	-10.5	0.39	2.305	0.444	9.131	3.146	6	839	ZINC000118936937
36.2	-8.6	-8.1	0.31	2.034	0.198	3.599	0.502	6	840	ZINC000257404677
36.2	-3.0	-1.2	0.86	1.625	0.429	10.674	4.086	6	841	ZINC000100828715
36.1	-11.2	-10.7	0.26	2.178	0.625	7.295	4.183	6	842	ZINC000257503095
36.1	-11.1	-10.7	0.24	2.374	0.437	9.129	3.156	6	843	ZINC000118936940
36.1	-3.2	-2.2	0.82	0.928	0.112	13.035	0.048	3	844	ZINC000100827969
36.1	-11.1	-10.6	0.27	2.608	0.428	10.777	0.122	6	845	ZINC000118936939
36.1	-11.1	-10.5	0.75	2.572	0.549	9.268	3.230	6	846	ZINC000118936938
36.1	-11.1	-10.5	0.37	2.605	0.454	10.835	0.159	6	847	ZINC000257503093
36.1	-2.8	-1.6	0.66	1.789	0.628	9.990	4.555	5	848	ZINC000100828714
36.1	-10.9	-9.0	1.12	2.611	0.690	6.099	1.636	6	849	ZINC000086034654
36.1	-9.5	-8.9	0.42	1.865	0.262	5.857	4.290	6	850	ZINC000100828179
36.0	-11.3	-10.1	0.98	2.343	0.221	8.915	1.611	6	851	ZINC000257480100

36.0	-9.4	-7.4	1.10	2.267	0.587	5.324	3.962	5	852	ZINC000257551678
36.0	-11.7	-10.6	0.64	1.785	0.187	3.359	0.694	6	853	ZINC000100777789
36.0	-11.0	-10.5	0.34	1.993	0.328	6.750	3.168	6	854	ZINC000257389065
35.9	-11.0	-10.7	0.22	2.066	0.302	7.967	2.635	6	855	ZINC000257389064
35.9	-10.9	-10.2	0.61	1.980	0.640	2.608	0.810	6	856	ZINC000118936973
35.9	-11.0	-10.6	0.24	2.074	0.347	7.955	2.634	6	857	ZINC000118936974
35.9	-12.7	-11.3	0.77	2.365	0.553	8.027	2.375	6	858	ZINC000100777927
35.8	-9.8	-8.9	0.68	1.774	0.564	4.170	2.951	6	859	ZINC000257394116
35.8	-2.6	-1.2	0.73	1.782	0.576	10.571	4.111	6	860	ZINC000257546729
35.8	-2.4	-1.9	0.50	1.025	0.000	13.066	0.000	2	861	ZINC000232408197
35.8	-8.9	-7.5	1.12	2.194	0.884	7.793	4.289	6	862	ZINC000257528260
35.7	-2.5	-1.3	0.67	1.713	0.568	10.599	4.143	6	863	ZINC000257546728
35.7	-8.8	-6.8	1.04	2.366	0.948	7.204	4.235	5	864	ZINC000100825203
35.7	-11.0	-9.4	1.07	2.103	0.310	7.250	3.313	6	865	ZINC000100783817
35.7	-8.8	-7.2	1.18	1.842	1.141	5.900	4.533	6	866	ZINC000257528262

35.7	-2.9	-1.5	1.20	1.246	0.315	9.462	5.155	4	867	ZINC000257578216
35.7	-8.8	-7.7	1.22	2.232	0.898	9.419	3.725	6	868	ZINC000257528261
35.7	-9.8	-9.0	0.69	2.136	0.167	10.208	0.268	6	869	ZINC000257394118
35.7	-9.8	-9.1	0.48	1.767	0.344	2.839	0.136	6	870	ZINC000100829038
35.7	-8.8	-8.6	0.22	1.299	0.584	2.304	0.308	3	871	ZINC000100825202
35.7	-9.8	-9.1	0.49	1.591	0.481	2.791	0.363	6	872	ZINC000257394117
35.7	-2.8	-1.5	1.18	1.209	0.309	9.545	4.810	4	873	ZINC000100827974
35.7	-8.5	-7.1	1.07	2.327	0.274	7.633	2.695	6	874	ZINC000150357038
35.7	-9.8	-9.2	0.46	2.546	0.043	9.561	0.256	3	875	ZINC000257517754
35.7	-8.8	-7.9	1.07	1.792	0.932	2.643	0.760	4	876	ZINC000100825199
35.6	-10.9	-10.4	0.23	2.225	0.336	6.999	2.964	6	877	ZINC000118936861
35.6	-8.6	-7.4	0.91	2.316	0.708	3.368	1.357	4	878	ZINC000085907193
35.6	-9.7	-9.0	0.60	1.850	0.569	8.557	3.334	6	879	ZINC000257394119
35.6	-10.9	-10.4	0.25	2.257	0.367	6.987	2.960	6	880	ZINC000253501034
35.6	-9.8	-9.2	0.53	1.628	0.520	4.157	2.908	6	881	ZINC000100829037

35.6	-8.7	-7.6	1.18	1.862	1.048	5.266	4.295	4	882	ZINC000100825198
35.6	-10.6	-10.2	0.24	2.077	0.785	7.652	4.277	6	883	ZINC000257611504
35.6	-9.8	-9.2	0.46	1.742	0.365	5.516	3.577	6	884	ZINC000100829036
35.6	-8.7	-7.3	1.07	2.600	0.750	3.832	1.024	3	885	ZINC000257528263
35.6	-2.9	-2.8	0.15	0.858	0.000	13.094	0.000	2	886	ZINC000257578222
35.5	-11.1	-10.6	0.25	2.079	0.304	6.859	2.973	6	887	ZINC000257374920
35.5	-11.1	-10.5	0.27	2.112	0.338	5.665	3.043	6	888	ZINC000257374921
35.5	-11.1	-10.6	0.24	2.065	0.275	6.886	2.960	6	889	ZINC000118936867
35.5	-10.9	-10.4	0.25	2.097	0.402	6.869	3.114	6	890	ZINC000253501027
35.5	-10.9	-10.6	0.18	1.742	0.378	4.026	3.296	6	891	ZINC000119385362
35.5	-10.9	-10.4	0.25	2.212	0.333	7.001	2.961	6	892	ZINC000118936858
35.5	-12.3	-11.9	0.25	1.899	0.295	6.989	3.603	6	893	ZINC000100777787
35.5	-11.1	-10.5	0.27	2.115	0.335	5.666	3.043	6	894	ZINC000257374922
35.5	-11.1	-10.5	0.42	2.102	0.325	5.648	3.059	6	895	ZINC000257374923
35.5	-6.7	-6.6	0.06	1.885	0.456	3.311	0.621	6	896	ZINC000257594433

35.5	-2.7	-1.2	1.18	1.104	0.280	8.661	5.260	6	897	ZINC000100827970
35.4	-12.7	-11.8	0.62	2.831	0.290	8.302	1.795	6	898	ZINC000257515265
35.4	-2.7	-1.1	0.93	1.144	0.241	7.673	5.309	5	899	ZINC000257407457
35.4	-10.9	-10.4	0.25	2.222	0.337	6.987	2.972	6	900	ZINC000118936860
35.4	-12.1	-11.0	0.83	2.039	0.821	5.327	3.683	6	901	ZINC000100781864
35.4	-9.9	-8.5	0.83	2.596	0.514	8.160	3.062	6	902	ZINC000257483003
35.4	-11.1	-10.6	0.24	2.068	0.284	6.884	2.961	6	903	ZINC000118936869
35.4	-10.9	-10.4	0.27	2.146	0.392	6.782	3.218	6	904	ZINC000253501031
35.4	-2.5	-1.1	1.04	1.060	0.236	5.936	5.096	4	905	ZINC000100822717
35.4	-2.7	-1.2	1.03	1.327	0.328	6.144	4.829	4	906	ZINC000257407459
35.4	-2.6	-1.3	0.89	1.091	0.101	9.471	5.057	4	907	ZINC000257407460
35.4	-11.1	-10.5	0.27	2.127	0.338	5.674	3.038	6	908	ZINC000118936866
35.4	-10.9	-9.9	0.81	2.229	0.362	8.007	2.658	6	909	ZINC000253501030
35.3	-2.0	-0.8	0.65	1.546	0.424	10.465	4.368	6	910	ZINC000257546726
35.3	-2.6	-1.3	0.88	1.195	0.291	9.377	5.080	4	911	ZINC000257407458

35.3	-12.6	-11.4	0.80	1.878	0.472	5.082	1.635	6	912	ZINC000257616269
35.3	-2.0	-1.0	0.49	1.707	0.579	10.457	4.346	6	913	ZINC000100828712
35.3	-12.1	-11.5	0.76	2.161	0.219	8.063	2.740	5	914	ZINC000100783644
35.3	-2.8	-1.8	1.23	1.504	0.817	8.345	4.758	3	915	ZINC000100822716
35.3	-8.6	-6.9	0.88	2.167	0.278	5.296	0.981	6	916	ZINC000150367995
35.3	-12.6	-11.6	0.48	1.980	0.375	4.424	2.585	6	917	ZINC000257613187
35.2	-10.1	-9.7	0.25	2.620	0.685	7.717	4.100	6	918	ZINC000118936987
35.2	-6.4	-6.2	0.19	0.937	0.677	3.052	0.524	6	919	ZINC000100826499
35.2	-11.1	-10.6	0.37	1.907	0.366	8.254	3.154	6	920	ZINC000257386811
35.2	-11.1	-10.5	0.50	2.031	0.415	6.905	3.346	6	921	ZINC000257386809
35.2	-10.1	-9.7	0.24	2.353	0.444	6.282	3.989	6	922	ZINC000118936988
35.2	-6.4	-6.3	0.12	0.728	0.583	2.894	0.587	6	923	ZINC000100826500
35.2	-10.1	-9.8	0.18	2.953	0.472	10.948	0.253	6	924	ZINC000118936986
35.2	-6.4	-6.2	0.11	1.131	0.583	2.806	0.457	6	925	ZINC000100826497
35.2	-6.4	-6.3	0.08	0.729	0.544	2.550	0.418	6	926	ZINC000100826496

35.2	-10.1	-9.8	0.22	2.600	0.430	9.427	3.101	6	927	ZINC000257556981
35.2	-10.1	-9.8	0.23	2.495	0.372	9.547	3.162	6	928	ZINC000257556984
35.2	-6.5	-6.2	0.14	0.999	0.682	3.111	0.514	6	929	ZINC000257594434
35.1	-6.3	-6.2	0.05	1.470	0.565	3.487	0.328	6	930	ZINC000257594436
35.1	-10.0	-9.7	0.16	1.806	0.692	4.105	3.459	6	931	ZINC000257556982
35.1	-12.6	-12.0	0.29	1.793	0.078	6.716	3.049	6	932	ZINC000100776735
35.1	-10.0	-9.8	0.19	2.431	0.423	7.798	4.097	6	933	ZINC000118936985
35.1	-6.3	-6.2	0.07	0.824	0.563	3.024	0.459	6	934	ZINC000257594435
35.0	-2.4	-1.3	1.05	2.247	0.000	12.887	0.000	2	935	ZINC000100822718
35.0	-2.4	-0.9	1.20	1.042	0.165	10.353	4.804	5	936	ZINC000257578219
34.9	-9.5	-8.2	0.95	2.285	0.384	7.169	2.480	6	937	ZINC000150368679
34.9	-10.7	-9.6	0.88	3.230	1.663	6.372	3.727	6	938	ZINC000257448856
34.9	-11.6	-10.5	0.64	3.018	0.787	6.480	2.602	6	939	ZINC000095619893
34.9	-8.7	-7.8	0.95	1.891	0.000	8.804	0.000	2	940	ZINC000085907199
34.9	-9.5	-8.0	1.08	2.182	0.691	5.728	2.907	6	941	ZINC000257540267

34.8	-10.1	-9.6	0.28	2.535	0.526	7.902	3.657	6	942	ZINC000257390406
34.8	-10.0	-9.7	0.17	2.882	0.441	10.876	0.194	6	943	ZINC000257390405
34.8	-10.0	-9.6	0.20	2.305	0.861	9.053	3.703	6	944	ZINC000257390404
34.8	-9.5	-8.5	0.81	2.168	0.517	6.010	2.821	6	945	ZINC000150368680
34.8	-10.8	-9.6	0.93	2.719	1.293	5.032	3.007	6	946	ZINC000257578150
34.8	-9.5	-8.5	0.77	1.860	0.511	4.733	3.049	6	947	ZINC000150368676
34.8	-12.8	-12.1	0.62	1.766	0.480	3.441	1.763	4	948	ZINC000085511003
34.8	-1.5	-1.0	0.40	1.724	0.563	10.581	4.072	6	949	ZINC000257546727
34.8	-10.0	-9.7	0.19	2.454	0.376	8.089	3.617	6	950	ZINC000118937016
34.7	-10.7	-10.0	0.40	1.955	0.678	5.442	3.550	6	951	ZINC000257471918
34.7	-10.1	-10.1	0.00	0.000	0.000	0.000	0.000	1	952	ZINC000079269160
34.7	-12.0	-11.4	0.41	2.415	0.575	6.133	3.018	6	953	ZINC000257566124
34.7	-7.5	-5.8	0.96	2.718	0.170	7.998	2.156	5	954	ZINC000261499650
34.7	-11.6	-9.8	1.14	1.848	0.313	4.421	2.829	6	955	ZINC000257496517
34.6	-9.8	-9.1	0.53	2.000	0.290	4.287	2.861	6	956	ZINC000100829040

34.6	-9.8	-9.6	0.14	2.093	0.483	6.121	3.585	6	957	ZINC000118937017
34.5	-10.6	-9.3	0.99	2.052	0.570	3.541	0.933	6	958	ZINC000118937091
34.5	-9.9	-8.9	1.01	2.618	0.578	6.662	3.467	6	959	ZINC000021981222
34.5	-10.6	-9.2	1.16	3.900	1.836	8.642	3.830	5	960	ZINC000257578149
34.5	-10.6	-9.4	0.88	1.729	0.384	2.874	0.741	6	961	ZINC000118937093
34.5	-11.1	-10.5	0.49	1.941	0.213	7.161	3.776	6	962	ZINC000257507763
34.5	-11.1	-10.3	0.68	2.127	0.511	6.082	3.487	6	963	ZINC000257507762
34.5	-1.8	-0.4	0.93	1.746	0.520	5.428	4.384	5	964	ZINC000100827966
34.5	-10.6	-9.8	0.66	2.488	1.410	4.681	3.152	6	965	ZINC000257448859
34.5	-11.1	-10.2	0.48	2.669	0.581	10.516	0.280	6	966	ZINC000257507764
34.5	-11.1	-10.3	0.45	2.778	0.562	10.546	0.213	6	967	ZINC000257507765
34.4	-9.4	-8.2	1.25	1.677	0.000	7.741	0.000	2	968	ZINC000085907240
34.4	-11.9	-10.3	0.78	2.475	0.605	8.516	2.805	6	969	ZINC000100777655
34.4	-9.0	-8.6	0.48	3.163	0.062	4.362	0.689	3	970	ZINC000100822311
34.4	-11.2	-10.8	0.32	2.101	0.280	7.118	2.745	6	971	ZINC000100777788

34.4	-10.4	-9.7	0.58	3.038	1.625	6.286	3.707	6	972	ZINC000118937092
34.4	-12.3	-11.9	0.23	1.870	0.654	5.400	3.582	6	973	ZINC000015121640
34.3	-7.7	-6.4	0.80	3.371	0.258	12.116	0.240	6	974	ZINC000257551680
34.3	-10.4	-9.3	0.85	3.389	1.655	6.443	3.796	6	975	ZINC000257448857
34.3	-10.3	-9.7	0.56	2.921	1.934	5.969	4.085	6	976	ZINC000257578154
34.2	-10.7	-10.2	0.40	1.937	0.685	5.327	3.646	6	977	ZINC000100828775
34.2	-10.7	-10.1	0.53	2.043	0.689	5.404	3.579	6	978	ZINC000257471916
34.2	-11.2	-10.4	0.60	1.966	0.332	4.530	2.260	5	979	ZINC000257408653
34.2	-10.4	-9.4	0.62	3.231	1.742	6.226	3.902	6	980	ZINC000257578151
34.2	-9.8	-9.4	0.26	1.585	0.393	7.406	3.709	6	981	ZINC000150366167
34.2	-10.2	-9.7	0.25	2.171	0.861	7.396	4.090	6	982	ZINC000095618133
34.2	-10.7	-9.5	0.70	2.062	0.890	4.106	2.434	6	983	ZINC000100825800
34.2	-10.4	-9.5	0.73	2.482	1.288	4.879	3.097	6	984	ZINC000118937090
34.0	-10.7	-10.1	0.52	2.160	0.765	6.839	3.866	6	985	ZINC000100828772
34.0	-9.3	-8.6	0.52	2.509	0.793	8.836	3.359	6	986	ZINC000257752816

33.9	-12.6	-11.4	0.77	2.598	0.628	5.921	3.263	6	987	ZINC000085774752
33.9	-10.7	-10.1	0.39	3.221	0.256	8.633	2.751	6	988	ZINC000150361550
33.9	-10.7	-10.3	0.34	2.950	0.424	7.596	2.758	6	989	ZINC000257440478
33.9	-10.5	-10.3	0.27	2.384	0.497	4.426	2.329	6	990	ZINC000150362039
33.9	-11.5	-10.8	0.65	2.813	0.498	6.299	1.464	6	991	ZINC000257458620
33.9	-10.4	-9.5	0.44	2.020	0.166	6.801	2.957	6	992	ZINC000257386810
33.8	-10.8	-10.3	0.28	1.706	0.377	7.549	4.358	6	993	ZINC000118936836
33.8	-9.9	-9.7	0.18	1.845	0.081	7.373	3.597	6	994	ZINC000150366170
33.8	-9.9	-9.8	0.15	1.644	0.388	5.734	3.797	6	995	ZINC000150366172
33.7	-9.9	-9.6	0.16	1.608	0.377	5.753	3.832	6	996	ZINC000257495593
33.7	-9.8	-9.5	0.20	1.640	0.368	7.395	3.637	6	997	ZINC000257495592
33.7	-9.8	-9.4	0.25	1.688	0.302	7.241	3.785	6	998	ZINC000257495594
33.7	-10.4	-9.9	0.53	2.883	0.427	6.803	3.168	6	999	ZINC000257440479
33.7	-10.4	-9.9	0.47	2.676	0.511	5.590	2.638	6	1000	ZINC000257440480
33.7	-10.7	-10.1	0.46	2.187	0.795	6.750	3.793	6	1001	ZINC000100828771

33.6	-10.4	-9.7	0.56	2.470	0.349	4.283	0.551	6	1002	ZINC000085814987
33.6	-10.7	-9.4	1.07	2.162	0.307	5.607	3.350	6	1003	ZINC000085907222
33.6	-10.4	-10.0	0.39	2.422	0.310	5.692	2.489	6	1004	ZINC000150361554
33.5	-11.4	-10.7	0.41	1.683	0.283	3.379	1.218	6	1005	ZINC000257604306
33.5	-11.1	-10.1	1.05	2.442	0.000	8.124	0.000	2	1006	ZINC000073331412
33.5	-11.1	-9.5	0.99	2.998	0.262	9.799	0.186	4	1007	ZINC000086034659
33.5	-9.9	-9.7	0.19	1.690	0.389	7.040	4.065	6	1008	ZINC000257495591
33.5	-1.0	0.2	0.89	1.892	0.739	5.505	3.881	6	1009	ZINC000100822719
33.5	-12.7	-11.9	0.50	1.778	0.782	5.989	3.707	6	1010	ZINC000100777928
33.5	-12.3	-11.0	0.74	1.946	0.398	5.476	3.517	6	1011	ZINC000257414053
33.5	-10.2	-9.4	0.51	2.318	0.357	6.103	3.611	6	1012	ZINC000150361556
33.4	-12.4	-11.5	0.56	1.902	0.307	6.875	2.816	6	1013	ZINC000085741871
33.4	-11.0	-11.0	0.00	0.000	0.000	0.000	0.000	1	1014	ZINC000028115894
33.4	-9.6	-9.3	0.20	1.474	0.294	5.528	3.720	6	1015	ZINC000150366175
33.4	-10.7	-9.3	0.85	2.281	0.597	5.631	3.071	6	1016	ZINC000100825803

33.3	-10.8	-9.9	0.62	1.906	0.303	5.688	3.404	6	1017	ZINC000257496520
33.3	-10.7	-9.4	0.99	1.644	0.652	4.577	3.151	6	1018	ZINC000100825802
33.2	-11.4	-10.7	0.46	1.841	0.411	4.181	1.131	6	1019	ZINC000100824392
33.2	-6.2	-5.4	0.80	2.171	0.220	7.320	0.304	5	1020	ZINC000095911652
33.2	-10.8	-10.4	0.22	1.690	0.572	5.646	4.604	6	1021	ZINC000257524961
33.1	-11.2	-10.3	0.48	1.982	0.562	6.347	3.559	6	1022	ZINC000257483678
33.1	-11.1	-10.6	0.31	2.178	0.473	9.757	0.335	6	1023	ZINC000257551199
33.1	-11.1	-10.5	0.45	2.253	0.459	8.449	2.847	6	1024	ZINC000257551197
33.1	-11.4	-10.8	0.32	1.748	0.339	3.271	1.265	6	1025	ZINC000100824390
33.1	-13.4	-12.1	0.76	2.187	0.472	4.872	2.858	6	1026	ZINC000100824459
33.1	-11.2	-10.6	0.35	1.739	0.535	3.321	1.398	6	1027	ZINC000100827930
33.1	-11.1	-10.6	0.28	2.067	0.559	8.102	3.188	6	1028	ZINC000257551196
33.1	-11.4	-10.6	0.50	1.947	0.446	5.330	2.914	6	1029	ZINC000100824391
33.1	-11.4	-10.8	0.38	1.543	0.409	3.527	1.490	6	1030	ZINC000100824387
33.0	-11.4	-10.7	0.41	1.899	0.409	3.906	1.121	6	1031	ZINC000257604307

33.0	-12.7	-11.5	0.82	2.212	0.562	7.083	3.363	6	1032	ZINC000257615333
33.0	-11.1	-10.6	0.30	2.351	0.554	9.641	0.308	6	1033	ZINC000100776721
33.0	-8.6	-7.8	0.48	2.130	0.351	5.531	2.717	6	1034	ZINC000085907216
32.9	-10.9	-9.7	0.68	2.192	0.244	5.031	0.412	6	1035	ZINC000257426831
32.9	-11.4	-10.7	0.44	2.485	0.320	5.448	1.838	6	1036	ZINC000257612631
32.9	-10.7	-10.1	0.49	2.576	0.255	8.426	3.114	6	1037	ZINC000257506532
32.9	-8.3	-7.0	1.15	1.991	0.094	8.811	0.074	3	1038	ZINC000085907189
32.9	-10.9	-9.6	1.30	2.055	0.000	9.322	0.000	2	1039	ZINC000257517756
32.9	-12.7	-11.5	0.65	2.431	0.718	6.540	3.228	6	1040	ZINC000257401055
32.9	-11.1	-10.6	0.31	2.067	0.593	8.234	3.300	6	1041	ZINC000257551198
32.8	-12.9	-12.3	0.41	2.633	0.707	6.957	3.397	6	1042	ZINC000100781862
32.8	-10.5	-10.5	0.00	0.000	0.000	0.000	0.000	1	1043	ZINC000257552147
32.8	-12.7	-11.8	0.50	2.390	0.702	7.891	2.154	6	1044	ZINC000100777923
32.7	-12.7	-11.6	0.75	2.260	0.597	9.133	0.248	6	1045	ZINC000100777926
32.7	-11.4	-10.9	0.62	2.926	0.489	8.105	1.992	6	1046	ZINC000150353094

32.7	-10.5	-9.7	0.52	2.248	0.788	7.133	3.924	6	1047	ZINC000257604305
32.7	-10.5	-10.1	0.23	2.467	0.680	6.063	3.344	6	1048	ZINC000100774383
32.7	-12.6	-11.7	0.64	1.989	0.383	6.543	3.036	6	1049	ZINC000100772796
32.7	-10.8	-10.0	0.42	2.346	0.207	6.075	2.243	6	1050	ZINC000257426830
32.7	-9.6	-8.3	0.68	2.266	0.431	5.703	2.436	6	1051	ZINC000086034594
32.7	-11.0	-10.1	0.94	2.747	0.024	8.133	2.494	4	1052	ZINC000257534241
32.7	-10.8	-9.8	0.62	2.255	0.284	6.147	2.178	6	1053	ZINC000150361576
32.6	-11.4	-10.7	0.44	2.351	0.148	4.409	0.829	6	1054	ZINC000100825468
32.6	-11.4	-10.8	0.49	2.156	0.393	7.995	1.422	6	1055	ZINC000257458622
32.6	-10.6	-10.3	0.14	1.910	0.555	5.798	3.563	6	1056	ZINC000257506533
32.6	-10.1	-8.5	1.09	1.979	0.610	5.380	3.462	6	1057	ZINC000085907195
32.6	-10.1	-8.5	1.12	2.616	0.362	10.168	0.159	5	1058	ZINC000095619921
32.5	-11.4	-10.5	0.47	2.416	0.240	4.798	0.842	6	1059	ZINC000257516669
32.5	-11.4	-10.5	0.59	2.038	0.462	6.539	3.424	6	1060	ZINC000257516671
32.5	-11.4	-10.1	0.72	2.259	0.208	7.899	3.410	6	1061	ZINC000100825473

32.5	-10.5	-10.0	0.36	2.720	0.483	7.336	3.438	6	1062	ZINC000257506535
32.5	-9.5	-8.6	0.75	2.198	0.492	6.107	2.746	6	1063	ZINC000257540265
32.5	-10.5	-10.0	0.33	2.559	0.502	7.241	3.502	6	1064	ZINC000100774381
32.4	-12.4	-11.6	0.51	2.385	0.752	7.915	2.117	6	1065	ZINC000100777938
32.4	-10.6	-10.0	0.46	2.887	0.556	8.899	2.551	6	1066	ZINC000257506534
32.4	-10.5	-9.6	0.75	2.096	1.118	4.202	2.358	6	1067	ZINC000100829255
32.4	-9.7	-8.4	1.25	1.843	0.000	3.661	0.000	2	1068	ZINC000150367986
32.4	-11.4	-10.6	0.52	2.400	0.177	5.848	2.449	6	1069	ZINC000257516670
32.4	-14.8	-13.8	0.86	2.339	0.767	6.922	2.219	4	1070	ZINC000085852994
32.4	-11.0	-10.7	0.17	3.026	0.900	6.745	2.650	6	1071	ZINC000095619137
32.4	-9.3	-8.2	0.85	1.952	0.671	7.388	4.091	6	1072	ZINC000100825359
32.4	-11.2	-10.3	0.61	2.114	0.423	4.489	2.006	6	1073	ZINC000150361646
32.4	-8.2	-6.8	0.91	2.459	0.277	5.988	1.652	5	1074	ZINC000095669920
32.4	-10.5	-10.1	0.27	2.541	0.839	7.091	3.745	6	1075	ZINC000100774384
32.3	-9.5	-8.8	0.57	2.108	0.679	6.998	2.704	6	1076	ZINC000150368682

32.3	-10.2	-9.7	0.26	2.134	0.626	7.928	4.192	6	1077	ZINC000085552284
32.3	-10.6	-10.0	0.31	1.842	0.613	2.966	0.886	6	1078	ZINC000118936851
32.3	-9.2	-8.1	0.88	2.336	0.950	7.589	4.096	6	1079	ZINC000100825356
32.2	-11.2	-10.3	0.61	2.119	0.420	4.555	1.977	6	1080	ZINC000150361642
32.2	-7.4	-6.6	0.80	2.209	0.000	6.345	0.000	2	1081	ZINC000261499652
32.2	-9.5	-8.5	0.77	2.162	0.461	6.061	2.778	6	1082	ZINC000257540266
32.2	-11.2	-10.2	1.19	2.207	0.236	5.612	2.373	4	1083	ZINC000230073923
32.2	-11.2	-9.8	1.18	2.080	0.274	6.349	2.032	6	1084	ZINC000257408652
32.2	-11.2	-9.7	1.13	1.923	0.195	5.625	2.658	6	1085	ZINC000150361640
32.2	-10.2	-9.7	0.45	2.286	0.546	6.062	3.223	6	1086	ZINC000100774378
32.1	-11.2	-10.9	0.24	2.232	0.285	4.221	1.590	3	1087	ZINC000257408651
32.1	-10.4	-9.7	0.36	2.552	0.377	8.136	2.950	6	1088	ZINC000257604308
32.1	-10.1	-9.5	0.34	1.806	0.187	6.474	3.698	6	1089	ZINC000100824476
32.0	-8.9	-8.6	0.23	2.261	0.788	8.697	3.424	6	1090	ZINC000100825357
32.0	-10.1	-9.7	0.36	2.307	0.343	5.367	2.529	6	1091	ZINC000150361572

31.9	-10.0	-9.7	0.20	2.458	0.380	8.063	3.756	6	1092	ZINC000257556983
31.9	-10.5	-9.5	0.56	2.217	0.719	5.438	2.557	6	1093	ZINC000100829258
31.9	-10.5	-9.5	0.54	2.386	0.710	4.347	2.229	6	1094	ZINC000100829256
31.8	-10.2	-9.6	0.30	2.584	0.446	8.278	3.676	6	1095	ZINC000085552287
31.8	-10.5	-9.5	0.56	2.218	0.728	5.440	2.557	6	1096	ZINC000100829257
31.8	-11.4	-10.0	0.86	2.298	0.281	6.517	2.668	6	1097	ZINC000100783850
31.8	-10.2	-9.6	0.28	2.275	0.163	6.697	3.738	6	1098	ZINC000085552279
31.8	-9.5	-8.6	0.68	2.205	0.474	7.148	2.433	6	1099	ZINC000257540264
31.8	-10.7	-10.1	0.46	2.195	0.798	6.720	3.811	6	1100	ZINC000257471917
31.7	-9.0	-8.4	0.41	3.054	0.136	7.098	2.777	5	1101	ZINC000100822314
31.7	-11.2	-10.6	0.40	1.843	0.508	6.022	3.280	6	1102	ZINC000150368561
31.7	-10.6	-9.5	0.90	1.927	1.093	3.893	1.884	6	1103	ZINC000004098553
31.7	-8.9	-8.5	0.40	3.062	0.156	6.190	2.580	4	1104	ZINC000100822316
31.7	-8.9	-8.5	0.42	3.060	0.156	6.155	2.594	4	1105	ZINC000100822313
31.7	-9.3	-8.8	0.28	3.035	0.124	9.032	1.853	6	1106	ZINC000085907187

31.7	-10.2	-9.6	0.31	2.462	0.301	9.742	3.046	6	1107	ZINC000085552281
31.6	-12.8	-11.5	0.94	1.781	0.430	4.122	2.894	6	1108	ZINC000100826204
31.6	-8.7	-7.8	0.57	2.510	0.658	6.339	0.350	6	1109	ZINC000085637240
31.6	-12.0	-11.6	0.39	2.053	0.436	7.973	2.267	6	1110	ZINC000100772785
31.6	-12.6	-11.6	0.69	2.539	0.547	6.761	3.458	6	1111	ZINC000257804823
31.6	-8.8	-7.0	1.03	2.750	0.654	7.570	2.674	5	1112	ZINC000085907221
31.5	-12.1	-11.1	0.46	2.408	0.667	4.863	2.734	6	1113	ZINC000044358570
31.5	-11.5	-11.1	0.41	1.897	0.292	5.897	3.141	6	1114	ZINC000257613188
31.5	-8.4	-7.5	0.69	2.461	0.652	8.955	3.447	6	1115	ZINC000257752815
31.5	-11.9	-10.9	0.72	1.991	0.432	7.909	2.950	6	1116	ZINC000100822159
31.5	-9.7	-8.7	0.48	2.730	0.903	8.439	3.219	6	1117	ZINC000100828600
31.5	-8.7	-7.5	1.05	2.293	0.210	6.726	0.883	6	1118	ZINC000095098884
31.4	-11.2	-10.3	0.45	2.165	0.227	4.557	0.789	6	1119	ZINC000100827932
31.4	-10.4	-9.8	0.58	1.045	0.303	2.093	0.247	3	1120	ZINC000100825317
31.3	-10.7	-10.4	0.21	1.779	0.449	5.714	4.374	6	1121	ZINC000257495941

31.3	-11.2	-10.2	0.56	2.304	0.267	6.946	3.075	6	1122	ZINC000257483679
31.3	-10.4	-9.5	1.05	2.354	0.291	8.512	2.483	6	1123	ZINC000257452352
31.3	-11.5	-10.8	0.38	2.366	0.516	4.882	2.950	6	1124	ZINC000257390901
31.3	-8.4	-8.3	0.10	3.109	0.119	5.837	2.339	6	1125	ZINC000257752817
31.3	-10.4	-9.9	0.36	0.695	0.113	2.135	0.372	4	1126	ZINC000100825310
31.2	-10.0	-9.7	0.22	2.872	0.965	6.213	3.722	6	1127	ZINC000150340218
31.2	-11.7	-10.7	0.71	1.630	0.641	5.734	3.619	5	1128	ZINC000238760838
31.2	-10.4	-10.1	0.30	1.212	0.000	2.275	0.000	2	1129	ZINC000257423919
31.2	-10.0	-8.8	1.25	2.199	0.306	6.433	3.816	3	1130	ZINC000085907186
31.2	-10.4	-9.8	0.42	0.874	0.184	2.224	0.341	4	1131	ZINC000257423920
31.2	-10.0	-9.7	0.18	2.343	0.460	7.711	4.057	6	1132	ZINC000085552271
31.2	-10.9	-10.6	0.19	1.793	0.434	5.622	4.258	6	1133	ZINC000257463851
31.1	-10.8	-10.5	0.18	2.001	0.548	6.419	3.783	6	1134	ZINC000100783856
31.1	-10.1	-9.7	0.26	2.210	0.556	6.340	3.793	6	1135	ZINC000257483680
31.1	-12.5	-11.8	0.42	1.844	0.243	5.128	3.429	6	1136	ZINC000100822158

31.1	-10.4	-9.9	0.50	1.268	0.000	2.935	0.000	2	1137	ZINC000100825319
31.1	-11.5	-10.8	0.32	2.364	0.664	4.964	2.940	6	1138	ZINC000257390903
31.0	-9.9	-9.5	0.18	2.666	1.202	4.949	2.964	6	1139	ZINC000040165214
31.0	-10.4	-9.9	0.36	0.696	0.107	2.135	0.368	4	1140	ZINC000257423922
31.0	-11.5	-10.7	0.39	2.351	0.660	5.824	3.516	6	1141	ZINC000118937427
31.0	-12.1	-11.5	0.39	1.968	0.778	4.428	2.767	6	1142	ZINC000100777657
31.0	-11.5	-10.9	0.27	1.940	0.771	3.168	1.296	6	1143	ZINC000118937429
30.9	-10.7	-10.4	0.33	2.805	0.475	6.219	2.424	6	1144	ZINC000150361552
30.9	-10.4	-9.8	0.47	1.066	0.342	2.380	0.436	4	1145	ZINC000257423921
30.8	-11.5	-10.8	0.33	2.574	0.222	4.101	0.861	6	1146	ZINC000257390902
30.8	-13.5	-11.8	0.95	1.958	0.519	4.957	2.476	6	1147	ZINC000257616270
30.8	-10.8	-10.3	0.24	2.009	1.024	4.492	3.262	6	1148	ZINC000256079354
30.8	-11.5	-10.9	0.32	2.634	0.221	4.444	0.858	6	1149	ZINC000118937428
30.8	-10.6	-9.6	0.68	1.790	1.063	3.176	1.064	6	1150	ZINC000095617891
30.8	-11.4	-10.6	0.84	1.785	0.173	2.948	0.394	6	1151	ZINC000257613186

30.8	-10.8	-10.3	0.26	1.708	0.605	2.935	0.506	6	1152	ZINC000256079356
30.7	-10.9	-10.2	0.65	2.860	0.000	4.593	0.000	2	1153	ZINC000257376561
30.7	-10.6	-9.6	0.74	1.730	1.130	3.383	1.646	6	1154	ZINC000095617892
30.7	-10.9	-10.2	0.65	2.872	0.000	4.601	0.000	2	1155	ZINC000257376562
30.7	-10.9	-10.2	0.65	2.859	0.000	4.593	0.000	2	1156	ZINC000100829920
30.7	-8.0	-8.0	0.00	0.000	0.000	0.000	0.000	1	1157	ZINC000150368540
30.7	-13.2	-12.4	0.90	2.564	0.323	4.390	0.251	6	1158	ZINC000100781620
30.6	-10.9	-10.1	0.68	2.423	0.986	3.741	1.457	4	1159	ZINC000100829922
30.6	-10.9	-10.2	0.65	2.859	0.000	4.592	0.000	2	1160	ZINC000100829926
30.6	-10.1	-9.2	0.88	1.634	0.210	5.928	3.807	6	1161	ZINC000100777899
30.6	-10.9	-9.9	0.76	2.947	0.095	4.686	0.124	3	1162	ZINC000257376560
30.6	-10.5	-9.5	0.71	1.833	1.051	3.272	1.208	6	1163	ZINC000253532196
30.6	-10.6	-9.6	0.76	1.734	1.144	3.418	1.638	6	1164	ZINC000095617893
30.6	-11.5	-10.8	0.39	2.651	0.242	5.344	2.315	6	1165	ZINC000118937430
30.6	-10.9	-10.2	0.65	2.863	0.000	4.563	0.000	2	1166	ZINC000257376563

30.6	-10.9	-9.9	0.63	2.373	1.050	3.525	1.738	4	1167	ZINC000100829924
30.6	-10.1	-9.7	0.21	2.536	0.384	6.797	3.698	6	1168	ZINC000085552265
30.5	-10.5	-9.5	1.03	2.277	0.599	6.976	3.237	6	1169	ZINC000257549070
30.5	-11.0	-10.4	0.43	2.346	0.151	4.506	0.914	6	1170	ZINC000100825428
30.5	-10.7	-9.9	0.49	2.364	0.709	8.316	3.098	6	1171	ZINC000100828769
30.5	-11.5	-10.9	0.29	2.286	0.491	3.554	1.095	6	1172	ZINC000257390900
30.5	-9.9	-9.6	0.26	2.394	1.399	4.909	2.988	6	1173	ZINC000040165215
30.5	-11.0	-10.2	0.59	2.419	0.128	7.022	2.916	6	1174	ZINC000100825425
30.5	-13.4	-12.3	0.71	2.640	0.394	4.911	2.315	6	1175	ZINC000257394166
30.5	-11.0	-10.0	0.51	2.302	0.153	7.171	2.724	6	1176	ZINC000257460260
30.5	-11.0	-10.2	0.43	2.062	0.471	5.496	2.830	6	1177	ZINC000100825421
30.4	-10.9	-10.3	0.29	2.427	1.038	6.190	3.869	6	1178	ZINC000118915335
30.4	-13.3	-12.4	0.63	2.609	0.411	4.935	2.315	6	1179	ZINC000100772022
30.4	-10.9	-10.3	0.27	2.219	0.952	4.644	3.181	6	1180	ZINC000118915334
30.4	-13.4	-12.5	0.60	2.662	0.491	4.180	1.055	6	1181	ZINC000100772024

30.4	-13.3	-12.5	0.61	2.433	0.544	4.744	2.472	6	1182	ZINC000100772020
30.4	-10.9	-10.3	0.30	2.136	0.798	4.618	3.117	6	1183	ZINC000256079353
30.4	-13.3	-12.3	0.86	2.835	0.446	4.851	0.733	6	1184	ZINC000257394167
30.4	-13.4	-12.1	0.93	2.768	0.410	7.035	3.174	6	1185	ZINC000257394165
30.4	-10.9	-10.4	0.27	1.709	0.580	2.926	0.475	6	1186	ZINC000118913657
30.3	-9.7	-9.3	0.32	2.201	0.870	5.896	1.080	6	1187	ZINC000085637243
30.3	-10.9	-10.6	0.20	2.023	0.233	5.881	4.055	6	1188	ZINC000257463854
30.3	-11.1	-10.5	0.35	2.131	0.550	7.338	3.888	6	1189	ZINC000257415286
30.3	-10.5	-9.1	0.95	2.848	0.847	5.567	2.502	6	1190	ZINC000257616744
30.3	-10.8	-10.3	0.23	2.222	0.948	4.646	3.181	6	1191	ZINC000118913655
30.3	-10.8	-10.3	0.29	1.911	0.547	3.097	0.383	6	1192	ZINC000256079355
30.3	-10.5	-9.1	0.99	2.835	0.862	5.545	2.523	6	1193	ZINC000257616743
30.2	-13.2	-12.5	0.47	2.459	0.640	3.976	1.247	6	1194	ZINC000100772026
30.2	-10.5	-9.1	0.97	2.837	0.856	5.493	2.585	6	1195	ZINC000100774044
30.2	-11.8	-10.0	0.94	2.025	0.343	5.639	2.165	5	1196	ZINC000085996810

30.2	-10.5	-9.0	0.92	2.814	0.942	5.500	2.591	6	1197	ZINC000257616745
30.2	-13.2	-12.4	0.45	2.100	0.051	7.296	2.432	6	1198	ZINC000085664941
30.2	-12.0	-11.4	0.45	1.679	0.436	6.473	3.371	6	1199	ZINC000257616268
30.2	-11.8	-11.5	0.23	2.073	0.449	6.686	3.213	6	1200	ZINC000100777920
30.2	-10.5	-8.8	1.06	2.641	0.855	4.994	1.449	6	1201	ZINC000257616746
30.2	-13.2	-11.8	1.02	3.592	0.389	8.894	2.213	6	1202	ZINC000257394168
30.2	-10.5	-9.3	0.87	2.660	0.868	4.426	1.265	5	1203	ZINC000100774052
30.2	-10.5	-9.0	0.97	2.868	0.818	5.605	2.460	6	1204	ZINC000100774049
30.2	-10.3	-9.8	0.39	2.015	0.393	5.574	2.829	6	1205	ZINC000100827936
30.1	-11.8	-10.8	1.05	2.189	0.000	7.571	0.000	2	1206	ZINC000013383665
30.1	-9.9	-9.7	0.17	2.539	0.407	9.501	3.201	6	1207	ZINC000085552256
30.1	-10.7	-10.3	0.28	2.115	1.396	4.334	3.078	6	1208	ZINC000257414911
30.1	-11.4	-10.4	0.75	2.138	0.429	6.423	3.642	6	1209	ZINC000104876194
30.1	-10.5	-10.2	0.15	2.864	0.996	7.633	3.923	6	1210	ZINC000118937372
30.1	-11.0	-10.1	0.59	2.277	0.144	5.798	2.410	6	1211	ZINC000257476924

30.1	-10.6	-9.8	0.41	2.282	0.389	5.531	2.199	6	1212	ZINC000257460262
30.1	-10.6	-9.6	0.53	2.205	0.316	8.055	2.850	6	1213	ZINC000257460261
30.0	-10.5	-10.2	0.20	2.442	1.006	6.259	3.883	6	1214	ZINC000257484336
30.0	-10.6	-9.4	0.59	2.154	0.336	8.736	3.280	6	1215	ZINC000257460263
30.0	-9.9	-9.5	0.17	2.669	0.358	9.570	2.917	6	1216	ZINC000085552258
30.0	-10.5	-10.2	0.15	2.782	1.088	7.636	3.930	6	1217	ZINC000257484337
30.0	-11.0	-10.1	0.56	2.246	0.142	4.728	0.630	6	1218	ZINC000100825489
30.0	-10.9	-8.9	0.92	2.837	0.369	5.832	1.928	6	1219	ZINC000257618310
30.0	-10.5	-10.0	0.35	1.120	0.268	2.379	0.358	4	1220	ZINC000100825290
30.0	-10.9	-9.7	1.06	2.040	1.142	3.426	1.554	6	1221	ZINC000100825297
30.0	-12.8	-12.3	0.30	1.768	0.299	6.549	3.445	6	1222	ZINC000100776733
30.0	-9.3	-8.3	0.76	2.482	0.042	6.028	0.211	3	1223	ZINC000100828693
30.0	-10.5	-10.1	0.28	2.163	1.434	4.370	3.089	6	1224	ZINC000118936781
30.0	-10.9	-9.0	0.96	2.761	0.372	5.822	2.159	5	1225	ZINC000100825294
29.9	-11.0	-10.1	0.58	2.271	0.169	5.767	2.382	6	1226	ZINC000257476923

29.9	-10.9	-9.2	1.04	2.376	0.839	5.079	2.436	6	1227	ZINC000257618311
29.9	-10.4	-10.0	0.31	2.903	1.685	6.042	3.635	6	1228	ZINC000257414909
29.9	-10.9	-9.6	1.00	2.140	1.029	3.547	1.420	6	1229	ZINC000257618313
29.9	-10.5	-10.0	0.31	2.223	1.408	4.558	2.987	6	1230	ZINC000118936784
29.9	-10.5	-10.0	0.33	2.039	1.478	4.263	3.067	6	1231	ZINC000118936783
29.9	-10.4	-10.1	0.21	2.618	1.079	7.693	3.954	6	1232	ZINC000118937375
29.9	-10.5	-9.9	0.36	2.260	1.297	4.813	2.945	6	1233	ZINC000118936782
29.9	-10.9	-9.1	0.90	2.386	0.819	5.132	2.368	6	1234	ZINC000100825291
29.9	-11.0	-10.1	0.58	2.305	0.180	5.851	2.366	6	1235	ZINC000100825486
29.9	-10.9	-9.3	1.09	1.955	0.884	4.787	2.773	6	1236	ZINC000100825298
29.9	-12.7	-11.5	0.77	1.911	0.412	8.510	3.115	6	1237	ZINC000095619775
29.9	-10.9	-8.8	0.97	2.781	0.341	5.348	2.149	6	1238	ZINC000257618312
29.8	-10.2	-9.7	0.30	2.359	1.246	4.864	2.863	6	1239	ZINC000257414908
29.8	-13.2	-12.7	0.51	2.139	0.775	3.608	1.086	6	1240	ZINC000100781614
29.8	-10.2	-10.0	0.15	2.006	0.740	5.779	4.012	6	1241	ZINC000118937373

29.8	-12.4	-11.6	0.59	2.251	0.599	2.653	0.752	6	1242	ZINC000257401054
29.8	-12.5	-11.7	0.88	2.143	0.829	5.379	3.865	6	1243	ZINC000100781856
29.8	-10.4	-10.1	0.32	2.008	1.481	4.332	3.089	6	1244	ZINC000257414910
29.8	-10.1	-9.4	0.43	2.135	0.527	5.099	1.526	6	1245	ZINC000257458621
29.8	-10.3	-10.0	0.24	2.165	0.581	5.840	3.970	6	1246	ZINC000257484339
29.8	-10.8	-10.2	0.39	1.979	0.636	3.636	1.001	6	1247	ZINC000257479449
29.8	-10.5	-9.4	0.84	2.244	1.075	3.920	1.514	6	1248	ZINC000253532195
29.8	-12.1	-11.2	0.41	2.460	0.993	7.130	2.249	6	1249	ZINC000257693326
29.7	-9.5	-8.2	0.73	2.510	0.451	7.101	2.336	6	1250	ZINC000085558121
29.7	-10.7	-10.0	0.48	2.014	0.602	4.940	3.062	6	1251	ZINC000100825447
29.7	-10.3	-9.9	0.30	2.130	0.518	5.468	2.861	6	1252	ZINC000100827934
29.7	-11.0	-10.1	0.56	2.245	0.135	4.400	0.775	6	1253	ZINC000100825496
29.7	-11.9	-11.6	0.20	1.977	0.541	5.759	3.052	6	1254	ZINC000100777917
29.7	-10.7	-9.8	0.41	2.288	0.215	6.180	2.305	6	1255	ZINC000257479451
29.6	-10.5	-9.4	1.05	1.915	1.060	3.844	1.795	6	1256	ZINC000253388707

29.6	-12.2	-11.3	0.73	2.083	0.315	6.870	3.288	6	1257	ZINC000100776741
29.6	-10.5	-9.3	0.84	2.375	1.006	4.214	1.547	4	1258	ZINC000253388708
29.6	-10.4	-10.2	0.16	2.816	1.138	7.618	3.945	6	1259	ZINC000257484338
29.6	-10.5	-9.2	0.66	2.165	1.147	3.693	1.108	6	1260	ZINC000100825285
29.6	-6.9	-5.5	0.93	2.529	0.245	5.459	1.412	4	1261	ZINC000095098885
29.6	-10.7	-9.9	0.49	2.460	0.747	8.398	3.132	6	1262	ZINC000257471915
29.6	-10.3	-9.9	0.30	2.542	0.246	7.095	3.062	6	1263	ZINC000257483677
29.5	-10.5	-9.6	0.90	3.012	0.000	4.585	0.000	2	1264	ZINC000100825287
29.5	-11.2	-10.5	0.44	1.342	0.498	2.301	0.622	6	1265	ZINC000086029824
29.5	-9.1	-8.7	0.29	2.317	0.396	3.516	0.164	3	1266	ZINC000095620843
29.5	-10.5	-8.9	0.87	2.652	0.863	4.464	1.021	6	1267	ZINC000253388706
29.5	-10.8	-9.8	0.69	2.309	0.122	6.911	3.064	6	1268	ZINC000100829170
29.5	-10.5	-8.8	1.20	3.147	0.022	5.998	0.094	3	1269	ZINC000100825281
29.5	-12.7	-11.7	0.58	2.451	0.639	6.796	2.746	6	1270	ZINC000257401057
29.4	-10.4	-9.4	0.44	2.133	0.333	6.559	2.652	6	1271	ZINC000257476922

29.4	-11.2	-10.3	0.46	1.480	0.580	2.871	1.220	6	1272	ZINC000257503535
29.4	-10.7	-10.1	0.40	2.009	0.469	4.043	1.290	6	1273	ZINC000257479450
29.4	-11.2	-9.2	0.91	2.469	0.140	10.192	0.334	6	1274	ZINC000100825394
29.4	-9.2	-8.5	0.61	1.950	0.354	4.273	1.689	3	1275	ZINC000095620842
29.4	-10.7	-9.9	0.52	2.359	0.122	7.011	3.049	6	1276	ZINC000256122405
29.4	-11.2	-10.2	0.73	1.729	0.748	4.481	3.363	6	1277	ZINC000100825395
29.4	-10.7	-10.0	0.46	2.108	0.299	4.130	1.138	6	1278	ZINC000100829171
29.4	-10.5	-9.4	0.78	2.385	0.889	3.771	0.999	6	1279	ZINC000028537120
29.4	-10.5	-8.9	0.95	2.709	0.966	4.668	1.480	6	1280	ZINC000028537111
29.4	-10.5	-9.3	0.99	1.935	0.999	3.830	1.787	6	1281	ZINC000253532198
29.4	-10.7	-10.1	0.40	2.066	0.439	4.134	1.251	6	1282	ZINC000256122402
29.4	-11.2	-10.3	0.62	1.677	0.496	3.024	1.056	6	1283	ZINC000257503536
29.4	-11.2	-10.0	0.75	1.972	0.680	5.685	3.940	6	1284	ZINC000100825391
29.4	-10.7	-9.7	0.57	1.854	0.766	6.034	3.970	6	1285	ZINC000256122404
29.4	-11.2	-10.5	0.46	1.509	0.518	2.556	0.666	6	1286	ZINC000100825389

29.3	-12.9	-11.0	1.04	2.650	0.511	8.429	2.864	5	1287	ZINC000100781618
29.3	-10.7	-10.4	0.17	3.185	0.251	4.528	0.462	6	1288	ZINC000095620039
29.3	-11.2	-10.5	0.51	1.514	0.471	2.579	0.681	6	1289	ZINC000257503537
29.3	-10.7	-10.3	0.27	3.001	1.173	7.633	3.916	6	1290	ZINC000253611314
29.3	-11.1	-10.4	0.42	2.352	0.295	4.700	0.677	6	1291	ZINC000100825670
29.3	-9.8	-9.5	0.21	2.229	1.061	7.567	3.956	6	1292	ZINC000254749820
29.3	-12.9	-12.0	0.87	2.474	0.646	4.846	2.770	6	1293	ZINC000257533118
29.3	-11.1	-10.3	0.54	2.070	0.445	6.237	3.586	6	1294	ZINC000256111249
29.3	-12.6	-11.8	0.50	2.365	0.682	4.474	2.200	6	1295	ZINC000100784156
29.3	-12.8	-11.2	0.96	2.344	0.272	7.126	3.440	6	1296	ZINC000257615335
29.3	-10.9	-10.6	0.23	2.198	0.242	4.446	0.903	6	1297	ZINC000257515950
29.3	-9.8	-9.4	0.30	2.966	0.701	9.393	2.641	6	1298	ZINC000014584319
29.3	-9.8	-9.5	0.20	2.635	0.755	9.232	2.831	6	1299	ZINC000014584315
29.3	-10.9	-10.5	0.36	2.219	0.366	3.792	1.118	6	1300	ZINC000100825223
29.3	-10.6	-10.3	0.22	3.291	0.103	4.354	0.456	6	1301	ZINC000261498310

29.3	-9.8	-9.5	0.21	3.384	0.758	10.435	0.307	6	1302	ZINC000001531878
29.3	-10.5	-9.3	0.61	1.766	1.066	3.269	1.203	6	1303	ZINC000253532197
29.3	-11.1	-10.5	0.38	2.227	0.220	4.745	0.686	6	1304	ZINC000256111253
29.2	-10.7	-10.3	0.26	3.577	0.681	9.458	3.008	6	1305	ZINC000118915332
29.2	-10.9	-10.3	0.34	1.975	0.489	3.535	1.559	6	1306	ZINC000257515953
29.2	-9.7	-9.5	0.22	2.160	0.803	7.678	4.040	6	1307	ZINC000254749819
29.2	-10.7	-10.4	0.17	3.444	0.723	8.103	3.412	6	1308	ZINC000118915333
29.2	-11.8	-11.5	0.23	1.931	0.410	6.674	3.190	6	1309	ZINC000100777918
29.2	-10.5	-9.4	1.04	1.835	1.109	3.776	1.843	6	1310	ZINC000028537115
29.2	-10.7	-10.3	0.20	3.608	0.480	9.317	2.881	6	1311	ZINC000040165204
29.2	-10.7	-10.4	0.19	3.006	1.265	7.698	3.903	6	1312	ZINC000118915330
29.2	-10.7	-10.3	0.24	2.941	1.262	7.718	3.893	6	1313	ZINC000253611310
29.2	-10.9	-9.3	1.17	2.160	0.946	5.186	2.642	5	1314	ZINC000017545537
29.2	-11.1	-10.3	0.60	2.189	0.130	5.664	2.450	6	1315	ZINC000100825671
29.2	-9.8	-9.5	0.17	2.885	0.697	10.760	0.250	6	1316	ZINC000253615056

29.2	-11.1	-10.2	0.71	2.407	0.206	8.025	3.108	6	1317	ZINC000256111246
29.2	-10.7	-10.4	0.20	2.689	1.105	6.399	3.664	6	1318	ZINC000253611308
29.2	-10.5	-9.3	0.65	2.211	1.131	3.738	1.133	6	1319	ZINC000028537105
29.2	-10.7	-10.4	0.19	2.590	1.093	4.986	3.028	6	1320	ZINC000253611311
29.2	-9.7	-8.5	0.73	2.284	0.694	8.592	0.615	4	1321	ZINC000257463094
29.2	-9.7	-9.5	0.16	2.751	0.598	10.673	0.226	6	1322	ZINC000014584317
29.2	-10.5	-9.3	1.09	1.931	1.081	3.856	1.844	6	1323	ZINC000253388709
29.2	-12.1	-11.3	0.64	2.862	0.478	7.893	1.737	6	1324	ZINC000086034624
29.1	-11.4	-10.4	0.77	1.982	0.797	6.523	3.342	6	1325	ZINC000100824432
29.1	-10.8	-10.0	0.41	2.131	0.677	3.997	1.439	6	1326	ZINC000238743467
29.1	-12.8	-10.8	1.03	2.862	0.764	7.062	3.263	6	1327	ZINC000257533116
29.0	-10.5	-9.9	0.41	2.446	0.415	4.342	0.196	6	1328	ZINC000100825220
29.0	-11.6	-10.1	1.09	2.995	0.091	5.245	0.220	3	1329	ZINC000070450876
29.0	-9.6	-9.4	0.15	2.200	0.417	6.174	3.875	6	1330	ZINC000014584321
29.0	-10.4	-10.0	0.33	2.056	0.538	5.441	3.878	6	1331	ZINC000095620040

29.0	-10.5	-10.3	0.23	2.579	1.075	6.376	3.825	6	1332	ZINC000085552274
29.0	-10.5	-10.2	0.17	1.591	0.469	2.286	0.555	6	1333	ZINC000118915331
29.0	-11.1	-11.1	0.00	0.000	0.000	0.000	0.000	1	1334	ZINC000257554952
29.0	-10.7	-10.0	0.45	2.398	0.140	5.696	2.610	6	1335	ZINC000100825452
28.9	-11.1	-10.4	0.43	3.292	1.064	6.616	2.771	6	1336	ZINC000118936891
28.9	-11.3	-10.5	0.47	1.518	0.415	2.447	0.557	6	1337	ZINC000100773029
28.9	-11.1	-10.5	0.35	2.896	1.113	6.461	3.147	6	1338	ZINC000008220551
28.9	-10.8	-10.5	0.30	2.588	0.493	8.657	0.226	6	1339	ZINC000257493816
28.9	-10.7	-9.3	1.07	2.745	1.039	5.468	2.628	6	1340	ZINC000253387539
28.9	-11.1	-10.5	0.35	3.183	0.973	6.751	2.890	6	1341	ZINC000257451293
28.9	-11.1	-10.5	0.37	3.207	0.995	6.657	2.806	6	1342	ZINC000118936893
28.9	-10.8	-10.4	0.21	2.322	1.005	5.705	3.909	6	1343	ZINC000085552254
28.9	-11.1	-10.5	0.38	3.328	1.038	6.833	2.817	6	1344	ZINC000118936890
28.9	-10.7	-9.7	0.54	2.280	0.173	7.280	2.743	6	1345	ZINC000100825450
28.9	-11.3	-10.6	0.50	1.386	0.520	2.422	0.707	6	1346	ZINC000257464972

28.9	-11.1	-10.5	0.43	3.903	0.483	8.176	1.758	6	1347	ZINC000257451292
28.9	-11.1	-10.4	0.39	3.449	1.184	6.842	2.938	6	1348	ZINC000118936892
28.9	-11.1	-10.4	0.45	3.141	0.991	5.891	2.590	6	1349	ZINC000257451295
28.9	-11.1	-10.6	0.36	3.178	0.973	6.750	2.888	6	1350	ZINC000257451294
28.9	-10.7	-9.7	0.98	2.264	1.029	4.033	1.476	6	1351	ZINC000100774159
28.9	-10.7	-9.7	0.97	2.271	1.039	4.043	1.529	6	1352	ZINC000165783065
28.9	-12.4	-11.5	0.64	2.152	0.528	4.495	2.782	6	1353	ZINC000257615334
28.8	-10.6	-9.2	1.13	2.675	1.156	5.699	2.839	5	1354	ZINC000100774158
28.8	-11.9	-10.4	0.90	2.349	0.244	7.087	2.867	6	1355	ZINC000100823248
28.8	-10.2	-9.1	0.54	2.017	0.673	5.684	2.764	6	1356	ZINC000085546859
28.8	-11.3	-10.6	0.46	1.227	0.525	2.076	0.718	6	1357	ZINC000257464970
28.8	-10.6	-9.5	1.14	2.407	1.156	5.095	2.808	6	1358	ZINC000100774161
28.8	-10.6	-9.7	0.46	1.887	0.103	5.138	2.582	6	1359	ZINC000100825669
28.8	-11.3	-10.5	0.47	1.430	0.539	2.448	0.722	6	1360	ZINC000100773028
28.8	-11.3	-10.2	0.77	1.758	0.584	3.235	1.131	6	1361	ZINC000100773036

28.8	-11.3	-10.6	0.42	1.138	0.173	2.005	0.214	6	1362	ZINC000100773033
28.8	-10.7	-8.9	1.08	2.212	0.478	7.380	2.929	4	1363	ZINC000085907212
28.7	-10.5	-9.5	0.78	2.401	1.105	3.748	1.322	6	1364	ZINC000253387538
28.7	-11.5	-10.4	0.86	1.750	0.366	5.718	3.485	6	1365	ZINC000257481898
28.7	-10.5	-8.8	0.96	3.247	0.193	6.394	2.087	5	1366	ZINC000100774164
28.7	-13.1	-12.1	0.92	2.880	0.644	5.450	2.535	6	1367	ZINC000257533115
28.7	-11.3	-10.4	0.60	1.642	0.485	3.025	1.187	6	1368	ZINC000257464969
28.7	-10.3	-9.8	0.26	1.481	0.438	3.789	3.097	6	1369	ZINC000017044426
28.7	-10.7	-9.6	0.58	2.388	0.188	7.261	2.787	6	1370	ZINC000100825455
28.7	-11.3	-10.7	0.49	1.559	0.532	3.037	1.179	6	1371	ZINC000257464971
28.7	-9.8	-9.5	0.20	2.115	0.631	4.761	3.208	6	1372	ZINC000100829169
28.6	-12.4	-11.5	0.46	2.061	0.688	6.367	3.315	6	1373	ZINC000100777936
28.6	-12.9	-11.5	1.17	2.495	0.533	7.254	3.117	6	1374	ZINC000100781619
28.6	-12.7	-10.9	0.93	2.942	0.121	8.710	2.239	6	1375	ZINC000257533117
28.6	-12.4	-11.7	0.38	1.859	0.746	5.974	3.746	6	1376	ZINC000257540223

28.6	-10.5	-9.9	0.44	2.266	0.399	7.543	4.104	6	1377	ZINC000100825668
28.6	-11.2	-10.4	0.41	1.520	0.572	3.399	1.393	6	1378	ZINC000100827695
28.5	-10.8	-10.3	0.41	1.584	0.369	3.044	0.940	6	1379	ZINC000100825194
28.5	-10.9	-9.3	1.19	2.162	0.951	5.141	2.685	5	1380	ZINC000253388745
28.5	-10.9	-9.2	1.11	2.334	0.916	5.018	2.388	6	1381	ZINC000017545534
28.5	-11.6	-10.4	1.04	2.199	0.291	7.083	3.411	6	1382	ZINC000257414052
28.5	-10.7	-9.9	0.47	2.080	0.442	6.621	3.452	6	1383	ZINC000256122403
28.5	-9.7	-8.9	0.47	3.036	0.409	6.606	2.686	6	1384	ZINC000085567825
28.5	-9.8	-9.0	0.54	2.279	0.384	5.186	2.842	6	1385	ZINC000230075599
28.5	-9.9	-9.0	0.55	2.188	0.682	7.156	2.015	6	1386	ZINC000085546857
28.5	-10.9	-10.2	0.40	1.831	0.445	4.433	1.183	6	1387	ZINC000257524149
28.5	-12.4	-11.3	0.67	2.372	0.656	9.156	0.268	6	1388	ZINC000100777933
28.5	-10.9	-9.6	1.03	2.112	1.053	3.502	1.431	6	1389	ZINC000017545540
28.5	-10.9	-9.8	1.24	1.808	1.196	3.740	1.698	3	1390	ZINC000017545543
28.5	-10.9	-9.9	0.59	2.276	0.271	8.254	2.894	6	1391	ZINC000257524151

28.5	-8.8	-7.4	0.92	2.909	0.050	6.322	2.622	6	1392	ZINC000100774050
28.4	-10.8	-10.3	0.39	2.065	0.257	4.976	0.303	6	1393	ZINC000100825193
28.4	-11.5	-11.0	0.47	2.244	0.356	5.778	2.752	6	1394	ZINC000095617903
28.4	-10.9	-8.9	1.03	2.766	0.341	5.763	2.088	5	1395	ZINC000253388742
28.4	-10.9	-9.6	1.03	2.106	1.049	3.533	1.377	6	1396	ZINC000253388744
28.4	-12.4	-11.6	0.42	2.236	0.849	6.337	3.376	6	1397	ZINC000100777930
28.4	-12.4	-11.4	0.53	2.549	0.617	5.733	2.785	6	1398	ZINC000257540222
28.4	-10.5	-10.0	0.23	2.220	0.157	5.748	1.827	6	1399	ZINC000150352512
28.4	-10.9	-9.4	1.00	2.162	1.004	3.532	1.390	6	1400	ZINC000253388743
28.4	-12.4	-11.5	0.59	2.427	0.722	8.032	2.176	6	1401	ZINC000257540225
28.4	-10.8	-10.3	0.35	1.889	0.184	4.555	0.783	6	1402	ZINC000100825197
28.3	-9.6	-8.9	0.51	2.552	0.780	5.181	2.756	6	1403	ZINC000257549067
28.3	-9.8	-9.0	0.60	2.143	1.257	3.729	1.534	6	1404	ZINC000095619645
28.3	-10.9	-10.5	0.21	2.814	0.752	5.982	2.588	6	1405	ZINC000100777700
28.3	-12.4	-11.6	0.41	2.268	0.784	6.485	3.152	6	1406	ZINC000257540224

28.3	-10.7	-10.0	0.41	3.078	0.283	7.009	3.149	6	1407	ZINC000261498311
28.3	-11.8	-11.6	0.22	2.079	0.521	5.454	3.108	6	1408	ZINC000100777921
28.3	-10.8	-10.1	0.36	1.955	0.458	4.201	3.225	6	1409	ZINC000100825195
28.3	-10.9	-10.5	0.29	2.348	0.907	5.869	2.425	6	1410	ZINC000248340922
28.2	-9.9	-9.0	0.62	2.072	1.195	3.677	1.554	6	1411	ZINC000095619642
28.2	-10.9	-10.5	0.21	2.531	1.012	7.061	2.430	6	1412	ZINC000248340924
28.2	-10.9	-10.4	0.22	2.268	0.900	5.991	2.313	6	1413	ZINC000100777699
28.2	-11.9	-10.9	0.54	1.806	0.357	3.600	0.925	6	1414	ZINC000118912913
28.2	-11.8	-10.3	0.89	2.105	0.720	5.868	1.022	6	1415	ZINC000150359943
28.2	-10.9	-10.5	0.27	2.342	0.910	5.900	2.427	6	1416	ZINC000248340923
28.2	-10.9	-10.5	0.25	3.527	0.681	6.037	2.187	6	1417	ZINC000248340925
28.2	-10.9	-10.5	0.22	2.519	1.023	7.073	2.435	6	1418	ZINC000100777695
28.2	-12.6	-11.5	0.66	2.560	0.586	6.089	3.011	6	1419	ZINC000100777661
28.1	-12.0	-11.0	0.69	1.621	0.624	3.042	0.995	6	1420	ZINC000255974230
28.1	-12.1	-11.1	0.43	2.377	0.897	7.958	2.019	6	1421	ZINC000257693325

28.1	-10.8	-9.7	0.61	3.049	0.180	10.170	0.333	6	1422	ZINC000100824461
28.1	-10.4	-9.7	0.44	1.736	0.506	4.119	3.350	6	1423	ZINC000257524150
28.1	-11.0	-9.1	1.01	2.218	0.406	7.965	3.032	5	1424	ZINC000086044911
28.1	-6.6	-6.1	0.27	2.726	0.270	10.014	3.004	6	1425	ZINC000257775068
28.1	-11.5	-10.5	0.44	2.658	0.511	7.326	3.464	6	1426	ZINC000257526034
28.1	-10.2	-9.6	0.29	1.847	0.837	3.016	1.130	6	1427	ZINC000100829173
28.1	-12.1	-11.2	0.41	2.460	0.994	7.138	2.234	6	1428	ZINC000257693324
28.1	-12.1	-11.1	0.45	2.756	0.865	6.274	2.050	6	1429	ZINC000257693323
28.1	-9.5	-8.9	0.60	2.130	0.000	5.711	0.000	2	1430	ZINC000257378052
28.0	-11.5	-10.8	0.43	2.321	0.571	5.801	3.485	6	1431	ZINC000040165249
28.0	-9.7	-9.5	0.10	2.677	1.039	6.268	4.019	6	1432	ZINC000085552272
28.0	-11.5	-10.9	0.35	2.320	1.213	4.590	2.846	6	1433	ZINC000257526032
28.0	-11.4	-10.6	0.44	2.267	1.407	5.744	3.719	6	1434	ZINC000040165265
28.0	-11.5	-10.6	0.42	2.923	0.621	7.333	3.323	6	1435	ZINC000118915340
28.0	-11.3	-10.7	0.33	2.180	1.396	5.528	3.724	6	1436	ZINC000040165263

28.0	-10.4	-9.0	1.01	1.952	0.393	6.934	3.180	6	1437	ZINC000072176260
28.0	-11.4	-10.6	0.38	2.316	1.493	5.537	3.715	6	1438	ZINC000261496054
28.0	-11.5	-10.9	0.35	2.555	1.157	5.853	3.447	6	1439	ZINC000118915343
28.0	-11.5	-10.7	0.38	2.418	1.230	5.697	3.512	6	1440	ZINC000118915342
28.0	-11.5	-10.6	0.41	2.698	0.904	7.193	3.501	6	1441	ZINC000118915341
28.0	-11.4	-10.7	0.39	1.889	0.735	3.162	0.704	6	1442	ZINC000257526033
28.0	-11.4	-10.5	0.40	2.651	1.135	5.906	3.578	6	1443	ZINC000040165247
28.0	-9.8	-9.1	0.52	1.610	1.149	3.080	1.093	6	1444	ZINC000095619643
28.0	-11.4	-10.5	0.42	3.237	1.021	8.705	3.073	6	1445	ZINC000040165264
28.0	-10.2	-8.9	1.25	1.350	0.000	8.860	0.000	2	1446	ZINC000013383663
28.0	-11.5	-10.7	0.41	2.357	1.045	5.780	3.448	6	1447	ZINC000257526031
27.9	-11.3	-10.5	0.39	2.473	1.169	5.766	3.637	6	1448	ZINC000118913667
27.9	-11.3	-10.6	0.33	1.706	1.070	4.204	2.934	6	1449	ZINC000261496055
27.9	-9.8	-9.1	0.52	1.613	1.147	3.059	1.099	6	1450	ZINC000095619644
27.9	-7.3	-6.8	0.29	1.815	0.728	4.676	3.379	6	1451	ZINC000100825314

27.9	-11.3	-10.6	0.39	2.770	1.440	7.231	3.641	6	1452	ZINC000118913666
27.9	-10.4	-10.0	0.24	2.069	0.162	4.271	0.634	6	1453	ZINC000100825849
27.9	-11.3	-10.6	0.32	2.378	1.436	5.700	3.611	6	1454	ZINC000008234384
27.9	-9.8	-9.6	0.17	2.215	0.578	5.115	3.080	6	1455	ZINC000257479448
27.9	-10.4	-9.9	0.36	2.083	0.173	5.496	2.266	6	1456	ZINC000257553956
27.9	-11.4	-10.5	0.41	2.814	0.885	6.191	3.461	6	1457	ZINC000040165266
27.8	-12.0	-11.0	0.67	1.812	0.502	3.190	0.961	6	1458	ZINC000255974231
27.8	-11.3	-10.6	0.38	2.268	1.171	5.689	3.689	6	1459	ZINC000008234385
27.8	-9.9	-9.8	0.11	2.758	0.114	4.277	0.221	6	1460	ZINC000085552253
27.8	-12.0	-10.6	0.64	1.786	0.488	2.380	0.591	6	1461	ZINC000255974229
27.8	-11.3	-10.6	0.41	1.982	1.061	5.719	3.676	6	1462	ZINC000261496053
27.8	-12.0	-10.7	0.93	1.950	0.747	4.625	3.017	6	1463	ZINC000100825384
27.8	-12.0	-10.7	0.91	1.827	0.679	5.232	3.886	6	1464	ZINC000100825381
27.8	-11.6	-11.1	0.34	2.028	0.151	6.735	2.711	6	1465	ZINC000257671262
27.8	-9.7	-8.1	1.01	2.479	0.568	5.616	2.914	5	1466	ZINC000257549069

27.8	-12.0	-10.8	0.88	1.840	0.480	3.645	1.032	6	1467	ZINC000100825378
27.8	-7.9	-6.8	1.00	2.210	0.880	6.917	3.519	5	1468	ZINC000004096918
27.8	-12.0	-11.1	0.55	1.599	0.548	2.590	0.776	6	1469	ZINC000255974228
27.8	-12.0	-10.8	0.87	2.035	0.603	3.654	1.153	6	1470	ZINC000100825386
27.7	-10.5	-10.1	0.28	1.835	0.561	3.959	2.876	6	1471	ZINC000100773809
27.7	-10.5	-10.1	0.26	1.501	0.361	2.344	0.461	6	1472	ZINC000100773813
27.7	-11.3	-10.5	0.36	3.367	1.107	8.685	3.081	6	1473	ZINC000118913668
27.7	-8.0	-7.7	0.24	2.466	0.480	5.397	3.385	6	1474	ZINC000100822192
27.7	-11.4	-10.3	0.63	2.342	0.142	7.117	2.504	6	1475	ZINC000100825476
27.6	-9.8	-9.1	0.53	2.191	0.501	6.898	2.510	6	1476	ZINC000085558132
27.6	-10.4	-9.9	0.39	1.935	0.685	5.083	2.693	6	1477	ZINC000059765659
27.6	-9.7	-9.0	0.49	3.006	0.222	10.258	0.279	6	1478	ZINC000257549068
27.6	-5.9	-5.7	0.18	3.156	0.500	7.471	2.993	6	1479	ZINC000100822182
27.6	-10.0	-9.7	0.18	2.083	0.787	7.605	4.224	6	1480	ZINC000118937019
27.6	-12.5	-10.9	1.10	1.870	0.368	5.813	2.518	5	1481	ZINC000086053091

27.5	-10.4	-9.5	1.02	2.048	1.249	4.173	2.074	3	1482	ZINC000059730881
27.5	-7.7	-7.3	0.21	1.985	0.236	3.711	0.373	6	1483	ZINC000100822196
27.5	-5.8	-5.6	0.24	2.296	0.580	5.525	3.129	6	1484	ZINC000100822178
27.5	-9.9	-8.8	1.15	2.040	0.000	3.016	0.000	2	1485	ZINC000085907214
27.5	-7.7	-7.5	0.13	1.954	0.453	5.012	3.371	6	1486	ZINC000100822202
27.5	-7.7	-7.5	0.09	1.871	0.516	4.956	3.396	6	1487	ZINC000100822200
27.5	-9.0	-7.2	1.09	2.595	0.105	7.089	1.165	4	1488	ZINC000038146181
27.5	-6.0	-5.7	0.24	1.953	0.869	5.106	3.599	6	1489	ZINC000100822177
27.5	-10.0	-9.7	0.20	2.246	0.938	7.516	4.163	6	1490	ZINC000257390407
27.5	-7.7	-7.3	0.34	2.172	0.206	5.209	3.427	6	1491	ZINC000100822191
27.5	-7.7	-7.4	0.20	1.861	0.516	3.278	0.832	6	1492	ZINC000257379999
27.5	-7.7	-7.4	0.24	2.033	0.608	4.992	3.369	6	1493	ZINC000257379998
27.5	-11.1	-10.3	0.39	1.737	0.745	3.190	0.736	6	1494	ZINC000004099004
27.5	-10.1	-8.6	1.10	2.122	0.092	6.513	1.185	3	1495	ZINC000100773130
27.5	-7.7	-7.3	0.22	2.379	0.344	8.663	3.927	6	1496	ZINC000100822195

27.5	-14.5	-12.5	0.98	2.469	0.761	5.062	2.897	6	1497	ZINC000150353463
27.5	-11.3	-10.6	0.56	1.771	0.332	4.443	1.855	6	1498	ZINC000257671271
27.5	-7.7	-7.4	0.21	2.084	0.196	4.952	3.520	6	1499	ZINC000257506060
27.5	-7.9	-7.7	0.16	2.097	0.272	4.950	3.556	6	1500	ZINC000257506057
27.5	-10.4	-9.9	0.39	2.173	0.314	5.703	2.101	6	1501	ZINC000257553957
27.5	-5.9	-5.6	0.26	2.093	0.616	5.254	3.477	6	1502	ZINC000257775070
27.4	-11.6	-10.2	0.94	2.027	0.097	3.644	0.335	5	1503	ZINC000085886068
27.4	-10.6	-10.1	0.26	1.852	0.552	4.019	2.889	6	1504	ZINC000100773823
27.4	-10.2	-9.9	0.21	2.064	0.173	5.630	2.702	6	1505	ZINC000100825847
27.4	-11.3	-10.4	0.66	1.791	0.334	3.677	1.045	6	1506	ZINC000257671258
27.4	-11.1	-10.1	0.61	2.117	0.443	6.413	3.727	6	1507	ZINC000256111251
27.4	-7.6	-7.4	0.15	1.999	0.478	4.976	3.432	6	1508	ZINC000100822199
27.4	-7.7	-7.2	0.34	2.101	0.373	3.485	0.552	6	1509	ZINC000257506058
27.4	-7.5	-5.8	1.03	2.098	0.740	6.861	2.596	5	1510	ZINC000100773124
27.4	-10.6	-10.1	0.29	1.871	0.496	4.182	2.840	6	1511	ZINC000100773822

27.4	-7.7	-7.2	0.34	1.958	0.510	4.727	3.603	6	1512	ZINC000100822194
27.3	-9.9	-8.8	0.51	3.262	0.358	6.530	1.608	6	1513	ZINC000095617640
27.3	-11.2	-10.5	0.58	1.860	0.325	4.951	2.319	6	1514	ZINC000257671266
27.3	-11.0	-10.2	0.45	2.367	0.292	6.952	2.700	6	1515	ZINC000257612632
27.3	-10.8	-9.3	0.80	1.769	0.347	8.522	3.171	6	1516	ZINC000257496518
27.3	-7.5	-7.2	0.26	2.069	0.304	5.441	3.110	6	1517	ZINC000257379997
27.3	-7.6	-7.3	0.19	2.262	0.297	5.231	3.316	6	1518	ZINC000257506059
27.3	-10.1	-8.8	0.81	2.504	0.295	4.791	0.807	6	1519	ZINC000150351526
27.3	-12.1	-11.5	0.48	1.986	0.466	4.590	2.773	6	1520	ZINC000257546885
27.3	-10.6	-10.2	0.30	2.478	0.916	5.908	3.907	6	1521	ZINC000040165221
27.3	-10.7	-9.8	0.57	2.823	0.406	6.225	2.348	6	1522	ZINC000100829260
27.2	-12.6	-11.8	0.50	2.636	0.419	7.552	2.982	6	1523	ZINC000100825987
27.2	-10.5	-10.2	0.26	2.859	0.893	8.967	3.223	6	1524	ZINC000040165220
27.2	-11.3	-10.6	0.46	2.105	0.481	6.188	3.290	6	1525	ZINC000257778808
27.2	-10.6	-10.2	0.26	1.945	0.992	4.154	3.178	6	1526	ZINC000040165219

27.2	-5.7	-5.2	0.38	1.774	0.677	3.196	1.122	6	1527	ZINC000100822180
27.1	-11.2	-10.7	0.31	1.458	0.509	5.776	3.466	6	1528	ZINC000138372595
27.1	-10.5	-10.2	0.26	1.998	1.103	5.834	3.784	6	1529	ZINC000008234405
27.1	-12.6	-11.9	0.57	2.708	0.467	6.349	2.967	6	1530	ZINC000100825990
27.1	-10.9	-10.6	0.22	2.843	0.782	6.022	2.577	6	1531	ZINC000100777697
27.1	-10.5	-10.1	0.27	2.743	0.851	7.656	3.754	6	1532	ZINC000102136971
27.1	-12.4	-12.0	0.25	2.817	0.454	6.427	2.923	6	1533	ZINC000257472637
27.1	-10.5	-10.1	0.31	2.565	0.930	7.374	4.088	6	1534	ZINC000102136963
27.0	-11.9	-11.0	0.93	1.624	0.383	5.154	3.921	6	1535	ZINC000100826207
27.0	-10.7	-10.2	0.26	1.727	0.497	4.015	0.964	6	1536	ZINC000253529537
27.0	-11.6	-10.4	0.87	1.619	0.614	3.180	1.145	6	1537	ZINC000100773022
27.0	-10.6	-10.0	0.33	2.109	0.273	6.638	2.730	6	1538	ZINC000100825605
27.0	-12.1	-11.8	0.41	2.681	0.665	7.262	3.562	6	1539	ZINC000100825989
27.0	-10.6	-10.1	0.31	2.001	0.568	5.760	2.092	6	1540	ZINC000100825610
26.9	-11.6	-9.8	0.96	2.069	0.536	5.412	2.675	6	1541	ZINC000100773024

26.9	-10.6	-10.1	0.37	2.015	0.579	5.949	3.332	6	1542	ZINC000253529538
26.9	-12.1	-11.8	0.24	2.422	0.663	5.901	3.149	6	1543	ZINC000100825991
26.9	-11.6	-10.6	0.71	1.510	0.444	2.956	1.116	6	1544	ZINC000100773019
26.9	-10.6	-10.3	0.26	1.927	0.547	4.998	2.582	6	1545	ZINC000100825607
26.9	-10.8	-10.1	0.38	2.273	0.125	5.343	0.195	6	1546	ZINC000150361570
26.9	-11.6	-10.5	0.84	1.434	0.397	2.862	1.149	6	1547	ZINC000257527293
26.9	-11.9	-10.7	0.59	2.128	0.218	8.932	2.172	6	1548	ZINC000085567725
26.9	-10.4	-10.2	0.11	2.039	0.458	3.374	0.306	6	1549	ZINC000261496056
26.9	-12.1	-11.7	0.37	2.373	0.497	6.069	3.039	6	1550	ZINC000257472635
26.9	-11.6	-10.4	0.87	1.795	0.621	4.124	3.104	6	1551	ZINC000257527294
26.9	-12.4	-12.1	0.20	3.012	0.214	7.608	2.345	6	1552	ZINC000100779189
26.9	-11.6	-10.4	0.92	1.887	0.691	4.583	3.060	6	1553	ZINC000086029853
26.9	-12.4	-10.7	1.11	2.024	0.343	7.716	3.371	4	1554	ZINC000257475639
26.9	-11.6	-10.6	0.75	1.669	0.619	2.913	0.767	6	1555	ZINC000257527295
26.8	-10.7	-10.2	0.31	2.401	0.690	6.679	3.123	6	1556	ZINC000100780754

26.8	-11.0	-10.7	0.23	1.841	0.321	8.280	2.602	6	1557	ZINC000085863878
26.8	-11.2	-10.4	0.44	2.101	0.565	6.477	2.991	6	1558	ZINC000257567964
26.8	-10.9	-10.1	0.48	1.995	0.176	4.222	1.136	6	1559	ZINC000257427756
26.7	-12.2	-11.9	0.19	2.105	0.912	7.331	2.346	6	1560	ZINC000100779187
26.7	-10.5	-9.9	0.41	2.332	0.477	5.693	2.068	6	1561	ZINC000100825225
26.7	-10.4	-10.0	0.17	1.645	0.258	6.575	3.801	6	1562	ZINC000230075702
26.7	-10.5	-9.9	0.39	2.287	0.656	3.955	1.000	6	1563	ZINC000257515951
26.7	-9.7	-8.9	0.68	2.666	0.395	6.732	2.701	6	1564	ZINC000095617639
26.7	-10.1	-9.7	0.20	2.077	0.795	7.582	4.252	6	1565	ZINC000118937018
26.7	-12.3	-11.2	0.71	2.009	0.437	5.842	3.114	6	1566	ZINC000085664948
26.7	-9.2	-8.2	0.56	1.975	0.778	7.094	1.082	6	1567	ZINC000085637247
26.7	-10.7	-9.2	1.04	2.159	0.978	6.061	4.337	6	1568	ZINC000100825347
26.7	-10.7	-9.4	0.98	1.927	0.803	5.222	3.213	6	1569	ZINC000100825345
26.7	-10.4	-9.2	0.64	1.943	0.504	7.389	3.577	6	1570	ZINC000257476925
26.7	-12.1	-11.0	0.72	2.655	0.212	4.483	0.481	6	1571	ZINC000257472638

26.7	-11.9	-11.5	0.36	1.900	0.285	5.619	3.301	6	1572	ZINC000257468788
26.7	-10.6	-10.2	0.25	1.716	0.261	4.417	3.447	6	1573	ZINC000257697353
26.6	-11.4	-10.7	0.51	1.287	0.514	2.361	0.789	6	1574	ZINC000100825397
26.6	-10.5	-9.8	0.68	1.510	0.022	2.317	0.288	3	1575	ZINC000100777077
26.6	-12.1	-11.4	0.59	2.529	0.229	8.772	1.946	6	1576	ZINC000257472636
26.6	-9.4	-8.9	0.33	2.015	0.736	6.116	2.654	6	1577	ZINC000085558136
26.6	-10.8	-10.3	0.32	1.514	0.285	7.142	4.077	6	1578	ZINC000257697351
26.6	-11.1	-10.3	0.43	1.827	0.988	4.411	3.106	6	1579	ZINC000040165230
26.6	-7.9	-6.2	0.96	1.940	0.260	5.871	1.878	6	1580	ZINC000100828906
26.5	-10.0	-8.5	1.50	1.343	0.000	9.228	0.000	2	1581	ZINC000257378051
26.5	-10.7	-10.5	0.21	1.704	0.184	6.723	2.874	6	1582	ZINC000014495570
26.5	-11.1	-10.5	0.32	2.167	1.330	5.611	3.817	6	1583	ZINC000040165229
26.5	-12.5	-11.9	0.42	3.118	0.698	7.520	2.845	6	1584	ZINC000257712439
26.5	-12.6	-11.8	0.45	2.614	0.570	7.900	2.812	6	1585	ZINC000257712437
26.5	-10.3	-9.6	0.38	2.516	0.593	4.861	2.709	6	1586	ZINC000100825222

26.5	-11.1	-10.3	0.37	3.018	0.988	8.683	3.098	6	1587	ZINC000040165231
26.5	-12.6	-12.2	0.19	1.784	0.472	4.666	2.836	6	1588	ZINC000257410668
26.5	-11.1	-10.3	0.37	2.759	0.836	8.736	3.094	6	1589	ZINC000040165258
26.5	-11.1	-10.4	0.35	2.074	1.210	5.689	3.903	6	1590	ZINC000040165228
26.4	-9.5	-9.1	0.25	1.599	0.213	2.803	0.567	6	1591	ZINC000257379609
26.4	-10.8	-10.3	0.29	1.669	0.341	5.688	3.837	6	1592	ZINC000100828898
26.4	-12.6	-11.5	0.72	2.274	0.390	7.601	2.295	6	1593	ZINC000085741836
26.4	-8.8	-7.5	0.67	2.317	0.223	6.415	0.278	6	1594	ZINC000257628367
26.4	-10.8	-10.4	0.29	2.104	0.410	4.832	2.372	6	1595	ZINC000257399034
26.3	-11.6	-9.7	0.88	1.933	0.579	5.322	1.132	6	1596	ZINC000085886539
26.3	-11.2	-10.8	0.32	3.335	0.832	5.801	2.020	6	1597	ZINC000257382341
26.3	-13.7	-12.6	0.94	1.633	0.625	2.837	0.645	4	1598	ZINC000257534788
26.3	-8.7	-7.3	0.95	2.056	0.150	5.946	1.910	5	1599	ZINC000100828904
26.3	-10.2	-9.7	0.32	2.068	0.537	5.936	4.015	6	1600	ZINC000100825471
26.3	-11.3	-10.6	0.55	2.352	0.549	5.232	2.172	6	1601	ZINC000257526822

26.3	-10.8	-10.1	0.34	1.790	1.132	4.341	3.154	6	1602	ZINC000004096817
26.3	-10.7	-10.2	0.34	2.164	0.670	7.032	2.976	6	1603	ZINC000028541894
26.3	-6.5	-4.7	1.06	2.570	0.602	7.728	1.079	5	1604	ZINC000013383661
26.3	-10.8	-10.2	0.37	2.337	0.506	6.644	3.194	6	1605	ZINC000257399031
26.3	-10.8	-10.4	0.29	1.984	0.458	5.711	2.891	6	1606	ZINC000028541902
26.2	-8.8	-8.0	1.20	2.872	0.922	7.055	3.391	4	1607	ZINC000085907198
26.2	-10.8	-10.0	0.36	2.855	0.951	7.393	3.599	6	1608	ZINC000150374443
26.2	-10.8	-10.1	0.36	2.720	1.213	7.225	3.777	6	1609	ZINC000008551674
26.2	-10.9	-10.1	0.39	2.792	0.889	7.314	3.438	6	1610	ZINC000256310200
26.2	-10.8	-10.0	0.35	2.925	0.930	8.769	3.061	6	1611	ZINC000013515304
26.2	-7.2	-5.8	1.00	2.513	0.103	6.923	0.504	5	1612	ZINC000257397847
26.2	-11.2	-10.8	0.35	3.342	0.831	5.023	1.611	6	1613	ZINC000100777724
26.2	-11.2	-10.8	0.30	3.327	0.832	5.807	2.023	6	1614	ZINC000257382343
26.2	-11.2	-10.7	0.37	3.841	0.531	6.170	1.745	6	1615	ZINC000100777720
26.2	-10.7	-10.3	0.29	1.976	0.464	5.697	2.882	6	1616	ZINC000257399032

26.2	-10.7	-9.8	0.54	1.670	0.652	3.641	1.454	6	1617	ZINC000100825407
26.2	-10.5	-9.9	0.38	2.432	0.174	7.375	2.346	6	1618	ZINC000100777080
26.2	-10.8	-10.1	0.35	2.913	1.025	7.381	3.578	6	1619	ZINC000013515302
26.2	-10.8	-10.2	0.31	2.048	1.214	4.401	2.974	6	1620	ZINC000012496781
26.2	-11.2	-10.7	0.36	3.679	0.427	5.957	1.875	6	1621	ZINC000257382342
26.2	-9.0	-8.8	0.24	2.253	0.328	7.680	3.806	6	1622	ZINC000015152843
26.2	-11.2	-10.6	0.35	3.331	0.832	5.759	2.053	6	1623	ZINC000100777725
26.1	-10.4	-10.2	0.12	2.688	1.161	7.641	3.912	6	1624	ZINC000118937374
26.1	-10.1	-9.3	0.52	2.143	0.538	7.139	3.344	6	1625	ZINC000095619924
26.1	-10.1	-9.0	0.54	2.615	0.753	4.044	0.989	6	1626	ZINC000095617896
26.1	-10.9	-9.9	0.62	2.050	0.361	4.760	2.519	6	1627	ZINC000257427757
26.1	-10.7	-9.0	1.02	2.210	0.593	8.629	3.322	6	1628	ZINC000257379607
26.1	-10.9	-10.0	0.55	2.259	0.185	5.983	2.285	6	1629	ZINC000150361574
26.1	-11.4	-10.5	0.45	2.314	0.420	4.718	2.251	6	1630	ZINC000095619717
26.1	-10.1	-9.7	0.21	1.509	0.513	5.633	3.143	6	1631	ZINC000100825214

26.1	-11.9	-10.7	0.66	2.168	0.734	3.359	0.649	6	1632	ZINC000118937175
26.1	-10.5	-9.0	0.99	2.088	0.334	5.303	2.134	5	1633	ZINC000150368767
26.1	-10.1	-8.7	0.90	2.142	0.720	6.220	2.649	6	1634	ZINC000095617895
26.1	-11.9	-10.6	0.66	2.669	0.745	3.954	0.781	6	1635	ZINC000118912915
26.1	-10.8	-10.0	0.36	2.608	1.146	7.321	3.781	6	1636	ZINC000013515303
26.1	-11.2	-10.7	0.34	3.500	0.947	5.242	1.592	6	1637	ZINC000086027213
26.1	-11.9	-10.7	0.54	1.815	0.349	3.212	0.268	6	1638	ZINC000118937176
26.1	-11.7	-10.4	0.91	1.753	0.509	3.547	1.035	6	1639	ZINC000255984829
26.0	-10.0	-8.5	0.98	1.767	0.459	6.519	3.242	6	1640	ZINC000095617894
26.0	-10.9	-10.2	0.34	1.751	0.266	3.582	1.007	6	1641	ZINC000257427759
26.0	-10.2	-9.6	0.31	1.844	0.487	5.811	2.936	6	1642	ZINC000100825241
26.0	-10.2	-9.6	0.31	1.965	0.348	7.157	2.618	6	1643	ZINC000100825245
26.0	-10.9	-9.6	0.75	1.997	0.778	6.772	2.780	6	1644	ZINC000059353055
26.0	-10.9	-9.8	0.74	1.796	0.446	4.532	2.633	6	1645	ZINC000100828753
26.0	-10.2	-9.7	0.36	1.936	0.749	3.728	1.290	6	1646	ZINC000257429748

26.0	-10.9	-9.5	0.85	2.398	0.494	7.552	3.379	6	1647	ZINC000257427758
26.0	-10.2	-10.0	0.14	2.208	0.375	7.570	3.339	6	1648	ZINC000034944433
26.0	-10.2	-9.6	0.30	1.971	0.344	7.140	2.604	6	1649	ZINC000257429745
26.0	-11.0	-10.5	0.34	3.373	0.448	6.797	2.579	6	1650	ZINC000100777722
25.9	-10.7	-10.0	0.38	1.665	0.489	3.183	1.295	6	1651	ZINC000100825412
25.9	-10.7	-9.8	0.47	1.886	0.368	3.774	1.296	6	1652	ZINC000100825414
25.9	-10.9	-10.2	0.34	1.992	0.271	3.905	0.909	6	1653	ZINC000100828749
25.9	-11.7	-10.6	0.87	2.438	0.575	6.478	2.869	6	1654	ZINC000033963962
25.9	-10.9	-10.3	0.31	1.875	0.384	3.737	1.079	6	1655	ZINC000100828751
25.9	-11.1	-9.9	0.65	1.900	0.624	5.932	1.983	6	1656	ZINC000257535736
25.9	-10.7	-8.8	0.88	2.407	0.407	8.879	3.053	6	1657	ZINC000100825409
25.9	-10.0	-8.7	0.74	2.632	0.428	5.046	2.147	6	1658	ZINC000095617897
25.9	-10.6	-10.1	0.35	2.115	0.691	6.267	3.180	6	1659	ZINC000257558556
25.9	-11.8	-10.9	0.67	2.342	0.682	7.496	2.890	6	1660	ZINC000085648932
25.9	-10.7	-9.8	0.61	1.361	0.335	2.417	0.360	6	1661	ZINC000257379608

25.8	-11.5	-11.1	0.31	2.842	0.938	4.157	2.366	6	1662	ZINC000257394486
25.8	-10.4	-9.7	0.37	2.745	0.851	7.095	3.376	6	1663	ZINC000257404500
25.8	-10.4	-9.4	0.51	2.457	0.355	8.513	2.301	6	1664	ZINC000100827282
25.8	-10.4	-9.9	0.41	2.004	0.887	5.761	3.575	6	1665	ZINC000257404501
25.8	-11.4	-9.9	1.13	2.249	0.811	4.428	2.571	6	1666	ZINC000255984827
25.8	-11.4	-10.4	0.71	1.638	0.570	3.166	1.142	6	1667	ZINC000100825400
25.8	-11.4	-10.4	0.78	1.653	0.603	3.221	1.165	6	1668	ZINC000255984828
25.8	-11.5	-11.0	0.34	2.221	0.484	6.020	2.535	6	1669	ZINC000150351563
25.8	-11.5	-11.1	0.34	3.064	0.470	4.683	1.857	6	1670	ZINC000100777332
25.8	-9.9	-9.0	0.72	2.541	0.555	7.783	3.391	6	1671	ZINC000257426832
25.8	-11.4	-9.4	0.96	2.703	0.499	7.892	2.377	6	1672	ZINC000255984826
25.8	-12.4	-10.6	0.87	2.145	0.195	5.356	2.405	6	1673	ZINC000085567723
25.8	-10.4	-9.8	0.39	2.142	0.131	5.518	2.281	6	1674	ZINC000100825850
25.8	-11.3	-10.6	0.41	1.517	0.348	5.075	2.023	6	1675	ZINC000257548675
25.8	-10.4	-9.8	0.50	2.285	0.553	7.184	3.214	6	1676	ZINC000257404498

25.8	-11.5	-11.1	0.23	3.216	0.357	5.808	2.508	6	1677	ZINC000257394485
25.8	-10.4	-9.6	0.41	2.211	0.345	7.393	2.645	6	1678	ZINC000100827284
25.8	-11.1	-10.3	0.37	2.443	0.201	6.309	2.658	6	1679	ZINC000257414163
25.8	-10.7	-9.8	0.47	1.792	0.512	3.734	1.480	6	1680	ZINC000257379610
25.8	-10.9	-9.7	0.76	1.816	0.113	3.277	0.268	6	1681	ZINC000100828747
25.8	-10.4	-9.6	0.48	2.242	0.933	6.885	3.719	6	1682	ZINC000257404499
25.8	-9.9	-9.7	0.12	1.565	0.335	4.380	2.879	6	1683	ZINC000014709107
25.8	-11.4	-10.4	0.76	1.773	0.638	3.590	1.382	6	1684	ZINC000100825405
25.8	-11.5	-11.2	0.28	3.014	0.484	5.677	2.588	6	1685	ZINC000085939531
25.8	-10.8	-10.4	0.22	3.010	0.948	6.665	2.190	6	1686	ZINC000252516950
25.8	-11.4	-10.4	0.81	1.803	0.536	3.245	1.121	6	1687	ZINC000100825402
25.8	-11.5	-11.1	0.27	2.859	0.952	4.163	2.368	6	1688	ZINC000257394487
25.7	-10.4	-9.6	0.50	2.265	0.294	5.937	2.435	6	1689	ZINC000100825851
25.7	-11.6	-10.5	0.54	2.273	0.532	4.745	1.957	6	1690	ZINC000015252012
25.7	-11.4	-10.5	0.81	2.653	0.303	7.156	1.782	6	1691	ZINC000100783660

25.7	-12.4	-11.5	0.47	2.845	0.747	6.223	3.122	6	1692	ZINC000257520704
25.7	-12.0	-11.3	0.45	2.135	0.412	5.876	2.359	6	1693	ZINC000100784153
25.7	-12.2	-10.9	0.73	2.445	0.974	4.627	2.680	6	1694	ZINC000119383728
25.7	-11.3	-10.0	0.79	2.213	0.370	7.298	3.442	6	1695	ZINC000086044920
25.7	-10.2	-9.6	0.29	1.975	0.347	7.142	2.603	6	1696	ZINC000100825244
25.7	-12.2	-10.9	0.66	3.915	0.188	8.824	2.196	6	1697	ZINC000119383945
25.6	-10.7	-10.0	0.59	1.650	0.252	2.435	0.672	6	1698	ZINC000040164454
25.6	-12.2	-10.9	0.71	2.292	0.971	4.981	2.768	6	1699	ZINC000119383846
25.6	-11.3	-10.7	0.45	2.008	0.448	6.402	3.340	6	1700	ZINC000257548677
25.6	-11.1	-10.2	0.58	1.623	0.385	4.308	3.094	6	1701	ZINC000100775271
25.6	-11.1	-10.7	0.42	2.289	0.498	6.618	3.330	6	1702	ZINC000257415872
25.5	-10.5	-10.1	0.25	2.152	0.437	5.682	2.907	6	1703	ZINC000257534178
25.5	-9.9	-9.7	0.12	1.886	0.944	3.028	1.054	6	1704	ZINC000257399033
25.5	-11.0	-10.2	0.54	2.245	0.140	4.399	0.799	6	1705	ZINC000100825492
25.5	-10.7	-9.0	1.12	2.536	0.911	7.810	4.170	6	1706	ZINC000257454263

25.5	-12.0	-11.4	0.45	3.057	0.645	7.509	2.808	6	1707	ZINC000257712438
25.5	-10.5	-10.2	0.21	2.227	0.435	5.739	2.834	6	1708	ZINC000257534176
25.4	-11.1	-10.6	0.44	2.878	0.350	7.576	1.810	6	1709	ZINC000004098619
25.4	-10.5	-9.6	0.56	1.832	0.771	5.878	2.498	6	1710	ZINC000085546865
25.4	-11.3	-10.3	0.65	1.855	0.814	3.887	2.780	6	1711	ZINC000257772035
25.4	-10.8	-10.3	0.34	1.742	0.445	4.026	3.021	6	1712	ZINC000014765653
25.3	-11.2	-10.5	0.48	1.750	0.188	4.433	1.249	6	1713	ZINC000100827702
25.3	-9.5	-9.1	0.26	2.586	0.844	7.650	4.049	6	1714	ZINC000004261857
25.3	-10.4	-10.2	0.18	1.966	0.307	7.130	2.519	6	1715	ZINC000257455943
25.3	-10.3	-9.4	0.59	2.307	0.548	7.180	1.980	6	1716	ZINC000085546850
25.3	-11.2	-10.4	0.43	1.665	0.311	3.815	1.350	6	1717	ZINC000257461308
25.3	-11.2	-10.5	0.39	1.827	0.381	4.629	1.146	6	1718	ZINC000257461307
25.3	-7.5	-5.6	1.12	2.290	0.659	5.563	2.860	4	1719	ZINC000150357730
25.3	-11.2	-10.6	0.38	1.932	0.363	3.870	1.077	6	1720	ZINC000100827699
25.3	-11.2	-10.5	0.35	1.589	0.524	3.108	1.394	6	1721	ZINC000257461305

25.3	-10.4	-10.0	0.22	2.369	0.619	8.255	1.569	6	1722	ZINC000100827940
25.3	-11.2	-10.4	0.47	1.835	0.264	4.223	1.132	6	1723	ZINC000257461306
25.3	-11.8	-11.1	0.55	2.249	0.280	4.075	0.360	6	1724	ZINC000257712436
25.3	-11.2	-10.5	0.36	1.596	0.521	3.344	1.441	6	1725	ZINC000100827697
25.2	-9.8	-9.3	0.24	1.901	0.504	7.431	3.648	6	1726	ZINC000002097391
25.2	-10.7	-10.3	0.29	1.972	0.461	5.671	2.927	6	1727	ZINC000028541898
25.2	-9.8	-9.4	0.21	1.691	0.573	5.855	3.698	6	1728	ZINC000002097389
25.2	-9.8	-9.3	0.25	1.813	0.390	8.784	3.217	6	1729	ZINC000015058149
25.2	-9.8	-9.3	0.22	1.498	0.598	4.229	3.002	6	1730	ZINC000012654549
25.2	-11.7	-10.4	0.87	2.420	0.208	7.806	2.107	6	1731	ZINC000257548676
25.2	-10.6	-10.2	0.34	1.887	0.625	4.676	2.869	6	1732	ZINC000100827313
25.2	-9.8	-9.3	0.27	1.675	0.532	5.788	3.738	6	1733	ZINC000253616140
25.2	-9.8	-9.4	0.20	1.718	0.584	5.811	3.748	6	1734	ZINC000253616141
25.2	-10.6	-10.3	0.33	2.055	0.733	5.809	3.508	6	1735	ZINC000257558554
25.1	-9.1	-7.8	0.78	2.282	0.550	3.774	1.300	5	1736	ZINC000085907207

25.1	-11.9	-11.0	0.75	2.110	0.603	6.112	3.593	6	1737	ZINC000100824434
25.1	-10.6	-10.0	0.41	2.480	0.376	7.913	2.643	6	1738	ZINC000100827318
25.1	-10.6	-10.0	0.47	2.368	0.888	6.035	3.412	6	1739	ZINC000100827312
25.1	-10.7	-10.4	0.27	3.034	1.368	5.747	3.635	6	1740	ZINC000040165257
25.1	-10.6	-10.2	0.34	2.102	0.677	6.315	3.240	6	1741	ZINC000257558555
25.1	-9.6	-8.7	0.48	2.676	0.997	8.421	3.274	6	1742	ZINC000257768592
25.1	-10.6	-10.1	0.42	2.235	0.831	6.299	3.221	6	1743	ZINC000100827315
25.1	-10.6	-10.3	0.17	2.061	0.807	4.613	2.640	6	1744	ZINC000004099002
25.1	-10.5	-10.2	0.37	1.916	0.646	4.466	2.950	6	1745	ZINC000257558557
25.1	-10.7	-9.6	0.72	1.864	0.502	4.626	1.258	6	1746	ZINC000085944635
25.1	-10.1	-9.7	0.26	1.640	0.394	7.796	2.741	6	1747	ZINC000100825219
25.0	-10.6	-10.0	0.40	3.717	1.145	9.054	3.443	6	1748	ZINC000040165256
25.0	-10.8	-10.2	0.38	2.264	0.139	4.999	0.587	6	1749	ZINC000257426833
25.0	-10.4	-9.1	0.93	2.734	0.557	8.523	2.405	6	1750	ZINC000100827279
25.0	-11.7	-10.1	0.86	2.567	0.554	6.693	2.289	6	1751	ZINC000100829132

25.0	-10.3	-9.4	0.54	2.288	0.211	6.016	1.385	6	1752	ZINC000042806945
25.0	-11.2	-10.4	0.60	1.813	0.687	6.226	4.203	6	1753	ZINC000100824477
25.0	-11.3	-11.0	0.21	2.584	1.024	6.113	3.045	6	1754	ZINC000100781596
25.0	-8.6	-7.0	0.76	2.707	0.161	4.703	0.939	6	1755	ZINC000257752819
24.9	-10.7	-9.9	0.80	2.535	0.099	6.943	0.510	3	1756	ZINC000028116057
24.9	-11.0	-10.1	0.69	2.334	0.139	8.123	3.158	6	1757	ZINC000100825418
24.9	-10.9	-10.0	0.66	1.433	0.678	3.360	3.108	6	1758	ZINC000086044917
24.9	-8.6	-7.3	0.83	2.269	0.098	6.194	0.978	4	1759	ZINC000100828695
24.8	-10.3	-9.7	0.37	2.006	0.720	3.971	2.787	6	1760	ZINC000100823809
24.8	-10.3	-9.7	0.45	1.619	0.399	3.855	2.502	6	1761	ZINC000100823811
24.8	-9.4	-9.0	0.19	1.857	0.584	2.998	0.722	6	1762	ZINC000100773025
24.8	-9.9	-9.4	0.27	1.954	0.312	8.546	3.014	6	1763	ZINC000014709110
24.8	-10.3	-9.6	0.53	2.002	0.629	5.384	3.309	6	1764	ZINC000257404341
24.8	-10.8	-10.1	0.47	1.527	0.504	3.054	1.121	6	1765	ZINC000257374392
24.8	-10.7	-9.8	0.54	1.711	0.513	3.108	1.232	6	1766	ZINC000257454264

24.8	-10.4	-9.7	0.48	2.228	0.297	5.652	2.623	6	1767	ZINC000257553955
24.8	-10.3	-9.5	0.53	1.986	0.525	4.398	2.398	6	1768	ZINC000257404339
24.8	-10.7	-9.5	0.82	1.724	0.717	3.259	1.323	6	1769	ZINC000100825342
24.8	-10.4	-9.9	0.37	1.537	0.576	5.210	3.722	6	1770	ZINC000100822245
24.8	-11.4	-10.8	0.39	2.603	0.342	8.923	2.042	6	1771	ZINC000100829131
24.8	-10.7	-9.8	0.57	1.574	0.628	2.993	1.314	6	1772	ZINC000100825350
24.8	-10.3	-9.5	0.43	2.005	0.354	4.476	2.202	6	1773	ZINC000257404340
24.7	-12.7	-11.0	1.12	2.702	0.315	6.942	1.217	6	1774	ZINC000003978545
24.7	-11.6	-10.5	0.95	2.181	0.392	4.702	2.889	6	1775	ZINC000257415287
24.7	-10.6	-10.2	0.22	2.026	0.185	5.632	2.731	6	1776	ZINC000100776723
24.7	-9.4	-8.2	0.68	1.991	0.592	5.379	3.673	6	1777	ZINC000085907188
24.7	-11.2	-10.3	0.59	1.914	0.519	7.171	1.953	6	1778	ZINC000257548678
24.7	-10.9	-9.7	0.70	1.711	0.578	4.073	2.578	6	1779	ZINC000150351802
24.7	-10.6	-9.5	0.55	1.942	0.371	4.710	1.233	6	1780	ZINC000257454262
24.7	-10.3	-9.4	0.49	2.370	0.626	6.857	3.115	6	1781	ZINC000100823814

24.6	-10.9	-9.9	0.68	1.505	0.529	3.516	1.430	6	1782	ZINC000100771758
24.6	-11.1	-10.4	0.45	2.601	0.429	5.279	0.403	6	1783	ZINC000100827925
24.6	-10.6	-10.3	0.16	2.966	0.570	7.529	1.800	6	1784	ZINC000118937106
24.5	-11.1	-10.6	0.51	2.148	0.562	4.693	2.299	6	1785	ZINC000257439850
24.5	-10.7	-10.1	0.48	1.634	0.341	2.260	0.255	6	1786	ZINC000118936966
24.5	-11.5	-11.4	0.13	2.273	0.241	7.047	1.414	6	1787	ZINC000257410667
24.5	-10.3	-9.8	0.29	2.496	0.424	3.733	0.948	6	1788	ZINC000095619920
24.4	-11.3	-10.9	0.34	2.007	0.994	3.381	1.596	6	1789	ZINC000085935130
24.4	-11.4	-10.8	0.57	1.915	0.337	5.549	3.369	6	1790	ZINC000100829928
24.4	-11.1	-10.6	0.41	2.430	0.294	4.820	0.718	6	1791	ZINC000100827924
24.4	-10.4	-10.1	0.21	1.894	0.455	6.888	2.966	6	1792	ZINC000257455944
24.4	-9.9	-9.0	0.41	2.260	0.344	6.508	1.178	6	1793	ZINC000230104783
24.4	-10.9	-9.4	0.84	2.468	0.364	5.917	0.900	6	1794	ZINC000085886550
24.4	-10.5	-9.8	0.35	1.742	0.131	7.496	2.019	6	1795	ZINC000257557539
24.4	-9.8	-9.3	0.35	2.045	0.600	6.059	1.993	6	1796	ZINC000085546853

24.4	-11.3	-10.0	0.73	2.413	0.557	5.920	2.890	6	1797	ZINC000100829930
24.4	-10.5	-10.1	0.24	2.156	0.553	8.407	1.595	6	1798	ZINC000100827938
24.4	-10.0	-9.5	0.39	2.052	0.588	6.484	0.682	6	1799	ZINC000257535734
24.3	-10.4	-10.1	0.19	2.024	0.372	7.079	2.668	6	1800	ZINC000100827941
24.3	-10.5	-9.9	0.76	2.162	0.647	5.269	3.353	4	1801	ZINC000085907224
24.3	-10.5	-10.2	0.23	2.228	0.424	5.769	2.836	6	1802	ZINC000100825216
24.3	-10.0	-9.7	0.17	2.066	0.672	8.481	3.327	6	1803	ZINC000028541906
24.3	-10.1	-8.7	0.65	2.170	0.840	6.698	2.660	6	1804	ZINC000085550330
24.3	-11.1	-10.6	0.41	2.434	0.293	4.821	0.735	6	1805	ZINC000257600996
24.3	-10.3	-9.1	0.71	2.304	0.265	7.040	0.748	5	1806	ZINC000030726947
24.3	-11.1	-10.6	0.44	2.506	0.210	4.547	0.929	6	1807	ZINC000257600995
24.3	-10.9	-10.1	0.63	1.518	0.527	3.213	1.100	6	1808	ZINC000257526882
24.2	-9.6	-9.4	0.09	2.312	0.457	7.046	3.718	6	1809	ZINC000014709115
24.2	-9.8	-8.8	0.84	1.960	0.525	6.655	2.425	6	1810	ZINC000085558138
24.2	-10.9	-10.0	0.70	1.510	0.530	3.196	1.126	6	1811	ZINC000257526885

24.2	-11.3	-9.7	0.78	2.385	0.316	7.584	3.200	6	1812	ZINC000257778809
24.2	-12.0	-11.4	0.31	2.508	0.560	5.688	2.260	6	1813	ZINC000100783657
24.1	-9.3	-8.7	0.37	1.706	0.945	4.318	2.980	6	1814	ZINC000100825306
24.1	-10.7	-10.2	0.29	1.542	0.328	8.802	3.197	6	1815	ZINC000257697352
24.1	-10.9	-10.0	0.72	1.710	0.613	3.196	1.173	6	1816	ZINC000100771760
24.1	-9.6	-9.2	0.26	2.369	0.457	8.022	3.098	6	1817	ZINC000257404342
24.1	-10.9	-10.0	0.58	1.685	0.596	3.304	1.211	6	1818	ZINC000257526883
24.1	-10.9	-10.1	0.60	1.501	0.510	3.168	1.104	6	1819	ZINC000257526884
24.1	-9.6	-9.1	0.24	2.368	0.477	7.134	3.028	6	1820	ZINC000100823810
24.1	-10.4	-8.8	0.82	2.079	0.310	5.734	3.302	6	1821	ZINC000100830092
24.1	-10.1	-9.4	0.39	2.013	0.584	7.984	2.899	6	1822	ZINC000257534177
24.1	-10.9	-9.6	0.84	2.055	0.755	7.026	3.872	6	1823	ZINC000100771754
24.0	-9.9	-9.1	0.58	2.241	0.738	5.218	2.745	6	1824	ZINC000257403665
24.0	-10.2	-10.0	0.21	2.126	0.358	5.488	2.772	6	1825	ZINC000100780753
24.0	-10.1	-9.6	0.29	1.936	0.204	9.316	0.345	6	1826	ZINC000257534179

23.9	-12.1	-11.8	0.17	2.844	0.540	5.292	2.458	6	1827	ZINC000100777962
23.9	-10.8	-10.3	0.38	1.670	0.304	5.592	4.089	6	1828	ZINC000257697354
23.9	-9.7	-8.7	0.74	2.001	0.100	7.872	0.251	4	1829	ZINC000150338875
23.9	-10.6	-9.4	0.68	1.887	0.613	4.449	2.490	6	1830	ZINC000085907209
23.9	-12.1	-11.8	0.19	3.062	0.394	5.496	2.264	6	1831	ZINC000257445832
23.9	-11.1	-9.4	1.13	1.911	0.175	2.747	0.363	4	1832	ZINC000257458623
23.9	-12.1	-11.8	0.17	2.836	0.540	5.292	2.454	6	1833	ZINC000100777968
23.9	-10.4	-9.4	1.30	3.586	0.539	7.307	0.196	3	1834	ZINC000100828617
23.9	-11.8	-11.3	0.39	2.346	0.963	6.254	3.118	6	1835	ZINC000253629642
23.8	-11.4	-11.0	0.26	2.469	0.915	5.396	2.784	6	1836	ZINC000256602002
23.8	-12.1	-11.3	0.39	3.156	0.572	5.918	2.681	6	1837	ZINC000257544175
23.8	-10.1	-9.7	0.23	1.733	0.440	6.946	2.804	6	1838	ZINC000100825218
23.8	-10.8	-10.1	0.45	1.545	0.507	3.087	1.278	6	1839	ZINC000100825688
23.8	-12.1	-11.6	0.35	3.377	0.098	7.115	1.748	6	1840	ZINC000257445833
23.8	-10.1	-9.7	0.21	2.461	0.513	5.988	3.379	6	1841	ZINC000001630828

23.8	-10.8	-10.1	0.42	2.444	0.388	5.842	2.636	6	1842	ZINC000257514463
23.8	-11.2	-10.5	0.55	1.704	0.178	4.860	2.931	6	1843	ZINC000085886072
23.8	-10.8	-9.9	0.45	2.443	0.368	5.515	2.290	6	1844	ZINC000100825698
23.8	-12.1	-11.7	0.23	3.079	0.387	5.668	2.148	6	1845	ZINC000100777966
23.8	-9.8	-8.5	0.98	2.382	0.333	6.587	1.228	6	1846	ZINC000257628366
23.8	-9.8	-8.7	0.76	2.074	0.218	6.078	1.331	6	1847	ZINC000100828903
23.8	-11.1	-10.5	0.46	1.803	0.506	3.445	1.197	6	1848	ZINC000100829875
23.8	-11.2	-10.9	0.18	2.095	0.904	3.956	2.307	6	1849	ZINC000257411676
23.8	-12.1	-11.7	0.27	3.289	0.110	6.695	2.242	6	1850	ZINC000257445834
23.8	-11.8	-11.1	0.38	2.763	0.409	7.905	2.054	6	1851	ZINC000253629641
23.8	-11.8	-11.1	0.39	2.842	0.343	7.788	2.093	6	1852	ZINC000253629643
23.8	-10.4	-10.3	0.11	1.897	0.595	5.736	3.094	6	1853	ZINC000257507134
23.8	-12.1	-11.8	0.19	3.067	0.370	5.506	2.264	6	1854	ZINC000100777964
23.8	-9.4	-7.8	0.98	2.065	0.504	6.190	2.122	5	1855	ZINC000100773122
23.8	-11.8	-11.1	0.43	2.642	0.533	7.873	2.049	6	1856	ZINC000253629644

23.8	-12.1	-11.6	0.32	2.980	0.381	4.777	1.920	6	1857	ZINC000257445831
23.8	-10.7	-10.2	0.41	1.759	0.189	3.996	2.320	6	1858	ZINC000257557540
23.8	-10.8	-9.9	0.58	1.680	0.518	3.549	1.491	6	1859	ZINC000257374393
23.7	-11.3	-10.1	0.70	2.595	0.157	6.163	1.974	6	1860	ZINC000257452354
23.7	-11.7	-10.2	0.88	2.394	0.727	6.061	3.260	6	1861	ZINC000100829929
23.7	-10.7	-9.9	0.52	1.654	0.497	3.093	1.077	6	1862	ZINC000100825689
23.7	-11.1	-10.7	0.21	2.498	0.209	7.551	2.230	6	1863	ZINC000100824480
23.7	-10.7	-10.3	0.20	1.853	0.311	5.508	2.856	6	1864	ZINC000118937107
23.7	-10.8	-10.2	0.35	1.662	0.391	7.456	3.886	6	1865	ZINC000100828901
23.7	-12.2	-10.9	0.72	2.467	0.703	5.883	2.582	6	1866	ZINC000085567728
23.7	-10.7	-9.9	0.53	1.582	0.484	3.072	1.139	6	1867	ZINC000100825687
23.7	-12.1	-11.4	0.37	2.909	0.591	5.512	2.907	6	1868	ZINC000257544174
23.7	-10.8	-9.9	0.54	2.349	0.260	6.602	3.044	6	1869	ZINC000100825701
23.7	-10.8	-9.5	0.84	2.182	0.873	5.680	3.559	6	1870	ZINC000257374394
23.7	-11.4	-10.3	0.74	2.336	0.119	10.109	0.339	6	1871	ZINC000257475641

23.7	-11.7	-10.5	0.77	1.900	0.389	5.787	2.600	6	1872	ZINC000257455688
23.7	-10.9	-10.1	0.44	2.152	0.270	7.812	1.674	6	1873	ZINC000257493815
23.7	-9.2	-7.9	0.87	2.718	0.820	9.098	3.535	6	1874	ZINC000100825352
23.7	-12.3	-11.4	0.50	2.431	0.360	5.636	1.888	6	1875	ZINC000095619531
23.6	-10.1	-9.6	0.29	2.108	0.319	6.054	3.134	6	1876	ZINC000001630830
23.6	-10.1	-10.0	0.11	2.129	0.290	3.175	0.688	6	1877	ZINC000014829482
23.6	8.2	9.4	0.70	2.474	0.257	3.714	0.651	6	1878	ZINC000169712282
23.6	-11.6	-10.5	0.95	2.130	0.382	6.462	2.624	6	1879	ZINC000100777076
23.6	-10.5	-10.2	0.22	2.048	0.739	5.580	3.150	6	1880	ZINC000257507135
23.5	-10.2	-10.1	0.12	2.215	0.447	8.213	2.640	6	1881	ZINC000257507133
23.5	-13.8	-12.2	1.13	2.450	0.377	5.086	2.758	5	1882	ZINC000100782354
23.5	-13.7	-12.8	0.82	2.141	0.721	3.593	0.881	6	1883	ZINC000100782357
23.5	-10.4	-10.3	0.12	1.932	0.619	5.830	3.201	6	1884	ZINC000100825704
23.5	-10.4	-9.2	0.98	2.582	0.694	6.083	2.726	6	1885	ZINC000100823246
23.5	-10.8	-9.9	0.58	1.565	0.475	3.070	1.066	6	1886	ZINC000257374395

23.4	-13.0	-12.4	0.31	2.222	0.798	4.165	2.124	6	1887	ZINC000257538750
23.4	-12.0	-11.2	0.40	2.001	0.381	7.189	2.377	6	1888	ZINC000085664954
23.4	-13.0	-12.2	0.49	2.407	0.847	4.791	1.838	6	1889	ZINC000257538751
23.4	-12.6	-12.0	0.41	2.987	0.248	5.471	2.505	6	1890	ZINC000257749093
23.4	-13.0	-12.2	0.43	2.296	0.717	6.219	2.423	6	1891	ZINC000257538753
23.4	-12.6	-11.9	0.48	2.708	0.625	6.325	2.372	6	1892	ZINC000257749089
23.4	-9.4	-9.2	0.14	1.819	0.127	7.340	3.232	6	1893	ZINC000257395220
23.4	-13.0	-12.5	0.33	2.157	0.703	4.109	2.116	6	1894	ZINC000100782754
23.4	-13.7	-12.8	0.76	2.847	0.562	6.464	2.780	6	1895	ZINC000257534786
23.4	-12.6	-11.9	0.46	2.742	0.654	6.432	2.469	6	1896	ZINC000100777913
23.4	-10.8	-9.8	0.56	1.777	0.451	3.617	1.427	6	1897	ZINC000100825686
23.4	-12.6	-11.9	0.47	2.289	0.716	5.486	2.509	6	1898	ZINC000257749091
23.4	-13.0	-12.4	0.33	2.255	0.799	4.200	2.094	6	1899	ZINC000100782759
23.4	-11.0	-10.7	0.17	1.755	0.364	5.890	3.051	6	1900	ZINC000100780009
23.4	-12.6	-12.0	0.36	2.679	0.453	4.072	2.176	6	1901	ZINC000100777915

23.4	-9.8	-9.5	0.20	2.816	0.548	6.958	3.760	6	1902	ZINC000013322022
23.4	-12.6	-12.0	0.40	2.820	0.518	5.155	2.830	6	1903	ZINC000100777911
23.4	-10.7	-9.1	1.27	2.373	0.188	7.245	3.066	4	1904	ZINC000085907219
23.4	-12.6	-12.0	0.43	2.502	0.728	5.161	2.854	6	1905	ZINC000257749090
23.4	-13.0	-12.5	0.31	2.421	0.824	4.531	1.858	6	1906	ZINC000100782753
23.4	-11.4	-9.7	0.79	2.713	0.227	6.776	1.959	6	1907	ZINC000100829259
23.4	-13.0	-12.3	0.40	2.111	0.671	4.986	2.699	6	1908	ZINC000257538752
23.4	-10.1	-9.6	0.21	1.746	0.347	6.829	3.591	6	1909	ZINC000001630827
23.4	-10.8	-10.0	0.43	1.769	0.077	6.064	3.037	6	1910	ZINC000100775270
23.4	-13.7	-12.9	0.65	2.530	0.452	3.874	0.527	6	1911	ZINC000100782362
23.4	-13.7	-12.7	0.77	2.390	0.689	4.994	2.504	6	1912	ZINC000100782359
23.4	-12.6	-11.9	0.46	3.089	0.171	6.427	2.466	6	1913	ZINC000100777909
23.3	-10.9	-10.3	0.34	1.772	0.120	3.348	0.205	6	1914	ZINC000257673738
23.3	-10.8	-10.1	0.45	1.354	0.463	2.555	0.760	6	1915	ZINC000257534557
23.3	-10.6	-10.0	0.37	2.100	0.496	7.244	3.301	6	1916	ZINC000253529539

23.3	-13.0	-12.4	0.35	2.073	0.888	5.520	2.454	6	1917	ZINC000100782757
23.3	-10.8	-9.5	0.84	2.009	0.839	5.650	3.486	6	1918	ZINC000100783659
23.3	-11.3	-10.7	0.32	2.422	0.507	5.391	2.826	6	1919	ZINC000095619977
23.3	-13.7	-12.7	0.64	2.440	0.989	5.894	3.235	6	1920	ZINC000257534787
23.3	-10.4	-10.2	0.15	1.869	0.586	4.509	2.643	6	1921	ZINC000100825705
23.3	-9.6	-7.6	1.00	2.352	0.316	6.888	1.115	5	1922	ZINC000257532060
23.3	-10.6	-10.2	0.27	1.599	0.452	3.791	1.154	6	1923	ZINC000253529536
23.2	-11.6	-10.7	0.58	1.828	0.212	5.225	3.235	6	1924	ZINC000150366684
23.2	-11.0	-10.1	0.42	2.425	0.341	8.215	1.483	6	1925	ZINC000100776724
23.2	-11.0	-10.0	0.66	2.051	0.392	5.007	2.557	6	1926	ZINC000257673736
23.2	-10.2	-9.7	0.31	2.648	0.422	5.106	2.479	6	1927	ZINC000257514461
23.2	-11.1	-10.9	0.19	2.018	0.419	3.758	1.136	6	1928	ZINC000100826055
23.2	-11.1	-10.0	0.68	2.421	0.606	7.133	2.579	6	1929	ZINC000257439849
23.1	-10.5	-10.3	0.17	1.975	0.459	5.905	2.896	6	1930	ZINC000257507136
23.1	-11.0	-10.2	0.64	2.125	0.093	6.034	2.255	6	1931	ZINC000257415288

23.1	-10.1	-10.1	0.00	0.000	0.000	0.000	0.000	1	1932	ZINC000095098883
23.1	-10.9	-10.4	0.25	1.937	0.284	3.996	0.829	6	1933	ZINC000257673733
23.1	-10.9	-10.4	0.26	1.804	0.187	3.728	0.913	6	1934	ZINC000100828894
23.1	-10.2	-9.4	0.64	1.856	0.364	5.566	3.717	6	1935	ZINC000100777079
23.1	-11.0	-10.0	0.71	1.889	0.526	4.750	2.707	6	1936	ZINC000100828895
23.1	-10.9	-10.4	0.29	1.878	0.289	3.474	0.470	6	1937	ZINC000100828891
23.1	-6.9	-6.0	0.81	1.772	0.291	6.619	2.379	4	1938	ZINC000100773128
23.1	-10.5	-9.9	0.35	1.614	0.411	5.868	3.687	6	1939	ZINC000100776698
23.1	-11.3	-10.1	0.79	1.963	0.466	8.026	2.743	6	1940	ZINC000257415873
23.1	-10.9	-10.2	0.49	1.775	0.095	3.304	0.137	6	1941	ZINC000257673739
23.1	-10.8	-9.9	0.59	1.655	0.569	3.234	1.127	6	1942	ZINC000257534554
23.1	-11.2	-10.5	0.39	2.412	0.378	6.592	0.963	6	1943	ZINC000042806955
23.0	-11.1	-10.8	0.23	1.572	0.437	2.703	0.779	6	1944	ZINC000257439848
23.0	-10.4	-10.3	0.15	1.909	0.591	5.665	3.127	6	1945	ZINC000100825706
23.0	-13.3	-12.0	0.90	2.253	0.359	5.775	2.656	6	1946	ZINC000257534789

23.0	-11.1	-10.7	0.21	1.917	0.195	4.822	2.552	6	1947	ZINC000100782170
23.0	-10.2	-10.0	0.20	1.802	0.289	5.566	3.125	6	1948	ZINC000100825708
23.0	-8.5	-8.2	0.21	1.407	0.397	4.234	3.143	3	1949	ZINC000257397849
23.0	-10.9	-10.3	0.37	1.665	0.156	3.135	0.348	6	1950	ZINC000100828892
23.0	-11.2	-10.2	0.52	3.221	0.669	6.910	3.020	6	1951	ZINC000100774257
23.0	-10.8	-10.1	0.45	1.645	0.314	7.331	4.014	6	1952	ZINC000100828897
23.0	-11.2	-10.1	0.53	3.217	0.686	6.907	3.047	6	1953	ZINC000256070131
22.9	-11.1	-10.6	0.46	1.619	0.390	2.847	0.674	6	1954	ZINC000100829876
22.9	-9.2	-8.8	0.24	2.887	0.359	9.345	3.138	6	1955	ZINC000004228270
22.9	-11.4	-10.4	0.92	2.097	0.763	5.995	1.586	6	1956	ZINC000150359924
22.9	-11.4	-11.2	0.20	1.805	0.187	3.114	0.869	6	1957	ZINC000085645395
22.9	-11.2	-10.7	0.28	2.473	0.560	6.857	1.969	6	1958	ZINC000257738518
22.9	-10.3	-9.8	0.43	1.897	0.179	7.221	3.549	6	1959	ZINC000100829100
22.9	-10.8	-9.8	0.53	2.082	0.187	5.185	2.683	6	1960	ZINC000100780750
22.9	-10.1	-9.9	0.14	2.299	0.379	3.931	1.040	6	1961	ZINC000100825608

22.9	-10.8	-10.3	0.31	1.673	0.404	8.961	3.298	6	1962	ZINC000100828899
22.9	-11.4	-9.6	0.88	1.918	0.141	5.820	1.630	6	1963	ZINC000257455691
22.8	-9.5	-9.0	0.24	1.960	0.366	5.428	3.116	6	1964	ZINC000100771764
22.8	-10.9	-10.0	0.53	1.684	0.530	3.194	1.280	6	1965	ZINC000100820515
22.8	-11.2	-10.7	0.33	1.946	0.104	5.187	2.607	6	1966	ZINC000257511791
22.8	-11.6	-10.8	0.51	2.410	0.542	5.266	2.387	6	1967	ZINC000136693003
22.8	-11.1	-10.3	0.53	1.995	0.680	5.246	3.209	6	1968	ZINC000100829878
22.8	-11.3	-10.7	0.36	1.726	0.461	5.081	3.824	6	1969	ZINC000257475638
22.8	-11.1	-10.7	0.22	2.877	0.780	6.830	2.703	6	1970	ZINC000256602007
22.7	-10.9	-9.9	0.60	1.590	0.562	3.418	1.565	6	1971	ZINC000257534555
22.7	-11.6	-10.9	0.34	2.203	0.418	5.425	2.416	6	1972	ZINC000100823644
22.7	-10.9	-9.9	0.62	1.601	0.615	3.443	1.531	6	1973	ZINC000100820517
22.7	-10.8	-9.9	0.60	1.662	0.628	3.751	1.398	6	1974	ZINC000257534556
22.7	-10.7	-9.4	0.85	2.045	0.456	4.751	2.519	6	1975	ZINC000095619922
22.7	-9.4	-8.4	0.62	2.265	0.683	6.331	3.567	6	1976	ZINC000162000754

22.7	-11.9	-11.0	0.46	2.637	0.709	6.329	2.166	6	1977	ZINC000014720200
22.7	-10.6	-9.6	0.70	1.898	0.824	5.807	2.375	6	1978	ZINC000000897910
22.6	-10.9	-10.5	0.46	1.647	0.896	6.306	3.962	6	1979	ZINC000056874794
22.6	-8.9	-8.5	0.34	2.562	0.320	7.925	3.616	6	1980	ZINC000100048501
22.6	-11.9	-10.5	0.68	2.605	0.739	6.147	2.195	6	1981	ZINC000100783655
22.6	-10.9	-9.8	0.66	1.693	0.585	3.787	1.391	6	1982	ZINC000100820514
22.6	-10.1	-9.6	0.23	2.112	0.297	6.202	2.773	6	1983	ZINC000100776695
22.6	-10.3	-9.9	0.23	2.191	0.627	5.335	3.212	6	1984	ZINC000257392525
22.6	-11.1	-10.8	0.27	1.590	0.400	2.846	0.595	6	1985	ZINC000100829872
22.6	-10.9	-9.8	0.61	1.613	0.442	3.298	1.037	6	1986	ZINC000100820516
22.6	-12.4	-11.8	0.42	2.495	0.412	7.054	2.553	6	1987	ZINC000086034619
22.6	-10.0	-9.0	0.56	2.412	0.211	7.292	2.754	6	1988	ZINC000150352530
22.6	-11.7	-11.0	0.38	1.692	0.108	4.944	2.559	6	1989	ZINC000257380437
22.6	-10.3	-9.8	0.22	2.862	0.116	9.343	0.118	6	1990	ZINC000238730635
22.6	-10.6	-10.2	0.23	1.969	0.168	5.108	2.819	6	1991	ZINC000257557541

22.6	-11.1	-10.6	0.35	2.187	0.131	3.790	0.935	6	1992	ZINC000257600998
22.6	-10.3	-10.0	0.25	2.352	0.670	6.681	3.363	6	1993	ZINC000257392527
22.5	-9.3	-8.7	0.36	1.847	1.062	5.579	3.447	6	1994	ZINC000100825304
22.5	-11.0	-9.8	0.60	2.865	0.764	8.042	2.502	6	1995	ZINC000257435769
22.5	-10.2	-9.9	0.29	2.310	0.555	8.162	2.676	6	1996	ZINC000100826032
22.5	-12.6	-12.2	0.25	2.115	0.827	5.127	2.227	6	1997	ZINC000257512907
22.5	-12.6	-12.2	0.29	2.106	0.821	5.136	2.223	6	1998	ZINC000100778072
22.5	-9.0	-7.9	0.55	2.365	0.622	5.958	3.668	6	1999	ZINC000150362035
22.5	-11.1	-10.4	0.45	2.364	0.751	7.098	1.848	6	2000	ZINC000100775819
22.5	-11.4	-10.9	0.31	1.990	0.678	4.576	2.904	6	2001	ZINC000014610661
22.5	-10.2	-9.9	0.20	2.512	0.669	6.731	3.277	6	2002	ZINC000100826031
22.5	-11.1	-10.5	0.37	1.945	0.711	5.606	2.146	6	2003	ZINC000100775823
22.5	-11.8	-10.9	0.57	2.514	0.353	7.705	1.022	6	2004	ZINC000003978504
22.5	-10.8	-9.9	0.96	2.564	0.307	7.828	2.839	4	2005	ZINC000085886071
22.5	-10.2	-10.0	0.17	2.269	0.698	6.786	3.325	6	2006	ZINC000100826033

22.5	-9.3	-8.7	0.40	1.278	0.896	2.880	1.231	6	2007	ZINC000257508868
22.5	-11.1	-10.5	0.37	1.919	0.725	5.660	2.118	6	2008	ZINC000257435770
22.5	-12.6	-12.2	0.30	1.763	0.841	4.554	2.733	6	2009	ZINC000100778074
22.5	-11.1	-10.4	0.44	2.237	0.894	6.433	2.648	6	2010	ZINC000257435772
22.5	-10.2	-9.9	0.20	2.554	0.639	8.105	2.659	6	2011	ZINC000100826030
22.5	-11.1	-10.5	0.35	2.007	0.753	5.668	2.285	6	2012	ZINC000257435771
22.5	-11.9	-11.1	0.45	2.072	0.303	6.582	2.506	6	2013	ZINC000085953513
22.5	-9.3	-8.7	0.34	1.490	0.965	2.916	1.226	6	2014	ZINC000257508867
22.4	-10.2	-9.9	0.22	2.349	0.837	6.460	3.403	6	2015	ZINC000257392526
22.4	-10.0	-9.1	0.56	2.208	0.657	5.747	2.532	6	2016	ZINC000085550327
22.4	-12.6	-12.2	0.24	1.986	0.733	4.218	1.815	6	2017	ZINC000257512906
22.4	-11.0	-10.3	0.41	2.160	0.938	6.689	2.931	6	2018	ZINC000100775825
22.4	-9.9	-8.8	0.54	3.150	0.508	8.389	3.732	6	2019	ZINC000095620839
22.4	-11.1	-10.0	0.62	2.060	0.560	6.660	3.638	6	2020	ZINC000085991643
22.4	-11.8	-10.5	0.90	2.479	0.394	7.615	1.829	6	2021	ZINC000257612629

22.4	-9.3	-9.1	0.17	1.785	0.980	4.813	2.873	6	2022	ZINC000410428651
22.4	-11.8	-11.3	0.27	2.556	0.718	5.613	2.474	6	2023	ZINC000100777779
22.4	-12.5	-12.1	0.28	2.659	0.718	6.226	2.276	6	2024	ZINC000257512905
22.4	-8.9	-7.9	0.70	2.583	0.330	8.063	1.287	6	2025	ZINC000257501004
22.4	-10.5	-9.3	0.88	2.337	0.167	7.763	2.178	5	2026	ZINC000150362041
22.4	-11.0	-10.5	0.32	1.673	0.361	4.863	1.246	6	2027	ZINC000100775821
22.4	-12.6	-12.2	0.22	2.299	0.722	4.234	1.852	6	2028	ZINC000257512908
22.4	-11.4	-9.9	0.77	2.412	0.090	7.039	1.694	6	2029	ZINC000085664944
22.4	-10.7	-9.5	0.74	2.038	0.832	6.439	2.532	6	2030	ZINC000044351309
22.4	-11.6	-10.9	0.36	1.732	0.327	5.104	3.050	6	2031	ZINC000100830091
22.3	-11.8	-11.3	0.26	2.373	0.756	5.287	2.794	6	2032	ZINC000257544173
22.3	-10.1	-9.6	0.42	1.684	0.621	3.256	0.955	6	2033	ZINC000257505558
22.3	-8.5	-7.0	0.92	2.119	0.127	6.759	1.480	5	2034	ZINC000257463095
22.3	-11.7	-10.6	0.65	2.297	0.305	6.503	2.162	6	2035	ZINC000150361952
22.3	-9.3	-8.6	0.41	1.509	0.892	4.268	2.994	6	2036	ZINC000100825308

22.3	-11.7	-11.1	0.29	2.628	0.894	6.016	2.150	6	2037	ZINC000257544176
22.3	-10.1	-9.6	0.42	1.288	0.172	2.406	0.361	6	2038	ZINC000100822626
22.3	-10.1	-9.3	0.43	1.899	0.745	3.018	0.985	6	2039	ZINC000100822622
22.3	-10.1	-9.6	0.37	1.711	0.600	3.133	0.951	6	2040	ZINC000257505557
22.3	-11.1	-10.9	0.09	2.408	0.361	6.609	2.279	6	2041	ZINC000257480079
22.3	-10.1	-9.5	0.39	1.831	0.541	3.380	0.854	6	2042	ZINC000100822625
22.3	-10.0	-9.7	0.21	1.668	0.188	6.274	2.439	6	2043	ZINC000257396948
22.2	-8.3	-7.8	0.26	1.963	0.194	6.344	0.784	6	2044	ZINC000257628369
22.2	-10.1	-9.5	0.42	1.788	0.621	3.045	1.023	6	2045	ZINC000100822623
22.2	-10.1	-9.6	0.32	1.504	0.461	2.908	0.740	6	2046	ZINC000257505556
22.2	-11.7	-11.2	0.31	2.964	0.869	5.351	1.916	6	2047	ZINC000257610573
22.2	-10.1	-9.5	0.42	1.944	0.672	3.553	1.067	6	2048	ZINC000257505555
22.2	-11.0	-10.7	0.15	3.178	0.557	7.550	1.700	6	2049	ZINC000014720198
22.2	-11.2	-10.2	0.64	2.026	0.307	5.928	1.131	6	2050	ZINC000100783888
22.2	-10.1	-9.2	0.59	2.157	0.297	7.243	1.612	6	2051	ZINC000257435967

22.2	-10.8	-10.4	0.29	1.695	0.345	5.729	3.345	6	2052	ZINC000257567963
22.2	-11.8	-11.2	0.28	2.223	0.758	4.414	2.255	6	2053	ZINC000100777783
22.2	-11.7	-11.2	0.31	2.625	0.646	5.916	2.307	6	2054	ZINC000100778566
22.2	-11.5	-10.9	0.37	2.646	0.442	5.831	1.588	6	2055	ZINC000100823643
22.2	-10.6	-10.0	0.44	1.806	0.486	6.218	3.532	6	2056	ZINC000100776692
22.1	-10.8	-10.0	0.80	1.664	0.722	4.424	3.161	4	2057	ZINC000150361957
22.1	-11.5	-10.8	0.34	2.239	0.873	5.030	2.996	6	2058	ZINC000034037251
22.1	-11.2	-11.1	0.14	1.822	0.418	6.248	2.499	6	2059	ZINC000100823637
22.1	-10.8	-10.1	0.41	2.047	0.309	5.612	2.243	6	2060	ZINC000257514460
22.1	-11.0	-9.8	0.82	2.254	0.634	6.321	2.542	6	2061	ZINC000100823808
22.1	-11.9	-11.6	0.44	2.810	0.599	3.463	0.746	4	2062	ZINC000230085618
22.1	-11.8	-11.2	0.37	2.795	0.875	6.233	2.140	6	2063	ZINC000100778574
22.1	-10.5	-9.6	0.49	2.017	0.398	5.858	3.867	6	2064	ZINC000257595497
22.1	-6.2	-5.2	0.92	2.590	0.465	6.054	0.789	6	2065	ZINC000257397848
22.1	-10.6	-10.2	0.24	2.136	0.999	8.097	3.254	6	2066	ZINC000257414162

22.1	-10.7	-10.1	0.44	2.070	0.092	8.510	2.333	6	2067	ZINC000150361960
22.1	-11.7	-11.2	0.32	2.783	0.872	6.223	2.148	6	2068	ZINC000100778569
22.1	-11.2	-10.7	0.24	2.194	0.587	4.031	1.965	6	2069	ZINC000014768740
22.1	-10.6	-10.4	0.15	2.591	0.378	8.952	2.848	6	2070	ZINC000042805482
22.1	-11.7	-11.2	0.33	3.151	0.600	5.264	1.747	6	2071	ZINC000257610576
22.0	-9.3	-8.8	0.33	1.469	0.979	2.898	1.225	6	2072	ZINC000257508869
22.0	-10.8	-10.3	0.34	1.950	0.496	3.821	0.870	6	2073	ZINC000257399447
22.0	-11.1	-10.4	0.45	1.893	0.629	4.109	2.615	6	2074	ZINC000257439851
22.0	-12.7	-11.5	0.64	2.293	0.220	7.074	2.205	6	2075	ZINC000257613185
22.0	-11.7	-10.8	0.57	2.580	0.412	6.142	1.944	6	2076	ZINC000095619716
22.0	-11.1	-10.0	0.75	2.153	0.199	6.336	1.453	6	2077	ZINC000257526824
22.0	-10.8	-10.2	0.32	2.068	0.671	5.680	2.818	6	2078	ZINC000257399446
21.9	-11.6	-10.7	0.52	1.786	0.363	6.596	2.324	6	2079	ZINC000100783883
21.9	-10.7	-9.5	0.80	2.642	0.549	6.525	0.756	6	2080	ZINC000085886552
21.9	-9.3	-8.8	0.31	1.468	0.980	2.899	1.232	6	2081	ZINC000257508870

21.9	-10.0	-9.7	0.22	2.054	0.426	7.117	3.488	6	2082	ZINC000002572533
21.9	-11.6	-11.1	0.27	2.516	0.829	7.137	2.190	6	2083	ZINC000257610569
21.9	-9.3	-8.8	0.34	1.602	1.118	4.154	2.862	6	2084	ZINC000100825301
21.9	-11.2	-10.7	0.27	2.512	0.427	4.465	1.101	6	2085	ZINC000085956584
21.9	-11.7	-11.1	0.28	2.904	0.899	6.277	2.094	6	2086	ZINC000257610577
21.9	-10.8	-10.4	0.32	2.360	0.285	5.176	1.753	6	2087	ZINC000257399445
21.8	-10.1	-9.7	0.27	2.150	0.975	3.413	1.158	4	2088	ZINC000257573398
21.8	-10.8	-10.4	0.20	1.924	0.860	3.632	2.499	6	2089	ZINC000100778648
21.8	-10.9	-10.5	0.33	1.764	0.863	3.246	1.607	6	2090	ZINC000085935134
21.8	-10.7	-10.2	0.36	2.186	0.102	5.186	2.041	6	2091	ZINC000100828644
21.8	-5.5	-5.0	0.30	1.893	0.709	8.078	1.262	6	2092	ZINC000100491657
21.8	-11.6	-11.1	0.27	2.555	0.824	7.138	2.193	6	2093	ZINC000100778571
21.8	-10.1	-9.8	0.19	2.260	0.885	6.511	3.355	6	2094	ZINC000257542399
21.8	-10.1	-9.8	0.21	2.035	0.724	6.532	3.374	6	2095	ZINC000100829072
21.8	-6.4	-5.0	0.99	2.072	0.466	6.647	1.085	6	2096	ZINC000100773127

21.8	-11.9	-11.5	0.31	2.259	0.514	7.102	2.709	6	2097	ZINC000086034583
21.7	-10.4	-10.2	0.19	2.116	0.316	4.132	0.631	6	2098	ZINC000100826064
21.7	-10.0	-9.7	0.30	2.009	0.722	6.425	3.293	6	2099	ZINC000100829075
21.7	-10.0	-9.8	0.15	1.985	0.766	5.209	3.287	6	2100	ZINC000257542396
21.7	-11.5	-9.9	0.80	2.345	0.488	5.507	1.807	6	2101	ZINC000100823804
21.7	-10.0	-9.7	0.18	1.905	0.801	5.096	3.349	6	2102	ZINC000100829071
21.7	-10.7	-10.3	0.22	2.205	0.546	5.262	2.643	6	2103	ZINC000253604951
21.7	-10.0	-9.7	0.23	2.511	0.618	8.035	2.843	6	2104	ZINC000085530963
21.7	-10.0	-9.7	0.20	2.130	0.823	6.509	3.355	6	2105	ZINC000257542398
21.7	-12.3	-11.2	0.62	2.144	0.502	5.346	1.902	6	2106	ZINC000257526821
21.7	-10.0	-9.8	0.16	1.951	0.720	5.224	3.284	6	2107	ZINC000257542397
21.7	-11.8	-11.3	0.42	3.023	0.573	5.353	2.827	6	2108	ZINC000257399764
21.7	-11.8	-11.2	0.53	2.894	0.828	6.711	2.552	6	2109	ZINC000257399762
21.6	-9.0	-8.8	0.11	1.330	0.779	4.048	3.231	6	2110	ZINC000095619631
21.6	-12.2	-11.5	0.59	2.365	0.277	6.510	2.374	6	2111	ZINC000230066700

21.6	-9.0	-8.8	0.16	1.488	0.792	4.189	3.171	6	2112	ZINC000100825268
21.6	-9.0	-8.7	0.23	1.641	0.734	5.763	3.854	6	2113	ZINC000257517335
21.6	-9.0	-8.8	0.13	1.494	0.800	5.643	3.921	6	2114	ZINC000059727283
21.6	-9.0	-8.8	0.16	1.420	0.760	3.916	3.329	6	2115	ZINC000059727301
21.6	-9.9	-9.6	0.15	2.433	0.560	7.908	2.886	6	2116	ZINC000100829069
21.6	-9.0	-8.8	0.16	1.496	0.789	4.194	3.173	6	2117	ZINC000085804579
21.6	-9.0	-8.8	0.16	1.447	0.774	4.249	3.155	6	2118	ZINC000257517333
21.6	-9.0	-8.8	0.16	1.455	0.767	5.714	4.007	6	2119	ZINC000257517334
21.6	-9.0	-8.8	0.16	1.448	0.777	5.714	4.008	6	2120	ZINC000059727308
21.6	-11.5	-11.1	0.22	2.600	0.955	5.908	2.784	6	2121	ZINC000013375695
21.6	-9.0	-8.8	0.16	1.408	0.767	4.101	3.205	6	2122	ZINC000100825266
21.5	-12.7	-12.1	0.31	1.919	0.623	5.692	3.134	6	2123	ZINC000100823673
21.5	-12.7	-11.5	1.20	3.193	0.000	5.775	0.000	2	2124	ZINC000257526823
21.5	-11.3	-11.0	0.20	1.755	0.452	3.985	1.926	6	2125	ZINC000100777654
21.5	-10.8	-10.1	0.37	2.319	0.426	5.529	2.269	6	2126	ZINC000100825702

21.5	-12.7	-12.1	0.30	2.743	0.805	8.645	0.432	6	2127	ZINC000256371658
21.5	-11.9	-10.8	0.84	2.090	0.084	6.886	3.178	5	2128	ZINC000100783650
21.5	-11.0	-10.6	0.30	2.318	0.269	4.392	0.856	6	2129	ZINC000230075609
21.5	-11.7	-10.8	0.41	1.881	0.377	6.000	2.667	6	2130	ZINC000257480081
21.5	-10.6	-9.8	0.51	2.474	0.737	6.051	2.809	6	2131	ZINC000257573397
21.5	-12.7	-12.2	0.29	2.389	0.735	5.253	2.843	6	2132	ZINC000256371656
21.5	-11.4	-10.8	0.43	2.190	0.356	6.842	2.964	6	2133	ZINC000100783813
21.5	-10.7	-10.2	0.41	1.743	0.246	3.981	3.000	6	2134	ZINC000257612630
21.5	-12.3	-10.5	0.94	2.110	0.429	5.838	2.022	6	2135	ZINC000024215062
21.5	-12.7	-12.2	0.31	2.480	0.707	6.469	2.559	6	2136	ZINC000256371659
21.5	-10.5	-9.5	0.56	1.749	0.725	5.797	2.576	6	2137	ZINC000085744299
21.5	-8.9	-7.3	0.88	2.227	0.786	6.260	2.157	6	2138	ZINC000150351797
21.4	-12.7	-12.3	0.27	2.648	0.732	6.684	2.358	6	2139	ZINC000100823671
21.4	-11.0	-10.1	0.65	1.861	0.572	5.408	3.123	6	2140	ZINC000257772037
21.4	-9.5	-8.1	1.26	2.196	0.320	6.586	0.868	6	2141	ZINC000086053088

21.4	-11.5	-10.3	0.54	2.822	0.822	5.540	3.013	6	2142	ZINC000256070129
21.4	-12.5	-12.0	0.37	2.763	0.502	5.139	2.733	6	2143	ZINC000257399763
21.4	-12.5	-12.0	0.32	2.769	0.517	4.212	2.243	6	2144	ZINC000257399761
21.4	-12.6	-12.1	0.24	2.448	0.758	6.188	2.850	6	2145	ZINC000256371657
21.4	-10.2	-9.8	0.27	1.663	0.327	5.901	2.431	6	2146	ZINC000100828646
21.4	-12.5	-12.0	0.32	2.767	0.499	4.211	2.234	6	2147	ZINC000100777750
21.4	-10.7	-9.8	0.52	2.495	0.779	7.156	3.529	6	2148	ZINC000085931567
21.4	-12.5	-11.7	0.37	2.431	0.640	7.471	1.864	6	2149	ZINC000100777742
21.4	-10.8	-10.3	0.32	1.921	0.173	6.926	1.034	6	2150	ZINC000257391641
21.3	-9.0	-8.8	0.12	1.993	0.263	8.333	2.223	6	2151	ZINC000257452007
21.3	-10.2	-9.7	0.39	1.676	0.300	6.575	2.567	6	2152	ZINC000100828641
21.3	-11.1	-10.8	0.16	2.246	0.397	4.191	2.840	6	2153	ZINC000085826835
21.3	-10.2	-9.7	0.32	1.653	0.481	5.289	1.994	6	2154	ZINC000100828643
21.3	-10.7	-10.5	0.25	1.619	0.801	6.355	3.399	6	2155	ZINC000100826638
21.3	-9.8	-9.3	0.23	2.015	0.507	7.481	3.681	6	2156	ZINC000012654552

21.3	-11.0	-10.6	0.22	2.696	0.611	5.810	2.160	6	2157	ZINC000014610655
21.3	-10.2	-9.7	0.39	2.288	0.068	4.252	0.331	6	2158	ZINC000095617917
21.3	-11.5	-10.5	0.63	2.364	0.291	6.188	2.605	6	2159	ZINC000085991637
21.3	-10.6	-9.7	0.43	1.765	0.195	8.637	2.953	6	2160	ZINC000100038169
21.2	-11.2	-10.8	0.40	2.341	0.214	6.071	2.014	6	2161	ZINC000150351561
21.2	-10.7	-10.3	0.32	2.321	0.625	4.998	1.883	6	2162	ZINC000100772623
21.2	-11.0	-10.7	0.23	2.446	0.912	5.795	3.189	6	2163	ZINC000256602011
21.2	-10.7	-9.9	0.56	2.109	0.513	5.970	2.464	6	2164	ZINC000100772621
21.2	-10.2	-9.5	0.33	1.746	0.570	6.457	3.044	6	2165	ZINC000100825240
21.1	-9.1	-8.7	0.21	1.687	0.539	4.211	2.996	6	2166	ZINC000100825685
21.1	-9.4	-9.0	0.35	1.782	0.268	7.253	4.043	6	2167	ZINC000257775069
21.1	-11.1	-10.3	0.52	3.039	0.194	7.109	1.863	6	2168	ZINC000100777360
21.1	-11.6	-10.4	0.61	2.006	0.278	6.078	2.595	6	2169	ZINC000100783890
21.1	-11.1	-10.4	0.46	2.392	0.710	6.303	1.943	6	2170	ZINC000100777369
21.1	-10.2	-10.0	0.15	2.361	0.493	5.240	2.616	6	2171	ZINC000031156987

21.1	-10.6	-10.3	0.31	2.319	0.623	4.988	1.882	6	2172	ZINC000100772618
21.1	-11.1	-10.6	0.36	2.889	0.760	5.426	1.726	6	2173	ZINC000100777366
21.1	-9.7	-9.5	0.10	1.990	0.287	7.263	3.202	6	2174	ZINC000043704779
21.1	-10.5	-9.8	0.43	2.069	0.277	7.117	2.676	6	2175	ZINC000100825696
21.1	-11.1	-10.5	0.38	2.873	0.750	5.425	1.743	6	2176	ZINC000100777362
21.1	-10.7	-10.3	0.33	2.324	0.625	4.997	1.887	6	2177	ZINC000100772616
21.1	-12.4	-11.1	0.82	2.601	0.352	6.302	2.609	6	2178	ZINC000049889144
21.0	-10.5	-10.3	0.11	1.946	0.419	3.726	1.023	6	2179	ZINC000100826058
21.0	-10.1	-9.3	0.59	2.218	0.609	6.469	3.524	6	2180	ZINC000095620800
21.0	15.2	16.5	0.90	2.292	0.554	3.171	0.927	3	2181	ZINC000095618352
21.0	-12.3	-10.7	0.89	2.057	0.495	4.603	2.854	6	2182	ZINC000100826209
21.0	-11.4	-10.9	0.25	1.931	0.292	4.169	2.052	6	2183	ZINC000253477227
20.9	-12.7	-11.1	1.16	2.315	1.005	4.612	2.670	6	2184	ZINC000257480399
20.9	-11.2	-10.7	0.29	2.128	0.618	5.704	2.604	6	2185	ZINC000257738523
20.9	-10.2	-9.8	0.26	2.722	0.509	5.366	2.495	6	2186	ZINC000257514462

20.9	-11.5	-10.4	0.56	3.396	0.714	7.012	2.881	6	2187	ZINC000256070132
20.9	-10.5	-9.8	0.46	2.481	0.455	5.253	2.182	6	2188	ZINC000100827928
20.9	-10.6	-10.3	0.22	2.368	0.356	9.184	2.302	6	2189	ZINC000086033451
20.9	-9.1	-9.0	0.11	2.048	0.570	5.286	3.272	6	2190	ZINC000257452006
20.8	-8.6	-7.5	1.10	2.215	0.000	7.736	0.000	2	2191	ZINC000257378053
20.8	-11.8	-10.9	0.52	2.530	0.408	6.949	1.514	6	2192	ZINC000095619715
20.8	-11.1	-10.7	0.34	2.386	0.396	5.428	2.400	6	2193	ZINC000013373231
20.8	-11.0	-9.9	0.64	2.202	0.630	5.378	3.235	6	2194	ZINC000100777352
20.8	-9.8	-9.1	0.45	2.860	0.327	6.734	1.009	6	2195	ZINC000100829298
20.8	-10.8	-10.5	0.20	3.084	0.744	5.190	1.907	6	2196	ZINC000008219992
20.8	-10.2	-9.9	0.21	2.149	0.747	8.021	2.668	6	2197	ZINC000014439706
20.8	-11.1	-10.5	0.29	2.660	0.900	5.869	2.309	6	2198	ZINC000100774347
20.8	-10.9	-9.4	0.86	2.718	0.469	8.184	1.342	6	2199	ZINC000100777350
20.8	-10.4	-10.0	0.25	2.239	0.374	7.207	2.810	6	2200	ZINC000001554609
20.7	-10.4	-9.1	0.94	1.836	0.631	5.453	2.024	6	2201	ZINC000150359921

20.7	-10.9	-9.5	0.82	2.285	0.621	5.211	2.288	6	2202	ZINC000100777357
20.7	-10.4	-10.1	0.20	2.667	1.037	6.275	4.121	6	2203	ZINC000004099050
20.7	-11.0	-9.4	0.92	2.743	0.408	8.222	1.355	6	2204	ZINC000100777353
20.7	-9.3	-9.1	0.12	2.356	0.675	7.098	3.556	6	2205	ZINC000100825681
20.7	-11.7	-11.4	0.33	2.065	0.570	4.802	1.386	6	2206	ZINC000150361855
20.7	-10.4	-9.6	0.62	1.778	0.706	4.851	2.136	6	2207	ZINC000085550996
20.7	-11.3	-9.8	1.05	2.349	0.256	6.541	1.439	6	2208	ZINC000257514436
20.7	-10.3	-9.8	0.29	2.148	0.677	5.371	3.601	6	2209	ZINC000014829487
20.7	-11.8	-11.1	0.41	2.574	1.012	5.131	2.703	6	2210	ZINC000100774339
20.6	-11.7	-10.2	0.75	2.241	0.515	4.465	1.531	6	2211	ZINC000257546886
20.6	-10.4	-10.0	0.31	1.770	0.253	6.121	3.087	6	2212	ZINC000257557542
20.6	-10.9	-10.5	0.18	2.895	1.235	5.996	2.815	6	2213	ZINC000252516952
20.6	-10.2	-10.0	0.13	2.434	0.185	6.745	2.518	6	2214	ZINC000100256163
20.6	-10.7	-10.5	0.20	2.789	0.269	8.818	0.293	6	2215	ZINC000100822020
20.6	-11.2	-10.3	0.43	2.012	0.472	5.661	3.179	6	2216	ZINC000257493817

20.6	-11.3	-10.8	0.27	2.474	0.818	5.328	2.800	6	2217	ZINC000100777774
20.6	-11.3	-10.8	0.24	2.627	0.764	6.624	2.472	6	2218	ZINC000100777781
20.5	-11.1	-9.5	0.83	1.685	0.501	5.095	1.819	6	2219	ZINC000150359939
20.5	-11.1	-10.4	0.43	2.724	0.336	8.553	1.667	6	2220	ZINC000257804485
20.5	-11.4	-10.3	0.63	2.087	0.265	4.969	2.035	6	2221	ZINC000257458423
20.5	-10.6	-10.1	0.30	2.599	0.560	7.292	2.411	6	2222	ZINC000257470147
20.5	-9.0	-8.8	0.12	1.840	0.310	4.441	3.071	6	2223	ZINC000100825871
20.5	-11.9	-11.0	0.47	2.835	0.630	7.393	1.759	6	2224	ZINC000257411677
20.5	-9.7	-8.5	0.88	2.187	0.209	6.092	3.666	3	2225	ZINC000230096436
20.5	-11.8	-11.3	0.42	2.612	0.873	7.546	2.880	6	2226	ZINC000257556924
20.5	-10.9	-10.3	0.28	1.926	0.165	6.282	2.543	6	2227	ZINC000257511790
20.4	-10.1	-9.9	0.22	2.146	0.263	4.547	0.918	6	2228	ZINC000257595496
20.4	-11.1	-10.6	0.40	2.358	0.630	4.925	1.967	6	2229	ZINC000096016069
20.4	-9.5	-9.2	0.27	2.701	0.836	9.094	3.050	6	2230	ZINC000257516879
20.4	-8.8	-8.6	0.11	1.551	0.380	6.844	3.754	6	2231	ZINC000257374814

20.4	-9.6	-9.5	0.15	1.935	0.554	5.904	3.791	6	2232	ZINC000257430069
20.4	-10.6	-9.8	0.46	1.654	0.292	3.642	1.230	6	2233	ZINC000100823236
20.4	-11.6	-11.2	0.24	2.450	0.783	4.787	2.119	6	2234	ZINC000257556926
20.4	-11.0	-9.4	0.97	2.751	0.216	8.989	2.018	5	2235	ZINC000085907227
20.4	-11.6	-10.4	0.62	2.502	0.712	6.570	2.203	6	2236	ZINC000085798093
20.4	-10.3	-10.2	0.10	1.873	0.503	6.419	3.055	6	2237	ZINC000100778438
20.4	-10.5	-10.0	0.31	2.029	0.167	6.612	2.153	6	2238	ZINC000100775272
20.4	-9.4	-9.1	0.18	1.493	0.538	5.481	3.972	6	2239	ZINC000257379996
20.4	-12.4	-11.9	0.34	2.687	0.390	7.355	2.497	6	2240	ZINC000100829135
20.4	-11.5	-10.9	0.37	2.490	0.310	6.285	1.701	6	2241	ZINC000100781814
20.4	-12.3	-11.8	0.33	2.128	0.784	6.573	2.786	6	2242	ZINC000044351169
20.4	-10.0	-9.6	0.33	1.826	0.188	4.199	2.128	6	2243	ZINC000257639039
20.3	-9.5	-9.2	0.23	2.914	0.777	9.106	3.057	6	2244	ZINC000257516877
20.3	-11.2	-10.8	0.25	2.920	0.645	6.655	2.213	6	2245	ZINC000257411675
20.3	-9.5	-8.8	0.32	1.819	0.394	2.817	0.392	6	2246	ZINC000014488600

20.3	-9.5	-9.2	0.22	3.118	0.728	10.598	0.105	6	2247	ZINC000257516878
20.3	-10.8	-10.0	0.52	2.391	0.295	3.782	0.688	6	2248	ZINC000100828613
20.3	-9.5	-9.2	0.24	3.021	0.817	9.045	3.023	6	2249	ZINC000002585425
20.3	-10.7	-10.6	0.05	2.837	0.000	7.133	0.000	2	2250	ZINC000086053090
20.3	-11.4	-10.3	0.60	2.072	0.304	5.742	2.663	6	2251	ZINC000100773910
20.3	-10.2	-10.1	0.17	1.977	0.574	7.589	2.655	6	2252	ZINC000100778436
20.3	-9.5	-9.1	0.29	2.572	1.014	6.062	3.634	6	2253	ZINC000014488598
20.3	-9.5	-9.2	0.21	2.710	0.452	6.272	3.548	6	2254	ZINC000004228244
20.3	-10.2	-10.2	0.05	2.051	0.621	6.291	3.160	6	2255	ZINC000100778435
20.3	-9.5	-8.6	0.46	2.430	0.468	5.928	1.192	6	2256	ZINC000257514435
20.3	-9.5	-9.2	0.20	2.546	0.837	6.008	3.626	6	2257	ZINC000014488602
20.3	-9.5	-9.1	0.30	2.761	1.060	7.401	3.923	6	2258	ZINC000014488604
20.3	-10.3	-10.2	0.11	1.870	0.502	6.434	3.029	6	2259	ZINC000100778432
20.3	-11.8	-10.4	0.78	2.701	0.269	6.334	1.152	6	2260	ZINC000100824463
20.2	-11.3	-10.9	0.25	2.427	0.577	4.055	1.906	6	2261	ZINC000014768737

20.2	-11.7	-11.2	0.29	2.835	0.475	5.284	2.758	6	2262	ZINC000100777739
20.2	-10.6	-10.4	0.22	2.119	0.430	3.679	1.009	6	2263	ZINC000100826060
20.2	-10.7	-9.8	0.46	2.217	0.704	7.610	2.518	6	2264	ZINC000257403662
20.2	-11.6	-10.8	0.59	2.141	0.506	5.167	2.118	6	2265	ZINC000150366674
20.2	-11.9	-10.8	0.58	2.024	0.433	4.510	1.232	6	2266	ZINC000150366676
20.2	-9.7	-8.6	0.78	2.445	0.663	5.210	2.727	6	2267	ZINC000086034599
20.2	-11.7	-11.2	0.28	2.985	0.577	4.541	2.195	6	2268	ZINC000100777746
20.2	-12.0	-11.0	0.50	2.085	0.108	6.989	0.783	6	2269	ZINC000150446310
20.2	-10.0	-9.9	0.09	2.037	0.267	7.331	3.350	6	2270	ZINC000005339606
20.2	-9.7	-9.4	0.17	2.039	0.267	3.155	0.346	6	2271	ZINC000059770512
20.2	-11.0	-9.9	0.60	2.656	0.334	7.048	0.619	6	2272	ZINC000042806939
20.2	-11.8	-11.0	0.60	2.333	0.400	4.747	2.600	6	2273	ZINC000200417767
20.2	-12.1	-11.8	0.20	3.424	0.473	7.188	2.153	6	2274	ZINC000014720190
20.1	-10.5	-10.2	0.21	2.064	0.263	4.063	0.566	6	2275	ZINC000257595498
20.1	-11.6	-10.9	0.40	2.735	0.206	6.720	1.836	6	2276	ZINC000100827359

20.1	-10.7	-10.1	0.34	2.204	0.589	4.897	2.237	6	2277	ZINC000085946092
20.1	-10.4	-9.8	0.47	1.673	0.461	4.538	2.798	6	2278	ZINC000100827280
20.1	-11.1	-10.5	0.28	2.743	0.427	5.108	1.796	6	2279	ZINC000013373237
20.1	-10.1	-9.9	0.12	2.016	0.245	5.366	2.185	6	2280	ZINC000100778819
20.1	-10.9	-10.4	0.31	2.318	0.649	6.335	2.481	6	2281	ZINC000118937108
20.1	-11.8	-10.9	0.82	1.814	0.760	4.338	2.338	4	2282	ZINC000086053089
20.1	-11.5	-10.9	0.48	2.025	0.364	5.009	2.843	6	2283	ZINC000230066692
20.1	-7.8	-7.3	0.42	2.322	0.190	6.777	0.592	6	2284	ZINC000100828689
20.0	-11.0	-10.4	0.37	1.806	0.102	7.483	1.378	6	2285	ZINC000100772197
20.0	-8.6	-7.0	1.15	2.542	0.495	6.767	0.461	6	2286	ZINC000100774812
20.0	-11.0	-9.6	0.69	2.297	0.120	6.251	1.212	6	2287	ZINC000257455689
20.0	-11.4	-10.6	0.55	2.480	0.304	4.463	1.646	6	2288	ZINC000257444396
20.0	-12.1	-11.4	0.40	2.199	0.329	5.161	2.247	6	2289	ZINC000257410670
20.0	-9.9	-9.3	0.49	2.356	0.154	4.485	0.839	6	2290	ZINC000256119451
20.0	-10.9	-10.0	0.52	2.488	0.333	4.318	0.869	6	2291	ZINC000253527788

20.0	-8.8	-8.5	0.21	2.111	0.783	4.505	3.176	6	2292	ZINC000085859014
20.0	-9.9	-8.6	0.82	2.334	0.215	6.827	1.984	6	2293	ZINC000257452353
20.0	-10.0	-9.7	0.22	1.908	0.652	5.231	2.713	6	2294	ZINC000150369274
20.0	-10.2	-9.6	0.31	1.970	0.355	7.159	2.618	6	2295	ZINC000257429746
20.0	-10.5	-10.0	0.36	2.159	0.654	7.308	3.723	6	2296	ZINC000015104406
20.0	-10.1	-9.6	0.38	1.737	0.205	8.731	2.496	6	2297	ZINC000095620838
20.0	-10.6	-10.0	0.32	2.334	0.480	7.334	2.381	6	2298	ZINC000248332069
20.0	-9.9	-8.9	0.53	2.492	0.452	6.982	2.621	6	2299	ZINC000100828609
19.9	-11.0	-10.0	0.49	2.138	0.734	5.942	3.276	6	2300	ZINC000085991639
19.9	-10.5	-10.2	0.20	3.063	0.760	7.213	1.882	6	2301	ZINC000100778646
19.9	-11.4	-10.4	0.52	2.521	0.580	5.632	2.501	6	2302	ZINC000100829137
19.9	-11.0	-9.6	1.11	1.992	0.242	4.802	0.759	3	2303	ZINC000257535735
19.9	-12.3	-11.7	0.44	2.701	0.231	6.414	2.311	6	2304	ZINC000257615336
19.8	-10.7	-10.3	0.22	1.985	0.548	5.601	2.010	6	2305	ZINC000095619500
19.8	-10.4	-10.1	0.21	1.874	0.193	5.367	2.496	6	2306	ZINC000100777954

19.8	-10.8	-10.1	0.57	2.026	0.339	3.622	0.798	6	2307	ZINC000150367788
19.8	-10.5	-10.2	0.20	2.557	0.287	7.462	1.638	6	2308	ZINC000100777810
19.8	-9.2	-9.0	0.11	1.848	0.637	5.357	3.310	6	2309	ZINC000100825684
19.8	-11.4	-10.7	0.41	2.187	0.617	4.411	2.719	6	2310	ZINC000118936833
19.8	-9.3	-9.2	0.08	2.311	0.532	6.736	2.831	6	2311	ZINC000257406752
19.8	-11.3	-10.7	0.32	2.293	0.425	7.278	3.501	6	2312	ZINC000085826837
19.7	-11.7	-11.0	0.56	1.628	0.678	5.173	3.201	6	2313	ZINC000257488656
19.7	-10.6	-10.2	0.18	1.951	0.302	6.492	2.109	6	2314	ZINC000257404830
19.7	-11.1	-10.0	0.62	1.960	0.593	4.731	2.610	6	2315	ZINC000085567739
19.7	-11.4	-11.0	0.30	2.029	0.534	2.999	0.624	6	2316	ZINC000257384131
19.7	-9.7	-9.4	0.27	2.491	0.357	6.624	3.287	6	2317	ZINC000014488605
19.7	-11.7	-11.3	0.38	1.745	0.330	5.029	3.303	6	2318	ZINC000024215056
19.7	-11.1	-10.9	0.18	2.758	1.046	5.875	2.687	6	2319	ZINC000100781599
19.7	-11.6	-11.1	0.24	2.646	0.563	6.735	2.126	6	2320	ZINC000095619511
19.7	-10.2	-9.9	0.17	1.884	0.543	6.124	4.070	6	2321	ZINC000410428652

19.6	-10.6	-9.8	0.45	2.044	0.484	4.697	2.570	6	2322	ZINC000257530992
19.6	-11.4	-10.8	0.34	2.191	0.803	4.358	2.791	6	2323	ZINC000118936831
19.6	-10.3	-9.8	0.34	2.201	0.652	2.901	0.726	6	2324	ZINC000085955997
19.6	-10.9	-10.1	0.36	1.865	0.402	6.142	2.235	6	2325	ZINC000248328654
19.6	-9.1	-8.6	0.23	2.022	0.364	7.809	3.529	6	2326	ZINC000031474771
19.6	-9.8	-9.4	0.20	1.475	0.585	2.609	0.548	6	2327	ZINC000015058148
19.6	-11.2	-11.0	0.18	1.848	0.193	3.367	0.660	6	2328	ZINC000257434587
19.6	-10.2	-9.3	0.49	2.465	0.206	6.474	1.718	6	2329	ZINC000100823805
19.6	-11.2	-10.7	0.38	2.075	1.147	3.702	1.529	6	2330	ZINC000014829484
19.6	-10.7	-10.1	0.36	1.961	0.325	6.152	3.196	6	2331	ZINC000257386808
19.6	-9.5	-9.5	0.00	0.000	0.000	0.000	0.000	1	2332	ZINC000100773119
19.6	-10.5	-10.3	0.11	2.133	0.282	4.234	0.682	6	2333	ZINC000257595499
19.6	-11.4	-10.3	0.66	2.808	0.714	7.041	3.032	6	2334	ZINC000257384133
19.6	-10.8	-9.8	0.51	2.724	0.385	6.643	2.098	6	2335	ZINC000257376544
19.6	-10.6	-10.2	0.29	1.929	0.222	7.196	1.670	6	2336	ZINC000014617843

19.6	-10.8	-10.0	0.42	2.589	0.198	4.355	0.558	6	2337	ZINC000256119456
19.6	-11.2	-10.7	0.24	2.503	1.012	5.855	3.035	6	2338	ZINC000256601998
19.6	-11.4	-10.8	0.37	2.036	0.856	4.165	2.884	6	2339	ZINC000257384132
19.6	-11.2	-10.0	0.76	2.219	0.529	6.595	2.254	6	2340	ZINC000095912680
19.6	-10.7	-10.7	0.08	2.030	0.487	6.818	3.623	6	2341	ZINC000014765648
19.6	-10.8	-10.3	0.27	2.001	0.479	6.312	2.636	6	2342	ZINC000257567965
19.6	-11.6	-11.1	0.24	2.679	0.627	5.396	2.527	6	2343	ZINC000095619513
19.6	-11.3	-10.8	0.38	2.110	0.804	4.218	2.848	6	2344	ZINC000257384134
19.6	-9.8	-9.3	0.25	2.272	0.432	6.300	3.501	6	2345	ZINC000014645015
19.5	-11.3	-11.0	0.20	1.869	0.371	2.709	0.506	6	2346	ZINC000118936830
19.5	-12.7	-11.9	0.42	2.588	0.404	5.803	2.257	6	2347	ZINC000257466111
19.5	-11.3	-10.3	0.52	2.574	0.763	6.588	3.167	6	2348	ZINC000118936832
19.5	-10.0	-8.7	0.70	1.945	0.472	4.367	3.061	6	2349	ZINC000238776308
19.5	-12.7	-12.2	0.34	2.676	1.068	6.136	2.835	6	2350	ZINC000118937425
19.5	-10.9	-10.2	0.49	2.041	0.351	4.794	2.125	6	2351	ZINC000095617641

19.5	-12.7	-12.0	0.34	2.627	0.388	5.924	2.085	6	2352	ZINC000257466110
19.5	-10.9	-10.7	0.17	1.705	0.153	7.425	1.963	6	2353	ZINC000257380439
19.5	-10.5	-10.2	0.26	2.109	1.410	6.628	2.266	6	2354	ZINC000085550541
19.5	-10.6	-10.2	0.22	1.978	0.185	7.648	0.122	6	2355	ZINC000257808144
19.5	-12.7	-11.9	0.44	2.661	0.443	4.959	1.807	6	2356	ZINC000118914173
19.5	-9.0	-8.7	0.16	2.674	0.526	7.189	2.767	6	2357	ZINC000257563725
19.5	-12.0	-11.4	0.32	3.062	0.720	7.287	2.362	6	2358	ZINC000100780297
19.5	-12.7	-11.9	0.38	2.897	0.779	5.731	2.175	6	2359	ZINC000118937426
19.5	-9.5	-9.1	0.29	2.016	0.527	5.735	3.804	6	2360	ZINC000014488607
19.5	-12.7	-11.9	0.37	2.505	0.420	4.904	1.823	6	2361	ZINC000257466108
19.5	-7.7	-7.0	0.83	3.169	0.499	7.197	1.315	3	2362	ZINC000028115899
19.5	-12.7	-12.2	0.36	2.293	0.727	5.303	2.699	6	2363	ZINC000257466109
19.5	-10.4	-10.2	0.22	1.646	0.114	6.333	2.824	6	2364	ZINC000014495575
19.4	-11.8	-11.4	0.30	2.109	0.767	4.632	2.314	6	2365	ZINC000100782766
19.4	-11.2	-10.4	0.39	2.273	0.192	4.144	0.391	6	2366	ZINC000257458422

19.4	-11.0	-10.8	0.11	2.121	0.371	3.737	0.793	6	2367	ZINC000257395113
19.4	-10.9	-10.6	0.24	2.528	0.352	6.018	2.203	6	2368	ZINC000100822015
19.4	-11.6	-10.8	0.65	1.669	0.390	5.462	3.818	6	2369	ZINC000257452355
19.4	-11.8	-11.5	0.29	2.056	0.781	4.595	2.357	6	2370	ZINC000257449821
19.4	-11.8	-11.5	0.23	2.186	0.771	5.062	1.963	6	2371	ZINC000100782764
19.4	-12.7	-12.0	0.36	2.776	0.802	5.636	2.227	6	2372	ZINC000118937424
19.4	-10.6	-9.8	0.52	1.992	0.452	4.694	2.593	6	2373	ZINC000100772375
19.4	-11.1	-9.4	0.99	2.213	0.397	3.387	1.160	5	2374	ZINC000085907217
19.4	-11.8	-11.5	0.27	2.154	0.778	5.052	1.968	6	2375	ZINC000100782760
19.4	-5.8	-4.3	1.10	2.564	0.643	6.388	1.845	6	2376	ZINC000013383666
19.4	-9.9	-9.2	0.56	1.827	0.272	5.189	2.390	6	2377	ZINC000257620361
19.4	-11.8	-11.4	0.28	2.104	0.792	4.710	2.316	6	2378	ZINC000257449819
19.4	-11.8	-11.3	0.41	2.298	0.840	6.766	2.180	6	2379	ZINC000257449818
19.4	-10.6	-9.7	0.50	1.886	0.174	5.595	3.629	6	2380	ZINC000100829094
19.4	-10.9	-10.7	0.16	2.010	0.270	5.933	2.490	6	2381	ZINC000100782164

19.4	-11.8	-11.5	0.29	2.166	0.775	5.062	1.959	6	2382	ZINC000257449820
19.4	-11.6	-10.0	0.96	2.364	0.232	7.993	0.561	4	2383	ZINC000100783894
19.4	-9.3	-9.0	0.14	2.622	0.606	7.107	2.832	6	2384	ZINC000013435205
19.4	-11.1	-10.3	0.40	2.203	0.490	6.143	2.419	6	2385	ZINC000150369283
19.4	-10.8	-9.7	0.73	2.062	0.383	4.521	2.893	6	2386	ZINC000253527787
19.4	-11.8	-11.5	0.26	2.166	0.774	5.060	1.962	6	2387	ZINC000100782762
19.4	-10.6	-9.7	0.52	1.582	0.477	5.398	3.779	6	2388	ZINC000257476479
19.3	-12.2	-11.6	0.31	2.967	0.349	6.306	2.100	6	2389	ZINC000257535078
19.3	-11.8	-11.0	0.57	1.776	0.175	7.084	1.507	6	2390	ZINC000257465217
19.3	-9.2	-8.8	0.20	2.807	0.478	7.197	2.823	6	2391	ZINC000257563726
19.3	-11.1	-10.3	0.39	1.857	0.592	5.176	2.879	6	2392	ZINC000257442037
19.3	-11.1	-10.3	0.39	2.050	0.469	5.659	1.648	6	2393	ZINC000150369278
19.3	-12.2	-11.6	0.29	2.709	0.469	4.316	1.912	6	2394	ZINC000257535076
19.3	-10.7	-10.5	0.15	1.927	0.255	6.620	1.834	6	2395	ZINC000254697461
19.3	-11.1	-10.3	0.37	2.524	0.603	7.760	1.456	6	2396	ZINC000257470150

19.3	-12.3	-10.8	1.02	2.619	0.288	6.899	1.107	5	2397	ZINC000100783815
19.3	-9.5	-9.0	0.31	2.267	0.832	8.615	3.248	6	2398	ZINC000100828602
19.3	-10.2	-9.6	0.33	2.017	0.413	6.975	2.484	6	2399	ZINC000257429747
19.3	-10.6	-9.7	0.60	2.215	0.252	6.097	3.153	6	2400	ZINC000100772377
19.3	-10.6	-9.5	0.54	1.787	0.353	5.491	3.734	6	2401	ZINC000257476480
19.3	-13.1	-11.6	0.76	2.078	0.598	4.283	2.534	6	2402	ZINC000100781859
19.3	-11.1	-10.3	0.39	2.042	0.469	5.664	1.652	6	2403	ZINC000150369281
19.3	-10.9	-10.4	0.29	2.544	0.685	6.511	3.146	6	2404	ZINC000230104301
19.3	-10.1	-9.8	0.24	1.951	0.138	5.506	2.412	6	2405	ZINC000257639037
19.3	-10.8	-10.4	0.20	1.842	0.299	7.011	2.221	6	2406	ZINC000253477225
19.2	-8.7	-8.4	0.19	1.966	0.801	5.560	3.782	6	2407	ZINC000014435236
19.2	-12.1	-11.6	0.26	2.885	0.491	5.162	2.458	6	2408	ZINC000100776968
19.2	-10.3	-9.3	0.59	2.118	0.165	7.703	3.942	6	2409	ZINC000257525941
19.2	-11.2	-10.8	0.38	1.980	0.420	6.550	2.877	6	2410	ZINC000100829931
19.2	-12.1	-11.7	0.22	2.653	0.534	4.100	2.002	6	2411	ZINC000085847606

19.2	-11.8	-10.9	0.46	2.239	0.182	7.600	0.935	6	2412	ZINC000257435965
19.2	-10.5	-9.5	0.51	2.992	0.333	6.072	2.093	6	2413	ZINC000100358741
19.2	-10.7	-10.3	0.24	2.241	0.628	7.064	1.983	6	2414	ZINC000014495572
19.2	-11.5	-10.7	0.38	1.776	0.309	3.687	2.421	6	2415	ZINC000257621916
19.2	-12.1	-11.6	0.29	2.961	0.373	6.303	2.103	6	2416	ZINC000100776970
19.2	-10.1	-9.6	0.27	1.921	0.199	7.300	3.609	6	2417	ZINC000014489963
19.2	-10.2	-9.7	0.31	1.755	0.285	5.337	1.929	6	2418	ZINC000257399448
19.2	-12.1	-11.6	0.27	2.968	0.303	5.631	2.236	6	2419	ZINC000100776966
19.2	-12.1	-11.6	0.26	2.894	0.494	5.165	2.456	6	2420	ZINC000257535077
19.2	-11.4	-10.5	0.51	1.528	0.149	2.437	0.639	6	2421	ZINC000257409647
19.1	-9.2	-8.7	0.25	3.303	1.328	7.604	3.859	6	2422	ZINC000014811584
19.1	-10.6	-10.1	0.25	2.945	0.336	5.607	1.850	6	2423	ZINC000100777958
19.1	-10.6	-9.7	0.51	2.133	0.330	4.779	2.528	6	2424	ZINC000100772379
19.1	-9.8	-9.5	0.20	2.665	0.635	7.834	3.278	6	2425	ZINC000257501323
19.1	-11.6	-10.5	0.78	2.541	0.082	8.143	1.758	6	2426	ZINC000257481896

19.1	-10.3	-8.7	0.87	2.278	0.580	6.353	2.268	6	2427	ZINC000150351800
19.1	-9.5	-9.3	0.12	2.142	0.392	5.735	3.444	6	2428	ZINC000257374815
19.1	-8.8	-8.6	0.13	1.666	0.784	2.781	1.384	6	2429	ZINC000014435232
19.1	-11.2	-11.0	0.23	1.752	0.187	5.258	2.434	6	2430	ZINC000257380436
19.1	-11.0	-10.7	0.20	1.954	0.207	5.366	2.196	6	2431	ZINC000257511789
19.1	-11.5	-11.0	0.35	2.041	0.849	4.964	2.786	6	2432	ZINC000014720202
19.1	-12.1	-11.6	0.26	2.712	0.477	4.312	1.919	6	2433	ZINC000100776973
19.0	-10.7	-10.2	0.41	2.648	0.854	6.324	2.661	6	2434	ZINC000257607428
19.0	-9.6	-9.4	0.15	1.851	0.563	5.221	3.149	6	2435	ZINC000015260671
19.0	-11.3	-10.0	0.61	2.383	0.443	7.108	0.741	6	2436	ZINC000038139485
19.0	-11.4	-10.5	0.65	2.301	0.144	8.667	2.274	6	2437	ZINC000100783653
19.0	-11.7	-11.1	0.40	2.259	0.530	6.823	2.642	6	2438	ZINC000257548479
19.0	-9.2	-8.7	0.24	1.920	0.802	4.170	3.158	6	2439	ZINC000014811586
19.0	-8.8	-8.5	0.17	1.742	0.741	3.986	3.185	6	2440	ZINC000100829041
19.0	-11.1	-10.7	0.22	2.229	0.647	5.169	1.899	6	2441	ZINC000100773913

18.9	-11.7	-11.0	0.34	2.495	0.421	7.438	1.427	6	2442	ZINC000257548482
18.9	-9.2	-8.7	0.32	1.444	0.752	3.673	3.250	6	2443	ZINC000014658124
18.9	-11.7	-11.2	0.34	2.287	0.496	6.187	2.639	6	2444	ZINC000118937501
18.9	-11.7	-11.2	0.34	2.318	0.519	6.187	2.642	6	2445	ZINC000257548480
18.9	-10.3	-9.8	0.29	2.629	0.596	3.797	0.842	6	2446	ZINC000085956000
18.9	-10.0	-9.8	0.12	1.796	0.171	8.670	0.060	6	2447	ZINC000100777897
18.9	-10.6	-10.1	0.41	2.337	0.456	3.877	1.055	6	2448	ZINC000242543351
18.9	-11.7	-11.1	0.44	2.059	0.588	4.980	3.007	6	2449	ZINC000118937502
18.9	-10.5	-10.3	0.15	3.212	1.052	6.563	2.582	6	2450	ZINC000100778653
18.9	-10.2	-10.0	0.23	2.460	0.317	7.399	1.483	6	2451	ZINC000100256150
18.9	-9.7	-9.5	0.09	1.774	0.598	5.673	3.869	6	2452	ZINC000014489961
18.9	-11.2	-10.2	0.58	2.395	0.129	6.985	1.243	6	2453	ZINC000257546883
18.9	-10.1	-9.8	0.19	1.645	0.193	3.004	0.668	6	2454	ZINC000257624274
18.9	-11.7	-11.1	0.36	2.243	0.469	5.781	2.977	6	2455	ZINC000118937500
18.9	-10.9	-10.4	0.35	2.450	0.378	6.378	3.295	6	2456	ZINC000100824470

18.9	-9.6	-9.3	0.17	2.001	0.757	5.735	3.914	6	2457	ZINC000100828777
18.9	-12.5	-12.1	0.32	2.686	0.632	5.237	2.684	6	2458	ZINC000095620014
18.9	-9.8	-9.5	0.17	2.260	0.227	8.393	2.858	6	2459	ZINC000013775871
18.9	-12.5	-12.2	0.24	2.817	0.682	4.266	2.034	6	2460	ZINC000100782748
18.9	-10.9	-10.2	0.52	2.417	0.818	5.842	2.803	6	2461	ZINC000100825512
18.9	-10.8	-10.3	0.34	2.252	0.519	5.406	2.218	6	2462	ZINC000257488042
18.9	-11.0	-10.7	0.34	1.672	0.248	5.903	2.815	6	2463	ZINC000257511792
18.9	-12.5	-12.2	0.26	2.894	0.747	5.054	2.636	6	2464	ZINC000100782749
18.9	-12.5	-12.1	0.30	2.946	0.441	6.352	2.289	6	2465	ZINC000073334979
18.9	-11.7	-11.2	0.34	2.286	0.495	6.189	2.645	6	2466	ZINC000118937499
18.9	-10.9	-10.6	0.13	2.298	0.409	6.172	1.968	6	2467	ZINC000095619135
18.9	-12.4	-10.9	0.73	2.256	0.201	6.359	2.529	6	2468	ZINC000150366679
18.9	-10.5	-10.1	0.19	1.854	0.262	5.662	2.277	6	2469	ZINC000257455711
18.8	-10.1	-9.8	0.28	2.274	0.241	7.943	1.390	6	2470	ZINC000100822021
18.8	-11.2	-10.5	0.37	2.624	0.638	5.974	2.267	6	2471	ZINC000150351595

18.8	-11.6	-11.3	0.22	2.684	0.586	5.253	2.530	6	2472	ZINC000257565590
18.8	-11.6	-11.2	0.31	2.994	0.350	6.276	2.048	6	2473	ZINC000257565588
18.8	-11.6	-11.3	0.18	2.301	0.666	5.297	2.267	6	2474	ZINC000257565589
18.8	-10.6	-10.3	0.17	2.246	0.240	7.460	1.941	6	2475	ZINC000257577148
18.8	-11.6	-11.3	0.18	2.299	0.664	5.296	2.268	6	2476	ZINC000257388026
18.8	-11.7	-11.1	0.40	2.295	0.497	6.853	2.586	6	2477	ZINC000257548481
18.8	-11.6	-11.3	0.23	2.926	0.473	6.336	2.149	6	2478	ZINC000100824058
18.8	-10.4	-10.1	0.23	1.515	0.296	4.867	3.334	6	2479	ZINC000014495578
18.8	-10.8	-10.2	0.46	2.625	0.914	5.496	2.583	6	2480	ZINC000100825519
18.8	-11.6	-11.4	0.14	2.385	0.206	5.382	0.337	6	2481	ZINC000001531664
18.8	-11.6	-11.3	0.18	2.088	0.614	4.892	2.636	6	2482	ZINC000257388025
18.8	-11.0	-10.1	0.58	2.217	0.446	5.942	2.274	6	2483	ZINC000100825762
18.8	-11.6	-11.3	0.18	2.247	0.639	5.290	2.276	6	2484	ZINC000100779246
18.8	-11.6	-11.3	0.16	2.293	0.659	5.288	2.275	6	2485	ZINC000100779244
18.8	-11.6	-11.3	0.20	2.928	0.503	5.158	2.417	6	2486	ZINC000257565591

18.8	-10.4	-10.0	0.29	2.189	0.281	6.980	1.280	6	2487	ZINC000257621033
18.8	-11.6	-11.3	0.17	2.293	0.658	5.285	2.277	6	2488	ZINC000100824061
18.8	-9.1	-8.8	0.17	2.675	0.547	5.659	2.626	6	2489	ZINC000015260668
18.7	-11.5	-11.2	0.21	2.422	0.641	3.248	0.794	6	2490	ZINC000100779241
18.7	-9.4	-9.2	0.16	2.424	0.726	6.962	2.941	6	2491	ZINC000015260677
18.7	-10.7	-9.8	0.60	1.867	0.514	4.373	3.030	6	2492	ZINC000257525938
18.7	-11.5	-11.3	0.14	2.949	0.434	5.306	2.262	6	2493	ZINC000100824060
18.7	-10.5	-10.1	0.20	2.330	0.794	5.689	2.744	6	2494	ZINC000118937028
18.7	-10.6	-9.5	0.60	1.917	0.176	7.206	3.556	6	2495	ZINC000257476482
18.7	-9.3	-8.6	0.33	1.756	0.539	2.825	0.544	6	2496	ZINC000257374817
18.7	-11.5	-11.3	0.14	2.950	0.428	5.305	2.260	6	2497	ZINC000100824059
18.7	-10.6	-10.1	0.35	2.240	0.261	6.028	3.739	6	2498	ZINC000100826074
18.7	-11.5	-11.2	0.19	2.600	0.720	7.061	1.783	6	2499	ZINC000100781815
18.7	-8.9	-8.6	0.16	2.868	0.312	8.347	2.102	6	2500	ZINC000085946106
18.7	-9.8	-9.5	0.14	1.776	0.280	5.975	2.198	6	2501	ZINC000257443454

18.7	-11.6	-10.6	0.68	2.584	0.680	5.449	1.707	6	2502	ZINC000257478361
18.7	-9.4	-8.9	0.30	2.019	0.823	6.811	2.708	6	2503	ZINC000003775575
18.7	-13.4	-12.6	0.65	3.083	0.601	5.856	1.335	6	2504	ZINC000100780019
18.7	-11.3	-11.0	0.19	2.270	0.290	6.184	2.407	6	2505	ZINC000100824462
18.7	-11.6	-11.0	0.36	2.646	0.336	6.049	1.663	6	2506	ZINC000100827365
18.7	-10.3	-9.4	0.46	2.621	0.771	6.746	3.363	6	2507	ZINC000257384658
18.7	-10.3	-9.9	0.26	1.931	0.489	5.591	3.351	6	2508	ZINC000001630829
18.7	-11.5	-11.3	0.16	2.887	0.551	5.137	2.458	6	2509	ZINC000257388024
18.7	-11.6	-11.0	0.34	2.697	0.233	5.635	1.714	6	2510	ZINC000100827363
18.7	-10.4	-9.5	0.63	2.634	0.125	6.221	0.827	6	2511	ZINC000150360868
18.7	-13.4	-12.6	0.65	3.081	0.599	5.860	1.334	6	2512	ZINC000100780022
18.6	-11.5	-11.3	0.16	2.949	0.430	5.296	2.261	6	2513	ZINC000257388027
18.6	-11.7	-9.9	1.01	2.340	0.460	6.458	2.180	6	2514	ZINC000136692919
18.6	-11.7	-10.6	0.63	2.097	0.524	4.728	2.904	6	2515	ZINC000257510214
18.6	-10.0	-9.7	0.22	1.996	0.344	5.693	2.271	6	2516	ZINC000100822013

18.6	-11.5	-11.1	0.28	2.139	0.537	6.345	2.151	6	2517	ZINC000100779243
18.6	-10.6	-9.8	0.52	2.162	0.352	4.792	2.512	6	2518	ZINC000100772372
18.6	-11.3	-10.9	0.20	2.324	0.380	6.365	2.230	6	2519	ZINC000100778888
18.6	-10.4	-9.8	0.37	2.130	0.063	7.014	3.151	6	2520	ZINC000257442036
18.6	-12.2	-11.5	0.40	2.106	0.236	5.178	1.821	6	2521	ZINC000014513020
18.6	-10.7	-10.1	0.37	2.497	0.205	7.319	2.135	6	2522	ZINC000059728593
18.6	-9.9	-9.2	0.49	2.129	0.623	6.183	2.917	6	2523	ZINC000150367793
18.6	-10.4	-10.0	0.21	2.192	0.318	5.604	2.915	6	2524	ZINC000257442038
18.5	-9.8	-9.3	0.24	1.813	0.211	5.777	3.232	6	2525	ZINC000238741755
18.5	-10.8	-9.9	0.51	1.904	0.273	5.890	1.909	6	2526	ZINC000257421471
18.5	-9.9	-9.8	0.13	1.588	0.170	8.082	0.168	6	2527	ZINC000100777735
18.5	-10.7	-10.0	0.44	2.143	0.346	5.953	3.736	6	2528	ZINC000100826073
18.5	-10.2	-9.8	0.30	2.787	0.374	8.806	0.577	6	2529	ZINC000150352529
18.5	-11.0	-10.4	0.32	2.690	0.259	6.031	1.393	6	2530	ZINC000257504418
18.5	-8.8	-8.3	0.33	2.331	0.297	5.895	3.801	6	2531	ZINC000014488269

18.5	-11.8	-10.5	0.77	2.243	0.336	6.909	2.098	6	2532	ZINC000257546884
18.5	-11.0	-10.8	0.21	2.308	0.642	6.368	2.699	6	2533	ZINC000013373230
18.5	-9.4	-9.3	0.07	2.578	0.556	7.230	2.432	6	2534	ZINC000008552019
18.5	-13.2	-12.2	0.76	3.037	0.623	6.761	2.021	6	2535	ZINC000100780026
18.5	-10.7	-10.3	0.21	1.729	0.343	5.610	2.732	6	2536	ZINC000014619924
18.5	-10.6	-9.8	0.53	2.454	0.405	6.057	3.484	6	2537	ZINC000257530993
18.5	-10.1	-9.6	0.24	1.687	0.166	5.972	3.540	6	2538	ZINC000095620783
18.5	-10.2	-9.8	0.27	2.192	0.170	5.062	2.196	6	2539	ZINC000257387714
18.5	-9.1	-8.9	0.09	1.865	0.804	5.447	3.458	6	2540	ZINC000257374816
18.5	-10.3	-9.5	0.55	2.037	0.754	3.951	2.793	6	2541	ZINC000100825639
18.5	-10.3	-9.7	0.38	1.989	0.901	5.265	3.568	6	2542	ZINC000257384659
18.5	-9.6	-9.2	0.38	1.870	0.454	4.591	2.541	6	2543	ZINC000059732394
18.5	-10.8	-10.4	0.41	2.467	0.786	7.372	2.624	6	2544	ZINC000257376546
18.5	-11.0	-10.4	0.31	2.428	0.398	6.174	1.211	6	2545	ZINC000257504415
18.5	-11.0	-10.3	0.36	2.174	0.562	5.000	2.177	6	2546	ZINC000257504416

18.5	-11.0	-10.7	0.18	1.883	0.297	5.599	2.425	6	2547	ZINC000257494289
18.5	-13.2	-12.4	0.61	3.138	0.625	5.971	1.338	6	2548	ZINC000100780023
18.4	-10.7	-10.0	0.30	2.373	0.589	5.529	2.329	6	2549	ZINC000095619499
18.4	-9.8	-9.3	0.29	2.518	0.465	6.053	0.284	6	2550	ZINC000100827899
18.4	-11.5	-11.2	0.31	2.881	0.612	7.044	2.520	6	2551	ZINC000095619575
18.4	-11.0	-10.5	0.34	2.719	0.219	7.985	1.423	6	2552	ZINC000257376545
18.4	-11.5	-11.0	0.24	1.989	0.750	3.881	2.316	6	2553	ZINC000257409648
18.4	-10.8	-10.1	0.45	2.831	0.171	6.381	1.679	6	2554	ZINC000100778815
18.4	-10.9	-10.5	0.37	2.334	0.264	5.909	2.405	6	2555	ZINC000257515952
18.4	-10.9	-10.4	0.31	2.637	0.843	6.124	2.854	6	2556	ZINC000252516953
18.4	-11.0	-10.5	0.28	1.803	0.595	4.163	2.417	6	2557	ZINC000257504417
18.4	-11.0	-10.5	0.46	1.761	0.955	5.801	3.912	6	2558	ZINC000100826636
18.4	-10.5	-9.8	0.39	1.604	0.513	5.376	3.802	6	2559	ZINC000257476481
18.4	-9.6	-9.2	0.24	1.272	0.335	2.033	0.575	6	2560	ZINC000257498515
18.4	-11.3	-10.3	0.55	2.677	0.920	4.990	1.780	6	2561	ZINC000256005424

18.4	-11.7	-10.5	1.05	1.905	0.760	5.062	2.849	6	2562	ZINC000257573395
18.4	-10.4	-10.1	0.20	2.124	0.231	5.912	2.693	6	2563	ZINC000257442039
18.3	-10.1	-9.5	0.41	1.912	0.926	5.213	3.693	6	2564	ZINC000100825640
18.3	-11.3	-10.7	0.42	3.086	0.785	7.352	1.932	6	2565	ZINC000014720203
18.3	-10.1	-9.7	0.45	1.749	0.665	6.464	3.360	6	2566	ZINC000257496149
18.3	-10.4	-9.9	0.30	1.622	0.137	7.584	3.773	6	2567	ZINC000257525939
18.3	-10.6	-10.3	0.17	2.275	0.217	5.883	2.290	6	2568	ZINC000100777599
18.3	-9.5	-9.0	0.22	2.259	0.551	7.574	2.724	6	2569	ZINC000257563727
18.3	-9.6	-9.2	0.19	2.433	0.593	5.177	2.935	6	2570	ZINC000014960821
18.3	-9.6	-9.4	0.14	2.455	0.590	7.361	2.556	6	2571	ZINC000257533755
18.2	-8.9	-8.5	0.28	1.931	0.393	6.060	3.534	6	2572	ZINC000014488275
18.2	-9.5	-9.0	0.50	1.695	0.561	4.244	2.762	6	2573	ZINC000100829097
18.2	-10.1	-9.4	0.43	1.910	0.939	5.247	3.648	6	2574	ZINC000257384657
18.2	-10.5	-10.0	0.54	2.116	0.193	6.784	0.674	6	2575	ZINC000257675762
18.2	-10.7	-10.2	0.32	1.758	0.561	4.182	3.032	6	2576	ZINC000100826637

18.2	-10.6	-10.4	0.19	1.974	0.300	3.012	0.492	6	2577	ZINC000118937194
18.2	-8.2	-7.8	0.27	3.417	0.977	7.848	3.709	6	2578	ZINC000014657880
18.2	-10.7	-9.9	0.59	2.047	0.195	3.210	0.504	6	2579	ZINC000257525940
18.2	-8.8	-8.8	0.00	0.000	0.000	0.000	0.000	1	2580	ZINC000100774809
18.2	-10.2	-9.6	0.49	2.046	0.156	6.551	2.243	6	2581	ZINC000085863883
18.1	-10.5	-10.4	0.07	1.933	0.532	5.082	2.274	6	2582	ZINC000014720437
18.1	-9.8	-9.6	0.17	2.646	0.488	6.649	2.346	6	2583	ZINC000100822019
18.1	-11.1	-10.6	0.43	2.431	0.292	4.806	0.737	6	2584	ZINC000257600997
18.1	-11.5	-11.2	0.15	2.187	0.634	5.902	2.528	6	2585	ZINC000100778891
18.1	-10.9	-10.2	0.48	2.636	0.790	6.375	2.750	6	2586	ZINC000257607425
18.1	-7.8	-6.0	0.82	2.125	0.160	5.265	1.048	6	2587	ZINC000100828692
18.1	-9.3	-9.0	0.24	2.037	0.403	4.107	3.230	6	2588	ZINC000257501324
18.1	-13.4	-13.0	0.30	1.045	0.949	6.275	0.533	6	2589	ZINC000085974153
18.1	-11.1	-10.8	0.13	2.245	0.320	5.887	2.238	6	2590	ZINC000014720433
18.1	-10.5	-10.0	0.44	2.111	0.089	6.811	3.186	6	2591	ZINC000038852739

18.1	-11.2	-10.8	0.25	2.161	0.538	5.810	2.301	6	2592	ZINC000257402588
18.1	-10.6	-10.2	0.32	2.468	0.786	4.755	2.597	6	2593	ZINC000118937193
18.0	-9.3	-7.8	0.85	3.495	0.923	6.938	1.042	4	2594	ZINC000100774817
18.0	-11.0	-10.6	0.21	2.674	0.728	7.178	2.718	6	2595	ZINC000257556923
18.0	-9.0	-8.6	0.18	2.151	0.638	5.712	2.840	6	2596	ZINC000100827308
18.0	-11.1	-10.5	0.39	2.243	0.350	3.626	0.568	6	2597	ZINC000257505904
18.0	-9.8	-9.3	0.31	2.416	0.609	6.740	3.464	6	2598	ZINC000257384656
18.0	-11.7	-10.8	0.46	2.126	0.179	7.185	2.706	6	2599	ZINC000085954796
18.0	-9.9	-9.4	0.33	2.450	0.377	4.144	1.011	6	2600	ZINC000100774089
18.0	-10.4	-10.2	0.22	1.794	0.418	5.869	3.386	6	2601	ZINC000100776720
18.0	-8.9	-8.7	0.15	2.007	0.330	9.764	0.213	6	2602	ZINC000100825682
18.0	-8.2	-8.0	0.11	1.317	0.439	2.603	0.641	6	2603	ZINC000100822172
18.0	-11.3	-10.2	0.83	2.506	0.939	7.182	1.031	6	2604	ZINC000100828147
18.0	-10.6	-9.5	0.60	1.912	0.178	7.177	3.586	6	2605	ZINC000100829099
18.0	-11.2	-10.9	0.22	2.654	0.748	6.111	1.728	6	2606	ZINC000013373236

18.0	-11.0	-10.7	0.22	2.069	0.484	5.474	2.659	6	2607	ZINC000014858847
18.0	-9.2	-8.9	0.15	1.914	0.595	5.302	3.131	6	2608	ZINC000085946001
17.9	-11.0	-10.5	0.26	1.975	0.532	4.341	1.290	6	2609	ZINC000253497558
17.9	-10.6	-10.3	0.32	2.407	0.447	6.716	3.183	6	2610	ZINC000118937228
17.9	-9.5	-8.3	0.81	2.889	0.797	6.856	0.617	6	2611	ZINC000100076290
17.9	-9.9	-9.3	0.40	2.807	0.832	5.134	2.040	6	2612	ZINC000100829300
17.9	-11.0	-9.6	1.07	1.997	0.524	6.137	2.711	6	2613	ZINC000257415871
17.9	-10.6	-10.3	0.19	3.404	0.817	6.564	1.932	6	2614	ZINC000257475406
17.9	-9.9	-9.0	0.53	2.855	0.331	6.844	1.128	6	2615	ZINC000257503208
17.9	-11.6	-11.0	0.32	2.525	0.445	5.780	2.052	6	2616	ZINC000100827361
17.9	-10.4	-10.1	0.16	2.066	0.268	6.238	2.166	6	2617	ZINC000257621036
17.9	-11.5	-10.0	0.85	2.409	0.172	8.399	0.174	6	2618	ZINC000100772787
17.8	-9.0	-8.8	0.10	2.276	0.622	5.037	3.124	6	2619	ZINC000257533757
17.8	-10.8	-10.1	0.51	2.564	0.820	5.561	2.554	6	2620	ZINC000100825516
17.8	-11.5	-10.3	0.64	2.251	0.568	6.270	3.681	6	2621	ZINC000085879591

17.8	-9.9	-9.2	0.45	2.646	0.292	7.894	1.156	6	2622	ZINC000257396339
17.8	-9.9	-9.8	0.11	1.911	0.130	8.484	0.027	6	2623	ZINC000257567962
17.8	-9.3	-7.8	0.92	2.131	0.472	6.300	2.846	4	2624	ZINC000100358352
17.8	-10.4	-10.0	0.28	2.325	0.881	5.355	1.841	6	2625	ZINC000100774800
17.8	-10.7	-9.4	0.75	1.943	0.685	5.151	2.075	6	2626	ZINC000257455690
17.8	-11.3	-10.7	0.32	1.802	0.378	5.296	2.245	6	2627	ZINC000257395114
17.8	-11.8	-11.5	0.18	2.058	0.302	4.739	1.931	6	2628	ZINC000257453495
17.8	-10.3	-9.2	0.74	2.253	0.432	3.229	0.978	6	2629	ZINC000230092786
17.8	-10.4	-10.0	0.33	2.743	0.853	6.234	2.251	6	2630	ZINC000100774806
17.8	-10.0	-9.9	0.06	1.908	0.192	5.993	2.657	6	2631	ZINC000248338812
17.8	-8.3	-7.9	0.26	2.063	0.343	4.953	2.076	6	2632	ZINC000257620363
17.8	-9.5	-8.9	0.37	2.510	0.542	6.308	3.617	6	2633	ZINC000014488271
17.7	-9.8	-9.2	0.38	2.534	0.265	6.514	1.786	6	2634	ZINC000261499739
17.7	-9.8	-8.9	0.64	2.442	0.290	6.879	1.275	6	2635	ZINC000261499737
17.7	-9.9	-9.0	0.49	1.953	0.373	4.404	1.438	6	2636	ZINC000100827983

17.7	-9.8	-9.0	0.50	2.583	0.760	6.040	2.160	6	2637	ZINC000100829299
17.7	-9.9	-9.1	0.49	3.070	0.328	5.679	0.520	6	2638	ZINC000100829301
17.7	-10.4	-10.0	0.24	2.708	0.817	5.397	1.828	6	2639	ZINC000100774803
17.7	-10.5	-9.9	0.34	1.797	0.319	7.123	2.347	6	2640	ZINC000248332066
17.7	-9.3	-8.8	0.24	1.959	0.626	3.057	0.935	6	2641	ZINC000100828605
17.7	-10.8	-10.2	0.46	2.547	0.821	5.608	2.514	6	2642	ZINC000257607426
17.7	-9.8	-9.3	0.39	2.058	0.524	5.361	3.446	6	2643	ZINC000257530995
17.7	-11.3	-11.0	0.21	2.254	0.865	4.680	2.914	6	2644	ZINC000261496414
17.7	-10.4	-10.0	0.24	2.723	0.834	5.371	1.849	6	2645	ZINC000100774799
17.7	-11.2	-11.0	0.17	2.957	0.204	7.252	1.444	6	2646	ZINC000034037250
17.7	-10.7	-9.8	0.63	2.868	0.180	5.634	1.485	6	2647	ZINC000257377455
17.7	-9.9	-9.2	0.49	2.592	0.391	7.074	1.905	6	2648	ZINC000257396338
17.7	-9.8	-9.2	0.43	2.518	0.322	6.340	1.958	6	2649	ZINC000100827980
17.7	-9.2	-9.0	0.13	2.250	0.334	5.938	3.477	6	2650	ZINC000095620837
17.7	-9.8	-8.9	0.48	2.459	0.336	6.795	1.118	6	2651	ZINC000100827978

17.7	-9.3	-9.0	0.19	1.947	0.628	6.661	3.050	6	2652	ZINC000014718953
17.7	-10.9	-10.5	0.23	1.777	0.439	4.607	2.439	6	2653	ZINC000257514656
17.7	-9.8	-9.2	0.40	2.680	0.249	7.384	1.702	6	2654	ZINC000261499742
17.7	-9.9	-9.2	0.48	1.897	0.468	2.678	0.785	6	2655	ZINC000257530994
17.6	-10.9	-10.4	0.31	1.959	0.564	4.852	2.375	6	2656	ZINC000095619498
17.6	-9.3	-9.2	0.08	1.881	0.586	5.589	2.952	6	2657	ZINC000059732398
17.6	-12.2	-11.0	0.56	1.985	0.561	3.851	1.866	6	2658	ZINC000257483114
17.6	-11.1	-10.8	0.16	2.257	0.505	5.700	2.407	6	2659	ZINC000257402586
17.6	-9.8	-9.2	0.41	2.636	0.335	7.339	1.768	6	2660	ZINC000261499738
17.6	-10.9	-10.0	0.55	2.696	0.390	4.897	1.386	6	2661	ZINC000033963966
17.6	-9.8	-9.2	0.42	2.732	0.278	8.078	1.094	6	2662	ZINC000100827996
17.6	-10.2	-9.9	0.24	1.749	0.218	6.119	2.376	6	2663	ZINC000257577141
17.6	-9.8	-9.2	0.54	2.371	0.382	6.355	2.101	6	2664	ZINC000257396337
17.6	-10.1	-9.9	0.16	2.288	0.307	7.286	3.783	6	2665	ZINC000059770539
17.6	-11.8	-11.0	0.65	1.910	0.448	6.268	3.406	6	2666	ZINC000095619788

17.6	-9.8	-9.3	0.32	2.634	0.254	6.664	1.884	6	2667	ZINC000257396336
17.6	-11.2	-10.6	0.45	2.348	0.702	4.832	2.511	6	2668	ZINC000257619760
17.6	-9.1	-7.1	0.92	2.442	0.302	7.188	0.727	6	2669	ZINC000032787250
17.6	-9.5	-9.0	0.29	1.917	0.470	7.184	4.006	6	2670	ZINC000014821373
17.6	-9.9	-9.1	0.50	2.869	0.324	6.884	1.117	6	2671	ZINC000257503209
17.6	-11.3	-10.9	0.30	2.749	0.445	6.155	2.327	6	2672	ZINC000014720415
17.6	-10.9	-9.7	0.75	2.252	0.197	6.701	2.802	6	2673	ZINC000085567731
17.6	-9.5	-8.7	0.57	3.023	0.426	10.355	0.629	6	2674	ZINC000257768596
17.6	-11.2	-10.9	0.18	1.777	0.340	4.874	2.494	6	2675	ZINC000257380438
17.6	-10.9	-10.5	0.24	1.931	0.315	4.367	2.037	6	2676	ZINC000100780011
17.6	-9.8	-9.2	0.39	2.512	0.280	7.001	1.521	6	2677	ZINC000100827976
17.6	-9.1	-8.7	0.20	1.804	0.209	5.969	3.915	6	2678	ZINC000014760575
17.6	-12.5	-11.4	0.55	2.392	0.674	6.104	2.381	6	2679	ZINC000100781811
17.6	-11.3	-10.5	0.40	1.700	0.163	7.620	2.096	6	2680	ZINC000100779517
17.6	-9.8	-9.0	0.54	2.493	0.349	7.432	1.809	6	2681	ZINC000100827995

17.5	-11.1	-10.6	0.55	3.070	1.020	4.939	2.002	6	2682	ZINC000100772739
17.5	-9.4	-9.0	0.26	2.633	0.404	5.336	2.256	6	2683	ZINC000257451584
17.5	-9.4	-9.2	0.12	1.960	0.263	4.361	3.070	6	2684	ZINC000100825875
17.5	-11.0	-10.8	0.16	2.388	0.665	6.096	2.608	6	2685	ZINC000100780293
17.5	-10.0	-9.6	0.27	2.683	1.145	6.273	2.271	6	2686	ZINC000257475404
17.5	-12.1	-11.5	0.36	1.342	0.142	4.826	2.350	6	2687	ZINC000257434588
17.5	-9.2	-9.0	0.09	2.042	0.535	5.630	2.898	6	2688	ZINC000085946104
17.5	-11.3	-10.8	0.37	1.811	0.193	5.643	2.192	6	2689	ZINC000257466735
17.5	-9.7	-9.2	0.38	2.103	0.684	5.915	2.799	6	2690	ZINC000100828000
17.4	-9.9	-9.2	0.32	2.265	0.640	5.437	3.789	6	2691	ZINC000257461969
17.4	-9.1	-8.8	0.21	2.493	0.789	8.542	3.030	6	2692	ZINC000014644029
17.4	-12.9	-11.4	1.05	2.066	0.563	5.406	2.569	6	2693	ZINC000150361860
17.4	-9.5	-8.9	0.33	2.340	0.765	8.595	3.245	6	2694	ZINC000100828597
17.4	-9.7	-9.3	0.23	2.085	0.236	7.857	3.744	6	2695	ZINC000004095909
17.4	-11.9	-11.7	0.15	2.597	0.605	5.772	2.096	6	2696	ZINC000257488510

17.4	-9.6	-8.7	0.60	2.430	0.256	7.170	1.520	6	2697	ZINC000100827992
17.4	-10.1	-9.7	0.22	1.918	0.491	4.407	1.927	6	2698	ZINC000252463744
17.4	-11.0	-10.3	0.56	2.529	0.719	5.621	2.259	6	2699	ZINC000257480400
17.4	-11.0	-10.0	0.43	2.573	0.234	7.597	2.586	6	2700	ZINC000100297808
17.4	-10.9	-10.6	0.16	2.843	0.865	5.894	2.988	6	2701	ZINC000252516951
17.4	-9.4	-9.1	0.29	2.847	0.818	7.508	2.658	6	2702	ZINC000014718951
17.4	-10.1	-9.7	0.36	2.344	0.752	5.728	3.325	6	2703	ZINC000095617637
17.4	-11.6	-11.1	0.31	1.540	0.305	6.765	1.533	6	2704	ZINC000257561920
17.4	-10.0	-9.8	0.14	2.316	0.448	7.281	2.165	6	2705	ZINC000257443455
17.3	-10.6	-10.5	0.13	1.860	0.240	4.576	2.191	6	2706	ZINC000014617364
17.3	-10.9	-10.7	0.19	2.358	0.305	7.034	2.193	6	2707	ZINC000257377456
17.3	-10.7	-10.1	0.33	2.739	0.489	6.635	2.511	6	2708	ZINC000085648934
17.3	-10.4	-9.8	0.29	2.198	0.105	5.446	2.336	6	2709	ZINC000257640754
17.3	-11.0	-10.5	0.23	2.704	0.155	7.758	1.265	6	2710	ZINC000150352527
17.3	-9.2	-8.8	0.24	2.125	0.843	4.300	2.947	6	2711	ZINC000014811716

17.3	-11.0	-10.7	0.24	1.654	0.367	3.778	1.991	6	2712	ZINC000014720435
17.3	-11.0	-10.9	0.13	1.847	0.118	6.603	1.706	6	2713	ZINC000100782745
17.3	-8.6	-8.5	0.11	1.976	0.359	5.858	3.342	6	2714	ZINC000015152846
17.3	-10.0	-9.7	0.27	2.172	0.570	6.933	3.838	6	2715	ZINC000257451583
17.3	-10.8	-10.3	0.25	2.378	0.485	8.063	3.028	6	2716	ZINC000085991642
17.2	-11.0	-9.4	1.00	2.118	0.445	6.484	1.168	6	2717	ZINC000150360874
17.2	-11.8	-10.6	0.79	2.092	0.592	6.280	1.697	6	2718	ZINC000085886536
17.2	-10.7	-10.3	0.24	2.560	0.220	6.295	1.779	6	2719	ZINC000085637986
17.2	-11.6	-10.4	0.75	2.181	0.351	8.363	2.018	6	2720	ZINC000072173638
17.2	-9.7	-9.0	0.37	2.479	0.133	10.135	0.208	6	2721	ZINC000257768589
17.2	-10.3	-9.6	0.40	2.695	0.539	7.051	2.447	6	2722	ZINC000100825517
17.2	-10.2	-10.1	0.10	1.897	0.251	5.708	2.196	6	2723	ZINC000257531618
17.2	-10.1	-9.8	0.22	2.174	0.171	7.332	1.797	6	2724	ZINC000100777953
17.2	-10.6	-10.1	0.35	2.394	0.330	6.679	1.293	6	2725	ZINC000100777886
17.2	-10.0	-9.6	0.29	2.001	0.339	4.303	1.939	6	2726	ZINC000257546996

17.2	-9.9	-9.5	0.17	2.305	0.333	7.220	2.983	6	2727	ZINC000014709118
17.2	-11.3	-10.6	0.38	1.998	0.442	5.173	2.502	6	2728	ZINC000100780014
17.2	-11.5	-10.3	0.68	2.383	0.085	7.974	0.214	6	2729	ZINC000085991692
17.2	-10.6	-10.2	0.30	2.272	0.379	6.197	2.783	6	2730	ZINC000150368588
17.2	-11.9	-11.3	0.39	1.854	0.194	6.976	1.330	6	2731	ZINC000257465215
17.2	-11.9	-11.3	0.38	1.871	0.434	5.769	2.252	6	2732	ZINC000100771781
17.2	-10.2	-8.9	0.69	2.051	0.095	7.272	2.103	6	2733	ZINC000095914543
17.2	-11.9	-10.8	0.60	2.217	0.379	3.971	0.417	6	2734	ZINC000257505906
17.2	-11.4	-10.3	0.50	2.655	0.377	5.803	2.682	6	2735	ZINC000150368162
17.2	-10.7	-10.0	0.42	2.221	0.286	4.711	2.984	6	2736	ZINC000100826077
17.2	-10.3	-9.6	0.45	1.989	0.276	5.526	3.516	6	2737	ZINC000100825637
17.2	-10.6	-10.4	0.12	2.367	1.010	4.765	2.400	6	2738	ZINC000257392669
17.1	-11.4	-10.8	0.45	2.896	1.628	5.394	3.819	6	2739	ZINC000257658471
17.1	-10.9	-10.6	0.19	2.284	0.278	5.622	1.490	6	2740	ZINC000150362907
17.1	-11.1	-11.0	0.11	2.096	0.208	5.932	1.868	6	2741	ZINC000261496026

17.1	-11.2	-10.9	0.24	2.233	0.216	7.361	1.371	6	2742	ZINC000096016064
17.1	-11.4	-10.8	0.47	2.883	1.276	5.099	3.162	6	2743	ZINC000118937035
17.1	-8.6	-8.5	0.10	1.530	0.484	4.133	2.921	6	2744	ZINC000085946091
17.1	-10.6	-10.0	0.38	2.114	0.655	4.120	2.743	6	2745	ZINC000118937026
17.1	-11.5	-11.2	0.24	1.636	0.123	5.393	2.513	6	2746	ZINC000257561919
17.1	-9.3	-8.7	0.33	2.269	0.678	3.493	0.833	6	2747	ZINC000257768586
17.1	-8.7	-8.5	0.17	1.794	0.376	7.143	3.583	6	2748	ZINC000095618138
17.1	-10.3	-9.7	0.46	2.525	0.763	5.557	2.542	6	2749	ZINC000257607427
17.1	-11.2	-10.7	0.28	1.668	0.505	3.836	2.105	6	2750	ZINC000100777648
17.1	-10.7	-10.1	0.43	1.750	0.122	3.754	2.352	6	2751	ZINC000257488043
17.1	-11.5	-10.6	0.42	2.797	0.277	7.936	0.293	6	2752	ZINC000257738528
17.1	-9.2	-9.1	0.07	2.019	0.523	7.305	3.637	6	2753	ZINC000257507079
17.1	-11.6	-11.4	0.12	1.499	0.121	4.537	2.653	6	2754	ZINC000014720087
17.1	-10.6	-9.7	0.58	2.405	0.451	5.050	2.905	6	2755	ZINC000253604921
17.1	-9.9	-9.5	0.31	2.179	0.566	3.166	0.459	6	2756	ZINC000118937195

17.1	-9.9	-9.2	0.49	2.667	0.801	5.764	2.435	6	2757	ZINC000257503207
17.1	-11.3	-11.1	0.12	2.305	0.226	5.854	1.699	6	2758	ZINC000015105349
17.0	-11.3	-10.7	0.29	2.038	0.301	6.017	2.415	6	2759	ZINC000100777618
17.0	-11.0	-10.0	0.43	1.779	0.375	5.065	2.499	6	2760	ZINC000095619501
17.0	-11.3	-10.8	0.24	2.259	0.615	5.673	2.485	6	2761	ZINC000256507439
17.0	-12.4	-11.5	0.45	2.627	0.694	7.162	1.391	6	2762	ZINC000100779618
17.0	-10.2	-10.1	0.08	2.539	0.383	7.078	1.456	6	2763	ZINC000100779062
17.0	-9.9	-9.7	0.13	2.172	0.598	5.713	2.842	6	2764	ZINC000013435203
17.0	-12.4	-11.5	0.45	2.409	0.601	7.412	1.262	6	2765	ZINC000257565511
17.0	-11.4	-10.8	0.37	2.277	1.187	3.898	2.923	6	2766	ZINC000118937036
17.0	-10.7	-10.2	0.39	2.555	0.143	8.001	1.646	6	2767	ZINC000100822016
17.0	-12.5	-11.6	0.60	2.131	0.153	6.194	1.833	6	2768	ZINC000085977724
17.0	-11.4	-10.8	0.27	1.677	0.565	4.094	2.718	6	2769	ZINC000095918729
17.0	-10.9	-10.6	0.18	2.337	0.349	6.464	1.817	6	2770	ZINC000257414988
17.0	-11.4	-10.6	0.50	4.148	1.219	8.169	3.030	6	2771	ZINC000118937037

17.0	-11.4	-10.8	0.41	2.285	1.223	3.990	2.894	6	2772	ZINC000257658473
17.0	-12.4	-11.5	0.46	2.660	0.632	7.158	1.384	6	2773	ZINC000100779621
17.0	-11.2	-10.5	0.35	1.884	0.248	5.016	2.438	6	2774	ZINC000100780013
17.0	-12.4	-11.8	0.43	2.921	1.077	7.257	3.234	6	2775	ZINC000257498924
17.0	-11.4	-10.6	0.49	3.294	1.313	5.844	3.351	6	2776	ZINC000257658474
17.0	-11.4	-10.6	0.50	3.587	1.397	6.725	3.512	6	2777	ZINC000257658472
17.0	-12.4	-11.5	0.45	2.407	0.604	7.416	1.249	6	2778	ZINC000257565512
17.0	-10.2	-10.0	0.14	1.859	0.126	5.166	2.461	6	2779	ZINC000100829675
17.0	-10.1	-9.8	0.21	2.781	0.336	8.629	2.321	6	2780	ZINC000257455851
17.0	-10.4	-9.9	0.26	1.941	0.448	4.962	2.726	6	2781	ZINC000014766825
17.0	-10.6	-9.9	0.37	2.312	0.342	7.503	0.902	6	2782	ZINC000257391640
17.0	-11.9	-11.5	0.22	2.245	0.182	5.575	1.354	6	2783	ZINC000095617885
17.0	-10.0	-9.4	0.42	2.171	0.338	5.321	2.226	6	2784	ZINC000256119448
16.9	-10.2	-9.8	0.27	2.340	0.511	6.456	2.771	6	2785	ZINC000150361525
16.9	-9.8	-9.7	0.21	1.978	0.614	5.485	2.743	6	2786	ZINC000256463111

16.9	-10.4	-10.2	0.12	2.419	0.375	5.278	2.624	6	2787	ZINC000015105775
16.9	-10.9	-10.6	0.21	2.427	0.435	6.815	2.220	6	2788	ZINC000014617324
16.9	-10.0	-9.7	0.17	2.823	0.869	7.212	3.442	6	2789	ZINC000118937233
16.9	-9.2	-8.9	0.17	2.242	0.574	3.346	0.760	6	2790	ZINC000004098984
16.9	-10.1	-10.0	0.09	2.140	0.617	7.230	2.571	6	2791	ZINC000100772609
16.9	-8.5	-7.4	0.76	2.483	0.527	7.954	1.426	6	2792	ZINC000150362037
16.9	-11.6	-10.9	0.30	1.954	0.354	6.355	1.539	6	2793	ZINC000015105347
16.9	-10.1	-9.7	0.21	2.155	0.304	7.075	3.280	6	2794	ZINC000100827211
16.9	-11.4	-10.8	0.38	2.363	1.145	4.019	2.891	6	2795	ZINC000118937034
16.9	-9.0	-8.8	0.11	2.346	0.797	6.609	3.286	6	2796	ZINC000257406751
16.9	-10.9	-10.6	0.22	2.292	0.347	6.597	1.768	6	2797	ZINC000257414990
16.9	-9.7	-9.1	0.52	2.173	0.553	7.219	3.977	6	2798	ZINC000257437483
16.9	-11.9	-11.0	0.45	1.943	0.315	4.190	1.821	6	2799	ZINC000013335902
16.9	-10.8	-10.2	0.31	2.010	0.520	7.066	3.903	6	2800	ZINC000257528196
16.9	-10.7	-10.6	0.07	1.866	0.162	4.967	2.028	6	2801	ZINC000014617321

16.9	-9.6	-8.7	0.49	2.918	0.759	6.853	2.637	6	2802	ZINC000040164362
16.9	-11.1	-10.0	1.15	1.882	0.530	4.957	1.956	6	2803	ZINC000150351798
16.9	-10.9	-10.7	0.17	1.974	0.638	5.649	2.575	6	2804	ZINC000257414989
16.9	-10.9	-10.7	0.18	1.802	0.600	4.352	2.372	6	2805	ZINC000150362916
16.9	-11.0	-10.1	0.47	2.245	0.077	7.320	1.539	6	2806	ZINC000257396946
16.9	-11.1	-10.2	0.48	2.674	0.516	7.041	2.960	6	2807	ZINC000261494616
16.9	-10.0	-9.6	0.49	2.162	0.321	6.352	2.724	6	2808	ZINC000100823824
16.9	-10.9	-10.6	0.21	2.220	0.438	6.585	1.713	6	2809	ZINC000150362913
16.9	-10.0	-9.6	0.27	2.045	0.320	6.086	2.057	6	2810	ZINC000242543352
16.9	-10.9	-10.7	0.22	1.873	0.340	5.045	2.258	6	2811	ZINC000257483310
16.9	-11.2	-10.3	0.58	2.115	0.488	5.388	2.138	6	2812	ZINC000257501128
16.9	-10.6	-10.4	0.19	1.930	0.271	5.854	2.245	6	2813	ZINC000257422803
16.8	-6.6	-5.2	1.09	2.371	0.468	6.926	1.894	6	2814	ZINC000100773134
16.8	-11.8	-11.2	0.51	2.161	0.198	5.466	2.790	6	2815	ZINC000257481895
16.8	-10.8	-10.6	0.23	2.397	0.408	6.514	1.764	6	2816	ZINC000257414987

16.8	-11.3	-10.4	0.45	2.102	0.317	6.991	1.539	6	2817	ZINC000038143792
16.8	-10.1	-9.9	0.12	2.108	0.564	6.318	2.553	6	2818	ZINC000100772611
16.8	-11.4	-10.7	0.37	2.150	0.253	7.651	2.237	6	2819	ZINC000257465214
16.8	-11.0	-10.6	0.37	1.702	0.435	5.189	1.807	6	2820	ZINC000100824611
16.8	-10.3	-9.3	0.59	2.248	0.095	4.887	1.144	6	2821	ZINC000100772199
16.8	-11.4	-11.0	0.22	2.751	0.914	7.242	3.023	6	2822	ZINC000257498922
16.8	-11.1	-9.9	0.79	2.368	0.331	6.308	0.409	6	2823	ZINC000150360865
16.8	-10.6	-10.2	0.23	2.471	0.969	5.785	2.514	6	2824	ZINC000100771609
16.8	-10.9	-10.4	0.33	1.691	0.227	5.595	2.960	6	2825	ZINC000100779523
16.8	-11.4	-10.2	0.77	2.038	0.737	5.145	2.972	6	2826	ZINC000085860845
16.8	-10.3	-10.0	0.14	2.531	0.230	4.373	1.510	6	2827	ZINC000257531617
16.8	-8.8	-8.5	0.24	2.142	0.502	3.288	0.365	6	2828	ZINC000014644023
16.8	-10.1	-10.0	0.07	2.078	0.617	7.130	2.518	6	2829	ZINC000100772614
16.8	-10.8	-10.6	0.21	2.161	0.433	6.405	1.856	6	2830	ZINC000150362902
16.8	-9.8	-9.4	0.31	2.007	0.330	4.953	2.642	6	2831	ZINC000257590967

16.8	-11.4	-10.5	0.43	3.453	0.220	7.380	2.060	6	2832	ZINC000119384068
16.8	-11.2	-10.5	0.35	2.333	0.214	5.670	2.416	6	2833	ZINC000257449431
16.8	-12.4	-11.1	0.82	2.145	0.393	5.863	1.237	6	2834	ZINC000150351528
16.8	-11.5	-11.1	0.32	3.098	0.868	6.883	1.416	6	2835	ZINC000001611808
16.7	-10.9	-10.8	0.11	1.909	0.523	5.965	2.992	6	2836	ZINC000100773907
16.7	-10.3	-9.5	0.36	1.891	0.268	5.204	2.108	6	2837	ZINC000252463741
16.7	-10.2	-10.0	0.14	2.167	0.449	5.624	2.134	6	2838	ZINC000254685395
16.7	-12.4	-11.5	0.55	1.981	0.317	6.234	1.741	6	2839	ZINC000095619532
16.7	-11.3	-11.1	0.13	1.724	0.275	7.178	1.832	6	2840	ZINC000100779186
16.7	-11.9	-11.3	0.33	2.079	0.446	5.444	1.927	6	2841	ZINC000024215027
16.7	-10.7	-10.5	0.14	1.620	0.134	3.746	1.881	6	2842	ZINC000014617322
16.7	-12.3	-12.1	0.15	2.080	0.473	4.293	2.076	6	2843	ZINC000024215034
16.7	-8.6	-7.3	1.00	3.144	0.629	5.816	1.882	6	2844	ZINC000026671872
16.7	-11.4	-10.9	0.31	2.221	0.611	6.292	1.790	6	2845	ZINC000038842195
16.7	-11.3	-10.8	0.29	1.798	0.235	5.253	1.727	6	2846	ZINC000085954782

16.7	-10.7	-10.2	0.28	2.634	0.891	5.656	3.440	6	2847	ZINC000100828143
16.7	-11.0	-10.7	0.15	2.169	0.115	6.402	2.279	6	2848	ZINC000100780831
16.7	-10.1	-9.6	0.30	2.428	0.160	4.963	2.100	6	2849	ZINC000257521780
16.7	-10.0	-9.9	0.11	2.296	0.717	6.398	2.645	6	2850	ZINC000100772613
16.7	-11.4	-10.3	0.71	1.946	0.578	4.955	2.104	6	2851	ZINC000042806950
16.7	-12.1	-11.3	0.49	2.180	0.412	5.162	2.152	6	2852	ZINC000014720213
16.7	-11.4	-11.0	0.26	1.619	0.397	2.386	0.358	6	2853	ZINC000118936770
16.7	-8.4	-7.1	0.91	2.384	0.987	6.871	1.131	5	2854	ZINC000135867865
16.6	-10.2	-9.5	0.45	2.131	0.739	5.380	3.553	6	2855	ZINC000100825636
16.6	-11.5	-11.3	0.14	1.669	0.363	3.901	2.008	6	2856	ZINC000257434589
16.6	-11.4	-10.8	0.36	1.761	0.292	2.668	0.304	6	2857	ZINC000257412746
16.6	-10.4	-9.3	0.52	2.702	0.742	6.950	0.357	6	2858	ZINC000100828146
16.6	-10.6	-10.3	0.15	1.921	0.406	4.909	2.641	6	2859	ZINC000100779524
16.6	-10.0	-9.6	0.30	1.644	0.356	4.315	3.154	6	2860	ZINC000100825181
16.6	-10.8	-10.5	0.15	2.037	0.296	5.586	2.135	6	2861	ZINC000257621913

16.6	-11.4	-10.9	0.30	2.001	0.549	3.763	2.678	6	2862	ZINC000118936769
16.6	-10.5	-9.9	0.33	1.890	0.327	5.993	2.854	6	2863	ZINC000100777956
16.6	-10.8	-10.3	0.30	2.559	0.117	4.542	0.580	6	2864	ZINC000100774082
16.6	-11.4	-10.8	0.38	2.003	0.766	4.035	3.140	6	2865	ZINC000118936772
16.6	-9.8	-9.4	0.19	2.440	0.416	4.945	2.626	6	2866	ZINC000257426304
16.6	-11.1	-10.9	0.13	2.703	0.223	6.041	2.008	6	2867	ZINC000032790299
16.6	-10.6	-10.2	0.29	1.879	0.196	4.615	1.836	6	2868	ZINC000257621034
16.6	-11.4	-10.8	0.39	2.058	0.578	3.926	2.925	6	2869	ZINC000257412743
16.6	-11.3	-11.0	0.22	1.770	0.457	5.969	2.741	6	2870	ZINC000100781810
16.6	-11.2	-11.0	0.16	2.089	0.522	5.177	2.056	6	2871	ZINC000014720417
16.6	-11.3	-10.7	0.42	1.753	0.226	5.276	2.161	6	2872	ZINC000254697458
16.6	-11.4	-10.9	0.35	1.610	0.323	2.340	0.241	6	2873	ZINC000257412745
16.6	-11.0	-10.3	0.37	2.182	0.219	7.963	2.782	6	2874	ZINC000072173637
16.6	-11.3	-10.7	0.34	2.520	0.305	7.168	1.109	6	2875	ZINC000085850638
16.6	-10.7	-10.3	0.23	1.678	0.210	4.139	1.848	6	2876	ZINC000100783642

16.6	-10.8	-10.0	0.39	2.053	0.374	5.510	1.537	6	2877	ZINC000257639038
16.6	-11.1	-10.9	0.13	1.541	0.057	5.732	2.495	6	2878	ZINC000100779428
16.6	-11.3	-9.9	0.68	2.331	0.139	6.793	0.369	6	2879	ZINC000095914547
16.5	-9.8	-9.3	0.29	2.611	0.229	5.483	2.196	6	2880	ZINC000257426306
16.5	-10.4	-10.3	0.09	2.680	1.017	5.880	2.493	6	2881	ZINC000257568260
16.5	-10.5	-9.6	0.72	2.235	0.378	6.568	2.632	6	2882	ZINC000085853635
16.5	-11.0	-10.0	0.43	2.428	0.252	5.462	1.755	6	2883	ZINC000253497555
16.5	-11.1	-10.5	0.31	2.323	0.495	6.158	1.898	6	2884	ZINC000100829677
16.5	-10.8	-10.3	0.32	2.280	0.329	4.658	2.261	6	2885	ZINC000257505907
16.5	-11.1	-10.6	0.41	2.432	0.294	4.808	0.710	6	2886	ZINC000100827921
16.5	-10.4	-9.8	0.33	2.516	0.570	10.340	0.108	6	2887	ZINC000015104412
16.5	-11.2	-10.5	0.41	2.157	0.130	6.146	1.245	6	2888	ZINC000150364957
16.5	-9.6	-8.0	0.90	2.032	0.698	4.591	2.625	5	2889	ZINC000085599272
16.5	-9.8	-9.3	0.22	2.675	0.161	5.760	2.015	6	2890	ZINC000257426305
16.5	-9.8	-9.4	0.24	2.632	0.245	5.518	2.187	6	2891	ZINC000100829447

16.5	-11.0	-10.4	0.38	2.122	0.695	4.587	2.956	6	2892	ZINC000100297810
16.5	-9.7	-9.1	0.28	2.716	0.187	5.862	1.983	6	2893	ZINC000100829449
16.5	-9.7	-9.2	0.27	2.638	0.126	5.661	2.046	6	2894	ZINC000257426307
16.5	-11.0	-10.3	0.39	2.343	0.352	5.214	2.555	6	2895	ZINC000100297807
16.5	-8.2	-7.4	0.58	1.975	0.430	5.561	2.231	6	2896	ZINC000257397850
16.5	-10.7	-10.0	0.40	2.238	0.317	5.957	1.136	6	2897	ZINC000150361956
16.5	-9.8	-9.4	0.20	2.381	0.324	4.829	2.604	6	2898	ZINC000100829455
16.5	-10.8	-10.6	0.16	2.705	0.445	7.761	2.154	6	2899	ZINC000100826027
16.5	-11.0	-10.3	0.41	2.187	0.582	5.240	2.728	6	2900	ZINC000100297813
16.5	-11.0	-10.5	0.36	1.937	0.442	4.120	1.904	6	2901	ZINC000257435389
16.5	-11.6	-10.5	0.60	1.898	0.584	6.378	3.496	6	2902	ZINC000085991695
16.5	-11.4	-10.5	0.41	1.997	0.614	6.053	2.503	6	2903	ZINC000095619503
16.5	-10.6	-10.4	0.14	2.076	0.315	5.647	2.310	6	2904	ZINC000100777329
16.5	-11.4	-10.8	0.39	1.817	0.722	3.870	2.963	6	2905	ZINC000257412744
16.5	-11.0	-10.7	0.17	1.721	0.062	5.021	2.089	6	2906	ZINC000257566878

16.5	-11.4	-10.8	0.42	2.090	0.944	5.169	3.721	6	2907	ZINC000118936771
16.5	-10.9	-9.9	0.51	2.330	0.139	7.407	1.941	6	2908	ZINC000085991691
16.5	-10.5	-10.3	0.13	2.467	0.386	5.694	1.893	6	2909	ZINC000100829676
16.5	-11.0	-10.5	0.31	2.224	0.420	3.939	0.958	6	2910	ZINC000253497556
16.5	-10.4	-9.5	0.98	1.456	0.363	4.392	2.000	6	2911	ZINC000100824613
16.4	-10.0	-9.6	0.34	2.217	1.120	5.393	1.619	6	2912	ZINC000100771610
16.4	-10.3	-10.1	0.17	2.065	0.680	7.078	2.587	6	2913	ZINC000100777511
16.4	-10.5	-10.0	0.25	1.786	0.329	5.340	2.187	6	2914	ZINC000257451381
16.4	-10.3	-10.0	0.17	1.773	0.250	6.878	1.999	6	2915	ZINC000247952289
16.4	-10.2	-9.9	0.18	2.444	0.265	7.482	2.494	6	2916	ZINC000013775870
16.4	-10.9	-10.7	0.20	1.858	1.205	4.350	2.843	6	2917	ZINC000012496345
16.4	-7.9	-6.7	0.67	3.437	1.259	5.951	0.695	6	2918	ZINC000100277550
16.4	-9.9	-9.2	0.32	2.484	0.673	7.004	3.829	6	2919	ZINC000257461967
16.4	-12.4	-11.7	0.38	2.783	0.756	5.786	2.514	6	2920	ZINC000242547644
16.4	-11.3	-10.9	0.37	2.653	0.548	5.743	0.829	6	2921	ZINC000257413142

16.4	-10.6	-9.8	0.88	2.089	0.139	7.071	1.952	6	2922	ZINC000150351529
16.4	-12.4	-11.5	0.49	2.857	0.619	6.540	1.639	6	2923	ZINC000242547643
16.4	-9.7	-9.6	0.11	3.812	0.672	7.283	1.828	6	2924	ZINC000014960825
16.4	-10.8	-10.1	0.48	2.449	0.265	4.323	0.744	6	2925	ZINC000253527791
16.4	-11.5	-10.0	0.84	2.763	0.485	7.867	1.699	6	2926	ZINC000257478362
16.4	-10.3	-10.1	0.13	2.117	0.738	6.240	2.794	6	2927	ZINC000100777508
16.4	-11.2	-10.5	0.36	2.638	0.540	6.671	0.426	6	2928	ZINC000257461489
16.4	-10.0	-9.7	0.23	2.215	0.354	7.489	2.629	6	2929	ZINC000257376543
16.4	-10.8	-10.5	0.22	1.986	0.419	4.763	2.405	6	2930	ZINC000014617361
16.4	-11.9	-11.1	0.43	2.317	0.196	6.081	1.723	6	2931	ZINC000100778886
16.4	-10.5	-10.3	0.12	2.944	0.323	6.926	1.218	6	2932	ZINC000100777331
16.4	-12.4	-11.7	0.33	2.452	0.849	6.588	2.589	6	2933	ZINC000100779159
16.4	-10.3	-10.0	0.19	2.070	0.682	6.251	2.622	6	2934	ZINC000100777510
16.4	-10.5	-9.8	0.83	1.727	0.440	4.878	2.662	6	2935	ZINC000257620364
16.4	-9.9	-9.5	0.24	2.361	0.248	4.440	2.478	6	2936	ZINC000085955994

16.4	-11.1	-10.6	0.31	2.427	0.717	5.871	2.640	6	2937	ZINC000253477226
16.4	-11.0	-10.3	0.45	2.628	0.469	5.390	1.813	6	2938	ZINC000256005425
16.4	-9.9	-9.3	0.47	2.520	0.575	5.003	2.633	6	2939	ZINC000012153680
16.4	-12.4	-11.7	0.34	2.493	0.789	6.620	2.529	6	2940	ZINC000242547645
16.4	-11.2	-10.9	0.20	2.025	0.327	6.753	1.294	6	2941	ZINC000168534108
16.4	-10.7	-10.6	0.08	2.224	0.247	6.196	2.040	6	2942	ZINC000095619989
16.3	-10.8	-9.8	0.64	2.637	0.117	4.752	0.501	6	2943	ZINC000100828606
16.3	-10.6	-10.1	0.42	2.547	0.352	5.442	2.225	6	2944	ZINC000100776699
16.3	-10.8	-10.5	0.18	1.733	0.254	3.731	2.210	6	2945	ZINC000257474272
16.3	-10.5	-9.8	0.32	1.898	0.241	5.931	2.638	6	2946	ZINC000100777733
16.3	-11.7	-11.2	0.25	2.719	0.243	7.407	1.650	6	2947	ZINC000257427003
16.3	-11.0	-10.7	0.19	1.780	0.131	5.777	2.047	6	2948	ZINC000100777623
16.3	-10.0	-9.3	0.39	1.868	0.762	5.517	1.692	6	2949	ZINC000257475407
16.3	-11.2	-9.8	1.06	1.718	0.898	5.148	2.997	6	2950	ZINC000085977728
16.3	-12.4	-11.5	0.50	2.860	0.628	6.550	1.634	6	2951	ZINC000100779155

16.3	-10.9	-10.2	0.42	2.571	0.146	4.786	0.590	6	2952	ZINC000100774086
16.3	-10.8	-10.4	0.26	2.238	0.394	5.401	2.215	6	2953	ZINC000014617792
16.3	-10.1	-9.7	0.19	2.426	0.377	6.435	1.903	6	2954	ZINC000257544502
16.3	-11.2	-10.5	0.61	2.475	0.644	6.441	2.965	6	2955	ZINC000150359320
16.3	-10.0	-9.6	0.30	2.118	1.006	5.840	1.975	6	2956	ZINC000257475405
16.3	-10.8	-9.5	0.95	2.503	0.823	8.813	3.423	6	2957	ZINC000257772034
16.2	-10.5	-10.2	0.20	2.744	0.394	7.355	1.699	6	2958	ZINC000247952264
16.2	-10.3	-9.8	0.26	2.691	0.940	6.173	3.324	6	2959	ZINC000257392248
16.2	-9.1	-8.9	0.13	1.836	0.624	7.398	3.687	6	2960	ZINC000014821371
16.2	-11.0	-10.3	0.33	1.807	0.256	6.217	1.912	6	2961	ZINC000257546336
16.2	-9.3	-8.9	0.37	2.016	1.308	5.376	3.889	6	2962	ZINC000150338771
16.2	-10.2	-10.1	0.14	2.064	0.676	6.133	2.709	6	2963	ZINC000100777507
16.2	-10.5	-10.1	0.21	2.332	0.343	6.250	1.626	6	2964	ZINC000257402141
16.2	-10.8	-9.9	0.58	2.384	0.172	6.088	0.729	6	2965	ZINC000230071369
16.2	-11.5	-10.8	0.33	1.769	0.462	4.009	1.987	6	2966	ZINC000014720431

16.2	-10.9	-10.5	0.31	2.187	0.712	5.159	2.185	6	2967	ZINC000014610681
16.2	-11.6	-11.0	0.48	2.302	0.453	5.829	1.808	6	2968	ZINC000257474271
16.2	-10.0	-9.8	0.11	2.697	0.376	6.931	2.089	6	2969	ZINC000257377833
16.2	-11.6	-10.7	0.44	1.892	0.192	7.544	0.211	6	2970	ZINC000100777620
16.2	-9.7	-9.5	0.18	2.287	0.372	7.131	2.649	6	2971	ZINC000257403663
16.1	-10.8	-9.4	0.68	2.674	0.260	6.495	2.229	6	2972	ZINC000257749069
16.1	-10.5	-10.0	0.33	2.446	0.129	5.725	1.529	6	2973	ZINC000100826860
16.1	-11.2	-10.5	0.34	2.846	0.466	5.986	3.108	6	2974	ZINC000118915220
16.1	-10.1	-9.8	0.16	1.727	0.489	5.125	2.589	6	2975	ZINC000100777942
16.1	-10.8	-9.6	0.59	1.877	0.238	4.660	1.710	6	2976	ZINC000257461490
16.1	-10.5	-10.0	0.29	2.063	0.290	5.808	2.010	6	2977	ZINC000100775273
16.1	-11.1	-10.5	0.26	2.339	0.288	6.148	2.181	6	2978	ZINC000015106717
16.1	-11.2	-10.7	0.30	1.939	0.405	3.851	2.082	6	2979	ZINC000100783640
16.1	-10.9	-10.0	0.58	2.539	0.277	5.892	0.518	6	2980	ZINC000257749070
16.1	-10.6	-10.0	0.49	2.444	0.373	5.377	2.708	6	2981	ZINC000253604920

16.1	-11.2	-10.4	0.39	2.613	0.542	5.743	3.250	6	2982	ZINC000118915218
16.1	-10.4	-9.7	0.38	1.982	0.315	5.574	3.644	6	2983	ZINC000257477343
16.1	-11.3	-10.2	0.71	2.521	0.411	6.609	2.399	6	2984	ZINC000100830075
16.1	-9.7	-9.2	0.28	2.171	0.653	6.329	3.468	6	2985	ZINC000257487332
16.1	-10.0	-9.2	0.55	2.020	0.243	6.849	2.014	6	2986	ZINC000086047511
16.1	-10.6	-9.6	0.60	2.204	0.666	6.759	3.197	6	2987	ZINC000257414161
16.1	-9.6	-9.4	0.12	2.528	0.370	3.616	0.514	6	2988	ZINC000257455859
16.1	-11.3	-10.3	0.67	2.423	0.481	4.784	2.570	6	2989	ZINC000100830072
16.1	-11.0	-10.6	0.26	2.282	0.217	7.983	1.123	6	2990	ZINC000029043135
16.1	-10.7	-10.5	0.10	2.515	0.395	8.080	1.682	6	2991	ZINC000100779379
16.1	-11.3	-10.6	0.41	2.110	0.260	3.885	0.908	6	2992	ZINC000100830074
16.1	-11.3	-10.3	0.63	2.275	0.571	5.542	2.196	6	2993	ZINC000257706817
16.1	-10.2	-9.8	0.18	2.495	0.304	6.345	2.609	6	2994	ZINC000100779929
16.1	-11.3	-10.5	0.47	2.175	0.353	4.130	1.206	6	2995	ZINC000257706819
16.1	-11.0	-10.5	0.26	2.128	0.141	6.021	1.674	6	2996	ZINC000014720331

16.1	-11.2	-10.3	0.43	2.583	0.569	5.968	2.992	6	2997	ZINC000118915221
16.1	-10.7	-10.2	0.33	1.860	0.108	7.356	0.105	6	2998	ZINC000100781610
16.1	-10.5	-10.1	0.24	2.302	0.219	3.984	0.721	6	2999	ZINC000095620386
16.0	-11.6	-10.6	0.51	1.958	0.397	4.670	2.439	6	3000	ZINC000257474270
16.0	-11.2	-10.5	0.32	2.182	0.229	3.945	0.875	6	3001	ZINC000257706820
16.0	-8.9	-7.8	0.90	2.248	0.289	4.292	0.917	5	3002	ZINC000100827213
16.0	-10.6	-10.2	0.21	1.771	0.292	4.759	2.584	6	3003	ZINC000257514657
16.0	-10.4	-9.8	0.43	2.256	0.675	5.032	2.307	6	3004	ZINC000100777893
16.0	-9.5	-9.4	0.16	2.128	0.663	3.295	0.727	6	3005	ZINC000014921631
16.0	-10.8	-10.2	0.28	2.229	0.499	6.676	3.182	6	3006	ZINC000257420546
16.0	-10.6	-10.1	0.30	2.381	0.321	4.079	0.757	6	3007	ZINC000253604918
16.0	-8.2	-8.0	0.07	3.066	0.804	4.999	2.769	6	3008	ZINC000001591042
16.0	-10.6	-9.5	0.56	2.583	0.403	7.322	2.504	6	3009	ZINC000100825595
16.0	-9.8	-9.5	0.23	2.454	0.746	7.277	3.561	6	3010	ZINC000100771913
16.0	-11.1	-10.4	0.43	2.266	0.371	5.059	2.054	6	3011	ZINC000257458424

16.0	-10.7	-10.3	0.21	2.352	0.393	5.195	2.260	6	3012	ZINC000015106714
16.0	-10.4	-10.2	0.14	1.699	0.146	5.887	2.394	6	3013	ZINC000257488041
16.0	-9.1	-8.8	0.20	2.504	0.393	6.104	2.139	6	3014	ZINC000085926508
16.0	-11.9	-11.2	0.34	1.777	0.209	6.897	2.346	6	3015	ZINC000095619530
16.0	-11.2	-10.1	0.59	2.346	0.282	5.765	2.087	6	3016	ZINC000095619789
16.0	-7.2	-5.7	0.73	1.521	0.130	6.688	0.372	6	3017	ZINC000257808239
16.0	-10.8	-10.2	0.27	2.319	0.256	7.180	2.613	6	3018	ZINC000100825539
16.0	-9.9	-9.5	0.36	2.229	0.320	4.744	0.724	6	3019	ZINC000100828610
16.0	-10.1	-9.8	0.27	2.078	0.196	6.782	1.354	6	3020	ZINC000100777805
16.0	-10.8	-10.4	0.22	2.526	0.258	4.088	0.637	6	3021	ZINC000100825540
16.0	-10.5	-10.0	0.24	2.444	0.330	8.844	0.167	6	3022	ZINC000100822017
16.0	-10.8	-10.5	0.18	2.217	0.502	4.918	2.219	6	3023	ZINC000100825543
16.0	-11.7	-11.2	0.45	2.386	0.400	6.308	1.175	6	3024	ZINC000150366669
16.0	-11.1	-10.3	0.43	2.664	0.557	6.017	3.087	6	3025	ZINC000261494619
16.0	-10.8	-10.3	0.25	2.276	0.305	6.535	2.447	6	3026	ZINC000257420545

16.0	-10.8	-10.3	0.33	3.103	0.278	6.705	1.564	6	3027	ZINC000014243547
16.0	-11.1	-10.3	0.45	2.604	0.512	4.536	2.542	6	3028	ZINC000261494618
16.0	-9.5	-9.2	0.24	2.366	0.859	4.461	2.740	6	3029	ZINC000257455861
16.0	-11.2	-10.4	0.40	2.140	0.456	3.533	0.803	6	3030	ZINC000257706818
16.0	-11.6	-10.9	0.39	2.220	0.449	4.269	2.342	6	3031	ZINC000257482003
16.0	-10.5	-9.7	0.42	2.021	0.521	6.031	3.858	6	3032	ZINC000100827833
16.0	-11.1	-10.4	0.40	2.626	0.503	6.943	3.329	6	3033	ZINC000261494617
16.0	-8.4	-7.5	1.06	3.290	0.744	7.605	1.770	5	3034	ZINC000032786290
16.0	-11.5	-11.0	0.26	2.049	0.154	4.879	1.871	6	3035	ZINC000257516997
16.0	-7.3	-5.7	0.76	1.462	0.094	7.140	1.171	6	3036	ZINC000257808240
16.0	-8.6	-6.9	0.86	2.472	0.231	6.940	1.718	6	3037	ZINC000257628368
16.0	-11.2	-10.8	0.31	2.089	0.222	6.297	1.993	6	3038	ZINC000257480082
16.0	-11.2	-10.5	0.33	2.848	0.457	5.981	3.111	6	3039	ZINC000118915219
16.0	-10.8	-10.4	0.21	2.075	0.406	4.967	2.232	6	3040	ZINC000100825537
16.0	-10.8	-10.3	0.27	2.211	0.278	5.467	2.452	6	3041	ZINC000257420544

16.0	-9.5	-9.2	0.21	2.056	0.872	4.282	2.798	6	3042	ZINC000257455858
16.0	-9.3	-9.0	0.22	2.406	0.133	8.172	2.574	6	3043	ZINC000100829452
15.9	-8.9	-7.8	0.80	2.500	0.186	7.948	0.632	6	3044	ZINC000100774814
15.9	-10.4	-10.1	0.22	2.262	0.546	8.439	1.608	6	3045	ZINC000257455946
15.9	-7.2	-5.7	0.72	1.454	0.090	7.142	1.165	6	3046	ZINC000100830088
15.9	-9.8	-9.6	0.13	2.368	0.538	7.078	1.670	6	3047	ZINC000257048100
15.9	-9.5	-9.2	0.19	3.437	0.300	4.678	0.540	6	3048	ZINC000014587247
15.9	-10.4	-10.1	0.22	2.190	0.537	7.215	2.592	6	3049	ZINC000100827939
15.9	-9.7	-9.4	0.18	2.247	0.251	6.948	2.893	6	3050	ZINC000256630153
15.9	-10.7	-10.3	0.22	2.769	0.572	4.998	1.340	6	3051	ZINC000257392668
15.9	-9.7	-9.4	0.20	2.237	0.258	6.938	2.893	6	3052	ZINC000100824040
15.9	-9.7	-9.5	0.16	1.919	0.461	6.639	3.291	6	3053	ZINC000256630157
15.9	-10.4	-10.1	0.22	2.057	0.352	7.182	2.510	6	3054	ZINC000257455945
15.9	-8.8	-8.6	0.14	1.855	0.474	5.655	3.894	6	3055	ZINC000014821369
15.9	-9.5	-9.2	0.22	3.651	0.440	5.721	2.052	6	3056	ZINC000257437435

15.9	-10.5	-10.1	0.30	2.274	0.540	4.021	1.117	6	3057	ZINC000252463742
15.9	-10.1	-10.0	0.07	2.550	0.336	4.211	2.007	6	3058	ZINC000257414578
15.9	-10.0	-9.7	0.17	2.425	0.344	6.239	1.796	6	3059	ZINC000100777731
15.9	-9.7	-9.3	0.20	2.220	0.731	7.064	2.951	6	3060	ZINC000100824043
15.9	-7.3	-5.7	0.80	1.521	0.140	7.256	1.154	6	3061	ZINC000257808242
15.9	-9.5	-9.2	0.18	3.492	0.318	4.718	0.545	6	3062	ZINC000014587249
15.9	-10.7	-10.2	0.25	1.867	0.310	5.859	2.401	6	3063	ZINC000096085883
15.9	-9.5	-9.2	0.23	3.674	0.439	5.795	2.022	6	3064	ZINC000014587248
15.9	-9.6	-9.4	0.19	2.064	0.361	4.969	3.036	6	3065	ZINC000095620795
15.9	-10.8	-10.3	0.30	2.432	0.273	4.142	0.696	6	3066	ZINC000256119454
15.9	-7.2	-5.7	0.72	1.464	0.094	7.145	1.154	6	3067	ZINC000100830086
15.9	-9.4	-9.2	0.17	1.525	0.385	2.588	0.485	6	3068	ZINC000257455860
15.9	-11.6	-11.1	0.29	1.569	0.138	3.431	2.134	6	3069	ZINC000095620011
15.9	-8.8	-8.6	0.11	2.801	0.690	6.319	2.909	6	3070	ZINC000257533756
15.9	-9.5	-9.2	0.19	3.428	0.314	4.599	0.555	6	3071	ZINC000257437433

15.9	-9.7	-9.2	0.28	2.655	0.513	7.503	1.916	6	3072	ZINC000256630158
15.9	-10.6	-10.4	0.18	2.914	0.611	4.952	1.755	6	3073	ZINC000013411949
15.9	-9.4	-9.2	0.21	2.323	0.836	4.404	2.758	6	3074	ZINC000014921636
15.9	-11.2	-10.5	0.38	2.341	0.162	4.420	0.466	6	3075	ZINC000100830071
15.9	-10.6	-10.1	0.31	2.393	0.291	4.084	0.769	6	3076	ZINC000100825591
15.9	-9.2	-8.9	0.16	2.009	0.346	8.488	3.176	6	3077	ZINC000003947503
15.9	-10.0	-9.9	0.10	2.178	0.143	6.147	2.346	6	3078	ZINC000031158164
15.9	-11.6	-11.0	0.30	2.193	0.150	6.188	1.286	6	3079	ZINC000003979028
15.9	-9.4	-9.0	0.25	1.934	0.561	5.390	2.997	6	3080	ZINC000100827304
15.8	-9.5	-9.1	0.23	3.709	0.528	6.412	2.472	6	3081	ZINC000257437434
15.8	-11.6	-10.4	0.83	2.610	0.671	6.059	2.147	6	3082	ZINC000100824455
15.8	-8.7	-8.6	0.05	2.612	0.276	7.730	2.203	6	3083	ZINC000014718950
15.8	-10.0	-9.9	0.08	2.187	0.228	4.388	1.749	6	3084	ZINC000014617841
15.8	-11.4	-10.9	0.39	2.092	0.332	6.066	1.664	6	3085	ZINC000003978827
15.8	-8.9	-8.8	0.11	2.424	0.584	6.480	2.794	6	3086	ZINC000059732365

15.8	-10.5	-10.1	0.28	1.650	0.266	5.053	2.420	6	3087	ZINC000003964578
15.8	-7.3	-5.8	0.74	1.460	0.091	7.144	1.166	6	3088	ZINC000257808241
15.8	-9.7	-9.4	0.20	2.386	0.530	7.295	2.578	6	3089	ZINC000256630150
15.8	-10.8	-10.6	0.18	2.173	0.534	5.854	3.590	6	3090	ZINC000257470886
15.8	-9.8	-9.2	0.33	2.340	0.427	5.416	2.552	6	3091	ZINC000100825593
15.8	-9.3	-9.1	0.16	1.678	0.474	2.868	0.523	6	3092	ZINC000014921634
15.8	-10.8	-10.6	0.17	1.953	0.523	7.330	3.830	6	3093	ZINC000100826065
15.8	-12.1	-11.2	0.43	2.483	0.262	6.384	2.190	6	3094	ZINC000100778889
15.8	-10.8	-10.4	0.31	2.375	0.279	6.584	3.293	6	3095	ZINC000100826070
15.8	-9.8	-9.7	0.18	2.039	0.539	5.520	2.699	6	3096	ZINC000100829950
15.8	-10.9	-10.6	0.24	1.769	0.239	5.416	2.165	6	3097	ZINC000253477224
15.8	-10.6	-9.2	0.69	3.616	0.329	5.918	1.855	6	3098	ZINC000100780967
15.8	-7.2	-5.7	0.75	1.456	0.090	7.137	1.157	6	3099	ZINC000100830085
15.8	-9.8	-9.5	0.30	2.221	0.236	7.902	1.823	6	3100	ZINC000100829954
15.8	-10.1	-9.2	0.41	2.901	0.431	9.832	0.531	6	3101	ZINC000257498009

15.8	-10.5	-10.2	0.26	1.699	0.257	4.211	1.885	6	3102	ZINC000100779057
15.8	-10.9	-10.6	0.18	1.793	0.437	4.775	2.216	6	3103	ZINC000013370664
15.8	-9.5	-9.2	0.15	3.504	0.356	4.631	0.551	6	3104	ZINC000014587250
15.8	-10.6	-9.8	0.44	2.565	0.205	4.142	0.503	6	3105	ZINC000253604919
15.8	-9.3	-9.2	0.14	1.959	0.950	4.083	2.955	6	3106	ZINC000014921639
15.8	-9.9	-9.5	0.32	1.995	0.653	4.194	2.130	6	3107	ZINC000100829951
15.8	-7.2	-5.7	0.72	1.484	0.094	7.137	1.187	6	3108	ZINC000100830089
15.8	-9.5	-9.2	0.18	3.535	0.438	5.500	2.134	6	3109	ZINC000257437432
15.8	-9.7	-9.4	0.20	1.914	0.467	6.652	3.288	6	3110	ZINC000100824044
15.8	-11.5	-11.1	0.23	1.738	0.198	5.812	1.711	6	3111	ZINC000095619517
15.8	-9.9	-9.5	0.35	2.441	0.587	6.008	1.870	6	3112	ZINC000256463121
15.8	-10.8	-10.6	0.22	2.191	0.537	7.274	3.767	6	3113	ZINC000257470885
15.7	-10.4	-10.2	0.35	2.534	0.789	7.604	3.474	6	3114	ZINC000118937231
15.7	-10.7	-10.5	0.09	2.538	0.356	5.968	2.406	6	3115	ZINC000257471474
15.7	-9.8	-9.6	0.27	2.182	0.314	6.846	2.948	6	3116	ZINC000100829953

15.7	-9.9	-9.7	0.16	2.341	0.511	7.067	2.390	6	3117	ZINC000257443453
15.7	-10.5	-10.3	0.28	2.419	0.267	6.109	1.993	6	3118	ZINC000257404250
15.7	-11.9	-11.1	0.40	2.249	0.359	5.490	1.662	6	3119	ZINC000100779567
15.7	-10.2	-9.8	0.20	2.397	0.073	6.672	2.487	6	3120	ZINC000253497557
15.7	-10.7	-10.5	0.12	2.208	0.505	5.783	2.562	6	3121	ZINC000257471472
15.7	-10.5	-10.2	0.21	2.044	0.239	6.703	1.409	6	3122	ZINC000100777904
15.7	-11.0	-10.8	0.09	1.464	0.078	4.636	2.360	6	3123	ZINC000100782741
15.7	-12.0	-10.7	0.69	2.252	0.620	5.194	2.889	6	3124	ZINC000140413066
15.7	-10.7	-10.5	0.09	2.542	0.352	6.925	2.482	6	3125	ZINC000100779382
15.7	-10.0	-9.8	0.15	1.953	0.451	5.200	2.346	6	3126	ZINC000100778813
15.7	-10.6	-9.9	0.60	2.200	0.679	5.780	3.183	6	3127	ZINC000257471998
15.7	-10.8	-10.4	0.24	2.164	0.405	6.276	2.243	6	3128	ZINC000257455712
15.7	-10.3	-10.0	0.27	1.892	0.279	4.030	1.724	6	3129	ZINC000257731811
15.7	-11.5	-10.7	0.41	2.196	0.158	5.429	1.524	6	3130	ZINC000085918237
15.7	-11.1	-10.9	0.12	2.160	0.743	5.530	2.866	6	3131	ZINC000257434590

15.7	-11.1	-10.4	0.37	2.191	0.300	6.234	1.847	6	3132	ZINC000014617789
15.7	-10.0	-9.7	0.18	2.090	0.317	6.189	2.185	6	3133	ZINC000100777728
15.7	-10.8	-10.5	0.14	1.819	0.277	5.852	2.147	6	3134	ZINC000100779514
15.7	-11.6	-10.5	0.62	2.020	0.514	5.057	1.989	6	3135	ZINC000100822997
15.7	-11.5	-10.7	0.74	2.777	0.582	5.192	2.192	6	3136	ZINC000257482004
15.7	-11.6	-10.7	0.52	2.139	0.269	7.036	0.769	6	3137	ZINC000024215070
15.7	-10.3	-9.9	0.31	2.509	0.405	4.779	1.959	6	3138	ZINC000253477157
15.7	-11.1	-10.6	0.26	1.931	0.262	4.976	2.308	6	3139	ZINC000014980278
15.7	-11.3	-10.7	0.30	2.361	0.500	3.533	1.231	6	3140	ZINC000085991684
15.7	-10.1	-9.6	0.32	2.229	0.553	4.153	2.888	6	3141	ZINC000015058161
15.7	-10.7	-10.5	0.09	2.499	0.403	6.906	2.522	6	3142	ZINC000100779385
15.7	-11.6	-10.6	0.45	1.920	0.191	5.884	2.671	6	3143	ZINC000257480080
15.7	-10.7	-10.5	0.09	2.493	0.413	6.896	2.535	6	3144	ZINC000257471471
15.7	-9.6	-9.4	0.15	2.228	0.246	6.950	2.913	6	3145	ZINC000100824042
15.7	-10.0	-9.8	0.21	2.423	0.434	7.644	1.580	6	3146	ZINC000014766828

15.6	-11.3	-10.7	0.32	2.550	0.680	6.358	2.913	6	3147	ZINC000100823641
15.6	-12.4	-11.5	0.46	2.641	0.656	7.164	1.389	6	3148	ZINC000257565514
15.6	-10.5	-10.3	0.10	1.763	0.557	6.429	1.898	6	3149	ZINC000257474269
15.6	-10.3	-10.1	0.25	2.239	0.292	5.621	1.741	6	3150	ZINC000256113612
15.6	-10.5	-10.2	0.16	1.490	0.440	4.321	2.888	6	3151	ZINC000257470887
15.6	-11.2	-10.1	0.60	1.931	0.358	5.459	2.436	6	3152	ZINC000100777880
15.6	-10.1	-9.2	0.85	2.475	0.000	5.549	0.000	2	3153	ZINC000085599273
15.6	-10.5	-10.2	0.25	1.734	0.538	5.958	3.370	6	3154	ZINC000100826068
15.6	-9.3	-8.9	0.22	2.381	0.602	6.740	3.087	6	3155	ZINC000257451581
15.6	-11.5	-10.6	0.53	1.807	0.322	5.071	1.730	6	3156	ZINC000085567736
15.6	-10.8	-9.7	0.54	1.812	0.380	4.543	2.461	6	3157	ZINC000257498578
15.6	-11.5	-10.9	0.30	2.190	0.223	4.195	1.537	6	3158	ZINC000257541114
15.6	-11.7	-11.2	0.37	3.038	0.415	5.102	1.460	6	3159	ZINC000256005423
15.6	-9.3	-9.1	0.19	1.823	0.126	3.322	0.871	6	3160	ZINC000100823235
15.6	-11.5	-10.9	0.36	1.874	0.300	6.868	1.832	6	3161	ZINC000257566875

15.6	-11.7	-11.2	0.25	1.913	0.353	6.629	1.961	6	3162	ZINC000014513017
15.6	-10.6	-9.5	0.53	2.551	0.311	7.084	2.627	6	3163	ZINC000100827894
15.6	-11.6	-10.9	0.37	2.241	0.854	5.530	2.813	6	3164	ZINC000005178688
15.6	-11.0	-10.2	0.37	1.887	0.361	6.779	1.887	6	3165	ZINC000100783638
15.6	-10.1	-9.9	0.15	1.639	0.213	7.044	1.756	6	3166	ZINC000257451380
15.6	-11.0	-10.6	0.29	2.207	0.919	4.234	2.284	6	3167	ZINC000100774349
15.6	-12.9	-11.7	0.59	1.707	0.284	4.615	2.166	6	3168	ZINC000049872295
15.5	-10.2	-9.7	0.26	2.207	0.340	3.701	0.647	6	3169	ZINC000100822014
15.5	-10.6	-9.9	0.46	2.105	0.453	5.272	3.440	6	3170	ZINC000257472001
15.5	-9.5	-9.3	0.12	2.597	0.213	6.316	2.612	6	3171	ZINC000256765741
15.5	-9.5	-9.3	0.11	2.517	0.155	6.186	2.695	6	3172	ZINC000100824035
15.5	-9.5	-9.3	0.12	2.549	0.191	6.242	2.650	6	3173	ZINC000256765755
15.5	-12.1	-10.9	0.57	3.371	0.601	6.081	1.029	6	3174	ZINC000257402587
15.5	-11.0	-10.5	0.34	1.752	0.395	6.968	2.100	6	3175	ZINC000257417468
15.5	-10.9	-10.0	0.39	2.074	0.287	6.156	2.356	6	3176	ZINC000257577145

15.5	-10.3	-9.9	0.25	2.022	0.648	4.042	1.800	6	3177	ZINC000257420543
15.5	-9.5	-9.3	0.10	2.564	0.151	5.268	2.256	6	3178	ZINC000100824036
15.5	-11.0	-10.8	0.13	1.980	0.370	5.594	1.764	6	3179	ZINC000013375699
15.5	-9.5	-9.3	0.11	2.556	0.139	6.200	2.656	6	3180	ZINC000100824038
15.5	-9.3	-9.1	0.15	2.527	1.005	4.355	2.761	6	3181	ZINC000015044249
15.5	-9.9	-9.6	0.15	2.169	0.463	5.976	2.216	6	3182	ZINC000257544501
15.5	-10.0	-9.4	0.42	2.349	0.157	4.576	0.874	6	3183	ZINC000253527793
15.5	-9.5	-9.3	0.26	2.240	0.268	6.852	2.947	6	3184	ZINC000256463116
15.5	-10.7	-10.5	0.18	2.454	0.250	6.393	1.713	6	3185	ZINC000004273446
15.5	-10.5	-10.2	0.23	1.882	0.420	5.954	3.373	6	3186	ZINC000100826069
15.5	-11.0	-10.7	0.21	2.211	0.172	6.715	1.392	6	3187	ZINC000100783763
15.5	-10.0	-9.7	0.21	2.329	0.613	5.898	3.556	6	3188	ZINC000257547797
15.5	-9.5	-9.3	0.11	2.676	0.290	7.253	2.787	6	3189	ZINC000256765731
15.5	-9.5	-9.3	0.10	2.517	0.174	6.148	2.711	6	3190	ZINC000100824039
15.5	-11.2	-10.6	0.47	2.788	0.558	5.547	1.228	6	3191	ZINC000014693307

15.4	-10.1	-9.8	0.19	2.618	0.509	8.670	2.340	6	3192	ZINC000100825582
15.4	-10.6	-9.5	0.52	2.610	0.315	8.390	2.101	6	3193	ZINC000257471999
15.4	-9.1	-8.4	0.56	2.246	0.655	4.864	2.764	6	3194	ZINC000014488273
15.4	-10.1	-9.8	0.18	2.757	0.343	8.623	2.320	6	3195	ZINC000100825580
15.4	-10.1	-9.7	0.26	2.326	0.287	6.507	2.191	6	3196	ZINC000257468174
15.4	-10.1	-9.8	0.16	2.657	0.496	9.794	0.327	6	3197	ZINC000257455853
15.4	-9.7	-9.4	0.15	2.215	0.247	5.213	2.280	6	3198	ZINC000257435430
15.4	-7.9	-7.1	0.86	1.863	1.211	4.389	1.070	6	3199	ZINC000150338646
15.4	-10.2	-10.0	0.10	1.934	0.550	4.704	2.629	6	3200	ZINC000100779927
15.4	-10.1	-9.8	0.16	2.618	0.507	8.666	2.339	6	3201	ZINC000238731015
15.4	-10.1	-9.8	0.19	2.626	0.515	8.673	2.342	6	3202	ZINC000100825585
15.4	-10.4	-10.0	0.31	2.009	0.332	4.863	2.281	6	3203	ZINC000257464992
15.4	-12.2	-11.4	0.42	1.726	0.108	4.859	1.663	6	3204	ZINC000095619534
15.4	-10.7	-10.3	0.28	1.869	0.762	3.971	3.112	6	3205	ZINC000100771770
15.4	-10.1	-9.8	0.21	2.732	0.483	9.818	0.323	6	3206	ZINC000257455852

15.4	-9.3	-9.2	0.07	2.127	0.411	3.193	0.570	6	3207	ZINC000257464900
15.4	-8.4	-8.3	0.07	1.971	0.573	6.885	3.714	6	3208	ZINC000015152837
15.4	-11.4	-10.9	0.35	2.117	0.537	4.541	2.736	6	3209	ZINC000100824454
15.4	-9.9	-9.0	0.54	2.842	0.305	6.654	1.356	6	3210	ZINC000257503210
15.4	-9.3	-9.2	0.13	2.457	1.004	4.401	2.712	6	3211	ZINC000257464899
15.4	-11.5	-10.8	0.33	1.733	0.118	5.433	2.262	6	3212	ZINC000257422805
15.4	-9.3	-9.0	0.18	2.450	1.004	4.284	2.750	6	3213	ZINC000015044245
15.4	-10.5	-10.2	0.33	2.578	0.526	5.292	2.334	6	3214	ZINC000257772036
15.4	-10.1	-9.8	0.16	2.483	0.643	8.345	2.957	6	3215	ZINC000100825583
15.4	-9.5	-9.2	0.30	2.184	0.320	6.779	3.026	6	3216	ZINC000256463127
15.4	-10.6	-10.0	0.51	2.053	0.354	4.881	2.521	6	3217	ZINC000100827900
15.4	-10.5	-10.0	0.28	1.685	0.176	6.934	1.647	6	3218	ZINC000257421470
15.4	-10.9	-10.2	0.42	2.198	0.272	6.448	1.498	6	3219	ZINC000006017976
15.4	-9.5	-9.3	0.12	2.639	0.849	5.788	3.423	6	3220	ZINC000014689159
15.4	-11.5	-11.0	0.38	2.801	0.567	6.977	2.469	6	3221	ZINC000257411678

15.4	-12.4	-11.5	0.41	2.310	0.688	6.334	2.563	6	3222	ZINC000100779622
15.4	-11.7	-11.1	0.60	1.639	0.987	7.135	2.948	6	3223	ZINC000100825768
15.4	-11.3	-10.7	0.31	1.761	0.162	5.659	2.381	6	3224	ZINC000100781829
15.3	-10.8	-10.3	0.26	2.060	0.570	7.403	0.394	6	3225	ZINC000253624626
15.3	-10.7	-10.1	0.31	2.559	0.255	6.534	2.912	6	3226	ZINC000100771773
15.3	-11.1	-10.5	0.27	1.563	0.203	4.980	2.658	6	3227	ZINC000014619923
15.3	-11.6	-10.9	0.32	2.505	0.423	5.332	1.808	6	3228	ZINC000095619173
15.3	-10.7	-10.3	0.25	2.174	0.570	5.247	2.649	6	3229	ZINC000253604949
15.3	-9.1	-8.9	0.11	2.044	0.494	7.592	2.700	6	3230	ZINC000257406749
15.3	-10.3	-10.2	0.07	2.006	0.675	5.133	2.585	6	3231	ZINC000257471473
15.3	-10.3	-10.1	0.15	2.391	0.489	6.856	2.874	6	3232	ZINC000100779383
15.3	-10.6	-10.1	0.41	2.232	0.356	4.959	2.174	6	3233	ZINC000100827897
15.3	-10.3	-10.2	0.09	2.238	0.414	6.783	2.072	6	3234	ZINC000100777944
15.3	-9.5	-9.3	0.11	2.594	0.879	5.724	3.467	6	3235	ZINC000238732497
15.3	-11.6	-11.1	0.39	1.899	0.387	4.529	1.729	6	3236	ZINC000095617886

15.3	-9.3	-9.1	0.12	2.263	0.253	2.979	0.109	6	3237	ZINC000059732353
15.3	-10.7	-10.2	0.29	2.270	0.471	4.509	2.790	6	3238	ZINC000253604950
15.3	-9.9	-9.6	0.14	1.456	0.145	2.616	0.979	6	3239	ZINC000059067035
15.3	-11.3	-10.5	0.56	2.430	0.234	6.231	0.707	6	3240	ZINC000230071361
15.3	-10.9	-10.7	0.15	1.844	0.490	3.813	2.032	6	3241	ZINC000095619512
15.3	-10.5	-10.2	0.13	1.946	0.399	4.160	2.165	6	3242	ZINC000257519432
15.3	-9.8	-9.5	0.16	1.920	0.269	5.154	3.335	6	3243	ZINC000100037755
15.3	-10.3	-10.0	0.21	2.283	0.640	3.836	0.945	6	3244	ZINC000257521781
15.3	-10.5	-10.0	0.36	1.911	0.256	6.680	2.250	6	3245	ZINC000100777587
15.3	-11.1	-10.6	0.35	2.498	0.575	5.261	2.734	6	3246	ZINC000085860881
15.3	-10.5	-10.2	0.20	2.063	0.335	3.887	1.183	6	3247	ZINC000257519433
15.3	-10.4	-9.7	0.41	2.420	0.323	7.997	0.436	6	3248	ZINC000257048097
15.3	-11.7	-10.7	0.52	2.139	0.663	5.390	1.964	6	3249	ZINC000085529962
15.3	-9.7	-9.5	0.20	2.981	0.554	4.938	2.377	6	3250	ZINC000100822578
15.3	-10.3	-9.8	0.34	1.769	0.351	4.163	3.265	6	3251	ZINC000100826079

15.3	-10.3	-10.0	0.22	2.585	0.299	5.073	1.870	6	3252	ZINC000100777804
15.3	-11.2	-10.7	0.30	2.101	0.609	5.367	2.771	6	3253	ZINC000257449429
15.3	-11.3	-11.0	0.29	2.087	0.616	5.429	2.471	6	3254	ZINC000100780309
15.3	-10.8	-10.4	0.23	3.174	0.591	6.364	1.423	6	3255	ZINC000257392667
15.3	-9.4	-9.2	0.11	2.115	0.349	7.731	3.501	6	3256	ZINC000085863150
15.3	-10.9	-10.5	0.22	2.077	0.347	5.025	1.937	6	3257	ZINC000015105339
15.3	-10.5	-10.2	0.15	1.992	0.336	4.620	1.889	6	3258	ZINC000100829475
15.3	-11.8	-11.5	0.16	2.261	0.328	6.577	1.505	6	3259	ZINC000257435966
15.2	-11.2	-10.3	0.56	2.183	0.711	4.318	2.311	6	3260	ZINC000015251989
15.2	-9.6	-9.3	0.23	2.352	0.683	4.587	2.931	6	3261	ZINC000014811813
15.2	-11.6	-10.7	0.50	2.160	0.198	7.191	1.329	6	3262	ZINC000257435964
15.2	-9.3	-8.9	0.27	2.193	0.369	5.062	2.039	6	3263	ZINC000014819524
15.2	-11.2	-10.4	0.41	2.330	0.296	6.374	2.683	6	3264	ZINC000100826028
15.2	-10.4	-10.1	0.16	2.279	0.264	4.632	1.694	6	3265	ZINC000031495353
15.2	-10.7	-10.4	0.20	1.992	0.459	3.760	1.281	6	3266	ZINC000100771774

15.2	-11.2	-10.7	0.29	2.322	0.489	4.531	2.259	6	3267	ZINC000257449430
15.2	-9.8	-9.5	0.22	2.103	0.238	6.567	2.137	6	3268	ZINC000100829403
15.2	-9.1	-8.9	0.13	2.064	0.462	3.227	0.479	6	3269	ZINC000238752624
15.2	-11.2	-10.7	0.26	2.247	0.601	5.477	2.663	6	3270	ZINC000100826029
15.2	-9.1	-8.9	0.16	2.404	0.971	4.369	2.506	6	3271	ZINC000015044252
15.2	-11.1	-10.7	0.27	1.758	0.772	4.828	0.590	6	3272	ZINC000085850634
15.2	-10.8	-10.3	0.27	1.755	0.150	5.870	2.317	6	3273	ZINC000257544499
15.2	-10.5	-9.9	0.33	1.756	0.367	4.625	2.664	6	3274	ZINC000257621035
15.2	-9.5	-9.3	0.11	2.522	0.408	6.785	3.242	6	3275	ZINC000256765746
15.2	-10.6	-9.9	0.40	2.056	0.731	4.262	2.730	6	3276	ZINC000257472000
15.2	-9.3	-9.2	0.07	2.554	0.334	3.426	0.557	6	3277	ZINC000257528224
15.2	-10.4	-10.2	0.20	2.080	0.352	4.618	1.476	6	3278	ZINC000257519434
15.2	-11.3	-10.4	0.40	2.318	0.111	4.503	1.597	6	3279	ZINC000100777600
15.2	-9.8	-9.5	0.20	2.082	0.200	6.139	0.915	6	3280	ZINC000253388216
15.2	-9.9	-9.2	0.34	2.275	0.640	5.495	3.740	6	3281	ZINC000014728225

15.2	-9.8	-9.3	0.22	3.191	0.421	6.564	3.061	6	3282	ZINC000100820462
15.2	-11.7	-11.3	0.27	1.855	0.452	4.862	2.018	6	3283	ZINC000257453497
15.2	-9.5	-9.2	0.17	1.655	0.386	4.120	2.400	6	3284	ZINC000257406750
15.2	-9.3	-9.1	0.09	2.796	0.710	3.704	0.953	6	3285	ZINC000257528225
15.2	-9.9	-9.2	0.35	2.274	0.649	5.492	3.720	6	3286	ZINC000257461968
15.2	-10.2	-9.3	0.54	3.096	0.226	6.017	0.192	6	3287	ZINC000150351593
15.2	-11.0	-10.4	0.34	2.743	0.391	5.023	1.343	6	3288	ZINC000015117343
15.2	-10.6	-10.2	0.23	2.068	0.376	6.027	3.111	6	3289	ZINC000257767190
15.2	-10.7	-10.5	0.11	2.551	0.533	6.810	2.837	6	3290	ZINC000257528537
15.2	-10.7	-9.9	0.47	2.846	0.394	6.635	2.076	6	3291	ZINC000095617638
15.2	-9.9	-9.0	0.46	2.360	0.606	4.487	2.893	6	3292	ZINC000014728224
15.2	-9.9	-9.3	0.34	2.148	0.753	5.273	3.901	6	3293	ZINC000257461970
15.2	-10.0	-9.6	0.22	1.915	0.346	6.994	1.950	6	3294	ZINC000257640757
15.2	-9.3	-9.2	0.06	3.052	0.357	4.201	0.524	6	3295	ZINC000014689164
15.1	-9.3	-9.1	0.15	2.252	0.343	5.740	2.270	6	3296	ZINC000100827305

15.1	-11.0	-10.3	0.35	2.368	0.599	6.427	1.457	6	3297	ZINC000014720322
15.1	-9.0	-8.8	0.20	2.547	0.713	4.819	2.624	6	3298	ZINC000015044242
15.1	-11.2	-10.9	0.27	2.156	0.361	6.154	1.949	6	3299	ZINC000257501771
15.1	-10.8	-10.6	0.12	1.953	0.325	7.598	2.032	6	3300	ZINC000100780840
15.1	-10.9	-10.6	0.14	1.640	0.224	4.869	2.394	6	3301	ZINC000257557432
15.1	-11.0	-10.6	0.27	2.640	0.252	6.783	1.168	6	3302	ZINC000256507448
15.1	-9.2	-8.9	0.21	2.450	0.897	8.485	3.185	6	3303	ZINC000257487565
15.1	-10.9	-10.4	0.35	2.076	0.055	5.830	3.116	6	3304	ZINC000257462123
15.1	-11.1	-10.6	0.24	2.224	0.644	5.476	2.669	6	3305	ZINC000100826026
15.1	-10.4	-10.1	0.21	2.248	0.257	4.361	0.652	6	3306	ZINC000256113618
15.1	-9.2	-9.1	0.09	3.172	0.417	5.245	2.144	6	3307	ZINC000014689160
15.1	-10.8	-9.4	0.66	1.695	0.409	5.637	1.748	6	3308	ZINC000257514756
15.1	-10.7	-10.5	0.16	2.785	0.579	5.171	0.984	6	3309	ZINC000013130935
15.1	-10.5	-10.0	0.31	2.388	0.410	6.325	3.058	6	3310	ZINC000253604948
15.1	-11.5	-10.8	0.43	2.185	0.358	5.026	1.246	6	3311	ZINC000014619926

15.1	-11.1	-10.5	0.28	1.777	0.142	5.708	2.409	6	3312	ZINC000257566876
15.1	-9.6	-9.3	0.23	2.626	1.024	4.633	2.285	6	3313	ZINC000004261846
15.1	-11.4	-11.1	0.17	2.371	0.339	6.021	1.891	6	3314	ZINC000261496025
15.1	-9.7	-9.2	0.23	2.476	0.353	7.202	3.351	6	3315	ZINC000257472649
15.1	-9.2	-9.0	0.10	3.067	0.401	4.156	0.548	6	3316	ZINC000257528226
15.1	-9.5	-9.3	0.18	2.381	1.085	4.411	2.881	6	3317	ZINC000257440477
15.1	-11.6	-11.1	0.38	2.665	0.242	6.419	1.503	6	3318	ZINC000085648920
15.1	-9.6	-9.2	0.31	1.436	0.604	2.487	0.944	6	3319	ZINC000100822186
15.1	-10.9	-10.3	0.33	1.864	0.231	6.418	2.260	6	3320	ZINC000261498230
15.1	-10.5	-10.0	0.28	1.530	0.773	4.682	2.837	6	3321	ZINC000257517851
15.1	-11.5	-11.0	0.37	2.071	0.579	5.953	2.382	6	3322	ZINC000257407331
15.1	-11.0	-10.5	0.28	1.842	0.183	6.116	1.909	6	3323	ZINC000257514658
15.1	-9.9	-9.2	0.39	2.309	0.665	4.443	2.934	6	3324	ZINC000014728226
15.1	-9.9	-9.2	0.35	2.258	0.652	5.451	3.762	6	3325	ZINC000014728227
15.1	-9.3	-9.2	0.07	2.999	0.286	4.317	0.572	6	3326	ZINC000014689162

15.1	-9.7	-9.4	0.23	2.733	0.717	5.028	2.315	6	3327	ZINC000014806833
15.1	-10.7	-10.4	0.18	2.069	0.449	4.213	1.228	6	3328	ZINC000100771769
15.1	-10.9	-10.3	0.30	2.546	0.273	7.782	1.876	6	3329	ZINC000014617790
15.1	-9.4	-9.3	0.11	1.979	0.445	2.832	0.686	6	3330	ZINC000257440476
15.1	-8.9	-8.7	0.16	2.287	0.545	6.105	3.550	6	3331	ZINC000003947504
15.1	-10.8	-10.3	0.33	1.737	0.172	4.818	2.574	6	3332	ZINC000257486645
15.1	-9.5	-9.1	0.33	1.463	0.545	2.585	0.877	6	3333	ZINC000257408776
15.1	-9.0	-8.9	0.13	2.124	0.534	3.235	0.589	6	3334	ZINC000257464898
15.0	-11.0	-10.0	0.49	2.636	0.696	6.001	2.521	6	3335	ZINC000150351592
15.0	-10.5	-10.1	0.23	1.926	0.170	2.790	0.336	6	3336	ZINC000015106720
15.0	-9.5	-9.0	0.39	1.832	0.579	3.339	1.205	6	3337	ZINC000100822188
15.0	-9.5	-9.2	0.26	1.665	0.638	2.994	1.492	6	3338	ZINC000257408775
15.0	-9.5	-9.2	0.24	1.588	0.475	2.920	1.157	6	3339	ZINC000100822187
15.0	-9.8	-9.5	0.20	2.030	0.217	6.239	0.928	6	3340	ZINC000257550497
15.0	-9.3	-9.1	0.14	3.155	0.645	7.984	2.874	6	3341	ZINC000014645009

15.0	-9.8	-9.5	0.20	2.079	0.208	6.138	0.916	6	3342	ZINC000100825674
15.0	-10.3	-9.9	0.24	1.725	0.625	4.046	1.368	6	3343	ZINC000257521778
15.0	-12.1	-11.6	0.29	2.603	0.602	6.699	2.277	6	3344	ZINC000086041820
15.0	-10.8	-9.6	0.66	2.339	0.536	6.451	2.432	6	3345	ZINC000085926503
15.0	-9.3	-9.0	0.18	3.494	0.534	5.371	1.888	6	3346	ZINC000000898308
15.0	-10.0	-9.5	0.24	1.986	0.285	6.383	2.098	6	3347	ZINC000100777576
15.0	-11.0	-10.1	0.52	2.710	0.670	6.842	0.830	6	3348	ZINC000085862054
15.0	-9.9	-9.4	0.39	2.145	0.236	3.469	0.807	6	3349	ZINC000100774084
15.0	-9.8	-9.3	0.27	2.416	0.399	4.335	0.948	6	3350	ZINC000100825597
15.0	-7.8	-7.3	0.38	1.951	0.649	5.862	4.774	6	3351	ZINC000014594127
15.0	-10.4	-9.3	0.68	3.161	0.451	6.849	2.036	6	3352	ZINC000060183860
15.0	-12.5	-11.0	0.79	2.550	0.433	6.888	2.608	6	3353	ZINC000257480592
15.0	-11.2	-10.8	0.21	1.799	0.317	7.418	0.180	6	3354	ZINC000100823670
15.0	-9.0	-8.8	0.16	2.628	0.522	7.148	2.985	6	3355	ZINC000100827302
15.0	-11.0	-10.2	0.38	1.824	0.456	5.575	1.990	6	3356	ZINC000257461491

15.0	-9.4	-9.2	0.25	2.418	0.427	6.705	2.707	6	3357	ZINC000085946004
15.0	-10.8	-10.4	0.26	2.191	0.914	3.843	2.174	6	3358	ZINC000014617181
15.0	-11.2	-10.5	0.43	2.620	0.598	6.780	1.043	6	3359	ZINC000257480398
15.0	-11.6	-10.6	0.46	1.816	0.341	4.796	2.542	6	3360	ZINC000095620012
15.0	-12.6	-10.8	0.83	2.826	0.492	7.120	0.948	6	3361	ZINC000027766588
15.0	-9.5	-9.3	0.22	1.845	0.767	3.693	1.816	6	3362	ZINC000257408774
15.0	-11.4	-11.0	0.21	2.318	0.373	5.805	1.768	6	3363	ZINC000014720194
15.0	-10.0	-9.3	0.33	2.180	0.409	4.693	2.948	6	3364	ZINC000014645013
15.0	-12.4	-11.6	0.40	2.300	0.713	6.316	2.602	6	3365	ZINC000257565513
14.9	-10.7	-9.9	0.45	2.317	0.448	6.716	3.283	6	3366	ZINC000095619583
14.9	-11.0	-10.5	0.33	1.749	0.349	5.959	2.170	6	3367	ZINC000095620004
14.9	-10.3	-9.9	0.27	2.395	0.822	4.277	2.462	6	3368	ZINC000100773815
14.9	-10.9	-10.8	0.09	1.794	0.481	4.809	2.506	6	3369	ZINC000100779425
14.9	-9.6	-9.1	0.28	1.648	0.366	5.816	3.919	6	3370	ZINC000015104408
14.9	-10.4	-10.2	0.27	2.793	0.706	8.308	2.966	6	3371	ZINC000118937229

14.9	-7.9	-6.8	1.01	2.528	0.888	4.975	1.930	5	3372	ZINC000257515480
14.9	-10.9	-10.7	0.15	1.820	0.381	5.698	2.351	6	3373	ZINC000015105341
14.9	-9.3	-9.2	0.06	2.882	0.730	3.967	1.018	6	3374	ZINC000014587230
14.9	-11.3	-10.3	0.51	1.783	0.261	6.413	1.916	6	3375	ZINC000100782679
14.9	-10.9	-10.3	0.26	1.976	0.377	5.052	2.151	6	3376	ZINC000015117347
14.9	-8.4	-7.3	0.84	2.754	0.779	7.332	1.330	4	3377	ZINC000100116841
14.9	-10.4	-10.2	0.13	1.745	0.152	5.039	2.008	6	3378	ZINC000257456727
14.9	-11.0	-10.9	0.13	1.769	0.183	4.470	1.519	6	3379	ZINC000100825211
14.9	-9.2	-9.0	0.16	3.540	0.514	5.442	1.872	6	3380	ZINC000003604297
14.9	-10.6	-10.3	0.26	2.091	0.736	4.824	2.545	6	3381	ZINC000257435390
14.9	-9.5	-9.2	0.23	1.942	0.616	3.787	1.413	6	3382	ZINC000257408777
14.9	-12.7	-11.1	0.88	3.095	0.665	7.191	1.328	6	3383	ZINC000137735231
14.9	-9.7	-9.4	0.16	2.062	0.236	5.876	0.558	6	3384	ZINC000100825527
14.9	-10.5	-10.0	0.36	2.173	0.411	6.070	2.472	6	3385	ZINC000257377832
14.9	-9.3	-9.2	0.06	1.691	0.509	7.211	3.783	6	3386	ZINC000100825870

14.9	-11.0	-10.7	0.20	2.366	0.814	5.280	2.692	6	3387	ZINC000100783682
14.9	-9.5	-9.1	0.27	2.046	0.535	3.847	1.382	6	3388	ZINC000100822190
14.9	-10.4	-10.1	0.25	2.304	0.188	6.487	2.121	6	3389	ZINC000100256144
14.9	-11.3	-10.8	0.33	1.697	0.361	5.523	2.615	6	3390	ZINC000003978797
14.9	-9.3	-8.7	0.29	1.810	0.323	5.874	3.930	6	3391	ZINC000070455478
14.9	-10.1	-9.8	0.22	1.815	0.378	5.887	3.839	6	3392	ZINC000257624275
14.9	-8.9	-8.6	0.21	2.079	0.673	4.351	2.789	6	3393	ZINC000014644026
14.9	-10.3	-9.8	0.30	2.416	0.191	4.939	2.101	6	3394	ZINC000256113615
14.9	-9.3	-9.0	0.18	3.180	0.350	4.467	0.332	6	3395	ZINC000014587229
14.9	-10.3	-10.0	0.29	2.157	0.330	5.535	1.778	6	3396	ZINC000008143604
14.8	-9.9	-9.3	0.28	2.288	0.763	5.673	3.593	6	3397	ZINC000257538548
14.8	-11.2	-10.9	0.21	2.207	0.410	5.873	2.236	6	3398	ZINC000100777650
14.8	-10.7	-10.3	0.34	2.208	0.258	4.634	2.077	6	3399	ZINC000257505905
14.8	-11.0	-10.8	0.26	1.667	0.336	4.842	2.125	6	3400	ZINC000100825212
14.8	-11.6	-11.0	0.30	1.988	0.126	8.146	0.228	6	3401	ZINC000049872350

14.8	-11.4	-11.1	0.18	2.272	0.414	7.004	1.655	6	3402	ZINC000150361517
14.8	-10.9	-10.3	0.45	2.725	1.002	5.864	3.279	6	3403	ZINC000257498921
14.8	-11.0	-10.6	0.41	1.873	0.315	4.822	2.003	6	3404	ZINC000100822825
14.8	-12.4	-11.5	0.41	2.315	0.690	6.337	2.562	6	3405	ZINC000100779624
14.8	-10.3	-9.9	0.19	2.044	0.254	5.996	2.346	6	3406	ZINC000257470618
14.8	-9.2	-9.0	0.25	2.461	0.980	5.880	3.304	6	3407	ZINC000014587227
14.8	-10.2	-9.4	0.51	2.615	0.552	6.659	2.696	6	3408	ZINC000257419524
14.8	-10.7	-10.1	0.31	1.823	0.312	5.768	2.827	6	3409	ZINC000086006924
14.8	-9.2	-9.0	0.15	3.553	0.512	5.450	1.883	6	3410	ZINC000014587228
14.8	-11.9	-11.0	0.49	1.889	0.525	5.802	2.155	6	3411	ZINC000100825780
14.8	-11.8	-11.1	0.38	2.155	0.297	5.737	2.226	6	3412	ZINC000015105351
14.8	-12.2	-12.0	0.15	2.130	0.354	6.802	2.726	6	3413	ZINC000024215043
14.8	-10.9	-10.6	0.20	1.937	0.183	5.895	2.153	6	3414	ZINC000257422802
14.8	-10.6	-10.0	0.29	1.953	0.412	5.841	2.159	6	3415	ZINC000257387716
14.8	-8.8	-8.6	0.12	2.068	0.444	4.471	3.089	6	3416	ZINC000014594131

14.8	-10.8	-10.3	0.42	2.655	0.311	6.662	2.386	6	3417	ZINC000257449432
14.8	-9.6	-9.4	0.21	2.889	0.868	5.855	2.859	6	3418	ZINC000100822580
14.8	-8.9	-8.5	0.30	2.616	0.306	7.625	3.235	6	3419	ZINC000011666970
14.8	-10.1	-9.5	0.37	2.186	0.204	7.214	1.976	6	3420	ZINC000257470617
14.8	-11.7	-11.3	0.24	1.625	0.146	5.860	1.881	6	3421	ZINC000100779561
14.8	-10.8	-10.3	0.37	1.613	0.148	4.780	2.720	6	3422	ZINC000248328867
14.8	-11.1	-10.7	0.19	2.491	0.412	6.481	1.542	6	3423	ZINC000005518250
14.8	-10.4	-10.2	0.16	1.895	0.347	3.610	2.120	6	3424	ZINC000257435387
14.8	-7.5	-7.3	0.26	1.278	0.147	1.761	0.239	5	3425	ZINC000014594129
14.8	-10.7	-10.3	0.21	2.958	0.881	8.306	3.331	6	3426	ZINC000257546980
14.8	-10.4	-9.9	0.41	2.339	0.432	7.308	1.996	6	3427	ZINC000118937029
14.7	-10.5	-9.4	0.55	3.536	0.444	5.806	1.942	6	3428	ZINC000100780973
14.7	-9.9	-9.8	0.07	2.122	0.476	5.568	2.593	6	3429	ZINC000100779173
14.7	-9.9	-9.5	0.21	1.945	0.331	5.902	2.247	6	3430	ZINC000100777567
14.7	-10.4	-10.2	0.21	2.123	0.306	4.449	0.708	6	3431	ZINC000095099274

14.7	-10.3	-9.7	0.30	1.771	0.508	5.771	2.745	6	3432	ZINC000257443452
14.7	-11.3	-10.7	0.34	1.667	0.486	4.912	2.950	6	3433	ZINC000100773678
14.7	-10.0	-9.6	0.21	2.181	0.472	5.702	2.590	6	3434	ZINC000257567513
14.7	-11.0	-10.3	0.45	2.175	0.479	5.554	2.089	6	3435	ZINC000100777808
14.7	-11.2	-10.7	0.27	2.685	0.240	7.498	2.228	6	3436	ZINC000257455120
14.7	-10.7	-10.6	0.08	2.424	0.502	6.089	2.617	6	3437	ZINC000100830018
14.7	-10.8	-10.6	0.11	2.369	0.307	5.074	2.128	6	3438	ZINC000257744787
14.7	-10.1	-10.0	0.10	2.449	0.470	6.578	2.919	6	3439	ZINC000257528540
14.7	-9.9	-9.5	0.22	2.156	0.329	3.415	0.601	6	3440	ZINC000100777569
14.7	-9.1	-8.8	0.23	2.677	0.678	7.616	3.161	6	3441	ZINC000002065942
14.7	-10.5	-10.1	0.25	1.912	0.158	7.048	0.937	6	3442	ZINC000100772239
14.7	-10.5	-10.2	0.17	1.729	0.563	5.962	3.375	6	3443	ZINC000257470888
14.7	-9.9	-9.4	0.28	2.128	0.721	4.132	2.869	6	3444	ZINC000257538546
14.7	-11.9	-10.7	0.76	1.896	0.457	6.114	2.136	6	3445	ZINC000100825764
14.7	-9.9	-9.7	0.19	2.216	0.347	5.576	1.765	6	3446	ZINC000100825362

14.7	-11.2	-10.9	0.21	2.335	0.457	5.439	1.910	6	3447	ZINC000100779191
14.7	-10.4	-10.3	0.10	1.889	0.291	5.912	2.135	6	3448	ZINC000257557433
14.7	-11.5	-10.8	0.49	2.094	0.494	4.399	1.999	6	3449	ZINC000100780833
14.7	-9.6	-9.3	0.26	1.955	0.417	7.128	3.312	6	3450	ZINC000100826048
14.7	-9.8	-9.4	0.24	1.914	0.322	2.827	0.442	6	3451	ZINC000015058167
14.7	-8.9	-8.5	0.28	2.083	0.456	7.831	1.777	6	3452	ZINC000086047375
14.7	-11.5	-10.8	0.41	2.626	0.588	6.459	2.301	6	3453	ZINC000100780298
14.7	-10.0	-9.7	0.24	2.372	0.495	5.583	2.393	6	3454	ZINC000257415728
14.7	-9.9	-9.3	0.35	1.897	0.434	5.852	4.016	6	3455	ZINC000257547796
14.7	-9.9	-9.2	0.40	2.227	0.502	4.407	2.769	6	3456	ZINC000012153679
14.7	-10.8	-10.5	0.20	2.096	0.432	4.214	2.161	6	3457	ZINC000118937236
14.7	-11.6	-11.0	0.28	1.879	0.448	5.114	2.866	6	3458	ZINC000027758239
14.7	-11.8	-11.6	0.19	1.900	0.424	5.067	1.948	6	3459	ZINC000004098620
14.6	-8.1	-7.7	0.24	2.276	0.226	8.492	4.310	6	3460	ZINC000014594126
14.6	-11.7	-11.0	0.44	1.945	0.256	4.737	2.165	6	3461	ZINC000014617363

14.6	-10.5	-10.0	0.33	1.888	0.175	7.025	0.934	6	3462	ZINC000100772243
14.6	-10.1	-9.9	0.09	2.284	0.613	5.975	2.913	6	3463	ZINC000257528538
14.6	-10.3	-10.2	0.11	1.736	0.222	5.871	2.188	6	3464	ZINC000257531616
14.6	-9.6	-9.2	0.26	2.106	0.417	8.394	2.758	6	3465	ZINC000100826050
14.6	-10.3	-9.9	0.26	2.043	0.357	6.098	2.496	6	3466	ZINC000257451382
14.6	-11.3	-10.5	0.39	1.786	0.142	4.894	2.085	6	3467	ZINC000100781613
14.6	-11.3	-11.0	0.30	1.648	0.378	4.602	2.432	6	3468	ZINC000257466732
14.6	-10.2	-10.0	0.16	2.957	1.015	7.590	3.446	6	3469	ZINC000118937232
14.6	-10.1	-10.0	0.07	2.763	0.498	7.851	2.246	6	3470	ZINC000100830015
14.6	-10.7	-10.1	0.33	2.635	0.183	6.761	1.544	6	3471	ZINC000257462122
14.6	-11.0	-10.9	0.16	1.743	0.174	4.903	1.314	6	3472	ZINC000100825207
14.6	-10.0	-9.9	0.12	2.042	0.454	4.855	2.410	6	3473	ZINC000100777602
14.6	-9.8	-9.5	0.23	1.910	0.349	2.882	0.426	6	3474	ZINC000015058165
14.6	-9.7	-9.4	0.23	1.677	0.205	5.489	3.528	6	3475	ZINC000238749139
14.6	-10.1	-10.0	0.06	2.425	0.546	5.890	2.163	6	3476	ZINC000257528539

14.6	-11.5	-10.7	0.57	2.373	0.356	5.871	1.438	6	3477	ZINC000257385902
14.6	-11.0	-10.9	0.14	1.920	0.373	5.786	2.280	6	3478	ZINC000257793282
14.6	-9.7	-9.4	0.23	2.105	0.507	5.879	3.342	6	3479	ZINC000238770157
14.6	-10.0	-9.5	0.29	1.954	0.460	7.348	3.637	6	3480	ZINC000095620831
14.6	-8.5	-7.8	0.46	1.913	0.728	6.365	4.570	6	3481	ZINC000014594128
14.6	-10.7	-10.2	0.26	2.096	0.239	6.267	1.717	6	3482	ZINC000100777940
14.6	-9.8	-9.3	0.34	1.504	0.363	5.747	3.504	6	3483	ZINC000014807638
14.6	-10.8	-10.6	0.22	1.819	0.254	4.909	0.329	6	3484	ZINC000257793281
14.6	-11.0	-10.8	0.20	1.854	0.278	4.422	1.435	6	3485	ZINC000100825209
14.6	-9.9	-9.7	0.17	2.085	0.226	5.520	2.210	6	3486	ZINC000100777578
14.5	-11.1	-10.5	0.36	2.505	0.232	5.660	1.911	6	3487	ZINC000085954773
14.5	-10.4	-9.8	0.37	2.005	0.294	4.320	1.712	6	3488	ZINC000100777559
14.5	-10.8	-10.5	0.15	1.902	0.354	5.528	2.465	6	3489	ZINC000100783769
14.5	-10.9	-10.7	0.17	1.778	0.256	4.391	1.598	6	3490	ZINC000257793279
14.5	-9.6	-9.3	0.22	1.943	0.399	5.659	3.547	6	3491	ZINC000257566338

14.5	-11.5	-10.8	0.42	2.162	0.507	4.754	2.297	6	3492	ZINC000257472502
14.5	-9.3	-9.0	0.29	2.007	0.286	3.861	1.125	6	3493	ZINC000257373614
14.5	-10.4	-10.2	0.12	2.823	1.152	6.082	2.142	6	3494	ZINC000100829691
14.5	-10.7	-10.1	0.30	2.387	0.308	5.952	2.124	6	3495	ZINC000100773897
14.5	-10.0	-9.7	0.18	1.978	0.418	5.201	2.109	6	3496	ZINC000242543353
14.5	-9.6	-9.3	0.28	1.771	0.169	7.014	3.218	6	3497	ZINC000257566337
14.5	-8.6	-8.1	0.48	2.384	1.098	5.451	2.425	6	3498	ZINC000004235524
14.5	-9.6	-9.4	0.19	1.951	0.423	5.801	3.239	6	3499	ZINC000100826052
14.5	-10.3	-9.9	0.21	2.240	0.386	4.988	1.498	6	3500	ZINC000257421469
14.5	-9.6	-9.3	0.21	1.775	0.154	4.275	1.876	6	3501	ZINC000100829407
14.5	-9.6	-9.3	0.26	1.990	0.486	7.293	3.447	6	3502	ZINC000100826046
14.5	-11.8	-10.8	0.52	2.068	0.583	3.840	2.023	6	3503	ZINC000100779212
14.5	-10.9	-10.7	0.13	1.877	0.274	6.784	1.410	6	3504	ZINC000014720333
14.5	-9.8	-9.5	0.23	2.356	0.897	6.049	3.508	6	3505	ZINC000100771910
14.5	-10.6	-10.4	0.12	2.007	0.124	6.700	1.337	6	3506	ZINC000014617362

14.5	-10.1	-10.0	0.09	2.327	0.465	6.090	2.553	6	3507	ZINC000100830017
14.5	-10.7	-10.4	0.17	2.594	0.224	5.279	1.772	6	3508	ZINC000100299712
14.5	-10.9	-10.4	0.31	2.542	0.469	6.074	1.136	6	3509	ZINC000013130933
14.5	-9.8	-9.5	0.19	3.158	1.039	4.529	1.406	6	3510	ZINC000013379901
14.5	-11.1	-10.7	0.33	1.766	0.195	6.612	1.664	6	3511	ZINC000100773683
14.5	-10.7	-10.4	0.23	2.562	1.281	5.043	1.903	6	3512	ZINC000027755888
14.5	-10.7	-10.3	0.22	1.810	0.342	6.214	2.187	6	3513	ZINC000257422804
14.5	-10.1	-10.0	0.07	2.428	0.534	7.036	2.410	6	3514	ZINC000100830020
14.5	-10.5	-10.0	0.24	1.885	0.158	5.947	2.140	6	3515	ZINC000100777908
14.4	-10.3	-9.8	0.27	2.271	0.415	5.016	2.322	6	3516	ZINC000257382701
14.4	-10.0	-9.7	0.16	1.881	0.164	4.017	2.421	6	3517	ZINC000257388121
14.4	-9.6	-9.4	0.17	3.002	0.693	7.310	2.282	6	3518	ZINC000100773806
14.4	-11.0	-10.5	0.31	2.818	0.609	6.566	1.968	6	3519	ZINC000100783677
14.4	-10.8	-10.4	0.24	2.078	0.464	5.800	2.607	6	3520	ZINC000257488044
14.4	-10.1	-9.6	0.31	2.090	0.398	4.191	2.983	6	3521	ZINC000015058160

14.4	-10.0	-9.3	0.44	2.127	0.686	6.260	3.405	6	3522	ZINC000257573396
14.4	-10.9	-10.1	0.51	2.802	0.614	5.150	0.768	6	3523	ZINC000085648924
14.4	-10.8	-10.6	0.15	1.997	0.348	5.251	1.683	6	3524	ZINC000257793280
14.4	-10.2	-10.0	0.10	1.647	0.217	4.960	2.386	6	3525	ZINC000257668334
14.4	-10.1	-9.6	0.33	2.343	0.601	5.589	3.617	6	3526	ZINC000013322028
14.4	-10.0	-9.8	0.11	2.037	0.265	5.530	3.068	6	3527	ZINC000100829154
14.4	-10.0	-9.7	0.18	1.935	0.151	5.431	2.911	6	3528	ZINC000257388123
14.4	-10.0	-9.8	0.12	1.970	0.282	5.350	3.248	6	3529	ZINC000257388124
14.4	-10.0	-9.7	0.16	2.066	0.239	5.548	3.055	6	3530	ZINC000100829153
14.4	-11.5	-11.2	0.20	2.107	0.388	5.164	1.893	6	3531	ZINC000014720215
14.4	-9.4	-9.2	0.11	2.227	0.444	3.844	0.937	6	3532	ZINC000059067044
14.4	-11.5	-10.7	0.51	2.162	0.456	4.860	1.384	6	3533	ZINC000257744792
14.4	-10.3	-10.1	0.12	2.088	0.314	5.282	1.969	6	3534	ZINC000100829405
14.4	-10.7	-10.2	0.23	2.379	0.175	6.472	1.502	6	3535	ZINC000100824375
14.4	-8.9	-8.5	0.24	1.904	0.204	6.973	3.704	6	3536	ZINC000257544402

14.4	-11.1	-10.1	0.54	1.826	0.192	5.926	3.095	6	3537	ZINC000150368423
14.4	-12.3	-11.7	0.43	2.618	0.528	5.362	1.014	6	3538	ZINC000257488509
14.4	-10.1	-9.7	0.26	1.895	0.221	2.543	0.560	6	3539	ZINC000015058162
14.4	-10.0	-9.8	0.12	1.973	0.296	5.338	3.248	6	3540	ZINC000257388122
14.3	-10.2	-9.7	0.28	2.052	0.356	5.095	2.260	6	3541	ZINC000257546995
14.3	-11.5	-11.1	0.23	1.751	0.454	4.672	2.786	6	3542	ZINC000257395111
14.3	-10.7	-10.5	0.16	1.958	0.387	7.870	0.075	6	3543	ZINC000257417466
14.3	-8.7	-8.5	0.11	2.971	0.367	4.392	0.727	6	3544	ZINC000014594130
14.3	-9.7	-8.8	0.45	1.518	0.284	3.149	1.061	6	3545	ZINC000100827647
14.3	-9.9	-9.7	0.13	2.083	0.232	6.791	3.073	6	3546	ZINC000100829151
14.3	-10.1	-9.5	0.31	2.201	0.581	4.245	2.837	6	3547	ZINC000257498010
14.3	-11.6	-11.2	0.28	1.662	0.330	7.709	0.112	6	3548	ZINC000095620005
14.3	-8.6	-8.4	0.09	2.189	1.011	5.524	3.343	6	3549	ZINC000257452004
14.3	-10.8	-10.4	0.29	1.701	0.193	5.710	2.372	6	3550	ZINC000100777616
14.3	-11.3	-10.9	0.32	2.056	0.203	6.616	2.127	6	3551	ZINC000257478363

14.3	-10.6	-10.1	0.28	2.129	0.218	4.767	1.882	6	3552	ZINC000257435429
14.3	-9.2	-9.0	0.22	2.140	0.606	5.902	3.309	6	3553	ZINC000014811764
14.3	-11.0	-10.4	0.34	2.377	0.287	6.859	1.807	6	3554	ZINC000013414448
14.3	-10.1	-9.6	0.31	2.093	0.395	4.140	3.017	6	3555	ZINC000015058163
14.3	-11.2	-10.5	0.39	1.887	0.257	5.973	1.810	6	3556	ZINC000100777797
14.3	-10.3	-10.0	0.21	2.121	0.674	4.178	1.025	6	3557	ZINC000257521779
14.3	-10.3	-10.1	0.12	2.215	0.487	5.050	2.548	6	3558	ZINC000257444397
14.3	-10.2	-9.7	0.32	2.112	0.385	6.083	2.466	6	3559	ZINC000257415725
14.3	-10.9	-10.6	0.17	1.942	0.463	5.052	2.067	6	3560	ZINC000014858850
14.3	-10.6	-10.1	0.26	2.479	0.401	6.947	3.296	6	3561	ZINC000086033455
14.3	-10.8	-10.4	0.27	1.670	0.313	5.877	2.207	6	3562	ZINC000014720335
14.3	-10.4	-10.0	0.29	2.305	0.622	3.796	0.927	6	3563	ZINC000100821981
14.3	-10.1	-9.5	0.39	1.980	0.123	3.882	2.488	6	3564	ZINC000257498008
14.3	-11.2	-10.4	0.37	2.156	0.748	6.048	2.823	6	3565	ZINC000003197535
14.3	-10.2	-8.8	0.65	2.216	0.335	4.271	1.030	6	3566	ZINC000085853640

14.3	-11.4	-10.6	0.39	2.093	0.351	4.900	1.901	6	3567	ZINC000257541116
14.3	-10.4	-10.0	0.24	2.027	0.299	6.289	1.637	6	3568	ZINC000257421472
14.3	-9.0	-8.8	0.15	2.336	0.590	4.124	2.345	6	3569	ZINC000257563724
14.3	-10.0	-9.8	0.15	2.026	0.264	5.548	3.054	6	3570	ZINC000100829150
14.3	-12.4	-11.1	0.67	2.371	0.581	5.969	2.363	6	3571	ZINC000200417803
14.3	-9.3	-9.2	0.11	1.900	0.423	5.717	3.344	6	3572	ZINC000013132549
14.2	-10.8	-10.4	0.20	1.787	0.068	5.590	1.932	6	3573	ZINC000013335894
14.2	-10.1	-9.8	0.23	2.198	0.389	7.202	1.591	6	3574	ZINC000257470619
14.2	-11.5	-10.8	0.41	2.201	0.651	5.846	2.340	6	3575	ZINC000014610659
14.2	-13.1	-12.5	0.33	2.498	0.485	6.345	0.937	6	3576	ZINC000257427004
14.2	-11.6	-10.9	0.44	1.841	0.335	5.346	2.527	6	3577	ZINC000257486646
14.2	-10.5	-10.4	0.07	1.807	0.573	3.600	2.416	6	3578	ZINC000095619504
14.2	-10.6	-10.2	0.21	1.785	0.275	5.996	2.323	6	3579	ZINC000085531644
14.2	-10.9	-10.7	0.13	2.324	0.506	5.409	2.465	6	3580	ZINC000256102635
14.2	-11.3	-10.5	0.56	2.769	0.364	6.753	1.110	6	3581	ZINC000026490620

14.2	-10.7	-10.2	0.36	1.849	0.358	4.941	2.391	6	3582	ZINC000014243549
14.2	-9.4	-8.4	0.95	1.988	0.000	7.117	0.000	2	3583	ZINC000100828905
14.2	-10.1	-9.9	0.12	1.771	0.427	5.775	1.898	6	3584	ZINC000100779320
14.2	-11.7	-11.2	0.37	2.213	0.528	4.072	1.470	6	3585	ZINC000100829134
14.2	-10.8	-10.6	0.09	2.422	0.503	5.515	1.997	6	3586	ZINC000253624624
14.2	-9.8	-9.5	0.18	2.103	0.250	5.742	0.564	6	3587	ZINC000257550495
14.2	-10.9	-10.8	0.09	2.398	0.970	5.963	2.591	6	3588	ZINC000014617315
14.2	-11.1	-10.2	0.60	2.137	0.724	4.954	2.368	6	3589	ZINC000100822464
14.2	-11.0	-10.3	0.50	2.096	0.539	6.022	2.751	6	3590	ZINC000015251992
14.2	-11.0	-10.6	0.24	2.052	0.314	4.992	2.293	6	3591	ZINC000085640526
14.2	-10.1	-9.7	0.30	2.346	0.516	4.652	2.743	6	3592	ZINC000257501322
14.2	-12.2	-11.6	0.33	2.969	0.790	4.842	1.928	6	3593	ZINC000003984030
14.2	-10.6	-10.2	0.22	2.562	0.586	5.055	2.217	6	3594	ZINC000257568261
14.2	-10.7	-10.3	0.27	1.745	0.128	6.735	1.771	6	3595	ZINC000257456726
14.1	-10.9	-10.4	0.25	2.525	0.412	8.053	0.812	6	3596	ZINC000257619759

14.1	-11.1	-10.8	0.18	2.116	0.435	4.886	2.193	6	3597	ZINC000014617323
14.1	-9.3	-9.0	0.20	2.282	0.326	6.946	3.083	6	3598	ZINC000014930813
14.1	-10.1	-10.0	0.15	1.915	0.508	6.594	3.189	6	3599	ZINC000253477158
14.1	-9.2	-9.0	0.12	2.201	0.761	5.351	3.160	6	3600	ZINC000014760468
14.1	-10.6	-10.0	0.35	2.229	0.216	5.368	0.873	6	3601	ZINC000100825771
14.1	-10.2	-9.9	0.16	1.776	0.321	5.582	2.677	6	3602	ZINC000100780144
14.1	-10.1	-9.8	0.17	1.817	0.318	5.963	2.605	6	3603	ZINC000100777894
14.1	-10.9	-10.5	0.21	2.419	0.556	6.505	2.794	6	3604	ZINC000100825187
14.1	-10.4	-10.0	0.30	1.985	0.627	3.376	1.081	6	3605	ZINC000100821977
14.1	-10.5	-10.1	0.20	2.222	0.250	4.923	2.270	6	3606	ZINC000027521328
14.1	-11.4	-11.0	0.21	2.149	0.262	7.235	1.864	6	3607	ZINC000100781358
14.1	-11.0	-10.6	0.27	1.916	0.313	5.861	2.069	6	3608	ZINC000100783767
14.1	-9.1	-9.0	0.14	2.264	0.478	5.896	3.328	6	3609	ZINC000001565353
14.1	-10.8	-10.6	0.14	1.659	0.139	5.629	2.242	6	3610	ZINC000257435730
14.1	-9.4	-8.9	0.30	2.080	0.686	5.729	3.636	6	3611	ZINC000014811712

14.0	-10.3	-10.1	0.17	1.940	0.667	3.969	1.054	6	3612	ZINC000100821979
14.0	-9.1	-8.9	0.17	2.437	0.904	8.475	3.179	6	3613	ZINC000014644020
14.0	-10.8	-10.6	0.27	2.899	0.472	6.442	0.944	6	3614	ZINC000257417469
14.0	-12.0	-11.3	0.41	2.277	0.105	8.190	0.610	6	3615	ZINC000150361858
14.0	-10.2	-9.4	0.46	2.252	0.595	3.568	0.685	6	3616	ZINC000238754427
14.0	-10.9	-10.1	0.42	2.169	0.476	6.015	2.919	6	3617	ZINC000118937027
14.0	-11.3	-10.6	0.40	2.021	0.438	5.722	2.477	6	3618	ZINC000257436634
14.0	-11.1	-10.5	0.40	1.905	0.452	4.199	1.153	6	3619	ZINC000086039720
14.0	-10.4	-9.4	0.46	2.258	0.228	6.102	1.220	6	3620	ZINC000042804873
14.0	-10.3	-9.9	0.30	2.101	0.360	5.856	2.559	6	3621	ZINC000248338811
14.0	-11.3	-10.9	0.32	2.446	0.527	5.371	1.876	6	3622	ZINC000033963983
14.0	-10.4	-10.1	0.25	2.157	0.346	4.799	2.399	6	3623	ZINC000027521326
14.0	-8.3	-8.3	0.05	1.476	0.395	2.997	0.424	6	3624	ZINC000004098929
14.0	-10.7	-10.5	0.14	1.579	0.322	3.786	2.251	6	3625	ZINC000095619506
14.0	-10.9	-10.5	0.26	1.872	0.109	5.918	1.901	6	3626	ZINC000257514655

14.0	-11.3	-10.7	0.29	1.742	0.245	4.875	2.206	6	3627	ZINC000095619220
14.0	-10.9	-10.4	0.30	3.044	0.895	5.899	1.744	6	3628	ZINC000257501129
14.0	-9.6	-9.3	0.16	2.282	0.399	7.093	3.172	6	3629	ZINC000015152840
14.0	-9.2	-8.8	0.45	3.338	0.000	5.806	0.000	2	3630	ZINC000685933357
14.0	-9.7	-9.5	0.12	1.757	0.260	7.269	2.247	6	3631	ZINC000257405384
14.0	-11.1	-10.7	0.23	2.865	0.121	7.673	0.091	6	3632	ZINC000248330744
14.0	-11.5	-10.8	0.37	2.734	0.915	6.189	2.958	6	3633	ZINC000257556925
14.0	-11.3	-10.9	0.31	2.453	0.272	7.067	1.766	6	3634	ZINC000038143714
14.0	-10.3	-9.7	0.30	2.024	0.270	4.483	1.755	6	3635	ZINC000100777563
14.0	-11.6	-10.8	0.36	2.133	0.418	5.122	1.985	6	3636	ZINC000257436632
13.9	-9.1	-8.7	0.27	1.930	0.506	5.656	3.560	6	3637	ZINC000002563648
13.9	-10.4	-10.1	0.18	2.303	0.185	5.249	2.076	6	3638	ZINC000017653861
13.9	-8.8	-8.6	0.15	1.716	0.424	4.205	3.167	6	3639	ZINC000014811718
13.9	-10.1	-9.7	0.23	2.407	0.155	4.037	0.662	6	3640	ZINC000100821976
13.9	-9.6	-8.9	0.33	1.907	0.492	4.755	2.764	6	3641	ZINC000015657768

13.9	-10.1	-10.0	0.12	1.606	0.366	4.170	2.703	6	3642	ZINC000254685402
13.9	-9.8	-8.9	0.53	2.978	0.649	6.208	2.661	6	3643	ZINC000013414442
13.9	-8.8	-8.5	0.27	2.062	0.570	4.497	2.974	6	3644	ZINC000257684165
13.9	-11.0	-10.4	0.32	2.430	0.267	6.651	1.763	6	3645	ZINC000253500916
13.9	-10.9	-10.5	0.41	1.864	0.396	4.886	3.069	6	3646	ZINC000257461515
13.9	-10.1	-9.8	0.19	2.103	0.278	5.433	2.356	6	3647	ZINC000100779060
13.9	-9.8	-9.4	0.24	2.638	0.548	6.437	2.903	6	3648	ZINC000085946006
13.9	-10.6	-10.4	0.11	1.893	0.295	4.097	2.680	6	3649	ZINC000100825185
13.9	-11.2	-10.3	0.45	2.186	0.430	5.954	2.796	6	3650	ZINC000150364734
13.9	-9.7	-9.5	0.14	2.640	0.796	4.884	2.416	6	3651	ZINC000014806829
13.9	-11.2	-10.6	0.32	2.796	0.398	7.508	2.225	6	3652	ZINC000257455123
13.9	-7.7	-7.0	0.69	2.079	0.507	5.179	1.282	5	3653	ZINC000004095530
13.9	-11.7	-10.4	0.60	2.520	0.447	5.361	2.157	6	3654	ZINC000085648943
13.9	-11.7	-10.9	0.47	2.151	0.212	7.021	1.744	6	3655	ZINC000257465216
13.9	-11.7	-11.1	0.33	2.277	0.174	5.296	1.866	6	3656	ZINC000014720089

13.9	-10.5	-10.0	0.25	2.076	0.200	7.386	1.532	6	3657	ZINC000257493818
13.9	-10.4	-10.1	0.29	2.341	0.312	4.085	0.925	6	3658	ZINC000100774214
13.9	-10.7	-9.8	0.45	1.993	0.470	5.796	3.925	6	3659	ZINC000002563649
13.9	-10.8	-10.4	0.31	1.771	0.305	5.010	2.174	6	3660	ZINC000100778532
13.8	-10.0	-9.6	0.29	2.115	0.161	7.131	0.850	6	3661	ZINC000257400084
13.8	-10.4	-10.1	0.18	2.301	0.187	5.236	2.078	6	3662	ZINC000017653863
13.8	-9.7	-9.1	0.43	2.099	0.528	6.440	3.121	6	3663	ZINC000257527556
13.8	-11.2	-10.4	0.59	1.885	0.344	5.925	2.752	6	3664	ZINC000004098612
13.8	-10.5	-10.3	0.17	1.830	0.242	4.985	1.385	6	3665	ZINC000100829478
13.8	-8.8	-8.3	0.28	1.788	0.528	5.432	3.988	6	3666	ZINC000238737454
13.8	-10.5	-10.3	0.13	1.804	0.218	4.831	2.549	6	3667	ZINC000257731812
13.8	-9.4	-8.9	0.25	1.872	0.327	4.333	3.182	6	3668	ZINC000095619662
13.8	-11.6	-10.6	0.69	2.823	0.515	7.705	0.443	6	3669	ZINC000100825781
13.8	-11.1	-10.7	0.24	1.993	0.164	5.928	1.860	6	3670	ZINC000100777798
13.8	-10.6	-10.5	0.07	2.289	0.357	5.885	2.313	6	3671	ZINC000003785416

13.8	-11.1	-10.9	0.24	2.838	0.849	6.067	2.478	6	3672	ZINC000095619574
13.8	-10.9	-10.5	0.38	2.228	0.519	5.880	3.251	6	3673	ZINC000257427006
13.8	-10.4	-10.2	0.21	1.696	0.162	4.448	1.643	6	3674	ZINC000100777607
13.8	-11.6	-11.0	0.29	2.000	0.230	5.856	1.737	6	3675	ZINC000100779562
13.8	-10.3	-10.0	0.20	1.868	0.269	3.274	1.071	6	3676	ZINC000257382699
13.8	-10.4	-10.0	0.25	2.058	0.271	5.668	2.326	6	3677	ZINC000257415727
13.8	-10.9	-10.0	0.47	1.887	0.127	2.830	0.486	6	3678	ZINC000257462537
13.8	-9.0	-8.8	0.15	2.460	0.668	6.896	2.447	6	3679	ZINC000257479497
13.8	-8.9	-8.7	0.14	2.160	0.256	9.007	2.869	6	3680	ZINC000014760580
13.7	-12.5	-11.7	0.44	2.162	0.728	5.699	2.382	6	3681	ZINC000085648928
13.7	-10.0	-9.8	0.17	2.175	0.303	6.809	1.560	6	3682	ZINC000257048099
13.7	-10.0	-9.6	0.27	1.958	0.525	7.073	3.587	6	3683	ZINC000100825182
13.7	-11.1	-10.0	0.84	2.213	0.703	4.156	1.407	6	3684	ZINC000086044921
13.7	-10.2	-10.0	0.16	2.026	0.556	6.991	1.166	6	3685	ZINC000247952280
13.7	-10.4	-10.0	0.21	2.946	0.736	6.298	2.568	6	3686	ZINC000085599508

13.7	-11.3	-10.3	0.54	2.376	0.214	6.624	1.843	6	3687	ZINC000150366681
13.7	-9.7	-9.2	0.31	1.961	0.779	3.976	3.063	6	3688	ZINC000257442486
13.7	-9.9	-9.3	0.49	1.808	0.265	5.783	2.267	6	3689	ZINC000100772673
13.7	-11.0	-10.7	0.23	2.235	0.321	5.626	1.674	6	3690	ZINC000257494288
13.7	-11.1	-10.7	0.27	2.471	0.598	5.821	1.998	6	3691	ZINC000085744660
13.7	-10.8	-10.0	0.42	2.017	0.786	4.089	3.184	6	3692	ZINC000118937239
13.7	-10.4	-10.0	0.28	1.829	0.125	4.190	1.772	6	3693	ZINC000100829479
13.7	-10.6	-10.4	0.28	2.386	0.580	5.337	1.866	6	3694	ZINC000257493890
13.7	-10.1	-9.7	0.25	1.789	0.294	5.699	2.464	6	3695	ZINC000248338809
13.7	-11.3	-10.8	0.31	2.107	0.319	5.957	1.939	6	3696	ZINC000248330745
13.7	-10.6	-9.3	0.59	2.295	0.373	5.216	0.765	6	3697	ZINC000150352518
13.7	-11.1	-10.9	0.12	1.813	0.351	4.146	1.835	6	3698	ZINC000257494291
13.7	-9.7	-9.5	0.13	1.967	0.397	5.848	1.905	6	3699	ZINC000257639040
13.7	-9.4	-9.0	0.28	1.868	0.444	2.703	1.045	6	3700	ZINC000014762965
13.7	-9.5	-9.4	0.05	2.209	0.283	6.940	1.687	6	3701	ZINC000257396945

13.7	-11.1	-10.0	0.95	2.325	0.086	5.888	0.738	6	3702	ZINC000044405958
13.7	-10.8	-10.6	0.16	1.992	0.373	6.136	1.677	6	3703	ZINC000017545552
13.7	-9.5	-9.0	0.34	1.846	0.507	5.512	3.795	6	3704	ZINC000257408604
13.7	-10.2	-9.9	0.30	2.489	0.349	6.041	2.062	6	3705	ZINC000085862071
13.7	-11.5	-11.2	0.30	2.142	0.322	7.797	1.819	6	3706	ZINC000085954800
13.6	-10.3	-9.9	0.22	1.842	0.270	6.104	1.833	6	3707	ZINC000257731814
13.6	-10.9	-10.7	0.15	2.147	0.634	4.537	2.148	6	3708	ZINC000256102637
13.6	-11.1	-10.8	0.32	2.077	0.490	5.005	2.098	6	3709	ZINC000100777802
13.6	-10.2	-9.9	0.24	2.605	0.170	5.063	2.561	6	3710	ZINC000014819530
13.6	-10.6	-10.2	0.21	2.216	0.326	5.243	2.104	6	3711	ZINC000100823659
13.6	-9.9	-9.7	0.16	2.088	0.513	5.827	2.286	6	3712	ZINC000100779926
13.6	-10.5	-10.4	0.16	2.066	0.558	4.329	0.772	6	3713	ZINC000100829163
13.6	-10.7	-10.3	0.25	1.744	0.146	5.657	2.439	6	3714	ZINC000257668342
13.6	-9.6	-9.2	0.21	1.954	0.325	5.789	2.975	6	3715	ZINC000100825164
13.6	-11.5	-10.3	0.56	1.979	0.316	6.401	1.398	6	3716	ZINC000005085290

13.6	-11.6	-10.1	0.97	2.499	0.424	6.858	1.065	6	3717	ZINC000100825765
13.6	-10.6	-9.9	0.41	2.983	0.630	6.988	1.058	6	3718	ZINC000031560977
13.6	-9.7	-9.5	0.13	2.266	0.419	4.672	2.509	6	3719	ZINC000100825601
13.6	-10.8	-10.3	0.26	1.842	0.425	5.080	2.687	6	3720	ZINC000247952272
13.6	-11.2	-10.8	0.30	2.076	0.381	6.982	2.320	6	3721	ZINC000150361515
13.6	-10.9	-10.6	0.16	2.613	0.119	5.738	2.116	6	3722	ZINC000256102642
13.6	-10.6	-10.2	0.21	1.991	0.350	6.144	1.881	6	3723	ZINC000015105773
13.6	-11.0	-10.5	0.25	2.080	0.462	5.261	2.435	6	3724	ZINC000100778527
13.6	-10.3	-10.0	0.26	2.121	0.302	4.403	1.811	6	3725	ZINC000257402142
13.6	-9.4	-9.1	0.20	2.665	0.548	6.093	3.709	6	3726	ZINC000014645011
13.6	-10.5	-9.9	0.39	1.804	0.395	5.967	2.141	6	3727	ZINC000257440862
13.6	-9.4	-9.0	0.18	1.775	0.499	3.920	3.273	6	3728	ZINC000002554914
13.6	-11.6	-11.2	0.21	1.946	0.545	6.310	2.213	6	3729	ZINC000085744650
13.6	-10.5	-10.2	0.35	2.245	0.652	5.108	1.937	6	3730	ZINC000100829162
13.6	-10.7	-10.4	0.18	1.482	0.094	4.702	2.515	6	3731	ZINC000253624627

13.6	-10.4	-10.2	0.11	2.833	0.848	5.777	2.390	6	3732	ZINC000100829800
13.5	-9.4	-9.0	0.27	2.375	0.216	6.698	3.022	6	3733	ZINC000014811795
13.5	-10.7	-10.5	0.21	2.899	0.777	6.374	2.232	6	3734	ZINC000100829694
13.5	-10.6	-10.2	0.33	2.083	0.127	7.937	2.054	6	3735	ZINC000014441533
13.5	-12.2	-11.6	0.30	1.974	0.710	6.664	2.654	6	3736	ZINC000085977691
13.5	-11.2	-10.7	0.27	2.200	0.388	6.595	1.721	6	3737	ZINC000100779510
13.5	-11.0	-10.4	0.32	2.538	0.624	5.442	1.694	6	3738	ZINC000085570907
13.5	-11.4	-11.0	0.25	2.214	0.608	5.486	2.145	6	3739	ZINC000257491953
13.5	-9.9	-9.8	0.14	2.198	0.361	5.148	2.465	6	3740	ZINC000100777572
13.5	-11.0	-10.5	0.34	1.910	0.259	4.083	1.789	6	3741	ZINC000100778524
13.5	-9.3	-8.3	0.62	2.247	0.418	5.567	2.665	6	3742	ZINC000013397717
13.5	-9.7	-9.5	0.18	2.015	0.552	7.213	4.039	6	3743	ZINC000257416097
13.5	-10.9	-10.6	0.18	2.468	0.238	5.894	1.986	6	3744	ZINC000100825191
13.5	-10.5	-10.1	0.32	1.628	0.408	4.600	2.938	6	3745	ZINC000150368420
13.5	-10.9	-10.7	0.17	1.918	0.684	5.003	2.853	6	3746	ZINC000100825189

13.5	-10.9	-10.6	0.17	2.196	0.495	5.325	2.540	6	3747	ZINC000256099269
13.5	-10.7	-10.2	0.27	2.183	0.618	6.089	2.384	6	3748	ZINC000257404829
13.5	-10.9	-10.7	0.10	2.053	0.657	4.157	2.259	6	3749	ZINC000256099266
13.5	-12.1	-11.5	0.37	2.454	0.370	5.185	1.911	6	3750	ZINC000230085597
13.5	-11.0	-10.7	0.18	1.944	0.378	4.988	1.924	6	3751	ZINC000257516998
13.5	-10.5	-10.3	0.29	2.409	0.556	5.479	1.674	6	3752	ZINC000257493891
13.5	-10.5	-10.0	0.43	2.189	0.368	5.761	2.225	6	3753	ZINC000257414576
13.5	-11.4	-11.0	0.26	1.970	0.251	5.628	1.681	6	3754	ZINC000257483113
13.5	-10.9	-10.5	0.23	2.431	0.304	7.094	2.040	6	3755	ZINC000100822573
13.5	-10.9	-10.6	0.21	2.290	0.474	5.506	2.418	6	3756	ZINC000256099263
13.5	-10.5	-10.3	0.29	2.201	0.254	4.359	0.767	6	3757	ZINC000257493892
13.5	-10.9	-10.6	0.18	2.597	0.107	5.784	2.078	6	3758	ZINC000100822571
13.5	-10.4	-9.8	0.38	1.854	0.601	4.829	2.143	6	3759	ZINC000085551053
13.5	-10.9	-10.5	0.27	2.299	0.532	5.484	2.466	6	3760	ZINC000100825188
13.5	-10.9	-10.6	0.17	2.431	0.340	5.337	2.445	6	3761	ZINC000256102639

13.5	-10.9	-10.5	0.24	2.488	0.266	6.836	2.372	6	3762	ZINC000100822575
13.5	-10.3	-9.6	0.38	2.279	0.589	4.024	2.381	6	3763	ZINC000085955992
13.5	-10.9	-10.5	0.23	2.064	0.488	5.708	2.287	6	3764	ZINC000100822576
13.5	-10.9	-10.6	0.14	2.357	0.438	5.402	2.463	6	3765	ZINC000256099268
13.4	-11.7	-11.4	0.31	1.891	0.847	4.574	2.267	6	3766	ZINC000100824405
13.4	-11.7	-10.8	0.46	2.800	0.594	6.125	0.772	6	3767	ZINC000004098482
13.4	-8.5	-8.3	0.13	2.805	0.458	4.532	2.247	6	3768	ZINC000003830558
13.4	-8.8	-8.4	0.27	2.108	0.219	8.873	3.095	6	3769	ZINC000014811844
13.4	-10.9	-10.6	0.20	2.045	0.380	6.309	1.903	6	3770	ZINC000257407332
13.4	-11.2	-10.8	0.23	2.403	0.721	5.826	2.355	6	3771	ZINC000100823685
13.4	-10.5	-10.0	0.28	1.852	0.269	7.478	0.051	6	3772	ZINC000252463743
13.4	-11.6	-11.1	0.37	2.316	0.228	5.726	1.443	6	3773	ZINC000257413559
13.4	-11.5	-10.5	0.48	2.142	0.194	6.929	1.441	6	3774	ZINC000257744782
13.4	-11.0	-10.3	0.35	1.871	0.477	3.034	1.130	6	3775	ZINC000257435729
13.4	-10.4	-10.0	0.35	1.957	0.451	5.235	2.136	6	3776	ZINC000100829160

13.4	-11.0	-10.3	0.49	1.889	0.741	3.369	1.339	6	3777	ZINC000257398184
13.4	-9.3	-9.2	0.15	2.411	0.314	8.180	2.837	6	3778	ZINC000014644772
13.4	-12.0	-11.4	0.39	2.037	0.459	5.499	2.317	6	3779	ZINC000257491951
13.4	-10.4	-9.9	0.29	1.933	0.309	6.809	1.569	6	3780	ZINC000014617842
13.4	-10.9	-10.6	0.20	3.082	0.236	8.773	2.434	6	3781	ZINC000040165209
13.4	-9.1	-8.9	0.13	2.737	0.436	5.739	2.891	6	3782	ZINC000095620854
13.4	-8.6	-7.4	0.83	2.773	0.444	6.705	1.490	4	3783	ZINC000100774811
13.4	-10.3	-9.5	0.43	2.543	0.234	8.239	1.992	6	3784	ZINC000257493889
13.4	-8.9	-8.7	0.15	2.811	0.413	4.890	2.378	6	3785	ZINC000106893759
13.4	-11.0	-10.6	0.29	2.083	0.222	5.335	1.432	6	3786	ZINC000257429806
13.4	-10.9	-10.6	0.28	2.112	0.446	5.566	2.046	6	3787	ZINC000257483309
13.4	-10.5	-9.4	0.83	1.982	0.152	5.370	1.511	6	3788	ZINC000257514757
13.4	-9.6	-9.2	0.21	2.783	0.535	9.080	2.908	6	3789	ZINC000256080316
13.4	-11.0	-10.4	0.28	2.302	0.284	5.371	1.929	6	3790	ZINC000014617791
13.4	-10.4	-10.0	0.33	2.057	0.153	6.005	1.986	6	3791	ZINC000014243546

13.4	-9.7	-9.2	0.21	2.936	0.436	8.924	1.954	6	3792	ZINC000253500829
13.4	-10.8	-10.5	0.14	2.151	0.461	6.139	2.207	6	3793	ZINC000014858849
13.4	-11.6	-10.9	0.38	1.969	0.595	3.776	2.131	6	3794	ZINC000253589779
13.4	-9.2	-9.0	0.13	2.340	0.495	6.275	2.572	6	3795	ZINC000257495606
13.4	-10.6	-10.4	0.13	2.053	0.532	4.709	2.809	6	3796	ZINC000257404831
13.4	-10.6	-10.2	0.21	2.244	0.461	5.566	1.964	6	3797	ZINC000100821963
13.3	-10.4	-9.8	0.29	2.303	0.573	4.265	2.539	6	3798	ZINC000014439708
13.3	-11.4	-10.4	0.57	2.178	0.683	4.537	2.363	6	3799	ZINC000100829803
13.3	-11.1	-10.8	0.23	1.741	0.265	5.643	2.296	6	3800	ZINC000257566877
13.3	-11.6	-11.1	0.29	1.995	0.406	5.093	2.041	6	3801	ZINC000100779572
13.3	-11.3	-10.9	0.37	2.017	0.194	6.165	1.776	6	3802	ZINC000100781832
13.3	-9.6	-8.7	0.52	3.057	0.388	6.266	2.459	6	3803	ZINC000013414439
13.3	-11.2	-10.7	0.40	2.322	0.850	5.118	2.486	6	3804	ZINC000257738514
13.3	-10.9	-10.7	0.14	2.096	0.408	5.172	2.276	6	3805	ZINC000014720318
13.3	-10.4	-10.2	0.11	1.949	0.130	4.863	1.730	6	3806	ZINC000017545555

13.3	-11.4	-10.6	0.52	1.989	0.521	4.839	2.033	6	3807	ZINC000006017975
13.3	-11.9	-10.9	0.56	2.645	0.401	5.617	1.428	6	3808	ZINC000257438012
13.3	-9.6	-9.2	0.30	2.329	0.471	7.638	3.934	6	3809	ZINC000257472647
13.3	-9.7	-9.5	0.14	2.211	0.241	4.537	2.543	6	3810	ZINC000253615812
13.3	-11.4	-10.7	0.48	2.228	0.350	4.401	0.910	6	3811	ZINC000085991680
13.3	-10.1	-9.9	0.15	1.664	0.703	3.894	3.137	6	3812	ZINC000014709123
13.3	-10.9	-9.9	0.50	2.698	0.186	5.623	1.529	6	3813	ZINC000257488805
13.3	-9.8	-9.4	0.28	2.450	0.969	4.331	2.817	6	3814	ZINC000257538545
13.3	-10.4	-10.0	0.27	3.006	0.460	8.745	2.542	6	3815	ZINC000257549722
13.3	-11.0	-10.2	0.37	1.876	0.237	6.451	1.357	6	3816	ZINC000257546335
13.3	-8.7	-8.5	0.17	2.421	0.609	6.139	2.635	6	3817	ZINC000257452005
13.3	-11.1	-10.5	0.30	2.401	0.756	5.406	2.463	6	3818	ZINC000100829695
13.3	-10.8	-10.4	0.22	2.054	0.488	5.302	2.786	6	3819	ZINC000100777653
13.3	-10.6	-10.3	0.22	2.167	0.152	5.723	1.807	6	3820	ZINC000100826090
13.3	-10.3	-10.0	0.11	1.690	0.244	5.683	2.406	6	3821	ZINC000257731813

13.3	-9.1	-9.0	0.07	2.973	0.832	8.682	2.831	6	3822	ZINC000004228243
13.3	-11.4	-11.1	0.30	2.009	0.364	4.882	2.052	6	3823	ZINC000257658448
13.3	-9.9	-9.8	0.16	2.167	0.454	5.467	1.953	6	3824	ZINC000257433858
13.3	-12.3	-11.8	0.43	1.903	0.517	5.072	2.838	6	3825	ZINC000257396099
13.3	-9.8	-9.2	0.43	3.093	0.437	6.361	2.782	6	3826	ZINC000015062978
13.3	-10.7	-10.2	0.30	1.914	0.480	3.996	2.133	6	3827	ZINC000100777565
13.3	-9.8	-9.3	0.33	2.407	0.847	4.439	2.820	6	3828	ZINC000015062976
13.3	-11.2	-10.3	0.40	2.115	0.387	5.767	2.129	6	3829	ZINC000095619496
13.3	-11.7	-11.0	0.35	1.904	0.499	4.379	2.307	6	3830	ZINC000257541115
13.3	-10.3	-9.8	0.22	1.857	0.398	7.124	1.776	6	3831	ZINC000100829382
13.2	-11.0	-10.7	0.15	2.347	0.260	4.507	1.493	6	3832	ZINC000014617313
13.2	-9.9	-9.4	0.33	2.014	0.445	4.744	2.812	6	3833	ZINC000014819526
13.2	-10.8	-10.5	0.17	2.045	0.436	5.850	2.280	6	3834	ZINC000014610657
13.2	-10.2	-9.8	0.25	2.222	0.559	3.994	2.996	6	3835	ZINC000043465449
13.2	-12.6	-11.3	1.02	2.304	0.608	6.118	2.452	6	3836	ZINC000100829129

13.2	-11.3	-10.9	0.27	1.467	0.128	4.337	2.379	6	3837	ZINC000257485977
13.2	-11.4	-11.0	0.33	1.679	0.376	4.156	1.629	6	3838	ZINC000257483307
13.2	-9.3	-9.1	0.16	2.207	0.268	5.706	3.112	6	3839	ZINC000257544404
13.2	-11.6	-11.2	0.34	2.435	0.418	6.494	3.213	6	3840	ZINC000085860876
13.2	-9.5	-9.3	0.17	2.588	0.466	8.250	2.918	6	3841	ZINC000100825604
13.2	-11.3	-10.6	0.34	2.688	0.669	6.139	2.846	6	3842	ZINC000100824451
13.2	-10.8	-10.3	0.28	1.940	0.344	4.999	3.472	6	3843	ZINC000118937041
13.2	-10.9	-10.2	0.40	1.992	0.329	6.505	2.061	6	3844	ZINC000150364955
13.2	-9.7	-9.1	0.53	2.073	0.656	6.181	2.388	6	3845	ZINC000100824606
13.2	-12.0	-11.2	0.48	1.977	0.268	4.571	2.514	6	3846	ZINC000085991683
13.2	-9.9	-9.7	0.23	2.247	0.353	5.734	1.837	6	3847	ZINC000100825363
13.2	-10.5	-9.6	0.49	2.413	0.368	4.328	0.586	6	3848	ZINC000013382493
13.2	-10.6	-10.1	0.29	1.851	0.431	4.633	2.587	6	3849	ZINC000100779170
13.2	-10.7	-10.0	0.43	2.475	0.177	8.885	2.447	6	3850	ZINC000070455321
13.2	-10.5	-10.2	0.21	2.642	0.371	9.046	0.305	6	3851	ZINC000257455121

13.2	-9.8	-9.3	0.39	2.419	0.834	4.457	2.763	6	3852	ZINC000015062971
13.2	-11.2	-10.8	0.29	2.412	0.746	5.689	1.875	6	3853	ZINC000085740393
13.2	-11.2	-10.7	0.27	2.664	0.221	7.494	2.242	6	3854	ZINC000257455122
13.2	-11.4	-11.1	0.20	2.181	0.768	4.657	2.314	6	3855	ZINC000257472503
13.2	-9.1	-8.6	0.32	2.257	0.495	5.436	2.635	6	3856	ZINC000257590968
13.2	-10.8	-10.4	0.21	2.837	0.754	6.427	2.237	6	3857	ZINC000100829802
13.2	-11.6	-10.6	0.60	2.515	0.543	6.765	0.663	6	3858	ZINC000100821966
13.2	-11.4	-10.9	0.25	2.053	0.244	5.000	1.890	6	3859	ZINC000100779391
13.2	-11.5	-11.2	0.19	1.636	0.373	4.599	2.492	6	3860	ZINC000012296274
13.2	-10.5	-10.1	0.24	1.985	0.240	5.694	1.935	6	3861	ZINC000100777333
13.1	-10.5	-10.1	0.25	1.909	0.154	7.040	0.931	6	3862	ZINC000100772240
13.1	-11.3	-11.0	0.24	2.032	0.318	6.739	1.336	6	3863	ZINC000014720083
13.1	-10.5	-10.0	0.38	2.028	0.197	7.446	0.659	6	3864	ZINC000257440861
13.1	-10.5	-10.1	0.21	2.091	0.283	6.230	2.383	6	3865	ZINC000100780759
13.1	-10.6	-10.3	0.21	2.064	0.330	5.114	2.332	6	3866	ZINC000257198429

13.1	-9.5	-9.3	0.16	1.689	0.312	8.822	3.430	6	3867	ZINC000014489965
13.1	-10.4	-10.1	0.26	2.262	0.539	5.097	2.582	6	3868	ZINC000100777714
13.1	-9.8	-9.3	0.38	2.553	0.760	4.655	2.673	6	3869	ZINC000015062974
13.1	-10.4	-9.8	0.32	1.666	0.411	3.567	2.532	6	3870	ZINC000257604351
13.1	-10.6	-10.0	0.38	1.995	0.493	6.503	1.906	6	3871	ZINC000100780141
13.1	-10.9	-10.6	0.19	2.450	0.432	5.140	2.440	6	3872	ZINC000257480397
13.1	-11.8	-10.9	0.46	2.251	0.477	6.038	1.805	6	3873	ZINC000014617314
13.1	-8.6	-8.1	0.29	2.417	0.559	4.869	3.235	6	3874	ZINC000095618169
13.1	-11.6	-11.0	0.42	1.845	0.591	4.090	2.318	6	3875	ZINC000150352528
13.1	-9.5	-9.3	0.15	1.778	0.409	4.527	3.051	6	3876	ZINC000257782821
13.1	-9.8	-9.2	0.33	2.982	0.549	4.710	0.688	6	3877	ZINC000014728343
13.1	-9.1	-8.9	0.24	2.703	0.918	8.181	3.125	6	3878	ZINC000257487564
13.1	-11.2	-10.7	0.37	2.151	0.565	7.234	1.406	6	3879	ZINC000040950680
13.1	-10.5	-10.1	0.23	1.957	0.377	4.873	2.114	6	3880	ZINC000100778531
13.1	-11.5	-10.9	0.40	3.403	0.178	7.081	0.361	6	3881	ZINC000256005426

13.1	-9.5	-9.3	0.14	1.948	0.485	6.700	3.702	6	3882	ZINC000253615813
13.1	-11.3	-10.7	0.41	1.422	0.109	5.915	2.745	6	3883	ZINC000095620003
13.1	-11.1	-10.4	0.39	2.587	1.320	5.512	2.956	6	3884	ZINC000118937234
13.1	-10.4	-9.8	0.27	2.208	0.159	6.042	1.621	6	3885	ZINC000100777583
13.1	-10.5	-10.1	0.26	1.913	0.153	7.040	0.929	6	3886	ZINC000100772242
13.0	-6.9	-5.0	0.94	2.476	0.714	8.883	4.688	6	3887	ZINC000003978844
13.0	-10.0	-8.8	0.62	2.995	0.839	7.839	0.542	6	3888	ZINC000100823807
13.0	-9.6	-9.2	0.18	2.569	0.857	4.351	2.687	6	3889	ZINC000012495974
13.0	-10.5	-10.2	0.20	1.983	0.352	5.686	1.326	6	3890	ZINC000257198430
13.0	-11.6	-11.2	0.29	2.330	0.657	5.547	2.132	6	3891	ZINC000257405316
13.0	-11.7	-10.4	0.68	2.107	0.151	6.329	1.944	6	3892	ZINC000004098552
13.0	-10.4	-9.6	0.43	2.474	0.388	5.526	1.754	6	3893	ZINC000085862050
13.0	-10.5	-10.4	0.13	2.137	0.250	5.909	2.140	6	3894	ZINC000257808147
13.0	-11.6	-11.2	0.30	1.997	0.371	5.662	2.210	6	3895	ZINC000254697454
13.0	-10.8	-10.5	0.16	1.651	0.449	4.932	2.689	6	3896	ZINC000095620002

13.0	-10.2	-9.6	0.34	3.968	0.876	5.708	0.325	6	3897	ZINC000100044664
13.0	-10.8	-10.4	0.25	1.736	0.304	5.957	2.536	6	3898	ZINC000100782678
13.0	-10.6	-10.4	0.09	1.937	0.185	4.805	2.012	6	3899	ZINC000100825843
13.0	-9.8	-9.5	0.21	2.022	0.752	4.059	2.881	6	3900	ZINC000015058164
13.0	-8.9	-8.7	0.12	2.018	0.600	7.224	3.995	6	3901	ZINC000014821375
13.0	-9.8	-9.4	0.25	2.039	0.390	6.779	3.259	6	3902	ZINC000257567736
13.0	-11.4	-11.0	0.26	1.876	0.258	5.896	2.035	6	3903	ZINC000257541113
13.0	-11.5	-11.3	0.19	1.792	0.979	4.908	3.182	6	3904	ZINC000100825777
13.0	-10.3	-9.8	0.29	1.951	0.321	3.937	1.963	6	3905	ZINC000014243548
13.0	-11.7	-11.1	0.34	3.397	0.493	7.434	1.346	6	3906	ZINC000039166887
13.0	-10.5	-10.2	0.17	2.023	0.295	5.266	1.687	6	3907	ZINC000257198431
13.0	-10.8	-10.1	0.45	2.415	0.441	4.382	2.370	6	3908	ZINC000257414768
13.0	-10.8	-10.4	0.20	2.095	0.402	4.377	1.713	6	3909	ZINC000100778528
13.0	-9.5	-9.5	0.08	2.264	0.311	3.884	0.250	6	3910	ZINC000013382491
13.0	-9.6	-9.2	0.25	2.866	1.260	5.718	3.326	6	3911	ZINC000012495977

13.0	-10.2	-9.6	0.41	1.717	0.437	3.152	1.103	6	3912	ZINC000100827832
13.0	-10.4	-9.9	0.36	1.627	0.571	5.100	3.156	6	3913	ZINC000059728603
13.0	-11.6	-10.8	0.53	2.051	0.485	5.941	1.972	6	3914	ZINC000085570901
13.0	-10.9	-10.7	0.09	1.870	0.306	5.349	2.317	6	3915	ZINC000100779512
12.9	-9.7	-9.5	0.16	2.444	0.727	5.796	3.252	6	3916	ZINC000253615811
12.9	-10.2	-9.9	0.16	2.146	0.179	5.712	1.925	6	3917	ZINC000100777907
12.9	-10.4	-9.9	0.36	2.081	0.207	4.766	1.837	6	3918	ZINC000238751951
12.9	-10.7	-10.4	0.19	1.772	0.328	4.835	2.169	6	3919	ZINC000100299709
12.9	-11.0	-10.3	0.45	2.301	0.321	5.640	1.231	6	3920	ZINC000248332072
12.9	-11.0	-10.8	0.14	2.190	0.392	5.894	2.711	6	3921	ZINC000257455052
12.9	-10.7	-10.3	0.28	2.015	0.592	4.356	2.594	6	3922	ZINC000100778817
12.9	-10.4	-10.2	0.13	1.887	0.110	4.587	0.746	6	3923	ZINC000100825844
12.9	-10.6	-10.2	0.17	2.433	0.478	6.137	2.407	6	3924	ZINC000015105771
12.9	-10.6	-10.2	0.34	2.305	0.591	8.543	2.656	6	3925	ZINC000257567516
12.9	-11.3	-10.6	0.38	1.760	0.122	5.394	2.620	6	3926	ZINC000100780250

12.9	-10.2	-9.9	0.23	1.811	0.266	6.186	2.051	6	3927	ZINC000247973592
12.9	-11.1	-10.5	0.43	1.791	0.379	6.600	1.184	6	3928	ZINC000095619502
12.9	-10.1	-9.8	0.23	2.247	0.185	7.014	0.799	6	3929	ZINC000100827292
12.9	-10.4	-10.2	0.17	2.016	0.290	5.247	1.692	6	3930	ZINC000198011939
12.9	-11.3	-10.6	0.36	1.941	0.168	5.937	1.405	6	3931	ZINC000257566000
12.9	-10.5	-10.3	0.19	2.024	0.522	4.577	0.685	6	3932	ZINC000100829164
12.9	-11.0	-10.7	0.14	2.086	0.449	6.843	2.378	6	3933	ZINC000100824732
12.9	-11.4	-10.8	0.43	2.276	0.443	5.986	1.930	6	3934	ZINC000100823684
12.9	-10.4	-10.0	0.22	2.028	0.081	4.433	0.656	6	3935	ZINC000100822827
12.9	-10.4	-10.2	0.17	1.963	0.323	5.215	1.714	6	3936	ZINC000100825842
12.9	-10.4	-10.2	0.19	1.915	0.133	4.654	0.765	6	3937	ZINC000100825846
12.9	-10.3	-10.1	0.18	1.729	0.125	4.807	2.431	6	3938	ZINC000100777793
12.9	-11.3	-10.8	0.35	1.984	0.558	4.708	2.441	6	3939	ZINC000257415285
12.9	-10.3	-9.9	0.32	1.876	0.431	5.206	1.995	6	3940	ZINC000257640756
12.9	-9.8	-9.3	0.32	2.024	0.460	4.216	3.045	6	3941	ZINC000015058166

12.9	-10.6	-10.3	0.22	2.129	0.507	8.984	1.656	6	3942	ZINC000100828997
12.8	-11.0	-10.5	0.31	1.763	0.335	4.450	2.544	6	3943	ZINC000100779211
12.8	-11.3	-10.8	0.40	1.872	0.218	5.973	1.968	6	3944	ZINC000100779507
12.8	-10.0	-9.7	0.27	2.276	0.265	7.052	0.925	6	3945	ZINC000100827288
12.8	-11.5	-11.2	0.17	2.019	0.455	5.605	1.914	6	3946	ZINC000257405313
12.8	-10.0	-9.6	0.33	1.914	0.614	6.549	2.768	6	3947	ZINC000100827170
12.8	-9.7	-9.1	0.33	2.215	0.408	5.342	2.508	6	3948	ZINC000100775215
12.8	-10.1	-9.7	0.22	2.163	0.527	5.093	2.800	6	3949	ZINC000257458914
12.8	-10.4	-10.0	0.29	1.968	0.341	5.987	2.458	6	3950	ZINC000257404828
12.8	-10.6	-10.3	0.25	2.128	0.504	8.989	1.660	6	3951	ZINC000257567515
12.8	-12.3	-11.5	0.44	1.936	0.421	5.122	1.642	6	3952	ZINC000100781367
12.8	-11.7	-11.1	0.36	1.903	0.486	5.597	1.822	6	3953	ZINC000095619536
12.8	-10.9	-10.6	0.14	2.265	0.475	4.235	1.976	6	3954	ZINC000100823662
12.8	-11.1	-10.7	0.22	2.245	0.199	7.597	0.093	6	3955	ZINC000257483308
12.8	-11.3	-11.0	0.24	2.331	0.420	5.113	1.829	6	3956	ZINC000013335899

12.8	-11.6	-11.0	0.43	2.271	0.545	4.467	2.308	6	3957	ZINC000257482001
12.8	-9.3	-9.0	0.18	1.513	0.223	5.449	4.143	6	3958	ZINC000014811714
12.8	-10.4	-10.2	0.17	2.056	0.365	4.451	1.664	6	3959	ZINC000100829476
12.8	-9.6	-9.4	0.15	2.254	0.301	9.300	0.187	6	3960	ZINC000004016718
12.8	-11.1	-10.8	0.24	1.682	0.532	4.368	2.246	6	3961	ZINC000086035336
12.8	-9.8	-9.5	0.16	1.713	0.350	7.779	0.036	6	3962	ZINC000248332064
12.8	-11.5	-11.2	0.27	1.937	1.046	5.077	3.085	6	3963	ZINC000100825759
12.8	-10.4	-10.1	0.28	2.031	0.268	4.412	2.085	6	3964	ZINC000085956005
12.8	-9.0	-8.6	0.22	1.972	0.732	5.585	3.579	6	3965	ZINC000014811789
12.8	-10.9	-10.6	0.16	1.893	0.578	3.643	2.284	6	3966	ZINC000257385901
12.8	-10.7	-10.4	0.21	2.284	0.038	7.762	0.650	6	3967	ZINC000086039723
12.8	-11.0	-10.7	0.17	2.350	0.502	7.650	2.596	6	3968	ZINC000100824737
12.8	-9.9	-9.5	0.36	2.056	0.599	4.734	2.627	6	3969	ZINC000100823418
12.8	-10.0	-9.7	0.21	2.006	0.144	6.352	1.621	6	3970	ZINC000100827287
12.7	-10.2	-9.8	0.30	2.121	0.077	5.218	2.192	6	3971	ZINC000085561875

12.7	-9.6	-9.4	0.13	1.666	0.387	4.301	3.317	6	3972	ZINC000059728998
12.7	-10.9	-10.7	0.19	2.209	0.289	5.450	2.599	6	3973	ZINC000100822831
12.7	-10.1	-9.7	0.28	2.026	0.271	6.159	2.220	6	3974	ZINC000257400085
12.7	-9.5	-9.3	0.14	2.368	0.403	3.626	0.893	6	3975	ZINC000085588510
12.7	-8.8	-8.5	0.18	2.074	0.310	5.549	3.888	6	3976	ZINC000014811846
12.7	-11.6	-11.0	0.44	2.291	0.517	4.481	2.304	6	3977	ZINC000257482002
12.7	-11.3	-10.6	0.46	2.707	0.769	6.472	3.077	6	3978	ZINC000095617626
12.7	-9.7	-9.2	0.23	1.910	0.497	2.956	1.122	6	3979	ZINC000014819528
12.7	-10.5	-10.2	0.14	2.025	0.597	4.764	2.143	6	3980	ZINC000257507786
12.7	-10.9	-10.1	0.55	2.570	0.487	5.371	1.164	6	3981	ZINC000257397146
12.7	-9.4	-8.9	0.31	2.157	0.384	5.130	2.142	6	3982	ZINC000100823234
12.7	-10.4	-10.3	0.07	2.183	0.248	3.926	2.083	6	3983	ZINC000257470148
12.7	-10.8	-10.7	0.18	1.873	0.229	6.036	2.326	6	3984	ZINC000257621915
12.7	-9.6	-8.5	0.55	2.037	0.376	4.891	1.342	6	3985	ZINC000257620362
12.7	-11.1	-10.8	0.21	2.340	0.991	5.188	2.392	6	3986	ZINC000100783685

12.7	-10.0	-9.7	0.19	2.196	0.125	6.976	0.786	6	3987	ZINC000257400086
12.7	-11.5	-10.9	0.33	2.437	0.344	4.485	1.575	6	3988	ZINC000095918728
12.7	-9.4	-9.0	0.23	1.319	0.375	5.287	3.706	6	3989	ZINC000257574800
12.7	-11.9	-11.3	0.41	1.609	0.546	4.962	2.469	6	3990	ZINC000100825769
12.7	-10.9	-10.8	0.08	2.089	0.220	4.863	1.737	6	3991	ZINC000095620009
12.7	-9.2	-8.7	0.33	2.063	0.422	4.797	2.650	6	3992	ZINC000100771816
12.6	-11.3	-9.8	0.93	1.876	0.207	3.520	0.307	5	3993	ZINC000257498923
12.6	-12.1	-11.1	0.49	1.477	0.166	4.706	2.181	6	3994	ZINC000257413560
12.6	-11.1	-10.6	0.35	1.725	0.548	5.434	3.332	6	3995	ZINC000100782681
12.6	-10.2	-9.3	0.54	2.061	0.407	5.467	2.145	6	3996	ZINC000085744737
12.6	-10.9	-10.4	0.40	2.015	0.394	3.683	0.868	6	3997	ZINC000100777710
12.6	-10.1	-9.7	0.30	2.255	0.225	6.325	1.939	6	3998	ZINC000014617844
12.6	-11.3	-11.1	0.15	2.057	0.349	4.614	0.929	6	3999	ZINC000150363926
12.6	-11.3	-11.0	0.18	1.343	0.102	6.302	1.930	6	4000	ZINC000257467832
12.6	-11.4	-11.2	0.12	2.225	0.593	5.517	1.930	6	4001	ZINC000257405314

12.6	-11.0	-10.7	0.21	2.086	0.341	6.553	2.511	6	4002	ZINC000257455053
12.6	-10.9	-10.7	0.17	2.189	0.673	5.376	2.586	6	4003	ZINC000014720419
12.6	-9.8	-9.5	0.24	1.820	0.471	4.257	3.392	6	4004	ZINC000085863130
12.6	-11.0	-10.7	0.20	2.469	0.556	6.901	2.919	6	4005	ZINC000257455051
12.6	-10.5	-10.2	0.18	2.199	0.379	4.579	2.223	6	4006	ZINC000118937109
12.6	-10.9	-10.4	0.30	2.966	0.770	7.055	1.439	6	4007	ZINC000257577153
12.6	-10.4	-10.2	0.31	1.763	0.361	3.545	2.173	6	4008	ZINC000257387713
12.6	-11.2	-10.5	0.40	2.863	0.297	7.423	1.297	6	4009	ZINC000257413143
12.6	-9.9	-8.9	0.63	1.958	0.337	5.494	1.689	6	4010	ZINC000257373613
12.6	-10.2	-9.7	0.23	2.790	0.255	7.930	1.864	6	4011	ZINC000100774220
12.6	-10.7	-10.2	0.31	2.246	0.868	4.496	2.314	6	4012	ZINC000100773903
12.6	-11.1	-10.3	0.42	2.579	0.208	5.949	0.717	6	4013	ZINC000257429805
12.6	-12.4	-11.3	0.56	2.071	0.271	5.744	1.894	6	4014	ZINC000257538446
12.6	-10.2	-10.0	0.19	3.403	0.257	10.266	0.275	6	4015	ZINC000257546978
12.6	-11.3	-10.7	0.39	2.359	0.177	5.623	1.917	6	4016	ZINC000100822590

12.6	-11.9	-11.3	0.38	1.992	0.296	6.297	1.408	6	4017	ZINC000095619533
12.6	-9.7	-9.3	0.29	2.224	0.584	4.089	2.725	6	4018	ZINC000040164386
12.6	-9.9	-9.5	0.24	2.188	0.548	6.557	1.657	6	4019	ZINC000100829402
12.5	-11.0	-10.6	0.35	2.321	0.083	5.211	2.065	6	4020	ZINC000033963978
12.5	-10.4	-9.8	0.37	2.262	0.055	5.052	0.162	6	4021	ZINC000238762118
12.5	-10.7	-10.4	0.21	2.793	0.227	7.467	1.148	6	4022	ZINC000085883292
12.5	-9.4	-9.3	0.10	2.234	0.466	6.196	2.265	6	4023	ZINC000257511391
12.5	-10.5	-10.1	0.28	1.913	0.482	4.414	2.292	6	4024	ZINC000014858848
12.5	-10.9	-10.7	0.13	2.036	0.208	5.837	2.204	6	4025	ZINC000095619495
12.5	-8.6	-8.4	0.16	1.675	0.668	5.614	4.015	6	4026	ZINC000257796844
12.5	-9.4	-9.1	0.17	2.006	0.488	3.057	0.677	6	4027	ZINC000014488277
12.5	-9.7	-9.4	0.22	2.023	1.219	5.551	3.659	6	4028	ZINC000150338767
12.5	-9.7	-9.4	0.18	2.019	0.295	3.011	0.369	6	4029	ZINC000100825603
12.5	-10.3	-9.9	0.28	3.007	0.430	6.257	1.364	6	4030	ZINC000100777882
12.5	-11.7	-11.0	0.39	1.778	0.261	5.625	2.474	6	4031	ZINC000015105345

12.5	-9.6	-9.5	0.11	1.972	0.408	6.286	1.963	6	4032	ZINC000257546994
12.5	-11.6	-10.8	0.57	2.047	0.671	5.409	1.597	6	4033	ZINC000230078637
12.5	-9.9	-9.3	0.40	2.266	0.539	8.686	2.468	6	4034	ZINC000257403664
12.5	-5.2	-3.4	1.30	1.855	0.745	13.244	0.180	3	4035	ZINC000004097711
12.5	-9.5	-9.3	0.14	2.391	0.478	7.145	3.716	6	4036	ZINC000257472648
12.5	-9.1	-8.7	0.31	1.871	0.374	4.607	2.612	6	4037	ZINC000100773938
12.5	-12.5	-10.9	0.80	2.175	0.375	4.759	1.657	6	4038	ZINC000085991686
12.5	-11.5	-10.4	0.65	2.258	0.191	6.862	1.586	6	4039	ZINC000230065846
12.5	-10.7	-10.0	0.39	1.864	0.292	3.212	0.809	6	4040	ZINC000085956007
12.5	-9.4	-9.0	0.19	2.581	0.444	4.676	1.865	6	4041	ZINC000085758754
12.5	-11.2	-10.9	0.27	1.822	0.048	6.429	1.419	6	4042	ZINC000014720337
12.5	-9.7	-9.4	0.20	2.332	0.638	6.844	3.004	6	4043	ZINC000238735243
12.5	-11.6	-10.7	0.52	1.978	0.280	4.765	1.824	6	4044	ZINC000254697456
12.5	-11.0	-10.4	0.33	1.594	0.193	4.026	1.925	6	4045	ZINC000253624625
12.4	-11.4	-10.7	0.36	2.279	0.439	6.030	1.998	6	4046	ZINC000150366671

12.4	-9.4	-9.0	0.32	2.129	0.528	5.415	3.088	6	4047	ZINC000257451582
12.4	-11.6	-11.0	0.27	1.779	0.288	4.895	1.865	6	4048	ZINC000100779394
12.4	-10.0	-9.7	0.21	2.123	0.446	6.558	3.085	6	4049	ZINC000100829000
12.4	-11.6	-10.4	0.67	2.369	0.131	6.166	0.671	6	4050	ZINC000014693305
12.4	-10.6	-9.4	0.55	4.798	0.435	7.569	1.589	6	4051	ZINC000100048111
12.4	-11.3	-11.0	0.21	1.856	0.082	4.096	1.648	6	4052	ZINC000257485979
12.4	-10.8	-10.5	0.23	1.781	0.288	5.309	1.682	6	4053	ZINC000095619222
12.4	-9.7	-9.4	0.21	2.294	0.781	4.316	2.731	6	4054	ZINC000100825600
12.4	-11.5	-10.9	0.43	3.328	0.495	5.762	1.363	6	4055	ZINC000100779287
12.4	-10.9	-10.7	0.19	1.806	0.372	5.271	2.015	6	4056	ZINC000100781830
12.4	-11.2	-10.9	0.20	1.689	0.176	4.822	1.919	6	4057	ZINC000100779565
12.4	-10.1	-9.8	0.24	2.184	0.680	6.240	2.512	6	4058	ZINC000100825840
12.4	-11.0	-10.4	0.29	1.975	0.577	4.795	2.437	6	4059	ZINC000100783761
12.4	-11.5	-11.0	0.41	3.265	0.606	6.492	1.516	6	4060	ZINC000257480110
12.4	-10.2	-10.0	0.16	1.944	0.254	7.884	2.063	6	4061	ZINC000073170143

12.4	-9.4	-9.2	0.12	2.079	0.188	5.973	2.270	6	4062	ZINC000100777890
12.4	-10.8	-10.3	0.29	1.775	0.230	4.765	2.371	6	4063	ZINC000261498229
12.4	-10.7	-10.5	0.11	2.251	0.567	4.536	2.064	6	4064	ZINC000100779214
12.4	-11.7	-10.3	0.80	2.353	0.111	6.004	1.161	6	4065	ZINC000100822349
12.4	-11.2	-11.0	0.13	2.294	0.588	5.597	2.818	6	4066	ZINC000100828732
12.4	-10.3	-10.0	0.21	2.167	0.493	5.512	3.269	6	4067	ZINC000100823826
12.4	-10.4	-10.0	0.20	2.112	0.313	6.194	2.279	6	4068	ZINC000257396947
12.4	-9.4	-9.2	0.10	1.910	0.308	4.306	2.809	6	4069	ZINC000013132546
12.4	-10.9	-10.6	0.26	1.900	0.331	5.821	1.808	6	4070	ZINC000014619925
12.3	-10.0	-9.8	0.12	1.648	0.452	3.505	2.307	6	4071	ZINC000100829384
12.3	-10.7	-10.2	0.25	2.026	0.316	5.832	2.299	6	4072	ZINC000095619497
12.3	-9.6	-9.3	0.28	2.745	0.146	5.354	0.533	6	4073	ZINC000100823825
12.3	-11.1	-10.8	0.22	2.140	0.338	6.726	1.642	6	4074	ZINC000257449663
12.3	-11.4	-11.0	0.21	1.870	0.109	5.564	1.995	6	4075	ZINC000095619518
12.3	-11.2	-10.9	0.17	1.871	0.624	5.105	1.700	6	4076	ZINC000100829047

12.3	-10.6	-10.3	0.15	2.448	0.458	5.881	2.003	6	4077	ZINC000257580981
12.3	-11.3	-10.9	0.20	1.908	0.280	4.690	2.144	6	4078	ZINC000014720192
12.3	-10.6	-10.3	0.24	2.194	0.400	5.007	2.246	6	4079	ZINC000257410666
12.3	-10.1	-9.8	0.19	2.738	0.172	6.945	2.459	6	4080	ZINC000257453479
12.3	-10.0	-9.7	0.17	2.354	0.399	5.512	2.095	6	4081	ZINC000070454233
12.3	-11.2	-10.9	0.15	1.823	0.605	4.614	1.958	6	4082	ZINC000257383882
12.3	-9.2	-8.9	0.16	2.354	0.836	6.645	3.333	6	4083	ZINC000014760472
12.3	-12.2	-11.2	0.53	2.826	0.772	7.280	1.060	6	4084	ZINC000100779732
12.3	-12.0	-11.5	0.24	2.124	0.526	5.672	1.685	6	4085	ZINC000257658450
12.3	-8.8	-8.0	0.56	1.790	0.294	5.412	3.901	6	4086	ZINC000256166391
12.3	-11.8	-11.3	0.29	2.530	0.980	5.641	2.955	6	4087	ZINC000100774341
12.3	-10.7	-9.7	0.47	3.178	0.911	6.525	2.636	6	4088	ZINC000118937256
12.3	-11.4	-10.7	0.44	1.915	0.158	6.736	1.439	6	4089	ZINC000015105337
12.3	-11.1	-10.7	0.36	2.292	0.437	5.159	2.146	6	4090	ZINC000230088388
12.3	-9.7	-9.5	0.15	2.270	0.292	3.662	0.856	6	4091	ZINC000014488281

12.3	-10.4	-9.8	0.30	2.361	0.283	8.175	2.634	6	4092	ZINC000085842738
12.3	-10.2	-10.0	0.17	1.671	0.109	4.682	2.558	6	4093	ZINC000100777946
12.3	-11.6	-10.9	0.37	1.601	0.276	3.888	1.987	6	4094	ZINC000118937506
12.3	-10.1	-9.9	0.11	2.024	0.316	6.211	1.865	6	4095	ZINC000248338810
12.3	-10.8	-10.2	0.33	2.168	0.405	6.131	2.240	6	4096	ZINC000100779519
12.3	-10.7	-10.3	0.24	2.645	0.700	5.890	2.331	6	4097	ZINC000257392670
12.3	-10.0	-9.6	0.26	2.275	0.537	5.811	2.440	6	4098	ZINC000100828998
12.3	-11.8	-11.1	0.36	2.244	0.527	4.980	1.640	6	4099	ZINC000100779576
12.3	-12.7	-11.8	0.58	1.931	0.378	3.957	2.082	6	4100	ZINC000085917128
12.3	-11.6	-10.9	0.35	2.512	0.693	5.760	2.224	6	4101	ZINC000100783680
12.3	-10.5	-10.2	0.16	2.004	0.317	4.736	1.791	6	4102	ZINC000257519431
12.3	-11.2	-10.8	0.21	1.884	0.627	5.433	1.827	6	4103	ZINC000257383881
12.3	-10.6	-9.7	0.42	2.724	0.608	5.345	1.972	6	4104	ZINC000085883280
12.3	-9.7	-9.3	0.22	2.383	0.716	3.832	1.130	6	4105	ZINC000013382498
12.3	-11.0	-10.6	0.24	2.613	0.163	7.214	2.997	6	4106	ZINC000014829485

12.3	-12.5	-10.3	1.10	2.141	0.365	5.764	2.067	6	4107	ZINC000150368579
12.3	-9.9	-9.8	0.08	2.174	1.014	8.483	3.184	6	4108	ZINC000257435528
12.3	-9.2	-8.7	0.34	2.079	0.359	6.914	3.629	6	4109	ZINC000014811848
12.2	-10.5	-10.3	0.21	1.779	0.173	4.405	0.752	6	4110	ZINC000004096846
12.2	-9.4	-8.1	0.75	3.273	0.600	7.376	1.849	6	4111	ZINC000100045922
12.2	-11.7	-11.0	0.36	1.652	0.170	5.546	2.337	6	4112	ZINC000257615700
12.2	-9.9	-9.5	0.30	2.123	0.457	6.250	3.280	6	4113	ZINC000100829001
12.2	-10.6	-10.2	0.24	2.788	0.239	6.933	2.006	6	4114	ZINC000257649818
12.2	-10.7	-10.4	0.22	1.923	0.436	4.438	2.272	6	4115	ZINC000095619514
12.2	-9.4	-8.8	0.33	2.340	0.700	6.157	3.395	6	4116	ZINC000100773941
12.2	-11.0	-10.8	0.20	2.268	0.423	6.079	2.100	6	4117	ZINC000257536918
12.2	-11.1	-10.0	0.50	1.935	0.288	5.957	1.861	6	4118	ZINC000095619923
12.2	-11.1	-9.7	1.07	2.311	0.285	7.975	1.461	6	4119	ZINC000038139486
12.2	-11.2	-11.0	0.19	2.066	0.392	5.168	1.583	6	4120	ZINC000100829051
12.2	-10.9	-10.5	0.29	1.857	0.199	5.306	1.585	6	4121	ZINC000100781363

12.2	-10.2	-9.7	0.27	3.606	0.473	5.107	0.911	6	4122	ZINC000014813645
12.2	-11.2	-10.4	0.36	2.226	0.464	5.574	2.121	6	4123	ZINC000257507785
12.2	-11.1	-10.9	0.16	2.527	0.311	3.979	0.754	6	4124	ZINC000100825171
12.2	-9.5	-9.0	0.41	2.071	0.371	4.402	3.001	6	4125	ZINC000100771809
12.2	-12.3	-10.9	0.80	2.308	0.528	5.099	2.016	6	4126	ZINC000230100653
12.2	-9.2	-8.8	0.29	2.794	0.707	5.138	2.150	6	4127	ZINC000015260674
12.2	-10.1	-9.7	0.21	2.419	0.428	6.394	1.839	6	4128	ZINC000100777884
12.2	-12.0	-11.5	0.36	2.008	0.102	4.859	2.102	6	4129	ZINC000230066813
12.2	-10.4	-10.1	0.15	1.475	0.252	5.098	2.129	6	4130	ZINC000031495355
12.2	-11.0	-10.8	0.15	1.231	0.193	6.004	2.396	6	4131	ZINC000257467833
12.2	-11.9	-10.6	0.60	2.005	0.352	5.970	1.629	6	4132	ZINC000257449662
12.2	-10.9	-10.5	0.35	1.875	0.429	3.816	0.828	6	4133	ZINC000118937257
12.2	-10.9	-9.4	0.73	1.898	0.430	4.811	2.467	6	4134	ZINC000257478360
12.2	-10.1	-9.2	0.46	2.362	0.313	6.949	3.096	6	4135	ZINC000257407716
12.2	-11.4	-11.1	0.22	2.002	0.649	4.662	2.225	6	4136	ZINC000095619521

12.2	-11.9	-11.1	0.56	2.527	0.540	6.385	2.375	6	4137	ZINC000257804489
12.2	-10.2	-9.7	0.24	3.608	0.461	5.148	0.915	6	4138	ZINC000014813647
12.2	-11.2	-10.8	0.23	1.885	0.462	5.239	1.401	6	4139	ZINC000257383879
12.2	-11.1	-10.5	0.42	2.235	1.283	5.375	3.715	6	4140	ZINC000257398187
12.2	-10.9	-10.8	0.08	1.955	0.792	5.224	2.525	6	4141	ZINC000004097019
12.1	-9.9	-9.5	0.25	2.387	0.445	8.590	2.160	6	4142	ZINC000014931147
12.1	-9.6	-9.2	0.25	2.140	0.631	5.097	3.402	6	4143	ZINC000014781048
12.1	-11.3	-10.9	0.31	1.926	0.114	5.847	2.022	6	4144	ZINC000095619516
12.1	-9.4	-9.2	0.16	1.996	0.167	6.961	1.676	6	4145	ZINC000257546997
12.1	-11.1	-10.8	0.23	2.327	0.349	6.390	2.411	6	4146	ZINC000100827320
12.1	-10.0	-9.9	0.07	3.177	0.136	7.643	2.005	6	4147	ZINC000100773232
12.1	-10.7	-10.3	0.21	1.956	0.632	3.960	1.278	6	4148	ZINC000257472871
12.1	-11.1	-10.8	0.20	2.345	0.412	6.077	2.507	6	4149	ZINC000100827324
12.1	-9.4	-9.2	0.17	2.414	0.479	8.404	3.164	6	4150	ZINC000100825167
12.1	-10.7	-10.2	0.23	2.092	0.724	5.736	1.921	6	4151	ZINC000257422699

12.1	-10.4	-10.4	0.08	2.235	0.561	4.048	1.722	6	4152	ZINC000257568262
12.1	-10.0	-9.5	0.28	1.911	0.506	3.951	2.451	6	4153	ZINC000014610484
12.1	-9.7	-9.4	0.25	2.329	0.251	5.680	1.881	6	4154	ZINC000257462120
12.1	-11.2	-10.8	0.21	1.881	0.459	5.622	1.035	6	4155	ZINC000100829048
12.1	-10.3	-10.1	0.15	1.786	0.172	3.331	0.473	6	4156	ZINC000100825184
12.1	-10.0	-9.8	0.12	1.928	0.546	6.092	3.230	6	4157	ZINC000027755906
12.1	-11.0	-10.5	0.38	1.978	0.717	4.598	2.441	6	4158	ZINC000150368159
12.1	-10.4	-10.0	0.28	1.730	0.640	4.593	2.780	6	4159	ZINC000257377834
12.1	-11.3	-11.0	0.23	2.171	0.508	5.342	2.482	6	4160	ZINC000256110444
12.1	-9.6	-9.0	0.29	2.781	0.165	5.107	2.338	6	4161	ZINC000014728344
12.1	-11.1	-10.7	0.24	1.943	0.351	3.915	2.068	6	4162	ZINC000014612725
12.1	-10.0	-9.8	0.26	2.876	0.958	7.695	3.512	6	4163	ZINC000257504831
12.1	-10.7	-10.4	0.23	2.277	0.856	4.915	2.240	6	4164	ZINC000014089743
12.1	-10.4	-10.1	0.26	2.287	0.169	6.424	1.211	6	4165	ZINC000100890068
12.1	-12.0	-11.4	0.30	1.723	0.254	5.171	1.990	6	4166	ZINC000100778077

12.1	-11.6	-10.8	0.70	2.244	0.327	5.739	2.306	4	4167	ZINC000095617889
12.1	-12.5	-11.3	0.68	3.495	0.554	7.655	1.096	6	4168	ZINC000039166885
12.0	-9.3	-9.0	0.18	2.124	0.398	8.783	2.969	6	4169	ZINC000257493295
12.0	-10.6	-9.7	0.43	3.450	0.693	4.582	1.027	6	4170	ZINC000014728430
12.0	-11.1	-10.8	0.16	2.245	0.353	5.872	2.337	6	4171	ZINC000257422089
12.0	-11.0	-10.5	0.26	1.915	0.128	5.632	2.172	6	4172	ZINC000100779395
12.0	-10.5	-8.8	0.99	2.109	0.489	4.397	1.343	6	4173	ZINC000257464993
12.0	-11.5	-11.0	0.31	2.872	0.339	7.282	1.269	6	4174	ZINC000042806008
12.0	-9.9	-9.6	0.16	2.424	0.372	7.190	1.333	6	4175	ZINC000257405387
12.0	-10.4	-9.6	0.47	2.893	0.891	7.139	3.222	6	4176	ZINC000257398185
12.0	-11.4	-11.0	0.25	2.157	0.444	5.732	1.831	6	4177	ZINC000014720421
12.0	-10.0	-9.8	0.13	2.577	0.639	4.421	2.413	6	4178	ZINC000085552277
12.0	-11.9	-11.0	0.42	1.938	0.286	4.861	1.988	6	4179	ZINC000100779586
12.0	-11.1	-10.8	0.23	2.269	0.364	5.468	2.097	6	4180	ZINC000257422090
12.0	-9.8	-9.7	0.13	2.494	0.522	4.336	2.858	6	4181	ZINC000012496004

12.0	-11.1	-11.0	0.11	2.132	0.457	5.128	2.102	6	4182	ZINC000100827323
12.0	-11.6	-11.0	0.56	2.617	0.050	7.956	0.539	6	4183	ZINC000085954785
12.0	-9.3	-9.2	0.11	1.831	0.450	4.261	3.125	6	4184	ZINC000014760578
12.0	-10.5	-9.9	0.43	2.175	0.628	6.033	1.953	6	4185	ZINC000248328655
12.0	-11.1	-10.9	0.12	2.094	0.447	5.654	2.398	6	4186	ZINC000100827326
12.0	-10.6	-9.7	0.43	3.660	0.534	4.995	0.940	6	4187	ZINC000014728429
12.0	-10.7	-10.4	0.19	2.244	0.303	5.046	2.214	6	4188	ZINC000095910431
12.0	-11.1	-10.8	0.21	2.351	0.361	5.466	2.463	6	4189	ZINC000059765648
12.0	-10.9	-9.0	1.08	2.835	0.377	7.429	1.039	5	4190	ZINC000100078482
12.0	-10.7	-10.3	0.22	2.357	0.341	6.763	0.721	6	4191	ZINC000257422698
12.0	-11.0	-10.6	0.28	2.745	0.393	6.936	0.346	6	4192	ZINC000014720196
12.0	-10.1	-9.9	0.14	2.567	0.631	4.413	2.433	6	4193	ZINC000261496955
12.0	-11.1	-10.8	0.19	2.214	0.368	4.837	2.370	6	4194	ZINC000059765655
12.0	-9.4	-9.2	0.16	2.966	0.492	7.367	3.116	6	4195	ZINC000257411365
11.9	-10.7	-10.2	0.32	2.116	0.451	5.657	2.608	6	4196	ZINC000257462534

11.9	-10.3	-10.1	0.11	2.010	0.604	7.577	2.988	6	4197	ZINC000118936971
11.9	-11.0	-10.4	0.36	2.030	0.379	6.493	2.307	6	4198	ZINC000024215048
11.9	-9.6	-8.9	0.62	3.075	0.404	8.505	0.923	5	4199	ZINC000032787243
11.9	-10.9	-10.4	0.39	2.179	0.580	6.137	2.564	6	4200	ZINC000015106723
11.9	-10.9	-10.2	0.34	2.167	0.259	6.429	0.550	6	4201	ZINC000085954772
11.9	-10.3	-10.0	0.14	2.026	0.613	6.469	3.286	6	4202	ZINC000257421773
11.9	-10.1	-9.9	0.11	1.822	0.333	6.326	2.008	6	4203	ZINC000257444395
11.9	-11.4	-10.9	0.31	1.900	0.337	5.705	2.106	6	4204	ZINC000095619515
11.9	-9.8	-9.6	0.17	1.765	0.379	7.467	3.719	6	4205	ZINC000257477344
11.9	-10.3	-10.0	0.14	2.571	0.605	8.166	2.348	6	4206	ZINC000118936972
11.9	-11.1	-10.5	0.33	2.627	0.439	6.794	1.680	6	4207	ZINC000013414445
11.9	-10.8	-10.2	0.28	2.052	0.853	4.207	1.922	6	4208	ZINC000100829692
11.9	-11.6	-11.1	0.26	1.867	0.557	4.366	2.186	6	4209	ZINC000257658449
11.9	-10.4	-10.0	0.21	2.339	0.512	5.424	2.197	6	4210	ZINC000253500919
11.9	-10.3	-10.0	0.14	2.298	0.376	8.048	2.271	6	4211	ZINC000257421774

11.9	-11.6	-11.3	0.32	1.805	0.404	5.304	1.911	6	4212	ZINC000257561921
11.9	-9.7	-9.6	0.15	2.682	0.630	5.782	3.598	6	4213	ZINC000012495995
11.9	-10.3	-10.0	0.13	2.011	0.615	6.481	3.291	6	4214	ZINC000257421776
11.9	-10.8	-10.2	0.26	2.002	0.545	3.847	2.071	6	4215	ZINC000257385903
11.9	-11.8	-11.4	0.21	2.604	0.045	7.924	0.424	6	4216	ZINC000257552355
11.9	-9.9	-9.8	0.11	2.591	0.654	4.443	2.429	6	4217	ZINC000261496954
11.9	-9.6	-8.9	0.35	2.500	0.419	6.481	2.981	6	4218	ZINC000015261718
11.9	-11.2	-10.9	0.20	2.277	0.637	5.353	2.313	6	4219	ZINC000100823682
11.9	-10.7	-10.3	0.18	2.887	0.814	7.610	3.270	6	4220	ZINC000118937235
11.9	-10.8	-10.3	0.34	3.154	0.529	5.714	1.371	6	4221	ZINC000095619956
11.9	-11.1	-10.6	0.32	2.283	0.519	5.908	2.137	6	4222	ZINC000095619988
11.9	-11.1	-10.5	0.31	2.762	0.316	9.324	0.220	6	4223	ZINC000257455054
11.9	-10.0	-9.8	0.18	2.207	0.359	6.146	1.916	6	4224	ZINC000059736081
11.9	-10.0	-9.8	0.15	2.336	0.116	6.115	2.987	6	4225	ZINC000257498579
11.9	-10.6	-10.3	0.26	2.242	0.403	6.757	2.227	6	4226	ZINC000257517852

11.9	-10.3	-10.0	0.13	2.371	0.405	7.953	2.266	6	4227	ZINC000118936970
11.9	-10.8	-10.7	0.12	1.996	0.582	4.601	2.304	6	4228	ZINC000100823683
11.9	-10.3	-10.0	0.15	2.224	0.643	6.504	3.413	6	4229	ZINC000118936969
11.9	-8.8	-8.4	0.20	2.819	0.487	6.763	2.370	6	4230	ZINC000085946090
11.9	-10.8	-10.4	0.26	1.989	0.202	6.804	1.453	6	4231	ZINC000100778520
11.9	-11.0	-10.7	0.21	1.730	0.301	3.761	1.882	6	4232	ZINC000014610678
11.9	-10.3	-10.0	0.15	2.500	0.425	8.015	2.316	6	4233	ZINC000257421775
11.9	-12.4	-11.8	0.56	2.020	1.093	4.812	2.221	6	4234	ZINC000150359315
11.9	-10.7	-10.5	0.16	2.830	1.045	6.269	2.169	6	4235	ZINC000014617182
11.9	-10.5	-10.1	0.21	1.976	0.145	5.813	1.530	6	4236	ZINC000257546334
11.9	-9.7	-9.3	0.22	2.252	0.442	5.559	3.119	6	4237	ZINC000014558338
11.9	-11.3	-10.8	0.29	2.041	0.562	5.634	1.592	6	4238	ZINC000256507444
11.9	-12.7	-11.6	0.59	2.962	0.894	6.132	1.919	6	4239	ZINC000100825778
11.8	-9.8	-9.6	0.21	2.025	0.412	4.691	2.506	6	4240	ZINC000095620799
11.8	-10.6	-10.2	0.30	2.050	0.737	5.588	2.330	6	4241	ZINC000257580988

11.8	-10.7	-9.9	0.61	2.136	0.762	5.910	3.681	6	4242	ZINC000257445159
11.8	-10.0	-9.7	0.30	2.009	0.316	7.762	0.245	6	4243	ZINC000257451379
11.8	-10.9	-10.2	0.41	2.047	0.408	3.476	0.807	6	4244	ZINC000100829390
11.8	-9.6	-9.2	0.22	2.142	0.470	5.627	3.502	6	4245	ZINC000013132547
11.8	-9.9	-9.7	0.18	2.609	0.625	4.147	2.499	6	4246	ZINC000261496953
11.8	-10.1	-9.5	0.40	2.241	0.548	5.685	2.435	6	4247	ZINC000257559676
11.8	-9.4	-9.2	0.15	2.267	0.841	8.513	3.538	6	4248	ZINC000002566160
11.8	-11.1	-10.6	0.32	2.021	0.217	3.987	1.790	6	4249	ZINC000257516996
11.8	-11.5	-11.0	0.41	2.859	0.450	6.166	1.734	6	4250	ZINC000100779286
11.8	-10.4	-9.7	0.35	3.496	0.651	4.695	0.847	6	4251	ZINC000014806662
11.8	-9.9	-9.4	0.39	1.990	0.225	5.121	1.988	6	4252	ZINC000257399597
11.8	-9.6	-9.3	0.20	2.170	1.008	5.673	3.575	6	4253	ZINC000257374741
11.8	-9.9	-9.8	0.09	2.386	0.759	4.112	2.595	6	4254	ZINC000084386269
11.8	-8.9	-8.8	0.12	2.627	0.857	4.268	1.263	6	4255	ZINC000003830557
11.8	-10.6	-10.2	0.18	2.450	0.298	6.236	1.500	6	4256	ZINC000100826960

11.8	-9.9	-9.5	0.26	2.296	0.161	7.134	2.558	6	4257	ZINC000257567514
11.8	-10.0	-9.6	0.27	2.258	0.290	4.956	1.435	6	4258	ZINC000257435431
11.8	-14.4	-13.4	0.82	2.867	0.185	7.502	0.518	3	4259	ZINC000086041813
11.8	-10.7	-9.8	0.50	2.755	0.349	5.169	1.864	6	4260	ZINC000257547946
11.8	-11.5	-11.0	0.31	2.419	0.675	7.041	1.443	6	4261	ZINC000257508663
11.7	-11.5	-10.9	0.39	2.633	0.998	5.616	2.638	6	4262	ZINC000100779284
11.7	-11.0	-10.5	0.27	1.763	0.176	5.204	1.879	6	4263	ZINC000253589776
11.7	-10.1	-9.7	0.30	3.352	0.290	5.688	1.786	6	4264	ZINC000257693599
11.7	-10.3	-10.1	0.13	2.189	0.427	4.545	1.217	6	4265	ZINC000257462113
11.7	-10.3	-10.1	0.11	2.095	0.426	4.377	1.105	6	4266	ZINC000100774295
11.7	-11.0	-9.7	0.66	2.750	0.636	5.074	1.647	6	4267	ZINC000150351596
11.7	-10.9	-10.7	0.18	2.220	0.225	5.945	1.915	6	4268	ZINC000100825690
11.7	-9.5	-9.3	0.15	2.449	0.712	6.698	3.088	6	4269	ZINC000257453478
11.7	-10.7	-10.1	0.43	3.332	0.589	5.830	1.632	6	4270	ZINC000238733194
11.7	-9.6	-9.3	0.16	2.029	0.379	7.433	3.725	6	4271	ZINC000014760576

11.7	-11.0	-10.5	0.30	1.593	0.187	4.675	2.475	6	4272	ZINC000257417467
11.7	-10.7	-9.9	0.38	2.223	0.628	7.024	1.509	6	4273	ZINC000086044991
11.7	-11.6	-11.0	0.32	1.642	0.242	4.249	2.203	6	4274	ZINC000095619538
11.7	-11.3	-11.1	0.14	1.879	0.436	4.601	2.370	6	4275	ZINC000118937114
11.7	-10.3	-10.0	0.23	2.320	0.511	4.136	0.897	6	4276	ZINC000100774289
11.7	-10.3	-10.1	0.11	2.248	0.329	4.923	0.517	6	4277	ZINC000100774291
11.7	-9.6	-9.3	0.24	2.047	0.848	7.257	3.730	6	4278	ZINC000001530144
11.7	-9.4	-9.1	0.14	1.894	0.333	7.772	3.457	6	4279	ZINC000100829222
11.7	-10.3	-10.0	0.23	2.319	0.426	4.388	1.037	6	4280	ZINC000100774293
11.7	-11.0	-10.7	0.19	2.367	0.310	5.328	2.049	6	4281	ZINC000256080203
11.7	-9.9	-9.5	0.34	1.712	0.097	8.004	3.483	6	4282	ZINC000100829217
11.7	-10.6	-10.5	0.09	2.305	0.278	4.834	1.245	6	4283	ZINC000257427005
11.7	-11.6	-11.1	0.34	2.084	0.607	5.153	1.655	6	4284	ZINC000257658447
11.6	-11.5	-11.2	0.19	2.738	0.791	7.330	1.430	6	4285	ZINC000257480109
11.6	-10.6	-10.2	0.21	2.051	0.528	6.586	2.625	6	4286	ZINC000257410663

11.6	-11.0	-10.3	0.37	2.058	0.374	6.083	1.329	6	4287	ZINC000095619987
11.6	-10.4	-10.2	0.15	2.184	0.429	5.616	2.438	6	4288	ZINC000017545549
11.6	-10.0	-9.7	0.23	2.931	0.195	6.771	1.509	6	4289	ZINC000257423136
11.6	-11.4	-11.3	0.15	2.334	0.477	6.331	1.487	6	4290	ZINC000014720211
11.6	-10.8	-10.3	0.29	1.973	0.119	6.505	1.802	6	4291	ZINC000044405962
11.6	-10.0	-9.5	0.24	1.784	0.258	7.083	1.693	6	4292	ZINC000100779317
11.6	-11.2	-10.8	0.24	1.666	0.191	4.803	2.356	6	4293	ZINC000100780253
11.6	-8.5	-8.1	0.24	3.091	0.499	6.455	2.221	6	4294	ZINC000255974367
11.6	-11.5	-10.9	0.40	2.846	0.461	6.203	1.746	6	4295	ZINC000100779285
11.6	-11.6	-11.2	0.31	2.038	0.414	5.233	2.796	6	4296	ZINC000014951658
11.6	-10.2	-9.8	0.26	1.973	0.345	4.915	2.178	6	4297	ZINC000247978460
11.6	-10.8	-9.8	0.53	2.314	0.322	5.194	0.645	6	4298	ZINC000085561868
11.6	-10.3	-10.1	0.13	1.823	0.625	5.974	2.692	6	4299	ZINC000257640755
11.6	-11.2	-10.5	0.38	2.250	0.545	5.791	2.099	6	4300	ZINC000257565999
11.6	-8.8	-8.3	0.31	2.092	0.632	3.301	1.011	6	4301	ZINC000257803305

11.6	-11.3	-10.0	0.75	1.834	0.413	5.111	2.574	6	4302	ZINC000014693309
11.6	-11.9	-11.0	0.67	1.989	0.586	4.655	1.492	6	4303	ZINC000085532007
11.6	-10.0	-9.7	0.17	2.302	0.489	6.801	2.331	6	4304	ZINC000100827293
11.6	-12.1	-11.3	0.57	2.034	0.846	6.156	2.745	6	4305	ZINC000100775861
11.6	-10.3	-9.9	0.26	2.381	0.473	7.076	3.837	6	4306	ZINC000100829044
11.6	-9.1	-9.0	0.07	2.654	0.582	7.290	2.862	6	4307	ZINC000257533824
11.6	-11.2	-10.7	0.33	1.639	0.302	4.316	2.369	6	4308	ZINC000257615703
11.6	-10.3	-10.1	0.13	2.478	0.383	5.140	0.378	6	4309	ZINC000257462112
11.6	-11.6	-11.1	0.31	2.763	0.070	7.317	0.850	6	4310	ZINC000085774745
11.5	-10.7	-10.2	0.25	2.083	0.697	4.308	1.313	6	4311	ZINC000100824207
11.5	-10.0	-9.6	0.20	2.266	0.470	6.146	2.260	6	4312	ZINC000100827297
11.5	-11.3	-11.0	0.20	2.194	0.537	4.690	2.573	6	4313	ZINC000100825693
11.5	-9.9	-9.6	0.21	2.826	0.336	5.888	1.847	6	4314	ZINC000118937191
11.5	-10.6	-10.1	0.25	2.798	0.699	8.045	2.006	6	4315	ZINC000100824378
11.5	-10.9	-10.0	0.48	2.568	0.399	5.959	1.629	6	4316	ZINC000013373180

11.5	-9.6	-8.9	0.37	2.645	0.547	9.683	0.184	6	4317	ZINC000014781045
11.5	-11.4	-10.9	0.31	2.262	0.642	4.636	1.044	6	4318	ZINC000257395112
11.5	-10.6	-10.3	0.18	2.031	0.403	4.035	1.064	6	4319	ZINC000257407326
11.5	-10.0	-9.5	0.24	2.187	0.402	4.667	1.302	6	4320	ZINC000257783851
11.5	-10.6	-10.3	0.17	2.372	0.202	4.525	0.637	6	4321	ZINC000257407324
11.5	-12.1	-11.0	0.64	2.935	0.250	5.804	1.237	6	4322	ZINC000100822644
11.5	-11.0	-10.7	0.24	1.605	0.364	6.001	2.285	6	4323	ZINC000095619223
11.5	-11.2	-10.9	0.24	2.474	0.789	5.418	2.677	6	4324	ZINC000257472505
11.5	-9.9	-9.7	0.11	2.291	0.337	5.352	2.357	6	4325	ZINC000257414579
11.5	-10.9	-10.4	0.26	1.847	0.571	4.188	2.719	6	4326	ZINC000257378283
11.5	-9.1	-8.8	0.26	1.593	0.609	7.208	2.629	6	4327	ZINC000100824630
11.5	-10.8	-10.6	0.22	1.689	0.320	5.152	3.423	6	4328	ZINC000118937039
11.5	-10.0	-9.7	0.19	2.172	0.419	5.823	2.002	6	4329	ZINC000257783849
11.5	-9.6	-9.3	0.19	2.246	0.386	3.030	0.483	6	4330	ZINC000238750094
11.5	-11.5	-11.2	0.19	2.737	0.783	7.324	1.433	6	4331	ZINC000257480108

11.5	-9.6	-9.2	0.24	2.043	0.906	4.376	2.863	6	4332	ZINC000257374738
11.5	-11.3	-11.0	0.17	2.594	0.135	6.598	1.689	6	4333	ZINC000100825691
11.5	-11.1	-10.3	0.37	2.129	0.198	6.508	1.137	6	4334	ZINC000085954768
11.5	-10.5	-10.4	0.11	2.185	0.342	7.742	2.746	6	4335	ZINC000085541163
11.5	-10.0	-9.6	0.21	2.223	0.415	5.727	2.000	6	4336	ZINC000100827295
11.5	-10.7	-10.3	0.29	1.934	0.294	6.038	1.080	6	4337	ZINC000100823143
11.5	-10.0	-9.6	0.21	2.137	0.406	5.670	2.023	6	4338	ZINC000257783852
11.5	-11.5	-10.7	0.38	2.089	0.598	5.031	2.473	6	4339	ZINC000095619986
11.5	-10.5	-9.7	0.63	2.160	0.242	6.958	1.358	6	4340	ZINC000230091515
11.5	-10.6	-10.3	0.13	2.217	0.458	3.989	0.937	6	4341	ZINC000100774300
11.5	-9.9	-9.5	0.23	2.938	0.211	6.770	1.502	6	4342	ZINC000118937190
11.5	-10.6	-10.2	0.22	2.086	0.553	3.633	0.906	6	4343	ZINC000257407325
11.5	-10.6	-10.3	0.16	2.386	0.193	4.533	0.617	6	4344	ZINC000100774299
11.5	-11.0	-10.2	0.50	2.223	0.428	6.985	1.985	6	4345	ZINC000150364729
11.5	-10.7	-9.7	0.51	3.044	0.808	5.299	2.028	6	4346	ZINC000013382492

11.5	-8.7	-8.4	0.26	2.197	0.929	8.625	3.404	6	4347	ZINC000008662733
11.5	-11.3	-11.0	0.22	2.283	0.420	5.961	1.977	6	4348	ZINC000256110447
11.5	-10.8	-10.3	0.28	2.094	0.124	5.792	1.656	6	4349	ZINC000257621914
11.5	-10.0	-9.6	0.20	2.231	0.450	5.935	2.220	6	4350	ZINC000100827296
11.4	-9.5	-9.1	0.19	2.097	0.481	4.704	2.774	6	4351	ZINC000013328774
11.4	-11.2	-10.8	0.21	1.879	0.460	5.622	1.031	6	4352	ZINC000100829049
11.4	-11.2	-10.9	0.21	2.149	0.630	5.266	2.472	6	4353	ZINC000100779424
11.4	-9.6	-9.2	0.22	2.279	1.051	6.856	3.687	6	4354	ZINC000100823213
11.4	-10.6	-10.3	0.19	2.194	0.486	4.326	1.038	6	4355	ZINC000100774305
11.4	-9.8	-9.4	0.31	2.054	0.263	7.106	2.170	6	4356	ZINC000257405385
11.4	-10.0	-9.7	0.16	2.133	0.252	5.670	1.419	6	4357	ZINC000085744724
11.4	-10.0	-9.3	0.34	2.321	0.684	4.541	2.716	6	4358	ZINC000014728340
11.4	-9.8	-9.5	0.21	2.857	0.329	6.457	1.923	6	4359	ZINC000257423138
11.4	-10.9	-10.2	0.36	2.079	0.347	5.001	1.852	6	4360	ZINC000100780136
11.4	-10.3	-9.9	0.21	2.291	0.418	6.721	1.242	6	4361	ZINC000257477723

11.4	-10.2	-9.8	0.22	1.880	0.610	6.249	3.921	6	4362	ZINC000049833293
11.4	-10.6	-10.2	0.24	2.298	0.208	5.183	1.478	6	4363	ZINC000257472870
11.4	-10.6	-10.3	0.14	2.055	0.523	3.587	1.255	6	4364	ZINC000257407323
11.4	-11.2	-10.6	0.41	2.245	0.450	6.119	3.232	6	4365	ZINC000257462535
11.4	-10.8	-10.6	0.11	1.680	0.591	3.698	2.118	6	4366	ZINC000095619519
11.4	-10.8	-10.2	0.33	3.711	0.044	10.196	0.219	6	4367	ZINC000257805857
11.4	-10.5	-10.2	0.21	1.876	0.182	6.340	1.840	6	4368	ZINC000257456729
11.4	-10.6	-10.4	0.14	1.964	0.649	4.081	1.391	6	4369	ZINC000100824206
11.4	-9.6	-9.2	0.26	2.466	1.007	7.056	3.579	6	4370	ZINC000257374739
11.4	-9.2	-9.1	0.06	2.108	0.789	6.047	3.475	6	4371	ZINC000100773234
11.4	-10.1	-9.8	0.17	2.172	0.488	6.488	1.248	6	4372	ZINC000248328868
11.4	-10.0	-9.7	0.21	2.554	0.377	6.869	2.241	6	4373	ZINC000118937230
11.4	-9.0	-8.6	0.24	1.745	0.279	7.105	2.541	6	4374	ZINC000257512430
11.4	-10.0	-9.8	0.13	2.260	0.484	3.977	0.820	6	4375	ZINC000257462115
11.4	-10.6	-10.2	0.20	2.091	0.442	3.672	1.106	6	4376	ZINC000100774304

11.4	-9.6	-9.2	0.24	2.183	0.929	5.666	3.435	6	4377	ZINC000100823212
11.4	-10.4	-10.1	0.21	1.681	0.368	2.732	0.936	6	4378	ZINC000257546979
11.4	-9.6	-9.2	0.29	2.638	0.855	5.190	2.745	6	4379	ZINC000013397720
11.4	-10.7	-10.5	0.18	2.175	0.489	4.734	2.327	6	4380	ZINC000257466734
11.4	-9.7	-9.4	0.19	2.157	0.319	3.390	0.834	6	4381	ZINC000100773931
11.4	-11.8	-10.2	0.86	2.467	0.228	6.608	0.217	6	4382	ZINC000100824458
11.4	-11.2	-10.8	0.21	1.797	0.588	4.717	1.888	6	4383	ZINC000257383880
11.4	-10.2	-9.7	0.25	1.962	0.468	5.996	2.655	6	4384	ZINC000257470620
11.4	-9.3	-9.0	0.18	1.729	0.526	3.844	2.717	6	4385	ZINC000257605397
11.4	-10.1	-9.8	0.28	1.740	0.126	6.061	1.941	6	4386	ZINC000257048101
11.4	-9.0	-8.8	0.21	1.564	0.553	4.921	2.783	6	4387	ZINC000100824632
11.4	-9.0	-8.7	0.25	1.958	0.445	6.455	2.291	6	4388	ZINC000257512428
11.4	-9.9	-9.7	0.16	2.172	0.420	5.824	2.004	6	4389	ZINC000257783853
11.4	-11.1	-10.8	0.20	2.270	0.360	4.782	1.882	6	4390	ZINC000095619171
11.3	-11.3	-10.8	0.38	2.348	0.382	4.926	1.538	6	4391	ZINC000119394826

11.3	-10.0	-9.8	0.20	2.324	0.589	3.944	1.014	6	4392	ZINC000257462114
11.3	-10.8	-10.2	0.28	2.183	0.427	5.429	2.331	6	4393	ZINC000257477217
11.3	-10.6	-10.3	0.23	2.883	0.462	8.071	2.866	6	4394	ZINC000238743139
11.3	-13.3	-12.4	0.43	2.474	0.513	6.273	1.272	6	4395	ZINC000085561975
11.3	-11.3	-10.8	0.36	1.689	0.112	4.738	1.991	6	4396	ZINC000100783636
11.3	-10.4	-10.0	0.21	1.944	0.284	5.937	2.114	6	4397	ZINC000100777906
11.3	-9.1	-8.9	0.20	1.640	0.660	6.150	2.747	6	4398	ZINC000257512427
11.3	-9.8	-9.3	0.27	2.568	0.698	9.029	2.355	6	4399	ZINC000014811818
11.3	-11.3	-11.0	0.21	2.040	0.284	4.306	1.176	6	4400	ZINC000100829058
11.3	-11.3	-10.8	0.31	2.382	0.286	5.673	2.451	6	4401	ZINC000257404248
11.3	-10.5	-10.0	0.26	2.597	1.117	5.525	3.577	6	4402	ZINC000257392247
11.3	-11.9	-10.9	0.47	1.933	0.545	5.454	2.317	6	4403	ZINC000257541177
11.3	-9.5	-8.8	0.45	2.494	0.335	6.419	2.780	6	4404	ZINC000014757411
11.3	-10.2	-9.9	0.16	2.232	0.369	6.940	1.538	6	4405	ZINC000100826553
11.3	-9.6	-9.1	0.31	2.173	0.896	5.722	3.541	6	4406	ZINC000100823217

11.3	-9.0	-8.6	0.25	2.159	0.743	8.643	0.408	6	4407	ZINC000257512429
11.3	-9.2	-9.0	0.20	1.803	0.624	4.555	2.101	6	4408	ZINC000256079723
11.3	-9.5	-9.1	0.27	2.330	0.592	5.666	3.351	6	4409	ZINC000257549821
11.3	-9.1	-8.8	0.24	1.278	0.444	5.836	3.143	6	4410	ZINC000100824633
11.3	-11.3	-11.0	0.22	2.119	0.446	5.496	1.998	6	4411	ZINC000100829062
11.3	-12.7	-11.6	0.68	2.350	0.255	6.118	1.927	6	4412	ZINC000086041817
11.3	-10.2	-9.9	0.20	2.382	0.403	6.340	1.148	6	4413	ZINC000257477726
11.3	-11.1	-10.4	0.39	2.349	0.601	5.188	1.715	6	4414	ZINC000257446299
11.3	-9.2	-8.5	0.33	2.959	0.411	7.684	2.713	6	4415	ZINC000257423025
11.3	-9.8	-9.5	0.24	3.525	0.629	4.849	0.910	6	4416	ZINC000013379902
11.3	-11.3	-10.9	0.27	2.403	0.221	6.406	1.833	6	4417	ZINC000100829060
11.3	-10.5	-10.3	0.13	2.290	0.317	8.067	1.813	6	4418	ZINC000085862067
11.3	-10.2	-9.9	0.17	1.909	0.180	4.157	2.206	6	4419	ZINC000073170142
11.3	-9.6	-9.2	0.22	2.070	0.992	4.324	2.902	6	4420	ZINC000257374740
11.3	-8.8	-8.7	0.11	1.915	0.390	6.589	2.373	6	4421	ZINC000085587788

11.3	-11.2	-11.0	0.19	1.956	0.362	5.546	2.027	6	4422	ZINC000100779398
11.3	-11.3	-10.8	0.24	2.687	0.234	7.249	1.524	6	4423	ZINC000059733047
11.3	-10.1	-9.8	0.18	2.422	0.356	6.855	1.162	6	4424	ZINC000257437254
11.3	-10.3	-10.0	0.17	1.808	0.088	6.750	1.250	6	4425	ZINC000247973602
11.3	-9.0	-8.6	0.27	1.860	0.651	6.496	2.597	6	4426	ZINC000100824628
11.3	-10.9	-10.4	0.27	2.292	0.490	4.966	2.272	6	4427	ZINC000150601222
11.3	-9.8	-9.5	0.23	3.115	0.297	6.912	1.330	6	4428	ZINC000257423139
11.3	-10.2	-10.0	0.14	2.435	0.391	6.495	1.597	6	4429	ZINC000257477724
11.3	-10.7	-10.5	0.14	1.728	0.551	4.128	1.958	6	4430	ZINC000014953943
11.3	-11.3	-10.9	0.33	2.760	1.224	7.020	3.640	6	4431	ZINC000257398186
11.3	-9.0	-8.7	0.20	2.290	0.394	4.943	2.416	6	4432	ZINC000257437482
11.3	-9.6	-9.2	0.26	1.733	0.593	3.061	0.634	6	4433	ZINC000100823215
11.3	-10.3	-10.1	0.24	3.216	0.242	7.073	0.776	6	4434	ZINC000257528518
11.3	-11.2	-10.8	0.29	2.026	0.464	4.230	2.047	6	4435	ZINC000253500790
11.3	-10.6	-10.3	0.14	1.952	0.611	4.069	1.364	6	4436	ZINC000257472868

11.2	-9.5	-9.0	0.39	1.858	0.590	4.025	2.519	6	4437	ZINC000257549822
11.2	-10.6	-10.2	0.41	1.771	0.158	5.100	2.288	6	4438	ZINC000257767189
11.2	-9.9	-9.3	0.31	2.030	0.369	5.674	3.554	6	4439	ZINC000013813075
11.2	-10.2	-9.9	0.15	2.529	0.365	6.183	1.976	6	4440	ZINC000257477725
11.2	-12.4	-11.4	0.62	2.266	0.294	6.080	1.145	6	4441	ZINC000169316283
11.2	-11.2	-10.8	0.30	2.197	0.455	5.517	2.358	6	4442	ZINC000257404249
11.2	-11.4	-10.6	0.40	1.722	0.412	4.984	2.533	6	4443	ZINC000100781603
11.2	-11.1	-10.7	0.24	2.446	0.483	4.845	1.850	6	4444	ZINC000257568259
11.2	-9.9	-9.8	0.11	1.795	0.460	5.275	2.457	6	4445	ZINC000100829380
11.2	-11.1	-10.9	0.15	2.183	0.414	5.720	1.998	6	4446	ZINC000100826017
11.2	-11.6	-11.0	0.46	1.627	0.422	4.473	2.393	6	4447	ZINC000257491954
11.2	-11.4	-10.9	0.35	2.614	0.796	5.501	1.498	6	4448	ZINC000100779476
11.2	-11.4	-10.9	0.32	3.332	0.305	6.297	1.640	6	4449	ZINC000257508660
11.2	-10.9	-10.6	0.18	2.451	0.373	5.635	2.179	6	4450	ZINC000256110451
11.2	-11.1	-10.2	0.43	2.347	0.177	6.260	1.139	6	4451	ZINC000085662192

11.2	-11.0	-10.5	0.24	2.216	0.614	6.030	0.147	6	4452	ZINC000100822778
11.2	-9.6	-9.5	0.06	1.955	0.361	7.312	2.615	6	4453	ZINC000100824735
11.2	-8.5	-8.2	0.20	3.574	0.450	7.431	1.507	6	4454	ZINC000006018661
11.2	-11.3	-10.7	0.36	2.457	0.823	5.977	2.589	6	4455	ZINC000150368575
11.2	-10.0	-9.7	0.24	2.084	0.141	6.112	2.251	6	4456	ZINC000150364737
11.2	-10.5	-10.2	0.18	2.509	0.240	6.353	1.113	6	4457	ZINC000257454107
11.2	-10.8	-10.2	0.35	2.305	0.206	5.783	2.755	6	4458	ZINC000257419091
11.2	-10.4	-10.1	0.15	2.299	0.496	5.187	2.333	6	4459	ZINC000017545559
11.2	-10.2	-9.8	0.20	2.018	0.357	4.253	1.360	6	4460	ZINC000257462121
11.2	-10.4	-10.1	0.16	2.326	0.301	7.841	1.855	6	4461	ZINC000100824733
11.2	-10.1	-9.9	0.16	2.418	0.159	6.293	1.381	6	4462	ZINC000100826559
11.2	-11.5	-11.1	0.26	2.370	0.394	6.422	1.642	6	4463	ZINC000100779474
11.2	-11.2	-10.9	0.25	2.443	0.193	5.751	2.418	6	4464	ZINC000100829061
11.2	-10.7	-10.3	0.27	1.918	0.151	5.399	2.073	6	4465	ZINC000257767188
11.2	-10.7	-10.5	0.12	1.621	0.150	5.174	2.417	6	4466	ZINC000095619221

11.2	-10.8	-10.4	0.21	2.133	0.383	4.355	1.578	6	4467	ZINC000100778523
11.2	-10.2	-9.9	0.17	2.523	0.363	7.022	1.528	6	4468	ZINC000100826551
11.2	-9.9	-9.7	0.13	1.968	0.155	6.957	2.082	6	4469	ZINC000100773901
11.2	-11.9	-11.5	0.32	2.293	0.643	5.473	2.686	6	4470	ZINC000085648919
11.2	-11.4	-11.0	0.29	3.077	0.647	6.384	1.498	6	4471	ZINC000100779473
11.2	-11.4	-10.8	0.36	2.273	0.558	5.285	2.146	6	4472	ZINC000200417732
11.2	-9.4	-9.1	0.16	2.382	0.527	6.016	3.380	6	4473	ZINC000014779801
11.2	-10.7	-9.5	0.65	2.487	0.434	6.324	0.614	6	4474	ZINC000257501130
11.2	-10.9	-10.0	0.53	2.581	0.433	6.139	1.437	6	4475	ZINC000013373179
11.1	-10.9	-10.6	0.25	1.863	0.634	4.982	2.250	6	4476	ZINC000253500787
11.1	-10.6	-10.0	0.35	3.101	0.752	8.404	2.918	6	4477	ZINC000257546981
11.1	-10.0	-9.8	0.16	1.860	0.096	7.511	0.079	6	4478	ZINC000100779319
11.1	-9.5	-9.1	0.18	2.141	0.172	6.280	2.176	6	4479	ZINC000118937189
11.1	-11.9	-10.6	0.60	1.862	0.510	5.422	2.364	6	4480	ZINC000257615702
11.1	-9.2	-9.0	0.21	2.037	0.378	5.021	2.431	6	4481	ZINC000100773946

11.1	-9.5	-9.1	0.24	2.262	0.044	6.162	2.328	6	4482	ZINC000257423137
11.1	-10.3	-10.0	0.19	2.854	0.366	6.764	0.890	6	4483	ZINC000015273917
11.1	-9.5	-9.3	0.16	3.150	0.439	8.089	2.214	6	4484	ZINC000257445160
11.1	-10.9	-10.3	0.41	1.810	0.145	5.602	2.340	6	4485	ZINC000014720320
11.1	-10.5	-9.4	0.58	2.440	0.389	4.870	2.161	6	4486	ZINC000257498576
11.1	-10.1	-9.8	0.18	2.127	0.684	5.605	2.359	6	4487	ZINC000100826555
11.1	-10.3	-10.0	0.23	2.551	0.104	8.111	0.747	6	4488	ZINC000100825663
11.1	-10.8	-10.2	0.30	2.584	0.281	7.387	1.294	6	4489	ZINC000086041845
11.1	-9.9	-9.5	0.23	2.102	0.228	6.093	1.638	6	4490	ZINC000257470913
11.1	-10.5	-10.1	0.21	2.032	0.176	5.630	2.163	6	4491	ZINC000247973597
11.1	-11.3	-10.9	0.32	2.953	0.854	6.983	1.134	6	4492	ZINC000257508661
11.1	-11.4	-11.0	0.29	3.085	0.638	6.380	1.496	6	4493	ZINC000257508662
11.1	-9.2	-8.9	0.15	2.698	0.740	3.951	0.744	6	4494	ZINC000059765124
11.1	-10.3	-10.1	0.24	2.593	0.164	8.361	0.593	6	4495	ZINC000257628154
11.1	-11.2	-11.0	0.15	2.005	0.431	5.069	2.182	6	4496	ZINC000257472504

11.1	-9.4	-9.1	0.14	3.027	0.130	9.508	0.124	6	4497	ZINC000257476594
11.1	-10.8	-10.2	0.33	1.848	0.298	5.854	1.746	6	4498	ZINC000100773685
11.1	-10.2	-9.9	0.19	2.418	0.107	7.711	0.949	6	4499	ZINC000257421408
11.1	-10.3	-10.2	0.14	2.567	0.153	8.406	0.534	6	4500	ZINC000100824979
11.1	-10.4	-9.9	0.24	2.489	0.177	7.108	0.261	6	4501	ZINC000100824467
11.1	-9.4	-9.2	0.08	1.931	0.365	3.012	1.179	6	4502	ZINC000013382497
11.1	-10.7	-10.3	0.24	2.960	0.378	5.178	2.188	6	4503	ZINC000013411952
11.1	-10.5	-10.2	0.19	1.748	0.256	4.806	1.786	6	4504	ZINC000257546333
11.0	-10.3	-10.2	0.17	2.621	0.184	8.452	0.488	6	4505	ZINC000100824982
11.0	-10.3	-10.2	0.14	2.614	0.184	8.453	0.483	6	4506	ZINC000257628155
11.0	-10.0	-9.7	0.27	2.838	0.495	8.682	3.317	6	4507	ZINC000253500828
11.0	-10.2	-9.8	0.22	1.901	0.195	6.865	1.501	6	4508	ZINC000014720317
11.0	-10.1	-9.0	0.69	1.905	0.259	4.659	2.020	6	4509	ZINC000100822466
11.0	-10.9	-10.7	0.24	2.032	0.733	5.607	2.693	6	4510	ZINC000017545485
11.0	-12.0	-10.8	0.64	3.125	0.386	5.957	2.309	6	4511	ZINC000031415836

11.0	-9.8	-9.2	0.32	1.846	0.564	6.849	3.795	6	4512	ZINC000100773936
11.0	-10.3	-10.2	0.07	2.635	0.184	8.257	0.502	6	4513	ZINC000257628153
11.0	-9.6	-9.2	0.23	1.952	0.058	9.104	0.104	6	4514	ZINC000257775071
11.0	-11.4	-10.7	0.42	2.036	0.317	4.764	2.331	6	4515	ZINC000100822678
11.0	-10.1	-9.7	0.24	2.034	0.699	6.556	2.849	6	4516	ZINC000256080206
11.0	-10.2	-10.0	0.13	2.190	0.260	6.797	1.276	6	4517	ZINC000257421407
11.0	-10.8	-9.8	0.46	2.671	0.724	5.665	1.750	6	4518	ZINC000257375888
11.0	-10.3	-10.0	0.20	2.154	0.709	6.629	2.540	6	4519	ZINC000100825667
11.0	-11.1	-10.8	0.24	1.829	0.361	3.769	1.976	6	4520	ZINC000014513023
11.0	-10.3	-10.1	0.24	2.594	0.167	8.337	0.622	6	4521	ZINC000100824981
11.0	-9.3	-8.8	0.24	2.354	0.656	7.042	3.830	6	4522	ZINC000238750729
11.0	-11.6	-11.4	0.17	2.804	0.312	7.980	0.210	6	4523	ZINC000100775864
11.0	-10.8	-10.2	0.31	2.222	0.303	6.277	2.222	6	4524	ZINC000015117349
11.0	-10.1	-9.4	0.46	2.222	0.446	3.484	1.473	6	4525	ZINC000257445161
11.0	-9.4	-9.0	0.26	2.502	0.943	4.841	2.182	6	4526	ZINC000059588219

11.0	-10.3	-10.1	0.24	2.603	0.169	8.345	0.614	6	4527	ZINC000257628152
11.0	-9.3	-9.1	0.11	2.362	0.521	5.664	3.183	6	4528	ZINC000257445162
11.0	-9.8	-9.4	0.24	1.925	0.273	4.917	2.410	6	4529	ZINC000100827887
11.0	-10.3	-10.1	0.19	2.037	0.567	6.228	2.294	6	4530	ZINC000118937405
11.0	-9.0	-8.9	0.12	1.552	0.637	4.388	1.971	6	4531	ZINC000100247849
11.0	-9.5	-9.0	0.32	3.377	1.016	6.499	2.360	6	4532	ZINC000095619150
11.0	-11.2	-11.0	0.21	2.168	0.389	4.886	1.895	6	4533	ZINC000100779571
11.0	-10.3	-10.0	0.25	2.399	0.353	7.009	1.578	6	4534	ZINC000100825665
11.0	-10.6	-10.2	0.25	1.687	0.184	4.801	2.446	6	4535	ZINC000100823663
11.0	-10.4	-10.1	0.21	2.251	0.233	4.886	1.029	6	4536	ZINC000100826884
11.0	-10.5	-9.9	0.31	2.162	0.481	3.121	0.991	6	4537	ZINC000257547948
11.0	-11.3	-11.0	0.27	3.098	0.610	6.391	1.499	6	4538	ZINC000100779470
11.0	-10.5	-10.2	0.18	2.567	0.254	5.853	1.312	6	4539	ZINC000100826887
11.0	-10.3	-10.1	0.18	2.443	0.386	7.688	1.491	6	4540	ZINC000100824980
11.0	-10.5	-10.4	0.05	1.813	0.625	4.389	2.263	6	4541	ZINC000254685400

11.0	-9.4	-9.0	0.26	2.997	1.093	5.925	2.659	6	4542	ZINC000095619151
11.0	-9.6	-9.2	0.27	2.607	0.570	4.795	2.363	6	4543	ZINC000085956002
11.0	-9.1	-8.8	0.22	2.565	0.429	7.704	3.802	6	4544	ZINC000257472650
10.9	-9.4	-8.9	0.34	3.465	1.054	6.520	2.290	6	4545	ZINC000095619149
10.9	-10.9	-10.5	0.26	2.147	0.251	4.526	1.168	6	4546	ZINC000257435096
10.9	-10.1	-9.7	0.22	1.996	0.613	5.714	2.447	6	4547	ZINC000256080209
10.9	-11.0	-10.7	0.25	2.185	0.425	5.653	2.045	6	4548	ZINC000100825655
10.9	-10.3	-9.7	0.29	2.147	0.387	6.474	2.763	6	4549	ZINC000100822247
10.9	-10.7	-10.0	0.35	1.720	0.214	4.199	1.605	6	4550	ZINC000257402140
10.9	-10.8	-10.5	0.24	2.305	0.380	4.776	2.330	6	4551	ZINC000017545483
10.9	-9.6	-9.2	0.22	2.672	0.674	5.438	2.439	6	4552	ZINC000004416136
10.9	-10.4	-10.1	0.21	2.254	0.346	6.085	1.769	6	4553	ZINC000257808145
10.9	-9.0	-8.9	0.11	1.449	0.610	3.207	0.820	6	4554	ZINC000100247855
10.9	-11.2	-10.8	0.20	1.383	0.194	3.993	2.630	6	4555	ZINC000257485976
10.9	-10.4	-10.1	0.21	2.274	0.425	5.983	2.292	6	4556	ZINC000257618447

10.9	-11.6	-11.1	0.27	1.728	0.281	4.504	1.803	6	4557	ZINC000257453496
10.9	-10.9	-10.3	0.26	2.636	0.504	9.412	2.151	6	4558	ZINC000257431094
10.9	-10.3	-9.8	0.23	2.093	0.266	6.647	2.053	6	4559	ZINC000257394919
10.9	-10.2	-9.9	0.22	2.441	0.340	6.405	3.435	6	4560	ZINC000257528197
10.9	-11.0	-10.0	0.49	2.134	0.246	6.170	0.747	6	4561	ZINC000014921514
10.9	-10.1	-9.8	0.18	2.036	0.649	6.125	2.577	6	4562	ZINC000100825839
10.9	-11.8	-11.0	0.45	2.721	0.575	5.621	1.768	6	4563	ZINC000100828145
10.9	-11.1	-10.7	0.22	2.242	0.479	4.526	2.120	6	4564	ZINC000257494290
10.9	-11.5	-10.8	0.35	1.911	0.458	5.056	1.856	6	4565	ZINC000086035344
10.9	-10.8	-10.5	0.24	2.503	0.362	5.978	2.278	6	4566	ZINC000253500791
10.9	-10.4	-10.0	0.17	2.021	0.609	4.896	1.888	6	4567	ZINC000257422696
10.9	-11.2	-10.6	0.33	2.009	0.417	3.801	0.731	6	4568	ZINC000257517853
10.9	-10.1	-9.8	0.16	2.042	0.630	6.164	2.581	6	4569	ZINC000256080211
10.9	-11.1	-10.7	0.29	2.231	0.418	5.046	1.530	6	4570	ZINC000017545581
10.9	-10.0	-9.7	0.24	2.059	0.197	5.946	1.426	6	4571	ZINC000257387715

10.9	-10.9	-10.6	0.22	2.088	0.239	6.244	1.385	6	4572	ZINC000100257275
10.8	-10.9	-10.4	0.29	2.276	0.342	5.086	0.984	6	4573	ZINC000257435094
10.8	-11.0	-10.3	0.37	2.429	0.475	7.347	0.514	6	4574	ZINC000026189526
10.8	-10.5	-10.1	0.31	1.869	0.291	6.579	3.396	6	4575	ZINC000033833262
10.8	-10.1	-9.7	0.23	2.414	0.191	7.656	1.221	6	4576	ZINC000100825837
10.8	-11.2	-10.7	0.27	1.736	0.143	5.582	1.588	6	4577	ZINC000014513026
10.8	-9.5	-9.2	0.15	4.178	0.260	6.877	2.027	6	4578	ZINC000014757344
10.8	-10.2	-9.9	0.25	2.152	0.300	5.255	2.291	6	4579	ZINC000257382700
10.8	-9.7	-9.2	0.34	2.327	0.277	5.214	2.269	6	4580	ZINC000257454349
10.8	-10.0	-9.6	0.25	2.539	0.878	6.563	3.599	6	4581	ZINC000118936878
10.8	-10.6	-10.4	0.13	2.272	0.359	4.401	1.787	6	4582	ZINC000257435428
10.8	-11.7	-11.3	0.28	2.201	0.415	6.516	1.989	6	4583	ZINC000085744663
10.8	-11.0	-10.7	0.23	1.667	0.153	3.981	1.423	6	4584	ZINC000257485978
10.8	-9.0	-8.5	0.28	1.866	0.396	5.636	3.794	6	4585	ZINC000257416098
10.8	-10.8	-10.5	0.30	2.112	0.505	4.971	2.357	6	4586	ZINC000015117345

10.8	-10.9	-10.8	0.10	2.079	0.337	5.528	1.915	6	4587	ZINC000100825662
10.8	-10.3	-10.1	0.11	2.607	0.521	8.110	2.276	6	4588	ZINC000257710434
10.8	-9.8	-9.3	0.43	2.661	0.600	7.211	2.417	6	4589	ZINC000257530290
10.8	-11.0	-10.7	0.17	2.470	0.361	5.455	1.668	6	4590	ZINC000253500918
10.8	-11.7	-10.6	0.50	1.888	0.128	5.475	1.730	6	4591	ZINC000015148339
10.8	-11.0	-10.3	0.46	1.535	0.323	3.134	2.654	6	4592	ZINC000118937404
10.8	-10.5	-10.4	0.14	1.878	0.207	5.706	2.722	6	4593	ZINC000257406859
10.8	-11.5	-10.9	0.36	2.268	0.407	5.569	1.712	6	4594	ZINC000257446300
10.8	-10.1	-9.7	0.26	2.590	0.258	7.564	1.162	6	4595	ZINC000100825836
10.7	-9.2	-9.0	0.16	1.939	0.592	3.167	1.059	6	4596	ZINC000118936829
10.7	-10.9	-10.4	0.29	2.360	0.282	5.846	0.756	6	4597	ZINC000257435093
10.7	-10.9	-10.5	0.24	2.097	0.120	5.113	2.134	6	4598	ZINC000257435095
10.7	-10.9	-10.5	0.23	2.282	0.382	6.229	2.266	6	4599	ZINC000100829083
10.7	-11.3	-11.0	0.22	2.399	0.112	6.198	1.050	6	4600	ZINC000100780311
10.7	-8.0	-7.8	0.21	2.054	1.100	5.283	4.041	6	4601	ZINC000013374806

10.7	-11.1	-10.4	0.39	2.276	0.646	6.438	0.718	6	4602	ZINC000100257268
10.7	-9.1	-8.8	0.17	1.947	0.465	2.793	0.522	6	4603	ZINC000257408603
10.7	-10.4	-9.7	0.34	3.728	0.541	4.965	0.862	6	4604	ZINC000014806656
10.7	-11.0	-10.5	0.28	2.934	0.857	5.994	2.650	6	4605	ZINC000100827883
10.7	-11.0	-10.5	0.22	2.513	0.316	6.458	2.241	6	4606	ZINC000253500920
10.7	-11.8	-10.4	0.90	2.148	0.242	5.825	1.370	6	4607	ZINC000026189535
10.7	-8.9	-8.7	0.10	2.872	0.635	6.128	1.846	6	4608	ZINC000014718954
10.7	-10.4	-9.7	0.45	2.587	0.866	3.926	1.103	6	4609	ZINC000257513024
10.7	-8.0	-7.8	0.15	3.232	0.828	5.573	2.805	6	4610	ZINC000012496045
10.7	-9.7	-9.4	0.17	2.094	0.601	6.025	3.128	6	4611	ZINC000257455625
10.7	-10.9	-10.5	0.26	2.276	0.278	5.213	1.244	6	4612	ZINC000100829081
10.7	-12.4	-11.5	0.51	2.583	0.663	7.310	1.577	6	4613	ZINC000100779156
10.7	-11.6	-10.9	0.41	1.994	0.430	5.205	1.944	6	4614	ZINC000014720217
10.7	-9.0	-8.9	0.09	3.025	0.275	6.493	3.088	6	4615	ZINC000257377502
10.7	-10.3	-9.9	0.24	2.436	0.108	7.774	1.060	6	4616	ZINC000257529701

10.7	-11.2	-10.5	0.35	1.758	0.244	4.803	1.968	6	4617	ZINC000257615701
10.7	-9.7	-9.5	0.19	2.074	0.499	4.121	2.234	6	4618	ZINC000014610486
10.7	-11.2	-11.0	0.12	1.883	0.399	6.621	2.183	6	4619	ZINC000150602720
10.7	-9.7	-9.3	0.27	2.354	0.757	7.394	3.830	6	4620	ZINC000257547097
10.7	-10.9	-10.5	0.23	2.403	0.378	6.771	1.802	6	4621	ZINC000100829076
10.7	-11.2	-10.1	0.56	2.357	0.683	4.446	2.330	6	4622	ZINC000001849810
10.7	-10.9	-10.5	0.25	2.133	0.258	4.511	1.149	6	4623	ZINC000100829078
10.7	-11.4	-10.5	0.39	2.064	0.204	4.962	1.901	6	4624	ZINC000253589780
10.7	-11.9	-11.4	0.37	1.877	0.399	4.762	1.842	6	4625	ZINC000095619537
10.7	-10.7	-10.4	0.19	2.130	0.192	5.967	1.186	6	4626	ZINC000100825661
10.7	-9.2	-9.0	0.18	2.844	0.875	7.417	3.650	6	4627	ZINC000008844372
10.7	-10.6	-10.5	0.18	2.290	0.363	5.904	1.640	6	4628	ZINC000257566001
10.7	-10.8	-10.6	0.12	1.827	0.274	4.007	2.370	6	4629	ZINC000118937406
10.7	-11.5	-11.2	0.19	2.036	0.475	4.914	2.001	6	4630	ZINC000100780307
10.7	-10.0	-9.6	0.27	2.179	0.642	5.882	2.278	6	4631	ZINC000257472869

10.7	-11.4	-11.2	0.23	1.812	0.317	5.615	2.332	6	4632	ZINC000257467831
10.6	-9.5	-9.3	0.18	2.360	0.299	5.586	3.210	6	4633	ZINC000014930787
10.6	-10.0	-9.7	0.26	1.723	0.447	4.646	3.047	6	4634	ZINC000257454350
10.6	-10.4	-10.0	0.22	2.656	0.421	6.377	2.498	6	4635	ZINC000238750977
10.6	-10.1	-9.8	0.21	2.317	0.513	5.386	2.720	6	4636	ZINC000257506114
10.6	-9.0	-8.8	0.19	2.559	0.913	5.771	3.674	6	4637	ZINC000095620845
10.6	-10.4	-10.0	0.23	1.779	0.100	6.420	1.481	6	4638	ZINC000100778525
10.6	-10.3	-9.9	0.25	1.736	0.165	6.639	1.276	6	4639	ZINC000100779058
10.6	-10.6	-9.3	0.63	3.523	0.459	5.842	1.921	6	4640	ZINC000100780964
10.6	-10.9	-10.6	0.17	2.546	0.210	6.816	1.498	6	4641	ZINC000410428657
10.6	-10.4	-9.8	0.30	1.849	0.099	5.513	1.810	6	4642	ZINC000257456728
10.6	-11.2	-10.4	0.38	1.932	0.126	5.136	1.667	6	4643	ZINC000100781607
10.6	-9.7	-9.1	0.29	2.217	0.281	6.203	1.245	6	4644	ZINC000100822877
10.6	-11.1	-10.6	0.29	2.740	0.264	6.598	0.641	6	4645	ZINC000410428659
10.6	-10.1	-9.8	0.20	2.156	0.224	4.652	1.335	6	4646	ZINC000257410664

10.6	-11.9	-11.1	0.54	2.780	1.129	7.182	3.423	6	4647	ZINC000257504829
10.6	-10.7	-10.2	0.30	1.948	0.105	5.848	2.375	6	4648	ZINC000257455713
10.6	-11.1	-10.5	0.31	2.158	0.466	5.059	1.746	6	4649	ZINC000014887101
10.6	-10.5	-10.3	0.12	2.605	0.120	5.726	0.732	6	4650	ZINC000100826885
10.6	-10.6	-10.1	0.28	2.365	0.145	4.280	0.322	6	4651	ZINC000014811793
10.6	-10.5	-10.0	0.27	2.771	0.957	5.264	2.738	6	4652	ZINC000100824503
10.6	-10.7	-10.2	0.27	2.495	0.354	6.910	1.795	6	4653	ZINC000014617183
10.6	-10.6	-10.1	0.27	1.946	0.535	4.140	2.187	6	4654	ZINC000257476395
10.6	-10.5	-10.0	0.30	1.873	0.115	4.127	2.104	6	4655	ZINC000257378284
10.6	-10.8	-10.0	0.48	1.897	0.700	4.587	2.243	6	4656	ZINC000013373263
10.6	-10.2	-9.7	0.30	2.486	0.885	4.253	2.788	6	4657	ZINC000058603845
10.6	-9.5	-9.2	0.16	2.203	0.557	6.359	2.823	6	4658	ZINC000257479498
10.5	-10.7	-10.4	0.16	1.927	0.193	6.301	2.295	6	4659	ZINC000257447359
10.5	-10.8	-10.5	0.31	2.286	1.124	6.289	1.064	6	4660	ZINC000095619156
10.5	-10.6	-10.1	0.31	2.130	0.238	4.476	1.554	6	4661	ZINC000100824202

10.5	-9.9	-9.8	0.11	1.808	0.197	6.677	1.506	6	4662	ZINC000100777796
10.5	-10.5	-10.1	0.28	1.978	0.482	5.460	2.316	6	4663	ZINC000257491418
10.5	-9.1	-8.8	0.23	2.256	0.173	4.421	2.468	6	4664	ZINC000100824508
10.5	-9.9	-9.7	0.20	1.440	0.218	2.081	0.273	6	4665	ZINC000257520468
10.5	-11.3	-10.7	0.56	1.647	0.841	4.677	2.406	6	4666	ZINC000085862074
10.5	-10.4	-10.0	0.26	2.101	0.671	6.418	2.403	6	4667	ZINC000253500785
10.5	-10.4	-10.1	0.27	2.191	0.679	6.780	2.357	6	4668	ZINC000017545477
10.5	-12.4	-11.7	0.41	2.852	0.621	7.157	1.467	6	4669	ZINC000100779152
10.5	-11.9	-11.5	0.23	2.243	0.303	5.559	2.028	6	4670	ZINC000085561952
10.5	-9.6	-8.6	0.61	2.974	0.572	6.086	1.350	6	4671	ZINC000100829704
10.5	-10.2	-9.7	0.36	2.793	0.580	6.584	2.493	6	4672	ZINC000257392249
10.5	-10.2	-9.8	0.21	2.197	0.289	6.669	2.050	6	4673	ZINC000257415726
10.5	-10.1	-9.6	0.28	3.205	0.207	7.988	1.644	6	4674	ZINC000013374707
10.5	-10.4	-9.8	0.26	1.753	0.603	4.963	2.650	6	4675	ZINC000017545480
10.5	-11.6	-11.3	0.23	1.887	0.501	3.819	2.038	6	4676	ZINC000095619520

10.5	-10.2	-9.9	0.20	1.975	0.606	6.125	2.548	6	4677	ZINC000100825692
10.5	-10.5	-10.4	0.07	2.283	0.111	6.089	0.628	6	4678	ZINC000257404247
10.5	-9.9	-9.7	0.15	2.023	0.183	6.041	1.780	6	4679	ZINC000100777791
10.5	-10.6	-10.4	0.11	2.258	0.263	4.694	1.599	6	4680	ZINC000100825659
10.5	-11.4	-11.1	0.16	2.673	0.318	5.796	2.587	6	4681	ZINC000100828736
10.5	-10.1	-9.9	0.12	2.360	0.488	5.471	1.107	6	4682	ZINC000257621272
10.5	-10.9	-10.6	0.29	2.676	0.750	6.457	2.960	6	4683	ZINC000014765649
10.5	-10.9	-10.2	0.37	1.870	0.415	4.685	2.331	6	4684	ZINC000257382698
10.5	-8.4	-8.3	0.10	2.455	0.413	6.265	3.041	6	4685	ZINC000014887393
10.5	-10.5	-10.0	0.26	2.234	0.464	6.738	2.798	6	4686	ZINC000257491284
10.5	-9.6	-9.2	0.30	2.853	1.021	6.021	3.553	6	4687	ZINC000034000389
10.5	-10.6	-10.2	0.16	1.825	0.542	4.509	1.474	6	4688	ZINC000257400031
10.5	-11.4	-10.1	0.64	2.435	0.478	6.613	1.301	6	4689	ZINC000257442892
10.5	-10.8	-10.0	0.61	2.302	0.520	5.024	2.195	6	4690	ZINC000150368141
10.5	-10.5	-9.8	0.36	2.876	0.697	6.710	2.894	6	4691	ZINC000257573779

10.5	-10.8	-10.4	0.25	2.159	0.206	5.628	1.972	6	4692	ZINC000095620013
10.5	-11.8	-11.3	0.27	2.060	0.555	5.646	2.600	6	4693	ZINC000257788400
10.4	-10.1	-9.9	0.19	2.492	0.325	6.197	1.770	6	4694	ZINC000257477219
10.4	-12.4	-11.7	0.41	2.406	0.914	6.565	2.657	6	4695	ZINC000242547642
10.4	-11.7	-11.2	0.31	2.135	0.478	6.493	2.734	6	4696	ZINC000257788398
10.4	-11.8	-11.3	0.36	2.289	0.384	6.807	2.582	6	4697	ZINC000257788401
10.4	-10.5	-10.2	0.24	2.479	0.126	6.526	0.381	6	4698	ZINC000685933136
10.4	-10.7	-10.2	0.25	2.822	0.904	6.289	2.308	6	4699	ZINC000257512056
10.4	-10.2	-10.0	0.20	1.664	0.595	4.357	2.331	6	4700	ZINC000256110441
10.4	-9.3	-9.0	0.16	2.021	0.343	4.382	2.978	6	4701	ZINC000014813775
10.4	-8.3	-8.2	0.13	2.753	0.607	7.478	2.952	6	4702	ZINC000257588236
10.4	-10.8	-10.2	0.30	1.950	0.667	5.663	2.614	6	4703	ZINC000257435388
10.4	-10.3	-9.6	0.49	3.379	0.746	5.104	0.783	6	4704	ZINC000013322077
10.4	-12.0	-11.1	0.45	1.869	0.242	4.498	2.453	6	4705	ZINC000150364725
10.4	-11.7	-11.1	0.41	2.234	0.333	4.138	1.364	6	4706	ZINC000100779578

10.4	-10.5	-10.2	0.14	2.235	0.449	4.128	1.797	6	4707	ZINC000100779172
10.4	-9.6	-9.2	0.26	1.677	0.497	5.792	4.154	6	4708	ZINC000137197396
10.4	-11.1	-10.5	0.37	1.942	0.679	5.579	2.297	6	4709	ZINC000100778300
10.4	-10.3	-10.0	0.18	2.089	0.222	6.037	1.892	6	4710	ZINC000100829678
10.4	-11.5	-11.1	0.26	2.805	0.536	4.719	1.961	6	4711	ZINC000100257257
10.4	-10.5	-10.2	0.19	2.486	0.291	6.575	0.902	6	4712	ZINC000086045273
10.4	-10.4	-10.0	0.24	2.189	0.416	4.275	2.096	6	4713	ZINC000004098487
10.4	-10.8	-10.5	0.25	2.547	0.290	6.819	1.892	6	4714	ZINC000257392553
10.4	-9.4	-9.2	0.14	2.550	1.078	6.842	3.999	6	4715	ZINC000138108194
10.4	-10.8	-10.5	0.20	2.597	0.452	6.318	1.612	6	4716	ZINC000254685398
10.4	-11.2	-11.0	0.17	1.767	0.326	4.972	1.812	6	4717	ZINC000118937504
10.4	-11.7	-11.2	0.24	2.069	0.514	5.662	2.587	6	4718	ZINC000257788399
10.4	-11.8	-10.6	0.77	2.248	0.311	4.482	1.339	6	4719	ZINC000014693303
10.4	-9.8	-9.6	0.15	1.779	0.389	3.948	3.200	6	4720	ZINC000257544403
10.4	-10.3	-10.0	0.16	3.421	0.570	6.634	2.794	6	4721	ZINC000040165218

10.3	-9.6	-9.1	0.34	1.892	0.389	4.885	2.626	6	4722	ZINC000257511390
10.3	-11.5	-11.1	0.29	1.781	0.291	3.545	1.697	6	4723	ZINC000257445594
10.3	-11.6	-10.8	0.39	1.930	0.382	4.528	2.162	6	4724	ZINC000100781834
10.3	-10.4	-9.8	0.34	2.080	0.694	5.247	2.793	6	4725	ZINC000257604349
10.3	-10.2	-10.0	0.15	3.424	0.570	6.603	2.795	6	4726	ZINC000040165255
10.3	-11.5	-11.0	0.30	1.916	0.382	4.729	2.020	6	4727	ZINC000100826011
10.3	-11.5	-11.2	0.30	2.127	0.736	6.494	3.150	6	4728	ZINC000257541178
10.3	-10.1	-9.7	0.27	2.090	0.113	6.174	1.521	6	4729	ZINC000100827290
10.3	-10.2	-10.0	0.12	1.899	0.562	5.706	2.355	6	4730	ZINC000100826025
10.3	-10.2	-10.0	0.17	3.416	0.580	6.604	2.813	6	4731	ZINC000040165217
10.3	-11.5	-11.0	0.37	2.054	0.539	4.571	2.564	6	4732	ZINC000100826010
10.3	-8.8	-8.7	0.10	2.515	0.737	5.900	2.858	6	4733	ZINC000014559828
10.3	-11.1	-10.2	0.45	2.517	0.278	6.892	1.275	6	4734	ZINC000100824404
10.3	-10.3	-9.6	0.50	3.035	0.840	4.792	0.726	6	4735	ZINC000015058181
10.3	-10.1	-9.3	0.39	2.709	0.315	5.271	2.266	6	4736	ZINC000014728339

10.3	-11.5	-10.9	0.33	1.852	0.459	4.030	2.621	6	4737	ZINC000257445595
10.3	-8.9	-8.8	0.09	2.331	0.130	5.850	1.755	6	4738	ZINC000100822875
10.3	-10.1	-9.7	0.19	2.325	0.738	5.781	3.187	6	4739	ZINC000257457868
10.3	-11.5	-11.0	0.31	2.081	0.455	5.302	2.686	6	4740	ZINC000257445596
10.3	-11.3	-11.0	0.14	1.861	0.307	5.841	1.801	6	4741	ZINC000014720085
10.3	-11.3	-10.6	0.41	2.697	0.438	5.480	1.182	6	4742	ZINC000100823760
10.3	-9.9	-9.8	0.11	3.011	0.664	5.226	2.067	6	4743	ZINC000014679174
10.3	-9.5	-9.0	0.21	2.791	0.535	7.383	3.582	6	4744	ZINC000100774223
10.3	-9.2	-9.0	0.18	2.115	0.520	5.380	3.192	6	4745	ZINC000100828783
10.3	-10.6	-9.9	0.32	2.463	0.709	5.470	2.669	6	4746	ZINC000257410706
10.3	-9.9	-9.5	0.30	2.582	0.203	5.939	1.815	6	4747	ZINC000015251141
10.3	-10.3	-10.0	0.21	2.060	0.549	5.564	3.138	6	4748	ZINC000257475722
10.3	-9.4	-9.3	0.11	2.450	1.001	5.024	2.578	6	4749	ZINC000014779839
10.3	-10.2	-9.5	0.35	2.060	0.231	5.275	2.741	6	4750	ZINC000100038173
10.3	-9.3	-8.7	0.26	2.579	0.386	4.693	2.553	6	4751	ZINC000014757095

10.3	-9.1	-8.8	0.15	3.784	0.348	6.677	2.303	6	4752	ZINC000257542991
10.3	-10.9	-10.5	0.16	2.266	0.844	5.133	2.160	6	4753	ZINC000014953928
10.3	-10.8	-10.6	0.11	2.158	0.437	5.892	1.536	6	4754	ZINC000015105343
10.3	-12.2	-11.3	0.59	1.705	0.162	3.953	1.572	6	4755	ZINC000095619539
10.3	-11.0	-10.5	0.32	2.255	0.230	6.708	0.762	6	4756	ZINC000026490614
10.3	-10.5	-9.3	0.59	3.410	0.281	5.631	2.014	6	4757	ZINC000257546802
10.3	-12.0	-10.7	0.63	3.217	0.433	6.799	2.545	6	4758	ZINC000031415830
10.3	-10.2	-9.9	0.20	2.206	0.250	6.512	1.434	6	4759	ZINC000100826020
10.3	-10.6	-10.0	0.30	1.978	0.117	6.554	0.832	6	4760	ZINC000086041879
10.3	-11.2	-10.8	0.22	2.107	0.706	6.211	0.337	6	4761	ZINC000100822776
10.3	-11.5	-11.0	0.28	1.746	0.245	2.880	1.210	6	4762	ZINC000100826014
10.3	-11.8	-10.1	1.25	2.031	0.407	5.531	1.695	5	4763	ZINC000150360872
10.3	-10.0	-9.6	0.28	1.985	0.116	6.539	1.727	6	4764	ZINC000257400087
10.2	-10.3	-9.9	0.25	2.115	0.446	6.538	2.576	6	4765	ZINC000257529704
10.2	-9.3	-9.0	0.18	1.964	0.962	6.664	2.239	6	4766	ZINC000257404969

10.2	-10.4	-9.7	0.36	3.305	0.775	4.618	1.114	6	4767	ZINC000014806666
10.2	-10.1	-9.8	0.20	1.974	0.252	4.405	2.097	6	4768	ZINC000257444944
10.2	-10.2	-9.9	0.19	2.424	0.168	7.701	0.929	6	4769	ZINC000257421409
10.2	-9.3	-9.0	0.19	1.808	0.884	6.730	2.274	6	4770	ZINC000100772253
10.2	-11.5	-11.0	0.37	1.975	0.417	4.143	1.427	6	4771	ZINC000257445593
10.2	-9.5	-9.1	0.20	2.369	0.500	6.947	2.865	6	4772	ZINC000005854568
10.2	-11.2	-10.8	0.27	2.086	0.280	6.400	1.350	6	4773	ZINC000100827210
10.2	-10.2	-9.8	0.18	2.580	0.151	5.305	1.715	6	4774	ZINC000100773918
10.2	-10.4	-9.7	0.35	3.690	0.545	5.117	0.984	6	4775	ZINC000014806658
10.2	-10.6	-10.4	0.13	2.192	0.543	6.497	2.220	6	4776	ZINC000013335897
10.2	-9.3	-9.0	0.17	1.797	0.884	6.731	2.275	6	4777	ZINC000100772255
10.2	-10.3	-10.0	0.23	2.438	0.124	7.845	0.989	6	4778	ZINC000257529702
10.2	-10.9	-10.2	0.35	1.812	0.329	7.350	1.915	6	4779	ZINC000257406858
10.2	-11.0	-10.1	0.41	2.275	0.591	4.667	2.264	6	4780	ZINC000100772851
10.2	-10.1	-9.6	0.24	2.504	0.679	5.759	2.084	6	4781	ZINC000095617635

10.2	-10.8	-10.4	0.27	1.891	0.424	4.144	1.856	6	4782	ZINC000119394678
10.2	-9.3	-9.0	0.18	1.806	0.878	6.725	2.271	6	4783	ZINC000100772259
10.2	-9.3	-9.0	0.24	1.766	0.855	6.798	2.302	6	4784	ZINC000257404970
10.2	-9.0	-8.8	0.15	1.779	0.456	5.396	3.434	6	4785	ZINC000257533822
10.2	-10.6	-10.1	0.33	1.708	0.564	4.102	1.841	6	4786	ZINC000100824205
10.2	-9.6	-9.5	0.09	2.372	1.134	6.390	3.091	6	4787	ZINC000014779830
10.2	-10.9	-10.5	0.26	2.039	0.392	5.172	1.630	6	4788	ZINC000100299705
10.2	-10.1	-9.8	0.21	2.456	0.223	7.098	1.249	6	4789	ZINC000100826022
10.2	-11.3	-10.7	0.46	2.745	0.543	8.174	2.845	6	4790	ZINC000257504830
10.2	-10.8	-10.1	0.40	1.903	0.500	4.137	2.065	6	4791	ZINC000257447361
10.2	-11.2	-10.9	0.23	2.430	0.359	6.349	1.567	6	4792	ZINC000004083904
10.2	-10.0	-9.6	0.24	3.079	0.993	5.702	3.064	6	4793	ZINC000059789415
10.2	-10.4	-10.1	0.20	2.350	0.190	5.428	1.027	6	4794	ZINC000257454108
10.2	-10.2	-9.7	0.41	3.235	0.450	6.091	1.523	6	4795	ZINC000257511392
10.2	-11.4	-10.9	0.36	2.013	0.494	5.139	2.484	6	4796	ZINC000100826012

10.2	-9.5	-9.2	0.18	2.846	0.299	8.477	2.016	6	4797	ZINC000014436822
10.2	-9.9	-9.6	0.21	2.483	0.559	6.584	2.655	6	4798	ZINC000118937238
10.2	-11.1	-10.8	0.19	2.263	0.432	6.083	2.148	6	4799	ZINC000257536919
10.2	-11.3	-10.6	0.32	3.083	0.298	5.473	0.785	6	4800	ZINC000014953932
10.2	-11.4	-10.9	0.24	1.898	0.469	4.925	1.979	6	4801	ZINC000257379452
10.2	-11.6	-10.2	0.64	2.192	0.256	6.803	1.554	6	4802	ZINC000257502006
10.2	-10.2	-9.9	0.22	2.469	0.173	7.052	1.221	6	4803	ZINC000257421406
10.1	-11.0	-10.4	0.35	2.384	0.239	6.777	2.222	6	4804	ZINC000257541176
10.1	-10.2	-9.4	0.42	2.483	0.570	7.239	3.293	6	4805	ZINC000100823823
10.1	-9.3	-8.7	0.29	2.785	0.771	4.899	2.410	6	4806	ZINC000014559825
10.1	-12.2	-10.8	0.67	3.018	0.908	6.012	1.677	6	4807	ZINC000100257251
10.1	-10.3	-9.9	0.25	2.350	0.352	7.149	1.405	6	4808	ZINC000257529703
10.1	-11.7	-11.6	0.14	1.966	0.294	4.867	2.099	6	4809	ZINC000257391698
10.1	-10.3	-10.0	0.21	2.431	0.191	7.487	1.025	6	4810	ZINC000100825666
10.1	-11.0	-10.2	0.40	2.385	0.369	5.725	1.608	6	4811	ZINC000257530281

10.1	-10.7	-10.3	0.25	2.075	0.267	5.135	2.370	6	4812	ZINC000257405129
10.1	-9.3	-8.9	0.29	1.994	0.633	4.133	2.538	6	4813	ZINC000100826509
10.1	-8.3	-8.2	0.07	1.850	0.872	3.379	0.764	6	4814	ZINC000005663096
10.1	-11.4	-10.8	0.30	2.098	0.479	5.280	1.634	6	4815	ZINC000257379455
10.1	-11.2	-10.9	0.23	2.766	0.461	6.304	1.186	6	4816	ZINC000039166886
10.1	-10.3	-9.2	0.52	2.207	0.394	5.614	1.171	6	4817	ZINC000230085022
10.1	-9.2	-9.0	0.16	1.891	0.927	6.718	2.269	6	4818	ZINC000257404968
10.1	-11.4	-10.9	0.24	1.964	0.426	5.576	1.545	6	4819	ZINC000257379453
10.1	-9.2	-9.0	0.16	1.892	0.938	6.711	2.267	6	4820	ZINC000257404967
10.1	-10.5	-9.9	0.41	2.477	0.209	6.488	1.803	6	4821	ZINC000014441535
10.1	-10.2	-9.9	0.21	2.024	0.970	6.447	2.203	6	4822	ZINC000100822989
10.1	-10.1	-9.9	0.13	2.345	0.338	5.773	2.430	6	4823	ZINC000100773900
10.1	-11.5	-10.4	0.53	2.287	0.378	5.458	1.822	6	4824	ZINC000014617184
10.1	-9.3	-9.2	0.14	4.146	0.905	6.895	1.210	6	4825	ZINC000014502661
10.1	-11.4	-10.9	0.24	1.896	0.466	4.924	1.982	6	4826	ZINC000100827338

10.1	-10.0	-9.7	0.15	1.833	0.455	5.308	2.573	6	4827	ZINC000257447358
10.1	-10.3	-10.1	0.14	2.454	0.563	7.141	2.748	6	4828	ZINC000257491427
10.1	-10.0	-9.7	0.16	2.258	0.633	7.681	2.600	6	4829	ZINC000257649819
10.1	-11.4	-10.9	0.24	1.859	0.460	4.884	1.968	6	4830	ZINC000100827332
10.1	-10.2	-9.5	0.41	2.383	0.380	5.006	1.575	6	4831	ZINC000038750551
10.1	-10.0	-9.8	0.11	2.032	0.389	5.747	2.262	6	4832	ZINC000100780137
10.0	-11.2	-10.9	0.17	2.330	0.547	4.747	1.528	6	4833	ZINC000257547463
10.0	-11.3	-10.5	0.45	1.757	0.114	5.976	1.705	6	4834	ZINC000118937503
10.0	-10.3	-9.8	0.26	2.511	0.369	3.884	0.693	6	4835	ZINC000014762963
10.0	-11.0	-10.2	0.35	2.640	0.336	8.029	2.083	6	4836	ZINC000100824380
10.0	-10.2	-9.8	0.32	2.143	0.370	3.568	1.099	6	4837	ZINC000014762961
10.0	-10.8	-10.3	0.34	2.699	0.562	5.463	1.480	6	4838	ZINC000100829801
10.0	-10.0	-9.8	0.12	2.377	0.241	4.466	1.892	6	4839	ZINC000015251147
10.0	-12.5	-11.4	0.63	2.439	0.505	5.984	1.809	6	4840	ZINC000086041836
10.0	-10.7	-10.4	0.33	2.011	0.549	5.976	3.648	6	4841	ZINC000100827828

10.0	-9.7	-9.3	0.30	1.950	0.319	5.100	2.290	6	4842	ZINC000100256155
10.0	-11.0	-10.6	0.43	2.461	0.926	3.315	0.956	6	4843	ZINC000257469141
10.0	-11.5	-10.6	0.47	1.924	0.212	5.860	0.542	6	4844	ZINC000095914682
10.0	-10.3	-9.8	0.24	2.037	0.279	5.187	1.728	6	4845	ZINC000100829379
10.0	-9.3	-9.1	0.13	2.243	0.694	4.394	2.812	6	4846	ZINC000001531401
10.0	-11.8	-10.9	0.50	2.317	0.666	4.980	2.269	6	4847	ZINC000100774253
10.0	-9.6	-9.0	0.35	1.735	0.324	2.733	0.615	6	4848	ZINC000085588504
10.0	-9.2	-9.0	0.14	2.506	0.238	5.534	1.957	6	4849	ZINC000256079923
10.0	-10.6	-10.3	0.26	2.098	0.618	6.258	3.037	6	4850	ZINC000100784247
10.0	-11.5	-10.0	0.94	2.381	0.745	5.629	0.845	6	4851	ZINC000085860770
10.0	-9.8	-9.7	0.11	3.370	1.147	4.025	1.282	6	4852	ZINC000013382495
10.0	-10.1	-10.0	0.11	2.725	0.926	7.269	1.129	6	4853	ZINC000257523144
10.0	-9.5	-9.1	0.26	2.244	0.676	6.785	3.830	6	4854	ZINC000100889861
10.0	-10.1	-9.8	0.18	2.361	0.794	5.249	2.739	6	4855	ZINC000257377831
10.0	-11.3	-10.8	0.31	2.516	0.127	5.925	1.247	6	4856	ZINC000257396096

10.0	-9.7	-9.3	0.30	3.005	1.008	4.662	2.537	6	4857	ZINC000015261716
10.0	-10.6	-10.3	0.25	2.077	0.271	5.919	1.691	6	4858	ZINC000100782683
10.0	-11.3	-10.8	0.45	2.141	0.198	6.530	1.625	6	4859	ZINC000085915289
10.0	-10.6	-9.6	0.74	2.236	0.238	5.978	0.739	6	4860	ZINC000100824401
10.0	-11.5	-11.0	0.29	2.749	0.326	6.910	0.828	6	4861	ZINC000100827207
10.0	-11.4	-10.7	0.38	2.222	0.423	5.627	2.025	6	4862	ZINC000085561959
10.0	-8.7	-8.4	0.16	2.345	0.413	7.858	2.658	6	4863	ZINC000100774179
10.0	-11.0	-10.7	0.29	2.255	0.410	5.882	1.983	6	4864	ZINC000100825657
10.0	-11.4	-10.8	0.28	1.758	0.285	5.111	1.009	6	4865	ZINC000100827333
10.0	-9.5	-9.2	0.24	1.935	1.001	4.453	3.106	6	4866	ZINC000014757099
10.0	-10.4	-9.7	0.37	2.415	0.739	6.368	1.227	6	4867	ZINC000257604348
9.9	-9.5	-9.3	0.10	2.563	0.492	5.957	1.334	6	4868	ZINC000257486516
9.9	-10.1	-9.9	0.11	2.479	0.771	4.646	2.416	6	4869	ZINC000095620781
9.9	-11.1	-10.8	0.25	1.728	0.184	5.133	1.580	6	4870	ZINC000150361503
9.9	-11.8	-11.3	0.29	1.834	0.370	5.665	0.818	6	4871	ZINC000150366301

9.9	-10.6	-10.1	0.26	2.148	0.664	5.858	2.229	6	4872	ZINC000257803252
9.9	-11.3	-10.8	0.29	2.434	1.027	5.791	1.076	6	4873	ZINC000014636455
9.9	-10.3	-9.9	0.32	2.533	0.286	6.169	1.403	6	4874	ZINC000100784094
9.9	-11.1	-10.9	0.11	1.980	0.317	6.484	2.845	6	4875	ZINC000257557711
9.9	-11.0	-10.8	0.19	2.314	0.237	6.382	1.937	6	4876	ZINC000257573447
9.9	-9.8	-9.2	0.29	2.353	0.421	6.303	2.818	6	4877	ZINC000257435900
9.9	-9.7	-9.6	0.16	2.058	0.545	3.136	1.167	6	4878	ZINC000014762967
9.9	-10.6	-10.1	0.29	1.935	0.811	3.637	2.597	6	4879	ZINC000118937258
9.9	-9.7	-9.4	0.22	2.177	0.907	4.409	2.800	6	4880	ZINC000257559340
9.9	-10.3	-9.8	0.38	2.016	0.347	5.987	1.794	6	4881	ZINC000100779176
9.9	-10.5	-9.9	0.35	1.930	0.204	6.108	2.156	6	4882	ZINC000100825509
9.9	-8.8	-8.6	0.09	2.395	0.199	6.537	2.906	6	4883	ZINC000014811672
9.9	-11.0	-10.7	0.24	2.172	0.411	5.633	2.038	6	4884	ZINC000257536916
9.9	-9.4	-9.2	0.14	3.065	0.422	3.928	0.622	6	4885	ZINC000014651017
9.9	-8.3	-8.1	0.16	0.914	0.446	5.351	3.121	6	4886	ZINC000105395106

9.9	-11.1	-10.8	0.18	1.810	0.185	5.774	1.565	6	4887	ZINC000100779558
9.9	-10.5	-10.0	0.27	1.922	0.863	4.117	2.230	6	4888	ZINC000086006927
9.9	-11.4	-10.9	0.25	1.965	0.429	5.584	1.543	6	4889	ZINC000100827337
9.9	-11.2	-10.5	0.34	2.046	0.327	5.487	1.827	6	4890	ZINC000015105769
9.9	-12.0	-10.8	0.65	3.218	0.460	6.142	2.269	6	4891	ZINC000040165202
9.9	-9.1	-8.9	0.15	1.948	0.489	4.526	2.810	6	4892	ZINC000100773944
9.9	-9.9	-9.2	0.37	2.486	0.582	4.801	1.850	6	4893	ZINC000118936828
9.9	-8.3	-8.1	0.13	1.656	0.748	5.448	3.041	6	4894	ZINC000014648304
9.9	-10.6	-10.2	0.20	1.919	0.438	4.851	2.504	6	4895	ZINC000257513123
9.8	-9.8	-9.6	0.16	2.352	0.564	5.119	2.197	6	4896	ZINC000261497407
9.8	-10.3	-9.6	0.49	3.087	0.797	4.504	1.152	6	4897	ZINC000013322079
9.8	-10.5	-10.1	0.26	2.099	0.114	7.321	0.795	6	4898	ZINC000100825502
9.8	-10.6	-10.3	0.18	2.974	0.894	8.158	3.211	6	4899	ZINC000118937237
9.8	-10.5	-10.0	0.30	2.056	0.175	7.069	0.880	6	4900	ZINC000257549039
9.8	-10.1	-10.0	0.10	2.215	0.500	4.374	2.064	6	4901	ZINC000118936932

9.8	-9.6	-9.4	0.17	2.133	0.731	5.443	3.281	6	4902	ZINC000015251144
9.8	-12.4	-11.3	0.51	2.278	0.289	6.600	0.724	6	4903	ZINC000085862056
9.8	-11.4	-10.8	0.30	1.757	0.283	5.107	1.023	6	4904	ZINC000257379454
9.8	-9.9	-9.2	0.32	2.276	0.249	6.632	2.689	6	4905	ZINC000257431093
9.8	-10.8	-10.5	0.16	1.848	0.076	5.003	1.561	6	4906	ZINC000104364018
9.8	-10.1	-9.7	0.26	1.691	0.317	5.441	2.458	6	4907	ZINC000100779318
9.8	-10.6	-10.4	0.17	2.430	0.273	3.989	0.685	6	4908	ZINC000118937040
9.8	-10.7	-9.8	0.46	2.711	0.517	5.119	2.028	6	4909	ZINC000257649820
9.8	-9.9	-9.7	0.11	2.394	0.181	7.743	0.873	6	4910	ZINC000257493562
9.8	-11.3	-10.6	0.58	3.106	0.131	6.470	1.881	6	4911	ZINC000014559285
9.8	-9.6	-9.3	0.25	3.751	0.658	6.007	2.710	6	4912	ZINC000013374851
9.8	-9.1	-8.8	0.15	2.571	0.490	5.965	2.337	6	4913	ZINC000031391961
9.8	-10.0	-9.6	0.17	2.033	0.174	6.739	1.440	6	4914	ZINC000257544500
9.8	-7.9	-7.4	0.23	2.382	0.468	5.667	3.341	6	4915	ZINC000030731384
9.8	-11.1	-10.5	0.34	2.137	0.415	3.728	1.422	6	4916	ZINC000257488806

9.8	-11.8	-11.3	0.45	1.673	0.344	2.995	1.369	6	4917	ZINC000100822999
9.8	-10.0	-9.8	0.20	2.207	0.366	6.171	1.896	6	4918	ZINC000257455991
9.8	-10.9	-10.6	0.27	1.944	0.178	5.554	0.723	6	4919	ZINC000257399011
9.8	-10.5	-10.0	0.28	1.941	0.285	6.190	2.073	6	4920	ZINC000100825504
9.8	-10.5	-10.0	0.26	2.083	0.141	6.829	0.994	6	4921	ZINC000100825506
9.8	-10.9	-10.5	0.26	2.064	0.326	6.138	1.096	6	4922	ZINC000257399010
9.8	-9.9	-9.6	0.17	2.446	0.308	6.310	2.155	6	4923	ZINC000100773921
9.8	-10.0	-9.8	0.23	2.086	0.282	5.282	1.797	6	4924	ZINC000059736073
9.8	-9.9	-9.7	0.09	2.106	0.667	6.287	2.369	6	4925	ZINC000257493563
9.8	-11.8	-10.8	0.49	3.456	0.901	6.453	2.063	6	4926	ZINC000100774260
9.8	-10.5	-9.9	0.31	2.006	0.169	6.762	1.001	6	4927	ZINC000257549038
9.8	-12.0	-11.4	0.33	2.028	0.323	5.488	1.079	6	4928	ZINC000257507607
9.8	-9.2	-9.0	0.08	1.876	0.484	6.700	3.869	6	4929	ZINC000257442485
9.8	-10.9	-10.6	0.24	2.030	0.329	6.101	1.108	6	4930	ZINC000100823145
9.8	-9.9	-9.1	0.45	2.308	0.495	3.738	1.811	6	4931	ZINC000014502664

9.8	-11.3	-11.1	0.19	1.630	0.245	5.480	2.234	6	4932	ZINC000100779584
9.8	-10.5	-10.0	0.30	1.968	0.210	7.529	0.806	6	4933	ZINC000257440863
9.8	-10.9	-10.6	0.28	1.962	0.179	5.552	0.708	6	4934	ZINC000257399013
9.8	-11.6	-9.9	0.89	2.148	0.243	7.046	0.133	5	4935	ZINC000100822468
9.8	-10.5	-10.0	0.27	2.092	0.176	7.321	0.798	6	4936	ZINC000257549040
9.8	-10.3	-10.0	0.19	2.354	0.471	4.764	1.708	6	4937	ZINC000005085287
9.8	-9.0	-8.6	0.21	2.172	0.659	4.870	2.642	6	4938	ZINC000095619630
9.8	-9.4	-9.2	0.17	2.399	0.452	7.821	2.702	6	4939	ZINC000100825168
9.8	-10.0	-9.8	0.23	2.162	0.313	5.792	1.665	6	4940	ZINC000100825376
9.8	-10.2	-9.7	0.35	2.760	1.048	5.688	3.598	6	4941	ZINC000261495061
9.8	-10.2	-10.1	0.09	2.643	0.274	8.356	2.483	6	4942	ZINC000100829045
9.7	-11.0	-10.4	0.27	2.363	0.473	6.570	1.658	6	4943	ZINC000119395091
9.7	-9.8	-9.4	0.31	2.069	0.331	4.577	2.789	6	4944	ZINC000085879594
9.7	-11.3	-10.6	0.32	2.276	0.415	4.862	1.752	6	4945	ZINC000100828743
9.7	-11.1	-10.4	0.49	1.908	0.483	5.659	2.796	6	4946	ZINC000257573435

9.7	-9.8	-9.7	0.13	2.318	0.100	7.864	0.989	6	4947	ZINC000257493564
9.7	-10.1	-10.0	0.11	2.766	0.946	7.999	1.065	6	4948	ZINC000100822988
9.7	-9.2	-8.9	0.19	2.160	0.440	5.870	2.071	6	4949	ZINC000013382499
9.7	-9.1	-8.9	0.09	2.709	0.516	6.408	2.315	6	4950	ZINC000014645282
9.7	-10.5	-9.3	0.60	3.404	0.337	5.633	2.002	6	4951	ZINC000257546801
9.7	-10.7	-10.3	0.26	2.291	0.384	5.691	1.959	6	4952	ZINC000257536917
9.7	-10.9	-10.5	0.27	1.806	0.633	5.327	2.059	6	4953	ZINC000257399012
9.7	-10.3	-9.5	0.38	2.826	0.558	4.305	1.634	6	4954	ZINC000014594639
9.7	-9.8	-9.5	0.21	2.735	0.426	6.350	2.072	6	4955	ZINC000257387411
9.7	-10.6	-10.3	0.19	1.864	0.397	4.674	2.082	6	4956	ZINC000257808146
9.7	-10.4	-9.7	0.38	3.740	0.550	5.079	0.905	6	4957	ZINC000257481912
9.7	-10.2	-9.9	0.20	2.501	0.238	4.750	2.030	6	4958	ZINC000257468173
9.7	-9.2	-8.9	0.26	1.743	0.357	2.913	0.804	6	4959	ZINC000104871274
9.7	-10.7	-10.0	0.41	2.334	0.458	6.268	1.903	6	4960	ZINC000257458915
9.7	-10.4	-9.7	0.34	3.749	0.473	5.211	0.865	6	4961	ZINC000257481910

9.7	-11.2	-10.6	0.30	2.208	0.549	6.471	2.418	6	4962	ZINC000238737929
9.7	-11.4	-10.7	0.44	2.113	0.202	6.437	2.007	6	4963	ZINC000100822828
9.7	-10.4	-9.6	0.39	2.866	0.904	4.072	1.053	6	4964	ZINC000015058182
9.7	-10.6	-10.4	0.19	1.863	0.233	4.872	2.304	6	4965	ZINC000003881983
9.7	-9.1	-8.8	0.25	2.547	0.395	7.734	2.185	6	4966	ZINC000085475843
9.7	-9.4	-8.9	0.26	2.434	0.290	5.494	2.144	6	4967	ZINC000257423026
9.7	-9.6	-9.2	0.24	2.142	0.188	3.732	0.788	6	4968	ZINC000014488283
9.7	-9.8	-9.7	0.12	2.492	0.087	7.202	1.033	6	4969	ZINC000100825658
9.7	-9.1	-8.8	0.21	2.404	0.607	5.395	2.844	6	4970	ZINC000014781054
9.7	-10.6	-9.8	0.35	2.326	0.596	5.135	2.229	6	4971	ZINC000030729909
9.7	-11.5	-11.1	0.27	2.873	0.787	6.460	1.005	6	4972	ZINC000086043949
9.7	-10.8	-10.5	0.25	1.894	0.211	5.944	1.971	6	4973	ZINC000257385900
9.7	-10.0	-9.7	0.24	2.608	0.817	6.260	2.915	6	4974	ZINC000257523142
9.7	-10.9	-10.4	0.27	2.098	0.812	4.973	2.392	6	4975	ZINC000100828157
9.7	-9.1	-8.7	0.26	2.342	0.624	5.947	1.153	6	4976	ZINC000014858258

9.7	-10.9	-10.6	0.27	1.933	0.182	5.533	0.722	6	4977	ZINC000100823138
9.7	-10.9	-10.4	0.27	1.882	1.006	5.055	2.957	6	4978	ZINC000257406162
9.7	-9.0	-8.7	0.17	2.296	0.905	7.050	3.515	6	4979	ZINC000014644771
9.6	-10.4	-9.7	0.43	2.405	0.436	5.947	1.782	6	4980	ZINC000257389782
9.6	-10.4	-10.0	0.22	2.015	0.151	6.822	0.990	6	4981	ZINC000257549037
9.6	-11.1	-10.7	0.27	2.014	0.615	4.435	2.277	6	4982	ZINC000257547466
9.6	-10.5	-10.3	0.17	2.678	0.193	6.068	1.013	6	4983	ZINC000257454106
9.6	-8.5	-8.4	0.09	2.424	0.675	6.831	3.282	6	4984	ZINC000014757347
9.6	-10.8	-10.4	0.26	2.828	0.779	7.310	3.417	6	4985	ZINC000257504828
9.6	-10.9	-10.5	0.22	2.082	0.670	5.378	1.810	6	4986	ZINC000257404083
9.6	-10.3	-9.9	0.31	2.651	0.562	6.438	2.538	6	4987	ZINC000255222363
9.6	-11.5	-11.1	0.24	1.756	0.244	3.873	1.574	6	4988	ZINC000257396098
9.6	-9.2	-9.1	0.11	2.800	0.297	7.274	2.997	6	4989	ZINC000014760542
9.6	-11.2	-10.5	0.43	2.536	0.494	6.287	0.762	6	4990	ZINC000059730509
9.6	-9.2	-9.0	0.11	1.717	0.073	2.974	0.830	6	4991	ZINC000100827649

9.6	-8.5	-8.3	0.11	1.995	0.722	3.217	0.951	6	4992	ZINC000056870823
9.6	-11.4	-10.9	0.36	1.825	0.234	6.472	1.433	6	4993	ZINC000257435728
9.6	-11.1	-10.0	0.54	3.292	0.320	6.837	1.430	6	4994	ZINC000257379673
9.6	-10.6	-10.1	0.30	2.343	0.431	4.996	2.106	6	4995	ZINC000100784243
9.6	-9.6	-9.3	0.16	1.796	0.314	5.362	2.493	6	4996	ZINC000253590063
9.6	-10.7	-10.1	0.33	2.587	0.601	6.324	1.933	6	4997	ZINC000257470149
9.6	-9.5	-9.2	0.14	2.658	0.354	6.241	1.212	6	4998	ZINC000100772675
9.6	-11.2	-10.4	0.44	1.808	0.628	5.991	0.873	6	4999	ZINC000100826918
9.6	-10.3	-9.8	0.23	2.470	0.208	6.118	0.857	6	5000	ZINC000150352515
9.6	-10.7	-10.4	0.25	2.463	0.374	6.134	0.847	6	5001	ZINC000150369713
9.6	-10.7	-10.4	0.26	1.713	0.574	5.117	1.870	6	5002	ZINC000100823142
9.5	-10.1	-9.7	0.30	1.959	0.187	5.289	3.231	6	5003	ZINC000257517539
9.5	-9.5	-9.4	0.14	2.769	0.306	9.193	2.309	6	5004	ZINC000257618751
9.5	-9.9	-9.5	0.29	3.747	0.757	6.710	2.074	6	5005	ZINC000257407076
9.5	-10.4	-10.0	0.26	2.074	0.460	6.204	1.004	6	5006	ZINC000059762881

9.5	-9.4	-9.1	0.20	1.965	0.397	7.266	3.897	6	5007	ZINC000257544401
9.5	-10.0	-9.6	0.26	3.372	0.668	6.056	1.609	6	5008	ZINC000257493866
9.5	-9.4	-9.0	0.28	2.564	0.635	5.103	1.542	6	5009	ZINC000003978523
9.5	-9.7	-9.4	0.17	1.691	0.400	2.801	0.890	6	5010	ZINC000014760474
9.5	-9.4	-9.1	0.17	2.251	0.389	6.179	2.617	6	5011	ZINC000005158782
9.5	-11.3	-10.9	0.27	2.369	0.624	6.927	1.380	6	5012	ZINC000257460141
9.5	-10.1	-9.6	0.35	3.160	0.448	7.252	2.826	6	5013	ZINC000257710433
9.5	-10.9	-10.6	0.15	1.985	0.778	4.104	1.427	6	5014	ZINC000257404082
9.5	-9.7	-9.3	0.24	2.300	0.169	5.694	1.632	6	5015	ZINC000257457516
9.5	-9.9	-9.5	0.25	3.746	0.736	6.873	1.930	6	5016	ZINC000257407075
9.5	-10.4	-10.2	0.18	1.946	0.398	4.070	2.130	6	5017	ZINC000257386780
9.5	-9.5	-9.3	0.16	1.819	0.276	4.866	2.084	6	5018	ZINC000257405386
9.5	-10.6	-9.8	0.40	3.379	0.825	4.970	1.068	6	5019	ZINC000095619705
9.5	-10.5	-10.3	0.21	2.280	0.171	4.386	1.501	6	5020	ZINC000100823661
9.5	-10.3	-10.1	0.17	2.638	0.825	5.852	2.259	6	5021	ZINC000100778650

9.5	-9.9	-9.5	0.24	2.322	0.157	5.702	1.632	6	5022	ZINC000100772235
9.5	-11.2	-10.8	0.38	1.845	0.331	5.257	1.606	6	5023	ZINC000100779430
9.5	-11.9	-11.5	0.22	2.246	0.489	6.987	1.396	6	5024	ZINC000150361498
9.5	-9.9	-9.5	0.23	3.127	0.301	6.921	1.345	6	5025	ZINC000118937192
9.5	-11.0	-10.4	0.31	1.845	0.239	4.705	2.162	6	5026	ZINC000100773680
9.5	-10.8	-10.6	0.10	3.167	0.712	5.305	2.170	6	5027	ZINC000040164488
9.5	-10.0	-9.7	0.14	1.862	0.607	5.408	3.103	6	5028	ZINC000005085286
9.5	-12.3	-11.5	0.64	1.879	0.667	5.967	0.601	6	5029	ZINC000100826921
9.5	-11.0	-10.3	0.42	2.182	0.572	4.302	1.810	6	5030	ZINC000014951196
9.5	-11.0	-10.7	0.26	2.168	0.562	5.590	2.652	6	5031	ZINC000118937115
9.5	-10.9	-10.4	0.26	1.983	0.618	5.665	1.864	6	5032	ZINC000100828162
9.5	-9.7	-9.4	0.23	2.635	0.996	5.294	2.900	6	5033	ZINC000095619782
9.4	-10.8	-10.0	0.40	2.583	0.424	7.851	3.260	6	5034	ZINC000257431095
9.4	-9.9	-9.5	0.25	3.762	0.759	6.880	1.935	6	5035	ZINC000015203078
9.4	-9.9	-9.5	0.25	3.769	0.758	6.880	1.929	6	5036	ZINC000015203083

9.4	-9.9	-9.5	0.25	3.763	0.750	6.882	1.924	6	5037	ZINC000015203081
9.4	-10.9	-10.6	0.16	1.964	0.764	4.092	1.421	6	5038	ZINC000100828158
9.4	-9.9	-9.5	0.26	3.774	0.736	6.877	1.930	6	5039	ZINC000257407073
9.4	-9.8	-9.4	0.24	2.315	0.156	5.693	1.641	6	5040	ZINC000100772228
9.4	-10.8	-10.4	0.21	1.922	0.540	4.657	2.040	6	5041	ZINC000257580984
9.4	-9.9	-9.3	0.35	1.981	0.654	4.177	3.246	6	5042	ZINC000257573885
9.4	-9.8	-9.2	0.31	2.276	0.673	6.023	3.549	6	5043	ZINC000104871269
9.4	-10.0	-9.8	0.18	2.162	0.452	5.499	1.915	6	5044	ZINC000257433859
9.4	-9.5	-8.7	0.41	2.543	0.788	7.428	3.683	6	5045	ZINC000257493294
9.4	-11.4	-10.9	0.28	2.526	0.271	5.910	1.602	6	5046	ZINC000257405315
9.4	-10.9	-10.6	0.27	2.229	0.427	6.270	1.892	6	5047	ZINC000085883295
9.4	-9.1	-9.0	0.05	1.975	0.120	6.408	2.061	6	5048	ZINC000257511389
9.4	-8.5	-8.3	0.16	2.213	0.737	3.584	1.280	6	5049	ZINC000085552379
9.4	-9.2	-8.8	0.29	3.793	1.178	6.962	1.597	6	5050	ZINC000014610083
9.4	-11.3	-10.7	0.29	2.110	0.624	4.784	1.835	6	5051	ZINC000100784103

9.4	-9.9	-9.5	0.25	3.762	0.749	6.882	1.928	6	5052	ZINC000015203075
9.4	-9.0	-8.8	0.15	1.919	0.357	2.935	0.681	6	5053	ZINC000238770680
9.4	-9.8	-9.5	0.17	2.183	0.232	6.443	0.652	6	5054	ZINC000086033209
9.4	-11.1	-10.9	0.09	2.230	0.251	7.117	1.216	6	5055	ZINC000257426052
9.4	-9.7	-9.4	0.15	2.026	0.220	6.224	0.933	6	5056	ZINC000100825524
9.4	-12.2	-11.1	0.61	1.765	0.241	3.152	0.742	6	5057	ZINC000100778075
9.4	-9.2	-9.0	0.16	2.593	0.149	4.682	0.361	6	5058	ZINC000100247852
9.4	-9.1	-8.7	0.22	1.936	0.449	7.282	4.027	6	5059	ZINC000070455473
9.4	-9.8	-9.4	0.24	2.318	0.159	5.698	1.637	6	5060	ZINC000100772230
9.4	-11.2	-10.8	0.23	2.713	0.347	7.488	2.086	6	5061	ZINC000100822461
9.4	-10.5	-10.0	0.32	1.952	0.226	7.532	0.793	6	5062	ZINC000257440860
9.4	-10.6	-10.2	0.29	2.372	0.254	7.484	2.616	6	5063	ZINC000100829219
9.4	-9.9	-9.5	0.25	3.761	0.750	6.879	1.937	6	5064	ZINC000257407074
9.4	-8.7	-8.4	0.16	2.425	0.542	5.180	3.013	6	5065	ZINC000015257352
9.3	-11.1	-10.7	0.34	3.099	1.094	4.163	1.009	6	5066	ZINC000257469143

9.3	-8.5	-8.3	0.14	2.003	0.696	3.252	0.882	6	5067	ZINC000085552381
9.3	-10.8	-10.6	0.22	2.378	0.281	4.865	0.911	6	5068	ZINC000253530770
9.3	-10.4	-10.2	0.15	2.466	0.306	6.241	1.106	6	5069	ZINC000100826881
9.3	-9.3	-9.0	0.25	3.031	0.115	7.477	1.437	6	5070	ZINC000100825773
9.3	-11.4	-10.6	0.43	3.506	0.413	7.567	1.593	6	5071	ZINC000257670142
9.3	-9.0	-8.9	0.11	1.512	0.663	3.398	0.923	6	5072	ZINC000100247861
9.3	-9.7	-9.3	0.24	2.301	0.166	5.694	1.631	6	5073	ZINC000257457518
9.3	-9.9	-9.5	0.24	2.095	0.115	6.913	0.707	6	5074	ZINC000100825365
9.3	-10.0	-9.7	0.23	2.093	0.305	6.471	2.575	6	5075	ZINC000257458885
9.3	-10.8	-10.7	0.15	2.493	0.335	5.430	0.794	6	5076	ZINC000253530769
9.3	-9.4	-8.6	0.43	3.992	0.481	7.437	1.125	6	5077	ZINC000059762085
9.3	-10.1	-9.6	0.28	2.380	0.706	6.057	2.924	6	5078	ZINC000100823421
9.3	-9.8	-9.7	0.10	2.003	0.564	6.457	2.529	6	5079	ZINC000257493565
9.3	-10.6	-10.2	0.16	2.034	0.165	5.553	1.060	6	5080	ZINC000100823107
9.3	-10.8	-10.7	0.11	2.353	0.363	5.143	1.705	6	5081	ZINC000100829067

9.3	-10.6	-10.5	0.08	2.339	0.299	5.677	0.911	6	5082	ZINC000100823102
9.3	-11.4	-10.6	0.43	3.530	0.434	7.578	1.620	6	5083	ZINC000257670145
9.3	-8.6	-8.3	0.13	2.776	0.592	5.904	2.387	6	5084	ZINC000032296553
9.3	-8.9	-8.5	0.28	2.324	0.242	4.514	0.790	6	5085	ZINC000014757408
9.3	-11.4	-10.6	0.43	3.519	0.428	7.563	1.598	6	5086	ZINC000100776825
9.3	-10.6	-10.2	0.17	2.044	0.669	4.666	1.637	6	5087	ZINC000100823106
9.3	-11.4	-10.6	0.43	3.524	0.437	7.575	1.620	6	5088	ZINC000100776823
9.3	-11.4	-10.3	0.60	2.193	0.447	6.003	0.335	6	5089	ZINC000100826923
9.3	-9.3	-9.2	0.11	2.074	0.429	4.412	2.397	6	5090	ZINC000257693601
9.3	-11.3	-10.9	0.29	2.049	0.313	5.341	2.404	6	5091	ZINC000257402589
9.3	-10.4	-10.1	0.29	2.676	0.801	5.751	2.874	6	5092	ZINC000118937196
9.3	-11.5	-11.1	0.21	2.470	0.353	6.317	1.254	6	5093	ZINC000095619535
9.3	-9.7	-9.3	0.24	2.301	0.169	5.693	1.632	6	5094	ZINC000257457515
9.3	-10.8	-10.7	0.10	2.303	0.402	5.163	1.714	6	5095	ZINC000100829065
9.3	-10.6	-10.2	0.18	1.863	0.610	4.417	1.427	6	5096	ZINC000257400029

9.3	-12.0	-10.9	0.52	2.617	0.414	6.644	0.692	6	5097	ZINC000026189523
9.3	-10.6	-9.4	0.60	3.499	0.420	5.820	1.925	6	5098	ZINC000257546803
9.3	-11.4	-10.6	0.43	3.516	0.420	7.566	1.612	6	5099	ZINC000257670143
9.3	-10.9	-9.8	0.54	2.385	0.382	5.600	1.778	6	5100	ZINC000257517537
9.3	-10.1	-9.2	0.41	2.397	0.656	6.159	2.765	6	5101	ZINC000257458218
9.3	-11.1	-10.7	0.43	2.628	1.063	3.741	1.161	6	5102	ZINC000100772740
9.3	-9.4	-9.0	0.24	3.277	0.287	6.307	2.102	6	5103	ZINC000238744083
9.3	-8.6	-8.3	0.20	1.816	0.756	4.406	2.091	6	5104	ZINC000008551166
9.3	-10.8	-10.7	0.11	2.316	0.395	5.166	1.715	6	5105	ZINC000100829066
9.3	-11.2	-10.7	0.29	2.794	0.530	5.755	2.844	6	5106	ZINC000118937129
9.3	-9.7	-9.4	0.21	2.301	0.160	5.661	1.663	6	5107	ZINC000100772233
9.3	-11.4	-10.6	0.43	3.519	0.424	7.575	1.597	6	5108	ZINC000257670144
9.3	-10.8	-10.7	0.10	2.316	0.308	4.776	1.106	6	5109	ZINC000253530772
9.3	-10.8	-10.7	0.10	2.286	0.327	5.170	0.660	6	5110	ZINC000100829063
9.3	-11.5	-10.8	0.33	2.288	0.468	6.459	2.260	6	5111	ZINC000150361496

9.3	-10.0	-9.5	0.30	2.446	0.230	6.611	0.518	6	5112	ZINC000013373245
9.3	-10.5	-10.1	0.33	1.967	0.419	5.433	2.988	6	5113	ZINC000257461513
9.3	-9.9	-9.8	0.17	2.234	0.336	5.585	1.761	6	5114	ZINC000257433860
9.3	-10.2	-9.7	0.28	2.055	0.261	6.422	3.512	6	5115	ZINC000014488611
9.3	-11.1	-10.6	0.49	2.569	0.931	3.743	1.094	6	5116	ZINC000257469144
9.3	-9.5	-9.1	0.20	3.234	0.368	7.297	2.403	6	5117	ZINC000100827218
9.2	-10.3	-9.8	0.30	3.598	0.697	5.846	2.080	6	5118	ZINC000033839051
9.2	-7.8	-7.6	0.12	3.087	0.256	9.488	0.130	6	5119	ZINC000100051308
9.2	-10.3	-9.6	0.51	2.969	0.950	4.559	1.009	6	5120	ZINC000257513025
9.2	-9.7	-8.7	0.53	2.082	0.449	6.432	3.553	6	5121	ZINC000100771811
9.2	-9.8	-9.6	0.12	1.852	0.372	2.941	0.740	6	5122	ZINC000100779925
9.2	-12.1	-10.8	0.71	2.647	0.518	6.815	1.176	6	5123	ZINC000086041839
9.2	-10.4	-9.9	0.36	2.738	0.345	6.809	0.781	6	5124	ZINC000100825226
9.2	-10.1	-9.8	0.22	2.458	0.276	4.932	2.030	6	5125	ZINC000257649817
9.2	-9.6	-9.3	0.22	2.298	0.168	5.691	1.631	6	5126	ZINC000257457517

9.2	-9.7	-9.2	0.35	1.976	0.298	5.876	1.115	6	5127	ZINC000100825522
9.2	-12.3	-11.8	0.40	2.559	0.986	5.757	1.162	6	5128	ZINC000095619383
9.2	-10.2	-9.7	0.29	2.539	0.598	6.304	2.867	6	5129	ZINC000257562846
9.2	-10.6	-10.2	0.16	2.051	0.675	4.521	1.554	6	5130	ZINC000257400030
9.2	-11.0	-10.5	0.55	2.294	0.718	3.511	0.859	6	5131	ZINC000100772736
9.2	-11.0	-10.6	0.43	2.565	0.933	3.642	1.098	6	5132	ZINC000100772734
9.2	-11.6	-10.5	0.53	2.196	0.805	4.492	1.988	6	5133	ZINC000100784106
9.2	-11.3	-11.0	0.19	2.141	0.576	4.257	2.106	6	5134	ZINC000118937163
9.2	-10.7	-10.0	0.42	2.620	0.619	6.778	0.403	6	5135	ZINC000017653878
9.2	-9.5	-9.1	0.20	3.026	0.551	6.959	2.717	6	5136	ZINC000257384359
9.2	-9.2	-9.0	0.18	2.205	0.391	2.946	0.688	6	5137	ZINC000014811850
9.2	-9.9	-9.5	0.31	3.051	0.254	8.508	2.758	6	5138	ZINC000012496007
9.2	-9.9	-9.7	0.23	2.097	0.428	4.785	2.200	6	5139	ZINC000100825367
9.2	-11.0	-10.7	0.22	1.571	0.897	4.682	2.742	6	5140	ZINC000100824474
9.2	-9.5	-9.1	0.19	3.015	0.578	7.037	2.770	6	5141	ZINC000100827216

9.2	-10.6	-10.3	0.15	2.124	0.255	5.267	0.946	6	5142	ZINC000257400028
9.2	-11.1	-10.5	0.36	2.044	0.307	6.483	2.322	6	5143	ZINC000257470604
9.2	-11.1	-10.7	0.26	2.753	0.597	5.580	3.028	6	5144	ZINC000118937127
9.2	-9.5	-9.1	0.19	3.258	0.378	7.402	2.467	6	5145	ZINC000100827215
9.2	-8.5	-8.3	0.16	1.935	0.579	4.100	2.261	6	5146	ZINC000085552386
9.2	-9.3	-8.8	0.26	1.588	0.785	5.575	1.488	6	5147	ZINC000001580528
9.2	-8.6	-8.3	0.17	1.431	0.692	4.013	2.214	6	5148	ZINC000040164548
9.2	-10.2	-9.9	0.20	2.463	0.207	5.290	0.741	6	5149	ZINC000018007499
9.2	-8.9	-8.2	0.48	2.303	0.544	4.850	1.893	6	5150	ZINC000195759750
9.2	-11.4	-11.0	0.24	2.286	0.597	4.563	1.974	6	5151	ZINC000118937161
9.2	-11.1	-10.7	0.42	2.764	1.014	3.903	0.929	6	5152	ZINC000257469142
9.2	-10.6	-9.4	0.57	3.565	0.399	5.851	1.913	6	5153	ZINC000100780972
9.2	-11.1	-10.4	0.38	1.470	0.271	4.168	2.646	6	5154	ZINC000100299716
9.2	-9.5	-9.0	0.21	2.557	0.446	5.754	2.518	6	5155	ZINC000257384360
9.2	-10.4	-9.6	0.45	2.226	0.522	7.279	3.106	6	5156	ZINC000014813793

9.2	-9.2	-9.1	0.14	2.523	0.065	5.452	1.770	6	5157	ZINC000256079724
9.2	-9.5	-9.1	0.20	3.240	0.450	8.239	1.940	6	5158	ZINC000100827217
9.2	-8.6	-8.4	0.12	1.950	0.345	5.602	3.849	6	5159	ZINC000008844373
9.2	-9.7	-9.5	0.17	2.118	0.249	6.463	0.643	6	5160	ZINC000100825528
9.2	-10.5	-10.2	0.17	2.619	0.173	6.625	0.603	6	5161	ZINC000257454105
9.1	-9.5	-9.3	0.14	2.827	0.944	5.779	3.514	6	5162	ZINC000004098610
9.1	-10.8	-10.3	0.37	2.757	0.287	6.631	0.230	6	5163	ZINC000257547465
9.1	-11.1	-10.7	0.25	2.321	0.816	4.307	2.545	6	5164	ZINC000118937128
9.1	-10.3	-10.2	0.09	2.586	0.464	5.635	1.279	6	5165	ZINC000100784250
9.1	-8.6	-8.4	0.22	2.552	0.270	4.465	0.339	6	5166	ZINC000253617699
9.1	-9.2	-8.6	0.31	2.283	0.294	5.803	3.293	6	5167	ZINC000100771814
9.1	-10.4	-9.2	0.78	1.962	0.356	5.399	2.704	6	5168	ZINC000100824608
9.1	-10.8	-10.1	0.39	2.401	0.760	5.407	2.621	6	5169	ZINC000257618448
9.1	-9.5	-9.2	0.23	2.154	0.080	6.449	0.950	6	5170	ZINC000070454710
9.1	-9.6	-9.2	0.24	2.106	0.225	7.936	2.952	6	5171	ZINC000100829220

9.1	-10.7	-10.7	0.05	3.232	0.845	5.878	2.680	6	5172	ZINC000100778908
9.1	-10.2	-9.5	0.41	2.186	0.439	6.701	2.560	6	5173	ZINC000230066600
9.1	-9.8	-9.4	0.28	1.959	0.269	5.923	1.131	6	5174	ZINC000257550496
9.1	-10.7	-10.3	0.27	1.983	0.194	5.452	2.856	6	5175	ZINC000257378282
9.1	-11.1	-10.1	0.51	2.908	0.889	5.964	2.383	6	5176	ZINC000100827889
9.1	-10.9	-10.3	0.27	2.108	0.097	5.993	2.570	6	5177	ZINC000257549773
9.1	-8.6	-8.4	0.11	1.595	0.712	3.202	0.894	6	5178	ZINC000006920404
9.1	-9.6	-9.4	0.17	2.425	0.187	5.406	2.000	6	5179	ZINC000257419521
9.1	-10.5	-10.3	0.12	2.176	0.491	5.289	2.571	6	5180	ZINC000118936930
9.1	-11.3	-10.9	0.29	2.409	0.295	5.168	2.372	6	5181	ZINC000118937160
9.1	-11.7	-11.0	0.42	2.855	0.603	5.465	0.934	6	5182	ZINC000070455089
9.1	-10.2	-9.7	0.35	2.287	0.342	5.064	1.668	6	5183	ZINC000261498679
9.1	-9.0	-8.8	0.15	1.513	0.671	4.172	2.124	6	5184	ZINC000256079721
9.1	-11.1	-10.7	0.24	2.511	0.403	4.397	2.461	6	5185	ZINC000118937126
9.1	-9.5	-9.1	0.23	3.078	0.405	5.559	2.279	6	5186	ZINC000014644683

9.1	-8.9	-8.4	0.29	2.127	0.370	4.652	3.094	6	5187	ZINC000015156994
9.1	-10.6	-9.2	0.66	2.633	0.508	6.763	2.578	6	5188	ZINC000253589634
9.1	-10.3	-10.2	0.09	2.946	0.455	5.513	2.403	6	5189	ZINC000257549721
9.1	-10.3	-10.0	0.34	2.429	0.295	5.636	1.938	6	5190	ZINC000257397149
9.1	-12.0	-11.1	0.41	1.909	0.445	5.679	2.067	6	5191	ZINC000257467834
9.1	-11.0	-10.8	0.13	2.435	0.409	5.146	2.022	6	5192	ZINC000257404085
9.1	-11.2	-10.8	0.31	2.383	0.564	7.663	2.424	6	5193	ZINC000257379674
9.1	-10.3	-10.0	0.17	2.214	0.648	4.829	2.742	6	5194	ZINC000257491428
9.0	-10.5	-10.0	0.32	2.069	0.329	6.507	2.775	6	5195	ZINC000257488807
9.0	-9.9	-9.3	0.30	2.110	0.305	5.312	2.225	6	5196	ZINC000257490154
9.0	-11.6	-10.9	0.32	2.601	0.482	6.506	1.377	6	5197	ZINC000257396097
9.0	-11.1	-10.6	0.51	2.219	0.660	4.764	2.044	6	5198	ZINC000029043232
9.0	-9.0	-8.8	0.14	2.475	0.319	6.540	1.975	6	5199	ZINC000086041882
9.0	-9.9	-9.5	0.26	2.371	0.485	6.038	2.964	6	5200	ZINC000005158748
9.0	-10.7	-10.2	0.34	2.596	0.500	6.903	2.021	6	5201	ZINC000257525014

9.0	-10.3	-10.2	0.09	2.523	0.586	6.452	2.303	6	5202	ZINC000014765651
9.0	-9.5	-9.2	0.16	3.462	0.621	4.529	0.515	6	5203	ZINC000005567794
9.0	-10.4	-10.0	0.22	2.654	0.170	8.100	2.124	6	5204	ZINC000257426053
9.0	-10.9	-10.5	0.23	1.830	0.653	4.763	1.900	6	5205	ZINC000100828161
9.0	-11.1	-11.0	0.18	2.101	0.146	4.726	1.932	6	5206	ZINC000100779580
9.0	-10.9	-10.5	0.24	2.311	0.159	5.901	0.556	6	5207	ZINC000257404084
9.0	-9.3	-9.2	0.10	2.203	0.779	6.771	3.594	6	5208	ZINC000014931144
9.0	-8.7	-8.4	0.20	2.424	0.199	5.291	2.018	6	5209	ZINC000253617706
9.0	-10.5	-10.2	0.16	2.372	0.280	6.490	1.543	6	5210	ZINC000100826965
9.0	-9.9	-9.5	0.25	2.552	0.277	8.462	0.253	6	5211	ZINC000257389781
9.0	-9.7	-9.6	0.08	2.546	0.865	6.394	3.147	6	5212	ZINC000257771087
9.0	-9.9	-9.8	0.19	1.803	0.480	4.469	2.570	6	5213	ZINC000095620798
9.0	-10.0	-9.7	0.16	3.607	0.481	5.498	0.550	6	5214	ZINC000085961347
9.0	-11.4	-10.8	0.27	1.882	0.249	7.260	0.056	6	5215	ZINC000118937505
9.0	-9.2	-8.9	0.16	1.738	0.298	3.655	1.128	6	5216	ZINC000014645694

9.0	-10.2	-10.0	0.15	1.967	0.485	4.916	2.285	6	5217	ZINC000257803254
9.0	-11.6	-11.1	0.31	2.114	0.563	4.129	1.557	6	5218	ZINC000086003248
9.0	-10.7	-10.2	0.32	2.155	0.716	4.568	2.322	6	5219	ZINC000257507787
9.0	-9.8	-9.6	0.17	2.344	1.126	4.516	2.618	6	5220	ZINC000253589639
8.9	-8.9	-8.6	0.16	1.365	0.410	3.828	3.240	6	5221	ZINC000257805170
8.9	-9.0	-8.7	0.19	2.552	0.366	5.610	1.643	6	5222	ZINC000014826856
8.9	-10.0	-9.3	0.40	2.703	1.359	7.690	1.465	6	5223	ZINC000002557132
8.9	-9.9	-9.3	0.38	2.473	0.594	7.006	2.642	6	5224	ZINC000100826532
8.9	-10.6	-10.2	0.21	2.755	0.384	5.042	1.650	6	5225	ZINC000014951190
8.9	-11.6	-10.7	0.48	1.782	0.358	5.215	2.211	6	5226	ZINC000257466733
8.9	-10.1	-9.7	0.26	2.435	0.606	6.458	2.355	6	5227	ZINC000257392058
8.9	-10.3	-9.7	0.31	2.524	0.777	5.342	2.603	6	5228	ZINC000257444906
8.9	-10.8	-9.8	0.60	2.036	0.153	5.432	1.972	6	5229	ZINC000100822463
8.9	-11.1	-10.5	0.32	2.255	0.522	5.533	1.846	6	5230	ZINC000100784095
8.9	-11.6	-10.7	0.71	2.105	0.244	6.910	0.265	6	5231	ZINC000014612724

8.9	-7.9	-7.6	0.21	2.197	0.624	4.454	2.774	6	5232	ZINC000012496837
8.9	-9.5	-9.2	0.16	2.084	0.577	3.124	1.194	6	5233	ZINC000085936716
8.9	-9.8	-9.4	0.19	2.100	0.205	6.085	0.937	6	5234	ZINC000100825673
8.9	-10.1	-9.5	0.35	3.516	0.478	4.914	0.843	6	5235	ZINC000257759416
8.9	-9.8	-9.6	0.20	2.125	0.174	7.140	0.910	6	5236	ZINC000085615793
8.9	-10.4	-10.1	0.20	2.419	0.175	5.645	1.309	6	5237	ZINC000100826961
8.9	-10.1	-9.5	0.33	3.354	0.531	4.565	0.873	6	5238	ZINC000257759414
8.9	-11.9	-11.5	0.22	1.912	0.563	4.616	2.564	6	5239	ZINC000257552356
8.9	-11.0	-10.2	0.42	2.611	0.722	5.999	2.801	6	5240	ZINC000257504567
8.9	-10.1	-9.4	0.39	3.189	0.478	4.558	0.833	6	5241	ZINC000100826041
8.9	-9.5	-9.2	0.17	3.030	0.801	5.541	2.379	6	5242	ZINC000040165531
8.9	-10.2	-10.0	0.14	2.187	0.373	5.478	2.683	6	5243	ZINC000257488808
8.9	-10.2	-9.8	0.31	2.528	0.618	5.534	1.784	6	5244	ZINC000013373246
8.9	-10.5	-10.4	0.06	3.483	0.319	6.118	2.007	6	5245	ZINC000257541752
8.9	-10.1	-9.4	0.39	3.539	0.444	4.954	0.792	6	5246	ZINC000100826045

8.9	-10.5	-9.9	0.32	2.456	0.549	6.139	2.602	6	5247	ZINC000118937162
8.9	-8.8	-8.5	0.16	2.041	0.561	5.899	3.109	6	5248	ZINC000003644978
8.9	-9.9	-9.6	0.25	2.219	0.278	6.322	0.739	6	5249	ZINC000100825654
8.9	-11.1	-10.7	0.29	1.956	0.145	4.848	1.727	6	5250	ZINC000257557431
8.9	-9.9	-9.6	0.18	1.950	0.580	4.204	2.319	6	5251	ZINC000014828688
8.9	-10.1	-9.5	0.35	3.526	0.514	4.921	0.889	6	5252	ZINC000238780928
8.9	-10.1	-9.4	0.47	3.497	0.351	5.628	1.695	6	5253	ZINC000257759415
8.9	-9.4	-9.0	0.26	2.700	0.653	7.462	3.697	6	5254	ZINC000014811587
8.9	-10.1	-9.5	0.36	3.532	0.450	4.920	0.795	6	5255	ZINC000100826042
8.9	-10.6	-9.5	0.73	2.115	0.255	4.594	1.371	6	5256	ZINC000100826094
8.9	-10.8	-10.3	0.29	2.217	0.272	5.858	1.206	6	5257	ZINC000085742170
8.9	-8.6	-8.3	0.21	1.702	0.758	3.179	0.865	6	5258	ZINC000008217411
8.9	-9.8	-9.3	0.29	2.584	0.658	5.272	2.795	6	5259	ZINC000257541039
8.9	-9.0	-8.4	0.34	2.300	0.603	4.731	2.809	6	5260	ZINC000257452427
8.9	-10.1	-9.4	0.39	3.550	0.459	4.972	0.805	6	5261	ZINC000100826039

8.9	-9.9	-9.5	0.32	2.314	0.197	6.997	0.550	6	5262	ZINC000100825656
8.9	-8.6	-8.4	0.19	2.440	0.324	4.223	0.353	6	5263	ZINC000040164549
8.9	-8.5	-8.4	0.08	2.988	0.591	7.172	2.343	6	5264	ZINC000014757342
8.8	-9.6	-9.1	0.23	3.404	0.906	6.020	2.314	6	5265	ZINC000013374852
8.8	-8.6	-8.4	0.14	2.672	0.142	4.488	0.278	6	5266	ZINC000253617708
8.8	-11.7	-11.7	0.08	2.131	0.356	5.639	1.850	6	5267	ZINC000100823153
8.8	-10.8	-10.1	0.41	2.666	0.692	5.704	2.630	6	5268	ZINC000257570254
8.8	-8.6	-8.3	0.20	2.605	0.170	4.433	0.307	6	5269	ZINC000008551169
8.8	-12.3	-11.6	0.43	1.464	0.401	6.005	0.952	6	5270	ZINC000095620572
8.8	-11.7	-11.6	0.09	2.245	0.399	6.773	0.802	6	5271	ZINC000100823151
8.8	-10.3	-9.5	0.54	2.394	0.223	7.254	1.207	6	5272	ZINC000044430967
8.8	-9.3	-9.0	0.19	3.016	0.925	6.346	2.672	6	5273	ZINC000040165530
8.8	-11.1	-10.2	0.52	2.535	0.698	6.288	0.976	6	5274	ZINC000095619714
8.8	-9.8	-9.6	0.13	2.120	0.353	5.192	2.343	6	5275	ZINC000000897618
8.8	-8.9	-8.5	0.18	3.660	0.443	6.206	1.899	6	5276	ZINC000014613167

8.8	-10.4	-9.6	0.39	2.097	0.973	3.043	1.115	6	5277	ZINC000015262018
8.8	-10.6	-9.5	0.53	2.159	0.753	4.992	3.173	6	5278	ZINC000253589635
8.8	-10.6	-10.3	0.15	2.251	0.242	5.358	0.952	6	5279	ZINC000100823105
8.8	-11.5	-10.7	0.40	2.160	0.347	5.227	2.119	6	5280	ZINC000257409646
8.8	-11.7	-11.6	0.12	1.922	0.278	4.306	1.456	6	5281	ZINC000100823147
8.8	-10.4	-10.0	0.19	3.300	0.664	6.652	2.822	6	5282	ZINC000013515300
8.8	-10.4	-9.8	0.31	2.102	0.559	5.531	2.389	6	5283	ZINC000150352517
8.8	-10.5	-10.3	0.10	2.938	0.643	6.609	2.920	6	5284	ZINC000257541754
8.8	-8.6	-8.5	0.10	3.257	0.353	5.392	1.381	6	5285	ZINC000015257355
8.8	-11.7	-11.5	0.21	2.096	0.389	4.801	1.266	6	5286	ZINC000257391701
8.8	-10.4	-10.4	0.08	3.054	0.757	5.647	2.326	6	5287	ZINC000257541751
8.8	-9.7	-9.3	0.27	2.117	0.420	6.454	1.856	6	5288	ZINC000257415567
8.8	-10.3	-9.8	0.37	1.957	0.462	4.880	1.934	6	5289	ZINC000257504269
8.8	-10.3	-9.6	0.38	2.816	0.454	6.285	2.310	6	5290	ZINC000257461822
8.8	-10.5	-10.3	0.15	2.692	0.142	6.472	1.360	6	5291	ZINC000100826964

8.8	-9.5	-9.3	0.19	2.416	1.255	4.843	2.818	6	5292	ZINC000118936954
8.8	-8.7	-8.4	0.23	2.638	0.122	5.433	1.959	6	5293	ZINC000008215921
8.8	-11.0	-10.0	0.63	2.083	0.824	3.526	1.294	6	5294	ZINC000013358410
8.8	-9.8	-9.3	0.27	2.401	0.526	6.128	2.874	6	5295	ZINC000257546800
8.8	-10.8	-10.7	0.09	2.496	0.366	7.601	2.073	6	5296	ZINC000100828734
8.8	-11.1	-10.2	0.41	2.075	0.306	5.478	2.934	6	5297	ZINC000257462536
8.8	-10.9	-10.5	0.35	2.329	0.506	5.137	1.898	6	5298	ZINC000257386778
8.8	-8.5	-8.3	0.20	2.915	0.888	5.411	2.573	6	5299	ZINC000014648318
8.8	-11.6	-11.0	0.45	1.869	0.241	6.752	2.039	6	5300	ZINC000085744654
8.7	-8.5	-8.3	0.15	1.678	0.773	3.159	0.877	6	5301	ZINC000008216112
8.7	-9.6	-9.3	0.20	2.553	0.903	5.128	3.257	6	5302	ZINC000005761332
8.7	-8.7	-8.5	0.12	1.828	0.276	4.520	2.624	6	5303	ZINC000014645697
8.7	-10.3	-10.1	0.12	3.244	0.661	5.460	2.249	6	5304	ZINC000013515301
8.7	-10.3	-10.1	0.16	3.340	0.633	6.647	2.866	6	5305	ZINC000004099035
8.7	-9.3	-8.9	0.20	2.540	0.692	4.086	2.148	6	5306	ZINC000014491678

8.7	-10.7	-10.4	0.23	1.809	0.295	5.920	2.799	6	5307	ZINC000257470605
8.7	-11.0	-10.5	0.24	2.270	0.700	5.757	0.559	6	5308	ZINC000100822773
8.7	-10.4	-10.3	0.15	2.134	0.821	4.836	2.777	6	5309	ZINC000100778910
8.7	-9.0	-8.8	0.17	2.312	0.469	5.615	1.558	6	5310	ZINC000257704824
8.7	-11.7	-11.6	0.11	1.997	0.279	5.317	1.814	6	5311	ZINC000100823150
8.7	-9.9	-9.3	0.40	2.319	0.971	4.123	2.813	6	5312	ZINC000014658117
8.7	-10.2	-10.1	0.11	1.944	0.161	4.350	2.171	6	5313	ZINC000253530771
8.7	-10.3	-10.1	0.13	3.244	0.653	5.454	2.241	6	5314	ZINC000013515299
8.7	-11.1	-10.0	0.55	1.824	0.386	5.320	1.710	6	5315	ZINC000100780836
8.7	-10.3	-10.1	0.12	3.262	0.631	5.457	2.230	6	5316	ZINC000150374455
8.7	-10.1	-9.8	0.20	2.513	0.087	7.101	3.203	6	5317	ZINC000014930811
8.7	-10.0	-9.6	0.25	2.370	0.546	4.739	1.970	6	5318	ZINC000257549390
8.7	-10.3	-10.1	0.12	3.267	0.638	5.462	2.246	6	5319	ZINC000150374458
8.7	-10.8	-10.5	0.19	2.326	0.289	7.084	1.591	6	5320	ZINC000100825169
8.7	-10.2	-10.0	0.17	2.362	0.366	5.714	1.383	6	5321	ZINC000253388215

8.7	-10.0	-9.8	0.17	1.921	0.500	3.827	2.112	6	5322	ZINC000257476393
8.7	-11.1	-10.9	0.21	2.388	0.347	6.444	1.401	6	5323	ZINC000100826091
8.7	-8.6	-8.3	0.24	2.319	0.645	3.983	0.961	6	5324	ZINC000008215914
8.7	-10.4	-10.3	0.04	3.478	0.336	7.161	2.391	6	5325	ZINC000100778913
8.7	-10.8	-10.5	0.30	2.429	0.434	6.275	1.714	6	5326	ZINC000014953941
8.7	-11.5	-11.0	0.29	2.454	0.812	6.832	1.570	6	5327	ZINC000014612728
8.7	-11.0	-10.5	0.26	2.701	0.346	7.077	0.576	6	5328	ZINC000100825717
8.7	-10.9	-10.0	0.46	1.819	0.269	4.764	2.103	6	5329	ZINC000100822824
8.7	-9.9	-9.7	0.12	2.465	0.654	4.161	1.933	6	5330	ZINC000085589043
8.7	-8.6	-8.3	0.23	2.284	0.567	4.934	2.304	6	5331	ZINC000008551168
8.7	-9.9	-9.5	0.21	2.051	0.252	6.149	2.321	6	5332	ZINC000257389783
8.7	-9.8	-9.2	0.42	2.589	0.301	7.469	2.328	6	5333	ZINC000005158783
8.7	-9.7	-9.6	0.05	2.613	0.823	5.376	2.879	6	5334	ZINC000201392712
8.7	-10.9	-10.2	0.36	3.155	0.594	5.680	2.287	6	5335	ZINC000100776827
8.7	-10.5	-9.7	0.39	2.030	0.169	4.934	1.515	6	5336	ZINC000257399599

8.7	-10.3	-9.9	0.30	1.936	0.258	6.989	3.119	6	5337	ZINC000257567734
8.7	-10.8	-10.3	0.29	2.392	0.275	6.639	2.109	6	5338	ZINC000100821964
8.7	-8.6	-8.4	0.18	2.573	0.099	5.424	1.955	6	5339	ZINC000008215923
8.7	-11.0	-10.6	0.26	1.734	0.307	4.970	2.383	6	5340	ZINC000100779099
8.7	-11.6	-11.1	0.31	2.101	0.448	6.858	0.318	6	5341	ZINC000027757497
8.7	-10.3	-9.2	0.53	2.342	0.560	5.721	2.029	6	5342	ZINC000100822475
8.7	-7.6	-7.4	0.11	4.257	1.810	8.289	1.807	6	5343	ZINC000034417894
8.7	-10.6	-9.8	0.35	2.001	0.301	4.672	1.503	6	5344	ZINC000257513125
8.7	-9.8	-9.5	0.19	2.219	0.223	5.120	2.282	6	5345	ZINC000238753105
8.7	-10.4	-10.2	0.21	2.859	0.270	5.932	2.481	6	5346	ZINC000100778915
8.7	-10.4	-10.3	0.07	2.528	0.468	4.380	2.374	6	5347	ZINC000257541753
8.7	-10.4	-10.3	0.11	2.673	0.151	7.629	1.506	6	5348	ZINC000100825480
8.7	-10.8	-9.9	0.47	2.730	1.094	5.264	2.127	6	5349	ZINC000014859661
8.7	-9.5	-9.3	0.17	2.312	0.364	5.260	2.322	6	5350	ZINC000038852736
8.7	-11.0	-10.5	0.26	2.342	0.485	6.708	2.232	6	5351	ZINC000150363934

8.7	-10.5	-9.8	0.47	2.096	0.361	4.798	2.162	6	5352	ZINC000038750548
8.7	-11.2	-10.9	0.26	1.775	0.453	3.453	1.649	6	5353	ZINC000100784091
8.7	-10.8	-10.3	0.38	3.215	0.514	6.580	0.594	6	5354	ZINC000015061786
8.6	-10.1	-9.5	0.26	1.882	0.456	4.139	2.171	6	5355	ZINC000257497572
8.6	-9.3	-9.2	0.10	2.494	0.815	5.300	2.618	6	5356	ZINC000040165529
8.6	-11.5	-11.0	0.28	2.113	0.389	5.077	1.784	6	5357	ZINC000005699594
8.6	-10.2	-10.1	0.08	2.674	0.890	7.552	1.231	6	5358	ZINC000100822990
8.6	-10.0	-9.3	0.34	2.288	0.401	7.091	2.257	6	5359	ZINC000257383375
8.6	-8.8	-8.6	0.12	2.024	0.669	6.822	3.981	6	5360	ZINC000257493296
8.6	-10.5	-9.6	0.46	2.613	0.709	6.120	2.373	6	5361	ZINC000257392060
8.6	-10.4	-9.8	0.32	3.038	0.194	6.895	2.038	6	5362	ZINC000028535504
8.6	-10.9	-10.6	0.20	2.354	0.331	6.727	0.751	6	5363	ZINC000150369715
8.6	-9.2	-9.0	0.12	2.588	0.905	5.937	3.173	6	5364	ZINC000014491682
8.6	-10.3	-10.0	0.23	2.696	0.360	5.446	1.116	6	5365	ZINC000257458886
8.6	-12.3	-11.2	0.62	2.117	0.388	6.282	0.417	6	5366	ZINC000136707361

8.6	-9.3	-9.0	0.27	2.584	0.620	7.259	3.735	6	5367	ZINC000014813787
8.6	-10.0	-9.4	0.31	2.234	0.705	5.833	3.325	6	5368	ZINC000014644774
8.6	-10.6	-10.0	0.41	2.019	0.522	3.733	2.963	6	5369	ZINC000004096163
8.6	-8.6	-8.4	0.19	2.242	0.598	3.969	0.940	6	5370	ZINC000008215918
8.6	-8.6	-8.4	0.21	2.633	0.121	5.406	1.888	6	5371	ZINC000253617704
8.6	-8.6	-8.4	0.14	2.136	0.486	4.103	2.359	6	5372	ZINC000256068537
8.6	-10.1	-9.8	0.18	2.183	0.293	7.671	2.295	6	5373	ZINC000257559678
8.6	-10.3	-9.8	0.40	1.814	0.282	6.354	2.852	6	5374	ZINC000257470602
8.6	-10.0	-9.6	0.29	2.511	0.483	5.656	3.523	6	5375	ZINC000100826864
8.6	-8.9	-8.7	0.09	2.215	0.524	6.380	2.989	6	5376	ZINC000100048689
8.6	-10.9	-10.2	0.36	3.534	0.416	7.495	1.580	6	5377	ZINC000100776826
8.6	-10.4	-9.9	0.33	2.176	0.339	5.840	1.485	6	5378	ZINC000013374463
8.6	-10.5	-10.0	0.32	2.812	0.251	7.028	1.617	6	5379	ZINC000100829042
8.6	-10.0	-9.6	0.24	2.450	0.316	8.060	0.725	6	5380	ZINC000257458219
8.6	-8.9	-8.5	0.27	2.603	0.479	4.991	2.102	6	5381	ZINC000014757413

8.6	-10.9	-10.2	0.37	3.261	0.289	6.298	2.380	6	5382	ZINC000100824381
8.6	-10.1	-9.8	0.14	1.928	0.257	6.398	3.135	6	5383	ZINC000257562845
8.6	-10.8	-10.2	0.33	1.911	0.631	5.425	3.279	6	5384	ZINC000118937038
8.6	-10.7	-10.3	0.20	3.008	0.166	8.136	0.215	6	5385	ZINC000257423420
8.6	-8.5	-8.3	0.18	1.795	0.722	4.208	2.228	6	5386	ZINC000008551167
8.6	-9.4	-9.2	0.23	1.671	0.375	5.653	3.866	6	5387	ZINC000014489967
8.6	-9.6	-9.4	0.26	2.714	0.247	6.564	2.431	6	5388	ZINC000257565710
8.6	-9.9	-9.4	0.26	3.758	0.615	6.466	1.103	6	5389	ZINC000014650504
8.6	-10.0	-9.8	0.16	2.074	0.279	6.994	1.133	6	5390	ZINC000257470912
8.6	-11.5	-11.1	0.28	2.155	0.658	4.989	2.557	6	5391	ZINC000100773808
8.6	-9.9	-9.6	0.22	2.143	0.217	6.710	0.674	6	5392	ZINC000257470911
8.6	-10.2	-9.8	0.24	2.882	0.679	6.275	2.250	6	5393	ZINC000100822987
8.6	-9.6	-9.2	0.27	2.050	0.446	5.564	3.671	6	5394	ZINC000000622260
8.6	-9.8	-9.5	0.24	2.687	0.877	6.726	3.099	6	5395	ZINC000003652281
8.5	-10.1	-9.9	0.27	2.030	0.574	4.836	3.080	6	5396	ZINC000257567737

8.5	-11.3	-10.9	0.27	2.052	0.590	3.383	0.724	6	5397	ZINC000118937216
8.5	-10.3	-9.8	0.35	2.550	0.571	6.045	2.191	6	5398	ZINC000253590164
8.5	-10.1	-9.9	0.18	2.431	1.027	8.078	1.105	6	5399	ZINC000257523143
8.5	-11.3	-11.0	0.19	2.225	0.307	6.630	1.017	6	5400	ZINC000150368153
8.5	-9.4	-9.2	0.14	2.924	0.887	5.039	2.738	6	5401	ZINC000040165532
8.5	-10.2	-9.6	0.30	2.620	1.019	6.187	2.622	6	5402	ZINC000006416346
8.5	-10.9	-10.6	0.20	2.745	0.381	7.764	2.013	6	5403	ZINC000257489244
8.5	-9.6	-9.0	0.38	2.490	0.449	6.806	2.336	6	5404	ZINC000015261717
8.5	-9.5	-9.3	0.19	2.241	0.403	5.855	2.727	6	5405	ZINC000085863159
8.5	-11.4	-11.3	0.09	2.058	0.681	5.902	2.121	6	5406	ZINC000257391699
8.5	-10.5	-10.4	0.09	1.991	0.350	6.197	2.483	6	5407	ZINC000257382538
8.5	-11.6	-10.9	0.56	2.366	0.875	6.333	0.468	6	5408	ZINC000100822780
8.5	-11.3	-10.9	0.27	1.956	0.522	3.154	0.578	6	5409	ZINC000118937217
8.5	-10.7	-10.4	0.30	2.332	0.294	6.020	1.822	6	5410	ZINC000257596846
8.5	-9.5	-8.8	0.50	1.853	0.533	5.506	2.001	6	5411	ZINC000257554974

8.5	-8.9	-8.7	0.22	2.973	0.901	7.986	3.166	6	5412	ZINC000006129939
8.5	-9.6	-9.1	0.23	2.121	0.363	7.193	2.625	6	5413	ZINC000100775216
8.5	-11.6	-11.3	0.28	2.089	0.721	5.239	1.179	6	5414	ZINC000014636470
8.5	-12.8	-11.0	1.02	2.321	0.197	6.512	0.501	5	5415	ZINC000026189531
8.5	-9.5	-9.0	0.28	2.135	0.808	4.318	2.880	6	5416	ZINC000014757246
8.5	-10.6	-10.1	0.28	1.839	0.656	4.633	2.376	6	5417	ZINC000100772853
8.5	-10.2	-9.8	0.25	2.068	0.383	5.675	3.330	6	5418	ZINC000257457869
8.5	-10.3	-10.0	0.19	2.363	0.367	5.703	1.440	6	5419	ZINC000067911928
8.5	-11.2	-10.1	0.63	1.951	0.644	5.601	2.722	6	5420	ZINC000100890069
8.5	-8.4	-8.2	0.09	1.210	0.622	6.539	2.870	6	5421	ZINC000005663092
8.5	-9.6	-9.3	0.17	2.059	0.201	6.044	2.193	6	5422	ZINC000086044997
8.5	-11.1	-11.0	0.12	1.670	0.179	6.931	3.151	6	5423	ZINC000118937377
8.5	-10.6	-10.2	0.25	2.944	0.195	6.558	1.125	6	5424	ZINC000032785896
8.5	-10.1	-9.6	0.26	2.427	0.464	8.120	2.982	6	5425	ZINC000257392554
8.5	-10.9	-9.8	0.53	2.946	0.211	7.102	2.411	6	5426	ZINC000100824383

8.5	-10.4	-10.2	0.19	2.368	0.322	6.459	2.778	6	5427	ZINC000253589626
8.5	-9.9	-9.5	0.23	2.106	0.223	6.100	1.632	6	5428	ZINC000100823798
8.5	-9.6	-9.3	0.18	2.336	1.342	3.845	2.454	6	5429	ZINC000257500356
8.5	-10.8	-10.2	0.38	2.052	0.230	5.245	2.994	6	5430	ZINC000257378281
8.5	-10.6	-10.0	0.40	2.265	0.530	4.334	2.193	6	5431	ZINC000257477218
8.5	-8.9	-8.7	0.13	2.546	0.482	4.841	2.827	6	5432	ZINC000014813773
8.5	-7.8	-7.5	0.16	2.216	0.746	4.218	2.770	6	5433	ZINC000030731390
8.5	-11.1	-10.6	0.30	2.535	0.798	5.754	1.613	6	5434	ZINC000257507606
8.5	-11.3	-10.9	0.23	2.012	0.585	3.150	0.601	6	5435	ZINC000118937218
8.5	-10.3	-10.0	0.27	2.211	0.582	6.473	2.968	6	5436	ZINC000118937362
8.4	-10.4	-10.1	0.22	2.749	0.532	7.408	0.555	6	5437	ZINC000257631299
8.4	-10.0	-9.3	0.42	1.387	0.672	5.276	1.187	6	5438	ZINC000085561004
8.4	-9.5	-8.9	0.37	2.626	0.745	7.270	2.590	6	5439	ZINC000015261719
8.4	-11.5	-11.0	0.28	2.209	0.180	6.808	0.810	6	5440	ZINC000238771764
8.4	-9.8	-9.5	0.23	2.179	0.228	6.155	0.919	6	5441	ZINC000100825672

8.4	-11.4	-10.8	0.45	3.895	0.316	7.619	1.050	6	5442	ZINC000257443654
8.4	-9.5	-9.2	0.21	2.104	0.517	4.164	1.879	6	5443	ZINC000257500358
8.4	-10.5	-10.0	0.22	2.570	0.264	5.707	1.208	6	5444	ZINC000257736999
8.4	-10.3	-9.3	0.51	2.273	0.584	4.726	2.114	6	5445	ZINC000257444908
8.4	-10.2	-9.9	0.21	2.545	0.236	6.026	1.721	6	5446	ZINC000095617636
8.4	-12.0	-10.8	0.63	2.672	0.554	6.785	1.096	6	5447	ZINC000257438013
8.4	-9.9	-9.7	0.12	1.632	0.702	5.801	2.584	6	5448	ZINC000005425409
8.4	-11.4	-11.3	0.11	2.004	0.273	5.340	1.786	6	5449	ZINC000257391700
8.4	-8.9	-8.5	0.22	1.961	0.225	4.768	2.580	6	5450	ZINC000257505314
8.4	-10.7	-10.0	0.36	2.404	0.071	6.699	0.635	6	5451	ZINC000257484773
8.4	-10.6	-10.4	0.21	2.036	0.474	4.062	2.341	6	5452	ZINC000257512053
8.4	-10.9	-10.5	0.22	2.656	0.281	6.590	2.414	6	5453	ZINC000118937123
8.4	-11.4	-10.8	0.45	3.889	0.313	7.626	1.035	6	5454	ZINC000257713657
8.4	-9.5	-9.3	0.14	2.181	1.360	4.041	2.374	6	5455	ZINC000118936955
8.4	-9.9	-9.2	0.34	2.014	0.381	5.201	3.359	6	5456	ZINC000257487950

8.4	-9.9	-9.5	0.28	2.935	0.321	6.090	1.012	6	5457	ZINC000015210128
8.4	-10.8	-9.9	0.50	2.186	0.663	4.939	2.769	6	5458	ZINC000257430575
8.4	-10.7	-10.2	0.24	1.964	0.363	5.128	2.497	6	5459	ZINC000257406857
8.4	-12.0	-10.9	0.57	2.545	0.499	5.321	0.954	6	5460	ZINC000257438011
8.4	-10.6	-10.1	0.24	1.963	0.200	4.952	1.990	6	5461	ZINC000100777713
8.4	-12.0	-10.9	0.59	2.491	0.506	6.159	1.283	6	5462	ZINC000100779102
8.4	-9.8	-9.4	0.28	2.406	0.262	8.285	0.278	6	5463	ZINC000118937305
8.4	-11.4	-10.8	0.45	3.898	0.318	7.627	1.043	6	5464	ZINC000257713645
8.4	-10.4	-10.2	0.15	2.055	0.672	4.974	1.931	6	5465	ZINC000257422697
8.4	-11.0	-10.2	0.39	2.306	0.487	6.677	3.567	6	5466	ZINC000085842743
8.4	-9.3	-9.1	0.17	2.459	0.620	7.194	2.521	6	5467	ZINC000014611791
8.4	-11.4	-10.8	0.45	3.897	0.319	7.623	1.044	6	5468	ZINC000257713649
8.4	-11.4	-10.7	0.47	3.696	0.564	6.838	1.557	6	5469	ZINC000257443655
8.4	-10.9	-10.8	0.10	2.172	0.150	6.645	2.417	6	5470	ZINC000257513308
8.4	-9.8	-9.6	0.14	2.238	0.145	6.119	0.560	6	5471	ZINC000257484772

8.4	-9.5	-9.3	0.16	2.318	1.351	4.136	2.310	6	5472	ZINC000257500359
8.4	-10.2	-9.9	0.21	2.333	0.360	6.002	1.720	6	5473	ZINC000253388217
8.4	-11.4	-10.8	0.47	3.636	0.545	7.008	1.661	6	5474	ZINC000257443652
8.4	-10.3	-10.1	0.12	3.229	0.971	5.538	1.919	6	5475	ZINC000031460595
8.4	-10.8	-10.4	0.24	1.993	0.233	6.608	2.130	6	5476	ZINC000257402143
8.4	-11.4	-10.8	0.45	3.886	0.311	7.609	1.055	6	5477	ZINC000257713653
8.4	-11.3	-10.8	0.31	2.163	0.236	6.446	0.968	6	5478	ZINC000150366295
8.4	-9.9	-9.5	0.28	2.137	0.208	6.149	1.596	6	5479	ZINC000100823800
8.4	-9.5	-9.3	0.17	2.693	1.087	4.144	2.381	6	5480	ZINC000118936956
8.4	-9.8	-9.0	0.41	2.578	0.087	8.564	0.173	6	5481	ZINC000257495603
8.4	-11.2	-10.8	0.22	2.502	0.396	4.803	2.527	6	5482	ZINC000118937219
8.4	-9.2	-9.0	0.20	3.010	1.014	7.131	1.744	6	5483	ZINC000204637817
8.4	-9.8	-9.4	0.22	2.523	0.157	8.150	0.249	6	5484	ZINC000118937307
8.4	-9.5	-9.3	0.16	2.537	1.159	4.347	2.275	6	5485	ZINC000118936953
8.4	-10.2	-9.7	0.24	2.135	0.434	4.824	1.722	6	5486	ZINC000242543350

8.4	-9.7	-8.9	0.46	1.706	0.102	2.788	0.274	6	5487	ZINC000257693600
8.4	-9.5	-9.3	0.14	2.332	1.351	4.169	2.362	6	5488	ZINC000257500357
8.4	-9.9	-9.6	0.23	2.144	0.217	6.706	0.669	6	5489	ZINC000100823802
8.4	-10.2	-9.9	0.21	2.824	0.646	6.352	3.372	6	5490	ZINC000257710435
8.4	-11.4	-10.8	0.45	3.897	0.319	7.636	1.035	6	5491	ZINC000257443653
8.4	-9.9	-9.5	0.24	2.101	0.227	6.105	1.633	6	5492	ZINC000257470910
8.4	-10.3	-10.1	0.20	2.495	0.682	5.395	1.654	6	5493	ZINC000257507788
8.4	-9.8	-9.5	0.21	2.324	0.185	7.298	1.943	6	5494	ZINC000118937304
8.4	-9.9	-9.6	0.21	2.140	0.220	6.702	0.657	6	5495	ZINC000100823801
8.3	-8.6	-8.4	0.12	2.223	0.512	7.962	2.999	6	5496	ZINC000014708535
8.3	-11.7	-11.3	0.31	2.095	0.271	6.319	3.304	6	5497	ZINC000257395001
8.3	-10.2	-10.0	0.17	2.432	0.496	3.998	1.193	6	5498	ZINC000077301904
8.3	-10.9	-10.4	0.25	2.711	0.271	6.563	2.375	6	5499	ZINC000118937124
8.3	-12.0	-10.9	0.57	2.555	0.480	5.340	0.953	6	5500	ZINC000100779101
8.3	-9.4	-9.2	0.13	2.466	0.672	6.079	2.397	6	5501	ZINC000100823827

8.3	-9.1	-8.8	0.27	2.911	0.583	4.902	1.983	6	5502	ZINC000085956026
8.3	-11.6	-11.5	0.07	2.629	0.311	6.307	0.398	6	5503	ZINC000024215123
8.3	-8.9	-8.3	0.33	2.764	0.443	5.027	2.373	6	5504	ZINC000014757409
8.3	-9.5	-9.4	0.06	2.826	0.474	5.853	2.900	6	5505	ZINC000257586854
8.3	-11.1	-10.7	0.25	2.765	0.847	5.555	1.987	6	5506	ZINC000085915840
8.3	-9.7	-9.3	0.22	2.400	0.456	6.368	3.047	6	5507	ZINC000031159715
8.3	-11.0	-10.6	0.29	2.684	1.011	7.186	3.580	6	5508	ZINC000257805858
8.3	-11.3	-11.1	0.12	2.361	1.155	5.770	0.432	6	5509	ZINC000100826929
8.3	-13.1	-12.7	0.35	2.259	0.712	6.517	0.907	6	5510	ZINC000100777472
8.3	-9.4	-9.0	0.28	2.152	0.181	5.189	2.910	6	5511	ZINC000014488279
8.3	-9.7	-9.3	0.21	2.249	0.612	5.094	2.195	6	5512	ZINC000257476014
8.3	-10.8	-10.4	0.28	2.469	0.492	9.180	0.516	6	5513	ZINC000034083350
8.3	-11.4	-10.7	0.41	2.427	0.544	6.022	1.972	6	5514	ZINC000100784098
8.3	-11.8	-11.2	0.44	2.029	0.727	5.613	1.477	6	5515	ZINC000100825234
8.3	-11.0	-10.5	0.29	2.178	0.600	6.627	2.884	6	5516	ZINC000034083353

8.3	-10.4	-10.0	0.33	2.472	0.338	6.569	0.717	6	5517	ZINC000100773046
8.3	-10.1	-9.7	0.24	2.260	0.654	5.021	2.870	6	5518	ZINC000118937139
8.3	-9.5	-8.9	0.28	1.760	0.178	3.069	0.519	6	5519	ZINC000253497581
8.3	-10.0	-9.5	0.33	2.265	0.333	4.803	1.857	6	5520	ZINC000004097718
8.3	-10.3	-9.8	0.29	2.074	0.147	4.758	2.173	6	5521	ZINC000257618449
8.3	-10.0	-9.8	0.18	2.202	0.357	6.159	1.935	6	5522	ZINC000100825370
8.3	-10.8	-10.2	0.27	2.491	0.707	5.694	2.714	6	5523	ZINC000100829396
8.3	-7.9	-7.7	0.19	2.582	1.573	3.776	1.663	6	5524	ZINC000012358651
8.3	-8.3	-8.1	0.21	0.942	0.797	2.307	0.282	6	5525	ZINC000096077628
8.3	-9.1	-8.9	0.10	2.376	0.308	9.787	0.154	6	5526	ZINC000100820461
8.3	-10.1	-9.8	0.31	2.324	0.651	4.797	2.424	6	5527	ZINC000257430574
8.3	-10.6	-10.3	0.18	2.146	0.367	5.553	2.728	6	5528	ZINC000085813329
8.3	-10.1	-9.8	0.23	2.321	0.473	5.294	1.906	6	5529	ZINC000044430969
8.3	-10.4	-9.9	0.37	1.680	0.521	5.053	3.088	6	5530	ZINC000257675761
8.3	-10.8	-10.6	0.12	2.474	0.313	5.768	2.009	6	5531	ZINC000257666334

8.3	-10.9	-10.4	0.25	2.543	0.451	7.185	2.588	6	5532	ZINC000118937122
8.3	-9.2	-9.0	0.11	1.971	0.743	4.751	3.374	6	5533	ZINC000070457358
8.3	-13.1	-12.7	0.41	2.655	0.466	6.629	1.704	6	5534	ZINC000100777467
8.3	-11.0	-10.6	0.27	1.856	0.364	4.482	2.195	6	5535	ZINC000100779097
8.3	-11.4	-10.5	0.43	2.075	0.319	6.093	1.640	6	5536	ZINC000100779215
8.3	-9.0	-8.7	0.18	2.430	0.739	6.306	3.577	6	5537	ZINC000100046881
8.3	-11.1	-9.8	0.74	2.423	0.908	3.719	1.341	6	5538	ZINC000118937259
8.2	-9.0	-8.8	0.16	2.288	0.113	5.835	1.912	6	5539	ZINC000100772249
8.2	-10.9	-10.6	0.31	2.610	0.381	7.680	0.351	6	5540	ZINC000085813338
8.2	-9.5	-9.2	0.16	3.349	0.907	6.458	2.861	6	5541	ZINC000014760591
8.2	-11.5	-10.9	0.35	2.155	0.523	4.570	2.014	6	5542	ZINC000150369718
8.2	-10.4	-10.1	0.18	2.348	0.353	5.077	1.350	6	5543	ZINC000013374469
8.2	-8.9	-8.8	0.10	2.024	0.420	3.234	0.892	6	5544	ZINC000014491680
8.2	-10.9	-10.4	0.29	2.514	0.670	6.550	2.446	6	5545	ZINC000100829043
8.2	-10.8	-10.5	0.18	2.356	0.636	5.697	2.864	6	5546	ZINC000118937125

8.2	-10.6	-10.3	0.18	1.705	0.170	6.336	2.638	6	5547	ZINC000257573443
8.2	-9.8	-9.0	0.40	2.171	0.655	6.753	3.024	6	5548	ZINC000015060718
8.2	-10.1	-9.9	0.11	2.260	0.090	6.039	1.894	6	5549	ZINC000100777795
8.2	-9.4	-8.9	0.35	2.888	1.049	6.745	3.257	6	5550	ZINC000248267900
8.2	-10.3	-10.1	0.13	2.689	0.160	4.808	1.628	6	5551	ZINC000119379906
8.2	-8.7	-8.5	0.14	3.586	0.543	6.750	2.171	6	5552	ZINC000013783867
8.2	-10.5	-9.9	0.38	2.457	0.065	5.705	1.482	6	5553	ZINC000100826533
8.2	-13.4	-11.6	0.87	2.233	0.251	5.507	0.764	6	5554	ZINC000085561967
8.2	-10.3	-9.7	0.29	3.241	0.269	5.977	0.951	6	5555	ZINC000014829022
8.2	-9.5	-9.2	0.19	3.335	0.834	7.787	1.407	6	5556	ZINC000015252018
8.2	-10.6	-9.9	0.45	2.130	0.519	4.240	2.340	6	5557	ZINC000257477216
8.2	-11.2	-10.8	0.23	2.011	1.001	4.543	3.273	6	5558	ZINC000257555912
8.2	-11.9	-11.0	0.49	2.692	0.608	5.507	0.786	6	5559	ZINC000257438014
8.2	-8.3	-8.2	0.13	1.499	0.704	4.355	2.579	6	5560	ZINC000014648306
8.2	-8.8	-8.5	0.19	2.261	0.739	5.365	2.640	6	5561	ZINC000003644977

8.2	-11.1	-10.7	0.32	2.362	0.289	6.625	2.513	6	5562	ZINC000118937137
8.2	-10.1	-9.4	0.33	2.305	0.610	5.265	2.410	6	5563	ZINC000257462922
8.2	-10.9	-10.5	0.37	2.118	1.009	5.891	1.881	6	5564	ZINC000085917114
8.2	-10.7	-10.1	0.27	2.089	0.229	6.468	1.126	6	5565	ZINC000059762823
8.2	-9.7	-9.5	0.15	2.419	0.108	8.025	0.235	6	5566	ZINC000118937306
8.2	-11.3	-10.8	0.31	1.873	0.378	4.836	1.948	6	5567	ZINC000036370165
8.2	-10.2	-9.5	0.43	2.520	0.465	7.154	2.027	6	5568	ZINC000257592432
8.2	-11.9	-10.9	0.56	2.539	0.493	5.321	0.967	6	5569	ZINC000100779108
8.2	-11.4	-11.1	0.16	2.340	0.351	5.248	1.606	6	5570	ZINC000100784100
8.2	-10.4	-10.0	0.28	1.768	0.269	4.918	2.356	6	5571	ZINC000257388909
8.2	-11.1	-10.8	0.20	1.774	0.858	5.304	2.437	6	5572	ZINC000027758247
8.2	-10.0	-9.6	0.26	2.708	0.334	6.002	2.179	6	5573	ZINC000257394920
8.2	-10.3	-10.1	0.11	1.910	0.468	3.209	0.847	6	5574	ZINC000004216287
8.2	-9.2	-9.0	0.08	3.263	0.185	6.980	2.688	6	5575	ZINC000238733145
8.2	-11.6	-11.1	0.37	2.436	0.363	6.135	1.293	6	5576	ZINC000100780304

8.2	-9.1	-8.9	0.12	2.696	0.778	6.764	1.975	6	5577	ZINC000014695525
8.1	-10.7	-10.1	0.35	2.320	0.597	4.183	2.138	6	5578	ZINC000085935111
8.1	-9.7	-9.5	0.13	2.625	0.497	6.351	1.964	6	5579	ZINC000257602912
8.1	-8.8	-8.6	0.12	4.211	0.516	7.482	1.571	6	5580	ZINC000014450977
8.1	-9.6	-9.3	0.18	1.813	0.390	7.325	3.854	6	5581	ZINC000100825873
8.1	-10.0	-9.7	0.21	2.551	0.208	7.551	1.196	6	5582	ZINC000005158749
8.1	-9.0	-8.9	0.11	2.341	0.091	6.026	1.777	6	5583	ZINC000257374615
8.1	-10.7	-10.1	0.30	2.184	0.567	4.753	1.951	6	5584	ZINC000014858268
8.1	-10.6	-10.1	0.29	2.802	0.935	6.005	2.581	6	5585	ZINC000100776811
8.1	-8.9	-8.7	0.14	2.939	0.603	7.641	3.512	6	5586	ZINC000059728952
8.1	-11.8	-11.3	0.29	2.121	0.622	5.867	1.437	6	5587	ZINC000014612723
8.1	-9.0	-8.9	0.11	2.302	0.146	5.298	1.518	6	5588	ZINC000257374614
8.1	-10.6	-10.1	0.30	2.860	0.825	6.169	2.290	6	5589	ZINC000257419940
8.1	-9.0	-8.9	0.09	2.355	0.098	6.021	1.772	6	5590	ZINC000100772246
8.1	-8.9	-8.7	0.11	2.876	0.209	6.158	2.577	6	5591	ZINC000004212164

8.1	-9.7	-9.4	0.21	2.522	0.603	6.860	2.665	6	5592	ZINC000100823420
8.1	-11.0	-10.3	0.44	2.167	0.295	5.456	1.684	6	5593	ZINC000015148333
8.1	-10.9	-10.8	0.14	2.082	0.197	4.018	1.063	6	5594	ZINC000150363923
8.1	-9.5	-9.2	0.24	2.262	0.646	5.492	2.317	6	5595	ZINC000086041876
8.1	-11.0	-10.8	0.15	2.344	0.683	6.565	2.487	6	5596	ZINC000118937138
8.1	-9.0	-8.9	0.11	2.471	0.160	5.621	1.704	6	5597	ZINC000100772252
8.1	-9.6	-9.2	0.29	2.498	0.262	6.416	1.282	6	5598	ZINC000257439619
8.1	-9.0	-8.9	0.11	2.348	0.098	6.026	1.772	6	5599	ZINC000257374616
8.1	-9.8	-9.1	0.36	1.985	0.462	5.572	2.375	6	5600	ZINC000086039800
8.1	-10.0	-9.8	0.19	2.203	0.356	6.179	1.896	6	5601	ZINC000100825372
8.1	-11.5	-10.7	0.52	1.801	0.138	4.573	1.800	6	5602	ZINC000015148335
8.1	-10.4	-9.8	0.41	2.518	0.740	5.971	2.453	6	5603	ZINC000095619685
8.1	-10.6	-10.1	0.35	2.930	0.887	6.180	2.352	6	5604	ZINC000257419939
8.1	-9.0	-8.9	0.09	2.307	0.154	5.304	1.514	6	5605	ZINC000100772247
8.1	-8.7	-8.5	0.17	2.562	0.464	6.100	2.532	6	5606	ZINC000257432898

8.1	-9.9	-9.6	0.20	2.940	0.394	7.700	2.969	6	5607	ZINC000014813791
8.1	-10.4	-9.8	0.35	2.090	0.489	3.956	2.396	6	5608	ZINC000253589623
8.1	-9.0	-8.9	0.11	2.312	0.155	5.309	1.514	6	5609	ZINC000257374613
8.1	-10.0	-9.8	0.23	2.153	0.306	5.716	1.648	6	5610	ZINC000257455992
8.1	-10.6	-10.1	0.32	2.865	0.830	6.142	2.327	6	5611	ZINC000257419941
8.1	-9.5	-9.1	0.31	2.889	0.877	6.015	1.836	6	5612	ZINC000261498643
8.1	-10.6	-10.1	0.32	2.822	0.755	6.076	2.205	6	5613	ZINC000257419942
8.1	-11.0	-10.7	0.26	2.827	0.409	7.565	1.713	6	5614	ZINC000118937140
8.1	-10.6	-10.1	0.31	2.972	0.837	6.059	2.108	6	5615	ZINC000100776813
8.1	-10.6	-10.1	0.40	2.258	0.401	6.321	2.835	6	5616	ZINC000257487040
8.1	-9.2	-8.8	0.30	2.915	0.223	8.958	1.988	6	5617	ZINC000085487712
8.1	-9.7	-9.4	0.20	1.729	0.681	5.090	2.065	6	5618	ZINC000257523141
8.1	-10.9	-10.4	0.29	2.542	0.414	8.602	0.449	6	5619	ZINC000015150607
8.1	-10.6	-10.0	0.38	3.117	0.583	6.552	1.342	6	5620	ZINC000100776807
8.1	-11.5	-11.0	0.24	2.014	0.207	7.088	0.623	6	5621	ZINC000100773038

8.1	-11.2	-10.9	0.19	1.973	0.397	4.473	2.123	6	5622	ZINC000100784093
8.1	-9.8	-9.4	0.26	1.914	0.191	5.828	1.089	6	5623	ZINC000100825675
8.1	-10.4	-10.0	0.27	1.695	0.513	3.267	1.402	6	5624	ZINC000100775315
8.0	-11.0	-10.6	0.29	2.713	0.777	5.779	0.921	6	5625	ZINC000095619157
8.0	-9.6	-9.2	0.29	2.518	0.449	6.979	2.397	6	5626	ZINC000257476744
8.0	-9.2	-8.9	0.20	2.928	0.528	5.023	1.517	6	5627	ZINC000250744444
8.0	-10.9	-10.3	0.26	2.612	0.229	6.660	1.683	6	5628	ZINC000014441532
8.0	-10.6	-10.1	0.26	2.126	0.626	4.295	2.173	6	5629	ZINC000100772850
8.0	-12.0	-11.3	0.47	2.267	0.811	3.813	1.291	6	5630	ZINC000257555913
8.0	-9.5	-9.2	0.26	2.167	0.899	4.156	2.815	6	5631	ZINC000040164387
8.0	-11.3	-10.9	0.25	2.547	0.320	5.686	0.357	6	5632	ZINC000257461098
8.0	-11.6	-11.0	0.32	2.848	0.531	5.451	0.943	6	5633	ZINC000100779014
8.0	-10.4	-10.1	0.18	2.295	0.198	6.976	0.764	6	5634	ZINC000257415729
8.0	-10.7	-10.4	0.22	2.230	0.680	4.360	2.122	6	5635	ZINC000118937197
8.0	-8.6	-8.3	0.15	2.252	0.928	5.506	1.888	6	5636	ZINC000015148363

8.0	-10.7	-10.5	0.13	2.103	0.601	6.367	2.436	6	5637	ZINC000118937346
8.0	-12.0	-11.4	0.39	2.326	0.348	7.486	0.764	6	5638	ZINC000150369714
8.0	-10.7	-10.0	0.32	2.568	0.233	8.807	1.951	6	5639	ZINC000257710431
8.0	-10.6	-10.1	0.35	2.968	0.816	6.246	2.241	6	5640	ZINC000100776809
8.0	-11.3	-10.8	0.36	1.849	0.336	4.831	2.366	6	5641	ZINC000257565998
8.0	-9.9	-9.7	0.21	2.067	0.493	3.903	1.739	6	5642	ZINC000257476745
8.0	-10.9	-10.6	0.22	2.180	0.485	7.783	2.444	6	5643	ZINC000257379675
8.0	-10.1	-9.8	0.17	2.854	0.819	5.359	2.047	6	5644	ZINC000100822249
8.0	-10.3	-9.2	0.58	3.117	0.438	6.133	1.604	6	5645	ZINC000257375890
8.0	-10.1	-9.7	0.24	3.227	1.110	5.621	2.148	6	5646	ZINC000031290887
8.0	-11.3	-10.8	0.34	1.749	0.231	6.118	1.676	6	5647	ZINC000257413558
8.0	-9.7	-9.2	0.43	2.463	0.460	5.720	0.612	6	5648	ZINC000257439622
8.0	-10.8	-10.4	0.26	2.222	1.025	5.738	1.067	6	5649	ZINC000095619158
8.0	-10.0	-9.4	0.26	1.975	0.318	4.808	2.483	6	5650	ZINC000253590093
8.0	-10.7	-10.3	0.33	1.993	0.437	5.514	1.908	6	5651	ZINC000017653882

8.0	-9.1	-8.9	0.08	2.826	0.265	6.047	2.675	6	5652	ZINC000257441268
8.0	-9.3	-9.1	0.09	2.761	0.817	5.876	2.338	6	5653	ZINC000014930785
8.0	-8.9	-8.8	0.08	1.430	0.294	5.358	4.152	6	5654	ZINC000014807641
8.0	-9.6	-9.4	0.14	3.050	0.167	6.604	0.830	6	5655	ZINC000261495758
8.0	-10.4	-9.6	0.42	3.281	0.327	6.624	1.157	6	5656	ZINC000034294468
8.0	-10.4	-10.0	0.30	1.637	0.498	3.212	1.444	6	5657	ZINC000257437773
8.0	-10.4	-10.0	0.31	1.941	0.787	4.339	2.442	6	5658	ZINC000100775319
8.0	-10.0	-9.8	0.20	2.231	0.128	7.027	1.435	6	5659	ZINC000247978452
8.0	-11.4	-11.2	0.13	2.093	0.488	4.832	2.272	6	5660	ZINC000100825179
8.0	-10.0	-9.8	0.23	2.162	0.314	5.814	1.629	6	5661	ZINC000100825373
8.0	-12.0	-11.7	0.22	1.911	0.287	5.787	1.334	6	5662	ZINC000150366298
8.0	-10.4	-9.9	0.26	2.802	0.477	6.488	1.817	6	5663	ZINC000257410707
8.0	-9.2	-8.9	0.15	2.063	0.740	5.076	2.853	6	5664	ZINC000257457018
8.0	-10.8	-10.3	0.39	2.435	0.249	6.236	1.397	6	5665	ZINC000014887097
8.0	-9.8	-9.2	0.39	2.440	0.313	5.122	2.011	6	5666	ZINC000257464991

7.9	-10.2	-9.9	0.18	2.275	0.587	4.582	2.216	6	5667	ZINC000118937110
7.9	-10.2	-10.0	0.25	2.471	0.373	5.149	1.444	6	5668	ZINC000100826093
7.9	-11.6	-10.9	0.45	2.708	0.490	6.245	1.364	6	5669	ZINC000257383251
7.9	-8.6	-8.5	0.16	2.359	0.349	6.604	2.107	6	5670	ZINC000257432899
7.9	-9.8	-9.6	0.12	2.993	0.280	5.488	1.169	6	5671	ZINC000015210172
7.9	-10.3	-9.5	0.57	3.024	0.971	5.249	1.988	6	5672	ZINC000100772893
7.9	-10.2	-9.9	0.18	2.575	0.423	5.797	2.183	6	5673	ZINC000257428227
7.9	-10.4	-10.1	0.14	2.397	0.388	8.428	0.402	6	5674	ZINC000150363931
7.9	-9.1	-8.6	0.28	2.578	0.559	6.144	3.957	6	5675	ZINC000015156996
7.9	-10.9	-10.4	0.30	2.326	0.393	6.358	2.376	6	5676	ZINC000100828738
7.9	-11.6	-11.1	0.26	2.774	0.495	5.576	0.893	6	5677	ZINC000100779011
7.9	-10.2	-9.5	0.35	2.354	0.235	5.671	1.022	6	5678	ZINC000257596852
7.9	-9.7	-9.5	0.15	1.950	0.695	6.218	3.273	6	5679	ZINC000004097705
7.9	-9.3	-9.1	0.10	2.811	1.024	6.704	3.207	6	5680	ZINC000003861169
7.9	-8.2	-8.0	0.14	2.373	0.742	6.781	3.042	6	5681	ZINC000014708538

7.9	-9.8	-9.5	0.14	2.226	0.837	5.688	3.102	6	5682	ZINC000014931142
7.9	-11.4	-10.7	0.44	2.057	0.087	5.741	1.356	6	5683	ZINC000014617316
7.9	-9.3	-8.5	0.49	2.366	0.758	5.619	2.290	6	5684	ZINC000255974368
7.9	-10.7	-10.5	0.16	2.108	0.567	6.332	2.434	6	5685	ZINC000118937352
7.9	-10.7	-10.5	0.13	2.137	0.578	5.350	2.582	6	5686	ZINC000118937345
7.9	-11.6	-11.0	0.34	2.698	0.507	6.179	1.448	6	5687	ZINC000100779010
7.9	-11.6	-11.0	0.28	2.753	0.494	5.558	0.899	6	5688	ZINC000169406130
7.9	-9.2	-9.0	0.10	2.519	1.793	6.701	1.929	6	5689	ZINC000014765869
7.9	-11.1	-10.8	0.20	2.145	0.499	5.570	1.702	6	5690	ZINC000257491952
7.9	-11.0	-10.9	0.07	2.212	0.312	5.834	2.590	6	5691	ZINC000100829053
7.9	-10.2	-9.9	0.17	2.442	0.625	5.568	2.418	6	5692	ZINC000118937111
7.9	-10.2	-9.5	0.46	2.426	0.302	6.783	0.676	6	5693	ZINC000257383047
7.9	-10.2	-9.9	0.18	2.403	0.666	5.492	2.480	6	5694	ZINC000118937112
7.9	-11.6	-10.9	0.41	2.563	0.442	5.829	1.743	6	5695	ZINC000257383250
7.9	-10.0	-9.8	0.14	2.243	0.525	5.322	2.649	6	5696	ZINC000015063091

7.9	-9.9	-9.8	0.10	2.050	0.430	5.388	0.916	6	5697	ZINC000095620397
7.9	-11.6	-11.1	0.26	2.761	0.506	5.574	0.895	6	5698	ZINC000257383249
7.9	-9.4	-9.1	0.21	2.193	0.476	4.207	2.316	6	5699	ZINC000014887449
7.9	-9.6	-9.2	0.24	2.683	0.936	5.806	3.139	6	5700	ZINC000257377503
7.9	-10.9	-10.2	0.41	2.923	0.668	5.574	2.676	6	5701	ZINC000257491281
7.9	-10.7	-10.4	0.20	2.320	0.711	4.279	2.166	6	5702	ZINC000118937199
7.9	-10.1	-9.6	0.25	3.241	1.277	5.667	2.229	6	5703	ZINC000031290884
7.9	-10.6	-9.8	0.55	2.113	0.540	4.856	1.968	6	5704	ZINC000015210032
7.9	-9.8	-9.1	0.41	2.428	0.822	5.284	2.885	6	5705	ZINC000253497580
7.9	-11.1	-10.3	0.38	2.539	0.660	6.245	1.676	6	5706	ZINC000257549774
7.9	-10.7	-10.5	0.14	2.324	0.314	6.402	2.397	6	5707	ZINC000118937347
7.9	-9.6	-9.3	0.18	2.094	0.177	9.387	0.378	6	5708	ZINC000257538547
7.9	-10.2	-9.9	0.18	2.435	0.618	5.548	2.425	6	5709	ZINC000118937113
7.9	-10.7	-10.5	0.12	2.142	0.587	5.364	2.550	6	5710	ZINC000118937351
7.9	-11.7	-10.6	0.55	2.311	0.236	5.836	0.647	6	5711	ZINC000100826919

7.9	-10.2	-9.5	0.34	2.637	0.525	4.907	2.524	6	5712	ZINC000096095401
7.9	-10.7	-10.4	0.16	2.110	0.524	6.386	2.405	6	5713	ZINC000118937350
7.9	-9.9	-9.4	0.25	2.751	0.387	6.673	2.241	6	5714	ZINC000100824517
7.9	-10.2	-9.8	0.17	2.731	0.487	5.893	2.161	6	5715	ZINC000257428226
7.8	-10.2	-9.9	0.18	2.388	0.653	5.514	2.457	6	5716	ZINC000257428225
7.8	-9.7	-9.5	0.15	2.329	0.617	6.739	1.653	6	5717	ZINC000100827885
7.8	-11.9	-11.0	0.52	1.790	0.499	4.687	2.369	6	5718	ZINC000257436633
7.8	-9.3	-9.0	0.15	1.904	0.314	6.741	1.449	6	5719	ZINC000257554975
7.8	-10.6	-10.3	0.23	2.739	0.885	6.712	2.478	6	5720	ZINC000118937198
7.8	-10.0	-9.4	0.38	2.731	0.341	5.272	0.662	6	5721	ZINC000257608681
7.8	-10.0	-9.7	0.21	2.383	0.491	7.859	2.953	6	5722	ZINC000100774216
7.8	-10.2	-10.0	0.21	2.792	0.289	6.277	0.603	6	5723	ZINC000257488523
7.8	-10.5	-10.2	0.18	1.993	0.644	6.410	3.238	6	5724	ZINC000257553794
7.8	-9.8	-9.3	0.30	2.612	0.721	5.883	3.428	6	5725	ZINC000100825544
7.8	-10.9	-10.2	0.36	1.959	0.500	4.656	2.203	6	5726	ZINC000100780300

7.8	-9.5	-9.1	0.18	2.395	0.701	6.304	2.874	6	5727	ZINC000015150599
7.8	-10.6	-10.4	0.13	2.264	0.530	5.505	2.450	6	5728	ZINC000118937349
7.8	-10.9	-10.7	0.17	2.375	0.493	6.006	1.968	6	5729	ZINC000100824471
7.8	-9.8	-9.5	0.16	2.641	0.540	5.424	2.226	6	5730	ZINC000257423422
7.8	-10.6	-10.3	0.15	3.061	0.449	6.526	2.010	6	5731	ZINC000118937204
7.8	-10.5	-10.1	0.23	2.230	0.206	5.311	1.109	6	5732	ZINC000150368138
7.8	-9.6	-9.3	0.18	2.923	0.428	6.620	3.128	6	5733	ZINC000008662732
7.8	-12.1	-11.4	0.32	1.999	0.589	4.687	2.133	6	5734	ZINC000024215108
7.8	-9.8	-9.5	0.17	2.146	0.304	6.387	1.786	6	5735	ZINC000100777718
7.8	-10.8	-10.3	0.27	2.477	0.409	6.459	1.386	6	5736	ZINC000257549772
7.8	-10.7	-9.7	0.50	3.136	0.327	6.510	0.584	6	5737	ZINC000015219772
7.8	-9.8	-9.0	0.39	2.448	0.515	5.176	1.845	6	5738	ZINC000004096710
7.8	-11.1	-10.4	0.43	2.761	0.434	5.378	1.422	6	5739	ZINC000257379676
7.8	-10.3	-9.2	0.52	2.039	0.348	4.695	1.590	6	5740	ZINC000257439621
7.8	-9.9	-9.7	0.21	1.439	0.561	3.595	1.847	6	5741	ZINC000100828067

7.8	-10.1	-9.9	0.17	2.536	0.511	4.604	2.388	6	5742	ZINC000095620830
7.8	-11.0	-10.6	0.24	2.680	0.303	6.311	0.279	6	5743	ZINC000014858809
7.8	-9.2	-8.9	0.18	2.419	0.868	7.177	3.448	6	5744	ZINC000014811760
7.8	-9.9	-9.6	0.23	2.257	0.343	4.669	1.942	6	5745	ZINC000257468172
7.8	-10.1	-9.8	0.16	2.614	0.619	9.149	0.356	6	5746	ZINC000257461514
7.8	-10.4	-9.5	0.61	1.978	1.043	3.371	1.114	6	5747	ZINC000006498737
7.8	-10.6	-10.2	0.20	2.686	0.494	4.937	1.883	6	5748	ZINC000257382541
7.8	-9.9	-9.6	0.27	1.906	0.464	4.706	1.852	6	5749	ZINC000257495604
7.8	-8.6	-8.3	0.20	1.178	0.714	3.944	2.189	6	5750	ZINC000257493910
7.7	-8.8	-8.6	0.14	2.354	0.461	5.737	2.919	6	5751	ZINC000257450390
7.7	-10.3	-10.0	0.20	2.558	0.781	5.307	2.609	6	5752	ZINC000118937013
7.7	-10.1	-9.9	0.13	2.106	0.271	4.696	1.451	6	5753	ZINC000257390533
7.7	-10.9	-10.2	0.31	1.841	0.479	3.930	2.109	6	5754	ZINC000100829393
7.7	-10.1	-9.9	0.11	2.100	0.260	4.726	1.464	6	5755	ZINC000257390532
7.7	-9.4	-8.7	0.38	3.901	0.680	6.841	2.173	6	5756	ZINC000014610081

7.7	-10.5	-9.7	0.44	2.577	0.665	5.994	1.476	6	5757	ZINC000257771088
7.7	-10.3	-9.9	0.24	2.461	0.908	5.668	2.607	6	5758	ZINC000118937012
7.7	-10.1	-9.9	0.14	2.103	0.256	4.714	1.478	6	5759	ZINC000100824073
7.7	-9.9	-9.5	0.24	2.583	0.249	7.678	2.133	6	5760	ZINC000257479182
7.7	-9.0	-8.6	0.21	1.825	0.580	2.951	1.059	6	5761	ZINC000014645287
7.7	-9.6	-9.2	0.20	2.656	0.792	4.716	2.588	6	5762	ZINC000014931146
7.7	-10.1	-9.9	0.13	2.097	0.266	4.719	1.463	6	5763	ZINC000100824075
7.7	-13.6	-12.9	0.36	3.176	0.917	6.540	1.188	6	5764	ZINC000257409037
7.7	-10.6	-10.1	0.25	2.779	0.571	8.207	1.861	6	5765	ZINC000257439872
7.7	-10.3	-9.9	0.25	2.044	0.755	5.420	2.672	6	5766	ZINC000257411331
7.7	-10.2	-10.1	0.11	1.935	0.712	5.027	2.992	6	5767	ZINC000257504570
7.7	-10.1	-10.0	0.11	1.947	0.522	4.568	1.690	6	5768	ZINC000257390530
7.7	-8.3	-8.2	0.07	1.823	1.480	6.386	3.300	6	5769	ZINC000005665087
7.7	-9.9	-9.8	0.08	2.561	0.428	8.365	0.368	6	5770	ZINC000119379746
7.7	-10.9	-9.9	0.75	2.780	0.791	6.050	1.931	6	5771	ZINC000072320099

7.7	-10.4	-10.1	0.15	2.162	0.311	5.342	2.425	6	5772	ZINC000257444943
7.7	-10.1	-9.9	0.16	2.143	0.233	5.389	1.275	6	5773	ZINC000100824077
7.7	-9.9	-9.8	0.11	2.321	0.451	8.086	0.479	6	5774	ZINC000119380029
7.7	-10.2	-10.0	0.17	1.991	0.279	5.932	1.670	6	5775	ZINC000257447360
7.7	-9.8	-9.4	0.25	3.021	0.888	6.946	3.641	6	5776	ZINC000118913618
7.7	-10.0	-9.5	0.23	2.458	0.324	5.418	1.009	6	5777	ZINC000015210134
7.7	-10.6	-10.0	0.38	2.094	0.530	5.128	2.657	6	5778	ZINC000100825723
7.7	-10.3	-9.8	0.25	2.824	0.436	6.810	2.297	6	5779	ZINC000118937014
7.7	-11.4	-10.5	0.52	2.561	0.420	6.350	1.211	6	5780	ZINC000100778997
7.7	-10.3	-9.9	0.22	2.127	0.712	4.258	2.241	6	5781	ZINC000257411329
7.7	-8.7	-8.5	0.21	2.199	0.878	5.270	2.581	6	5782	ZINC000014658084
7.7	-10.8	-10.7	0.13	2.435	0.357	5.833	1.752	6	5783	ZINC000100825710
7.7	-10.3	-9.9	0.25	2.030	0.747	4.330	2.190	6	5784	ZINC000118937015
7.7	-9.6	-9.0	0.38	1.986	0.505	3.937	1.342	6	5785	ZINC000257486517
7.7	-9.7	-9.0	0.37	2.512	0.812	6.032	2.195	6	5786	ZINC000015257574

7.7	-10.2	-10.0	0.19	2.866	0.214	6.128	0.813	6	5787	ZINC000100822596
7.7	-9.7	-9.2	0.26	2.649	0.564	6.289	1.081	6	5788	ZINC000257791897
7.7	-10.8	-10.4	0.26	2.945	0.786	6.328	0.792	6	5789	ZINC000008214766
7.7	-9.3	-8.9	0.26	2.073	0.942	4.032	2.298	6	5790	ZINC000257561351
7.7	-10.6	-10.3	0.20	2.130	0.700	3.175	1.090	6	5791	ZINC000100774255
7.7	-10.3	-9.9	0.25	2.609	0.875	5.848	2.422	6	5792	ZINC000257411330
7.7	-9.5	-9.0	0.23	2.631	0.656	5.176	1.882	6	5793	ZINC000013547732
7.7	-10.0	-9.6	0.29	2.500	1.000	5.423	3.173	6	5794	ZINC000257392246
7.7	-10.5	-10.2	0.19	3.003	0.413	6.451	1.958	6	5795	ZINC000118937205
7.7	-10.6	-10.2	0.22	1.834	0.535	4.670	2.117	6	5796	ZINC000013374448
7.7	-10.5	-10.3	0.15	3.060	0.436	6.526	2.002	6	5797	ZINC000118937206
7.7	-10.5	-10.3	0.14	2.302	0.648	5.619	2.205	6	5798	ZINC000118937348
7.7	-10.3	-9.9	0.24	2.078	0.662	3.553	0.786	6	5799	ZINC000257411332
7.7	-9.8	-9.3	0.21	2.628	0.608	4.136	0.859	6	5800	ZINC000013382496
7.7	-9.2	-8.9	0.18	3.295	0.628	7.392	1.928	6	5801	ZINC000014586261

7.7	-9.7	-9.1	0.31	2.864	0.321	6.928	1.550	6	5802	ZINC000257498126
7.7	-9.8	-9.5	0.18	2.086	0.511	3.554	1.055	6	5803	ZINC000257547947
7.7	-9.0	-8.7	0.23	1.973	0.473	5.516	3.277	6	5804	ZINC000100773764
7.7	-10.9	-10.4	0.22	2.305	0.504	6.284	3.387	6	5805	ZINC000100825172
7.7	-9.2	-9.0	0.16	3.004	0.079	7.647	1.311	6	5806	ZINC000014650503
7.7	-10.1	-9.5	0.31	1.681	0.573	7.139	3.847	6	5807	ZINC000035454640
7.7	-11.5	-10.6	0.52	2.717	0.517	6.273	1.281	6	5808	ZINC000257393662
7.7	-9.9	-9.7	0.11	2.624	0.331	5.792	2.128	6	5809	ZINC000257428224
7.7	-9.9	-9.8	0.10	2.294	0.441	8.097	0.463	6	5810	ZINC000119380146
7.7	-11.5	-11.2	0.24	2.223	0.521	8.664	0.726	6	5811	ZINC000150368146
7.7	-12.0	-11.3	0.58	2.469	0.185	5.787	0.803	6	5812	ZINC000096903804
7.7	-10.5	-10.3	0.12	3.148	0.427	7.193	1.837	6	5813	ZINC000118937207
7.7	-9.9	-9.3	0.33	1.997	0.342	5.947	1.707	6	5814	ZINC000257458220
7.7	-10.4	-10.3	0.07	3.153	0.078	5.291	1.551	6	5815	ZINC000257386779
7.7	-13.6	-12.9	0.36	2.933	1.227	6.480	2.392	6	5816	ZINC000257409038

7.7	-9.6	-9.3	0.21	1.928	0.716	5.211	3.232	6	5817	ZINC000014645276
7.6	-10.2	-9.9	0.26	2.936	0.513	6.454	2.141	6	5818	ZINC000005920547
7.6	-12.0	-11.4	0.58	2.155	0.951	4.742	2.773	6	5819	ZINC000257437256
7.6	-9.9	-9.6	0.25	1.760	0.463	5.184	3.356	6	5820	ZINC000257517538
7.6	-10.4	-10.1	0.25	2.578	0.309	5.766	2.299	6	5821	ZINC000118937213
7.6	-10.9	-10.4	0.27	2.584	1.200	5.902	3.623	6	5822	ZINC000257805856
7.6	-12.0	-11.4	0.56	2.529	0.781	5.526	2.189	6	5823	ZINC000100823559
7.6	-9.3	-9.0	0.15	2.584	0.867	4.599	2.775	6	5824	ZINC000257377501
7.6	-11.4	-10.5	0.51	2.269	0.783	4.987	2.363	6	5825	ZINC000100778994
7.6	-11.4	-10.5	0.50	2.539	0.433	5.469	0.893	6	5826	ZINC000257393661
7.6	-12.0	-11.4	0.56	2.529	0.778	5.530	2.188	6	5827	ZINC000100823554
7.6	-10.4	-10.1	0.21	2.659	0.162	6.638	2.028	6	5828	ZINC000118937215
7.6	-13.5	-13.0	0.29	2.290	0.654	5.446	1.790	6	5829	ZINC000247933187
7.6	-7.5	-7.4	0.07	3.666	1.215	8.158	1.611	6	5830	ZINC000095617916
7.6	-10.2	-9.7	0.27	2.354	0.388	7.489	2.043	6	5831	ZINC000257476675

7.6	-10.2	-9.7	0.35	2.466	0.532	7.170	1.049	6	5832	ZINC000150368584
7.6	-10.1	-9.8	0.23	2.178	0.622	5.089	2.773	6	5833	ZINC000257410709
7.6	-9.4	-9.2	0.15	3.269	0.476	7.429	1.746	6	5834	ZINC000004095717
7.6	-9.3	-9.0	0.19	2.291	0.719	5.694	2.674	6	5835	ZINC000085863138
7.6	-10.0	-9.7	0.14	3.305	1.058	6.615	2.168	6	5836	ZINC000013397733
7.6	-10.2	-9.9	0.22	2.931	0.513	6.470	2.187	6	5837	ZINC000118937273
7.6	-8.0	-7.6	0.23	3.646	1.036	7.107	2.431	6	5838	ZINC000001529772
7.6	-10.0	-9.6	0.26	2.748	0.630	5.238	1.480	6	5839	ZINC000257470251
7.6	-10.2	-9.8	0.21	2.597	0.598	7.257	1.989	6	5840	ZINC000100777278
7.6	-10.2	-9.7	0.22	2.424	0.417	6.554	2.332	6	5841	ZINC000257476673
7.6	-9.2	-9.0	0.14	2.523	0.366	6.281	2.640	6	5842	ZINC000257604600
7.6	-10.2	-9.9	0.22	2.616	0.500	5.433	2.238	6	5843	ZINC000118937275
7.6	-10.2	-9.6	0.39	2.518	0.633	4.837	2.469	6	5844	ZINC000257381186
7.6	-10.2	-9.9	0.20	2.935	0.514	6.481	2.185	6	5845	ZINC000118937274
7.6	-10.0	-9.7	0.26	1.771	1.077	3.137	2.441	6	5846	ZINC000257460907

7.6	-9.9	-9.6	0.17	2.142	0.338	5.876	2.409	6	5847	ZINC000257392059
7.6	-11.4	-10.5	0.51	2.500	0.441	6.183	1.391	6	5848	ZINC000100778996
7.6	-12.0	-11.4	0.56	2.530	0.779	5.520	2.187	6	5849	ZINC000100823555
7.6	-13.5	-13.0	0.29	2.299	0.656	5.463	1.776	6	5850	ZINC000247933192
7.6	-10.2	-9.6	0.41	2.571	0.841	5.657	2.939	6	5851	ZINC000100824510
7.6	-10.0	-9.7	0.19	2.760	0.811	6.676	3.153	6	5852	ZINC000257472415
7.6	-10.2	-9.7	0.23	2.361	0.405	7.495	2.087	6	5853	ZINC000100777280
7.6	-9.5	-9.2	0.16	1.987	0.392	5.456	3.627	6	5854	ZINC000100827841
7.6	-10.1	-9.9	0.16	1.931	0.515	4.676	1.799	6	5855	ZINC000257390531
7.6	-8.7	-8.4	0.20	2.407	0.819	7.522	2.993	6	5856	ZINC000013371191
7.6	-10.2	-9.6	0.27	2.091	0.255	4.345	2.095	6	5857	ZINC000253589629
7.6	-9.3	-9.0	0.27	2.534	0.726	7.388	3.314	6	5858	ZINC000030730350
7.6	-11.3	-10.7	0.41	1.936	0.325	5.805	2.676	6	5859	ZINC000206658454
7.6	-10.0	-9.7	0.34	1.934	0.345	6.869	2.971	6	5860	ZINC000257373527
7.6	-9.9	-9.5	0.24	2.372	0.354	6.710	3.374	6	5861	ZINC000014436816

7.6	-11.9	-10.7	0.61	2.217	0.470	6.377	1.834	6	5862	ZINC000044305779
7.6	-10.4	-9.6	0.45	2.059	0.281	6.245	2.353	6	5863	ZINC000100825560
7.6	-10.7	-10.2	0.28	1.881	0.387	4.837	3.251	6	5864	ZINC000257461823
7.6	-10.1	-9.9	0.21	2.159	0.238	5.050	1.375	6	5865	ZINC000100824072
7.6	-10.9	-10.7	0.19	2.216	0.152	5.915	1.687	6	5866	ZINC000014887090
7.6	-10.2	-9.6	0.30	2.236	0.203	4.502	0.496	6	5867	ZINC000100827471
7.6	-12.0	-11.4	0.56	2.530	0.782	5.529	2.190	6	5868	ZINC000257437255
7.6	-12.0	-11.3	0.64	2.266	0.839	4.843	2.654	6	5869	ZINC000100823557
7.6	-9.5	-9.0	0.28	2.519	0.447	5.690	2.416	6	5870	ZINC000255974366
7.6	-10.4	-10.0	0.27	2.543	0.264	4.771	1.919	6	5871	ZINC000118937212
7.6	-10.4	-9.8	0.35	2.354	0.713	4.344	1.910	6	5872	ZINC000014688693
7.6	-11.3	-11.0	0.20	2.079	0.440	5.135	1.159	6	5873	ZINC000257446298
7.6	-11.0	-10.7	0.22	2.055	0.557	6.040	1.985	6	5874	ZINC000257512054
7.6	-9.4	-9.3	0.07	2.416	0.563	7.614	2.793	6	5875	ZINC000014813771
7.6	-9.4	-9.3	0.07	2.940	0.659	5.625	0.789	6	5876	ZINC000031460590

7.6	-10.2	-9.8	0.24	2.437	0.956	5.962	3.124	6	5877	ZINC000257805859
7.6	-10.5	-10.1	0.31	3.707	0.285	6.801	0.904	6	5878	ZINC000057509131
7.6	-7.5	-7.4	0.08	2.632	1.150	6.441	1.896	6	5879	ZINC000085980896
7.6	-11.4	-10.5	0.51	2.511	0.439	6.197	1.384	6	5880	ZINC000257393660
7.6	-10.3	-10.1	0.21	2.517	0.731	5.681	2.440	6	5881	ZINC000100827591
7.5	-9.8	-9.1	0.34	2.339	0.677	5.430	3.141	6	5882	ZINC000059762514
7.5	-9.8	-9.2	0.37	3.024	1.274	6.888	3.821	6	5883	ZINC000118913619
7.5	-9.7	-9.2	0.29	2.213	0.426	4.616	1.783	6	5884	ZINC000052650193
7.5	-10.7	-9.1	0.95	3.234	1.055	6.892	0.704	6	5885	ZINC000100288506
7.5	-10.5	-10.0	0.31	1.599	0.211	3.287	0.797	6	5886	ZINC000257419089
7.5	-10.9	-10.7	0.21	1.865	0.334	6.452	1.291	6	5887	ZINC000100778302
7.5	-10.1	-9.8	0.26	2.908	0.406	6.366	2.013	6	5888	ZINC000118937272
7.5	-9.3	-9.0	0.19	2.324	0.891	4.372	3.255	6	5889	ZINC000014811589
7.5	-9.8	-9.3	0.26	2.495	0.585	5.626	2.715	6	5890	ZINC000014759027
7.5	-8.7	-8.5	0.13	2.133	1.229	5.285	3.103	6	5891	ZINC000014610079

7.5	-10.4	-9.9	0.27	1.724	0.752	3.890	2.675	6	5892	ZINC000257525015
7.5	-9.3	-9.2	0.08	1.843	0.189	4.194	2.843	6	5893	ZINC000035466077
7.5	-9.0	-8.8	0.13	2.824	0.341	6.520	2.170	6	5894	ZINC000002563650
7.5	-11.2	-10.7	0.34	1.777	0.452	4.753	2.306	6	5895	ZINC000150602724
7.5	-9.6	-9.0	0.30	3.335	0.981	5.936	2.093	6	5896	ZINC000014502667
7.5	-10.2	-9.6	0.32	2.348	0.318	6.755	1.628	6	5897	ZINC000257397147
7.5	-10.8	-10.2	0.26	2.124	0.169	5.412	1.559	6	5898	ZINC000100779096
7.5	-8.9	-8.7	0.15	2.441	0.559	5.322	2.295	6	5899	ZINC000005167145
7.5	-10.2	-9.7	0.26	2.439	0.317	8.553	0.430	6	5900	ZINC000257476674
7.5	-9.9	-9.6	0.22	2.649	0.259	6.663	1.489	6	5901	ZINC000014811797
7.5	-10.2	-9.9	0.20	1.824	0.525	3.391	1.988	6	5902	ZINC000095619584
7.5	-8.8	-8.5	0.27	2.268	0.494	8.001	1.587	6	5903	ZINC000014727570
7.5	-10.2	-9.7	0.23	2.711	0.495	8.343	0.551	6	5904	ZINC000100777281
7.5	-10.2	-9.7	0.21	2.829	0.451	8.188	0.514	6	5905	ZINC000100777277
7.5	-10.2	-9.6	0.31	1.891	0.570	3.977	1.223	6	5906	ZINC000014435167

7.5	-9.4	-9.4	0.05	2.604	0.424	7.240	2.804	6	5907	ZINC000059211656
7.5	-10.2	-9.6	0.29	1.997	0.541	3.857	1.151	6	5908	ZINC000014435163
7.5	-10.4	-10.2	0.18	1.955	0.276	3.770	0.830	6	5909	ZINC000257767193
7.5	-9.8	-9.3	0.33	2.699	0.324	6.059	0.197	6	5910	ZINC000257602913
7.5	-9.8	-9.5	0.22	2.037	0.286	3.737	0.974	6	5911	ZINC000257791898
7.5	-9.8	-9.2	0.62	2.230	0.456	7.141	3.331	6	5912	ZINC000100823237
7.5	-10.9	-10.3	0.32	2.227	0.623	4.247	1.877	6	5913	ZINC000118937200
7.5	-10.1	-9.9	0.13	2.292	0.754	4.674	1.878	6	5914	ZINC000257546503
7.5	-10.6	-10.0	0.33	2.058	0.636	6.372	2.523	6	5915	ZINC000257444394
7.5	-9.6	-9.5	0.06	2.165	0.454	4.931	2.299	6	5916	ZINC000257430036
7.5	-10.3	-10.1	0.20	2.683	0.206	6.775	1.856	6	5917	ZINC000118937214
7.5	-9.9	-9.2	0.36	2.204	0.511	6.125	2.222	6	5918	ZINC000257383377
7.5	-10.8	-9.7	0.55	2.295	0.599	5.431	1.618	6	5919	ZINC000257612466
7.4	-9.9	-9.7	0.15	2.623	0.956	4.718	2.504	6	5920	ZINC000238744080
7.4	-10.4	-9.9	0.27	2.544	0.528	4.557	2.122	6	5921	ZINC000257373529

7.4	-10.5	-10.1	0.22	2.451	0.255	5.706	0.511	6	5922	ZINC000137664569
7.4	-11.3	-10.9	0.26	2.491	0.236	6.986	0.898	6	5923	ZINC000070455322
7.4	-9.9	-9.7	0.15	2.933	1.044	5.186	2.368	6	5924	ZINC000014642822
7.4	-10.4	-9.6	0.39	1.870	0.348	5.438	2.941	6	5925	ZINC000257559677
7.4	-9.9	-9.7	0.12	2.647	0.972	3.636	0.915	6	5926	ZINC000014642816
7.4	-9.7	-9.2	0.28	2.293	0.349	5.818	1.873	6	5927	ZINC000257490152
7.4	-9.9	-9.5	0.30	2.074	0.266	6.658	2.162	6	5928	ZINC000100826522
7.4	-10.1	-9.7	0.27	2.251	0.508	5.415	2.026	6	5929	ZINC000085883677
7.4	-8.3	-8.0	0.24	3.064	0.668	7.749	2.565	6	5930	ZINC000022061296
7.4	-10.8	-10.6	0.09	2.067	0.627	5.976	0.565	6	5931	ZINC000014686358
7.4	-8.6	-8.3	0.18	1.385	0.674	5.249	2.704	6	5932	ZINC000100828387
7.4	-10.5	-10.0	0.26	2.567	0.388	4.990	1.870	6	5933	ZINC000257458887
7.4	-9.4	-9.2	0.13	2.943	0.395	6.374	3.065	6	5934	ZINC000014760546
7.4	-10.1	-9.7	0.28	2.752	0.211	7.102	1.468	6	5935	ZINC000257512055
7.4	-10.0	-9.7	0.20	1.673	0.583	4.164	2.040	6	5936	ZINC000257559420

7.4	-8.6	-8.3	0.20	1.307	0.665	3.022	0.713	6	5937	ZINC000257493913
7.4	-8.6	-8.4	0.12	1.304	0.670	2.998	0.703	6	5938	ZINC000100828384
7.4	-9.7	-9.3	0.27	1.983	0.701	5.386	3.136	6	5939	ZINC000014647291
7.4	-9.5	-9.2	0.18	3.054	0.549	7.492	2.044	6	5940	ZINC000257771089
7.4	-10.0	-9.8	0.11	1.999	0.473	4.504	1.993	6	5941	ZINC000257546505
7.4	-11.0	-10.4	0.45	1.998	0.538	4.879	2.159	6	5942	ZINC000100773819
7.4	-9.8	-9.4	0.28	2.277	0.248	5.162	2.328	6	5943	ZINC000257388911
7.4	-10.3	-9.9	0.24	2.481	0.446	6.888	2.206	6	5944	ZINC000013423819
7.4	-11.4	-10.7	0.40	1.944	0.280	6.784	1.258	6	5945	ZINC000059763171
7.4	-9.9	-9.6	0.18	3.067	0.730	5.082	2.291	6	5946	ZINC000257808142
7.4	-9.9	-9.7	0.16	2.473	0.414	5.664	2.460	6	5947	ZINC000118937364
7.4	-10.8	-10.5	0.20	2.195	0.355	3.982	1.681	6	5948	ZINC000257386781
7.4	-8.6	-8.3	0.16	1.349	0.669	3.937	2.470	6	5949	ZINC000100828386
7.4	-10.3	-9.9	0.29	2.953	0.626	6.934	2.288	6	5950	ZINC000257419523
7.4	-9.2	-9.1	0.10	2.387	0.568	5.289	2.335	6	5951	ZINC000257565709

7.4	-10.5	-10.1	0.26	2.599	0.507	7.445	2.087	6	5952	ZINC000100825230
7.4	-9.9	-9.7	0.10	2.723	0.976	3.777	1.007	6	5953	ZINC000257808143
7.4	-9.9	-9.6	0.20	2.680	0.288	6.026	2.162	6	5954	ZINC000118937366
7.4	-9.5	-8.7	0.49	2.298	0.689	6.620	2.271	6	5955	ZINC000058593464
7.4	-10.1	-10.0	0.09	2.920	0.368	8.775	0.444	6	5956	ZINC000257476672
7.4	-9.9	-9.7	0.15	2.908	1.082	5.153	2.409	6	5957	ZINC000014642819
7.4	-11.0	-10.1	0.40	2.172	0.478	6.022	1.876	6	5958	ZINC000257668338
7.4	-7.8	-7.6	0.13	3.670	0.432	6.728	1.827	6	5959	ZINC000034467810
7.4	-8.6	-8.3	0.21	1.319	0.693	2.878	0.555	6	5960	ZINC000257493911
7.4	-9.9	-9.7	0.15	2.634	0.943	3.623	0.883	6	5961	ZINC000257808141
7.4	-9.6	-9.0	0.40	1.967	0.597	3.438	0.920	6	5962	ZINC000014611793
7.4	-10.8	-9.9	0.41	2.593	1.172	5.330	2.076	6	5963	ZINC000014859658
7.4	-10.1	-9.5	0.26	1.973	0.569	3.856	1.186	6	5964	ZINC000100827469
7.4	-9.0	-8.9	0.06	2.721	0.905	4.688	1.978	6	5965	ZINC000015262020
7.4	-11.2	-10.6	0.37	1.814	0.398	4.175	2.150	6	5966	ZINC000100779110

7.4	-8.6	-8.3	0.22	1.291	0.640	3.006	0.710	6	5967	ZINC000100828389
7.4	-8.8	-8.5	0.28	2.868	0.146	5.486	0.569	6	5968	ZINC000255974369
7.4	-9.9	-9.6	0.19	2.650	0.309	5.936	2.209	6	5969	ZINC000118937367
7.3	-9.9	-9.7	0.17	2.659	0.313	5.986	2.184	6	5970	ZINC000118937365
7.3	-9.2	-9.0	0.12	2.536	0.491	6.239	2.326	6	5971	ZINC000257485775
7.3	-11.9	-11.7	0.18	1.870	0.095	5.979	1.735	6	5972	ZINC000100829274
7.3	-9.4	-9.2	0.18	3.196	1.215	4.570	1.982	6	5973	ZINC000014708289
7.3	-10.2	-9.8	0.23	1.895	0.393	5.557	3.216	6	5974	ZINC000257517536
7.3	-10.8	-10.4	0.26	2.335	0.367	6.796	1.092	6	5975	ZINC000100825232
7.3	-9.7	-9.3	0.18	2.254	0.777	5.254	2.758	6	5976	ZINC000257416818
7.3	-9.7	-9.5	0.14	2.964	0.798	5.250	2.448	6	5977	ZINC000238742410
7.3	-10.7	-10.5	0.15	2.337	0.216	5.078	1.319	6	5978	ZINC000257623037
7.3	-10.1	-9.2	0.47	2.104	0.182	5.851	1.733	6	5979	ZINC000100826525
7.3	-8.6	-8.3	0.16	1.352	0.679	3.969	2.453	6	5980	ZINC000257493912
7.3	-11.4	-10.9	0.31	2.397	0.367	4.218	0.943	6	5981	ZINC000040165254

7.3	-10.6	-10.2	0.26	2.066	0.372	5.204	2.767	6	5982	ZINC000257406860
7.3	-9.8	-9.6	0.13	2.756	0.573	5.040	2.298	6	5983	ZINC000014642813
7.3	-10.1	-10.0	0.11	2.037	0.723	3.926	2.561	6	5984	ZINC000085612047
7.3	-10.2	-9.7	0.39	2.049	0.325	5.375	2.589	6	5985	ZINC000257397148
7.3	-9.7	-9.3	0.29	2.769	1.082	5.538	3.035	6	5986	ZINC000085598621
7.3	-9.9	-9.1	0.46	1.998	0.453	4.045	2.428	6	5987	ZINC000257487948
7.3	-11.5	-10.7	0.38	2.425	0.873	5.295	2.499	6	5988	ZINC000150368150
7.3	-9.9	-9.8	0.11	1.955	0.530	4.592	1.856	6	5989	ZINC000257546504
7.3	-9.6	-9.3	0.32	2.447	0.881	5.193	2.337	6	5990	ZINC000030729885
7.3	-9.3	-8.9	0.31	3.549	1.245	7.220	2.351	6	5991	ZINC000015261720
7.3	-11.1	-10.5	0.53	2.281	0.418	4.233	1.630	6	5992	ZINC000257596857
7.3	-9.1	-8.7	0.19	2.299	0.656	7.334	3.509	6	5993	ZINC000013132548
7.3	-9.4	-9.1	0.23	2.723	0.713	5.887	1.805	6	5994	ZINC000100823828
7.3	-10.9	-10.5	0.32	1.615	0.277	4.606	3.182	6	5995	ZINC000118937407
7.3	-11.0	-10.5	0.33	1.914	0.281	6.700	2.765	6	5996	ZINC000257464498

7.3	-10.6	-10.3	0.23	2.620	0.697	7.311	2.284	6	5997	ZINC000257489245
7.3	-9.7	-9.5	0.14	2.871	0.341	5.825	2.791	6	5998	ZINC000059762520
7.3	-11.3	-11.1	0.12	2.245	0.080	5.902	1.931	6	5999	ZINC000003984043
7.3	-9.9	-9.1	0.39	2.167	0.263	6.922	2.127	6	6000	ZINC000257430039
7.3	-10.8	-10.5	0.19	1.600	0.231	4.866	1.350	6	6001	ZINC000014636463
7.3	-10.4	-10.0	0.45	2.101	0.249	6.752	0.975	6	6002	ZINC000257429807
7.3	-9.5	-9.3	0.13	2.312	0.818	7.139	3.981	6	6003	ZINC000014811799
7.3	-11.5	-11.1	0.37	2.379	0.298	6.972	0.365	6	6004	ZINC000056874788
7.3	-9.7	-9.5	0.13	2.290	0.599	5.477	3.597	6	6005	ZINC000014811803
7.3	-9.9	-9.7	0.11	2.589	0.268	8.809	0.371	6	6006	ZINC000014488609
7.3	-8.9	-8.7	0.15	2.192	0.289	6.221	2.632	6	6007	ZINC000257615280
7.3	-10.3	-10.0	0.23	1.998	0.590	4.840	2.013	6	6008	ZINC000013373243
7.3	-10.3	-9.8	0.26	3.304	0.604	8.105	1.974	6	6009	ZINC000257415838
7.3	-9.3	-9.0	0.21	2.275	0.634	8.867	0.495	6	6010	ZINC000014644559
7.3	-9.1	-9.0	0.12	2.477	0.361	6.183	2.889	6	6011	ZINC000014930809

7.2	-9.4	-9.2	0.11	2.656	0.361	8.413	1.851	6	6012	ZINC000257493864
7.2	-11.4	-11.0	0.27	1.825	0.404	3.703	1.799	6	6013	ZINC000257623035
7.2	-9.0	-8.6	0.22	2.886	0.905	8.743	1.929	6	6014	ZINC000014781051
7.2	-9.1	-9.0	0.13	2.025	0.526	9.092	0.246	6	6015	ZINC000100829911
7.2	-9.0	-8.6	0.24	2.400	0.493	6.582	2.034	6	6016	ZINC000014959367
7.2	-10.8	-10.2	0.31	2.649	0.115	7.192	0.195	6	6017	ZINC000257580978
7.2	-8.7	-8.6	0.11	2.524	0.596	5.798	2.556	6	6018	ZINC000014759029
7.2	-10.4	-10.2	0.19	2.161	0.410	5.918	1.089	6	6019	ZINC000257445510
7.2	-11.0	-10.5	0.31	2.573	0.843	4.718	2.757	6	6020	ZINC000100778993
7.2	-8.9	-8.7	0.23	2.043	0.776	5.787	2.895	6	6021	ZINC000257557274
7.2	-9.6	-8.9	0.43	3.042	0.269	7.204	0.835	6	6022	ZINC000003860325
7.2	-8.6	-8.5	0.14	2.350	0.678	4.520	2.296	6	6023	ZINC000015257347
7.2	-8.6	-8.5	0.14	2.821	0.136	5.770	1.422	6	6024	ZINC000230163200
7.2	-9.5	-8.6	0.47	2.412	0.503	6.500	2.286	6	6025	ZINC000086039796
7.2	-10.1	-9.8	0.21	2.795	0.522	6.633	2.095	6	6026	ZINC000257525012

7.2	-9.8	-9.5	0.27	2.570	0.292	7.720	1.853	6	6027	ZINC000257573781
7.2	-9.5	-9.3	0.14	1.955	0.656	6.121	2.882	6	6028	ZINC000014728432
7.2	-10.3	-9.6	0.32	2.287	0.753	4.303	2.000	6	6029	ZINC000004096816
7.2	-11.4	-11.2	0.21	1.906	0.123	5.246	0.525	6	6030	ZINC000150366304
7.2	-10.5	-10.2	0.26	2.032	0.271	5.740	1.574	6	6031	ZINC000085956010
7.2	-8.7	-8.6	0.05	2.323	0.428	8.016	2.567	6	6032	ZINC000257533823
7.2	-11.0	-10.4	0.35	2.549	0.883	4.692	2.779	6	6033	ZINC000257393659
7.2	-10.7	-10.4	0.16	2.419	0.920	5.455	2.633	6	6034	ZINC000118937361
7.2	-9.4	-9.2	0.22	2.919	0.891	6.490	2.104	6	6035	ZINC000256430526
7.2	-10.2	-9.6	0.31	1.571	0.488	5.362	0.923	6	6036	ZINC000257416175
7.2	-10.8	-10.2	0.37	2.581	0.675	5.937	0.861	6	6037	ZINC000014435557
7.2	-10.4	-10.0	0.19	3.507	0.476	7.570	1.155	6	6038	ZINC000085589038
7.2	-10.0	-9.7	0.17	2.527	0.395	6.128	2.585	6	6039	ZINC000014760595
7.2	-10.9	-10.7	0.20	1.627	0.181	5.415	1.449	6	6040	ZINC000085532003
7.2	-10.5	-10.2	0.16	2.468	0.223	6.603	1.710	6	6041	ZINC000086041842

7.2	-9.9	-9.8	0.15	2.335	0.242	5.509	1.522	6	6042	ZINC000257430038
7.2	-9.0	-8.8	0.14	2.795	0.246	5.500	2.086	6	6043	ZINC000013416202
7.2	-9.5	-9.1	0.24	2.347	0.253	4.276	0.784	6	6044	ZINC000100825548
7.2	-12.0	-11.0	0.69	1.711	0.694	5.478	2.137	6	6045	ZINC000100824400
7.1	-9.9	-9.4	0.27	2.994	0.860	6.421	2.902	6	6046	ZINC000261495059
7.1	-9.5	-9.2	0.22	2.299	0.400	5.741	1.713	6	6047	ZINC000257554977
7.1	-11.3	-10.8	0.28	1.960	0.349	4.878	0.963	6	6048	ZINC000100773041
7.1	-10.3	-9.8	0.35	2.795	0.344	7.474	0.848	6	6049	ZINC000100825647
7.1	-10.6	-10.1	0.25	2.803	0.566	8.256	1.888	6	6050	ZINC000257439875
7.1	-10.7	-9.5	0.55	2.620	0.289	5.623	0.191	6	6051	ZINC000013429510
7.1	-10.6	-9.8	0.36	2.428	0.649	3.897	0.787	6	6052	ZINC000257439874
7.1	-10.5	-10.2	0.17	2.482	0.281	6.078	1.686	6	6053	ZINC000257459143
7.1	-9.9	-9.5	0.24	1.861	0.357	4.695	2.288	6	6054	ZINC000257532630
7.1	-10.6	-10.4	0.21	2.527	0.057	6.635	2.280	6	6055	ZINC000005085289
7.1	-10.3	-9.5	0.68	2.221	0.575	7.393	1.880	6	6056	ZINC000085862052

7.1	-10.1	-9.7	0.27	3.973	0.655	8.171	2.448	6	6057	ZINC000257402073
7.1	-9.7	-9.0	0.35	3.138	0.248	7.472	1.276	6	6058	ZINC000004096020
7.1	-9.2	-8.8	0.24	2.699	0.636	3.952	0.965	6	6059	ZINC000257493863
7.1	-9.8	-9.4	0.36	2.018	0.631	3.540	2.342	6	6060	ZINC000100821975
7.1	-9.8	-9.5	0.24	1.813	0.766	3.523	2.302	6	6061	ZINC000014435240
7.1	-10.6	-10.3	0.19	2.603	0.903	5.714	2.394	6	6062	ZINC000118937363
7.1	-9.1	-8.7	0.22	1.846	0.330	6.441	3.219	6	6063	ZINC000014727575
7.1	-10.8	-10.3	0.31	2.568	0.521	3.296	0.836	6	6064	ZINC000100781705
7.1	-11.0	-10.8	0.11	2.135	0.525	5.645	2.090	6	6065	ZINC000033831805
7.1	-9.9	-9.3	0.29	1.662	0.538	3.752	1.886	6	6066	ZINC000118937147
7.1	-9.6	-9.2	0.21	2.906	0.901	5.881	2.901	6	6067	ZINC000006763464
7.1	-10.6	-10.0	0.34	2.533	0.356	5.813	2.509	6	6068	ZINC000257439873
7.1	-12.1	-11.6	0.43	1.975	0.603	5.908	1.179	6	6069	ZINC000003978478
7.1	-9.5	-9.1	0.33	3.189	0.892	5.570	2.030	6	6070	ZINC000006394163
7.1	-10.2	-10.1	0.11	3.058	0.366	8.439	1.682	6	6071	ZINC000085589146

7.1	-10.5	-10.1	0.18	2.453	0.354	6.539	1.634	6	6072	ZINC000257459140
7.1	-9.3	-8.9	0.23	2.313	0.269	7.742	0.919	6	6073	ZINC000100829103
7.1	-9.9	-9.6	0.27	2.346	0.254	6.588	2.050	6	6074	ZINC000257485999
7.1	-9.7	-9.4	0.26	2.515	0.385	5.276	1.562	6	6075	ZINC000257505313
7.1	-9.3	-9.1	0.17	2.112	0.562	4.661	2.538	6	6076	ZINC000014813651
7.1	-10.1	-9.7	0.22	3.618	1.350	7.787	3.246	6	6077	ZINC000257402074
7.1	-10.0	-9.5	0.24	2.182	0.601	3.688	1.108	6	6078	ZINC000014645285
7.1	-9.3	-9.0	0.20	2.773	0.518	5.570	0.585	6	6079	ZINC000257507307
7.1	-10.1	-9.6	0.26	3.440	1.180	6.700	3.391	6	6080	ZINC000257402076
7.1	-8.8	-8.2	0.41	2.538	0.221	6.616	1.606	6	6081	ZINC000195759751
7.1	-10.1	-9.7	0.23	3.278	0.987	5.132	1.655	6	6082	ZINC000014502290
7.1	-10.5	-10.2	0.22	2.458	0.348	6.551	1.624	6	6083	ZINC000257459141
7.1	-10.5	-10.3	0.18	2.305	0.370	4.377	1.598	6	6084	ZINC000118915336
7.1	-9.8	-9.2	0.35	1.858	0.581	4.708	1.963	6	6085	ZINC000257389780
7.1	-10.5	-10.2	0.19	2.321	0.179	5.930	1.800	6	6086	ZINC000118915338

7.1	-9.5	-9.4	0.05	2.424	0.727	5.978	3.040	6	6087	ZINC000006036380
7.1	-9.8	-9.3	0.30	1.819	0.457	5.094	3.738	6	6088	ZINC000257487949
7.1	-11.3	-11.1	0.14	2.251	0.516	6.247	2.253	6	6089	ZINC000150361500
7.1	-10.7	-9.9	0.43	2.381	0.814	4.714	2.187	6	6090	ZINC000257562848
7.1	-11.3	-11.1	0.20	2.109	0.711	3.975	1.557	6	6091	ZINC000100781693
7.1	-10.5	-10.2	0.20	2.407	0.313	4.359	0.600	6	6092	ZINC000257459142
7.1	-9.2	-8.9	0.20	1.647	0.249	5.569	3.851	6	6093	ZINC000257493297
7.1	-12.1	-11.0	0.67	1.922	0.552	4.752	2.275	6	6094	ZINC000100775855
7.1	-10.1	-9.8	0.20	3.292	1.332	6.504	3.466	6	6095	ZINC000257402075
7.1	-10.9	-10.4	0.26	2.520	0.588	5.496	2.036	6	6096	ZINC000014612721
7.1	-8.6	-8.5	0.12	3.146	1.314	7.342	2.479	6	6097	ZINC000015148361
7.1	-9.4	-9.2	0.12	2.430	0.518	5.642	3.077	6	6098	ZINC000004349687
7.1	-10.5	-10.2	0.18	2.437	0.276	6.125	1.646	6	6099	ZINC000118915337
7.1	-10.8	-10.1	0.38	3.058	0.762	5.568	2.864	6	6100	ZINC000257549723
7.1	-9.8	-9.7	0.10	2.208	0.359	5.453	1.984	6	6101	ZINC000257414769

7.1	-10.8	-10.4	0.21	2.190	0.393	6.662	2.431	6	6102	ZINC000100825482
7.1	-10.5	-10.2	0.19	2.369	0.340	4.925	1.425	6	6103	ZINC000118915339
7.1	-10.9	-10.3	0.34	2.261	0.233	6.935	2.024	6	6104	ZINC000095617890
7.0	-12.2	-11.2	0.50	2.828	0.775	7.280	1.059	6	6105	ZINC000100779736
7.0	-10.1	-9.8	0.17	2.420	0.402	6.404	2.343	6	6106	ZINC000100829786
7.0	-10.0	-9.8	0.11	2.487	0.288	5.407	0.883	6	6107	ZINC000257592429
7.0	-10.1	-9.4	0.35	3.328	0.912	5.975	2.753	6	6108	ZINC000013374706
7.0	-9.5	-9.3	0.11	3.318	0.653	7.007	2.400	6	6109	ZINC000100778452
7.0	-10.0	-9.3	0.32	4.612	0.468	7.469	1.198	6	6110	ZINC000004416666
7.0	-9.6	-9.3	0.23	2.050	1.004	4.204	0.981	6	6111	ZINC000256235055
7.0	-9.5	-9.2	0.18	1.930	0.736	4.607	2.626	6	6112	ZINC000257474407
7.0	-11.6	-9.8	0.81	3.224	0.528	5.717	1.115	6	6113	ZINC000257562752
7.0	-10.1	-10.0	0.11	2.452	0.352	6.810	0.941	6	6114	ZINC000100830135
7.0	-9.8	-9.2	0.28	1.892	0.409	3.642	2.586	6	6115	ZINC000014887434
7.0	-12.2	-11.3	0.45	2.624	0.983	6.244	2.409	6	6116	ZINC000257535639

7.0	-10.1	-9.8	0.15	2.599	0.457	5.470	2.303	6	6117	ZINC000100829788
7.0	-9.8	-9.3	0.31	2.785	0.480	5.541	1.703	6	6118	ZINC000100826977
7.0	-10.1	-9.8	0.17	2.572	0.481	5.469	2.288	6	6119	ZINC000257474184
7.0	-10.9	-10.7	0.21	2.015	0.501	5.204	2.289	6	6120	ZINC000257623038
7.0	-9.6	-9.2	0.30	1.785	0.432	6.047	2.186	6	6121	ZINC000257544779
7.0	-12.2	-11.2	0.49	2.830	0.779	7.280	1.055	6	6122	ZINC000257535637
7.0	-10.6	-10.1	0.29	2.463	0.600	6.151	2.883	6	6123	ZINC000257504568
7.0	-10.8	-10.3	0.23	1.843	0.219	3.957	2.108	6	6124	ZINC000257573439
7.0	-10.1	-9.2	0.51	2.164	0.192	7.722	0.221	6	6125	ZINC000257504268
7.0	-9.4	-9.0	0.21	1.884	0.343	2.757	0.517	6	6126	ZINC000257488512
7.0	-12.2	-11.2	0.50	2.827	0.780	7.277	1.060	6	6127	ZINC000100779735
7.0	-10.4	-9.9	0.24	2.018	0.532	6.293	3.400	6	6128	ZINC000085955327
7.0	-9.5	-9.3	0.11	3.222	0.689	7.812	1.956	6	6129	ZINC000257508763
7.0	-10.1	-9.8	0.17	2.566	0.466	5.457	2.288	6	6130	ZINC000100829791
7.0	-8.9	-8.4	0.26	2.731	1.482	5.540	2.402	6	6131	ZINC000014506013

7.0	-9.5	-8.9	0.31	2.202	0.410	9.547	0.315	6	6132	ZINC000257487566
7.0	-9.5	-9.3	0.13	2.353	0.185	7.545	3.329	6	6133	ZINC000014811801
7.0	-10.5	-10.4	0.13	2.584	0.924	4.621	2.315	6	6134	ZINC000118937360
7.0	-11.5	-10.4	0.50	2.517	0.182	5.289	0.856	6	6135	ZINC000100772962
7.0	-10.1	-9.7	0.26	1.723	0.398	3.959	1.911	6	6136	ZINC000100829395
7.0	-9.7	-8.7	0.49	2.072	0.273	5.926	3.568	6	6137	ZINC000257504788
7.0	-10.1	-9.3	0.40	2.035	0.585	5.007	2.555	6	6138	ZINC000257462918
7.0	-9.4	-9.1	0.21	2.829	0.529	4.996	1.776	6	6139	ZINC000257498123
7.0	-10.1	-9.9	0.11	3.168	0.752	7.879	0.667	6	6140	ZINC000100830132
7.0	-9.5	-9.3	0.09	2.931	0.431	6.208	2.714	6	6141	ZINC000257442786
7.0	-10.1	-9.8	0.25	2.200	0.464	6.384	3.343	6	6142	ZINC000257498577
7.0	-12.2	-11.1	0.56	2.796	0.738	7.802	0.424	6	6143	ZINC000100779734
7.0	-9.0	-8.8	0.12	2.533	0.263	5.779	1.259	6	6144	ZINC000013373238
7.0	-9.3	-8.9	0.24	3.527	0.426	5.967	0.982	6	6145	ZINC000015257555
7.0	-9.7	-9.2	0.25	2.317	0.372	6.543	0.834	6	6146	ZINC000256073695

7.0	-12.2	-11.2	0.50	2.826	0.773	7.280	1.060	6	6147	ZINC000257535636
7.0	-13.3	-11.5	0.90	2.103	0.591	5.174	1.138	6	6148	ZINC000086041863
7.0	-10.5	-10.1	0.21	1.745	0.621	5.025	1.903	6	6149	ZINC000100822600
7.0	-10.9	-10.2	0.31	2.776	0.834	6.717	2.920	6	6150	ZINC000118937221
7.0	-9.2	-8.8	0.21	3.292	0.549	5.522	0.486	6	6151	ZINC000014450975
7.0	-10.1	-9.8	0.13	2.562	0.469	4.426	2.012	6	6152	ZINC000257474183
7.0	-9.8	-9.5	0.30	2.248	0.351	6.565	3.085	6	6153	ZINC000014757252
7.0	-9.5	-9.0	0.22	3.265	0.729	7.016	1.450	6	6154	ZINC000000338230
7.0	-12.2	-11.1	0.54	2.797	0.747	7.802	0.425	6	6155	ZINC000257535638
7.0	-10.0	-9.4	0.27	3.071	0.579	7.496	3.134	6	6156	ZINC000100820464
7.0	-9.0	-8.8	0.15	3.373	1.975	5.666	2.988	6	6157	ZINC000012494196
7.0	-10.5	-10.0	0.26	2.470	0.694	5.701	2.064	6	6158	ZINC000100772862
7.0	-13.0	-12.1	0.60	3.228	0.690	6.295	1.868	6	6159	ZINC000039166884
6.9	-9.9	-9.5	0.26	2.328	0.475	6.404	1.424	6	6160	ZINC000085589041
6.9	-10.4	-10.0	0.30	1.632	0.506	3.253	1.384	6	6161	ZINC000257437771

6.9	-10.8	-10.3	0.24	2.363	0.927	4.364	2.457	6	6162	ZINC000118937222
6.9	-10.8	-10.3	0.31	2.854	0.589	4.414	2.368	6	6163	ZINC000100781700
6.9	-9.1	-8.9	0.20	2.279	0.461	6.074	0.333	6	6164	ZINC000085863097
6.9	-8.9	-8.6	0.21	3.420	1.036	7.206	2.579	6	6165	ZINC000004365609
6.9	-8.7	-8.5	0.13	2.121	0.252	5.951	1.902	6	6166	ZINC000100822878
6.9	-10.1	-9.7	0.37	2.206	0.191	5.115	1.846	6	6167	ZINC000257415566
6.9	-9.3	-9.1	0.19	2.321	0.652	6.904	2.730	6	6168	ZINC000257544598
6.9	-10.6	-10.0	0.28	2.860	0.608	9.235	0.348	6	6169	ZINC000100779951
6.9	-9.0	-8.7	0.15	2.290	0.540	5.269	2.827	6	6170	ZINC000014658112
6.9	-9.7	-9.3	0.18	3.143	0.360	6.641	0.895	6	6171	ZINC000014610287
6.9	-11.0	-10.7	0.32	2.186	0.644	3.957	2.577	6	6172	ZINC000100823757
6.9	-11.0	-10.5	0.43	2.265	0.467	4.362	2.459	6	6173	ZINC000100823759
6.9	-10.7	-9.8	0.55	2.149	0.100	5.985	0.603	6	6174	ZINC000257410665
6.9	-10.1	-9.4	0.38	2.344	0.637	4.489	2.135	6	6175	ZINC000118937303
6.9	-11.0	-10.6	0.46	1.979	0.428	4.149	2.585	6	6176	ZINC000257381187

6.9	-11.0	-10.5	0.26	2.589	0.580	8.081	2.807	6	6177	ZINC000100825174
6.9	-10.3	-9.8	0.32	2.445	0.509	5.954	2.119	6	6178	ZINC000253589675
6.9	-10.4	-9.8	0.33	2.139	0.562	4.586	2.239	6	6179	ZINC000100775316
6.9	-13.2	-12.4	0.72	1.443	0.705	5.446	2.129	6	6180	ZINC000013378117
6.9	-11.1	-10.3	0.39	2.436	0.551	6.367	2.312	6	6181	ZINC000257513124
6.9	-10.8	-10.4	0.32	2.203	0.374	2.869	0.709	6	6182	ZINC000257549724
6.9	-9.1	-8.8	0.14	2.763	0.396	8.646	2.213	6	6183	ZINC000257562471
6.9	-10.4	-9.9	0.38	2.237	0.414	5.218	1.141	6	6184	ZINC000261498680
6.9	-10.4	-9.9	0.34	1.989	0.786	4.458	2.337	6	6185	ZINC000257437770
6.9	-11.0	-10.5	0.44	2.274	0.477	4.420	2.414	6	6186	ZINC000257381185
6.9	-11.0	-10.5	0.56	2.217	0.466	4.775	2.632	6	6187	ZINC000100823761
6.9	-9.7	-9.2	0.28	2.052	0.350	5.536	2.000	6	6188	ZINC000014921511
6.9	-10.8	-10.3	0.23	2.054	1.006	4.275	2.837	6	6189	ZINC000118937223
6.9	-10.3	-9.9	0.30	2.735	1.082	6.151	3.641	6	6190	ZINC000040164384
6.9	-9.6	-9.4	0.17	2.489	0.728	4.598	2.862	6	6191	ZINC000014811817

6.9	-12.4	-11.2	0.62	2.177	0.118	5.826	1.574	6	6192	ZINC000014612722
6.9	-11.0	-10.5	0.42	2.271	0.467	4.430	2.401	6	6193	ZINC000257381188
6.9	-10.8	-10.6	0.11	2.059	0.100	5.772	1.480	6	6194	ZINC000059730503
6.9	-10.4	-10.0	0.32	1.957	0.800	4.453	2.343	6	6195	ZINC000257437772
6.9	-9.2	-9.0	0.16	2.580	0.196	6.915	2.135	6	6196	ZINC000100823419
6.9	-10.4	-10.0	0.27	1.674	0.513	3.233	1.404	6	6197	ZINC000100775320
6.9	-10.9	-10.2	0.41	2.157	0.445	6.706	1.921	6	6198	ZINC000100827595
6.9	-11.1	-10.8	0.19	2.032	0.184	5.858	1.186	6	6199	ZINC000100824983
6.9	-8.7	-8.4	0.15	2.366	0.657	4.271	2.359	6	6200	ZINC000014436818
6.9	-10.4	-10.2	0.19	2.310	0.292	6.599	0.280	6	6201	ZINC000257488524
6.9	-10.7	-10.3	0.29	2.062	0.388	5.046	2.079	6	6202	ZINC000100772855
6.9	-10.3	-9.8	0.27	2.953	0.380	5.813	1.448	6	6203	ZINC000059727326
6.9	-9.4	-9.0	0.23	2.513	0.420	6.333	2.454	6	6204	ZINC000014728346
6.9	-10.8	-10.3	0.24	2.672	0.910	5.866	2.866	6	6205	ZINC000118937220
6.9	-9.8	-9.3	0.34	2.465	0.580	4.113	1.657	6	6206	ZINC000019418979

6.9	-10.8	-10.3	0.28	2.860	0.781	6.463	2.456	6	6207	ZINC000257464499
6.9	-9.5	-9.1	0.18	2.439	0.915	4.995	2.798	6	6208	ZINC000257484780
6.9	-11.2	-11.0	0.17	2.166	0.196	5.252	1.728	6	6209	ZINC000085977720
6.9	-9.6	-9.1	0.24	2.209	0.482	5.310	2.933	6	6210	ZINC000014887445
6.8	-7.4	-7.1	0.14	1.750	0.491	6.606	3.064	6	6211	ZINC000085910443
6.8	-9.6	-9.2	0.28	2.297	0.784	4.879	1.975	6	6212	ZINC000261498268
6.8	-11.2	-10.9	0.22	2.167	0.373	5.925	0.630	6	6213	ZINC000100778297
6.8	-9.9	-9.2	0.60	2.160	0.185	6.397	1.450	6	6214	ZINC000257399598
6.8	-9.8	-9.0	0.34	2.289	0.127	4.295	0.956	6	6215	ZINC000257490155
6.8	-10.2	-9.6	0.37	2.070	0.845	2.975	1.094	6	6216	ZINC000015262016
6.8	-10.2	-10.0	0.17	2.868	0.213	6.129	0.809	6	6217	ZINC000100822599
6.8	-10.2	-9.9	0.22	3.193	0.804	9.270	0.315	6	6218	ZINC000257565508
6.8	-10.7	-10.5	0.18	2.438	0.210	6.617	0.825	6	6219	ZINC000257445511
6.8	-10.5	-10.3	0.15	2.359	0.371	5.955	1.201	6	6220	ZINC000100827593
6.8	-10.1	-9.8	0.17	2.161	0.864	4.042	1.165	6	6221	ZINC000014858269

6.8	-10.3	-9.2	0.52	2.515	0.311	6.321	0.920	6	6222	ZINC000257444946
6.8	-9.9	-9.5	0.27	2.786	0.303	7.317	0.316	6	6223	ZINC000005195808
6.8	-9.5	-8.7	0.39	2.254	0.631	5.264	2.467	6	6224	ZINC000033833244
6.8	-9.9	-9.5	0.22	2.464	0.544	4.759	2.751	6	6225	ZINC000257462239
6.8	-9.2	-8.6	0.33	2.646	0.203	5.246	1.325	6	6226	ZINC000052650196
6.8	-8.5	-8.4	0.07	1.002	0.587	3.817	2.226	6	6227	ZINC000004098933
6.8	-12.3	-11.6	0.49	2.125	0.495	7.263	1.042	6	6228	ZINC000100825712
6.8	-9.1	-8.8	0.18	2.517	0.603	6.355	2.304	6	6229	ZINC000014695527
6.8	-10.2	-9.8	0.28	2.460	0.302	6.611	2.373	6	6230	ZINC000013374704
6.8	-9.0	-8.8	0.13	2.386	0.625	7.917	2.864	6	6231	ZINC000257603264
6.8	-9.8	-9.3	0.32	2.012	0.434	6.600	1.981	6	6232	ZINC000100824589
6.8	-11.6	-10.9	0.38	2.245	0.913	4.370	2.885	6	6233	ZINC000085588754
6.8	-11.4	-10.9	0.27	2.313	0.237	7.526	1.407	6	6234	ZINC000257512510
6.8	-11.1	-10.8	0.24	2.061	0.324	5.521	2.113	6	6235	ZINC000014951634
6.8	-9.5	-9.2	0.19	2.193	0.459	4.232	2.549	6	6236	ZINC000014887439

6.8	-7.4	-7.2	0.17	1.620	0.706	3.883	2.576	6	6237	ZINC000014504560
6.8	-9.3	-9.3	0.07	2.314	0.816	5.847	3.255	6	6238	ZINC000100828779
6.8	-11.0	-10.7	0.18	2.508	0.229	6.116	1.827	6	6239	ZINC000100825720
6.8	-9.3	-9.1	0.13	3.964	0.612	7.206	0.946	6	6240	ZINC000257431556
6.8	-9.5	-9.1	0.19	2.167	0.555	6.342	3.377	6	6241	ZINC000014644563
6.8	-9.9	-9.8	0.11	2.604	0.697	5.784	2.947	6	6242	ZINC000257535031
6.8	-9.2	-8.9	0.16	1.973	0.265	7.000	2.398	6	6243	ZINC000013547730
6.8	-9.5	-9.3	0.11	3.311	0.652	6.992	2.413	6	6244	ZINC000100778447
6.8	-9.2	-9.0	0.12	3.757	1.349	6.905	2.069	6	6245	ZINC000257431555
6.8	-7.9	-7.9	0.04	0.296	0.171	5.838	2.864	6	6246	ZINC000034024620
6.8	-10.6	-9.9	0.37	3.650	0.241	7.402	1.747	6	6247	ZINC000095619783
6.8	-10.9	-10.5	0.17	2.017	0.406	6.120	2.440	6	6248	ZINC000100825229
6.8	-10.2	-9.8	0.24	2.947	1.050	4.981	2.310	6	6249	ZINC000049878230
6.8	-11.6	-10.9	0.42	2.706	0.826	4.912	1.764	6	6250	ZINC000257555288
6.7	-10.9	-10.6	0.25	2.422	0.734	4.759	2.067	6	6251	ZINC000040165203

6.7	-9.4	-9.2	0.17	3.042	0.866	6.008	2.578	6	6252	ZINC000100776835
6.7	-8.8	-8.7	0.09	2.653	0.927	5.817	2.708	6	6253	ZINC000012494340
6.7	-11.4	-11.2	0.22	2.098	0.720	3.962	1.557	6	6254	ZINC000257417335
6.7	-10.2	-9.8	0.28	2.035	0.468	6.131	2.812	6	6255	ZINC000257736992
6.7	-9.8	-9.5	0.21	2.451	0.538	6.342	2.516	6	6256	ZINC000257439656
6.7	-9.1	-9.0	0.15	1.850	0.824	6.682	2.993	6	6257	ZINC000100829914
6.7	-11.0	-10.5	0.31	2.270	0.452	5.192	1.482	6	6258	ZINC000015148337
6.7	-10.2	-9.9	0.23	3.024	0.212	6.796	0.864	6	6259	ZINC000257488522
6.7	-10.4	-10.0	0.29	2.600	0.321	8.966	0.043	6	6260	ZINC000100784253
6.7	-7.6	-6.9	0.34	4.094	1.311	5.692	2.553	6	6261	ZINC000001714282
6.7	-10.5	-9.9	0.29	2.212	0.615	5.056	2.599	6	6262	ZINC000257491426
6.7	-8.8	-8.6	0.16	3.776	0.588	7.597	2.160	6	6263	ZINC000014610077
6.7	-9.4	-9.3	0.11	2.045	0.613	5.111	3.024	6	6264	ZINC000257441269
6.7	-11.2	-11.0	0.18	1.907	1.145	5.197	3.523	6	6265	ZINC000086043943
6.7	-9.1	-8.7	0.22	2.649	0.605	8.494	2.953	6	6266	ZINC000059732983

6.7	-10.5	-10.4	0.08	1.997	0.182	4.458	2.048	6	6267	ZINC000024215131
6.7	-9.3	-9.2	0.10	1.959	0.255	6.808	2.941	6	6268	ZINC000257607437
6.7	-9.9	-9.5	0.28	2.513	0.776	5.158	2.320	6	6269	ZINC000257525458
6.7	-10.6	-10.4	0.15	2.780	0.629	6.881	0.950	6	6270	ZINC000257431427
6.7	-7.4	-7.1	0.18	1.896	0.421	5.462	3.108	6	6271	ZINC000039025773
6.7	-10.4	-9.7	0.41	2.407	0.696	4.303	2.061	6	6272	ZINC000257484779
6.7	-10.2	-10.0	0.21	2.844	0.242	6.107	0.837	6	6273	ZINC000100822598
6.7	-13.4	-12.9	0.29	2.152	0.725	5.736	2.419	6	6274	ZINC000100777478
6.7	-10.2	-10.0	0.16	1.797	0.447	4.122	2.433	6	6275	ZINC000095912140
6.7	-9.6	-9.2	0.28	2.657	0.728	7.082	2.543	6	6276	ZINC000015261715
6.7	-12.0	-11.2	0.59	2.158	0.740	4.796	2.334	6	6277	ZINC000086041814
6.7	-9.4	-9.2	0.12	2.061	0.623	3.243	1.094	6	6278	ZINC000257388910
6.7	-10.3	-10.0	0.22	2.481	0.718	5.998	1.658	6	6279	ZINC000049771447
6.7	-8.7	-8.5	0.13	2.066	0.738	4.704	2.341	6	6280	ZINC000100829888
6.7	-10.2	-10.0	0.19	2.731	0.438	6.056	0.907	6	6281	ZINC000257488521

6.7	-11.4	-10.7	0.37	2.095	0.314	6.029	1.424	6	6282	ZINC000085742437
6.7	-8.6	-8.3	0.18	2.597	0.836	5.646	2.110	6	6283	ZINC000257432897
6.7	-8.8	-8.6	0.13	3.648	0.883	8.259	1.783	6	6284	ZINC000014613168
6.7	-9.0	-8.7	0.17	2.206	0.635	5.775	3.539	6	6285	ZINC000015060717
6.7	-10.0	-9.6	0.19	2.489	0.244	6.028	2.015	6	6286	ZINC000257382540
6.7	-9.4	-9.1	0.14	2.541	0.594	5.652	1.940	6	6287	ZINC000257534410
6.7	-10.1	-9.5	0.42	2.000	0.455	5.423	1.976	6	6288	ZINC000257419090
6.7	-8.3	-7.9	0.20	4.720	1.227	7.908	2.439	6	6289	ZINC000003792789
6.7	-9.4	-9.0	0.21	2.842	0.519	6.471	1.322	6	6290	ZINC000257608680
6.7	-9.2	-8.7	0.31	1.803	0.392	4.239	1.781	6	6291	ZINC000100772891
6.7	-13.4	-12.9	0.27	2.163	0.735	5.747	2.420	6	6292	ZINC000257409035
6.7	-13.4	-13.0	0.25	2.453	1.146	5.678	2.368	6	6293	ZINC000100777474
6.7	-9.1	-8.8	0.29	2.672	0.742	5.630	2.311	6	6294	ZINC000100829702
6.7	-9.0	-8.8	0.13	2.490	0.430	7.841	1.672	6	6295	ZINC000257397620
6.7	-9.3	-9.0	0.29	2.570	0.534	5.995	2.824	6	6296	ZINC000257447799

6.7	-10.7	-10.0	0.49	2.585	1.398	5.224	2.933	6	6297	ZINC000006069771
6.7	-10.0	-9.1	0.64	2.172	0.509	5.394	2.186	6	6298	ZINC000100822477
6.7	-6.7	-6.5	0.09	1.823	0.230	4.431	1.620	6	6299	ZINC000001850792
6.6	-10.1	-9.5	0.29	2.341	0.136	6.305	1.489	6	6300	ZINC000247978456
6.6	-9.0	-9.0	0.05	2.447	0.215	7.643	2.543	6	6301	ZINC000085541181
6.6	-11.8	-11.3	0.37	1.984	0.218	5.167	0.812	6	6302	ZINC000100829275
6.6	-9.7	-9.2	0.33	3.051	0.251	6.780	1.527	6	6303	ZINC000118936792
6.6	-9.9	-9.6	0.17	2.805	0.402	4.815	0.866	6	6304	ZINC000257615277
6.6	-10.0	-9.2	0.38	2.643	0.555	7.785	2.539	6	6305	ZINC000257567289
6.6	-10.9	-10.5	0.24	2.232	0.567	5.269	1.522	6	6306	ZINC000257461099
6.6	-10.1	-9.7	0.45	2.289	0.815	5.302	1.422	6	6307	ZINC000257375887
6.6	-10.0	-9.4	0.38	2.933	0.143	5.299	1.015	6	6308	ZINC000015219770
6.6	-12.0	-10.8	0.64	2.330	0.255	5.940	0.398	6	6309	ZINC000085862070
6.6	-9.0	-8.7	0.15	2.007	0.435	5.814	2.368	6	6310	ZINC000000712672
6.6	-9.2	-8.9	0.16	2.527	0.413	5.917	2.607	6	6311	ZINC000100771917

6.6	-7.4	-7.2	0.18	2.030	1.051	5.398	3.592	6	6312	ZINC000100826593
6.6	-8.9	-8.7	0.17	2.470	0.566	5.740	2.201	6	6313	ZINC000059772474
6.6	-8.6	-8.3	0.21	1.960	0.461	7.454	3.418	6	6314	ZINC000011666971
6.6	-9.2	-9.0	0.15	2.871	0.776	8.926	3.017	6	6315	ZINC000011666642
6.6	-7.6	-7.4	0.10	4.233	1.810	7.098	2.943	6	6316	ZINC000012496191
6.6	-10.9	-10.4	0.31	2.087	1.064	4.628	2.485	6	6317	ZINC000150368156
6.6	-9.9	-9.7	0.22	1.602	0.414	3.575	1.311	6	6318	ZINC000100828720
6.6	-10.1	-9.7	0.22	2.031	0.517	6.366	3.509	6	6319	ZINC000257471568
6.6	-10.3	-9.8	0.30	2.515	0.485	6.400	2.310	6	6320	ZINC000100826879
6.6	-10.5	-9.9	0.30	2.846	0.432	7.581	1.154	6	6321	ZINC000100825435
6.6	-10.0	-9.4	0.27	2.463	0.831	5.511	2.839	6	6322	ZINC000257476394
6.6	-9.1	-8.5	0.38	2.413	0.587	5.169	1.581	6	6323	ZINC000085589158
6.6	-9.9	-9.4	0.30	1.744	0.725	2.970	0.624	6	6324	ZINC000015209464
6.6	-10.1	-9.5	0.30	3.867	0.504	8.349	0.615	6	6325	ZINC000085935100
6.6	-10.2	-9.4	0.40	2.405	0.605	4.926	1.938	6	6326	ZINC000257771090

6.6	-10.3	-10.0	0.15	2.126	0.407	5.369	2.163	6	6327	ZINC000257623736
6.6	-9.1	-8.6	0.36	3.150	1.047	6.505	1.824	6	6328	ZINC000014727641
6.6	-8.9	-8.5	0.20	3.404	0.511	5.256	1.388	6	6329	ZINC000148605973
6.6	-8.9	-8.5	0.17	3.664	1.666	5.735	2.316	6	6330	ZINC000410428656
6.6	-9.0	-8.8	0.12	2.107	0.749	3.879	1.536	6	6331	ZINC000257423423
6.6	-9.8	-9.5	0.21	2.067	0.721	5.113	2.677	6	6332	ZINC000257378883
6.6	-9.9	-9.3	0.32	2.273	0.757	4.230	2.186	6	6333	ZINC000137649401
6.6	-11.3	-10.7	0.36	2.299	0.490	5.377	2.075	6	6334	ZINC000257623036
6.6	-10.7	-10.2	0.24	2.429	0.351	6.039	1.038	6	6335	ZINC000257516675
6.6	-10.6	-10.1	0.31	1.973	0.253	5.396	1.693	6	6336	ZINC000257416177
6.6	-10.5	-10.3	0.16	2.613	0.181	6.874	1.769	6	6337	ZINC000100824377
6.6	-9.9	-9.7	0.20	2.487	0.596	7.852	1.681	6	6338	ZINC000014930843
6.6	-9.9	-9.1	0.39	2.506	0.108	6.424	1.494	6	6339	ZINC000257468171
6.6	-9.2	-9.0	0.15	2.189	0.319	5.609	2.282	6	6340	ZINC000085833800
6.6	-10.7	-10.3	0.26	2.396	0.596	6.435	2.595	6	6341	ZINC000100824376

6.6	-10.2	-9.9	0.17	2.253	0.452	4.414	2.082	6	6342	ZINC000257470603
6.6	-9.7	-9.4	0.28	1.816	0.640	3.917	2.401	6	6343	ZINC000257485776
6.6	-9.3	-9.1	0.19	2.570	0.257	7.192	2.093	6	6344	ZINC000257505608
6.6	-8.6	-8.5	0.07	1.752	0.433	3.101	0.986	6	6345	ZINC000085863064
6.5	-9.5	-9.4	0.14	2.875	0.322	6.429	2.526	6	6346	ZINC000100825550
6.5	-9.9	-9.3	0.34	2.854	1.015	5.192	2.607	6	6347	ZINC000085758509
6.5	-9.2	-8.8	0.22	1.962	0.755	3.824	1.872	6	6348	ZINC000100821972
6.5	-9.1	-8.7	0.22	2.534	0.637	6.401	2.559	6	6349	ZINC000256430531
6.5	-10.4	-9.7	0.35	2.699	0.084	8.505	0.130	6	6350	ZINC000257439654
6.5	-9.5	-9.1	0.21	1.663	0.371	2.395	0.395	6	6351	ZINC000012495612
6.5	-8.8	-8.6	0.17	2.813	0.699	5.723	2.240	6	6352	ZINC000257702853
6.5	-9.9	-9.3	0.31	2.290	0.764	5.012	2.555	6	6353	ZINC000015209457
6.5	-10.1	-9.5	0.38	3.903	0.539	6.324	0.995	6	6354	ZINC000006763466
6.5	-10.7	-9.6	0.59	2.423	0.499	6.081	2.349	6	6355	ZINC000257373612
6.5	-9.8	-9.4	0.23	2.282	0.573	7.209	3.700	6	6356	ZINC000013328772

6.5	-9.2	-9.0	0.24	2.397	0.392	6.964	2.366	6	6357	ZINC000257477826
6.5	-9.9	-9.3	0.35	2.479	1.113	4.212	2.048	6	6358	ZINC000257522740
6.5	-9.9	-9.3	0.32	1.998	0.989	4.244	2.173	6	6359	ZINC000015209462
6.5	-9.4	-8.3	0.68	2.623	0.532	5.834	2.312	6	6360	ZINC000230163207
6.5	-9.9	-9.4	0.26	2.105	0.229	5.123	1.512	6	6361	ZINC000085871712
6.5	-8.8	-8.7	0.07	2.568	0.512	7.919	1.898	6	6362	ZINC000014760535
6.5	-9.4	-8.7	0.38	2.736	0.401	7.465	1.061	6	6363	ZINC000257477825
6.5	-11.5	-10.6	0.54	2.108	0.375	6.736	0.349	6	6364	ZINC000150368143
6.5	-13.2	-12.8	0.20	2.684	0.830	6.681	0.846	6	6365	ZINC000100777479
6.5	-10.5	-9.9	0.31	2.365	0.597	8.663	3.587	6	6366	ZINC000100826861
6.5	-10.3	-9.5	0.38	2.859	0.518	6.987	1.967	6	6367	ZINC000003824120
6.5	-10.1	-9.6	0.28	3.168	0.713	5.258	2.005	6	6368	ZINC000257494860
6.5	-9.9	-9.5	0.29	2.090	0.941	4.023	2.285	6	6369	ZINC000257522739
6.5	-9.2	-8.9	0.24	1.753	0.744	3.399	2.087	6	6370	ZINC000100821974
6.5	-13.1	-12.8	0.37	2.336	0.616	6.678	0.902	6	6371	ZINC000100777470

6.5	-9.1	-8.9	0.17	1.721	0.429	7.542	0.302	6	6372	ZINC000013378554
6.5	-13.2	-12.8	0.20	2.687	0.828	6.686	0.837	6	6373	ZINC000100777476
6.5	-9.9	-9.5	0.30	2.147	0.492	5.018	2.942	6	6374	ZINC000085955988
6.5	-9.9	-9.4	0.28	2.274	0.668	4.651	2.267	6	6375	ZINC000253641056
6.5	-13.2	-12.8	0.22	2.665	0.821	6.608	0.974	6	6376	ZINC000257409036
6.5	-10.3	-9.8	0.30	2.982	0.513	5.530	2.069	6	6377	ZINC000257521374
6.5	-9.7	-9.2	0.24	2.153	0.842	4.752	2.918	6	6378	ZINC000261497586
6.5	-10.1	-9.6	0.40	2.281	0.446	5.065	1.479	6	6379	ZINC000015219777
6.5	-9.8	-9.2	0.28	1.925	0.417	6.650	3.196	6	6380	ZINC000257573780
6.5	-11.0	-10.9	0.07	2.400	0.706	4.200	2.385	6	6381	ZINC000118936942
6.5	-8.6	-8.4	0.21	3.585	0.713	7.604	2.910	6	6382	ZINC000015112142
6.5	-9.4	-9.1	0.22	2.246	0.273	5.522	1.408	6	6383	ZINC000100826517
6.5	-9.8	-9.5	0.15	2.511	0.475	4.102	1.231	6	6384	ZINC000012153882
6.5	-9.4	-9.2	0.16	2.594	0.773	5.945	3.500	6	6385	ZINC000015060719
6.5	-9.9	-9.4	0.27	1.819	0.874	3.958	2.296	6	6386	ZINC000015209460

6.5	-9.2	-8.8	0.18	2.397	0.205	5.040	1.558	6	6387	ZINC000100826225
6.5	-9.1	-8.9	0.13	2.329	0.165	6.480	2.324	6	6388	ZINC000257520469
6.5	-13.1	-12.9	0.31	1.927	0.631	5.517	2.259	6	6389	ZINC000247933185
6.5	-11.3	-10.8	0.30	2.185	0.658	5.771	1.823	6	6390	ZINC000248330746
6.5	-13.1	-12.9	0.19	2.156	0.820	5.004	2.074	6	6391	ZINC000100777471
6.5	-9.6	-9.4	0.12	4.200	0.519	7.790	1.549	6	6392	ZINC000064220350
6.5	-9.3	-8.8	0.27	1.485	0.185	6.470	3.246	6	6393	ZINC000014814771
6.5	-9.5	-9.2	0.21	2.522	0.818	4.730	2.561	6	6394	ZINC000014728345
6.5	-7.4	-7.1	0.16	2.012	0.531	4.214	2.419	6	6395	ZINC000014504555
6.5	-9.5	-8.9	0.29	2.654	0.413	6.140	0.555	6	6396	ZINC000015219774
6.5	-9.5	-9.2	0.17	2.053	0.597	5.099	2.731	6	6397	ZINC000085833801
6.5	-9.2	-9.0	0.16	2.302	0.116	5.684	1.095	6	6398	ZINC000100771564
6.5	-8.7	-8.5	0.11	2.235	0.167	5.668	3.151	6	6399	ZINC000257542990
6.5	-10.3	-9.6	0.36	2.255	0.452	4.715	2.213	6	6400	ZINC000118937009
6.5	-10.0	-9.4	0.27	2.294	0.557	4.390	1.775	6	6401	ZINC000257622340

6.5	-13.1	-12.8	0.32	2.300	0.590	5.970	1.832	6	6402	ZINC000247933190
6.5	-10.2	-9.5	0.35	2.451	0.563	7.130	2.458	6	6403	ZINC000257430576
6.5	-9.3	-8.9	0.20	3.583	0.870	6.723	2.600	6	6404	ZINC000013130928
6.5	-9.8	-9.1	0.44	2.186	0.189	5.804	0.141	6	6405	ZINC000100826878
6.5	-8.6	-8.2	0.28	2.365	0.931	6.530	1.054	6	6406	ZINC000003860707
6.5	-9.9	-9.2	0.34	2.181	0.632	6.267	2.996	6	6407	ZINC000257387409
6.5	-10.4	-10.1	0.20	1.885	0.244	5.174	3.334	6	6408	ZINC000118937376
6.5	-9.6	-9.4	0.12	1.696	0.735	3.937	2.809	6	6409	ZINC000257530282
6.4	-10.2	-9.8	0.26	2.085	0.524	4.453	3.016	6	6410	ZINC000257491283
6.4	-8.8	-8.5	0.21	2.649	1.445	5.330	2.753	6	6411	ZINC000013367232
6.4	-10.3	-9.7	0.35	1.999	0.448	7.051	3.304	6	6412	ZINC000077293264
6.4	-8.9	-8.5	0.20	2.615	1.214	6.685	3.497	6	6413	ZINC000033816043
6.4	-10.4	-9.8	0.36	2.254	0.111	6.220	0.801	6	6414	ZINC000100825562
6.4	-8.1	-7.9	0.11	2.623	0.699	5.848	3.130	6	6415	ZINC000034660468
6.4	-9.6	-9.0	0.38	2.323	1.404	4.027	2.608	6	6416	ZINC000118937340

6.4	-9.8	-9.3	0.29	2.771	0.528	6.453	2.258	6	6417	ZINC000118936839
6.4	-9.7	-9.5	0.13	2.637	0.343	6.927	2.165	6	6418	ZINC000118937209
6.4	-10.2	-9.8	0.23	2.277	0.660	5.268	2.326	6	6419	ZINC000257394472
6.4	-9.3	-9.1	0.14	3.235	0.690	6.032	2.102	6	6420	ZINC000014502669
6.4	-9.9	-9.6	0.15	1.509	0.315	6.943	4.057	6	6421	ZINC000257442390
6.4	-10.6	-9.8	0.56	2.131	0.408	4.689	1.586	6	6422	ZINC000257394918
6.4	-9.9	-9.5	0.32	2.837	1.126	6.806	2.280	6	6423	ZINC000257419522
6.4	-9.0	-8.6	0.20	2.771	0.819	7.183	0.990	6	6424	ZINC000015063086
6.4	-9.3	-9.1	0.17	2.716	0.645	7.848	3.020	6	6425	ZINC000014813789
6.4	-8.9	-8.8	0.15	2.471	0.657	4.972	2.232	6	6426	ZINC000257607436
6.4	-10.2	-9.7	0.30	3.298	0.430	7.264	0.753	6	6427	ZINC000257521371
6.4	-9.6	-9.2	0.24	2.323	0.878	3.743	2.300	6	6428	ZINC000040165501
6.4	-9.2	-8.9	0.21	2.510	0.284	7.127	0.220	6	6429	ZINC000085587783
6.4	-9.6	-9.0	0.36	2.435	0.320	5.945	1.661	6	6430	ZINC000014691748
6.4	-7.7	-7.4	0.21	2.764	1.186	6.115	1.801	6	6431	ZINC000001531669

6.4	-9.0	-8.8	0.18	2.248	0.605	5.478	1.963	6	6432	ZINC000257534411
6.4	-10.8	-10.6	0.15	2.720	0.761	4.359	1.402	6	6433	ZINC000100773313
6.4	-11.2	-10.9	0.24	2.256	0.172	4.217	1.364	6	6434	ZINC000085896638
6.4	-10.9	-10.5	0.17	3.514	0.629	6.514	1.431	6	6435	ZINC000005430837
6.4	-9.3	-9.0	0.18	2.396	0.554	7.260	3.344	6	6436	ZINC000011666641
6.4	-9.9	-9.4	0.24	3.213	0.263	6.315	1.158	6	6437	ZINC000118937397
6.4	-11.6	-11.2	0.22	2.254	0.929	5.119	2.273	6	6438	ZINC000257459425
6.4	-9.1	-8.9	0.09	3.017	0.321	6.937	1.680	6	6439	ZINC000059589161
6.4	-9.9	-9.4	0.41	2.228	0.628	4.151	2.114	6	6440	ZINC000257541040
6.4	-9.6	-8.9	0.34	3.281	0.165	7.271	0.819	6	6441	ZINC000040164568
6.4	-10.7	-10.1	0.26	2.636	0.275	5.539	1.865	6	6442	ZINC000014757137
6.4	-11.0	-10.5	0.24	2.366	0.372	6.025	1.610	6	6443	ZINC000100889840
6.4	-8.4	-8.3	0.09	2.390	0.417	5.691	2.558	6	6444	ZINC000100829891
6.4	-9.9	-9.5	0.21	1.976	0.410	5.893	1.721	6	6445	ZINC000000900910
6.4	-9.8	-9.0	0.47	1.821	0.471	5.485	1.745	6	6446	ZINC000257379841

6.4	-10.0	-9.5	0.24	3.204	1.070	6.732	3.590	6	6447	ZINC000005822376
6.4	-10.0	-9.9	0.11	2.563	0.319	5.556	0.511	6	6448	ZINC000085589153
6.4	-11.0	-10.7	0.19	2.188	0.299	6.638	1.647	6	6449	ZINC000238736614
6.4	-9.7	-9.5	0.11	2.427	0.321	6.777	2.308	6	6450	ZINC000118937211
6.4	-9.9	-9.5	0.24	1.818	0.316	4.920	2.837	6	6451	ZINC000257439639
6.4	-8.9	-8.6	0.18	1.963	0.284	5.636	3.531	6	6452	ZINC000014811815
6.4	-9.9	-9.4	0.27	2.506	0.568	6.478	2.565	6	6453	ZINC000257497574
6.4	-9.5	-9.4	0.09	3.694	1.072	6.536	2.048	6	6454	ZINC000261496044
6.4	-9.0	-8.8	0.15	2.299	0.824	5.199	2.732	6	6455	ZINC000013309939
6.4	-9.9	-9.6	0.16	2.376	0.092	5.303	1.379	6	6456	ZINC000014921508
6.4	-9.7	-9.3	0.26	2.132	0.410	8.634	3.040	6	6457	ZINC000036375370
6.4	-9.9	-9.1	0.40	2.282	0.720	5.780	1.703	6	6458	ZINC000100772909
6.4	-11.5	-11.0	0.28	2.799	0.389	6.369	0.489	6	6459	ZINC000085977710
6.4	-9.8	-9.5	0.25	2.015	0.331	3.927	0.412	6	6460	ZINC000118936840
6.4	-12.0	-11.3	0.48	3.244	0.253	5.915	1.313	6	6461	ZINC000257555290

6.4	-9.0	-8.8	0.09	2.525	0.549	5.767	2.276	6	6462	ZINC000015257529
6.4	-9.0	-8.6	0.28	2.845	0.114	5.905	1.481	6	6463	ZINC000257783875
6.3	-9.5	-9.1	0.19	2.109	0.806	4.305	2.417	6	6464	ZINC000257380054
6.3	-11.0	-10.5	0.38	2.166	0.408	4.592	1.864	6	6465	ZINC000257513122
6.3	-10.7	-10.5	0.16	2.744	0.120	7.000	1.445	6	6466	ZINC000059763775
6.3	-9.4	-9.0	0.30	3.464	0.344	7.229	1.623	6	6467	ZINC000100776828
6.3	-9.7	-8.8	0.48	2.534	0.320	5.967	0.611	6	6468	ZINC000100822478
6.3	-9.8	-9.7	0.15	2.363	0.469	6.083	0.716	6	6469	ZINC000013108877
6.3	-8.8	-8.5	0.22	2.291	0.728	5.185	2.641	6	6470	ZINC000257431553
6.3	-10.2	-9.9	0.30	3.165	1.201	5.309	2.060	6	6471	ZINC000003984116
6.3	-10.1	-9.8	0.20	2.028	0.311	5.971	1.820	6	6472	ZINC000257423421
6.3	-9.7	-9.5	0.10	2.513	0.372	5.815	2.065	6	6473	ZINC000118937208
6.3	-9.4	-9.1	0.23	3.381	0.423	6.735	1.494	6	6474	ZINC000100772064
6.3	-8.5	-8.4	0.11	1.933	1.034	4.289	2.459	6	6475	ZINC000013309933
6.3	-9.0	-8.9	0.11	2.221	0.605	6.415	2.521	6	6476	ZINC000100829913

6.3	-9.5	-9.2	0.21	2.645	1.072	5.632	3.375	6	6477	ZINC000004084618
6.3	-11.1	-10.4	0.37	2.823	0.459	8.363	2.177	6	6478	ZINC000257622071
6.3	-9.0	-8.9	0.13	2.094	0.486	6.164	2.720	6	6479	ZINC000100829910
6.3	-8.8	-8.6	0.12	2.344	0.226	5.912	2.075	6	6480	ZINC000015257521
6.3	-7.5	-7.2	0.15	1.908	0.564	5.795	3.184	6	6481	ZINC000001614080
6.3	-11.1	-10.5	0.31	2.192	0.345	7.275	2.404	6	6482	ZINC000238763329
6.3	-9.4	-9.1	0.23	3.377	0.420	6.729	1.501	6	6483	ZINC000100776831
6.3	-9.1	-8.9	0.08	3.054	0.533	4.908	2.206	6	6484	ZINC000014695523
6.3	-10.5	-9.7	0.54	2.917	0.406	5.821	0.862	6	6485	ZINC000013430427
6.3	-8.0	-7.9	0.10	2.946	0.424	6.284	2.568	6	6486	ZINC000034660471
6.3	-9.9	-9.6	0.21	3.676	0.281	5.861	0.962	6	6487	ZINC000096030148
6.3	-11.1	-10.5	0.29	2.388	0.420	4.582	2.561	6	6488	ZINC000100783777
6.3	-10.0	-9.5	0.30	2.353	0.241	5.151	1.960	6	6489	ZINC000012493582
6.3	-9.6	-9.3	0.21	2.072	0.587	5.029	2.088	6	6490	ZINC000003870056
6.3	-9.4	-8.8	0.32	2.891	0.748	6.013	1.035	6	6491	ZINC000005854773

6.3	-9.9	-9.6	0.20	2.696	0.806	4.844	1.461	6	6492	ZINC000006069773
6.3	-9.0	-8.8	0.12	2.521	0.272	5.348	2.091	6	6493	ZINC000257557271
6.3	-10.6	-10.3	0.21	2.095	0.149	4.839	1.946	6	6494	ZINC000100772857
6.3	-9.6	-9.2	0.18	1.886	0.505	4.639	2.794	6	6495	ZINC000261499075
6.3	-9.4	-9.2	0.17	3.061	0.841	6.018	2.557	6	6496	ZINC000100772058
6.3	-10.3	-9.4	0.42	2.066	0.453	6.949	0.658	6	6497	ZINC000257383046
6.3	-9.9	-9.4	0.24	2.515	0.549	5.411	2.144	6	6498	ZINC000013125764
6.3	-10.0	-9.7	0.25	3.597	0.519	7.879	1.960	6	6499	ZINC000261497817
6.3	-9.4	-9.1	0.23	3.382	0.422	6.413	1.894	6	6500	ZINC000100772068
6.3	-9.8	-9.3	0.30	3.313	0.552	6.049	1.739	6	6501	ZINC000015058239
6.3	-9.3	-9.2	0.11	3.781	0.602	7.609	1.574	6	6502	ZINC000261496043
6.3	-10.2	-9.8	0.34	2.028	0.909	4.185	2.487	6	6503	ZINC000257544599
6.3	-9.3	-9.2	0.13	2.745	0.415	5.597	2.119	6	6504	ZINC000085863140
6.3	-9.3	-9.0	0.18	2.422	0.859	4.014	2.079	6	6505	ZINC000204637797
6.3	-10.2	-9.8	0.21	2.487	0.484	4.433	2.037	6	6506	ZINC000257555735

6.3	-9.8	-9.7	0.12	3.008	0.281	4.323	0.765	6	6507	ZINC000257528532
6.3	-9.5	-9.1	0.21	2.801	0.799	5.454	2.182	6	6508	ZINC000030729880
6.3	-9.1	-9.0	0.10	2.045	0.585	7.258	2.236	6	6509	ZINC000013125765
6.3	-9.2	-8.8	0.22	2.533	0.659	6.434	1.273	6	6510	ZINC000015209927
6.3	-9.7	-9.4	0.19	3.542	0.441	5.873	2.307	6	6511	ZINC000015252160
6.3	-9.6	-9.1	0.25	3.144	0.632	4.981	1.328	6	6512	ZINC000257559339
6.3	-10.2	-9.6	0.44	2.390	0.308	5.812	0.540	6	6513	ZINC000059732385
6.3	-9.4	-9.1	0.23	3.378	0.422	6.714	1.505	6	6514	ZINC000100772062
6.3	-9.4	-9.0	0.24	3.483	0.300	6.521	1.798	6	6515	ZINC000100776834
6.3	-12.0	-11.2	0.54	2.607	0.176	6.180	0.966	6	6516	ZINC000261499280
6.3	-8.0	-7.8	0.09	2.476	0.626	5.861	3.139	6	6517	ZINC000034660469
6.3	-10.4	-9.8	0.36	2.730	0.473	6.137	0.917	6	6518	ZINC000015168933
6.3	-9.7	-9.5	0.11	2.506	0.367	5.819	2.057	6	6519	ZINC000118937210
6.3	-9.2	-9.0	0.19	2.298	0.661	7.245	3.393	6	6520	ZINC000012495605
6.3	-9.7	-9.5	0.11	2.558	0.401	5.873	2.025	6	6521	ZINC000257444909

6.3	-9.1	-9.0	0.07	2.210	0.685	4.850	2.715	6	6522	ZINC000085863160
6.2	-9.4	-9.3	0.11	1.944	0.403	5.721	2.672	6	6523	ZINC000100779148
6.2	-10.3	-9.8	0.30	2.309	0.614	6.016	2.410	6	6524	ZINC000257612464
6.2	-10.1	-9.8	0.17	3.165	0.367	8.609	0.507	6	6525	ZINC000257498124
6.2	-9.2	-8.8	0.20	2.536	0.661	6.433	1.275	6	6526	ZINC000257385998
6.2	-9.1	-8.8	0.15	2.268	0.586	4.815	2.515	6	6527	ZINC000255256195
6.2	-10.9	-10.1	0.37	2.679	0.398	6.043	1.736	6	6528	ZINC000238761666
6.2	-9.8	-9.4	0.23	2.160	0.399	5.425	1.012	6	6529	ZINC000257484770
6.2	-8.7	-8.3	0.36	2.341	0.859	4.261	3.232	6	6530	ZINC000085673589
6.2	-8.8	-8.6	0.12	3.035	0.193	6.878	0.599	6	6531	ZINC000014645919
6.2	-9.7	-9.5	0.11	2.291	1.067	7.397	0.231	6	6532	ZINC000014686696
6.2	-9.4	-9.3	0.14	2.215	0.572	5.051	2.521	6	6533	ZINC000100779146
6.2	-10.1	-9.6	0.27	2.718	0.809	6.295	2.734	6	6534	ZINC000257530289
6.2	-10.7	-10.5	0.15	2.345	0.416	6.227	2.585	6	6535	ZINC000100822023
6.2	-10.5	-9.6	0.54	3.597	0.222	6.248	0.846	6	6536	ZINC000006037481

6.2	-9.5	-9.2	0.23	2.437	0.313	6.682	1.424	6	6537	ZINC000257531900
6.2	-10.3	-10.0	0.21	2.590	0.244	6.822	1.638	6	6538	ZINC000257516676
6.2	-9.2	-8.8	0.23	2.301	0.449	5.499	1.882	6	6539	ZINC000257385996
6.2	-9.4	-9.2	0.12	2.482	0.403	6.828	0.733	6	6540	ZINC000257562365
6.2	-9.7	-8.8	0.45	2.514	0.435	6.475	1.913	6	6541	ZINC000100824591
6.2	-9.7	-9.5	0.14	2.314	0.527	8.199	1.823	6	6542	ZINC000040422816
6.2	-9.0	-8.8	0.17	1.999	0.719	6.074	2.918	6	6543	ZINC000257557272
6.2	-9.7	-9.5	0.18	1.802	0.448	3.355	0.990	6	6544	ZINC000118936838
6.2	-10.3	-9.8	0.22	2.890	0.448	5.094	1.818	6	6545	ZINC000095619632
6.2	-9.7	-9.4	0.27	2.928	0.530	6.138	1.755	6	6546	ZINC000100825546
6.2	-9.6	-9.2	0.19	2.993	1.207	5.497	2.664	6	6547	ZINC000006050191
6.2	-9.7	-9.6	0.05	1.844	0.605	6.184	2.889	6	6548	ZINC000257442785
6.2	-11.7	-10.8	0.46	2.251	0.516	6.320	2.553	6	6549	ZINC000100775859
6.2	-7.6	-7.4	0.12	2.369	0.608	4.405	2.690	6	6550	ZINC000030731379
6.2	-9.0	-8.8	0.19	1.864	0.562	7.305	2.643	6	6551	ZINC000257557273

6.2	-10.2	-9.4	0.41	3.212	0.290	6.662	0.655	6	6552	ZINC000015168936
6.2	-8.9	-8.7	0.15	2.184	0.759	7.523	2.998	6	6553	ZINC000014759032
6.2	-9.2	-8.8	0.21	2.418	0.702	6.659	1.198	6	6554	ZINC000100772681
6.2	-7.5	-7.4	0.04	3.221	0.387	5.908	2.182	6	6555	ZINC000014828306
6.2	-9.6	-9.5	0.18	2.238	0.851	5.651	2.955	6	6556	ZINC000095618008
6.2	-9.6	-9.3	0.16	3.064	0.160	6.579	2.703	6	6557	ZINC000257459855
6.2	-10.3	-10.1	0.14	3.018	0.348	5.757	1.042	6	6558	ZINC000118936841
6.2	-10.5	-10.1	0.20	2.073	0.109	6.632	1.015	6	6559	ZINC000100826876
6.1	-10.2	-10.1	0.11	2.554	0.553	5.692	2.380	6	6560	ZINC000257378885
6.1	-10.3	-9.9	0.21	2.849	0.414	6.427	3.046	6	6561	ZINC000100772054
6.1	-9.3	-8.9	0.23	3.104	0.516	7.018	1.732	6	6562	ZINC000257431554
6.1	-9.7	-9.4	0.23	2.632	0.811	5.572	2.968	6	6563	ZINC000257399923
6.1	-10.2	-10.1	0.09	2.645	0.211	6.710	0.852	6	6564	ZINC000100827209
6.1	-9.6	-9.2	0.25	2.592	0.599	6.523	1.448	6	6565	ZINC000257602910
6.1	-9.3	-9.2	0.10	3.011	0.773	4.801	1.533	6	6566	ZINC000257411657

6.1	-9.9	-9.7	0.15	2.589	0.530	9.252	2.812	6	6567	ZINC000015104410
6.1	-10.3	-10.0	0.15	2.744	0.533	5.372	2.975	6	6568	ZINC000100772052
6.1	-9.9	-9.5	0.23	1.758	0.385	4.698	3.032	6	6569	ZINC000015207575
6.1	-10.8	-10.0	0.39	3.167	0.079	6.366	0.287	6	6570	ZINC000100784083
6.1	-7.6	-7.3	0.17	2.175	0.239	6.111	2.279	6	6571	ZINC000002558160
6.1	-11.4	-10.7	0.33	2.301	0.653	5.810	2.554	6	6572	ZINC000257460139
6.1	-9.9	-9.5	0.22	1.997	0.377	5.925	2.887	6	6573	ZINC000015207578
6.1	-10.3	-9.8	0.22	2.812	0.468	4.810	1.451	6	6574	ZINC000059727321
6.1	-10.3	-9.8	0.22	2.893	0.451	5.097	1.821	6	6575	ZINC000100825275
6.1	-10.5	-9.6	0.80	1.625	0.169	4.426	0.490	6	6576	ZINC000064624409
6.1	-9.6	-9.0	0.29	2.367	0.358	5.788	3.115	6	6577	ZINC000014813649
6.1	-9.3	-9.0	0.21	1.997	0.203	5.951	0.159	6	6578	ZINC000014921505
6.1	-9.9	-9.5	0.20	2.667	0.545	7.804	2.866	6	6579	ZINC000012153178
6.1	-9.2	-8.8	0.20	2.395	0.667	6.585	1.156	6	6580	ZINC000100772680
6.1	-9.0	-8.7	0.16	4.110	0.520	6.992	1.966	6	6581	ZINC000015063088

6.1	-11.8	-11.5	0.17	2.407	0.260	7.435	2.252	6	6582	ZINC000100822026
6.1	-9.2	-9.0	0.17	2.453	0.187	5.463	1.000	6	6583	ZINC000100771562
6.1	-9.7	-9.3	0.25	2.293	0.375	6.225	0.940	6	6584	ZINC000257602943
6.1	-9.5	-9.3	0.16	2.995	0.637	3.953	0.903	6	6585	ZINC000005854718
6.1	-10.3	-10.0	0.15	2.729	0.541	5.371	2.986	6	6586	ZINC000257439993
6.1	-9.2	-8.8	0.21	2.556	0.694	6.506	1.323	6	6587	ZINC000257385997
6.1	-8.8	-8.6	0.17	1.901	0.535	6.559	3.616	6	6588	ZINC000014760470
6.1	-10.3	-9.8	0.22	2.889	0.439	5.102	1.816	6	6589	ZINC000100825278
6.1	-9.9	-9.7	0.17	2.268	0.716	5.615	2.855	6	6590	ZINC000014613244
6.1	-9.2	-8.8	0.19	2.255	0.210	5.777	1.426	6	6591	ZINC000100772258
6.1	-9.9	-9.4	0.25	2.039	0.388	5.805	2.734	6	6592	ZINC000257439638
6.1	-9.7	-9.1	0.29	2.131	0.526	6.658	2.129	6	6593	ZINC000257476747
6.1	-10.4	-9.9	0.32	2.205	0.539	4.789	2.261	6	6594	ZINC000000900914
6.1	-10.5	-10.4	0.09	1.745	0.829	5.795	2.815	6	6595	ZINC000100822459
6.1	-9.7	-9.2	0.27	3.073	0.797	6.411	3.287	6	6596	ZINC000014760544

6.1	-11.5	-10.7	0.45	2.056	0.090	4.445	1.181	6	6597	ZINC000253589773
6.1	-9.8	-9.3	0.29	2.693	0.676	6.327	2.109	6	6598	ZINC000014691746
6.1	-10.0	-9.3	0.33	2.209	1.034	5.294	3.429	6	6599	ZINC000004096712
6.1	-10.0	-9.5	0.29	1.714	0.512	4.769	1.104	6	6600	ZINC000100826880
6.1	-9.9	-9.5	0.22	1.824	0.361	4.915	2.861	6	6601	ZINC000257439640
6.1	-10.0	-9.7	0.24	2.397	0.656	5.086	3.000	6	6602	ZINC000257521372
6.1	-9.0	-8.8	0.12	2.325	0.305	6.622	0.618	6	6603	ZINC000085863077
6.1	-9.3	-8.7	0.29	2.215	0.415	6.898	0.332	6	6604	ZINC000257484771
6.1	-9.9	-9.4	0.24	1.893	0.409	4.831	2.789	6	6605	ZINC000015207573
6.1	-10.3	-9.8	0.27	3.115	0.324	6.282	0.836	6	6606	ZINC000095620500
6.1	-11.0	-10.4	0.27	2.135	0.621	5.875	1.596	6	6607	ZINC000086041866
6.1	-10.3	-10.0	0.15	2.568	0.471	4.091	2.435	6	6608	ZINC000100772055
6.1	-9.5	-9.2	0.18	2.517	0.745	5.534	2.700	6	6609	ZINC000014760593
6.1	-10.3	-9.8	0.22	2.813	0.464	4.810	1.447	6	6610	ZINC000085796910
6.1	-9.2	-8.9	0.25	2.017	0.280	5.588	3.055	6	6611	ZINC000257498513

6.1	-10.3	-9.8	0.23	2.894	0.449	5.097	1.818	6	6612	ZINC000059727293
6.1	-9.4	-9.4	0.04	2.160	0.388	6.029	2.778	4	6613	ZINC000257768740
6.1	-10.4	-9.6	0.39	2.744	1.128	4.619	2.387	6	6614	ZINC000257460908
6.1	-9.6	-9.2	0.33	2.461	0.597	6.380	2.315	6	6615	ZINC000013374485
6.1	-9.0	-8.5	0.22	2.311	0.438	7.583	2.398	6	6616	ZINC000238765926
6.1	-10.3	-10.0	0.15	2.728	0.524	5.356	2.979	6	6617	ZINC000257439992
6.0	-11.1	-10.6	0.26	1.854	0.285	3.886	0.759	6	6618	ZINC000100773176
6.0	-9.1	-8.3	0.48	2.619	0.628	6.324	2.416	6	6619	ZINC000004062194
6.0	-10.9	-10.7	0.18	1.913	0.565	4.535	2.526	6	6620	ZINC000257419088
6.0	-11.1	-10.6	0.29	1.854	0.276	3.872	0.745	6	6621	ZINC000100773178
6.0	-11.1	-10.6	0.28	1.649	0.358	3.652	0.734	6	6622	ZINC000100773183
6.0	-8.8	-8.4	0.25	2.394	0.348	7.152	1.117	6	6623	ZINC000085833888
6.0	-11.1	-10.6	0.29	1.853	0.278	3.863	0.780	6	6624	ZINC000257472027
6.0	-9.6	-9.5	0.17	2.222	0.322	5.520	2.259	6	6625	ZINC000257382539
6.0	-10.3	-10.0	0.14	2.874	0.424	6.735	2.800	6	6626	ZINC000257439995

6.0	-9.2	-9.0	0.25	3.211	0.524	6.314	1.425	6	6627	ZINC000257411660
6.0	-9.8	-9.2	0.32	2.556	0.375	5.852	1.260	6	6628	ZINC000014435216
6.0	-10.3	-10.0	0.16	2.843	0.408	5.676	2.723	6	6629	ZINC000257439994
6.0	-10.9	-9.9	0.69	1.985	0.490	4.171	1.709	6	6630	ZINC000136707317
6.0	-9.8	-9.5	0.24	2.362	0.514	7.287	2.389	6	6631	ZINC000253590162
6.0	-9.3	-9.2	0.11	3.199	0.878	5.697	1.997	6	6632	ZINC000100778552
6.0	-9.1	-9.0	0.07	2.186	0.263	5.473	2.155	6	6633	ZINC000100825806
6.0	-11.2	-10.7	0.26	1.938	0.188	6.318	0.784	6	6634	ZINC000257415730
6.0	-10.7	-10.4	0.19	2.211	0.255	6.295	1.858	6	6635	ZINC000100825176
6.0	-9.8	-9.4	0.27	2.974	0.196	6.544	2.867	6	6636	ZINC000100824507
6.0	-11.1	-10.6	0.29	1.844	0.282	3.862	0.790	6	6637	ZINC000100773186
6.0	-9.6	-9.2	0.19	3.079	0.468	6.440	1.729	6	6638	ZINC000013416199
6.0	-11.1	-10.8	0.19	2.536	0.087	5.524	0.620	6	6639	ZINC000257596863
6.0	-9.6	-9.2	0.25	1.940	0.179	6.230	2.676	6	6640	ZINC000257549393
6.0	-8.9	-8.5	0.26	3.574	1.154	6.858	2.473	6	6641	ZINC000013783866

6.0	-10.3	-10.1	0.20	2.321	0.756	5.776	2.996	6	6642	ZINC000085955321
6.0	-9.9	-9.7	0.14	2.001	0.257	7.335	3.574	6	6643	ZINC000100824384
6.0	-8.5	-8.4	0.06	1.472	0.780	5.303	2.601	6	6644	ZINC000008655654
6.0	-9.9	-9.3	0.31	2.087	0.640	5.947	2.693	6	6645	ZINC000257439637
6.0	-10.1	-9.5	0.31	2.415	0.684	6.664	3.197	6	6646	ZINC000028713627
6.0	-10.9	-10.5	0.24	2.517	0.399	5.606	2.325	6	6647	ZINC000257666337
6.0	-10.2	-9.7	0.32	2.671	0.345	4.907	1.052	6	6648	ZINC000040164534
6.0	-10.4	-9.8	0.26	1.947	0.511	3.317	1.503	6	6649	ZINC000257471569
6.0	-9.9	-9.7	0.11	1.714	0.407	5.019	3.632	6	6650	ZINC000095620855
6.0	-9.9	-9.5	0.25	2.053	1.089	4.333	2.796	6	6651	ZINC000257565507
6.0	-11.2	-10.7	0.37	1.933	0.483	4.605	1.424	6	6652	ZINC000118937117
6.0	-10.1	-9.7	0.20	2.456	0.563	6.460	2.183	6	6653	ZINC000257408373
6.0	-10.0	-9.7	0.22	1.490	0.664	4.330	0.319	6	6654	ZINC000038298811
6.0	-10.3	-10.0	0.23	1.796	0.570	4.198	2.271	6	6655	ZINC000257666368
6.0	-10.3	-10.0	0.13	2.382	0.127	7.974	1.717	6	6656	ZINC000100822025

6.0	-9.8	-9.3	0.29	3.517	0.609	5.745	1.165	6	6657	ZINC000014708292
6.0	-9.8	-9.5	0.29	2.583	0.359	6.357	0.189	6	6658	ZINC000100826515
6.0	-9.1	-9.0	0.13	1.737	0.186	4.616	2.322	6	6659	ZINC000013378555
6.0	-9.5	-9.4	0.14	1.979	0.200	6.252	2.760	6	6660	ZINC000257378884
6.0	-10.5	-9.8	0.37	1.912	0.655	4.202	2.993	6	6661	ZINC000257485997
6.0	-9.5	-9.3	0.12	2.224	0.723	7.216	3.134	6	6662	ZINC000257648690
6.0	-9.1	-8.9	0.14	2.294	0.640	5.224	2.203	6	6663	ZINC000014592517
6.0	-10.6	-9.7	0.52	1.888	0.755	5.519	1.948	6	6664	ZINC000013484752
6.0	-10.3	-10.0	0.16	2.730	0.430	6.364	3.194	6	6665	ZINC000100772050
6.0	-9.9	-9.5	0.33	2.012	0.935	4.333	2.775	6	6666	ZINC000257493865
6.0	-10.4	-9.7	0.49	2.321	0.180	7.093	1.358	6	6667	ZINC000100825718
6.0	-9.9	-9.5	0.24	1.837	0.326	4.800	2.729	6	6668	ZINC000015207580
6.0	-11.1	-10.6	0.28	1.983	0.461	4.081	1.027	6	6669	ZINC000257472029
6.0	-11.3	-10.0	0.61	3.585	0.614	5.328	1.130	6	6670	ZINC000095620856
6.0	-9.6	-9.1	0.36	3.152	0.860	7.549	0.189	6	6671	ZINC000006394168

6.0	-10.3	-9.8	0.23	3.625	0.308	5.849	0.837	6	6672	ZINC000257437411
6.0	-10.3	-10.2	0.09	2.174	0.210	7.055	0.108	6	6673	ZINC000119394955
6.0	-10.0	-9.8	0.21	2.194	0.696	4.302	2.025	6	6674	ZINC000013373234
5.9	-10.0	-9.4	0.32	2.256	0.290	7.160	1.291	6	6675	ZINC000257495605
5.9	-9.5	-9.2	0.18	2.842	0.284	6.535	2.306	6	6676	ZINC000015062954
5.9	-10.1	-9.5	0.27	2.221	0.669	4.971	1.430	6	6677	ZINC000257523876
5.9	-9.0	-8.6	0.24	2.849	0.631	5.472	1.601	6	6678	ZINC000257590405
5.9	-9.7	-9.3	0.21	2.255	0.406	5.882	0.676	6	6679	ZINC000100778084
5.9	-9.5	-9.2	0.13	2.945	0.549	7.192	2.056	6	6680	ZINC000257498748
5.9	-9.5	-9.3	0.13	3.313	0.653	7.007	2.399	6	6681	ZINC000257508762
5.9	-9.5	-9.4	0.13	2.615	1.262	5.068	2.463	6	6682	ZINC000257435527
5.9	-10.0	-9.7	0.16	2.361	0.529	7.222	2.444	6	6683	ZINC000100826224
5.9	-10.0	-9.5	0.42	3.365	1.195	5.872	2.939	6	6684	ZINC000005357595
5.9	-11.2	-11.0	0.21	2.788	0.480	5.838	1.619	6	6685	ZINC000085588759
5.9	-9.3	-9.0	0.15	2.789	0.266	7.898	3.427	6	6686	ZINC000013373888

5.9	-10.4	-10.0	0.30	2.595	0.626	6.767	1.069	6	6687	ZINC000085589047
5.9	-8.7	-8.6	0.07	2.754	0.531	7.513	2.323	6	6688	ZINC000257487951
5.9	-8.7	-8.5	0.09	1.898	0.521	5.258	3.452	6	6689	ZINC000257542989
5.9	-10.1	-9.5	0.29	2.726	0.740	6.932	2.917	6	6690	ZINC000253589638
5.9	-10.5	-9.4	0.67	2.353	0.551	5.557	0.977	6	6691	ZINC000257621274
5.9	-10.1	-9.9	0.11	2.609	0.267	5.866	1.374	6	6692	ZINC000257390557
5.9	-11.6	-10.8	0.41	2.163	0.833	5.522	2.682	6	6693	ZINC000014612726
5.9	-11.1	-10.7	0.21	2.378	0.348	5.051	1.876	6	6694	ZINC000100828740
5.9	-9.7	-9.5	0.17	3.354	0.123	8.423	1.824	6	6695	ZINC000257535029
5.9	-11.4	-11.1	0.19	2.993	0.590	4.211	1.111	6	6696	ZINC000257555289
5.9	-9.5	-9.4	0.10	2.328	0.704	4.557	2.868	6	6697	ZINC000022058370
5.9	-10.3	-9.9	0.25	2.279	0.407	5.043	1.897	6	6698	ZINC000257516674
5.9	-11.1	-10.5	0.40	2.883	0.709	5.419	1.785	6	6699	ZINC000038563014
5.9	-8.6	-8.5	0.07	2.233	0.583	5.338	1.893	6	6700	ZINC000100829890
5.9	-9.9	-9.4	0.27	1.996	0.567	4.168	1.257	6	6701	ZINC000014435220

5.9	-8.6	-8.5	0.11	1.594	0.540	5.393	2.924	6	6702	ZINC000256068536
5.9	-9.8	-9.7	0.07	2.688	0.254	7.662	1.753	6	6703	ZINC000100824419
5.9	-11.6	-10.6	0.48	1.971	0.325	5.241	1.660	6	6704	ZINC000014887093
5.9	-9.6	-9.4	0.20	2.481	0.650	5.048	2.901	6	6705	ZINC000257431092
5.9	-10.2	-9.9	0.26	1.732	0.619	3.608	2.152	6	6706	ZINC000100828718
5.9	-9.9	-9.5	0.22	2.745	0.262	6.013	0.494	6	6707	ZINC000257383048
5.9	-10.1	-9.8	0.21	2.725	0.728	4.848	2.289	6	6708	ZINC000014760590
5.9	-10.0	-9.5	0.32	2.416	0.958	5.507	2.701	6	6709	ZINC000257438069
5.9	-9.8	-9.5	0.16	2.687	0.992	6.546	2.926	6	6710	ZINC000257513023
5.9	-10.4	-10.1	0.24	3.355	0.530	6.115	1.075	6	6711	ZINC000257504569
5.9	-9.8	-9.4	0.29	2.800	0.612	5.993	3.518	6	6712	ZINC000257507080
5.9	-10.1	-9.6	0.39	1.986	0.332	6.589	3.268	6	6713	ZINC000257573782
5.9	-9.0	-8.8	0.12	2.986	0.598	6.447	1.594	6	6714	ZINC000257494861
5.9	-9.1	-8.7	0.22	2.991	0.519	6.228	1.811	6	6715	ZINC000014782112
5.9	-10.2	-9.9	0.28	1.829	0.653	4.441	2.115	6	6716	ZINC000100828722

5.9	-11.0	-10.5	0.34	2.452	0.558	6.582	0.739	6	6717	ZINC000257526398
5.9	-9.9	-9.5	0.21	2.622	1.288	3.883	1.312	6	6718	ZINC000013382494
5.9	-10.6	-9.9	0.50	2.163	0.429	4.926	0.768	6	6719	ZINC000013374486
5.9	-10.8	-9.5	0.61	2.478	0.552	5.445	2.834	6	6720	ZINC000257373530
5.9	-9.7	-9.0	0.33	3.412	0.368	4.657	0.942	6	6721	ZINC000257525457
5.9	-9.3	-9.1	0.14	2.841	0.645	6.915	2.240	6	6722	ZINC000257478192
5.9	-10.2	-9.8	0.33	1.770	0.658	4.434	2.105	6	6723	ZINC000257666370
5.9	-9.0	-8.7	0.24	2.118	0.522	4.628	1.520	6	6724	ZINC000257379840
5.9	-7.6	-7.5	0.14	3.119	1.843	8.476	3.030	6	6725	ZINC000095617908
5.9	-9.4	-9.2	0.16	2.081	0.476	4.379	2.387	6	6726	ZINC000257384362
5.9	-7.7	-7.4	0.18	3.687	0.542	4.889	0.810	6	6727	ZINC000012496188
5.9	-10.9	-10.3	0.29	2.152	0.387	4.371	1.689	6	6728	ZINC000257547464
5.8	-10.0	-9.8	0.14	2.871	0.458	5.090	1.823	6	6729	ZINC000257633499
5.8	-9.3	-9.2	0.08	1.987	1.003	7.264	3.054	6	6730	ZINC000014779851
5.8	-9.2	-8.9	0.20	2.724	1.007	5.064	2.208	6	6731	ZINC000256368967

5.8	-10.3	-9.8	0.26	2.836	0.363	5.969	1.364	6	6732	ZINC000257553795
5.8	-7.8	-7.5	0.18	1.882	0.279	5.623	2.628	6	6733	ZINC000001614086
5.8	-9.9	-9.6	0.21	2.448	0.542	4.580	1.577	6	6734	ZINC000004097719
5.8	-9.5	-9.3	0.14	2.480	0.478	7.578	2.159	6	6735	ZINC000015150595
5.8	-10.6	-10.3	0.23	2.684	0.070	6.136	1.777	6	6736	ZINC000257557712
5.8	-9.2	-8.9	0.17	2.582	0.737	5.646	2.470	6	6737	ZINC000256416871
5.8	-10.7	-10.2	0.30	2.213	0.731	6.377	2.538	6	6738	ZINC000014436719
5.8	-9.2	-8.9	0.18	2.679	0.367	4.316	0.488	6	6739	ZINC000257545876
5.8	-10.3	-10.0	0.36	2.042	0.142	6.893	0.373	6	6740	ZINC000257373611
5.8	-8.9	-8.8	0.08	2.326	0.223	6.303	2.025	6	6741	ZINC000257474186
5.8	-9.0	-8.8	0.15	3.074	0.161	4.912	0.293	6	6742	ZINC000085810308
5.8	-11.9	-11.7	0.20	2.108	0.723	3.977	1.553	6	6743	ZINC000257417336
5.8	-9.3	-9.0	0.14	3.447	0.302	7.791	0.217	6	6744	ZINC000257507304
5.8	-11.0	-10.4	0.32	2.142	0.381	5.140	1.623	6	6745	ZINC000100828730
5.8	-11.7	-11.0	0.43	2.542	0.842	5.705	1.279	6	6746	ZINC000095619884

5.8	-8.9	-8.8	0.09	2.253	0.218	7.043	1.771	6	6747	ZINC000257474185
5.8	-10.8	-10.1	0.39	2.084	0.645	5.194	2.032	6	6748	ZINC000014858267
5.8	-9.3	-9.0	0.23	1.950	1.130	4.549	3.003	6	6749	ZINC000014779842
5.8	-10.1	-10.1	0.05	2.421	0.616	8.278	3.088	6	6750	ZINC000100826865
5.8	-8.9	-8.4	0.30	2.335	0.702	5.590	1.813	6	6751	ZINC000100784294
5.8	-8.9	-8.8	0.08	2.325	0.225	6.303	2.030	6	6752	ZINC000100829787
5.8	-9.2	-9.1	0.06	1.835	0.455	5.348	2.563	6	6753	ZINC000014757227
5.8	-11.0	-9.9	0.52	2.322	0.383	4.920	1.303	6	6754	ZINC000014828690
5.8	-9.0	-8.8	0.11	2.977	0.413	5.045	0.981	6	6755	ZINC000014504952
5.8	-10.4	-10.2	0.12	2.441	0.614	6.528	1.158	6	6756	ZINC000003644256
5.8	-9.1	-8.8	0.26	2.477	0.639	6.363	1.900	6	6757	ZINC000257704827
5.8	-9.4	-9.0	0.23	2.637	0.178	6.350	2.386	6	6758	ZINC000014647287
5.8	-9.4	-9.0	0.33	1.934	0.460	4.497	3.011	6	6759	ZINC000014811791
5.8	-10.2	-10.0	0.19	2.014	0.696	4.302	2.131	6	6760	ZINC000257405261
5.8	-9.8	-9.2	0.41	1.797	0.542	4.158	1.274	6	6761	ZINC000100821969

5.8	-9.5	-9.2	0.18	2.425	0.365	6.094	2.261	6	6762	ZINC000257531898
5.8	-9.9	-9.6	0.32	1.656	0.602	3.729	1.442	6	6763	ZINC000100828071
5.8	-9.7	-9.3	0.24	2.291	0.342	6.425	0.626	6	6764	ZINC000257602944
5.8	-9.9	-9.7	0.24	1.923	0.691	5.233	2.262	6	6765	ZINC000100828073
5.8	-10.5	-9.9	0.35	2.249	0.388	6.166	0.441	6	6766	ZINC000014858808
5.8	-9.5	-9.2	0.28	2.232	0.412	5.155	2.232	6	6767	ZINC000257377357
5.8	-8.5	-8.3	0.07	2.124	0.210	6.058	2.092	6	6768	ZINC000095912863
5.8	-9.5	-9.1	0.24	2.442	0.587	5.646	2.065	6	6769	ZINC000005919482
5.8	-9.4	-9.3	0.09	2.450	0.117	4.950	1.585	6	6770	ZINC000257615284
5.8	-9.0	-8.7	0.17	1.985	0.202	7.634	1.273	6	6771	ZINC000085833884
5.8	-10.3	-9.5	0.39	2.900	0.589	5.944	2.890	6	6772	ZINC000040164388
5.8	-9.9	-9.4	0.26	2.588	0.722	6.453	2.093	6	6773	ZINC000015168938
5.8	-9.7	-9.4	0.27	2.799	0.563	5.161	2.205	6	6774	ZINC000014708286
5.8	-10.1	-9.7	0.30	2.210	0.246	4.869	2.371	6	6775	ZINC000257458913
5.8	-10.4	-10.0	0.25	1.820	0.536	4.939	1.296	6	6776	ZINC000013378362

5.8	-9.3	-8.9	0.26	2.534	0.299	5.322	2.344	6	6777	ZINC000014680913
5.8	-9.5	-9.1	0.29	3.040	0.620	5.709	1.062	6	6778	ZINC000100782664
5.8	-9.8	-9.7	0.11	1.653	0.162	5.210	2.418	6	6779	ZINC000014647190
5.8	-9.5	-9.3	0.17	2.107	0.313	6.014	2.397	6	6780	ZINC000257530291
5.8	-9.3	-9.1	0.17	2.018	0.295	5.175	2.419	6	6781	ZINC000261495756
5.8	-10.2	-9.8	0.26	3.406	0.830	6.038	1.515	6	6782	ZINC000261498133
5.8	-10.7	-10.5	0.18	1.901	0.589	3.979	2.373	6	6783	ZINC000257493212
5.8	-10.5	-9.7	0.37	2.633	0.951	4.627	2.224	6	6784	ZINC000100257573
5.8	-9.7	-9.2	0.30	2.084	0.322	6.353	2.433	6	6785	ZINC000257544596
5.8	-9.5	-8.9	0.36	2.503	0.256	6.607	0.796	6	6786	ZINC000100827232
5.8	-9.0	-8.6	0.30	2.172	0.647	4.831	2.549	6	6787	ZINC000257548388
5.8	-9.3	-8.8	0.25	2.362	0.554	5.630	2.085	6	6788	ZINC000014727590
5.8	-10.4	-10.2	0.09	1.678	0.360	4.293	2.418	6	6789	ZINC000100777799
5.7	-8.9	-8.4	0.26	2.176	1.029	4.825	2.997	6	6790	ZINC000006393492
5.7	-11.2	-10.4	0.40	1.938	0.502	5.814	2.908	6	6791	ZINC000029229735

5.7	-9.4	-8.6	0.41	2.336	0.315	5.580	1.691	6	6792	ZINC000261495755
5.7	-10.0	-9.8	0.09	3.306	0.657	5.692	1.678	6	6793	ZINC000253609252
5.7	-9.3	-9.1	0.16	2.052	0.355	5.844	2.996	6	6794	ZINC000118928393
5.7	-11.4	-11.1	0.18	2.750	0.523	4.021	1.199	6	6795	ZINC000257555291
5.7	-9.5	-9.3	0.11	3.219	0.693	7.826	1.952	6	6796	ZINC000257508761
5.7	-7.4	-7.2	0.12	1.880	0.447	5.459	3.105	6	6797	ZINC000085910445
5.7	-10.8	-10.2	0.28	2.035	0.614	4.824	2.996	6	6798	ZINC000257497571
5.7	-9.7	-9.3	0.22	3.804	0.455	6.111	0.847	6	6799	ZINC000100825153
5.7	-9.3	-9.0	0.25	2.929	0.816	5.267	1.548	6	6800	ZINC000015257558
5.7	-10.0	-9.9	0.11	3.272	0.416	7.457	1.990	6	6801	ZINC000257511056
5.7	-10.4	-9.9	0.32	2.327	1.124	4.879	3.014	6	6802	ZINC000257399921
5.7	-10.4	-9.8	0.29	3.181	0.450	6.008	0.674	6	6803	ZINC000118936889
5.7	-9.4	-9.2	0.13	2.161	0.417	6.948	3.321	6	6804	ZINC000095620825
5.7	-9.2	-9.0	0.19	1.776	0.775	4.701	1.403	6	6805	ZINC000140695327
5.7	-9.0	-8.7	0.16	2.389	0.515	7.071	2.427	6	6806	ZINC000100771842

5.7	-10.1	-9.7	0.25	2.560	0.628	6.399	2.376	6	6807	ZINC000257439655
5.7	-9.9	-9.5	0.42	2.216	0.534	4.888	1.687	6	6808	ZINC000257459232
5.7	-9.0	-8.8	0.10	2.435	0.486	6.522	2.639	6	6809	ZINC000014727569
5.7	-10.2	-9.7	0.34	2.755	0.518	7.456	3.241	6	6810	ZINC000014807644
5.7	-9.7	-9.2	0.26	2.310	0.351	6.438	0.656	6	6811	ZINC000100778082
5.7	-10.0	-9.9	0.11	3.378	0.254	7.461	2.013	6	6812	ZINC000100825001
5.7	-9.7	-9.3	0.30	3.134	0.594	8.059	2.500	6	6813	ZINC000257485774
5.7	-10.0	-9.7	0.15	2.850	0.523	7.846	2.686	6	6814	ZINC000257511055
5.7	-10.6	-10.3	0.16	3.624	0.854	5.895	1.685	6	6815	ZINC000257416820
5.7	-9.6	-9.1	0.40	3.072	0.976	5.544	1.753	6	6816	ZINC000257702855
5.7	-9.5	-8.9	0.26	2.559	0.395	5.234	1.943	6	6817	ZINC000261498642
5.7	-10.8	-10.5	0.17	3.321	0.719	5.220	1.382	6	6818	ZINC000100773312
5.7	-9.5	-9.0	0.37	2.891	0.564	6.547	1.451	6	6819	ZINC000238779152
5.7	-10.1	-9.6	0.26	3.098	0.766	6.174	1.036	6	6820	ZINC000014502285
5.7	-9.1	-8.6	0.28	2.308	0.581	6.869	2.154	6	6821	ZINC000100826510

5.7	-10.2	-10.2	0.08	2.361	0.442	4.803	2.257	6	6822	ZINC000257453476
5.7	-10.0	-9.7	0.21	2.045	0.208	5.929	2.113	6	6823	ZINC000257405263
5.7	-9.3	-9.0	0.20	2.840	0.858	6.081	2.632	6	6824	ZINC000040164308
5.7	-9.5	-9.2	0.24	2.136	0.152	6.805	1.330	6	6825	ZINC000253613402
5.7	-9.6	-9.4	0.12	2.061	0.690	3.757	2.520	6	6826	ZINC000253589677
5.7	-8.7	-8.3	0.25	1.841	0.299	3.835	1.325	6	6827	ZINC000085833876
5.7	-10.0	-9.7	0.17	1.633	0.425	3.637	1.290	6	6828	ZINC000257666367
5.7	-9.3	-9.2	0.14	2.917	0.669	6.053	2.624	6	6829	ZINC000257462238
5.7	-10.7	-9.8	0.51	2.778	0.426	5.919	1.008	6	6830	ZINC000015210126
5.7	-10.1	-9.8	0.16	2.653	0.196	6.346	2.415	6	6831	ZINC000257560117
5.7	-10.1	-9.8	0.15	2.620	0.192	6.293	2.404	6	6832	ZINC000118937155
5.7	-10.0	-9.9	0.11	2.280	1.289	5.346	3.570	6	6833	ZINC000253609253
5.7	-10.2	-9.9	0.23	2.399	0.486	7.092	2.956	6	6834	ZINC000257459856
5.7	-8.9	-8.5	0.28	2.958	0.971	6.143	1.859	6	6835	ZINC000040165474
5.7	-10.5	-10.3	0.10	1.967	0.671	5.076	2.224	6	6836	ZINC000257459424

5.7	-10.7	-10.5	0.16	2.179	0.807	5.072	2.606	6	6837	ZINC000100827958
5.7	-9.6	-9.5	0.11	2.502	0.606	7.524	2.784	6	6838	ZINC000257506116
5.7	-9.1	-8.9	0.15	3.347	0.711	7.481	0.467	6	6839	ZINC000100827476
5.7	-10.5	-10.1	0.20	2.572	0.763	5.887	0.762	6	6840	ZINC000013373244
5.7	-11.1	-10.1	0.45	3.103	0.709	5.685	2.260	6	6841	ZINC000059735698
5.7	-10.0	-9.8	0.11	3.147	0.316	8.329	1.621	6	6842	ZINC000257511057
5.7	-9.3	-9.2	0.15	2.214	0.897	6.067	3.285	6	6843	ZINC000257507305
5.7	-8.9	-8.5	0.22	2.208	0.650	5.686	2.634	6	6844	ZINC000015268299
5.7	-10.4	-9.7	0.35	1.783	0.734	4.605	2.336	6	6845	ZINC000013461268
5.7	-11.9	-11.7	0.27	2.564	0.695	5.880	0.538	6	6846	ZINC000257417334
5.7	-10.0	-9.8	0.14	3.342	0.297	7.546	2.109	6	6847	ZINC000100824999
5.7	-10.8	-10.5	0.18	2.855	0.794	4.797	1.519	6	6848	ZINC000104352764
5.7	-9.7	-9.4	0.19	2.484	0.744	6.563	2.195	6	6849	ZINC000015168931
5.7	-9.8	-9.2	0.28	3.024	0.203	6.708	0.870	6	6850	ZINC000040165472
5.7	-9.7	-9.5	0.19	3.417	0.545	6.786	1.293	6	6851	ZINC000257505605

5.7	-10.3	-9.8	0.24	2.617	0.774	5.128	2.348	6	6852	ZINC000034326242
5.7	-10.8	-10.5	0.23	3.481	0.623	5.676	1.232	6	6853	ZINC000104352760
5.7	-10.1	-10.0	0.08	2.478	0.337	7.241	0.768	6	6854	ZINC000100830134
5.7	-10.4	-9.6	0.54	2.882	0.717	5.169	1.294	6	6855	ZINC000100257569
5.7	-10.1	-9.6	0.38	2.030	0.217	4.816	1.490	6	6856	ZINC000257375845
5.7	-10.0	-9.8	0.13	3.110	0.466	7.550	2.090	6	6857	ZINC000257511054
5.7	-10.1	-9.9	0.11	3.375	0.640	5.692	1.673	6	6858	ZINC000034951215
5.7	-9.1	-8.7	0.30	2.585	0.909	6.237	0.960	6	6859	ZINC000033831437
5.7	-9.7	-9.3	0.27	2.558	0.899	5.559	2.931	6	6860	ZINC000012153134
5.7	-10.0	-9.6	0.35	2.139	0.878	3.126	0.956	6	6861	ZINC000100825004
5.7	-10.1	-9.8	0.16	3.159	0.676	6.466	1.129	6	6862	ZINC000118937339
5.7	-9.9	-9.6	0.16	2.263	0.869	4.045	2.293	6	6863	ZINC000257378778
5.7	-7.6	-7.3	0.11	4.780	0.562	7.455	1.908	6	6864	ZINC000003831417
5.6	-9.3	-8.8	0.30	2.103	0.712	6.106	1.881	6	6865	ZINC000059766546
5.6	-10.4	-9.3	0.53	3.291	0.576	6.457	1.397	6	6866	ZINC000013430430

5.6	-10.3	-9.8	0.33	2.419	0.738	4.725	1.930	6	6867	ZINC000257399922
5.6	-9.2	-8.6	0.42	2.525	0.476	4.907	1.025	6	6868	ZINC000257485545
5.6	-10.0	-9.6	0.21	2.845	0.380	6.140	1.142	6	6869	ZINC000257498125
5.6	-9.9	-9.6	0.26	1.894	0.469	4.185	1.801	6	6870	ZINC000100828717
5.6	-9.7	-9.2	0.26	2.501	0.249	5.710	1.580	6	6871	ZINC000261495757
5.6	-10.6	-10.1	0.52	2.271	0.452	6.274	0.742	6	6872	ZINC000056874789
5.6	-8.7	-8.5	0.13	3.000	0.415	7.073	0.609	6	6873	ZINC000085833849
5.6	-10.5	-9.8	0.38	2.139	0.803	5.984	0.864	6	6874	ZINC000257459510
5.6	-10.5	-10.2	0.16	2.269	0.170	4.210	0.615	6	6875	ZINC000100825178
5.6	-11.1	-9.8	0.61	2.698	0.740	5.675	0.670	6	6876	ZINC000238757829
5.6	-10.0	-9.6	0.26	2.343	0.656	4.497	2.608	6	6877	ZINC000257486000
5.6	-10.5	-9.8	0.35	1.933	0.324	5.208	1.658	6	6878	ZINC000014921500
5.6	-9.9	-9.5	0.22	2.053	0.393	4.553	2.041	6	6879	ZINC000257666369
5.6	-9.6	-9.3	0.16	2.891	0.280	7.326	2.800	6	6880	ZINC000059728289
5.6	-8.8	-8.5	0.15	2.962	1.061	5.129	3.156	6	6881	ZINC000014762761

5.6	-11.3	-10.5	0.45	2.676	0.482	5.995	0.875	6	6882	ZINC000013307621
5.6	-9.5	-9.3	0.13	3.234	0.700	7.837	1.970	6	6883	ZINC000100778450
5.6	-9.9	-9.8	0.11	3.134	0.807	6.757	2.538	6	6884	ZINC000100825002
5.6	-9.1	-8.9	0.09	2.161	0.458	6.933	3.460	6	6885	ZINC000038940470
5.6	-11.2	-10.9	0.21	2.392	0.591	6.506	1.240	6	6886	ZINC000106385146
5.6	-9.6	-9.3	0.14	1.998	0.439	5.122	2.376	6	6887	ZINC000257430037
5.6	-10.5	-10.2	0.26	2.242	0.683	4.376	2.064	6	6888	ZINC000118936854
5.6	-8.7	-8.5	0.11	2.367	0.670	5.377	1.919	6	6889	ZINC000257706791
5.6	-9.7	-9.1	0.27	2.314	0.352	6.578	0.846	6	6890	ZINC000238778596
5.6	-9.5	-8.9	0.33	2.369	0.232	8.495	0.256	6	6891	ZINC000100827831
5.6	-10.2	-9.8	0.33	1.787	0.651	5.448	3.200	6	6892	ZINC000257373528
5.6	-9.9	-9.7	0.26	1.772	0.858	4.087	1.663	6	6893	ZINC000100828070
5.6	-9.3	-9.0	0.24	2.775	0.834	5.966	2.684	6	6894	ZINC000119383480
5.6	-9.2	-8.6	0.35	2.921	0.482	6.145	1.314	6	6895	ZINC000261498596
5.6	-11.7	-10.8	0.64	2.359	0.694	6.796	2.595	6	6896	ZINC000014612727

5.6	-9.4	-9.1	0.15	1.889	0.593	2.811	0.697	6	6897	ZINC000014757346
5.6	-9.6	-8.9	0.36	2.504	0.236	7.134	0.401	6	6898	ZINC000257430034
5.6	-9.7	-9.5	0.12	2.557	0.313	4.740	2.528	6	6899	ZINC000000899800
5.6	-9.4	-9.3	0.11	2.444	0.541	5.985	1.039	6	6900	ZINC000257413123
5.6	-10.1	-9.5	0.29	2.728	0.550	6.054	2.300	6	6901	ZINC000257458217
5.6	-8.9	-8.6	0.19	1.746	0.145	4.422	2.319	6	6902	ZINC000015148365
5.6	-9.9	-9.4	0.24	2.157	0.761	6.909	3.837	6	6903	ZINC000014811759
5.6	-11.0	-10.0	0.55	1.977	0.535	6.087	2.149	6	6904	ZINC000013484755
5.6	-10.0	-9.3	0.31	3.069	0.930	5.763	2.423	6	6905	ZINC000030729894
5.6	-11.0	-10.3	0.36	2.807	0.489	5.787	1.509	6	6906	ZINC000100257577
5.6	-10.1	-9.9	0.21	2.378	0.841	4.680	2.615	6	6907	ZINC000012153136
5.6	-10.5	-10.2	0.26	2.570	0.955	5.392	2.507	6	6908	ZINC000118936855
5.6	-10.4	-9.6	0.36	3.033	0.336	5.884	0.768	6	6909	ZINC000257377355
5.6	-10.0	-9.7	0.31	1.989	0.785	5.200	2.248	6	6910	ZINC000257559419
5.6	-8.3	-8.0	0.15	3.401	0.326	6.063	1.700	6	6911	ZINC000256166394

5.6	-10.5	-10.3	0.13	2.082	0.112	5.722	2.586	6	6912	ZINC000100825477
5.6	-10.0	-9.1	0.47	3.261	0.416	5.883	0.954	6	6913	ZINC000118937342
5.6	-8.7	-8.2	0.24	2.266	0.288	5.714	1.376	6	6914	ZINC000238735766
5.6	-10.5	-10.2	0.26	2.246	0.690	4.374	2.060	6	6915	ZINC000118936857
5.6	-9.9	-9.6	0.25	1.586	0.643	4.189	2.007	6	6916	ZINC000257559421
5.6	-9.4	-9.0	0.24	1.769	0.693	5.897	1.795	6	6917	ZINC000031494948
5.6	-9.9	-9.5	0.28	2.670	0.798	6.293	3.040	6	6918	ZINC000257416817
5.6	-10.6	-9.8	0.45	2.899	0.355	7.151	1.454	6	6919	ZINC000257551109
5.6	-10.5	-10.2	0.26	2.337	0.714	4.283	2.114	6	6920	ZINC000118936856
5.6	-9.8	-9.7	0.09	1.765	0.704	7.459	3.482	6	6921	ZINC000014779848
5.6	-9.7	-9.2	0.29	2.844	0.992	5.653	1.763	6	6922	ZINC000040165458
5.6	-10.5	-10.2	0.26	2.268	0.673	4.413	2.039	6	6923	ZINC000257438068
5.6	-10.4	-9.1	0.59	2.608	0.200	6.092	1.876	6	6924	ZINC000257446876
5.6	-7.4	-7.2	0.09	2.873	0.622	6.289	2.822	6	6925	ZINC000015271796
5.5	-9.9	-9.7	0.15	1.986	0.306	3.775	1.801	6	6926	ZINC000059766553

5.5	-10.2	-9.8	0.21	2.159	0.816	4.547	3.081	6	6927	ZINC000257553793
5.5	-11.1	-10.7	0.21	2.242	0.783	5.381	2.052	6	6928	ZINC000100826926
5.5	-10.6	-9.7	0.39	2.448	0.388	5.931	1.279	6	6929	ZINC000257564221
5.5	-10.0	-9.9	0.11	2.547	0.657	5.160	2.324	6	6930	ZINC000118936943
5.5	-9.6	-9.2	0.30	3.518	0.998	7.811	2.687	6	6931	ZINC000257460304
5.5	-9.2	-9.0	0.20	2.788	0.831	5.997	2.661	6	6932	ZINC000119383209
5.5	-10.4	-9.7	0.58	2.491	0.590	5.016	1.826	6	6933	ZINC000261497252
5.5	-10.1	-9.9	0.12	2.292	0.413	4.870	2.140	6	6934	ZINC000013374456
5.5	-10.0	-9.7	0.26	2.231	0.772	4.168	2.377	6	6935	ZINC000257506113
5.5	-8.0	-7.7	0.18	1.980	0.183	5.992	2.006	6	6936	ZINC000014709355
5.5	-8.7	-8.5	0.11	2.125	0.524	6.102	2.471	6	6937	ZINC000257706792
5.5	-9.1	-8.6	0.26	3.196	0.618	6.435	2.694	6	6938	ZINC000000899894
5.5	-9.0	-8.8	0.11	2.853	0.898	6.343	2.313	6	6939	ZINC000096095406
5.5	-9.6	-9.2	0.27	3.746	0.830	7.844	2.250	6	6940	ZINC000257460305
5.5	-8.7	-8.5	0.13	2.296	0.626	5.353	1.954	6	6941	ZINC000257706793

5.5	-9.5	-9.3	0.11	3.317	0.651	7.010	2.392	6	6942	ZINC000257508764
5.5	-10.3	-9.8	0.28	2.637	0.237	7.040	0.781	6	6943	ZINC000100828907
5.5	-9.8	-9.6	0.11	2.160	0.556	5.391	2.425	6	6944	ZINC000118937104
5.5	-11.0	-10.2	0.44	3.049	0.506	7.157	1.167	6	6945	ZINC000015150593
5.5	-10.3	-9.7	0.31	2.185	0.772	5.247	3.254	6	6946	ZINC000257476396
5.5	-9.2	-9.1	0.07	2.564	1.031	6.085	2.775	6	6947	ZINC000012153181
5.5	-9.3	-9.2	0.12	3.073	0.867	4.869	1.558	6	6948	ZINC000100778554
5.5	-10.2	-10.0	0.11	3.224	0.431	4.739	0.735	6	6949	ZINC000118936931
5.5	-10.0	-9.6	0.21	2.601	0.302	5.868	0.895	6	6950	ZINC000257539987
5.5	-9.2	-8.9	0.22	1.764	0.924	3.871	2.911	6	6951	ZINC000253497582
5.5	-9.0	-8.8	0.15	2.852	0.447	6.155	2.064	6	6952	ZINC000257402550
5.5	-11.4	-10.9	0.26	2.720	1.010	6.767	0.954	6	6953	ZINC000095619882
5.5	-9.6	-9.1	0.23	2.057	0.545	4.766	2.186	6	6954	ZINC000118913642
5.5	-9.7	-9.3	0.24	2.286	0.325	6.382	1.009	6	6955	ZINC000100778081
5.5	-9.2	-8.8	0.22	3.919	0.966	6.277	2.475	6	6956	ZINC000015262054

5.5	-9.2	-8.9	0.22	2.848	0.820	6.061	2.538	6	6957	ZINC000119383618
5.5	-9.1	-8.9	0.21	2.227	0.213	5.864	1.737	6	6958	ZINC000100771616
5.5	-10.5	-9.9	0.29	2.415	0.225	6.814	1.842	6	6959	ZINC000100829052
5.5	-10.4	-9.8	0.34	2.294	0.460	5.431	1.191	6	6960	ZINC000257477342
5.5	-9.2	-9.0	0.17	2.333	0.313	6.296	1.474	6	6961	ZINC000100772677
5.5	-10.0	-9.8	0.18	1.966	0.516	5.749	2.799	6	6962	ZINC000257736995
5.5	-10.1	-9.5	0.27	2.430	0.553	5.324	2.008	6	6963	ZINC000257507306
5.5	-10.3	-9.7	0.41	2.142	0.511	4.479	1.589	6	6964	ZINC000257375889
5.5	-10.8	-10.5	0.19	2.658	0.628	6.408	1.369	6	6965	ZINC000100776538
5.5	-8.5	-8.2	0.17	1.930	0.608	3.661	1.389	6	6966	ZINC000085833877
5.5	-10.9	-10.4	0.26	1.782	0.375	3.806	1.936	6	6967	ZINC000100825722
5.5	-8.7	-8.5	0.13	1.805	0.582	5.596	2.121	6	6968	ZINC000257379843
5.5	-9.2	-9.1	0.07	2.232	0.484	6.578	3.223	6	6969	ZINC000257472414
5.5	-8.8	-8.3	0.32	2.123	0.255	6.176	0.302	6	6970	ZINC000257377987
5.5	-9.8	-9.7	0.10	2.107	0.640	5.350	2.473	6	6971	ZINC000118937103

5.5	-9.8	-9.1	0.45	2.271	0.477	4.755	1.684	6	6972	ZINC000085847327
5.5	-10.0	-9.9	0.11	2.337	0.811	5.051	2.378	6	6973	ZINC000118936944
5.5	-9.8	-9.6	0.12	1.930	0.484	4.405	2.936	6	6974	ZINC000070455474
5.5	-8.7	-8.6	0.09	1.902	0.881	4.917	2.386	6	6975	ZINC000257706790
5.5	-10.3	-9.9	0.22	1.677	0.852	4.852	2.894	6	6976	ZINC000257435155
5.5	-8.7	-8.5	0.15	2.721	0.259	4.518	0.232	6	6977	ZINC000100829889
5.5	-10.5	-10.1	0.26	1.969	0.562	5.259	2.072	6	6978	ZINC000100824984
5.5	-9.8	-9.6	0.11	1.929	0.392	5.300	2.508	6	6979	ZINC000256457385
5.5	-11.1	-10.8	0.22	2.476	0.262	7.678	1.509	6	6980	ZINC000100828744
5.5	-9.2	-8.9	0.18	2.809	1.230	5.522	3.400	6	6981	ZINC000118936827
5.5	-10.1	-9.4	0.31	2.904	0.592	6.160	1.956	6	6982	ZINC000118937354
5.5	-9.2	-9.1	0.07	2.751	0.435	6.980	2.136	6	6983	ZINC000013409111
5.5	-8.6	-8.3	0.21	2.484	0.544	3.174	0.677	6	6984	ZINC000004097700
5.5	-10.9	-10.4	0.26	2.197	0.484	5.509	2.424	6	6985	ZINC000257541175
5.5	-9.6	-9.2	0.29	3.340	0.686	6.912	2.640	6	6986	ZINC000257460307

5.5	-9.8	-9.6	0.12	2.060	0.444	5.532	2.343	6	6987	ZINC000256457389
5.5	-9.6	-8.6	0.54	2.447	0.432	5.276	2.705	6	6988	ZINC000014727969
5.5	-9.5	-9.2	0.18	1.528	0.529	4.755	2.466	6	6989	ZINC000014647188
5.5	-11.5	-10.9	0.29	1.978	0.643	5.716	1.802	6	6990	ZINC000086043948
5.5	-11.4	-10.9	0.23	2.340	0.877	4.069	1.772	6	6991	ZINC000030731319
5.5	-8.6	-8.2	0.21	2.130	0.445	5.329	1.411	6	6992	ZINC000085863095
5.5	-9.8	-9.6	0.14	2.347	0.779	4.157	2.260	6	6993	ZINC000257378780
5.4	-10.9	-10.5	0.26	2.411	0.286	5.754	2.073	6	6994	ZINC000100822462
5.4	-8.9	-8.6	0.19	2.232	1.135	6.804	2.496	6	6995	ZINC000004098750
5.4	-9.6	-9.5	0.13	2.720	0.719	6.063	2.448	6	6996	ZINC000257783877
5.4	-9.9	-9.0	0.47	2.473	0.266	5.884	0.313	6	6997	ZINC000085883673
5.4	-8.6	-8.4	0.15	2.578	0.623	5.322	2.617	6	6998	ZINC000014762969
5.4	-8.6	-8.4	0.11	3.558	0.670	7.970	1.166	6	6999	ZINC000013783869
5.4	-9.7	-9.6	0.11	3.372	0.632	5.681	1.723	6	7000	ZINC000034951217
5.4	-9.7	-9.6	0.08	2.009	0.409	5.293	2.498	6	7001	ZINC000118937105

5.4	-9.3	-8.9	0.21	2.758	0.571	5.475	1.900	6	7002	ZINC000015257511
5.4	-9.2	-8.9	0.19	2.212	0.688	7.848	3.069	6	7003	ZINC000012153682
5.4	-10.0	-9.8	0.13	2.294	0.580	5.503	3.227	6	7004	ZINC000014436728
5.4	-9.5	-9.2	0.26	3.762	0.650	7.991	2.347	6	7005	ZINC000257460306
5.4	-11.3	-11.0	0.28	1.856	0.176	5.038	1.473	6	7006	ZINC000100825237
5.4	-9.5	-8.8	0.35	2.573	0.803	6.186	1.045	6	7007	ZINC000015257540
5.4	-11.0	-10.7	0.20	1.619	0.494	3.374	1.890	6	7008	ZINC000100827962
5.4	-9.5	-9.0	0.26	2.461	1.046	4.764	2.168	6	7009	ZINC000030729890
5.4	-10.7	-10.5	0.10	2.317	0.304	6.332	2.380	6	7010	ZINC000100825177
5.4	-9.7	-9.6	0.07	2.244	1.241	4.496	2.831	6	7011	ZINC000253609254
5.4	-9.7	-9.5	0.10	2.306	0.492	6.369	2.435	6	7012	ZINC000118937102
5.4	-9.3	-8.6	0.32	2.303	0.566	4.736	1.936	6	7013	ZINC000030730049
5.4	-9.9	-9.2	0.35	2.069	0.291	6.405	2.629	6	7014	ZINC000014709125
5.4	-9.6	-9.4	0.13	2.065	0.632	5.153	2.833	6	7015	ZINC000257530280
5.4	-9.5	-8.9	0.33	3.038	0.606	6.953	2.017	6	7016	ZINC000257783878

5.4	-10.0	-9.9	0.08	2.533	0.677	5.129	2.373	6	7017	ZINC000118936941
5.4	-8.5	-8.4	0.07	2.865	1.064	5.418	2.052	6	7018	ZINC000014984540
5.4	-9.7	-9.5	0.12	1.873	1.079	3.997	2.896	6	7019	ZINC000253609251
5.4	-9.3	-8.8	0.33	2.802	0.756	5.335	2.465	6	7020	ZINC000257789844
5.4	-10.9	-10.5	0.25	1.813	0.254	4.958	1.741	6	7021	ZINC000100778304
5.4	-10.4	-10.0	0.26	2.089	0.287	6.155	0.926	6	7022	ZINC000257416176
5.4	-9.4	-8.7	0.37	3.243	0.651	6.855	0.265	6	7023	ZINC000014645921
5.4	-9.8	-9.5	0.20	2.384	0.606	6.361	2.740	6	7024	ZINC000257408374
5.4	-9.7	-9.4	0.18	2.436	0.640	4.758	2.440	6	7025	ZINC000014727602
5.4	-10.0	-9.5	0.23	1.569	0.479	5.630	3.187	6	7026	ZINC000014814120
5.4	-9.8	-9.2	0.32	1.864	0.484	3.929	1.938	6	7027	ZINC000261495605
5.4	-9.3	-9.1	0.18	2.796	0.990	4.039	1.201	6	7028	ZINC000014684666
5.4	-9.2	-8.9	0.22	2.688	0.513	6.742	2.647	6	7029	ZINC000014760539
5.4	-9.5	-9.2	0.15	2.771	0.611	7.201	3.107	6	7030	ZINC000012494532
5.4	-9.8	-9.3	0.27	2.771	0.659	5.922	2.646	6	7031	ZINC000257415836

5.4	-10.0	-9.5	0.29	2.782	0.742	4.711	1.493	6	7032	ZINC000253609460
5.4	-9.7	-9.5	0.10	1.712	1.230	3.779	3.029	6	7033	ZINC000034951214
5.4	-10.6	-10.1	0.39	2.345	0.495	5.254	1.694	6	7034	ZINC000022066501
5.4	-10.6	-9.6	0.48	2.801	0.513	6.051	1.713	6	7035	ZINC000014647507
5.4	-9.0	-8.8	0.12	2.491	0.469	4.202	1.770	6	7036	ZINC000012494015
5.4	-8.9	-8.6	0.19	3.016	0.946	5.281	2.243	6	7037	ZINC000014811670
5.4	-9.8	-9.6	0.22	3.087	1.182	5.375	1.628	6	7038	ZINC000015147368
5.4	-10.5	-10.1	0.20	2.251	0.735	4.650	2.032	6	7039	ZINC000044075095
5.4	-10.3	-9.8	0.27	2.773	0.328	6.358	2.824	6	7040	ZINC000013409107
5.4	-9.5	-9.4	0.11	2.235	0.603	4.668	0.173	6	7041	ZINC000257388908
5.4	-9.0	-8.8	0.14	2.742	0.786	4.970	1.615	6	7042	ZINC000014504958
5.4	-10.3	-10.0	0.24	2.192	0.614	5.551	1.243	6	7043	ZINC000100825235
5.4	-8.9	-8.8	0.14	2.059	0.692	4.577	2.729	6	7044	ZINC000014811762
5.4	-8.3	-8.0	0.16	2.180	0.914	6.515	3.090	6	7045	ZINC000005430808
5.4	-10.1	-10.0	0.11	2.347	0.883	7.180	0.931	6	7046	ZINC000257551727

5.4	-11.1	-10.7	0.33	1.739	0.550	4.335	0.687	6	7047	ZINC000257472028
5.4	-9.2	-8.9	0.21	3.279	0.396	7.364	1.446	6	7048	ZINC000119383357
5.4	-10.9	-10.0	0.52	2.433	0.534	5.454	1.275	6	7049	ZINC000003197581
5.4	-10.1	-9.7	0.24	3.143	0.318	5.843	0.524	6	7050	ZINC000257531899
5.3	-9.7	-9.6	0.11	2.455	0.382	5.583	1.103	6	7051	ZINC000003644941
5.3	-10.5	-10.2	0.15	2.167	0.612	5.698	2.365	6	7052	ZINC000005452569
5.3	-9.7	-9.5	0.11	2.473	0.554	5.245	2.182	6	7053	ZINC000100829776
5.3	-9.9	-9.8	0.18	1.651	0.599	4.188	2.008	6	7054	ZINC000257559418
5.3	-10.5	-9.8	0.35	1.977	0.748	5.424	1.493	6	7055	ZINC000014689171
5.3	-9.7	-9.5	0.10	2.389	0.517	4.381	1.844	6	7056	ZINC000252515358
5.3	-9.7	-9.5	0.12	2.478	0.565	5.237	2.170	6	7057	ZINC000252515361
5.3	-10.7	-9.8	0.51	2.698	0.502	6.304	2.733	6	7058	ZINC000014436717
5.3	-8.9	-8.7	0.10	3.209	0.331	5.993	1.532	6	7059	ZINC000014504949
5.3	-10.3	-10.1	0.13	2.768	0.369	6.690	0.859	6	7060	ZINC000230067902
5.3	-9.7	-9.5	0.11	2.300	0.395	3.757	0.625	6	7061	ZINC000252515359

5.3	-10.8	-10.0	0.39	2.053	0.230	6.234	1.027	6	7062	ZINC000257446875
5.3	-10.1	-9.6	0.27	2.150	0.448	5.648	2.522	6	7063	ZINC000098044023
5.3	-9.6	-9.5	0.11	2.503	1.312	5.343	3.479	6	7064	ZINC000013548878
5.3	-9.8	-9.0	0.39	2.376	0.469	6.639	0.412	6	7065	ZINC000257510195
5.3	-10.8	-10.5	0.16	2.249	0.521	4.736	1.766	6	7066	ZINC000014441537
5.3	-9.6	-9.3	0.19	3.022	0.672	8.572	2.005	6	7067	ZINC000096095400
5.3	-9.2	-8.9	0.20	3.100	0.594	4.290	0.870	6	7068	ZINC000015058240
5.3	-9.1	-8.6	0.27	2.851	0.387	5.778	1.421	6	7069	ZINC000257485548
5.3	-10.0	-9.5	0.23	2.751	0.437	4.658	1.882	6	7070	ZINC000085727128
5.3	-9.5	-9.3	0.15	2.295	0.306	5.636	2.686	6	7071	ZINC000257435899
5.3	-9.6	-8.7	0.43	2.742	0.143	7.138	1.421	6	7072	ZINC000257478195
5.3	-9.9	-9.2	0.42	2.369	0.631	4.868	1.752	6	7073	ZINC000261498267
5.3	-10.8	-10.3	0.55	1.736	0.604	4.351	1.618	6	7074	ZINC000257461826
5.3	-9.6	-9.1	0.38	2.777	0.109	6.019	0.921	6	7075	ZINC000013375634
5.3	-9.7	-9.5	0.12	2.471	0.547	5.240	2.165	6	7076	ZINC000100829774

5.3	-9.4	-9.1	0.22	4.825	0.463	8.416	1.237	6	7077	ZINC000013414436
5.3	-9.5	-9.1	0.23	2.598	0.605	4.237	0.985	6	7078	ZINC000014681171
5.3	-10.3	-9.3	0.47	2.433	0.690	4.395	1.254	6	7079	ZINC000014650506
5.3	-9.6	-9.4	0.12	2.069	0.466	5.514	2.054	6	7080	ZINC000256457394
5.3	-9.9	-9.7	0.16	2.276	0.494	4.846	2.364	6	7081	ZINC000257592431
5.3	-9.1	-8.9	0.17	2.483	1.216	4.084	0.933	6	7082	ZINC000014779796
5.3	-10.9	-10.0	0.54	2.546	0.580	6.400	0.740	6	7083	ZINC000085956032
5.3	-9.7	-9.5	0.11	2.348	0.462	4.316	1.691	6	7084	ZINC000100829773
5.3	-10.1	-9.8	0.24	2.652	0.491	7.789	0.556	6	7085	ZINC000085843930
5.3	-9.6	-9.4	0.11	2.247	0.470	4.136	0.255	6	7086	ZINC000257450438
5.3	-9.6	-9.1	0.26	2.954	0.249	6.298	1.921	6	7087	ZINC000039283191
5.3	-11.2	-10.4	0.38	3.202	0.395	5.922	1.166	6	7088	ZINC000257570250
5.3	-9.0	-8.8	0.10	2.864	0.395	5.827	1.711	6	7089	ZINC000014504955
5.3	-9.7	-9.5	0.11	2.350	0.459	4.315	1.679	6	7090	ZINC000252515360
5.3	-9.0	-8.7	0.21	2.118	0.653	4.553	2.273	6	7091	ZINC000257789847

5.3	-9.8	-9.3	0.34	3.340	0.906	5.516	1.663	6	7092	ZINC000136198513
5.3	-9.4	-9.1	0.18	2.688	0.241	6.578	1.747	6	7093	ZINC000059732029
5.3	-9.7	-9.5	0.11	2.348	0.463	4.297	1.688	6	7094	ZINC000100829771
5.3	-9.5	-8.9	0.38	2.537	0.470	6.695	2.411	6	7095	ZINC000257506932
5.3	-10.5	-9.8	0.38	1.822	0.702	6.096	0.768	6	7096	ZINC000257459509
5.3	-10.0	-9.9	0.09	3.065	0.750	6.259	1.777	6	7097	ZINC000085589027
5.3	-9.3	-9.0	0.18	2.436	0.202	5.530	1.931	6	7098	ZINC000014610289
5.3	-9.7	-9.5	0.11	2.491	0.576	5.230	2.168	6	7099	ZINC000008234373
5.3	-9.6	-9.1	0.29	2.703	0.477	4.563	0.865	6	7100	ZINC000257430040
5.3	-10.5	-9.8	0.38	1.943	0.730	4.977	0.983	6	7101	ZINC000001531453
5.3	-9.9	-9.6	0.30	2.150	0.629	5.538	2.657	6	7102	ZINC000257483639
5.3	-9.8	-9.5	0.22	2.516	0.621	4.030	1.807	6	7103	ZINC000100784238
5.3	-9.1	-8.9	0.13	2.289	0.974	5.120	2.651	6	7104	ZINC000257470254
5.3	-8.8	-8.7	0.13	2.550	0.343	6.107	3.107	6	7105	ZINC000014757250
5.3	-9.9	-9.7	0.23	2.378	0.718	5.170	2.011	6	7106	ZINC000257430033

5.3	-10.8	-10.2	0.38	2.213	0.696	5.703	1.578	6	7107	ZINC000014585978
5.3	-9.9	-9.7	0.15	2.127	0.619	5.631	3.131	6	7108	ZINC000257737003
5.3	-9.3	-9.1	0.12	3.366	0.411	6.810	1.580	6	7109	ZINC000257498746
5.3	-10.6	-9.6	0.55	2.754	0.826	8.118	0.730	6	7110	ZINC000013484754
5.3	-9.5	-9.3	0.14	2.327	0.497	6.782	0.767	6	7111	ZINC000257544780
5.3	-9.4	-9.0	0.25	2.658	0.867	4.743	2.469	6	7112	ZINC000100775198
5.3	-10.7	-9.9	0.46	1.900	0.334	4.300	1.630	6	7113	ZINC000019418977
5.3	-10.5	-9.8	0.33	1.821	0.596	5.680	1.402	6	7114	ZINC000014689168
5.3	-9.1	-8.8	0.23	2.442	0.822	6.083	2.153	6	7115	ZINC000238764818
5.3	-10.4	-10.2	0.23	2.970	0.659	4.981	1.131	6	7116	ZINC000014858270
5.3	-9.7	-9.4	0.21	2.430	0.340	7.417	2.905	6	7117	ZINC000014813777
5.3	-9.5	-9.3	0.11	3.244	0.693	7.857	1.971	6	7118	ZINC000100778445
5.3	-9.1	-8.8	0.15	2.028	0.508	4.634	2.480	6	7119	ZINC000015060714
5.3	-10.1	-9.8	0.13	2.244	0.240	6.186	2.173	6	7120	ZINC000100827596
5.3	-10.0	-9.6	0.22	2.489	0.374	7.269	0.511	6	7121	ZINC000085896636

5.3	-8.7	-8.5	0.17	2.500	0.756	5.442	2.198	6	7122	ZINC000257549714
5.3	-7.6	-7.4	0.17	3.204	0.906	6.087	1.728	6	7123	ZINC000006018558
5.3	-8.8	-8.6	0.24	2.272	0.427	5.321	1.789	6	7124	ZINC000257371391
5.3	-10.0	-9.5	0.26	2.073	0.275	5.199	2.372	6	7125	ZINC000257493209
5.3	-10.2	-9.8	0.19	2.764	0.609	5.553	2.314	6	7126	ZINC000100822024
5.3	-8.9	-8.7	0.15	2.084	0.317	3.875	1.781	6	7127	ZINC000014727331
5.3	-9.9	-9.5	0.32	3.004	0.574	5.382	1.567	6	7128	ZINC000118937302
5.2	-10.1	-9.6	0.26	2.540	0.521	6.718	2.349	6	7129	ZINC000257528517
5.2	-11.1	-10.6	0.34	1.739	0.555	4.336	0.686	6	7130	ZINC000257472030
5.2	-10.1	-10.0	0.13	2.507	0.734	5.991	1.939	6	7131	ZINC000257537711
5.2	-9.5	-9.2	0.20	2.096	1.110	6.147	2.973	6	7132	ZINC000014779845
5.2	-10.0	-9.6	0.26	3.247	0.403	8.061	1.532	6	7133	ZINC000257513022
5.2	-9.5	-8.9	0.29	2.888	0.341	7.943	2.380	6	7134	ZINC000238755905
5.2	-9.1	-8.6	0.41	2.069	0.543	3.415	1.218	6	7135	ZINC000014506023
5.2	-9.0	-8.8	0.09	2.741	0.675	6.158	2.812	6	7136	ZINC000014811674

5.2	-8.8	-8.4	0.22	1.897	0.397	5.057	2.441	6	7137	ZINC000257434138
5.2	-8.9	-8.7	0.12	3.098	0.298	6.518	1.713	6	7138	ZINC000257402548
5.2	-10.4	-9.6	0.49	2.640	0.226	5.841	1.519	6	7139	ZINC000257444945
5.2	-9.2	-9.0	0.14	3.356	0.246	7.907	3.071	6	7140	ZINC000257377504
5.2	-9.7	-9.3	0.23	2.460	0.093	5.880	0.693	6	7141	ZINC000013307307
5.2	-9.6	-9.4	0.13	2.087	0.452	4.149	0.295	6	7142	ZINC000257450437
5.2	-10.3	-9.4	0.43	2.244	0.559	5.437	1.558	6	7143	ZINC000257554976
5.2	-11.2	-10.6	0.33	1.862	0.661	6.903	2.980	6	7144	ZINC000257487039
5.2	-9.7	-9.7	0.05	2.351	0.641	4.482	2.463	6	7145	ZINC000257528531
5.2	-10.0	-9.8	0.14	2.638	0.641	4.557	1.144	6	7146	ZINC000100783800
5.2	-11.3	-11.1	0.21	2.081	0.708	3.970	1.555	6	7147	ZINC000100781691
5.2	-10.0	-9.4	0.32	2.375	0.241	5.757	2.214	6	7148	ZINC000118936901
5.2	-9.7	-9.4	0.21	2.611	0.482	5.440	2.812	6	7149	ZINC000014760855
5.2	-11.2	-10.2	0.52	2.509	0.768	4.718	1.269	6	7150	ZINC000015219798
5.2	-10.4	-10.1	0.19	2.077	0.359	6.723	1.769	6	7151	ZINC000254309074

5.2	-10.3	-9.8	0.25	2.663	0.406	5.435	2.084	6	7152	ZINC000033830927
5.2	-9.3	-8.5	0.36	2.522	0.777	4.783	1.921	6	7153	ZINC000261496901
5.2	-9.5	-8.5	0.47	2.840	0.388	5.683	0.425	6	7154	ZINC000257485789
5.2	-9.7	-9.2	0.21	2.230	0.381	5.966	0.660	6	7155	ZINC000100778078
5.2	-10.7	-10.2	0.31	2.036	0.576	6.890	1.594	6	7156	ZINC000082123410
5.2	-8.5	-8.1	0.33	3.614	1.763	4.824	1.516	6	7157	ZINC000000236891
5.2	-9.7	-9.3	0.19	2.355	0.767	5.692	3.096	6	7158	ZINC000257510194
5.2	-10.0	-9.2	0.38	2.370	0.318	5.818	2.170	6	7159	ZINC000118936899
5.2	-9.3	-9.0	0.24	2.315	1.123	6.143	2.961	6	7160	ZINC000257453621
5.2	-9.9	-9.7	0.16	1.574	0.380	3.623	2.261	6	7161	ZINC000257604350
5.2	-9.5	-9.4	0.09	2.242	0.640	5.538	2.047	6	7162	ZINC000256457398
5.2	-9.8	-9.5	0.21	2.681	0.669	3.516	0.893	6	7163	ZINC000085955990
5.2	-10.5	-9.8	0.37	2.064	0.308	7.394	2.173	6	7164	ZINC000257461516
5.2	-9.3	-9.0	0.27	3.416	0.734	6.837	1.709	6	7165	ZINC000015449241
5.2	-10.1	-9.3	0.41	1.859	0.424	5.735	1.925	6	7166	ZINC000100826631

5.2	-10.3	-9.6	0.36	2.977	0.425	6.411	0.698	6	7167	ZINC000015168915
5.2	-10.2	-9.7	0.29	1.839	0.679	4.153	3.147	6	7168	ZINC000015060716
5.2	-9.9	-9.6	0.16	2.155	0.242	7.252	3.316	6	7169	ZINC000034774793
5.2	-9.8	-9.7	0.08	2.958	0.738	6.716	0.944	6	7170	ZINC000257551596
5.2	-9.7	-9.5	0.07	2.390	0.579	4.771	1.914	6	7171	ZINC000015150591
5.2	-9.9	-9.4	0.31	1.968	0.555	4.930	2.560	6	7172	ZINC000253589630
5.2	-10.3	-9.6	0.38	2.894	0.720	5.613	1.854	6	7173	ZINC000015168917
5.2	-9.4	-9.3	0.07	3.064	0.683	6.296	1.849	6	7174	ZINC000015058242
5.2	-11.3	-11.1	0.20	2.108	0.714	3.973	1.553	6	7175	ZINC000100781689
5.2	-9.6	-9.3	0.24	1.992	0.793	4.084	1.809	6	7176	ZINC000008215581
5.2	-10.2	-9.5	0.36	1.948	0.357	5.967	0.554	6	7177	ZINC000230078108
5.2	-11.3	-10.8	0.24	2.074	0.489	4.367	2.268	6	7178	ZINC000257460140
5.2	-10.5	-10.2	0.23	2.751	0.835	6.013	1.148	6	7179	ZINC000008215403
5.2	-9.6	-9.1	0.27	2.831	0.703	6.299	2.455	6	7180	ZINC000000899109
5.2	-10.0	-9.8	0.17	2.471	0.517	4.243	0.894	6	7181	ZINC000257488907

5.2	-9.9	-9.4	0.32	1.939	0.752	3.894	2.591	6	7182	ZINC000257399920
5.2	-9.2	-9.0	0.15	2.878	0.612	6.539	1.489	6	7183	ZINC000257459234
5.2	-8.9	-8.5	0.41	2.234	0.166	4.395	1.226	6	7184	ZINC000014826860
5.2	-9.6	-9.4	0.14	2.282	0.713	6.641	2.219	6	7185	ZINC000257623737
5.1	-10.8	-10.3	0.40	2.724	0.602	4.455	2.382	6	7186	ZINC000100781703
5.1	-9.8	-9.7	0.12	2.324	0.940	5.309	3.047	6	7187	ZINC000004097812
5.1	-10.1	-9.8	0.16	1.995	0.153	5.517	1.859	6	7188	ZINC000257420085
5.1	-10.3	-9.4	0.41	2.490	0.814	3.876	1.386	6	7189	ZINC000004095721
5.1	-9.2	-9.1	0.11	2.426	0.594	4.200	2.441	6	7190	ZINC000257556656
5.1	-9.1	-8.9	0.15	3.182	0.585	6.700	0.902	6	7191	ZINC000013334298
5.1	-11.3	-10.8	0.33	2.296	0.601	6.876	0.662	6	7192	ZINC000100821962
5.1	-10.6	-9.6	0.50	2.719	0.218	5.434	0.267	6	7193	ZINC000257621275
5.1	-7.8	-7.6	0.13	2.972	1.067	3.854	1.398	6	7194	ZINC000004215527
5.1	-10.2	-9.6	0.45	2.360	0.531	5.378	1.629	6	7195	ZINC000085589030
5.1	-9.5	-9.2	0.18	2.518	0.598	6.000	2.688	6	7196	ZINC000030729869

5.1	-9.4	-9.3	0.11	1.752	1.083	5.167	3.430	6	7197	ZINC000200468525
5.1	-10.6	-10.2	0.27	3.012	0.770	5.033	1.534	6	7198	ZINC000257784406
5.1	-10.4	-9.3	0.53	3.719	0.309	6.729	0.374	6	7199	ZINC000257608679
5.1	-10.0	-9.3	0.35	2.037	0.444	5.278	2.664	6	7200	ZINC000118936900
5.1	-8.2	-7.7	0.28	3.145	0.818	4.027	0.991	6	7201	ZINC000014504988
5.1	-11.3	-11.1	0.20	2.582	0.704	5.710	0.379	6	7202	ZINC000100781690
5.1	-10.6	-10.2	0.28	2.785	0.737	4.865	1.586	6	7203	ZINC000257784416
5.1	-10.1	-9.2	0.44	2.706	0.727	5.015	1.698	6	7204	ZINC000257783876
5.1	-9.4	-9.1	0.23	1.845	0.620	3.545	2.408	6	7205	ZINC000257510196
5.1	-10.1	-9.7	0.27	2.516	0.295	6.153	0.929	6	7206	ZINC000100823157
5.1	-10.6	-10.2	0.22	2.637	0.587	4.765	1.580	6	7207	ZINC000100778939
5.1	-9.5	-9.2	0.21	3.449	1.175	7.042	2.428	6	7208	ZINC000015261714
5.1	-10.6	-10.2	0.26	2.701	0.676	4.559	1.584	6	7209	ZINC000100778940
5.1	-9.2	-8.9	0.18	3.409	0.558	6.847	2.829	6	7210	ZINC000257574313
5.1	-10.1	-9.4	0.39	1.451	0.611	4.420	1.963	6	7211	ZINC000257795033

5.1	-11.3	-11.1	0.18	2.106	0.702	3.998	1.568	6	7212	ZINC000257417337
5.1	-8.9	-8.7	0.10	3.118	0.276	5.916	1.615	6	7213	ZINC000257402547
5.1	-9.8	-9.6	0.17	2.238	0.185	4.962	1.030	6	7214	ZINC000100829054
5.1	-10.6	-10.3	0.23	2.828	0.826	4.039	0.685	6	7215	ZINC000257784409
5.1	-9.3	-9.1	0.14	3.375	0.946	6.130	1.916	6	7216	ZINC000261495541
5.1	-10.4	-9.4	0.47	2.344	0.681	5.291	2.589	6	7217	ZINC000257387408
5.1	-9.4	-9.2	0.19	2.574	1.193	5.175	2.455	6	7218	ZINC000257483367
5.1	-10.1	-9.8	0.24	2.593	0.291	6.483	1.239	6	7219	ZINC000100823155
5.1	-9.0	-8.9	0.14	2.673	0.173	6.251	2.267	6	7220	ZINC000238742363
5.1	-9.5	-9.2	0.20	2.708	0.562	6.458	1.876	6	7221	ZINC000257450436
5.1	-10.1	-9.8	0.19	2.944	0.342	6.812	1.340	6	7222	ZINC000100828910
5.1	-10.8	-10.3	0.31	2.328	0.477	4.597	1.465	6	7223	ZINC000013374494
5.1	-9.1	-8.8	0.13	2.276	0.488	3.963	2.175	6	7224	ZINC000257485788
5.1	-10.6	-10.2	0.24	2.699	0.672	4.561	1.582	6	7225	ZINC000257784412
5.1	-10.1	-10.0	0.06	2.967	1.052	6.850	2.314	6	7226	ZINC000014689064

5.1	-9.6	-9.4	0.16	2.245	0.513	7.816	2.109	6	7227	ZINC000257378882
5.1	-9.9	-9.2	0.33	2.240	0.207	5.848	2.132	6	7228	ZINC000118936898
5.1	-9.3	-8.9	0.23	2.952	0.469	6.721	2.023	6	7229	ZINC000040165471
5.1	-10.1	-9.8	0.26	2.611	0.321	6.130	0.878	6	7230	ZINC000100823154
5.1	-9.7	-9.3	0.31	3.560	0.422	6.575	1.892	6	7231	ZINC000118937341
5.1	-10.6	-10.2	0.29	2.879	0.836	4.282	0.879	6	7232	ZINC000100778938
5.1	-9.9	-9.7	0.19	2.724	0.434	5.447	1.658	6	7233	ZINC000004098695
5.1	-10.2	-9.8	0.30	2.605	0.579	5.503	1.413	6	7234	ZINC000095620782
5.1	-8.6	-8.3	0.24	2.635	0.721	4.788	2.532	6	7235	ZINC000014763011
5.1	-9.5	-9.0	0.31	2.318	0.598	6.591	2.294	6	7236	ZINC000085664580
5.1	-11.0	-10.8	0.11	2.104	0.563	5.930	0.852	6	7237	ZINC000085977717
5.1	-9.5	-9.3	0.17	2.744	0.683	5.434	2.330	6	7238	ZINC000257450439
5.1	-10.1	-9.6	0.31	2.478	0.231	5.717	1.211	6	7239	ZINC000006920437
5.1	-10.0	-9.4	0.33	2.590	0.338	6.676	2.207	6	7240	ZINC000257392057
5.1	-9.3	-9.2	0.09	2.457	0.479	9.794	0.310	6	7241	ZINC000238776397

5.1	-10.7	-10.3	0.31	2.307	0.625	4.374	2.394	6	7242	ZINC000100781697
5.1	-9.3	-9.1	0.21	2.304	0.453	5.248	2.165	6	7243	ZINC000261497585
5.1	-9.3	-9.0	0.19	3.039	0.845	5.766	1.603	6	7244	ZINC000261498223
5.1	-10.6	-10.2	0.23	2.738	0.748	4.710	1.578	6	7245	ZINC000100778941
5.1	-9.0	-8.6	0.24	2.687	0.246	6.087	1.534	6	7246	ZINC000014681006
5.1	-9.2	-8.8	0.25	2.657	0.706	6.066	2.669	6	7247	ZINC000257505607
5.1	-10.3	-10.1	0.16	2.272	0.309	4.809	2.045	6	7248	ZINC000014930821
5.1	-9.2	-9.0	0.17	2.248	0.526	5.232	2.073	6	7249	ZINC000257478194
5.1	-10.0	-9.7	0.16	2.484	0.628	7.756	3.023	6	7250	ZINC000012153135
5.1	-10.7	-10.3	0.21	2.560	0.561	5.171	1.519	6	7251	ZINC000100824423
5.1	-9.5	-9.1	0.24	2.421	0.399	4.313	2.678	6	7252	ZINC000257388900
5.0	-9.8	-9.5	0.19	2.295	0.437	6.345	2.278	6	7253	ZINC000100773464
5.0	-9.3	-8.9	0.22	2.454	0.592	5.516	1.893	6	7254	ZINC000001763468
5.0	-8.5	-8.4	0.07	2.058	0.331	7.229	1.355	6	7255	ZINC000257434137
5.0	-10.2	-9.6	0.32	2.328	0.565	6.102	2.065	6	7256	ZINC000257530279

5.0	-10.2	-9.9	0.19	1.852	0.516	5.688	0.516	6	7257	ZINC000014435559
5.0	-10.8	-10.2	0.36	2.015	0.450	5.712	0.605	6	7258	ZINC000003197580
5.0	-9.1	-8.7	0.24	2.321	0.293	4.805	1.641	6	7259	ZINC000014826854
5.0	-8.5	-8.3	0.10	2.124	0.450	7.722	2.777	6	7260	ZINC000014644881
5.0	-10.6	-10.3	0.24	1.878	0.027	4.704	1.901	6	7261	ZINC000118937116
5.0	-8.4	-8.3	0.12	2.268	0.569	5.853	0.897	6	7262	ZINC000100829104
5.0	-9.9	-9.4	0.31	2.532	0.228	6.655	1.907	6	7263	ZINC000257549392
5.0	-10.1	-9.8	0.22	2.012	0.755	4.725	2.128	6	7264	ZINC000238745229
5.0	-9.8	-9.5	0.23	2.683	0.406	6.141	0.697	6	7265	ZINC000257618973
5.0	-10.0	-9.7	0.15	3.847	0.619	7.785	1.833	6	7266	ZINC000142924020
5.0	-8.4	-8.0	0.23	2.973	0.567	5.616	2.400	6	7267	ZINC000014762755
5.0	-9.8	-9.6	0.12	2.993	0.223	6.091	1.358	6	7268	ZINC000059737024
5.0	-9.7	-9.3	0.20	1.947	0.108	5.050	2.091	6	7269	ZINC000257531901
5.0	-10.4	-10.0	0.22	1.796	0.248	4.070	1.809	6	7270	ZINC000257420088
5.0	-9.8	-9.6	0.15	2.068	1.081	4.517	1.675	6	7271	ZINC000257618974

5.0	-9.8	-9.4	0.21	2.492	0.471	5.554	0.979	6	7272	ZINC000015271304
5.0	-9.0	-8.9	0.08	2.417	0.275	6.582	1.810	6	7273	ZINC000015046506
5.0	-9.8	-9.5	0.20	2.540	0.462	5.956	0.424	6	7274	ZINC000257618971
5.0	-10.4	-10.1	0.19	2.081	0.952	3.348	2.082	6	7275	ZINC000100771607
5.0	-10.6	-10.1	0.24	3.713	0.481	6.571	1.315	6	7276	ZINC000257447822
5.0	-10.3	-9.9	0.29	2.001	0.256	4.989	1.718	6	7277	ZINC000257450977
5.0	-9.7	-9.3	0.21	2.508	0.655	4.922	1.517	6	7278	ZINC000230054498
5.0	-9.6	-9.4	0.09	1.977	0.448	4.135	2.908	6	7279	ZINC000257532629
5.0	-9.6	-9.2	0.23	2.463	0.482	6.034	2.005	6	7280	ZINC000014727349
5.0	-9.6	-9.4	0.17	3.219	0.614	6.045	1.424	6	7281	ZINC000257416819
5.0	-9.6	-9.4	0.10	2.184	0.550	4.992	2.860	6	7282	ZINC000012153879
5.0	-9.9	-9.6	0.20	1.825	0.380	4.957	2.314	6	7283	ZINC000238760477
5.0	-11.8	-10.9	0.47	2.461	0.300	6.724	0.477	6	7284	ZINC000086041870
5.0	-9.8	-9.5	0.18	2.360	0.368	7.471	1.918	6	7285	ZINC000100773467
5.0	-9.8	-9.5	0.18	2.864	0.425	8.035	0.330	6	7286	ZINC000257435348

5.0	-9.6	-9.3	0.17	2.903	0.340	7.256	1.511	6	7287	ZINC000015150584
5.0	-9.8	-9.5	0.15	2.589	0.590	7.127	1.895	6	7288	ZINC000257435346
5.0	-9.6	-9.4	0.17	2.613	1.279	6.261	2.856	6	7289	ZINC000014779804
5.0	-9.8	-9.3	0.26	2.002	0.635	4.907	2.422	6	7290	ZINC000261495606
5.0	-9.7	-9.0	0.32	2.970	0.210	5.888	1.138	6	7291	ZINC000014618516
5.0	-9.4	-9.2	0.13	3.457	0.453	6.109	1.085	6	7292	ZINC000100772908
5.0	-9.3	-8.9	0.23	2.618	0.416	5.484	1.745	6	7293	ZINC000014680915
5.0	-9.3	-8.9	0.23	2.744	0.699	6.384	1.701	6	7294	ZINC000118937344
5.0	-8.5	-8.3	0.10	2.459	0.769	5.519	1.891	6	7295	ZINC000014645915
5.0	-10.5	-10.1	0.26	2.059	0.311	5.590	1.993	6	7296	ZINC000257547692
5.0	-9.8	-9.6	0.19	2.153	1.109	4.927	1.691	6	7297	ZINC000015271309
5.0	-8.2	-8.0	0.17	2.503	1.506	4.529	1.383	6	7298	ZINC000095617907
5.0	-10.5	-9.6	0.43	2.756	0.536	6.925	0.722	6	7299	ZINC000014647509
5.0	-7.8	-7.6	0.14	2.041	0.724	5.133	2.257	6	7300	ZINC000238782887
5.0	-9.6	-9.3	0.16	2.453	0.620	4.493	1.837	6	7301	ZINC000008217042

5.0	-10.2	-9.5	0.40	2.949	0.249	4.524	0.410	6	7302	ZINC000100824504
5.0	-9.7	-9.5	0.19	3.389	0.418	5.458	2.364	6	7303	ZINC000014757094
5.0	-9.8	-9.4	0.21	2.469	0.488	5.595	0.972	6	7304	ZINC000257618972
5.0	-10.1	-9.4	0.41	2.147	0.752	5.632	3.200	6	7305	ZINC000257462919
5.0	-9.8	-9.5	0.17	2.580	0.380	7.222	1.891	6	7306	ZINC000257435349
5.0	-10.2	-10.0	0.16	2.811	0.804	4.298	0.890	6	7307	ZINC000257461704
5.0	-9.8	-9.5	0.21	2.068	1.039	4.175	1.595	6	7308	ZINC000015271300
5.0	-10.4	-10.0	0.25	2.398	0.276	5.053	1.508	6	7309	ZINC000031156118
5.0	-9.8	-9.6	0.21	2.053	1.081	4.518	1.745	6	7310	ZINC000015271302
5.0	-8.7	-8.6	0.12	3.463	0.901	5.306	1.425	6	7311	ZINC000006484761
5.0	-10.7	-10.2	0.37	2.243	0.178	6.750	1.224	6	7312	ZINC000100772865
5.0	-10.3	-9.6	0.37	2.242	0.278	4.051	1.604	6	7313	ZINC000067665085
4.9	-9.7	-9.2	0.30	1.975	0.226	4.428	2.141	6	7314	ZINC000085955986
4.9	-10.7	-10.5	0.13	1.974	0.565	4.195	1.940	6	7315	ZINC000257385149
4.9	-8.9	-8.5	0.25	2.562	0.660	6.124	1.305	6	7316	ZINC000014645688

4.9	-10.0	-9.7	0.21	1.996	0.510	3.820	1.747	6	7317	ZINC000257420488
4.9	-11.1	-10.5	0.35	2.634	0.756	7.052	3.068	6	7318	ZINC000257622070
4.9	-9.3	-9.0	0.24	2.476	0.317	6.748	0.714	6	7319	ZINC000100825439
4.9	-9.9	-9.3	0.30	1.811	0.251	3.596	1.920	6	7320	ZINC000100773765
4.9	-11.2	-10.8	0.35	2.273	0.449	5.620	2.161	6	7321	ZINC000100825714
4.9	-9.6	-9.2	0.19	2.521	0.779	4.900	2.522	6	7322	ZINC000006394164
4.9	-10.5	-9.8	0.44	1.856	0.404	5.125	2.964	6	7323	ZINC000015150603
4.9	-11.1	-10.4	0.35	2.564	0.747	6.192	2.789	6	7324	ZINC000257488906
4.9	-10.2	-9.6	0.39	2.117	0.129	5.883	0.292	6	7325	ZINC000257416178
4.9	-10.0	-9.6	0.20	2.584	0.259	6.064	1.466	6	7326	ZINC000100830204
4.9	-9.4	-9.0	0.31	2.961	0.252	6.204	1.548	6	7327	ZINC000005758782
4.9	-10.3	-10.1	0.21	1.777	0.599	3.634	2.264	6	7328	ZINC000014814122
4.9	-10.4	-9.9	0.26	3.466	0.321	6.779	0.699	6	7329	ZINC000033988549
4.9	-10.2	-9.8	0.28	2.210	0.220	6.342	0.435	6	7330	ZINC000100775757
4.9	-11.8	-11.2	0.37	1.069	0.464	6.061	0.717	6	7331	ZINC000003780340

4.9	-9.4	-9.3	0.14	2.210	0.569	5.059	2.508	6	7332	ZINC000257768737
4.9	-11.3	-10.4	0.48	2.121	0.288	4.903	1.853	6	7333	ZINC000015219795
4.9	-10.5	-9.9	0.32	2.562	0.928	4.113	1.668	6	7334	ZINC000257378568
4.9	-8.6	-8.4	0.15	2.937	0.251	6.309	2.036	6	7335	ZINC000014586263
4.9	-11.1	-10.4	0.39	2.603	0.774	8.064	2.766	6	7336	ZINC000100783803
4.9	-9.9	-9.2	0.34	2.751	0.690	4.491	2.562	6	7337	ZINC000014757097
4.9	-9.2	-9.0	0.20	2.437	0.397	5.183	2.290	6	7338	ZINC000013416205
4.9	-8.0	-7.6	0.23	3.383	1.110	6.461	1.878	6	7339	ZINC000034290496
4.9	-10.4	-9.7	0.36	1.564	0.422	4.272	0.278	6	7340	ZINC000257428874
4.9	-10.0	-9.6	0.21	2.537	0.340	6.036	1.514	6	7341	ZINC000100830205
4.9	-9.2	-8.9	0.16	2.342	1.063	5.195	3.534	6	7342	ZINC000118936826
4.9	-11.1	-10.8	0.27	2.476	0.234	6.544	1.383	6	7343	ZINC000024215118
4.9	-10.0	-9.6	0.20	2.600	0.235	6.085	1.436	6	7344	ZINC000100830203
4.9	-11.1	-10.5	0.33	2.498	0.831	7.012	3.100	6	7345	ZINC000257622069
4.9	-9.8	-9.5	0.18	2.704	0.512	5.288	2.132	6	7346	ZINC000084707297

4.9	-9.4	-9.1	0.24	2.884	0.386	7.398	3.099	6	7347	ZINC000059211655
4.9	-10.6	-10.1	0.27	2.420	0.794	5.550	1.371	6	7348	ZINC000014585990
4.9	-9.2	-9.1	0.11	2.381	0.440	4.741	1.647	6	7349	ZINC000257378777
4.9	-9.2	-9.0	0.13	2.206	0.524	4.683	1.643	6	7350	ZINC000257378779
4.9	-10.0	-9.7	0.18	2.264	0.378	7.271	1.692	6	7351	ZINC000015150580
4.9	-10.0	-9.6	0.19	1.792	0.573	3.924	2.057	6	7352	ZINC000095619314
4.9	-8.9	-8.8	0.07	1.989	0.920	6.413	2.005	6	7353	ZINC000100782669
4.9	-10.8	-9.8	0.44	1.918	0.427	5.214	2.530	6	7354	ZINC000100829854
4.9	-9.8	-9.3	0.26	3.339	1.157	5.417	2.817	6	7355	ZINC000257586850
4.9	-8.5	-8.1	0.25	1.941	0.598	6.001	2.724	6	7356	ZINC000014717727
4.9	-10.0	-9.6	0.34	3.019	1.090	5.269	2.061	6	7357	ZINC000100826981
4.9	-10.2	-9.8	0.25	2.590	0.447	6.228	1.917	6	7358	ZINC000100775753
4.9	-9.1	-8.8	0.17	1.996	0.495	4.849	2.419	6	7359	ZINC000004521575
4.9	-10.9	-10.3	0.29	3.721	0.510	5.940	1.283	6	7360	ZINC000014860718
4.9	-9.0	-8.7	0.16	2.792	0.542	7.028	1.952	6	7361	ZINC000118936791

4.9	-10.4	-10.0	0.20	2.842	0.358	5.947	1.327	6	7362	ZINC000100772858
4.9	-9.7	-9.0	0.35	2.157	0.411	4.665	1.900	6	7363	ZINC000039036685
4.9	-8.3	-8.2	0.07	1.995	1.249	5.126	2.439	6	7364	ZINC000049601658
4.9	-10.9	-10.4	0.32	2.660	0.587	6.257	1.326	6	7365	ZINC000038497796
4.9	-11.1	-10.3	0.37	2.973	0.394	9.486	0.489	6	7366	ZINC000257488905
4.9	-10.7	-10.4	0.23	2.187	0.357	6.394	1.264	6	7367	ZINC000150368133
4.9	-9.9	-9.6	0.18	2.448	0.492	4.182	1.483	6	7368	ZINC000014512154
4.9	-9.8	-9.3	0.27	2.929	0.228	5.404	0.374	6	7369	ZINC000084428482
4.9	-9.0	-8.8	0.15	2.953	0.357	6.159	0.844	6	7370	ZINC000257515728
4.8	-10.9	-10.5	0.22	1.869	0.508	3.909	1.411	6	7371	ZINC000257376312
4.8	-10.7	-10.3	0.17	2.222	0.171	5.949	1.031	6	7372	ZINC000100829483
4.8	-9.5	-9.0	0.29	2.585	0.773	5.986	2.337	6	7373	ZINC000014417208
4.8	-9.3	-8.8	0.25	2.629	0.311	6.176	1.268	6	7374	ZINC000014645690
4.8	-8.7	-8.5	0.12	3.200	0.829	6.627	1.825	6	7375	ZINC000015165530
4.8	-9.5	-9.0	0.32	2.376	0.507	5.920	2.321	6	7376	ZINC000257506931

4.8	-11.1	-10.4	0.40	1.846	0.219	6.729	0.181	6	7377	ZINC000100779018
4.8	-8.7	-8.5	0.12	3.104	0.798	5.613	1.550	6	7378	ZINC000230087349
4.8	-10.1	-10.0	0.06	2.725	0.952	7.598	0.995	6	7379	ZINC000257551725
4.8	-9.6	-9.3	0.16	2.616	0.536	7.148	2.396	6	7380	ZINC000004097814
4.8	-9.7	-9.0	0.34	2.582	0.389	5.659	2.015	6	7381	ZINC000003860283
4.8	-11.4	-10.1	0.61	2.370	0.677	5.605	2.983	6	7382	ZINC000085935104
4.8	-9.5	-9.2	0.17	2.764	1.027	7.008	3.739	6	7383	ZINC000008536462
4.8	-10.1	-10.0	0.11	2.383	0.789	7.452	0.838	6	7384	ZINC000100830133
4.8	-9.4	-9.0	0.27	3.203	1.069	5.529	2.165	6	7385	ZINC000004096562
4.8	-9.9	-9.2	0.34	2.904	0.233	6.790	2.796	6	7386	ZINC000014759109
4.8	-9.6	-9.0	0.35	2.534	0.744	5.345	1.563	6	7387	ZINC000100771617
4.8	-9.6	-9.2	0.27	2.475	0.850	5.841	2.387	6	7388	ZINC000253609461
4.8	-10.4	-10.1	0.19	2.629	0.650	6.110	1.429	6	7389	ZINC000012494712
4.8	-10.3	-9.5	0.50	1.895	0.565	3.808	1.570	6	7390	ZINC000040164518
4.8	-10.0	-9.7	0.20	2.521	0.706	5.423	2.363	6	7391	ZINC000013382490

4.8	-9.5	-9.3	0.12	2.463	0.700	4.921	1.985	6	7392	ZINC000008551772
4.8	-9.5	-9.3	0.11	1.809	0.699	5.005	3.424	6	7393	ZINC000257603265
4.8	-9.0	-8.8	0.15	2.725	0.350	5.933	1.865	6	7394	ZINC000257633500
4.8	-9.3	-8.7	0.37	2.210	0.397	5.311	2.201	6	7395	ZINC000257553844
4.8	-9.7	-9.3	0.24	2.803	0.502	5.781	1.706	6	7396	ZINC000014691743
4.8	-9.0	-8.5	0.26	2.765	0.150	6.640	0.811	6	7397	ZINC000257423771
4.8	-9.7	-8.8	0.61	2.241	0.317	5.261	1.751	6	7398	ZINC000059065202
4.8	-8.7	-8.5	0.12	3.111	0.798	5.630	1.535	6	7399	ZINC000006032353
4.8	-10.0	-9.6	0.20	2.461	0.313	5.302	1.486	6	7400	ZINC000257420487
4.8	-9.0	-8.7	0.26	2.121	0.380	5.080	0.600	6	7401	ZINC000257377988
4.8	-7.7	-7.4	0.24	2.377	0.513	5.614	2.027	6	7402	ZINC000014436447
4.8	-9.0	-8.9	0.11	2.327	0.624	5.430	1.373	6	7403	ZINC000008216005
4.8	-9.0	-8.8	0.15	2.867	0.537	6.129	2.131	6	7404	ZINC000257633501
4.8	-7.9	-7.4	0.25	4.377	1.680	6.600	3.097	6	7405	ZINC000001531693
4.8	-10.8	-10.3	0.39	2.663	0.771	4.719	1.872	6	7406	ZINC000257385148

4.8	-10.0	-9.6	0.19	2.460	0.316	5.300	1.489	6	7407	ZINC000100830201
4.8	-9.0	-8.8	0.13	2.184	0.538	4.433	2.115	6	7408	ZINC000013309941
4.8	-8.4	-8.2	0.12	2.682	0.305	6.949	2.005	6	7409	ZINC000100782891
4.8	-10.7	-10.1	0.49	2.374	0.204	6.054	0.451	6	7410	ZINC000100772966
4.8	-10.9	-10.8	0.13	2.739	0.658	7.100	0.572	6	7411	ZINC000015251996
4.8	-10.4	-10.1	0.20	2.442	0.399	5.401	2.811	6	7412	ZINC000014930845
4.8	-10.9	-10.4	0.37	1.956	0.210	5.942	0.320	6	7413	ZINC000085797750
4.8	-8.9	-8.7	0.19	3.195	0.492	6.764	2.282	6	7414	ZINC000257397621
4.8	-9.3	-8.9	0.17	3.339	0.343	7.532	1.637	6	7415	ZINC000014757230
4.8	-9.1	-8.9	0.19	3.026	0.710	5.330	1.095	6	7416	ZINC000085801175
4.8	-11.9	-11.5	0.27	2.110	0.333	5.372	1.017	6	7417	ZINC000100826931
4.8	-9.5	-9.3	0.16	2.378	0.283	6.542	2.232	6	7418	ZINC000015252015
4.8	-9.8	-9.4	0.28	2.266	0.334	6.362	2.448	6	7419	ZINC000257592430
4.8	-9.4	-9.2	0.17	2.307	0.817	4.127	2.501	6	7420	ZINC000013551306
4.8	-10.2	-9.6	0.29	2.042	0.677	4.613	2.807	6	7421	ZINC000257547949

4.8	-9.3	-9.0	0.14	2.046	0.324	7.382	2.493	6	7422	ZINC000257635543
4.8	-9.0	-8.8	0.14	3.264	1.109	8.286	1.277	6	7423	ZINC000031291853
4.8	-9.5	-9.1	0.22	2.738	0.330	5.572	0.846	6	7424	ZINC000256233361
4.8	-10.6	-10.1	0.30	4.165	0.251	6.090	0.740	6	7425	ZINC000015147375
4.8	-10.1	-9.9	0.11	2.559	0.437	6.986	1.058	6	7426	ZINC000257551728
4.8	-10.1	-9.6	0.33	2.834	0.526	5.775	0.564	6	7427	ZINC000015209924
4.8	-9.8	-9.4	0.19	1.884	0.476	3.951	2.539	6	7428	ZINC000257537504
4.8	-10.0	-9.7	0.19	2.325	0.522	4.943	1.970	6	7429	ZINC000238749979
4.8	-9.2	-8.9	0.15	2.184	1.115	4.865	3.317	6	7430	ZINC000257498749
4.8	-9.4	-8.1	0.85	2.528	0.640	4.676	1.434	6	7431	ZINC000008214556
4.8	-8.9	-8.7	0.17	2.394	0.400	6.724	2.790	6	7432	ZINC000014644885
4.8	-9.5	-9.4	0.09	3.401	0.189	8.340	1.821	6	7433	ZINC000257535030
4.8	-8.8	-8.4	0.23	2.283	0.395	7.333	0.192	6	7434	ZINC000257619394
4.8	-9.6	-9.4	0.14	2.444	0.544	5.575	2.428	6	7435	ZINC000257435347
4.8	-11.0	-10.8	0.13	2.211	0.152	5.002	1.524	6	7436	ZINC000100779094

4.8	-9.8	-9.5	0.19	2.218	0.536	5.726	2.752	6	7437	ZINC000014436820
4.8	-9.6	-9.4	0.14	2.464	0.515	5.587	2.286	6	7438	ZINC000100773470
4.8	-10.9	-10.1	0.45	2.035	0.563	3.803	1.749	6	7439	ZINC000257524852
4.8	-10.4	-9.9	0.27	1.669	0.363	3.826	1.305	6	7440	ZINC000257428876
4.8	-9.3	-9.1	0.13	2.465	0.334	5.061	2.171	6	7441	ZINC000012153880
4.8	-11.4	-10.8	0.36	2.272	0.069	5.555	0.765	6	7442	ZINC000105386968
4.8	-10.3	-9.9	0.26	2.890	0.263	6.373	2.209	6	7443	ZINC000257479183
4.8	-8.8	-8.3	0.29	2.775	0.372	6.241	1.207	6	7444	ZINC000015210061
4.8	-9.6	-9.5	0.15	1.972	0.319	4.386	2.039	6	7445	ZINC000257411454
4.7	-10.1	-9.2	0.43	3.213	0.276	6.194	1.328	6	7446	ZINC000030725719
4.7	-9.2	-8.9	0.21	2.714	0.406	5.369	0.801	6	7447	ZINC000257379842
4.7	-9.1	-8.3	0.38	3.404	0.762	7.142	2.082	6	7448	ZINC000095620665
4.7	-9.0	-8.8	0.12	2.722	0.721	6.474	2.252	6	7449	ZINC000100825155
4.7	-8.8	-8.6	0.12	2.439	0.421	6.110	1.925	6	7450	ZINC000014757229
4.7	-10.0	-9.8	0.26	3.384	0.469	6.494	0.711	6	7451	ZINC000257498747

4.7	-12.2	-11.8	0.26	4.569	1.109	7.349	1.035	6	7452	ZINC000257448433
4.7	-11.4	-10.7	0.37	2.041	0.903	4.875	2.152	6	7453	ZINC000100823228
4.7	-7.9	-7.7	0.16	3.077	1.290	5.675	3.047	6	7454	ZINC000013352621
4.7	-9.9	-9.4	0.26	2.316	0.549	4.932	1.893	6	7455	ZINC000238755709
4.7	-9.9	-9.5	0.24	2.396	0.630	4.683	1.485	6	7456	ZINC000014642602
4.7	-8.0	-7.9	0.13	2.332	0.330	8.525	0.071	6	7457	ZINC000040165528
4.7	-8.7	-8.5	0.12	3.210	1.053	7.431	1.488	6	7458	ZINC000100780943
4.7	-9.2	-8.9	0.27	2.270	0.384	4.893	2.832	6	7459	ZINC000015156993
4.7	-9.5	-9.2	0.19	3.756	0.425	6.203	1.703	6	7460	ZINC000012495985
4.7	-8.8	-8.6	0.13	3.020	0.620	5.880	1.302	6	7461	ZINC000015257518
4.7	-9.9	-9.4	0.27	2.801	0.480	7.482	2.096	6	7462	ZINC000257562847
4.7	-12.2	-11.8	0.26	4.306	1.552	6.377	2.183	6	7463	ZINC000257448435
4.7	-9.9	-9.5	0.23	2.315	0.617	4.567	1.632	6	7464	ZINC000014642605
4.7	-10.6	-10.2	0.28	2.710	1.703	5.495	2.722	6	7465	ZINC000096014960
4.7	-9.3	-9.1	0.15	2.499	0.599	5.870	2.528	6	7466	ZINC000118937369

4.7	-8.8	-8.5	0.21	3.314	0.121	6.317	1.555	6	7467	ZINC000014437033
4.7	-9.5	-8.9	0.38	2.644	0.515	4.913	1.980	6	7468	ZINC000014417210
4.7	-10.6	-10.3	0.33	2.276	0.173	5.572	1.159	6	7469	ZINC000014827091
4.7	-10.0	-9.9	0.08	2.170	0.449	7.055	0.635	6	7470	ZINC000257435157
4.7	-8.7	-8.5	0.14	3.197	0.839	6.415	1.487	6	7471	ZINC000257475753
4.7	-9.6	-9.2	0.32	2.119	0.383	5.674	1.850	6	7472	ZINC000014647511
4.7	-9.4	-9.2	0.10	2.977	0.368	4.550	0.419	6	7473	ZINC000014644685
4.7	-10.1	-9.7	0.20	1.986	0.301	8.288	3.113	6	7474	ZINC000257567735
4.7	-9.3	-8.8	0.24	2.380	0.409	6.045	2.587	6	7475	ZINC000257607435
4.7	-9.3	-8.9	0.30	2.315	0.247	6.822	1.599	6	7476	ZINC000257789845
4.7	-9.5	-8.8	0.35	4.396	1.101	6.058	1.880	6	7477	ZINC000014712188
4.7	-9.4	-9.3	0.17	2.210	0.534	5.049	2.496	6	7478	ZINC000100779147
4.7	-10.4	-10.2	0.12	2.592	0.314	5.981	0.768	6	7479	ZINC000014827094
4.7	-9.5	-9.2	0.18	2.194	0.549	4.610	1.933	6	7480	ZINC000119382196
4.7	-10.2	-9.3	0.45	2.758	0.830	5.868	2.217	6	7481	ZINC000014612457

4.7	-9.9	-9.6	0.24	2.461	0.539	4.297	1.556	6	7482	ZINC000014642608
4.7	-9.0	-8.9	0.08	2.333	0.530	4.216	1.982	6	7483	ZINC000085871715
4.7	-9.9	-9.6	0.17	2.641	0.583	5.365	2.383	6	7484	ZINC000085589150
4.7	-9.5	-9.0	0.31	2.560	0.403	6.011	2.184	6	7485	ZINC000014417204
4.7	-11.2	-10.8	0.21	2.214	0.943	4.348	2.035	6	7486	ZINC000085611174
4.7	-10.3	-10.1	0.13	2.850	0.234	4.760	0.827	6	7487	ZINC000100824512
4.7	-8.5	-8.2	0.23	2.984	0.496	6.426	1.096	6	7488	ZINC000005851338
4.7	-12.2	-11.8	0.26	4.543	1.118	7.356	1.029	6	7489	ZINC000257448434
4.7	-9.6	-9.0	0.29	3.016	0.525	6.921	1.646	6	7490	ZINC000036402459
4.7	-9.2	-8.7	0.28	2.570	0.666	4.329	2.395	6	7491	ZINC000014644557
4.7	-9.3	-9.0	0.23	2.379	0.542	6.026	2.226	6	7492	ZINC000118937371
4.7	-9.1	-8.5	0.34	2.975	2.027	4.722	2.939	6	7493	ZINC000000538453
4.7	-9.1	-8.7	0.29	2.899	0.417	5.096	1.649	6	7494	ZINC000015268308
4.7	-9.7	-9.3	0.24	2.738	0.359	6.208	1.462	6	7495	ZINC000257785082
4.7	-9.4	-8.9	0.27	2.394	0.445	5.326	1.710	6	7496	ZINC000257380055

4.7	-9.7	-9.3	0.24	2.057	0.620	4.858	2.531	6	7497	ZINC000257405262
4.7	-9.3	-9.2	0.13	3.096	0.852	5.667	1.990	6	7498	ZINC000100778551
4.7	-9.3	-9.1	0.16	2.682	0.359	6.374	0.814	6	7499	ZINC000100773769
4.7	-9.2	-8.6	0.40	2.877	0.305	6.396	1.008	6	7500	ZINC000257590407
4.7	-8.4	-8.3	0.09	1.889	0.316	5.733	1.360	6	7501	ZINC000036443189
4.7	-9.3	-8.8	0.23	2.251	0.502	4.788	1.683	6	7502	ZINC000014680903
4.7	-9.8	-9.5	0.28	1.957	0.329	4.988	3.317	6	7503	ZINC000014709120
4.7	-9.7	-9.2	0.29	2.382	0.241	7.317	1.121	6	7504	ZINC000257548389
4.7	-9.4	-8.9	0.31	2.314	0.348	5.871	2.232	6	7505	ZINC000257506930
4.7	-9.9	-9.5	0.29	2.656	0.567	5.125	1.594	6	7506	ZINC000257695264
4.7	-12.2	-11.8	0.27	4.565	1.112	7.362	1.028	6	7507	ZINC000257448436
4.7	-8.7	-8.5	0.12	2.858	1.100	7.131	1.409	6	7508	ZINC000100780946
4.7	-10.3	-9.6	0.40	2.415	0.910	4.476	2.583	6	7509	ZINC000257578814
4.7	-10.3	-9.8	0.31	2.165	0.508	5.176	1.194	6	7510	ZINC000014585977
4.7	-9.9	-9.6	0.26	2.459	0.643	4.062	1.408	6	7511	ZINC000257695265

4.7	-9.5	-8.7	0.44	4.742	0.790	8.460	1.401	6	7512	ZINC000014712185
4.7	-9.1	-8.9	0.18	2.560	0.449	6.700	2.253	6	7513	ZINC000014586259
4.7	-12.0	-11.5	0.39	2.555	0.365	6.307	0.953	6	7514	ZINC000086041847
4.7	-9.6	-9.4	0.21	3.315	0.786	6.974	2.519	6	7515	ZINC000257551110
4.7	-9.5	-9.0	0.28	2.511	0.529	6.143	2.377	6	7516	ZINC000257602911
4.7	-10.5	-10.3	0.13	2.770	0.364	6.077	0.672	6	7517	ZINC000085850882
4.7	-9.9	-9.5	0.21	2.352	0.529	5.160	2.036	6	7518	ZINC000014642599
4.7	-9.5	-9.4	0.14	2.236	0.582	5.049	2.506	6	7519	ZINC000257768738
4.7	-9.8	-9.5	0.26	3.287	0.265	6.502	1.111	6	7520	ZINC000034445678
4.7	-8.4	-8.2	0.13	3.624	0.461	5.051	1.637	6	7521	ZINC000014762745
4.7	-7.7	-7.4	0.19	3.111	1.702	5.266	3.116	6	7522	ZINC000012496184
4.7	-9.9	-9.6	0.24	2.551	0.652	4.269	1.456	6	7523	ZINC000238771781
4.6	-10.3	-9.8	0.26	1.739	0.349	3.750	1.774	6	7524	ZINC000257574919
4.6	-9.9	-9.7	0.13	2.491	0.861	7.723	1.575	6	7525	ZINC000059777175
4.6	-10.7	-10.2	0.27	2.661	0.725	5.704	1.305	6	7526	ZINC000100773101

4.6	-9.6	-9.0	0.36	2.539	0.417	6.026	2.224	6	7527	ZINC000014417206
4.6	-9.3	-9.1	0.15	2.139	0.452	4.299	1.644	6	7528	ZINC000003824124
4.6	-9.8	-9.4	0.24	2.428	0.719	4.791	2.028	6	7529	ZINC000257439653
4.6	-10.1	-9.5	0.32	2.803	0.211	5.935	1.628	6	7530	ZINC000028646753
4.6	-9.2	-8.9	0.21	1.945	0.516	6.417	2.541	6	7531	ZINC000257564220
4.6	-9.7	-9.2	0.31	2.260	0.908	4.959	2.568	6	7532	ZINC000005459396
4.6	-9.3	-8.9	0.27	2.636	0.436	5.921	2.046	6	7533	ZINC000015043410
4.6	-9.7	-9.4	0.19	2.196	1.110	4.847	2.539	6	7534	ZINC000015168919
4.6	-10.1	-10.0	0.11	2.412	0.241	7.253	0.775	6	7535	ZINC000257551726
4.6	-9.3	-9.0	0.18	2.311	0.611	5.379	2.636	6	7536	ZINC000257387410
4.6	-9.9	-9.6	0.21	2.656	0.221	7.727	1.908	6	7537	ZINC000004098720
4.6	-9.4	-9.1	0.23	2.907	0.786	5.810	1.525	6	7538	ZINC000118937343
4.6	-9.3	-9.1	0.12	2.978	0.518	7.399	1.767	6	7539	ZINC000014760567
4.6	-9.3	-9.1	0.16	2.342	0.583	5.554	2.797	6	7540	ZINC000118937368
4.6	-9.5	-9.3	0.12	2.155	0.407	4.528	2.318	6	7541	ZINC000014759031

4.6	-8.7	-8.5	0.12	2.907	1.146	7.332	1.608	6	7542	ZINC000100780949
4.6	-8.8	-8.6	0.11	2.273	0.510	6.203	1.779	6	7543	ZINC000006363551
4.6	-8.9	-8.6	0.18	1.971	0.302	5.299	2.733	6	7544	ZINC000002386791
4.6	-10.3	-9.8	0.27	3.318	0.891	4.625	1.186	6	7545	ZINC000003596884
4.6	-9.5	-9.3	0.13	3.521	0.404	5.785	0.775	6	7546	ZINC000257525946
4.6	-9.2	-8.8	0.20	1.358	0.589	4.248	3.049	6	7547	ZINC000100829907
4.6	-10.1	-9.3	0.35	2.901	0.423	5.927	1.864	6	7548	ZINC000006036327
4.6	-10.3	-9.7	0.35	2.437	0.286	9.040	0.234	6	7549	ZINC000257388902
4.6	-9.8	-9.5	0.15	1.626	0.238	8.816	3.069	6	7550	ZINC000045789238
4.6	-10.3	-9.7	0.37	2.132	0.304	4.928	1.056	6	7551	ZINC000014435560
4.6	-10.5	-9.5	0.50	3.514	0.720	6.339	1.724	6	7552	ZINC000257460909
4.6	-10.5	-9.8	0.39	1.941	0.685	5.498	1.417	6	7553	ZINC000014689169
4.6	-10.5	-10.1	0.18	2.216	0.774	3.043	1.018	6	7554	ZINC000014930841
4.6	-10.0	-9.8	0.12	2.245	0.440	7.274	1.208	6	7555	ZINC000100772326
4.6	-9.5	-9.4	0.11	2.554	0.598	5.683	1.983	6	7556	ZINC000100773466

4.6	-9.6	-9.5	0.11	3.213	0.616	4.479	0.705	6	7557	ZINC000014644687
4.6	-10.1	-9.8	0.20	2.515	0.877	6.848	2.936	6	7558	ZINC000100822251
4.6	-10.2	-10.0	0.17	2.622	0.584	4.100	1.997	6	7559	ZINC000261499073
4.6	-9.8	-9.5	0.15	2.969	0.575	6.426	1.920	6	7560	ZINC000257384361
4.6	-9.9	-9.2	0.31	2.457	0.587	4.594	2.150	6	7561	ZINC000118936886
4.6	-10.0	-9.5	0.25	2.429	0.728	5.347	2.266	6	7562	ZINC000014436807
4.6	-11.7	-11.0	0.35	2.602	0.410	6.793	0.315	6	7563	ZINC000085977714
4.6	-10.0	-8.9	0.52	3.913	0.326	6.238	0.942	6	7564	ZINC000014437253
4.6	-9.2	-8.8	0.23	2.046	0.399	5.781	0.329	6	7565	ZINC000100823159
4.6	-10.4	-9.9	0.32	2.203	0.242	6.075	0.331	6	7566	ZINC000013429511
4.6	-9.1	-8.8	0.20	3.255	0.930	5.224	1.992	6	7567	ZINC000014452747
4.6	-12.3	-11.1	0.62	2.227	0.216	4.847	1.277	6	7568	ZINC000028642721
4.6	-9.8	-9.5	0.31	3.175	0.168	6.217	0.912	6	7569	ZINC000257567288
4.6	-9.3	-8.2	0.48	1.877	0.211	4.705	1.872	6	7570	ZINC000257402167
4.6	-9.9	-9.7	0.15	2.077	0.076	5.300	2.466	6	7571	ZINC000100829056

4.6	-9.5	-9.2	0.18	1.699	0.629	5.528	3.469	6	7572	ZINC000100827843
4.5	-9.9	-9.4	0.33	2.442	0.824	4.691	2.548	6	7573	ZINC000257383376
4.5	-7.8	-7.5	0.19	4.096	0.962	7.264	1.821	6	7574	ZINC000005423072
4.5	-9.1	-9.0	0.09	3.057	0.844	5.201	1.919	6	7575	ZINC000238783414
4.5	-9.8	-9.7	0.07	2.214	0.165	5.386	2.045	6	7576	ZINC000257435156
4.5	-9.3	-9.2	0.14	3.791	0.422	9.030	2.206	6	7577	ZINC000004228267
4.5	-10.4	-10.2	0.25	1.748	0.542	2.435	0.756	6	7578	ZINC000085589035
4.5	-10.0	-9.5	0.27	2.452	1.012	6.154	1.634	6	7579	ZINC000000000706
4.5	-9.1	-8.7	0.27	3.277	0.965	7.621	0.558	6	7580	ZINC000014450979
4.5	-9.6	-9.0	0.29	2.685	0.455	6.139	2.339	6	7581	ZINC000004404494
4.5	-9.6	-9.3	0.23	2.536	0.204	5.946	1.092	6	7582	ZINC000100784087
4.5	-9.2	-9.0	0.19	2.755	0.915	4.576	1.788	6	7583	ZINC000014681025
4.5	-9.5	-9.2	0.22	2.766	0.273	5.919	1.529	6	7584	ZINC000085964220
4.5	-9.1	-8.7	0.25	2.595	0.497	7.824	1.823	6	7585	ZINC000257474406
4.5	-9.9	-9.5	0.20	2.812	0.383	6.138	1.682	6	7586	ZINC000118937378

4.5	-9.6	-9.2	0.24	1.947	0.615	5.062	2.546	6	7587	ZINC000257503167
4.5	-9.4	-9.2	0.15	2.083	0.190	6.177	2.591	6	7588	ZINC000014613245
4.5	-11.5	-10.8	0.50	3.405	0.215	6.012	0.554	6	7589	ZINC000014828693
4.5	-10.4	-10.1	0.26	3.557	0.433	5.800	0.823	6	7590	ZINC000100775755
4.5	-9.1	-8.9	0.20	2.450	0.553	6.925	2.557	6	7591	ZINC000014644887
4.5	-9.9	-9.4	0.72	2.158	0.473	6.766	0.361	6	7592	ZINC000085862077
4.5	-9.1	-8.8	0.18	2.933	0.721	7.388	1.663	6	7593	ZINC000014637748
4.5	-9.3	-8.8	0.33	2.139	0.141	5.961	1.266	6	7594	ZINC000085936060
4.5	-10.0	-9.3	0.34	2.531	0.349	5.601	0.403	6	7595	ZINC000015273921
4.5	-9.9	-9.4	0.30	2.474	0.573	5.368	2.530	6	7596	ZINC000253589673
4.5	-9.7	-9.4	0.20	2.828	0.685	5.409	1.718	6	7597	ZINC000008551144
4.5	-9.4	-9.1	0.18	2.242	0.917	3.772	1.633	6	7598	ZINC000013309935
4.5	-9.6	-9.2	0.32	2.606	0.497	4.330	0.698	6	7599	ZINC000257503968
4.5	-10.3	-9.6	0.31	2.455	0.358	6.385	1.537	6	7600	ZINC000239402956
4.5	-9.2	-9.0	0.11	2.864	0.372	5.781	0.384	6	7601	ZINC000100784081

4.5	-8.5	-8.3	0.10	2.838	0.396	7.217	2.645	6	7602	ZINC000014644883
4.5	-9.1	-8.7	0.31	2.565	0.883	5.174	2.523	6	7603	ZINC000248290294
4.5	-8.4	-8.2	0.18	0.467	0.451	4.707	2.547	6	7604	ZINC000049601654
4.5	-9.9	-9.5	0.23	2.390	0.722	5.155	2.601	6	7605	ZINC000086045279
4.5	-8.2	-7.8	0.29	3.259	0.860	4.169	1.083	6	7606	ZINC000257466462
4.5	-9.3	-9.1	0.13	1.925	0.405	4.388	2.038	6	7607	ZINC000119382108
4.5	-9.3	-9.0	0.16	2.353	0.251	7.020	0.500	6	7608	ZINC000085954739
4.5	-10.0	-9.9	0.07	2.266	0.367	5.101	2.589	6	7609	ZINC000100824422
4.5	-9.6	-9.1	0.25	2.718	0.469	5.286	1.926	6	7610	ZINC000100825863
4.5	-9.4	-8.9	0.26	2.984	0.446	5.047	1.521	6	7611	ZINC000257565420
4.5	-9.5	-8.8	0.33	2.213	0.755	4.941	1.803	6	7612	ZINC000253641054
4.5	-9.8	-9.2	0.35	2.479	0.688	5.851	2.273	6	7613	ZINC000014858260
4.5	-10.9	-10.1	0.39	2.170	0.818	4.909	2.295	6	7614	ZINC000257424029
4.5	-7.9	-7.5	0.29	2.763	1.279	8.746	2.245	6	7615	ZINC000012496719
4.5	-10.5	-9.8	0.36	1.810	0.600	5.654	1.424	6	7616	ZINC000002110660

4.5	-9.5	-8.9	0.30	2.209	0.463	5.755	2.075	6	7617	ZINC000257383049
4.5	-9.4	-9.1	0.15	2.254	0.588	3.770	0.787	6	7618	ZINC000257411452
4.5	-10.5	-9.8	0.37	2.616	0.243	5.208	1.305	6	7619	ZINC000100783476
4.5	-8.3	-7.8	0.23	4.511	0.921	6.624	2.275	6	7620	ZINC000053298354
4.5	-9.2	-9.0	0.17	2.517	0.634	5.103	2.225	6	7621	ZINC000118937337
4.5	-9.5	-9.3	0.25	2.714	0.735	6.084	1.596	6	7622	ZINC000034944434
4.5	-9.6	-9.0	0.25	2.399	0.729	4.531	1.855	6	7623	ZINC000004096611
4.5	-8.6	-8.4	0.14	2.030	0.374	4.848	2.388	6	7624	ZINC000085598416
4.5	-9.3	-8.9	0.22	3.102	0.462	6.731	1.843	6	7625	ZINC000045337018
4.5	-8.1	-7.9	0.15	2.439	0.468	6.857	3.708	6	7626	ZINC000014708529
4.5	-8.8	-8.6	0.15	2.421	0.296	5.709	1.964	6	7627	ZINC000015257532
4.5	-9.5	-9.2	0.30	2.402	0.994	5.509	3.750	6	7628	ZINC000012496906
4.5	-10.4	-9.7	0.37	2.689	0.194	4.596	0.822	6	7629	ZINC000257510389
4.5	-10.0	-9.7	0.18	3.977	0.822	7.009	1.333	6	7630	ZINC000094437783
4.5	-9.2	-8.9	0.20	2.488	0.480	6.224	1.586	6	7631	ZINC000257430035

4.5	-9.4	-8.9	0.25	1.844	0.243	5.151	1.586	6	7632	ZINC000100772660
4.5	-9.1	-8.7	0.22	3.246	0.551	5.914	0.855	6	7633	ZINC000165071264
4.5	-9.5	-9.3	0.19	2.031	0.416	5.002	2.190	6	7634	ZINC000257476746
4.5	-10.1	-9.6	0.30	2.979	0.910	5.806	2.027	6	7635	ZINC000002557433
4.5	-9.6	-9.2	0.21	2.558	0.346	5.505	1.128	6	7636	ZINC000257398588
4.5	-8.6	-8.2	0.26	2.440	0.311	5.356	1.884	6	7637	ZINC000257789846
4.5	-9.1	-8.7	0.33	3.220	0.949	6.619	0.953	6	7638	ZINC000014819612
4.5	-9.5	-9.2	0.12	3.013	0.422	7.327	2.245	6	7639	ZINC000012153137
4.5	-10.0	-9.2	0.35	2.778	0.352	5.528	0.735	6	7640	ZINC000014618541
4.5	-9.9	-9.6	0.20	2.333	0.495	6.401	2.775	6	7641	ZINC000257513739
4.5	-9.4	-8.8	0.26	2.025	0.570	5.644	1.931	6	7642	ZINC000104351592
4.4	-9.2	-8.8	0.22	1.237	0.435	5.535	3.734	6	7643	ZINC000100829904
4.4	-10.5	-9.8	0.35	2.142	0.780	5.860	0.935	6	7644	ZINC000014689166
4.4	-9.3	-8.9	0.18	2.345	0.586	4.517	1.682	6	7645	ZINC000002516840
4.4	-9.3	-9.1	0.18	1.842	0.438	5.324	2.499	6	7646	ZINC000257411453

4.4	-10.4	-9.9	0.27	3.090	1.387	5.371	2.631	6	7647	ZINC000095619148
4.4	-9.3	-8.9	0.20	2.375	0.405	6.574	2.088	6	7648	ZINC000257411451
4.4	-9.8	-9.1	0.40	3.375	0.235	6.903	0.745	6	7649	ZINC000040164493
4.4	-9.2	-8.9	0.19	2.244	0.555	5.922	2.645	6	7650	ZINC000005758832
4.4	-9.4	-9.0	0.30	3.328	0.842	5.354	1.521	6	7651	ZINC000031705519
4.4	-10.0	-9.7	0.13	2.372	0.698	5.472	2.282	6	7652	ZINC000013547326
4.4	-8.7	-8.6	0.05	1.332	0.648	4.926	2.011	6	7653	ZINC000011612826
4.4	-8.5	-8.1	0.20	1.963	1.029	6.398	4.011	6	7654	ZINC000014708514
4.4	-10.9	-10.5	0.32	2.820	1.197	5.446	2.237	6	7655	ZINC000013376905
4.4	-9.5	-9.0	0.28	2.897	0.524	6.584	1.786	6	7656	ZINC000030729704
4.4	-9.1	-8.8	0.19	2.208	0.299	5.875	1.838	6	7657	ZINC000261496326
4.4	-8.0	-7.8	0.17	1.873	1.412	4.523	2.660	6	7658	ZINC000003830993
4.4	-9.5	-9.2	0.21	3.084	0.237	6.858	0.953	6	7659	ZINC000085589300
4.4	-9.6	-9.2	0.26	2.234	0.740	6.435	2.081	6	7660	ZINC000033835364
4.4	-9.8	-9.4	0.34	2.384	0.651	5.006	2.164	6	7661	ZINC000040165394

4.4	-9.3	-9.2	0.07	3.455	0.623	7.566	1.630	6	7662	ZINC000257481911
4.4	-9.9	-9.4	0.28	2.869	0.976	6.403	2.308	6	7663	ZINC000100825730
4.4	-9.0	-8.8	0.13	2.032	0.366	4.755	2.179	6	7664	ZINC000257474405
4.4	-9.9	-9.5	0.26	3.270	0.330	6.436	2.158	6	7665	ZINC000100257564
4.4	-8.2	-7.7	0.29	2.992	0.699	4.033	0.968	6	7666	ZINC000014504992
4.4	-9.7	-9.1	0.34	2.777	0.310	5.519	1.023	6	7667	ZINC000118913640
4.4	-9.3	-9.0	0.18	2.094	0.415	5.574	2.158	6	7668	ZINC000119381945
4.4	-9.3	-9.1	0.19	3.450	0.376	5.239	1.372	6	7669	ZINC000040164385
4.4	-9.4	-9.2	0.14	2.041	0.419	4.693	2.318	6	7670	ZINC000257506115
4.4	-11.4	-9.6	0.82	3.493	0.886	6.278	1.679	6	7671	ZINC000013398322
4.4	-10.1	-9.5	0.27	2.170	0.428	4.434	1.606	6	7672	ZINC000100773576
4.4	-9.4	-9.0	0.24	1.698	0.454	4.340	2.152	6	7673	ZINC000100772656
4.4	-10.9	-10.6	0.31	3.829	0.733	6.913	0.439	6	7674	ZINC000014859662
4.4	-10.1	-9.3	0.42	1.541	0.524	4.747	1.998	6	7675	ZINC000100828449
4.4	-7.7	-7.3	0.17	2.093	0.336	6.049	2.445	6	7676	ZINC000005733763

4.4	-8.8	-8.5	0.17	1.999	0.417	5.120	2.528	6	7677	ZINC000014645202
4.4	-9.6	-9.1	0.25	3.188	0.474	6.459	2.669	6	7678	ZINC000257397623
4.4	-9.4	-9.1	0.19	2.551	0.254	6.951	1.164	6	7679	ZINC000257423878
4.4	-9.7	-9.3	0.32	2.719	0.358	6.626	1.762	6	7680	ZINC000257556654
4.4	-9.0	-8.8	0.15	2.719	0.771	5.306	2.324	6	7681	ZINC000257402549
4.4	-8.2	-7.8	0.24	3.212	0.736	4.170	0.816	6	7682	ZINC000014504989
4.4	-9.3	-8.7	0.33	2.160	0.679	4.571	2.368	6	7683	ZINC000004215814
4.4	-9.4	-9.3	0.15	2.196	0.537	5.052	2.518	6	7684	ZINC000100779150
4.4	-9.8	-9.6	0.11	2.289	0.295	4.844	2.130	6	7685	ZINC000100824420
4.4	-9.2	-9.0	0.15	2.058	0.522	5.463	2.277	6	7686	ZINC000119382331
4.4	-10.0	-9.5	0.28	2.461	0.823	4.830	1.724	6	7687	ZINC000012153857
4.4	-9.7	-9.3	0.22	2.153	0.703	4.370	2.450	6	7688	ZINC000014757135
4.4	-8.7	-8.5	0.14	2.136	0.301	7.007	2.107	6	7689	ZINC000014680909
4.4	-10.1	-9.4	0.39	1.271	0.516	4.345	2.261	6	7690	ZINC000100828453
4.4	-11.3	-10.9	0.29	1.934	0.229	6.596	1.554	6	7691	ZINC000257502005

4.4	-10.0	-9.7	0.24	2.403	0.690	3.431	0.620	6	7692	ZINC000261499074
4.4	-10.0	-9.7	0.27	2.249	0.279	4.948	1.382	6	7693	ZINC000004692015
4.4	-10.5	-10.0	0.26	3.251	1.165	7.490	2.355	6	7694	ZINC000253477262
4.4	-9.1	-9.0	0.07	3.068	0.208	5.668	0.672	6	7695	ZINC000257515726
4.4	-8.1	-7.5	0.25	3.637	0.544	5.384	2.103	6	7696	ZINC000000899200
4.4	-9.4	-9.1	0.15	2.675	0.564	6.543	2.650	6	7697	ZINC000014930807
4.4	-8.2	-7.8	0.34	3.277	0.869	4.345	1.010	6	7698	ZINC000014504985
4.4	-9.8	-8.8	0.65	2.293	0.329	5.505	1.746	6	7699	ZINC000257423772
4.4	-9.2	-8.9	0.19	1.351	0.376	7.094	3.648	6	7700	ZINC000257525364
4.4	-9.5	-9.0	0.30	2.319	0.384	6.599	3.289	6	7701	ZINC000015060715
4.4	-8.3	-8.2	0.10	2.593	0.787	5.748	1.930	6	7702	ZINC000085833847
4.4	-8.5	-8.4	0.07	3.581	1.025	6.194	1.863	6	7703	ZINC000001575028
4.4	-10.5	-9.7	0.40	2.350	0.599	5.106	1.568	6	7704	ZINC000014585988
4.4	-8.5	-8.4	0.07	1.581	0.508	4.981	2.176	6	7705	ZINC000034173086
4.4	-9.5	-9.0	0.32	3.235	1.268	4.690	1.790	6	7706	ZINC000085964214

4.4	-9.9	-9.5	0.23	1.912	0.444	6.370	2.103	6	7707	ZINC000015150586
4.4	-9.8	-9.5	0.25	3.167	0.656	5.884	1.814	6	7708	ZINC000238764738
4.4	-9.3	-9.2	0.09	3.116	0.899	5.390	1.895	6	7709	ZINC000100778557
4.4	-9.9	-9.5	0.24	2.529	0.379	6.809	1.878	6	7710	ZINC000253590100
4.4	-9.6	-9.3	0.24	2.559	0.906	5.524	2.084	6	7711	ZINC000015168921
4.4	-9.4	-9.0	0.22	2.697	0.334	6.425	1.267	6	7712	ZINC000012496510
4.4	-9.1	-8.9	0.12	2.575	0.524	6.806	2.142	6	7713	ZINC000014691744
4.4	-9.7	-9.3	0.27	2.697	0.466	5.360	0.994	6	7714	ZINC000256543466
4.4	-11.5	-11.0	0.26	2.859	0.585	5.657	1.567	6	7715	ZINC000257727950
4.4	-9.4	-8.8	0.46	2.461	0.457	5.077	2.099	6	7716	ZINC000013311815
4.4	-9.2	-8.8	0.21	2.356	0.554	7.517	0.403	6	7717	ZINC000014727335
4.4	-9.3	-9.0	0.27	1.820	0.379	4.888	3.365	6	7718	ZINC000059775273
4.4	-8.2	-7.6	0.36	3.591	0.951	4.946	0.918	6	7719	ZINC000257466461
4.4	-9.8	-9.5	0.24	2.666	0.553	6.494	1.344	6	7720	ZINC000118928395
4.4	-9.6	-9.2	0.29	3.255	0.344	5.722	1.085	6	7721	ZINC000257567286

4.4	-9.5	-9.1	0.23	3.056	0.107	8.455	0.280	6	7722	ZINC000257476013
4.3	-9.7	-9.4	0.17	2.068	0.218	6.091	1.322	6	7723	ZINC000100827233
4.3	-10.2	-9.7	0.29	2.571	0.550	5.344	3.164	6	7724	ZINC000257388901
4.3	-11.7	-11.2	0.30	2.601	0.884	5.387	1.161	6	7725	ZINC000095619885
4.3	-10.1	-9.8	0.17	2.701	0.079	6.436	2.227	6	7726	ZINC000118937153
4.3	-9.2	-8.9	0.16	1.414	0.643	4.064	3.030	6	7727	ZINC000257428933
4.3	-11.5	-11.0	0.42	2.106	0.440	6.078	0.627	6	7728	ZINC000100826924
4.3	-9.1	-8.9	0.17	2.132	0.743	6.108	1.911	6	7729	ZINC000256235048
4.3	-9.1	-8.5	0.35	3.263	0.672	6.733	0.312	6	7730	ZINC000261498131
4.3	-10.1	-9.7	0.27	2.838	1.012	4.650	2.054	6	7731	ZINC000257373886
4.3	-10.5	-9.7	0.40	2.660	0.186	5.171	1.273	6	7732	ZINC000100783482
4.3	-8.9	-8.6	0.21	2.228	0.339	5.045	1.296	6	7733	ZINC000059772455
4.3	-9.8	-9.4	0.20	3.172	0.645	5.737	1.432	6	7734	ZINC000257565421
4.3	-9.4	-8.8	0.34	2.515	0.445	4.382	2.151	6	7735	ZINC000015257341
4.3	-8.7	-8.5	0.20	3.593	0.927	5.152	1.725	6	7736	ZINC000014819503

4.3	-10.1	-9.4	0.34	1.316	0.341	4.076	2.096	6	7737	ZINC000257795040
4.3	-9.2	-8.8	0.26	1.993	0.166	4.820	0.342	6	7738	ZINC000257552401
4.3	-10.4	-10.1	0.19	2.048	0.168	5.237	2.973	6	7739	ZINC000257546502
4.3	-9.6	-9.1	0.24	1.555	0.186	2.285	0.416	6	7740	ZINC000100827839
4.3	-11.7	-11.1	0.32	2.695	0.985	5.591	1.244	6	7741	ZINC000095619883
4.3	-9.1	-8.8	0.14	2.610	0.219	4.920	1.359	6	7742	ZINC000257704825
4.3	-9.7	-9.4	0.17	3.446	0.591	7.192	1.754	6	7743	ZINC000014760569
4.3	-8.2	-8.0	0.21	1.285	0.555	4.472	2.541	6	7744	ZINC000013411715
4.3	-10.4	-9.7	0.38	2.614	0.223	5.173	1.439	6	7745	ZINC000257510387
4.3	-8.9	-8.5	0.21	3.015	0.541	6.286	2.233	6	7746	ZINC000014708521
4.3	-9.8	-9.7	0.21	1.992	0.480	5.553	2.695	6	7747	ZINC000085664588
4.3	-9.3	-8.8	0.37	2.509	0.692	5.368	2.212	6	7748	ZINC000006491648
4.3	-9.9	-9.5	0.32	2.261	1.456	4.211	1.876	6	7749	ZINC000257560455
4.3	-8.3	-8.2	0.15	1.936	0.487	4.559	1.897	6	7750	ZINC000100827204
4.3	-10.3	-10.0	0.19	2.915	0.869	7.866	1.808	6	7751	ZINC000014760860

4.3	-9.3	-9.2	0.12	2.964	0.857	5.296	1.790	6	7752	ZINC000257411659
4.3	-9.8	-9.7	0.07	2.349	0.944	5.115	2.548	6	7753	ZINC000257483366
4.3	-9.3	-8.9	0.20	2.537	0.691	4.700	1.581	6	7754	ZINC000040164325
4.3	-9.5	-9.3	0.12	2.420	0.436	5.017	2.118	6	7755	ZINC000257549391
4.3	-9.9	-9.6	0.23	1.844	0.965	4.176	0.802	6	7756	ZINC000100776822
4.3	-10.3	-9.7	0.29	2.703	0.591	4.766	1.504	6	7757	ZINC000001618681
4.3	-10.1	-9.8	0.14	2.632	0.181	5.404	2.371	6	7758	ZINC000257560115
4.3	-9.4	-9.4	0.00	2.014	0.424	5.226	2.763	5	7759	ZINC000257768739
4.3	-10.2	-9.7	0.25	1.855	0.394	5.369	2.974	6	7760	ZINC000015150610
4.3	-9.9	-9.7	0.15	2.349	0.629	5.310	2.761	6	7761	ZINC000257555127
4.3	-10.1	-9.8	0.15	2.739	0.098	7.394	1.803	6	7762	ZINC000118937152
4.3	-10.1	-9.8	0.16	2.695	0.079	6.442	2.226	6	7763	ZINC000257560114
4.3	-10.3	-9.6	0.42	2.974	0.517	6.328	0.656	6	7764	ZINC000014952338
4.3	-9.6	-9.1	0.23	2.616	0.393	4.640	1.576	6	7765	ZINC000257475564
4.3	-9.9	-9.4	0.39	2.923	1.678	5.471	1.815	6	7766	ZINC000257560454

4.3	-9.6	-9.2	0.26	2.294	0.525	5.276	1.569	6	7767	ZINC000257539986
4.3	-9.2	-9.0	0.13	2.509	0.743	7.045	2.964	6	7768	ZINC000014760571
4.3	-10.5	-10.0	0.31	2.216	0.930	4.297	1.836	6	7769	ZINC000257387061
4.3	-11.0	-10.5	0.39	2.746	0.305	6.957	1.020	6	7770	ZINC000257491282
4.3	-9.4	-8.6	0.39	2.073	0.521	6.310	0.946	6	7771	ZINC000014760677
4.3	-9.1	-8.7	0.31	1.938	0.283	4.558	1.992	6	7772	ZINC000014858255
4.3	-9.7	-9.0	0.32	2.153	0.379	6.201	2.222	6	7773	ZINC000014760413
4.3	-9.3	-9.2	0.08	2.834	0.932	3.821	1.290	6	7774	ZINC000014814264
4.3	-9.1	-8.8	0.16	2.683	0.706	5.631	2.286	6	7775	ZINC000118936789
4.3	-10.5	-10.0	0.35	2.103	0.539	3.537	1.205	6	7776	ZINC000257553435
4.3	-10.0	-9.8	0.22	1.997	0.901	4.098	2.914	6	7777	ZINC000257774398
4.3	-7.8	-7.5	0.19	4.414	1.312	9.547	0.733	6	7778	ZINC000001531698
4.3	-9.9	-9.4	0.41	3.023	1.777	5.605	1.964	6	7779	ZINC000100776819
4.3	-9.3	-8.9	0.38	2.273	0.299	6.750	1.441	6	7780	ZINC000013458990
4.3	-9.7	-9.5	0.15	3.161	0.891	5.978	2.267	6	7781	ZINC000040164361

4.3	-9.7	-9.1	0.31	2.879	0.276	6.402	1.931	6	7782	ZINC000003875383
4.3	-9.5	-9.1	0.26	2.435	0.531	5.904	2.607	6	7783	ZINC000257475563
4.3	-10.2	-9.5	0.45	2.586	0.403	5.607	0.668	6	7784	ZINC000257389648
4.3	-11.2	-10.6	0.29	2.988	0.547	7.081	0.226	6	7785	ZINC000257459423
4.3	-9.3	-8.8	0.28	1.380	0.600	3.828	2.984	6	7786	ZINC000257525361
4.3	-9.8	-9.2	0.35	2.802	0.635	6.604	1.838	6	7787	ZINC000004096711
4.3	-9.9	-9.5	0.26	1.839	0.958	4.138	0.774	6	7788	ZINC000100776820
4.3	-8.9	-8.5	0.27	2.681	0.637	5.519	2.128	6	7789	ZINC000004539100
4.3	-10.4	-10.0	0.29	1.849	0.599	5.556	2.484	6	7790	ZINC000118937399
4.3	-10.0	-9.2	0.41	2.114	0.316	5.263	1.750	6	7791	ZINC000257420086
4.3	-10.4	-9.4	0.58	2.550	0.661	5.191	0.869	6	7792	ZINC000118916857
4.3	-8.9	-8.6	0.21	2.344	0.078	5.273	2.405	6	7793	ZINC000005854565
4.3	-8.2	-7.8	0.35	2.462	1.803	4.655	2.999	6	7794	ZINC000085552312
4.3	-9.9	-9.6	0.24	1.839	0.961	4.141	0.776	6	7795	ZINC000104359826
4.3	-9.7	-9.6	0.12	2.501	0.346	7.484	3.429	6	7796	ZINC000100827835

4.3	-9.3	-9.1	0.15	2.070	0.263	6.042	1.038	6	7797	ZINC000253374245
4.3	-9.2	-8.8	0.18	1.607	0.512	6.155	3.285	6	7798	ZINC000100828377
4.3	-10.4	-10.2	0.12	2.277	0.079	4.692	0.976	6	7799	ZINC000014759040
4.3	-9.8	-9.3	0.30	1.939	0.846	4.480	1.372	6	7800	ZINC000100782657
4.3	-11.8	-10.7	0.54	2.335	0.895	3.180	0.770	6	7801	ZINC000021981190
4.3	-10.7	-9.9	0.37	1.945	0.134	6.120	1.480	6	7802	ZINC000100772848
4.3	-9.9	-9.4	0.37	2.130	1.087	4.588	1.356	6	7803	ZINC000100776817
4.3	-10.1	-9.4	0.36	1.657	0.421	4.573	1.799	6	7804	ZINC000100828448
4.3	-8.7	-8.5	0.12	2.690	0.428	7.351	2.450	6	7805	ZINC000014757226
4.3	-9.3	-9.0	0.23	2.973	0.999	5.840	3.191	6	7806	ZINC000012494535
4.3	-11.8	-10.5	0.62	4.203	1.221	5.804	1.933	6	7807	ZINC000021981188
4.3	-9.3	-9.0	0.21	2.942	0.729	8.249	2.859	6	7808	ZINC000014491684
4.2	-9.6	-9.4	0.12	2.580	0.249	7.507	1.845	6	7809	ZINC000100771845
4.2	-9.6	-9.3	0.18	1.878	0.521	4.115	1.069	6	7810	ZINC000014759052
4.2	-9.2	-8.8	0.25	2.287	0.557	5.192	2.035	6	7811	ZINC000014658120

4.2	-9.2	-9.1	0.12	2.889	0.393	5.792	1.336	6	7812	ZINC000257411658
4.2	-9.7	-9.5	0.14	3.393	1.435	5.795	2.492	6	7813	ZINC000238751323
4.2	-8.8	-8.5	0.15	2.411	0.511	6.519	2.278	6	7814	ZINC000014727333
4.2	-8.3	-8.2	0.08	2.277	0.349	4.288	2.022	6	7815	ZINC000033832262
4.2	-8.8	-8.4	0.29	1.805	0.332	4.501	2.514	6	7816	ZINC000014437039
4.2	-9.2	-8.6	0.30	2.412	0.381	5.741	1.831	6	7817	ZINC000015268302
4.2	-9.4	-9.0	0.20	2.574	0.523	6.250	2.159	6	7818	ZINC000004683730
4.2	-9.6	-9.4	0.11	1.980	0.563	3.692	2.070	6	7819	ZINC000257408376
4.2	-9.1	-8.8	0.16	2.466	0.410	5.456	1.303	6	7820	ZINC000257523877
4.2	-10.2	-9.7	0.29	2.028	0.278	4.779	0.467	6	7821	ZINC000257420087
4.2	-10.2	-9.5	0.34	1.910	0.639	4.558	1.259	6	7822	ZINC000014759048
4.2	-10.1	-9.6	0.33	2.030	0.708	4.833	2.846	6	7823	ZINC000257383378
4.2	-10.5	-9.7	0.39	2.648	0.225	5.337	1.074	6	7824	ZINC000100783472
4.2	-9.6	-9.2	0.25	2.148	0.329	4.074	1.708	6	7825	ZINC000100774372
4.2	-10.1	-9.5	0.35	1.265	0.463	4.033	2.130	6	7826	ZINC000100828451

4.2	-10.5	-10.3	0.19	2.859	0.385	5.909	0.943	6	7827	ZINC000085862556
4.2	-7.6	-7.4	0.12	2.221	0.274	5.515	2.083	6	7828	ZINC000013386178
4.2	-9.6	-9.4	0.15	2.895	0.242	5.870	0.849	6	7829	ZINC000257621273
4.2	-8.4	-8.3	0.11	3.169	1.196	5.479	2.262	6	7830	ZINC000003870838
4.2	-9.9	-9.7	0.11	2.939	0.081	8.265	0.186	6	7831	ZINC000257476012
4.2	-10.7	-10.2	0.25	1.588	0.134	3.915	0.965	6	7832	ZINC000257428875
4.2	-9.6	-9.4	0.21	3.421	1.146	7.098	0.978	6	7833	ZINC000261497818
4.2	-9.3	-9.1	0.12	2.204	0.936	4.615	2.390	6	7834	ZINC000257679253
4.2	-8.0	-7.5	0.24	4.444	0.762	5.940	0.432	6	7835	ZINC000014644868
4.2	-9.8	-9.3	0.27	4.105	0.389	6.378	1.333	6	7836	ZINC000014502292
4.2	-9.7	-8.9	0.53	3.461	0.506	6.251	0.599	6	7837	ZINC000118937355
4.2	-9.4	-9.2	0.15	2.464	0.654	6.399	2.632	6	7838	ZINC000257484778
4.2	-9.5	-9.1	0.26	2.254	0.494	4.580	1.023	6	7839	ZINC000118937007
4.2	-11.3	-10.5	0.46	2.450	0.174	6.562	0.418	6	7840	ZINC000014829499
4.2	-9.6	-9.2	0.25	2.400	0.334	6.355	2.067	6	7841	ZINC000257406847

4.2	-8.2	-8.0	0.07	2.325	0.329	6.196	2.785	6	7842	ZINC000085933544
4.2	-9.9	-9.6	0.16	2.296	0.712	5.633	2.534	6	7843	ZINC000257450975
4.2	-10.0	-9.6	0.23	2.061	0.358	5.525	2.272	6	7844	ZINC000257430577
4.2	-9.0	-8.7	0.23	2.431	0.225	5.262	1.957	6	7845	ZINC000085863067
4.2	-9.8	-9.7	0.13	2.322	0.964	5.703	2.486	6	7846	ZINC000257527572
4.2	-10.0	-9.7	0.22	1.826	1.044	4.068	2.820	6	7847	ZINC000014436733
4.2	-8.8	-8.5	0.22	1.948	0.587	6.019	2.475	6	7848	ZINC000257470253
4.2	-10.0	-9.8	0.11	2.650	0.189	6.302	2.378	6	7849	ZINC000118937154
4.2	-9.7	-9.4	0.21	2.372	0.745	5.792	1.595	6	7850	ZINC000014681077
4.2	-9.4	-9.2	0.19	2.424	0.680	6.209	2.451	6	7851	ZINC000003978489
4.2	-9.5	-9.1	0.23	2.239	0.492	7.577	2.953	6	7852	ZINC000014684664
4.2	-9.8	-9.1	0.35	2.043	0.701	4.110	2.448	6	7853	ZINC000086052089
4.2	-10.3	-9.7	0.32	1.660	0.254	2.849	0.554	6	7854	ZINC000015115502
4.2	-10.1	-9.8	0.16	2.587	0.123	5.692	1.041	6	7855	ZINC000257435407
4.2	-10.1	-9.7	0.27	2.665	0.562	7.376	1.737	6	7856	ZINC000257616576

4.2	-9.7	-9.5	0.17	2.487	0.422	4.707	1.560	6	7857	ZINC000040164357
4.2	-10.5	-10.3	0.11	1.874	0.124	6.410	0.582	6	7858	ZINC000100826633
4.2	-10.0	-9.9	0.06	3.029	0.474	6.142	1.987	6	7859	ZINC000040164319
4.2	-9.8	-9.3	0.24	2.390	0.413	5.845	3.077	6	7860	ZINC000045287165
4.2	-10.6	-9.9	0.44	1.999	0.610	5.371	1.000	6	7861	ZINC000095619153
4.2	-9.7	-9.3	0.22	2.512	0.562	5.958	0.814	6	7862	ZINC000014503434
4.2	-9.2	-8.8	0.22	1.550	0.848	4.524	3.030	6	7863	ZINC000257525363
4.2	-7.9	-7.7	0.15	2.549	0.632	4.699	1.910	6	7864	ZINC000001614079
4.2	-9.3	-9.0	0.23	3.374	0.440	6.890	2.260	6	7865	ZINC000015062951
4.2	-10.0	-9.7	0.17	2.294	0.465	7.886	1.450	6	7866	ZINC000257561217
4.2	-7.7	-7.4	0.16	1.767	0.116	3.307	2.380	6	7867	ZINC000006018556
4.2	-8.9	-8.4	0.39	2.372	0.972	5.217	2.157	6	7868	ZINC000014681163
4.2	-10.1	-9.7	0.18	2.729	0.640	6.623	3.301	6	7869	ZINC000257435898
4.2	-10.1	-9.5	0.36	1.515	0.590	4.226	2.203	6	7870	ZINC000257795030
4.2	-9.7	-9.5	0.13	1.772	0.613	3.748	2.665	6	7871	ZINC000095620826

4.2	-9.7	-9.3	0.35	1.707	0.530	4.399	1.747	6	7872	ZINC000014759046
4.2	-10.1	-9.4	0.39	1.641	0.604	4.469	1.944	6	7873	ZINC000257795037
4.2	-10.2	-9.8	0.23	2.573	0.637	4.957	1.678	6	7874	ZINC000257408375
4.2	-9.4	-9.0	0.21	2.378	0.429	5.461	2.648	6	7875	ZINC000257444907
4.2	-8.6	-8.3	0.32	2.041	0.490	2.696	0.658	6	7876	ZINC000086010712
4.2	-10.5	-10.0	0.27	2.399	0.772	4.614	2.051	6	7877	ZINC000257410708
4.2	-9.6	-9.4	0.18	2.715	0.952	4.992	2.340	6	7878	ZINC000014814315
4.2	-10.9	-10.4	0.45	1.852	0.807	4.521	2.183	6	7879	ZINC000013484753
4.2	-10.9	-9.9	0.55	2.575	0.838	4.865	2.519	6	7880	ZINC000000899909
4.2	-10.0	-9.8	0.17	1.928	0.508	5.018	3.319	6	7881	ZINC000257559679
4.2	-10.0	-9.4	0.47	3.116	0.364	6.422	0.448	6	7882	ZINC000257525013
4.2	-10.6	-9.6	0.46	3.139	0.586	6.218	1.249	6	7883	ZINC000253387561
4.2	-10.0	-9.7	0.13	2.710	0.065	6.522	2.280	6	7884	ZINC000257560116
4.2	-10.1	-9.8	0.18	2.501	0.420	4.866	2.211	6	7885	ZINC000100778483
4.2	-9.7	-9.6	0.09	2.077	0.236	2.917	0.348	6	7886	ZINC000014757131

4.2	-10.0	-9.7	0.22	2.344	0.743	6.178	3.039	6	7887	ZINC000257497573
4.2	-8.6	-8.4	0.08	2.123	0.434	3.813	1.498	6	7888	ZINC000014681012
4.2	-9.4	-9.0	0.26	2.606	0.776	8.275	0.508	6	7889	ZINC000005854545
4.2	-9.9	-9.1	0.41	2.779	0.809	7.210	0.702	6	7890	ZINC000006036223
4.2	-9.0	-8.8	0.14	2.577	0.225	4.907	1.819	6	7891	ZINC000014680917
4.1	-10.1	-9.6	0.33	2.172	0.122	4.655	1.553	6	7892	ZINC000257394470
4.1	-9.9	-9.8	0.11	1.516	0.977	3.550	2.211	6	7893	ZINC000257483640
4.1	-9.5	-9.1	0.28	3.049	0.538	6.605	0.268	6	7894	ZINC000147974383
4.1	-9.2	-9.0	0.17	2.989	0.192	5.185	1.831	6	7895	ZINC000014645206
4.1	-8.8	-8.5	0.20	2.130	0.146	5.202	1.262	6	7896	ZINC000100822480
4.1	-9.0	-8.8	0.13	2.960	0.344	6.512	1.843	6	7897	ZINC000015257344
4.1	-9.1	-8.9	0.18	2.915	0.520	6.749	2.500	6	7898	ZINC000257377356
4.1	-10.0	-9.7	0.22	1.920	1.021	4.191	2.577	6	7899	ZINC000200468557
4.1	-9.3	-9.1	0.15	1.996	0.304	7.077	2.615	6	7900	ZINC000034374848
4.1	-10.4	-10.0	0.23	4.007	0.576	7.229	2.029	6	7901	ZINC000015202158

4.1	-9.5	-9.1	0.20	3.690	0.488	6.072	1.858	6	7902	ZINC000012495981
4.1	-9.5	-9.1	0.26	3.202	1.155	5.397	2.726	6	7903	ZINC000040164400
4.1	-9.5	-9.3	0.13	2.459	0.317	5.227	2.424	6	7904	ZINC000040164358
4.1	-9.3	-9.0	0.20	2.271	0.342	8.492	0.253	6	7905	ZINC000015150601
4.1	-10.2	-9.8	0.29	1.895	1.059	4.625	1.847	6	7906	ZINC000100782626
4.1	-9.5	-9.3	0.12	3.294	0.439	7.053	1.395	6	7907	ZINC000003978459
4.1	-9.9	-9.5	0.18	2.291	0.245	4.686	2.621	6	7908	ZINC000014757133
4.1	-10.4	-10.0	0.25	1.891	0.642	4.234	2.134	6	7909	ZINC000253505157
4.1	-10.5	-9.9	0.43	2.708	0.315	5.527	0.516	6	7910	ZINC000015268477
4.1	-9.5	-9.1	0.22	3.557	0.846	5.879	2.036	6	7911	ZINC000261495063
4.1	-9.2	-9.1	0.07	2.291	1.218	5.757	2.840	6	7912	ZINC000410428650
4.1	-9.7	-9.4	0.21	2.819	0.352	9.141	2.558	6	7913	ZINC000014644689
4.1	-10.3	-9.8	0.36	1.827	0.608	4.501	2.419	6	7914	ZINC000085862453
4.1	-9.3	-9.1	0.13	2.090	0.264	6.243	2.604	6	7915	ZINC000012494068
4.1	-9.5	-8.9	0.36	2.148	0.419	5.219	1.896	6	7916	ZINC000257423773

4.1	-9.6	-9.4	0.19	2.478	0.413	8.014	1.820	6	7917	ZINC000238748266
4.1	-10.4	-10.0	0.32	2.596	0.712	3.670	0.675	6	7918	ZINC000059211654
4.1	-8.4	-8.3	0.09	4.058	0.256	6.499	0.426	6	7919	ZINC000014648502
4.1	-9.8	-9.3	0.25	3.098	1.051	5.300	2.140	6	7920	ZINC000040165506
4.1	-10.2	-9.8	0.27	1.780	0.953	4.484	1.655	6	7921	ZINC000253390104
4.1	-10.2	-9.8	0.28	1.814	0.931	4.499	1.640	6	7922	ZINC000014584577
4.1	-9.7	-9.5	0.17	2.737	0.710	6.944	3.128	6	7923	ZINC000012153179
4.1	-10.3	-9.7	0.37	3.023	1.072	4.137	1.775	6	7924	ZINC000015104592
4.1	-10.1	-9.7	0.21	2.055	0.357	5.064	1.941	6	7925	ZINC000100825433
4.1	-9.9	-9.8	0.07	2.742	0.743	5.842	2.282	6	7926	ZINC000030729371
4.1	-10.4	-9.6	0.44	2.585	0.328	4.914	2.042	6	7927	ZINC000257521373
4.1	-9.9	-9.8	0.07	2.196	0.127	5.113	0.723	6	7928	ZINC000257503168
4.1	-9.5	-9.2	0.14	2.267	0.757	4.207	1.733	6	7929	ZINC000014680901
4.1	-9.7	-9.4	0.20	2.601	0.498	4.626	1.555	6	7930	ZINC000257565422
4.1	-9.5	-8.9	0.40	2.833	0.940	5.353	2.403	6	7931	ZINC000261495617

4.1	-9.4	-8.9	0.34	1.982	0.374	4.259	1.612	6	7932	ZINC000257423876
4.1	-9.9	-9.5	0.22	2.013	0.557	4.227	1.749	6	7933	ZINC000257427194
4.1	-10.1	-9.3	0.41	3.468	0.765	6.092	1.462	6	7934	ZINC000256414364
4.1	-9.5	-9.2	0.16	3.213	0.897	5.699	2.425	6	7935	ZINC000118913621
4.1	-10.5	-9.7	0.39	2.421	0.452	4.662	1.702	6	7936	ZINC000100783477
4.1	-9.3	-8.9	0.25	2.233	0.554	5.256	2.167	6	7937	ZINC000257387095
4.1	-9.0	-8.8	0.15	2.721	0.650	5.397	1.509	6	7938	ZINC000014645917
4.1	-9.4	-9.2	0.14	2.930	0.577	8.735	2.947	6	7939	ZINC000009212428
4.1	-10.3	-10.0	0.22	3.766	0.297	6.141	1.173	6	7940	ZINC000015147372
4.1	-9.4	-9.0	0.21	2.761	0.875	4.993	2.036	6	7941	ZINC000261496045
4.1	-10.0	-9.6	0.35	2.359	0.531	5.430	1.511	6	7942	ZINC000257390556
4.1	-9.8	-9.7	0.10	2.169	0.236	5.331	0.946	6	7943	ZINC000013429517
4.1	-9.7	-9.5	0.14	3.043	0.606	5.102	1.490	6	7944	ZINC000015046508
4.1	-9.0	-8.9	0.11	4.354	0.436	7.249	0.497	6	7945	ZINC000009212425
4.1	-9.8	-8.9	0.59	2.861	1.031	5.262	2.336	6	7946	ZINC000257785071

4.1	-9.5	-9.2	0.19	3.235	0.898	5.677	2.461	6	7947	ZINC000040164398
4.1	-9.6	-9.4	0.15	2.146	0.353	6.388	1.366	6	7948	ZINC000253613401
4.1	-9.7	-9.4	0.25	2.730	0.521	8.001	2.192	6	7949	ZINC000014684667
4.1	-9.3	-9.0	0.23	2.400	0.408	5.566	0.549	6	7950	ZINC000059736600
4.1	-9.8	-9.3	0.28	2.655	0.589	6.362	1.128	6	7951	ZINC000031457169
[truncated: 781,518 more chars]
